# Exploring the diagnostic markers of essential tremor: A study based on machine learning algorithms

**DOI:** 10.1515/biol-2022-0622

**Published:** 2023-06-22

**Authors:** Yuan Gao, Li Ding, Jiang Liu, Xiaoyan Wang, Qiang Meng

**Affiliations:** Department of Neurology, The First People’s Hospital of Yunnan Province, The Affiliated Hospital of Kunming University of Science and Technology, Kunming 650000, Yunnan, China; Medicine School, Kunming University of Science and Technology, Kunming 650000, Yunnan, China

**Keywords:** essential tremor, diagnosis markers, high-throughput sequencing, immune microenvironment, functional enrichment analysis

## Abstract

Essential tremor (ET) is a common neurological disorder with a difficult clinical diagnosis, primarily due to the lack of relevant biomarkers. The current study aims to identify possible biomarkers for ET by screening miRNAs using machine learning algorithms. In this investigation, public datasets and our own datasets were used to examine the ET disorder. The ET datasets originated from public sources. To generate our own dataset, high-throughput sequencing analyses were performed on ET and control samples from the First People’s Hospital of Yunnan Province. Functional enrichment analysis was employed to identify the potential function of differentially expressed genes (DEGs). Using datasets from the Gene Expression Omnibus database, Lasso regression analysis and support vector machine recursive feature elimination were used to screen potential diagnostic genes for ET. To identify the genes responsible for the final diagnosis, area under the curves (AUCs) of the receiver operating characteristic was examined. Finally, an ssGSEA representing an ET immune landscape was created. The sample exhibited expression profiles that corresponded with six genes in the public database. Three diagnostic genes were discovered with AUCs >0.7 that can distinguish ET from normal data: APOE, SENP6, and ZNF148. Single-gene GSEA indicated that these diagnostic genes were closely associated with the cholinergic, GABAergic, and dopaminergic synapse networks. The immune microenvironment of ET was also affected by these diagnostic genes. According to the findings, these three DEGs (*APOE*, *SENP6*, and *ZNF148*) may successfully differentiate between samples from ET patients and normal controls, serving as a helpful diagnostic tool. This effort provided a theoretical foundation for elucidating the pathogenesis of ET and raised hopes of overcoming the diagnostic difficulty of ET clinically.

## Graphical abstract

**Figure:**
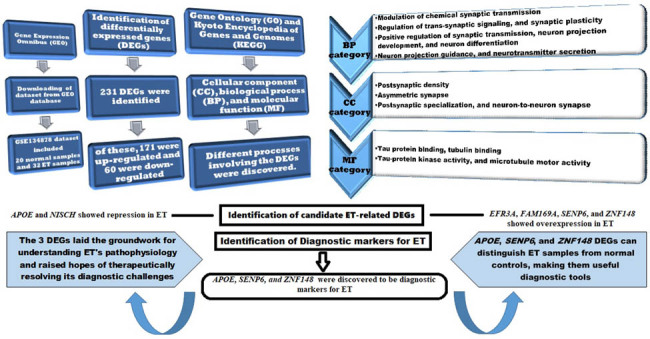

## 1 Introduction

Essential tremor (ET) is a widely prevalent movement disorder that is characterized by bilateral tremors in the upper limbs during both postural and kinetic activities. It has been suggested that ET is among the most frequently occurring neurodegenerative conditions [1–3]. Clinical symptoms and epidemiology in ET appear to be related. For instance, a study discovered that the incidence of ET has two peaks: early-onset (before the age of 25) and late-onset (after the age of 65) [4]. Positive family history and sensitivity to alcohol are more frequent characteristics of young-onset ET. A higher incidence of dementia has been related to late-onset ET [5]. A meta-analysis conducted globally revealed that the prevalence of ET was 0.4–0.9% across all age groups, while it was 4.6–6.3% in populations aged 65 years and above [6,7]. In addition to tremor, other symptoms of ET include gait and balance impairment, moderate cognitive deficiency, psychiatric symptoms, and hearing loss [8–11]. Recently, the term ET-Plus has been used to characterize these soft signs [12,13].

The pathogenesis of ET is complex and inconclusive due to the involvement of multiple genetic and environmental etiologies. The precise etiology and pathogenesis of ET remains uncertain. Numerous studies in academic literature have indicated the significant influence of genetic factors [14]. The etiology of ET is supported by the prevalence of positive family history of tremor in patients with ET, indicating the involvement of genetic factors. It is estimated that genetic factors contribute to ET in a range of 20–90% of patients. Additionally, genetic anticipation, which refers to the earlier onset of tremor in the next generation, has been observed in ET patients. Furthermore, twin studies have shown higher concordance rates of ET for monozygotic twins compared to dizygotic twins [15,16]. The diagnosis of ET poses a challenge due to its diverse clinical manifestations, resulting in a high rate of misdiagnosis [17,18].

At present, the clinical diagnosis of ET heavily relies on the subjective clinical evaluation of medical professionals, and a minimum disease duration of 3 years is typically necessary for diagnosis [19]. Globally, there is a severe shortage of experienced neurologists. Therefore, researchers are focusing on incorporating objective measurement indices into the auxiliary diagnosis of ET [20]. Neuroimaging and neurophysiology technologies provide new ideas for confirming the specific pathogenesis of ET. High-precision invasiveness diagnosis, such as ^123^I-FP-CIT SPECT, has been determined to be the most effective diagnostic instrument in this respect; however, only developed nations can afford it due to its high cost and high consumption [21]. The International Parkinson and Movement Disorder Society (IPMDS) standardized the diagnostic criteria for ET, stating that a 3-year history of isolated action tremor in the absence of any other neurologic condition is required [19]. In order to accurately diagnose a patient, doctors meticulously collect clinical information, including historical features (age at onset, family history), tremor characteristics (body distribution, activation condition), related indicators, and laboratory testing (electrophysiology, imaging, and scales). Due to their age and poor mobility, many ET patients may be unable to see a doctor right away, which would delay receiving an accurate diagnosis and prompt treatment. To help with diagnosis and to distinguish between diagnoses at the early stage of ET, various sensitive and specific detection indexes are still anticipated. As a result, noninvasive auxiliary solutions that are low cost and high efficiency are a new hotspot for ET early diagnosis.

The ET has been linked to cerebellar function, according to the research. The cerebellar degeneration hypothesis is now regarded as a significant player on the ET stage, encompassing movement disorders, cognitive disorders, and affective disorders [22,23]. According to certain scholars, a minimum of 27 miRNAs participate in the regulation of gene expression after transcription and are implicated in the processes of apoptosis and necrosis in cerebellar neurons [24]. Microarrays and bioinformatics analytical tools have been extensively utilized for the identification of differentially expressed miRNAs in ET over the years [25]. A number of miRNAs have been documented to exhibit anomalous expression in individuals with ET, and could potentially have clinical implications [26,27]. In addition, miRNAs have been suggested as potential non-invasive biomarkers for the diagnosis, prognosis, and treatment response of various neurodegenerative conditions, including Parkinson’s disease (PD) and Alzheimer’s disease (AD) [28].

The field of bioinformatics has undergone significant advancements, enabling the screening of a greater number of genes in a more efficient and precise manner compared to traditional experimental research, which is often costly and time-consuming. Bioinformatics analysis has the potential to offer exploratory predictions at a reduced cost, thereby informing subsequent biological experiments and clinical applications [29]. The Hub gene, due to its significant connectivity within the gene expression network, is believed to have a crucial impact on the advancement of the disease [30]. In earlier investigations, hub genes were frequently discovered using the STRING (Search Tool for the Retrieval of Interacting Genes) or cytoHubba program [30]. However, the quality of the screening process and the reproducibility of the experiment are compromised when researchers are allowed to use their personal preferences to determine whether the top 5 or 10 of total differential expression genes (DEGs) should be chosen as hub genes [31]. Various machine learning (ML) approaches have recently been introduced to bioinformatics analysis in an effort to reduce this type of inaccuracy, and it has been demonstrated that doing so improves the accuracy and stability of the screening procedure [32]. As a normalized linear regression method, the least absolute shrinkage and selection operator (LASSO) regression can disregard unimportant features and construct a sparse and easily interpretable model to prevent overfitting. With the support vector machine recursive feature elimination (SVM-RFE) method, the support vector machine is incorporated into the recursive feature elimination approach and its inherent feature selection function is used to continuously screen important features. In several sectors, the combination of LASSO and SVM-RFE algorithms has demonstrated adequate sensitivity and accuracy. High classification performance is achieved by this model, which is recognized to remove irrelevant information effectively [33,34]. Additionally, the most effective techniques currently employed in studies on miRNA biomarkers include bioinformatics and biological tools [35].

In order to assess the potential of the miRNAs to serve as ET biomarkers, this study screened miRNAs based on ML methods utilizing sequencing data from both public databases and the own dataset. Three diagnostic genes, *APOE*, *SENP6*, and *ZNF148*, all with area under the curves (AUCs) >0.7, were found to have significant diagnostic values for distinguishing ET from normal samples. The synaptic signaling transmission and immunological microenvironment of neurons were thought to be closely related to these diagnostic genes. The present study employed an innovative bioinformatics approach to identify putative biomarkers for ET, which could potentially enhance the accuracy of clinical diagnosis.

## 2 Materials and methods

### 2.1 The public data source

The Gene Expression Omnibus (GEO) database, which can be accessed at  https://www.ncbi.nlm.nih.gov/geo/ as of November 1, 2022, is a publicly available international repository that contains functional genome datasets generated through high-throughput microarray and next-generation sequencing techniques. The National Center for Biotechnology Information is responsible for the creation and maintenance of this database [36]. This database contains virtually all gene expression assay data that is useful for scientific investigation [37]. Twenty normal samples and 32 ET samples were included in the GSE134878 dataset, which was downloaded for this investigation from the GEO database. For the primary analysis of this study, this dataset was used.

### 2.2 Patient preparation

The Department of Neurology at the First People’s Hospital of Yunnan Province, China, recruited three patients with ET. All patients underwent clinical and neuropsychological examinations, brain magnetic resonance imaging, and thyroid function evaluations in accordance with IPMDS [19] criteria. Exclusion criteria included concurrent or recent exposure to tremorgenic drugs, hyper- and hypothyroidism, hyperparathyroidism, physiological and psychogenic tremor, premorbid clinically significant psychiatric disorders, alcohol and narcotic addictions, inflammatory diseases, cancer, chronic diseases, and a history of major surgery. All of the patients had a 3-year history of bilateral upper limb tremors. In addition, three age- and gender-matched healthy subjects were recruited as controls [38]. Fahn Tolosa Marin scale was utilized to evaluate the severity of tremor [39]. The Chinese version of the Montreal Cognitive Assessment (score 0–30) was used to evaluate cognitive function. The Beck Depression Inventory (score 0–63) was used to assess affective symptoms.


**Informed consent:** Informed consent has been obtained from all individuals included in this study.
**Ethical approval:** The research related to human use has been complied with all the relevant national regulations, institutional policies and in accordance with the tenets of the Helsinki Declaration, and has been approved by Medical Ethics Committee of the First People’s Hospital of Yunnan Province (YYLH005, February 2019).

### 2.3 RNA-seq analysis

Peripheral blood mononuclear cells (PBMCs) were isolated from ET patients and healthy controls by Ficoll density gradient centrifugation and Lymphoprep (Stemcell, USA). Following their separation, PBMCs were lysed using a TRIzol Reagent (Invitrogen, USA) and kept at −80°C for further processing. Then, according to the manufacturer’s recommendations, total RNA was isolated using a mirVana miRNA Isolation Kit (Ambion, Foster City, CA). The quantity of RNA was assessed using a NanoDrop 2000 (Thermo Fisher Scientific, Waltham, MA), while RNA quality was evaluated using an Agilent 2100 bioanalyzer (Agilent Technologies, Santa Clara, CA). Purified libraries were created using Illumina TruSeq Stranded Total RNA Sample Preparation Kits (Illumina, San Diego, CA) in accordance with the manufacturer’s instructions, and their quantities were determined using an Agilent 2100 bioanalyzer and a Qubit 2.0 Fluorometer (Life Technologies, Carlsbad, CA). Libraries were utilized to create a cluster using the cBot software. Subsequently, the cluster underwent sequencing utilizing the Illumina HiSeq 2500 platform, which is located in San Diego, CA. The sequencing procedures were conducted by Origin-Biotech Inc. (Ao-Ji Bio-Tech, Shanghai, China).

### 2.4 Analysis of DEGs

The differential expression files, which had undergone DESeq2 processing, were acquired directly from the GEO database (Table S1). DEGs were identified based on the statistical criteria of a significance level of *P* < 0.05 and a false discovery rate threshold of <0.25. [41]. The *t*-test assay was utilized to obtain the expression pattern of the DEGs in the own dataset, as indicated in Table S2.

### 2.5 Functional enrichment analysis

The DEGs underwent functional enrichment analysis using the clusterProfiler package, specifically through the utilization of Gene Ontology (GO) and Kyoto Encyclopedia of Genes and Genomes (KEGG). Cellular component (CC), biological process (BP), and molecular function (MF) were the three basic categories used in the GO analysis. The KEGG signaling pathway was considered a pre-established gene set, and subsequently, gene set enrichment analysis (GSEA) was conducted on individual diagnostic markers to identify the specific pathway associated with each diagnostic marker. The adjusted (adj.) *P* < 0.05 values were regarded as significant for all statistical analyses.

### 2.6 LASSO and SVM-RFE algorithms

DEGs with consistent expression trends in their own and GSE134878 datasets were regarded to be potential ET-related DEGs. The LASSO and SVM-RFE algorithms were utilized to filter candidate diagnostic genes for ET from among the candidate DEGs associated with ET. The glmnet (version 3.0) and e1071 (version 1.7−3) R package (version 3.6.0) were utilized to conduct LASSO and SVM-RFE, respectively. The genes that were identified by both algorithms were considered as potential diagnostic genes. By comparing the AUC of the receiver operating characteristic (ROC) curves, the efficacy of candidate diagnostic genes in differentiating ET samples from normal samples was evaluated. A gene with AUCs greater than 0.7 in both the GSE134878 and own datasets was considered the diagnostic gene.

### 2.7 Single sample GSEA (ssGSEA)

ssGSEA is a GSEA deconvolution algorithm that translates gene expression profiles into quantitative fractions of immune cells in a single sample. The GSVA function in the R package was utilized to evaluate the distribution of 24 immune cell subtypes in each sample of the GSE134878 dataset [42]. Since no sample included evidence of the expression of the IL3RA-only gene in pDC cells, these cells were specifically excluded of all subsequent analyses.

### 2.8 Statistical analysis

Pearson correlation analysis was employed to calculate correlations, and a correlation was deemed significant if it satisfied the criteria of |correlation (cor)| ≥0.3 and *P* < 0.05. The regulatory networks in which diagnostic genes may be engaged were revealed using Ingenuity Pathway Analysis (IPA). The statistical analyses were primarily conducted using the R package (version 3.6.0). Statistical analyses were conducted with a significance level of *P* < 0.05, indicating statistical significance.

## 3 Results

### 3.1 Filtering of ET-related DEGs and unveiling of their potential functions

**Figure 1:**
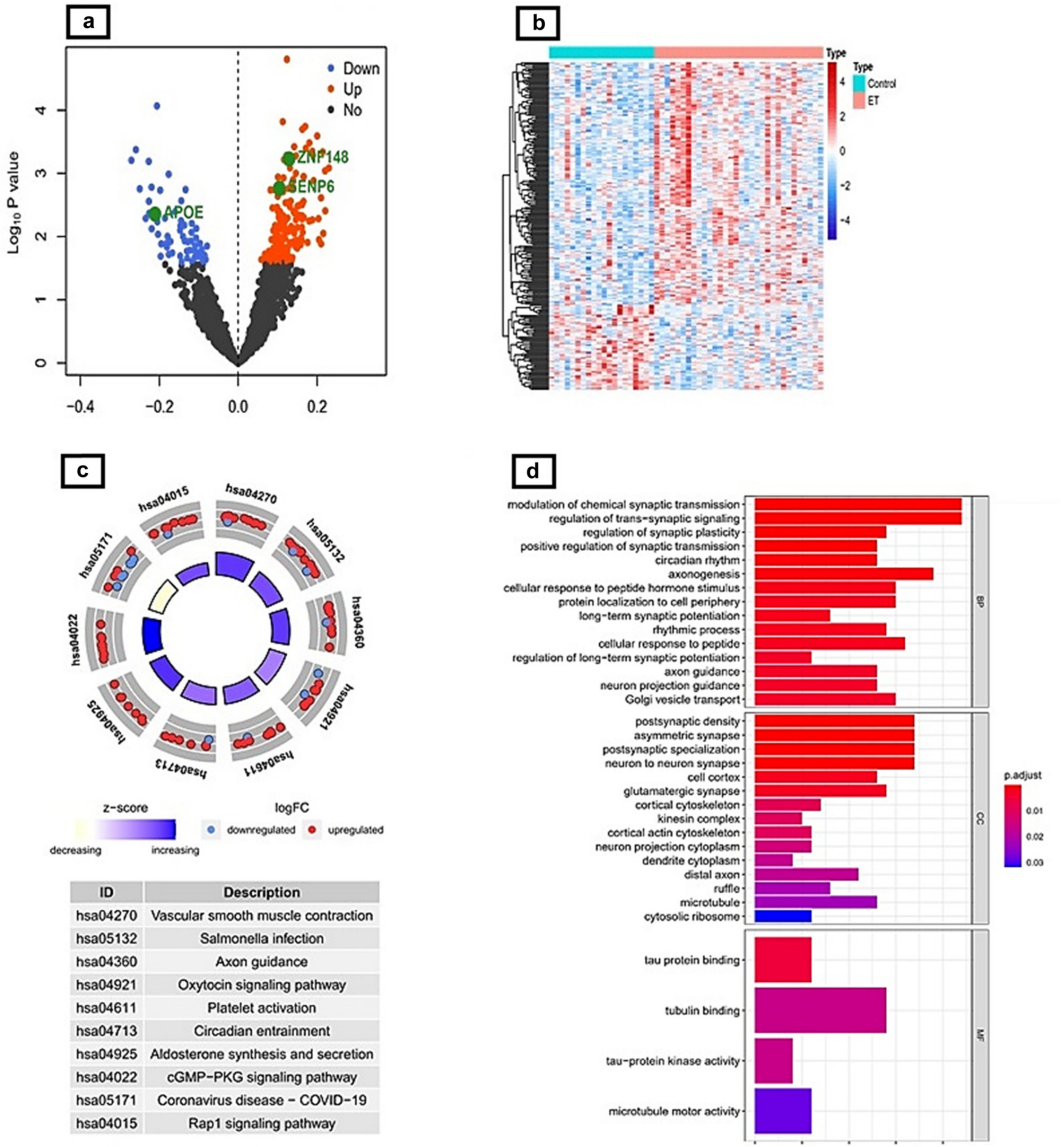
(a) Volcano plot showing DEGs for the ET and control groups (black dots denote no significant difference, red dots denote differential expression that was up-regulated, blue dots denote differential expression that was down-regulated). (b) Heat map of DEGs between the ET and control groups. (c) Results of GO enrichment analysis for DEGs. (d) Results of the investigation of KEGG enrichment for DEGs.

A total of 231 DEGs related to ET were identified in the GSE134878 dataset, as shown in Table S3. Figure 1a shows that of these, 171 were up-regulated and 60 were down-regulated. Furthermore, the expression pattern of the DEGs in the GSE134878 dataset was demonstrated through a heat map, as depicted in Figure 1b.

The potential roles of DEGs in the pathogenic progression of ET were then investigated using GO analysis (Figure 1c; Table S4). The DEGs identified in the BP category were observed to have a close association with synaptic transmission and neuronal conduction. Specifically, the DEGs were found to be involved in various processes such as modulation of chemical synaptic transmission, regulation of trans-synaptic signaling, regulation of synaptic plasticity, positive regulation of synaptic transmission, neuron projection guidance, positive regulation of neuron projection development, neurotransmitter secretion, and positive regulation of neuron differentiation. The identified genes were observed to have significant involvement in cellular structures such as the postsynaptic density, asymmetric synapse, postsynaptic specialization, and neuron-to-neuron synapse. In addition, it was observed that four terms exhibited significant enrichment in the MF category. These terms include tau protein binding, tubulin binding, tau-protein kinase activity, and microtubule motor activity. In addition, the KEGG analysis revealed that the DEGs were primarily associated with various biological processes such as vascular smooth muscle contraction, Salmonella infection, axon guidance, and oxytocin signaling pathway (as shown in Figure 1d and Table S5).

### 3.2 Identification of candidate ET-related DEGs

**Figure 2:**
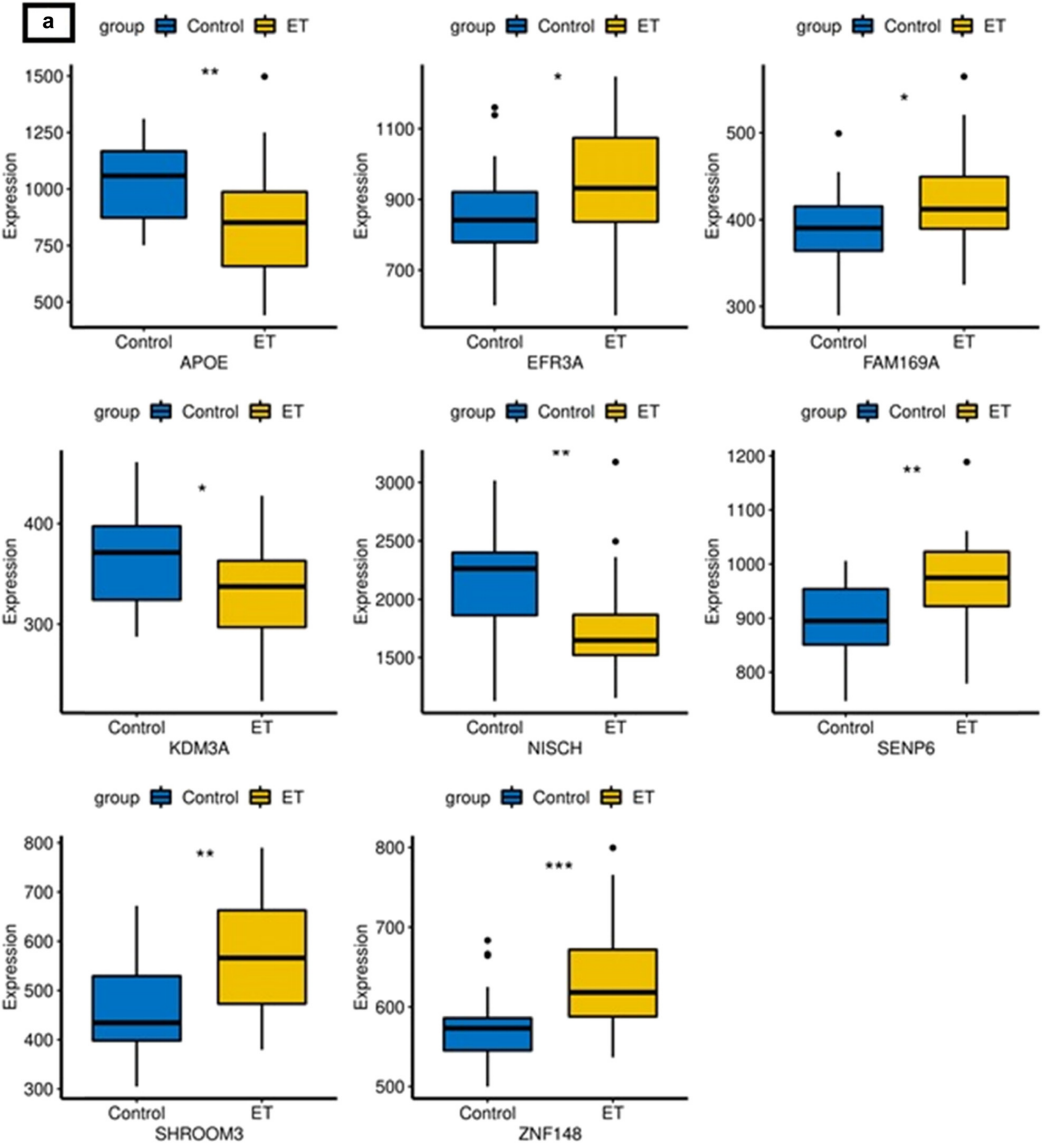

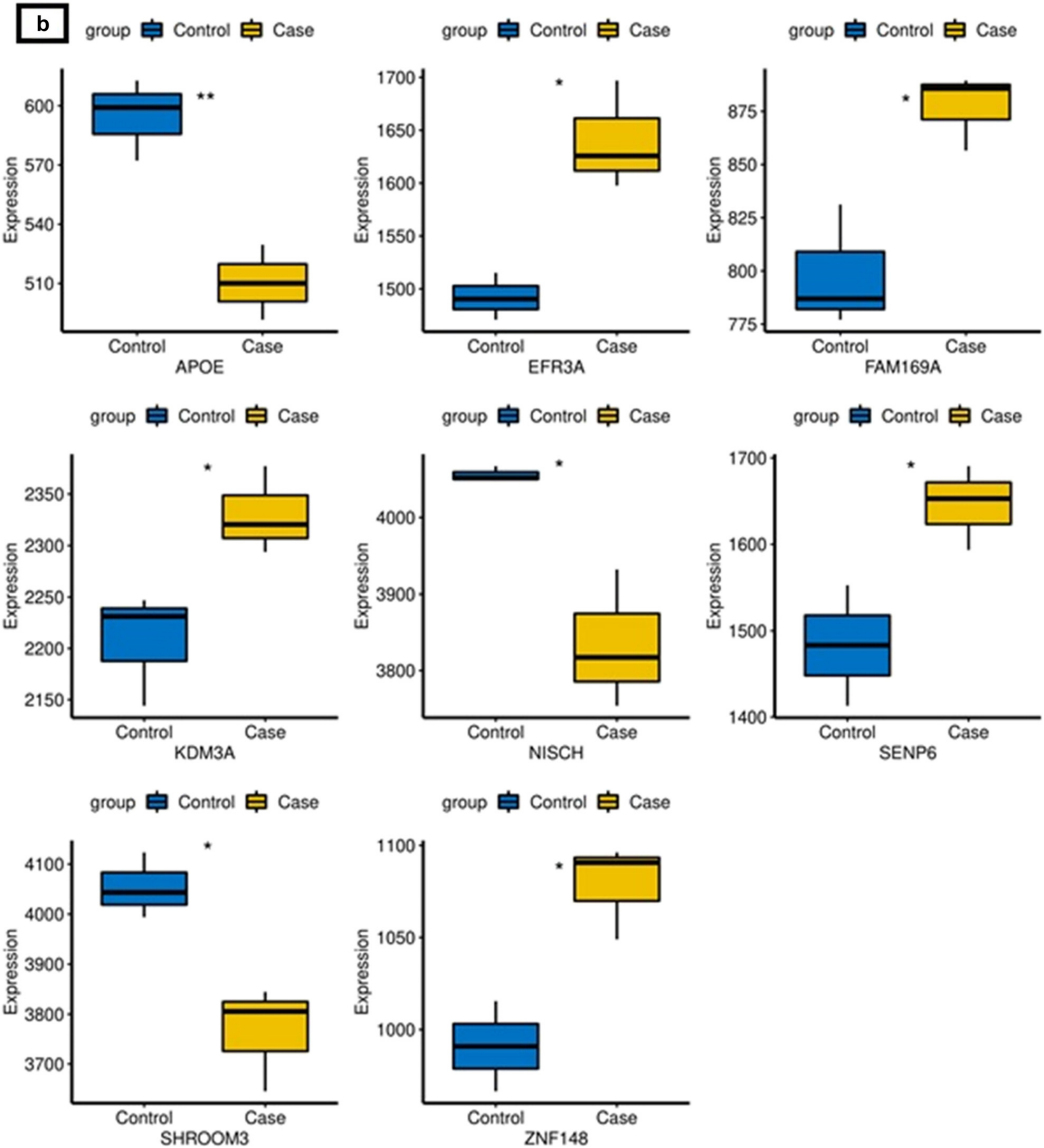
Expression boxplot of eight different genes in GSE134878 (a) and the own dataset (b), respectively.

The expression pattern of the aforementioned DEGs was verified between a collection of three normal and three ET samples using *t*-tests. A total of 231 DEGs were identified, out of which eight DEGs exhibited differential expression in the dataset under investigation. Subsequently, six out of the eight DEGs exhibited consistent expression patterns in the GSE134878 dataset. The study found that the genes *EFR3A*, *FAM169A*, *SENP6*, and *ZNF148* exhibited overexpression in ET, while *APOE* and *NISCH* showed repression in ET, as depicted in Figure 2. These genes were identified as potential ET-related DEGs and were selected for further analysis (Figure S1).

### 3.3 Screening for ET diagnostic markers and assessment of their diagnostic validity

**Figure 3:**
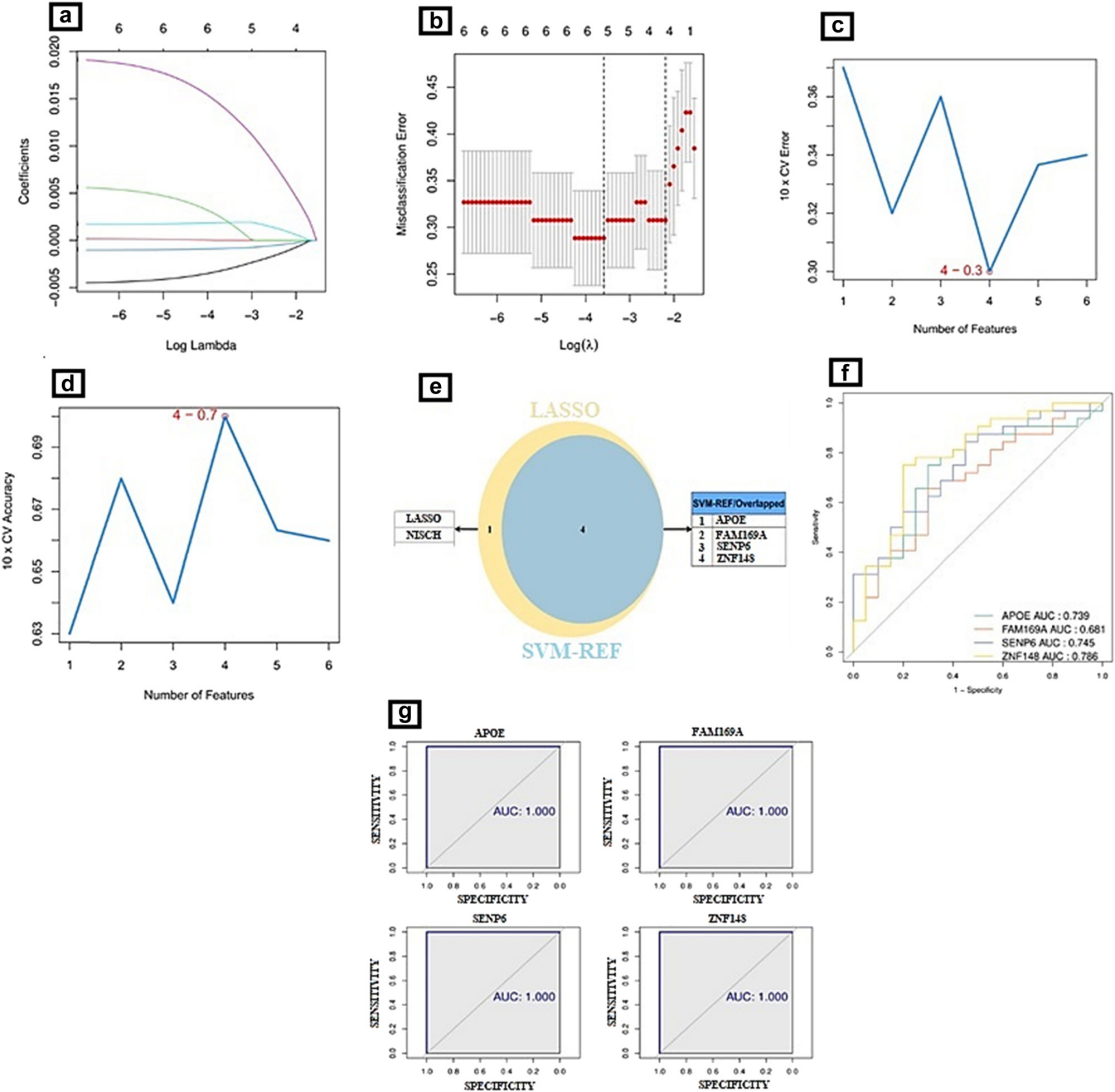
(a and b) The LASSO algorithm identified a characteristic gene set containing five DEGs. (c and d) The SVM-RFE algorithm identified a characteristic gene set containing four DEGs. (e) Four DEGs were discovered by overlapping the characteristic genes selected by the LASSO algorithm and the SVM-RFE algorithm. (f) ROC curve analysis of characteristic genes in GSE134878 dataset. (g) Examination of the ROC curves for the characteristic genes in the own dataset. (The results of the ROC curve analysis indicate that *APOE*, *SENP6*, and *ZNF148* exhibited high classification accuracy in both the GSE134878 dataset [with all AUCs greater than 0.7] and the researcher’s own dataset [with all AUCs equal to 1]).

Two distinct methodologies were utilized to identify the most effective diagnostic markers for ET by analyzing potential DEGs associated with ET. The LASSO algorithm was utilized to identify a set of characteristic genes, which consisted of five DEGs, as depicted in Figure 3a and b. On the other hand, the SVM-RFE algorithm was employed to select a set of four DEGs, as illustrated in Figure 3c and d. Through the process of overlapping the biomarkers selected by the two algorithms, a total of four DEGs were identified, as illustrated in Figure 3e. These DEGs were subsequently identified as potential feature genes for the classification of ET. In this study, a cohort of 52 test samples sourced from the GSE134878 dataset, alongside six validation samples from an own dataset, were classified into ET and normal groups based on the utilization of four potential feature genes. The results of the ROC curve analysis indicate that *APOE*, *SENP6*, and *ZNF148* achieved a high level of classification accuracy in both the GSE134878 dataset (with all AUCs greater than 0.7, as shown in Figure 3f) and the researcher’s own dataset (with all AUCs equal to 1, as shown in Figure 3g). Therefore, these genes have been validated as diagnostic markers for ET. Figure 1a depicts the annotation of ET diagnostic markers’ expression patterns in the GSE134878 dataset.

### 3.4 Single-gene GSEA for ET diagnostic markers

**Figure 4:**
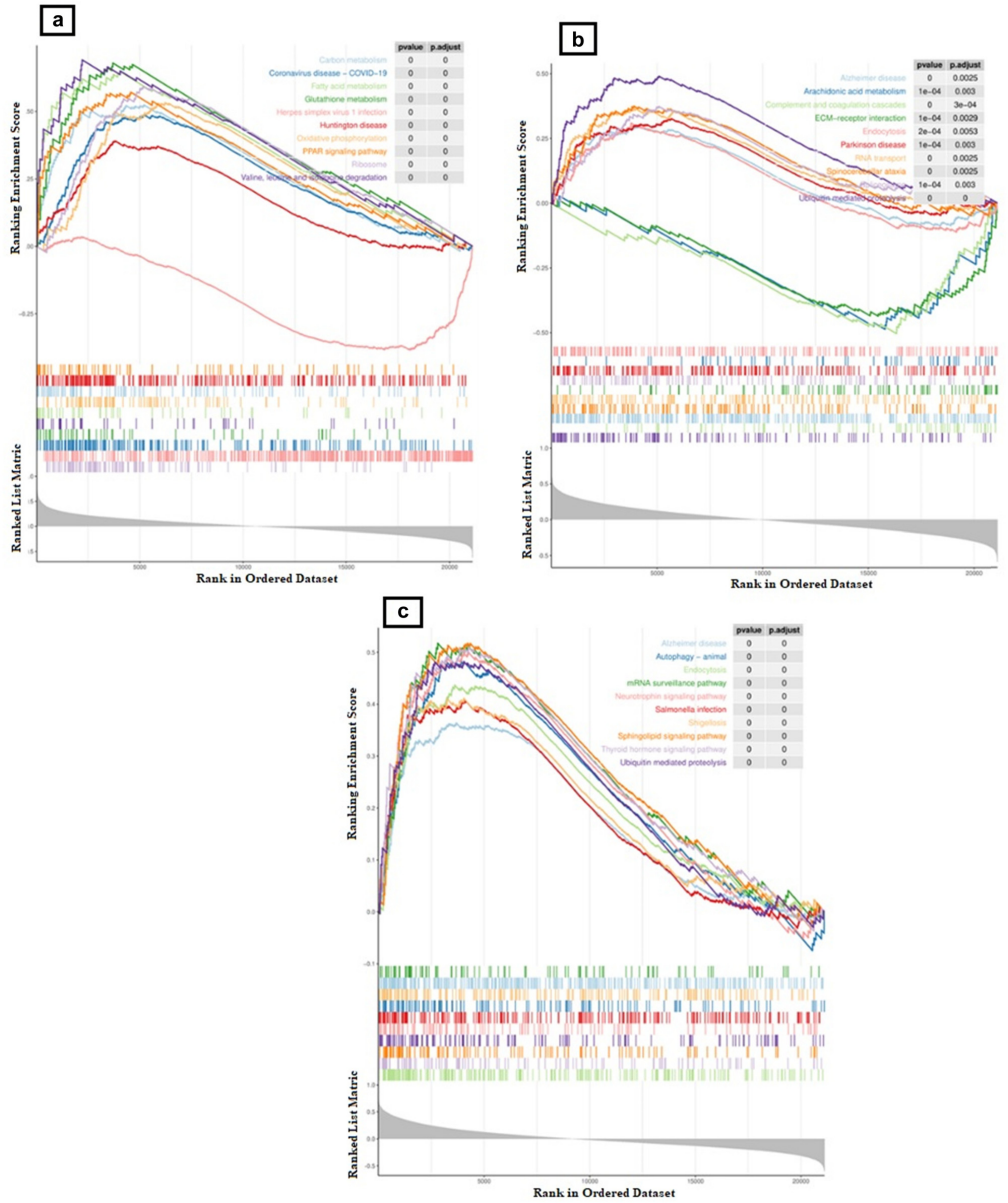
(a) Single-gene GSEA for *APOE*, (b) single-gene GSEA for *SENP6*, and (c) single-gene GSEA for *ZNF148.*

The KEGG signaling pathway was utilized as a pre-gene set, and GSEA was used to reveal the signaling pathways implicated in each of the three diagnostic markers. The study found that *APOE* was associated with 151 enriched pathways. The three most enriched pathways were identified as “Ribosome,” “Herpes simplex virus 1 infection,” and “Coronavirus disease – COVID-19,” as shown in Figure 4a and Table S6. The study identified 32 pathways associated with *SENP6*, with the “Ubiquitin mediated proteolysis,” “Complement and coagulation cascades,” and “AD” pathways being the most significant, as depicted in Figure 4b and Table S7. The study identified 145 pathways associated with *ZNF148* through GSEA. Among these pathways, “Endocytosis,” “Thyroid hormone signaling pathway,” and “Sphingolipid signaling pathway” were found to have the strongest correlation with *ZNF148*, as depicted in Figure 4c and Table S8. Remarkably, the investigation revealed a significant correlation between the genes and specific synaptic pathways, namely “GABAergic synapse” [43] (*APOE*, *ZNF148*), “Cholinergic synapse” [44] (*ZNF148*), “Dopaminergic synapse” [45] (*ZNF148*), and “Serotonergic synapse” [46] (*ZNF148*). Moreover, these diagnostic markers were implicated in diverse neurodegenerative disease pathways, including Huntington’s disease (HD), PD, amyotrophic lateral sclerosis, AD, and spinocerebellar ataxia. The findings indicate that the aforementioned genes could potentially play a role in the development of ET and other associated neurodegenerative disorders. Additionally, Figure S1 illustrates a potential regulatory network implicated in the diagnostic markers identified by IPA.

### 3.5 Immune landscape analysis of ET patients

**Figure 5:**
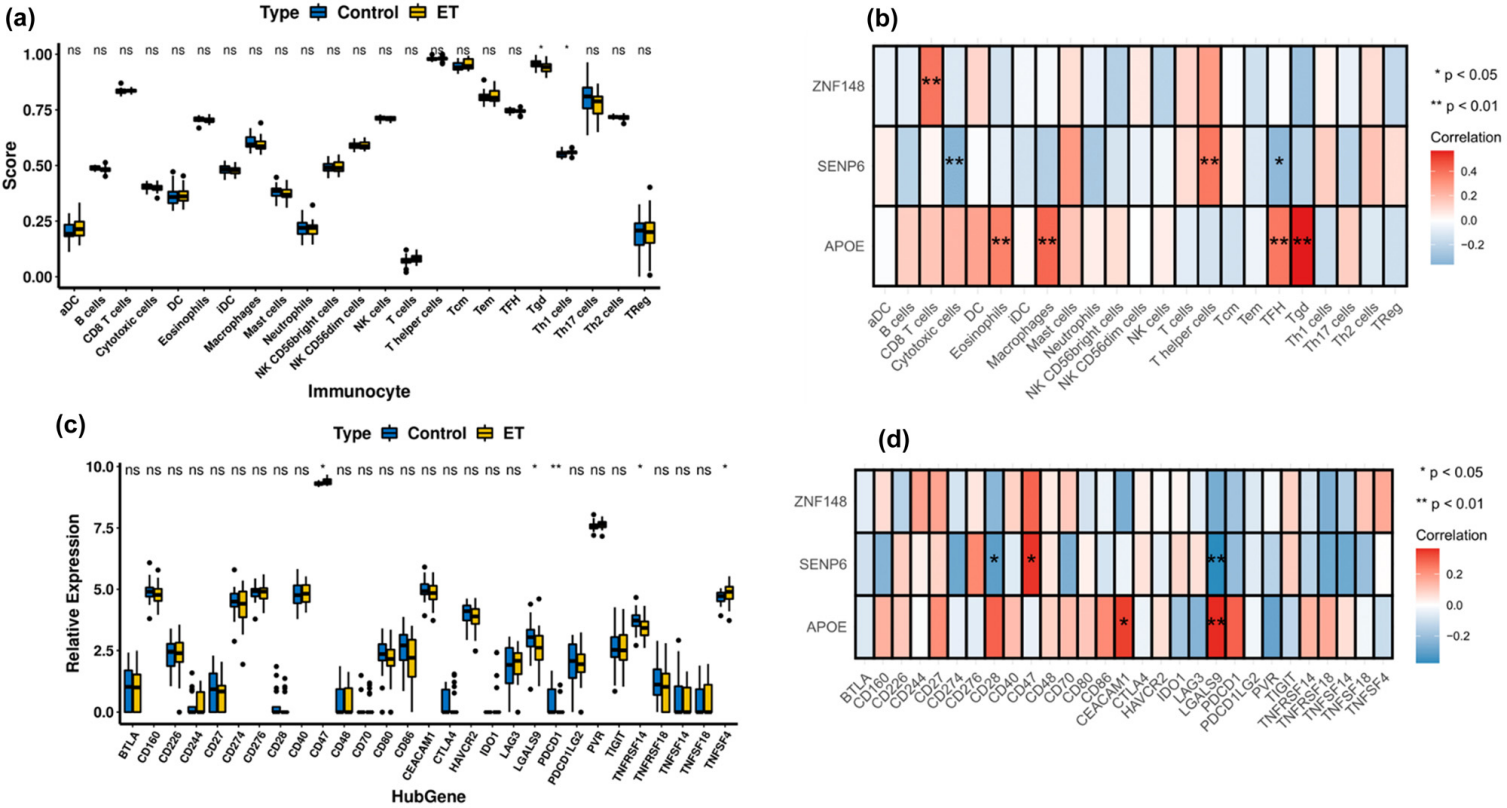
(a) Expression of immune infiltrating cells between the ET and control groups (only the fractions of Tgd and Th1 cells were significant between normal and ET patients, with Tgd being less infiltrated in ET patients, while Th1 cells were more widely distributed in ET patients). (b) Relationship between diagnostic markers and immune infiltrating cells by Pearson correlation analysis. (c) Expression levels of 29 immune checkpoint molecules in the ET and control groups. (d) Relationship between diagnostic markers and immune checkpoint molecules by Pearson correlation analysis.

The study conducted a functional enrichment analysis of DEGs and diagnostic markers associated with ET. The results indicated a strong association between these genes and the immune response, including pathways such as the cAMP signaling pathway, cell adhesion molecules, antigen processing and presentation, Th17 cell differentiation, ECM–receptor interaction, T cell receptor signaling pathway, and AMPK signaling pathway. Inspired by this, the ssGSEA algorithm was used to estimate the proportion of immune infiltrating cells between normal and ET patients in the GSE134878 dataset. The findings indicate that the fractions of Tgd and Th1 cells were the only statistically significant differences observed between the two groups. Specifically, Tgd cells were found to be less infiltrated in ET patients, while Th1 cells were more widely distributed in ET patients (Figure 5a and Figure S1). Subsequently, the correlation between diagnostic markers and immune infiltrating cells was calculated using Pearson correlation analysis (Figure 5b and Table S9). The findings indicate a significant positive association between *APOE* and Tgd (cor = 0.56, *P* = 1.32 × 10^−5^), macrophages (cor = 0.42, *P* = 0.00185), TFH (cor = 0.37, *P* = 0.006253), and eosinophils (cor = 0.36, *P* = 0.009681). The findings suggest that SENP6 exhibits a negative correlation with cytotoxic cells (cor = −0.36, *P* = 0.008547) and TFH (cor = −0.33, *P* = 0.015557), whereas a positive correlation was observed with T helper cells (cor = 0.38, *P* = 0.005584). Only CD8 T cells exhibited a positive correlation with *ZNF148* (cor = 0.39, *P* = 0.004593) (Figure S3).

The study conducted revealed a significant association between *ZNF148* and the expression of PD-L1 and the PD-1 checkpoint pathway in cancer. As a result, an investigation was carried out to determine the expression levels of 29 immune checkpoint molecules in both normal and ET patients, as illustrated in Figures S2 and S3. The results revealed that CD47 and TNFSF4 exhibited high expression levels in patients with ET, while LGALS9, PDCD1, and TNFRSF14 were highly expressed in the normal group, as depicted in Figure 5c. The results of Pearson correlation analysis indicated that CD47 exhibited a strong positive correlation with *SENP6* (cor = 0.35, *P* = 0.010676). Additionally, LGALS9 demonstrated significant positive and negative correlations with *APOE* (cor = 0.36, *P* = 0.008381) and *SENP6* (cor = −0.37, *P* = 0.006177), respectively. However, no significant correlation was observed among TNFSF4, TNFRSF14, and the diagnostic markers, as shown in Figure 5d and Table S10 (Figure S2).

## 4 Discussion

This study represents a novel investigation into the diagnostic markers of ET utilizing ML algorithms, with the aim of elucidating the underlying pathogenesis. During the preliminary investigation, it was observed that three diagnostic genes played a significant role in the diagnosis process and were implicated in complex pathophysiological mechanisms. The aforementioned discoveries have facilitated a redefinition of our comprehension of the mechanism and have provided clarification regarding the complexity of clinical manifestations.

The manifestation of tremors, as observed in clinical settings, can be attributed to the interaction between the cerebellar thalamus, cortex, and muscle, resulting in involuntary muscle contractions [47,48]. Several neuroimaging studies have demonstrated the existence of functional, metabolic, and structural defects in the cerebellum of individuals with ET [49–51]. The postmortem literature has been expanding and has identified microscopic abnormalities in the brain of individuals with ET. These abnormalities are primarily focused on the Purkinje cells and synaptic transmission [52–54]. According to certain researchers, compared to controls, ET patients had significantly lower dendritic complexity and spine density [55]. The Purkinje cells, acting as efferent neurons within the cerebellar cortex, receive primary excitatory inputs from the climbing fibers of olivary cells via synapses. Subsequently, they transmit information through synapses to establish a one-to-one relationship [56,57]. The cerebellum of individuals with ET has been found to exhibit anomalies in Glu synapses and GABA synapses, particularly in terms of synapse density, as indicated by postmortem studies [58–60]. The aberrant formation of Purkinje cells and their synapses, leading to cellular remodeling and degeneration, has been found to give rise to novel or atypical cortical circuits in individuals with ET, thereby contributing to the manifestation of associated clinical symptoms [61,62].

The potential functions of DEGs in the pathogenic course of ET were investigated via GO and KEGG pathway enrichment analysis. Despite the lack of clear correlation between DEGs and the nervous system in the KEGG analysis, it is postulated that the DEGs primarily impact synaptic transmission and neuronal conduction, as indicated by the results of the GO analysis (Figure 1c and d). The findings of the analysis indicated a close correlation between the DEGs and the functions of tau protein and microtubule-associated protein. These DEGs were found to be responsible for inducing abnormalities in the cytoskeleton. These genes were also discovered to play crucial roles in CCs such postsynaptic density, asymmetric synapses, and postsynaptic specialization. This finding explained the axonal and dendritic structural alterations in compromised Purkinje cells, as well as the underlying degenerative process involving the Purkinje cells and/or their microenvironment [63].

Three diagnostic genes were identified as the optimal markers for ET diagnosis from candidate DEGs associated with ET. The three diagnostic genes were found to have significant involvement in the process of cellular structure remodeling and metabolic function anomalies specifically in Purkinje cells. The GSEA analysis revealed that three diagnostic genes were significantly linked to diverse neurodegenerative disease pathways across distinct synapses, as depicted in Figure 4.

The *APOE* gene, which controls multifactorial/complex processes resulting in the early death of neurons, had been thoroughly investigated in ET. The *APOE* gene is known to regulate the metabolism of amyloid precursor protein and the accumulation of amyloid-beta (Aβ). It has also been found to promote the hyperphosphorylation of tau-protein, reduce choline acetyltransferase activity, increase oxidative processes, modify inflammation-related neuroimmunotropic activity, and alter synapse structure, among other effects [64,65]. An elevation in insoluble Aβ42 levels was previously observed in the cerebral parietal cortex of all individuals with ET, as well as in the cerebellar white matter of the majority of ET cases [66]. This accumulation of cerebellar Aβ42 indicated continuous neurodegeneration in a subset of ET patients and clarified the cognitive impairment of ET-plus patients [65]. The findings of this investigation indicate a decrease in the expression of *APOE* in individuals with ET, as demonstrated in Figure 2. The results were in agreement with prior research indicating that the reduction of plasma *APOE* levels could serve as a peripheral biomarker. This decrease may indicate an increase in ET brains, which has also been noted in individuals with AD [67].

The close association between *SENP6* and hereditary stability has been suggested as a role that is evolutionarily conserved in regulating chromatin dynamics and genome stability networks. This is achieved through the maintenance of a balance in the chromatin residency of protein complexes [68]. The results of the GSEA conducted on *SENP6* indicated that the pathways of “Ubiquitin mediated proteolysis” and “AD” were notably prominent, as illustrated in Figure 4b. Extensive research has been carried out suggesting that *SENP6* was responsible for modifying synaptic transmission and neuronal excitability in neurons via small ubiquitin-related modifier, which is compatible with the finding of this work [69]. *ZNF148* has been linked to various cellular processes, such as cell proliferation, differentiation, and programmed cell death, and has been identified as a potential tumor suppressor [70,71]. In research for HD, as well as a progressive neurodegenerative disorder, the researchers described that *ZNF148* directly interacted with three known-HD genes (*BCL2*, *CASP6*, and *IRS2*) [72]. Additionally, *ZNF148* had been identified as a previously unknown yet significant transcription factor in the corpus callosum’s development [73,74]. The evidence suggests that *ZNF148* may have a significant impact on abnormal neuronal proliferation or migration, as well as abnormal axonal growth or guidance [74]. The aforementioned results indicate that *SENP6* and *ZNF148* could potentially contribute to the development of ET and other associated neurodegenerative disorders.

It had been established that an imbalance in GABAergic inhibition can result in excitotoxicity and the upregulation of glutamatergic and cholinergic neurons, ultimately leading to the manifestation of delirium and involuntary tremors in patients with ET [75,76]. These three genes were found to be significantly linked to synapses and were observed to participate in the gene network of neurotransmitters. Learning disabilities and memory deficits were observed as a consequence of abnormal *APOE* gene expression in GABAergic interneurons [77,78]. The post-translational modifications occurring at both presynaptic buttons and post-synaptic terminals were found to regulate the activity of *SENP6* [69]. *SENP6* controlled a number of synaptic functions, including the degradation of target proteins, which is crucial for the creation and recall of memories [79]. Furthermore, it was demonstrated that *ZNF148* exhibited the ability to stimulate the promoter of *SLC6A1*, a transporter responsible for the termination of GABA activity through the elimination of GABA from the synaptic cleft [80]. Conversely, certain antiepileptic drugs (AEDs) have potential for employment in the clinical treatment of ET, including Mysoline (Primidone), Klonopin (Clonazepam), Ativan (Lorazepam), and Valium (Diazepam). The pharmacological targets of these AEDs were mainly focused on glutamatergic and GABAergic synapses [43]. The involvement of *APOE* and *ZNF148* in GABAergic synapses has been observed, indicating that these synapses may serve as potential targets for the treatment of ET using AEDs.

It was observed that the DEGs associated with (ETs were linked to the immune response, as depicted in Figure 5a–d. Despite ET being classified as a neurodegenerative disorder, increasing evidence has suggested that the activation of lymphocytes and microglia may contribute to neuroinflammation in ET [81–83]. The findings indicate that there were significant differences in the fractions of Tgd and Th1 cells between normal individuals and those with ET. Specifically, Tgd cells were found to be less infiltrated in ET patients, while Th1 cells were more widely distributed in ET patients, as depicted in Figure 5a. Th1 cells stimulated macrophages by means of interleukin 2 (IL-2), which caused the release of interleukin 8 (IL-8) [84]. A recent study has reported that patients with ET exhibit considerably elevated serum levels of IL-8 in comparison to control patients. Additionally, the study found a positive correlation between the severity of tremor and the serum IL-8 level, which further supports our conclusion [81]. Tgd cells have been observed to participate in a multifaceted immune response within the gut–brain axis, which has been shown to exert a substantial impact on the pathogenesis of central nervous system neurodegenerative disorders, including PD and AD, through immune cell-mediated mechanisms [85,86]. The intestinal microflora in the context of autoimmune disease may also have an impact on the pathological and clinical outcome of ET [87].

The findings of our study indicate a significant positive association between *APOE* and Tgd, macrophages, TFH, and eosinophils. The study revealed that *SENP6* exhibited an inverse association with cytotoxic cells and TFH, whereas it displayed a direct association with T helper cells. The results presented in Figure 5b indicate a positive correlation between *ZNF148* and CD8 T cells. The relevant research was examined, revealing that *APOE* has an impact on physiology and pathophysiology at various levels. These levels include the inhibition of CD4^+^ and CD8^+^ lymphocytes, regulation of macrophage function, modulation of inflammation and oxidation, and reduction of IL-2 production [88,89]. The inhibition of neuroinflammation was achieved through the dampening of nuclear factor kappa-B (NF-κB) activation by *SENP6* [90]. *ZNF148* was found to exert an influence on immunoreactivity in T cells through its regulatory role in the expression of immune molecules [91]. The findings of this study suggest an indirect association between neuroimmune activity and the onset of ET.

The study conducted an in-depth analysis of the expression levels of immune checkpoint molecules in patients with ET and explored the correlation between immune checkpoint molecules and diagnostic markers, representing truly an innovative approach in the field. In particular, it was discovered that CD47 had a high level of expression in ET patients, and that this expression was both positive and substantially linked with *SENP6*. The aforementioned results have exposed the fundamental inflammatory mechanisms involved in ET and have also identified *SENP6* as a potentially efficacious gene therapy tool that could be further explored in the context of treating ET patients.

## 5 Conclusions

In conclusion, the findings revealed that these three DEGs (*APOE*, *SENP6*, and *ZNF148*) may effectively differentiate between samples from ET patients and normal controls, hence providing a useful diagnostic tool. The study employed bioinformatic methodology to anticipate the potential functional roles of DEGs and investigate their plausible involvement in the pathogenesis of ET. The finding had the potential to contribute to an improved understanding of the pathogenic processes that ultimately result in ET. However, the validation trial for detecting plasma miRNA expression levels in a large sample size of ET patients and controls has not been conducted due to financial limitations. Subsequently, additional verification experiments will be conducted in the forthcoming work. In addition, consistency of the findings of ML approaches needs to be evaluated on a larger dataset in the future to further establish the universality of ML. The precise biological roles and molecular processes by which these three DEGs contribute to the pathogenesis of ET will also be the subject of future studies.

## Supplementary Material

Supplementary Figures

Supplementary Table 1

Supplementary Table 2

Supplementary Table 3

Supplementary Table 4

Supplementary Table 5

Supplementary Table 6

Supplementary Table 7

Supplementary Table 8

Supplementary Table 9

Supplementary Table 10

## References

[1] Ma C, Zhang P, Pan L, Li X, Yin C, Li A, et al. Two-stage framework for automatic diagnosis of multi-task in essential tremor via multi-sensory fusion parameters. J King Saud Univ. 2022;34(10):8284–96.

[2] Louis ED, Faust PL. Essential tremor: the most common form of cerebellar degeneration? Cerebellum Ataxias. 2020;7(1):1–10. 10.1186/s40673-020-00121-1 PMC7427947 32922824

[3] Huang H, Yang X, Zhao Q, Ning P, Shen Q, Wang H, et al. Clinical characteristics of patients with essential tremor or essential tremor plus. Acta Neurol Scand. 2020;141(4):335–41. 10.1111/ane.13209 31863462

[4] Lou J-S, Jankovic J. Essential tremor: clinical correlates in 350 patients. Neurology. 1991;41(2 Part 1):234. 10.1212/wnl.41.2_part_1.234 1992367

[5] Louis ED. Essential tremor and the cerebellum. Handb Clin Neurol. 2018;155:245–58. 10.1016/B978-0-444-64189-2.00016-0 29891062

[6] Louis ED, Ferreira JJ. How common is the most common adult movement disorder? Update on the worldwide prevalence of essential tremor. Mov Disord. 2010;25(5):534–41. 10.1002/mds.22838 20175185

[7] Louis ED, Ottman R, Allen Hauser W. How common is the most common adult movement disorder? Estimates of the prevalence of essential tremor throughout the world. J Mov Disord. 1998;13(1):5–10. 10.1002/mds.870130105 9452318

[8] Fois AF, Briceño HM, Fung VS. Nonmotor symptoms in essential tremor and other tremor disorders. Int Rev Neurobiol. 2017;134:1373–96. 10.1016/bs.irn.2017.05.010 28805576

[9] Hopfner F, Deuschl G. Is essential tremor a single entity? Eur J Neurol. 2018;25(1):71–82. 10.1111/ene.13454 28905504

[10] Louis ED. Essential tremor:“Plus” or “Minus”. Perhaps now is the time to adopt the term “the essential tremors”. Park Relat Disord. 2018;56:111–2. 10.1016/j.parkreldis.2018.06.026 29937098

[11] Fabbrini G, Berardelli I, Falla M, Moretti G, Pasquini M, Altieri M, et al. Psychiatric disorders in patients with essential tremor. Park Relat Disord. 2012;18(8):971–3. 10.1016/j.parkreldis.2012.05.005 22658234

[12] Bellows ST, Jankovic J. Phenotypic features of isolated essential tremor, essential tremor plus, and essential tremor-Parkinson’s disease in a movement disorders clinic. Tremor Other Hyperkinet Mov. 2021;11:12–22. 10.5334/tohm.581 PMC8015706 33828900

[13] Bologna M, Berardelli I, Paparella G, Ferrazzano G, Angelini L, Giustini P, et al. Tremor distribution and the variable clinical presentation of essential tremor. Cerebellum. 2019;18:866–72. 10.1007/s12311-019-01070-0 31422549

[14] Jiménez-Jiménez FJ, Alonso-Navarro H, García-Martín E, Álvarez I, Pastor P, Agúndez JA. Genomic markers for essential tremor. Pharmaceuticals. 2021;14(6):516. 10.3390/ph14060516 PMC8226734 34072005

[15] Soto MCS, Fasano A. Essential tremor: new advances. Clin Park Relat Disord. 2020;3:100031. 10.1016/j.prdoa.2019.100031 PMC8298793 34316617

[16] Jiménez‐Jiménez F, Alonso‐Navarro H, García‐Martín E, Lorenzo‐Betancor O, Pastor P, Agúndez J. Update on genetics of essential tremor. Acta Neurol Scand. 2013;128(6):359–71. 10.1111/ane.12148 23682623

[17] Jain S, Lo SE, Louis ED. Common misdiagnosis of a common neurological disorder: how are we misdiagnosing essential tremor? Arch Neurol. 2006;63(8):1100–4. 10.1001/archneur.63.8.1100 16908735

[18] Schrag A, Münchau A, Bhatia K, Quinn N, Marsden C. Essential tremor: an overdiagnosed condition? J Neurol. 2000;247:955–9. 10.1007/s004150070053 11200689

[19] Bhatia KP, Bain P, Bajaj N, Elble RJ, Hallett M, Louis ED, et al. Consensus Statement on the classification of tremors. From the task force on tremor of the International Parkinson and Movement Disorder Society. Mov Disord. 2018;33(1):75–87. 10.1002/mds.27121 PMC6530552 29193359

[20] Pan M-K, Kuo S-H. Essential tremor: clinical perspectives and pathophysiology. J Neurol Sci. 2022;435:120198. 10.1016/j.jns.2022.120198 PMC10363990 35299120

[21] Wang Y, Yang J, Cai M, Liu X, Lu K, Lou Y, et al. Application of optimized convolutional neural networks for early aided diagnosis of essential tremor: automatic handwriting recognition and feature analysis. Med Eng Phys. 2023;113:103962. 10.1016/j.medengphy.2023.103962 36966002

[22] Louis ED. The evolving definition of essential tremor: what are we dealing with? Park Relat Disord. 2018;46:S87–91. 10.1016/j.parkreldis.2017.07.004 PMC5696078 28747280

[23] Lawrenson C, Bares M, Kamondi A, Kovács A, Lumb B, Apps R, et al. The mystery of the cerebellum: clues from experimental and clinical observations. Cerebellum Ataxias. 2018;5(1):1–11. 10.1186/s40673-018-0087-9 PMC5877388 29610671

[24] Pieczora L, Stracke L, Vorgerd M, Hahn S, Theiss C, Theis V. Unveiling of miRNA expression patterns in Purkinje cells during development. Cerebellum. 2017;16:376–87. 10.1007/s12311-016-0814-9 27387430

[25] Houle G, Ambalavanan A, Schmouth J-F, Leblond CS, Spiegelman D, Laurent SB, et al. No rare deleterious variants from STK32B, PPARGC1A, and CTNNA3 are associated with essential tremor. Neurol Genet. 2017;3(5):e195–7. 10.1212/NXG.0000000000000195 PMC6281551 30584593

[26] Delay C, Tremblay C, Brochu E, Paris‐Robidas S, Emond V, Rajput AH, et al. Increased LINGO1 in the cerebellum of essential tremor patients. Mov Disord. 2014;29(13):1637–47. 10.1002/mds.25819 24531928

[27] Kosmowska B, Ossowska K, Głowacka U, Wardas J. Tremorolytic effect of 5′‐chloro‐5′‐deoxy‐( ±)‐ENBA, a potent and selective adenosine A1 receptor agonist, evaluated in the harmaline‐induced model in rats. CNS Neurosci Ther. 2017;23(5):438–46. 10.1111/cns.12692 PMC6492700 28371468

[28] Batistela MS, Josviak ND, Sulzbach CD, de Souza RLR. An overview of circulating cell-free microRNAs as putative biomarkers in Alzheimer’s and Parkinson’s diseases. Int J Neurosci. 2017;127(6):547–58. 10.1080/00207454.2016.1209754 27381850

[29] Blauwendraat C, Nalls MA, Singleton AB. The genetic architecture of Parkinson’s disease. Lancet Neurol. 2020;19(2):170–8. 10.1016/S1474-4422(19)30287-X PMC8972299 31521533

[30] Chen H, Yang J, Wu W. Seven key hub genes identified by gene co-expression network in cutaneous squamous cell carcinoma. BMC Cancer. 2021;21:1–12. 10.1186/s12885-021-08604-y PMC8306372 34301206

[31] Fang KY, Liang GN, Zhuang ZQ, Fang YX, Dong YQ, Liang CJ, et al. Screening the hub genes and analyzing the mechanisms in discharged COVID‐19 patients retesting positive through bioinformatics analysis. J Clin Lab. 2022;36(7):e24495. 10.1002/jcla.24495 PMC9279949 35657140

[32] Moradi S, Tapak L, Afshar S. Identification of novel noninvasive diagnostics biomarkers in the Parkinson’s diseases and improving the disease classification using support vector machine. Biomed Res Int. 2022;2022:8. 10.1155/2022/5009892 PMC8941533 35342758

[33] Goñi M, Eickhoff SB, Far MS, Patil KR, Dukart J. Smartphone-based digital biomarkers for Parkinson’s disease in a remotely-administered setting. IEEE Access. 2022;10:28361–84.

[34] Wang Y, Huang X, Xian B, Jiang H, Zhou T, Chen S, et al. Machine learning and bioinformatics-based insights into the potential targets of saponins in Paris polyphylla smith against non-small cell lung cancer. Front Genet. 2022;3123:19. 10.3389/fgene.2022.1005896 PMC9649596 36386821

[35] Xiao F, Zuo Z, Cai G, Kang S, Gao X, Li T. miRecords: an integrated resource for microRNA–target interactions. Nucleic Acids Res. 2009;37(suppl_1):D105–10. 10.1093/nar/gkn851 PMC2686554 18996891

[36] Barrett T, Wilhite SE, Ledoux P, Evangelista C, Kim IF, Tomashevsky M, et al. NCBI GEO: archive for functional genomics data sets – update. Nucleic Acids Res. 2012;41(D1):D991–5. 10.1093/nar/gks1193 PMC3531084 23193258

[37] Bao Y, Wang L, Yu F, Yang J, Huang D. Parkinson’s disease gene biomarkers screened by the LASSO and SVM algorithms. Brain Sci. 2023;13(2):175. 10.3390/brainsci13020175 PMC9953979 36831718

[38] Huang D, Liu J, Cao Y, Wan L, Jiang H, Sun Y, et al. RNA sequencing for gene expression profiles in peripheral blood mononuclear cells with ankylosing spondylitis RNA. Biomed Res Int. 2020;2020:13. 10.1155/2020/5304578 PMC7298317 32596323

[39] Jankovic J. Parkinson’s disease and movement disorders: moving forward. Lancet Neurol. 2008;7(1):9–11. 10.1016/S1474-4422(07)70302-2 18093549

[40] Association WM. World medical association declaration of Helsinki. Ethical principles for medical research involving human subjects. Bull World Health Organ. 2001;79(4):373. PMC2566407 11357217

[41] Martuscello RT, Kerridge CA, Chatterjee D, Hartstone WG, Kuo S-H, Sims PA, et al. Gene expression analysis of the cerebellar cortex in essential tremor. Neurosci Lett. 2020;721:134540. 10.1016/j.neulet.2019.134540 PMC7593093 31707044

[42] Bindea G, Mlecnik B, Tosolini M, Kirilovsky A, Waldner M, Obenauf AC, et al. Spatiotemporal dynamics of intratumoral immune cells reveal the immune landscape in human cancer. Immunity. 2013;39(4):782–95. 10.1016/j.immuni.2013.10.003 24138885

[43] Landmark CJ. Antiepileptic drugs in non-epilepsy disorders: relations between mechanisms of action and clinical efficacy. CNS Drugs. 2008;22:27–47. 10.2165/00023210-200822010-00003 18072813

[44] Hampel H, Mesulam M-M, Cuello AC, Farlow MR, Giacobini E, Grossberg GT, et al. The cholinergic system in the pathophysiology and treatment of Alzheimer’s disease. Brain. 2018;141(7):1917–33. 10.1093/brain/awy132 PMC6022632 29850777

[45] Antonini A, Moresco R, Gobbo C, De Notaris R, Panzacchi A, Barone P, et al. The status of dopamine nerve terminals in Parkinson’s disease and essential tremor: a PET study with the tracer [11-C] FE-CIT. Neurol Sci. 2001;22:47–8. 10.1007/s100720170040 11487195

[46] Bang D, Kishida KT, Lohrenz T, White JP, Laxton AW, Tatter SB, et al. Sub-second dopamine and serotonin signaling in human striatum during perceptual decision-making. Neuron. 2020;108(5):999–1010.e6. 10.1016/j.neuron.2020.09.015 PMC7736619 33049201

[47] Elble RJ, Higgins C, Elble S. Electrophysiologic transition from physiologic tremor to essential tremor. Mov Disorders: J Mov Disord. 2005;20(8):1038–42. 10.1002/mds.20487 15852370

[48] Kavanagh JJ, Keogh JW. Correlates between force and postural tremor in older individuals with essential tremor. Cerebellum. 2016;15:688–95. 10.1007/s12311-015-0732-2 26490155

[49] Louis ED, Huang CC, Dyke JP, Long Z, Dydak U. Neuroimaging studies of essential tremor: how well do these studies support/refute the neurodegenerative hypothesis? Tremor Other Hyperkinet Mov. 2014;4:235–44. 10.7916/D8DF6PB8 PMC4038743 24918024

[50] Sharifi S, Nederveen AJ, Booij J, van Rootselaar A-F. Neuroimaging essentials in essential tremor: a systematic review. Neuroimage Clin. 2014;5:217–31. 10.1016/j.nicl.2014.05.003 PMC4110352 25068111

[51] Mavroudis I, Petridis F, Kazis D. Neuroimaging and neuropathological findings in essential tremor. Acta Neurol Scand. 2019;139(6):491–6. 10.1111/ane.13101 30977113

[52] Louis ED, Vonsattel JPG. The emerging neuropathology of essential tremor. Mov Disord. 2008;23(2):174–82. 10.1002/mds.21731 PMC2692583 17999421

[53] Louis ED, Yi H, Erickson-Davis C, Vonsattel J-PG, Faust PL. Structural study of Purkinje cell axonal torpedoes in essential tremor. Neurosci Lett. 2009;450(3):287–91. 10.1016/j.neulet.2008.11.043 PMC2662443 19047012

[54] Louis ED, Faust PL, Vonsattel JPG, Honig LS, Rajput A, Rajput A, et al. Torpedoes in Parkinson’s disease, Alzheimer’s disease, essential tremor, and control brains. J Mov Disord. 2009;24(11):1600–5. 10.1002/mds.22567 PMC2736313 19526585

[55] Louis ED, Lee M, Babij R, Ma K, Cortes E, Vonsattel J-PG, et al. Reduced Purkinje cell dendritic arborization and loss of dendritic spines in essential tremor. Brain. 2014;137(12):3142–8. 10.1093/brain/awu314 PMC4240305 25367027

[56] Louis ED, Faust PL, Vonsattel J-PG, Honig LS, Rajput A, Robinson CA, et al. Neuropathological changes in essential tremor: 33 cases compared with 21 controls. Brain. 2007;130(12):3297–307. 10.1093/brain/awm266 18025031

[57] Babij R, Lee M, Cortes E, Vonsattel J-PG, Faust PL, Louis ED. Purkinje cell axonal anatomy: quantifying morphometric changes in essential tremor versus control brains. Brain. 2013;136(10):3051–61. 10.1093/brain/awt238 PMC3784286 24030953

[58] Lee D, Gan S-R, Faust PL, Louis ED, Kuo S-H. Climbing fiber-Purkinje cell synaptic pathology across essential tremor subtypes. Park Relat Disord. 2018;51:24–9. 10.1016/j.parkreldis.2018.02.032 PMC6089250 29482925

[59] Zhang X, Santaniello S. Role of cerebellar GABAergic dysfunctions in the origins of essential tremor. Proc Natl Acad Sci. 2019;116(27):13592–601. 10.1073/pnas.1817689116 PMC6612915 31209041

[60] Marin-Lahoz J, Gironell A. Linking essential tremor to the cerebellum: neurochemical evidence. Cerebellum. 2016;15:243–52. 10.1007/s12311-015-0735-z 26498765

[61] Zhou M, Melin MD, Xu W, Südhof TC. Dysfunction of parvalbumin neurons in the cerebellar nuclei produces an action tremor. J Clin Investig. 2020;130(10):5142–56. 10.1172/JCI135802 PMC7524475 32634124

[62] Schaefer SM, Vives Rodriguez A, Louis ED. Brain circuits and neurochemical systems in essential tremor: insights into current and future pharmacotherapeutic approaches. Expert Rev Neurother. 2018;18(2):101–10. 10.1080/14737175.2018.1413353 29206482

[63] Yu M, Ma K, Faust PL, Honig LS, Cortés E, Vonsattel JP, et al. Increased number of Purkinje cell dendritic swellings in essential tremor. Eur J Neurol. 2012;19(4):625–30. 10.1111/j.1468-1331.2011.03598.x PMC3297734 22136494

[64] Filippini N, Ebmeier KP, MacIntosh BJ, Trachtenberg AJ, Frisoni GB, Wilcock G, et al. Differential effects of the APOE genotype on brain function across the lifespan. Neuroimage. 2011;54(1):602–10. 10.1016/j.neuroimage.2010.08.009 20705142

[65] Saunders A, Schmader K, Breitner J, Benson M, Brown W, Goldfarb L, et al. Apolipoprotein E epsilon 4 allele distributions in late-onset Alzheimer’s disease and in other amyloid-forming diseases. Lancet (London, England). 1993;342(8873):710–1. 10.1016/0140-6736(93)91709-u 8103823

[66] Béliveau E, Tremblay C, Aubry-Lafontaine É, Paris-Robidas S, Delay C, Robinson C, et al. Accumulation of amyloid-β in the cerebellar cortex of essential tremor patients. Neurobiol Dis. 2015;82:397–408. 10.1016/j.nbd.2015.07.016 26253607

[67] Wang Z, Qin W, Wei C, Tang Y, Zhao L, Jin H, et al. The microRNA‐1908 up‐regulation in the peripheral blood cells impairs amyloid clearance by targeting ApoE. Int J Geriatr Psychiatry. 2018;33(7):980–6. 10.1002/gps.4881 29635818

[68] Keiten-Schmitz J, Schunck K, Müller S. SUMO chains rule on chromatin occupancy. Front Cell Dev Biol. 2020;7:343. 10.3389/fcell.2019.00343 PMC6965010 31998715

[69] Colnaghi L, Conz A, Russo L, Musi CA, Fioriti L, Borsello T, et al. Neuronal localization of SENP proteins with super resolution microscopy. Brain Sci. 2020;10(11):778. 10.3390/brainsci10110778 PMC7693135 33113832

[70] Yan S-M, Wu H-N, He F, Hu X-P, Zhang Z-Y, Huang M-Y, et al. High expression of zinc-binding protein-89 predicts decreased survival in esophageal squamous cell cancer. Ann Thorac Surg. 2014;97(6):1966–73. 10.1016/j.athoracsur.2014.01.058 24698505

[71] Wang N, Li M-Y, Liu Y, Yu J, Ren J, Zheng Z, et al. ZBP-89 negatively regulates self-renewal of liver cancer stem cells via suppression of Notch1 signaling pathway. Cancer Lett. 2020;472:70–80. 10.1016/j.canlet.2019.12.026 PMC7226908 31874246

[72] Chandrasekaran S, Bonchev D. Network analysis of human post-mortem microarrays reveals novel genes, microRNAs, and mechanistic scenarios of potential importance in fighting Huntington’s disease. Comput Struct Biotechnol J. 2016;14:117–30. 10.1016/j.csbj.2016.02.001 PMC5128196 27924190

[73] Stevens SJ, van Essen AJ, van Ravenswaaij CM, Elias AF, Haven JA, Lelieveld SH, et al. Truncating de novo mutations in the Krüppel-type zinc-finger gene ZNF148 in patients with corpus callosum defects, developmental delay, short stature, and dysmorphisms. Genome Med. 2016;8:1–10. 10.1186/s13073-016-0386-9 PMC5155377 27964749

[74] Edwards TJ, Sherr EH, Barkovich AJ, Richards LJ. Clinical, genetic and imaging findings identify new causes for corpus callosum development syndromes. Brain. 2014;137(6):1579–613. 10.1093/brain/awt358 PMC4032094 24477430

[75] Liu D, Cao H, Kural KC, Fang Q, Zhang F. Integrative analysis of shared genetic pathogenesis by autism spectrum disorder and obsessive-compulsive disorder. Biosci Rep. 2019;39(12):9. 10.1042/BSR20191942 PMC6928520 31808517

[76] Aisa B, Gil-Bea FJ, Solas M, Garcia-Alloza M, Chen CP, Lai MK, et al. Altered NCAM expression associated with the cholinergic system in Alzheimer’s disease. J Alzheimer’s Dis. 2010;20(2):659–68. 10.3233/JAD-2010-1398 20164549

[77] Knoferle J, Yoon SY, Walker D, Leung L, Gillespie AK, Tong LM, et al. Apolipoprotein E4 produced in GABAergic interneurons causes learning and memory deficits in mice. J Neurosci Res. 2014;34(42):14069–78. 10.1523/JNEUROSCI.2281-14.2014 PMC4198545 25319703

[78] Huang Y, Mucke L. Alzheimer mechanisms and therapeutic strategies. Cell. 2012;148(6):1204–22. 10.1016/j.cell.2012.02.040 PMC3319071 22424230

[79] Fioravante D, Byrne JH. Protein degradation and memory formation. Brain Res Bull. 2011;85(1–2):14–20. 10.1016/j.brainresbull.2010.11.002 PMC3079012 21078374

[80] Hirunsatit R, George ED, Lipska BK, Elwafi HM, Sander L, Yrigollen CM, et al. Twenty-one-base-pair insertion polymorphism creates an enhancer element and potentiates SLC6A1 GABA transporter promoter activity. Pharmacogenet Genomics. 2009;19(1):53–65. 10.1097/FPC.0b013e328318b21a PMC2791799 19077666

[81] Muruzheva ZM, Ivleva IS, Traktirov DS, Zubov AS, Karpenko MN. The relationship between serum interleukin-1β, interleukin-6, interleukin-8, interleukin-10, tumor necrosis factor-α levels and clinical features in essential tremor. Int J Neurosci. 2022;132(11):1143–9. 10.1080/00207454.2020.1865952 33345671

[82] Shi T, Xie J, Xiong Y, Deng W, Guo J, Wang F, et al. Human HS1BP3 induces cell apoptosis and activates AP-1. BMB Rep. 2011;44(6):381–6. 10.5483/BMBRep.2011.44.6.381 21699750

[83] Gannushkina I, Zhirnova I, Toropova N, Toporkina T, Markova E. Quantitative evaluation of the T-and B-systems of immunity in various hereditary diseases of the nervous system. Zh Nevrol Psikhiatr im SS Korsakova. 1981;81(7):1009–13. 6974936

[84] Teijeira A, Garasa S, Ochoa MDC, Cirella A, Olivera I, Glez‐Vaz J, et al. Differential Interleukin‐8 thresholds for chemotaxis and netosis in human neutrophils. Eur J Immunol. 2021;51(9):2274–80. 10.1002/eji.202049029 33963542

[85] Giau VV, Wu SY, Jamerlan A, An SSA, Kim S, Hulme J. Gut microbiota and their neuroinflammatory implications in Alzheimer’s disease. Nutrients. 2018;10(11):1765. 10.3390/nu10111765 PMC6266223 30441866

[86] Kaur G, Behl T, Bungau S, Kumar A, Uddin MS, Mehta V, et al. Dysregulation of the gut–brain axis, dysbiosis and influence of numerous factors on gut microbiota associated Parkinson’s disease. Curr Neuropharmacol. 2021;19(2):233–47. 10.2174/1570159X18666200606233050 PMC8033978 32504503

[87] Agirman G, Hsiao EY. SnapShot: the microbiota–gut–brain axis. Cell. 2021;184(9):2524.e1. 10.1016/j.cell.2021.03.022 33930299

[88] Tudorache IF, Trusca VG, Gafencu AV. Apolipoprotein E – a multifunctional protein with implications in various pathologies as a result of its structural features. Comput Struct Biotechnol J. 2017;15:359–65. 10.1016/j.csbj.2017.05.003 PMC5476973 28660014

[89] Kockx M, Traini M, Kritharides L. Cell-specific production, secretion, and function of apolipoprotein E. J Mol Med. 2018;96:361–71. 10.1007/s00109-018-1632-y 29516132

[90] Li Q, Liu D, Pan F, Ho CS, Ho R. Ethanol exposure induces microglia activation and neuroinflammation through TLR4 activation and SENP6 modulation in the adolescent rat hippocampus. Neural Plast. 2019;2019:12. 10.1155/2019/1648736 PMC6874951 31781182

[91] Isogai S, Yamamoto N, Hiramatsu N, Goto Y, Hayashi M, Kondo M, et al. Preparation of induced pluripotent stem cells using human peripheral blood monocytes. Cell Reprogramming. 2018;20(6):347–55. 10.1089/cell.2018.0024 PMC6302673 31107605

