# Supplementary material for: Exploring the diagnostic markers of essential tremor: A study based on machine learning algorithms
Source: Open Life Sci. 2023 Jun 22;18(1):20220622. doi: 10.1515/biol-2022-0622 (PMC10290283; doi:10.1515/biol-2022-0622)
Supplement: Supplementary Figures [file biol-2022-0622-sm1.pdf]

# Supplementary material

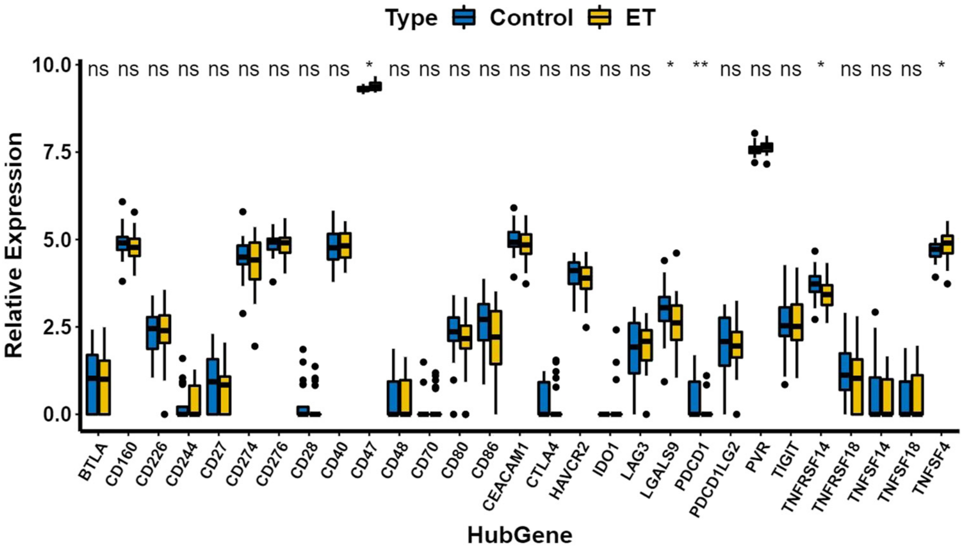


**Figure S1:** Identification of candidate ET-related DEGs.

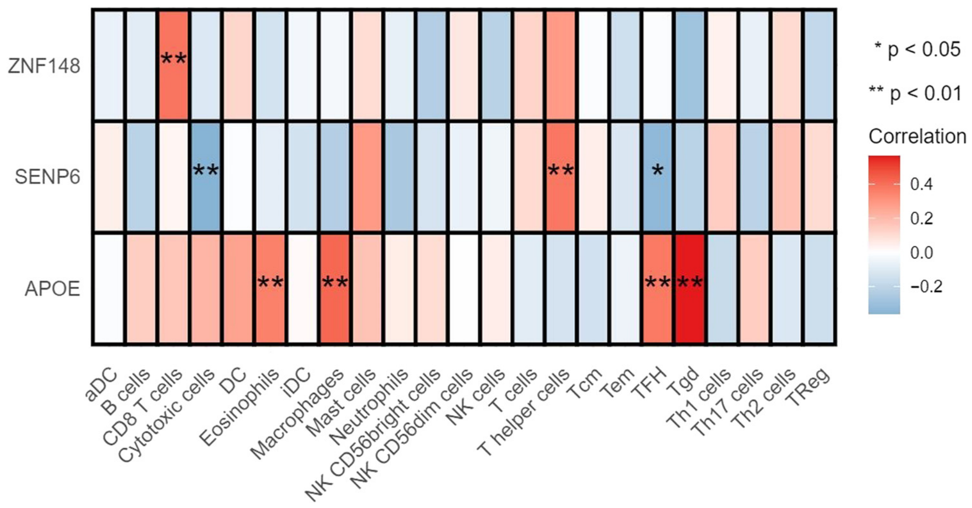


**Figure S2:** Immune landscape analysis of ET patients.

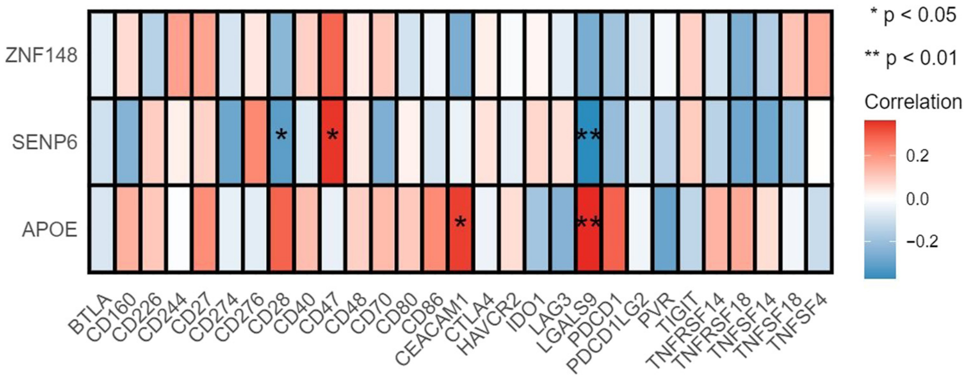


**Figure S3:** Functional enrichment analysis of ET-associated DEGs and diagnostic markers.
