# Supplementary material for: Exploring the diagnostic markers of essential tremor: A study based on machine learning algorithms
Source: Open Life Sci. 2023 Jun 22;18(1):20220622. doi: 10.1515/biol-2022-0622 (PMC10290283; doi:10.1515/biol-2022-0622)
Supplement: Supplementary Table 1 [file biol-2022-0622-sm2.pdf]

Table S1. Scales to assess ET patients and control groups

	ET 1	ET 2	ET 3	Control 1	Control 2	Control 3
FTMS A: position and severity	7	10	8	/	/	/
FTMS B: drawing/handwriting	7	11	9	/	/	/
FTMS C: disability	8	16	12	/	/	/
MoCA (score 0-30)	25	29	30	29	28	30
BDI (score 0-63)	8	16	10	2	3	3

MoCA (the Montreal Cognitive Assessment): ≥ 26 = normal;
BDI (the Beck Depression Inventory) functional scores range from: 0-4 = normal; 5-13 = mildly depression; 14-20 = moderately depression; > 21 = severely depression
