# Supplementary material for: Exploring the diagnostic markers of essential tremor: A study based on machine learning algorithms
Source: Open Life Sci. 2023 Jun 22;18(1):20220622. doi: 10.1515/biol-2022-0622 (PMC10290283; doi:10.1515/biol-2022-0622)
Supplement: Supplementary Table 2 [file biol-2022-0622-sm3.pdf]

Table S2: The expression pattern of these DEGs via a t-test assay

Symbol	Base Mean	log_2FoldC	lfcSE	stat	p-value	p-adj
LOC10013	0	NA	NA	NA	NA	NA
USP17L30	0	NA	NA	NA	NA	NA
RNU6-16	0	NA	NA	NA	NA	NA
PHOX2B	0	NA	NA	NA	NA	NA
PISRT1	0	NA	NA	NA	NA	NA
C4orf17	0	NA	NA	NA	NA	NA
KRTAP2-3	0	NA	NA	NA	NA	NA
KRTAP2-2	0	NA	NA	NA	NA	NA
KRTAP2-1	0	NA	NA	NA	NA	NA
KRTAP2-4	0	NA	NA	NA	NA	NA
MIR4708	0	NA	NA	NA	NA	NA
MIR4709	0	NA	NA	NA	NA	NA
MIR4707	0	NA	NA	NA	NA	NA
MIR4700	0	NA	NA	NA	NA	NA
MIR3169	0	NA	NA	NA	NA	NA
INO80B-W	0	NA	NA	NA	NA	NA
HOXA11	0	NA	NA	NA	NA	NA
HOXA10	0	NA	NA	NA	NA	NA
LRRC24	0	NA	NA	NA	NA	NA
CCDC172	0	NA	NA	NA	NA	NA
MIR193B	0	NA	NA	NA	NA	NA
LOC10050	0	NA	NA	NA	NA	NA
INMT-FAM	0	NA	NA	NA	NA	NA
OR5R1	0	NA	NA	NA	NA	NA
GOLGA8G	0	NA	NA	NA	NA	NA
GOLGA8F	0	NA	NA	NA	NA	NA
CT45A1	0	NA	NA	NA	NA	NA
CT45A2	0	NA	NA	NA	NA	NA
CT45A4	0	NA	NA	NA	NA	NA
CT45A6	0	NA	NA	NA	NA	NA
GATA3-AS	0	NA	NA	NA	NA	NA
ERCC-0005	0	NA	NA	NA	NA	NA
ERCC-0005	0	NA	NA	NA	NA	NA
ERCC-0005	0	NA	NA	NA	NA	NA
ERCC-0005	0	NA	NA	NA	NA	NA
OR1S1	0	NA	NA	NA	NA	NA
OR1S2	0	NA	NA	NA	NA	NA
TRIML2	0	NA	NA	NA	NA	NA
TRIML1	0	NA	NA	NA	NA	NA
FOXN1	0	NA	NA	NA	NA	NA
UGT2A1	0	NA	NA	NA	NA	NA
DUX4L7	0	NA	NA	NA	NA	NA
DUX4L6	0	NA	NA	NA	NA	NA
DUX4L5	0	NA	NA	NA	NA	NA
DUX4L4	0	NA	NA	NA	NA	NA
DUX4L3	0	NA	NA	NA	NA	NA

DUX4L2	0	NA	NA	NA	NA	NA
TSPY8	0	NA	NA	NA	NA	NA
MIR4773-2	0	NA	NA	NA	NA	NA
MIR4773-1	0	NA	NA	NA	NA	NA
TSPY2	0	NA	NA	NA	NA	NA
TSPY1	0	NA	NA	NA	NA	NA
SLC17A2	0	NA	NA	NA	NA	NA
TRIM49D2	0	NA	NA	NA	NA	NA
HOXB13	0	NA	NA	NA	NA	NA
TEX28	0	NA	NA	NA	NA	NA
HSFX2	0	NA	NA	NA	NA	NA
HSFX1	0	NA	NA	NA	NA	NA
SFTA3	0	NA	NA	NA	NA	NA
SFTA2	0	NA	NA	NA	NA	NA
KRTAP1-4	0	NA	NA	NA	NA	NA
CSHL1	0	NA	NA	NA	NA	NA
AGXT	0	NA	NA	NA	NA	NA
HOXA11-A	0	NA	NA	NA	NA	NA
OR51F2	0	NA	NA	NA	NA	NA
MIR1-1	0	NA	NA	NA	NA	NA
GAGE1	0	NA	NA	NA	NA	NA
GAGE5	0	NA	NA	NA	NA	NA
GAGE6	0	NA	NA	NA	NA	NA
LOC60672	0	NA	NA	NA	NA	NA
MIR1972-2	0	NA	NA	NA	NA	NA
MIR1972-1	0	NA	NA	NA	NA	NA
ADAM18	0	NA	NA	NA	NA	NA
RNU6-81	0	NA	NA	NA	NA	NA
RPL13AP5	0	NA	NA	NA	NA	NA
OR2T34	0	NA	NA	NA	NA	NA
OR2T35	0	NA	NA	NA	NA	NA
MIR147B	0	NA	NA	NA	NA	NA
MIR1471	0	NA	NA	NA	NA	NA
OR4A47	0	NA	NA	NA	NA	NA
MIR936	0	NA	NA	NA	NA	NA
MIR937	0	NA	NA	NA	NA	NA
MIR935	0	NA	NA	NA	NA	NA
MIR5583-2	0	NA	NA	NA	NA	NA
MIR5583-1	0	NA	NA	NA	NA	NA
MIR939	0	NA	NA	NA	NA	NA
CPN1	0	NA	NA	NA	NA	NA
IL21	0	NA	NA	NA	NA	NA
IL20	0	NA	NA	NA	NA	NA
IL26	0	NA	NA	NA	NA	NA
MLIP-IT1	0	NA	NA	NA	NA	NA
OR8J3	0	NA	NA	NA	NA	NA
OR8J1	0	NA	NA	NA	NA	NA

OR51A2	0	NA	NA	NA	NA	NA
REG1A	0	NA	NA	NA	NA	NA
REG1B	0	NA	NA	NA	NA	NA
REG1P	0	NA	NA	NA	NA	NA
ABHD14A-	0	NA	NA	NA	NA	NA
SERPINB3	0	NA	NA	NA	NA	NA
SERPINB5	0	NA	NA	NA	NA	NA
SPATA16	0	NA	NA	NA	NA	NA
TRIM53AP	0	NA	NA	NA	NA	NA
MIR4268	0	NA	NA	NA	NA	NA
MIR4261	0	NA	NA	NA	NA	NA
MIR4260	0	NA	NA	NA	NA	NA
MIR4267	0	NA	NA	NA	NA	NA
MIR4266	0	NA	NA	NA	NA	NA
OR5J2	0	NA	NA	NA	NA	NA
LINC00633	0	NA	NA	NA	NA	NA
BIVM-ERC	0	NA	NA	NA	NA	NA
OR4L1	0	NA	NA	NA	NA	NA
TYR	0	NA	NA	NA	NA	NA
INSL6	0	NA	NA	NA	NA	NA
PDX1	0	NA	NA	NA	NA	NA
MIR5692A	0	NA	NA	NA	NA	NA
GPR89C	0	NA	NA	NA	NA	NA
MIR3591	0	NA	NA	NA	NA	NA
KRT33B	0	NA	NA	NA	NA	NA
KRT33A	0	NA	NA	NA	NA	NA
LOC10012	0	NA	NA	NA	NA	NA
DEFB130	0	NA	NA	NA	NA	NA
DEFB131	0	NA	NA	NA	NA	NA
DEFB133	0	NA	NA	NA	NA	NA
KRTAP10-7	0	NA	NA	NA	NA	NA
KRTAP10-6	0	NA	NA	NA	NA	NA
KRTAP10-3	0	NA	NA	NA	NA	NA
KRTAP10-2	0	NA	NA	NA	NA	NA
MIR770	0	NA	NA	NA	NA	NA
MIR483	0	NA	NA	NA	NA	NA
MIR489	0	NA	NA	NA	NA	NA
ERCC-0015	0	NA	NA	NA	NA	NA
ERCC-0015	0	NA	NA	NA	NA	NA
ERCC-0015	0	NA	NA	NA	NA	NA
ERCC-0015	0	NA	NA	NA	NA	NA
ERCC-0015	0	NA	NA	NA	NA	NA
NXF2B	0	NA	NA	NA	NA	NA
ATXN3L	0	NA	NA	NA	NA	NA
LEUTX	0	NA	NA	NA	NA	NA
LOC10013	0	NA	NA	NA	NA	NA
HTN3	0	NA	NA	NA	NA	NA

FAM45A	0	NA	NA	NA	NA	NA
SPRR4	0	NA	NA	NA	NA	NA
SPRR3	0	NA	NA	NA	NA	NA
PFN3	0	NA	NA	NA	NA	NA
MIR4632	0	NA	NA	NA	NA	NA
MIR4633	0	NA	NA	NA	NA	NA
CES1P2	0	NA	NA	NA	NA	NA
MIR3945	0	NA	NA	NA	NA	NA
SATB2-AS1	0	NA	NA	NA	NA	NA
RFPL4A	0	NA	NA	NA	NA	NA
LYPD4	0	NA	NA	NA	NA	NA
MIR523	0	NA	NA	NA	NA	NA
MIR522	0	NA	NA	NA	NA	NA
MIR525	0	NA	NA	NA	NA	NA
MIR524	0	NA	NA	NA	NA	NA
OR4F29	0	NA	NA	NA	NA	NA
CT45A3	0	NA	NA	NA	NA	NA
OR8B2	0	NA	NA	NA	NA	NA
OR8B4	0	NA	NA	NA	NA	NA
DEFA1B	0	NA	NA	NA	NA	NA
FAM27L	0	NA	NA	NA	NA	NA
LHX8	0	NA	NA	NA	NA	NA
WT1-AS	0	NA	NA	NA	NA	NA
MGC2738	0	NA	NA	NA	NA	NA
ERCC-0005	0	NA	NA	NA	NA	NA
SNORD125	0	NA	NA	NA	NA	NA
NOBOX	0	NA	NA	NA	NA	NA
PGCP1	0	NA	NA	NA	NA	NA
OR4D6	0	NA	NA	NA	NA	NA
HOXA6	0	NA	NA	NA	NA	NA
HOXA9	0	NA	NA	NA	NA	NA
PRTN3	0	NA	NA	NA	NA	NA
CTSG	0	NA	NA	NA	NA	NA
LOC64466	0	NA	NA	NA	NA	NA
LOC10050	0	NA	NA	NA	NA	NA
OR14J1	0	NA	NA	NA	NA	NA
RNU6-6	0	NA	NA	NA	NA	NA
LOC64344	0	NA	NA	NA	NA	NA
C9orf62	0	NA	NA	NA	NA	NA
RPTN	0	NA	NA	NA	NA	NA
MIR4509-2	0	NA	NA	NA	NA	NA
MIR4509-3	0	NA	NA	NA	NA	NA
MIR4509-1	0	NA	NA	NA	NA	NA
FAM223A	0	NA	NA	NA	NA	NA
MIR4309	0	NA	NA	NA	NA	NA
MIR4302	0	NA	NA	NA	NA	NA
MIR4307	0	NA	NA	NA	NA	NA

MIR3656	0	NA	NA	NA	NA	NA
MIR3655	0	NA	NA	NA	NA	NA
MIR3654	0	NA	NA	NA	NA	NA
LINC00489	0	NA	NA	NA	NA	NA
MIR3652	0	NA	NA	NA	NA	NA
MIR3651	0	NA	NA	NA	NA	NA
DEFA6	0	NA	NA	NA	NA	NA
DEFA5	0	NA	NA	NA	NA	NA
DEFA3	0	NA	NA	NA	NA	NA
DEFA1	0	NA	NA	NA	NA	NA
NUP210P1	0	NA	NA	NA	NA	NA
MAGED4B	0	NA	NA	NA	NA	NA
GSTA5	0	NA	NA	NA	NA	NA
GSTA1	0	NA	NA	NA	NA	NA
GSTA2	0	NA	NA	NA	NA	NA
GSTA3	0	NA	NA	NA	NA	NA
DMRT3	0	NA	NA	NA	NA	NA
CCL4L1	0	NA	NA	NA	NA	NA
CCL4L2	0	NA	NA	NA	NA	NA
FCRL2	0	NA	NA	NA	NA	NA
FCRL4	0	NA	NA	NA	NA	NA
PNMA6A	0	NA	NA	NA	NA	NA
CHIAP2	0	NA	NA	NA	NA	NA
PI3	0	NA	NA	NA	NA	NA
FMO9P	0	NA	NA	NA	NA	NA
MIR1257	0	NA	NA	NA	NA	NA
TSPY4	0	NA	NA	NA	NA	NA
TSPY3	0	NA	NA	NA	NA	NA
CRYBA4	0	NA	NA	NA	NA	NA
LINC00523	0	NA	NA	NA	NA	NA
MC3R	0	NA	NA	NA	NA	NA
MIR548AA	0	NA	NA	NA	NA	NA
CLDN10-A	0	NA	NA	NA	NA	NA
DEFA9P	0	NA	NA	NA	NA	NA
MGC3958	0	NA	NA	NA	NA	NA
H3F3AP4	0	NA	NA	NA	NA	NA
RNA5SP24	0	NA	NA	NA	NA	NA
IL9R	0	NA	NA	NA	NA	NA
TAAR1	0	NA	NA	NA	NA	NA
TAAR2	0	NA	NA	NA	NA	NA
SPANXA1	0	NA	NA	NA	NA	NA
SPANXA2	0	NA	NA	NA	NA	NA
FLJ20518	0	NA	NA	NA	NA	NA
MIR4485	0	NA	NA	NA	NA	NA
MIR4483	0	NA	NA	NA	NA	NA
ABCC6P2	0	NA	NA	NA	NA	NA
OR51S1	0	NA	NA	NA	NA	NA

C8A	0	NA	NA	NA	NA	NA
HLA-DRB3	0	NA	NA	NA	NA	NA
MTPN	0	NA	NA	NA	NA	NA
RNU6-57	0	NA	NA	NA	NA	NA
CHRNA9	0	NA	NA	NA	NA	NA
AQP12A	0	NA	NA	NA	NA	NA
LINC00317	0	NA	NA	NA	NA	NA
ERCC-0002	0	NA	NA	NA	NA	NA
ERCC-0002	0	NA	NA	NA	NA	NA
ERCC-0002	0	NA	NA	NA	NA	NA
ERCC-0002	0	NA	NA	NA	NA	NA
IL3RA	0	NA	NA	NA	NA	NA
ARHGEF38	0	NA	NA	NA	NA	NA
LOC39070	0	NA	NA	NA	NA	NA
TRIM6-TRI	0	NA	NA	NA	NA	NA
RBAK-LOC	0	NA	NA	NA	NA	NA
TRIM49D1	0	NA	NA	NA	NA	NA
SST	0	NA	NA	NA	NA	NA
SYNJ2BP-C	0	NA	NA	NA	NA	NA
LOC72784	0	NA	NA	NA	NA	NA
MIR155	0	NA	NA	NA	NA	NA
LOC14474	0	NA	NA	NA	NA	NA
MIR514A1	0	NA	NA	NA	NA	NA
MIR514A3	0	NA	NA	NA	NA	NA
MIR514A2	0	NA	NA	NA	NA	NA
GTF2A1L	0	NA	NA	NA	NA	NA
LACRT	0	NA	NA	NA	NA	NA
AADAC	0	NA	NA	NA	NA	NA
C19orf33	0	NA	NA	NA	NA	NA
KLK15	0	NA	NA	NA	NA	NA
MIR218-1	0	NA	NA	NA	NA	NA
PEG3-AS1	0	NA	NA	NA	NA	NA
ANKRD20A	0	NA	NA	NA	NA	NA
ANKRD20A	0	NA	NA	NA	NA	NA
OR11A1	0	NA	NA	NA	NA	NA
TMEM110	0	NA	NA	NA	NA	NA
CDRT7	0	NA	NA	NA	NA	NA
C17orf112	0	NA	NA	NA	NA	NA
KRTAP1-1	0	NA	NA	NA	NA	NA
KRTAP1-5	0	NA	NA	NA	NA	NA
TMED7-TI	0	NA	NA	NA	NA	NA
MIR2113	0	NA	NA	NA	NA	NA
MIR2110	0	NA	NA	NA	NA	NA
MIR2114	0	NA	NA	NA	NA	NA
LOC14908	0	NA	NA	NA	NA	NA
MIR5681B	0	NA	NA	NA	NA	NA
SNAR-H	0	NA	NA	NA	NA	NA

MIR516B2	0	NA	NA	NA	NA	NA
MIR516B1	0	NA	NA	NA	NA	NA
LOC28478	0	NA	NA	NA	NA	NA
KRT6B	0	NA	NA	NA	NA	NA
KRT6A	0	NA	NA	NA	NA	NA
BPY2B	0	NA	NA	NA	NA	NA
BPY2C	0	NA	NA	NA	NA	NA
ISL1	0	NA	NA	NA	NA	NA
MS4A5	0	NA	NA	NA	NA	NA
DNTT	0	NA	NA	NA	NA	NA
MIR4775	0	NA	NA	NA	NA	NA
MIR3201	0	NA	NA	NA	NA	NA
CUZD1	0	NA	NA	NA	NA	NA
CCL11	0	NA	NA	NA	NA	NA
CCL13	0	NA	NA	NA	NA	NA
CCL14	0	NA	NA	NA	NA	NA
CCL15	0	NA	NA	NA	NA	NA
CCL18	0	NA	NA	NA	NA	NA
SERPINA9	0	NA	NA	NA	NA	NA
GAGE13	0	NA	NA	NA	NA	NA
PRR23A	0	NA	NA	NA	NA	NA
ERCC-0014	0	NA	NA	NA	NA	NA
ERCC-0014	0	NA	NA	NA	NA	NA
ERCC-0014	0	NA	NA	NA	NA	NA
MIR548F4	0	NA	NA	NA	NA	NA
LOC10050	0	NA	NA	NA	NA	NA
OR5W2	0	NA	NA	NA	NA	NA
RBMY1J	0	NA	NA	NA	NA	NA
MIR4465	0	NA	NA	NA	NA	NA
SLX1A-SUL	0	NA	NA	NA	NA	NA
RBMY1D	0	NA	NA	NA	NA	NA
MIR4524A	0	NA	NA	NA	NA	NA
MUCL1	0	NA	NA	NA	NA	NA
SCGB1D1	0	NA	NA	NA	NA	NA
SCGB1D4	0	NA	NA	NA	NA	NA
CDY1	0	NA	NA	NA	NA	NA
C16orf82	0	NA	NA	NA	NA	NA
MS4A8B	0	NA	NA	NA	NA	NA
GAGE8	0	NA	NA	NA	NA	NA
OR52J3	0	NA	NA	NA	NA	NA
GAGE4	0	NA	NA	NA	NA	NA
LOC10012	0	NA	NA	NA	NA	NA
MIR3910-1	0	NA	NA	NA	NA	NA
MIR3910-2	0	NA	NA	NA	NA	NA
SLC25A3P	0	NA	NA	NA	NA	NA
RSPH10B	0	NA	NA	NA	NA	NA
CGB8	0	NA	NA	NA	NA	NA

CGB5	0	NA	NA	NA	NA	NA
CGB7	0	NA	NA	NA	NA	NA
CGB1	0	NA	NA	NA	NA	NA
MIR1323	0	NA	NA	NA	NA	NA
LOC10012	0	NA	NA	NA	NA	NA
MIR3199-1	0	NA	NA	NA	NA	NA
MIR3199-2	0	NA	NA	NA	NA	NA
NEDD8-M	0	NA	NA	NA	NA	NA
SMR3B	0	NA	NA	NA	NA	NA
SMR3A	0	NA	NA	NA	NA	NA
TRIM51	0	NA	NA	NA	NA	NA
ERCC-0010	0	NA	NA	NA	NA	NA
ERCC-0010	0	NA	NA	NA	NA	NA
ERCC-0010	0	NA	NA	NA	NA	NA
ALDH3B2	0	NA	NA	NA	NA	NA
C10orf32-	0	NA	NA	NA	NA	NA
HTN1	0	NA	NA	NA	NA	NA
OR10Z1	0	NA	NA	NA	NA	NA
APOC4	0	NA	NA	NA	NA	NA
APOC2	0	NA	NA	NA	NA	NA
MIR3918	0	NA	NA	NA	NA	NA
SPDYE2L	0	NA	NA	NA	NA	NA
PAGE5	0	NA	NA	NA	NA	NA
PAGE1	0	NA	NA	NA	NA	NA
MIR4641	0	NA	NA	NA	NA	NA
MIR4647	0	NA	NA	NA	NA	NA
LINC00297	0	NA	NA	NA	NA	NA
LINC00293	0	NA	NA	NA	NA	NA
MIR211	0	NA	NA	NA	NA	NA
MIR217	0	NA	NA	NA	NA	NA
MIR214	0	NA	NA	NA	NA	NA
OR4A16	0	NA	NA	NA	NA	NA
OR4A15	0	NA	NA	NA	NA	NA
LOC64242	0	NA	NA	NA	NA	NA
MIR3198-2	0	NA	NA	NA	NA	NA
LOC64364	0	NA	NA	NA	NA	NA
MIR1260A	0	NA	NA	NA	NA	NA
MIR1260B	0	NA	NA	NA	NA	NA
MIR1282	0	NA	NA	NA	NA	NA
MIR4436A	0	NA	NA	NA	NA	NA
SPATA8	0	NA	NA	NA	NA	NA
SPATA3	0	NA	NA	NA	NA	NA
OR5D13	0	NA	NA	NA	NA	NA
OR5D14	0	NA	NA	NA	NA	NA
OR5D16	0	NA	NA	NA	NA	NA
PYURF	0	NA	NA	NA	NA	NA
HMHB1	0	NA	NA	NA	NA	NA

ERCC-0009	0	NA	NA	NA	NA	NA
USP17L21	0	NA	NA	NA	NA	NA
MIR1295A	0	NA	NA	NA	NA	NA
MIR1295B	0	NA	NA	NA	NA	NA
HOXD9	0	NA	NA	NA	NA	NA
HOXD4	0	NA	NA	NA	NA	NA
HSPE1-MO	0	NA	NA	NA	NA	NA
MIR135A1	0	NA	NA	NA	NA	NA
TMEM207	0	NA	NA	NA	NA	NA
TTTY3B	0	NA	NA	NA	NA	NA
DEFB4B	0	NA	NA	NA	NA	NA
DEFB4A	0	NA	NA	NA	NA	NA
PRR9	0	NA	NA	NA	NA	NA
ADAM6	0	NA	NA	NA	NA	NA
GOLGA6D	0	NA	NA	NA	NA	NA
UGT2B15	0	NA	NA	NA	NA	NA
UGT2B10	0	NA	NA	NA	NA	NA
UGT2B11	0	NA	NA	NA	NA	NA
GML	0	NA	NA	NA	NA	NA
OR1M1	0	NA	NA	NA	NA	NA
TTTY17A	0	NA	NA	NA	NA	NA
TTTY17B	0	NA	NA	NA	NA	NA
TTTY17C	0	NA	NA	NA	NA	NA
BPIFB3	0	NA	NA	NA	NA	NA
BPIFB2	0	NA	NA	NA	NA	NA
BPIFB6	0	NA	NA	NA	NA	NA
BPIFB4	0	NA	NA	NA	NA	NA
RNASE12	0	NA	NA	NA	NA	NA
C9orf53	0	NA	NA	NA	NA	NA
SNAR-G2	0	NA	NA	NA	NA	NA
FAM74A4	0	NA	NA	NA	NA	NA
FAM74A1	0	NA	NA	NA	NA	NA
FAM74A2	0	NA	NA	NA	NA	NA
MIR4532	0	NA	NA	NA	NA	NA
MIR499B	0	NA	NA	NA	NA	NA
PTPN20B	0	NA	NA	NA	NA	NA
PTPN20A	0	NA	NA	NA	NA	NA
LOC72767	0	NA	NA	NA	NA	NA
HDGFL1	0	NA	NA	NA	NA	NA
MIR18B	0	NA	NA	NA	NA	NA
MIR18A	0	NA	NA	NA	NA	NA
MIR187	0	NA	NA	NA	NA	NA
MIR183	0	NA	NA	NA	NA	NA
LCN1	0	NA	NA	NA	NA	NA
MIA-RAB4	0	NA	NA	NA	NA	NA
PRAMEF18	0	NA	NA	NA	NA	NA
PRAMEF19	0	NA	NA	NA	NA	NA

PRAMEF16	0	NA	NA	NA	NA	NA
PRAMEF17	0	NA	NA	NA	NA	NA
PRAMEF14	0	NA	NA	NA	NA	NA
PRAMEF15	0	NA	NA	NA	NA	NA
PRAMEF12	0	NA	NA	NA	NA	NA
PRAMEF13	0	NA	NA	NA	NA	NA
PRAMEF10	0	NA	NA	NA	NA	NA
PRAMEF11	0	NA	NA	NA	NA	NA
GUCY2GP	0	NA	NA	NA	NA	NA
CST13P	0	NA	NA	NA	NA	NA
ACTR3BP2	0	NA	NA	NA	NA	NA
ACTR3BP5	0	NA	NA	NA	NA	NA
OR5H15	0	NA	NA	NA	NA	NA
OR5H14	0	NA	NA	NA	NA	NA
MIR153-2	0	NA	NA	NA	NA	NA
MIR550A1	0	NA	NA	NA	NA	NA
MIR550A2	0	NA	NA	NA	NA	NA
OR7E24	0	NA	NA	NA	NA	NA
OR10R2	0	NA	NA	NA	NA	NA
NUDT4P1	0	NA	NA	NA	NA	NA
LOC28468	0	NA	NA	NA	NA	NA
GCOM1	0	NA	NA	NA	NA	NA
FLJ26245	0	NA	NA	NA	NA	NA
LOC10110	0	NA	NA	NA	NA	NA
DEFB107B	0	NA	NA	NA	NA	NA
DEFB107A	0	NA	NA	NA	NA	NA
MIR2277	0	NA	NA	NA	NA	NA
OR2V1	0	NA	NA	NA	NA	NA
LOC10028	0	NA	NA	NA	NA	NA
PRSS2	0	NA	NA	NA	NA	NA
SPATA31A	0	NA	NA	NA	NA	NA
LOC64933	0	NA	NA	NA	NA	NA
KNCN	0	NA	NA	NA	NA	NA
SPANXN5	0	NA	NA	NA	NA	NA
SPANXN4	0	NA	NA	NA	NA	NA
SPANXN2	0	NA	NA	NA	NA	NA
SPANXD	0	NA	NA	NA	NA	NA
SPANXE	0	NA	NA	NA	NA	NA
C17orf64	0	NA	NA	NA	NA	NA
OR2M7	0	NA	NA	NA	NA	NA
MIR4458	0	NA	NA	NA	NA	NA
MIR4453	0	NA	NA	NA	NA	NA
MIR4454	0	NA	NA	NA	NA	NA
MIR4456	0	NA	NA	NA	NA	NA
MIR4457	0	NA	NA	NA	NA	NA
CTAG1A	0	NA	NA	NA	NA	NA
CTAG1B	0	NA	NA	NA	NA	NA

RSPH10B2	0	NA	NA	NA	NA	NA
TNXA	0	NA	NA	NA	NA	NA
FATE1	0	NA	NA	NA	NA	NA
FAM133D	0	NA	NA	NA	NA	NA
SERPINB13	0	NA	NA	NA	NA	NA
SERPINB12	0	NA	NA	NA	NA	NA
GSTT2B	0	NA	NA	NA	NA	NA
HNRNPA1	0	NA	NA	NA	NA	NA
FAM187B	0	NA	NA	NA	NA	NA
OR8B12	0	NA	NA	NA	NA	NA
FLJ46446	0	NA	NA	NA	NA	NA
ZNRF4	0	NA	NA	NA	NA	NA
MIR34C	0	NA	NA	NA	NA	NA
MIR34B	0	NA	NA	NA	NA	NA
SLMO2-AT	0	NA	NA	NA	NA	NA
C11orf52	0	NA	NA	NA	NA	NA
USP17L28	0	NA	NA	NA	NA	NA
CST8	0	NA	NA	NA	NA	NA
CST9	0	NA	NA	NA	NA	NA
CST2	0	NA	NA	NA	NA	NA
CST1	0	NA	NA	NA	NA	NA
CST4	0	NA	NA	NA	NA	NA
CST5	0	NA	NA	NA	NA	NA
SUMO1P1	0	NA	NA	NA	NA	NA
HOXB-AS5	0	NA	NA	NA	NA	NA
KRTAP20-2	0	NA	NA	NA	NA	NA
KRTAP20-3	0	NA	NA	NA	NA	NA
KRTAP20-1	0	NA	NA	NA	NA	NA
MIR3606	0	NA	NA	NA	NA	NA
DYDC1	0	NA	NA	NA	NA	NA
MIR10B	0	NA	NA	NA	NA	NA
MIR10A	0	NA	NA	NA	NA	NA
EDN2	0	NA	NA	NA	NA	NA
OPN1MW	0	NA	NA	NA	NA	NA
AZGP1P1	0	NA	NA	NA	NA	NA
SLC13A2	0	NA	NA	NA	NA	NA
IGFL1	0	NA	NA	NA	NA	NA
OR10J5	0	NA	NA	NA	NA	NA
OR10J3	0	NA	NA	NA	NA	NA
GTF2IP1	0	NA	NA	NA	NA	NA
MIR103B1	0	NA	NA	NA	NA	NA
MIR103B2	0	NA	NA	NA	NA	NA
OR6P1	0	NA	NA	NA	NA	NA
INE2	0	NA	NA	NA	NA	NA
KERA	0	NA	NA	NA	NA	NA
HOXD-AS2	0	NA	NA	NA	NA	NA
MIR182	0	NA	NA	NA	NA	NA

MIR548AJ	*0*	*NA*	*NA*	*NA*	*NA*	*NA*
CSF2RA	0	NA	NA	NA	NA	*NA*
KRTAP24-1	0	NA	NA	NA	NA	*NA*
OR8U8	0	NA	NA	NA	NA	*NA*
OR8U1	0	NA	NA	NA	NA	*NA*
PPIAL4A	0	NA	NA	NA	NA	*NA*
KRTAP19-3	0	NA	NA	NA	NA	*NA*
PPIAL4C	0	NA	NA	NA	NA	*NA*
PPIAL4B	0	NA	NA	NA	NA	*NA*
PPIAL4E	0	NA	NA	NA	NA	*NA*
PPIAL4D	0	NA	NA	NA	NA	*NA*
PPIAL4F	0	NA	NA	NA	NA	*NA*
TTTY23B	0	NA	NA	NA	NA	*NA*
RNU6-72	0	NA	NA	NA	NA	*NA*
LOC38904	0	NA	NA	NA	NA	*NA*
SPANXF1	0	NA	NA	NA	NA	*NA*
MIR4724	0	NA	NA	NA	NA	*NA*
DAZ3	0	NA	NA	NA	NA	*NA*
DAZ1	0	NA	NA	NA	NA	*NA*
DAZ4	0	NA	NA	NA	NA	*NA*
LINC00442	0	NA	NA	NA	NA	*NA*
MIR3185	0	NA	NA	NA	NA	*NA*
MIR3184	0	NA	NA	NA	NA	*NA*
MIR3182	0	NA	NA	NA	NA	*NA*
CCL23	0	NA	NA	NA	NA	*NA*
FAM230C	0	NA	NA	NA	NA	*NA*
LOC44102	0	NA	NA	NA	NA	*NA*
MYF5	0	NA	NA	NA	NA	*NA*
MYF6	0	NA	NA	NA	NA	*NA*
MIR548AX	0	NA	NA	NA	NA	*NA*
MIR548AS	0	NA	NA	NA	NA	*NA*
MIR548AQ	0	NA	NA	NA	NA	*NA*
EVX1	0	NA	NA	NA	NA	*NA*
MIR548A1	0	NA	NA	NA	NA	*NA*
OR5T1	0	NA	NA	NA	NA	*NA*
OR5T3	0	NA	NA	NA	NA	*NA*
OR5T2	0	NA	NA	NA	NA	*NA*
MAGEA2B	0	NA	NA	NA	NA	*NA*
MIR1912	0	NA	NA	NA	NA	*NA*
MIR1911	0	NA	NA	NA	NA	*NA*
FDCSP	0	NA	NA	NA	NA	*NA*
MIR615	0	NA	NA	NA	NA	*NA*
MIR614	0	NA	NA	NA	NA	*NA*
MIR610	0	NA	NA	NA	NA	*NA*
RPL36A-H	0	NA	NA	NA	NA	*NA*
LINC00343	0	NA	NA	NA	NA	*NA*
LINC00348	*0*	*NA*	*NA*	*NA*	*NA*	*NA*

ERCC-0007	0	NA	NA	NA	NA	NA
ERCC-0007	0	NA	NA	NA	NA	NA
ERCC-0007	0	NA	NA	NA	NA	NA
CENPVP2	0	NA	NA	NA	NA	NA
CENPVP1	0	NA	NA	NA	NA	NA
ERCC-0007	0	NA	NA	NA	NA	NA
ERCC-0007	0	NA	NA	NA	NA	NA
ERCC-0007	0	NA	NA	NA	NA	NA
LOC65029	0	NA	NA	NA	NA	NA
KRT3	0	NA	NA	NA	NA	NA
KRT2	0	NA	NA	NA	NA	NA
KRT4	0	NA	NA	NA	NA	NA
KRT9	0	NA	NA	NA	NA	NA
ARPC4-TTL	0	NA	NA	NA	NA	NA
LOC10013	0	NA	NA	NA	NA	NA
DMRTC1	0	NA	NA	NA	NA	NA
TMEM189	0	NA	NA	NA	NA	NA
MIR5680	0	NA	NA	NA	NA	NA
MIR5689	0	NA	NA	NA	NA	NA
SPHAR	0	NA	NA	NA	NA	NA
GP1BB	0	NA	NA	NA	NA	NA
LINC00102	0	NA	NA	NA	NA	NA
RAB4B-EG	0	NA	NA	NA	NA	NA
MIR302F	0	NA	NA	NA	NA	NA
MIR4283-2	0	NA	NA	NA	NA	NA
MIR4283-1	0	NA	NA	NA	NA	NA
DUSP21	0	NA	NA	NA	NA	NA
MIR890	0	NA	NA	NA	NA	NA
LOC10013	0	NA	NA	NA	NA	NA
FAM197Y2	0	NA	NA	NA	NA	NA
MIR429	0	NA	NA	NA	NA	NA
ERCC-0013	0	NA	NA	NA	NA	NA
ERCC-0013	0	NA	NA	NA	NA	NA
ERCC-0013	0	NA	NA	NA	NA	NA
ERCC-0013	0	NA	NA	NA	NA	NA
ERCC-0013	0	NA	NA	NA	NA	NA
GOLGA2P3	0	NA	NA	NA	NA	NA
EPYC	0	NA	NA	NA	NA	NA
OR6C75	0	NA	NA	NA	NA	NA
OR6C74	0	NA	NA	NA	NA	NA
OR6C76	0	NA	NA	NA	NA	NA
OR6C70	0	NA	NA	NA	NA	NA
KIR3DL3	0	NA	NA	NA	NA	NA
LOC10013	0	NA	NA	NA	NA	NA
MIR521-1	0	NA	NA	NA	NA	NA
MIR521-2	0	NA	NA	NA	NA	NA
KRTAP21-3	0	NA	NA	NA	NA	NA

KRTAP21-2	0 NA	NA	NA	NA	NA
OR2T11	0 NA	NA	NA	NA	NA
DYX1C1-C	0 NA	NA	NA	NA	NA
MIR3914-2	0 NA	NA	NA	NA	NA
OR7G2	0 NA	NA	NA	NA	NA
OR7G3	0 NA	NA	NA	NA	NA
OR7G1	0 NA	NA	NA	NA	NA
SPAG11A	0 NA	NA	NA	NA	NA
OR9Q2	0 NA	NA	NA	NA	NA
SNORD115	0 NA	NA	NA	NA	NA
SNORD115	0 NA	NA	NA	NA	NA
SNORD115	0 NA	NA	NA	NA	NA
NBPF6	0 NA	NA	NA	NA	NA
NBPF4	0 NA	NA	NA	NA	NA
ZNF816-ZN	0 NA	NA	NA	NA	NA
ZFP91-CNT	0 NA	NA	NA	NA	NA
OR8H1	0 NA	NA	NA	NA	NA
OR8H2	0 NA	NA	NA	NA	NA
OR8H3	0 NA	NA	NA	NA	NA
PLEKHS1	0 NA	NA	NA	NA	NA
ONECUT3	0 NA	NA	NA	NA	NA
MIR519D	0 NA	NA	NA	NA	NA
MIR519E	0 NA	NA	NA	NA	NA
MIR519C	0 NA	NA	NA	NA	NA
MIR941-3	0 NA	NA	NA	NA	NA
MIR941-2	0 NA	NA	NA	NA	NA
MIR941-4	0 NA	NA	NA	NA	NA
LOC10013	0 NA	NA	NA	NA	NA
ERCC-0004	0 NA	NA	NA	NA	NA
MIR92A1	0 NA	NA	NA	NA	NA
MIR92A2	0 NA	NA	NA	NA	NA
OR5L1	0 NA	NA	NA	NA	NA
OR5L2	0 NA	NA	NA	NA	NA
MIR4436B	0 NA	NA	NA	NA	NA
MIR4436B	0 NA	NA	NA	NA	NA
OR4B1	0 NA	NA	NA	NA	NA
URGCP-M	0 NA	NA	NA	NA	NA
HSPB3	0 NA	NA	NA	NA	NA
KRTAP25-1	0 NA	NA	NA	NA	NA
TTTY6B	0 NA	NA	NA	NA	NA
SCGB2A2	0 NA	NA	NA	NA	NA
TCF23	0 NA	NA	NA	NA	NA
DIAPH3-AS	0 NA	NA	NA	NA	NA
INSL4	0 NA	NA	NA	NA	NA
PRNT	0 NA	NA	NA	NA	NA
KTI12	0 NA	NA	NA	NA	NA
PRSS57	0 NA	NA	NA	NA	NA

MIR5692C	0	NA	NA	NA	NA	NA
MAFIP	0	NA	NA	NA	NA	NA
LOC72801	0	NA	NA	NA	NA	NA
C7orf55-L	0	NA	NA	NA	NA	NA
DEFB116	0	NA	NA	NA	NA	NA
DEFB114	0	NA	NA	NA	NA	NA
DEFB112	0	NA	NA	NA	NA	NA
DEFB113	0	NA	NA	NA	NA	NA
DEFB110	0	NA	NA	NA	NA	NA
DEFB118	0	NA	NA	NA	NA	NA
DEFB119	0	NA	NA	NA	NA	NA
CALML5	0	NA	NA	NA	NA	NA
MIR5011	0	NA	NA	NA	NA	NA
MIR96	0	NA	NA	NA	NA	NA
XAGE1A	0	NA	NA	NA	NA	NA
XAGE1B	0	NA	NA	NA	NA	NA
XAGE1C	0	NA	NA	NA	NA	NA
XAGE1D	0	NA	NA	NA	NA	NA
XAGE1E	0	NA	NA	NA	NA	NA
MIR3622A	0	NA	NA	NA	NA	NA
MIR711	0	NA	NA	NA	NA	NA
LINC00424	0	NA	NA	NA	NA	NA
OR4D10	0	NA	NA	NA	NA	NA
OR4D11	0	NA	NA	NA	NA	NA
MIR3202-2	0	NA	NA	NA	NA	NA
MIR3202-1	0	NA	NA	NA	NA	NA
BCL2L2-PA	0	NA	NA	NA	NA	NA
TREML5P	0	NA	NA	NA	NA	NA
CCL3L3	0	NA	NA	NA	NA	NA
MIR550B1	0	NA	NA	NA	NA	NA
MIR550B2	0	NA	NA	NA	NA	NA
C10orf71	0	NA	NA	NA	NA	NA
TMIGD1	0	NA	NA	NA	NA	NA
SHOX	0	NA	NA	NA	NA	NA
LINC00229	0	NA	NA	NA	NA	NA
LINC00226	0	NA	NA	NA	NA	NA
FAM41AY1	0	NA	NA	NA	NA	NA
FAM41AY2	0	NA	NA	NA	NA	NA
HOXA10-H	0	NA	NA	NA	NA	NA
ITLN1	0	NA	NA	NA	NA	NA
MACC1-AS	0	NA	NA	NA	NA	NA
INS-IGF2	0	NA	NA	NA	NA	NA
MIR507	0	NA	NA	NA	NA	NA
MIR504	0	NA	NA	NA	NA	NA
LIMS3	0	NA	NA	NA	NA	NA
SUN5	0	NA	NA	NA	NA	NA
RBM14-RB	0	NA	NA	NA	NA	NA

FAM25C	0	NA	NA	NA	NA	NA
FAM25B	0	NA	NA	NA	NA	NA
FAM25G	0	NA	NA	NA	NA	NA
OR51F1	0	NA	NA	NA	NA	NA
PLGLB1	0	NA	NA	NA	NA	NA
PLGLB2	0	NA	NA	NA	NA	NA
SNORD115	0	NA	NA	NA	NA	NA
FLJ46257	0	NA	NA	NA	NA	NA
SNORD107	0	NA	NA	NA	NA	NA
SNORD108	0	NA	NA	NA	NA	NA
DCAF8L2	0	NA	NA	NA	NA	NA
MIR4792	0	NA	NA	NA	NA	NA
MIR4422	0	NA	NA	NA	NA	NA
MIR4426	0	NA	NA	NA	NA	NA
MIR105-2	0	NA	NA	NA	NA	NA
MIR105-1	0	NA	NA	NA	NA	NA
OR2A20P	0	NA	NA	NA	NA	NA
USP17L5	0	NA	NA	NA	NA	NA
OR2AP1	0	NA	NA	NA	NA	NA
POTEM	0	NA	NA	NA	NA	NA
POTEG	0	NA	NA	NA	NA	NA
POTEC	0	NA	NA	NA	NA	NA
POTEB	0	NA	NA	NA	NA	NA
MAGEA9B	0	NA	NA	NA	NA	NA
GH2	0	NA	NA	NA	NA	NA
C10orf120	0	NA	NA	NA	NA	NA
MIR338	0	NA	NA	NA	NA	NA
OR1J2	0	NA	NA	NA	NA	NA
SNAR-B2	0	NA	NA	NA	NA	NA
SNAR-B1	0	NA	NA	NA	NA	NA
MMP7	0	NA	NA	NA	NA	NA
MMP1	0	NA	NA	NA	NA	NA
LOC22112	0	NA	NA	NA	NA	NA
OR5AP2	0	NA	NA	NA	NA	NA
LOC10013	0	NA	NA	NA	NA	NA
LCE2C	0	NA	NA	NA	NA	NA
LCE2B	0	NA	NA	NA	NA	NA
LCE2A	0	NA	NA	NA	NA	NA
LCE2D	0	NA	NA	NA	NA	NA
HNRNPUL	0	NA	NA	NA	NA	NA
MIR5095	0	NA	NA	NA	NA	NA
MIR4320	0	NA	NA	NA	NA	NA
MIR4327	0	NA	NA	NA	NA	NA
MIR4325	0	NA	NA	NA	NA	NA
MIR4328	0	NA	NA	NA	NA	NA
MIR4329	0	NA	NA	NA	NA	NA
IL36RN	0	NA	NA	NA	NA	NA

FLJ25328	0	NA	NA	NA	NA	NA
MIR17	0	NA	NA	NA	NA	NA
ANXA8L2	0	NA	NA	NA	NA	NA
ANXA8L1	0	NA	NA	NA	NA	NA
KRTAP10-9	0	NA	NA	NA	NA	NA
KRTAP10-5	0	NA	NA	NA	NA	NA
KRTAP10-4	0	NA	NA	NA	NA	NA
MIR139	0	NA	NA	NA	NA	NA
MIR136	0	NA	NA	NA	NA	NA
MIR137	0	NA	NA	NA	NA	NA
FGF16	0	NA	NA	NA	NA	NA
CLPSL2	0	NA	NA	NA	NA	NA
OR11G2	0	NA	NA	NA	NA	NA
MIR4698	0	NA	NA	NA	NA	NA
MIR4699	0	NA	NA	NA	NA	NA
MIR4693	0	NA	NA	NA	NA	NA
MIR4696	0	NA	NA	NA	NA	NA
MIR4697	0	NA	NA	NA	NA	NA
MIR4695	0	NA	NA	NA	NA	NA
MIR518A1	0	NA	NA	NA	NA	NA
POM121L1	0	NA	NA	NA	NA	NA
MIR518A2	0	NA	NA	NA	NA	NA
LOC10012	0	NA	NA	NA	NA	NA
RNA5-8S5	0	NA	NA	NA	NA	NA
SPANXN1	0	NA	NA	NA	NA	NA
TFDP3	0	NA	NA	NA	NA	NA
OR5AC2	0	NA	NA	NA	NA	NA
LINC00502	0	NA	NA	NA	NA	NA
ZBED1	0	NA	NA	NA	NA	NA
NANOGNB	0	NA	NA	NA	NA	NA
CXorf49B	0	NA	NA	NA	NA	NA
PPY	0	NA	NA	NA	NA	NA
NME1-NM	0	NA	NA	NA	NA	NA
SPACA5B	0	NA	NA	NA	NA	NA
MIR484	0	NA	NA	NA	NA	NA
USP17L29	0	NA	NA	NA	NA	NA
USP17L22	0	NA	NA	NA	NA	NA
USP17L20	0	NA	NA	NA	NA	NA
USP17L26	0	NA	NA	NA	NA	NA
USP17L27	0	NA	NA	NA	NA	NA
USP17L24	0	NA	NA	NA	NA	NA
USP17L25	0	NA	NA	NA	NA	NA
CSN1S2AP	0	NA	NA	NA	NA	NA
FTMT	0	NA	NA	NA	NA	NA
MRGPRG-	0	NA	NA	NA	NA	NA
LOC28617	0	NA	NA	NA	NA	NA
KIR2DS4	0	NA	NA	NA	NA	NA

KIR2DS5	0	NA	NA	NA	NA	NA
KIR2DS2	0	NA	NA	NA	NA	NA
KIR2DS3	0	NA	NA	NA	NA	NA
KIR2DS1	0	NA	NA	NA	NA	NA
GRAPL	0	NA	NA	NA	NA	NA
MIR4713	0	NA	NA	NA	NA	NA
MIR4710	0	NA	NA	NA	NA	NA
TTTY2	0	NA	NA	NA	NA	NA
TTTY3	0	NA	NA	NA	NA	NA
TTTY1	0	NA	NA	NA	NA	NA
TTTY6	0	NA	NA	NA	NA	NA
TTTY7	0	NA	NA	NA	NA	NA
TTTY4	0	NA	NA	NA	NA	NA
TTTY5	0	NA	NA	NA	NA	NA
TTTY8	0	NA	NA	NA	NA	NA
METTL11B	0	NA	NA	NA	NA	NA
OR10G9	0	NA	NA	NA	NA	NA
DMRTC2	0	NA	NA	NA	NA	NA
DUX4L	0	NA	NA	NA	NA	NA
LRRC30	0	NA	NA	NA	NA	NA
KIAA1024L	0	NA	NA	NA	NA	NA
MIR622	0	NA	NA	NA	NA	NA
MIR621	0	NA	NA	NA	NA	NA
LINC00331	0	NA	NA	NA	NA	NA
LINC00330	0	NA	NA	NA	NA	NA
ERCC-0004	0	NA	NA	NA	NA	NA
ERCC-0004	0	NA	NA	NA	NA	NA
ERCC-0004	0	NA	NA	NA	NA	NA
ERCC-0004	0	NA	NA	NA	NA	NA
ERCC-0004	0	NA	NA	NA	NA	NA
ERCC-0004	0	NA	NA	NA	NA	NA
MIR24-2	0	NA	NA	NA	NA	NA
MIR24-1	0	NA	NA	NA	NA	NA
LOC73044	0	NA	NA	NA	NA	NA
SLC34A1	0	NA	NA	NA	NA	NA
OR9G4	0	NA	NA	NA	NA	NA
CCL3L1	0	NA	NA	NA	NA	NA
LYZL1	0	NA	NA	NA	NA	NA
LYZL2	0	NA	NA	NA	NA	NA
TCEB3CL	0	NA	NA	NA	NA	NA
MIR4508	0	NA	NA	NA	NA	NA
MIR4503	0	NA	NA	NA	NA	NA
MIR4500	0	NA	NA	NA	NA	NA
HSFY1	0	NA	NA	NA	NA	NA
HSFY2	0	NA	NA	NA	NA	NA
KRT16P3	0	NA	NA	NA	NA	NA
KRT16P1	0	NA	NA	NA	NA	NA

SLX1A	0	NA	NA	NA	NA	NA
SLX1B	0	NA	NA	NA	NA	NA
LOC28321	0	NA	NA	NA	NA	NA
CTSL3P	0	NA	NA	NA	NA	NA
OR10G7	0	NA	NA	NA	NA	NA
OR10G3	0	NA	NA	NA	NA	NA
OR10G8	0	NA	NA	NA	NA	NA
DNAJB3	0	NA	NA	NA	NA	NA
PPP1R3A	0	NA	NA	NA	NA	NA
HEPN1	0	NA	NA	NA	NA	NA
GUCA2B	0	NA	NA	NA	NA	NA
LOC40065	0	NA	NA	NA	NA	NA
PF4V1	0	NA	NA	NA	NA	NA
CASR	0	NA	NA	NA	NA	NA
KRTAP8-1	0	NA	NA	NA	NA	NA
ANXA8	0	NA	NA	NA	NA	NA
SSX6	0	NA	NA	NA	NA	NA
SSX7	0	NA	NA	NA	NA	NA
SSX4	0	NA	NA	NA	NA	NA
SSX2	0	NA	NA	NA	NA	NA
SSX3	0	NA	NA	NA	NA	NA
SSX1	0	NA	NA	NA	NA	NA
SSX8	0	NA	NA	NA	NA	NA
SSX9	0	NA	NA	NA	NA	NA
NDFIP2-AS	0	NA	NA	NA	NA	NA
NXF2	0	NA	NA	NA	NA	NA
HOXD10	0	NA	NA	NA	NA	NA
FAM150A	0	NA	NA	NA	NA	NA
LINC00589	0	NA	NA	NA	NA	NA
LOC10050	0	NA	NA	NA	NA	NA
MMP10	0	NA	NA	NA	NA	NA
KCNK18	0	NA	NA	NA	NA	NA
KCNK16	0	NA	NA	NA	NA	NA
SNAR-A14	0	NA	NA	NA	NA	NA
SNAR-A12	0	NA	NA	NA	NA	NA
IL37	0	NA	NA	NA	NA	NA
MIR3180-2	0	NA	NA	NA	NA	NA
MIR3180-3	0	NA	NA	NA	NA	NA
MIR3180-1	0	NA	NA	NA	NA	NA
OR8K1	0	NA	NA	NA	NA	NA
OR8K3	0	NA	NA	NA	NA	NA
LOC28358	0	NA	NA	NA	NA	NA
OPN1LW	0	NA	NA	NA	NA	NA
LOC38983	0	NA	NA	NA	NA	NA
LOC38983	0	NA	NA	NA	NA	NA
MIR519A1	0	NA	NA	NA	NA	NA
MIR519A2	0	NA	NA	NA	NA	NA

SNORA17	0	NA	NA	NA	NA	NA
ACSM2B	0	NA	NA	NA	NA	NA
FLJ25758	0	NA	NA	NA	NA	NA
KRTAP9-8	0	NA	NA	NA	NA	NA
KRTAP9-9	0	NA	NA	NA	NA	NA
KRTAP9-1	0	NA	NA	NA	NA	NA
KRTAP9-2	0	NA	NA	NA	NA	NA
KRTAP9-3	0	NA	NA	NA	NA	NA
KRTAP9-4	0	NA	NA	NA	NA	NA
GPR174	0	NA	NA	NA	NA	NA
RNY5	0	NA	NA	NA	NA	NA
ERCC-0008	0	NA	NA	NA	NA	NA
ERCC-0008	0	NA	NA	NA	NA	NA
ERCC-0008	0	NA	NA	NA	NA	NA
LOC10028	0	NA	NA	NA	NA	NA
MIR548D1	0	NA	NA	NA	NA	NA
MIR548D2	0	NA	NA	NA	NA	NA
OR5I1	0	NA	NA	NA	NA	NA
TLX1NB	0	NA	NA	NA	NA	NA
OR4M1	0	NA	NA	NA	NA	NA
MIR891A	0	NA	NA	NA	NA	NA
MIR891B	0	NA	NA	NA	NA	NA
STOML3	0	NA	NA	NA	NA	NA
AKR1CL1	0	NA	NA	NA	NA	NA
LOC38824	0	NA	NA	NA	NA	NA
MIR3689F	0	NA	NA	NA	NA	NA
IL17A	0	NA	NA	NA	NA	NA
HOXD12	0	NA	NA	NA	NA	NA
MIR320B1	0	NA	NA	NA	NA	NA
NLRP7	0	NA	NA	NA	NA	NA
IGF2BP1	0	NA	NA	NA	NA	NA
NLRP13	0	NA	NA	NA	NA	NA
DIRC1	0	NA	NA	NA	NA	NA
MIRLET7A	0	NA	NA	NA	NA	NA
DEFB129	0	NA	NA	NA	NA	NA
DEFB128	0	NA	NA	NA	NA	NA
DEFB127	0	NA	NA	NA	NA	NA
DEFB126	0	NA	NA	NA	NA	NA
DEFB124	0	NA	NA	NA	NA	NA
DEFB123	0	NA	NA	NA	NA	NA
DEFB122	0	NA	NA	NA	NA	NA
DEFB121	0	NA	NA	NA	NA	NA
LOC44011	0	NA	NA	NA	NA	NA
TRIM64C	0	NA	NA	NA	NA	NA
TRIM64B	0	NA	NA	NA	NA	NA
MAGEC2	0	NA	NA	NA	NA	NA
SNX15	0	NA	NA	NA	NA	NA

NEUROD4	0	NA	NA	NA	NA	NA
LINC00474	0	NA	NA	NA	NA	NA
LINC00479	0	NA	NA	NA	NA	NA
TRIM74	0	NA	NA	NA	NA	NA
TRIM73	0	NA	NA	NA	NA	NA
MIR208B	0	NA	NA	NA	NA	NA
ERCC-0016	0	NA	NA	NA	NA	NA
ERCC-0016	0	NA	NA	NA	NA	NA
ERCC-0016	0	NA	NA	NA	NA	NA
ERCC-0016	0	NA	NA	NA	NA	NA
MIR3680-1	0	NA	NA	NA	NA	NA
MS4A13	0	NA	NA	NA	NA	NA
PASD1	0	NA	NA	NA	NA	NA
MIR526A2	0	NA	NA	NA	NA	NA
MIR526A1	0	NA	NA	NA	NA	NA
ASMTL	0	NA	NA	NA	NA	NA
OR10T2	0	NA	NA	NA	NA	NA
IRX4	0	NA	NA	NA	NA	NA
LINC00516	0	NA	NA	NA	NA	NA
MIR944	0	NA	NA	NA	NA	NA
RPL17-C18	0	NA	NA	NA	NA	NA
AREG	0	NA	NA	NA	NA	NA
MRGPRX1	0	NA	NA	NA	NA	NA
MRGPRX2	0	NA	NA	NA	NA	NA
PITX2	0	NA	NA	NA	NA	NA
MIR4315-1	0	NA	NA	NA	NA	NA
OR9K2	0	NA	NA	NA	NA	NA
SNAR-A11	0	NA	NA	NA	NA	NA
SNAR-A10	0	NA	NA	NA	NA	NA
MIR4659A	0	NA	NA	NA	NA	NA
MIR4659B	0	NA	NA	NA	NA	NA
KRTAP13-4	0	NA	NA	NA	NA	NA
KRTAP13-1	0	NA	NA	NA	NA	NA
KRTAP13-3	0	NA	NA	NA	NA	NA
WFDC11	0	NA	NA	NA	NA	NA
WFDC12	0	NA	NA	NA	NA	NA
OR51A4	0	NA	NA	NA	NA	NA
OR51A7	0	NA	NA	NA	NA	NA
COMMD3-	0	NA	NA	NA	NA	NA
MIR4290	0	NA	NA	NA	NA	NA
MIR4291	0	NA	NA	NA	NA	NA
MIR4299	0	NA	NA	NA	NA	NA
OR5A2	0	NA	NA	NA	NA	NA
OR5A1	0	NA	NA	NA	NA	NA
RPS17L	0	NA	NA	NA	NA	NA
CXorf51A	0	NA	NA	NA	NA	NA
HOXB9	0	NA	NA	NA	NA	NA

HOXB1	0	NA	NA	NA	NA	NA
HOXB5	0	NA	NA	NA	NA	NA
LOC10016	0	NA	NA	NA	NA	NA
OR4E2	0	NA	NA	NA	NA	NA
OR4C46	0	NA	NA	NA	NA	NA
UPK3BL	0	NA	NA	NA	NA	NA
TTTY16	0	NA	NA	NA	NA	NA
TTTY11	0	NA	NA	NA	NA	NA
TTTY12	0	NA	NA	NA	NA	NA
TTTY13	0	NA	NA	NA	NA	NA
TTTY18	0	NA	NA	NA	NA	NA
RNU5D-1	0	NA	NA	NA	NA	NA
GAGE7	0	NA	NA	NA	NA	NA
MIR527	0	NA	NA	NA	NA	NA
TTTY1B	0	NA	NA	NA	NA	NA
DHRSX	0	NA	NA	NA	NA	NA
HRG	0	NA	NA	NA	NA	NA
AMELY	0	NA	NA	NA	NA	NA
CXorf49	0	NA	NA	NA	NA	NA
OR4X1	0	NA	NA	NA	NA	NA
OR4X2	0	NA	NA	NA	NA	NA
PRSS21	0	NA	NA	NA	NA	NA
KRTAP4-9	0	NA	NA	NA	NA	NA
KRTAP4-1	0	NA	NA	NA	NA	NA
KRTAP4-3	0	NA	NA	NA	NA	NA
KRTAP4-2	0	NA	NA	NA	NA	NA
KRTAP4-5	0	NA	NA	NA	NA	NA
KRTAP4-7	0	NA	NA	NA	NA	NA
KRTAP4-6	0	NA	NA	NA	NA	NA
KRTAP12-1	0	NA	NA	NA	NA	NA
KRTAP12-3	0	NA	NA	NA	NA	NA
KRTAP12-2	0	NA	NA	NA	NA	NA
KRTAP12-4	0	NA	NA	NA	NA	NA
SNAR-A9	0	NA	NA	NA	NA	NA
SNAR-A8	0	NA	NA	NA	NA	NA
SNAR-A3	0	NA	NA	NA	NA	NA
SNAR-A2	0	NA	NA	NA	NA	NA
SNAR-A1	0	NA	NA	NA	NA	NA
SNAR-A7	0	NA	NA	NA	NA	NA
SNAR-A6	0	NA	NA	NA	NA	NA
SNAR-A5	0	NA	NA	NA	NA	NA
SNAR-A4	0	NA	NA	NA	NA	NA
LOC72804	0	NA	NA	NA	NA	NA
RNASE9	0	NA	NA	NA	NA	NA
KRTAP17-1	0	NA	NA	NA	NA	NA
OTOL1	0	NA	NA	NA	NA	NA
CDY2B	0	NA	NA	NA	NA	NA

LOC40079	0	NA	NA	NA	NA	NA
MIR4313	0	NA	NA	NA	NA	NA
MIR4317	0	NA	NA	NA	NA	NA
MIR200C	0	NA	NA	NA	NA	NA
RNU5E-1	0	NA	NA	NA	NA	NA
NPS	0	NA	NA	NA	NA	NA
CNGA2	0	NA	NA	NA	NA	NA
FAM99A	0	NA	NA	NA	NA	NA
FAM99B	0	NA	NA	NA	NA	NA
RBMY2FP	0	NA	NA	NA	NA	NA
MIR520H	0	NA	NA	NA	NA	NA
MIR520B	0	NA	NA	NA	NA	NA
MIR520C	0	NA	NA	NA	NA	NA
MIR520A	0	NA	NA	NA	NA	NA
MIR520F	0	NA	NA	NA	NA	NA
MIR520G	0	NA	NA	NA	NA	NA
MIR520D	0	NA	NA	NA	NA	NA
MIR520E	0	NA	NA	NA	NA	NA
ARSH	0	NA	NA	NA	NA	NA
PRR5-ARH	0	NA	NA	NA	NA	NA
OR8K5	0	NA	NA	NA	NA	NA
LOC10028	0	NA	NA	NA	NA	NA
TEN1	0	NA	NA	NA	NA	NA
BOLA2	0	NA	NA	NA	NA	NA
MIR1233-2	0	NA	NA	NA	NA	NA
FAM224B	0	NA	NA	NA	NA	NA
FAM224A	0	NA	NA	NA	NA	NA
SNORD116	0	NA	NA	NA	NA	NA
HOXC-AS5	0	NA	NA	NA	NA	NA
SNORD116	0	NA	NA	NA	NA	NA
XAGE3	0	NA	NA	NA	NA	NA
FAM9C	0	NA	NA	NA	NA	NA
SNORD62A	0	NA	NA	NA	NA	NA
SNORD62B	0	NA	NA	NA	NA	NA
DEFB105A	0	NA	NA	NA	NA	NA
DEFB105B	0	NA	NA	NA	NA	NA
ZNF658B	0	NA	NA	NA	NA	NA
CSN1S2BP	0	NA	NA	NA	NA	NA
PRDM9	0	NA	NA	NA	NA	NA
RTP2	0	NA	NA	NA	NA	NA
SAA3P	0	NA	NA	NA	NA	NA
ACSM2A	0	NA	NA	NA	NA	NA
C17orf49	0	NA	NA	NA	NA	NA
MIR4471	0	NA	NA	NA	NA	NA
MIR4476	0	NA	NA	NA	NA	NA
PRR21	0	NA	NA	NA	NA	NA
HOXA-AS4	0	NA	NA	NA	NA	NA

S100A7A	0 NA	NA	NA	NA	NA
MIR3120	0 NA	NA	NA	NA	NA
MIR3122	0 NA	NA	NA	NA	NA
MIR3129	0 NA	NA	NA	NA	NA
TTTY9B	0 NA	NA	NA	NA	NA
MIR1181	0 NA	NA	NA	NA	NA
MIR1182	0 NA	NA	NA	NA	NA
MYL2	0 NA	NA	NA	NA	NA
MIR4800	0 NA	NA	NA	NA	NA
MIR449A	0 NA	NA	NA	NA	NA
OR5M10	0 NA	NA	NA	NA	NA
OR5M11	0 NA	NA	NA	NA	NA
LOC40008	0 NA	NA	NA	NA	NA
FRG2C	0 NA	NA	NA	NA	NA
MIR1973	0 NA	NA	NA	NA	NA
OR4P4	0 NA	NA	NA	NA	NA
MIR362	0 NA	NA	NA	NA	NA
ERCC-0001	0 NA	NA	NA	NA	NA
ERCC-0001	0 NA	NA	NA	NA	NA
ERCC-0001	0 NA	NA	NA	NA	NA
ERCC-0001	0 NA	NA	NA	NA	NA
ERCC-0001	0 NA	NA	NA	NA	NA
T	0 NA	NA	NA	NA	NA
HMX3	0 NA	NA	NA	NA	NA
HMX2	0 NA	NA	NA	NA	NA
LUZP4	0 NA	NA	NA	NA	NA
LUZP6	0 NA	NA	NA	NA	NA
LOC33924	0 NA	NA	NA	NA	NA
RBMY1A3	0 NA	NA	NA	NA	NA
SP8	0 NA	NA	NA	NA	NA
CSAG4	0 NA	NA	NA	NA	NA
TP53TG3	0 NA	NA	NA	NA	NA
OR4D9	0 NA	NA	NA	NA	NA
OR5AN1	0 NA	NA	NA	NA	NA
VCY	0 NA	NA	NA	NA	NA
PRAMEF4	0 NA	NA	NA	NA	NA
PRAMEF5	0 NA	NA	NA	NA	NA
PRAMEF6	0 NA	NA	NA	NA	NA
PRAMEF7	0 NA	NA	NA	NA	NA
PRAMEF1	0 NA	NA	NA	NA	NA
PRAMEF2	0 NA	NA	NA	NA	NA
PRAMEF3	0 NA	NA	NA	NA	NA
PRAMEF8	0 NA	NA	NA	NA	NA
PRAMEF9	0 NA	NA	NA	NA	NA
KIR3DS1	0 NA	NA	NA	NA	NA
MIR448	0 NA	NA	NA	NA	NA
PRSS3P2	0 NA	NA	NA	NA	NA

FER1L6-AS	0 NA	NA	NA	NA	NA
LGALS7	0 NA	NA	NA	NA	NA
NMS	0 NA	NA	NA	NA	NA
SRGAP2B	0 NA	NA	NA	NA	NA
ADH7	0 NA	NA	NA	NA	NA
SERPINA7	0 NA	NA	NA	NA	NA
LOC28378	0 NA	NA	NA	NA	NA
MIR216B	0 NA	NA	NA	NA	NA
MIR216A	0 NA	NA	NA	NA	NA
YY1P2	0 NA	NA	NA	NA	NA
MMP27	0 NA	NA	NA	NA	NA
MMP20	0 NA	NA	NA	NA	NA
TTTY21B	0 NA	NA	NA	NA	NA
MIR516A2	0 NA	NA	NA	NA	NA
MIR516A1	0 NA	NA	NA	NA	NA
HLA-DRB4	0 NA	NA	NA	NA	NA
LOC65348	0 NA	NA	NA	NA	NA
RNU6-52	0 NA	NA	NA	NA	NA
RNU6-53	0 NA	NA	NA	NA	NA
KRT75	0 NA	NA	NA	NA	NA
KRT76	0 NA	NA	NA	NA	NA
KRT73	0 NA	NA	NA	NA	NA
KRT79	0 NA	NA	NA	NA	NA
KRT78	0 NA	NA	NA	NA	NA
SNORD115	0 NA	NA	NA	NA	NA
SNORD115	0 NA	NA	NA	NA	NA
MIR4741	0 NA	NA	NA	NA	NA
MIR4745	0 NA	NA	NA	NA	NA
MIR4747	0 NA	NA	NA	NA	NA
OR2G6	0 NA	NA	NA	NA	NA
LOC10023	0 NA	NA	NA	NA	NA
DSCR4	0 NA	NA	NA	NA	NA
CRYGB	0 NA	NA	NA	NA	NA
CRYGC	0 NA	NA	NA	NA	NA
LINC00308	0 NA	NA	NA	NA	NA
LINC00307	0 NA	NA	NA	NA	NA
SNORA43	0 NA	NA	NA	NA	NA
RBMY1B	0 NA	NA	NA	NA	NA
RBMY1F	0 NA	NA	NA	NA	NA
RBMY1E	0 NA	NA	NA	NA	NA
OR51T1	0 NA	NA	NA	NA	NA
ISY1-RAB4	0 NA	NA	NA	NA	NA
C7orf33	0 NA	NA	NA	NA	NA
MSANTD3	0 NA	NA	NA	NA	NA
MIR517A	0 NA	NA	NA	NA	NA
MIR517B	0 NA	NA	NA	NA	NA
MIR517C	0 NA	NA	NA	NA	NA

ICOS	0 NA	NA	NA	NA	NA
MIR671	0 NA	NA	NA	NA	NA
MIR675	0 NA	NA	NA	NA	NA
LINC00366	0 NA	NA	NA	NA	NA
LIMS3L	0 NA	NA	NA	NA	NA
ERCC-0009	0 NA	NA	NA	NA	NA
ERCC-0009	0 NA	NA	NA	NA	NA
ERCC-0009	0 NA	NA	NA	NA	NA
ERCC-0009	0 NA	NA	NA	NA	NA
ERCC-0009	0 NA	NA	NA	NA	NA
LOC14648	0 NA	NA	NA	NA	NA
FLJ46300	0 NA	NA	NA	NA	NA
RIMBP3B	0 NA	NA	NA	NA	NA
C16orf97	0 NA	NA	NA	NA	NA
TRIM39-R	0 NA	NA	NA	NA	NA
SNORD58B	0 NA	NA	NA	NA	NA
SERPINA13	0 NA	NA	NA	NA	NA
OC90	0 NA	NA	NA	NA	NA
LINC00160	0 NA	NA	NA	NA	NA
MIR3155B	0 NA	NA	NA	NA	NA
MIR2964A	0 NA	NA	NA	NA	NA
PROL1	0 NA	NA	NA	NA	NA
LINC00838	0 NA	NA	NA	NA	NA
DNAJC25-	0 NA	NA	NA	NA	NA
SNORD86	0 NA	NA	NA	NA	NA
GTPBP6	0 NA	NA	NA	NA	NA
GPR75-AS	0 NA	NA	NA	NA	NA
LOC61303	0 NA	NA	NA	NA	NA
MAGED4	0 NA	NA	NA	NA	NA
LY75-CD30	0 NA	NA	NA	NA	NA
NEUROG1	0 NA	NA	NA	NA	NA
TRIM48	0 NA	NA	NA	NA	NA
TRIM49	0 NA	NA	NA	NA	NA
TRIM40	0 NA	NA	NA	NA	NA
ERCC-0011	0 NA	NA	NA	NA	NA
ERCC-0011	0 NA	NA	NA	NA	NA
ERCC-0011	0 NA	NA	NA	NA	NA
ERCC-0011	0 NA	NA	NA	NA	NA
ERCC-0011	0 NA	NA	NA	NA	NA
MIR3156-3	0 NA	NA	NA	NA	NA
MIR3156-2	0 NA	NA	NA	NA	NA
MIR3156-1	0 NA	NA	NA	NA	NA
GAGE12H	0 NA	NA	NA	NA	NA
GAGE12I	0 NA	NA	NA	NA	NA
GAGE12J	0 NA	NA	NA	NA	NA
GAGE12B	0 NA	NA	NA	NA	NA
GAGE12C	0 NA	NA	NA	NA	NA

GAGE12D	0	NA	NA	NA	NA	NA
GAGE12E	0	NA	NA	NA	NA	NA
GAGE12F	0	NA	NA	NA	NA	NA
GAGE12G	0	NA	NA	NA	NA	NA
POC1B-GA	0	NA	NA	NA	NA	NA
LOC28609	0	NA	NA	NA	NA	NA
MIR3908	0	NA	NA	NA	NA	NA
MIR205	0	NA	NA	NA	NA	NA
MIR206	0	NA	NA	NA	NA	NA
MIR202	0	NA	NA	NA	NA	NA
MIR203	0	NA	NA	NA	NA	NA
MIR20A	0	NA	NA	NA	NA	NA
MIR20B	0	NA	NA	NA	NA	NA
MIR642B	0	NA	NA	NA	NA	NA
MIR642A	0	NA	NA	NA	NA	NA
MIR1297	0	NA	NA	NA	NA	NA
MIR1298	0	NA	NA	NA	NA	NA
OR7A17	0	NA	NA	NA	NA	NA
PAEP	0	NA	NA	NA	NA	NA
MIR567	0	NA	NA	NA	NA	NA
MIR561	0	NA	NA	NA	NA	NA
KRTAP19-2	0	NA	NA	NA	NA	NA
KRTAP19-1	0	NA	NA	NA	NA	NA
KRTAP19-6	0	NA	NA	NA	NA	NA
KRTAP19-7	0	NA	NA	NA	NA	NA
KRTAP19-4	0	NA	NA	NA	NA	NA
KRTAP19-5	0	NA	NA	NA	NA	NA
OCM2	0	NA	NA	NA	NA	NA
BPY2	0	NA	NA	NA	NA	NA
LOC10065	0	NA	NA	NA	NA	NA
MOGAT3	0	NA	NA	NA	NA	NA
RGPD6	0	NA	NA	NA	NA	NA
RGPD5	0	NA	NA	NA	NA	NA
LOR	0	NA	NA	NA	NA	NA
MIR3714	0	NA	NA	NA	NA	NA
HYALP1	0	NA	NA	NA	NA	NA
MIR371B	0	NA	NA	NA	NA	NA
MIR371A	0	NA	NA	NA	NA	NA
PPP2R3B	0	NA	NA	NA	NA	NA
MIR2392	0	NA	NA	NA	NA	NA
OR4C15	0	NA	NA	NA	NA	NA
OR4C16	0	NA	NA	NA	NA	NA
OR4C11	0	NA	NA	NA	NA	NA
OR4C13	0	NA	NA	NA	NA	NA
OR4C12	0	NA	NA	NA	NA	NA
MIR3926-2	0	NA	NA	NA	NA	NA
MIR3926-1	0	NA	NA	NA	NA	NA

TTTY4C	0	NA	NA	NA	NA	NA
TTTY4B	0	NA	NA	NA	NA	NA
LOC10050	0	NA	NA	NA	NA	NA
PER4	0	NA	NA	NA	NA	NA
OR5AR1	0	NA	NA	NA	NA	NA
PRLH	0	NA	NA	NA	NA	NA
MS4A12	0	NA	NA	NA	NA	NA
LOC15100	0	NA	NA	NA	NA	NA
MIR451B	0	NA	NA	NA	NA	NA
MIR511-1	0	NA	NA	NA	NA	NA
MIR511-2	0	NA	NA	NA	NA	NA
DEFB125	0	NA	NA	NA	NA	NA
INS	0	NA	NA	NA	NA	NA
OR8G5	0	NA	NA	NA	NA	NA
MIR198	0	NA	NA	NA	NA	NA
MIR19A	0	NA	NA	NA	NA	NA
LOC64616	0	NA	NA	NA	NA	NA
MIR3659	0	NA	NA	NA	NA	NA
MIR3658	0	NA	NA	NA	NA	NA
MIR133A2	0	NA	NA	NA	NA	NA
RNA5SP41	0	NA	NA	NA	NA	NA
PRAMEF22	0	NA	NA	NA	NA	NA
PRAMEF21	0	NA	NA	NA	NA	NA
PRAMEF20	0	NA	NA	NA	NA	NA
ANKRD30B	0	NA	NA	NA	NA	NA
MIR5580	0	NA	NA	NA	NA	NA
LINC00207	0	NA	NA	NA	NA	NA
LINC00208	0	NA	NA	NA	NA	NA
ASMT	0	NA	NA	NA	NA	NA
BIRC8	0	NA	NA	NA	NA	NA
KRTAP4-11	0	NA	NA	NA	NA	NA
KRTAP4-12	0	NA	NA	NA	NA	NA
F8A3	0	NA	NA	NA	NA	NA
F8A2	0	NA	NA	NA	NA	NA
SPRR1A	0	NA	NA	NA	NA	NA
SPRR1B	0	NA	NA	NA	NA	NA
PPAN-P2R	0	NA	NA	NA	NA	NA
DEFB106A	0	NA	NA	NA	NA	NA
DEFB106B	0	NA	NA	NA	NA	NA
ARL17B	0	NA	NA	NA	NA	NA
OR12D2	0	NA	NA	NA	NA	NA
LOC10009	0	NA	NA	NA	NA	NA
REXO1L2P	0	NA	NA	NA	NA	NA
HLTF-AS1	0	NA	NA	NA	NA	NA
SERF1A	0	NA	NA	NA	NA	NA
SERF1B	0	NA	NA	NA	NA	NA
HIST2H3C	0	NA	NA	NA	NA	NA

HIST2H3A	0	NA	NA	NA	NA	NA
TP53TG3B	0	NA	NA	NA	NA	NA
TP53TG3C	0	NA	NA	NA	NA	NA
KIR2DL5B	0	NA	NA	NA	NA	NA
KIR2DL5A	0	NA	NA	NA	NA	NA
DAZ2	0	NA	NA	NA	NA	NA
OR51D1	0	NA	NA	NA	NA	NA
ERCC-0016	0	NA	NA	NA	NA	NA
ERCC-0016	0	NA	NA	NA	NA	NA
MIR3680-2	0	NA	NA	NA	NA	NA
C17orf99	0	NA	NA	NA	NA	NA
LOC10028	0	NA	NA	NA	NA	NA
MIR3187	0	NA	NA	NA	NA	NA
OR5F1	0	NA	NA	NA	NA	NA
VAMP7	0	NA	NA	NA	NA	NA
MIR4443	0	NA	NA	NA	NA	NA
CCL24	0	NA	NA	NA	NA	NA
JMJD7-PLA	0	NA	NA	NA	NA	NA
SMEK3P	0	NA	NA	NA	NA	NA
MAS1L	0	NA	NA	NA	NA	NA
OR4S1	0	NA	NA	NA	NA	NA
OR4S2	0	NA	NA	NA	NA	NA
CRCT1	0	NA	NA	NA	NA	NA
LOC44091	0	NA	NA	NA	NA	NA
MIR644B	0	NA	NA	NA	NA	NA
TNFSF11	0	NA	NA	NA	NA	NA
LCE4A	0	NA	NA	NA	NA	NA
ATP5J2-PT	0	NA	NA	NA	NA	NA
OR5M3	0	NA	NA	NA	NA	NA
SERF2-C15	0	NA	NA	NA	NA	NA
MIR3618	0	NA	NA	NA	NA	NA
MIR3610	0	NA	NA	NA	NA	NA
MIR3616	0	NA	NA	NA	NA	NA
MIR3615	0	NA	NA	NA	NA	NA
MIR3614	0	NA	NA	NA	NA	NA
MIR3160-2	0	NA	NA	NA	NA	NA
NEU2	0	NA	NA	NA	NA	NA
TLX1	0	NA	NA	NA	NA	NA
FLJ33581	0	NA	NA	NA	NA	NA
KAAG1	0	NA	NA	NA	NA	NA
TCHHL1	0	NA	NA	NA	NA	NA
CT64	0	NA	NA	NA	NA	NA
CBLC	0	NA	NA	NA	NA	NA
FAM90A10	0	NA	NA	NA	NA	NA
LOC34035	0	NA	NA	NA	NA	NA
SULT1A4	0	NA	NA	NA	NA	NA
SULT1A3	0	NA	NA	NA	NA	NA

OLIG3	0	NA	NA	NA	NA	NA
FAM156B	0	NA	NA	NA	NA	NA
TNFAIP8L2	0	NA	NA	NA	NA	NA
LINC00698	0	NA	NA	NA	NA	NA
CT47A7	0	NA	NA	NA	NA	NA
TCL1B	0	NA	NA	NA	NA	NA
TCL1A	0	NA	NA	NA	NA	NA
CT47A4	0	NA	NA	NA	NA	NA
CT47A3	0	NA	NA	NA	NA	NA
CT47A2	0	NA	NA	NA	NA	NA
CT47A1	0	NA	NA	NA	NA	NA
CT47A9	0	NA	NA	NA	NA	NA
CT47A8	0	NA	NA	NA	NA	NA
DMRTC1B	0	NA	NA	NA	NA	NA
PRY	0	NA	NA	NA	NA	NA
ESX1	0	NA	NA	NA	NA	NA
OPN1MW	0	NA	NA	NA	NA	NA
MEF2B	0	NA	NA	NA	NA	NA
FETUB	0	NA	NA	NA	NA	NA
RNU6-64	0	NA	NA	NA	NA	NA
RNU6-69	0	NA	NA	NA	NA	NA
CDY1B	0	NA	NA	NA	NA	NA
LOC10013	0	NA	NA	NA	NA	NA
PAX1	0	NA	NA	NA	NA	NA
MBD3L1	0	NA	NA	NA	NA	NA
MBD3L2	0	NA	NA	NA	NA	NA
MBD3L3	0	NA	NA	NA	NA	NA
MBD3L4	0	NA	NA	NA	NA	NA
MBD3L5	0	NA	NA	NA	NA	NA
DSG3	0	NA	NA	NA	NA	NA
OR2J2	0	NA	NA	NA	NA	NA
ELK2AP	0	NA	NA	NA	NA	NA
ATP6V1G2	0	NA	NA	NA	NA	NA
SNORA7A	0	NA	NA	NA	NA	NA
FOXR2	0	NA	NA	NA	NA	NA
MIR3689D	0	NA	NA	NA	NA	NA
LRRC52	0	NA	NA	NA	NA	NA
MIR5100	0	NA	NA	NA	NA	NA
ERCC-0007	0	NA	NA	NA	NA	NA
AP1M2	0	NA	NA	NA	NA	NA
RPS7P5	0	NA	NA	NA	NA	NA
MIR1909	0	NA	NA	NA	NA	NA
LINC00685	0	NA	NA	NA	NA	NA
LINC00684	0	NA	NA	NA	NA	NA
MIR190B	0	NA	NA	NA	NA	NA
MIR600	0	NA	NA	NA	NA	NA
LINC00351	0	NA	NA	NA	NA	NA

LINC00353	0	NA	NA	NA	NA	NA
OR4K5	0	NA	NA	NA	NA	NA
OR4K1	0	NA	NA	NA	NA	NA
OR4K2	0	NA	NA	NA	NA	NA
RNA45S5	0	NA	NA	NA	NA	NA
AADACL4	0	NA	NA	NA	NA	NA
AADACL3	0	NA	NA	NA	NA	NA
MIR509-2	0	NA	NA	NA	NA	NA
ERCC-0006	0	NA	NA	NA	NA	NA
ERCC-0006	0	NA	NA	NA	NA	NA
ERCC-0006	0	NA	NA	NA	NA	NA
APITD1-CO	0	NA	NA	NA	NA	NA
FKBP1A-SD	0	NA	NA	NA	NA	NA
FOXI1	0	NA	NA	NA	NA	NA
FOXI3	0	NA	NA	NA	NA	NA
MIR4520B	0	NA	NA	NA	NA	NA
ELSPBP1	0	NA	NA	NA	NA	NA
CCL1	0	NA	NA	NA	NA	NA
CCL7	0	NA	NA	NA	NA	NA
GJA10	0	NA	NA	NA	NA	NA
MIR5698	0	NA	NA	NA	NA	NA
MIR5696	0	NA	NA	NA	NA	NA
MIR3690	0	NA	NA	NA	NA	NA
LINC00112	0	NA	NA	NA	NA	NA
LINC00111	0	NA	NA	NA	NA	NA
MIR1245A	0	NA	NA	NA	NA	NA
MIR1245B	0	NA	NA	NA	NA	NA
CSH1	0	NA	NA	NA	NA	NA
MIR888	0	NA	NA	NA	NA	NA
LOC64674	0	NA	NA	NA	NA	NA
CABS1	0	NA	NA	NA	NA	NA
LOC44024	0	NA	NA	NA	NA	NA
OR1J4	0	NA	NA	NA	NA	NA
CHEK2P2	0	NA	NA	NA	NA	NA
BLOC1S5-T	0	NA	NA	NA	NA	NA
LOC72912	0	NA	NA	NA	NA	NA
OR10A3	0	NA	NA	NA	NA	NA
OR10A6	0	NA	NA	NA	NA	NA
UMOD	0	NA	NA	NA	NA	NA
GOLGA2P2	0	NA	NA	NA	NA	NA
OR6Y1	0	NA	NA	NA	NA	NA
CRISP1	0	NA	NA	NA	NA	NA
OR6C65	0	NA	NA	NA	NA	NA
OR6C68	0	NA	NA	NA	NA	NA
OR2T29	0	NA	NA	NA	NA	NA
OR2T27	0	NA	NA	NA	NA	NA
MIR3194	0	NA	NA	NA	NA	NA

MIR922	0	NA	NA	NA	NA	NA
MIR548AA	0	NA	NA	NA	NA	NA
LOC44089	0	NA	NA	NA	NA	NA
LOC10013	0	NA	NA	NA	NA	NA
TGIF2LY	0	NA	NA	NA	NA	NA
TGIF2LX	0	NA	NA	NA	NA	NA
MIR3119-1	0	NA	NA	NA	NA	NA
MIR3119-2	0	NA	NA	NA	NA	NA
KRT28	0	NA	NA	NA	NA	NA
KRT26	0	NA	NA	NA	NA	NA
KRT27	0	NA	NA	NA	NA	NA
FLJ43315	0	NA	NA	NA	NA	NA
SNORD115	0	NA	NA	NA	NA	NA
SNORD115	0	NA	NA	NA	NA	NA
SNORD115	0	NA	NA	NA	NA	NA
SNORD115	0	NA	NA	NA	NA	NA
SNORD115	0	NA	NA	NA	NA	NA
SNORD115	0	NA	NA	NA	NA	NA
BOLA2B	0	NA	NA	NA	NA	NA
OR8I2	0	NA	NA	NA	NA	NA
BHLHE23	0	NA	NA	NA	NA	NA
OR2B3	0	NA	NA	NA	NA	NA
OSTCP1	0	NA	NA	NA	NA	NA
MIR4315-2	0	NA	NA	NA	NA	NA
SOX14	0	NA	NA	NA	NA	NA
C4A	0	NA	NA	NA	NA	NA
C4B	0	NA	NA	NA	NA	NA
MIR199A2	0	NA	NA	NA	NA	NA
TM4SF4	0	NA	NA	NA	NA	NA
LOC44070	0	NA	NA	NA	NA	NA
MIR518C	0	NA	NA	NA	NA	NA
MIR518B	0	NA	NA	NA	NA	NA
MIR518E	0	NA	NA	NA	NA	NA
MIR518D	0	NA	NA	NA	NA	NA
MIR518F	0	NA	NA	NA	NA	NA
MIR5186	0	NA	NA	NA	NA	NA
SNORD73A	0	NA	NA	NA	NA	NA
MIR4278	0	NA	NA	NA	NA	NA
MIR4272	0	NA	NA	NA	NA	NA
MIR4273	0	NA	NA	NA	NA	NA
MIR4275	0	NA	NA	NA	NA	NA
MIR4276	0	NA	NA	NA	NA	NA
MIR4277	0	NA	NA	NA	NA	NA
OR5K4	0	NA	NA	NA	NA	NA
OR5K3	0	NA	NA	NA	NA	NA
LINC00608	0	NA	NA	NA	NA	NA
OR4C6	0	NA	NA	NA	NA	NA

OR4C3	0 NA	NA	NA	NA	NA
UBE2DNL	0 NA	NA	NA	NA	NA
FAM90A27	0 NA	NA	NA	NA	NA
TTTY7B	0 NA	NA	NA	NA	NA
OR4K15	0 NA	NA	NA	NA	NA
OR4K14	0 NA	NA	NA	NA	NA
OR4K17	0 NA	NA	NA	NA	NA
OR4K13	0 NA	NA	NA	NA	NA
DAOA-AS1	0 NA	NA	NA	NA	NA
PPP1R2P9	0 NA	NA	NA	NA	NA
CELA3B	0 NA	NA	NA	NA	NA
CELA3A	0 NA	NA	NA	NA	NA
RPL21P28	0 NA	NA	NA	NA	NA
PRAC	0 NA	NA	NA	NA	NA
EEF1E1-M	0 NA	NA	NA	NA	NA
UGT1A8	0 NA	NA	NA	NA	NA
UGT1A9	0 NA	NA	NA	NA	NA
UGT1A4	0 NA	NA	NA	NA	NA
UGT1A5	0 NA	NA	NA	NA	NA
UGT1A1	0 NA	NA	NA	NA	NA
UGT1A3	0 NA	NA	NA	NA	NA
OR2A42	0 NA	NA	NA	NA	NA
MIR3150A	0 NA	NA	NA	NA	NA
MIR3150B	0 NA	NA	NA	NA	NA
SNORD109	0 NA	NA	NA	NA	NA
SNORD109	0 NA	NA	NA	NA	NA
XAGE2B	0 NA	NA	NA	NA	NA
KRTAP10-8	0 NA	NA	NA	NA	NA
MAGEA9	0 NA	NA	NA	NA	NA
MAGEA3	0 NA	NA	NA	NA	NA
MAGEA2	0 NA	NA	NA	NA	NA
MAGEA1	0 NA	NA	NA	NA	NA
MAGEA6	0 NA	NA	NA	NA	NA
MAGEA5	0 NA	NA	NA	NA	NA
MAGEA4	0 NA	NA	NA	NA	NA
TEX13A	0 NA	NA	NA	NA	NA
MIR767	0 NA	NA	NA	NA	NA
MIR764	0 NA	NA	NA	NA	NA
MIR762	0 NA	NA	NA	NA	NA
MIR498	0 NA	NA	NA	NA	NA
ERCC-0005	0 NA	NA	NA	NA	NA
ERCC-0014	0 NA	NA	NA	NA	NA
ERCC-0014	0 NA	NA	NA	NA	NA
ERCC-0014	0 NA	NA	NA	NA	NA
SNORD64	0 NA	NA	NA	NA	NA
IPO11-LRR	0 NA	NA	NA	NA	NA
FAM46D	0 NA	NA	NA	NA	NA

MIR4435-2	0	NA	NA	NA	NA	NA
MIR4435-1	0	NA	NA	NA	NA	NA
MIR2054	0	NA	NA	NA	NA	NA
C10orf40	0	NA	NA	NA	NA	NA
DEFT1P2	0	NA	NA	NA	NA	NA
MIR526B	0	NA	NA	NA	NA	NA
SCARNA13	0	NA	NA	NA	NA	NA
MIR4679-1	0	NA	NA	NA	NA	NA
MIR4679-2	0	NA	NA	NA	NA	NA
SPINK2	0	NA	NA	NA	NA	NA
TMPRSS4-	0	NA	NA	NA	NA	NA
LINC00433	0	NA	NA	NA	NA	NA
IL22RA2	0	NA	NA	NA	NA	NA
MIR1283-1	0	NA	NA	NA	NA	NA
MIR1283-2	0	NA	NA	NA	NA	NA
CD200R1L	0	NA	NA	NA	NA	NA
MIR802	0	NA	NA	NA	NA	NA
CD1E	0	NA	NA	NA	NA	NA
CD1B	0	NA	NA	NA	NA	NA
SPATA31A	0	NA	NA	NA	NA	NA
SPATA31A	0	NA	NA	NA	NA	NA
SPATA31A	0	NA	NA	NA	NA	NA
SPATA31A	0	NA	NA	NA	NA	NA
SPATA31A	0	NA	NA	NA	NA	NA
SPATA31A	0	NA	NA	NA	NA	NA
SPAM1	0	NA	NA	NA	NA	NA
MIR510	0	NA	NA	NA	NA	NA
OR4F16	0	NA	NA	NA	NA	NA
OR4F17	0	NA	NA	NA	NA	NA
HIST2H4B	0	NA	NA	NA	NA	NA
HIST2H4A	0	NA	NA	NA	NA	NA
OR2Z1	0	NA	NA	NA	NA	NA
CBWD5	0	NA	NA	NA	NA	NA
OR51G2	0	NA	NA	NA	NA	NA
OR51G1	0	NA	NA	NA	NA	NA
SERPINA12	0	NA	NA	NA	NA	NA
SERPINA11	0	NA	NA	NA	NA	NA
CLC	0	NA	NA	NA	NA	NA
OR7A10	0	NA	NA	NA	NA	NA
KRTAP27-1	0	NA	NA	NA	NA	NA
DUX4	0	NA	NA	NA	NA	NA
DUX2	0	NA	NA	NA	NA	NA
MIR4417	0	NA	NA	NA	NA	NA
GAGE2D	0	NA	NA	NA	NA	NA
GAGE2E	0	NA	NA	NA	NA	NA
GAGE2C	0	NA	NA	NA	NA	NA
MIR3913-2	0	NA	NA	NA	NA	NA

MIR3913-1	0<br>NA	NA	NA	NA	NA
LOC10013	0<br>NA	NA	NA	NA	NA
CPXCR1	0<br>NA	NA	NA	NA	NA
C1orf151-	0<br>NA	NA	NA	NA	NA
XKRY2	0<br>NA	NA	NA	NA	NA
MAGEB18	0<br>NA	NA	NA	NA	NA
MAGEB10	0<br>NA	NA	NA	NA	NA
MAGEB16	0<br>NA	NA	NA	NA	NA
VCY1B	0<br>NA	NA	NA	NA	NA
GIP	0<br>NA	NA	NA	NA	NA
CXorf61	0<br>NA	NA	NA	NA	NA
CXorf66	0<br>NA	NA	NA	NA	NA
MIR3529	0<br>NA	NA	NA	NA	NA
TVP23C-C	0<br>NA	NA	NA	NA	NA
MIR374C	0<br>NA	NA	NA	NA	NA
SNAR-C5	0<br>NA	NA	NA	NA	NA
SNAR-C4	0<br>NA	NA	NA	NA	NA
SNAR-C1	0<br>NA	NA	NA	NA	NA
SNAR-C2	0<br>NA	NA	NA	NA	NA
LCE1F	0<br>NA	NA	NA	NA	NA
LCE1D	0<br>NA	NA	NA	NA	NA
LCE1C	0<br>NA	NA	NA	NA	NA
DSCR10	0<br>NA	NA	NA	NA	NA
MIR4330	0<br>NA	NA	NA	NA	NA
SMN2	0<br>NA	NA	NA	NA	NA
SMN1	0<br>NA	NA	NA	NA	NA
MIR3646	0<br>NA	NA	NA	NA	NA
OR5D18	0<br>NA	NA	NA	NA	NA

CXorf51B	0<br>NA	NA	NA	NA	NA	
NR1H4	0<br>NA	NA	NA	NA	NA	
AGSK1	0<br>NA	NA	NA	NA	NA	
TTTY8B	0<br>NA	NA	NA	NA	NA	
ZCCHC5	0<br>NA	NA	NA	NA	NA	
OR11H2	0<br>NA	NA	NA	NA	NA	
OR11H1	0<br>NA	NA	NA	NA	NA	
SLX1B-SUL	0<br>NA	NA	NA	NA	NA	
MIR4687	0<br>NA	NA	NA	NA	NA	
MIR4686	0<br>NA	NA	NA	NA	NA	
MIR4680	0<br>NA	NA	NA	NA	NA	
MIR4683	0<br>NA	NA	NA	NA	NA	
SSX4B	0<br>NA	NA	NA	NA	NA	
MIR653	0<br>NA	NA	NA	NA	NA	
FAM226A	0<br>NA	NA	NA	NA	NA	
FAM226B	0<br>NA	NA	NA	NA	NA	
MIR1248	0<br>NA	NA	NA	NA	NA	
MIR1246	0<br>NA	NA	NA	NA	NA	
MIR3689B	0<br>NA	NA	NA	NA	NA	

MIR3689C	0	NA	NA	NA	NA	NA
MIR3689E	0	NA	NA	NA	NA	NA
FAM197Y5	0	NA	NA	NA	NA	NA
LOC34009	0	NA	NA	NA	NA	NA
BLOC1S1-R	0	NA	NA	NA	NA	NA
CD99	0	NA	NA	NA	NA	NA
DEFB103B	0	NA	NA	NA	NA	NA
DEFB103A	0	NA	NA	NA	NA	NA
LOC10050	0	NA	NA	NA	NA	NA
CT47B1	0	NA	NA	NA	NA	NA
C14orf177	0	NA	NA	NA	NA	NA
USP17L15	0	NA	NA	NA	NA	NA
USP17L13	0	NA	NA	NA	NA	NA
USP17L12	0	NA	NA	NA	NA	NA
USP17L11	0	NA	NA	NA	NA	NA
USP17L19	0	NA	NA	NA	NA	NA
USP17L18	0	NA	NA	NA	NA	NA
RNU6-34	0	NA	NA	NA	NA	NA
RNU6-35	0	NA	NA	NA	NA	NA
CDY2A	0	NA	NA	NA	NA	NA
RNU6-33	0	NA	NA	NA	NA	NA
SPANXB1	0	NA	NA	NA	NA	NA
SPANXB2	0	NA	NA	NA	NA	NA
MIRLET7B	0	NA	NA	NA	NA	NA
SNORD116	0	NA	NA	NA	NA	NA
SNORD116	0	NA	NA	NA	NA	NA
SNORD116	0	NA	NA	NA	NA	NA
TCEB3CL2	0	NA	NA	NA	NA	NA
MIR4497	0	NA	NA	NA	NA	NA
MIR4490	0	NA	NA	NA	NA	NA
MIR4491	0	NA	NA	NA	NA	NA
MIR4492	0	NA	NA	NA	NA	NA
RHOXF2B	0	NA	NA	NA	NA	NA
MIR449B	0	NA	NA	NA	NA	NA
MIR449C	0	NA	NA	NA	NA	NA
ELF5	0	NA	NA	NA	NA	NA
MIR3148	0	NA	NA	NA	NA	NA
MIR3142	0	NA	NA	NA	NA	NA
MIR3141	0	NA	NA	NA	NA	NA
MIR3147	0	NA	NA	NA	NA	NA
VRTN	0	NA	NA	NA	NA	NA
MGC7087	0	NA	NA	NA	NA	NA
BPESC1	0	NA	NA	NA	NA	NA
FRG2	0	NA	NA	NA	NA	NA
OR5P3	0	NA	NA	NA	NA	NA
OR5P2	0	NA	NA	NA	NA	NA
SIX6	0	NA	NA	NA	NA	NA

LINC00309	*0 NA*	*NA*	*NA*	*NA*	*NA*
LINC00303	0 NA	NA	NA	NA	*NA*
LINC00301	0 NA	NA	NA	NA	*NA*
LINC00305	0 NA	NA	NA	NA	*NA*
ERCC-0003	0 NA	NA	NA	NA	*NA*
ERCC-0003	0 NA	NA	NA	NA	*NA*
ERCC-0003	0 NA	NA	NA	NA	*NA*
ERCC-0003	0 NA	NA	NA	NA	*NA*
ERCC-0003	0 NA	NA	NA	NA	*NA*
LOC28340	0 NA	NA	NA	NA	*NA*
KRTAP10-1	0 NA	NA	NA	NA	*NA*
GJA9	0 NA	NA	NA	NA	*NA*
CFHR2	0 NA	NA	NA	NA	*NA*
CFHR3	0 NA	NA	NA	NA	*NA*
CFHR1	0 NA	NA	NA	NA	*NA*
CFHR4	0 NA	NA	NA	NA	*NA*
CFHR5	0 NA	NA	NA	NA	*NA*
DEFA10P	0 NA	NA	NA	NA	*NA*
HIST2H2A	0 NA	NA	NA	NA	*NA*
HIST2H2A	0 NA	NA	NA	NA	*NA*
TSSK2	0 NA	NA	NA	NA	*NA*
PIGY	0 NA	NA	NA	NA	*NA*
FAM47B	0 NA	NA	NA	NA	*NA*
RAB9BP1	0 NA	NA	NA	NA	*NA*
MIR4510	0 NA	NA	NA	NA	*NA*
MIR451A	0 NA	NA	NA	NA	*NA*
PMF1-BGL	0 NA	NA	NA	NA	*NA*
MIR143	0 NA	NA	NA	NA	*NA*
MIR141	0 NA	NA	NA	NA	*NA*
MIR145	0 NA	NA	NA	NA	*NA*
AKAP17A	0 NA	NA	NA	NA	*NA*
PDILT	0 NA	NA	NA	NA	*NA*
FABP9	0 NA	NA	NA	NA	*NA*
TCEB3C	0 NA	NA	NA	NA	*NA*
TGIF2-C20	0 NA	NA	NA	NA	*NA*
AKAP4	0 NA	NA	NA	NA	*NA*
CETN1	0 NA	NA	NA	NA	*NA*
RBMY1A1	0 NA	NA	NA	NA	*NA*
P2RY8	0 NA	NA	NA	NA	*NA*
GUCA1C	0 NA	NA	NA	NA	*NA*
CT47A6	0 NA	NA	NA	NA	*NA*
CT47A5	0 NA	NA	NA	NA	*NA*
ADAM3A	0 NA	NA	NA	NA	*NA*
FFAR3	0 NA	NA	NA	NA	*NA*
RAET1L	0 NA	NA	NA	NA	*NA*
MIR509-1	0 NA	NA	NA	NA	*NA*
LINC00592	*0 NA*	*NA*	*NA*	*NA*	*NA*

MEF2BNB-	0	NA	NA	NA	NA	NA
SPDYE2	0	NA	NA	NA	NA	NA
TNFSF12-T	0	NA	NA	NA	NA	NA
LOC38789	0	NA	NA	NA	NA	NA
GRXCR1	0	NA	NA	NA	NA	NA
GRXCR2	0	NA	NA	NA	NA	NA
USP17L9P	0	NA	NA	NA	NA	NA
ACTRT2	0	NA	NA	NA	NA	NA
REG3G	0	NA	NA	NA	NA	NA
REG3A	0	NA	NA	NA	NA	NA
MIR4763	0	NA	NA	NA	NA	NA
OR2A1	0	NA	NA	NA	NA	NA
CHKB-CPT	0	NA	NA	NA	NA	NA
IL36A	0	NA	NA	NA	NA	NA
IL36B	0	NA	NA	NA	NA	NA
IL36G	0	NA	NA	NA	NA	NA
GAGE2A	0	NA	NA	NA	NA	NA
GAGE2B	0	NA	NA	NA	NA	NA
PRR20A	0	NA	NA	NA	NA	NA
PRR20B	0	NA	NA	NA	NA	NA
PRR20C	0	NA	NA	NA	NA	NA
PRR20D	0	NA	NA	NA	NA	NA
PRR20E	0	NA	NA	NA	NA	NA
ACTL9	0	NA	NA	NA	NA	NA
ACTL8	0	NA	NA	NA	NA	NA
OR5H6	0	NA	NA	NA	NA	NA
OR5H1	0	NA	NA	NA	NA	NA
OR5H2	0	NA	NA	NA	NA	NA
RAB4B	0	NA	NA	NA	NA	NA
MIR650	0	NA	NA	NA	NA	NA
OR4N2	0	NA	NA	NA	NA	NA
PRY2	0	NA	NA	NA	NA	NA
OR4N5	0	NA	NA	NA	NA	NA
NARR	0	NA	NA	NA	NA	NA
H2AFB1	0	NA	NA	NA	NA	NA
HYI	0	NA	NA	NA	NA	NA
EIF4A1	0	NA	NA	NA	NA	NA
CDX4	0	NA	NA	NA	NA	NA
NKX2-3	0	NA	NA	NA	NA	NA
NKX2-6	0	NA	NA	NA	NA	NA
OR2T3	0	NA	NA	NA	NA	NA
KLHL38	0	NA	NA	NA	NA	NA
OR5AS1	0	NA	NA	NA	NA	NA
ADIPOQ-A	0	NA	NA	NA	NA	NA
RPS17	0	NA	NA	NA	NA	NA
RHOXF2	0	NA	NA	NA	NA	NA
LOC10028	0	NA	NA	NA	NA	NA

PLCXD1	0	NA	NA	NA	NA	NA
SAA2-SAA	0	NA	NA	NA	NA	NA
GGTLC1	0	NA	NA	NA	NA	NA
MAGEB2	0	NA	NA	NA	NA	NA
MAGEB1	0	NA	NA	NA	NA	NA
MAGEB6	0	NA	NA	NA	NA	NA
MAGEB4	0	NA	NA	NA	NA	NA
MAGEB5	0	NA	NA	NA	NA	NA
LINC00462	0	NA	NA	NA	NA	NA
TRIM64	0	NA	NA	NA	NA	NA
ERCC-0017	0	NA	NA	NA	NA	NA
ERCC-0017	0	NA	NA	NA	NA	NA
PYDC2	0	NA	NA	NA	NA	NA
CRLF2	0	NA	NA	NA	NA	NA
LOC10013	0	NA	NA	NA	NA	NA
MIR200A	0	NA	NA	NA	NA	NA
MIR200B	0	NA	NA	NA	NA	NA
MIR3928	0	NA	NA	NA	NA	NA
MIR3924	0	NA	NA	NA	NA	NA
S100A7L2	0	NA	NA	NA	NA	NA
OR7C2	0	NA	NA	NA	NA	NA
MIR4657	0	NA	NA	NA	NA	NA
MIR4652	0	NA	NA	NA	NA	NA
OR6C3	0	NA	NA	NA	NA	NA
OR6C2	0	NA	NA	NA	NA	NA
OR6C1	0	NA	NA	NA	NA	NA
OR6C6	0	NA	NA	NA	NA	NA
OR6C4	0	NA	NA	NA	NA	NA
LINC00261	0	NA	NA	NA	NA	NA
LOC25502	0	NA	NA	NA	NA	NA
OR8D1	0	NA	NA	NA	NA	NA
OR8D2	0	NA	NA	NA	NA	NA
UGT2A3	0	NA	NA	NA	NA	NA
UGT2A2	0	NA	NA	NA	NA	NA
OR2Y1	0	NA	NA	NA	NA	NA
GOLGA6L6	0	NA	NA	NA	NA	NA
APOBEC3A	0	NA	NA	NA	NA	NA
ULK4P1	0	NA	NA	NA	NA	NA
ULK4P2	0	NA	NA	NA	NA	NA
FXYD6-FXY	0	NA	NA	NA	NA	NA
KRTAP22-2	0	NA	NA	NA	NA	NA
IDO2	0	NA	NA	NA	NA	NA
MIR4280	0	NA	NA	NA	NA	NA
SNORA59A	0	NA	NA	NA	NA	NA
MIR4287	0	NA	NA	NA	NA	NA
MIR106A	0	NA	NA	NA	NA	NA
MIR3688-1	0	NA	NA	NA	NA	NA

MIR3688-2	0	NA	NA	NA	NA	NA
MIR548Y	0	NA	NA	NA	NA	NA
MIR548Z	0	NA	NA	NA	NA	NA
LINC00651	0	NA	NA	NA	NA	NA
MIR548C	0	NA	NA	NA	NA	NA
MIR548M	0	NA	NA	NA	NA	NA
LINC00659	0	NA	NA	NA	NA	NA
HOXC9	0	NA	NA	NA	NA	NA
HOXC8	0	NA	NA	NA	NA	NA
OR4F3	0	NA	NA	NA	NA	NA
OR4F6	0	NA	NA	NA	NA	NA
OR4F5	0	NA	NA	NA	NA	NA
MIR3179-2	0	NA	NA	NA	NA	NA
MIR3179-3	0	NA	NA	NA	NA	NA
MIR3179-1	0	NA	NA	NA	NA	NA
UBE2Q2P2	0	NA	NA	NA	NA	NA
TTTY2B	0	NA	NA	NA	NA	NA
TTTY21	0	NA	NA	NA	NA	NA
TTTY20	0	NA	NA	NA	NA	NA
TTTY23	0	NA	NA	NA	NA	NA
TTTY22	0	NA	NA	NA	NA	NA
TMEM239	0	NA	NA	NA	NA	NA
MIR519B	0	NA	NA	NA	NA	NA
C21orf54	0	NA	NA	NA	NA	NA
CCT8L2	0	NA	NA	NA	NA	NA
FSBP	0	NA	NA	NA	NA	NA
MIR548I3	0	NA	NA	NA	NA	NA
MIR1184-1	0	NA	NA	NA	NA	NA
MIR1184-2	0	NA	NA	NA	NA	NA
MIR1184-3	0	NA	NA	NA	NA	NA
UGT2B28	0	NA	NA	NA	NA	NA
OR1N1	0	NA	NA	NA	NA	NA
MIR1244-3	0	NA	NA	NA	NA	NA
MIR1244-2	0	NA	NA	NA	NA	NA
MIR1244-1	0	NA	NA	NA	NA	NA
CDK3	0	NA	NA	NA	NA	NA
CKLF-CMT	0	NA	NA	NA	NA	NA
CCL15-CCL	0	NA	NA	NA	NA	NA
IQCJ-SCHIP	0	NA	NA	NA	NA	NA
KRTAP23-1	0	NA	NA	NA	NA	NA
MIR3689D	0	NA	NA	NA	NA	NA
MIR3130-2	0	NA	NA	NA	NA	NA
MIR3130-1	0	NA	NA	NA	NA	NA
LOC64681	0	NA	NA	NA	NA	NA
LOC72871	0	NA	NA	NA	NA	NA
MIR515-1	0	NA	NA	NA	NA	NA
MIR515-2	0	NA	NA	NA	NA	NA

RBMY2EP	0	NA	NA	NA	NA	NA
FTH1P18	0	NA	NA	NA	NA	NA
RBMY3AP	0	NA	NA	NA	NA	NA
MYH13	0	NA	NA	NA	NA	NA
OR6K3	0	NA	NA	NA	NA	NA
OR6K2	0	NA	NA	NA	NA	NA
LOC10028	0	NA	NA	NA	NA	NA
MIF	0	NA	NA	NA	NA	NA
MIR1279	0	NA	NA	NA	NA	NA
FAM223B	0	NA	NA	NA	NA	NA
CSPG4P1Y	0	NA	NA	NA	NA	NA
SLAMF1	0	NA	NA	NA	NA	NA
DEFB104B	0	NA	NA	NA	NA	NA
DEFB104A	0	NA	NA	NA	NA	NA
SPATA31D	0	NA	NA	NA	NA	NA
SPATA31D	0	NA	NA	NA	NA	NA
LINC00547	0	NA	NA	NA	NA	NA
APOBEC1	0	NA	NA	NA	NA	NA
USP17L6P	0	NA	NA	NA	NA	NA
TMEM183	0	NA	NA	NA	NA	NA
C1QTNF5	0	NA	NA	NA	NA	NA
LOC1720	0	NA	NA	NA	NA	NA
MIR1233-1	0	NA	NA	NA	NA	NA
MIR4460	0	NA	NA	NA	NA	NA
MIR4468	0	NA	NA	NA	NA	NA
BMS1P1	0	NA	NA	NA	NA	NA
BMS1P2	0	NA	NA	NA	NA	NA
BMS1P5	0	NA	NA	NA	NA	NA
BMS1P6	0	NA	NA	NA	NA	NA
NT5C1B-R	0	NA	NA	NA	NA	NA
LOC10050	0	NA	NA	NA	NA	NA
FAM90A7	0	NA	NA	NA	NA	NA
ERCC-0006	0	NA	NA	NA	NA	NA
ERCC-0006	0	NA	NA	NA	NA	NA
MIR196B	0	NA	NA	NA	NA	NA
GKN1	0	NA	NA	NA	NA	NA
GKN2	0	NA	NA	NA	NA	NA
MIR375	0	NA	NA	NA	NA	NA
MIR372	0	NA	NA	NA	NA	NA
MIR373	0	NA	NA	NA	NA	NA
OR4Q3	0	NA	NA	NA	NA	NA
ERCC-0000	0	NA	NA	NA	NA	NA
ERCC-0000	0	NA	NA	NA	NA	NA
ERCC-0000	0	NA	NA	NA	NA	NA
ERCC-0000	0	NA	NA	NA	NA	NA
OR2A9P	0	NA	NA	NA	NA	NA
CCR3	0	NA	NA	NA	NA	NA

CCDC169-	0	NA	NA	NA	NA	NA
CLDN22	0	NA	NA	NA	NA	NA
XAGE5	0	NA	NA	NA	NA	NA
XAGE2	0	NA	NA	NA	NA	NA
MIR4650-2	0	NA	NA	NA	NA	NA
MIR4650-1	0	NA	NA	NA	NA	NA
CORO7-PA	0	NA	NA	NA	NA	NA
PLAC1L	0	NA	NA	NA	NA	NA
MAGEA10	0	NA	NA	NA	NA	NA
CSN2	0	NA	NA	NA	NA	NA
CSN3	0	NA	NA	NA	NA	NA
BARX1	0	NA	NA	NA	NA	NA
MIR19B2	0	NA	NA	NA	NA	NA
MIR19B1	0	NA	NA	NA	NA	NA
PGLYRP2	0	NA	NA	NA	NA	NA
PGLYRP4	0	NA	NA	NA	NA	NA
RGS21	0	NA	NA	NA	NA	NA
LOC10013	0	NA	NA	NA	NA	NA
OR10K2	0	NA	NA	NA	NA	NA
OR10K1	0	NA	NA	NA	NA	NA
SYS1-DBN	0	NA	NA	NA	NA	NA
PHOSPHO	0	NA	NA	NA	NA	NA
OR6S1	0	NA	NA	NA	NA	NA
KRTAP6-3	0	NA	NA	NA	NA	NA
KRTAP6-2	0	NA	NA	NA	NA	NA
KRTAP6-1	0	NA	NA	NA	NA	NA
MIR196A2	0	NA	NA	NA	NA	NA
MIR196A1	0	NA	NA	NA	NA	NA
PRSS58	0	NA	NA	NA	NA	NA
KPRP	0	NA	NA	NA	NA	NA
KRTAP15-1	0	NA	NA	NA	NA	NA
LOC64913	0	NA	NA	NA	NA	NA
PGA3	0	NA	NA	NA	NA	NA
PGA4	0	NA	NA	NA	NA	NA
VCX3A	0	NA	NA	NA	NA	NA
LOC10028	0	NA	NA	NA	NA	NA
PCNA-AS1	0	NA	NA	NA	NA	NA
ENAM	0	NA	NA	NA	NA	NA
LOC10009	0	NA	NA	NA	NA	NA
MIR148A	0	NA	NA	NA	NA	NA
TARM1	0	NA	NA	NA	NA	NA
SNAR-I	0	NA	NA	NA	NA	NA
MIR4752	0	NA	NA	NA	NA	NA
MIR4751	0	NA	NA	NA	NA	NA
MIR4756	0	NA	NA	NA	NA	NA
FGFBP1	0	NA	NA	NA	NA	NA
ST20-MTH	0	NA	NA	NA	NA	NA

MIR3197	0	NA	NA	NA	NA	NA
MIR3191	0	NA	NA	NA	NA	NA
ASMTL-AS	0	NA	NA	NA	NA	NA
OR10AG1	0	NA	NA	NA	NA	NA
GPR139	0	NA	NA	NA	NA	NA
MFRP	0	NA	NA	NA	NA	NA
MIR662	0	NA	NA	NA	NA	NA
LINC00379	0	NA	NA	NA	NA	NA
GDF1	0	NA	NA	NA	NA	NA
SCXB	0	NA	NA	NA	NA	NA
SCXA	0	NA	NA	NA	NA	NA
UBTFL1	0	NA	NA	NA	NA	NA
ERCC-0008	0	NA	NA	NA	NA	NA
ERCC-0008	0	NA	NA	NA	NA	NA
ASIC5	0	NA	NA	NA	NA	NA
SNORD116	0	NA	NA	NA	NA	NA
SNORD116	0	NA	NA	NA	NA	NA
FOXD4L4	0	NA	NA	NA	NA	NA
FOXD4L2	0	NA	NA	NA	NA	NA
SOAT2	0	NA	NA	NA	NA	NA
C1orf68	0	NA	NA	NA	NA	NA
C1orf65	0	NA	NA	NA	NA	NA
CSF2	0	NA	NA	NA	NA	NA
LOC10050	0	NA	NA	NA	NA	NA
MIR1306	0	NA	NA	NA	NA	NA
ZG16B	0	NA	NA	NA	NA	NA
C15orf38-	0	NA	NA	NA	NA	NA
RNF186	0	NA	NA	NA	NA	NA
MIR433	0	NA	NA	NA	NA	NA
MIR432	0	NA	NA	NA	NA	NA
MIR431	0	NA	NA	NA	NA	NA
ERCC-0012	0	NA	NA	NA	NA	NA
ERCC-0012	0	NA	NA	NA	NA	NA
ERCC-0012	0	NA	NA	NA	NA	NA
CSAG3	0	NA	NA	NA	NA	NA
CSAG2	0	NA	NA	NA	NA	NA
FAM47E-S	0	NA	NA	NA	NA	NA
OR4F13P	0	NA	NA	NA	NA	NA
MIR9-2	0	NA	NA	NA	NA	NA
OR10X1	0	NA	NA	NA	NA	NA
LOC10028	0	NA	NA	NA	NA	NA
TBC1D3	0	NA	NA	NA	NA	NA
MIR4770	0	NA	NA	NA	NA	NA
OR7D4	0	NA	NA	NA	NA	NA
MIR4665	0	NA	NA	NA	NA	NA
CXXC1P1	0	NA	NA	NA	NA	NA
HNRNPCL1	0	NA	NA	NA	NA	NA

TMX2-CTN	0	NA	NA	NA	NA	NA
LINC00421	0	NA	NA	NA	NA	NA
ZNF705B	0	NA	NA	NA	NA	NA
TRIM49B	0	NA	NA	NA	NA	NA
TRIM49C	0	NA	NA	NA	NA	NA
SNORD115	0	NA	NA	NA	NA	NA
MIR509-3	0	NA	NA	NA	NA	NA
OR8G1	0	NA	NA	NA	NA	NA
OR8G2	0	NA	NA	NA	NA	NA
UGT2B7	0	NA	NA	NA	NA	NA
UGT2B4	0	NA	NA	NA	NA	NA
OR10A7	0	NA	NA	NA	NA	NA
TCP10L2	0	NA	NA	NA	NA	NA
MIR548H2	0	NA	NA	NA	NA	NA
TIFAB	0	NA	NA	NA	NA	NA
MIR4252	0	NA	NA	NA	NA	NA
MIR4255	0	NA	NA	NA	NA	NA
OR5M8	0	NA	NA	NA	NA	NA
OR5M9	0	NA	NA	NA	NA	NA
OR5M1	0	NA	NA	NA	NA	NA
XKRY	0	NA	NA	NA	NA	NA
MIR5707	0	NA	NA	NA	NA	NA
MIR5705	0	NA	NA	NA	NA	NA
MIR5704	0	NA	NA	NA	NA	NA
MIR5703	0	NA	NA	NA	NA	NA
MIR5700	0	NA	NA	NA	NA	NA
MIR5708	0	NA	NA	NA	NA	NA
XKR3	0	NA	NA	NA	NA	NA
AMTN	0	NA	NA	NA	NA	NA
OR4A5	0	NA	NA	NA	NA	NA
MIR4289	0	NA	NA	NA	NA	NA
PRR23B	0	NA	NA	NA	NA	NA
SNORA59B	0	NA	NA	NA	NA	NA
OR11H12	0	NA	NA	NA	NA	NA
C4B_2	0	NA	NA	NA	NA	NA
IL1F10	0	NA	NA	NA	NA	NA
MIR3545	0	NA	NA	NA	NA	NA
NBPF11	0	NA	NA	NA	NA	NA
SLC25A6	0	NA	NA	NA	NA	NA
TTTY9A	0	NA	NA	NA	NA	NA
MIR5003	0	NA	NA	NA	NA	NA
MIR5007	0	NA	NA	NA	NA	NA
RPL21	0	NA	NA	NA	NA	NA
LOC64414	0	NA	NA	NA	NA	NA
UGT1A10	0	NA	NA	NA	NA	NA
SNORA11E	0	NA	NA	NA	NA	NA
SNORA11D	0	NA	NA	NA	NA	NA

IQCF4	0	NA	NA	NA	NA	NA
TFF1	0	NA	NA	NA	NA	NA
TFF2	0	NA	NA	NA	NA	NA
MIR4776-2	0	NA	NA	NA	NA	NA
MIR4776-1	0	NA	NA	NA	NA	NA
FTHL17	0	NA	NA	NA	NA	NA
GIMAP1-G	0	NA	NA	NA	NA	NA
OR52R1	0	NA	NA	NA	NA	NA
OR10P1	0	NA	NA	NA	NA	NA
CYP11B2	0	NA	NA	NA	NA	NA
CYP11B1	0	NA	NA	NA	NA	NA
SSX2B	0	NA	NA	NA	NA	NA
LELP1	0	NA	NA	NA	NA	NA
MIR3976	0	NA	NA	NA	NA	NA
MIR3974	0	NA	NA	NA	NA	NA
TBC1D3C	0	NA	NA	NA	NA	NA
TBC1D3F	0	NA	NA	NA	NA	NA
TBC1D3H	0	NA	NA	NA	NA	NA
CEACAM3	0	NA	NA	NA	NA	NA
CEACAM7	0	NA	NA	NA	NA	NA
C20orf141	0	NA	NA	NA	NA	NA
ERCC-0007	0	NA	NA	NA	NA	NA
MIR1226	0	NA	NA	NA	NA	NA
MIR1270-2	0	NA	NA	NA	NA	NA
MIR1270-1	0	NA	NA	NA	NA	NA
LOC14651	0	NA	NA	NA	NA	NA
VENTXP1	0	NA	NA	NA	NA	NA
SPRR2E	0	NA	NA	NA	NA	NA
SPRR2A	0	NA	NA	NA	NA	NA
SPRR2C	0	NA	NA	NA	NA	NA
SPRR2B	0	NA	NA	NA	NA	NA
KRT84	0	NA	NA	NA	NA	NA
KRT85	0	NA	NA	NA	NA	NA
SPACA7	0	NA	NA	NA	NA	NA
OR9G1	0	NA	NA	NA	NA	NA
OR9G9	0	NA	NA	NA	NA	NA
OR6N2	0	NA	NA	NA	NA	NA
H2AFB3	0	NA	NA	NA	NA	NA
H2AFB2	0	NA	NA	NA	NA	NA
MIR3158-1	0	NA	NA	NA	NA	NA
MIR3158-2	0	NA	NA	NA	NA	NA
OR2T5	0	NA	NA	NA	NA	NA
OR2T2	0	NA	NA	NA	NA	NA
CYP4F30P	0	NA	NA	NA	NA	NA
AMY1A	0	NA	NA	NA	NA	NA
AMY1C	0	NA	NA	NA	NA	NA
AMY1B	0	NA	NA	NA	NA	NA

RPS10-NU	0	NA	NA	NA	NA	NA
CSN1S1	0	NA	NA	NA	NA	NA
ERCC-0013	0	NA	NA	NA	NA	NA
XGPY2	0	NA	NA	NA	NA	NA
HOXC12	0	NA	NA	NA	NA	NA
KIR2DL3	0	NA	NA	NA	NA	NA
KIR2DL2	0	NA	NA	NA	NA	NA
KIR2DL4	0	NA	NA	NA	NA	NA
MIR378C	0	NA	NA	NA	NA	NA
MIR378F	0	NA	NA	NA	NA	NA
SPIC	0	NA	NA	NA	NA	NA
MIR4780	0	NA	NA	NA	NA	NA
MIR4788	0	NA	NA	NA	NA	NA
SIM1	0	NA	NA	NA	NA	NA
MIR4437	0	NA	NA	NA	NA	NA
MIR4432	0	NA	NA	NA	NA	NA
DEFT1P	0	NA	NA	NA	NA	NA
FLJ22763	0	NA	NA	NA	NA	NA
LOC10050	0	NA	NA	NA	NA	NA
BAGE4	0	NA	NA	NA	NA	NA
BAGE5	0	NA	NA	NA	NA	NA
BAGE2	0	NA	NA	NA	NA	NA
BAGE3	0	NA	NA	NA	NA	NA
VGLL1	0	NA	NA	NA	NA	NA
ALX1	0	NA	NA	NA	NA	NA
GPR31	0	NA	NA	NA	NA	NA
ERCC-0001	0	NA	NA	NA	NA	NA
CCER1	0	NA	NA	NA	NA	NA
MIR934	0	NA	NA	NA	NA	NA
LOC73227	0	NA	NA	NA	NA	NA
PP12613	0	NA	NA	NA	NA	NA
SOHLH2	0	NA	NA	NA	NA	NA
CLDN17	0	NA	NA	NA	NA	NA
MIR512-2	0	NA	NA	NA	NA	NA
MIR512-1	0	NA	NA	NA	NA	NA
LCE3D	0	NA	NA	NA	NA	NA
LCE3E	0	NA	NA	NA	NA	NA
LCE3A	0	NA	NA	NA	NA	NA
LCE3B	0	NA	NA	NA	NA	NA
LCE3C	0	NA	NA	NA	NA	NA
SNORD103	0	NA	NA	NA	NA	NA
SNORD103	0	NA	NA	NA	NA	NA
MIR22	0	NA	NA	NA	NA	NA
FAM138C	0	NA	NA	NA	NA	NA
FAM138A	0	NA	NA	NA	NA	NA
FAM138F	0	NA	NA	NA	NA	NA
FAM138E	0	NA	NA	NA	NA	NA

LINC00705	0	NA	NA	NA	NA	NA
IFNA10	0	NA	NA	NA	NA	NA
MIR3668	0	NA	NA	NA	NA	NA
MIR3665	0	NA	NA	NA	NA	NA
MIR3663	0	NA	NA	NA	NA	NA
MIR3660	0	NA	NA	NA	NA	NA
MIR3661	0	NA	NA	NA	NA	NA
GPX6	0	NA	NA	NA	NA	NA
MIR127	0	NA	NA	NA	NA	NA
MIR122	0	NA	NA	NA	NA	NA
LOC10050	0	NA	NA	NA	NA	NA
TISP43	0	NA	NA	NA	NA	NA
SAPCD1	0	NA	NA	NA	NA	NA
UNC93A	0	NA	NA	NA	NA	NA
HOTTIP	0	NA	NA	NA	NA	NA
OR10H2	0	NA	NA	NA	NA	NA
OR10H1	0	NA	NA	NA	NA	NA
OR10H4	0	NA	NA	NA	NA	NA
PPBPP2	0	NA	NA	NA	NA	NA
OR6N1	0	NA	NA	NA	NA	NA
STATH	0	NA	NA	NA	NA	NA
CT47A12	0	NA	NA	NA	NA	NA
CT47A11	0	NA	NA	NA	NA	NA
CT47A10	0	NA	NA	NA	NA	NA
MT4	0	NA	NA	NA	NA	NA
HPVC1	0	NA	NA	NA	NA	NA
TRIM51HP	0	NA	NA	NA	NA	NA
SNAR-A13	0	NA	NA	NA	NA	NA
SLC7A13	0	NA	NA	NA	NA	NA
NPC2	10.87535	-0.3948	0.084334	-4.68141	2.85E-06	NA
SP140	8.318395	-0.32588	0.083617	-3.89722	9.73E-05	NA
BTN2A2	119.5551	-0.31701	0.07391	-4.2891	1.79E-05	NA
LAMC2	70.2828	0.316661	0.083494	3.792595	0.000149	NA
FLJ39639	23.78436	-0.3153	0.084142	-3.74725	0.000179	NA
CSF3R	9.321749	-0.31105	0.083214	-3.73797	0.000186	NA
MUC4	37.28175	-0.30894	0.084873	-3.64003	0.000273	NA
SRPX2	3.930409	-0.30885	0.078143	-3.95235	7.74E-05	NA
VRK2	8.532187	-0.30782	0.08491	-3.62522	0.000289	NA
IGFBP3	33.72187	-0.30508	0.081917	-3.72428	0.000196	NA
KIF19	11.27218	-0.29829	0.081251	-3.67118	0.000241	NA
SCRG1	63.30684	-0.2962	0.076373	-3.87831	0.000105	NA
EPN3	15.96986	0.296095	0.084843	3.489938	0.000483	NA
INPP5D	31.21398	-0.28855	0.081037	-3.56074	0.00037	NA
PSRC1	30.21588	-0.28839	0.081881	-3.52209	0.000428	NA
AZGP1	16.75851	-0.28786	0.075777	-3.79877	0.000145	NA
DIRC3	12.33163	-0.28501	0.084906	-3.35671	0.000789	NA
SFTPA2	12.30249	0.283929	0.084468	3.361382	0.000776	NA

BLOC1S1	4.123903	-0.28364	0.081496	-3.48047	0.000501	NA
TDH	4.049512	-0.28188	0.078073	-3.61046	0.000306	NA
FOXO1	41.18968	-0.281	0.083048	-3.38354	0.000716	NA
HLA-F-AS1	14.82	-0.2809	0.083228	-3.37505	0.000738	NA
GPR89B	5.180241	-0.28029	0.082714	-3.3887	0.000702	NA
PRAM1	19.24648	-0.27892	0.081719	-3.41321	0.000642	NA
SLC14A1	49.94938	-0.27272	0.075784	-3.59863	0.00032	NA
CPLX3	390.2462	-0.27091	0.079155	-3.42253	0.00062	0.075062
STAG3	181.8008	-0.26949	0.072637	-3.71008	0.000207	NA
SLC11A1	13.72045	-0.26907	0.075057	-3.58492	0.000337	NA
MAP1LC3B	12.7694	-0.26893	0.083172	-3.23343	0.001223	NA
TMEM80	31.01095	-0.2678	0.082296	-3.25414	0.001137	NA
VEPH1	25.61297	-0.26778	0.075376	-3.55254	0.000382	NA
PNPLA7	162.2811	-0.26614	0.084649	-3.14405	0.001666	NA
MT3	202.2214	-0.26606	0.084724	-3.14028	0.001688	NA
BTN3A1	78.73431	-0.26446	0.07998	-3.3066	0.000944	NA
FCGR2A	11.18903	-0.26441	0.080618	-3.27983	0.001039	NA
PLCD1	45.79362	-0.2643	0.070369	-3.75598	0.000173	NA
PIEZO1	174.496	-0.26251	0.074243	-3.53586	0.000406	NA
ARHGEF10	109.5329	-0.26152	0.084469	-3.09605	0.001961	NA
EML3	59.7703	-0.26128	0.068592	-3.80923	0.000139	NA
FLJ38109	8.719315	-0.26074	0.084914	-3.07068	0.002136	NA
SELL	6.690499	-0.26064	0.079329	-3.28558	0.001018	NA
DPYD	14.46597	-0.26022	0.084835	-3.06733	0.00216	NA
CAPG	8.677412	-0.26002	0.084746	-3.06824	0.002153	NA
C3	157.3342	-0.25979	0.082924	-3.13285	0.001731	NA
GOLGA8A	724.0041	-0.25939	0.073556	-3.5265	0.000421	0.075062
MUC20	224.9654	-0.2584	0.084202	-3.0688	0.002149	NA
TXNIP	297.6847	-0.25801	0.084823	-3.04175	0.002352	NA
SYCP2L	51.1063	-0.25738	0.084849	-3.03338	0.002418	NA
ZNF28	115.0098	0.255507	0.055161	4.631992	3.62E-06	NA
LINC00320	17.08277	-0.25523	0.084084	-3.03538	0.002402	NA
USH1C	5.685834	-0.25319	0.082017	-3.08701	0.002022	NA
TRAM2	42.50874	0.252665	0.07285	3.468289	0.000524	NA
RPS21	94.67474	-0.25254	0.084879	-2.9753	0.002927	NA
EPHA1-AS	5.469615	-0.25193	0.077382	-3.2556	0.001132	NA
TYROBP	5.846809	-0.25103	0.07786	-3.22411	0.001264	NA
LMAN1L	152.0533	-0.25	0.079701	-3.13665	0.001709	NA
PPP1R1B	369.6548	-0.24971	0.079793	-3.12952	0.001751	0.101784
PLIN2	11.50994	-0.24888	0.083669	-2.97462	0.002934	NA
GLRA2	77.16311	0.245643	0.075387	3.258424	0.00112	NA
XKR8	29.30279	0.245304	0.076857	3.191685	0.001414	NA
SNAI2	31.30379	0.244376	0.083973	2.910185	0.003612	NA
ARAP3	86.82579	-0.24417	0.082773	-2.9499	0.003179	NA
SLC43A1	10.24591	-0.24403	0.083815	-2.91154	0.003597	NA
GOLGA2P5	67.45395	-0.24351	0.076337	-3.18986	0.001423	NA
PVRL1	112.6415	0.242864	0.068174	3.56242	0.000367	NA

ENOSF1	34.25103	-0.24263	0.084929	-2.85685	0.004279	NA
SNORD22	22.20465	-0.24106	0.082947	-2.90623	0.003658	NA
GPR123	22.74038	0.239941	0.084864	2.827357	0.004693	NA
COL9A1	19.50573	-0.23976	0.083987	-2.85476	0.004307	NA
SSC5D	57.13397	0.239737	0.079833	3.002983	0.002673	NA
SLC3A1	25.38944	-0.23967	0.082265	-2.91337	0.003575	NA
LGI4	166.9954	-0.23954	0.082724	-2.8956	0.003784	NA
LIG4	112.944	0.239287	0.072911	3.281907	0.001031	NA
HCLS1	23.22363	-0.23823	0.081355	-2.92827	0.003408	NA
FUOM	3.473972	-0.23797	0.077672	-3.06373	0.002186	NA
NRARP	12.33336	-0.23789	0.084258	-2.82339	0.004752	NA
CD27-AS1	7.548073	-0.23786	0.084542	-2.81349	0.004901	NA
C10orf114	11.33516	0.237537	0.084881	2.798465	0.005135	NA
CSPG4	40.1379	-0.23721	0.079185	-2.99564	0.002739	NA
RGS1	12.16696	-0.2361	0.063421	-3.72278	0.000197	NA
GCM2	17.98751	-0.23532	0.084883	-2.77225	0.005567	NA
SLC12A1	12.54072	-0.23524	0.084886	-2.7712	0.005585	NA
CCDC148	54.26592	0.2346	0.082715	2.836234	0.004565	NA
COL16A1	140.5443	-0.2346	0.083367	-2.81406	0.004892	NA
RPS6	937.653	-0.23455	0.083806	-2.79866	0.005132	0.151649
LOC61920	15.93238	-0.23436	0.082441	-2.8427	0.004473	NA
IL10RA	5.967805	-0.2338	0.0803	-2.91156	0.003596	NA
IL15	2.753181	-0.23362	0.072937	-3.20308	0.00136	NA
CDH23	299.6641	-0.2336	0.083594	-2.79446	0.005199	NA
FBXO45	198.2127	0.23358	0.059648	3.915994	9.00E-05	NA
ITGB4	175.5214	-0.23323	0.083887	-2.78031	0.005431	NA
ADM	8.627532	-0.23293	0.077355	-3.01118	0.002602	NA
SF3B5	18.40826	-0.23258	0.083727	-2.77783	0.005472	NA
RTN4RL1	44.28377	0.232031	0.07249	3.200868	0.00137	NA
LMOD1	69.9811	-0.23171	0.083916	-2.76118	0.005759	NA
C10orf54	43.71126	-0.23096	0.081745	-2.8254	0.004722	NA
HK2	11.67343	-0.23087	0.082792	-2.78857	0.005294	NA
LOC33865	85.60161	0.230176	0.072671	3.16738	0.001538	NA
NDUFA2	32.939	-0.2301	0.082587	-2.78613	0.005334	NA
SNORD45C	2.799525	-0.2299	0.075655	-3.03881	0.002375	NA
SHROOM3	530.301	0.229575	0.068614	3.34591	0.00082	0.084436
IL11RA	49.1828	-0.22879	0.06661	-3.43475	0.000593	NA
HLA-DQB1	7.671241	-0.22873	0.077363	-2.95656	0.003111	NA
KCNIP1	19.22766	-0.22873	0.084323	-2.71251	0.006677	NA
RPL36	285.25	-0.22871	0.082756	-2.76367	0.005716	NA
SNORD117	7.714529	-0.22866	0.083288	-2.74542	0.006043	NA
ANKRD34C	56.88321	0.228658	0.066541	3.43633	0.00059	NA
DOCK8	20.03495	-0.22846	0.084665	-2.69842	0.006967	NA
IRAK3	27.80654	-0.22797	0.084202	-2.70749	0.006779	NA
RPS9	101.768	-0.22773	0.079178	-2.87615	0.004026	NA
CDKL3	56.47272	-0.22756	0.06336	-3.59149	0.000329	NA
GPR153	4.887574	-0.22734	0.082366	-2.76011	0.005778	NA

PCDHB16	58.69534	-0.2273	0.082454	-2.75666	0.00584	NA
CSF1R	40.07883	-0.22713	0.082575	-2.75064	0.005948	NA
ANKZF1	416.2827	-0.22703	0.066542	-3.41182	0.000645	0.075062
BTNL9	31.75659	-0.22697	0.084938	-2.67215	0.007537	NA
JAKMIP1	51.94893	0.226943	0.082346	2.755962	0.005852	NA
PTGDS	515.0879	-0.22657	0.075656	-2.99473	0.002747	0.130106
SPIN4	23.17462	-0.22651	0.078538	-2.88404	0.003926	NA
LOC10013	10.20064	-0.22633	0.084058	-2.69254	0.007091	NA
STON1	64.68805	-0.22614	0.079775	-2.8348	0.004586	NA
RPS8	358.3453	-0.22612	0.079106	-2.85848	0.004257	0.146084
RPL35	276.1975	-0.2256	0.079944	-2.822	0.004773	NA
NPEPL1	13.27531	-0.22554	0.0849	-2.65648	0.007896	NA
EFCC1	20.97798	-0.22546	0.084796	-2.65882	0.007841	NA
SCPEP1	41.88468	-0.2252	0.068469	-3.28902	0.001005	NA
CDH8	5.247147	0.224181	0.083179	2.695165	0.007035	NA
RPS15A	216.618	-0.22404	0.083713	-2.67632	0.007444	NA
RPS18	289.0216	-0.22356	0.084937	-2.63213	0.008485	NA
SERPING1	67.05276	-0.22318	0.08461	-2.63774	0.008346	NA
FAM89A	11.97355	-0.22301	0.084939	-2.62558	0.00865	NA
SCARA3	154.7687	-0.22299	0.082841	-2.69184	0.007106	NA
LOC40054	50.79774	-0.22253	0.077121	-2.88545	0.003909	NA
FCGRT	14.12622	-0.22247	0.081098	-2.74328	0.006083	NA
HIST1H4H	36.53049	0.222389	0.084433	2.633919	0.008441	NA
PLLP	16.65793	-0.22227	0.080572	-2.75865	0.005804	NA
EVA1C	54.69248	-0.22198	0.061578	-3.60477	0.000312	NA
GREB1L	46.89389	-0.22193	0.07379	-3.00757	0.002633	NA
ALDH1L1	152.9423	-0.22137	0.082173	-2.69399	0.00706	NA
HMCN1	8.225117	-0.22112	0.083863	-2.63671	0.008371	NA
ERBB2	32.81245	-0.22112	0.083003	-2.664	0.007722	NA
MS4A7	6.859872	-0.22105	0.077497	-2.85239	0.004339	NA
METTL20	24.04626	-0.22072	0.075113	-2.93846	0.003298	NA
PRR15L	4.029443	-0.22056	0.080449	-2.7416	0.006114	NA
NREP	544.0699	0.220339	0.076326	2.8868	0.003892	0.14186
PGBD2	36.82967	0.220173	0.076864	2.864446	0.004177	NA
NISCH	1915.82	-0.2201	0.069944	-3.14682	0.001651	0.101784
JUN	397.3504	0.219934	0.06629	3.317743	0.000907	0.089367
FAM177B	3.691465	-0.21982	0.080155	-2.74248	0.006098	NA
SPATA2L	15.45873	0.219642	0.083989	2.615125	0.008919	NA
CCDC88B	462.6303	-0.21961	0.082093	-2.67512	0.00747	0.171303
TAL1	6.710107	-0.21953	0.084685	-2.59226	0.009535	NA
PNPO	72.63726	-0.21914	0.069996	-3.13074	0.001744	NA
ACPT	6.812419	-0.21902	0.083234	-2.63143	0.008503	NA
RASAL1	4.546311	-0.21894	0.081105	-2.69951	0.006944	NA
CRYL1	75.93726	-0.21852	0.078532	-2.78251	0.005394	NA
RPS7	211.1808	-0.21824	0.083356	-2.61822	0.008839	NA
PFDN5	243.581	-0.21821	0.081165	-2.68852	0.007177	NA
CFP	9.610829	0.218117	0.084907	2.568889	0.010203	NA

RPS12	128.4034	-0.21788	0.081183	-2.68386	0.007278	NA
ATOH8	16.22313	-0.21788	0.083736	-2.60197	0.009269	NA
ANGPTL4	22.17883	-0.21772	0.083409	-2.6103	0.009046	NA
CCDC89	36.7727	0.217371	0.075279	2.887537	0.003883	NA
TTC9	80.84561	0.217358	0.076068	2.857412	0.004271	NA
GRM5	20.46402	-0.21728	0.080412	-2.70205	0.006891	NA
MGAT2	41.6905	0.216939	0.065453	3.314438	0.000918	NA
PRH1-PRR	14.36421	-0.21632	0.080951	-2.67225	0.007534	NA
LGALS3	33.10128	-0.2158	0.084772	-2.54567	0.010907	NA
OLFML2A	7.432727	0.215731	0.082356	2.619483	0.008806	NA
BAALC	420.1047	-0.21555	0.074887	-2.87839	0.003997	0.14186
CCKBR	27.60871	0.215548	0.079745	2.702958	0.006873	NA
RASD1	79.94628	-0.21547	0.084158	-2.56031	0.010458	NA
EPS8L2	139.8966	-0.21538	0.081458	-2.64411	0.008191	NA
PRKCB	411.8428	0.215355	0.082283	2.617246	0.008864	0.180879
GRB7	26.9408	-0.21526	0.082912	-2.59624	0.009425	NA
STC1	5.584104	-0.21519	0.061835	-3.48	0.000501	NA
SEMA7A	163.8238	0.215164	0.083089	2.589557	0.00961	NA
WDR49	49.92333	-0.21459	0.083536	-2.56885	0.010204	NA
FXYD1	49.39538	-0.21417	0.083739	-2.55758	0.01054	NA
HIF3A	122.9134	-0.2141	0.084863	-2.52285	0.011641	NA
AGT	407.1206	0.213945	0.061043	3.504835	0.000457	0.075062
LOC38979	3.945252	-0.21359	0.080116	-2.66606	0.007675	NA
CREB3L2	38.14358	-0.21318	0.070516	-3.0231	0.002502	NA
DLEU7-AS	4.737546	-0.21307	0.082646	-2.57808	0.009935	NA
GOLGA8B	319.2531	-0.21307	0.082392	-2.586	0.00971	NA
NLRP14	1.986811	-0.21298	0.067024	-3.17771	0.001484	NA
H1FX-AS1	14.21075	-0.21278	0.082363	-2.58349	0.009781	NA
C17orf103	70.27678	-0.21265	0.067336	-3.15799	0.001589	NA
CLEC7A	5.882454	-0.21261	0.079297	-2.68118	0.007336	NA
MUC1	8.376029	-0.21257	0.083989	-2.53089	0.011377	NA
SUPV3L1	94.65	-0.21253	0.048901	-4.34605	1.39E-05	NA
AASS	85.64354	-0.21232	0.072338	-2.93504	0.003335	NA
NHLRC1	4.369376	0.211503	0.08132	2.600854	0.009299	NA
APOE	911.6687	-0.21142	0.074193	-2.84961	0.004377	0.147979
SH2D3C	49.0812	0.211295	0.073269	2.883838	0.003929	NA
CXCL16	14.27694	-0.21122	0.080619	-2.61998	0.008794	NA
FAU	191.8377	-0.21103	0.079843	-2.64309	0.008215	NA
LINC00643	26.72032	0.210993	0.084722	2.490409	0.01276	NA
RPS14	108.3022	-0.21078	0.084928	-2.48183	0.013071	NA
DPYSL5	155.1172	0.210763	0.051427	4.09828	4.16E-05	NA
TREM2	3.279352	-0.21026	0.071229	-2.95183	0.003159	NA
SLC43A2	80.18786	-0.21002	0.06465	-3.24853	0.00116	NA
SPTBN5	464.4531	0.20987	0.084452	2.485064	0.012953	0.205975
OGFRL1	544.5606	0.209664	0.069128	3.032989	0.002421	0.12465
PHF11	46.69786	-0.20958	0.071765	-2.92029	0.003497	NA
APOBEC3H	5.263537	-0.20856	0.070429	-2.96135	0.003063	NA

RPL18A	141.652	-0.20845	0.080845	-2.57833	0.009928	NA
DHRS3	13.77355	-0.20842	0.083928	-2.48338	0.013014	NA
CEBPD	48.45116	-0.20822	0.081574	-2.55248	0.010696	NA
ETV6	23.53548	-0.20819	0.084522	-2.46319	0.013771	NA
C1RL-AS1	28.19284	-0.20785	0.084875	-2.44889	0.01433	NA
LOC73010	17.6457	0.207783	0.083739	2.481314	0.01309	NA
RPL27	218.0296	-0.20775	0.084429	-2.46066	0.013868	NA
RTKN	54.98704	-0.20755	0.076213	-2.72335	0.006462	NA
S100A13	70.73641	-0.20728	0.083356	-2.48668	0.012894	NA
C5orf64	5.449733	-0.20726	0.084108	-2.46419	0.013732	NA
ST7-OT3	20.3773	-0.20714	0.081335	-2.54678	0.010872	NA
NDUFA11	77.7507	-0.20704	0.06906	-2.99801	0.002718	NA
RHOC	44.64234	-0.20677	0.081435	-2.53908	0.011114	NA
DOCK4	700.3528	-0.20652	0.052555	-3.92954	8.51E-05	0.075062
GOLGA6L7	9.047359	0.206495	0.083611	2.469711	0.013522	NA
GAS5	41.93007	-0.20649	0.077615	-2.6605	0.007803	NA
CAP2	114.8627	0.206226	0.078192	2.637418	0.008354	NA
DOK6	202.6071	0.206154	0.076032	2.711406	0.0067	NA
IFI27L2	16.52805	-0.20598	0.08382	-2.45742	0.013994	NA
TMEM144	78.87487	-0.20598	0.084752	-2.43036	0.015084	NA
ZBTB22	30.69368	0.205937	0.077409	2.660365	0.007806	NA
DNAH17	37.45171	-0.20584	0.073803	-2.78912	0.005285	NA
NDRG2	1206.873	-0.2055	0.074471	-2.75944	0.00579	0.156126
VWA5A	10.7648	-0.20541	0.081452	-2.52187	0.011673	NA
C20orf202	5.375442	-0.20536	0.081366	-2.52385	0.011608	NA
TIMM10	21.41224	-0.20517	0.082573	-2.48472	0.012965	NA
SNORD97	52.63563	-0.20516	0.08485	-2.41791	0.01561	NA
LOC10099	27.80346	-0.20511	0.081043	-2.53089	0.011377	NA
GPR180	157.362	0.204401	0.05151	3.968167	7.24E-05	NA
CHGB	3077.89	0.204385	0.080507	2.538729	0.011126	0.201925
LRRC2	45.24384	-0.20414	0.084344	-2.4203	0.015508	NA
C17orf62	58.26567	-0.20383	0.069269	-2.94261	0.003255	NA
RPS3	522.6925	-0.20377	0.07816	-2.60711	0.009131	0.183021
RGS4	8.143418	0.203637	0.081092	2.511194	0.012032	NA
GLIS1	32.70879	0.203502	0.081476	2.497679	0.012501	NA
LAPTM5	17.32646	-0.20334	0.08025	-2.53384	0.011282	NA
SPHKAP	2622.335	0.203159	0.072647	2.796533	0.005165	0.151649
IFI16	66.49208	-0.20315	0.084701	-2.39844	0.016465	NA
SELM	84.88908	-0.20314	0.077989	-2.60468	0.009196	NA
TIGD2	44.96033	0.202805	0.064387	3.14976	0.001634	NA
OXLD1	11.84381	-0.20272	0.083463	-2.42886	0.015146	NA
INSRR	8.536294	0.202507	0.077608	2.609356	0.009071	NA
COX7B	121.7943	-0.2023	0.084177	-2.40329	0.016248	NA
HPN	9.861205	-0.2021	0.084688	-2.38644	0.017012	NA
TTF2	17.00971	-0.20208	0.083568	-2.41813	0.0156	NA
FAM102B	206.3917	0.201962	0.060466	3.340112	0.000837	NA
CYP4F11	19.35915	-0.20194	0.082974	-2.43382	0.01494	NA

ALOX12P2	13.79769	-0.2017	0.0828	-2.43599	0.014851	NA
ARHGAP4	53.52945	-0.20165	0.07456	-2.7045	0.006841	NA
GLYCTK	22.83162	-0.20155	0.076237	-2.64378	0.008199	NA
BMF	15.87377	-0.20154	0.084708	-2.37923	0.017349	NA
LIMS2	11.25796	-0.20142	0.084938	-2.37138	0.017722	NA
RPL24	300.0926	-0.20134	0.081908	-2.45817	0.013965	NA
SPTLC3	4.765351	-0.20127	0.079117	-2.54401	0.010959	NA
PTPLA	21.88876	0.201247	0.082736	2.432405	0.014999	NA
LOC72973	114.4748	-0.20116	0.071924	-2.7968	0.005161	NA
PLSCR4	97.28485	-0.20052	0.083379	-2.40494	0.016175	NA
SNORA37	4.785697	-0.20048	0.082054	-2.4432	0.014558	NA
DHRS4	4.012221	-0.20043	0.080409	-2.49262	0.012681	NA
LMCD1-AS	7.6868	-0.20041	0.083865	-2.38962	0.016866	NA
DEPDC7	2.799227	-0.20024	0.074524	-2.68685	0.007213	NA
SPDEF	4.66847	-0.20023	0.079837	-2.50799	0.012142	NA
UBXN8	23.15672	-0.20011	0.081367	-2.45931	0.01392	NA
GPR158	1040.506	0.199928	0.054668	3.657124	0.000255	0.075062
HHLA3	13.22646	-0.1998	0.083631	-2.38912	0.016889	NA
A2MP1	9.550538	-0.19972	0.084653	-2.35932	0.018309	NA
ABCC4	14.13214	-0.19968	0.084904	-2.35182	0.018682	NA
IFT122	239.8161	-0.19891	0.060974	-3.2623	0.001105	NA
FOXM1	9.279045	-0.19871	0.080945	-2.45489	0.014093	NA
PRODH	156.658	-0.19867	0.083226	-2.38715	0.016979	NA
APH1A	37.08134	-0.19833	0.066724	-2.97239	0.002955	NA
RYR1	623.2078	-0.19825	0.063654	-3.11452	0.001842	0.101784
RNASE6	1.789123	-0.19807	0.065868	-3.0071	0.002638	NA
RNF175	37.55384	0.197536	0.075647	2.611294	0.00902	NA
RPL18	194.6359	-0.19748	0.076085	-2.59555	0.009444	NA
GDF10	17.12806	-0.19735	0.0799	-2.47002	0.013511	NA
CCDC96	10.34049	0.197348	0.084933	2.323581	0.020148	NA
CHST11	79.58748	0.19709	0.080099	2.460568	0.013872	NA
EFNA5	81.94854	0.197013	0.084634	2.327829	0.019921	NA
S100B	677.6063	-0.19694	0.084927	-2.31898	0.020396	0.235785
STK31	22.73055	-0.19692	0.08322	-2.36628	0.017968	NA
DECR2	16.16983	-0.19676	0.082926	-2.37277	0.017655	NA
RNF125	5.998786	-0.19663	0.083306	-2.36036	0.018257	NA
VSIG8	23.70125	0.196408	0.080786	2.4312	0.015049	NA
PET100	23.88994	-0.19634	0.081338	-2.4139	0.015783	NA
MIR4427	7.960883	-0.19586	0.084933	-2.30609	0.021106	NA
CSRP1	239.2405	-0.19585	0.070902	-2.7622	0.005741	NA
PDK4	112.4935	-0.19584	0.081987	-2.38863	0.016912	NA
PIK3IP1	162.2318	-0.19568	0.062184	-3.14671	0.001651	NA
ADARB2	105.8616	-0.19565	0.075956	-2.57583	0.01	NA
RET	54.02349	0.195615	0.084706	2.30934	0.020925	NA
TMEM74	35.79748	0.195573	0.079866	2.448772	0.014334	NA
TRPT1	12.5407	-0.19555	0.084485	-2.31457	0.020637	NA
FAM107A	1700.57	-0.19554	0.078695	-2.48475	0.012964	0.205975

FSD1L	128.5864	0.195483	0.049569	3.943664	8.02E-05	NA
CREB3L4	22.10522	-0.19545	0.081557	-2.39643	0.016555	NA
PVALB	131.8983	-0.19544	0.084738	-2.30642	0.021087	NA
THAP1	59.60282	0.195435	0.06084	3.212297	0.001317	NA
ZNF229	75.10586	0.195419	0.077255	2.529516	0.011422	NA
CYP27A1	32.83746	-0.19508	0.078526	-2.48432	0.01298	NA
CTSF	285.3439	-0.19506	0.054993	-3.54709	0.00039	NA
ZNF853	68.28261	0.195056	0.058058	3.359655	0.00078	NA
AMDHD1	10.60247	0.195042	0.082426	2.366257	0.017969	NA
ZFP36L1	133.6424	-0.19494	0.081998	-2.37733	0.017438	NA
UBE2QL1	331.6432	0.19489	0.064088	3.040967	0.002358	NA
RHBDF2	17.06726	-0.19486	0.084936	-2.29417	0.021781	NA
SNX9	110.2902	-0.19444	0.048229	-4.03168	5.54E-05	NA
TSPAN1	18.11841	0.193576	0.084828	2.281988	0.02249	NA
RPL13A	61.49314	-0.19353	0.080082	-2.41667	0.015663	NA
CALHM1	14.19308	0.193505	0.081866	2.363691	0.018094	NA
ADAM28	14.00012	-0.1934	0.084556	-2.28719	0.022184	NA
ARHGAP26	6.896192	-0.19336	0.08374	-2.30906	0.02094	NA
C1RL	9.658139	-0.193	0.082864	-2.32908	0.019855	NA
SRGN	23.83286	-0.19262	0.084927	-2.2681	0.023323	NA
SERPINB8	5.652226	-0.19259	0.082129	-2.34493	0.019031	NA
GTF2H2B	4.146449	-0.19255	0.05855	-3.2886	0.001007	NA
RGS20	33.48939	-0.19245	0.083467	-2.30577	0.021124	NA
ZNF365	188.3108	0.192444	0.077054	2.497512	0.012507	NA
C15orf26	3.123303	0.192403	0.070711	2.720967	0.006509	NA
PGBD4	24.93407	0.192303	0.076471	2.514727	0.011912	NA
LOC10049	16.88766	-0.19219	0.082804	-2.32107	0.020283	NA
MDP1	8.456375	-0.19202	0.084926	-2.26108	0.023754	NA
F8A1	3.933226	0.191921	0.080101	2.395992	0.016575	NA
KRTAP5-2	7.47393	0.191798	0.082594	2.322175	0.020224	NA
APBB1IP	13.63407	-0.1916	0.082599	-2.31962	0.020361	NA
FLJ35024	6.497669	-0.19159	0.084479	-2.2679	0.023335	NA
SLC44A2	165.9878	-0.19151	0.065555	-2.92134	0.003485	NA
KBTBD8	32.98429	0.191372	0.070595	2.710834	0.006711	NA
MMAA	47.24433	0.191179	0.065298	2.927808	0.003414	NA
C1QB	35.63537	-0.19111	0.070634	-2.70565	0.006817	NA
C4orf19	19.01588	0.191061	0.084885	2.250813	0.024397	NA
RPS15AP1	11.9674	-0.19098	0.084647	-2.25618	0.024059	NA
TMEM170	414.9523	0.190855	0.059355	3.21551	0.001302	0.095139
FPR3	3.981374	-0.19069	0.07393	-2.57939	0.009897	NA
SLC16A7	303.1206	0.190656	0.055948	3.407753	0.000655	NA
MIR3607	3.621905	-0.19064	0.080448	-2.36972	0.017802	NA
LPXN	23.82792	-0.19048	0.077409	-2.46066	0.013868	NA
MNS1	308.8794	-0.19044	0.073132	-2.60401	0.009214	NA
LAIR1	8.090464	-0.19037	0.078984	-2.41019	0.015944	NA
ZDHHC12	7.472036	-0.1903	0.084919	-2.24089	0.025033	NA
COMMD4	17.56233	-0.19028	0.080665	-2.35888	0.01833	NA

TUBA3D	13.4007	-0.19014	0.083279	-2.28316	0.022421	NA
LMBR1L	98.7437	-0.1901	0.060671	-3.13321	0.001729	NA
MGST1	65.73142	-0.19006	0.084882	-2.23915	0.025146	NA
CMTM8	3.480676	-0.18997	0.074881	-2.53692	0.011183	NA
HLA-DRA	27.06193	-0.18988	0.08175	-2.32268	0.020196	NA
TMEM219	24.84253	-0.18988	0.079384	-2.39187	0.016763	NA
WHAMMP	60.22011	-0.18958	0.073824	-2.56797	0.01023	NA
KATNAL2	66.17333	-0.18935	0.068043	-2.78276	0.00539	NA
TSPYL4	556.4496	0.189341	0.054519	3.472966	0.000515	0.075062
ERBB3	52.79885	-0.18908	0.080856	-2.33854	0.019359	NA
TCF7L2	68.12373	-0.18906	0.071055	-2.66072	0.007797	NA
MPP6	119.5173	-0.18899	0.072208	-2.61734	0.008862	NA
CASP3	140.9385	0.188976	0.058224	3.245667	0.001172	NA
ACADL	7.963795	-0.18887	0.083037	-2.2746	0.02293	NA
LAMA2	204.0018	-0.18872	0.078171	-2.41425	0.015768	NA
MAP3K6	105.1705	-0.18867	0.074144	-2.54461	0.01094	NA
TATDN3	54.88267	-0.18864	0.074622	-2.52796	0.011473	NA
LPAR5	6.81928	-0.18848	0.080629	-2.33762	0.019407	NA
PIH1D1	71.46706	-0.1884	0.055047	-3.42253	0.00062	NA
C16orf86	29.43301	-0.18833	0.081861	-2.30059	0.021415	NA
LOC10012	10.61186	-0.18817	0.084498	-2.22687	0.025956	NA
SNRPD2	54.24088	-0.18811	0.08363	-2.24929	0.024494	NA
NRG1-IT3	3.070217	-0.18783	0.075459	-2.4892	0.012803	NA
ZIC1	2036.337	0.187753	0.06751	2.781135	0.005417	0.153366
OPCML	764.7454	0.187619	0.053434	3.511214	0.000446	0.075062
IRF7	43.89376	-0.18757	0.071881	-2.60937	0.009071	NA
CYP2G1P	9.398971	-0.18732	0.084924	-2.20577	0.0274	NA
TAS2R50	12.66175	-0.18731	0.08479	-2.20917	0.027163	NA
CLEC5A	6.986399	-0.18716	0.076399	-2.44977	0.014295	NA
ZNF10	84.79729	0.186875	0.054587	3.423439	0.000618	NA
FAM195A	20.5287	-0.1868	0.07892	-2.36698	0.017934	NA
RASL10B	17.03537	-0.18679	0.08022	-2.3284	0.019891	NA
NODAL	18.11566	-0.18677	0.082962	-2.25127	0.024368	NA
AGER	35.75158	-0.18667	0.081034	-2.30364	0.021243	NA
PCDHGA3	43.40419	0.186656	0.082554	2.26101	0.023759	NA
TNFRSF11	6.89655	-0.18646	0.080933	-2.30391	0.021227	NA
MIR570	24.53755	-0.18643	0.084622	-2.20309	0.027588	NA
GSTP1	71.94138	-0.18641	0.066638	-2.79742	0.005151	NA
FASN	1301.018	-0.18637	0.066494	-2.80283	0.005066	0.151649
MGC3937	10.08737	-0.18625	0.072749	-2.56019	0.010461	NA
SLC25A33	11.8037	-0.18621	0.084295	-2.20907	0.02717	NA
TEDDM1	20.70067	-0.18616	0.084105	-2.21344	0.026867	NA
HLA-DMB	6.364837	-0.18611	0.079811	-2.33183	0.019709	NA
NDUFA4	129.6884	-0.1861	0.083888	-2.21842	0.026526	NA
CHST3	72.26063	-0.18597	0.083152	-2.23648	0.025321	NA
RASL11B	22.71104	0.185945	0.080771	2.302142	0.021327	NA
ZNF516	78.44351	-0.18586	0.080938	-2.29628	0.02166	NA

SP110	24.32423	-0.1858	0.082702	-2.24658	0.024667	NA
RPL9	182.171	-0.18569	0.084271	-2.20346	0.027563	NA
PRKAR2B	64.40197	0.185512	0.067929	2.730968	0.006315	NA
RIN3	19.80531	-0.18548	0.082984	-2.23514	0.025408	NA
RPS3A	353.8534	-0.18542	0.084173	-2.20285	0.027605	0.263821
LMNB1	259.5086	0.184891	0.05617	3.291618	0.000996	NA
APOC1	3.390359	-0.18489	0.060614	-3.05024	0.002287	NA
HNMT	50.0066	-0.18485	0.076923	-2.403	0.016261	NA
ZNF100	121.6653	0.184728	0.068719	2.688141	0.007185	NA
SAE1	117.4357	0.184569	0.05471	3.373579	0.000742	NA
SAP30	148.0528	-0.18457	0.05288	-3.49028	0.000483	NA
PIM1	53.11451	-0.18456	0.071178	-2.59288	0.009518	NA
PSAPL1	3.419953	-0.18437	0.078542	-2.34742	0.018904	NA
ENPEP	3.499098	-0.1843	0.070749	-2.60496	0.009189	NA
KBTBD7	63.64754	0.184232	0.063056	2.921732	0.003481	NA
RPS5	144.7424	-0.18401	0.084298	-2.18285	0.029047	NA
PARP4	42.34103	-0.18401	0.075349	-2.44207	0.014603	NA
SURF1	15.71506	-0.184	0.082582	-2.22808	0.025875	NA
CCDC147	3.388633	-0.18395	0.077551	-2.37194	0.017695	NA
NOXRED1	3.904667	-0.18386	0.076254	-2.4112	0.0159	NA
ERBB2IP	344.0113	-0.18383	0.073991	-2.48451	0.012973	0.205975
CD74	170.5445	-0.18367	0.082635	-2.2227	0.026236	NA
SDHAP2	166.1765	-0.18364	0.080554	-2.27976	0.022622	NA
PDE4B	177.7164	-0.18354	0.081029	-2.26509	0.023507	NA
AIF1	5.772773	-0.18353	0.072917	-2.51701	0.011836	NA
CLDN18	29.54177	0.183462	0.084002	2.184032	0.02896	NA
GOLT1A	4.46497	-0.18346	0.080698	-2.27336	0.023005	NA
PZP	60.43131	-0.18339	0.079979	-2.29295	0.021851	NA
RABIF	30.62259	0.183335	0.072469	2.529846	0.011411	NA
FLJ34208	7.290105	-0.18327	0.084875	-2.15936	0.030822	NA
HLF	243.4817	0.183222	0.076469	2.396026	0.016574	NA
CERCAM	106.0234	-0.18308	0.079325	-2.308	0.020999	NA
C1orf54	9.757067	-0.18303	0.084328	-2.17044	0.029973	NA
SYT16	24.35576	0.182796	0.084899	2.153106	0.03131	NA
ARPC4	22.34784	-0.18273	0.07899	-2.3133	0.020706	NA
SPATA13	132.2091	-0.18264	0.07554	-2.41786	0.015612	NA
TRIM5	10.07768	-0.18258	0.084438	-2.16226	0.030598	NA
PROCA1	26.43744	-0.18257	0.07916	-2.30632	0.021093	NA
C10orf105	19.57929	-0.18238	0.084852	-2.14934	0.031607	NA
HTR2B	19.31937	-0.18231	0.080311	-2.27002	0.023207	NA
SCARNA12	12.38016	-0.18223	0.084649	-2.15279	0.031336	NA
TRIQK	130.9185	0.18223	0.05865	3.107072	0.00189	NA
GAA	60.97948	-0.18216	0.072098	-2.52651	0.01152	NA
SNHG6	58.00725	-0.18203	0.073935	-2.46199	0.013817	NA
SYTL4	30.40263	-0.18198	0.083617	-2.17634	0.02953	NA
DGCR9	229.2686	-0.18197	0.074524	-2.44173	0.014617	NA
CRNDE	9.335604	-0.18196	0.084789	-2.14608	0.031866	NA

LOC10050	23.75724	0.181962	0.081889	2.222063	0.026279	NA
KIF24	17.12224	-0.18191	0.08145	-2.23336	0.025525	NA
NPL	25.66084	-0.1819	0.083669	-2.17406	0.029701	NA
MYO7B	2.525699	-0.18186	0.067217	-2.70556	0.006819	NA
RGN	100.9851	-0.18156	0.054119	-3.35481	0.000794	NA
RPL13P5	8.089772	0.181479	0.084913	2.137242	0.032578	NA
PLCB2	20.36616	-0.18129	0.084874	-2.13597	0.032682	NA
FAM110A	5.893228	0.181256	0.082781	2.189593	0.028554	NA
QPCTL	21.62653	-0.18118	0.076188	-2.37811	0.017402	NA
VSTM5	2.496635	-0.18111	0.067309	-2.69068	0.007131	NA
PPP1R14D	1.763261	0.181041	0.063693	2.842414	0.004477	NA
INCENP	394.4628	-0.18098	0.074055	-2.44388	0.01453	0.212327
SIGMAR1	70.8257	0.180975	0.068901	2.626592	0.008624	NA
MUC5B	7.917795	-0.18097	0.068424	-2.64485	0.008173	NA
ASGR2	1.471602	-0.1809	0.061775	-2.92842	0.003407	NA
SNORD45A	10.48485	-0.18089	0.07884	-2.29437	0.021769	NA
MIR23C	2.720892	-0.18083	0.074737	-2.41952	0.015541	NA
GNRH1	35.45721	-0.18073	0.083811	-2.1564	0.031052	NA
RPS19	209.4363	-0.18054	0.075037	-2.40601	0.016128	NA
ITGA7	103.4111	-0.1804	0.0703	-2.56616	0.010283	NA
RPLP0	179.3093	-0.18022	0.077218	-2.33395	0.019598	NA
ZIC4	563.7506	0.180125	0.050152	3.591583	0.000329	0.075062
LOC10012	81.33471	0.180106	0.080244	2.244479	0.024802	NA
C20orf96	25.76932	-0.18007	0.084222	-2.13804	0.032514	NA
TP53AIP1	7.422505	-0.18007	0.084421	-2.13294	0.032929	NA
ZNF311	3.873349	-0.18003	0.076569	-2.35116	0.018715	NA
CNTN4	223.091	0.17987	0.058054	3.098309	0.001946	NA
SLC25A18	88.09512	-0.17985	0.077751	-2.31318	0.020713	NA
P2RX6	44.74241	0.179802	0.080044	2.246288	0.024686	NA
BTBD3	748.5475	0.179792	0.064943	2.768467	0.005632	0.155569
C19orf81	8.339856	-0.17973	0.084866	-2.11776	0.034195	NA
RPL19	843.0513	-0.17965	0.069613	-2.58072	0.00986	0.193485
CCNA2	34.42304	0.179619	0.084395	2.128314	0.033311	NA
GPR22	116.3699	0.179606	0.068076	2.638296	0.008332	NA
FAM103A1	12.51159	0.179529	0.084778	2.117632	0.034206	NA
CPAMD8	10.16892	-0.17947	0.079037	-2.27073	0.023163	NA
CNNM1	228.7435	0.179381	0.078733	2.278331	0.022707	NA
SAMD10	61.96813	-0.17936	0.062645	-2.86317	0.004194	NA
PCK2	18.89773	-0.17934	0.083174	-2.1562	0.031068	NA
SIGLEC8	4.269711	-0.1793	0.076872	-2.3324	0.019679	NA
LOC38884	4.331488	-0.17923	0.076391	-2.34628	0.018962	NA
IGDCC4	30.45486	0.179159	0.083543	2.144509	0.031992	NA
LOC72997	9.046347	-0.17915	0.084896	-2.11022	0.034839	NA
KANSL1-AS	6.26628	0.179107	0.078972	2.267965	0.023331	NA
EXTL1	14.61676	0.179094	0.084262	2.125439	0.03355	NA
GAP43	299.508	0.179074	0.06374	2.809457	0.004963	NA
PLEKHG1	52.97195	-0.17892	0.077054	-2.32195	0.020236	NA

LOXL4	17.94336	-0.17888	0.08096	-2.20943	0.027145	NA
VTRNA1-3	13.50498	0.178796	0.068246	2.619869	0.008796	NA
STX16-NPE	12.94279	-0.1787	0.084899	-2.10487	0.035303	NA
KLHL11	134.5759	0.178652	0.059753	2.98984	0.002791	NA
LOC28439	9.998293	-0.17863	0.081081	-2.20312	0.027586	NA
LOC10013	35.4996	-0.17833	0.080083	-2.22688	0.025955	NA
GRAMD3	124.9741	-0.17817	0.078436	-2.27152	0.023116	NA
KHDRBS3	68.48115	0.178165	0.071413	2.494867	0.0126	NA
KANK3	20.19869	-0.17805	0.081144	-2.19429	0.028215	NA
GSN	199.4259	-0.178	0.078082	-2.2796	0.022631	NA
C15orf60	6.212931	0.177989	0.084037	2.117982	0.034177	NA
FOXJ1	1.575995	0.177975	0.054442	3.269086	0.001079	NA
METTL9	117.7929	0.177737	0.055444	3.205704	0.001347	NA
MIR329-1	2.18706	-0.17764	0.071316	-2.49093	0.012741	NA
C19orf53	83.05148	-0.17763	0.067737	-2.62234	0.008733	NA
UXT	22.98816	-0.17758	0.077317	-2.29676	0.021633	NA
SLC1A5	5.649329	-0.17748	0.07879	-2.25259	0.024285	NA
ZNF286B	27.70109	0.177172	0.079836	2.219192	0.026474	NA
MRPL13	25.61604	-0.17707	0.080935	-2.18783	0.028682	NA
LOC10050	2.968228	-0.17689	0.076611	-2.3089	0.020949	NA
FAM214A	394.5956	-0.17679	0.053854	-3.28267	0.001028	0.092144
ZMPSTE24	82.01862	0.176737	0.067131	2.632721	0.00847	NA
AHCYL1	959.9883	-0.17657	0.075346	-2.34349	0.019104	0.231397
MIR760	5.532922	0.176478	0.083478	2.114064	0.03451	NA
METTL22	84.25399	-0.17644	0.062905	-2.80488	0.005034	NA
TSPAN4	45.32945	-0.17643	0.074531	-2.36716	0.017925	NA
AXL	55.19543	-0.17642	0.078943	-2.23479	0.025431	NA
TMBIM1	95.84138	-0.1764	0.073859	-2.38831	0.016926	NA
PGM5	39.55091	-0.17632	0.084828	-2.07852	0.037662	NA
CARD14	10.87175	-0.17625	0.083715	-2.10535	0.035261	NA
FAM162A	38.92924	-0.17616	0.082727	-2.12947	0.033216	NA
MT1E	19.20943	-0.17603	0.083492	-2.10837	0.034999	NA
PDZK1	49.43199	0.17603	0.078897	2.231151	0.025671	NA
FAM3A	69.82861	-0.17596	0.062898	-2.79758	0.005149	NA
HSBP1L1	32.75732	-0.17586	0.076464	-2.2999	0.021454	NA
ACSS1	191.6091	-0.17581	0.059038	-2.97791	0.002902	NA
CSPG5	57.70985	0.17576	0.076257	2.304826	0.021176	NA
RORC	87.95638	-0.17555	0.080742	-2.17424	0.029687	NA
ELTD1	13.5152	0.175537	0.083849	2.093494	0.036305	NA
EPCAM	4.73502	-0.17553	0.0657	-2.67172	0.007546	NA
RPL23AP7	13.71431	0.175476	0.082759	2.12032	0.033979	NA
NCKAP1L	11.24933	-0.17533	0.084217	-2.08191	0.037351	NA
COX5B	247.8065	-0.1753	0.084116	-2.08408	0.037153	NA
GUCY1A2	473.9457	0.175293	0.070031	2.50308	0.012312	0.205975
MYRF	104.9125	-0.17526	0.079283	-2.21059	0.027064	NA
C21orf62	17.57904	-0.17509	0.082913	-2.11176	0.034707	NA
LRRC14	115.1248	0.175033	0.075983	2.303601	0.021245	NA

RCC2	115.4041	0.174984	0.05998	2.917362	0.00353	NA
ZNF518B	232.9113	0.174948	0.050814	3.442924	0.000575	NA
C1orf213	16.76024	-0.17494	0.083368	-2.09837	0.035872	NA
CITED4	8.013377	-0.17467	0.084387	-2.06993	0.038459	NA
FADS3	63.02038	-0.17462	0.075433	-2.31492	0.020617	NA
PHKG1	46.61012	-0.17461	0.067368	-2.59192	0.009544	NA
LIMS1	33.54508	-0.17449	0.072617	-2.40283	0.016269	NA
TLCD1	16.02086	-0.1744	0.084935	-2.05333	0.040041	NA
C18orf32	6.109828	-0.17437	0.084501	-2.0635	0.039065	NA
SLC51B	2.317067	-0.17432	0.071473	-2.439	0.014728	NA
ZSWIM4	16.29436	-0.17432	0.08107	-2.15021	0.031539	NA
VWA3A	11.71068	-0.1743	0.083755	-2.08108	0.037426	NA
TLR10	1.56142	-0.17427	0.062386	-2.79348	0.005214	NA
MRE11A	245.8745	-0.1742	0.054932	-3.17116	0.001518	NA
PCDH9-AS	24.67068	-0.17411	0.081045	-2.14836	0.031685	NA
CRIPT	73.79983	-0.1741	0.061809	-2.8168	0.004851	NA
DNPH1	12.07662	-0.1741	0.084705	-2.05535	0.039845	NA
RPS25	208.6658	-0.174	0.073229	-2.37607	0.017498	NA
IDUA	163.6232	-0.17397	0.06669	-2.60867	0.009089	NA
PANX1	49.10689	0.173904	0.082772	2.100992	0.035642	NA
RPS27A	374.0795	-0.17381	0.082144	-2.11594	0.03435	0.288416
LOC14366	21.49913	-0.1738	0.078154	-2.22378	0.026163	NA
LSM6	57.38229	-0.17374	0.075261	-2.30852	0.02097	NA
FBXL19-AS	10.65089	0.173502	0.080149	2.164737	0.030408	NA
NDUFAF4	96.73145	0.173376	0.072253	2.39956	0.016415	NA
CD209	10.11603	0.173368	0.083075	2.086899	0.036897	NA
WNT7A	3.254836	0.17332	0.075585	2.293061	0.021845	NA
FGD5P1	2.762243	-0.17331	0.075425	-2.29776	0.021575	NA
SNHG1	61.48721	-0.17329	0.071072	-2.4382	0.014761	NA
DHRS7B	16.64836	-0.17325	0.081756	-2.1191	0.034082	NA
MTFP1	3.785572	-0.17322	0.076768	-2.25636	0.024048	NA
PXDN	9.930144	-0.17321	0.08276	-2.09288	0.03636	NA
UBOX5-AS	24.05479	-0.1732	0.078417	-2.20876	0.027191	NA
FAT4	43.12807	-0.17311	0.071316	-2.42736	0.015209	NA
PDE6C	11.70383	-0.17304	0.084718	-2.0425	0.041102	NA
ZNF525	136.5998	0.172969	0.058892	2.937074	0.003313	NA
TMEM176	9.502806	-0.17294	0.074495	-2.32144	0.020263	NA
KIF17	1.688588	0.172892	0.066441	2.602183	0.009263	NA
EGFL7	27.94087	-0.17283	0.084447	-2.04662	0.040696	NA
CD97	6.918317	-0.17282	0.084784	-2.03834	0.041516	NA
GADD45A	15.17495	-0.17274	0.079662	-2.16838	0.03013	NA
TSG1	8.540881	-0.1727	0.076382	-2.26104	0.023757	NA
UTS2	4.32819	0.172683	0.075037	2.301302	0.021375	NA
PCDP1	12.36729	-0.17268	0.084888	-2.03424	0.041927	NA
METTL13	75.14244	0.172535	0.056132	3.073741	0.002114	NA
UPB1	2.323994	-0.17252	0.067798	-2.54456	0.010942	NA
HMGB3	18.38882	-0.17247	0.084874	-2.03203	0.042151	NA

CRIPAK	53.45425	-0.17243	0.072335	-2.38373	0.017138	NA
GBP4	15.27211	0.172418	0.084236	2.046839	0.040674	NA
FCGR3A	15.17559	-0.1724	0.070051	-2.46103	0.013854	NA
C12orf49	71.2184	0.172386	0.064477	2.673623	0.007504	NA
VANGL2	81.95528	-0.17237	0.068978	-2.49892	0.012457	NA
ENPP1	24.12773	-0.1723	0.084757	-2.03285	0.042068	NA
ACOX2	13.46863	-0.17229	0.081597	-2.11151	0.034728	NA
BTN3A3	39.3183	-0.17224	0.079479	-2.16711	0.030226	NA
PAGR1	683.4095	-0.17224	0.068446	-2.51637	0.011857	0.201925
CSNK2B	161.9626	-0.17214	0.048794	-3.52785	0.000419	NA
STAB1	44.53234	-0.1721	0.081089	-2.12233	0.03381	NA
URM1	57.82468	-0.17196	0.066272	-2.59471	0.009467	NA
LRRC66	20.03522	0.171946	0.076793	2.239103	0.025149	NA
ZNF155	43.59119	0.171763	0.063919	2.687203	0.007205	NA
C9orf171	11.84937	-0.17174	0.084751	-2.02643	0.042721	NA
MASTL	36.28154	-0.17165	0.069365	-2.47462	0.013338	NA
RPL34	239.4617	-0.17163	0.079362	-2.16267	0.030567	NA
C6orf164	5.260977	-0.17156	0.081987	-2.09246	0.036398	NA
LOC10050	14.2913	-0.17154	0.082988	-2.0671	0.038724	NA
C20orf203	20.34055	-0.17151	0.082218	-2.08603	0.036976	NA
MAP3K1	200.4018	0.17141	0.047322	3.622201	0.000292	NA
HSPBAP1	29.08278	-0.17141	0.077142	-2.22196	0.026286	NA
THBS3	42.59963	-0.17135	0.078704	-2.1771	0.029473	NA
COL4A5	32.53419	-0.1713	0.084758	-2.02105	0.043275	NA
C11orf45	1.592152	-0.17128	0.065104	-2.63084	0.008517	NA
LRRC38	7.773445	-0.17125	0.080562	-2.12571	0.033527	NA
MRPS28	39.96162	-0.1712	0.067871	-2.52245	0.011654	NA
SSH1	537.7834	0.171143	0.048349	3.539731	0.000401	0.075062
CDKL5	104.92	0.171135	0.058556	2.922583	0.003471	NA
LOC72995	4.587177	0.17111	0.080423	2.127628	0.033368	NA
SLC32A1	13.03758	-0.17102	0.083117	-2.05763	0.039626	NA
COL6A2	19.70514	-0.17099	0.082444	-2.07396	0.038083	NA
MAP6D1	173.5937	0.170897	0.067012	2.550257	0.010764	NA
CD53	8.473406	-0.1709	0.082721	-2.06593	0.038835	NA
SNORD35B	4.247265	-0.17077	0.072748	-2.34736	0.018907	NA
DERA	26.25599	-0.17061	0.079154	-2.15537	0.031133	NA
SLC35F4	49.65822	-0.17053	0.070871	-2.40619	0.01612	NA
TMEM52	4.12416	-0.17047	0.07681	-2.2194	0.026459	NA
MRPL23-A	9.805399	-0.1704	0.080964	-2.10466	0.035321	NA
SKOR1	32.40769	-0.17039	0.076384	-2.23073	0.025699	NA
NEIL2	53.67193	0.170386	0.067975	2.50659	0.01219	NA
VSIG4	8.580609	-0.17034	0.067259	-2.53263	0.011321	NA
CENPQ	17.54576	-0.17034	0.081499	-2.09005	0.036613	NA
LOC64627	22.243	0.170112	0.084834	2.005236	0.044938	NA
EFEMP2	40.5699	-0.17007	0.068865	-2.46961	0.013526	NA
CCT5	188.1225	0.169978	0.071173	2.388223	0.01693	NA
GPANK1	78.40938	-0.16985	0.057522	-2.95278	0.003149	NA

C4orf32	16.98865	0.169833	0.083475	2.034534	0.041898	NA
ZNF821	134.2753	-0.16977	0.055843	-3.04012	0.002365	NA
ST7-AS1	6.027967	-0.16974	0.077608	-2.18718	0.02873	NA
PIK3R3	588.7223	0.169586	0.045286	3.744785	0.000181	0.075062
SCML1	35.51606	-0.16956	0.07672	-2.21005	0.027102	NA
C1S	52.14609	-0.16952	0.084936	-1.99583	0.045952	NA
RPP40	4.694448	0.169518	0.08237	2.058006	0.03959	NA
DENND3	70.06534	-0.16949	0.077177	-2.19607	0.028087	NA
RGS10	6.296765	-0.16936	0.080383	-2.10695	0.035122	NA
ITIH3	5.651136	-0.16931	0.082588	-2.05004	0.040361	NA
COQ4	49.77854	-0.16923	0.070129	-2.41312	0.015817	NA
TRPM4	28.70579	0.169171	0.079697	2.122683	0.03378	NA
CNTN6	222.3864	0.169095	0.07303	2.315414	0.02059	NA
RGCC	151.8111	-0.16897	0.078981	-2.13933	0.032409	NA
PTPRC	21.79379	-0.16896	0.084936	-1.98931	0.046667	NA
NUP43	72.03813	-0.16896	0.058012	-2.91245	0.003586	NA
ZFHX4	100.2814	-0.16895	0.084071	-2.00962	0.044472	NA
SOX9	243.4452	-0.1687	0.077507	-2.17658	0.029512	NA
MKNK1-AS	23.65803	-0.16867	0.081878	-2.05995	0.039403	NA
ZC3H12A	10.23374	0.168618	0.084938	1.985195	0.047123	NA
MVP	64.79899	-0.16861	0.082727	-2.03812	0.041538	NA
GALT	67.91296	-0.16859	0.07214	-2.33696	0.019441	NA
LHX5	8.352296	-0.16848	0.082692	-2.03746	0.041604	NA
MMP15	29.90963	0.168411	0.079261	2.124777	0.033605	NA
ESRG	2.65954	-0.16835	0.073722	-2.28358	0.022396	NA
PRKCQ-AS	3.24255	-0.16826	0.074552	-2.25696	0.024011	NA
PCDHGA1	51.72316	0.168223	0.083099	2.024362	0.042933	NA
DCX	10.83149	-0.16814	0.084911	-1.98018	0.047683	NA
MT1X	22.93786	-0.16808	0.084767	-1.98283	0.047387	NA
ZNHIT1	34.57051	-0.16803	0.078985	-2.1273	0.033395	NA
LOC15857	6.801748	-0.16797	0.084822	-1.98025	0.047676	NA
FOLR4	2.778365	-0.16796	0.072492	-2.31693	0.020508	NA
BTG3	4.218053	-0.16783	0.081564	-2.05766	0.039623	NA
SNORA4	11.87582	-0.16776	0.084747	-1.97952	0.047757	NA
SNORA55	4.68897	-0.16771	0.080587	-2.08114	0.037421	NA
MIF4GD	22.43424	-0.16767	0.076214	-2.19995	0.027811	NA
NFATC2	19.94249	-0.16764	0.084039	-1.99476	0.046069	NA
LOC10028	34.39897	0.167611	0.073682	2.274775	0.022919	NA
QRICH2	160.5082	0.167581	0.081208	2.063594	0.039056	NA
MLF1IP	10.87873	-0.16757	0.084599	-1.98076	0.047618	NA
RPL31	343.86	-0.16756	0.070857	-2.36484	0.018038	0.229527
POLR2L	36.02985	-0.16755	0.083331	-2.01067	0.04436	NA
NCF2	6.944659	-0.16751	0.084929	-1.97238	0.048567	NA
BRI3BP	54.65323	0.167435	0.059507	2.813694	0.004898	NA
EVI2B	41.86853	-0.16742	0.067668	-2.47414	0.013356	NA
FAM98A	69.69444	0.167293	0.061835	2.70548	0.006821	NA
BARHL1	73.43789	0.167181	0.07696	2.172302	0.029833	NA

KIAA2022	148.0382	0.167125	0.06183	2.702987	0.006872	NA
SNORA74A	1.644659	-0.16712	0.062266	-2.68393	0.007276	NA
TIMP4	111.2518	-0.16711	0.073517	-2.27307	0.023022	NA
CYP4V2	202.3072	0.167074	0.071465	2.337835	0.019396	NA
TACR1	5.053036	-0.16702	0.079399	-2.1036	0.035413	NA
CNTNAP5	77.21012	-0.16683	0.081735	-2.04114	0.041237	NA
HLA-DMA	7.360594	-0.16683	0.077907	-2.14139	0.032243	NA
C10orf113	24.60947	-0.16674	0.081667	-2.04175	0.041176	NA
ATP10D	64.60589	-0.16669	0.061419	-2.71401	0.006647	NA
MGC1291	2.551655	-0.16662	0.065868	-2.52962	0.011418	NA
RNF217	42.97994	-0.1666	0.063527	-2.62252	0.008728	NA
C17orf104	19.57569	0.166596	0.080261	2.075677	0.037924	NA
P2RY1	12.29862	-0.16655	0.084907	-1.9615	0.04982	NA
TOMM6	39.66516	-0.16646	0.069124	-2.40809	0.016036	NA
COX6A2	3.781923	-0.16642	0.080078	-2.07825	0.037686	NA
HILPDA	62.5352	-0.16641	0.080264	-2.07334	0.03814	NA
MPEG1	13.38686	0.166408	0.083709	1.987934	0.046819	NA
GLS	934.4241	0.166049	0.059108	2.809225	0.004966	0.151649
BMPR2	784.1397	0.165937	0.050951	3.256809	0.001127	0.092144
RNASE4	4.242902	-0.16594	0.080448	-2.06263	0.039148	NA
CDADC1	69.36565	0.165919	0.059521	2.787546	0.005311	NA
SYNGR2	47.09648	-0.16586	0.072591	-2.28487	0.022321	NA
IRF3	31.94103	-0.16585	0.073438	-2.25836	0.023923	NA
DACT3-AS	1.812458	0.165723	0.066791	2.48122	0.013093	NA
TRMT6	87.53014	0.165677	0.065071	2.54608	0.010894	NA
MSH4	37.70128	0.165658	0.084935	1.95041	0.051127	NA
HSD17B11	88.18569	-0.1656	0.067748	-2.4443	0.014513	NA
SHANK2-A	9.769597	-0.16554	0.084136	-1.9675	0.049126	NA
SHC4	10.16929	-0.16553	0.083276	-1.98768	0.046847	NA
KRT31	23.76086	0.165479	0.084857	1.9501	0.051164	NA
ITGAX	12.10985	-0.16542	0.084848	-1.94963	0.05122	NA
GAREML	49.29316	0.16538	0.084265	1.962623	0.04969	NA
GRAMD1C	44.84508	-0.16535	0.077949	-2.12133	0.033894	NA
MERTK	57.21293	-0.16532	0.082436	-2.00542	0.044919	NA
COX6B2	1.409689	-0.16519	0.061156	-2.70119	0.006909	NA
SYTL3	3.527361	-0.16514	0.076674	-2.15376	0.031259	NA
43346	1087.725	0.165123	0.055159	2.993585	0.002757	0.130106
GPR183	19.09133	-0.16509	0.077448	-2.13165	0.033036	NA
ZFAS1	77.65358	-0.16506	0.08386	-1.96827	0.049037	NA
LINC00641	542.1291	0.165054	0.050671	3.25735	0.001125	0.092144
KLF11	80.21612	0.164997	0.076763	2.149434	0.0316	NA
NEK7	110.7045	-0.16496	0.066895	-2.46596	0.013665	NA
ZNF547	46.57476	0.164955	0.076872	2.14585	0.031885	NA
CTDSP1	38.3146	-0.16495	0.0724	-2.27831	0.022708	NA
DLGAP1-A	25.10711	-0.16495	0.081777	-2.01701	0.043695	NA
KIAA1045	150.1796	0.164802	0.067347	2.447047	0.014403	NA
IMMP2L	23.79185	-0.16476	0.07754	-2.12479	0.033604	NA

PIM2	22.47216	-0.16465	0.078706	-2.09193	0.036445	NA
TNFRSF14	11.18363	-0.16462	0.084088	-1.95767	0.050268	NA
SNORA16A	6.234471	-0.16451	0.081524	-2.01789	0.043603	NA
DDRGK1	286.2298	-0.16447	0.054966	-2.99218	0.00277	NA
RNASEH2B	5.169211	-0.16434	0.079978	-2.0548	0.039898	NA
RNF149	102.1963	-0.16426	0.062966	-2.60866	0.00909	NA
LRRC4	257.6161	0.164249	0.06412	2.561612	0.010419	NA
HOPX	38.30926	-0.16425	0.083249	-1.97298	0.048498	NA
SCARNA16	24.28174	-0.16422	0.084939	-1.93333	0.053195	NA
ACOT11	147.96	-0.16421	0.078059	-2.1037	0.035405	NA
C2	12.78443	-0.16421	0.082796	-1.98328	0.047336	NA
NDRG1	162.8757	-0.16419	0.08107	-2.02535	0.042832	NA
KNSTRN	71.44775	0.164166	0.076415	2.14834	0.031687	NA
KIAA1683	100.2095	0.164152	0.082725	1.984321	0.04722	NA
PDIK1L	76.63518	0.164144	0.058604	2.800892	0.005096	NA
SYNPO2	68.74932	-0.16409	0.079126	-2.07384	0.038095	NA
XRCC4	10.70805	-0.16409	0.084764	-1.93584	0.052888	NA
SMAD3	73.19912	-0.16408	0.074191	-2.21164	0.026992	NA
LRRC2-AS1	0.771902	-0.16407	0.048981	-3.34964	0.000809	NA
SYNPR	276.7223	0.164037	0.079315	2.068188	0.038622	NA
RPS20	228.8197	-0.16397	0.062979	-2.60351	0.009228	NA
MYH15	26.01029	-0.16396	0.082849	-1.97907	0.047809	NA
ACE	15.69888	0.16396	0.083047	1.974298	0.048348	NA
C17orf97	21.62136	-0.16394	0.082342	-1.99096	0.046485	NA
TIMM8B	28.20526	-0.1639	0.077814	-2.10633	0.035176	NA
ARSA	75.52108	-0.1639	0.070008	-2.34109	0.019228	NA
CCDC157	4.530581	0.163892	0.081665	2.006888	0.044762	NA
PDE3A	160.1189	0.163841	0.079608	2.058088	0.039582	NA
SLC44A4	5.360314	0.163763	0.083461	1.962148	0.049745	NA
DNAH6	27.96252	0.163758	0.082682	1.980573	0.047639	NA
MRPL52	29.07335	-0.16375	0.080102	-2.04434	0.04092	NA
RASAL3	4.847275	-0.16372	0.079388	-2.06226	0.039183	NA
LINC00273	169.7519	-0.16371	0.081012	-2.02078	0.043302	NA
SH2D4A	6.101351	-0.16364	0.082693	-1.97894	0.047823	NA
CFL1P1	6.513508	0.163631	0.083788	1.952927	0.050828	NA
ZACN	4.513453	0.163571	0.072669	2.250901	0.024392	NA
FAM26E	1.888615	0.163375	0.066657	2.450992	0.014246	NA
RBBP5	232.2001	0.163343	0.050389	3.241622	0.001189	NA
ANKRD50	117.8544	0.163329	0.065588	2.490222	0.012766	NA
EXD3	46.41669	-0.16331	0.07739	-2.11026	0.034836	NA
LOC64551	79.36397	-0.16331	0.062622	-2.60787	0.009111	NA
ARHGEF26	16.83503	-0.16314	0.083955	-1.94312	0.052002	NA
SNORD114	4.039452	-0.16311	0.07309	-2.23171	0.025634	NA
DLGAP1-A	6.872515	-0.16298	0.084479	-1.92926	0.053699	NA
OR10V2P	4.773831	-0.16295	0.081229	-2.00611	0.044844	NA
PSTPIP1	5.737512	-0.16291	0.083031	-1.96199	0.049764	NA
YWHAG	1200.134	0.162869	0.061881	2.63195	0.00849	0.176787

RAB3C	77.05955	0.162859	0.075443	2.158714	0.030872	NA
RPS26	74.70279	-0.16286	0.083957	-1.93978	0.052406	NA
NUDT22	13.1053	-0.16271	0.083016	-1.95992	0.050005	NA
FAM161A	32.74413	-0.16267	0.079997	-2.03344	0.042008	NA
MIR101-1	2.095291	-0.16266	0.069138	-2.35276	0.018635	NA
DSG2	4.961233	-0.16259	0.076548	-2.12404	0.033667	NA
EIF4EBP1	10.64634	-0.16254	0.084898	-1.91459	0.055545	NA
SLC4A5	133.6833	-0.16249	0.074023	-2.1951	0.028156	NA
JUNB	48.6305	-0.16246	0.083608	-1.94312	0.052002	NA
OLFM3	160.9445	0.162349	0.055526	2.923822	0.003458	NA
ADO	100.5999	0.162287	0.065549	2.47583	0.013293	NA
CAND1	583.8895	0.162285	0.043637	3.718979	0.0002	0.075062
ZNF408	22.28727	0.1622	0.07989	2.030305	0.042326	NA
GPR18	28.65284	-0.16217	0.079236	-2.04665	0.040692	NA
HEXA	138.7664	-0.16216	0.060765	-2.66855	0.007618	NA
LACTB2	8.507027	-0.16205	0.084866	-1.90948	0.056201	NA
FLJ45513	25.4521	-0.16197	0.084788	-1.91027	0.056099	NA
RBM24	5.594625	0.16183	0.076952	2.102989	0.035467	NA
NRBP2	225.7276	-0.16183	0.057574	-2.81076	0.004942	NA
KIF5A	1974.778	0.161772	0.054688	2.958073	0.003096	0.135775
ADCY1	1262.301	0.161575	0.05785	2.79299	0.005222	0.151649
TLR2	18.92207	-0.16155	0.083856	-1.92656	0.054035	NA
FUT1	34.89715	0.161506	0.067988	2.37549	0.017526	NA
PURB	832.7022	0.161471	0.057066	2.82952	0.004662	0.150804
SAT1	223.9171	-0.16139	0.076559	-2.10808	0.035024	NA
COQ2	6.62246	0.161376	0.083096	1.942044	0.052132	NA
VKORC1L1	32.50382	0.16137	0.076613	2.106298	0.035178	NA
WDHD1	43.87425	-0.16132	0.06779	-2.37974	0.017325	NA
H1F0	372.9778	0.161309	0.077119	2.09169	0.036466	0.292894
MDFIC	26.05664	-0.16126	0.083837	-1.9235	0.054417	NA
NEUROD2	237.7531	0.161257	0.068108	2.367646	0.017902	NA
FAT2	6694.747	0.161156	0.059834	2.69339	0.007073	0.171303
C17orf76-	134.9822	-0.16104	0.080594	-1.99815	0.0457	NA
SLC35E2	143.3312	-0.16094	0.067862	-2.37158	0.017712	NA
GJB6	47.52711	-0.16087	0.084694	-1.89945	0.057506	NA
DBI	217.0829	-0.16082	0.07581	-2.12137	0.033891	NA
PABPC1L	12.50846	-0.16071	0.083387	-1.92729	0.053943	NA
GATS	686.734	-0.16065	0.05583	-2.8775	0.004008	0.14186
ANAPC15	14.28392	-0.16064	0.084641	-1.89786	0.057715	NA
EGOT	4.807954	-0.16061	0.081479	-1.97123	0.048697	NA
ITGA11	14.3321	-0.16056	0.083632	-1.91985	0.054877	NA
HPDL	18.71086	-0.16049	0.083217	-1.92863	0.053777	NA
LINC00852	17.39379	0.160467	0.083457	1.922738	0.054513	NA
SLC8A3	44.24177	-0.16039	0.084939	-1.88832	0.058983	NA
EPB41L1	732.1714	0.160385	0.063518	2.525043	0.011568	0.201925
SLC27A4	65.67059	0.160334	0.065598	2.444179	0.014518	NA
GFAP	7080.335	-0.16032	0.084593	-1.89517	0.05807	0.364493

TNFRSF1B	15.54249	-0.1603	0.084332	-1.90087	0.057319	NA
CD14	16.87269	-0.16027	0.083825	-1.91202	0.055874	NA
RBMS1	34.01456	-0.16023	0.075695	-2.11683	0.034274	NA
PSD4	5.412909	-0.1602	0.08338	-1.92127	0.054698	NA
TTYH1	178.1904	-0.16019	0.07846	-2.04166	0.041186	NA
NCAN	176.2161	0.16018	0.077795	2.059002	0.039494	NA
PION	24.71648	-0.16012	0.084074	-1.9045	0.056845	NA
TMED7	139.2869	0.160059	0.067969	2.354865	0.018529	NA
TMEM246	118.7555	0.160041	0.057328	2.791649	0.005244	NA
TMIE	11.58526	0.160021	0.08488	1.885257	0.059395	NA
LINC00634	7.590285	0.159971	0.083867	1.907436	0.056464	NA
PRTFDC1	31.90407	-0.15993	0.0777	-2.05835	0.039556	NA
CA14	17.70911	-0.15991	0.083906	-1.90579	0.056677	NA
SNORD12	16.50144	-0.15988	0.079403	-2.01357	0.044055	NA
HEPACAM	105.2161	-0.15988	0.075337	-2.12223	0.033819	NA
MYOF	38.69399	-0.15979	0.084745	-1.88551	0.059362	NA
GPR56	84.36532	-0.15969	0.081225	-1.96599	0.0493	NA
PTGES3L	2.913777	-0.15967	0.076706	-2.08161	0.037378	NA
CCDC92	318.6991	0.159659	0.051128	3.122744	0.001792	NA
PCDHGA5	88.51923	0.159619	0.078105	2.043655	0.040988	NA
PSMG1	28.99386	0.159614	0.072949	2.188032	0.028667	NA
LOC44017	3.751161	-0.15956	0.077011	-2.07197	0.038268	NA
ANKRD7	1.73152	-0.15956	0.0622	-2.56535	0.010307	NA
LURAP1L	90.98004	0.159467	0.066689	2.39122	0.016792	NA
HERC2P10	15.16814	0.159454	0.08491	1.877921	0.060392	NA
G0S2	8.384159	0.159441	0.079066	2.016559	0.043742	NA
PROZ	6.948163	0.159395	0.084574	1.884675	0.059474	NA
NUPR1	100.5596	-0.15938	0.081007	-1.96745	0.049131	NA
HTR1F	4.632406	0.159329	0.079472	2.004843	0.04498	NA
HMGB3P1	3.932328	0.159293	0.078257	2.035523	0.041798	NA
C12orf57	70.18065	-0.15925	0.081693	-1.94941	0.051247	NA
ZNF710	63.31222	-0.15924	0.076854	-2.07201	0.038264	NA
FAM179A	43.58174	-0.1592	0.084673	-1.88019	0.060083	NA
IL5	4.77286	-0.15917	0.078595	-2.02523	0.042843	NA
EAF2	17.29506	0.159167	0.079888	1.992384	0.046329	NA
AMER3	366.5572	0.159166	0.06215	2.560992	0.010437	0.198661
HLA-DOA	8.078069	-0.15916	0.076708	-2.07494	0.037992	NA
PPA2	123.5023	-0.15916	0.07351	-2.16514	0.030377	NA
RBM43	11.29739	-0.15916	0.0849	-1.87466	0.06084	NA
PRRT3-AS1	11.67514	-0.15909	0.084911	-1.87363	0.060982	NA
CCDC14	59.42404	-0.15904	0.078828	-2.01754	0.04364	NA
RBM3	111.9042	-0.15899	0.073172	-2.17282	0.029794	NA
DOCK5	155.6119	-0.15898	0.077089	-2.06224	0.039185	NA
RYBP	86.31871	0.158917	0.059208	2.684022	0.007274	NA
PRPH2	9.839943	0.158893	0.084933	1.870802	0.061373	NA
MLXIPL	231.983	-0.15885	0.058443	-2.71799	0.006568	NA
KRBOX1	9.213995	-0.15883	0.084682	-1.87564	0.060705	NA

NDUFB7	72.06955	-0.15874	0.080069	-1.98249	0.047424	NA
CECR6	32.55676	0.158729	0.075766	2.094999	0.036171	NA
FANCD2	16.6581	-0.15871	0.083523	-1.90021	0.057406	NA
ARHGAP30	15.97353	-0.1587	0.084654	-1.87468	0.060837	NA
MIR3911	20.0847	-0.1587	0.079503	-1.99611	0.045922	NA
COTL1	148.6671	-0.15869	0.064517	-2.45967	0.013906	NA
MRGPRD	4.025587	-0.15867	0.079197	-2.0035	0.045124	NA
INSM1	71.51478	0.15862	0.065665	2.415607	0.015709	NA
AKAP13	125.0371	-0.15856	0.067769	-2.33967	0.019301	NA
SNORD87	20.28686	-0.15853	0.083735	-1.89319	0.058333	NA
NEUROD1	741.4964	0.158431	0.063992	2.475781	0.013294	0.206483
ENTPD3	46.87195	0.158409	0.072742	2.177669	0.029431	NA
CRISPLD2	23.80224	-0.15829	0.084047	-1.88332	0.059657	NA
GINS4	8.581994	-0.15827	0.084914	-1.86388	0.062339	NA
CHGA	15.31993	0.158248	0.084317	1.876816	0.060543	NA
SNHG12	25.21418	-0.15823	0.074631	-2.12022	0.033987	NA
ACTG1P4	40.10555	-0.15818	0.074622	-2.1197	0.034031	NA
UBE2L6	65.66048	-0.15815	0.070325	-2.24877	0.024527	NA
FTH1P3	7.174477	-0.1581	0.084775	-1.86498	0.062185	NA
CHST9	4.461939	-0.15806	0.080552	-1.96221	0.049738	NA
ZNF597	20.90258	0.158038	0.078174	2.021616	0.043216	NA
CMTM1	21.07197	0.158033	0.080246	1.969358	0.048912	NA
PLIN3	17.96457	-0.15803	0.083717	-1.88763	0.059076	NA
COL6A3	8.888174	-0.1579	0.078566	-2.00977	0.044455	NA
LINC00161	1.525654	-0.15783	0.062807	-2.51288	0.011975	NA
LOC14913	14.70532	-0.15763	0.084335	-1.8691	0.061609	NA
MIR3613	1.435271	0.157547	0.061988	2.541558	0.011036	NA
LOC28646	4.99096	-0.1575	0.079717	-1.97577	0.048181	NA
DAXX	89.33176	0.157314	0.055826	2.817945	0.004833	NA
UBTF	475.2477	0.157251	0.053198	2.955945	0.003117	0.135775
SMTNL1	6.301867	-0.15724	0.084084	-1.87004	0.061478	NA
KCNT2	20.55638	-0.15724	0.084697	-1.85648	0.063385	NA
HSD17B8	3.86502	-0.15717	0.078183	-2.01025	0.044405	NA
GLRA1	17.14267	-0.15716	0.078757	-1.99555	0.045983	NA
HTRA1	221.0267	-0.1571	0.076874	-2.04366	0.040987	NA
ARRDC2	25.7735	-0.157	0.084018	-1.86865	0.061672	NA
DENND2C	14.91237	-0.15698	0.084905	-1.84886	0.064478	NA
SNORD32A	5.484421	-0.15681	0.066763	-2.34875	0.018837	NA
C9orf123	61.34689	-0.15677	0.076339	-2.05359	0.040015	NA
SYT13	236.29	0.156664	0.077338	2.025714	0.042794	NA
ZNF165	11.7028	0.156594	0.083432	1.876897	0.060532	NA
BOLA1	13.39093	-0.15654	0.082319	-1.9016	0.057224	NA
SNORA14B	88.6309	-0.15649	0.084169	-1.85921	0.062997	NA
TEK	37.34008	-0.15647	0.084887	-1.84324	0.065294	NA
DDIT4	215.2446	-0.15634	0.07907	-1.9773	0.048008	NA
ZNF703	27.65887	-0.15631	0.083205	-1.87865	0.060292	NA
RPS29	86.73849	-0.15627	0.084378	-1.85208	0.064014	NA

TAS2R14	19.7867	-0.15626	0.084129	-1.85735	0.063262	NA
FIGN	25.96962	-0.15625	0.084552	-1.84794	0.064611	NA
UNC119	49.74515	0.156226	0.0683	2.287353	0.022175	NA
SDHAP1	197.6925	-0.15621	0.08078	-1.9338	0.053138	NA
C12orf61	10.14006	-0.15619	0.084938	-1.83882	0.065941	NA
GYG2	17.31141	-0.15617	0.08392	-1.86098	0.062747	NA
HS3ST5	5.469771	0.156164	0.081327	1.9202	0.054833	NA
CYBB	23.9722	-0.15616	0.084659	-1.84453	0.065106	NA
SELK	75.14961	-0.15611	0.074866	-2.08521	0.037051	NA
ETFB	44.47551	-0.15608	0.080273	-1.94432	0.051857	NA
PPP4R4	72.07237	0.156013	0.081477	1.914794	0.055519	NA
LYSMD3	130.4093	0.156011	0.05226	2.985275	0.002833	NA
GSTZ1	7.732049	-0.15599	0.084685	-1.84199	0.065476	NA
YKT6	112.7287	0.15597	0.0526	2.965197	0.003025	NA
LOC64647	57.66277	-0.15596	0.059382	-2.62632	0.008631	NA
TMEM56-	5.519487	0.155949	0.081994	1.901951	0.057178	NA
ADAMTS4	21.48052	-0.15594	0.082358	-1.89339	0.058306	NA
TMEM72-	18.25123	-0.1559	0.084861	-1.83712	0.066192	NA
MRM1	8.006533	-0.15586	0.084771	-1.83861	0.065972	NA
LOC10050	105.5377	-0.15578	0.063707	-2.44529	0.014473	NA
LMF1	61.34894	-0.15573	0.07181	-2.16862	0.030112	NA
PAQR4	43.39888	0.155678	0.076103	2.045617	0.040794	NA
DCC	12.81535	-0.1556	0.084914	-1.83239	0.066893	NA
PKIG	63.55761	-0.1556	0.062296	-2.49766	0.012501	NA
ZMYM6NB	14.21041	-0.15559	0.084606	-1.83904	0.06591	NA
SCARNA3	3.762312	-0.15554	0.079344	-1.96033	0.049957	NA
ZNF720	39.73599	-0.15548	0.073482	-2.1159	0.034353	NA
KIAA1217	180.8707	-0.15542	0.080434	-1.93224	0.05333	NA
CYGB	45.31633	0.155293	0.079443	1.954767	0.050611	NA
SMIM11	27.37866	-0.15529	0.071675	-2.16655	0.030269	NA
EPHA10	8.336183	-0.15528	0.083374	-1.86247	0.062537	NA
CDK15	33.18033	-0.15521	0.079765	-1.94589	0.051668	NA
SLC24A5	32.75295	0.155201	0.078872	1.96774	0.049098	NA
TFPI	9.082131	-0.15519	0.084192	-1.84324	0.065294	NA
WDFY4	13.1487	-0.15515	0.084932	-1.82681	0.067728	NA
SLC5A12	0.791613	-0.15514	0.049597	-3.12803	0.00176	NA
EPB41L2	235.2165	-0.15507	0.072753	-2.13148	0.03305	NA
SNORD88C	2.645758	-0.15507	0.068673	-2.25804	0.023943	NA
ARHGDIB	14.82548	-0.15506	0.084319	-1.839	0.065915	NA
NAV1	303.841	-0.15494	0.061622	-2.51432	0.011926	NA
FAM96B	49.391	-0.15486	0.066111	-2.34239	0.019161	NA
CREB5	180.5808	0.154838	0.075483	2.051288	0.040239	NA
SFRP4	1.702903	-0.15482	0.066119	-2.34148	0.019207	NA
KRTAP5-1	109.6914	0.154785	0.083322	1.857668	0.063216	NA
PMP22	50.89314	-0.15467	0.083586	-1.85039	0.064258	NA
TAF1A	31.123	0.154664	0.071466	2.164159	0.030452	NA
DUSP8	184.5123	0.154658	0.061923	2.497577	0.012505	NA

SMARCD2	250.5604	-0.15463	0.058413	-2.64715	0.008117	NA
KCNMB3	23.69624	0.154603	0.084142	1.837408	0.06615	NA
MPG	36.88224	-0.15449	0.067915	-2.27468	0.022925	NA
GSTM2	40.15805	-0.15441	0.067864	-2.27533	0.022886	NA
FOXN3-AS	7.99861	-0.15438	0.084617	-1.8244	0.068092	NA
PGAP2	18.77721	-0.15433	0.07895	-1.95482	0.050604	NA
MIR4637	3.358194	0.154287	0.078809	1.957729	0.050262	NA
LOC10050	18.59846	-0.15424	0.082022	-1.88053	0.060036	NA
GRID2IP	90.47021	-0.15419	0.080743	-1.90962	0.056182	NA
CYTH4	6.215612	-0.15406	0.082812	-1.86035	0.062836	NA
CD109	13.37119	-0.15404	0.084865	-1.81507	0.069513	NA
LOC33879	115.5146	-0.15396	0.07281	-2.11455	0.034468	NA
SLCO2B1	57.86332	-0.15395	0.069412	-2.21788	0.026563	NA
ADAM12	18.54685	-0.15379	0.082801	-1.85736	0.06326	NA
RNLS	80.45267	-0.15372	0.06067	-2.53375	0.011285	NA
WDR19	295.2168	-0.15362	0.06271	-2.44975	0.014296	NA
HIC1	18.04458	0.15358	0.08489	1.809159	0.070426	NA
TMEM51	75.11291	0.15356	0.073623	2.085756	0.037001	NA
PTPN7	4.27329	-0.15352	0.078728	-1.94998	0.051178	NA
CNN3	278.9952	-0.15349	0.078902	-1.94538	0.051729	NA
TNFSF4	27.40235	0.15344	0.075775	2.024936	0.042874	NA
HSP90AA1	5937.993	0.153416	0.083787	1.831022	0.067097	0.378048
COL18A1-	5.920511	0.153392	0.069579	2.20458	0.027484	NA
TMEM178	167.5766	0.153386	0.059633	2.572167	0.010106	NA
PDE6B	22.49468	-0.15338	0.083273	-1.84192	0.065487	NA
CLDN11	40.35034	-0.15336	0.083228	-1.84265	0.06538	NA
C1QC	23.59621	-0.15329	0.066953	-2.28955	0.022047	NA
ZNF774	56.76787	0.153292	0.062096	2.468639	0.013563	NA
ARL4C	210.3389	0.153255	0.068649	2.232433	0.025586	NA
ERMAP	11.15057	-0.1532	0.08407	-1.82223	0.06842	NA
SOWAHA	59.63393	0.153192	0.07499	2.042838	0.041068	NA
LRRC39	22.52229	-0.15318	0.076492	-2.00257	0.045224	NA
CYP2A6	8.40943	-0.15318	0.084892	-1.80436	0.071175	NA
FAM109B	1.793432	-0.15315	0.067527	-2.268	0.023329	NA
FAM221A	31.82054	-0.15312	0.069724	-2.19608	0.028086	NA
CENPBD1	49.31353	0.153009	0.069953	2.187291	0.028721	NA
UCHL3	47.86144	0.152948	0.061627	2.481841	0.013071	NA
SEMA3G	172.7931	-0.15289	0.078999	-1.9353	0.052953	NA
TLR6	10.15457	-0.15285	0.083201	-1.83707	0.066199	NA
SYCE1L	7.013021	-0.15284	0.081379	-1.87808	0.060371	NA
HNF1A	8.540328	0.152792	0.084918	1.799294	0.071972	NA
CASP9	57.38824	0.152785	0.06403	2.386128	0.017027	NA
ZAP70	4.285862	0.15264	0.075251	2.028414	0.042518	NA
KIRREL2	4.4033	-0.15263	0.079847	-1.91158	0.05593	NA
FOXN3-AS	40.43904	-0.15262	0.075063	-2.03326	0.042026	NA
ENKD1	60.00884	-0.1526	0.070234	-2.17278	0.029797	NA
TFAP2A	26.15582	-0.1526	0.084936	-1.79664	0.072392	NA

EAPP	115.0073	-0.15258	0.059027	-2.58487	0.009742	NA
TMEM42	9.313404	-0.15257	0.084791	-1.7993	0.071971	NA
KIF23	4.30292	-0.15256	0.075691	-2.01558	0.043844	NA
FLJ22184	218.4285	0.152496	0.045316	3.365173	0.000765	NA
SNORA57	6.730467	-0.15249	0.080207	-1.90125	0.057269	NA
ACVR2B-A	31.8022	-0.15249	0.079316	-1.9225	0.054543	NA
SPATS2L	148.7264	0.152419	0.071541	2.130505	0.03313	NA
SNORD63	16.88194	-0.15237	0.079691	-1.91205	0.055869	NA
FABP7	214.8417	-0.15236	0.079646	-1.91296	0.055753	NA
SPAG1	85.58661	0.152355	0.062108	2.453063	0.014165	NA
PNPLA2	103.9007	-0.15235	0.062625	-2.43268	0.014988	NA
SFRP2	5.961911	0.1523	0.083549	1.822881	0.068321	NA
ZNF280B	63.30874	0.152284	0.064604	2.357208	0.018413	NA
ARHGAP6	13.51593	-0.15228	0.084932	-1.79301	0.072971	NA
TUBB2B	164.0646	-0.15219	0.084349	-1.80432	0.071181	NA
SEMA3B	46.28847	-0.15209	0.083596	-1.81935	0.068859	NA
NOMO3	12.48984	0.152007	0.084938	1.78963	0.073513	NA
LIMD2	9.329013	-0.152	0.084919	-1.78994	0.073464	NA
LGALS9	6.671121	-0.15192	0.079496	-1.91108	0.055994	NA
TESC	7.458784	0.151915	0.075496	2.012223	0.044196	NA
KIAA1024	296.5402	0.15191	0.051312	2.960532	0.003071	NA
ENTPD2	13.80494	0.151832	0.084329	1.800475	0.071786	NA
TADA1	57.56755	0.1518	0.065025	2.334496	0.01957	NA
NHLRC4	2.6485	0.151727	0.070386	2.155654	0.031111	NA
MAFF	19.4156	-0.1517	0.084917	-1.7864	0.074035	NA
SIGLEC7	1.983269	-0.15168	0.066495	-2.28114	0.02254	NA
CHRNB1	15.68616	-0.15162	0.082145	-1.84573	0.064932	NA
RACGAP1	34.34928	-0.1516	0.069745	-2.17358	0.029737	NA
RGL3	9.201911	-0.15158	0.082631	-1.83445	0.066588	NA
ARL11	2.366672	-0.15145	0.072864	-2.07846	0.037667	NA
MAPK1	624.742	0.151417	0.051454	2.942766	0.003253	0.137333
ZNF280C	51.03562	0.151403	0.061503	2.461729	0.013827	NA
DNM3	2008.576	0.151386	0.053266	2.842082	0.004482	0.14929
STXBP2	5.826372	-0.15126	0.0824	-1.83573	0.066398	NA
RGS12	396.8409	0.151237	0.071789	2.106686	0.035145	0.288416
LNP1	33.25056	-0.1512	0.077018	-1.96313	0.049631	NA
LOC10030	11.254	-0.15118	0.084561	-1.78787	0.073796	NA
DNAJA1	423.2455	0.151114	0.083817	1.802906	0.071403	0.38806
DLEC1	9.364612	-0.15104	0.084922	-1.77855	0.075313	NA
RPS16	122.6786	-0.15104	0.081818	-1.84599	0.064893	NA
CACNA1E	210.3995	-0.15102	0.082681	-1.82659	0.067761	NA
SNORD10	55.32784	-0.15101	0.084809	-1.78059	0.07498	NA
DDX19B	109.1318	-0.15096	0.069902	-2.15964	0.0308	NA
NXPH4	22.41978	0.15088	0.084881	1.777561	0.075476	NA
CBL	240.2813	0.150765	0.047325	3.185738	0.001444	NA
FAM163B	11.93481	0.150755	0.083894	1.79697	0.07234	NA
DNASE1L1	9.806438	-0.15072	0.084607	-1.78144	0.074841	NA

CUX2	24.87241	-0.15072	0.08381	-1.79832	0.072126	NA
ONECUT1	8.449459	0.150692	0.078561	1.918155	0.055091	NA
PSG4	18.88908	0.150691	0.083571	1.803151	0.071364	NA
SOX13	63.16019	-0.15067	0.077242	-1.95065	0.051099	NA
SCARNA4	9.548525	-0.15066	0.081139	-1.85678	0.063342	NA
JAZF1	139.6767	0.150657	0.062513	2.410007	0.015952	NA
TCTE3	3.842844	-0.15066	0.079868	-1.88632	0.059252	NA
HES6	15.16547	0.150588	0.084674	1.778456	0.075329	NA
SEPW1	255.8027	-0.15055	0.077	-1.95525	0.050553	NA
FLJ36777	4.665808	-0.15054	0.080582	-1.8682	0.061734	NA
MIR766	2.557391	-0.15042	0.073262	-2.05322	0.040051	NA
SNAP47	95.97659	0.150362	0.052909	2.841918	0.004484	NA
PLXNA1	285.4839	0.150298	0.083039	1.80997	0.0703	NA
DHRS4L2	8.060154	-0.15028	0.082287	-1.82628	0.067808	NA
C12orf29	58.11034	-0.15027	0.064937	-2.31408	0.020663	NA
MT2A	56.59257	-0.15023	0.084541	-1.77695	0.075577	NA
HIST3H2A	104.6126	-0.15014	0.082897	-1.81121	0.070108	NA
MORN1	165.9654	-0.15006	0.061826	-2.42708	0.015221	NA
RS1	4.836744	0.149934	0.080761	1.856519	0.06338	NA
PAPLN	60.24654	-0.1499	0.079368	-1.88872	0.05893	NA
PCDHB9	52.33721	-0.1498	0.082354	-1.81901	0.068909	NA
ABHD4	25.4365	-0.14978	0.075295	-1.98931	0.046667	NA
TIGD4	13.46664	0.149759	0.083208	1.799801	0.071892	NA
HSPA4	462.5379	0.149743	0.071009	2.108788	0.034963	0.288416
WDPCP	85.89473	-0.14974	0.057484	-2.60492	0.00919	NA
FAM118A	140.8387	0.149711	0.08352	1.792517	0.07305	NA
HS3ST1	97.0511	0.149699	0.060838	2.4606	0.01387	NA
PBXIP1	158.4301	-0.14968	0.073824	-2.02748	0.042613	NA
SLC18B1	51.18299	-0.14961	0.075762	-1.97467	0.048306	NA
PBLD	29.5593	-0.14957	0.070453	-2.12299	0.033754	NA
COL9A2	25.65609	-0.14953	0.081971	-1.82412	0.068135	NA
RNF6	291.8191	0.149494	0.059658	2.505857	0.012216	NA
ENHO	101.7284	-0.14949	0.080354	-1.86036	0.062834	NA
C5orf24	358.3277	0.149471	0.050871	2.938238	0.003301	0.137333
COL5A2	10.37348	0.149465	0.084457	1.769707	0.076776	NA
GRIK1-AS2	2.405809	-0.14945	0.071075	-2.10275	0.035487	NA
SNORD115	71.53405	-0.14944	0.083227	-1.79555	0.072567	NA
MAGT1	33.3269	-0.14944	0.073628	-2.02962	0.042395	NA
NDRG3	554.2505	0.149435	0.06151	2.429455	0.015122	0.216391
LITAF	40.20053	-0.14938	0.078302	-1.90779	0.056418	NA
C1orf131	28.39586	-0.14915	0.073643	-2.02534	0.042832	NA
RAB6B	415.6792	0.149085	0.055437	2.689243	0.007161	0.171303
SRGAP2D	201.4513	0.149021	0.073267	2.033935	0.041958	NA
PIGG	107.1205	-0.149	0.060065	-2.48068	0.013113	NA
SERTAD3	7.241935	-0.14898	0.084797	-1.75689	0.078937	NA
ARMCX6	13.65844	0.148973	0.083839	1.776893	0.075586	NA
GRIA1	612.4873	-0.14888	0.077253	-1.92714	0.053963	0.357364

FLRT1	86.93962	0.148818	0.069673	2.135971	0.032682	NA
CYP3A43	1.717498	-0.14879	0.063817	-2.33148	0.019728	NA
N4BP2L1	129.7076	-0.14875	0.064235	-2.31566	0.020577	NA
TTC23L	4.825245	-0.14873	0.07932	-1.87505	0.060786	NA
LRRC19	13.13893	-0.14871	0.083257	-1.78615	0.074075	NA
ARHGAP15	2.569781	-0.14871	0.072976	-2.03774	0.041576	NA
PLIN5	13.60539	-0.14866	0.084852	-1.75195	0.079783	NA
LOC10050	32.92847	-0.14862	0.075116	-1.97857	0.047864	NA
PCDHGA12	123.2702	0.14859	0.079491	1.869268	0.061586	NA
CITED1	2.721656	-0.14851	0.072695	-2.04287	0.041065	NA
MS4A6A	6.953924	-0.14848	0.067995	-2.18374	0.028982	NA
CYB561D2	17.85529	-0.14844	0.084059	-1.76589	0.077414	NA
FLJ41350	3.014599	-0.1484	0.075083	-1.97649	0.048099	NA
LOC10050	3.791693	-0.14834	0.078546	-1.88857	0.05895	NA
PSD3	3843.658	0.148303	0.07096	2.089961	0.036621	0.2931
LRRC56	23.8305	0.148247	0.084629	1.751739	0.079819	NA
SNORD96A	9.128471	-0.14823	0.080889	-1.83254	0.06687	NA
DAZAP1	192.5174	-0.14823	0.049445	-2.99782	0.002719	NA
TOR4A	2.471144	-0.14822	0.072247	-2.05152	0.040216	NA
ACSS3	32.65253	-0.14821	0.079057	-1.8747	0.060835	NA
SYK	11.72534	-0.14815	0.084453	-1.75422	0.079393	NA
ZNF470	195.6907	0.148013	0.052346	2.827596	0.00469	NA
ZNF561	93.7493	0.147925	0.05481	2.698893	0.006957	NA
CTBP1-AS1	52.79153	0.147913	0.065246	2.267005	0.02339	NA
MSTN	7.431561	-0.14789	0.083531	-1.77052	0.076641	NA
TSPYL6	39.28501	-0.14788	0.076281	-1.93863	0.052547	NA
NCOA5	204.4505	0.14786	0.065189	2.268187	0.023318	NA
STPG1	62.4075	0.147855	0.06041	2.447505	0.014385	NA
ZBTB21	128.1016	0.147849	0.059776	2.47336	0.013385	NA
RPS28	66.11642	-0.1478	0.076136	-1.94133	0.052219	NA
S100A16	21.24628	-0.14779	0.083137	-1.77768	0.075457	NA
SRSF11	1351.532	-0.14779	0.055621	-2.65712	0.007881	0.171303
MKNK1	129.9462	-0.14761	0.064398	-2.2921	0.0219	NA
RAP1GAP	613.8868	-0.14759	0.070892	-2.08187	0.037354	0.297915
LINC00092	9.477989	-0.14758	0.084895	-1.73842	0.082137	NA
TRIM67	168.663	0.14751	0.067166	2.196211	0.028077	NA
RPL13	467.2537	-0.14748	0.063489	-2.32289	0.020185	0.235785
PLEKHG7	3.8943	-0.14743	0.075604	-1.94997	0.051179	NA
GLIPR2	19.09805	-0.14737	0.084778	-1.7383	0.082158	NA
TMEM56	257.9471	0.147358	0.063967	2.303672	0.021241	NA
CLSPN	2.627655	-0.1473	0.068056	-2.16444	0.03043	NA
SLITRK4	539.1465	0.1473	0.058226	2.529812	0.011412	0.201925
MAGEC3	1.954221	-0.14728	0.066914	-2.20097	0.027738	NA
SPAG5	87.90371	0.147258	0.083895	1.755273	0.079213	NA
PLVAP	0.905035	-0.14726	0.050819	-2.89769	0.003759	NA
FBP1	0.760258	-0.14719	0.048847	-3.01331	0.002584	NA
CCDC24	16.1703	-0.14713	0.080936	-1.8179	0.06908	NA

SREK1IP1	118.9597	0.147092	0.051791	2.840093	0.00451	NA
SPG7	355.0169	-0.14705	0.054249	-2.7106	0.006716	0.167166
LYRM1	36.28512	-0.14703	0.082048	-1.79204	0.073126	NA
LINC00277	3.986589	0.146986	0.065907	2.230188	0.025735	NA
NOTCH2N	64.68114	-0.14698	0.07308	-2.0112	0.044304	NA
CCDC84	110.6213	-0.14693	0.063354	-2.31915	0.020387	NA
LOC10050	11.73899	-0.14691	0.083775	-1.75365	0.07949	NA
HLA-DRB1	22.70409	-0.1469	0.081891	-1.79381	0.072844	NA
CXorf57	8.055828	-0.14688	0.084931	-1.72939	0.083739	NA
CST3	460.2478	-0.14683	0.076949	-1.90812	0.056376	0.361261
CPT1A	73.38247	-0.14683	0.076705	-1.91416	0.055599	NA
SNORD105	7.73514	-0.14681	0.075693	-1.9395	0.052441	NA
SOCS2	15.67373	-0.14676	0.084241	-1.74214	0.081484	NA
CHAF1A	77.45677	-0.14673	0.067427	-2.17612	0.029547	NA
NDUFA4L2	33.86349	-0.14662	0.07311	-2.00545	0.044915	NA
PCDHB12	75.74574	-0.1466	0.068698	-2.13402	0.032841	NA
THEM5	3.426359	-0.1466	0.07856	-1.86604	0.062035	NA
FCGR3B	1.198723	0.146566	0.056323	2.602219	0.009262	NA
GDPD1	68.59251	-0.14656	0.060599	-2.41857	0.015582	NA
EFR3A	918.7088	0.146475	0.05875	2.493182	0.01266	0.205975
SNHG7	51.82604	-0.14647	0.080243	-1.82533	0.067951	NA
INPPL1	77.09942	-0.1464	0.074295	-1.97053	0.048777	NA
LOC10013	6.401514	-0.14638	0.084396	-1.73448	0.082833	NA
PLTP	41.57097	-0.14628	0.08079	-1.81059	0.070205	NA
SLC35D2	10.05046	-0.14625	0.084858	-1.72341	0.084814	NA
NKRF	101.3597	0.146228	0.049874	2.931958	0.003368	NA
INHA	3.97924	-0.14622	0.073224	-1.99685	0.045841	NA
TAP2	80.45	0.146197	0.076084	1.921535	0.054664	NA
ASH1L-AS1	13.08996	-0.14618	0.081581	-1.79189	0.07315	NA
ROBO3	23.06505	-0.14615	0.077211	-1.89292	0.058369	NA
TGFBR3L	16.99702	-0.14615	0.082663	-1.76802	0.077058	NA
CALN1	1330.712	0.146145	0.059794	2.444144	0.01452	0.212327
LOC72792	2.918126	-0.14615	0.048146	-3.03546	0.002402	NA
DSCAML1	71.96952	-0.14612	0.072506	-2.01523	0.043881	NA
SMIM13	271.3552	0.146079	0.054562	2.677302	0.007422	NA
COL8A2	39.4526	0.146058	0.077356	1.888138	0.059007	NA
ENDOG	7.837103	0.146	0.084935	1.718956	0.085622	NA
MRPL32	83.68108	-0.14597	0.067004	-2.17854	0.029366	NA
STXBP5L	2173.03	0.145951	0.052432	2.783601	0.005376	0.153366
DGCR10	60.10137	-0.14594	0.079674	-1.83177	0.066985	NA
KIAA0232	634.1604	0.145941	0.042098	3.466722	0.000527	0.075062
NOS1AP	272.9085	-0.14591	0.065048	-2.24306	0.024893	NA
ZHX1	369.2954	0.14583	0.044304	3.29156	0.000996	0.092144
SNORD67	33.48244	-0.14582	0.082374	-1.77027	0.076683	NA
ZNF252P-A	21.96422	0.145771	0.081623	1.78591	0.074114	NA
TUBA1C	12.05615	-0.14577	0.083309	-1.74974	0.080163	NA
COL13A1	235.7429	-0.14574	0.077263	-1.8863	0.059255	NA

DNHD1	219.327	-0.14563	0.065185	-2.23407	0.025478	NA
SAMD11	3.060104	0.14562	0.070187	2.07475	0.03801	NA
USF1	101.1219	-0.14562	0.049982	-2.91342	0.003575	NA
POGLUT1	46.74741	0.145513	0.070745	2.056876	0.039698	NA
IDH1-AS1	0.55838	-0.1455	0.043786	-3.32295	0.000891	NA
TMEM223	29.87199	-0.14547	0.071188	-2.04341	0.041012	NA
FAM136A	51.29498	0.145439	0.062643	2.321716	0.020248	NA
CNST	302.2239	0.145386	0.052653	2.76119	0.005759	NA
KLHL25	20.65626	0.145332	0.078254	1.85719	0.063284	NA
WASF2	149.5931	-0.14532	0.058665	-2.47715	0.013243	NA
TCF25	676.9431	-0.14531	0.052434	-2.77131	0.005583	0.155569
CASQ1	18.03742	-0.1453	0.079397	-1.83	0.06725	NA
SYT4	1146.028	0.145296	0.068801	2.111824	0.034702	0.288416
ITGAE	28.34186	-0.14523	0.078247	-1.85607	0.063443	NA
PAPPA2	12.32743	0.145211	0.084743	1.71355	0.086611	NA
C19orf57	22.33032	-0.1452	0.076059	-1.90905	0.056255	NA
MYO15B	157.2799	-0.14519	0.081306	-1.78569	0.07415	NA
SEZ6L	435.0505	0.145172	0.069904	2.076739	0.037826	0.299563
ST6GALNA	7.836492	-0.14514	0.082469	-1.7599	0.078425	NA
LOC10013	42.10974	-0.14507	0.074569	-1.94546	0.05172	NA
RNF150	179.0978	0.145008	0.062306	2.327361	0.019946	NA
CBLN3	396.4008	0.144998	0.084648	1.712944	0.086723	0.410078
NENF	49.16738	-0.14498	0.07647	-1.89585	0.057979	NA
TMEM68	34.48224	0.144935	0.070374	2.0595	0.039446	NA
SAPCD2	7.457544	-0.14488	0.082491	-1.75633	0.079033	NA
FGD4	133.9312	-0.14487	0.071206	-2.03447	0.041905	NA
MICALL2	78.29341	-0.1448	0.077912	-1.85844	0.063107	NA
MUM1	420.9292	-0.14477	0.048255	-3.00008	0.002699	0.130106
JMJD4	12.68099	0.144747	0.082877	1.74652	0.080721	NA
RTBDN	6.862122	-0.14474	0.081945	-1.76628	0.077348	NA
ITGB2	12.35298	-0.14468	0.078838	-1.83514	0.066484	NA
ST8SIA4	8.475722	-0.14465	0.084806	-1.70561	0.088081	NA
CAPS2	50.27395	-0.14462	0.06208	-2.32951	0.019832	NA
C19orf73	9.271514	0.144545	0.084856	1.70342	0.088489	NA
NTN5	6.736948	-0.14445	0.084142	-1.71675	0.086025	NA
DUSP7	47.08969	0.144445	0.075073	1.924062	0.054347	NA
PCDHGA7	109.9282	0.144425	0.081897	1.763491	0.077818	NA
SLC45A4	48.89793	0.1444	0.072552	1.990291	0.046559	NA
CDS1	149.1444	0.144374	0.067593	2.135938	0.032684	NA
KLF16	25.03963	0.144358	0.078709	1.834074	0.066643	NA
SEMA3C	13.11	0.144307	0.083199	1.734478	0.082833	NA
RAB41	6.228447	0.144298	0.083797	1.721998	0.08507	NA
GTF3C4	164.8841	0.144228	0.054418	2.650378	0.00804	NA
PABPC1L2	8.474094	0.144197	0.084883	1.698776	0.089361	NA
ZNF571	40.48459	0.14417	0.066965	2.15293	0.031324	NA
STAT4	5.460843	-0.14415	0.080686	-1.7865	0.074018	NA
KIAA0895	183.2599	0.144138	0.054123	2.663146	0.007741	NA

YPEL3	437.7585	-0.14408	0.065909	-2.18606	0.028811	0.270777
PPIH	29.82485	-0.14397	0.075358	-1.91044	0.056076	NA
AP1S3	20.25284	-0.14395	0.084468	-1.70419	0.088345	NA
ZNF252P	165.6233	0.143942	0.057293	2.512372	0.011992	NA
PIGL	45.75027	-0.14391	0.08469	-1.69927	0.089269	NA
OAT	115.0278	0.143758	0.061248	2.347128	0.018919	NA
LOC10050	21.64628	0.143756	0.082755	1.737136	0.082363	NA
ABCA17P	32.15396	-0.14376	0.082806	-1.73607	0.082552	NA
TEX40	10.2262	-0.14375	0.084813	-1.69496	0.090083	NA
SNORD21	21.32172	-0.14374	0.084939	-1.69228	0.090593	NA
MZT2A	18.55509	-0.14374	0.077956	-1.84384	0.065207	NA
ATHL1	10.19666	-0.14368	0.083591	-1.71891	0.085631	NA
APCDD1L-	3.17856	-0.14368	0.074185	-1.93681	0.052769	NA
ADAMTS1	255.493	0.143682	0.075711	1.897769	0.057727	NA
CERK	338.2441	-0.14368	0.064155	-2.2396	0.025117	0.248425
UNG	40.05584	-0.14367	0.067439	-2.13044	0.033135	NA
CTRL	18.85296	0.143628	0.08143	1.763816	0.077763	NA
ZSWIM3	31.26148	0.143589	0.075212	1.909125	0.056246	NA
RXFP4	16.04504	-0.14356	0.084114	-1.70673	0.087872	NA
ADAMTS9-	12.46411	-0.14348	0.084918	-1.68965	0.091094	NA
SCGN	79.12024	-0.14346	0.083779	-1.7124	0.086824	NA
TMA7	108.9567	-0.14346	0.074967	-1.91367	0.055662	NA
DOCK10	120.5285	-0.14345	0.079933	-1.79466	0.072708	NA
GORASP2	114.5346	0.143431	0.061002	2.351263	0.01871	NA
C9orf89	30.4993	-0.14341	0.07344	-1.9528	0.050844	NA
SULF1	35.60882	-0.14339	0.082328	-1.74167	0.081567	NA
ALDH1A2	9.096561	0.143375	0.078397	1.82884	0.067424	NA
LINGO4	22.27418	-0.14335	0.084174	-1.70306	0.088556	NA
NKAIN2	91.93204	0.143308	0.067866	2.111653	0.034716	NA
TEX9	27.06356	-0.1433	0.081373	-1.76097	0.078243	NA
GOLT1B	88.40343	-0.14325	0.079827	-1.79446	0.07274	NA
S100A9	8.773143	-0.14324	0.060647	-2.36182	0.018185	NA
TCF7	46.38324	-0.14311	0.076322	-1.87509	0.060781	NA
C1orf61	151.2705	-0.14307	0.07238	-1.97667	0.048078	NA
FAM65B	24.97805	-0.14304	0.080809	-1.77015	0.076702	NA
MIR5194	3.933244	-0.14303	0.076846	-1.86128	0.062704	NA
EMB	9.170213	-0.14301	0.084896	-1.68458	0.092069	NA
RTN4R	92.82984	0.143012	0.070981	2.014789	0.043927	NA
CRIP2	32.09824	-0.14301	0.076198	-1.87681	0.060544	NA
CD9	35.91672	-0.1429	0.084928	-1.68259	0.092454	NA
PDE8A	47.16997	-0.1429	0.079056	-1.80757	0.070674	NA
CTU2	25.41873	-0.1429	0.0762	-1.87526	0.060757	NA
MRPS12	10.75436	-0.14289	0.083988	-1.70131	0.088884	NA
TMSB10	186.8175	-0.14288	0.081166	-1.76037	0.078345	NA
CXorf36	19.73069	-0.14287	0.084939	-1.68208	0.092553	NA
LINC00854	4.060182	0.142848	0.079846	1.789048	0.073607	NA
MST4	25.1938	0.142802	0.077695	1.837984	0.066065	NA

CIRBP	808.8452	-0.14278	0.082538	-1.72989	0.083649	0.403119
RPL37A	292.7331	-0.14277	0.07918	-1.80306	0.071379	NA
L3MBTL4	5.467302	-0.14271	0.082057	-1.7391	0.082018	NA
LST1	2.371531	-0.14268	0.062496	-2.28301	0.02243	NA
MTFR2	1.400792	-0.14266	0.059394	-2.40188	0.016311	NA
FEM1C	283.0881	0.142617	0.050757	2.809784	0.004957	NA
LOC10012	5.091889	0.142593	0.078315	1.82075	0.068645	NA
KTN1-AS1	4.811573	-0.14259	0.080994	-1.76051	0.078322	NA
TXLNB	1.781717	-0.14258	0.063572	-2.24285	0.024907	NA
HBEGF	26.60599	0.14255	0.077789	1.832521	0.066874	NA
PXDC1	23.57897	-0.14254	0.083463	-1.70787	0.087661	NA
FMNL3	206.2634	-0.14251	0.06661	-2.13946	0.032398	NA
EIF4E3	201.1626	0.1425	0.055735	2.556757	0.010565	NA
FTH1	567.64	-0.14249	0.067211	-2.12	0.034006	0.288416
C1QA	8.599208	-0.14247	0.063086	-2.25829	0.023928	NA
TRO	116.2938	0.142436	0.064663	2.202756	0.027612	NA
MTMR11	22.90351	-0.14243	0.083217	-1.71154	0.086981	NA
USP46	307.3609	0.142402	0.045363	3.139148	0.001694	NA
SMC2	130.0349	0.142376	0.067048	2.123499	0.033712	NA
C21orf67	4.712353	0.142374	0.08098	1.758142	0.078723	NA
SLC6A6	196.8452	-0.14236	0.067974	-2.09434	0.036229	NA
CHST10	70.31731	0.142347	0.058662	2.426565	0.015243	NA
COL9A3	10.95059	-0.14233	0.083941	-1.69556	0.08997	NA
RASD2	22.07298	0.142308	0.080739	1.762571	0.077973	NA
TMEM127	122.4741	0.142296	0.056392	2.523319	0.011625	NA
NOLC1	361.2226	0.142219	0.049654	2.864198	0.004181	0.14568
CMKLR1	8.584388	-0.14221	0.077173	-1.84277	0.065362	NA
RNASE2	1.574902	-0.14218	0.060574	-2.34713	0.018919	NA
TMEM176	4.175771	-0.14216	0.071202	-1.9966	0.045869	NA
C1orf220	40.86947	-0.14213	0.075213	-1.88964	0.058806	NA
KIAA1211L	27.68722	-0.14201	0.084927	-1.6721	0.094505	NA
PCA3	25.62624	-0.142	0.083163	-1.70743	0.087742	NA
PDZD7	121.9359	0.141951	0.075915	1.869856	0.061504	NA
TEX2	343.5172	0.141904	0.049231	2.882396	0.003947	0.14186
ALDH1A1	451.2929	-0.14181	0.081541	-1.73915	0.082008	0.401988
DNASE1	85.71059	0.141805	0.068447	2.071751	0.038289	NA
CH25H	1.142585	-0.1418	0.051628	-2.74654	0.006023	NA
WDR66	22.88269	0.141793	0.083777	1.692512	0.090548	NA
SCAND1	23.71172	-0.14177	0.078339	-1.80968	0.070346	NA
CD300A	8.055358	-0.14176	0.08479	-1.67192	0.094541	NA
ATP4A	22.12262	0.141728	0.08377	1.691869	0.090671	NA
FAM13B	475.5334	0.141663	0.054064	2.620309	0.008785	0.180879
ABTB1	176.8869	-0.14166	0.056673	-2.49964	0.012432	NA
CLOCK	758.9671	0.14164	0.039848	3.55451	0.000379	0.075062
HRSP12	59.23169	-0.14163	0.080639	-1.75637	0.079025	NA
LOC10050	10.05953	-0.1416	0.082643	-1.71343	0.086634	NA
GXYLT2	2.497256	-0.1416	0.072033	-1.96577	0.049325	NA

BCL11B	5.96189	0.141544	0.066343	2.133508	0.032883	NA
FAM204A	117.9894	-0.14143	0.058655	-2.41118	0.015901	NA
CEP78	86.96122	0.141358	0.052549	2.689997	0.007145	NA
EFCAB1	1.898012	0.14133	0.062101	2.275795	0.022858	NA
GABRA3	7.522314	-0.14131	0.080028	-1.7658	0.07743	NA
OPALIN	24.82037	-0.14129	0.073038	-1.93446	0.053056	NA
CTNNA3	68.5811	-0.14121	0.074653	-1.89155	0.058551	NA
PCDHGA8	120.2432	0.141148	0.08149	1.732093	0.083257	NA
DUSP10	42.70962	-0.14113	0.084607	-1.66811	0.095294	NA
PARP12	7.868151	-0.1411	0.084321	-1.67335	0.094258	NA
ANKRD36B	6.506449	-0.14108	0.082985	-1.70012	0.089109	NA
MIR17HG	3.128933	-0.14108	0.068281	-2.06611	0.038818	NA
CD58	4.791436	-0.14105	0.081463	-1.7314	0.08338	NA
LAMTOR4	42.43597	-0.141	0.077827	-1.81172	0.07003	NA
PON2	142.0081	-0.14095	0.07714	-1.82716	0.067676	NA
LIN7C	534.0208	0.140933	0.055993	2.516992	0.011836	0.201925
SNORA63	10.43313	-0.14093	0.081929	-1.72018	0.0854	NA
AOC2	11.66961	-0.14087	0.084929	-1.65866	0.097185	NA
RREB1	44.93023	-0.14083	0.073422	-1.91815	0.055092	NA
UQCRQ	118.4606	-0.14083	0.078621	-1.79124	0.073255	NA
FAM111A	19.43004	-0.14081	0.080335	-1.75282	0.079634	NA
EFCAB9	6.023782	0.140771	0.082339	1.709652	0.08733	NA
MCM7	137.5455	-0.14073	0.06104	-2.30546	0.021141	NA
SAMD9	14.14309	-0.14063	0.08488	-1.65679	0.097563	NA
C8orf37	33.15224	-0.14062	0.072987	-1.92661	0.054028	NA
TSC22D4	91.24186	-0.14061	0.061843	-2.27369	0.022985	NA
ELAVL1	247.5565	0.140591	0.039764	3.535631	0.000407	NA
ZFPL1	21.84371	-0.14056	0.077631	-1.81057	0.070207	NA
MIER3	256.3626	0.140532	0.049668	2.829405	0.004663	NA
PLXNA4	140.6908	0.14052	0.080698	1.741314	0.081629	NA
ELFN2	14.75885	0.140457	0.083417	1.683793	0.092222	NA
MAN2B1	50.15167	-0.1404	0.064329	-2.18249	0.029073	NA
HUS1	106.6351	0.140368	0.056424	2.487731	0.012856	NA
BCAN	184.16	-0.14036	0.065762	-2.13435	0.032814	NA
TEC	2.531447	-0.14033	0.073992	-1.89651	0.057893	NA
CLK4	910.994	-0.14032	0.068201	-2.05747	0.039641	0.305061
SRRM3	821.6232	-0.1403	0.054396	-2.5792	0.009903	0.193485
MON1A	22.44142	0.140279	0.083318	1.683656	0.092248	NA
LOC15843	17.8212	-0.14027	0.084134	-1.66718	0.095478	NA
GINS3	32.29465	0.140243	0.075163	1.865846	0.062063	NA
PRKCE	488.4109	0.140132	0.048692	2.877914	0.004003	0.14186
URB2	36.57776	0.140103	0.077272	1.813112	0.069815	NA
PPIAP30	10.16925	-0.14007	0.084937	-1.64909	0.099129	NA
DUSP3	193.7929	0.140043	0.061014	2.295237	0.02172	NA
NUDT16P1	18.49795	-0.14001	0.083374	-1.6793	0.093095	NA
GAREM	209.1912	0.139937	0.053047	2.637995	0.00834	NA
FIBCD1	0.670515	0.139927	0.042694	3.277433	0.001048	NA

HIST1H4B	37.35075	0.139888	0.084783	1.649946	0.098954	NA
KCTD21	31.05848	0.139886	0.077813	1.797727	0.07222	NA
SIRPB2	2.407886	-0.13982	0.070778	-1.97544	0.048218	NA
GLIS3	28.65939	-0.13979	0.083677	-1.67059	0.094803	NA
THBS4	23.73179	-0.13977	0.080485	-1.73663	0.082452	NA
WRN	175.946	-0.13977	0.060439	-2.3126	0.020745	NA
C5orf27	3.93891	-0.1397	0.078544	-1.77862	0.075302	NA
TSGA10	125.8782	0.139686	0.064888	2.152741	0.031339	NA
MGAT3	236.6293	0.139672	0.068047	2.052572	0.040114	NA
ZNF184	65.06677	0.139621	0.055976	2.494321	0.01262	NA
SMAGP	7.687966	-0.13946	0.083883	-1.66253	0.096407	NA
PRSS30P	4.037135	-0.13941	0.079058	-1.7634	0.077834	NA
CPNE9	93.68031	0.139403	0.075	1.858696	0.06307	NA
GABRE	3.611954	-0.13939	0.072212	-1.93032	0.053567	NA
TCP1	261.9638	0.139375	0.056742	2.456277	0.014038	NA
PLA2G16	49.65629	-0.13935	0.075666	-1.84167	0.065523	NA
ATP5O	116.8437	-0.13929	0.076062	-1.83128	0.067059	NA
SUCLG2	22.85119	-0.13929	0.082509	-1.6882	0.091372	NA
RIN2	56.84568	-0.13927	0.066436	-2.09628	0.036057	NA
LUZP1	319.1962	0.139255	0.07051	1.974979	0.048271	NA
MORN2	15.6406	-0.13924	0.080671	-1.72609	0.084331	NA
FHOD3	57.58458	0.139236	0.078452	1.774799	0.075931	NA
TTC33	280.0176	0.139228	0.064145	2.170527	0.029967	NA
DDX58	90.5849	-0.1392	0.069636	-1.99894	0.045615	NA
RPL10	129.9867	-0.13919	0.070494	-1.97454	0.04832	NA
ACTR2	357.6343	0.139179	0.068664	2.026964	0.042666	0.318294
ABHD13	176.999	0.139124	0.049682	2.80026	0.005106	NA
DBH	4.797638	-0.13911	0.081225	-1.71271	0.086766	NA
LOC28475	2.044977	-0.13911	0.060748	-2.28986	0.022029	NA
PYGO1	37.777	0.139077	0.070052	1.985341	0.047107	NA
SIAH2	51.03547	0.139054	0.064305	2.1624	0.030587	NA
UQCRH	126.3505	-0.13898	0.076712	-1.81169	0.070034	NA
EFNB1	11.86888	0.138891	0.08449	1.643876	0.100202	NA
RPS15	491.5382	-0.13889	0.072909	-1.90499	0.056781	0.361261
ZMYND10	5.853375	-0.13889	0.081832	-1.69726	0.089647	NA
LOC25516	11.17508	0.138801	0.084907	1.634741	0.102103	NA
ADRB1	7.994219	0.13879	0.0849	1.634754	0.102101	NA
SPP1	137.6866	-0.13878	0.077357	-1.79408	0.072801	NA
ARCN1	399.5768	0.138754	0.055867	2.483655	0.013004	0.205975
LOC73066	2.225186	0.138709	0.068258	2.03211	0.042142	NA
LOC40094	4.434463	-0.13869	0.080436	-1.72418	0.084675	NA
FUT9	1979.706	0.138666	0.042996	3.225107	0.001259	0.095074
BID	78.50655	0.138615	0.067885	2.041907	0.041161	NA
SLC7A4	31.26432	0.138561	0.078025	1.775864	0.075755	NA
CLPB	100.42	0.138557	0.0582	2.380696	0.01728	NA
LMO4	85.60917	-0.13854	0.062353	-2.22191	0.02629	NA
RHEBL1	4.035268	-0.13852	0.078513	-1.76434	0.077675	NA

PURA	769.2127	0.138506	0.043982	3.149124	0.001638	0.101784
RPL17	7.631113	-0.13847	0.083692	-1.65453	0.09802	NA
WNT7B	6.006134	0.138459	0.080909	1.711283	0.087029	NA
CCDC173	12.36378	-0.13845	0.084074	-1.64675	0.09961	NA
NTRK3-AS	10.54832	-0.13844	0.084906	-1.63057	0.102981	NA
LOC10050	8.665408	-0.13842	0.083843	-1.65097	0.098745	NA
HSPB8	31.00939	-0.13841	0.084426	-1.6394	0.101131	NA
RPL27A	222.132	-0.13838	0.070973	-1.94978	0.051203	NA
FGFRL1	191.1785	-0.13837	0.074507	-1.85716	0.063289	NA
ZBTB18	2066.737	0.138331	0.052093	2.655463	0.00792	0.171303
UVSSA	116.7255	-0.1383	0.075491	-1.83197	0.066956	NA
ATP5S	26.10455	-0.13829	0.07402	-1.86833	0.061716	NA
SNORD68	345.4126	-0.13825	0.081382	-1.69873	0.08937	0.415114
ZCCHC10	45.25079	0.138226	0.068425	2.020097	0.043373	NA
LINC00475	8.844408	0.138206	0.084149	1.642396	0.100508	NA
KIF15	20.88759	0.138189	0.084903	1.627614	0.103607	NA
FZD10	3.530416	0.138151	0.073771	1.872702	0.06111	NA
WASH3P	11.67572	0.13814	0.08483	1.628432	0.103433	NA
SYT6	1.907129	-0.1381	0.065479	-2.10916	0.034931	NA
ST3GAL6	116.2182	-0.13808	0.056234	-2.45539	0.014073	NA
PHF13	23.94241	0.13807	0.080443	1.716358	0.086097	NA
IFITM3	28.54103	-0.13802	0.081657	-1.6902	0.090989	NA
SORCS3	72.43469	-0.138	0.078391	-1.76046	0.07833	NA
PNMAL1	236.2313	0.137998	0.063997	2.156304	0.03106	NA
DNAI1	6.233348	-0.13794	0.081631	-1.68974	0.091077	NA
NDP	40.62441	0.137905	0.076306	1.807267	0.070721	NA
NPRL3	150.2955	-0.13789	0.050177	-2.74814	0.005994	NA
SNORA3	5.656409	-0.13789	0.077454	-1.78023	0.075038	NA
PLOD2	93.78419	-0.13789	0.068756	-2.00542	0.044918	NA
GOLGA8I	5.434734	-0.13788	0.082808	-1.66511	0.09589	NA
SLC17A7	548.1609	0.13784	0.07492	1.839815	0.065795	0.375222
MAN2C1	192.1273	-0.13781	0.056146	-2.45446	0.01411	NA
NR4A3	32.73268	0.13773	0.077274	1.782347	0.074693	NA
SFMBT1	108.7171	0.137726	0.059833	2.301864	0.021343	NA
APOD	183.5328	-0.13772	0.083051	-1.65822	0.097274	NA
SNORD114	5.16526	-0.13759	0.079641	-1.72761	0.084058	NA
PCDHGA6	96.35688	0.137578	0.079354	1.733716	0.082968	NA
LOC10013	12.6233	0.137551	0.083146	1.654336	0.098059	NA
SNX33	55.04704	-0.13751	0.071496	-1.92329	0.054443	NA
TULP4	982.859	0.137486	0.059761	2.300591	0.021415	0.23963
ZNF81	128.5396	0.137482	0.057878	2.375389	0.01753	NA
LOC10013	17.54281	-0.13745	0.084231	-1.63187	0.102708	NA
SLC8A1	296.9032	0.137395	0.079859	1.720462	0.085348	NA
ARMCX2	83.21344	0.137389	0.060693	2.263682	0.023594	NA
TATDN2	185.0518	0.137359	0.051091	2.688496	0.007177	NA
COL20A1	19.78573	-0.13736	0.084933	-1.61724	0.105826	NA
HOOK1	271.4096	0.137307	0.048781	2.81473	0.004882	NA

ENTPD3-A	8.17874	0.137306	0.084928	1.616725	0.105938	NA
BLOC1S3	12.6227	-0.13728	0.08346	-1.64484	0.100003	NA
ALG8	19.13315	0.137206	0.08137	1.6862	0.091757	NA
TCP11L2	94.39585	-0.1372	0.05658	-2.4248	0.015317	NA
FEM1B	191.0539	0.137124	0.056841	2.412406	0.015848	NA
PLCH2	491.3059	0.137094	0.081703	1.677956	0.093356	0.419831
LOC10013	16.53567	0.137037	0.084327	1.625064	0.104149	NA
WDYHV1	23.56625	0.136983	0.082499	1.660431	0.096828	NA
JAZF1-AS1	11.84725	0.136964	0.084909	1.613062	0.106731	NA
MFI2-AS1	29.12033	-0.13696	0.075958	-1.80305	0.071381	NA
LENG8	1522.465	-0.13695	0.050377	-2.71861	0.006556	0.16684
ZCCHC24	142.7441	-0.13687	0.065966	-2.07478	0.038007	NA
TMEM258	5.204697	-0.1368	0.08372	-1.63399	0.102262	NA
TMEM126	60.10717	-0.13679	0.075961	-1.80075	0.071742	NA
MXRA5	3.510089	0.136766	0.068717	1.990284	0.04656	NA
MESP1	8.117066	-0.13676	0.084258	-1.62316	0.104554	NA
NIP7	21.57471	0.13675	0.080288	1.703234	0.088524	NA
EHHADH	8.039027	-0.13675	0.083539	-1.63691	0.10165	NA
VCAM1	10.32112	0.136735	0.070357	1.943443	0.051963	NA
LOC33980	10.42868	-0.13668	0.084931	-1.60926	0.107559	NA
SLC16A8	20.37776	-0.13666	0.084021	-1.62652	0.10384	NA
DCXR	71.80726	-0.13659	0.064187	-2.12793	0.033343	NA
FAM153A	37.51229	-0.13654	0.083652	-1.63225	0.102628	NA
LOC15843	9.197437	-0.13652	0.081489	-1.67532	0.093871	NA
SCIN	20.55311	-0.13651	0.074221	-1.83927	0.065876	NA
HRASLS	2.538377	-0.1365	0.070108	-1.94706	0.051528	NA
C11orf75	3.813526	-0.13649	0.080434	-1.69696	0.089705	NA
MRPL4	49.523	-0.13643	0.061845	-2.20599	0.027384	NA
SYNDIG1L	2.849952	-0.13643	0.075053	-1.81774	0.069104	NA
MFSD8	107.6879	-0.13638	0.067813	-2.01119	0.044306	NA
LOC10013	33.31205	-0.13637	0.077715	-1.75476	0.079301	NA
RPLP2	218.5942	-0.13636	0.065467	-2.08292	0.037258	NA
ZNF784	24.37151	-0.13634	0.073561	-1.85341	0.063824	NA
LDHD	22.87742	-0.1363	0.079061	-1.72405	0.084699	NA
BTN1A1	4.705116	-0.13625	0.077358	-1.76125	0.078197	NA
METTL7A	407.1267	-0.1362	0.066907	-2.0357	0.041781	0.316501
CD86	4.689063	-0.1362	0.073552	-1.85174	0.064063	NA
FTL	268.8481	-0.13617	0.075167	-1.81157	0.070053	NA
LOC15117	20.50797	0.136163	0.084926	1.603325	0.108863	NA
PALD1	65.84502	-0.13616	0.076256	-1.78561	0.074163	NA
FLNC	60.71386	-0.13609	0.083765	-1.62462	0.104244	NA
ABO	10.32314	0.136037	0.081971	1.65958	0.096999	NA
LOC10050	14.59139	-0.13601	0.081811	-1.66246	0.096422	NA
ZFAND6	158.1712	-0.13598	0.060899	-2.23289	0.025556	NA
JARID2	721.0708	-0.13598	0.053898	-2.52287	0.01164	0.201925
ADI1	59.76496	-0.13596	0.07332	-1.85432	0.063694	NA
FAM186A	12.34546	-0.13596	0.083487	-1.62847	0.103425	NA

MIR575	1.187903	-0.13593	0.057026	-2.38372	0.017139	NA
CRYBG3	93.45679	-0.13593	0.073785	-1.8422	0.065445	NA
UNC5CL	5.718836	-0.13589	0.076723	-1.77123	0.076523	NA
ALPL	15.73855	0.135881	0.084178	1.614224	0.106479	NA
SNORD50A	15.59544	-0.13588	0.077976	-1.7426	0.081403	NA
ZNF286A	86.31123	0.135878	0.053634	2.533426	0.011295	NA
CABLES1	190.0597	-0.13587	0.084273	-1.61231	0.106895	NA
RNF144A-	68.99105	0.135836	0.068105	1.994516	0.046096	NA
THAP2	100.4176	0.135813	0.063771	2.129702	0.033196	NA
DSEL	298.2552	0.135807	0.066391	2.045578	0.040798	NA
STOML1	14.33148	0.135801	0.083156	1.633073	0.102454	NA
ADAMTS9	91.59464	-0.13578	0.073361	-1.85088	0.064186	NA
DNAJC1	58.3616	-0.13577	0.059982	-2.26352	0.023604	NA
LPHN2	78.53656	-0.1357	0.084898	-1.59843	0.109947	NA
NUDT10	40.65114	-0.13569	0.074013	-1.83336	0.066749	NA
S100A6	203.8678	-0.13569	0.070876	-1.91445	0.055563	NA
SNORD99	67.75791	-0.13566	0.079267	-1.71138	0.087011	NA
EPB41L4A-	108.9383	-0.13563	0.074247	-1.82678	0.067733	NA
P2RX5-TAX	27.23923	0.13563	0.080874	1.677049	0.093533	NA
CALCA	5.145535	-0.13561	0.075589	-1.79409	0.072798	NA
RPF2	46.78496	0.135579	0.066072	2.051987	0.040171	NA
POP1	60.18635	0.13557	0.068061	1.9919	0.046382	NA
MCM5	50.10889	-0.13556	0.07203	-1.88204	0.05983	NA
SNORD116	8.313424	-0.13552	0.083499	-1.62297	0.104596	NA
TMEM251	12.69821	0.135502	0.083346	1.625786	0.103995	NA
MBL1P	11.11332	0.135472	0.084709	1.599256	0.109764	NA
SPDYA	10.90403	0.135414	0.084903	1.594918	0.110731	NA
SNORA21	17.03097	-0.1354	0.083912	-1.6136	0.106613	NA
GRIP2	28.98535	-0.13537	0.082738	-1.63618	0.101803	NA
UTP14C	146.1629	0.135327	0.051637	2.62074	0.008774	NA
MYO5C	556.3274	-0.13529	0.07536	-1.79525	0.072614	0.389252
FAM155B	36.78863	0.135289	0.06912	1.9573	0.050312	NA
PADI2	110.7394	-0.13524	0.080151	-1.68735	0.091536	NA
CCNG2	509.3523	0.135226	0.059929	2.256442	0.024043	0.246583
RAB11FIP1	58.82544	-0.13521	0.071568	-1.88918	0.058868	NA
C1QL1	10.97319	-0.1352	0.084936	-1.59173	0.111445	NA
DGCR11	31.00803	-0.13517	0.074812	-1.80681	0.070792	NA
DUS2L	21.81336	-0.13516	0.082661	-1.63507	0.102033	NA
ANGPTL7	32.00905	0.135131	0.074999	1.801781	0.07158	NA
TTC3P1	66.34425	0.135129	0.066431	2.034125	0.041939	NA
FXYD7	10.23481	-0.13511	0.082341	-1.64091	0.100817	NA
ZNF567	116.1481	0.135101	0.05906	2.287529	0.022165	NA
CPB2-AS1	25.25896	-0.13509	0.076541	-1.76488	0.077585	NA
NAIF1	11.9724	0.135072	0.084819	1.592478	0.111277	NA
NKX2-2	31.81117	-0.13504	0.080095	-1.68596	0.091803	NA
RPL26	355.6378	-0.13503	0.077026	-1.75306	0.079592	0.397742
SIX5	3.79688	-0.13503	0.080356	-1.68041	0.092877	NA

PLEKHH2	152.7832	-0.13502	0.071523	-1.88783	0.059049	NA
SOCS6	150.7406	0.134942	0.049807	2.70927	0.006743	NA
43170	6.336362	-0.13493	0.082086	-1.6438	0.100217	NA
LRP5	12.36942	0.134831	0.084733	1.591237	0.111556	NA
MAPK6	490.0018	0.13482	0.069082	1.951606	0.050985	0.348396
MCF2L-AS	31.25877	-0.13482	0.075541	-1.78466	0.074316	NA
BDH2	32.34881	-0.1348	0.082194	-1.64005	0.100996	NA
RNF126	46.45646	-0.13477	0.067527	-1.99574	0.045962	NA
LRRN4	7.478672	-0.13469	0.083263	-1.61768	0.105731	NA
PCP4	209.2688	-0.13462	0.084915	-1.58541	0.112874	NA
RPL14	248.5408	-0.13462	0.071608	-1.87999	0.060109	NA
EQTN	1.96811	-0.13455	0.064976	-2.0708	0.038377	NA
SP140L	7.011037	-0.13451	0.082867	-1.62324	0.104537	NA
RAB25	0.817984	-0.13451	0.051721	-2.60071	0.009303	NA
PXDNL	5.54934	-0.1345	0.082661	-1.62716	0.103703	NA
WDR81	80.48571	-0.1345	0.05835	-2.30504	0.021164	NA
FGF2	97.99176	-0.13448	0.081661	-1.64683	0.099593	NA
LOC10050	0.790082	-0.13447	0.049504	-2.71628	0.006602	NA
PGAM2	68.73099	0.134447	0.066819	2.012099	0.044209	NA
FAM200A	65.15715	0.134398	0.058409	2.300995	0.021392	NA
CCDC22	46.09141	-0.13438	0.063762	-2.10749	0.035075	NA
UNKL	245.2395	-0.13438	0.058816	-2.2847	0.02233	NA
FBXO33	88.27281	-0.13437	0.05314	-2.52852	0.011455	NA
PCDHB2	20.13478	0.134335	0.082945	1.619571	0.105324	NA
MYL6B	45.06765	-0.13431	0.070858	-1.89542	0.058036	NA
SNX16	41.37074	0.134302	0.067802	1.98079	0.047615	NA
C7orf29	7.7851	0.134284	0.082968	1.618492	0.105557	NA
NIPAL1	3.525147	0.134242	0.076491	1.754999	0.07926	NA
PICK1	113.2603	-0.13423	0.06293	-2.13297	0.032927	NA
SNORA26	10.19798	-0.13422	0.082751	-1.62193	0.104818	NA
NRG1	59.07805	-0.1341	0.069111	-1.94035	0.052338	NA
LIMK2	189.0595	-0.13404	0.071028	-1.88711	0.059145	NA
TRAF5	14.83544	-0.134	0.083991	-1.59538	0.110628	NA
MIR100HG	52.2007	-0.134	0.07818	-1.71394	0.086539	NA
C16orf59	4.197861	0.133966	0.078836	1.699289	0.089265	NA
TMEM259	340.691	-0.13395	0.042916	-3.1212	0.001801	0.101784
LIPC	0.950227	0.133932	0.052442	2.553921	0.010652	NA
MYCL1	5.206271	-0.13393	0.083687	-1.6004	0.109511	NA
LOC10050	18.86475	-0.13393	0.080407	-1.66563	0.095787	NA
CAMTA1	1077.72	0.133921	0.049577	2.701247	0.006908	0.170072
C10orf88	34.3056	0.133911	0.067247	1.991336	0.046444	NA
GSTK1	36.81378	-0.13388	0.073634	-1.81819	0.069035	NA
WDR25	27.72373	-0.13388	0.076973	-1.73926	0.081989	NA
PVRIG	406.4727	-0.13384	0.078081	-1.71415	0.086501	0.410078
APOBEC2	5.426078	-0.13383	0.078968	-1.69475	0.090123	NA
BEND5	13.32982	-0.13382	0.08435	-1.58654	0.112616	NA
HTR5A	91.00738	0.133823	0.078536	1.703962	0.088388	NA

LYRM5	47.23036	-0.13375	0.075372	-1.77447	0.075986	NA
CBLN1	752.2753	0.133683	0.07117	1.878354	0.060333	0.367348
FRRS1L	106.7235	0.133655	0.058182	2.297193	0.021608	NA
CLMN	151.0327	-0.13365	0.075952	-1.75962	0.078472	NA
LAT2	23.97491	-0.13363	0.08182	-1.63321	0.102425	NA
NEUROD6	24.20969	0.133564	0.077325	1.727314	0.084111	NA
STIP1	371.3293	0.133555	0.084626	1.57817	0.114527	0.465042
NGB	3.987969	0.133528	0.073191	1.824374	0.068095	NA
MIR337	1.11688	-0.13346	0.057186	-2.33373	0.01961	NA
SWI5	23.02219	-0.13345	0.074965	-1.78019	0.075045	NA
PECAM1	30.5105	-0.13344	0.084812	-1.57341	0.115623	NA
CROCCP2	281.1046	-0.13344	0.073118	-1.82506	0.067993	NA
SULT1A2	24.4468	-0.13318	0.084656	-1.57322	0.115667	NA
FOXE1	3.482975	-0.13311	0.069696	-1.90981	0.056158	NA
NAP1L3	351.7818	0.13308	0.060678	2.193235	0.02829	0.266991
GABPB1	71.20413	0.13306	0.062961	2.113385	0.034568	NA
PLXNB3	277.8415	-0.13301	0.067545	-1.96914	0.048937	NA
LEPRE1	66.37638	-0.133	0.062234	-2.13712	0.032588	NA
KDM5B-AS	2.553302	-0.13298	0.072217	-1.84144	0.065556	NA
FAM90A25	7.568115	0.132974	0.082589	1.610073	0.107382	NA
SPARC	773.9203	-0.13292	0.080779	-1.64551	0.099865	0.436963
IGDCC3	41.29447	0.132917	0.067812	1.960061	0.049989	NA
SYN2	493.5214	0.132888	0.044356	2.995962	0.002736	0.130106
MIR5695	1.743149	0.132863	0.064357	2.064482	0.038972	NA
TK2	209.9481	-0.13284	0.058004	-2.29017	0.022011	NA
ATP11A	221.1663	0.132803	0.050624	2.623307	0.008708	NA
KLHDC9	11.13567	-0.1328	0.084918	-1.56387	0.117849	NA
HIST1H2BI	2.065017	-0.13279	0.06329	-2.09807	0.035899	NA
VPS25	15.06121	-0.1326	0.081573	-1.62553	0.104049	NA
GABARAP	131.0623	-0.13259	0.070141	-1.89033	0.058714	NA
SDK1	21.03969	-0.13254	0.083271	-1.59166	0.11146	NA
SIGIRR	3.616404	-0.13243	0.076715	-1.72631	0.084291	NA
KCNS2	4.535559	-0.13242	0.081218	-1.63048	0.103	NA
ATMIN	229.9314	0.132414	0.065737	2.014309	0.043977	NA
KLRG1	16.09236	-0.13241	0.082552	-1.60401	0.108713	NA
MIR4292	4.212485	0.132403	0.078818	1.679851	0.092986	NA
STAP2	11.15137	-0.13236	0.084244	-1.57117	0.116142	NA
RPL35A	182.393	-0.13235	0.08038	-1.64656	0.099649	NA
GABRB3	337.6968	0.132346	0.049386	2.679841	0.007366	0.171303
FLJ36000	4.185911	0.132321	0.058246	2.271753	0.023101	NA
OVGP1	29.21578	-0.13232	0.079678	-1.66068	0.096778	NA
ZNF534	20.28408	-0.1323	0.084892	-1.55848	0.11912	NA
ATP1A1	1147.589	0.132265	0.071275	1.855709	0.063495	0.371619
CAT	40.33963	-0.13226	0.084102	-1.57266	0.115798	NA
TMEM35	77.99535	0.132262	0.069049	1.915479	0.055431	NA
KLF12	358.99	0.132231	0.04961	2.665428	0.007689	0.171303
LOC10049	14.43475	0.132176	0.08432	1.56755	0.116986	NA

SLAIN2	221.1783	0.132143	0.045607	2.897425	0.003762	NA
PLAG1	82.4054	-0.13208	0.062204	-2.12326	0.033732	NA
SASH3	5.540455	-0.13206	0.077596	-1.70194	0.088766	NA
BEAN1	31.86692	-0.13206	0.081423	-1.62188	0.104828	NA
ZNF256	73.42687	0.13205	0.067205	1.964874	0.049429	NA
AATK-AS1	7.331908	0.132025	0.084743	1.557948	0.119245	NA
LOC40132	68.58964	0.131994	0.066166	1.994885	0.046055	NA
KLHL35	10.96764	-0.13198	0.084913	-1.55424	0.120127	NA
AQP9	6.395169	0.131957	0.078739	1.675868	0.093764	NA
METTL5	29.53118	0.131949	0.074658	1.767375	0.077165	NA
DNAH1	273.1279	-0.13189	0.07263	-1.81599	0.069372	NA
HOMER1	327.7	0.131874	0.043104	3.05941	0.002218	NA
LOC10013	0.849378	-0.13186	0.049753	-2.65033	0.008041	NA
ARL6IP5	152.0229	0.131815	0.067552	1.951326	0.051018	NA
SGK2	10.69386	-0.13182	0.084938	-1.55189	0.120688	NA
PCDHB10	48.27288	-0.13181	0.078077	-1.68816	0.09138	NA
LHX4	23.25841	0.131802	0.081695	1.613341	0.10667	NA
BRCA2	10.34429	-0.13179	0.084915	-1.55204	0.120652	NA
PTPLAD1	342.703	0.131721	0.057624	2.285883	0.022261	0.23963
SNORD90	14.33758	-0.13172	0.07502	-1.75574	0.079132	NA
BCOR	133.1602	0.131688	0.063169	2.084681	0.037098	NA
CACNG8	55.05241	-0.13168	0.084423	-1.5598	0.118807	NA
RPL41	81.65818	-0.13166	0.082062	-1.60442	0.108623	NA
F10	7.620369	-0.13158	0.08469	-1.55369	0.120258	NA
CAPS	14.31695	-0.13158	0.084723	-1.55308	0.120403	NA
LOC38970	3.392444	0.131553	0.077856	1.689712	0.091083	NA
PRSS55	2.693468	-0.13153	0.065128	-2.01956	0.043429	NA
DLEU2L	27.20175	-0.13152	0.075721	-1.7369	0.082405	NA
PRLR	1.525572	0.1315	0.059544	2.208455	0.027213	NA
TM4SF18	7.731228	0.131497	0.077382	1.699329	0.089257	NA
SUN2	320.875	-0.13149	0.059978	-2.19227	0.02836	NA
KCTD15	64.53363	0.131481	0.072427	1.815355	0.069469	NA
WIF1	10.71275	-0.13143	0.072873	-1.80359	0.071295	NA
ZBTB8A	100.1013	0.13141	0.047928	2.74183	0.00611	NA
RRP7B	110.9624	-0.13141	0.069229	-1.89813	0.057679	NA
PUS1	33.76676	0.131351	0.06992	1.878574	0.060303	NA
TVP23B	30.53302	0.131329	0.076183	1.723863	0.084733	NA
MRPL23	30.33179	-0.13133	0.073236	-1.79321	0.07294	NA
SNORD89	410.9341	-0.13131	0.084508	-1.55379	0.120235	0.472798
CSNK2A2	62.01796	0.131283	0.055612	2.360691	0.018241	NA
COL23A1	40.4741	-0.13127	0.084413	-1.5551	0.119923	NA
CFLAR-AS1	15.28363	-0.13123	0.083292	-1.5756	0.115118	NA
MEIS3	43.89218	-0.13123	0.084927	-1.54519	0.1223	NA
LRRC33	3.23613	0.131226	0.07271	1.804789	0.071108	NA
EPDR1	327.7535	0.131226	0.073084	1.795556	0.072565	NA
ERVV-1	5.239262	0.131205	0.077399	1.695181	0.090041	NA
ZSCAN20	74.3562	0.131197	0.059441	2.207157	0.027303	NA

SORCS1	16.70752	-0.13117	0.084152	-1.55876	0.119052	NA
LOC10012	9.593063	-0.13114	0.082209	-1.59516	0.110676	NA
SDC4	84.54811	-0.13112	0.084917	-1.54412	0.122559	NA
EIF5A2	17.86087	-0.1311	0.084924	-1.54375	0.12265	NA
MIR3175	3.18783	0.131101	0.076578	1.711996	0.086897	NA
CCNB3	4.036796	-0.13107	0.076092	-1.72253	0.084973	NA
SEPHS1	93.90133	0.131071	0.062574	2.094639	0.036203	NA
KCTD10	82.4092	0.131071	0.057883	2.264413	0.023549	NA
CMTM5	5.806143	-0.13105	0.077163	-1.69835	0.089442	NA
EIF4G3	839.6219	0.131044	0.039149	3.347363	0.000816	0.084436
DENND2D	15.31804	-0.13104	0.082332	-1.59162	0.111469	NA
SLC6A11	25.80753	-0.13103	0.084898	-1.54338	0.122738	NA
TP53BP1	1192.164	0.130992	0.04463	2.935063	0.003335	0.137333
ABCA10	153.881	-0.13098	0.074613	-1.75547	0.079179	NA
CDK6	49.19724	-0.13098	0.0823	-1.59144	0.11151	NA
STAT5A	38.83628	-0.13095	0.077267	-1.6948	0.090113	NA
C9orf174	1.136955	-0.13091	0.054905	-2.3844	0.017107	NA
PACS1	273.5385	0.130914	0.041709	3.138721	0.001697	NA
YOD1	103.9672	0.130903	0.05829	2.245722	0.024722	NA
LOC28434	8.324067	0.130857	0.081132	1.612889	0.106769	NA
C11orf34	12.43136	-0.13085	0.084713	-1.54466	0.122429	NA
KCNJ16	177.4478	-0.13081	0.079043	-1.65487	0.09795	NA
DDX59	66.59976	-0.1308	0.059222	-2.20862	0.027201	NA
KIAA1984	111.4869	-0.13079	0.066788	-1.95834	0.05019	NA
CEP152	99.0405	-0.13075	0.084189	-1.55303	0.120416	NA
CSNK1E	246.9784	-0.13075	0.04851	-2.69529	0.007033	NA
ADAMTSL3	53.89661	-0.13074	0.076648	-1.70567	0.088069	NA
ZFP36L2	45.80112	-0.13072	0.079314	-1.64816	0.09932	NA
CTSL1	81.24284	-0.13072	0.061187	-2.13637	0.032649	NA
TFAP4	94.5624	-0.13069	0.060741	-2.15168	0.031423	NA
MANSC1	49.83277	0.130668	0.06663	1.961083	0.049869	NA
EIF4G2	1105.604	0.130654	0.052287	2.49876	0.012463	0.205975
RXRA	90.47769	-0.13062	0.068995	-1.89313	0.058341	NA
ZNF524	11.23584	-0.13059	0.084471	-1.54601	0.122102	NA
DOCK2	15.46059	-0.13058	0.08454	-1.54463	0.122435	NA
LRCH2	168.5316	0.130491	0.047225	2.763189	0.005724	NA
SC5DL	322.7675	0.13047	0.054757	2.382696	0.017186	NA
IL5RA	8.85015	0.130421	0.082479	1.581255	0.11382	NA
CLEC4E	1.716266	-0.13042	0.056433	-2.311	0.020833	NA
CDR1	165065.7	0.130413	0.068455	1.905096	0.056768	0.361261
ZNF185	46.42688	-0.13039	0.081887	-1.59228	0.111323	NA
TBXAS1	7.386527	-0.13038	0.082767	-1.57525	0.115198	NA
KDM3A	348.2205	-0.13035	0.054555	-2.38924	0.016883	0.224654
TERC	10.6392	-0.13032	0.084839	-1.53613	0.124507	NA
TXNDC16	271.7836	-0.13028	0.051268	-2.54117	0.011048	NA
NRL	8.516262	-0.13026	0.084276	-1.54563	0.122193	NA
PRKCD	28.27249	-0.13025	0.083306	-1.56354	0.117925	NA

SCAMP1	395.8267	0.130224	0.057166	2.277987	0.022727	0.23963
RPS11	475.1151	-0.13022	0.073254	-1.77762	0.075465	0.391143
CHST2	67.27401	-0.13014	0.082701	-1.57368	0.115561	NA
LOC10063	4.229472	-0.1301	0.079196	-1.64277	0.100431	NA
CPM	27.55117	-0.13009	0.084544	-1.53872	0.123873	NA
TPT1-AS1	42.73459	-0.13008	0.066123	-1.96723	0.049157	NA
AGAP5	15.31968	0.13006	0.081383	1.598127	0.110015	NA
CC2D2B	11.76561	-0.13004	0.083482	-1.55776	0.11929	NA
ABCG2	46.99401	0.130044	0.084934	1.531117	0.125741	NA
NCKIPSD	151.6619	-0.13004	0.050602	-2.56992	0.010172	NA
RASA4CP	6.382326	-0.13003	0.084469	-1.53942	0.123702	NA
SLC7A9	8.420389	0.13001	0.078596	1.654164	0.098094	NA
BACE1-AS	8.076242	-0.12999	0.084855	-1.53185	0.125559	NA
NBR2	29.62121	-0.12994	0.07479	-1.73736	0.082324	NA
YWHAQ	479.4921	0.129921	0.070329	1.847327	0.0647	0.375222
SNORD38B	7.321469	-0.12991	0.079458	-1.63494	0.102061	NA
ZBTB6	79.23046	0.129894	0.061341	2.117557	0.034213	NA
HNRNPU-A	488.6219	-0.12988	0.06154	-2.11056	0.03481	0.288416
RFX4	64.09734	-0.12984	0.08347	-1.55557	0.119811	NA
CCDC28A	63.46912	-0.12983	0.069361	-1.87183	0.061231	NA
LRRC17	4.852179	-0.12983	0.081045	-1.60194	0.10917	NA
TSPYL2	2269.608	-0.12982	0.056555	-2.29538	0.021711	0.23963
CDKN2AIP	78.56318	0.129751	0.05655	2.294464	0.021764	NA
SLC26A3	8.241447	0.129694	0.078535	1.651426	0.098652	NA
TEKT3	6.243718	-0.12969	0.078716	-1.64758	0.09944	NA
ARSD	25.06448	-0.12967	0.083654	-1.55005	0.12113	NA
ASB16	41.65376	-0.12966	0.069813	-1.85728	0.063271	NA
MTPAP	268.9957	0.129661	0.059769	2.169363	0.030055	NA
GPR63	36.32545	-0.12964	0.084805	-1.5287	0.126339	NA
BMP1	86.42616	0.12964	0.068818	1.883813	0.05959	NA
SLC12A3	4.97665	0.129609	0.080576	1.608536	0.107718	NA
ANXA4	10.69145	-0.12957	0.084559	-1.5323	0.125449	NA
LACC1	21.74604	-0.12948	0.08409	-1.53972	0.12363	NA
ZNF180	51.8693	0.129466	0.062212	2.081037	0.037431	NA
HSF2BP	3.713739	-0.12946	0.075492	-1.71493	0.086357	NA
LOC20278	41.66379	-0.12946	0.070389	-1.83922	0.065883	NA
IDI2-AS1	25.97966	-0.12945	0.077462	-1.67114	0.094695	NA
IL20RA	19.94543	-0.12941	0.084883	-1.5246	0.12736	NA
CROCC	698.9226	-0.12941	0.079736	-1.62293	0.104605	0.447883
ANGPT1	43.27297	-0.1294	0.082632	-1.56598	0.117354	NA
ZNF257	25.72676	0.129349	0.076479	1.691294	0.090781	NA
ZMAT5	11.99385	-0.12932	0.083954	-1.54037	0.12347	NA
OMD	26.03657	-0.12931	0.0832	-1.5542	0.120137	NA
RAB28	87.70372	0.129293	0.054885	2.355693	0.018488	NA
LOC10050	8.758927	-0.12928	0.084774	-1.52501	0.127257	NA
PELO	40.46547	0.129276	0.065519	1.973105	0.048484	NA
LPL	143.3631	-0.12925	0.084749	-1.52507	0.127242	NA

GBA	26.26831	-0.12921	0.076916	-1.67993	0.092971	NA
TMEM88B	1.541836	-0.1292	0.061214	-2.11069	0.034799	NA
CLMP	86.21903	-0.12919	0.082962	-1.55724	0.119413	NA
FAM186B	13.03501	-0.12919	0.083708	-1.54336	0.122742	NA
DCDC2	1.816513	-0.12916	0.067502	-1.91344	0.055692	NA
NFATC2IP	178.1311	-0.12915	0.051201	-2.52237	0.011657	NA
EOMES	48.92828	0.129078	0.083898	1.538515	0.123923	NA
PPP4C	47.90331	-0.12906	0.070666	-1.82629	0.067807	NA
MR1	11.72254	-0.12905	0.084556	-1.52618	0.126966	NA
ARL9	6.240874	-0.12903	0.084207	-1.53236	0.125435	NA
ERI3	75.74614	-0.12898	0.05149	-2.50491	0.012248	NA
CHTF18	73.1821	-0.12895	0.070895	-1.81885	0.068934	NA
TMEM229	120.8909	0.128927	0.058882	2.18959	0.028554	NA
POPDC2	7.807403	-0.12881	0.084917	-1.51684	0.129308	NA
C12orf4	93.01097	0.128754	0.059323	2.170376	0.029978	NA
ZNF333	161.2013	-0.12869	0.060579	-2.12439	0.033638	NA
ENKUR	13.40623	-0.12863	0.084877	-1.5155	0.129646	NA
MAP7	1472.859	-0.1286	0.064209	-2.00286	0.045193	0.333419
MRPL19	288.9235	0.128601	0.042947	2.994394	0.00275	NA
SMTN	213.6788	-0.12858	0.084923	-1.51411	0.129997	NA
OR6A2	0.815231	-0.12858	0.048193	-2.66807	0.007629	NA
ZNF148	610.9009	0.128544	0.037403	3.436762	0.000589	0.075062
CMTM6	60.61229	-0.12853	0.072155	-1.78131	0.074862	NA
SLC29A3	13.76876	-0.12852	0.082774	-1.55262	0.120513	NA
ST6GAL1	34.80387	-0.12848	0.081612	-1.57423	0.115433	NA
STARD4-A	297.2799	0.12847	0.064993	1.976676	0.048078	NA
SERHL2	12.1114	-0.12845	0.084482	-1.52042	0.128405	NA
CASP10	14.63895	0.128422	0.084367	1.522179	0.127964	NA
LOC64366	6.225665	-0.12838	0.082929	-1.54811	0.121596	NA
NLRX1	17.52939	-0.12837	0.081821	-1.56889	0.116674	NA
ZDHHC2	377.4813	0.128351	0.042995	2.98525	0.002833	0.130976
THSD1	16.35714	-0.12832	0.084928	-1.51094	0.130803	NA
PIK3AP1	10.34067	-0.1283	0.081757	-1.56932	0.116573	NA
C16orf11	132.073	0.128302	0.076454	1.678158	0.093316	NA
BAG5	275.3236	0.12819	0.045125	2.840743	0.004501	NA
NIT1	51.05431	-0.12818	0.060903	-2.10462	0.035325	NA
FAM171A2	66.26162	0.128165	0.063601	2.015139	0.04389	NA
DCAF4	23.0751	0.12814	0.079703	1.607722	0.107896	NA
DPH1	98.25404	-0.12812	0.058067	-2.20644	0.027353	NA
OCLN	25.42888	0.128117	0.081146	1.578854	0.11437	NA
F8	25.23492	-0.12811	0.081438	-1.57306	0.115705	NA
FAM229A	13.8243	-0.12807	0.084928	-1.50799	0.131557	NA
FLJ42709	145.881	-0.12805	0.053965	-2.37281	0.017654	NA
CNN2	7.416823	-0.12804	0.084548	-1.51442	0.129919	NA
FBXW10	9.711018	0.128036	0.084803	1.50981	0.131092	NA
ST3GAL2	153.0453	0.128032	0.049714	2.575351	0.010014	NA
SNORD115	33.90087	-0.12803	0.083716	-1.52929	0.126193	NA

CDK5R2	37.36321	0.127975	0.07841	1.632119	0.102654	NA
REM2	6.34871	-0.12797	0.083198	-1.53812	0.124018	NA
RAD21L1	2.997613	-0.12796	0.065307	-1.9594	0.050067	NA
CALD1	696.9177	-0.12794	0.050908	-2.51311	0.011967	0.202283
SLC22A13	15.90237	0.12792	0.084919	1.506378	0.13197	NA
ALDOC	1111.455	-0.12786	0.083516	-1.53102	0.125764	0.478748
ATOX1	13.61399	-0.12784	0.083159	-1.53735	0.124208	NA
ZBTB16	445.8507	-0.12783	0.084038	-1.52115	0.128223	0.48338
GPCPD1	277.0069	0.127833	0.071775	1.781038	0.074906	NA
EPHA4	347.997	0.127722	0.05569	2.29346	0.021822	0.23963
HTRA2	18.92572	0.127705	0.079651	1.603312	0.108866	NA
SYT9	227.0321	0.127705	0.061268	2.084361	0.037127	NA
HBP1	212.1583	-0.12769	0.055582	-2.29726	0.021604	NA
MGST3	72.561	-0.12768	0.063572	-2.00843	0.044598	NA
HMGN3	145.4687	-0.12766	0.054973	-2.32229	0.020218	NA
NPTXR	345.6111	0.127609	0.060712	2.101869	0.035565	0.289763
RPL26L1	9.780877	-0.12753	0.084908	-1.50202	0.133093	NA
SGCA	12.13218	-0.12749	0.081894	-1.55673	0.119534	NA
TSNARE1	38.09105	-0.12748	0.072983	-1.74676	0.080679	NA
AKR1C6P	2.043351	-0.12747	0.068428	-1.86285	0.062484	NA
FAM228A	16.01432	-0.12745	0.084824	-1.50254	0.132957	NA
ZFP36	57.60563	-0.12739	0.081274	-1.56745	0.11701	NA
NOL6	82.37299	-0.12737	0.068498	-1.85951	0.062955	NA
BTF3L4	102.0595	-0.12736	0.05793	-2.19848	0.027915	NA
NLRP3	4.107066	-0.12736	0.080917	-1.5739	0.115511	NA
NR1D2	672.1388	0.127353	0.048178	2.643387	0.008208	0.173751
FBLN2	7.86137	-0.12734	0.07932	-1.60538	0.108411	NA
OGFOD3	133.6387	-0.12733	0.066713	-1.90868	0.056303	NA
CREB1	464.2112	0.127313	0.040619	3.13431	0.001723	0.101784
FBXL19	89.82046	0.12725	0.055051	2.311479	0.020806	NA
SNORD15B	163.0509	-0.12724	0.083583	-1.52234	0.127923	NA
OARD1	31.01737	-0.12721	0.08092	-1.5721	0.115927	NA
TMEM159	47.88601	-0.1272	0.078488	-1.62061	0.105101	NA
CLYBL	171.5156	-0.1272	0.062508	-2.03492	0.041858	NA
SSR3	160.2694	-0.1272	0.050986	-2.49476	0.012604	NA
TP53INP1	90.97048	-0.12717	0.06688	-1.90147	0.057241	NA
PIP4K2A	172.0678	-0.12716	0.076802	-1.65561	0.0978	NA
NEBL-AS1	2.406317	-0.12715	0.074872	-1.69826	0.089459	NA
RNF138P1	81.05454	-0.12714	0.063609	-1.99872	0.045639	NA
NARF	178.4514	-0.1271	0.057687	-2.2033	0.027573	NA
CCDC103	4.641924	-0.1271	0.073548	-1.72807	0.083976	NA
TMEM61	8.207094	-0.12709	0.084927	-1.49645	0.134535	NA
ZIM2	18.74328	-0.12704	0.084795	-1.49816	0.134093	NA
CRK	166.3994	0.127033	0.049025	2.591195	0.009564	NA
PCP2	39.79307	-0.12695	0.082557	-1.53772	0.124117	NA
ARC	5.67695	-0.12685	0.080972	-1.5666	0.117209	NA
RPSA	119.9924	-0.12685	0.078327	-1.61949	0.105343	NA

NUDT12	38.31346	-0.12683	0.080429	-1.5769	0.11482	NA
PHB2	135.9928	-0.12681	0.063816	-1.98716	0.046905	NA
FAAH2	40.83819	-0.12678	0.076818	-1.65039	0.098863	NA
UBE2C	2.661455	0.126776	0.07019	1.806185	0.07089	NA
MIR181A2	3.565678	-0.12675	0.07834	-1.618	0.105663	NA
EFHD1	42.05072	-0.12675	0.084484	-1.50022	0.133557	NA
SNHG11	6.497595	0.126743	0.084423	1.501279	0.133283	NA
ITGA8	38.29861	0.126723	0.083556	1.516631	0.12936	NA
PSPH	37.02297	0.126715	0.078691	1.610277	0.107337	NA
PGAP3	16.30115	-0.12669	0.079668	-1.59024	0.11178	NA
QARS	80.24299	-0.12668	0.057424	-2.20612	0.027375	NA
ECE2	196.8894	-0.12668	0.052122	-2.43035	0.015084	NA
MRPL35	107.847	0.126648	0.05604	2.259969	0.023823	NA
GADD45G	10.92449	-0.12664	0.084914	-1.49138	0.135862	NA
KRAS	310.6199	0.1266	0.053045	2.386677	0.017001	NA
NAPB	1092.784	0.126591	0.067824	1.866472	0.061975	0.37053
RPL29	110.5143	-0.12659	0.073942	-1.71199	0.086899	NA
TAS2R19	26.43188	-0.12657	0.079923	-1.58367	0.11327	NA
PPM1J	14.51124	-0.1265	0.0848	-1.4918	0.135752	NA
BTK	3.661366	-0.12647	0.071598	-1.76634	0.07734	NA
DUSP11	97.81791	-0.12646	0.0538	-2.35062	0.018742	NA
LEMD1-AS	5.182206	-0.12645	0.077187	-1.6382	0.101379	NA
ENTPD8	6.880056	-0.12644	0.081182	-1.55746	0.119362	NA
LDOC1L	152.7449	0.126435	0.054777	2.308194	0.020988	NA
PPCDC	18.89523	-0.1264	0.080269	-1.5747	0.115325	NA
INA	569.7317	0.126345	0.074982	1.685002	0.091988	0.419222
MIR635	5.0121	0.126268	0.082078	1.538394	0.123952	NA
CORO1A	38.78995	-0.12626	0.069567	-1.81493	0.069534	NA
FAAH	101.2049	-0.12625	0.066341	-1.90312	0.057024	NA
STIM2	137.9647	0.126236	0.056815	2.221863	0.026293	NA
NMNAT2	1151.673	0.126219	0.054127	2.331924	0.019705	0.234966
C1orf198	98.36646	-0.12618	0.069773	-1.80838	0.070547	NA
BMP4	26.62235	0.126139	0.084622	1.49061	0.136064	NA
COL4A4	29.11328	-0.12613	0.075932	-1.66113	0.096687	NA
LEMD1	7.389225	-0.12613	0.083064	-1.51848	0.128895	NA
FOLH1	10.24353	-0.12613	0.079729	-1.58199	0.113653	NA
VWC2	203.3399	0.126125	0.051564	2.445984	0.014446	NA
POP4	39.54148	0.126118	0.07261	1.736925	0.0824	NA
C8orf44	33.82586	-0.12611	0.074042	-1.70323	0.088526	NA
RAB39B	116.3928	0.12611	0.061568	2.048301	0.040531	NA
AKR1C3	7.697173	-0.1261	0.084148	-1.4985	0.134003	NA
HEXDC	90.74471	-0.12609	0.066683	-1.89092	0.058635	NA
BCDIN3D	12.27485	-0.12607	0.083681	-1.50658	0.131917	NA
FBXO28	151.0595	0.126064	0.050562	2.493241	0.012658	NA
CLEC2L	26.50897	-0.12603	0.084486	-1.49172	0.135772	NA
IFT20	76.82608	-0.12603	0.063938	-1.97107	0.048716	NA
UBQLN1	424.1992	0.126011	0.051527	2.445522	0.014464	0.212327

LOC10050	6.734936	-0.12597	0.083064	-1.51655	0.12938	NA
ZNF556	38.53699	0.125936	0.075273	1.673055	0.094316	NA
CDC26	29.22009	0.125894	0.073435	1.714345	0.086465	NA
CHST5	14.25112	0.125893	0.082986	1.517025	0.12926	NA
TUBB6	37.96338	-0.12586	0.080946	-1.55488	0.119974	NA
GJC1	171.8784	0.125832	0.061353	2.050937	0.040273	NA
C6orf170	54.33435	-0.12583	0.079317	-1.58643	0.112641	NA
SNORD114	3.328941	-0.1258	0.07716	-1.63034	0.10303	NA
APLF	12.00024	-0.12577	0.08454	-1.48773	0.136823	NA
PALMD	20.12876	0.125731	0.084855	1.481716	0.138416	NA
CSF1	43.75149	-0.12571	0.077386	-1.62444	0.104283	NA
MIR663A	165.714	-0.12567	0.081555	-1.54089	0.123345	NA
MMP16	195.6068	0.125649	0.063398	1.981907	0.04749	NA
FOSL1	4.637618	-0.12565	0.080122	-1.56821	0.116831	NA
BTBD19	4.21184	0.125647	0.079773	1.575057	0.115243	NA
CXCL12	9.033925	0.125566	0.07792	1.61147	0.107077	NA
TAGLN	48.48293	-0.12556	0.08464	-1.48347	0.137949	NA
CELF6	73.14824	-0.12556	0.064037	-1.96072	0.049912	NA
CDKN1A	19.87177	-0.12551	0.079366	-1.58135	0.113799	NA
FBXO10	46.10563	-0.12549	0.070682	-1.77542	0.075829	NA
RWDD2B	33.75111	-0.12547	0.080052	-1.56734	0.117035	NA
LRRC1	27.6343	-0.12547	0.078276	-1.6029	0.108957	NA
LAMTOR3	124.0282	0.125423	0.063415	1.977805	0.047951	NA
METTL15	54.06562	0.125411	0.065153	1.924878	0.054245	NA
TNFRSF1A	24.84863	-0.12539	0.083829	-1.49573	0.134724	NA
L3HYPDH	54.29794	0.12538	0.064462	1.945035	0.051771	NA
CCDC146	58.56962	-0.12536	0.076493	-1.63889	0.101236	NA
IGFBPL1	15.78574	0.125339	0.079087	1.584826	0.113006	NA
MIR3936	5.701691	-0.12534	0.083456	-1.50181	0.133146	NA
LOC10050	2.192044	-0.1253	0.067554	-1.85483	0.063621	NA
NTSR2	10.98504	-0.12528	0.08424	-1.48723	0.136954	NA
ZNF704	1232.866	0.125272	0.060663	2.065033	0.03892	0.300865
TANK	93.91514	0.125229	0.060968	2.054034	0.039972	NA
HMOX1	4.39863	-0.12519	0.068248	-1.83441	0.066593	NA
LOC10027	1.960474	-0.12517	0.06884	-1.81829	0.06902	NA
UBL5	41.35424	-0.12514	0.082208	-1.52224	0.12795	NA
PCDHA1	17.10641	0.125103	0.081438	1.536179	0.124494	NA
IL21R-AS1	9.780922	-0.12509	0.084035	-1.48854	0.136609	NA
TRIM55	22.82491	0.125087	0.084443	1.481326	0.13852	NA
PCOLCE	21.88	-0.12502	0.079768	-1.56731	0.117043	NA
AFTPH	351.6055	0.125007	0.044579	2.804169	0.005045	0.151649
TTLL11	169.4014	0.124983	0.057051	2.190706	0.028473	NA
DCPS	25.61523	0.12497	0.073252	1.706027	0.088003	NA
VAMP8	2.954332	-0.12493	0.071306	-1.752	0.079774	NA
GJB4	2.96563	-0.12491	0.074941	-1.66677	0.095561	NA
ACAN	1.863021	-0.1249	0.065697	-1.90114	0.057284	NA
VWF	218.0608	-0.12484	0.08419	-1.48282	0.138121	NA

UBE2G1	92.92384	0.124826	0.057756	2.161256	0.030676	NA
WAC	1667.732	0.124798	0.047069	2.651361	0.008017	0.171303
ZNF76	129.3518	-0.12474	0.047456	-2.6286	0.008574	NA
PDLIM4	8.98335	-0.12471	0.082148	-1.51806	0.129	NA
COL11A1	29.23913	-0.12469	0.080395	-1.55098	0.120907	NA
SNX7	9.594427	-0.12465	0.083999	-1.48392	0.137829	NA
NCK2	196.4683	0.124631	0.054734	2.277009	0.022786	NA
PKP4	795.8632	0.124584	0.051894	2.400732	0.016362	0.223611
MAL	13.50062	-0.12458	0.084289	-1.47799	0.139411	NA
IRF2	101.8046	-0.12457	0.046464	-2.68104	0.007339	NA
EGLN3	25.89553	-0.12457	0.08365	-1.4892	0.136435	NA
PRKD3	152.3685	-0.12455	0.051384	-2.42392	0.015354	NA
KLC2	497.0602	0.124547	0.051479	2.419368	0.015547	0.218727
SFT2D2	5.704838	-0.12451	0.080856	-1.53993	0.123578	NA
PRH2	56.96512	-0.1245	0.080711	-1.54258	0.122934	NA
BCL11A	10.23976	-0.1245	0.08409	-1.48056	0.138724	NA
CEP72	29.11037	-0.12449	0.080154	-1.55319	0.120379	NA
LOC25412	48.54454	-0.12449	0.061413	-2.02712	0.04265	NA
GEM	3.605438	-0.12447	0.073319	-1.69768	0.089568	NA
SNCG	49.32602	-0.12445	0.082948	-1.50035	0.133523	NA
PDE6A	13.57506	0.124435	0.084939	1.464993	0.142923	NA
C17orf59	18.77552	0.124368	0.080853	1.538195	0.124001	NA
PKLR	7.025594	0.124304	0.083971	1.480313	0.13879	NA
SCN2B	362.2664	0.124296	0.071815	1.730769	0.083493	0.403119
C1orf226	40.22072	-0.12422	0.080716	-1.53898	0.12381	NA
SERPINI2	16.98496	-0.12421	0.084883	-1.46329	0.143389	NA
MSX2	18.70181	-0.1242	0.084544	-1.46903	0.141825	NA
SORCS2	49.12663	-0.12418	0.078503	-1.58183	0.113689	NA
SLC22A7	19.21713	0.124161	0.08493	1.461918	0.143764	NA
EPHX2	65.86999	-0.12414	0.068651	-1.80821	0.070574	NA
SLC37A2	9.824359	-0.12413	0.084428	-1.4703	0.141481	NA
DHRS13	55.97597	0.124132	0.06926	1.792264	0.073091	NA
CSDA	38.2374	-0.12405	0.078507	-1.58014	0.114074	NA
LOC10027	2.955768	-0.12403	0.074737	-1.65961	0.096994	NA
C8orf59	14.32059	-0.12402	0.084415	-1.46923	0.14177	NA
MAP2K4	306.1307	0.124022	0.053438	2.320832	0.020296	NA
ABCA9	98.76422	-0.12401	0.072701	-1.70571	0.088062	NA
RHOQ	68.27053	-0.124	0.067716	-1.83125	0.067063	NA
C2CD2	47.40026	-0.12399	0.082917	-1.49538	0.134815	NA
CXCL17	9.654627	-0.12396	0.084935	-1.45949	0.14443	NA
PSENEN	10.1144	-0.12389	0.084533	-1.46559	0.142761	NA
ANGPTL2	156.2061	0.123863	0.057977	2.136423	0.032645	NA
RAB11FIP2	239.3996	0.123847	0.052254	2.370073	0.017785	NA
NDUFAF1	40.54588	-0.12381	0.068171	-1.8162	0.06934	NA
APRT	17.91628	-0.12381	0.080642	-1.53531	0.124708	NA
ADHFE1	31.78802	-0.12377	0.084556	-1.46382	0.143244	NA
NECAP2	138.3926	-0.12377	0.054776	-2.25954	0.02385	NA

HEY2	26.67159	-0.12376	0.075381	-1.64186	0.100619	NA
EPHA8	5.256115	-0.12376	0.075629	-1.63643	0.101751	NA
C2CD4A	1.533017	-0.12376	0.057689	-2.14525	0.031933	NA
EVPL	13.4946	0.123735	0.084721	1.460499	0.144153	NA
NCOA6	589.7335	0.12372	0.03634	3.404523	0.000663	0.075062
C1orf51	34.59485	-0.1237	0.082663	-1.49647	0.134531	NA
MEIS1-AS3	13.20321	-0.12366	0.084627	-1.4613	0.143933	NA
ERCC1	61.34548	-0.12364	0.057394	-2.1543	0.031217	NA
ZNF121	107.7892	0.123598	0.047013	2.62904	0.008563	NA
FIS1	79.30891	-0.1236	0.073469	-1.6823	0.09251	NA
EXTL3	186.585	0.123521	0.051444	2.401093	0.016346	NA
SNORD59B	18.91473	-0.12352	0.083236	-1.48398	0.137813	NA
AGBL2	10.57885	0.12351	0.084135	1.467991	0.142107	NA
RAB23	121.5971	0.123463	0.058489	2.110869	0.034784	NA
TMEM129	56.43383	-0.12344	0.061834	-1.99631	0.045901	NA
FSTL3	22.39014	-0.12336	0.079625	-1.54922	0.12133	NA
OIP5-AS1	836.7025	0.123316	0.056656	2.176577	0.029512	0.274979
PI16	4.597272	-0.12331	0.078965	-1.56154	0.118395	NA
FTSJ2	51.36016	0.123228	0.068828	1.790365	0.073395	NA
SIGLEC10	4.396892	-0.12323	0.067112	-1.83613	0.066339	NA
C21orf58	19.65322	-0.12322	0.079405	-1.55182	0.120705	NA
ARFGAP3	116.288	0.12321	0.054891	2.244622	0.024792	NA
CAPN2	360.2258	-0.12319	0.051307	-2.40107	0.016347	0.223611
ANKRD17	1292.872	0.123172	0.028514	4.319776	1.56E-05	0.035377
NSUN7	3.810498	-0.12317	0.076564	-1.60868	0.107686	NA
CCDC115	37.18585	0.123138	0.078539	1.567866	0.116912	NA
NFATC1	18.24516	-0.1231	0.081488	-1.51064	0.13088	NA
YPEL1	130.1413	-0.12308	0.071972	-1.71017	0.087235	NA
TAF13	27.07385	0.12307	0.073868	1.666078	0.095698	NA
DNAH12	4.84436	-0.12303	0.080249	-1.53315	0.125239	NA
NELFE	61.45885	-0.12303	0.057581	-2.13669	0.032623	NA
HMGCL	14.43528	-0.12303	0.084544	-1.45516	0.145624	NA
GALNT6	6.096067	-0.12301	0.082075	-1.49874	0.13394	NA
SNRK	300.9118	0.123006	0.052395	2.34768	0.018891	NA
SLC16A12	2.087983	-0.12299	0.065234	-1.88542	0.059374	NA
GPLD1	284.2389	-0.12299	0.04534	-2.71261	0.006676	NA
IER3IP1	81.06886	0.122987	0.065742	1.870756	0.061379	NA
SOCS4	160.3807	0.12294	0.047257	2.601523	0.009281	NA
R3HDM4	30.54429	0.122938	0.074124	1.658542	0.097208	NA
TMEM63A	89.51551	-0.12293	0.082146	-1.49655	0.134511	NA
KLHL28	141.3536	0.122922	0.051528	2.385548	0.017054	NA
LRTM1	5.639542	-0.1229	0.083065	-1.47958	0.138984	NA
PTPN13	149.9185	-0.12277	0.073917	-1.66097	0.096719	NA
PPP1R14C	16.8625	0.122761	0.084897	1.445991	0.14818	NA
LTB	2.261093	-0.12276	0.069253	-1.77263	0.076291	NA
ENPP6	9.606962	0.122757	0.079962	1.53519	0.124737	NA
STK32C	30.50953	0.122747	0.078079	1.57208	0.115932	NA

ZNF625	29.24707	0.122725	0.071085	1.726446	0.084267	NA
RPL23AP3	38.09902	-0.12272	0.073924	-1.66011	0.096893	NA
SNORA30	3.641443	0.122721	0.07699	1.593995	0.110937	NA
SLC7A14	505.3722	0.122706	0.062165	1.973877	0.048396	0.342551
ZNF14	94.54214	0.122686	0.057104	2.148448	0.031678	NA
GALNT15	76.50267	-0.12267	0.080267	-1.52826	0.126448	NA
TAF5L	94.04346	0.122659	0.051198	2.395779	0.016585	NA
PLD4	18.50554	-0.12265	0.084362	-1.4538	0.146002	NA
ZNF614	120.5164	0.122643	0.052901	2.318326	0.020432	NA
UGCG	45.9022	0.122636	0.064297	1.907328	0.056478	NA
GLI4	25.61189	-0.12263	0.081896	-1.49737	0.134296	NA
NPHS1	2.737525	-0.12263	0.073594	-1.66628	0.095657	NA
PRDM16	9.511128	-0.12262	0.08488	-1.44463	0.148562	NA
SCARNA2	68.04407	-0.12257	0.084876	-1.44407	0.148718	NA
MAP7D3	12.8593	-0.12252	0.084351	-1.45253	0.146354	NA
HAPLN2	53.72608	-0.12251	0.079913	-1.5331	0.125251	NA
MYC	10.2216	0.122479	0.084623	1.447357	0.147797	NA
KIF11	6.693078	-0.12244	0.084625	-1.44682	0.147948	NA
UBE2E2	39.32314	0.122433	0.066071	1.853052	0.063875	NA
SNORD8	326.8453	-0.12243	0.080704	-1.51704	0.129258	NA
LOC64285	209.0137	0.122412	0.064833	1.888108	0.059011	NA
LOC10013	3.145826	-0.1224	0.072234	-1.69456	0.090159	NA
XRCC3	48.1366	-0.1224	0.072054	-1.69877	0.089362	NA
SLC39A4	6.9521	0.12237	0.072692	1.683408	0.092296	NA
POLD4	35.18951	-0.12237	0.07455	-1.64143	0.100709	NA
RUNX1	10.21255	-0.12232	0.082032	-1.4911	0.135935	NA
SNORA45	11.81706	-0.12231	0.073264	-1.66941	0.095036	NA
PPP1R3B	25.08507	-0.1223	0.083434	-1.4658	0.142703	NA
KCNJ6	130.666	0.122286	0.061208	1.997863	0.045732	NA
SYT12	211.9488	0.122269	0.069586	1.757099	0.078901	NA
C16orf13	33.63566	-0.12226	0.068121	-1.79472	0.072699	NA
VPS37D	12.92011	0.122201	0.083874	1.456951	0.14513	NA
CSNK1A1L	1.065771	-0.12218	0.054126	-2.25736	0.023985	NA
SPATC1	13.22423	0.122061	0.082378	1.481726	0.138413	NA
MSR1	19.04384	-0.12202	0.084871	-1.43771	0.150516	NA
ZNF74	39.7536	0.122001	0.068506	1.780871	0.074934	NA
LOC11511	0.936884	-0.122	0.050869	-2.39827	0.016473	NA
MROH9	1.389405	-0.12199	0.062292	-1.95839	0.050184	NA
SNORD116	7.770245	-0.12194	0.084491	-1.44322	0.14896	NA
EFNA3	10.31969	0.121938	0.0842	1.448199	0.147561	NA
TSPY26P	13.87308	0.121925	0.082466	1.478495	0.139275	NA
SET	482.8691	0.121908	0.05665	2.151942	0.031402	0.277495
TLR5	2.758182	-0.12189	0.070299	-1.73391	0.082935	NA
B3GALT6	28.80863	0.121884	0.075926	1.60531	0.108426	NA
ZNF300P1	62.22039	0.12188	0.08487	1.436083	0.150979	NA
ANXA2P3	3.1235	0.121874	0.06761	1.802615	0.071449	NA
MCEE	13.37793	-0.12187	0.083229	-1.46427	0.14312	NA

FAM86A	8.884895	-0.12186	0.08493	-1.43482	0.151337	NA
KIAA1984-	80.19056	-0.12184	0.072901	-1.67133	0.094656	NA
PSMB10	30.58799	-0.12181	0.069735	-1.74677	0.080678	NA
UGGT1	346.119	0.121801	0.035108	3.469368	0.000522	0.075062
ATG4A	35.17865	0.121784	0.070514	1.727098	0.08415	NA
ABCA6	93.61124	-0.12177	0.076425	-1.59334	0.111084	NA
DEFB1	1.545104	-0.12176	0.056828	-2.14262	0.032144	NA
RIT2	72.51917	0.121725	0.066968	1.817664	0.069115	NA
RUNDC1	117.571	0.121722	0.070842	1.718225	0.085756	NA
KIAA0087	30.37673	-0.12172	0.084939	-1.43305	0.151843	NA
PCDHAC2	73.2552	0.121658	0.063236	1.92387	0.054371	NA
PSIMCT-1	20.80944	-0.12163	0.079815	-1.52395	0.127522	NA
ABCA8	57.62098	-0.12163	0.08414	-1.44557	0.148297	NA
RHOU	52.29704	-0.12158	0.07609	-1.5979	0.110065	NA
MMP19	6.767846	-0.12158	0.082241	-1.47828	0.139334	NA
GPR133	8.95197	-0.12157	0.084222	-1.44344	0.148895	NA
COLQ	47.57434	-0.12157	0.084311	-1.44189	0.149333	NA
TDRD9	21.20414	0.121523	0.073249	1.659045	0.097107	NA
TTC12	29.80099	-0.12152	0.084732	-1.43413	0.151535	NA
ACSL4	195.5212	0.121496	0.065366	1.858698	0.06307	NA
DALRD3	19.75139	0.12149	0.077024	1.577301	0.114726	NA
ART3	69.06021	-0.12146	0.082888	-1.46533	0.14283	NA
TBC1D7	26.61931	-0.12143	0.082006	-1.48079	0.138662	NA
LINC00339	9.355773	-0.12139	0.084568	-1.43542	0.151167	NA
ARNTL2	58.07065	0.121372	0.077579	1.564493	0.117702	NA
ZNF583	82.90513	0.121366	0.053815	2.255231	0.024119	NA
CDHR5	30.40648	-0.12135	0.084239	-1.44054	0.149715	NA
ZNF239	25.53576	0.121326	0.077928	1.556898	0.119495	NA
ZNF880	257.6561	0.121275	0.050337	2.409239	0.015986	NA
GSS	44.23742	-0.12126	0.069934	-1.73397	0.082923	NA
PRTG	23.92556	-0.12126	0.08153	-1.48729	0.136938	NA
MAU2	259.6933	-0.12123	0.048743	-2.48721	0.012875	NA
MT1M	9.880573	-0.12123	0.077344	-1.56742	0.117017	NA
HHIP	39.71722	-0.12123	0.078315	-1.54795	0.121633	NA
TXN	51.01731	-0.12122	0.076168	-1.59145	0.111508	NA
C21orf119	3.499927	-0.12114	0.07936	-1.52645	0.126897	NA
MRPL11	29.86472	-0.12112	0.075892	-1.5959	0.11051	NA
SPCS1	43.45504	-0.12109	0.077843	-1.55562	0.119799	NA
ALDH2	188.2433	-0.12109	0.067759	-1.7871	0.073922	NA
TSHR	16.91908	0.121087	0.084661	1.430256	0.152644	NA
GVINP1	10.57173	-0.12105	0.083273	-1.45365	0.146043	NA
MEG9	30.40671	-0.12095	0.080913	-1.49485	0.134954	NA
DUSP16	289.0359	0.120949	0.042399	2.852633	0.004336	NA
PACRGL	46.43573	-0.12092	0.064076	-1.88713	0.059142	NA
IFT27	30.87164	-0.12091	0.074057	-1.63271	0.10253	NA
SUPT4H1	47.66662	-0.12091	0.072133	-1.67622	0.093695	NA
ICOSLG	14.53487	-0.12091	0.084823	-1.42541	0.154039	NA

ZNF296	13.05684	0.120904	0.08333	1.450917	0.146803	NA
MIR655	1.150029	-0.12089	0.05564	-2.17275	0.029799	NA
C22orf43	23.37595	-0.12088	0.084841	-1.42474	0.154231	NA
STMN4	256.2924	0.120771	0.068847	1.754211	0.079394	NA
GPRIN2	51.31895	0.120763	0.082073	1.471407	0.141181	NA
ARL5A	212.0442	0.120757	0.049668	2.431278	0.015046	NA
FZD8	1.876342	-0.12067	0.066398	-1.81738	0.069158	NA
GABRG1	207.9234	-0.12057	0.082251	-1.46582	0.142698	NA
BAI2	140.6451	0.120563	0.073632	1.637363	0.101555	NA
SPTY2D1	111.2947	0.120558	0.049398	2.440546	0.014665	NA
LOC10029	84.84804	0.120536	0.064718	1.862475	0.062536	NA
ANAPC11	34.21517	-0.12053	0.078794	-1.52968	0.126096	NA
SLC25A45	3.03197	-0.12051	0.073819	-1.63249	0.102575	NA
MKS1	40.70308	-0.12049	0.071953	-1.67462	0.094008	NA
IL33	28.8079	-0.12044	0.084819	-1.41994	0.155626	NA
LOC10050	32.62691	-0.12042	0.083732	-1.4381	0.150406	NA
ZNF430	109.7267	0.120398	0.052161	2.308208	0.020988	NA
SHANK2	348.8199	-0.12038	0.077246	-1.55845	0.119127	0.472798
SYTL2	98.52983	-0.12037	0.0752	-1.60067	0.10945	NA
FRMD4B	36.86083	-0.12033	0.08389	-1.43434	0.151475	NA
ARMC12	1.414259	-0.12027	0.054399	-2.21094	0.02704	NA
MIR4312	4.2896	-0.12026	0.080653	-1.49104	0.135952	NA
MLPH	1.461509	-0.12025	0.063238	-1.90151	0.057235	NA
PAR1	190.6512	-0.12025	0.071184	-1.68924	0.091173	NA
NOD2	2.319515	-0.12024	0.068393	-1.75805	0.078739	NA
NUCB2	205.8825	0.120197	0.051546	2.331853	0.019708	NA
ZNF75A	79.32619	0.120177	0.060369	1.990712	0.046513	NA
PRR11	4.434553	-0.12017	0.080553	-1.49187	0.135734	NA
DCAF8	650.3343	-0.12017	0.047699	-2.51946	0.011754	0.201925
PSME1	75.3748	-0.12014	0.06815	-1.76291	0.077916	NA
KCND2	1159.267	0.120099	0.053245	2.255606	0.024095	0.246583
PRRC1	77.05348	0.120057	0.055888	2.148159	0.031701	NA
OLIG2	13.22382	-0.12005	0.084842	-1.41499	0.157073	NA
FKBP3	212.1636	-0.12004	0.057774	-2.07782	0.037726	NA
NT5DC2	123.2033	-0.12003	0.083307	-1.44084	0.14963	NA
MACROD1	32.17733	-0.12	0.08164	-1.46992	0.141584	NA
CDKN1C	7.14895	-0.11998	0.08035	-1.49322	0.135379	NA
GPR34	20.42954	-0.11997	0.081609	-1.47009	0.141537	NA
TAS2R13	19.75545	-0.11995	0.08303	-1.44465	0.148556	NA
ARMCX5	39.44534	-0.11995	0.078242	-1.53304	0.125265	NA
C21orf91	39.88471	-0.11989	0.084617	-1.41687	0.156521	NA
FAM63A	151.9526	-0.11984	0.055827	-2.14657	0.031828	NA
NECAB1	21.59445	0.119832	0.08492	1.411118	0.15821	NA
SNRNP70	1227.61	-0.11982	0.060278	-1.98786	0.046828	0.337641
TAF12	53.38241	0.119792	0.062885	1.904949	0.056787	NA
TMEM117	12.10804	-0.11979	0.084586	-1.41615	0.156731	NA
DYNC1H1	6394.84	0.11977	0.040211	2.978521	0.002896	0.131209

PROSER1	176.1112	0.119763	0.05385	2.22399	0.026149	NA
MRPL33	45.21436	-0.11975	0.07838	-1.52787	0.126544	NA
GMFG	1.489404	-0.11975	0.06071	-1.97242	0.048561	NA
ZNF287	116.8062	0.119686	0.051426	2.327326	0.019948	NA
THG1L	12.94153	-0.11963	0.082642	-1.44755	0.147744	NA
COA3	19.44694	-0.1196	0.08316	-1.43821	0.150373	NA
BTG2	101.6236	-0.11959	0.05726	-2.08851	0.036751	NA
SPATA6	33.83553	-0.11956	0.077806	-1.53662	0.124386	NA
FBXO5	20.45041	0.119558	0.078842	1.516417	0.129414	NA
SNX29P2	11.73915	-0.11943	0.084773	-1.40883	0.158886	NA
DDX1	309.5937	0.119418	0.052892	2.257772	0.02396	NA
ALDH4A1	28.99715	0.119367	0.077132	1.547571	0.121726	NA
SYNC	8.977853	-0.11934	0.084854	-1.40638	0.15961	NA
DBN1	295.5314	0.119332	0.069093	1.727121	0.084146	NA
TMEM134	16.50879	-0.11932	0.081795	-1.45881	0.144618	NA
ABHD15	6.748233	-0.11932	0.083711	-1.42536	0.154053	NA
RSPO4	1.017379	-0.11931	0.052733	-2.26256	0.023663	NA
C14orf183	8.722784	0.119271	0.078176	1.52568	0.12709	NA
C7orf76	1.364585	0.119246	0.05877	2.02905	0.042453	NA
SPTSSB	47.70255	0.119197	0.084573	1.409395	0.158718	NA
QSER1	916.4451	0.119166	0.052781	2.257759	0.023961	0.246583
CLK1	764.5958	-0.11916	0.05833	-2.04278	0.041074	0.31324
C15orf40	41.26559	-0.11913	0.07481	-1.59245	0.111284	NA
ARL2BP	81.9359	0.119126	0.062913	1.893493	0.058292	NA
LIX1L	36.27169	-0.11911	0.069184	-1.7217	0.085123	NA
KLHL42	227.4644	0.119114	0.053355	2.232466	0.025584	NA
MRPS18B	46.83263	-0.11909	0.073091	-1.62932	0.103244	NA
APPL2	261.2967	-0.11908	0.042909	-2.77523	0.005516	NA
LY6G5C	10.32345	0.119071	0.084917	1.402212	0.160852	NA
SNORD52	2.014299	-0.11907	0.067493	-1.7642	0.077699	NA
MIR4691	1.97073	-0.11904	0.069892	-1.70315	0.08854	NA
GPS1	185.7433	-0.11903	0.041382	-2.87643	0.004022	NA
SNORD49A	2.333694	-0.11902	0.069151	-1.7212	0.085214	NA
KLHL7-AS1	1.623694	-0.119	0.060739	-1.95915	0.050095	NA
RPL32	262.8722	-0.11899	0.079385	-1.49892	0.133895	NA
LOC10050	70.3078	-0.11898	0.072701	-1.63656	0.101723	NA
ACOT7	90.99493	0.118968	0.074027	1.607092	0.108034	NA
FKBP5	123.6285	-0.11897	0.080281	-1.48189	0.13837	NA
GNMT	15.89374	-0.11895	0.084921	-1.4007	0.161303	NA
FAM213A	127.2644	-0.11894	0.071587	-1.66149	0.096615	NA
LOC10012	3.255115	-0.11894	0.07567	-1.57183	0.115989	NA
SLC25A51	51.80583	-0.11887	0.061087	-1.94592	0.051665	NA
UBQLN4	100.0411	0.118864	0.058523	2.031053	0.04225	NA
BAIAP2L2	18.36745	-0.11883	0.082909	-1.43323	0.151791	NA
ACTR3	228.8833	0.118826	0.057906	2.052048	0.040165	NA
SAP30BP	147.4934	-0.11878	0.048363	-2.45602	0.014048	NA
SRA1	100.7958	0.118774	0.064853	1.831433	0.067036	NA

PHRF1	701.9736	-0.11876	0.045135	-2.63123	0.008508	0.176787
CES4A	206.6299	-0.11875	0.072044	-1.64826	0.099299	NA
TRIP10	37.53241	-0.11874	0.083216	-1.42691	0.153607	NA
GLCCI1	308.6186	0.118741	0.049114	2.417669	0.01562	NA
COPB2	425.106	0.118734	0.046525	2.552054	0.010709	0.200462
TLN2	470.8939	0.118724	0.052152	2.276475	0.022818	0.23963
CPB2	7.439538	-0.1187	0.084867	-1.39864	0.16192	NA
ARL15	40.64751	0.118674	0.072089	1.64622	0.099719	NA
COMTD1	8.78326	0.118616	0.084869	1.397633	0.162223	NA
NLGN1	290.7631	0.118595	0.050782	2.335388	0.019523	NA
FBN1	52.39569	-0.11851	0.080544	-1.47141	0.14118	NA
PRELID2	3.48354	-0.11851	0.077434	-1.53048	0.125898	NA
MRPL41	50.82143	-0.11849	0.066843	-1.77265	0.076287	NA
HNRNPUL	181.8888	0.118486	0.054464	2.175487	0.029594	NA
BEST1	22.04411	-0.11848	0.083603	-1.4172	0.156423	NA
SSFA2	949.2567	-0.11842	0.043214	-2.74035	0.006137	0.161641
TTLL12	140.5753	-0.11841	0.061729	-1.91824	0.05508	NA
RB1	228.8784	0.11839	0.045738	2.588425	0.009642	NA
LAS1L	290.9246	-0.11839	0.061052	-1.93914	0.052485	NA
MAGEE2	33.76356	0.118384	0.071739	1.650213	0.098899	NA
HIST1H4A	13.76186	0.11838	0.084444	1.401874	0.160953	NA
SNORD115	4.217746	-0.11838	0.079777	-1.48383	0.137855	NA
GTPBP1	187.4217	-0.11836	0.049126	-2.40935	0.015981	NA
RAB11FIP4	493.521	0.118316	0.044091	2.683458	0.007287	0.171303
ACSM5	7.812503	-0.1183	0.081781	-1.44653	0.14803	NA
SLITRK3	156.4144	0.118266	0.060197	1.96464	0.049456	NA
FAM178B	10.01773	0.118255	0.083743	1.41213	0.157912	NA
TRIM11	158.8526	-0.11824	0.062521	-1.89126	0.05859	NA
PJA1	133.0271	0.118203	0.055358	2.135234	0.032742	NA
SLC35F3	187.4664	-0.1182	0.060444	-1.95559	0.050513	NA
OR10A5	1.115482	-0.1182	0.056409	-2.09545	0.036131	NA
GPD2	289.9399	0.118198	0.069715	1.695434	0.089993	NA
ARHGAP18	18.95766	-0.11819	0.083352	-1.41798	0.156196	NA
KBTBD4	37.67608	0.118185	0.072947	1.620134	0.105204	NA
LONRF2	1586.164	0.118178	0.044285	2.668603	0.007617	0.171303
SMIM4	15.16394	-0.11817	0.084448	-1.39934	0.16171	NA
PGRMC1	176.0192	0.118142	0.072855	1.62161	0.104887	NA
ACRC	65.55543	0.118132	0.084939	1.390787	0.16429	NA
SYNPO	96.23959	0.118103	0.078137	1.511489	0.130664	NA
LOC10012	93.10553	0.118097	0.07001	1.686858	0.091631	NA
GMPR	21.33723	-0.11807	0.082954	-1.42339	0.154624	NA
NOTCH4	46.33078	-0.11805	0.079938	-1.47673	0.139748	NA
CGGBP1	240.6791	0.117986	0.057517	2.051318	0.040236	NA
ITPK1-AS1	31.7786	-0.11797	0.080566	-1.46425	0.143125	NA
TPM2	223.4211	-0.11796	0.083981	-1.40466	0.160122	NA
TMEM179	20.62657	0.117962	0.079357	1.486484	0.137151	NA
IMPG2	12.35173	0.117934	0.083785	1.407577	0.159256	NA

MIR4666A	2.608574	-0.11793	0.071708	-1.64452	0.100068	NA
KLC1	1513.245	0.117923	0.042728	2.759884	0.005782	0.156126
GIPC2	3.451205	-0.11791	0.078046	-1.51075	0.130853	NA
NOV	13.22533	0.117829	0.082257	1.432448	0.152016	NA
DCTN3	54.94763	-0.11782	0.071867	-1.63946	0.101117	NA
PCDHGB8P	84.43469	0.117818	0.082713	1.42442	0.154325	NA
CTNND1	39.63987	-0.11782	0.074407	-1.58342	0.113326	NA
BDKRB1	1.9108	0.117813	0.062755	1.877345	0.060471	NA
C18orf56	0.961154	-0.11781	0.049755	-2.36774	0.017897	NA
ZBTB41	494.4834	0.117798	0.045157	2.608639	0.00909	0.183021
DENND1B	80.01189	0.117783	0.06062	1.942958	0.052021	NA
TYMS	3.874452	-0.11778	0.076506	-1.53945	0.123694	NA
KCNH2	252.2316	0.117772	0.046082	2.555702	0.010597	NA
MIS18A	21.5892	-0.11771	0.078565	-1.4983	0.134056	NA
SLC4A7	264.9398	0.117694	0.056877	2.069272	0.038521	NA
PIGB	37.58787	-0.11767	0.0764	-1.54013	0.123528	NA
ADORA1	94.97812	0.117638	0.075953	1.548826	0.121424	NA
MORN3	1.942721	-0.11764	0.066445	-1.77041	0.076659	NA
SLC5A9	5.257418	-0.11763	0.081	-1.45228	0.146424	NA
GEMIN8	44.08477	-0.11763	0.073329	-1.60415	0.108681	NA
CACNA2D4	1.025726	-0.11755	0.053434	-2.19982	0.027819	NA
PSMB3	29.99622	-0.11747	0.079625	-1.47527	0.140141	NA
DAND5	2.519397	-0.11747	0.071548	-1.64177	0.100637	NA
ZMYND15	2.005438	0.117461	0.066922	1.755197	0.079226	NA
CSTF2T	151.7521	0.117456	0.051026	2.301881	0.021342	NA
ACHE	89.54316	0.117442	0.07842	1.497608	0.134235	NA
MBOAT1	3.328421	0.117433	0.071271	1.647709	0.099412	NA
PCDHGA9	94.28467	0.11741	0.079203	1.482384	0.138238	NA
GJC2	7.995665	0.117391	0.084361	1.391537	0.164063	NA
TCTN1	20.55318	-0.11735	0.081082	-1.44734	0.147802	NA
NFE2L3	133.3803	-0.11734	0.070297	-1.66927	0.095064	NA
HGSNAT	157.9013	-0.11734	0.077766	-1.50891	0.131323	NA
STAT6	29.68217	-0.11728	0.079305	-1.47888	0.139173	NA
CFLAR	1204.36	-0.11725	0.052287	-2.24248	0.02493	0.247662
HSPA8	2497.533	0.11725	0.078336	1.496749	0.134459	0.487416
PDE8B	65.07025	-0.11719	0.072794	-1.60992	0.107416	NA
CSTF1	68.30783	0.117185	0.062816	1.86554	0.062106	NA
SALL4	5.868538	-0.11718	0.07912	-1.48105	0.138594	NA
NDUFB9	101.6895	-0.11715	0.08479	-1.38168	0.167069	NA
HLA-DPB1	101.5934	-0.11715	0.075478	-1.55205	0.120649	NA
TMC2	116.3608	-0.1171	0.076377	-1.5332	0.125227	NA
KCNK10	122.5035	0.117101	0.073877	1.585081	0.112948	NA
CRYBA1	8.920686	-0.11709	0.083977	-1.39429	0.163229	NA
SLC27A6	2.849493	-0.11706	0.066691	-1.75533	0.079203	NA
PMAIP1	1.561949	0.117059	0.060506	1.934654	0.053033	NA
CEP63	280.421	0.117013	0.045293	2.583461	0.009781	NA
COL6A6	32.11967	0.117012	0.079342	1.474793	0.140268	NA

FAM96A	37.37576	-0.11699	0.069918	-1.67322	0.094285	NA
IKZF3	1.889349	-0.11694	0.064776	-1.80529	0.071029	NA
ADARB2-A	3.516504	-0.11694	0.077827	-1.50251	0.132965	NA
LNX1	43.20267	0.116935	0.072672	1.609091	0.107596	NA
RMND5A	1150.859	0.116867	0.051429	2.272377	0.023064	0.23963
VTRNA1-1	4.446814	0.116863	0.073567	1.588518	0.112169	NA
DDX39B	6.430934	-0.11685	0.081002	-1.44261	0.149132	NA
ITGA9	31.70867	-0.11683	0.078195	-1.49414	0.135139	NA
GABBR2	634.1225	0.116808	0.058453	1.998314	0.045683	0.334577
HOMER2	146.3431	0.116806	0.050311	2.321656	0.020251	NA
KLHL18	100.1314	0.116772	0.056394	2.070662	0.03839	NA
ZNF581	10.83403	0.116765	0.084281	1.385424	0.165923	NA
CCR5	1.124182	-0.11676	0.049099	-2.37812	0.017401	NA
CENPK	3.351737	-0.11675	0.072268	-1.61548	0.106206	NA
HMGB2	125.2982	-0.11671	0.077956	-1.49715	0.134354	NA
TBCEL	117.2813	0.116694	0.055693	2.095317	0.036143	NA
SUMF1	36.11874	-0.11669	0.079032	-1.47652	0.139804	NA
TEX29	2.634746	0.116669	0.073848	1.579854	0.11414	NA
CLP1	11.88803	0.116664	0.083857	1.391227	0.164157	NA
PPP2CA	580.5247	0.116635	0.06343	1.838808	0.065943	0.375222
CCDC81	27.27782	0.116631	0.08438	1.382201	0.16691	NA
VAX2	28.64025	0.116618	0.083277	1.400359	0.161406	NA
METTL2B	50.86215	-0.1166	0.065164	-1.78926	0.073572	NA
MRPS21	81.53742	-0.11659	0.066704	-1.7479	0.080482	NA
ANKS4B	67.10162	-0.11658	0.067097	-1.73742	0.082314	NA
LOC40065	54.88782	-0.11657	0.065557	-1.77817	0.075376	NA
MIPEPP3	8.757516	0.116566	0.084887	1.373193	0.169692	NA
PROP1	5.366726	-0.11654	0.082343	-1.41533	0.156971	NA
TMEM158	6.22861	0.116529	0.078321	1.487848	0.136791	NA
DCAF16	224.5723	-0.11651	0.05546	-2.10081	0.035658	NA
SCARNA17	61.19495	-0.11651	0.07428	-1.56847	0.116771	NA
SNORA11B	1.834672	-0.11649	0.067239	-1.73256	0.083175	NA
HEPH	34.32566	-0.11649	0.081713	-1.42555	0.153997	NA
CARD11	10.67425	-0.11648	0.08473	-1.3747	0.169226	NA
NRP2	20.31469	-0.11647	0.080731	-1.44272	0.149101	NA
CALML4	149.7606	-0.1164	0.04759	-2.44597	0.014446	NA
PTPRU	145.7455	0.116364	0.071369	1.630465	0.103003	NA
INE1	27.61776	-0.11636	0.07528	-1.54565	0.12219	NA
REEP4	7.353879	-0.11635	0.084938	-1.36979	0.170752	NA
IQCH	12.71269	-0.11632	0.08427	-1.38036	0.167475	NA
FAM134B	152.088	-0.1163	0.060572	-1.92007	0.054849	NA
ANKHD1-E	4.619224	0.116296	0.080942	1.436785	0.150779	NA
BCL2L10	1.448684	-0.11629	0.062551	-1.85915	0.063007	NA
WNT5B	3.516922	-0.11629	0.075323	-1.54389	0.122615	NA
SNORD84	11.76668	-0.11627	0.081488	-1.42685	0.153624	NA
F13A1	11.92468	-0.11626	0.070649	-1.64557	0.099853	NA
TRIM17	306.7023	-0.11625	0.065957	-1.76246	0.077992	NA

PLK1S1	401.6596	-0.11622	0.053857	-2.158	0.030928	0.277495
ZIK1	48.91546	0.116218	0.063963	1.816958	0.069224	NA
HRASLS5	1.949928	0.116218	0.064687	1.796606	0.072398	NA
MAN2A1	76.10161	-0.11617	0.083262	-1.39523	0.162948	NA
OXCT2	4.081058	0.11613	0.077967	1.489467	0.136365	NA
LINC00173	59.04904	-0.11612	0.08145	-1.4257	0.153954	NA
AMD1	318.891	0.116092	0.053663	2.16333	0.030516	NA
MAB21L2	14.65433	-0.11608	0.083492	-1.39036	0.164421	NA
LY96	2.364434	-0.11604	0.072312	-1.60474	0.10855	NA
PAPSS2	26.49483	-0.11603	0.084805	-1.36825	0.171234	NA
CHSY1	113.1559	0.116029	0.056592	2.050269	0.040338	NA
BEND6	277.6523	0.115988	0.063807	1.817798	0.069095	NA
MRPL54	16.01293	-0.11598	0.083604	-1.38728	0.165358	NA
ENDOU	4.150213	-0.11596	0.07985	-1.45225	0.146432	NA
GALNT14	13.4408	-0.11596	0.084918	-1.36553	0.172087	NA
PAXIP1	518.1292	0.115947	0.061618	1.88171	0.059875	0.366535
C11orf70	4.546351	0.115931	0.073653	1.574023	0.115482	NA
DLGAP2	13.13619	-0.11592	0.084726	-1.36816	0.171261	NA
BCL2	62.45412	-0.1159	0.07245	-1.59978	0.109647	NA
ARHGEF33	217.2066	-0.1159	0.071966	-1.61045	0.1073	NA
ZNF493	526.8488	0.11587	0.062925	1.841398	0.065563	0.375222
AAMDC	13.91566	-0.11584	0.083268	-1.39121	0.164161	NA
DMC1	5.343678	-0.11584	0.081468	-1.42188	0.155061	NA
ABCC1	122.7856	0.115825	0.064873	1.785419	0.074193	NA
SUV420H1	421.8393	0.115816	0.033766	3.42991	0.000604	0.075062
BEST4	15.48084	0.11581	0.08173	1.416981	0.156488	NA
FUK	44.08217	-0.11579	0.071034	-1.63008	0.103084	NA
RAPGEF2	702.5461	0.115777	0.048252	2.399424	0.016421	0.223611
SNORD98	3.552091	0.115707	0.07607	1.521064	0.128244	NA
ULK4	65.46244	-0.11566	0.071794	-1.61106	0.107166	NA
C19orf40	1.084838	0.115648	0.05514	2.097354	0.035962	NA
SLC25A53	20.84759	0.115644	0.079956	1.446351	0.148079	NA
NDUFA6	153.4597	-0.11564	0.068739	-1.68237	0.092498	NA
NCAM2	221.3585	-0.11564	0.069599	-1.66154	0.096604	NA
DNAJA4	412.3598	0.115632	0.060268	1.918636	0.05503	0.358172
BMI1	32.19096	0.115618	0.079705	1.450583	0.146896	NA
CDRT15P1	3.090327	-0.1156	0.074058	-1.56097	0.11853	NA
SNORA5C	2.911615	-0.1156	0.073482	-1.57322	0.115669	NA
ANKRD13A	128.9027	-0.1156	0.049715	-2.32527	0.020058	NA
FERMT3	9.303722	0.115576	0.082182	1.406342	0.159623	NA
COL11A2	115.1713	-0.11557	0.07449	-1.55143	0.120798	NA
NOL11	194.75	0.115559	0.051952	2.224337	0.026126	NA
OR7D2	0.816102	-0.11556	0.041823	-2.76299	0.005727	NA
ZMYND11	764.1487	0.115525	0.050261	2.298511	0.021533	0.23963
ZNF800	255.6145	0.115501	0.064284	1.796732	0.072378	NA
COX4I1	416.4993	-0.11548	0.076807	-1.50356	0.132695	0.487416
C2orf80	1.089203	-0.11544	0.053434	-2.1605	0.030734	NA

FNTA	168.6955	-0.11543	0.052454	-2.20054	0.027769	NA
RPL13AP3	4.595709	0.115413	0.073178	1.57715	0.114761	NA
COX10-AS	26.05392	0.115408	0.07524	1.533868	0.125062	NA
FAM22A	5.832558	-0.1154	0.082341	-1.40145	0.16108	NA
CALM1	5734.555	0.115388	0.049712	2.321115	0.020281	0.235785
CCNL2	703.6613	-0.11536	0.053759	-2.14593	0.031878	0.27878
ASPRV1	25.93739	-0.11534	0.08172	-1.41139	0.158129	NA
C1orf56	22.46236	-0.11533	0.073365	-1.57202	0.115945	NA
PITPNA	371.7425	0.11533	0.060048	1.920643	0.054777	0.35783
MYL5	6.178331	0.115304	0.080346	1.435096	0.15126	NA
SNORD35A	5.510903	-0.1153	0.076447	-1.50824	0.131494	NA
ACBD7	146.8428	-0.11526	0.079087	-1.45735	0.14502	NA
ASCC1	167.4253	-0.11524	0.046832	-2.46073	0.013866	NA
HES1	24.17321	-0.11523	0.084879	-1.35757	0.1746	NA
CDK5R1	269.1167	0.115212	0.062782	1.835123	0.066487	NA
GOLGA5	122.264	0.115206	0.052818	2.181179	0.02917	NA
LOC28562	3.068073	0.115159	0.072301	1.592773	0.111211	NA
LRRC42	22.61542	0.115148	0.074329	1.549174	0.12134	NA
HAVCR1P1	16.19361	-0.11513	0.083474	-1.37928	0.167807	NA
IL1RAPL1	54.65179	-0.11512	0.066709	-1.72578	0.084386	NA
YIF1B	28.91321	-0.11509	0.073928	-1.55675	0.119531	NA
RAP2C	41.26568	-0.11508	0.06649	-1.73072	0.083502	NA
IP6K3	5.762225	-0.11507	0.081127	-1.41835	0.156087	NA
ERO1LB	143.2769	-0.11503	0.062565	-1.83858	0.065977	NA
IVD	128.2145	-0.11498	0.056643	-2.02998	0.042359	NA
NDST3	196.1515	0.114982	0.075176	1.529503	0.12614	NA
C12orf5	47.43861	0.114962	0.084693	1.357401	0.174654	NA
SMPD2	12.34106	-0.11491	0.084471	-1.3604	0.173703	NA
KEL	5.21819	-0.11489	0.081659	-1.4069	0.159456	NA
ZNF91	1364.366	0.114881	0.042094	2.729125	0.00635	0.163447
ADRA2A	13.72531	0.114872	0.075341	1.524698	0.127334	NA
ESYT1	101.4906	-0.11487	0.046693	-2.46017	0.013887	NA
AHSA2	227.7761	-0.11486	0.067173	-1.70988	0.087288	NA
PPM1F	158.0963	0.114849	0.058602	1.959813	0.050018	NA
WBP2NL	13.59534	0.114825	0.084644	1.356572	0.174917	NA
TMEM19	91.93719	0.11482	0.063273	1.814677	0.069574	NA
GTF2H5	101.2294	0.114808	0.075868	1.51326	0.130214	NA
PRDM10	243.0388	-0.1148	0.06232	-1.84208	0.065464	NA
SSX2IP	537.043	0.114779	0.057852	1.984004	0.047255	0.337641
RUNX2	11.35893	-0.11476	0.084698	-1.35491	0.175446	NA
NDNL2	41.12409	0.114728	0.065354	1.755491	0.079175	NA
NMNAT3	15.95361	-0.11472	0.081635	-1.4053	0.159932	NA
LOC10050	44.08695	0.114685	0.067997	1.686613	0.091678	NA
KCMF1	184.1042	0.114681	0.053026	2.162726	0.030562	NA
ARHGEF28	33.08974	-0.11468	0.080152	-1.43074	0.152505	NA
WDR4	33.53351	0.114676	0.075336	1.522193	0.127961	NA
LEPROT	83.95589	-0.11461	0.063835	-1.79535	0.072598	NA

EXOSC9	57.42336	-0.1146	0.062256	-1.84082	0.065648	NA
GSR	145.7229	-0.11457	0.059038	-1.94064	0.052302	NA
PRKD2	14.50587	-0.11457	0.083607	-1.37035	0.170577	NA
COPE	90.74523	-0.11454	0.076858	-1.49028	0.13615	NA
PPP1R16A	85.59233	-0.11454	0.064546	-1.7745	0.07598	NA
C11orf87	43.33312	0.114536	0.084679	1.35259	0.176187	NA
NCKAP1	1149.88	0.114533	0.048771	2.348388	0.018855	0.231397
ITPRIPL1	17.03312	0.114519	0.082728	1.384286	0.166271	NA
PTPN4	1109.762	0.114516	0.035304	3.243727	0.00118	0.092144
TRMT112	47.86068	-0.11447	0.078098	-1.46573	0.142721	NA
PAQR9	4.516187	0.114468	0.079673	1.436724	0.150797	NA
DNAJB6	144.8031	0.114448	0.074855	1.528916	0.126285	NA
LOC10050	28.38767	-0.11441	0.082078	-1.39397	0.163327	NA
HIVEP2	1574.396	0.114412	0.047756	2.395765	0.016586	0.223611
NFAM1	2.318009	-0.1144	0.066914	-1.70972	0.087318	NA
ZNF835	46.85284	0.114391	0.068517	1.66954	0.09501	NA
ZNF770	299.19	0.114366	0.042873	2.667525	0.007641	NA
C11orf68	50.10017	0.114342	0.069774	1.638748	0.101266	NA
HADHB	170.523	-0.11433	0.064323	-1.77747	0.075492	NA
SNORD121	3.118544	-0.1143	0.072075	-1.58578	0.112789	NA
LSM10	14.07443	0.114241	0.082555	1.383814	0.166415	NA
GPR65	2.483868	-0.11423	0.069918	-1.63372	0.102317	NA
SLC26A10	171.9844	-0.11422	0.053301	-2.14302	0.032112	NA
C2orf15	17.46214	-0.11421	0.080791	-1.41359	0.157481	NA
LINC00260	10.26761	-0.1142	0.084939	-1.34448	0.178794	NA
GDPGP1	14.55465	0.114197	0.082156	1.390008	0.164526	NA
KLF7	446.4035	0.114183	0.046768	2.441455	0.014628	0.212391
LOC10012	8.710323	-0.11418	0.07154	-1.59599	0.110491	NA
POLR2A	920.3927	0.114144	0.052662	2.167476	0.030199	0.275997
ZNF382	277.5234	0.114135	0.052817	2.160964	0.030698	NA
TBK1	147.4955	0.114073	0.048406	2.356578	0.018444	NA
TSGA10IP	3.30658	0.114052	0.071432	1.596657	0.110342	NA
SELPLG	11.66425	-0.11405	0.084521	-1.34933	0.177232	NA
THAP7	39.66239	0.114006	0.068518	1.663888	0.096135	NA
BHLHE40	97.40381	-0.11397	0.073192	-1.55721	0.11942	NA
F5	34.56916	-0.11397	0.082966	-1.37375	0.169519	NA
NHS	86.49929	0.113961	0.061401	1.856022	0.06345	NA
GPR101	6.781772	-0.11391	0.076361	-1.49179	0.135755	NA
LRP10	36.33573	-0.11388	0.081882	-1.39083	0.164276	NA
NAE1	208.9789	-0.11384	0.051453	-2.21254	0.026929	NA
MTMR9LP	13.74252	-0.11383	0.084532	-1.34662	0.178104	NA
LOC15077	148.4315	-0.11379	0.081052	-1.40393	0.160339	NA
FZD3	442.836	0.113792	0.036698	3.100785	0.00193	0.104087
AKAP11	1594.13	0.113789	0.052876	2.15202	0.031396	0.277495
TPBGL	1.7216	-0.11375	0.058709	-1.93759	0.052673	NA
RPS13	87.91087	-0.11372	0.083291	-1.36532	0.172152	NA
C19orf24	16.8735	0.113702	0.084938	1.338644	0.180687	NA

EDEM1	89.85833	-0.11369	0.056192	-2.02321	0.043052	NA
LDB1	254.6106	-0.11363	0.04746	-2.39434	0.01665	NA
NHSL1	237.0324	-0.11362	0.065071	-1.74602	0.080808	NA
THY1	216.305	0.113593	0.07651	1.484686	0.137627	NA
SUPT20H	187.4313	0.113546	0.046227	2.456255	0.014039	NA
KIAA0754	236.7998	-0.11352	0.057748	-1.96571	0.049333	NA
RAB22A	201.3221	0.113489	0.059439	1.909343	0.056218	NA
ZNF566	99.60506	0.113484	0.04746	2.391124	0.016797	NA
FAM153B	110.9238	-0.11348	0.076879	-1.47609	0.139921	NA
ERV3-1	77.76848	0.113471	0.076052	1.492015	0.135695	NA
GPR116	47.88613	0.113434	0.080145	1.415354	0.156965	NA
TMED10P1	65.56548	0.113389	0.067775	1.673024	0.094323	NA
RBM12	440.2849	0.113347	0.040037	2.83107	0.004639	0.150804
HCG9	3.113646	0.113345	0.073658	1.538799	0.123853	NA
TOB1	121.6276	0.113344	0.055771	2.032307	0.042123	NA
GPR85	83.68921	0.113324	0.057039	1.986775	0.046947	NA
ZFAND4	232.3736	0.113313	0.051146	2.215491	0.026726	NA
HNRNPA0	186.3203	0.113309	0.058547	1.935362	0.052946	NA
METTL18	13.13924	0.113305	0.082631	1.371221	0.170306	NA
CDH6	19.89356	0.113285	0.084721	1.337157	0.181171	NA
OLFM1	455.8483	0.113232	0.057795	1.959205	0.050089	0.34688
CORO6	78.24115	-0.1132	0.079041	-1.43217	0.152095	NA
RSBN1L-AS	17.10057	-0.11318	0.081265	-1.39268	0.163717	NA
MFN1	268.8142	-0.11316	0.066255	-1.70799	0.087637	NA
MYOT	32.56721	-0.11316	0.077976	-1.45121	0.146721	NA
PRLHR	3.982193	-0.11311	0.066048	-1.71249	0.086807	NA
PCDHGC4	136.1749	0.113043	0.056893	1.986958	0.046927	NA
CCT6A	239.6644	0.113043	0.05273	2.143792	0.03205	NA
GLDC	48.38025	-0.11304	0.08392	-1.34695	0.177997	NA
LZIC	77.38169	0.113019	0.05315	2.126437	0.033467	NA
MTRNR2L	1.406042	-0.11298	0.062736	-1.80085	0.071727	NA
SLC9A6	278.5359	0.112975	0.064968	1.738924	0.082048	NA
ANGPTL1	38.81961	-0.11296	0.075791	-1.49037	0.136126	NA
ZNF626	98.63235	0.112952	0.061236	1.844529	0.065106	NA
TMTC1	52.45491	-0.11295	0.083015	-1.36055	0.173656	NA
LOC10012	5.011583	-0.11294	0.072958	-1.54797	0.12163	NA
SLA	14.13693	-0.11292	0.082749	-1.36461	0.172377	NA
C19orf66	57.23103	-0.11289	0.065865	-1.71398	0.086533	NA
ZMIZ1-AS1	14.58059	-0.11288	0.084928	-1.32916	0.183795	NA
PRR18	11.02857	-0.11286	0.0839	-1.34522	0.178553	NA
RALY	173.192	-0.11281	0.044703	-2.52347	0.01162	NA
BIVM	79.63707	-0.11277	0.059885	-1.88313	0.059682	NA
KIAA0317	139.7533	0.112746	0.05414	2.082497	0.037297	NA
WEE2	2.290148	-0.11269	0.070409	-1.60045	0.109499	NA
HAS2	0.769634	-0.11268	0.047154	-2.38969	0.016863	NA
CABP7	31.50991	-0.11268	0.083923	-1.34265	0.179384	NA
TTC39A	9.523128	0.112667	0.08491	1.326908	0.184539	NA

GPBP1	690.9469	0.112666	0.029735	3.788987	0.000151	0.075062
GPC2	3.925165	-0.11266	0.071286	-1.58045	0.114003	NA
RAB34	8.394133	-0.11264	0.084771	-1.32881	0.183912	NA
GPM6B	834.6444	-0.11264	0.071197	-1.58211	0.113623	0.464543
ZNF134	136.0607	0.112636	0.051769	2.175754	0.029574	NA
PRKAB2	242.4895	-0.11263	0.059697	-1.88675	0.059194	NA
TMEM232	22.6794	-0.11262	0.080294	-1.40259	0.160739	NA
LOC10050	4.552051	0.112604	0.078938	1.426484	0.153729	NA
CRLF3	505.2206	-0.1126	0.053415	-2.10797	0.035033	0.288416
NUP62	99.3925	0.112592	0.060786	1.852272	0.063987	NA
MBNL3	8.817499	-0.11259	0.084404	-1.33393	0.182226	NA
C6orf120	65.98587	0.112549	0.062947	1.788014	0.073774	NA
EFNB2	130.7303	0.112501	0.063196	1.780206	0.075042	NA
CHST7	21.49131	-0.11248	0.078326	-1.43606	0.150985	NA
PCDHA4	39.12443	0.112481	0.07567	1.486469	0.137155	NA
RPL10A	172.4107	-0.11242	0.081799	-1.37439	0.169321	NA
CD84	7.962481	-0.1124	0.080834	-1.39045	0.164394	NA
ACACB	248.8567	-0.11238	0.067806	-1.65736	0.097447	NA
PRR3	145.4406	-0.11237	0.067925	-1.65429	0.098068	NA
MIR2909	1.946312	-0.11236	0.068764	-1.63393	0.102274	NA
GTF2H3	54.37654	-0.11235	0.06891	-1.63035	0.103027	NA
TST	8.569659	-0.11234	0.084183	-1.3345	0.182041	NA
PEX11G	4.85685	-0.11233	0.081779	-1.37357	0.169575	NA
SPSB3	198.7633	-0.11232	0.047301	-2.37454	0.017571	NA
VILL	14.7387	-0.11231	0.084829	-1.32393	0.185525	NA
HADHA	257.3965	-0.1123	0.056276	-1.99559	0.045979	NA
NUDT21	383.1519	0.112296	0.062901	1.78529	0.074214	0.390538
PLAUR	20.30302	0.112274	0.081164	1.383289	0.166576	NA
EPT1	356.9956	0.112267	0.065426	1.715934	0.086174	0.410051
HIBADH	68.6685	-0.11227	0.060804	-1.84635	0.064841	NA
ZNF75D	45.15791	0.112264	0.065536	1.713007	0.086711	NA
ZNRF2	124.8396	0.112208	0.057839	1.940009	0.052379	NA
BCL2L14	3.149781	0.112181	0.073828	1.519483	0.128641	NA
COL27A1	872.4978	0.112123	0.084927	1.320219	0.186762	0.538188
GMPR2	74.19301	-0.11211	0.053189	-2.10779	0.035049	NA
SEZ6L2	357.333	0.112108	0.070845	1.582445	0.113548	0.464543
POLR2G	25.66494	-0.11207	0.08073	-1.38816	0.165089	NA
SNORD46	12.35392	-0.11204	0.08281	-1.353	0.176057	NA
SMG8	52.22981	0.112034	0.066422	1.686693	0.091662	NA
MOG	54.04768	-0.11199	0.084309	-1.32838	0.184053	NA
PDCD7	206.9705	-0.11199	0.050859	-2.20188	0.027674	NA
SNORA67	12.48523	-0.11197	0.084924	-1.31851	0.187334	NA
BRE-AS1	5.20427	0.11196	0.083083	1.347566	0.177798	NA
LRP4	252.6127	-0.11196	0.076929	-1.45531	0.145582	NA
ST6GALNA	12.18053	-0.11195	0.08481	-1.32001	0.186832	NA
JAK3	43.14949	-0.11193	0.08303	-1.34806	0.177638	NA
TRIB2	144.6851	-0.11193	0.073789	-1.51682	0.129312	NA

SLC37A4	28.85608	-0.11192	0.07214	-1.55141	0.120803	NA
APEX2	11.12431	0.111884	0.084731	1.320452	0.186684	NA
SLCO1C1	12.24164	-0.11188	0.084793	-1.3194	0.187036	NA
GABRA6	708.6503	0.111858	0.065601	1.705121	0.088172	0.41454
CHCHD2	101.3198	-0.11184	0.081156	-1.37804	0.168192	NA
DNAJB5	143.1302	0.111832	0.066764	1.675024	0.09393	NA
MGAT5	501.2921	0.111777	0.049755	2.246559	0.024668	0.247228
LSMD1	38.0581	-0.11177	0.081503	-1.37133	0.170271	NA
NEGR1	368.2771	0.111759	0.058156	1.92172	0.054641	0.35783
CBX3	138.272	0.111733	0.054636	2.045044	0.040851	NA
USP18	4.995087	-0.11171	0.081514	-1.37043	0.170552	NA
PLOD1	53.24527	0.111704	0.074944	1.490494	0.136094	NA
NEK5	4.792412	-0.11169	0.081403	-1.37201	0.170061	NA
ABCA2	1276.469	-0.11166	0.048554	-2.29977	0.021461	0.23963
HAUS1	29.26461	-0.11157	0.070583	-1.5807	0.113946	NA
ASB17	2.029946	0.111567	0.063482	1.757475	0.078837	NA
MTRNR2L	5.227333	-0.11154	0.079444	-1.40405	0.160303	NA
VHLL	5.635076	0.111537	0.070167	1.589596	0.111926	NA
NFKBID	37.70922	-0.11154	0.073968	-1.50789	0.131584	NA
TTC37	710.4581	-0.11154	0.046543	-2.3964	0.016557	0.223611
ASCL3	2.096788	-0.11152	0.065455	-1.70383	0.088413	NA
RAP1GDS1	155.6761	0.111521	0.061149	1.823769	0.068187	NA
C4orf27	44.81181	-0.11151	0.067468	-1.65278	0.098376	NA
RFTN1	81.85161	-0.1115	0.081866	-1.36196	0.173209	NA
TXNDC15	260.6425	-0.11149	0.063744	-1.74901	0.080289	NA
PRAP1	1.12676	-0.11147	0.057843	-1.92719	0.053956	NA
SCLT1	261.4407	0.11147	0.052331	2.130081	0.033165	NA
LMCD1	24.01112	-0.11146	0.084939	-1.31229	0.189422	NA
TMEM128	25.29218	-0.11146	0.077802	-1.43261	0.151969	NA
CNTN1	1944.521	0.111458	0.051338	2.171074	0.029926	0.275534
COL4A2	67.00527	-0.11145	0.084749	-1.315	0.18851	NA
C17orf78	15.41155	-0.11142	0.083672	-1.33166	0.182972	NA
STRC	100.3685	-0.11142	0.080007	-1.39265	0.163725	NA
ST8SIA3	774.601	0.111409	0.040722	2.735836	0.006222	0.161992
LOC10012	35.58234	0.111409	0.081144	1.372971	0.169761	NA
IGF1R	691.3003	-0.11141	0.059915	-1.85942	0.062967	0.37053
ACTRT3	12.96233	0.111385	0.084384	1.31998	0.186842	NA
SETD5-AS1	47.00039	0.111385	0.070099	1.588977	0.112066	NA
LIPH	2.854898	-0.11136	0.073657	-1.51194	0.130549	NA
FOSB	2.683132	0.111356	0.059148	1.882677	0.059744	NA
LOC28486	6.160495	0.111354	0.078421	1.419954	0.155621	NA
PGS1	63.78681	-0.11134	0.060878	-1.82885	0.067421	NA
UHRF2	232.4715	-0.11133	0.04585	-2.42818	0.015175	NA
GCNT2	99.65464	-0.11133	0.073094	-1.52311	0.127731	NA
LOC10028	9.760351	-0.11132	0.08485	-1.31201	0.189517	NA
WDR83	67.08953	-0.11132	0.069319	-1.60592	0.108292	NA
PARD3	76.89118	-0.11131	0.078963	-1.40965	0.158644	NA

STL	16.0979	-0.1113	0.084938	-1.31041	0.190059	NA
CCDC34	14.95733	0.111278	0.082874	1.342733	0.179358	NA
ABCB4	1.823335	-0.11127	0.066801	-1.66569	0.095776	NA
TSPAN14	49.72153	-0.11123	0.078035	-1.42534	0.154059	NA
HSH2D	3.195541	0.111215	0.074627	1.490278	0.136151	NA
TMEM41A	24.26188	-0.11118	0.078978	-1.40768	0.159226	NA
PROSER2	3.926445	0.111167	0.079015	1.406923	0.15945	NA
C8orf76	9.511874	-0.11116	0.084237	-1.31961	0.186967	NA
ARID5B	149.873	-0.11115	0.075994	-1.46258	0.143583	NA
TGFBRAP1	78.30199	0.111135	0.055326	2.008719	0.044567	NA
RPGR	108.8512	-0.11112	0.070101	-1.5852	0.11292	NA
SIK1	27.41374	-0.11111	0.081453	-1.36405	0.172553	NA
UBQLN2	189.7461	0.111091	0.063298	1.755065	0.079248	NA
CDK2	15.49439	-0.11101	0.084927	-1.30715	0.191163	NA
POPDC3	3.045374	-0.111	0.07072	-1.56953	0.116525	NA
PRKCA	357.9456	-0.11099	0.072099	-1.53942	0.123702	0.477296
CCDC110	24.61114	0.110985	0.083953	1.32199	0.186171	NA
GPX2	29.65213	-0.11097	0.079037	-1.40402	0.160314	NA
FGD2	8.046508	-0.11094	0.07674	-1.44572	0.148255	NA
TNNC1	2.940754	-0.11094	0.065971	-1.68159	0.092648	NA
TECPR1	103.0133	-0.11092	0.058661	-1.89081	0.05865	NA
C20orf201	2.447465	0.110871	0.070548	1.571576	0.116049	NA
43162	35.53136	0.110865	0.079717	1.390735	0.164306	NA
LURAP1	49.81622	0.110834	0.064498	1.718418	0.08572	NA
EXOSC3	133.3213	0.110827	0.047216	2.347239	0.018913	NA
PCDHA13	47.17983	0.11082	0.07504	1.476827	0.139722	NA
PRDM4	203.7934	0.110804	0.047886	2.313916	0.020672	NA
NEK3	20.20622	-0.1108	0.081547	-1.35874	0.174228	NA
ATP2B1	1153.623	0.110789	0.046095	2.403514	0.016238	0.223611
ZNF317	162.6141	0.110789	0.044061	2.514465	0.011921	NA
SULT1A1	117.7103	-0.11075	0.07386	-1.4994	0.133771	NA
LINC00222	12.49059	0.110735	0.084907	1.304192	0.192168	NA
FAM169A	411.0722	0.110731	0.047528	2.329782	0.019818	0.235011
KIAA0556	103.9931	0.110711	0.060094	1.842294	0.065432	NA
SGK494	206.9509	0.110704	0.075982	1.456979	0.145122	NA
MALSU1	31.72056	-0.1107	0.069813	-1.58571	0.112805	NA
USP22	1061.871	0.110684	0.058323	1.89778	0.057725	0.364493
S100A1	34.11077	-0.11065	0.084748	-1.30564	0.191677	NA
USP53	264.215	-0.11063	0.049621	-2.22953	0.025779	NA
MRPS25	279.4954	-0.11061	0.052788	-2.0954	0.036136	NA
ADPRHL2	30.11952	0.110593	0.078153	1.41509	0.157042	NA
FAM198A	32.11837	-0.11059	0.078598	-1.40706	0.159411	NA
PSMA2	74.43699	-0.11056	0.071667	-1.54274	0.122893	NA
DACT3	21.1404	-0.11056	0.079478	-1.39112	0.16419	NA
CHST15	36.76037	-0.11055	0.081821	-1.35108	0.176669	NA
CYFIP1	125.5654	-0.11054	0.058122	-1.90191	0.057183	NA
ZCCHC17	300.9055	-0.1105	0.044118	-2.50474	0.012254	NA

SMUG1	15.9914	0.110481	0.081433	1.356715	0.174872	NA
TSPYL1	1014.944	0.110474	0.052737	2.094818	0.036187	0.292449
STK32A	60.32493	-0.11046	0.074407	-1.48449	0.13768	NA
PEX2	70.30653	-0.11042	0.060103	-1.83712	0.066193	NA
LOC73009	10.17982	-0.11041	0.0817	-1.35144	0.176556	NA
GUCY1A3	66.63875	0.110402	0.082289	1.341629	0.179716	NA
SLC48A1	113.6654	-0.1104	0.060609	-1.82146	0.068538	NA
CNIH4	25.82899	-0.11037	0.076187	-1.44872	0.147415	NA
ZXDB	62.20932	0.11035	0.062445	1.76715	0.077203	NA
DCUN1D3	42.92477	0.110317	0.071402	1.545004	0.122345	NA
DSTNP2	32.37152	0.110284	0.069275	1.591989	0.111387	NA
IRG1	1.854355	-0.11027	0.06091	-1.81039	0.070235	NA
43347	1379.101	-0.11025	0.067653	-1.6297	0.103165	0.445167
SYCE2	18.11101	-0.11025	0.081913	-1.34594	0.178321	NA
CD80	3.979028	-0.11025	0.079451	-1.38764	0.165246	NA
KCNQ3	60.50157	-0.1102	0.082546	-1.33503	0.181867	NA
GPR125	84.68744	0.110159	0.075556	1.457975	0.144847	NA
NUMBL	137.9955	-0.11014	0.053179	-2.07121	0.03834	NA
NETO1	3.72473	-0.11014	0.07798	-1.41243	0.157824	NA
ZNF117	148.7701	0.110118	0.067837	1.623265	0.104533	NA
CCDC141	12.20836	-0.11011	0.080694	-1.36451	0.172408	NA
MOCOS	1.537178	0.110068	0.050235	2.191041	0.028449	NA
ADAMTSL4	22.09355	-0.11005	0.083716	-1.31461	0.18864	NA
INPP5B	255.6197	-0.11005	0.057718	-1.90674	0.056555	NA
SNORD111	3.939574	-0.11005	0.078236	-1.40661	0.159542	NA
NTRK3	433.503	-0.11004	0.059879	-1.83771	0.066105	0.375222
ZNF92	91.06723	0.110035	0.062919	1.748819	0.080322	NA
KANK4	8.757364	0.110034	0.072126	1.525575	0.127116	NA
RNASEL	17.88035	-0.11003	0.084923	-1.29565	0.195097	NA
FKBP4	786.3329	0.110017	0.078673	1.398418	0.161988	0.519328
MEX3B	33.05651	0.110015	0.072043	1.527066	0.126745	NA
GSDMD	9.00708	-0.10998	0.084926	-1.29502	0.195314	NA
ENC1	20.89116	-0.10996	0.080859	-1.35987	0.173872	NA
PMF1	9.387898	0.109956	0.084539	1.300654	0.193377	NA
PRR26	159.7268	-0.10995	0.066837	-1.64504	0.099961	NA
ZNF468	45.38036	0.109931	0.072494	1.516414	0.129415	NA
TMEM119	3.212387	-0.10993	0.071376	-1.54011	0.123534	NA
ZNF572	12.53662	0.109886	0.084776	1.296197	0.194908	NA
RASSF4	204.6052	-0.10989	0.056406	-1.94812	0.051401	NA
SLC2A13	74.32639	0.10987	0.08149	1.348266	0.177573	NA
LLPH	22.42786	0.109859	0.076714	1.432063	0.152126	NA
TNIP2	23.80614	-0.10984	0.084394	-1.30146	0.1931	NA
MEPCE	108.4957	0.109831	0.057619	1.90615	0.056631	NA
EEF1B2	140.2242	-0.10983	0.082686	-1.32823	0.184102	NA
SHISA6	255.0056	-0.10982	0.081429	-1.34862	0.177458	NA
ATP6V0B	36.81306	-0.10976	0.080495	-1.36358	0.172699	NA
SNORD85	11.31868	-0.10975	0.080974	-1.3554	0.17529	NA

ZNF808	132.5676	0.109745	0.054628	2.008948	0.044543	NA
FAM20B	255.0794	0.109737	0.052589	2.08668	0.036917	NA
MINOS1P1	67.29861	-0.10972	0.080685	-1.35986	0.173874	NA
DMGDH	3.303029	-0.10971	0.075963	-1.4443	0.148654	NA
HTATSF1	459.775	0.10971	0.061216	1.792195	0.073102	0.389589
C5orf51	136.7678	0.109701	0.048795	2.24819	0.024564	NA
MTHFD1	210.8759	-0.10968	0.053708	-2.04207	0.041144	NA
LINC00574	2.969192	0.109658	0.075732	1.447974	0.147624	NA
LRRTM2	287.917	0.109654	0.043766	2.50548	0.012229	NA
PYROXD1	46.48353	0.109633	0.07241	1.514055	0.130012	NA
GGCT	33.46405	-0.10962	0.076642	-1.43034	0.152621	NA
KCNN4	1.71147	-0.1096	0.06422	-1.70664	0.087889	NA
EIF2AK3	156.2066	0.109594	0.052428	2.090381	0.036584	NA
ULK3	239.2124	-0.10955	0.058103	-1.88552	0.059359	NA
HGFAC	18.87938	0.109546	0.078164	1.401482	0.16107	NA
JMJD6	245.7412	0.109538	0.079923	1.370541	0.170518	NA
CTF1	2.628335	-0.10953	0.074347	-1.47318	0.140704	NA
SREBF1	108.85	-0.10953	0.072293	-1.51502	0.129767	NA
PRRT2	909.0313	-0.1095	0.044505	-2.4604	0.013878	0.210965
LOC40068	4.230029	0.109469	0.079759	1.372502	0.169907	NA
MRP63	78.50074	0.109453	0.06078	1.800804	0.071734	NA
HINT1	110.4262	-0.10945	0.083125	-1.31664	0.187959	NA
RECQL4	101.1637	0.109417	0.083581	1.309109	0.190497	NA
MIR340	5.550106	-0.10942	0.081757	-1.33829	0.180801	NA
ST3GAL5	81.95079	-0.10939	0.061291	-1.78476	0.074301	NA
CD163	31.73139	-0.10939	0.058927	-1.85629	0.063412	NA
C6orf57	25.44329	-0.10938	0.084168	-1.2996	0.193739	NA
TIMM22	66.66205	-0.10935	0.061921	-1.76592	0.077409	NA
LPIN3	8.891825	-0.10934	0.084721	-1.29064	0.196829	NA
SLC12A6	268.2387	-0.10933	0.043885	-2.49122	0.012731	NA
GPX1	32.70647	-0.1093	0.081977	-1.33335	0.182418	NA
GOT1	164.1698	0.10928	0.074931	1.458416	0.144726	NA
NBPF16	8.150963	-0.10927	0.068863	-1.58679	0.11256	NA
SLC4A1	5.715203	0.109267	0.081443	1.341643	0.179712	NA
HLA-DPA1	49.00965	-0.10923	0.084916	-1.28636	0.198316	NA
SLC2A6	56.17231	0.109224	0.064502	1.693333	0.090392	NA
OR4N4	2.136033	-0.10922	0.042604	-2.56356	0.01036	NA
MIR641	4.489316	-0.10921	0.080971	-1.34877	0.177412	NA
BANF1	25.30397	0.109204	0.080609	1.354733	0.175503	NA
IL18	7.177739	-0.1092	0.084577	-1.29111	0.196667	NA
NBPF15	21.42086	0.109185	0.080204	1.361355	0.173402	NA
DPM2	15.64252	0.109183	0.082434	1.324485	0.185342	NA
OXTR	13.20076	-0.10918	0.081085	-1.34643	0.178164	NA
ABCD2	242.8384	0.109152	0.057203	1.908136	0.056374	NA
RGS18	2.548176	-0.10915	0.065471	-1.6671	0.095494	NA
CAMK2N2	108.3433	0.109147	0.073397	1.487066	0.136997	NA
FOXD2-AS	0.700947	-0.10912	0.04425	-2.46611	0.013659	NA

MSX1	3.774393	0.109119	0.076453	1.427268	0.153503	NA
REXO4	140.2661	0.109114	0.049771	2.192338	0.028355	NA
ELOVL2	71.75843	-0.10911	0.081822	-1.33352	0.18236	NA
PIK3CG	3.048824	-0.10907	0.074651	-1.46104	0.144005	NA
FAM117A	49.19086	0.109068	0.062749	1.738161	0.082182	NA
LOC10050	1.870593	-0.10905	0.066531	-1.6391	0.101193	NA
BTN2A3P	4.394584	-0.10905	0.080332	-1.35746	0.174636	NA
TBC1D22A	81.66785	-0.10904	0.065011	-1.67727	0.093489	NA
KIF26B	130.1415	0.10904	0.072118	1.511973	0.130541	NA
AP5M1	114.4486	0.10902	0.048141	2.264592	0.023538	NA
FTX	167.9757	-0.10897	0.053537	-2.03544	0.041807	NA
BTF3P11	1.006523	-0.10896	0.050234	-2.16912	0.030074	NA
CRHR1	154.1445	0.108956	0.064287	1.694842	0.090105	NA
C2orf61	5.460391	-0.10894	0.083518	-1.30436	0.19211	NA
MOSPD3	13.14091	0.108925	0.082686	1.317337	0.187726	NA
ZFP69B	31.76524	0.108919	0.074564	1.460744	0.144086	NA
FCER1G	4.842066	-0.1089	0.068997	-1.57835	0.114484	NA
RAMP1	47.19315	-0.1089	0.074065	-1.47029	0.141483	NA
TLE6	8.999071	0.108895	0.076629	1.421065	0.155298	NA
TTYH2	52.70391	-0.1089	0.08095	-1.34521	0.178558	NA
KCNG1	13.48371	-0.10889	0.078925	-1.37969	0.167681	NA
API5	317.9145	0.108881	0.049349	2.20634	0.02736	NA
ROCK2	1187.448	0.108848	0.043668	2.492594	0.012681	0.205975
PRICKLE3	4.841568	0.108834	0.080913	1.345069	0.178603	NA
CRYM-AS1	3.103687	-0.10882	0.067166	-1.62022	0.105185	NA
SNORD115	49.04514	-0.10882	0.084756	-1.28392	0.199169	NA
DDX43	4.461765	0.108813	0.061368	1.773121	0.076209	NA
LUC7L3	3175.687	-0.1088	0.0653	-1.66614	0.095685	0.427469
HAUS5	114.0846	-0.1088	0.063089	-1.72447	0.084623	NA
KIAA1656	24.66201	-0.10879	0.077896	-1.39661	0.16253	NA
OR52B6	1.555297	-0.10878	0.058738	-1.85202	0.064023	NA
KIAA0141	275.3367	-0.10877	0.043168	-2.51973	0.011744	NA
MSRB2	37.38215	0.108748	0.073808	1.47339	0.140646	NA
LPPR3	2.469368	-0.10874	0.070048	-1.55233	0.120582	NA
RPS6KB2	85.09748	-0.10872	0.057474	-1.89167	0.058535	NA
MIR3117	5.19095	-0.10872	0.083105	-1.30818	0.190812	NA
MPPED2	70.81561	-0.10871	0.072742	-1.49446	0.135055	NA
USP43	5.626958	-0.1087	0.080339	-1.35299	0.176059	NA
BDKRB2	55.19336	0.108679	0.076932	1.41266	0.157756	NA
LOC10050	145.9862	-0.10866	0.068328	-1.59026	0.111775	NA
DNAH5	27.06398	0.108643	0.083559	1.300187	0.193537	NA
ZBTB8OS	55.08655	-0.10864	0.059353	-1.83033	0.067201	NA
CYP2D7P1	15.81624	-0.10861	0.082927	-1.30966	0.190312	NA
ZNF700	82.00171	0.108605	0.05447	1.993858	0.046168	NA
TBX3	9.831874	-0.1086	0.084049	-1.29205	0.196341	NA
GPR45	1.743353	0.108556	0.066586	1.630303	0.103038	NA
MAGEE1	150.9985	0.108548	0.062253	1.743655	0.081219	NA

SNORD115	90.11932	-0.10855	0.084891	-1.27865	0.201021	NA
TCF19	52.79354	0.108542	0.079927	1.358027	0.174455	NA
CERS6	207.9002	0.108539	0.053884	2.014322	0.043976	NA
PIGK	81.34515	0.108516	0.075164	1.443717	0.148819	NA
NAP1L2	336.1402	0.10851	0.063662	1.704483	0.088291	0.41454
HSD17B6	21.29671	0.108502	0.084234	1.288106	0.197709	NA
ZNF645	5.576941	-0.10848	0.082299	-1.31816	0.18745	NA
BASP1P1	1.492234	-0.10848	0.062483	-1.73609	0.082547	NA
MCTP1	624.1941	0.108474	0.048646	2.229871	0.025756	0.251454
EIF1AX	244.547	0.108422	0.062383	1.737997	0.082211	NA
DKFZp451	4.554965	-0.10842	0.08089	-1.34032	0.18014	NA
ZNF277	70.80885	0.108418	0.058347	1.858174	0.063144	NA
PCDHGB4	88.86336	0.108416	0.082493	1.314249	0.188762	NA
RNFT1	29.4635	0.108414	0.071718	1.511666	0.130619	NA
RDH13	84.94993	-0.10839	0.065153	-1.66364	0.096185	NA
C1QTNF2	15.80938	0.10839	0.083371	1.300094	0.193569	NA
FBXL7	58.1272	0.108343	0.084936	1.275582	0.202103	NA
PTAR1	483.4	0.108325	0.043703	2.478663	0.013188	0.206483
SEC11A	60.82378	-0.10829	0.06436	-1.6826	0.092452	NA
HPX	8.696388	-0.10829	0.084458	-1.28217	0.199782	NA
SNORD66	17.64087	-0.10828	0.080564	-1.34402	0.178942	NA
FOXF2	7.575219	0.108258	0.084322	1.283867	0.199189	NA
EN2	225.4406	0.108234	0.061005	1.774174	0.076034	NA
EXOSC6	92.54616	0.10823	0.063111	1.714909	0.086362	NA
CEBPG	160.6545	0.108223	0.050313	2.150981	0.031478	NA
CAMK2A	79.06027	-0.10821	0.072641	-1.48964	0.136319	NA
KIAA1407	45.39685	-0.1082	0.075623	-1.43078	0.152493	NA
IL32	3.86255	0.108197	0.066435	1.628623	0.103393	NA
CLVS1	95.53791	-0.1082	0.064649	-1.67359	0.094211	NA
SPI1	10.63865	-0.10818	0.084521	-1.2799	0.200579	NA
NMT2	349.4497	-0.10817	0.061966	-1.74569	0.080865	0.400784
HIST1H4F	1.796521	-0.10815	0.060042	-1.8013	0.071655	NA
CANT1	63.19421	0.108144	0.074658	1.44854	0.147466	NA
TIPARP-AS	2.37804	-0.10814	0.07253	-1.49097	0.13597	NA
FAM63B	525.6363	0.108127	0.044112	2.451164	0.014239	0.212327
PTPRN	271.5108	0.108096	0.056744	1.904991	0.056781	NA
VEZF1	180.6131	0.108084	0.05115	2.113088	0.034593	NA
SERPINA10	3.624178	0.108062	0.075201	1.436974	0.150725	NA
CCRN4L	31.52919	-0.10803	0.071357	-1.51393	0.130043	NA
UGGT2	55.05799	-0.10798	0.081243	-1.32907	0.183825	NA
KCNK3	50.39621	0.107969	0.074611	1.447077	0.147875	NA
DUSP5	67.27385	0.107933	0.084789	1.272966	0.20303	NA
TMEM120	30.10818	0.107913	0.075544	1.428481	0.153154	NA
PRKG1-AS	1.472767	-0.10789	0.06015	-1.79368	0.072865	NA
LOC28507	108.2884	-0.10789	0.065849	-1.6384	0.101338	NA
KCNH8	15.99965	-0.10784	0.083309	-1.29451	0.195488	NA
CHAC2	16.39575	0.107796	0.080367	1.341306	0.179821	NA

MAP6	167.7342	0.10779	0.057971	1.85937	0.062975	NA
FOXO3B	52.56643	0.107773	0.070525	1.528161	0.126472	NA
MFSD2A	12.57027	0.10775	0.083511	1.29025	0.196964	NA
SUGP1	125.7814	-0.10769	0.045468	-2.36853	0.017859	NA
NAMPT	123.9691	0.107679	0.06895	1.56171	0.118356	NA
BAIAP2-AS	42.48559	0.107644	0.078365	1.373634	0.169555	NA
C16orf3	7.592112	0.107644	0.084813	1.26919	0.204373	NA
ZNF786	98.39785	0.107637	0.065096	1.653502	0.098229	NA
DENND5B-	1.770927	-0.10763	0.065582	-1.64122	0.100753	NA
CEACAM1	29.83759	-0.10763	0.081219	-1.32519	0.185109	NA
PAWR	28.40746	-0.10762	0.084939	-1.267	0.205156	NA
NUAK1	368.6331	0.107611	0.067916	1.58448	0.113085	0.464015
CLTC	1268.297	0.107603	0.058857	1.828209	0.067518	0.378951
CDK9	204.5788	-0.10757	0.048721	-2.20794	0.027249	NA
SNORA12	134.2612	-0.10756	0.079554	-1.35208	0.176349	NA
FBXO30	136.8922	0.107558	0.049497	2.173023	0.029779	NA
HPSE	2.711509	-0.10755	0.073295	-1.46732	0.142289	NA
ZNF804A	162.6672	0.10754	0.08064	1.333588	0.182339	NA
HSF5	5.196273	0.107539	0.082477	1.303863	0.19228	NA
UNC80	2717.532	-0.1075	0.041971	-2.56127	0.010429	0.198661
HCN1	192.0447	0.107499	0.076626	1.402912	0.160643	NA
LOC10050	12.51328	-0.10749	0.084933	-1.26561	0.205655	NA
LYSMD1	73.39743	-0.10747	0.05351	-2.0084	0.044601	NA
43355	11.40198	-0.10747	0.079378	-1.35389	0.175772	NA
MANBAL	29.39836	-0.10739	0.078273	-1.37201	0.17006	NA
S100A11	15.57593	-0.10739	0.070902	-1.51459	0.129875	NA
KCNH7	161.8554	0.107386	0.083866	1.280449	0.200387	NA
PCDHA3	34.22891	0.107365	0.081544	1.316661	0.187952	NA
SNHG15	13.12711	0.107351	0.082951	1.29416	0.19561	NA
FAM184B	71.6679	-0.10733	0.062788	-1.70943	0.087371	NA
FAT1	1034.86	0.107331	0.077645	1.382328	0.166871	0.521454
TMEM253	6.809722	-0.10732	0.081563	-1.31578	0.188248	NA
SELO	152.3965	-0.10731	0.053928	-1.98991	0.046601	NA
DUSP6	20.0379	-0.10731	0.083975	-1.27787	0.201297	NA
UCN	2.404901	-0.10728	0.064866	-1.65387	0.098154	NA
LOC33952	6.552884	-0.10728	0.081709	-1.31291	0.189213	NA
ASB14	77.99169	-0.10727	0.071885	-1.49224	0.135637	NA
ARHGEF25	415.0923	-0.10726	0.052466	-2.04445	0.040909	0.313037
TNFRSF21	80.40055	0.10724	0.064528	1.661907	0.096531	NA
ZNF32	50.85779	-0.10724	0.071295	-1.50413	0.132548	NA
LOC20161	0.700228	-0.1072	0.04584	-2.33851	0.019361	NA
GMPPB	20.13727	-0.10718	0.080903	-1.32477	0.185246	NA
C1orf216	71.51281	0.107177	0.069483	1.542497	0.122953	NA
ZNF215	2.881822	-0.10717	0.069444	-1.54325	0.12277	NA
LRP1B	1026.335	0.107163	0.067834	1.579779	0.114157	0.465042
SLC30A10	12.64762	-0.10716	0.082935	-1.29209	0.196327	NA
ADORA3	7.670549	-0.10716	0.076232	-1.40568	0.159818	NA

PCDHB7	18.51669	-0.10716	0.082996	-1.2911	0.196669	NA
RAB26	153.4641	-0.10715	0.07452	-1.43788	0.150468	NA
NME3	44.74717	-0.1071	0.068267	-1.56883	0.116688	NA
AKAP8L	529.8712	-0.10709	0.058978	-1.81573	0.069412	0.385666
HAUS3	234.9814	0.107083	0.052156	2.053152	0.040058	NA
STK32B	4.34909	-0.10708	0.080483	-1.33043	0.183376	NA
DUSP18	20.2302	0.107059	0.08377	1.278019	0.201243	NA
UBP1	427.2674	0.107051	0.044018	2.431957	0.015018	0.216391
SNORD116	19.62497	-0.10705	0.083668	-1.27943	0.200745	NA
LRRC8B	35.81305	0.107016	0.079367	1.34837	0.177539	NA
LOC28314	15.34688	0.107014	0.083872	1.27592	0.201984	NA
PEBP4	6.312411	-0.10701	0.081327	-1.31585	0.188224	NA
PTCHD2	27.02243	-0.10696	0.083127	-1.2867	0.198199	NA
SNORA58	3.253767	-0.10695	0.07636	-1.40056	0.161347	NA
CAMKK2	1598.191	0.106945	0.05985	1.786875	0.073958	0.390538
LOC44166	61.68755	0.106931	0.080432	1.329466	0.183694	NA
PCDHGB2	136.5915	0.106929	0.072771	1.469385	0.141728	NA
LIME1	43.48138	-0.10689	0.082742	-1.29187	0.196401	NA
MIR26A2	0.855719	-0.10688	0.049522	-2.15818	0.030913	NA
SNORD115	3.558433	-0.10687	0.077407	-1.38062	0.167395	NA
SNPH	158.8495	0.106856	0.058009	1.842064	0.065466	NA
SARDH	11.46533	-0.10686	0.084933	-1.25811	0.208351	NA
GCAT	45.75124	-0.10685	0.067841	-1.57499	0.115258	NA
CALCOCO2	284.8294	-0.10683	0.058627	-1.82224	0.068419	NA
DDX17	3403.818	-0.10682	0.039879	-2.67862	0.007393	0.171303
SNORD113	3.790682	-0.10678	0.075409	-1.41598	0.156781	NA
ZNF233	91.7043	0.106754	0.07004	1.524194	0.12746	NA
RPP30	80.10923	-0.10673	0.052039	-2.051	0.040267	NA
CLU	3686.92	-0.10673	0.074409	-1.43438	0.151462	0.503763
TRAF6	194.8346	0.106723	0.048432	2.203553	0.027556	NA
LOC40068	3.134631	-0.10672	0.075549	-1.41256	0.157786	NA
PPT2	17.1866	-0.10671	0.078932	-1.35196	0.176388	NA
SNORD29	95.05264	-0.1067	0.082063	-1.30019	0.193535	NA
DLK2	20.58002	0.106683	0.082511	1.292948	0.196029	NA
CACFD1	55.78078	0.106678	0.077191	1.381988	0.166976	NA
SMA4	19.7695	-0.10668	0.084933	-1.256	0.209118	NA
WWTR1	78.29646	-0.10667	0.076334	-1.39746	0.162276	NA
TPRKB	59.74846	-0.10665	0.065449	-1.62948	0.103211	NA
SNORD34	8.680839	-0.10664	0.066662	-1.59975	0.109655	NA
DIRAS2	1150.586	0.106639	0.062978	1.693288	0.090401	0.415466
GPR144	1.086456	-0.10664	0.05196	-2.05227	0.040143	NA
TMEM150	16.31324	-0.10662	0.07957	-1.33998	0.180253	NA
PCDHGB5	71.05456	0.106616	0.07888	1.351612	0.1765	NA
TAS2R42	0.774446	-0.10661	0.049723	-2.14408	0.032026	NA
NR1D1	260.4156	-0.1066	0.061802	-1.72483	0.084557	NA
NUTF2	40.76072	0.106577	0.066226	1.60929	0.107553	NA
LTC4S	8.001856	-0.10658	0.084851	-1.25604	0.209102	NA

SLC16A2	54.7592	0.106552	0.064923	1.641195	0.100757	NA
AKAP5	61.78837	0.10653	0.059802	1.781382	0.07485	NA
STAC	56.65469	0.10651	0.072183	1.475564	0.140061	NA
CHRNB2	158.8236	0.10649	0.061296	1.737305	0.082333	NA
WAS	15.40631	-0.10648	0.082961	-1.28344	0.199338	NA
ZNF34	99.97461	-0.10646	0.067021	-1.5885	0.112173	NA
MEP1B	11.60329	0.106448	0.084939	1.253222	0.210125	NA
ACPP	1.185283	-0.10645	0.045071	-2.36175	0.018189	NA
NXNL2	1.222593	-0.10642	0.056331	-1.88912	0.058876	NA
BOLA3-AS	92.11253	0.106413	0.066159	1.608437	0.10774	NA
ATF1	100.708	0.106407	0.053455	1.990575	0.046528	NA
TENC1	190.5986	-0.1064	0.066933	-1.58965	0.111914	NA
RC3H2	418.3657	0.106387	0.038685	2.750105	0.005958	0.158753
PKDREJ	14.18001	-0.10636	0.083586	-1.27245	0.203212	NA
CD81	288.1099	-0.10635	0.06268	-1.69666	0.08976	NA
ADK	59.04098	-0.10634	0.064332	-1.65306	0.09832	NA
ENO1	528.5762	-0.10634	0.070291	-1.51285	0.130319	0.487283
EMC8	26.39214	0.106331	0.075981	1.399444	0.16168	NA
NAA30	124.9703	0.106324	0.050011	2.126022	0.033501	NA
NGRN	502.829	0.106305	0.051361	2.069764	0.038474	0.300499
RPL12	229.864	-0.1063	0.076541	-1.38877	0.164903	NA
CD68	44.65047	-0.10628	0.081782	-1.29957	0.193749	NA
MEF2A	459.3332	0.106281	0.045333	2.344457	0.019055	0.231397
SNORD20	4.092188	-0.10628	0.068944	-1.54153	0.123189	NA
ADORA2B	8.696066	0.106268	0.084766	1.253658	0.209966	NA
HSP90B2P	56.03725	-0.10624	0.067052	-1.5845	0.113079	NA
PON1	3.979388	0.106198	0.07996	1.328137	0.184133	NA
KIAA0586	295.3805	-0.10618	0.046228	-2.29698	0.02162	NA
ZNF143	144.89	0.106181	0.056373	1.883531	0.059628	NA
IDH2	119.7204	-0.10616	0.078425	-1.35366	0.175845	NA
C2orf72	46.49357	0.106158	0.078288	1.355996	0.1751	NA
C3orf65	10.2361	0.106142	0.084363	1.258159	0.208334	NA
HERC5	11.04555	-0.10613	0.084936	-1.2495	0.211483	NA
CKS2	4.482589	-0.10612	0.075535	-1.40491	0.160049	NA
FAM71D	9.352736	0.106112	0.084743	1.252159	0.210512	NA
FAM90A2	5.359909	0.106083	0.082057	1.292806	0.196078	NA
ATP10A	41.69336	-0.10607	0.084771	-1.25123	0.21085	NA
CLIC5	12.57474	-0.10605	0.084175	-1.25986	0.207721	NA
RHOT2	157.1913	-0.10603	0.053225	-1.99217	0.046352	NA
SNRPA1	71.32462	-0.10603	0.069903	-1.51683	0.129309	NA
RPL23AP5	141.2462	0.106025	0.060698	1.746756	0.08068	NA
RABGGTA	57.17614	-0.10601	0.063434	-1.67113	0.094697	NA
FBXL14	67.12981	0.105984	0.069792	1.518558	0.128874	NA
SLX4	368.2996	-0.10597	0.060236	-1.75924	0.078537	0.397742
C5orf56	22.92386	-0.10597	0.073089	-1.44986	0.147098	NA
ZNF268	165.4198	0.105934	0.054897	1.929684	0.053646	NA
ARTN	7.030167	-0.10591	0.084892	-1.24753	0.212202	NA

CYP2B7P1	2.162163	-0.1059	0.069074	-1.53314	0.125242	NA
ATP1A1OS	35.61227	-0.10589	0.075121	-1.40965	0.158643	NA
ERRFI1	221.0725	-0.10589	0.047913	-2.21004	0.027103	NA
HNRNPC	721.8801	0.10588	0.039619	2.672432	0.00753	0.171303
BOLA3	9.503699	-0.10584	0.084921	-1.24639	0.21262	NA
C8orf33	105.7856	-0.10582	0.062184	-1.70181	0.088791	NA
MIR1281	8.562353	-0.10581	0.084669	-1.24963	0.211435	NA
RBM7	61.23612	0.105804	0.060949	1.735947	0.082573	NA
SHISA4	73.74712	-0.10578	0.069233	-1.52787	0.126544	NA
PPAPDC3	52.91504	0.105776	0.061333	1.724607	0.084598	NA
DRAM2	101.1015	-0.10574	0.065394	-1.617	0.105879	NA
RNPEP	61.33286	-0.10573	0.064514	-1.63894	0.101225	NA
SNORD115	9.282858	-0.10573	0.084741	-1.24772	0.212135	NA
LINC00184	7.563737	-0.10573	0.08391	-1.26005	0.207652	NA
UBALD2	45.75916	-0.10572	0.075353	-1.40305	0.160603	NA
CX3CL1	28.49767	0.105718	0.084668	1.248612	0.211807	NA
RAI2	27.04548	0.10571	0.080681	1.310222	0.190121	NA
CLDN2	2.121299	-0.10571	0.067302	-1.57061	0.116273	NA
PHAX	227.6575	0.105698	0.05171	2.044071	0.040947	NA
SAMD13	3.43785	-0.10569	0.079299	-1.33283	0.182589	NA
DISC1	3.273646	-0.10566	0.075569	-1.39819	0.162055	NA
RANBP2	1198.221	0.105621	0.036685	2.879129	0.003988	0.14186
GRHL3	9.350163	0.105618	0.08451	1.249776	0.211381	NA
MTHFSD	40.88909	0.105595	0.069027	1.529764	0.126075	NA
NDUFA7	20.25873	-0.10559	0.08167	-1.29284	0.196066	NA
PTK2B	686.9859	-0.10557	0.072334	-1.4595	0.144427	0.495647
CDKL1	24.64699	-0.10557	0.074357	-1.41973	0.155685	NA
OR1K1	2.172562	-0.10554	0.07162	-1.47367	0.14057	NA
CNTN4-AS	16.72063	0.105529	0.082007	1.286839	0.19815	NA
CNTNAP3B	14.75299	-0.10553	0.080733	-1.30709	0.191181	NA
FANCI	53.21931	-0.10552	0.075388	-1.39973	0.161596	NA
SNORA54	58.43666	-0.10551	0.07809	-1.35107	0.176672	NA
SPAST	178.7333	0.105483	0.046173	2.284544	0.02234	NA
LINC00304	25.54365	-0.10547	0.084592	-1.24676	0.212484	NA
PI4KAP1	27.89248	-0.10543	0.068842	-1.53148	0.125652	NA
STC2	3.519299	-0.10541	0.074174	-1.42109	0.15529	NA
MED28	166.0994	-0.1054	0.04917	-2.14351	0.032072	NA
BEST3	53.60057	-0.10535	0.080657	-1.30619	0.191488	NA
ZNF271	146.3098	0.10534	0.052	2.025749	0.04279	NA
EXPH5	1890.167	0.105335	0.045777	2.301042	0.021389	0.23963
NEUROG3	1.896017	-0.10533	0.05867	-1.79527	0.07261	NA
MIR381	1.786363	-0.10532	0.064209	-1.64025	0.100954	NA
HDAC2	251.1001	0.105309	0.048078	2.190375	0.028497	NA
SNORD56	14.33877	-0.1053	0.083496	-1.26119	0.20724	NA
FAM46C	7.279174	-0.1053	0.082672	-1.27366	0.202785	NA
FXYD5	12.81197	-0.10529	0.08304	-1.26801	0.204795	NA
VEGFA	253.8189	-0.10529	0.084407	-1.24742	0.212243	NA

BAHCC1	576.7243	-0.10529	0.04797	-2.19486	0.028173	0.266991
RAB6A	664.9076	0.105269	0.058071	1.812756	0.06987	0.385986
MVD	52.0542	-0.10526	0.065694	-1.60226	0.109097	NA
PSTK	22.78532	-0.10525	0.080796	-1.30272	0.19267	NA
UBLCP1	66.36331	0.105252	0.081781	1.287001	0.198094	NA
TEX22	8.477336	-0.10521	0.084928	-1.23878	0.215425	NA
NOSIP	51.12511	-0.10519	0.066475	-1.58247	0.113543	NA
PPIL6	32.05791	0.105155	0.070239	1.497096	0.134368	NA
CCDC122	15.08017	-0.10513	0.082873	-1.26855	0.204601	NA
HS1BP3	33.53631	-0.10512	0.068841	-1.52707	0.126744	NA
SLC39A3	47.23949	0.10509	0.068665	1.530473	0.1259	NA
TAS2R20	27.9949	-0.10508	0.080931	-1.29843	0.194138	NA
MIR210HG	8.658346	-0.10508	0.08337	-1.26035	0.207542	NA
LOC10049	13.29379	-0.10507	0.075473	-1.39222	0.163857	NA
VPS35	252.7906	0.105074	0.055364	1.897884	0.057711	NA
LSP1	2.595204	-0.10505	0.067645	-1.55289	0.12045	NA
SNORD94	15.99142	-0.10503	0.084422	-1.24407	0.213476	NA
B3GNT7	6.234395	-0.105	0.079319	-1.32382	0.185564	NA
SNORA32	2.971806	-0.10499	0.073459	-1.42926	0.152928	NA
PPAP2B	109.3087	-0.10497	0.081778	-1.2836	0.199282	NA
FAM167A	63.52197	-0.10493	0.084933	-1.23547	0.216654	NA
MYLK4	19.28704	0.104918	0.082405	1.2732	0.202947	NA
SUFU	78.45828	0.104797	0.057491	1.822825	0.06833	NA
IQCJ	4.184227	0.104784	0.067375	1.555224	0.119893	NA
TMEM218	30.2596	-0.10478	0.07549	-1.38794	0.165156	NA
PSMA6	96.29514	-0.10477	0.064547	-1.62309	0.10457	NA
CXXC5	260.9753	0.10475	0.054736	1.913721	0.055656	NA
STARD5	99.10739	-0.10475	0.06624	-1.58129	0.113813	NA
AFAP1L2	265.6143	0.104732	0.080774	1.296605	0.194767	NA
CEP128	178.101	-0.1047	0.059749	-1.75241	0.079704	NA
UBE2I	157.9554	0.104701	0.045552	2.298527	0.021532	NA
CDNF	3.1379	-0.1047	0.065844	-1.59014	0.111804	NA
ALMS1P	17.29233	-0.10469	0.084511	-1.23871	0.215451	NA
GPNMB	13.91035	0.104671	0.083685	1.250774	0.211017	NA
GRM7	15.59357	0.104663	0.083297	1.256499	0.208935	NA
ACVR1	60.20935	0.104644	0.066459	1.57458	0.115353	NA
PRICKLE2	54.43001	-0.10464	0.078707	-1.3295	0.183683	NA
NUDT8	6.614349	-0.10463	0.084876	-1.23274	0.217674	NA
LIAS	24.6253	-0.10462	0.075042	-1.3941	0.163289	NA
SPC25	0.483051	-0.1046	0.040633	-2.57432	0.010044	NA
FBLN5	6.341389	0.104602	0.079936	1.308571	0.19068	NA
PATE4	13.78307	-0.10458	0.082781	-1.2633	0.206482	NA
FOXP4	67.47346	-0.10457	0.074179	-1.40975	0.158613	NA
LOC72873	90.96719	-0.10457	0.072033	-1.45171	0.146582	NA
COL5A3	63.00745	-0.10457	0.083794	-1.2479	0.212069	NA
KCNMA1	420.9289	-0.10456	0.078327	-1.33488	0.181916	0.530978
ECT2	54.31459	0.104551	0.072198	1.448113	0.147585	NA

TPCN1	750.0525	-0.10453	0.061805	-1.69121	0.090797	0.415466
SGMS2	55.65551	0.1045	0.064144	1.62915	0.103281	NA
NUB1	237.4553	-0.10449	0.04714	-2.21667	0.026645	NA
ANKRD20A	10.34039	0.104479	0.084628	1.234562	0.216994	NA
KLHL29	74.48655	0.104477	0.06293	1.660206	0.096873	NA
MAOA	53.49642	-0.10446	0.084342	-1.23852	0.215525	NA
SPG21	35.06216	0.10445	0.067266	1.552792	0.120473	NA
GLTSCR2	204.4077	-0.10443	0.059759	-1.74758	0.080536	NA
ESRRB	2.633701	-0.10443	0.071549	-1.4595	0.144428	NA
FAM21C	126.158	0.104409	0.053685	1.94484	0.051794	NA
DCLRE1B	50.49182	0.104408	0.061485	1.698107	0.089487	NA
FAM161B	83.79168	0.104407	0.053857	1.938594	0.052551	NA
KCNIP4-IT	1108.325	0.104392	0.073211	1.425916	0.153893	0.506644
SCGB2B3P	4.932398	-0.10436	0.08147	-1.28098	0.200199	NA
LOC10050	15.46767	-0.10436	0.083905	-1.24375	0.21359	NA
UNC5C	196.8747	0.104317	0.083767	1.245321	0.213014	NA
DSCAM-AS	1.206521	-0.10428	0.05725	-1.82154	0.068526	NA
RIN1	34.31213	-0.10428	0.07606	-1.37103	0.170366	NA
PTX3	5.731891	-0.10427	0.080844	-1.28974	0.197143	NA
RABEPK	26.5108	-0.10427	0.079772	-1.30706	0.191193	NA
PTRH2	25.50451	0.104265	0.071803	1.452113	0.14647	NA
LOC10013	52.63673	-0.10425	0.071991	-1.44809	0.147592	NA
GABARAPL	346.1703	-0.10422	0.075485	-1.38071	0.167367	0.521454
ANKRD12	2373.593	0.104223	0.050264	2.073492	0.038126	0.300499
GPR148	6.214135	0.104205	0.082093	1.269357	0.204314	NA
SNORD11B	2.39828	-0.1042	0.066031	-1.57799	0.114568	NA
LOC40277	3.303404	-0.10419	0.078329	-1.33015	0.183469	NA
SENP6	940.4799	0.104189	0.033232	3.135225	0.001717	0.101784
TMEM116	39.6179	-0.10416	0.071817	-1.45031	0.146972	NA
TAS2R41	0.529012	-0.10415	0.036286	-2.87025	0.004101	NA
C22orf31	3.190613	0.104132	0.075547	1.37838	0.168086	NA
ADIPOR2	146.4319	-0.1041	0.068639	-1.51665	0.129355	NA
MFSD4	126.8717	0.104082	0.061254	1.699192	0.089283	NA
B4GALT2	21.10409	0.104075	0.076897	1.353427	0.175919	NA
ZBTB11	316.1044	0.104051	0.045081	2.308068	0.020995	NA
HYOU1	270.8418	0.104028	0.06898	1.508101	0.131529	NA
LIN7A	162.4719	0.104018	0.056657	1.83593	0.066368	NA
AK1	154.3995	-0.10401	0.057685	-1.80304	0.071382	NA
ESF1	247.8387	0.104009	0.047643	2.183104	0.029028	NA
LMNB2	205.605	0.103966	0.050869	2.043802	0.040973	NA
CTNNB1	934.0727	0.103954	0.052385	1.984404	0.047211	0.337641
PER2	250.9578	0.103935	0.070402	1.47631	0.139861	NA
AP2S1	21.27787	-0.10391	0.079733	-1.30322	0.192499	NA
ACP1	126.3326	-0.10389	0.052617	-1.97439	0.048337	NA
ING4	68.04615	-0.10388	0.064636	-1.6071	0.108033	NA
ELF1	62.55859	-0.10384	0.062909	-1.65068	0.098805	NA
RNPC3	354.3664	-0.10384	0.055754	-1.86249	0.062535	0.37053

FNBP4	841.8916	-0.10384	0.044816	-2.31693	0.020508	0.235785
GHRLOS	24.29092	0.103826	0.084621	1.226954	0.21984	NA
LOC72819	55.24225	-0.10382	0.075042	-1.38355	0.166497	NA
LMBRD2	244.123	0.10381	0.047388	2.190647	0.028477	NA
GPR111	7.730704	-0.1038	0.080791	-1.28484	0.198849	NA
ARGLU1	1140.819	-0.10378	0.055334	-1.87553	0.06072	0.368716
ZNF507	403.2055	0.103762	0.044124	2.351585	0.018694	0.231397
RNF166	40.04463	-0.10375	0.065482	-1.58443	0.113096	NA
PP14571	21.60038	0.103751	0.083955	1.235789	0.216537	NA
PCDH9	579.1787	-0.10374	0.056431	-1.83842	0.066001	0.375222
DDX60	18.50498	-0.10373	0.083604	-1.24071	0.214714	NA
LOC64136	13.87882	0.103708	0.083912	1.235914	0.21649	NA
CALCOCO1	848.8513	-0.1037	0.06726	-1.54176	0.123133	0.476745
TBC1D17	138.5563	-0.10369	0.056381	-1.83916	0.065892	NA
UNC5A	326.3477	-0.10369	0.072057	-1.43895	0.150166	NA
CBWD2	38.88406	0.103681	0.069225	1.497749	0.134198	NA
EPHA1	16.54458	-0.10368	0.083807	-1.23714	0.216035	NA
GK5	233.9711	-0.10368	0.059512	-1.74211	0.081489	NA
NUBP1	39.96227	0.103663	0.070487	1.470668	0.141381	NA
MTRF1	42.97093	0.103647	0.070787	1.464211	0.143136	NA
SFRP5	8.162905	-0.10364	0.084517	-1.22631	0.220081	NA
SCN1B	302.3314	0.103624	0.06787	1.526791	0.126813	NA
TTC7A	76.62199	-0.1036	0.056367	-1.83792	0.066074	NA
UBE2L3	168.2349	0.103595	0.048996	2.114356	0.034485	NA
NACAD	299.3794	0.103568	0.048741	2.124864	0.033598	NA
ZBTB2	42.83053	0.103554	0.067434	1.535634	0.124628	NA
EXOC5	382.7537	0.103552	0.040205	2.575605	0.010006	0.193715
NLRP9	4.302824	-0.10355	0.077529	-1.33565	0.181665	NA
ALKBH6	17.49799	-0.10355	0.080519	-1.28598	0.198449	NA
ADAP2	10.51099	-0.1035	0.084588	-1.22352	0.221134	NA
TP53RK	47.78264	0.103485	0.065397	1.582408	0.113556	NA
FBXL12	36.19527	0.103462	0.067164	1.540436	0.123454	NA
MEOX1	1.739763	-0.10339	0.062023	-1.66701	0.095513	NA
RRAGC	92.57673	0.103381	0.063711	1.622663	0.104661	NA
KLRC2	1.363672	-0.10338	0.055001	-1.87955	0.06017	NA
RGP1	41.99932	0.103368	0.067719	1.526427	0.126903	NA
CRLF1	13.5659	0.103354	0.084158	1.228096	0.219411	NA
MAPRE1	133.2147	0.103352	0.055028	1.878174	0.060357	NA
SVIL	109.6641	-0.10333	0.065797	-1.57043	0.116315	NA
ADAMTSL1	24.97269	-0.10333	0.084367	-1.22476	0.220666	NA
ST7-OT4	7.372024	-0.10321	0.08447	-1.22185	0.221763	NA
TMEM254	34.62602	-0.1032	0.067612	-1.52633	0.126927	NA
ZC3H4	271.4606	0.103172	0.042522	2.426337	0.015252	NA
SNORD114	3.768042	-0.10317	0.071021	-1.45268	0.146313	NA
LINC00266	1.67166	-0.10316	0.059552	-1.73227	0.083226	NA
SNORD104	18.15696	-0.10316	0.081288	-1.26904	0.204426	NA
TBX15	3.211584	0.103152	0.07405	1.393015	0.163615	NA

PDPK1	329.4396	0.103141	0.045403	2.27166	0.023107	NA
GPR98	678.2472	-0.10313	0.078734	-1.30992	0.190224	0.54182
MYLK	94.90992	-0.10312	0.075595	-1.36407	0.172545	NA
ANO7	13.65251	-0.10311	0.084614	-1.21864	0.222982	NA
PATE1	2.485828	-0.10311	0.069101	-1.49221	0.135645	NA
ZFR	818.7619	0.103109	0.041755	2.46937	0.013535	0.208552
SLC10A7	31.02935	-0.1031	0.077325	-1.33327	0.182442	NA
ALG5	33.12428	-0.10309	0.073252	-1.40736	0.159321	NA
PIK3R1	953.0528	0.103087	0.055336	1.862912	0.062475	0.37053
SHKBP1	13.0591	-0.10309	0.084159	-1.2249	0.220611	NA
GFPT1	351.3817	0.103057	0.036479	2.82506	0.004727	0.150804
SLC25A28	70.98704	-0.10306	0.061895	-1.665	0.095913	NA
CNR1	284.8245	0.103027	0.078304	1.315731	0.188264	NA
GLB1L	14.03712	-0.10301	0.084936	-1.21283	0.225195	NA
BMP8B	16.10261	-0.10301	0.082959	-1.24174	0.214334	NA
CCDC78	50.24079	-0.103	0.073372	-1.40375	0.160392	NA
KIF2C	20.14002	0.102934	0.080344	1.28117	0.200134	NA
ZNF697	43.71881	-0.10293	0.067522	-1.52439	0.127411	NA
PDE7B	15.4534	-0.10289	0.083645	-1.23006	0.218675	NA
PUM2	998.5968	0.102879	0.036591	2.811607	0.004929	0.151649
TTC32	15.92175	-0.10286	0.080495	-1.27786	0.2013	NA
SNTG1	78.49233	-0.10284	0.084151	-1.22214	0.221656	NA
HCRTR1	2.132824	-0.10283	0.066141	-1.55473	0.120011	NA
NOS3	17.35001	-0.1028	0.083219	-1.23524	0.21674	NA
C9orf91	47.97585	0.102776	0.069575	1.477183	0.139627	NA
LOC10028	2.669498	0.102771	0.072606	1.415464	0.156933	NA
PCYOX1	262.7027	0.102759	0.053612	1.916711	0.055275	NA
SPINT1	30.79372	0.102727	0.077227	1.330201	0.183452	NA
CENPA	0.622992	-0.10271	0.041739	-2.46075	0.013865	NA
NOL7	139.2621	-0.10269	0.053313	-1.92624	0.054074	NA
DTX2	19.48973	-0.10267	0.080059	-1.28239	0.199705	NA
AGA	8.638798	-0.10267	0.083723	-1.22628	0.220094	NA
C9orf40	16.89711	0.102652	0.081222	1.263847	0.206285	NA
TMEM255	20.98383	-0.10264	0.084779	-1.21067	0.226022	NA
C9orf156	62.1718	-0.10264	0.065886	-1.55778	0.119285	NA
PYCARD	8.348846	-0.10262	0.08487	-1.20918	0.226592	NA
MDK	17.69471	-0.10262	0.080684	-1.27188	0.203415	NA
GPR68	51.80921	-0.10259	0.07815	-1.31277	0.18926	NA
SNORA13	1.786254	-0.10255	0.061273	-1.67372	0.094186	NA
PSMB8	11.01762	-0.10254	0.084695	-1.2107	0.226009	NA
DOK3	14.28833	0.102516	0.084757	1.209522	0.226462	NA
LMTK2	549.0593	0.102509	0.045811	2.237647	0.025244	0.248599
SNORD113	2.308973	-0.1025	0.066003	-1.553	0.120422	NA
FAM129A	18.26599	-0.10249	0.084196	-1.21723	0.223516	NA
RHOBTB3	319.1115	-0.10248	0.068646	-1.49295	0.135451	NA
GID8	186.555	0.102483	0.041692	2.45809	0.013968	NA
LINC00663	9.013144	0.102471	0.084913	1.206781	0.227517	NA

YBEY	29.73949	-0.10242	0.080031	-1.27973	0.200641	NA
RBM33	1567.093	-0.10241	0.063824	-1.60463	0.108576	0.452973
PLD1	32.97454	-0.10241	0.084763	-1.2082	0.226971	NA
BUD31	86.57305	-0.10238	0.068226	-1.50065	0.133446	NA
FAM71F2	18.77025	-0.10238	0.084169	-1.21632	0.223864	NA
TBRG4	40.51065	-0.10236	0.07159	-1.42981	0.152773	NA
PLCD3	108.6101	-0.10236	0.070757	-1.44663	0.148002	NA
C8orf86	6.123084	-0.10236	0.081971	-1.24868	0.211782	NA
ENAH	345.7586	0.102346	0.041346	2.475372	0.01331	0.206483
HSD11B2	13.44306	-0.10235	0.084043	-1.21778	0.223308	NA
KCNIP3	12.1959	-0.10234	0.084583	-1.20999	0.226284	NA
SPTSSA	55.91797	-0.10234	0.065583	-1.56043	0.118658	NA
SULT4A1	327.5645	0.102318	0.065855	1.553698	0.120257	NA
GNG5	9.438486	-0.1023	0.084939	-1.20439	0.228438	NA
FAM46B	8.764726	-0.1023	0.083268	-1.22852	0.219252	NA
LRRC34	24.10771	0.102282	0.079091	1.293217	0.195936	NA
SULF2	77.70375	-0.10228	0.079738	-1.28264	0.199617	NA
KLHDC3	201.5752	-0.10227	0.05081	-2.01271	0.044145	NA
CDK5RAP3	252.8013	-0.10226	0.058736	-1.74108	0.08167	NA
SLC37A1	61.26959	-0.10226	0.06113	-1.67277	0.094373	NA
RDH5	8.598951	-0.10225	0.08479	-1.20594	0.227839	NA
HSD17B1	44.29481	0.102246	0.062557	1.63445	0.102164	NA
IRS2	411.1539	-0.10224	0.050454	-2.02643	0.04272	0.318294
ACN9	7.790862	-0.10224	0.083728	-1.22105	0.222066	NA
ZFP3	194.1141	0.102208	0.050819	2.011203	0.044304	NA
SFT2D1	28.86825	-0.10219	0.069385	-1.4728	0.140804	NA
RCC1	27.43143	0.102178	0.084913	1.203332	0.228848	NA
GNLY	2.092349	-0.10217	0.062144	-1.64414	0.100147	NA
LRFN3	67.20399	0.102155	0.063049	1.620251	0.105178	NA
TAX1BP1	436.3982	0.102148	0.040434	2.526281	0.011528	0.201925
DHFR	15.85425	0.102148	0.084825	1.204215	0.228506	NA
RBM4	348.6841	-0.10213	0.05901	-1.73074	0.083499	0.403119
TAC4	20.75267	-0.10211	0.084161	-1.21324	0.225039	NA
RPS27L	39.65061	-0.10209	0.079856	-1.27838	0.201116	NA
NSMF	142.951	-0.10206	0.064196	-1.58988	0.111862	NA
LOC28438	40.39354	0.10206	0.067635	1.508998	0.131299	NA
CNTN3	48.03902	-0.10205	0.084939	-1.20145	0.229576	NA
FOXE3	0.841669	-0.10204	0.049738	-2.05151	0.040217	NA
TMEM150	2.402914	0.10203	0.071841	1.420223	0.155543	NA
MARS2	40.61013	0.102027	0.06859	1.48749	0.136885	NA
MRPS16	92.15405	0.102018	0.053656	1.901343	0.057257	NA
PAX3	44.2085	-0.10201	0.076668	-1.33049	0.183356	NA
FLJ16779	64.06976	-0.102	0.084732	-1.2038	0.228667	NA
CACHD1	69.22055	-0.10198	0.071614	-1.42407	0.154426	NA
TGS1	262.1787	0.101968	0.043236	2.358392	0.018354	NA
PQLC1	71.97504	0.10196	0.0661	1.542517	0.122948	NA
BAG4	310.1323	0.101957	0.053789	1.895489	0.058028	NA

SRSF5	769.5678	-0.10196	0.060899	-1.67418	0.094095	0.421656
KCNAB1-A	4.596347	-0.10196	0.080989	-1.25888	0.208072	NA
PRDM8	30.6078	-0.10194	0.084939	-1.2001	0.230101	NA
FRG1B	41.09814	0.101915	0.084815	1.201603	0.229517	NA
HPS6	21.36979	-0.1019	0.077779	-1.31016	0.190141	NA
BAG3	288.8715	-0.10188	0.083321	-1.22273	0.221431	NA
ALDH1B1	8.654771	0.101844	0.084836	1.200483	0.229952	NA
DPYSL3	199.0595	-0.10184	0.068725	-1.48178	0.138399	NA
CCDC13	19.63254	0.101833	0.084014	1.21209	0.225478	NA
ELAC1	19.54059	0.10182	0.084485	1.205187	0.228131	NA
FBXO46	33.736	-0.10182	0.072974	-1.39523	0.162945	NA
PEX11A	23.43532	-0.10181	0.0808	-1.26002	0.207661	NA
NFYB	137.114	0.101786	0.059856	1.70052	0.089033	NA
EDC3	105.9054	0.101784	0.053975	1.885783	0.059324	NA
PLD2	104.2579	-0.10178	0.065986	-1.54244	0.122966	NA
CDH2	296.5095	0.101771	0.050137	2.029847	0.042372	NA
COL4A3	27.63329	-0.10176	0.078969	-1.28867	0.197514	NA
MEDAG	0.982536	-0.10176	0.052551	-1.93643	0.052816	NA
MED31	9.421212	-0.10175	0.084862	-1.19898	0.230535	NA
NICN1	103.5426	-0.10169	0.058105	-1.75019	0.080086	NA
RNASEH2C	101.917	-0.10166	0.067877	-1.49776	0.134196	NA
TNFSF10	7.389468	0.101636	0.074644	1.361616	0.173319	NA
FBXO42	120.2583	0.101635	0.054603	1.861328	0.062698	NA
APOBEC3G	4.613968	-0.10162	0.079188	-1.28327	0.199398	NA
PRSS50	5.302838	0.10156	0.081508	1.246011	0.21276	NA
CD93	18.54376	-0.10155	0.071057	-1.42914	0.152963	NA
TMEM109	63.45168	0.101541	0.064889	1.564846	0.117619	NA
SMIM5	19.05345	-0.10153	0.08289	-1.22485	0.220633	NA
PER1	268.0638	-0.10152	0.068619	-1.47946	0.139016	NA
AKR7A2P1	5.559104	-0.1015	0.082224	-1.23445	0.217035	NA
PSMG3	24.66831	-0.10148	0.078368	-1.29497	0.195329	NA
FAM3C	85.13349	0.101479	0.070778	1.43377	0.151638	NA
ALAS2	2.15754	0.101475	0.066088	1.535466	0.124669	NA
P2RY11	2.352423	0.101471	0.067463	1.504107	0.132554	NA
CETN2	48.80717	-0.10147	0.077176	-1.31474	0.188598	NA
RHOB	325.8101	-0.10146	0.069923	-1.45097	0.146788	NA
ASAP3	33.97965	-0.10144	0.082679	-1.22697	0.219836	NA
INPP1	78.02431	-0.10142	0.068529	-1.47997	0.13888	NA
HIP1R	312.1646	-0.10141	0.063754	-1.5906	0.111699	NA
NRM	6.663988	0.101387	0.083133	1.219578	0.222625	NA
CAPN9	5.649122	-0.10135	0.082423	-1.22967	0.218819	NA
TMEM63B	185.8604	0.101347	0.068186	1.486339	0.137189	NA
SNHG4	2.699109	-0.10134	0.074726	-1.3562	0.175036	NA
NBPF10	85.06934	-0.10134	0.073277	-1.38296	0.166676	NA
HSF4	152.2893	-0.10134	0.067444	-1.50256	0.132952	NA
MTHFS	8.830829	0.101331	0.084685	1.196566	0.231476	NA
CCDC121	29.95279	0.1013	0.072376	1.399637	0.161622	NA

SNORD115	2.494345	-0.10127	0.072296	-1.40073	0.161294	NA
EIF2AK2	296.2096	0.10126	0.04606	2.198432	0.027918	NA
SYNJ1	502.748	0.10125	0.042565	2.378698	0.017374	0.227303
CORO1B	46.18512	0.101245	0.063322	1.598884	0.109846	NA
SEC61G	27.61337	-0.10124	0.077668	-1.30353	0.192394	NA
LOC10050	22.92399	0.101205	0.077738	1.301863	0.192963	NA
RRP15	135.3942	0.101197	0.058263	1.736908	0.082403	NA
FAM120C	114.8616	0.101184	0.046926	2.156257	0.031064	NA
N4BP1	266.3791	0.101166	0.042607	2.374426	0.017576	NA
EFCAB4B	3.226807	-0.10117	0.077081	-1.31245	0.189369	NA
ZCCHC16	0.844838	-0.10116	0.05056	-2.00087	0.045407	NA
PCDH12	36.54749	-0.10115	0.084673	-1.19456	0.232259	NA
LOC33989	2.553248	0.101135	0.064828	1.560042	0.11875	NA
TXNL1	237.1498	0.101126	0.05825	1.736075	0.082551	NA
ZNF304	127.6616	0.101116	0.055029	1.837489	0.066138	NA
TDRD7	64.33297	0.1011	0.058449	1.729727	0.083679	NA
NR3C1	648.497	0.101073	0.039677	2.547376	0.010854	0.201504
SMG1	2901.045	0.101071	0.056431	1.79104	0.073287	0.389659
ARFIP1	69.67135	0.101047	0.05726	1.764699	0.077614	NA
C7orf50	59.25523	-0.10104	0.075154	-1.34441	0.178815	NA
EEF2K	522.1596	-0.10103	0.042647	-2.36907	0.017833	0.2295
H2AFV	646.9898	0.101013	0.049798	2.028445	0.042515	0.318294
VPS37B	68.33628	0.101006	0.062286	1.621663	0.104875	NA
NDUFA9	116.2655	0.100977	0.054055	1.868042	0.061756	NA
GDF11	126.5787	0.100969	0.071848	1.405311	0.159929	NA
GPR21	12.1118	-0.10096	0.082456	-1.2244	0.220802	NA
TUBA4A	114.7003	0.100947	0.076264	1.323653	0.185618	NA
OSMR	41.19988	-0.10094	0.083495	-1.20893	0.226688	NA
CEP19	19.64493	0.10093	0.082311	1.226212	0.220119	NA
FNDC4	52.76176	-0.10092	0.065685	-1.53639	0.124442	NA
TAS2R31	26.14393	-0.10089	0.078072	-1.29225	0.196269	NA
MPZ	18.38441	-0.10086	0.080007	-1.2607	0.207417	NA
COX7A1	18.75793	-0.10084	0.083641	-1.20561	0.227969	NA
SP2	105.9676	0.100831	0.055023	1.832516	0.066875	NA
TRPC4	7.315044	-0.10083	0.083221	-1.21153	0.225692	NA
ITGB2-AS1	1.510989	-0.1008	0.062922	-1.60205	0.109146	NA
PBX4	5.422408	-0.10079	0.074458	-1.35366	0.175846	NA
AUH	171.3978	-0.10078	0.049922	-2.01874	0.043515	NA
KIAA1109	2380.784	0.100771	0.030978	3.25292	0.001142	0.092144
SLC2A3	175.1864	0.100767	0.063024	1.598872	0.109849	NA
CPOX	83.81817	-0.10075	0.053142	-1.89582	0.057984	NA
CD63	86.82728	-0.10072	0.074866	-1.3453	0.178529	NA
IMPDH1	70.52467	0.100654	0.083954	1.198907	0.230564	NA
TPPP	867.7721	0.100648	0.056756	1.773338	0.076173	0.392116
CLDN5	50.10641	-0.10063	0.084419	-1.19209	0.233226	NA
WIPF1	27.5434	-0.10062	0.082012	-1.22692	0.219853	NA
MAL2	143.7521	0.100618	0.075891	1.325827	0.184897	NA

MIR3679	0.591959	-0.10061	0.043698	-2.30249	0.021307	NA
ZNF385C	41.92192	-0.1006	0.078595	-1.28002	0.200538	NA
MIR130A	1.518298	-0.1006	0.064307	-1.56441	0.117722	NA
LINGO1	60.84621	0.10059	0.072782	1.382073	0.166949	NA
SMIM15	48.55378	0.100589	0.066317	1.51679	0.12932	NA
LOC54147	1.117241	0.100586	0.053388	1.88406	0.059557	NA
LYPD1	4.094577	0.100565	0.074332	1.352922	0.17608	NA
FBLIM1	4.691962	-0.10056	0.081716	-1.23065	0.218455	NA
AK8	6.355929	-0.10055	0.082379	-1.22061	0.222233	NA
C1orf122	17.9224	-0.10055	0.079746	-1.26089	0.207349	NA
CXCL14	3.900161	0.10055	0.073419	1.369546	0.170829	NA
LINC00693	6.403479	-0.10054	0.084021	-1.19655	0.231481	NA
OAZ3	15.92484	0.100534	0.08109	1.239793	0.215052	NA
PCDH11X	5.925962	0.100515	0.069971	1.436518	0.150855	NA
NR2C2AP	10.51007	0.10051	0.084016	1.196325	0.23157	NA
TBC1D19	45.826	0.1005	0.065869	1.525763	0.127069	NA
PLEKHH1	605.036	-0.10049	0.052521	-1.91338	0.0557	0.3598
MIR613	0.886472	-0.10048	0.050882	-1.97483	0.048288	NA
UPK3B	0.928649	-0.10048	0.048443	-2.07419	0.038061	NA
ROMO1	8.451933	-0.10046	0.08481	-1.18459	0.236181	NA
OAS1	4.027417	-0.10046	0.073528	-1.36629	0.171849	NA
LOC38894	1.211558	-0.10045	0.057093	-1.75944	0.078502	NA
DSC2	9.425229	-0.10044	0.082282	-1.22071	0.222195	NA
PRKX	47.50504	-0.10039	0.083959	-1.19574	0.231797	NA
PPP4R2	135.4523	0.100383	0.060106	1.670111	0.094897	NA
AGPHD1	7.332745	-0.10035	0.084934	-1.18154	0.237386	NA
PDE1A	702.4676	0.100304	0.046433	2.160187	0.030758	0.277495
KIAA1191	208.8355	0.100285	0.05273	1.901875	0.057187	NA
LOC64372	2.409085	-0.10025	0.067616	-1.48267	0.138162	NA
UBE2J1	149.1675	0.10025	0.052336	1.915534	0.055424	NA
KLF5	3.865008	-0.10025	0.074551	-1.34472	0.178716	NA
XKR7	85.66625	0.10025	0.065238	1.536681	0.124371	NA
CNKSR2	1694.856	0.100231	0.032854	3.050796	0.002282	0.120222
MLH3	420.3315	0.100205	0.034732	2.88509	0.003913	0.14186
LOC39219	10.83858	-0.10018	0.082356	-1.21644	0.223819	NA
SLC29A2	103.9066	-0.10015	0.061747	-1.62196	0.104812	NA
PDS5B	809.7541	0.10014	0.046751	2.142008	0.032193	0.279375
TOMM7	117.8303	-0.10014	0.074151	-1.35044	0.176874	NA
GJA1	425.3793	-0.10011	0.083482	-1.19924	0.230435	0.579285
KIAA0408	5.503477	0.100102	0.082749	1.209712	0.226389	NA
C11orf31	49.70613	-0.10006	0.060332	-1.65852	0.097213	NA
OR10A2	0.811389	-0.10006	0.050047	-1.99932	0.045574	NA
KCTD11	22.79927	0.100045	0.076409	1.309328	0.190423	NA
MRPL47	33.88464	-0.10004	0.071128	-1.40647	0.159585	NA
ACTN1	156.6086	-0.10004	0.06697	-1.49378	0.135233	NA
ALG6	41.06183	-0.10004	0.070114	-1.42675	0.153652	NA
PKD1	4119.094	-0.10003	0.055416	-1.80503	0.07107	0.38806

LOC55420	14.90001	-0.09998	0.083786	-1.1933	0.232751	NA
ZFYVE1	86.51151	-0.09998	0.055177	-1.81195	0.069994	NA
GZF1	147.577	0.099965	0.051751	1.931658	0.053402	NA
B4GALT5	184.3312	0.099936	0.049786	2.007301	0.044718	NA
PPP3R1	781.33	0.099927	0.055046	1.815346	0.069471	0.385666
SH3BP5-A	152.3586	-0.09991	0.058049	-1.72106	0.08524	NA
ETFA	95.61992	-0.09989	0.070042	-1.42614	0.153827	NA
TGFA	32.69701	0.099871	0.084475	1.182253	0.237105	NA
NAP1L6	2.780367	-0.09987	0.07124	-1.40183	0.160967	NA
ASRGL1	94.01447	-0.09986	0.050305	-1.98515	0.047128	NA
LEPROTL1	116.2934	0.099854	0.058729	1.70024	0.089086	NA
ZNF33BP1	7.313215	-0.09985	0.077431	-1.28954	0.19721	NA
SCARNA22	7.323626	0.099842	0.082737	1.206729	0.227537	NA
CEP170B	511.5794	0.099841	0.056597	1.764063	0.077721	0.397379
LOC10050	1.656765	0.09984	0.061209	1.631139	0.102861	NA
PRKAR1A	1162.672	0.099828	0.067251	1.484419	0.137698	0.489381
CA12	34.29342	-0.09982	0.082985	-1.20289	0.229019	NA
LOC10050	29.79023	-0.09982	0.074903	-1.33261	0.18266	NA
ARL14EP	59.7485	0.099803	0.061424	1.624808	0.104203	NA
SNORD115	30.07529	-0.09979	0.082349	-1.21181	0.225584	NA
MTMR9	264.1698	0.099791	0.056285	1.772965	0.076235	NA
RNF13	111.2022	-0.09978	0.053659	-1.85958	0.062945	NA
FLRT3	132.5906	0.099779	0.046654	2.13869	0.032461	NA
JPH1	317.788	0.099771	0.052253	1.909403	0.05621	NA
SNORD113	4.164438	-0.09975	0.076896	-1.29726	0.194541	NA
TENM2	63.19473	-0.09974	0.078174	-1.27586	0.202004	NA
SUV420H2	32.95214	-0.09972	0.077231	-1.29124	0.19662	NA
HMMR	1.1574	-0.09972	0.055126	-1.80895	0.070459	NA
LOC44035	6.322144	-0.0997	0.084334	-1.18224	0.237112	NA
SNORA31	4.764856	-0.09969	0.081286	-1.22639	0.22005	NA
RPL23AP6	26.62899	-0.09967	0.078112	-1.27604	0.201941	NA
PDXP	110.2603	0.099657	0.067588	1.474485	0.140351	NA
ARHGAP10	32.40846	-0.09965	0.079195	-1.25835	0.208265	NA
LOC25551	3.516618	-0.09965	0.077251	-1.29	0.19705	NA
LOC10028	42.44628	-0.09965	0.073452	-1.35668	0.174884	NA
ACP6	36.37247	-0.09964	0.081538	-1.22196	0.221721	NA
LOC10013	7.714637	-0.09962	0.084349	-1.18104	0.237586	NA
DLG3	224.776	0.099604	0.048291	2.062563	0.039154	NA
TMEM185	60.1751	0.099592	0.061489	1.619667	0.105304	NA
DBF4	35.87759	-0.09959	0.075973	-1.31086	0.189906	NA
SLC2A11	90.80379	-0.09959	0.062348	-1.59731	0.110197	NA
GTF2A1	532.9437	0.099585	0.045784	2.175102	0.029622	0.274979
NOTCH3	42.9793	0.099575	0.083174	1.197186	0.231234	NA
TFCP2L1	18.48334	-0.09957	0.080737	-1.23323	0.217492	NA
ATE1	165.0997	-0.09957	0.046761	-2.12925	0.033233	NA
NKD2	1.455104	0.099562	0.062461	1.593976	0.110941	NA
SRD5A1	46.00182	0.099548	0.068574	1.451693	0.146587	NA

BTBD17	2.996445	-0.09952	0.073413	-1.35569	0.175198	NA
LPHN1	860.0745	0.099513	0.057402	1.733632	0.082983	0.403119
FAP	1.737109	-0.09951	0.054729	-1.81825	0.069025	NA
EPHB3	68.41474	0.09951	0.068859	1.445125	0.148423	NA
ZNF132	105.371	0.099499	0.060917	1.633358	0.102394	NA
ATXN7L3	159.5108	0.099472	0.057802	1.720905	0.085268	NA
SFRP1	173.4916	0.099456	0.080098	1.241671	0.214358	NA
TFB2M	34.05157	0.099455	0.072675	1.368495	0.171157	NA
FBXW4P1	15.18645	0.099452	0.08404	1.183386	0.236656	NA
LPGAT1	460.0303	0.09945	0.055791	1.782552	0.074659	0.390538
DLAT	114.0291	0.0994	0.054734	1.816071	0.06936	NA
ST20	12.32451	0.099371	0.083714	1.18703	0.235216	NA
GDPD5	82.32413	-0.09937	0.062991	-1.57746	0.11469	NA
GNG11	10.40253	0.099353	0.084712	1.172835	0.240862	NA
SCG3	567.8941	-0.09935	0.078431	-1.26667	0.205274	0.553506
NPTX2	8.609818	-0.09934	0.067967	-1.46166	0.143835	NA
SNORD102	12.0933	-0.09934	0.083874	-1.18441	0.236251	NA
SCGB1D2	0.597947	-0.09931	0.041408	-2.39823	0.016475	NA
LRRCC1	42.28114	-0.09926	0.074691	-1.32894	0.183868	NA
SMG5	360.6662	-0.09922	0.042808	-2.31773	0.020464	0.235785
MAGEF1	150.554	0.099214	0.061922	1.602243	0.109102	NA
ARHGEF1	101.6499	-0.0992	0.066732	-1.48658	0.137126	NA
KIAA1644	65.17583	-0.09919	0.084932	-1.16791	0.242845	NA
TTBK2	493.0953	0.099193	0.039115	2.535952	0.011214	0.201925
SBNO1	933.5266	0.099176	0.03131	3.167533	0.001537	0.101784
MGC2889	3.048582	-0.09917	0.077166	-1.28514	0.198744	NA
TMEM132	11.41799	-0.09916	0.083259	-1.19099	0.233657	NA
C17orf51	669.1396	0.099146	0.063533	1.56056	0.118627	0.472798
DOCK6	38.3011	-0.09912	0.082445	-1.20231	0.229245	NA
SDC2	161.6478	0.099106	0.068423	1.448426	0.147498	NA
MORC2-AS	29.90991	-0.0991	0.078794	-1.25773	0.208489	NA
USP35	72.65404	-0.09909	0.065968	-1.50212	0.133066	NA
AOX1	6.503697	-0.09907	0.082792	-1.19663	0.231451	NA
STRADA	109.843	-0.09907	0.062424	-1.58703	0.112505	NA
KRT222	129.7074	0.099065	0.071128	1.392766	0.163691	NA
TP53I13	6.472065	-0.09906	0.081201	-1.2199	0.222502	NA
AK2	234.1413	-0.09905	0.04704	-2.10569	0.035232	NA
FDFT1	151.9412	-0.09904	0.061041	-1.6226	0.104676	NA
ERI1	74.70591	0.099036	0.058576	1.690715	0.090891	NA
SNORD30	5.598582	-0.09903	0.075799	-1.30654	0.191368	NA
KIAA1549	43.90534	0.099029	0.081049	1.221849	0.221765	NA
GSTM5	24.75085	-0.09901	0.084704	-1.1689	0.242444	NA
FCHSD1	57.81766	-0.099	0.078479	-1.26152	0.207121	NA
COX8A	37.15276	-0.09899	0.081963	-1.20775	0.227142	NA
LOC10050	17.82388	0.098991	0.081869	1.209146	0.226607	NA
YTHDF3	227.6613	0.098962	0.044673	2.215238	0.026744	NA
IFT74	127.0082	-0.09894	0.056697	-1.74502	0.080981	NA

ZFP1	116.3606	0.098934	0.063667	1.553938	0.120199	NA
PTPN22	6.918525	0.098921	0.084348	1.172774	0.240887	NA
HN1	112.9802	0.098917	0.062106	1.59273	0.111221	NA
SMOC1	69.39679	-0.09891	0.079717	-1.24076	0.214693	NA
TMPO-AS1	7.386177	0.098888	0.083265	1.187637	0.234976	NA
VCPIP1	293.4708	0.098885	0.046136	2.143324	0.032087	NA
HELLS	107.8113	-0.09888	0.064636	-1.52979	0.126068	NA
ADH1B	2.573105	-0.09888	0.056907	-1.73756	0.082288	NA
THAP9-AS	113.4381	0.098876	0.075649	1.30704	0.191199	NA
HORMAD1	0.584107	0.098875	0.043297	2.283619	0.022394	NA
SESN1	217.8754	-0.09887	0.043625	-2.26643	0.023425	NA
TTC25	17.259	-0.09885	0.084203	-1.174	0.240394	NA
LOC10050	8.810953	-0.09885	0.084939	-1.16382	0.244496	NA
GMEB2	93.07237	-0.09883	0.052804	-1.87159	0.061264	NA
C5orf45	62.4465	-0.09882	0.068101	-1.45115	0.146737	NA
FCGBP	13.4643	-0.09879	0.064795	-1.52468	0.127339	NA
DNM1P35	32.78025	0.098768	0.082656	1.194925	0.232116	NA
POFUT1	68.21063	0.098744	0.063466	1.555863	0.119741	NA
SIDT1	130.0514	0.098725	0.063194	1.562244	0.118231	NA
THPO	9.693027	0.098724	0.08456	1.167508	0.243005	NA
TSHZ3	19.69719	0.098709	0.084246	1.171678	0.241326	NA
HEG1	102.8148	0.098706	0.082847	1.191415	0.233491	NA
USP1	255.04	0.0987	0.043401	2.274119	0.022959	NA
MED11	11.9428	-0.09869	0.084116	-1.17321	0.240713	NA
TMEM161	74.85341	-0.09866	0.060801	-1.62269	0.104655	NA
MRS2	215.3177	-0.09864	0.040541	-2.43313	0.014969	NA
LOC73081	77.69454	0.098638	0.05904	1.670698	0.094781	NA
ALS2CR11	22.74723	-0.09863	0.081328	-1.21273	0.225232	NA
TXNRD3NB	3.76405	0.098629	0.074795	1.318646	0.187288	NA
HELZ2	13.82683	-0.09862	0.081962	-1.20327	0.228873	NA
CC2D2A	272.2629	-0.09862	0.059438	-1.65923	0.097069	NA
ETHE1	11.59541	0.098615	0.083973	1.174369	0.240247	NA
CASKIN1	372.568	0.098606	0.052197	1.88912	0.058876	0.36517
MIR4677	3.008004	0.098597	0.074067	1.33118	0.18313	NA
DNLZ	12.17077	0.098588	0.084799	1.16261	0.244988	NA
CPO	2.287267	-0.09856	0.071312	-1.38212	0.166934	NA
PPARG	13.67471	0.098547	0.084494	1.166322	0.243484	NA
SNTB1	30.27751	-0.09853	0.073281	-1.34455	0.178772	NA
MIR657	2.391147	-0.09853	0.068546	-1.43741	0.150603	NA
JAK2	273.0599	0.098518	0.038533	2.55674	0.010566	NA
EYA1	2.623481	0.09851	0.071231	1.382965	0.166675	NA
NDUFV2	116.4362	-0.09848	0.070135	-1.40414	0.160277	NA
FZR1	366.6284	-0.09847	0.058005	-1.69755	0.089594	0.415114
PJA2	1338.871	0.098458	0.051477	1.912637	0.055795	0.3598
FXR2	203.1234	0.098446	0.048228	2.041274	0.041224	NA
TGOLN2	1121.833	0.098423	0.033629	2.926705	0.003426	0.138559
RNH1	100.8202	0.098384	0.05418	1.815874	0.06939	NA

FAM86DP	21.58222	-0.09835	0.084384	-1.16548	0.243825	NA
LINC00319	2.270188	-0.09835	0.072383	-1.3587	0.174241	NA
ALOX5AP	3.171565	-0.09835	0.063929	-1.53836	0.123961	NA
HCK	5.115148	-0.09832	0.080928	-1.21495	0.224384	NA
PLS1	27.44241	0.098322	0.083724	1.174361	0.24025	NA
ZNF672	92.88596	0.098289	0.053234	1.846351	0.064841	NA
EAF1	130.3626	0.098257	0.062715	1.56672	0.11718	NA
SULT1C4	18.49337	-0.09825	0.084502	-1.16275	0.244932	NA
PXN	117.9916	-0.09822	0.069072	-1.42206	0.15501	NA
TGFB2	79.78817	-0.09822	0.080559	-1.21927	0.222741	NA
FAM50B	52.97207	0.098189	0.06609	1.485678	0.137364	NA
THNSL2	11.20408	-0.09819	0.08063	-1.21774	0.223324	NA
GPR115	5.405746	0.098165	0.078794	1.245837	0.212824	NA
DAPP1	1.305282	-0.09815	0.05817	-1.68722	0.09156	NA
LOC72855	7.640341	-0.09813	0.084677	-1.15885	0.246516	NA
KLF15	90.61691	-0.09812	0.078935	-1.24305	0.213851	NA
S100A7	1.194444	0.098109	0.051945	1.888715	0.05893	NA
LCT	4.367406	0.098099	0.076117	1.288791	0.197471	NA
KLHL33	4.629207	-0.09809	0.080957	-1.21167	0.225639	NA
MYOZ2	1.047511	-0.09808	0.055309	-1.77336	0.07617	NA
ZNF669	44.93354	0.098078	0.069535	1.410472	0.1584	NA
CLSTN1	2411.388	0.098073	0.050144	1.955827	0.050486	0.34688
ARRDC3	356.6397	-0.09806	0.060493	-1.621	0.105017	0.448055
MORF4L2-	13.66675	-0.09806	0.08348	-1.17462	0.240145	NA
RNF157-A	19.1582	-0.09805	0.084772	-1.15658	0.247443	NA
C1orf141	0.463236	-0.09803	0.037214	-2.63431	0.008431	NA
BFSP1	12.00167	0.098021	0.084389	1.161543	0.245421	NA
CTSO	28.5211	-0.09801	0.08363	-1.17199	0.241202	NA
SEMA3E	31.63626	-0.098	0.084641	-1.15781	0.246944	NA
DUSP26	102.3321	-0.09798	0.057149	-1.71449	0.086439	NA
EDA2R	2.556722	0.097975	0.071155	1.376938	0.168532	NA
NXPE4	4.853193	0.097962	0.074961	1.306844	0.191266	NA
WASH1	19.08538	0.097954	0.083429	1.174107	0.240352	NA
PACRG	5.575607	0.097936	0.083705	1.170004	0.241999	NA
SNORA76	1.95011	0.09791	0.065901	1.485717	0.137354	NA
B2M	227.483	-0.09791	0.082161	-1.19165	0.233398	NA
FAM108A1	64.68061	-0.0979	0.063552	-1.54053	0.123432	NA
COX4I2	1.200871	0.097869	0.055516	1.762888	0.077919	NA
LOC10028	1.145665	0.097856	0.057446	1.70344	0.088486	NA
POLR3F	127.0615	-0.09786	0.052517	-1.8633	0.062421	NA
CISD3	28.01951	0.097839	0.077662	1.259796	0.207743	NA
AHNAK	1499.937	-0.09782	0.076718	-1.27502	0.202302	0.549418
TDRD3	120.49	0.097804	0.058299	1.677627	0.09342	NA
CDK17	353.746	0.097801	0.042051	2.325774	0.020031	0.235785
KRT34	0.928796	0.097797	0.048504	2.016255	0.043773	NA
ITPKC	54.67423	-0.09777	0.067858	-1.44083	0.149633	NA
CD4	16.23435	-0.09776	0.08477	-1.15329	0.248791	NA

NDUFS4	106.7738	-0.09776	0.073005	-1.33911	0.180534	NA
MLANA	23.25404	0.097758	0.082817	1.180406	0.237839	NA
PLEKHB1	425.3384	-0.09776	0.071519	-1.36685	0.171673	0.525733
ARAP2	219.7495	-0.09773	0.063786	-1.53217	0.12548	NA
CENPT	252.1367	-0.09771	0.052911	-1.8467	0.064791	NA
C15orf41	35.55766	0.097705	0.067416	1.449294	0.147255	NA
SBK2	1.300737	0.097705	0.052897	1.847086	0.064735	NA
C16orf70	49.52249	0.097702	0.063954	1.5277	0.126587	NA
ATPAF2	67.67161	-0.09769	0.056457	-1.73041	0.083557	NA
C11orf83	20.83621	0.097688	0.080749	1.209765	0.226369	NA
PARD6B	63.0371	0.097674	0.057448	1.700203	0.089093	NA
NOP16	25.37237	0.09767	0.078144	1.24987	0.211347	NA
GNAZ	421.5917	0.097641	0.057304	1.703907	0.088399	0.41454
PCDHA9	112.6632	0.097611	0.082447	1.183931	0.236441	NA
C2orf82	5.005368	-0.09759	0.078463	-1.24382	0.213565	NA
CCDC11	85.36218	-0.09758	0.071701	-1.36091	0.173543	NA
SNORD116	119.878	-0.09757	0.083066	-1.17459	0.240158	NA
HIST1H4C	135.9975	-0.09753	0.084668	-1.15187	0.249376	NA
RAD9B	5.036764	0.097524	0.08328	1.171046	0.24158	NA
APEX1	103.1434	-0.0975	0.069752	-1.39785	0.162158	NA
CROCCP3	608.9235	-0.09745	0.057237	-1.70252	0.088657	0.414895
LAMC1	273.0751	0.097441	0.057724	1.688051	0.091401	NA
SLC25A27	760.8912	-0.09744	0.039114	-2.49106	0.012736	0.205975
LOC10050	2.992128	-0.09742	0.073976	-1.31692	0.187867	NA
PCBP4	318.5694	-0.09741	0.067634	-1.44019	0.149812	NA
NID1	42.29281	-0.0974	0.07686	-1.26726	0.205061	NA
SLC35E1	165.0944	0.097402	0.053603	1.817099	0.069202	NA
CTDNEP1	110.0051	0.097401	0.053068	1.835398	0.066447	NA
ZNF224	22.43621	0.097399	0.077069	1.263788	0.206306	NA
LOC10037	13.40974	0.097398	0.083715	1.163447	0.244648	NA
PDCD5	68.04612	-0.09739	0.062055	-1.56938	0.116559	NA
SRCRB4D	3.683155	0.097366	0.077653	1.253867	0.20989	NA
ABI3	7.727725	-0.09736	0.081197	-1.19906	0.230503	NA
HAPLN1	1.587072	0.097345	0.055097	1.766782	0.077265	NA
LOC25303	78.63353	-0.09734	0.08213	-1.18523	0.235925	NA
WRAP53	13.64641	0.097341	0.082024	1.186735	0.235332	NA
CUTA	57.14411	-0.09731	0.066636	-1.46028	0.144214	NA
NDUFS2	315.6792	-0.09726	0.050207	-1.93725	0.052715	NA
TMPRSS5	15.37669	-0.09726	0.084254	-1.15437	0.248349	NA
FRS2	323.5129	0.097247	0.044638	2.178587	0.029362	NA
TMEM60	9.727864	-0.09723	0.084862	-1.14574	0.251901	NA
LNPEP	562.3676	0.097222	0.036632	2.654034	0.007954	0.171303
RAP2A	187.4287	0.09722	0.048397	2.008788	0.04456	NA
ZNF610	65.8282	0.097211	0.077342	1.256898	0.20879	NA
ZFP91	267.3999	0.097202	0.049914	1.94737	0.05149	NA
MEGF8	547.92	0.09718	0.040065	2.425533	0.015286	0.216391
ADRBK1	479.8421	0.097166	0.052177	1.862235	0.06257	0.37053

SUZ12P1	177.2279	-0.09715	0.053129	-1.82856	0.067465	NA
CACNG1	0.540696	-0.09714	0.040171	-2.41805	0.015604	NA
IFI44	13.61629	-0.09713	0.084925	-1.14377	0.25272	NA
FIZ1	28.31449	0.097131	0.073399	1.323327	0.185727	NA
PKIA	354.7659	0.097127	0.068101	1.426211	0.153807	0.506644
MAT2B	169.804	-0.09713	0.072644	-1.337	0.181223	NA
FYTTD1	289.5207	0.097124	0.039055	2.486852	0.012888	NA
CHRNB3	12.49755	0.097121	0.071806	1.35255	0.176199	NA
DAPK3	101.1194	0.097112	0.057111	1.700415	0.089053	NA
MIR661	0.925475	-0.09711	0.051038	-1.90269	0.057081	NA
NRXN2	1626.839	0.097106	0.054713	1.774841	0.075924	0.391931
PTPRS	1036.933	0.097091	0.062161	1.561923	0.118306	0.472599
SNORA8	20.50313	-0.09708	0.084709	-1.14604	0.251778	NA
DEPDC1B	3.180125	-0.09707	0.074145	-1.30918	0.190472	NA
ABCA5	1247.13	-0.09706	0.048025	-2.02093	0.043287	0.321461
FAM66C	76.00431	-0.09703	0.061546	-1.57653	0.114904	NA
SSUH2	0.692145	-0.09703	0.047414	-2.04636	0.040721	NA
LIN28A	2.090204	-0.09701	0.06185	-1.5685	0.116765	NA
OXER1	12.27855	-0.09701	0.083249	-1.16524	0.243923	NA
ANP32A-IT	53.17346	-0.09696	0.069585	-1.39337	0.163508	NA
NES	141.3148	0.096954	0.082822	1.170639	0.241744	NA
C8orf56	1.300129	-0.09693	0.061123	-1.58575	0.112796	NA
PIDD	53.63259	-0.09692	0.076616	-1.265	0.20587	NA
PCDH19	10.76888	0.096903	0.084879	1.141668	0.253592	NA
LINC00657	1187.438	0.09689	0.052673	1.839482	0.065844	0.375222
PRIM1	58.42517	-0.09689	0.070492	-1.37447	0.169296	NA
YPEL4	152.6164	-0.09688	0.07256	-1.33524	0.181798	NA
RAMP2-AS	4.870257	-0.09687	0.079966	-1.21136	0.225759	NA
FARS2	54.45027	0.096865	0.066264	1.461796	0.143797	NA
IL1RL1	26.18681	-0.09686	0.045411	-2.13295	0.032929	NA
SPIN2B	7.539022	-0.09686	0.084939	-1.1403	0.254163	NA
MORF4L2	326.6736	0.096827	0.048984	1.976706	0.048075	NA
WNT11	4.048694	-0.09681	0.079799	-1.21319	0.225056	NA
AGAP11	3.078727	-0.09681	0.076051	-1.27293	0.203044	NA
ZNF90	29.87774	-0.09679	0.077452	-1.24969	0.211413	NA
STARD7	318.6403	0.096766	0.043785	2.210017	0.027104	NA
CYSTM1	23.1777	0.096765	0.076957	1.257395	0.20861	NA
NPIP	534.348	-0.09676	0.0603	-1.60461	0.108579	0.452973
BTBD8	40.47589	-0.09676	0.074276	-1.30267	0.192689	NA
FAM71F1	0.575797	-0.09675	0.044041	-2.19677	0.028037	NA
ERCC3	229.8605	0.096744	0.042638	2.268946	0.023272	NA
ZNF559-ZN	4.844107	-0.09674	0.082283	-1.17567	0.239728	NA
ADCY2	172.3935	-0.09673	0.080208	-1.20604	0.227801	NA
KIF13B	136.0316	-0.09673	0.060525	-1.59818	0.110003	NA
TTPA	17.33948	0.096718	0.084426	1.145606	0.251958	NA
FAM108C1	21.3379	0.096708	0.081904	1.180752	0.237701	NA
GABARAPL	8.20277	0.096692	0.081758	1.18267	0.23694	NA

OR1L4	1.297999	-0.09668	0.056893	-1.69932	0.089259	NA
DUOX1	337.7449	-0.09668	0.082361	-1.1738	0.240474	0.589893
CASC1	16.46727	-0.09665	0.080933	-1.19413	0.232425	NA
CISD2	48.06241	0.096634	0.067928	1.42258	0.154858	NA
NINJ2	1.819369	-0.09662	0.066111	-1.4615	0.143879	NA
TAF6L	33.60626	-0.09662	0.067826	-1.42451	0.1543	NA
WDR82	366.9334	0.096616	0.053549	1.804248	0.071193	0.38806
DYSF	42.12941	-0.09661	0.076777	-1.25838	0.208254	NA
DSCAM-IT	3.416553	-0.09658	0.076871	-1.25644	0.208956	NA
BCAP29	224.5622	0.096577	0.061825	1.562111	0.118262	NA
PENK	58.53351	0.096576	0.072841	1.325858	0.184887	NA
ABCA3	579.3106	0.096569	0.050393	1.916308	0.055326	0.359063
RELT	36.08557	-0.09656	0.070625	-1.36725	0.171546	NA
MAP3K3	213.879	-0.09654	0.056032	-1.723	0.084889	NA
REEP5	554.1203	0.09653	0.065902	1.464751	0.142989	0.495647
ZC2HC1C	14.46546	-0.09652	0.084237	-1.14585	0.251859	NA
ITGB7	13.37019	-0.09652	0.083998	-1.14909	0.250517	NA
TUSC1	25.34277	0.096518	0.078624	1.227588	0.219602	NA
HEATR6	167.0664	0.096506	0.071913	1.341986	0.1796	NA
COL10A1	20.37696	0.096503	0.082804	1.165442	0.24384	NA
DUSP2	3.299062	0.096503	0.073791	1.307795	0.190943	NA
LINC00094	64.03864	0.096496	0.061647	1.565314	0.117509	NA
SLC35F6	80.83324	0.096492	0.055327	1.744023	0.081155	NA
LOC64223	13.16223	-0.09644	0.083613	-1.15345	0.248725	NA
C14orf119	17.50398	-0.09643	0.08348	-1.15518	0.248016	NA
DBNDD1	67.05756	0.096384	0.068673	1.403517	0.160463	NA
PITRM1-A	51.95251	-0.09637	0.072686	-1.32587	0.184882	NA
SERPIND1	37.6925	0.09636	0.074888	1.286718	0.198192	NA
UTP3	90.84198	0.096351	0.0558	1.726728	0.084217	NA
PYGB	572.8088	-0.09632	0.051271	-1.87864	0.060294	0.367348
JHDM1D	303.7691	0.096304	0.047012	2.048487	0.040512	NA
RGMA	81.97912	0.096292	0.082038	1.173749	0.240496	NA
CAPRIN1	647.3256	0.096282	0.051784	1.85932	0.062982	0.37053
SLC5A5	8.642811	0.096265	0.071718	1.342263	0.179511	NA
FAM154B	11.42758	-0.09626	0.083063	-1.1589	0.246495	NA
BMP8A	47.64955	-0.09626	0.07153	-1.34569	0.178403	NA
SSH3	133.4157	-0.09626	0.065021	-1.48039	0.13877	NA
C7	11.33952	-0.09626	0.072542	-1.3269	0.184541	NA
ABLIM2	110.9161	0.09625	0.057608	1.670788	0.094764	NA
ZBTB43	217.332	0.09625	0.043774	2.198799	0.027892	NA
GNPTG	114.8325	-0.09624	0.048309	-1.99212	0.046358	NA
HNRNPAB	149.7574	0.096217	0.058768	1.637216	0.101585	NA
NXF5	2.897957	-0.09622	0.070493	-1.36491	0.172281	NA
SLC9A8	94.22117	-0.09621	0.078482	-1.22584	0.220258	NA
MYLK3	3.356184	0.096206	0.07133	1.348745	0.177419	NA
C9orf170	3.012269	-0.0962	0.075722	-1.27037	0.203951	NA
LAMP5	14.099	0.096193	0.084904	1.132965	0.257229	NA

NKAIN4	22.58964	-0.09619	0.084731	-1.13521	0.256287	NA
SEMA6C	196.6915	-0.09618	0.066161	-1.45377	0.146009	NA
HLA-H	25.26914	0.096162	0.083313	1.154227	0.248407	NA
EXOC3L1	10.96092	-0.09616	0.084896	-1.13263	0.25737	NA
RUSC1-AS	8.654166	-0.09612	0.084902	-1.1321	0.257591	NA
PRR24	14.922	-0.09612	0.082436	-1.16594	0.243638	NA
ZNF425	26.21121	0.096107	0.084318	1.139815	0.254363	NA
APLN	12.91797	-0.09611	0.083399	-1.15237	0.249168	NA
SLC25A4	452.6997	0.096102	0.063219	1.520145	0.128475	0.48338
SURF6	162.1887	0.096098	0.052045	1.846439	0.064828	NA
CD247	6.048141	-0.09609	0.082165	-1.16951	0.242197	NA
ZNF442	46.0417	0.096092	0.072957	1.31711	0.187802	NA
NNMT	2.858168	-0.09608	0.072464	-1.32595	0.184856	NA
LOC28317	2.237035	-0.09608	0.066442	-1.44604	0.148167	NA
ANKFY1	553.9048	-0.09607	0.043601	-2.20331	0.027573	0.263821
PAQR6	89.30807	-0.09606	0.076521	-1.25532	0.209361	NA
HEYL	15.62263	-0.09606	0.083554	-1.14962	0.2503	NA
OLFML1	17.45751	0.096026	0.084881	1.131308	0.257925	NA
COL4A1	51.10455	-0.09602	0.080955	-1.18614	0.235568	NA
ZNF483	1601.114	0.096018	0.039205	2.449151	0.014319	0.212327
ZNF300	41.5099	0.09601	0.071373	1.345174	0.178569	NA
CD160	28.94166	-0.096	0.082153	-1.1686	0.242566	NA
GRIN2A	711.0187	0.096003	0.048066	1.997306	0.045792	0.334577
SOCS1	11.95575	0.096	0.084854	1.131357	0.257905	NA
CXorf40B	25.59914	-0.096	0.074571	-1.28732	0.197984	NA
IL1RAP	10.30547	-0.09599	0.084279	-1.13897	0.254715	NA
AGAP1	758.7865	0.095945	0.038897	2.466638	0.013639	0.208729
AS3MT	3.717257	0.095939	0.07729	1.241292	0.214498	NA
ZNF174	39.86077	0.095934	0.066033	1.452813	0.146276	NA
RAB7A	518.8839	0.095926	0.061322	1.56431	0.117745	0.472599
S100A8	3.925949	-0.09592	0.046379	-2.06812	0.038629	NA
SHC2	43.35804	-0.09591	0.082796	-1.15833	0.246728	NA
ZSCAN23	75.50868	-0.09588	0.065693	-1.45949	0.14443	NA
MLLT10P1	42.00224	0.09585	0.084817	1.130084	0.258441	NA
LTK	5.889736	-0.09584	0.080593	-1.18915	0.234381	NA
UQCR11	65.13143	-0.09582	0.076029	-1.26028	0.20757	NA
SPECC1L	1333.383	0.095814	0.051606	1.856648	0.063361	0.371619
CD44	141.0074	-0.09581	0.079566	-1.20411	0.228549	NA
EIF3I	87.05413	-0.0958	0.06917	-1.38498	0.166058	NA
TTC30A	16.67386	-0.09579	0.084896	-1.12835	0.259171	NA
UBL7	43.92734	0.09579	0.069862	1.371131	0.170334	NA
FAM126B	623.087	0.095788	0.042123	2.274002	0.022966	0.23963
ZFP2	42.50141	0.095786	0.073013	1.311895	0.189556	NA
DPYSL4	76.48042	0.095768	0.069716	1.373698	0.169535	NA
SUSD2	10.5942	0.095755	0.079855	1.199111	0.230485	NA
PPP1R14A	25.24725	-0.09574	0.083965	-1.1402	0.254202	NA
CDCA4	18.51926	0.095737	0.080662	1.186898	0.235268	NA

CYP21A2	2.502116	-0.09573	0.068475	-1.39799	0.162118	NA
SNORD114	5.50952	0.095704	0.081162	1.179168	0.238331	NA
MBLAC2	100.4323	0.095703	0.071917	1.330751	0.183271	NA
MIR4656	1.16469	-0.0957	0.057338	-1.66901	0.095116	NA
PATE2	6.874823	-0.09569	0.084091	-1.13798	0.25513	NA
BAIAP2	249.4717	-0.09568	0.046415	-2.06148	0.039257	NA
NUCB1	255.4232	-0.09565	0.048168	-1.98574	0.047063	NA
C11orf49	104.1796	-0.09565	0.052006	-1.8391	0.0659	NA
NETO2	69.55297	0.095636	0.079575	1.201833	0.229428	NA
HIAT1	97.66545	0.095572	0.05468	1.747838	0.080492	NA
PLA2G3	8.468778	0.095571	0.083514	1.144372	0.252469	NA
CDC6	10.6316	0.095561	0.084738	1.127733	0.259433	NA
C11orf96	18.04608	-0.09555	0.080821	-1.18229	0.23709	NA
SLC13A5	9.151005	-0.09555	0.081707	-1.16937	0.242255	NA
TATDN1	50.07451	-0.09554	0.067771	-1.40976	0.158609	NA
ST7-AS2	20.41591	-0.09554	0.079476	-1.20208	0.229332	NA
RNF152	125.1493	0.095516	0.067993	1.404795	0.160082	NA
UNC13A	1300.308	0.095502	0.063559	1.502571	0.13295	0.487416
TAB1	131.7916	-0.0955	0.067835	-1.40781	0.159187	NA
SNORD114	1.507575	-0.09545	0.056245	-1.69706	0.089685	NA
CPD	343.3556	0.095431	0.04015	2.376839	0.017462	0.227303
RPL30	160.8535	-0.09543	0.075779	-1.25925	0.20794	NA
PDXDC2P	157.1225	-0.09542	0.066742	-1.42972	0.152798	NA
ZBTB38	938.3051	0.095417	0.04693	2.033188	0.042033	0.317353
KCNE3	2.239797	-0.09542	0.071072	-1.34252	0.179427	NA
C16orf88	103.2927	-0.0954	0.074545	-1.27977	0.200626	NA
MFI2	18.8088	-0.09539	0.078849	-1.20978	0.226363	NA
KPNA3	173.1435	0.095366	0.054696	1.743564	0.081235	NA
TBC1D10C	9.466883	-0.09537	0.084288	-1.13142	0.257877	NA
TRAPPC6A	7.282463	-0.09534	0.084484	-1.12851	0.259106	NA
SNORD115	16.74719	-0.09534	0.084853	-1.12355	0.261206	NA
ZNF675	101.8493	0.095277	0.052648	1.809711	0.070341	NA
SLC43A3	14.0364	-0.09522	0.084675	-1.12459	0.260761	NA
HCN4	9.557777	0.095191	0.083908	1.134473	0.256596	NA
COX6A1	44.47056	-0.09513	0.084356	-1.12777	0.259418	NA
CYB5B	100.7868	-0.09513	0.053122	-1.79076	0.073331	NA
HECTD1	2060.454	0.095112	0.040082	2.372951	0.017647	0.228398
RP2	28.72985	-0.0951	0.071137	-1.33688	0.181261	NA
SCNN1D	51.64082	-0.09509	0.073471	-1.29421	0.195593	NA
TNS1	237.972	-0.09508	0.066367	-1.43266	0.151956	NA
PDGFRL	10.12801	0.095072	0.084259	1.128338	0.259177	NA
ADRM1	158.9445	0.095034	0.052243	1.819069	0.068901	NA
CRHR2	4.645877	-0.09503	0.077458	-1.22691	0.219857	NA
PPM1L	218.7486	0.095024	0.056978	1.667738	0.095368	NA
CARD9	19.70334	0.09502	0.084788	1.120676	0.262426	NA
ARSI	0.882987	-0.095	0.051947	-1.8288	0.067429	NA
SNORD92	3.920716	-0.09499	0.075984	-1.25017	0.211238	NA

FANCM	102.4232	-0.09499	0.067182	-1.41391	0.157389	NA
SCAND2P	218.7096	-0.09498	0.055871	-1.69998	0.089134	NA
GPR87	13.83183	-0.09498	0.08382	-1.13309	0.257175	NA
RTN2	89.34104	-0.09498	0.060848	-1.56085	0.11856	NA
LGALSL	89.2557	0.09497	0.073648	1.289517	0.197218	NA
PTRF	118.8861	0.09496	0.065406	1.451849	0.146544	NA
MEGF10	121.949	-0.09496	0.082764	-1.14736	0.251235	NA
HHAT	15.91464	-0.09495	0.083182	-1.14151	0.253659	NA
SEMA5B	3.493921	0.094945	0.076003	1.249232	0.21158	NA
PLK5	97.37992	-0.09494	0.081636	-1.16291	0.244864	NA
RNF216P1	91.62856	0.09491	0.065299	1.453465	0.146095	NA
MAPKAPK	11.73496	-0.09491	0.084434	-1.12407	0.260983	NA
TRIB1	42.61977	0.0949	0.080576	1.177769	0.238889	NA
MRVI1	126.1899	-0.09489	0.084637	-1.12118	0.262209	NA
MMP25	30.47946	-0.09489	0.0768	-1.23559	0.216609	NA
ZNF595	138.6421	0.094889	0.055866	1.69852	0.08941	NA
LOC10013	1.250195	-0.09489	0.057505	-1.6501	0.098923	NA
LOC10028	41.30738	0.094888	0.081668	1.161877	0.245286	NA
MLLT11	230.3737	0.094887	0.065266	1.45384	0.145991	NA
MED19	33.49093	-0.09485	0.071366	-1.32908	0.183823	NA
BBS7	183.9725	0.094848	0.040386	2.348561	0.018846	NA
RNF219	99.52607	0.094838	0.05608	1.691126	0.090813	NA
DMD	495.638	-0.09483	0.060877	-1.55775	0.119292	0.472798
HEBP2	43.66376	-0.09483	0.077664	-1.22099	0.22209	NA
PCSK1	12.54972	-0.09482	0.082396	-1.15076	0.249831	NA
ZNF473	67.47773	0.094785	0.069037	1.372959	0.169765	NA
AP3M2	208.0025	0.094779	0.043449	2.181399	0.029154	NA
PRAF2	70.53752	0.09476	0.067755	1.398558	0.161946	NA
KIF2A	367.1364	0.09475	0.041579	2.278798	0.022679	0.23963
SNORD83A	1.639073	-0.09474	0.061937	-1.52961	0.126113	NA
ILDR2	79.93947	0.094731	0.073478	1.289252	0.19731	NA
RPL23	220.8601	-0.09466	0.071155	-1.33037	0.183396	NA
DNA2	62.79294	0.094634	0.08467	1.11769	0.2637	NA
CLCN1	10.05249	-0.0946	0.08242	-1.14783	0.25104	NA
KIAA0947	657.5347	0.0946	0.036681	2.578984	0.009909	0.193485
HSPH1	249.0497	0.094565	0.078233	1.208763	0.226754	NA
AGAP2	1042.105	-0.09456	0.06956	-1.35945	0.174006	0.526198
SNORD114	7.82518	-0.09456	0.083879	-1.12734	0.259597	NA
CHD3	532.1093	0.094555	0.063168	1.496896	0.13442	0.487416
LSM7	32.45419	0.094554	0.074982	1.261021	0.207301	NA
HSPD1	216.8922	0.094545	0.061939	1.526418	0.126906	NA
TUBE1	251.8083	-0.09454	0.056218	-1.68166	0.092635	NA
PCDHGA10	121.4597	0.094534	0.081208	1.164103	0.244382	NA
NPHP4	68.33616	-0.09453	0.075803	-1.24703	0.212386	NA
PTPLB	55.82139	0.094526	0.068948	1.370986	0.170379	NA
TESK1	115.3841	0.094523	0.051163	1.847504	0.064674	NA
DPY19L1P	40.55496	0.094491	0.075785	1.246826	0.212461	NA

RCAN2	232.9388	0.094475	0.074926	1.260905	0.207343	NA
SLC4A9	8.668925	0.094459	0.084355	1.119775	0.26281	NA
EFHB	2.596192	-0.09445	0.060896	-1.55108	0.120883	NA
AFF3	776.8776	0.094434	0.040257	2.34577	0.018988	0.231397
TNFRSF12	1.750121	0.094433	0.066249	1.42542	0.154036	NA
LY6E	92.46483	0.094429	0.068841	1.371707	0.170155	NA
LINC00265	58.68414	-0.09443	0.063847	-1.47896	0.13915	NA
LRRK2	111.6765	-0.0944	0.07157	-1.31899	0.187172	NA
CTSW	6.239416	-0.09438	0.08367	-1.12802	0.259311	NA
EPB41L4B	180.3732	0.094381	0.0713	1.323711	0.185599	NA
ZC3HAV1	33.05138	-0.09436	0.083592	-1.12886	0.258958	NA
OSTM1	86.87627	0.094359	0.068545	1.376603	0.168635	NA
CACNA1G	330.4814	-0.09435	0.08393	-1.12412	0.260963	NA
PLEK	5.72411	-0.09434	0.081744	-1.15405	0.24848	NA
ZYG11B	528.0045	0.094325	0.049776	1.894991	0.058094	0.364493
MRC2	10.35577	-0.09428	0.084906	-1.11045	0.266804	NA
CD59	441.7926	-0.09428	0.062766	-1.50214	0.13306	0.487416
SIPA1	17.47142	-0.09427	0.082741	-1.13937	0.254548	NA
JRKL	54.38975	0.094254	0.06447	1.461995	0.143743	NA
CHADL	23.81642	-0.09425	0.080071	-1.17703	0.239182	NA
LRRN3	112.7985	0.094232	0.05362	1.757417	0.078847	NA
AP1AR	132.8662	0.094228	0.058825	1.601852	0.109188	NA
PEX14	61.47365	-0.09422	0.060716	-1.55176	0.12072	NA
RPS2	89.16026	-0.09419	0.068107	-1.38289	0.166697	NA
LMO2	25.9681	-0.09418	0.084939	-1.10876	0.267536	NA
TRAPPC2L	33.86896	-0.09417	0.082401	-1.14281	0.253116	NA
SYNGR1	1189.311	0.094168	0.047049	2.001494	0.045339	0.333419
PINLYP	16.08536	-0.09417	0.082943	-1.1353	0.256248	NA
C6orf132	4.141403	0.094166	0.069855	1.34802	0.177652	NA
SPINK13	2.805251	0.094161	0.067991	1.384895	0.166085	NA
ZNF574	58.20969	0.094158	0.065259	1.442831	0.149068	NA
AMZ1	30.80641	-0.09415	0.077284	-1.21823	0.223136	NA
CRB2	47.00125	-0.09414	0.075494	-1.24694	0.212419	NA
AGXT2L1	134.8397	-0.09414	0.084724	-1.1111	0.266526	NA
KPNA5	206.6033	0.09413	0.047954	1.962934	0.049654	NA
MIR3685	5.212913	0.09413	0.082033	1.147462	0.251191	NA
C8orf42	94.16129	0.094128	0.059136	1.591717	0.111448	NA
AUNIP	9.476965	0.094128	0.08491	1.108565	0.267618	NA
CCDC117	140.2584	0.094121	0.067256	1.399444	0.16168	NA
AP4E1	184.998	0.094112	0.04523	2.08073	0.037459	NA
AHSA1	214.5957	0.094106	0.080325	1.171564	0.241372	NA
GRID1	67.34864	-0.0941	0.064368	-1.46186	0.143779	NA
FLJ44635	43.70924	0.094093	0.069157	1.360562	0.173652	NA
SLC1A1	133.8962	0.094091	0.062662	1.50155	0.133213	NA
GNG4	50.57873	0.094076	0.084654	1.111303	0.266438	NA
STARD13-	4.615336	-0.09406	0.080153	-1.17352	0.240589	NA
GIF	7.850494	0.094038	0.082321	1.142325	0.253319	NA

DGKE	339.9271	0.094024	0.045386	2.071625	0.0383	0.300499
ANXA1	18.09632	-0.09402	0.078786	-1.19336	0.232728	NA
UST	109.2437	0.094016	0.080211	1.172109	0.241153	NA
CHORDC1	247.472	0.094013	0.084506	1.112508	0.26592	NA
MRGPRF	37.64916	0.094012	0.074719	1.258204	0.208318	NA
ITFG2	126.2755	-0.09401	0.052495	-1.7908	0.073326	NA
ARHGAP21	3501.873	-0.094	0.051432	-1.82772	0.067592	0.378951
LOC10050	62.44309	-0.094	0.060911	-1.54328	0.122763	NA
HPRT1	122.8181	0.093985	0.066612	1.41093	0.158265	NA
UBOX5	45.24192	-0.09397	0.065078	-1.44396	0.148751	NA
SFR1	15.31296	-0.09396	0.080272	-1.17056	0.241775	NA
MIR125B1	0.755255	-0.09395	0.047261	-1.98791	0.046822	NA
SNAP91	749.9061	0.093949	0.049549	1.896067	0.057951	0.364493
NCK1	71.20564	0.093949	0.058745	1.599259	0.109763	NA
HSPA2	96.81389	-0.09394	0.084807	-1.1077	0.267991	NA
C11orf57	142.981	0.093939	0.047477	1.978594	0.047862	NA
ZNF345	50.48563	-0.09391	0.067734	-1.38641	0.165621	NA
SNORD114	2.901658	-0.09389	0.072959	-1.28694	0.198116	NA
CCDC58	12.16997	0.093887	0.084631	1.109361	0.267274	NA
LOC10013	69.47246	-0.09387	0.07813	-1.20148	0.229566	NA
CCT2	187.7258	0.093866	0.056223	1.66954	0.095011	NA
TPM4	884.3616	0.093849	0.060332	1.555542	0.119817	0.472798
ACP5	3.992931	-0.09385	0.076921	-1.22003	0.222452	NA
BBOX1	15.22878	-0.09381	0.084818	-1.10597	0.268738	NA
SMG7	554.333	0.093802	0.034559	2.71427	0.006642	0.167162
TLCD2	9.038228	0.093802	0.084046	1.116075	0.26439	NA
EPM2A	60.15465	0.093795	0.068223	1.374826	0.169185	NA
PMEPA1	31.7263	0.093794	0.078746	1.191097	0.233615	NA
ARL5B	105.0705	0.09378	0.052967	1.770542	0.076637	NA
STARD9	833.8193	-0.09376	0.066612	-1.40759	0.159252	0.51603
RNF216-IT	4.08777	-0.09376	0.078323	-1.19711	0.231264	NA
NPAP1	1.573782	-0.09374	0.054587	-1.71727	0.08593	NA
SRCAP	1072.233	0.09374	0.048815	1.920302	0.05482	0.35783
PDDC1	191.5966	-0.09373	0.039008	-2.40278	0.016271	NA
ZNF285	51.17286	0.093702	0.067293	1.392448	0.163787	NA
BAK1	15.08351	-0.0937	0.078901	-1.18753	0.235018	NA
SGCZ	47.88161	-0.09369	0.076087	-1.23137	0.218185	NA
LCP1	34.96389	-0.09368	0.078953	-1.18659	0.235391	NA
EPS8L1	1.946783	0.093671	0.068011	1.377283	0.168425	NA
P2RX4	146.8045	-0.09365	0.072251	-1.29624	0.194894	NA
GIN1	81.41518	0.093651	0.056766	1.649784	0.098987	NA
ELMOD1	163.4548	-0.09365	0.077308	-1.21137	0.225754	NA
LYSMD2	59.19759	0.093639	0.069464	1.348021	0.177652	NA
HIGD1A	141.6737	-0.09364	0.079239	-1.18171	0.237321	NA
SNORD116	0.846712	-0.09362	0.051305	-1.82484	0.068025	NA
CDS2	956.4319	0.093597	0.040949	2.285708	0.022271	0.23963
LOC44120	64.64064	-0.09355	0.084195	-1.11112	0.266518	NA

LTBP3	334.8928	-0.09352	0.062493	-1.49656	0.134508	0.487416
PLCL1	137.0965	-0.09349	0.077158	-1.21171	0.225622	NA
BCO2	46.86086	-0.09348	0.073682	-1.26874	0.204532	NA
ZFP82	68.77064	0.093479	0.057133	1.636167	0.101805	NA
GRM6	64.16379	0.093476	0.080226	1.165154	0.243957	NA
BOP1	53.59381	0.093463	0.068403	1.366355	0.171828	NA
TSPAN32	1.808599	-0.09346	0.06202	-1.50693	0.131827	NA
LOC14841	39.58095	0.093448	0.081258	1.150016	0.250137	NA
SPPL2B	116.3281	-0.09345	0.061461	-1.52041	0.128409	NA
ANGEL2	180.3126	0.093442	0.044972	2.077771	0.03773	NA
ASF1B	2.079085	-0.09344	0.067951	-1.37511	0.169097	NA
ZNF568	177.4684	0.093414	0.054945	1.700128	0.089107	NA
FAM92B	2.016208	-0.09341	0.049782	-1.87633	0.060609	NA
LUST	99.14153	-0.09338	0.0656	-1.42354	0.154579	NA
PSMB4	67.99221	-0.09338	0.057709	-1.61819	0.105621	NA
AARS	657.582	0.093374	0.043337	2.154599	0.031193	0.277495
SH3BGRL	81.22142	-0.09337	0.07634	-1.22308	0.221298	NA
EMILIN3	8.229819	-0.09337	0.078829	-1.18445	0.236236	NA
PRSS48	13.31972	-0.09335	0.084653	-1.10279	0.27012	NA
DPP6	898.3477	-0.09334	0.051534	-1.81118	0.070114	0.386393
ACVR1C	30.92299	-0.09332	0.078396	-1.1903	0.233928	NA
RNF213	612.8877	-0.09331	0.068106	-1.37014	0.170644	0.524434
FLJ35946	3.809716	-0.09331	0.076767	-1.21552	0.224168	NA
GTF2F1	402.4931	0.093308	0.063058	1.479711	0.13895	0.490222
BCL2L13	267.0601	0.093284	0.069304	1.346013	0.178298	NA
TRABD2A	0.97309	0.093278	0.053193	1.753573	0.079504	NA
MIR4670	1.014573	-0.09327	0.054925	-1.69807	0.089495	NA
PKP2	27.24482	-0.09324	0.082359	-1.13216	0.257567	NA
CD300E	10.84553	-0.09324	0.082933	-1.12429	0.260891	NA
FMO4	1.89473	-0.09323	0.066882	-1.39393	0.163337	NA
CXorf56	58.01413	-0.09322	0.069635	-1.33871	0.180665	NA
DGKA	83.69043	0.09322	0.069348	1.344226	0.178875	NA
MACROD2	30.08902	-0.09321	0.079615	-1.17078	0.241686	NA
FUZ	35.45456	-0.09316	0.073584	-1.26599	0.205517	NA
MSTO1	7.167382	-0.09316	0.084752	-1.09915	0.271701	NA
FAM185A	47.76246	-0.09315	0.065831	-1.41504	0.157055	NA
DNMBP-A	2.648206	-0.09315	0.073823	-1.26184	0.207006	NA
PSMB1	95.45657	-0.09313	0.055168	-1.68809	0.091394	NA
43344	42.68384	-0.09313	0.070106	-1.3284	0.184047	NA
CCDC135	1.314875	0.093125	0.058757	1.58493	0.112982	NA
LOC64418	1.515901	0.093123	0.063403	1.468749	0.141901	NA
APLNR	29.41564	-0.09312	0.072319	-1.28763	0.197874	NA
S1PR1	121.0947	-0.09312	0.078547	-1.18553	0.235808	NA
PHF21B	31.66624	-0.09311	0.078482	-1.18644	0.235448	NA
IL17RA	77.26324	-0.09311	0.08165	-1.14039	0.254124	NA
ZNF329	74.97395	0.093091	0.052747	1.764838	0.077591	NA
TPST1	95.03367	-0.09304	0.058953	-1.57825	0.114507	NA

DOK5	5.525616	0.09304	0.078712	1.182023	0.237196	NA
MIR124-2	2.475273	0.093023	0.07278	1.278139	0.2012	NA
ZNF569	83.05288	0.093023	0.05664	1.642359	0.100516	NA
OSTC	18.47577	-0.093	0.083123	-1.11877	0.263238	NA
IFT88	64.94487	-0.09299	0.063543	-1.46345	0.143344	NA
CC2D1A	281.4342	-0.09299	0.061428	-1.51379	0.130078	NA
FYN	494.9973	-0.09297	0.038578	-2.40996	0.015954	0.223064
SH3GL1P1	69.00269	-0.09295	0.078908	-1.17795	0.238816	NA
ATP2A2	1201.153	0.09295	0.060099	1.546604	0.121959	0.476542
PDZRN3	25.79045	-0.09295	0.082316	-1.12913	0.258843	NA
RHBDD3	35.35959	-0.09294	0.06891	-1.34871	0.17743	NA
CXXC4	89.9595	-0.09294	0.077048	-1.2062	0.227741	NA
CAV1	7.30077	-0.09293	0.083661	-1.11084	0.266639	NA
TRAF3IP1	161.0066	0.09293	0.052343	1.77541	0.07583	NA
UAP1L1	17.61309	-0.09293	0.084859	-1.09507	0.273488	NA
KCNIP2	84.7057	-0.09291	0.060712	-1.53029	0.125945	NA
SMIM17	22.52713	-0.09289	0.083842	-1.10791	0.267902	NA
SPN	4.21224	-0.09288	0.077486	-1.19865	0.230663	NA
STXBP1	2785.477	0.092857	0.048231	1.925245	0.054199	0.357364
C14orf64	3.360953	-0.09285	0.078052	-1.18954	0.234228	NA
TMEM44-	5.746361	-0.09284	0.082884	-1.12015	0.262649	NA
CHD1L	231.6722	-0.09284	0.050984	-1.82096	0.068613	NA
TXNRD1	163.5416	0.092817	0.048891	1.898472	0.057634	NA
PHGDH	70.13028	-0.09282	0.079387	-1.16914	0.242346	NA
STK11	243.7982	-0.0928	0.046655	-1.98901	0.0467	NA
USP21	70.57849	-0.09279	0.067246	-1.37987	0.167627	NA
SLC12A9	45.54833	0.09279	0.073598	1.260768	0.207392	NA
MSS51	77.00728	-0.09278	0.06048	-1.53402	0.125025	NA
NLGN4X	192.3701	0.092763	0.050986	1.819394	0.068851	NA
MB21D1	3.470039	-0.09276	0.074631	-1.24293	0.213893	NA
WDR63	0.972023	-0.09275	0.053652	-1.72875	0.083854	NA
NASP	403.2938	-0.09274	0.060677	-1.52844	0.126402	0.479566
GSDMB	101.2211	-0.09272	0.0723	-1.2824	0.199704	NA
ZCCHC2	212.8817	0.092639	0.055148	1.679838	0.092989	NA
ZC3HAV1L	1.281559	0.092617	0.058518	1.582716	0.113486	NA
TMEM173	4.248078	0.092612	0.079104	1.170768	0.241692	NA
POGK	218.27	0.092587	0.050747	1.824478	0.06808	NA
ATPAF1	200.3054	0.092565	0.04509	2.052913	0.040081	NA
FAM69C	6.100395	0.092543	0.080566	1.148657	0.250697	NA
PPP5D1	4.811983	0.092525	0.079349	1.166058	0.243591	NA
HEXA-AS1	6.010997	0.092508	0.081317	1.137626	0.255277	NA
NAA16	268.9633	-0.0925	0.05724	-1.61598	0.106098	NA
HNRPLL	160.4046	0.092497	0.057177	1.617737	0.105719	NA
RFXANK	12.04972	-0.0925	0.083711	-1.10495	0.269182	NA
ZFP28	126.1383	0.09249	0.051068	1.811122	0.070122	NA
MYO16	45.13003	-0.09249	0.084017	-1.10082	0.270976	NA
MED8	83.21895	0.092488	0.04928	1.876779	0.060548	NA

ISCA1	126.5301	-0.09248	0.05732	-1.61333	0.106674	NA
APMAP	328.6714	0.092451	0.063166	1.463614	0.1433	NA
ERCC2	42.92117	-0.09244	0.064983	-1.4226	0.154853	NA
BST1	2.506911	-0.09242	0.069411	-1.33153	0.183014	NA
SPTY2D1-A	4.197378	0.09241	0.080317	1.150563	0.249912	NA
METTL8	74.15972	0.092404	0.058773	1.572232	0.115897	NA
LOC64935	4.049632	0.092375	0.068471	1.349113	0.177301	NA
LOC10013	4.114676	-0.09237	0.078428	-1.17772	0.238909	NA
CYR61	11.32819	0.092358	0.071794	1.286428	0.198294	NA
CARNS1	69.00879	-0.09235	0.08487	-1.08811	0.276547	NA
HAUS4	35.78233	-0.09234	0.071774	-1.28661	0.198229	NA
FRMD6	162.038	0.092339	0.071195	1.296993	0.194634	NA
FSCN1	55.32992	-0.09233	0.07238	-1.27567	0.202073	NA
IRX3	20.88052	0.092333	0.084882	1.087774	0.276695	NA
TMEM69	37.75021	0.092315	0.066789	1.382188	0.166914	NA
MYH11	90.37361	0.092304	0.082666	1.116581	0.264174	NA
CYP2A7	5.287362	-0.0923	0.083133	-1.11025	0.266891	NA
ALDH6A1	128.8914	-0.0923	0.067675	-1.36382	0.172625	NA
USP31	489.8135	0.092281	0.049292	1.872122	0.06119	0.369586
ARPC5L	49.89929	0.09227	0.072173	1.278443	0.201093	NA
LOC64676	116.202	0.092268	0.065863	1.400904	0.161243	NA
OGFR	95.54938	-0.09227	0.066722	-1.38286	0.166708	NA
ZNF726	15.25535	-0.09226	0.083868	-1.10008	0.271295	NA
DLC1	92.96499	0.092233	0.071204	1.295325	0.195208	NA
MIRLET7F	5.351979	0.09222	0.081318	1.134066	0.256767	NA
DIRAS1	213.897	0.092216	0.073053	1.262317	0.206835	NA
DNAJB11	109.1861	0.092206	0.082809	1.113468	0.265507	NA
CCDC113	37.9024	-0.0922	0.084472	-1.0915	0.275054	NA
OGFOD1	138.2912	0.092196	0.044273	2.082453	0.037301	NA
SNORD16	2.634258	-0.09219	0.063355	-1.45509	0.145645	NA
PARVB	50.01247	-0.09217	0.078721	-1.17089	0.241642	NA
NR2F2	43.68436	-0.09217	0.078684	-1.17137	0.241451	NA
ARHGEF16	4.462913	0.092155	0.078549	1.173228	0.240705	NA
PPIL2	161.3359	-0.09215	0.055706	-1.65424	0.09808	NA
MATN1	1.008696	-0.09215	0.053145	-1.73392	0.082932	NA
RTN4IP1	56.09051	-0.09211	0.070894	-1.29924	0.19386	NA
CCND1	70.3846	0.092101	0.074086	1.243151	0.213812	NA
MED18	6.717206	-0.09209	0.084883	-1.08486	0.277984	NA
CTCFL	0.737667	-0.09208	0.046972	-1.96039	0.04995	NA
TAS2R40	3.413034	-0.09208	0.076764	-1.19946	0.230349	NA
SEC11C	106.9887	-0.09205	0.075616	-1.21738	0.22346	NA
FMO3	2.162587	-0.09205	0.068817	-1.33756	0.18104	NA
NSD1	1262.152	0.092042	0.035993	2.557178	0.010553	0.199179
C2CD4D	4.744064	-0.09203	0.082115	-1.12079	0.262379	NA
CXCR3	4.775018	0.092031	0.076601	1.20144	0.22958	NA
PCNXL4	155.7399	0.092015	0.051049	1.802498	0.071467	NA
TRIM69	16.36962	-0.092	0.084761	-1.08535	0.277765	NA

SLC16A11	9.5001	-0.09199	0.084791	-1.08491	0.277964	NA
MBTD1	239.0365	-0.09198	0.046496	-1.97828	0.047897	NA
ARHGEF12	2088.15	0.091974	0.029469	3.12108	0.001802	0.101784
DSN1	21.81483	-0.09196	0.078704	-1.16846	0.242622	NA
PIGF	12.70787	-0.09195	0.084546	-1.0876	0.276772	NA
KIF21A	886.2691	0.091886	0.044465	2.066503	0.038781	0.300865
43163	5.988119	-0.09188	0.079803	-1.15131	0.249604	NA
PRKG1	45.5479	-0.09187	0.081031	-1.13375	0.256901	NA
MYOZ3	6.115012	0.091868	0.081719	1.124189	0.260933	NA
LOC33881	7.2002	-0.09185	0.076437	-1.20167	0.229492	NA
LOC10050	30.42793	0.091834	0.084625	1.08518	0.277842	NA
C16orf52	199.539	0.091831	0.048015	1.912524	0.055809	NA
OR6X1	1.151972	-0.09181	0.053238	-1.72456	0.084606	NA
LRRC28	35.68166	-0.09181	0.065018	-1.412	0.157949	NA
TBC1D9	672.9614	0.091805	0.051377	1.7869	0.073954	0.390538
ZEB1-AS1	17.11832	0.091798	0.079382	1.156397	0.247519	NA
RHOD	4.647845	-0.0918	0.080027	-1.14708	0.25135	NA
CTAGE7P	7.032346	0.091787	0.084063	1.091888	0.274882	NA
ATP5I	92.50218	-0.09177	0.073191	-1.25391	0.209874	NA
REC8	156.1171	-0.09177	0.058453	-1.57	0.116415	NA
PGA5	1.072294	0.091758	0.047458	1.933451	0.053181	NA
PAK1	430.8123	0.091747	0.072859	1.259242	0.207943	0.55642
BCCIP	177.0842	0.091705	0.050566	1.813546	0.069748	NA
ETV1	1880.563	0.091698	0.06663	1.376223	0.168752	0.522164
DGKI	244.3202	0.091693	0.043015	2.131667	0.033034	NA
ATRN	434.4749	0.091665	0.052941	1.731458	0.08337	0.403119
MOV10L1	25.52546	0.091656	0.080908	1.132848	0.257278	NA
TJP3	6.983379	0.091645	0.078423	1.168603	0.242564	NA
TSPAN13	56.12548	0.091626	0.072912	1.25667	0.208873	NA
PTGFRN	23.3195	-0.09163	0.082363	-1.11246	0.26594	NA
ZNF718	40.97796	0.091602	0.082762	1.106822	0.268371	NA
ERMP1	254.4665	0.091602	0.042542	2.153214	0.031302	NA
PDZRN4	27.60087	-0.09159	0.081718	-1.12083	0.262359	NA
MAT2A	408.3346	-0.09158	0.04271	-2.14418	0.032018	0.27893
CIR1	227.0764	-0.09158	0.04976	-1.84039	0.065712	NA
BUB1B	9.212681	0.091568	0.084815	1.079618	0.280312	NA
ZNF12	254.7157	0.091565	0.04227	2.166168	0.030298	NA
NFATC3	279.4783	0.091564	0.040099	2.28348	0.022402	NA
RAB31	123.3731	-0.09154	0.069833	-1.31086	0.189904	NA
IGFBP5	549.0363	0.091538	0.082395	1.110963	0.266584	0.607287
MESDC1	133.5257	0.091538	0.04994	1.832939	0.066812	NA
LRP2	77.84791	-0.09152	0.084664	-1.08094	0.279722	NA
IDI2	10.26112	0.091492	0.083703	1.093053	0.274371	NA
ABTB2	57.28264	0.091492	0.07915	1.155935	0.247708	NA
IDI1	93.3629	0.091464	0.070078	1.305177	0.191833	NA
SNORD116	14.96207	-0.09146	0.084093	-1.08765	0.27675	NA
AHR	36.72103	-0.09145	0.073681	-1.24119	0.214536	NA

SNX20	19.07789	-0.09145	0.084464	-1.08273	0.27893	NA
C1QTNF7	26.91681	-0.09144	0.084506	-1.08205	0.279231	NA
FGL2	78.77889	-0.09141	0.084763	-1.0784	0.280853	NA
PLEKHM1P	113.9562	-0.0914	0.060556	-1.50941	0.131194	NA
DPP7	111.4093	-0.0914	0.054521	-1.67639	0.093663	NA
LINC00525	6.159392	-0.09139	0.083451	-1.09519	0.273433	NA
PRX	39.32244	-0.09137	0.081767	-1.11748	0.263791	NA
SLC35B4	216.1912	0.091345	0.058158	1.570647	0.116265	NA
PPP3CA	1093.617	0.091344	0.063029	1.449244	0.147269	0.500848
MXRA8	27.19329	-0.09134	0.081853	-1.1159	0.264467	NA
GCC1	94.51801	0.09134	0.056528	1.615834	0.10613	NA
PAFAH1B3	28.26018	0.09132	0.072433	1.260745	0.207401	NA
PREX1	375.6618	-0.09132	0.068456	-1.33398	0.18221	0.531153
PCDHB6	12.10126	-0.09132	0.084435	-1.08153	0.27946	NA
NELL2	190.4855	0.091319	0.062308	1.465612	0.142754	NA
IST1	246.7017	-0.09131	0.047307	-1.93018	0.053584	NA
KIAA1009	139.2105	-0.0913	0.0539	-1.69386	0.090292	NA
LOC90834	39.20898	-0.0913	0.08088	-1.12879	0.258985	NA
USP2	118.2148	0.091288	0.05757	1.585683	0.112811	NA
MYO6	697.3984	-0.09128	0.03859	-2.36532	0.018015	0.229527
CBX5	1147.022	0.091277	0.043005	2.122491	0.033797	0.288416
ZNF667	139.6007	0.09127	0.053029	1.721135	0.085226	NA
CBY3	10.50882	0.091261	0.084883	1.075135	0.282314	NA
HMGCR	187.2282	0.091261	0.054058	1.688209	0.091371	NA
SETD2	1832.993	0.091259	0.036141	2.525105	0.011566	0.201925
KIAA1324	26.12043	0.091258	0.079079	1.154016	0.248494	NA
SNORA62	82.59269	-0.09125	0.084738	-1.07689	0.28153	NA
CREBRF	604.7665	-0.09124	0.046443	-1.96454	0.049467	0.34589
LOC28317	797.3715	0.091213	0.051289	1.778398	0.075338	0.391143
NDUFA13	128.4051	-0.0912	0.071508	-1.27541	0.202164	NA
CUL3	393.7015	0.091201	0.047282	1.92889	0.053745	0.357364
LINC00035	5.540781	0.091173	0.081082	1.124459	0.260818	NA
BBS10	93.51934	0.091168	0.065565	1.390499	0.164377	NA
IL17RE	46.05441	0.091154	0.075276	1.210929	0.225923	NA
STX1A	318.0628	0.091144	0.062951	1.447865	0.147655	NA
DNAJC27	83.77655	0.09113	0.059946	1.520208	0.128459	NA
C19orf25	51.98147	-0.09112	0.067442	-1.35113	0.176654	NA
USP49	56.40974	0.091104	0.062263	1.463207	0.143411	NA
B4GALNT3	5.890759	0.091102	0.077065	1.182149	0.237147	NA
SLC36A4	112.3872	-0.09109	0.054786	-1.6627	0.096373	NA
CAND2	98.71603	0.09109	0.062007	1.469018	0.141828	NA
CXCR2	1.089783	0.091087	0.047418	1.920916	0.054742	NA
ANAPC1	131.6928	-0.09109	0.046825	-1.94523	0.051747	NA
LOC28575	7.461192	-0.09108	0.083243	-1.09411	0.273905	NA
STRAP	153.3648	0.09107	0.063432	1.43571	0.151085	NA
LINC00636	3.572766	-0.09107	0.07639	-1.19217	0.233194	NA
SCN5A	8.066086	0.091068	0.084671	1.075551	0.282128	NA

NSMCE4A	58.25736	-0.09106	0.061454	-1.48175	0.138408	NA
RPS19BP1	92.95078	-0.09105	0.058056	-1.56831	0.116808	NA
ZNF570	73.76349	0.091034	0.054717	1.663721	0.096168	NA
GAL3ST3	37.46157	-0.09103	0.073577	-1.23716	0.216028	NA
ZNF420	79.77168	0.090983	0.057421	1.584487	0.113083	NA
VSNL1	1314.134	0.090979	0.081311	1.118897	0.263184	0.60594
MIR1252	2.010297	-0.09097	0.066656	-1.3647	0.172346	NA
FBXO7	127.3529	0.090952	0.052884	1.719853	0.085459	NA
LPP-AS2	9.215315	-0.09093	0.084806	-1.07218	0.28364	NA
CADPS2	2349.544	0.090913	0.046488	1.955604	0.050512	0.34688
HSP90B1	746.5938	0.090909	0.078901	1.152181	0.249247	0.592704
FAM168B	393.5411	0.090899	0.058007	1.567028	0.117108	0.472599
SWAP70	77.61497	-0.09089	0.070149	-1.29565	0.195094	NA
DNAJC22	0.686885	-0.09088	0.046654	-1.94804	0.05141	NA
GGT7	211.9229	-0.09088	0.049685	-1.82916	0.067376	NA
HYDIN	567.7122	-0.09087	0.082316	-1.10392	0.269627	0.610306
SEC1P	7.393285	-0.09087	0.084199	-1.07922	0.28049	NA
DRP2	6.184906	-0.09086	0.084003	-1.08161	0.279426	NA
TPTE2P5	43.95032	0.090855	0.070622	1.286506	0.198267	NA
LOC14937	14.73979	0.090852	0.084865	1.070553	0.28437	NA
TNFAIP1	90.61284	0.090831	0.049857	1.821822	0.068482	NA
VRK1	46.969	0.090823	0.067526	1.345008	0.178623	NA
SLC29A4	18.55689	-0.09082	0.084763	-1.07148	0.283952	NA
IGBP1	112.2752	-0.09082	0.067987	-1.33586	0.181595	NA
C22orf29	120.4387	0.090813	0.05527	1.643069	0.100369	NA
ACSL1	116.3518	-0.09081	0.057214	-1.58713	0.112483	NA
GFRA2	15.10696	-0.09078	0.084848	-1.06989	0.284669	NA
TEX261	91.3966	0.090755	0.056786	1.598207	0.109997	NA
MIR4755	1.908132	-0.09075	0.062221	-1.4585	0.144702	NA
LOC25304	4.634649	-0.09074	0.080886	-1.12186	0.261922	NA
LCA5	191.2544	-0.09074	0.060359	-1.50337	0.132743	NA
GABPB1-A	133.446	-0.09072	0.076547	-1.18522	0.235931	NA
MYO10	231.6281	-0.09072	0.074605	-1.21605	0.223967	NA
GLIPR1	29.11208	0.09071	0.075272	1.205084	0.228171	NA
PTPRK	90.58505	-0.09068	0.071482	-1.26853	0.204609	NA
LPO	2.93816	-0.09067	0.071041	-1.27631	0.201847	NA
KATNBL1	110.5347	-0.09067	0.06457	-1.40415	0.160275	NA
LOC64383	215.5609	0.090662	0.054435	1.665519	0.095809	NA
FAM179B	274.2834	0.090662	0.034258	2.646426	0.008135	NA
B3GALT1	26.00146	0.090652	0.078333	1.157275	0.24716	NA
RBM11	8.382745	-0.09062	0.082686	-1.09598	0.273088	NA
CA1	10.58971	0.090616	0.084628	1.07076	0.284277	NA
MYO1H	3.532852	0.09061	0.077833	1.164151	0.244363	NA
RAB12	203.8751	0.0906	0.050774	1.784391	0.07436	NA
DCLRE1A	56.81	0.090583	0.070023	1.293621	0.195796	NA
UBE2K	474.2727	0.090569	0.051621	1.754491	0.079346	0.397742
DDB2	51.75235	-0.09057	0.06868	-1.31867	0.187278	NA

HSPB9	1.044468	0.090564	0.054412	1.664418	0.096029	NA
PPARD	167.8933	-0.09052	0.048184	-1.87863	0.060295	NA
ESPN	49.72996	-0.09052	0.081057	-1.11669	0.264128	NA
SIRT6	17.11149	-0.09051	0.081605	-1.10907	0.2674	NA
FNIP2	173.3329	0.090477	0.045442	1.991034	0.046477	NA
PAPL	99.10436	-0.09046	0.069293	-1.30542	0.191748	NA
RHOJ	23.25384	-0.09044	0.084666	-1.06823	0.285418	NA
RSPH4A	12.67257	-0.09043	0.079148	-1.14252	0.253238	NA
EIF1B-AS1	28.49627	-0.09042	0.07986	-1.13224	0.257532	NA
CPQ	42.65715	-0.09039	0.067333	-1.34245	0.17945	NA
C19orf54	8.900528	-0.09038	0.084796	-1.06589	0.286472	NA
USHBP1	8.693905	-0.09038	0.084862	-1.06503	0.286862	NA
FAM153C	151.6	-0.09038	0.066257	-1.36408	0.172542	NA
RIBC1	7.788195	-0.09038	0.084932	-1.0641	0.287285	NA
CDH20	87.01411	-0.09036	0.074333	-1.21559	0.224141	NA
MED21	150.5654	0.090353	0.057608	1.568426	0.116782	NA
KCNK7	4.803952	0.090336	0.078589	1.149476	0.25036	NA
FANCC	200.7508	-0.09032	0.056394	-1.60166	0.10923	NA
RBM15B	151.0492	0.090313	0.050338	1.794133	0.072792	NA
MNX1	0.718077	-0.09031	0.045288	-1.9941	0.046141	NA
FILIP1	13.09935	-0.09031	0.084716	-1.06601	0.286418	NA
ATP6V0D1	73.58209	0.090272	0.078013	1.157134	0.247218	NA
TMEM30A	642.662	0.090264	0.060284	1.497304	0.134314	0.487416
ETF1	119.6587	0.09026	0.05024	1.79658	0.072402	NA
CAST	260.8821	-0.09025	0.043843	-2.05846	0.039546	NA
FSTL4	100.2428	-0.09022	0.074111	-1.21742	0.223444	NA
FLT4	9.288131	0.090222	0.084735	1.064755	0.286987	NA
XRN1	727.7722	-0.09019	0.040719	-2.21493	0.026765	0.259072
RGS6	55.865	-0.09019	0.071306	-1.26483	0.205931	NA
SNORD6	2.943346	-0.09019	0.07506	-1.20152	0.229548	NA
CLDND1	308.6053	-0.09017	0.070839	-1.27291	0.203048	NA
EBF3	9.152587	-0.09016	0.084129	-1.07164	0.283883	NA
ARF6	137.5245	0.090143	0.062836	1.434574	0.151409	NA
PWWP2B	17.21272	0.090141	0.080199	1.123973	0.261024	NA
KPNA2	29.69253	0.090132	0.082444	1.093249	0.274285	NA
PCDHGA2	53.06806	0.090127	0.081222	1.109637	0.267155	NA
ZSCAN5A	3.042989	-0.09012	0.076443	-1.17895	0.238417	NA
TP53	10.58808	-0.09011	0.084509	-1.06632	0.286281	NA
ACRBP	14.99907	-0.09011	0.083649	-1.07727	0.281361	NA
TDRKH	237.9111	0.090112	0.065478	1.376203	0.168759	NA
LDHAL6A	27.20761	0.090109	0.082373	1.093912	0.273994	NA
LNX2	99.03542	0.090108	0.066369	1.357686	0.174563	NA
KIAA1107	577.6981	0.090103	0.0516	1.746189	0.080778	0.400784
PARP15	0.901146	0.090091	0.052392	1.719559	0.085513	NA
GPR137B	50.55432	-0.09006	0.068671	-1.31148	0.189696	NA
CTC1	387.5434	-0.09006	0.060214	-1.49566	0.134743	0.487416
FAM189A2	32.18868	-0.09005	0.08489	-1.06081	0.288777	NA

GNPNAT1	100.6289	0.090048	0.055732	1.615729	0.106153	NA
CCDC85A	30.02859	0.090034	0.084408	1.066645	0.286132	NA
DTX1	121.8892	0.090021	0.059229	1.519874	0.128543	NA
PXN-AS1	6.762301	-0.09002	0.083372	-1.07972	0.280268	NA
SEC14L4	1.927408	0.089995	0.058544	1.537214	0.124241	NA
EDA	4.178994	0.089986	0.079384	1.13355	0.256983	NA
GAB1	187.9307	-0.08998	0.06834	-1.31671	0.187935	NA
PCDHGB7	206.4253	0.089972	0.075814	1.186737	0.235331	NA
FSIP1	12.68701	-0.08997	0.083937	-1.07184	0.283792	NA
NEAT1	1270.278	-0.08992	0.078149	-1.15066	0.249873	0.592704
ABCB1	89.12277	0.08991	0.078095	1.151291	0.249613	NA
TM2D2	83.12857	0.089888	0.064026	1.403922	0.160342	NA
PPP1R17	72.213	-0.08988	0.081646	-1.10091	0.270935	NA
ZNF7	76.15695	0.089867	0.060214	1.492468	0.135577	NA
PDE9A	74.41315	-0.08982	0.070543	-1.27322	0.20294	NA
FAM76A	86.23837	0.089796	0.073905	1.21501	0.224362	NA
THAP8	13.65105	-0.08978	0.083637	-1.0734	0.283092	NA
CDCA3	4.211068	0.089756	0.075472	1.189265	0.234335	NA
COQ6	29.42066	-0.08974	0.076227	-1.17727	0.239086	NA
BCAR3	39.42221	-0.08974	0.071672	-1.25207	0.210545	NA
CHN2	1674.912	0.089737	0.041653	2.154406	0.031208	0.277495
C1R	25.95578	-0.08974	0.07684	-1.16785	0.242869	NA
N4BP2L2-I	418.6814	-0.08973	0.067071	-1.33787	0.18094	0.53091
WDR37	153.8882	-0.08972	0.04533	-1.97933	0.047778	NA
RBM4B	173.6942	-0.08972	0.063813	-1.40593	0.159745	NA
TMEM101	24.373	-0.08972	0.078606	-1.14134	0.253729	NA
HSPA12A	285.0534	0.089711	0.051021	1.75833	0.078691	NA
CCDC151	17.71861	0.089702	0.084762	1.058284	0.289926	NA
PIK3R5	4.868719	-0.08965	0.079223	-1.13163	0.257791	NA
SNTA1	86.09742	-0.08965	0.078728	-1.13869	0.254832	NA
TTC36	7.274182	0.089631	0.084901	1.055706	0.291103	NA
LMO7	156.1655	0.089631	0.057399	1.561542	0.118396	NA
SMAP1	89.81718	0.08963	0.053782	1.666535	0.095607	NA
TMED8	65.15586	0.089629	0.072247	1.2406	0.214754	NA
SEC22A	86.27046	0.089626	0.055186	1.624068	0.104361	NA
COX7A2	72.06415	-0.08962	0.082509	-1.08619	0.277393	NA
NHSL2	226.692	0.089581	0.061146	1.465044	0.142909	NA
ZBTB33	128.5271	0.08958	0.061158	1.464723	0.142996	NA
RSPH1	6.173707	0.089563	0.081158	1.103565	0.269782	NA
CLEC4A	3.915704	-0.08956	0.078534	-1.14043	0.254106	NA
RPL7A	328.1708	-0.08956	0.069439	-1.28977	0.197132	NA
ANP32C	1.141509	0.089541	0.053209	1.682815	0.092411	NA
RPL28	276.6743	-0.08951	0.059272	-1.51008	0.131022	NA
ANKRD31	12.89366	-0.08949	0.084933	-1.0537	0.292019	NA
BPIFB1	1.47838	0.089485	0.063566	1.40774	0.159208	NA
MANEA	54.01215	0.089478	0.065982	1.35611	0.175064	NA
IL4R	30.1378	-0.08945	0.081733	-1.09442	0.27377	NA

SCYL3	73.1325	-0.08945	0.065869	-1.35795	0.174479	NA
SH3GL2	956.6449	0.089445	0.048701	1.836631	0.066264	0.375222
TRIM38	12.11405	-0.08944	0.084781	-1.05491	0.291464	NA
LINC00271	2.328151	-0.08943	0.070704	-1.26483	0.205932	NA
EDNRB	135.8786	0.089428	0.084776	1.054881	0.29148	NA
TNR	331.2622	-0.08942	0.068584	-1.30384	0.192287	NA
LOC10028	3.417846	-0.08942	0.078728	-1.13585	0.256021	NA
PELI1	164.3877	0.089422	0.047693	1.87496	0.060798	NA
PHLPP2	312.1154	0.089412	0.040598	2.202358	0.02764	NA
SNORD116	644.4888	-0.0894	0.084397	-1.05931	0.289459	0.622625
ZC3H12C	236.4052	0.089397	0.041784	2.139516	0.032394	NA
PRPF8	1322.325	0.089377	0.039046	2.289033	0.022077	0.23963
TSSK4	32.35218	-0.08937	0.074209	-1.20435	0.228454	NA
WDR13	91.31693	-0.08937	0.053222	-1.67921	0.093111	NA
STK40	80.24072	0.089362	0.061285	1.458133	0.144804	NA
FONG	7.015866	-0.08935	0.084118	-1.06226	0.28812	NA
ZFAT-AS1	2.546887	-0.08934	0.072459	-1.23303	0.217565	NA
LEMD2	117.2385	-0.08934	0.061936	-1.44252	0.149155	NA
NBEA	2085.078	0.089336	0.036802	2.427495	0.015204	0.216391
DNAJC16	170.2855	0.089327	0.046012	1.941375	0.052213	NA
ERICH1	67.44026	-0.08932	0.08195	-1.08991	0.275754	NA
KLHDC1	81.22762	-0.08931	0.064688	-1.38067	0.167379	NA
EPB41L3	1293.58	0.089309	0.048738	1.832421	0.066889	0.377813
LRMP	5.356599	-0.08931	0.081331	-1.09809	0.272166	NA
MEF2C	179.7848	-0.08931	0.065675	-1.35985	0.173878	NA
CLIP4	211.8301	-0.08929	0.052112	-1.71344	0.086631	NA
CADM2	830.9452	0.089288	0.053223	1.67763	0.093419	0.419831
DTX4	181.9564	0.089283	0.047817	1.86716	0.061879	NA
PIGA	29.76248	-0.08928	0.075282	-1.18591	0.235656	NA
ATP11C	58.85424	-0.08927	0.084798	-1.05279	0.292436	NA
HIPK4	56.57395	-0.08927	0.075747	-1.17856	0.238574	NA
PTPN5	16.35788	-0.08925	0.084688	-1.05392	0.291918	NA
SNORD42B	11.17674	-0.08925	0.082809	-1.0778	0.281122	NA
TSC22D2	210.3259	0.089242	0.042776	2.086276	0.036954	NA
TMCC1	495.2965	0.08924	0.047418	1.881973	0.05984	0.366535
KCNC3	197.781	0.089235	0.054456	1.638671	0.101282	NA
FYCO1	53.89968	-0.08923	0.076799	-1.16187	0.24529	NA
CSRP2	24.88539	-0.08922	0.084883	-1.05107	0.293228	NA
NUMA1	2060.662	-0.08922	0.039636	-2.25087	0.024394	0.246583
ZNHIT6	155.0876	0.089207	0.047945	1.860623	0.062797	NA
ZNF746	99.68663	0.089176	0.051129	1.744125	0.081137	NA
HHIP-AS1	3.279756	-0.08917	0.078025	-1.14285	0.253099	NA
C5orf55	4.1417	0.089165	0.08016	1.112343	0.265991	NA
GOLPH3L	84.11706	0.08916	0.061323	1.453932	0.145965	NA
LDLRAP1	500.0897	-0.08915	0.054574	-1.63348	0.102368	0.443332
ZNF691	17.5015	-0.08914	0.084402	-1.05612	0.290915	NA
KPNA4	283.9222	0.089133	0.05074	1.75664	0.078979	NA

TMEM18	45.51967	-0.08913	0.069814	-1.27671	0.201704	NA
MTX2	57.89197	0.08912	0.061592	1.446931	0.147916	NA
ZNF620	43.90305	0.089112	0.067599	1.318246	0.187421	NA
CEP112	150.7509	0.08911	0.072864	1.22296	0.221345	NA
BSN-AS2	13.04299	0.089093	0.084488	1.054507	0.291651	NA
BLNK	6.730297	-0.08909	0.08417	-1.05844	0.289853	NA
A2ML1	27.31169	-0.08907	0.075604	-1.17811	0.238754	NA
SYT3	58.02126	0.089068	0.064683	1.376979	0.168519	NA
CPNE8	28.70847	-0.08905	0.08469	-1.0515	0.293028	NA
COL17A1	10.97763	0.089048	0.084923	1.048581	0.294371	NA
STK24	155.9871	0.088996	0.046271	1.92337	0.054434	NA
IGIP	176.6716	0.088992	0.058007	1.534149	0.124993	NA
RAD51B	5.506591	-0.08898	0.080515	-1.10518	0.269083	NA
CEP85L	409.2031	0.088983	0.041408	2.148916	0.031641	0.277779
TNFAIP8	2.834157	-0.08898	0.071408	-1.24603	0.212752	NA
MIR4782	1.289917	-0.08897	0.053996	-1.64767	0.099419	NA
PRIM2	30.94749	-0.08896	0.072882	-1.22062	0.222229	NA
TMPRSS9	28.01323	-0.08896	0.084449	-1.0534	0.29216	NA
PGM5P2	15.32104	-0.08895	0.084483	-1.05292	0.292379	NA
MIA3	940.0879	0.088931	0.033494	2.655135	0.007928	0.171303
PDE1C	57.93975	0.088931	0.084547	1.051852	0.292867	NA
HSD17B14	40.0296	-0.08893	0.06459	-1.37677	0.168582	NA
ZCCHC18	28.89833	0.088924	0.072783	1.221778	0.221792	NA
CAMK2B	1044.841	0.088919	0.041341	2.150874	0.031486	0.277495
PODNL1	21.90981	-0.0889	0.084908	-1.04704	0.295083	NA
OVCA2	18.41085	-0.0889	0.080724	-1.1013	0.270765	NA
APOO	32.32625	-0.08889	0.077293	-1.15008	0.250111	NA
GPR61	30.2982	-0.08889	0.072673	-1.22319	0.221257	NA
CNTNAP2	208.425	0.088888	0.073625	1.207303	0.227316	NA
ADM5	25.45962	-0.08889	0.082523	-1.07711	0.281431	NA
OPHN1	225.025	-0.08888	0.042359	-2.09834	0.035875	NA
SPATA21	8.444949	-0.08888	0.083067	-1.06998	0.28463	NA
RIOK2	72.66974	0.088878	0.061273	1.450526	0.146912	NA
VSIG10L	42.61642	0.088875	0.075464	1.177712	0.238912	NA
WDR47	332.9248	0.088852	0.049093	1.809891	0.070313	NA
RHBG	27.89366	-0.08884	0.084656	-1.04944	0.293976	NA
TCAIM	153.3342	0.088836	0.046279	1.919573	0.054912	NA
SERPINE1	4.205587	-0.08882	0.060663	-1.46421	0.143135	NA
TM7SF2	29.12767	-0.08879	0.073514	-1.20777	0.227135	NA
LOC10012	7.826005	0.088785	0.08466	1.048725	0.294305	NA
RND1	9.848982	-0.08877	0.083696	-1.06065	0.288847	NA
MYO1F	16.66152	-0.08877	0.083656	-1.06111	0.288638	NA
DUSP22	37.43859	-0.08876	0.066414	-1.33647	0.181397	NA
PEX7	8.568006	-0.08875	0.084561	-1.04955	0.293925	NA
LRRC7	14.98686	-0.08874	0.08423	-1.05359	0.292068	NA
DCP2	343.2709	0.088738	0.060228	1.473384	0.140648	0.492546
CAMSAP2	1285.017	0.088738	0.03942	2.251105	0.024379	0.246583

PVRL3	20.19918	-0.08873	0.081395	-1.09008	0.275679	NA
SLC22A20	11.75857	-0.08872	0.084792	-1.04634	0.295402	NA
GNB2	86.61528	-0.08872	0.051217	-1.73225	0.083229	NA
SYNCRIP	572.7026	0.088699	0.043699	2.02979	0.042378	0.318294
CCT7	244.7424	0.088698	0.066511	1.333578	0.182342	NA
AGMO	0.956252	-0.0887	0.052645	-1.68481	0.092025	NA
HECW1	300.6189	0.088692	0.051301	1.728862	0.083834	NA
CTNS	59.01051	-0.08869	0.077359	-1.14646	0.251603	NA
ELAVL2	111.5982	0.088686	0.070327	1.261061	0.207287	NA
LOC10050	1.934229	-0.08868	0.067364	-1.31648	0.188012	NA
ASIC3	80.25999	-0.08867	0.070966	-1.24948	0.211491	NA
INSC	0.983494	-0.08866	0.050945	-1.7404	0.081789	NA
ALCAM	78.83362	-0.08866	0.082387	-1.07616	0.281854	NA
FLI1-AS1	1.717794	-0.08864	0.061541	-1.4403	0.149783	NA
INTU	63.2326	-0.08859	0.075337	-1.17598	0.239601	NA
KIAA1671	130.9462	-0.08858	0.0707	-1.25292	0.210234	NA
CXCR5	8.681547	-0.08857	0.084937	-1.04282	0.297033	NA
NPY6R	58.41279	-0.08856	0.078347	-1.13037	0.258319	NA
SPATA20	116.0184	0.08856	0.066069	1.340412	0.180111	NA
TIMM23	45.63872	-0.08855	0.06908	-1.28178	0.199919	NA
EFHA1	173.8665	-0.08852	0.047832	-1.85074	0.064207	NA
GRIN3A	9.973277	0.088521	0.084875	1.042957	0.296968	NA
TRPM6	23.01206	-0.08851	0.082803	-1.06897	0.285084	NA
ZNF37BP	758.4737	-0.08849	0.045047	-1.96445	0.049478	0.34589
MTHFD2	24.80416	-0.08848	0.083556	-1.05897	0.289614	NA
ATP5L	222.6566	-0.08848	0.068923	-1.28374	0.199235	NA
MRPL2	46.31178	-0.08847	0.060937	-1.45183	0.146547	NA
CCDC90B	86.69971	0.088467	0.058085	1.52307	0.127741	NA
NUS1	59.3967	0.088466	0.058885	1.50236	0.133004	NA
PGRMC2	187.6934	0.088453	0.049225	1.796892	0.072353	NA
AEN	12.56649	0.088453	0.084029	1.052644	0.292504	NA
C7orf49	24.2198	-0.08845	0.07675	-1.1524	0.249157	NA
MLLT4-AS	3.80537	-0.08843	0.080353	-1.10052	0.271105	NA
LYAR	36.41892	0.088422	0.074574	1.185693	0.235744	NA
ZNF554	62.24316	-0.08841	0.07383	-1.19748	0.231118	NA
SP100	33.10803	-0.08841	0.082656	-1.0696	0.284801	NA
PPP2R5B	274.7216	0.0884	0.049194	1.796984	0.072338	NA
SPINT2	75.30859	0.088394	0.066011	1.339076	0.180546	NA
LOC10050	56.98462	-0.08839	0.072796	-1.21427	0.224645	NA
BRE	47.05724	-0.08839	0.074985	-1.1788	0.238479	NA
UBL4A	38.4666	0.088389	0.071733	1.232183	0.217881	NA
CTU1	8.440594	0.08836	0.084886	1.040935	0.297906	NA
HLA-DRB5	12.72232	-0.08832	0.067488	-1.30864	0.190658	NA
MMACHC	28.19759	-0.08828	0.072249	-1.22193	0.221733	NA
CD22	34.29425	-0.08828	0.084799	-1.04106	0.297848	NA
HRH2	4.545559	-0.08828	0.078683	-1.12192	0.261895	NA
NTRK2	1482.135	-0.08825	0.055081	-1.60223	0.109105	0.454268

SYNJ2	110.5653	-0.08825	0.079532	-1.10963	0.267158	NA
FIGF	2.885544	0.088228	0.074061	1.191294	0.233538	NA
PRNP	1379.309	0.088223	0.058669	1.503746	0.132647	0.487416
SH2D5	4.42059	-0.08822	0.080205	-1.09988	0.271385	NA
ZNF615	142.332	0.088204	0.045831	1.924568	0.054283	NA
CYP4F3	2.843786	-0.0882	0.056436	-1.5629	0.118077	NA
GOLGA1	145.955	-0.0882	0.055285	-1.59531	0.110643	NA
OPLAH	88.03307	0.088195	0.082419	1.070087	0.28458	NA
SNORD7	0.571488	-0.08818	0.04259	-2.07055	0.038401	NA
SNORD56B	2.944597	0.088167	0.07668	1.149803	0.250225	NA
ZNF790	125.0661	0.088141	0.048122	1.831601	0.067011	NA
AMT	75.40818	-0.08814	0.067136	-1.31282	0.189244	NA
SLC25A44	126.2458	0.088135	0.06096	1.445797	0.148234	NA
ZNF542	112.2836	0.088116	0.057572	1.530525	0.125887	NA
TSC1	914.4638	-0.08811	0.038627	-2.281	0.022548	0.23963
RSG1	6.318578	0.088079	0.082879	1.062748	0.287896	NA
RBAK	275.8457	0.088079	0.045912	1.918428	0.055057	NA
PM20D1	29.13415	0.088069	0.057093	1.542534	0.122944	NA
CCDC160	5.859659	-0.08806	0.083529	-1.05427	0.291761	NA
PIAS3	119.9445	0.088061	0.05374	1.638657	0.101285	NA
CYP17A1	9.383798	-0.08803	0.082929	-1.06152	0.288454	NA
DYNLT3	67.2424	0.08803	0.073257	1.201655	0.229497	NA
ATXN7L3B	442.7756	0.088024	0.054039	1.628897	0.103335	0.445167
ALKBH5	284.3136	0.088021	0.047204	1.864679	0.062226	NA
OIT3	10.58009	0.087973	0.07818	1.125263	0.260478	NA
ETS1	26.14847	-0.08797	0.083252	-1.05662	0.290684	NA
TMEM5	15.69382	0.08796	0.084163	1.045124	0.295966	NA
ZFHX3	87.725	-0.08796	0.062063	-1.41727	0.156404	NA
CAMK2D	662.5868	0.087946	0.044104	1.994064	0.046145	0.336072
PPFIBP2	80.5763	-0.08793	0.07358	-1.19505	0.232068	NA
SIRT7	47.00407	0.087916	0.083798	1.049136	0.294115	NA
CDH5	29.44599	-0.08789	0.084572	-1.03929	0.298668	NA
APPL1	518.5237	0.08789	0.038359	2.291239	0.02195	0.23963
MIR548T	1.031991	0.087884	0.05138	1.710491	0.087175	NA
CDIP1	197.1473	0.087881	0.062443	1.407385	0.159313	NA
ADAM21P	1.121712	-0.08786	0.056323	-1.55996	0.118769	NA
ENTPD7	81.9687	0.087857	0.059453	1.477764	0.139471	NA
TXNDC9	51.71628	-0.08785	0.064696	-1.35789	0.1745	NA
KLHL24	730.3117	-0.08785	0.048453	-1.81298	0.069835	0.385986
NRBP1	305.1831	0.087843	0.036081	2.434592	0.014909	NA
TRAPPC10	291.0656	0.087842	0.056437	1.556456	0.1196	NA
PRIMA1	53.31608	0.087816	0.076421	1.149104	0.250513	NA
TMEM99	4.249545	0.087807	0.076745	1.144136	0.252567	NA
POC1A	2.858839	-0.0878	0.076564	-1.14673	0.251492	NA
SLC46A2	13.04265	0.087793	0.084907	1.033997	0.301138	NA
PIK3CD	98.69773	-0.08777	0.078017	-1.125	0.260588	NA
KBTBD12	61.05185	0.087757	0.074778	1.173566	0.240569	NA

ARRDC3-A	4.739111	-0.08775	0.083434	-1.05175	0.292916	NA
RAD23B	566.1565	0.087743	0.046416	1.890369	0.058709	0.36517
FAM196A	21.67501	-0.08774	0.080779	-1.08615	0.277412	NA
HINT2	8.090311	-0.08773	0.084912	-1.03318	0.30152	NA
ANG	0.882733	-0.08773	0.047842	-1.83365	0.066705	NA
SNORD5	6.031352	-0.08772	0.084491	-1.03823	0.299165	NA
PHF7	31.7031	-0.08772	0.080576	-1.08863	0.276315	NA
ICAM3	8.036291	-0.08771	0.084904	-1.033	0.301603	NA
ITIH2	27.54894	0.087704	0.079693	1.100523	0.271104	NA
NUDT2	18.89759	-0.0877	0.081969	-1.06994	0.284647	NA
CHST6	127.8371	0.087695	0.077453	1.132226	0.257539	NA
TCP10L	84.34583	0.087686	0.083545	1.049564	0.293919	NA
ATP5J2	48.56009	-0.08767	0.08472	-1.03486	0.300736	NA
HERC2P4	6.347659	0.087671	0.077707	1.128235	0.259221	NA
RMDN2	40.09187	-0.08766	0.071135	-1.23233	0.217824	NA
MMP11	2.427697	0.087661	0.069317	1.264641	0.206	NA
C4orf52	27.33014	-0.08766	0.083323	-1.05205	0.292776	NA
TMEM169	35.37603	0.087657	0.079581	1.101482	0.270687	NA
FAM86B1	14.905	0.087643	0.084743	1.03422	0.301033	NA
RAB21	235.1336	0.087638	0.045422	1.929439	0.053676	NA
LOC81691	16.49365	-0.08763	0.084046	-1.04268	0.297095	NA
CCDC30	65.13957	0.08762	0.059615	1.469749	0.14163	NA
FAM134A	256.5036	0.087602	0.055094	1.590027	0.111829	NA
MIER2	65.60881	0.087589	0.059704	1.467051	0.142362	NA
IRAK1	66.55519	-0.08757	0.062281	-1.40611	0.159692	NA
AKIP1	28.96313	-0.08757	0.077857	-1.1247	0.260718	NA
XBP1	81.07197	0.087565	0.076771	1.140594	0.254039	NA
C4orf26	1.542837	-0.08755	0.056632	-1.54595	0.122116	NA
CLTB	297.3277	0.087541	0.052199	1.677053	0.093532	NA
LSM3	62.86583	0.08754	0.072895	1.200896	0.229792	NA
GPR108	47.79044	-0.08753	0.069466	-1.26009	0.207636	NA
MCCC1	215.186	-0.08753	0.050393	-1.73701	0.082385	NA
ULK1	344.7233	-0.08753	0.055217	-1.5852	0.112921	0.464015
RBM44	4.237412	0.087525	0.079379	1.102624	0.27019	NA
SMCR8	239.9825	0.087524	0.041558	2.106075	0.035198	NA
CPNE5	21.18235	0.08752	0.083898	1.043161	0.296874	NA
SNORD113	12.27515	-0.08752	0.078491	-1.11502	0.264841	NA
NFYA	345.3786	0.087517	0.056124	1.559359	0.118911	0.472798
PRSS12	13.19019	0.087514	0.081714	1.070988	0.284175	NA
PLSCR1	26.56297	-0.08751	0.083846	-1.04371	0.296621	NA
PPP2R2C	434.9699	0.087508	0.049871	1.754673	0.079315	0.397742
RPH3A	941.7941	0.087497	0.067685	1.29271	0.196111	0.545356
LOC10099	30.57845	0.087487	0.075937	1.152103	0.249279	NA
TSPAN7	581.6153	0.087478	0.069383	1.260798	0.207382	0.55642
RANBP6	193.7033	0.087465	0.054787	1.596451	0.110388	NA
MIR630	4.038594	0.087448	0.079841	1.095271	0.273398	NA
LOC40783	1.898216	0.087441	0.06144	1.4232	0.154678	NA

WDR88	6.915552	-0.08743	0.084073	-1.03997	0.298354	NA
CD83	83.88486	0.087417	0.070305	1.243391	0.213724	NA
OLFML3	3.946554	0.087391	0.078318	1.115859	0.264482	NA
SMIM1	2.289091	-0.08739	0.068051	-1.28421	0.199069	NA
LRRN1	139.9469	0.087389	0.067491	1.29482	0.195382	NA
ANKRD45	8.256818	-0.08739	0.084691	-1.03184	0.302146	NA
HIST1H3E	1.350836	0.087381	0.057333	1.524116	0.12748	NA
CCDC39	34.77371	-0.08737	0.069768	-1.25228	0.210467	NA
TSPAN2	4.60186	0.087365	0.042839	2.039392	0.041411	NA
ATF6B	235.298	-0.08736	0.043484	-2.00905	0.044532	NA
ZNF496	70.00224	-0.08736	0.065103	-1.34189	0.179633	NA
TDRD5	1.206805	0.087356	0.057884	1.509165	0.131257	NA
LRIT3	6.636186	-0.08732	0.08408	-1.03858	0.298999	NA
FSHR	2.723609	0.08732	0.054326	1.607328	0.107982	NA
COX6B1	83.82735	-0.08731	0.074372	-1.17393	0.240424	NA
IL20RB	6.325913	0.087306	0.082968	1.052285	0.292669	NA
ZNF709	113.8411	0.087303	0.048909	1.785002	0.074261	NA
MROH5	1.362928	-0.08726	0.057026	-1.53012	0.125986	NA
GSTCD	52.92128	-0.08725	0.067948	-1.28413	0.199097	NA
PPARGC1A	245.564	0.087252	0.061174	1.426279	0.153788	NA
RELB	3.09986	0.087249	0.074776	1.166817	0.243284	NA
ZC3H12D	5.781841	0.087234	0.083367	1.04638	0.295386	NA
LOC65443	2.543775	0.087232	0.064533	1.35175	0.176455	NA
RUSC2	446.7147	0.087223	0.029872	2.919908	0.003501	0.139132
IPW	539.933	-0.08722	0.056414	-1.54609	0.122082	0.476542
ZNF562	208.8002	0.087212	0.043151	2.021086	0.043271	NA
MIR3612	1.69526	0.087198	0.062692	1.390878	0.164262	NA
WDR78	17.28438	-0.08719	0.082583	-1.05583	0.291045	NA
C9orf78	178.7444	0.087189	0.060323	1.445369	0.148354	NA
SNORD116	48.21523	-0.08718	0.0846	-1.03047	0.302791	NA
NKTR	1715.972	-0.08716	0.052796	-1.65094	0.09875	0.435155
SFMBT2	247.0501	-0.08714	0.055477	-1.57069	0.116255	NA
LOC73022	1.575882	0.087133	0.055375	1.573524	0.115598	NA
GRN	44.52017	-0.08711	0.065143	-1.33716	0.181171	NA
ALDH3B1	4.401723	-0.08711	0.082009	-1.06216	0.288164	NA
FRMD4A	260.9214	-0.08708	0.047708	-1.82519	0.067973	NA
BCAS4	13.62631	-0.08707	0.081935	-1.06268	0.287927	NA
FAS	10.31293	-0.08707	0.079061	-1.10129	0.270769	NA
FAM86HP	4.422021	0.087067	0.07968	1.092717	0.274518	NA
SRP68	205.0437	0.08705	0.048245	1.804342	0.071178	NA
FLJ42351	4.133968	0.08704	0.081295	1.070674	0.284316	NA
BTN3A2	22.41047	-0.08702	0.08386	-1.03769	0.299414	NA
LOC25688	4.845101	0.087019	0.081373	1.069387	0.284895	NA
LARP1B	226.3273	0.087017	0.050109	1.736542	0.082468	NA
MAST1	938.0601	0.087	0.078825	1.103707	0.26972	0.610306
GEN1	23.15635	-0.087	0.079006	-1.10116	0.270826	NA
SYNM	457.1063	-0.087	0.081232	-1.07098	0.28418	0.618912

MIR4534	1.634391	-0.08698	0.062389	-1.39423	0.163249	NA
COL14A1	5.813614	0.08696	0.079744	1.090487	0.275498	NA
EIF4B	973.8751	-0.08696	0.066396	-1.3097	0.190299	0.54182
TEKT1	0.519968	-0.08696	0.040382	-2.15336	0.031291	NA
NMUR2	3.462443	-0.08695	0.070158	-1.23936	0.215214	NA
BRPF1	151.9691	0.086919	0.062013	1.401615	0.16103	NA
PDIA2	329.2124	-0.08692	0.076117	-1.1419	0.253496	NA
PGAM5	89.32402	0.086904	0.057754	1.504739	0.132391	NA
NINL	454.6449	-0.0869	0.069278	-1.25436	0.209711	0.558818
FEM1A	111.341	0.08689	0.048931	1.775761	0.075772	NA
ZBP1	0.4541	0.086852	0.037587	2.31067	0.020851	NA
SELRC1	32.6932	0.086849	0.081594	1.06441	0.287143	NA
PRR14L	684.099	0.086842	0.037138	2.338351	0.019369	0.233356
SLC26A11	22.15083	-0.08684	0.084939	-1.02239	0.306594	NA
MIR3115	1.11469	-0.08683	0.057835	-1.50128	0.133283	NA
ABCC5-AS	7.798073	-0.08682	0.084715	-1.0249	0.305409	NA
ATXN1L	140.7503	0.086824	0.049413	1.757103	0.0789	NA
ERAL1	78.00302	0.086804	0.052751	1.645543	0.099858	NA
RRAGB	97.17723	-0.08679	0.051277	-1.69267	0.090519	NA
LOC10050	2.871511	0.086788	0.076402	1.135948	0.255978	NA
RAB33B	111.3704	0.086785	0.059395	1.461143	0.143976	NA
MIR563	1.052638	0.086771	0.056321	1.540662	0.123399	NA
CHRM2	1.867619	0.086756	0.063422	1.367922	0.171337	NA
TAPT1-AS1	35.75735	-0.08675	0.075952	-1.14223	0.253359	NA
DTNA	1467.263	-0.08675	0.057937	-1.49729	0.134317	0.487416
ARSJ	113.2765	0.086747	0.079221	1.094999	0.273517	NA
VIPAS39	70.09224	-0.08672	0.063251	-1.37111	0.170342	NA
CCDC149	198.5436	0.086719	0.048139	1.801452	0.071632	NA
LOC38869	13.07548	0.086717	0.082561	1.050328	0.293567	NA
ATP6V1A	433.568	0.086712	0.06771	1.280635	0.200322	0.547633
OR52I2	0.784186	-0.0867	0.049427	-1.75413	0.079408	NA
CLN3	32.84148	-0.08669	0.075651	-1.14589	0.251839	NA
FXN	87.06312	-0.08667	0.05442	-1.59268	0.111232	NA
GNE	161.7092	0.086674	0.046846	1.850161	0.06429	NA
ITM2C	201.5032	-0.08666	0.079433	-1.09093	0.275306	NA
C7orf43	53.98143	-0.08665	0.070533	-1.22848	0.219268	NA
SERPINA1	12.11379	-0.08664	0.060021	-1.44349	0.148884	NA
EPHB1	421.7945	0.08663	0.055425	1.563008	0.118051	0.472599
CYP39A1	30.22008	-0.08661	0.08481	-1.02125	0.307136	NA
FIGNL1	92.59685	0.086604	0.065849	1.315191	0.188446	NA
RPL23A	334.3653	-0.08659	0.068567	-1.26288	0.206631	NA
MIS18BP1	45.30126	-0.08659	0.082247	-1.05276	0.29245	NA
TAOK1	1283.309	0.086585	0.036753	2.355856	0.01848	0.231397
RBL2	598.1947	-0.08657	0.046843	-1.84811	0.064586	0.375222
MIR7-3	0.625795	0.086564	0.045986	1.882385	0.059784	NA
ARHGEF6	90.51005	-0.08655	0.072642	-1.19152	0.23345	NA
THUMPD1	279.3302	0.086551	0.043463	1.991362	0.046441	NA

HCG11	98.3479	0.086549	0.068211	1.268855	0.204493	NA
TTLL6	2.106948	-0.08651	0.068784	-1.25766	0.208515	NA
VAT1	48.89356	-0.0865	0.070159	-1.23291	0.21761	NA
LRP3	121.1038	0.086496	0.058234	1.485327	0.137457	NA
ASPN	31.513	-0.08648	0.079283	-1.09084	0.275344	NA
SPDYE6	10.21873	-0.08648	0.08394	-1.03031	0.302864	NA
CAMK2N1	279.1853	0.086468	0.084843	1.019154	0.30813	NA
ATP5G2	65.31224	-0.08646	0.076809	-1.12569	0.260295	NA
DOC2A	135.4063	0.086433	0.062375	1.385692	0.165841	NA
RPS27	238.8584	-0.08642	0.071755	-1.20436	0.22845	NA
MRPL42P5	7.291997	-0.08641	0.08314	-1.03939	0.298625	NA
S100A12	0.913125	-0.08641	0.039899	-2.1657	0.030334	NA
ZNF785	99.96838	-0.08639	0.058599	-1.47428	0.140405	NA
LMOD2	0.868911	0.086379	0.04994	1.729644	0.083694	NA
HHIPL2	1.078805	0.086377	0.054085	1.597069	0.11025	NA
SH3GL3	131.5836	-0.08638	0.050783	-1.70089	0.088963	NA
ADAMTS7	5.196147	0.086375	0.079466	1.086944	0.277061	NA
ZNF564	184.8085	0.086359	0.058292	1.481485	0.138477	NA
GTDC2	130.6318	0.086357	0.053733	1.607161	0.108019	NA
CIDEB	61.05432	0.086344	0.063862	1.352037	0.176364	NA
HENMT1	14.34836	0.086343	0.083942	1.028607	0.303664	NA
TTC23	29.12461	-0.08634	0.073455	-1.17542	0.239828	NA
TNFSF13B	10.77742	-0.0863	0.084653	-1.01945	0.30799	NA
LOC10050	3.094091	-0.08629	0.070239	-1.22847	0.219269	NA
ZNF598	124.7891	0.086286	0.051855	1.663968	0.096119	NA
ZNF385D	185.5178	0.086279	0.053467	1.613691	0.106595	NA
PDHA1	263.0112	0.086278	0.049406	1.746293	0.08076	NA
FGF14-AS2	122.7918	-0.08628	0.067556	-1.2771	0.201568	NA
BCAS3	175.478	-0.08627	0.052186	-1.65304	0.098322	NA
FLYWCH2	51.24087	0.086254	0.071449	1.207217	0.227348	NA
PIWIL4	31.05083	-0.08625	0.078217	-1.10265	0.27018	NA
PLXNA3	334.9349	0.086233	0.07362	1.171334	0.241465	0.590833
CASP8AP2	523.3252	0.086229	0.039706	2.171659	0.029881	0.275534
ZNF79	70.09804	0.086223	0.070398	1.224793	0.220653	NA
BRD2	808.9304	0.086223	0.040513	2.128261	0.033315	0.286653
COX6C	251.4984	-0.08621	0.07048	-1.22317	0.221266	NA
BNIP3L	310.4629	-0.08619	0.058906	-1.46322	0.143408	NA
XKR4	65.22242	0.086169	0.072971	1.180879	0.237651	NA
OR6V1	3.616455	-0.08617	0.074641	-1.15442	0.248329	NA
LOC65316	13.91918	-0.08615	0.082997	-1.038	0.299271	NA
SDPR	40.42723	0.086142	0.084775	1.016128	0.309569	NA
PRELP	38.31148	-0.08613	0.083793	-1.02784	0.304025	NA
KCNJ8	4.586058	-0.08611	0.078195	-1.10125	0.270789	NA
AQP1	259.4703	-0.08611	0.084764	-1.01583	0.309711	NA
FAM219B	242.113	-0.0861	0.035979	-2.39315	0.016704	NA
METTL16	164.0656	0.086095	0.046663	1.845029	0.065033	NA
PLCXD3	600.9191	0.086092	0.068388	1.258879	0.208074	0.55642

DCTN5	370.6905	0.086089	0.044438	1.937288	0.05271	0.355768
PDZD8	284.5946	0.086087	0.046465	1.852727	0.063922	NA
ATP1A2	2460.905	-0.08608	0.076918	-1.11912	0.263088	0.60594
C1orf145	21.5127	-0.08608	0.084227	-1.022	0.306779	NA
CLINT1	190.713	0.086066	0.04089	2.104823	0.035307	NA
IFIH1	11.51529	0.086058	0.08288	1.038343	0.29911	NA
LAT	19.64711	-0.08603	0.082859	-1.0383	0.299129	NA
C6orf195	12.54064	-0.08603	0.084414	-1.01916	0.308125	NA
IFNGR1	107.509	-0.08603	0.058144	-1.47955	0.138992	NA
LOC44090	1.791187	-0.08602	0.046379	-1.85476	0.06363	NA
DSCAM	71.8783	-0.08601	0.072776	-1.18181	0.237283	NA
TICRR	3.689154	-0.08601	0.07481	-1.14966	0.250283	NA
UGT8	53.32647	0.086005	0.08286	1.037963	0.299287	NA
TMOD2	1555.619	0.086004	0.04468	1.924899	0.054242	0.357364
TGM4	4.335798	-0.08599	0.079206	-1.08561	0.27765	NA
LOC28588	0.510429	-0.08598	0.03998	-2.15063	0.031505	NA
SSR4	21.925	-0.08598	0.080903	-1.06277	0.287887	NA
RPGRIP1	3.104989	-0.08595	0.075883	-1.13271	0.257335	NA
SEPHS2	57.05209	0.085945	0.073387	1.171122	0.24155	NA
SNORD116	83.43415	-0.08592	0.084213	-1.02027	0.307601	NA
TUSC2	45.07307	-0.0859	0.070272	-1.22238	0.221565	NA
SNORD59A	27.74274	-0.08588	0.084643	-1.01467	0.310264	NA
MCOLN1	40.47187	-0.08588	0.070435	-1.21931	0.222728	NA
ANKRD27	219.396	-0.08586	0.052961	-1.6213	0.104954	NA
PITPNM1	195.2068	0.085857	0.060724	1.413889	0.157394	NA
NUDT1	5.576309	-0.08585	0.08384	-1.02399	0.305842	NA
PDCD10	108.6723	-0.08584	0.052616	-1.63149	0.102788	NA
CLDN4	7.255211	-0.08583	0.079254	-1.08301	0.278803	NA
CRTAP	234.501	-0.08582	0.046505	-1.84547	0.064969	NA
RASGRP4	40.91753	0.085819	0.080704	1.063386	0.287607	NA
LRFN1	51.10028	0.085797	0.064416	1.331918	0.182887	NA
COX17	72.52897	0.085753	0.070282	1.220129	0.222416	NA
PEG10	1369.218	0.085752	0.067963	1.261742	0.207042	0.55642
CCDC8	13.57409	0.085752	0.08429	1.017347	0.308989	NA
FNIP1	356.7268	-0.08575	0.036774	-2.33182	0.01971	0.234966
MIR4493	1.959537	-0.08574	0.069179	-1.23939	0.215201	NA
SIPA1L2	60.86854	-0.08571	0.083869	-1.02196	0.306799	NA
MESTIT1	5.580304	-0.08571	0.080188	-1.06883	0.285147	NA
SLC39A10	185.3743	0.085701	0.055948	1.531786	0.125575	NA
KCND1	69.65708	0.085684	0.075583	1.13363	0.25695	NA
RENBP	6.470623	0.085673	0.081691	1.048745	0.294295	NA
ZNF850	45.84351	0.085668	0.074429	1.15101	0.249728	NA
FSTL1	54.87352	-0.08567	0.080188	-1.06831	0.285382	NA
C16orf87	21.76328	0.08566	0.076455	1.120404	0.262542	NA
NAV2-AS4	6.854773	-0.08564	0.082664	-1.03602	0.300194	NA
CDH11	111.4066	0.08564	0.070339	1.217533	0.223401	NA
TRABD2B	14.0475	-0.08563	0.084531	-1.01304	0.311043	NA

IER3	8.62553	0.085621	0.083373	1.026967	0.304436	NA
LOC10050	28.92085	-0.0856	0.077567	-1.10359	0.26977	NA
KAL1	17.17967	-0.08558	0.084221	-1.0161	0.309582	NA
PRKRA	113.7994	-0.08558	0.058582	-1.4608	0.14407	NA
OVCH2	5.868033	0.085574	0.077319	1.106757	0.268399	NA
GJD3	5.86003	0.085574	0.080241	1.066457	0.286217	NA
IFITM2	10.35335	-0.08557	0.076833	-1.1137	0.265408	NA
ARR3	4.949617	-0.08556	0.081155	-1.05426	0.291763	NA
KRT19	39.20787	0.085545	0.081432	1.050498	0.293489	NA
UBALD1	59.24978	-0.08554	0.066622	-1.284	0.199143	NA
PRMT8	57.99903	-0.08551	0.078129	-1.09446	0.273754	NA
SLC30A5	88.34207	0.085507	0.056756	1.506581	0.131918	NA
SH3TC2	44.06958	-0.0855	0.084915	-1.00693	0.31397	NA
C17orf58	26.43362	0.085495	0.079617	1.073826	0.282901	NA
GYG1	52.33929	-0.08549	0.077893	-1.09754	0.272405	NA
STAM	223.4428	0.085484	0.046248	1.848363	0.06455	NA
ANKRD13B	188.0383	-0.08548	0.049892	-1.71327	0.086663	NA
GLT25D2	186.0715	0.085465	0.052723	1.621007	0.105016	NA
ADNP	565.4917	0.085463	0.036254	2.35735	0.018406	0.231397
LPCAT2	13.14575	-0.08545	0.084087	-1.01615	0.309556	NA
ZDHHC13	50.57069	-0.08542	0.060126	-1.42073	0.155395	NA
SCARNA23	2.395231	-0.08542	0.07036	-1.21401	0.224742	NA
PLEKHA4	7.872046	-0.08541	0.07821	-1.09209	0.274795	NA
BARX2	5.105559	-0.08541	0.08254	-1.03479	0.300768	NA
ODF3L1	4.441205	0.085386	0.079019	1.08057	0.279889	NA
KCTD5	34.76926	0.085366	0.066299	1.287596	0.197887	NA
SLC50A1	19.75165	0.085365	0.081568	1.046547	0.295308	NA
SDC1	6.053081	-0.08534	0.075697	-1.12736	0.259592	NA
H2AFZ	92.42471	-0.08534	0.069186	-1.23345	0.217407	NA
RTEL1-TNF	6.043631	-0.08533	0.080369	-1.06168	0.288382	NA
TIMM13	37.75278	-0.08532	0.070839	-1.20446	0.228413	NA
ST14	6.467748	-0.0853	0.082901	-1.02897	0.303495	NA
KLK8	0.602367	-0.08529	0.045251	-1.88491	0.059442	NA
APP	2211.28	0.085288	0.061495	1.386912	0.165469	0.52098
ZNF142	186.8796	0.085287	0.04285	1.990362	0.046551	NA
CHAMP1	142.7658	0.085279	0.05785	1.474137	0.140445	NA
DCAF13	56.43886	0.085266	0.065116	1.309448	0.190383	NA
TLR9	18.67807	0.085202	0.084782	1.004946	0.314923	NA
MIR30E	0.970815	-0.08519	0.053451	-1.59379	0.110984	NA
PHF20	285.6249	0.08517	0.038821	2.193921	0.028241	NA
GAMT	39.84229	-0.08515	0.067275	-1.26565	0.205637	NA
ZNF138	322.5758	0.085146	0.056036	1.519483	0.128641	NA
FBXO41	383.8321	0.085144	0.045157	1.885507	0.059361	0.365363
ARG1	3.673707	-0.08514	0.078822	-1.0801	0.280097	NA
AMPD2	149.2986	0.085135	0.061414	1.386252	0.16567	NA
ABHD3	35.4419	0.085075	0.079325	1.07249	0.2835	NA
PCDHGB3	80.30654	0.085071	0.082702	1.028642	0.303648	NA

ACSL3	291.4414	0.085067	0.06009	1.415663	0.156874	NA
U2AF1L4	19.59744	-0.08507	0.083274	-1.02152	0.307006	NA
FGL1	0.666842	-0.08507	0.044468	-1.91297	0.055752	NA
CREM	50.36809	-0.08506	0.065363	-1.3014	0.193121	NA
ZKSCAN4	29.72044	0.085046	0.073713	1.153737	0.248608	NA
TMEM59L	299.6509	0.084985	0.05922	1.435087	0.151262	NA
ALOX12B	5.195373	-0.08497	0.082774	-1.02648	0.304666	NA
RNFT2	69.70142	0.084944	0.068484	1.240353	0.214845	NA
C14orf79	12.55921	0.084935	0.084523	1.004874	0.314957	NA
SNUPN	51.10943	0.084931	0.061104	1.389933	0.164549	NA
HDAC7	99.36455	-0.08492	0.067194	-1.26375	0.206321	NA
SMEK1	436.3675	0.084906	0.035508	2.391198	0.016793	0.224654
GNA15	4.443331	0.0849	0.073739	1.151359	0.249585	NA
CCDC6	232.2318	0.084899	0.045548	1.863929	0.062332	NA
NPPA-AS1	4.477563	-0.08489	0.075526	-1.12393	0.261044	NA
SNORD115	1.740959	-0.08488	0.060107	-1.41223	0.157881	NA
ORC1	7.953812	-0.08487	0.084938	-0.99923	0.317684	NA
GTF2IRD2	2.735828	-0.08485	0.074035	-1.14606	0.251771	NA
SP6	4.708228	0.084847	0.078917	1.075133	0.282315	NA
TTC3	2261.711	0.084843	0.040522	2.093756	0.036282	0.292449
PRSS16	2.019024	-0.08484	0.067956	-1.24849	0.21185	NA
TMEM63C	391.2203	-0.08484	0.068104	-1.24572	0.212866	0.562782
YWHAB	651.0764	0.084826	0.066893	1.268083	0.204768	0.553506
KIF4B	10.32864	0.084798	0.084908	0.998701	0.31794	NA
GLUL	1284.025	-0.0848	0.076809	-1.10399	0.269599	0.610306
METTL21B	18.65394	-0.08479	0.08166	-1.03836	0.299104	NA
ADAM10	502.9825	0.084788	0.044846	1.890645	0.058672	0.36517
TBL1XR1	626.6687	0.084769	0.050183	1.689217	0.091178	0.416367
KIFC1	1.681737	-0.08477	0.063782	-1.329	0.183846	NA
TYK2	174.3516	-0.08476	0.052108	-1.62659	0.103823	NA
TYRO3P	6.458753	-0.08474	0.082126	-1.03178	0.302174	NA
SLC7A8	236.3458	-0.08473	0.076172	-1.11239	0.265969	NA
UNC5D	44.88686	-0.08473	0.084906	-0.99787	0.31834	NA
HMGB4	2.714353	-0.08472	0.076209	-1.11164	0.266291	NA
DCUN1D5	646.6994	0.084698	0.055185	1.5348	0.124833	0.47732
IGSF9B	734.9491	0.084678	0.059558	1.421774	0.155092	0.509106
GMNC	0.671579	-0.08466	0.040857	-2.07214	0.038252	NA
DYNLL2	300.3686	0.08466	0.05715	1.481368	0.138509	NA
ATF7IP	700.696	0.084648	0.033329	2.539738	0.011094	0.201925
EPHB4	13.3152	-0.08464	0.084911	-0.99685	0.318839	NA
FAM171B	385.6905	0.084628	0.057445	1.473203	0.140696	0.492546
FAM175A	27.48856	0.084626	0.074592	1.134514	0.256579	NA
MTRNR2L	944.9532	-0.08461	0.064015	-1.3217	0.186266	0.537444
SH3BP4	31.06898	-0.0846	0.081454	-1.03866	0.298964	NA
SOX3	0.811971	-0.08459	0.050371	-1.67938	0.093079	NA
SMIM16	13.01922	0.084592	0.084294	1.00353	0.315605	NA
MRPL30	165.7557	-0.08459	0.046132	-1.83363	0.066709	NA

DUOXA1	2.816837	-0.08459	0.064323	-1.31506	0.18849	NA
TTC38	31.54655	0.084587	0.082588	1.024197	0.305742	NA
ZNF467	21.00805	0.084579	0.082696	1.022768	0.306418	NA
TAF8	54.42431	-0.08457	0.065469	-1.29179	0.196431	NA
CYTIP	1.503327	-0.08456	0.062048	-1.3628	0.172945	NA
DCAF13P3	25.48493	-0.08455	0.07923	-1.06716	0.285901	NA
ADAMTSL5	10.02802	-0.08454	0.084892	-0.99586	0.31932	NA
POU3F3	66.93348	0.084517	0.07698	1.097914	0.272242	NA
F7	12.87537	-0.08451	0.084927	-0.99505	0.31971	NA
WDR93	33.69514	-0.0845	0.075565	-1.1183	0.263438	NA
SLC26A6	31.41369	-0.0845	0.073295	-1.15282	0.248983	NA
CNBP	835.2432	-0.08449	0.054347	-1.55472	0.120013	0.472798
HSD17B3	2.747365	-0.08448	0.063909	-1.3219	0.1862	NA
MAP3K19	2.166782	-0.08447	0.058969	-1.43248	0.152007	NA
TNFRSF11	2.142533	-0.08444	0.067817	-1.24507	0.213104	NA
CYFIP2	1505.832	0.084426	0.04884	1.728614	0.083878	0.403363
LUC7L	265.5449	-0.08441	0.0529	-1.59557	0.110586	NA
TMEM208	36.5977	-0.08439	0.071327	-1.18312	0.236763	NA
ALK	18.47986	-0.08438	0.08011	-1.05337	0.292173	NA
EXOC2	161.7244	0.084384	0.051234	1.647042	0.099549	NA
POFUT2	57.91	-0.08437	0.059963	-1.40698	0.159432	NA
KBTBD6	141.3717	0.084354	0.063836	1.321411	0.186364	NA
DCLK3	2.335147	0.084337	0.059815	1.409958	0.158552	NA
FAM86B2	9.89506	0.084327	0.079288	1.063548	0.287533	NA
KCNJ3	682.8312	0.084311	0.048064	1.754144	0.079406	0.397742
C10orf25	18.7836	0.084303	0.084765	0.994556	0.319952	NA
ARL13B	63.98526	-0.08428	0.056268	-1.49792	0.134154	NA
DLG5-AS1	5.91181	-0.08427	0.084068	-1.0024	0.316148	NA
CRABP1	3.882831	-0.08427	0.067328	-1.25164	0.210703	NA
PPM1E	147.0695	0.084253	0.054092	1.557579	0.119333	NA
ZSCAN31	105.5918	0.08424	0.084316	0.999099	0.317747	NA
PDIA3	186.6816	0.08423	0.058404	1.442212	0.149242	NA
ARHGAP11	7.156421	0.084221	0.084659	0.994821	0.319823	NA
MRPL27	42.84028	-0.08422	0.067075	-1.25556	0.209275	NA
LAMB2P1	7.350469	-0.0842	0.083999	-1.0024	0.316149	NA
TMEM216	16.7699	0.084178	0.083374	1.009635	0.31267	NA
SLC25A52	0.50433	-0.08417	0.041119	-2.047	0.040658	NA
GPATCH4	116.5843	0.084166	0.055726	1.510367	0.13095	NA
CCDC159	43.22996	-0.08416	0.073767	-1.14085	0.253933	NA
QPCT	0.885588	-0.08416	0.052562	-1.60108	0.10936	NA
NFS1	52.07618	0.084156	0.064949	1.295717	0.195073	NA
LGR5	3.485171	0.084153	0.075718	1.111391	0.2664	NA
MKI67IP	43.15332	-0.08414	0.064626	-1.30199	0.192921	NA
WDR54	22.47182	-0.08414	0.074256	-1.13313	0.257162	NA
COX18	33.70341	-0.08413	0.079752	-1.05488	0.291481	NA
HAVCR2	14.82542	-0.08413	0.083225	-1.01085	0.31209	NA
RPL7L1	31.1262	0.084113	0.077771	1.081539	0.279457	NA

STAU1	312.2449	0.084105	0.04559	1.844816	0.065064	NA
TUBA1B	270.5343	0.084103	0.073946	1.137346	0.255394	NA
EZH1	681.3141	-0.08409	0.057004	-1.47523	0.140151	0.492301
ANKRD18B	279.2186	0.084092	0.072759	1.155761	0.247779	NA
PPP2R5A	73.73972	-0.08407	0.065673	-1.28014	0.200496	NA
SOX5	76.36595	-0.08407	0.069967	-1.20155	0.229539	NA
DNALI1	43.48902	-0.08405	0.073198	-1.14827	0.250857	NA
SRP9	137.4278	-0.08405	0.068308	-1.23047	0.218522	NA
KCNJ11	51.60968	0.084013	0.07461	1.126021	0.260157	NA
RUNX1T1	1239.438	0.084012	0.041226	2.037841	0.041566	0.315928
PFN4	4.844682	-0.084	0.081188	-1.03463	0.30084	NA
SLFN5	34.37675	0.083995	0.079935	1.050787	0.293356	NA
SNORD91B	13.51254	-0.08399	0.080492	-1.04344	0.296743	NA
SLC7A10	7.312377	-0.08398	0.084592	-0.99278	0.320816	NA
LOC72902	18.45484	0.083973	0.08176	1.027072	0.304387	NA
SMARCB1	255.9198	0.083952	0.063831	1.315224	0.188435	NA
ZNF681	89.59167	0.083917	0.066624	1.259557	0.207829	NA
PTPRB	356.1058	0.08391	0.062857	1.334922	0.181902	0.530978
OTUB2	25.14017	-0.0839	0.084159	-0.99697	0.318778	NA
RANBP17	72.61077	0.083881	0.080229	1.045517	0.295784	NA
RGS16	13.27328	-0.08388	0.084334	-0.99457	0.319946	NA
UNC13C	4944.265	0.083847	0.047015	1.783406	0.07452	0.390538
C5orf15	60.7537	0.083832	0.059347	1.412565	0.157784	NA
LOC10013	24.60453	-0.08383	0.081712	-1.02592	0.304927	NA
CENPM	4.028168	0.083827	0.077722	1.078556	0.280786	NA
ARRB2	174.9454	-0.08382	0.044423	-1.88679	0.059189	NA
ELK4	391.4509	0.083807	0.039652	2.113546	0.034554	0.288416
SLC16A5	3.5402	-0.08379	0.076878	-1.08986	0.275775	NA
BTBD6	129.9966	0.083772	0.069025	1.213657	0.224879	NA
NSF	384.0509	0.08377	0.074	1.132023	0.257625	0.59833
SNORA64	5.542494	-0.08376	0.080766	-1.03706	0.299709	NA
SERF2	44.47714	0.083746	0.066857	1.252625	0.210342	NA
WFS1	300.7145	0.083736	0.082028	1.020824	0.307338	NA
LANCL2	107.5025	0.083726	0.069191	1.210061	0.226255	NA
L1CAM	1032.192	0.083702	0.046665	1.793665	0.072867	0.389252
GRIN2D	16.48858	-0.0837	0.08445	-0.99111	0.32163	NA
TDRD6	363.0539	-0.08369	0.068453	-1.22264	0.221464	0.570018
STPG2	5.238883	-0.08369	0.082445	-1.01507	0.310074	NA
LOC10012	45.60035	0.083671	0.076065	1.099991	0.271336	NA
SPRED1	172.9775	0.08367	0.075962	1.101467	0.270693	NA
CYCSP52	2.843821	0.083663	0.07208	1.160692	0.245767	NA
TRAK2	372.3488	0.083659	0.062954	1.328905	0.183879	0.534406
SLIT1	59.6411	0.083648	0.083511	1.001643	0.316516	NA
GPT2	98.29399	-0.08364	0.059014	-1.41736	0.156377	NA
DGCR14	66.00235	0.083635	0.065375	1.279303	0.20079	NA
PTH1R	22.09831	-0.08362	0.084929	-0.98461	0.324815	NA
GADD45B	41.39711	-0.08361	0.084324	-0.99153	0.321426	NA

NAP1L5	227.832	0.083602	0.0765	1.092835	0.274466	NA
MIR940	0.969188	-0.08359	0.054361	-1.53775	0.124109	NA
ZNF230	54.031	0.08358	0.061309	1.363263	0.1728	NA
DERL2	29.3515	0.083564	0.074384	1.123413	0.261262	NA
COQ7	67.29137	-0.08356	0.060669	-1.37737	0.168397	NA
SDR16C5	7.158766	0.083538	0.080326	1.039986	0.298347	NA
HSP90AB1	2725.588	0.083523	0.074657	1.11876	0.263243	0.60594
SLC17A9	1.803833	-0.08351	0.064209	-1.30065	0.193378	NA
SPRY2	211.9275	0.083506	0.054659	1.52776	0.126572	NA
FGF1	209.607	-0.0835	0.0662	-1.26131	0.207197	NA
DPY19L2P	87.42844	0.083491	0.059746	1.397427	0.162285	NA
AARS2	177.0447	-0.08345	0.054868	-1.52098	0.128264	NA
ZFX-AS1	1.736759	-0.08343	0.067703	-1.23233	0.217825	NA
TSR1	127.4777	-0.08342	0.046763	-1.78387	0.074445	NA
MAPK4	321.4357	0.083415	0.062431	1.336102	0.181516	NA
XRCC6BP1	12.06656	-0.08341	0.083073	-1.0041	0.31533	NA
ZNF85	49.3779	-0.08341	0.067902	-1.22836	0.219313	NA
FLJ40292	13.1447	0.083392	0.083292	1.001201	0.316729	NA
FAM86FP	33.39193	-0.08339	0.080886	-1.03099	0.302547	NA
PRDM12	1.220189	-0.08337	0.055036	-1.51491	0.129796	NA
AMY2B	117.6782	-0.08337	0.068949	-1.2092	0.226586	NA
GABRB2	1672.354	0.083358	0.051796	1.609353	0.107539	0.452973
LOC10013	10.94885	-0.08336	0.083566	-0.99748	0.318532	NA
NCF1	1.193593	-0.08335	0.056126	-1.4851	0.137518	NA
CD200R1	0.767832	-0.08335	0.047105	-1.76937	0.076832	NA
DCP1A	227.0897	0.083346	0.044625	1.867677	0.061807	NA
KLKB1	13.1158	0.083325	0.084471	0.986425	0.323924	NA
GLIPR1L1	18.48549	-0.08332	0.081066	-1.0278	0.304043	NA
RBMX	435.0043	-0.08329	0.05056	-1.6474	0.099476	0.436963
LOC10013	293.5646	0.083285	0.078349	1.063002	0.287781	NA
KIAA2013	42.68158	0.08328	0.062245	1.337953	0.180912	NA
CCNE2	45.97762	-0.08328	0.06803	-1.22412	0.220906	NA
MICA	43.16746	0.083272	0.084794	0.98205	0.326075	NA
APC	1813.327	0.083264	0.04946	1.683459	0.092286	0.419736
ITPKB	398.0976	-0.08326	0.072504	-1.1484	0.250804	0.592704
PRMT5	55.3365	0.083262	0.06349	1.311425	0.189714	NA
FLJ42627	27.33251	-0.08326	0.084938	-0.9802	0.326986	NA
TSSC4	15.67065	0.08323	0.082443	1.009541	0.312715	NA
RILP	26.41889	-0.08323	0.079533	-1.04647	0.295342	NA
IGSF3	17.76954	-0.08323	0.084937	-0.97985	0.327158	NA
IGSF10	11.37661	0.083222	0.084883	0.980428	0.326875	NA
ATXN10	246.9181	0.083199	0.0547	1.521013	0.128257	NA
LASP1	215.1095	0.083195	0.055131	1.509054	0.131285	NA
MAP2K1	224.7871	0.083187	0.061633	1.349706	0.17711	NA
ZMIZ1	563.377	0.083186	0.046743	1.779645	0.075134	0.391143
SLC52A3	7.239952	-0.08318	0.081964	-1.01488	0.310164	NA
PMS2P5	42.46396	0.083173	0.074241	1.120302	0.262585	NA

OFD1	254.3266	-0.08317	0.06179	-1.34603	0.178293	NA
MAK16	55.66995	0.08313	0.075614	1.099399	0.271594	NA
RIMS4	83.63257	0.083123	0.06617	1.256192	0.209046	NA
BSPRY	42.24068	-0.0831	0.084686	-0.98122	0.326483	NA
CHRNA3	22.20225	-0.08307	0.083112	-0.9995	0.317554	NA
SKIDA1	23.34321	-0.08305	0.0794	-1.04595	0.295583	NA
PCDHB17	9.303026	-0.08304	0.084507	-0.98267	0.325771	NA
NUF2	1.321692	-0.08304	0.060477	-1.37307	0.169731	NA
LOC10012	1.456748	-0.08302	0.053027	-1.5657	0.117419	NA
TNNT1	2.87342	-0.08302	0.073938	-1.12277	0.261533	NA
RPL37	346.5621	-0.08302	0.06254	-1.32739	0.184381	0.534729
KLF4	9.411694	0.083	0.075463	1.099876	0.271386	NA
KDELR2	113.5471	0.082982	0.048698	1.704024	0.088377	NA
MYD88	27.54462	0.082969	0.073336	1.131356	0.257905	NA
LOC10013	12.8858	-0.08295	0.084774	-0.9785	0.327829	NA
NADSYN1	111.2713	-0.08294	0.062746	-1.32177	0.186244	NA
RAB14	281.8192	0.082928	0.053966	1.536666	0.124375	NA
FBXO40	1.21532	-0.08292	0.055436	-1.49571	0.134729	NA
GNB1	1293.261	0.082913	0.05934	1.397269	0.162333	0.519328
ZNRD1-AS	45.3407	-0.0829	0.077841	-1.06496	0.286893	NA
SEC14L2	125.5042	-0.08287	0.060007	-1.38098	0.167286	NA
JAKMIP3	404.5128	0.082851	0.071742	1.154853	0.248151	0.592704
UBE4B	623.8662	-0.08283	0.036426	-2.27397	0.022968	0.23963
KIAA2018	716.1792	0.08283	0.037938	2.183273	0.029016	0.271573
TEX264	15.92469	0.082794	0.080189	1.032489	0.301843	NA
CORO2A	101.1002	0.082789	0.065087	1.271971	0.203383	NA
RPL39	18.09171	0.082774	0.084938	0.974532	0.329792	NA
ZNF69	12.90099	0.082772	0.084359	0.981188	0.3265	NA
DHPS	233.8387	-0.08276	0.050734	-1.63117	0.102854	NA
CA8	232.7934	-0.08275	0.083834	-0.98711	0.323587	NA
FOXJ3	540.5294	0.082751	0.037355	2.215265	0.026742	0.259072
STARD3NL	51.73683	-0.08273	0.07563	-1.09389	0.274004	NA
ATRNL1	87.68153	-0.08271	0.081556	-1.0141	0.310534	NA
DKFZp686	18.69223	-0.0827	0.079008	-1.04673	0.295223	NA
SNORA42	19.23133	-0.08268	0.083837	-0.98622	0.324026	NA
AAAS	52.40311	0.082673	0.06142	1.346038	0.17829	NA
SNORA48	34.38502	-0.08266	0.083678	-0.98784	0.323231	NA
RNF220	155.0993	-0.08266	0.054893	-1.50581	0.132115	NA
PANK1	168.6848	0.082655	0.059943	1.3789	0.167925	NA
CREB3	59.87706	0.082631	0.059558	1.3874	0.16532	NA
STK17B	63.94782	-0.08263	0.083555	-0.98892	0.322704	NA
KIAA1586	227.3712	0.082602	0.044473	1.857344	0.063262	NA
MTSS1	634.2196	0.0826	0.042243	1.955375	0.050539	0.34688
SDCCAG3	69.42301	-0.08257	0.060704	-1.36028	0.17374	NA
ANKHD1	14.3568	0.082571	0.082089	1.005874	0.314476	NA
NONO	652.5075	-0.08252	0.054662	-1.50973	0.131113	0.487416
ETV2	5.097921	0.082517	0.081207	1.01613	0.309568	NA

PPP2R2D	306.4757	0.082503	0.051238	1.610176	0.107359	NA
MCPH1	175.4033	0.082502	0.055566	1.484765	0.137606	NA
PRKCG	111.8814	-0.0825	0.084918	-0.97151	0.331293	NA
ZNF385A	75.63601	-0.08249	0.076877	-1.07301	0.283266	NA
FGFR1	1056.48	0.082485	0.061235	1.347027	0.177972	0.530729
GP1BA	55.78841	-0.08248	0.073198	-1.12678	0.259836	NA
PTTG2	5.677954	0.082461	0.083265	0.990342	0.322007	NA
PIAS2	374.2557	-0.08246	0.043494	-1.89586	0.057978	0.364493
MIR194-2	0.891657	-0.08243	0.049386	-1.66918	0.095082	NA
MCAM	410.3235	0.082422	0.071329	1.155527	0.247875	0.592704
EFEMP1	91.05844	-0.08242	0.084117	-0.97979	0.327191	NA
C9orf50	2.045358	0.082387	0.066153	1.245402	0.212984	NA
MEF2D	474.047	0.082387	0.042539	1.936748	0.052776	0.355768
CNP	265.4726	-0.08238	0.07196	-1.14486	0.252269	NA
ZCCHC11	802.6079	-0.08235	0.047375	-1.73822	0.082172	0.401988
EIF6	26.39901	0.082342	0.075924	1.08454	0.278125	NA
GM2A	74.81249	-0.08233	0.06191	-1.3299	0.18355	NA
QKI	1209.863	-0.08233	0.063056	-1.30563	0.191678	0.542492
PDLIM7	9.243984	-0.08232	0.083388	-0.98714	0.323573	NA
LOC44146	1.589026	-0.08231	0.06519	-1.26268	0.206705	NA
SELP	0.907587	-0.08231	0.049316	-1.66897	0.095124	NA
METRN	47.37672	-0.08227	0.070955	-1.15945	0.246273	NA
ZNF713	46.05015	0.082266	0.072324	1.137454	0.255348	NA
GALNT12	97.78664	-0.08226	0.081439	-1.0101	0.312447	NA
CRISPLD1	4.818924	-0.08225	0.068184	-1.20636	0.227678	NA
C10orf111	4.407183	0.082248	0.065876	1.248511	0.211844	NA
ADCY7	58.25037	-0.08224	0.061217	-1.3435	0.179112	NA
CYP2C8	6.706334	-0.08224	0.081625	-1.00748	0.313702	NA
CENPE	4.993874	-0.0822	0.076768	-1.07073	0.284292	NA
SLC7A5P2	183.2254	-0.08218	0.07113	-1.15531	0.247963	NA
OR1L6	0.831853	-0.08218	0.048211	-1.70448	0.088291	NA
LY75	2.620748	-0.08216	0.075232	-1.09205	0.27481	NA
LOC72960	1.797778	-0.08214	0.064612	-1.27135	0.203606	NA
RSL24D1	132.5049	-0.08214	0.077602	-1.05848	0.289839	NA
LDB3	21.14776	-0.08214	0.083671	-0.98165	0.326272	NA
SCD5	660.0031	-0.08213	0.065606	-1.25187	0.210617	0.560498
MEGF11	369.3429	-0.08213	0.053202	-1.54371	0.122659	0.476542
IP6K2	458.106	-0.08211	0.064768	-1.26774	0.204891	0.553506
IRF2BP1	68.8749	0.082103	0.060143	1.365125	0.172214	NA
C9orf139	19.80538	-0.0821	0.084871	-0.96737	0.333359	NA
LOC10050	0.53763	0.082088	0.038228	2.147303	0.031769	NA
FARSA	91.09163	-0.08208	0.052521	-1.56283	0.118094	NA
KLF3	134.143	0.082074	0.055523	1.478184	0.139358	NA
C5	32.56711	0.082069	0.080646	1.017654	0.308842	NA
IGFL4	9.505821	-0.08205	0.084842	-0.96714	0.333473	NA
STK33	40.3885	-0.08205	0.08165	-1.00484	0.314972	NA
MIR545	0.665852	-0.08204	0.040689	-2.01631	0.043767	NA

ACCS	18.97959	-0.08204	0.084898	-0.96628	0.333903	NA
GRM1	697.306	0.082011	0.048343	1.696433	0.089804	0.415114
OSBPL6	213.3155	0.08201	0.054885	1.494229	0.135116	NA
FAM83D	4.20879	0.081998	0.074875	1.095135	0.273457	NA
LYST	1378.301	0.081994	0.026301	3.117537	0.001824	0.101784
B3GNT9	10.95555	0.081993	0.084509	0.970224	0.331935	NA
NDUFB1	45.3112	-0.08199	0.080022	-1.02461	0.305545	NA
ODC1	49.99916	0.081976	0.069589	1.178002	0.238796	NA
TAB2	468.8142	0.081923	0.044199	1.853501	0.063811	0.372503
MTERF	50.99683	0.081921	0.063048	1.299343	0.193826	NA
PYCR2	54.02826	-0.08187	0.070374	-1.16341	0.244665	NA
SH3D21	103.1123	-0.08187	0.066178	-1.23711	0.216047	NA
FTSJD2	208.2457	-0.08185	0.052775	-1.55094	0.120917	NA
MIR199B	3.628748	-0.08185	0.078522	-1.04232	0.297262	NA
CCR10	16.11156	-0.08183	0.084359	-0.96996	0.332067	NA
PITPNC1	231.2037	-0.08181	0.061382	-1.33283	0.182586	NA
HSPA1B	143.6906	-0.08181	0.074603	-1.09663	0.272803	NA
SNORD115	3.580403	-0.08179	0.076656	-1.06697	0.285987	NA
NKAIN1	67.13956	0.081756	0.072764	1.123567	0.261197	NA
DENND5A	499.7441	0.08175	0.039491	2.070075	0.038445	0.300499
SLC12A8	3.829354	-0.08175	0.079261	-1.03135	0.302377	NA
MIR600HG	197.0368	0.081736	0.052118	1.568284	0.116815	NA
AFF2	396.1915	0.081727	0.067414	1.212314	0.225392	0.576749
FAM203A	1.4596	-0.08173	0.064232	-1.27234	0.203253	NA
TMEM198	20.21791	-0.0817	0.079137	-1.03243	0.301872	NA
USP6	12.15273	0.081678	0.084939	0.961611	0.336245	NA
LOC28559	5.316205	0.081666	0.080574	1.013547	0.310799	NA
CRLS1	58.52809	0.081647	0.066158	1.234123	0.217157	NA
CDK14	403.1756	0.081646	0.05028	1.623837	0.104411	0.447883
COPS7A	80.499	0.081644	0.078772	1.036459	0.299988	NA
FAM92A1	70.28192	0.08164	0.057568	1.41816	0.156144	NA
PYROXD2	17.74078	-0.08163	0.084386	-0.96735	0.333371	NA
CDH19	7.878836	-0.08162	0.084433	-0.96666	0.333716	NA
SNORD9	14.02695	-0.08161	0.081217	-1.00484	0.314974	NA
RBMS2	108.4993	-0.08161	0.071157	-1.14684	0.251448	NA
PCDHGB1	71.44228	0.0816	0.082224	0.99241	0.320998	NA
MAG	50.87083	-0.08159	0.083994	-0.97137	0.331365	NA
SULT1B1	2.327295	-0.08155	0.064484	-1.2647	0.205979	NA
LIMD1	16.39383	0.081548	0.08083	1.008887	0.313029	NA
LOC15117	1.442145	-0.08154	0.053992	-1.51019	0.130994	NA
TPRN	170.7614	0.081535	0.063191	1.290285	0.196952	NA
SPTAN1	5428.313	0.081512	0.035737	2.280874	0.022556	0.23963
ECH1	80.30039	-0.0815	0.065074	-1.25246	0.210401	NA
LYPD5	38.9339	0.081488	0.084129	0.968604	0.332743	NA
DTX2P1-U	22.93341	-0.08147	0.08122	-1.00308	0.315824	NA
NIPAL4	18.05142	-0.08145	0.084657	-0.96213	0.335986	NA
C6orf211	77.47766	0.081437	0.062778	1.297227	0.194553	NA

MSL3	70.29434	-0.08143	0.058529	-1.39124	0.164152	NA
WDR36	147.8359	0.081425	0.050096	1.625375	0.104083	NA
MAOB	67.22951	-0.08142	0.081501	-0.99905	0.31777	NA
LOC72875	3.827159	0.081418	0.080141	1.015943	0.309657	NA
PITPNB	251.1651	0.081413	0.05159	1.578081	0.114547	NA
MIR548AP	1.843477	0.081406	0.065489	1.243046	0.213851	NA
GAB2	221.6871	-0.0814	0.059522	-1.36762	0.171431	NA
LHFP	356.4602	0.081398	0.045663	1.78259	0.074653	0.390538
UBE4A	789.4835	-0.08137	0.049541	-1.64244	0.100498	0.43859
C15orf56	9.153049	0.081366	0.084936	0.957975	0.338075	NA
NRN1L	4.588278	-0.08136	0.082913	-0.98131	0.326438	NA
SLC25A26	31.42807	-0.08135	0.072278	-1.12546	0.260393	NA
LFNG	36.64555	-0.08133	0.080815	-1.00632	0.314262	NA
NET1	172.2161	-0.08131	0.077125	-1.05427	0.29176	NA
RCAN3	354.1569	0.0813	0.062987	1.290744	0.196792	0.545356
RDH12	2.089603	-0.08129	0.063022	-1.28984	0.197106	NA
PCED1B-A	1.366024	-0.08127	0.06284	-1.29334	0.195892	NA
TMC1	4.583955	0.081262	0.079477	1.022469	0.306559	NA
HMGXB4	137.6849	-0.08125	0.049733	-1.63377	0.102307	NA
SERTAD2	117.6452	0.081252	0.056607	1.43537	0.151182	NA
SNHG16	38.42481	-0.08125	0.068898	-1.17923	0.238307	NA
ANKRD9	33.12258	0.081241	0.072389	1.12229	0.261739	NA
PAAF1	61.63924	-0.08124	0.08275	-0.98171	0.326242	NA
FUT11	44.2224	0.081234	0.06093	1.333231	0.182456	NA
IGFALS	5.170362	-0.08123	0.083274	-0.97544	0.329341	NA
SLC26A2	195.3203	0.081222	0.070234	1.15645	0.247497	NA
SNORA27	2.326898	0.081188	0.069902	1.161449	0.245459	NA
RAMP3	3.376194	0.081172	0.071971	1.127845	0.259385	NA
LY6G5B	26.53183	-0.08117	0.081869	-0.99147	0.321454	NA
LOC15056	2.122954	-0.08116	0.06687	-1.21373	0.224849	NA
SNURF	5.168706	0.081117	0.083589	0.970421	0.331837	NA
TRAF4	37.38378	0.081107	0.071223	1.138783	0.254794	NA
RTN3	1223.344	0.081098	0.04767	1.701245	0.088897	0.415114
LRP12	127.7067	0.081091	0.055118	1.471243	0.141225	NA
BAI1	94.29172	-0.08109	0.079489	-1.0201	0.307681	NA
C6orf25	1.756869	0.081084	0.060516	1.339873	0.180287	NA
BOLL	1.515853	0.081072	0.053652	1.511077	0.130769	NA
ELOVL4	108.5437	0.081066	0.063267	1.281332	0.200077	NA
KRTAP5-9	0.81955	-0.08106	0.048298	-1.67827	0.093295	NA
TRERF1	152.6769	0.081048	0.058629	1.382384	0.166854	NA
DDAH2	19.29908	-0.08102	0.080331	-1.00856	0.313187	NA
PRR7	12.82601	-0.08101	0.083589	-0.96909	0.332501	NA
RPAP3	183.5469	0.080997	0.049201	1.64624	0.099714	NA
C1orf111	10.1798	-0.081	0.083865	-0.9658	0.334147	NA
C1orf112	56.6009	0.080991	0.069696	1.162051	0.245215	NA
LYPD6	25.24653	-0.08098	0.081836	-0.98953	0.322402	NA
GBX2	3.954621	0.080977	0.076636	1.056641	0.290675	NA

MYO1D	152.5452	0.080968	0.05721	1.415281	0.156986	NA
COX19	151.746	-0.08097	0.069332	-1.16783	0.242877	NA
HEATR3	79.27818	0.080949	0.069484	1.164992	0.244022	NA
NCAM1	1421.235	0.080936	0.038165	2.120674	0.033949	0.288416
SSTR1	5.02558	-0.08091	0.08037	-1.00671	0.314077	NA
MOB4	5.459196	0.080895	0.082421	0.981491	0.326351	NA
DAGLB	100.2707	-0.08088	0.060067	-1.34643	0.178165	NA
SEMA3A	8.251191	0.080868	0.069997	1.155309	0.247964	NA
PHEX-AS1	5.088865	-0.08087	0.076635	-1.05523	0.291319	NA
PHEX	15.21449	-0.08086	0.081943	-0.98673	0.323775	NA
ARID5A	22.29426	-0.08085	0.083904	-0.96356	0.335268	NA
43353	29.08998	-0.08085	0.078832	-1.02555	0.305105	NA
PPP3CB	667.5689	0.080841	0.060855	1.328437	0.184034	0.534406
MEST	61.09284	0.080818	0.074754	1.081119	0.279644	NA
POLR2D	80.21863	0.080805	0.054793	1.47472	0.140288	NA
LOC10050	91.97101	-0.08079	0.059602	-1.35557	0.175235	NA
DEXI	42.2983	-0.08079	0.068868	-1.17313	0.240742	NA
HK1	699.8919	0.080782	0.053442	1.511575	0.130642	0.487416
KLF10	16.55087	0.080778	0.084436	0.956686	0.338726	NA
ZNF788	70.22147	-0.08078	0.083146	-0.97152	0.33129	NA
SNORA68	12.36225	-0.08077	0.082296	-0.98144	0.326374	NA
CEP170P1	2.047962	-0.08076	0.06556	-1.23184	0.21801	NA
NR2F6	48.72926	0.080756	0.062905	1.28378	0.199219	NA
NUP50	203.3185	0.080743	0.048743	1.656508	0.097619	NA
GRIPAP1	919.2269	-0.08074	0.043316	-1.86389	0.062337	0.37053
MIR125A	0.366026	-0.08071	0.035411	-2.27923	0.022653	NA
XDH	2.852535	-0.0807	0.073146	-1.10328	0.269904	NA
SLC6A1	284.6409	-0.08069	0.083501	-0.96629	0.333899	NA
NPM1	417.6331	-0.08068	0.071168	-1.1337	0.256919	0.598301
DFNB59	6.779172	-0.08068	0.08288	-0.97344	0.330334	NA
MT1F	8.593852	-0.08067	0.080127	-1.00674	0.314058	NA
NOP10	17.44161	-0.08067	0.083206	-0.96949	0.332301	NA
PAFAH1B2	501.164	0.080655	0.056564	1.425902	0.153897	0.506644
PTPRZ1	713.4023	-0.08063	0.072593	-1.11075	0.266677	0.607287
ECHS1	101.1639	-0.08062	0.05541	-1.455	0.145669	NA
STK3	23.46125	-0.08061	0.082306	-0.97941	0.327377	NA
GNB2L1	409.217	-0.08061	0.053603	-1.50375	0.132647	0.487416
TMPRSS3	1.772263	-0.0806	0.054541	-1.47785	0.139447	NA
RRP1B	163.5659	0.080599	0.043945	1.83411	0.066638	NA
NCDN	621.7461	0.080597	0.052482	1.535703	0.124611	0.47732
ZNF791	192.2713	0.080584	0.046241	1.742701	0.081386	NA
TXNRD3	33.83573	0.080576	0.073079	1.102586	0.270207	NA
BHLHE22	130.8942	-0.08056	0.083323	-0.96688	0.333603	NA
LRRC37A6	15.42887	0.080558	0.084872	0.949174	0.342532	NA
SAMD3	2.290219	0.080553	0.0649	1.241181	0.214539	NA
XIAP	271.3873	0.080526	0.046472	1.732778	0.083135	NA
CIB1	11.78623	-0.08052	0.084073	-0.95771	0.338211	NA

CHMP2A	164.7085	-0.08049	0.06803	-1.1831	0.236768	NA
LINC00341	8.250364	0.080469	0.084476	0.952571	0.340808	NA
AGPS	221.8446	0.080446	0.042912	1.874665	0.060839	NA
MIEN1	27.36877	-0.08044	0.078667	-1.0225	0.306544	NA
KIAA1598	356.5048	0.080415	0.065422	1.229171	0.219008	0.569521
TTC13	134.379	0.0804	0.062904	1.278145	0.201198	NA
MED12L	304.3604	0.080378	0.042246	1.902602	0.057093	NA
GTF2E1	34.58993	0.080373	0.071136	1.129856	0.258537	NA
PLCE1	33.75067	-0.08037	0.082465	-0.9746	0.329757	NA
SNAPC5	44.99128	0.080368	0.076106	1.056009	0.290964	NA
ASPA	11.97751	-0.08034	0.084854	-0.94685	0.343715	NA
GRM4	1445.729	0.080341	0.059434	1.351753	0.176454	0.529395
KLHL15	94.8109	0.080325	0.060548	1.326633	0.18463	NA
PRSS23	14.48735	0.080318	0.080531	0.997358	0.318591	NA
PRDM5	18.48548	-0.08031	0.080037	-1.00336	0.315686	NA
GAS1	13.63856	0.080288	0.082051	0.978525	0.327815	NA
CAPNS1	165.882	-0.08028	0.071718	-1.11942	0.262959	NA
ABCA1	104.4187	-0.08028	0.078955	-1.01681	0.309246	NA
UBA1	416.5234	0.080267	0.046671	1.71987	0.085456	0.40835
GMFB	331.3111	0.080255	0.069665	1.152024	0.249311	NA
LRRIQ1	6.177253	0.08024	0.079162	1.013616	0.310766	NA
IFT80	158.7071	0.080239	0.057694	1.390767	0.164296	NA
OPTC	1.830993	-0.08024	0.061531	-1.30399	0.192236	NA
MIR1304	7.642632	-0.08022	0.084068	-0.95419	0.339988	NA
FAM19A1	1.079068	-0.08021	0.051261	-1.5648	0.11763	NA
ROGDI	269.205	0.080208	0.048038	1.669661	0.094986	NA
SEL1L3	793.848	0.080208	0.035659	2.249275	0.024495	0.246583
APOBEC4	47.34382	-0.08019	0.073162	-1.0961	0.273034	NA
MAP3K8	9.223013	-0.08019	0.082813	-0.96828	0.332902	NA
TM4SF20	8.918361	-0.08018	0.084932	-0.94405	0.345146	NA
OXNAD1	93.14931	-0.08017	0.054396	-1.47384	0.140524	NA
SLMO1	39.47043	-0.08016	0.075308	-1.06446	0.287121	NA
FAM41C	2.470147	0.080156	0.055579	1.442205	0.149245	NA
TUBB4A	2298.871	0.080151	0.064525	1.242177	0.214171	0.562782
PPFIA3	562.9037	0.080146	0.045495	1.761638	0.078131	0.397742
ANAPC7	138.1998	0.080119	0.0514	1.558729	0.119061	NA
LGI3	58.18237	0.080106	0.072853	1.099561	0.271523	NA
CUBN	46.37721	0.0801	0.082788	0.967536	0.333276	NA
RAP1GAP2	529.2658	0.080068	0.052187	1.534253	0.124967	0.47732
FAM182B	10.66963	-0.08007	0.078223	-1.02358	0.306034	NA
C10orf90	17.91691	-0.08007	0.084783	-0.94437	0.344979	NA
HARS	195.1128	-0.08006	0.036913	-2.16883	0.030095	NA
MARK1	174.8471	0.080053	0.044317	1.806352	0.070863	NA
ABCC12	2.198199	0.080044	0.070071	1.142316	0.253323	NA
COMMD9	51.25309	-0.08004	0.060473	-1.32358	0.185642	NA
YWHAZ	559.9947	0.080037	0.069087	1.158485	0.246666	0.592704
AKR1E2	15.29606	-0.08003	0.08452	-0.94684	0.343719	NA

TPTE2P3	1.176469	-0.08002	0.051719	-1.54716	0.121825	NA
CYP27B1	4.650557	0.080001	0.076963	1.039481	0.298581	NA
TSC2	649.3044	-0.08	0.04423	-1.80863	0.070509	0.387629
PRKDC	1113.718	0.079981	0.038037	2.102717	0.03549	0.289763
POLN	68.79135	0.079974	0.076825	1.04099	0.29788	NA
PAPD5	276.2703	0.079953	0.050845	1.572476	0.11584	NA
COMMD1	88.28161	-0.07992	0.054186	-1.47492	0.140233	NA
PAPOLB	43.68759	0.079904	0.07337	1.08905	0.276132	NA
MRPL48	71.5723	-0.0799	0.063971	-1.24904	0.211652	NA
AR	12.72564	-0.0799	0.084823	-0.94198	0.346204	NA
FKBP1A	47.94907	-0.07988	0.065951	-1.21126	0.225796	NA
HNRNPA1	19.90577	0.079884	0.078168	1.021956	0.306802	NA
BBS4	52.60875	-0.07987	0.074846	-1.06708	0.285934	NA
CHAD	76.38755	0.079866	0.084162	0.948955	0.342644	NA
CTCF	310.5977	0.079865	0.05078	1.572787	0.115768	NA
H2AFY2	77.39783	0.079852	0.058867	1.356485	0.174945	NA
INHBC	1.840291	0.07984	0.053721	1.486183	0.137231	NA
ZNF805	164.7315	0.079823	0.046618	1.71227	0.086847	NA
CCDC144C	283.5511	0.079821	0.077264	1.033088	0.301563	NA
LARP7	176.5352	0.079819	0.049769	1.603786	0.108761	NA
CCDC158	8.211079	-0.07982	0.081211	-0.98283	0.325689	NA
ZNF41	127.7491	0.079812	0.04615	1.729411	0.083736	NA
MTR	484.367	-0.07981	0.062265	-1.28177	0.199922	0.547633
LCP2	11.11248	-0.0798	0.084745	-0.94167	0.346361	NA
NOXA1	20.04428	-0.0798	0.084716	-0.94198	0.3462	NA
HDHD1	28.28452	-0.07978	0.077241	-1.03291	0.301648	NA
IL10RB	20.92819	-0.07977	0.08229	-0.96937	0.33236	NA
MAN1A2	1163.355	0.079744	0.053096	1.50187	0.133131	0.487416
SEC14L5	51.27047	-0.07974	0.076372	-1.04406	0.296457	NA
RIMS1	4235.286	0.079737	0.057816	1.379148	0.167849	0.521454
ADRB2	4.616313	-0.07973	0.081667	-0.97625	0.328943	NA
TGM6	0.307892	-0.07971	0.031296	-2.54696	0.010867	NA
DDTL	5.372636	0.079698	0.081404	0.979036	0.327562	NA
ZCWPW2	13.50166	-0.07969	0.081921	-0.97275	0.33068	NA
SLC47A2	15.64602	0.07967	0.084939	0.937963	0.348264	NA
SNORD115	103.6751	-0.07967	0.082152	-0.96973	0.33218	NA
C2CD5	585.2146	-0.07965	0.032429	-2.45616	0.014043	0.212051
C2CD2L	132.9053	0.079649	0.055377	1.438288	0.150352	NA
PIPSL	8.481036	0.079645	0.084916	0.937922	0.348284	NA
EPB41L4A	143.9756	-0.07964	0.054093	-1.47234	0.140929	NA
C11orf93	2.390496	-0.07964	0.068806	-1.15742	0.247102	NA
C18orf42	126.8293	0.079635	0.069316	1.148867	0.250611	NA
DNAH7	21.24746	-0.07963	0.084785	-0.93924	0.347607	NA
SUZ12	217.9855	0.079612	0.048944	1.626608	0.10382	NA
GUK1	238.9735	-0.07961	0.056719	-1.40357	0.160448	NA
EMC4	64.30475	-0.0796	0.070362	-1.13133	0.257916	NA
MYO1A	1.877076	-0.07959	0.067948	-1.17126	0.241494	NA

F11R	15.06992	-0.07958	0.084848	-0.93791	0.348291	NA
CAMK4	703.1866	0.079565	0.057717	1.378533	0.168039	0.521454
GPR114	1.239985	0.079542	0.05691	1.397678	0.16221	NA
MPV17L	42.0435	0.079541	0.069199	1.149451	0.25037	NA
CHRNG	6.639459	0.079495	0.082964	0.958188	0.337968	NA
IDE	165.3188	0.079494	0.046838	1.697198	0.089659	NA
FAM171A1	264.5787	0.079492	0.058501	1.35883	0.174201	NA
C9orf64	19.29888	-0.07949	0.079672	-0.99773	0.318409	NA
RNASET2	22.1846	-0.07948	0.083485	-0.95203	0.341083	NA
LRFN4	79.85819	0.079475	0.072867	1.09069	0.275409	NA
MASP2	119.9738	-0.07947	0.048256	-1.6468	0.099598	NA
SALL3	3.823554	-0.07946	0.079299	-1.00204	0.316326	NA
FAM159B	2.353956	-0.07944	0.070274	-1.13049	0.258268	NA
FADS6	36.5587	0.079434	0.075483	1.052348	0.29264	NA
RBFOX1	631.0696	-0.07942	0.050707	-1.56628	0.117282	0.472599
DRG2	57.27639	-0.07941	0.063422	-1.25215	0.210514	NA
BEST2	2.735241	0.07941	0.073427	1.08148	0.279484	NA
PLA2G4A	6.922061	-0.07935	0.08415	-0.94299	0.345685	NA
MIR548K	1.302175	-0.07935	0.062646	-1.26665	0.205279	NA
SIRPA	458.668	0.079345	0.045797	1.732562	0.083174	0.403119
UCKL1	212.3326	-0.07934	0.068873	-1.15196	0.249337	NA
PPWD1	211.77	-0.07933	0.043474	-1.82472	0.068043	NA
KIAA1239	22.92696	0.079323	0.084936	0.933913	0.350349	NA
KLHL10	4.172691	0.079315	0.0805	0.98528	0.324487	NA
CCDC155	45.48952	-0.07926	0.083284	-0.95175	0.341226	NA
NT5C3B	134.4572	-0.07926	0.060984	-1.29965	0.193721	NA
KXD1	48.84619	-0.07926	0.068976	-1.14902	0.250547	NA
C20orf111	90.92141	-0.07925	0.066444	-1.1928	0.232947	NA
SLCO2A1	1.604613	-0.07925	0.061876	-1.28074	0.200285	NA
PCTP	21.00083	-0.07924	0.078342	-1.0115	0.311776	NA
CPVL	61.88332	-0.07924	0.071689	-1.10533	0.269015	NA
PHTF2	137.128	-0.07924	0.061335	-1.29191	0.196387	NA
FAM22D	6.366794	0.079237	0.081327	0.974296	0.32991	NA
ALLC	0.435	-0.07923	0.03727	-2.12593	0.033509	NA
EPB41L5	652.7322	-0.07923	0.058536	-1.35352	0.175889	0.529097
UQCR10	71.2226	-0.07922	0.079018	-1.00252	0.31609	NA
ERAP1	86.17686	-0.07919	0.065288	-1.21289	0.225171	NA
KRI1	181.9374	-0.07919	0.070097	-1.12967	0.258617	NA
PRR19	1.706241	0.079179	0.064128	1.234714	0.216937	NA
MYCBP2	2509.309	0.079174	0.033295	2.377954	0.017409	0.227303
REPS2	354.9434	0.079169	0.053903	1.468736	0.141904	0.495647
TRPC6	8.135189	-0.07916	0.082183	-0.96323	0.33543	NA
PAPOLA	783.5716	0.079149	0.069051	1.146244	0.251694	0.592704
AIFM2	45	-0.07915	0.076081	-1.04032	0.29819	NA
SMIM12	112.4304	0.079078	0.060103	1.315714	0.18827	NA
ERVMER34	2.876248	0.079076	0.071608	1.104296	0.269465	NA
PFKFB2	32.99539	-0.07902	0.081948	-0.96427	0.334913	NA

TXNDC17	14.30122	0.079007	0.08252	0.957424	0.338353	NA
NUDT15	34.9025	0.079001	0.07106	1.111753	0.266244	NA
ADAMTS5	142.6658	-0.07898	0.07004	-1.12763	0.259478	NA
RCAN1	127.296	0.078975	0.054299	1.454449	0.145822	NA
ARHGAP1	59.88489	-0.07897	0.056542	-1.39663	0.162524	NA
HUS1B	4.679128	-0.07894	0.079504	-0.99285	0.320782	NA
ANKMY1	33.29122	-0.07893	0.080552	-0.97988	0.327143	NA
HIST1H1D	33.46659	-0.07893	0.084706	-0.93178	0.351449	NA
TRMT12	57.67973	0.078926	0.059359	1.329647	0.183635	NA
ECSIT	83.99073	-0.07892	0.051371	-1.53633	0.124458	NA
HEXB	62.04453	-0.07892	0.059398	-1.32865	0.183963	NA
CDO1	44.31475	0.078893	0.066795	1.181122	0.237554	NA
GALR3	2.245447	0.078875	0.06894	1.144103	0.252581	NA
TRPV1	131.9051	-0.07887	0.061181	-1.28916	0.197341	NA
NAALADL2	18.52657	-0.07887	0.083115	-0.94896	0.342642	NA
PHKA1	66.93753	0.078821	0.061802	1.275396	0.202169	NA
EGR4	8.293344	-0.07882	0.072755	-1.08333	0.278663	NA
SYVN1	131.4885	-0.07881	0.056056	-1.406	0.159724	NA
SFTPD	13.02825	0.078815	0.084475	0.932987	0.350827	NA
LRRC8D	105.0744	0.0788	0.058221	1.353461	0.175908	NA
RELL2	30.90489	-0.0788	0.080857	-0.97454	0.329788	NA
CGNL1	107.2978	-0.0788	0.073346	-1.07434	0.282673	NA
NKIRAS1	144.4071	0.078792	0.046514	1.693933	0.090278	NA
KDM8	32.61265	0.078765	0.076513	1.029429	0.303278	NA
ARID3A	61.33431	-0.07873	0.076939	-1.02331	0.306159	NA
LRRC61	14.04026	0.078722	0.084681	0.929625	0.352565	NA
SLC5A1	0.283883	-0.07871	0.030191	-2.60722	0.009128	NA
QTRT1	75.03705	-0.07869	0.064785	-1.21455	0.224536	NA
PRKAG2-A	3.470186	-0.07867	0.075106	-1.04752	0.29486	NA
MAD2L2	52.33047	0.07866	0.064297	1.223388	0.221183	NA
DYRK1B	88.43846	0.07866	0.072976	1.077877	0.281089	NA
MIR135B	1.303142	0.078642	0.059357	1.324884	0.18521	NA
PTPRM	264.2632	-0.07864	0.079533	-0.98876	0.322781	NA
ADPRH	6.125147	0.078627	0.083864	0.937563	0.348469	NA
RGS2	33.03039	0.078626	0.077424	1.015531	0.309853	NA
GAR1	9.603776	0.078619	0.084724	0.927938	0.35344	NA
ENTPD5	27.00106	-0.0786	0.079282	-0.99137	0.321504	NA
IL1R1	23.45632	-0.07859	0.083231	-0.94424	0.345046	NA
LOC10027	6.425564	-0.07856	0.082933	-0.94727	0.343502	NA
LOC28608	2.362028	-0.07856	0.070598	-1.11275	0.265817	NA
FES	13.63605	-0.07856	0.083724	-0.93827	0.348105	NA
TSPAN5	401.5718	-0.07856	0.03958	-1.98475	0.047172	0.337641
UPF3A	385.1884	-0.07854	0.051361	-1.5291	0.12624	0.479566
ITPK1	300.4062	-0.07854	0.053009	-1.48156	0.138458	NA
C2orf73	5.687245	-0.07853	0.083689	-0.93835	0.348063	NA
FAM98B	141.047	0.078528	0.053145	1.477617	0.13951	NA
DNAAF2	61.47342	0.078513	0.059053	1.329536	0.183671	NA

RAG2	2.009102	-0.07849	0.070287	-1.11671	0.264119	NA
NARS2	53.21426	-0.07847	0.066343	-1.18278	0.236896	NA
PPP1R35	20.9675	-0.07846	0.076228	-1.02928	0.303349	NA
RRM2B	113.4199	-0.07845	0.063607	-1.23338	0.217435	NA
ELP3	155.1013	0.078436	0.051378	1.526633	0.126852	NA
PNMA1	314.3357	0.078435	0.067698	1.158601	0.246619	NA
GPT	47.61544	-0.07842	0.07798	-1.00561	0.314604	NA
KIAA1199	167.8796	-0.07841	0.070807	-1.10733	0.26815	NA
KRT12	1.14222	0.078404	0.057958	1.352783	0.176125	NA
RHOT1	244.5916	0.078401	0.040943	1.914862	0.05551	NA
DPP4	8.359465	-0.07839	0.084892	-0.92337	0.355814	NA
TAF10	27.62407	-0.07837	0.071433	-1.09713	0.272586	NA
IRAK2	23.68504	0.078357	0.079446	0.986296	0.323988	NA
GATA1	0.234524	-0.07836	0.028871	-2.71403	0.006647	NA
LOC10028	1.201553	-0.07834	0.056671	-1.38242	0.166841	NA
CCDC69	45.3073	-0.07834	0.078105	-1.00299	0.315863	NA
IRX5	12.63463	-0.07834	0.084931	-0.92238	0.356332	NA
MORC3	528.8065	0.07831	0.045398	1.724956	0.084535	0.405663
GSG1L	7.657302	-0.0783	0.084472	-0.92691	0.353971	NA
SPATA13-A	1.392727	0.078294	0.05982	1.308816	0.190597	NA
SLC6A4	10.42911	-0.07829	0.084815	-0.92309	0.355958	NA
SHROOM2	48.05348	-0.07829	0.084936	-0.92175	0.35666	NA
RWDD1	113.9711	0.078271	0.060827	1.286768	0.198175	NA
SNORD17	69.97235	-0.07827	0.080915	-0.96727	0.33341	NA
TPMT	78.49797	0.07826	0.063787	1.226888	0.219865	NA
RPL8	363.0129	-0.07826	0.070266	-1.11374	0.26539	0.607287
ZNF550	35.72759	0.078241	0.072366	1.081187	0.279614	NA
GORAB	32.31798	0.078234	0.073038	1.071144	0.284105	NA
NECAP1	254.524	0.078216	0.068602	1.140139	0.254229	NA
PDHB	134.3457	-0.07821	0.056019	-1.39609	0.162688	NA
ARHGAP5	1622.553	0.0782	0.048579	1.609749	0.107453	0.452973
TPTEP1	2.17421	0.078196	0.067421	1.159822	0.246121	NA
ZNF668	37.80996	0.078188	0.072457	1.079098	0.280544	NA
NCAPD2	118.9429	-0.07818	0.059764	-1.30809	0.190844	NA
EMC7	47.12327	-0.07817	0.074353	-1.05139	0.293079	NA
GSE1	652.9207	0.078166	0.055786	1.401184	0.161159	0.519239
DFNA5	22.71299	-0.07816	0.081952	-0.95375	0.340212	NA
PLEKHG2	32.06483	-0.07815	0.081077	-0.96391	0.335089	NA
CMC1	15.27491	-0.07815	0.083956	-0.93085	0.351932	NA
GOLGA2	362.5983	0.078143	0.044931	1.73918	0.082003	0.401988
ST6GALNA	44.28538	0.078142	0.065912	1.185557	0.235797	NA
RBM18	92.12039	0.078138	0.059373	1.316049	0.188157	NA
NPR1	5.882506	-0.07812	0.078551	-0.99448	0.31999	NA
DTX3L	26.85056	-0.07809	0.084644	-0.92263	0.356201	NA
OR5AK4P	0.620406	-0.07809	0.039004	-2.00207	0.045277	NA
MTRNR2L	1.35991	-0.07806	0.054728	-1.4264	0.153752	NA
CNGB1	0.569402	0.078061	0.04172	1.871082	0.061334	NA

MIR3938	0.848307	0.078057	0.049423	1.579356	0.114254	NA
SRP14	448.9686	-0.07802	0.043261	-1.8034	0.071325	0.38806
THOC6	10.50494	-0.07801	0.084913	-0.91868	0.358262	NA
RNF111	310.5066	0.078006	0.035876	2.174314	0.029682	NA
RNF187	311.3353	0.078003	0.061828	1.261614	0.207088	NA
SIN3B	305.4749	-0.07799	0.048547	-1.60648	0.108169	NA
DPY19L3	194.9267	-0.07796	0.071574	-1.08919	0.276071	NA
TBPL1	71.79559	-0.07796	0.055111	-1.41453	0.157207	NA
TMSB4X	215.9335	-0.07796	0.082343	-0.94672	0.343779	NA
EPN2-IT1	12.17261	-0.07795	0.083345	-0.93525	0.349661	NA
NBPF3	56.15738	-0.07794	0.073199	-1.06484	0.286948	NA
LOC10028	61.55481	-0.07793	0.067077	-1.16183	0.245306	NA
CD47	655.0265	0.077926	0.032638	2.387559	0.016961	0.224654
ZFYVE27	103.6091	-0.07791	0.047143	-1.65259	0.098413	NA
MIRLET7E	2.054953	0.077893	0.069751	1.116728	0.264111	NA
CCDC144N	18.53567	0.077876	0.083885	0.928368	0.353217	NA
TNFAIP2	14.7735	0.077847	0.084808	0.917925	0.358658	NA
TRIM62	129.213	0.077828	0.049187	1.582286	0.113584	NA
FARP1	95.58235	-0.07783	0.076686	-1.01487	0.310166	NA
FABP6	11.36141	-0.07782	0.083803	-0.92862	0.353085	NA
KGFLP2	16.48765	-0.07781	0.084923	-0.91622	0.359553	NA
C8orf31	11.13648	0.077801	0.084855	0.916877	0.359207	NA
TMEM51-	136.0081	0.077793	0.071652	1.085702	0.277611	NA
SLC10A5	24.10336	0.077785	0.083672	0.929633	0.352561	NA
KIF20A	5.087624	0.077784	0.078395	0.992211	0.321095	NA
PQBP1	105.537	-0.07778	0.064569	-1.20461	0.228353	NA
TEAD2	5.60131	-0.07776	0.080616	-0.96459	0.334751	NA
ZNF367	11.66695	-0.07776	0.083829	-0.92757	0.353629	NA
POU1F1	0.750059	0.07775	0.048331	1.608676	0.107687	NA
FAM84B	67.81796	-0.07773	0.081475	-0.954	0.340081	NA
BTRC	324.5582	0.07772	0.032793	2.369998	0.017788	NA
WISP3	4.600542	-0.07772	0.081739	-0.95081	0.341699	NA
ABHD14A	25.45321	-0.0777	0.079214	-0.98089	0.326649	NA
DIAPH1	309.1282	-0.0777	0.046748	-1.66206	0.096501	NA
MIR329-2	1.275874	-0.07769	0.059454	-1.30679	0.191283	NA
CNTN2	542.5616	0.077687	0.060686	1.280161	0.200488	0.547633
ASB3	15.18622	-0.07769	0.084513	-0.91922	0.357978	NA
WWC3	279.8844	0.077686	0.051668	1.503555	0.132696	NA
BREA2	1.786434	-0.07767	0.063763	-1.21814	0.223171	NA
MIR3124	0.525786	0.077648	0.039865	1.947761	0.051444	NA
ZNF84	429.2356	0.077623	0.041741	1.859629	0.062938	0.37053
IHH	0.711609	0.077612	0.046295	1.676447	0.093651	NA
ZNF530	23.22872	0.077609	0.078216	0.992235	0.321083	NA
CDCA8	3.982309	-0.0776	0.079048	-0.98174	0.326227	NA
GPR17	13.56746	-0.07759	0.084578	-0.91742	0.358924	NA
ZSCAN12P	8.050505	0.077592	0.084581	0.91737	0.358949	NA
FADS2	91.22758	-0.07758	0.079209	-0.9794	0.327381	NA

NTPCR	36.62483	-0.07757	0.072084	-1.07612	0.281873	NA
FBXL17	172.0613	0.07757	0.063924	1.213468	0.224951	NA
DHH	1.477797	0.077561	0.055771	1.390701	0.164316	NA
NDUFS3	65.32291	-0.07756	0.069162	-1.12137	0.262132	NA
ABCA7	101.5468	0.077544	0.083637	0.92715	0.353848	NA
CLNS1A	308.706	-0.07754	0.045666	-1.69801	0.089507	NA
UBR7	117.8512	0.077539	0.051264	1.512543	0.130396	NA
LRGUK	6.855568	0.077528	0.083403	0.929563	0.352597	NA
NFAT5	1655.725	0.07752	0.036603	2.117842	0.034188	0.288416
SNORD47	14.27962	0.077508	0.084853	0.913433	0.361015	NA
CCT4	197.4166	0.077502	0.06774	1.144115	0.252576	NA
STXBP4	54.73081	-0.0775	0.062004	-1.2499	0.211337	NA
NDUFB11	45.41281	-0.07749	0.07309	-1.06021	0.289048	NA
ODF3B	12.83315	-0.07748	0.083911	-0.92331	0.355848	NA
F2RL3	1.020936	-0.07747	0.052768	-1.46808	0.142082	NA
COBL	376.3539	-0.07746	0.061936	-1.25059	0.211084	0.560498
LOC39981	1.016922	0.077446	0.054026	1.433495	0.151716	NA
HMGN4	93.60202	-0.07742	0.049771	-1.55558	0.119809	NA
EXOC8	159.6958	0.077419	0.065552	1.181038	0.237588	NA
C6orf106	446.4843	0.077417	0.051624	1.499649	0.133705	0.487416
GRIK1	52.67224	-0.07741	0.083982	-0.92174	0.356667	NA
LOC10021	7.386353	-0.07739	0.081978	-0.94404	0.345149	NA
FAM69A	85.65723	0.077341	0.056588	1.366743	0.171706	NA
CENPP	53.13008	-0.07732	0.07787	-0.99293	0.320745	NA
PROS1	10.49542	-0.07731	0.084914	-0.91051	0.362555	NA
SIX3	2.71958	-0.0773	0.071571	-1.08006	0.280113	NA
DDC	0.759137	-0.07729	0.050577	-1.52825	0.126451	NA
GIGYF1	683.0908	-0.07728	0.052932	-1.45995	0.144304	0.495647
NAT14	27.38621	0.077266	0.072237	1.069616	0.284792	NA
RPL5	831.1986	-0.07726	0.078614	-0.98279	0.325712	0.655185
CHIC1	181.7372	0.077254	0.057808	1.336395	0.18142	NA
CDC23	94.22282	0.077252	0.048851	1.58136	0.113796	NA
TUBB4B	162.1309	0.077223	0.066017	1.169747	0.242103	NA
OR1C1	0.462075	-0.07722	0.035668	-2.16493	0.030393	NA
EMP1	16.87115	-0.0772	0.07758	-0.9951	0.319688	NA
RDH16	4.346629	-0.07719	0.073935	-1.04408	0.296447	NA
E2F6	26.45254	-0.07719	0.074258	-1.03952	0.298563	NA
BBS12	13.28959	0.077184	0.081976	0.941548	0.346424	NA
TMEM132	140.2747	0.077177	0.056329	1.370107	0.170653	NA
MORC2	225.4204	-0.07717	0.042845	-1.80117	0.071676	NA
ZNF45	113.6006	0.077141	0.05149	1.498177	0.134087	NA
ASXL3	12.19475	-0.07714	0.084281	-0.91521	0.360081	NA
HERPUD1	144.2808	-0.07713	0.064117	-1.20292	0.229006	NA
DCUN1D1	138.1516	0.077126	0.04647	1.6597	0.096975	NA
ALOX5	132.2244	-0.07712	0.080443	-0.95867	0.337725	NA
KLHL34	39.76924	0.077114	0.079314	0.972256	0.330923	NA
SNORD54	5.415584	-0.0771	0.081888	-0.94158	0.346409	NA

DOCK9	877.0802	0.0771	0.044337	1.738948	0.082044	0.401988
TMEM151	853.0631	-0.0771	0.044325	-1.73932	0.081979	0.401988
COL7A1	335.8264	-0.07704	0.07766	-0.99202	0.321188	0.651336
CRTC3	171.247	0.077034	0.045282	1.701221	0.088901	NA
OXCT1	287.8586	0.077034	0.06367	1.209893	0.22632	NA
USP40	130.0095	-0.07703	0.069727	-1.10473	0.269278	NA
TPT1	793.3347	-0.07697	0.077495	-0.99317	0.320628	0.651322
RNF38	239.3818	0.076965	0.03801	2.024839	0.042884	NA
ZNF202	91.79673	0.076932	0.073232	1.050521	0.293479	NA
STIL	17.83871	-0.07693	0.079355	-0.96945	0.332323	NA
GARNL3	594.0812	-0.07693	0.042727	-1.80043	0.071792	0.388089
STEAP2	235.112	0.076914	0.065195	1.179759	0.238096	NA
B3GNT4	24.75897	-0.07691	0.076626	-1.00367	0.315538	NA
PRPSAP1	160.6307	0.076905	0.068565	1.121627	0.262021	NA
POLR2J4	114.8725	-0.0769	0.051051	-1.50642	0.13196	NA
ZFR2	21.47697	-0.07687	0.08468	-0.90779	0.363989	NA
EFCAB14	372.9861	0.076867	0.042738	1.798567	0.072087	0.388756
RAB5C	147.8901	0.076846	0.067521	1.138103	0.255078	NA
LIMA1	1024.899	-0.07685	0.070537	-1.08943	0.275962	0.61452
RCHY1	154.385	0.076842	0.043909	1.750038	0.080112	NA
SND1	354.9471	-0.0768	0.039878	-1.92586	0.054122	0.357364
CDCA2	0.736949	-0.0768	0.043794	-1.75361	0.079497	NA
LRRC37A	6.03692	0.076787	0.076283	1.006607	0.314124	NA
DIEXF	76.6772	0.076781	0.064352	1.193148	0.232811	NA
RP9P	14.68547	0.076766	0.081269	0.944587	0.34487	NA
KCNAB1-A	1.046971	-0.07676	0.054402	-1.41092	0.158267	NA
PCDHGA11	131.2945	0.076745	0.081385	0.942992	0.345685	NA
GREB1	93.3636	-0.07674	0.075163	-1.02093	0.307288	NA
BCKDHB	84.3857	-0.07673	0.062446	-1.2288	0.219145	NA
FHOD1	117.1194	-0.0767	0.066465	-1.15403	0.248488	NA
PPL	116.4559	0.076701	0.067315	1.139435	0.254522	NA
TOX3	5.99813	-0.07669	0.082837	-0.92584	0.35453	NA
PFKFB3	353.0795	-0.07669	0.067775	-1.13151	0.257838	0.59833
PPAP2A	53.15086	0.07668	0.069838	1.097972	0.272217	NA
MRPL17	49.82669	0.076662	0.067682	1.132677	0.25735	NA
GAS2L3	1.739552	-0.07666	0.064532	-1.18787	0.234884	NA
GAS5-AS1	6.324674	0.076643	0.083424	0.918714	0.358245	NA
MYADM	55.89988	0.076634	0.065823	1.164232	0.24433	NA
HIST1H2A	2.570149	-0.07662	0.070506	-1.08675	0.277146	NA
PPP1R7	123.6816	0.076621	0.063375	1.20901	0.226659	NA
CDC7	101.2673	-0.07662	0.065092	-1.17711	0.23915	NA
PTBP1	143.2381	-0.07662	0.057828	-1.32493	0.185193	NA
PTEN	643.5931	0.076611	0.044716	1.713286	0.08666	0.410078
DNAJC3	173.4407	0.07661	0.058391	1.31202	0.189513	NA
SPARCL1	2164.865	-0.07661	0.070642	-1.08447	0.278155	0.614792
CPSF4L	2.265603	-0.07661	0.069079	-1.10896	0.26745	NA
MIR7-1	1.207229	-0.07659	0.05522	-1.38695	0.165458	NA

FAM205A	2.943586	-0.07657	0.074111	-1.0332	0.301509	NA
RNF32	25.45953	-0.07656	0.082231	-0.93099	0.351859	NA
SEMA6B	141.2988	0.076552	0.054308	1.409585	0.158662	NA
CRKL	473.7369	0.07655	0.043354	1.765688	0.077448	0.396878
DEF6	12.13988	-0.07652	0.084483	-0.90575	0.365066	NA
CCDC57	279.9327	-0.07652	0.062733	-1.21971	0.222576	NA
HRK	1.30955	0.076503	0.055285	1.38381	0.166417	NA
SRI	270.6599	-0.0765	0.051979	-1.47177	0.141084	NA
C11orf35	38.84012	-0.07649	0.084925	-0.90068	0.367756	NA
CDC45	2.164523	-0.07647	0.060906	-1.25556	0.209275	NA
LRRTM3	262.7252	-0.07642	0.064171	-1.19095	0.233675	NA
SV2B	1339.296	0.076423	0.053244	1.435332	0.151192	0.503604
EML1	28.39293	-0.07642	0.08169	-0.93548	0.349543	NA
CAB39	302.4661	0.076416	0.053689	1.423321	0.154643	NA
VAPA	414.4709	0.076411	0.058568	1.304667	0.192006	0.542492
SEC23A	222.5336	0.076407	0.048526	1.574553	0.11536	NA
PDE4D	188.9668	-0.07639	0.063309	-1.2066	0.227587	NA
MIR382	0.454536	0.076377	0.038294	1.99446	0.046102	NA
CHDH	100.8178	-0.07637	0.069934	-1.09198	0.274844	NA
KY	42.83083	0.076351	0.078788	0.969064	0.332513	NA
UBA7	29.317	-0.07634	0.078526	-0.9722	0.330949	NA
MNDA	5.60815	-0.07634	0.074433	-1.02557	0.305094	NA
SNORD43	0.765823	0.076335	0.04644	1.643729	0.100232	NA
SFXN2	30.39788	-0.07632	0.081103	-0.94103	0.346689	NA
MMD2	22.81574	0.076314	0.084904	0.898827	0.368745	NA
TMC4	15.28314	-0.07631	0.084352	-0.90463	0.365662	NA
SUMO2	160.2014	0.076306	0.068027	1.121696	0.261992	NA
TARBP1	277.4419	-0.0763	0.081805	-0.93275	0.35095	NA
EXOC3	280.9324	-0.0763	0.051203	-1.49011	0.136196	NA
MED9	69.74822	0.076285	0.056781	1.343492	0.179113	NA
FHIT	6.481463	-0.07628	0.084658	-0.90101	0.367582	NA
SPNS1	30.1489	-0.07626	0.076794	-0.99298	0.320721	NA
ZNF280D	386.1838	0.07624	0.040425	1.88597	0.059299	0.365363
LOC72802	13.92571	0.07623	0.083334	0.914754	0.360321	NA
TMEM191	1.129943	-0.07622	0.052681	-1.44689	0.147928	NA
IGSF5	1.161147	0.076194	0.054227	1.405085	0.159996	NA
C9orf69	41.60328	0.076192	0.071232	1.069628	0.284787	NA
AOAH	13.72066	-0.07618	0.08458	-0.9007	0.367746	NA
NEFL	325.7741	0.076178	0.084241	0.90429	0.365842	NA
CSGALNAC	34.91157	-0.07617	0.0763	-0.99834	0.318112	NA
LCOR	720.8926	0.076166	0.042654	1.785663	0.074154	0.390538
CHRM5	6.717764	0.076161	0.078687	0.967897	0.333096	NA
FAM49B	268.3296	0.076156	0.04852	1.569581	0.116513	NA
KLK7	3.002953	-0.07615	0.055589	-1.36986	0.170732	NA
MRPL37	68.44839	0.076142	0.061808	1.231917	0.21798	NA
KIAA1609	55.65359	-0.07613	0.067239	-1.13227	0.257521	NA
HIPK1	912.8164	0.076118	0.049282	1.544543	0.122457	0.476542

MAPK12	125.4398	0.076115	0.071404	1.065982	0.286432	NA
MRPL24	37.18471	-0.07611	0.078653	-0.9677	0.333195	NA
SLC25A5-A	12.11936	0.076099	0.083519	0.91116	0.362211	NA
EOGT	33.43656	-0.07608	0.075141	-1.01251	0.311292	NA
CYYR1	14.08144	0.076077	0.083945	0.906265	0.364796	NA
MIR612	14.05128	-0.07607	0.083162	-0.91475	0.360323	NA
NID2	32.69091	-0.07606	0.078701	-0.96641	0.33384	NA
PTS	35.24887	-0.07606	0.073006	-1.04177	0.297518	NA
IKBKE	18.68311	-0.07604	0.084935	-0.8953	0.370625	NA
PAK6	104.4575	0.076032	0.062998	1.206881	0.227478	NA
CCDC109B	1.972722	-0.07602	0.068262	-1.11362	0.265443	NA
SMOX	71.72063	0.075987	0.066139	1.148894	0.2506	NA
ATG16L2	72.41314	-0.07598	0.070962	-1.07071	0.2843	NA
NRADDP	3.465733	0.075979	0.077894	0.975412	0.329356	NA
CLSTN3	880.3795	0.075963	0.044134	1.721191	0.085216	0.408065
KTN1	1314.719	0.075958	0.044545	1.705178	0.088161	0.41454
LAPTM4A	94.72357	-0.07596	0.064425	-1.17899	0.238404	NA
SHCBP1	11.22968	-0.07594	0.084788	-0.89568	0.370422	NA
SNORD33	26.93671	-0.07592	0.081484	-0.93176	0.351458	NA
SLC25A20	6.539499	-0.07592	0.084772	-0.89552	0.370507	NA
SLC5A4	30.54633	0.075906	0.082722	0.917595	0.358831	NA
MRPS2	34.16721	0.075896	0.066649	1.138745	0.254809	NA
FAM210A	48.72319	-0.07589	0.063973	-1.18626	0.23552	NA
GSG2	0.663439	0.075886	0.047632	1.593155	0.111125	NA
HIST1H2A	6.605407	-0.07587	0.081569	-0.93019	0.352273	NA
LOC10027	3.759397	-0.07587	0.074764	-1.01485	0.310178	NA
SCYL2	172.0308	0.075868	0.049995	1.517512	0.129138	NA
SOGA1	2755.584	0.075854	0.051075	1.485145	0.137505	0.489381
PNRC1	371.6687	-0.07584	0.061546	-1.23217	0.217887	0.569219
SRSF8	144.1565	0.075827	0.057059	1.32891	0.183878	NA
ADSSL1	4.609473	-0.07583	0.08197	-0.92503	0.354949	NA
ZNF789	71.02178	-0.07582	0.063934	-1.18584	0.235687	NA
ATP2A3	110.8789	-0.07581	0.08432	-0.89909	0.368605	NA
OSTF1	52.64467	0.075793	0.061086	1.240752	0.214697	NA
SCARNA15	3.628322	-0.07579	0.078742	-0.96253	0.335784	NA
CHI3L1	33.45177	-0.07577	0.060357	-1.25535	0.209351	NA
PIP5KL1	3.855727	0.075728	0.078357	0.966452	0.333818	NA
AGBL4	9.805041	-0.07571	0.083525	-0.90646	0.364694	NA
DNAAF1	12.58439	-0.0757	0.08467	-0.8941	0.371268	NA
MAPK9	497.5901	0.075695	0.057893	1.307501	0.191042	0.542245
NPFF	10.31795	0.075681	0.084727	0.893242	0.371728	NA
SNAPC2	22.18312	0.075661	0.078262	0.96676	0.333664	NA
MGC2752	67.00163	0.075658	0.055192	1.370816	0.170432	NA
EN1	1.826252	0.075644	0.056748	1.332986	0.182537	NA
AOX2P	0.448028	-0.07562	0.038189	-1.98012	0.04769	NA
NYAP1	130.882	0.075611	0.054853	1.37842	0.168074	NA
DPP8	333.0594	0.075609	0.053804	1.405271	0.159941	NA

BCKDHA	48.98626	-0.07561	0.069574	-1.08668	0.277176	NA
PGM2L1	149.3258	0.075604	0.076426	0.989251	0.322541	NA
MARK2	231.9767	0.075591	0.04599	1.643627	0.100253	NA
UHRF1	5.813417	-0.07559	0.081911	-0.92283	0.356096	NA
IREB2	512.8171	0.075576	0.057547	1.31328	0.189089	0.541449
KATNB1	80.01718	-0.07557	0.053111	-1.42286	0.154777	NA
AHRR	3.741345	-0.07556	0.077635	-0.97324	0.330436	NA
PIGC	21.00554	-0.07555	0.077553	-0.97413	0.329992	NA
MRI1	94.48701	-0.07554	0.064713	-1.16724	0.243114	NA
SLC25A15	29.46776	0.075534	0.076585	0.986267	0.324002	NA
GIT1	318.2503	0.075503	0.049152	1.53611	0.124511	NA
ZNF879	36.42293	0.07549	0.07323	1.030854	0.302609	NA
TM9SF2	184.6788	0.075473	0.054596	1.382381	0.166855	NA
E4F1	201.5939	0.075471	0.071448	1.056313	0.290825	NA
RAPGEF1	529.2737	0.075463	0.049047	1.538579	0.123907	0.477296
CISH	3.379526	0.075456	0.072293	1.043752	0.2966	NA
PI4KAP2	23.16866	0.075442	0.084395	0.893915	0.371367	NA
RARRES2	17.72181	-0.07542	0.080379	-0.93835	0.348065	NA
ACTN3	12.11029	-0.07542	0.084633	-0.89111	0.372873	NA
ING2	72.04757	0.075415	0.072996	1.033135	0.301541	NA
WIPF2	276.2194	0.07541	0.044652	1.688837	0.091251	NA
FGD3	3.372451	-0.07541	0.072127	-1.04545	0.295814	NA
ERAS	1.073571	0.075396	0.054282	1.388983	0.164838	NA
PGLS	17.89131	0.075393	0.079472	0.948672	0.342787	NA
GRK6	72.52693	-0.07538	0.069827	-1.07953	0.280352	NA
DDAH1	268.7499	0.075366	0.072358	1.041572	0.29761	NA
SGPP1	85.40462	0.075342	0.069052	1.091093	0.275232	NA
TMEM121	5.59642	-0.07534	0.082767	-0.91026	0.362683	NA
ANO2	17.65559	-0.07534	0.083824	-0.89879	0.368765	NA
GCSHP3	49.01244	0.075332	0.067495	1.116114	0.264374	NA
ZNF527	86.01771	0.075327	0.061139	1.232057	0.217928	NA
SCNN1A	0.794458	0.075319	0.041966	1.794772	0.07269	NA
MRPS33	13.15851	-0.07532	0.083831	-0.89845	0.368948	NA
LOC28392	92.06961	0.075314	0.065452	1.150677	0.249865	NA
CBX6	298.3492	0.075298	0.047174	1.596197	0.110445	NA
RPL39L	2.508738	-0.07529	0.07219	-1.043	0.296949	NA
PRSS27	4.368395	0.075271	0.075262	1.000122	0.317251	NA
UBE2Q2	120.5941	0.075268	0.062291	1.208328	0.226921	NA
UBQLNL	0.870223	-0.07523	0.048568	-1.54896	0.121392	NA
CTNNA1	239.25	-0.07523	0.063125	-1.19173	0.233368	NA
DAD1	45.38627	-0.07523	0.077967	-0.96484	0.334626	NA
CKAP2L	3.205755	-0.07518	0.068958	-1.09027	0.275596	NA
NACA	1204.673	-0.07517	0.057063	-1.31729	0.18774	0.540017
LOC64521	50.38659	-0.07516	0.068565	-1.09622	0.272985	NA
CRYZL1	166.8809	-0.07516	0.050313	-1.49386	0.135212	NA
SYNGR3	50.60461	0.075145	0.074512	1.008495	0.313217	NA
HSPB6	24.62861	0.07514	0.078749	0.954174	0.339995	NA

TYRO3	291.2159	0.075137	0.046789	1.605863	0.108304	NA
GSTO2	7.271754	-0.07513	0.083301	-0.90191	0.367105	NA
RFX3	250.0439	0.075123	0.043014	1.746451	0.080733	NA
DDX60L	22.36474	-0.07512	0.08428	-0.89135	0.372744	NA
MKL1	174.496	0.075107	0.049998	1.502186	0.133049	NA
PSMC4	141.2225	0.075097	0.058825	1.276609	0.20174	NA
B4GALT6	298.6215	0.075084	0.060866	1.233599	0.217352	NA
TTLL2	1.037927	-0.07506	0.052671	-1.42504	0.154146	NA
PREP	77.70623	0.075055	0.061631	1.21782	0.223292	NA
CBY1	54.23775	-0.07505	0.060207	-1.24654	0.212565	NA
HAX1	70.06441	-0.07503	0.072951	-1.02853	0.3037	NA
CDYL2	243.7714	0.075023	0.053267	1.408424	0.159005	NA
SAP30L	123.789	0.075022	0.058736	1.277279	0.201504	NA
MAPT	1074.989	0.075009	0.042831	1.751269	0.0799	0.397742
LINC00674	744.7538	-0.07499	0.047181	-1.58952	0.111944	0.461005
PCDHA7	28.48097	-0.07499	0.076797	-0.9765	0.328815	NA
DDX21	158.5893	0.074985	0.048525	1.545271	0.122281	NA
ZNF396	5.979612	-0.07498	0.084177	-0.89073	0.373075	NA
ARHGAP11	2.155538	-0.07498	0.06663	-1.12527	0.260473	NA
CHRDL2	0.810797	-0.07497	0.04863	-1.54174	0.123137	NA
CYP4X1	39.49359	0.074974	0.083479	0.89812	0.369121	NA
PITPNM3	470.9242	-0.07497	0.052447	-1.42948	0.152867	0.506203
SNORD116	49.2668	-0.07496	0.08488	-0.88311	0.377176	NA
S100A4	2.242874	-0.07496	0.066695	-1.1239	0.261056	NA
LOC10013	16.38357	0.074928	0.084431	0.887457	0.374833	NA
AVPI1	23.77587	-0.07493	0.076216	-0.98308	0.32557	NA
SUGT1P3	10.47456	-0.0749	0.084848	-0.88275	0.377373	NA
RNF10	459.5423	0.074888	0.0382	1.960409	0.049948	0.34688
EMX2	5.69047	-0.07488	0.08204	-0.91272	0.361389	NA
PKI55	119.5824	0.074875	0.077471	0.966493	0.333797	NA
LOC10013	1.773903	-0.07486	0.04586	-1.63225	0.102627	NA
KIF18B	0.569412	-0.07485	0.043238	-1.73108	0.083437	NA
STRA13	26.97901	-0.07485	0.079403	-0.94264	0.345864	NA
MRPS30	111.5922	0.074835	0.052367	1.429033	0.152995	NA
JUP	68.2708	0.074823	0.078761	0.950008	0.342108	NA
ERAP2	24.59726	-0.07482	0.084927	-0.881	0.378318	NA
NFIL3	72.83401	0.074819	0.067355	1.110813	0.266649	NA
CRYAB	607.9794	-0.07482	0.081792	-0.91471	0.360343	0.683565
MIR3198-1	2.962706	0.074813	0.075196	0.994917	0.319777	NA
SFXN1	186.6321	-0.07481	0.035478	-2.1086	0.034979	NA
NAA60	128.0831	0.074798	0.055172	1.355722	0.175188	NA
CRAT	82.39357	-0.0748	0.064774	-1.15473	0.248199	NA
RAB40AL	13.97951	-0.0748	0.084937	-0.8806	0.378532	NA
RNF214	228.2449	0.074789	0.038376	1.948863	0.051312	NA
MYEOV2	23.37292	-0.07478	0.084859	-0.88125	0.378185	NA
HAND2	18.17504	0.074764	0.059157	1.263828	0.206292	NA
SOCS7	631.8281	0.074758	0.04633	1.613586	0.106617	0.452973

CALM3	1188.884	0.074745	0.071581	1.044212	0.296387	0.628575
RAG1	2.227575	-0.07474	0.065125	-1.14767	0.251105	NA
BTNL2	1.959424	0.074734	0.059379	1.258579	0.208182	NA
GRIN2B	47.85471	-0.07473	0.084924	-0.88	0.378858	NA
ZNF563	42.57505	0.074723	0.070106	1.065858	0.286488	NA
BNIPL	20.92197	0.07472	0.084841	0.880705	0.378478	NA
RGAG1	13.51346	0.074707	0.084394	0.885222	0.376037	NA
TRMT61B	73.53075	0.074684	0.061107	1.222176	0.221641	NA
PDE12	93.38385	0.074679	0.055418	1.347555	0.177802	NA
PTMS	408.2964	0.07467	0.067966	1.098649	0.271921	0.612663
FAM126A	27.38965	-0.07467	0.082339	-0.90684	0.364489	NA
HNRNPKP	1.434281	0.074665	0.059221	1.260782	0.207387	NA
SSR4P1	5.629499	-0.07466	0.081986	-0.9107	0.362455	NA
SMAD7	76.69621	0.074636	0.064366	1.159555	0.24623	NA
ITPR1	2328.682	-0.07463	0.084932	-0.87874	0.379543	0.699234
OVOL3	7.217182	-0.07462	0.083854	-0.88985	0.373545	NA
SH3BP1	31.72307	0.074603	0.078327	0.952448	0.34087	NA
COL5A1	39.70746	-0.0746	0.083888	-0.88926	0.373864	NA
LOC34050	1.463221	-0.0746	0.060464	-1.23372	0.217308	NA
SH3TC1	8.581522	-0.07457	0.08145	-0.91558	0.359889	NA
MRPL16	44.13631	0.074572	0.061995	1.20288	0.229023	NA
PRKAG2	243.546	-0.07456	0.045814	-1.62734	0.103664	NA
CMPK2	4.052411	-0.07455	0.078089	-0.95473	0.339715	NA
FAM72B	1.455121	-0.07455	0.063902	-1.16667	0.243345	NA
KCNK1	359.9974	0.074549	0.051757	1.440364	0.149764	0.502511
SYT11	1197.855	0.074547	0.05377	1.386394	0.165627	0.52098
FAM115C	13.63279	-0.07455	0.081396	-0.91584	0.359749	NA
GABRG3	6.211439	-0.07454	0.080828	-0.92219	0.356431	NA
DUS3L	31.83951	0.074537	0.072839	1.023311	0.306161	NA
MIR3127	1.175307	0.074523	0.055343	1.346555	0.178124	NA
HIST1H1E	150.0267	-0.07452	0.081881	-0.9101	0.362772	NA
UPP1	139.5779	0.074502	0.076797	0.970116	0.331989	NA
SPRYD7	63.37884	0.074489	0.065799	1.132067	0.257606	NA
RPL11	273.3817	-0.07449	0.066645	-1.11769	0.263699	NA
EPS15	677.7102	-0.07448	0.048476	-1.53651	0.124414	0.47732
CLEC2D	10.02652	-0.07448	0.084928	-0.87698	0.380498	NA
FAM84A	21.46937	0.074452	0.0846	0.880048	0.378833	NA
MTF1	152.1807	0.074439	0.056429	1.31916	0.187116	NA
P2RX5	8.377031	0.074438	0.084836	0.877431	0.380253	NA
TMEM133	3.213505	-0.07444	0.071848	-1.03604	0.300181	NA
KANSL3	280.3421	-0.07444	0.038292	-1.94387	0.051911	NA
RIOK1	100.4775	0.074427	0.06762	1.100656	0.271046	NA
LINC00467	2.244257	-0.07442	0.068771	-1.0822	0.279164	NA
LOC65371	4.168716	0.074384	0.077912	0.954728	0.339715	NA
JUND	613.1369	0.074381	0.053057	1.401895	0.160947	0.519239
LOC10050	0.545535	0.074379	0.041272	1.802171	0.071519	NA
SHISA2	1.570466	-0.07437	0.061797	-1.20346	0.228799	NA

RABGAP1L	1115.612	0.074369	0.045176	1.646218	0.099719	0.436963
AGPAT6	573.4306	-0.07437	0.051734	-1.43748	0.150581	0.503048
SLC39A12	7.152199	-0.07436	0.082881	-0.89719	0.369618	NA
NDUFAF4P	10.91457	-0.07436	0.084637	-0.87855	0.379644	NA
KIF5C	4549.944	0.074355	0.044203	1.682123	0.092545	0.41977
TBCB	75.26899	0.074349	0.065305	1.138478	0.254921	NA
BMP7	35.11937	0.074348	0.081987	0.906831	0.364496	NA
PCDH9-AS	13.79605	-0.07434	0.08486	-0.87605	0.381005	NA
PTPN12	304.7957	0.074341	0.04794	1.550715	0.12097	NA
FBXL8	6.372615	0.074336	0.083766	0.887426	0.37485	NA
SNORD114	3.084117	0.074322	0.066205	1.122593	0.26161	NA
LOC10061	0.852814	0.074315	0.050391	1.474776	0.140273	NA
PLA2G5	7.295304	-0.0743	0.078226	-0.9498	0.342214	NA
LOC72968	6.314093	-0.0743	0.081603	-0.91045	0.362585	NA
ANP32AP1	1.52107	0.074295	0.062255	1.1934	0.232713	NA
PCED1A	60.12224	-0.07429	0.061526	-1.20753	0.22723	NA
TBX6	4.362561	0.07429	0.080139	0.927017	0.353918	NA
LINC00526	10.45191	-0.07429	0.084464	-0.8795	0.379129	NA
POLL	63.10029	-0.07428	0.066669	-1.11421	0.265189	NA
LOC72874	35.75495	-0.07428	0.066226	-1.12154	0.262058	NA
CKB	1006.325	-0.07427	0.063719	-1.16561	0.243772	0.592428
OLFM2	91.67572	0.074267	0.072081	1.03032	0.30286	NA
FBXL13	22.81184	-0.07427	0.077514	-0.95809	0.338017	NA
ZNF273	246.4864	0.074259	0.050961	1.457175	0.145068	NA
EBLN2	14.06082	-0.07425	0.082455	-0.90044	0.367885	NA
EPSTI1	8.131755	0.074244	0.078332	0.947816	0.343223	NA
ZNF680	247.1753	0.07423	0.060584	1.22525	0.220481	NA
PPP1CB	703.4206	0.074202	0.064055	1.158412	0.246696	0.592704
EFHA2	310.7173	0.074194	0.061079	1.214714	0.224475	NA
IGHMBP2	132.3208	0.074185	0.065113	1.139334	0.254564	NA
CD72	5.545628	-0.07418	0.078725	-0.9423	0.346038	NA
MYBL1	45.05888	0.074183	0.073905	1.003759	0.315495	NA
TECR	125.4513	-0.07418	0.052678	-1.40814	0.159089	NA
VAT1L	526.0712	0.074164	0.06754	1.09808	0.27217	0.612663
ZNF761	103.416	0.074164	0.073458	1.009613	0.312681	NA
KIRREL3	89.36159	0.074155	0.068116	1.088665	0.276302	NA
GJB3	10.50287	-0.07415	0.08479	-0.87455	0.38182	NA
BAG2	34.35002	0.074143	0.084149	0.881093	0.378268	NA
ZNF140	79.2566	0.074143	0.05323	1.392882	0.163655	NA
LINC00263	20.53657	-0.07414	0.082254	-0.90137	0.367394	NA
SLC52A1	11.93762	0.074124	0.083142	0.89154	0.37264	NA
ECM1	24.41129	0.074119	0.084791	0.874137	0.382043	NA
HSD11B1	57.63458	-0.07412	0.078335	-0.94615	0.34407	NA
CHKB	1.006388	0.074094	0.05404	1.371077	0.170351	NA
LOC15062	42.01651	-0.07407	0.084929	-0.87214	0.383131	NA
HPGD	0.446061	-0.07406	0.037135	-1.99438	0.04611	NA
TWF2	78.18927	-0.07406	0.05992	-1.236	0.216459	NA

POLE3	135.0458	-0.07403	0.059498	-1.24427	0.213402	NA
SYTL5	1.268658	0.07403	0.059136	1.251865	0.210619	NA
KCNE4	9.503447	-0.07402	0.067518	-1.09632	0.272938	NA
FBXO4	39.27854	-0.07401	0.076573	-0.96654	0.333773	NA
ZNF707	15.66699	0.073999	0.082081	0.901532	0.367306	NA
PPP1R14B	14.85347	-0.07399	0.082781	-0.89385	0.371403	NA
C1orf109	36.02069	0.073989	0.074036	0.99936	0.317621	NA
OR13J1	6.429741	-0.07398	0.082091	-0.90125	0.367456	NA
IPO5	592.4272	0.073977	0.047322	1.563276	0.117988	0.472599
PFDN6	29.37405	0.073958	0.070017	1.056286	0.290837	NA
GRID2	400.834	-0.07393	0.053057	-1.39345	0.163485	0.52098
FAM91A1	189.6897	0.073927	0.052271	1.414285	0.157278	NA
SEC14L1	658.4191	-0.07389	0.053262	-1.38727	0.165358	0.52098
ZNF721	494.4744	0.073879	0.038114	1.938359	0.052579	0.355768
ABCC6	5.349447	0.073878	0.075147	0.983112	0.325552	NA
CPNE1	126.2779	-0.07388	0.069775	-1.0588	0.289692	NA
SNORD19	1.094551	-0.07388	0.053016	-1.3935	0.163468	NA
PCDHA12	41.20067	0.07386	0.074781	0.987681	0.323309	NA
LYPLAL1	51.44638	-0.07386	0.063713	-1.15923	0.24636	NA
TSSK6	3.423097	0.073856	0.078146	0.945102	0.344607	NA
TMEM184	100.0948	0.073854	0.051632	1.430382	0.152607	NA
FAM173A	22.1564	0.073835	0.077481	0.952934	0.340623	NA
N6AMT1	22.85558	-0.07383	0.076315	-0.96739	0.333348	NA
PRPF40B	90.505	-0.07382	0.056833	-1.29884	0.194	NA
MSTO2P	22.00496	-0.0738	0.076291	-0.96735	0.333367	NA
RBP1	35.00492	-0.0738	0.080795	-0.91339	0.361036	NA
CLCN4	517.059	0.073785	0.061326	1.203166	0.228912	0.577536
C15orf55	7.724598	0.073782	0.082344	0.896021	0.370242	NA
C10orf11	2.871763	0.073776	0.075694	0.974658	0.32973	NA
SLC9A9	18.98118	-0.07375	0.084197	-0.87592	0.381075	NA
YIPF3	88.73201	-0.07375	0.052693	-1.39959	0.161635	NA
CPEB4	1329.615	0.073728	0.039448	1.869	0.061623	0.37053
MPHOSPH	166.3428	0.073718	0.059327	1.242566	0.214028	NA
LOC10013	8.464318	-0.07371	0.084116	-0.87632	0.380855	NA
PABPN1	182.7237	-0.07371	0.061494	-1.19862	0.230675	NA
C19orf60	61.72118	-0.07371	0.073034	-1.0092	0.31288	NA
C16orf45	317.5384	0.073696	0.051185	1.439797	0.149925	NA
VTA1	123.8298	0.073692	0.070229	1.049313	0.294034	NA
RARS	125.0924	-0.07369	0.05852	-1.25923	0.207948	NA
USP30-AS1	3.540104	-0.07369	0.075668	-0.97383	0.330139	NA
ZNF500	63.2312	-0.07368	0.062852	-1.17232	0.241067	NA
LOC64785	3.835659	-0.07368	0.04487	-1.64205	0.100581	NA
GPRIN3	688.9678	0.073661	0.054151	1.360297	0.173736	0.526085
LOC72917	2.525649	0.073649	0.073136	1.00702	0.313925	NA
ARGFXP2	24.62633	-0.07364	0.080023	-0.92021	0.357465	NA
LOC73018	14.30281	0.073622	0.08135	0.905011	0.36546	NA
ZC4H2	55.92741	-0.07361	0.061459	-1.19779	0.230998	NA

KCNF1	3.984585	0.073599	0.076613	0.960656	0.336725	NA
TMEM249	10.86507	0.073597	0.084308	0.872957	0.382686	NA
PDXDC1	454.6488	0.07358	0.047655	1.543993	0.12259	0.476542
CYLD	400.7439	0.073577	0.034535	2.130519	0.033129	0.2864
HYAL3	22.2622	0.073569	0.084026	0.875548	0.381276	NA
THEM6	22.6276	-0.07356	0.07859	-0.93601	0.349266	NA
KIF5B	1249.729	0.073551	0.032248	2.280782	0.022561	0.23963
ABCG1	163.5339	0.07353	0.063083	1.165594	0.243779	NA
SKA1	2.624116	0.073526	0.07291	1.008449	0.313239	NA
RNF208	31.80687	0.073515	0.069687	1.054926	0.291459	NA
CASP1	6.788262	-0.07351	0.079095	-0.92935	0.352707	NA
EIF2D	56.53857	-0.0735	0.063139	-1.16417	0.244357	NA
TCF20	509.4369	0.073503	0.050945	1.442809	0.149074	0.502511
CDK1	0.68687	-0.0735	0.044436	-1.65409	0.09811	NA
HPS5	129.0455	-0.07349	0.05444	-1.35001	0.177014	NA
UQCRHL	9.591311	-0.07346	0.084518	-0.86922	0.384729	NA
TRANK1	1018.235	-0.07346	0.078567	-0.935	0.349787	0.674334
CTSC	6.10276	-0.07346	0.079968	-0.9186	0.358306	NA
SPESP1	5.3341	0.073431	0.074148	0.990334	0.322011	NA
MDH1B	11.2726	-0.07342	0.083443	-0.8799	0.378912	NA
DNAJC21	328.4535	0.073421	0.041409	1.773037	0.076223	NA
BDNF-AS	21.24137	0.073413	0.083488	0.879319	0.379228	NA
MIR598	1.613529	-0.07341	0.063431	-1.15731	0.247146	NA
CRELD1	274.9829	0.073407	0.07134	1.028967	0.303495	NA
SCAF8	728.8349	0.073407	0.03451	2.127107	0.033411	0.286653
OSBP2	263.9014	0.073394	0.045547	1.611399	0.107093	NA
LOC64563	0.830942	0.073389	0.048351	1.517828	0.129058	NA
PLAT	45.77226	0.07338	0.081817	0.896876	0.369785	NA
APOBEC3F	2.092299	0.073368	0.063399	1.157245	0.247172	NA
C10orf35	43.42148	0.073354	0.064893	1.130386	0.258314	NA
HMGN2P4	2.584731	-0.07335	0.074516	-0.98432	0.32496	NA
TMEM30B	1.014332	-0.07334	0.046373	-1.58145	0.113776	NA
VENTX	1.076251	-0.07332	0.056346	-1.30132	0.19315	NA
LRRC25	0.728495	-0.07332	0.043972	-1.66746	0.095424	NA
CCDC68	4.586211	-0.0733	0.081702	-0.89722	0.369602	NA
IQSEC1	995.0755	0.0733	0.050141	1.461864	0.143779	0.495647
FANK1	2.844446	0.073281	0.076501	0.957912	0.338107	NA
SLC25A32	130.8022	0.07328	0.04949	1.480719	0.138681	NA
ASXL1	311.1768	-0.07328	0.041111	-1.78245	0.074676	NA
TMEM190	8.51524	0.07324	0.084622	0.865498	0.386765	NA
PIN4	26.31078	-0.07324	0.07843	-0.93383	0.350393	NA
MIER1	307.8663	0.073238	0.035743	2.048998	0.040462	NA
ARHGEF38	2.842833	-0.07323	0.074259	-0.98616	0.324056	NA
EHD3	201.4599	0.073219	0.064566	1.134019	0.256787	NA
TRIP6	24.26519	-0.07321	0.075661	-0.96762	0.333235	NA
ANXA6	315.2939	-0.07321	0.054165	-1.35155	0.17652	NA
CCDC94	63.09537	0.073204	0.060932	1.201411	0.229592	NA

SKA2	316.6083	0.073203	0.064732	1.130874	0.258108	NA
MPL	5.558702	-0.0732	0.082865	-0.8834	0.377022	NA
WNT16	2.268748	-0.07319	0.067768	-1.08002	0.280134	NA
WDR11-AS	28.59117	-0.07317	0.078479	-0.93237	0.351143	NA
GPN1	70.26868	0.073156	0.057337	1.275895	0.201993	NA
SDHD	21.90726	-0.07314	0.078617	-0.93038	0.352175	NA
VAMP4	324.3842	0.073138	0.066454	1.100581	0.271079	NA
ZBTB39	96.10756	0.073137	0.057524	1.271416	0.203581	NA
CAPN5	43.71484	0.07313	0.070234	1.041235	0.297766	NA
SMARCA2	1339.075	0.073124	0.033943	2.154323	0.031215	0.277495
ZNF514	200.1767	-0.07311	0.077958	-0.93781	0.348343	NA
DGKH	224.2105	0.073109	0.060531	1.207803	0.227123	NA
DYX1C1	0.756375	-0.0731	0.048766	-1.49902	0.133869	NA
FAM209A	0.607201	-0.07309	0.040245	-1.81611	0.069353	NA
DNAJC6	728.5656	0.073086	0.055621	1.313998	0.188847	0.54144
HNRNPH3	684.9321	0.073054	0.032556	2.243938	0.024836	0.247662
MLL3	2684.307	0.073045	0.037442	1.950914	0.051067	0.348396
AKAP12	7234.925	0.07304	0.055202	1.323139	0.185789	0.536751
GCSAML	53.13778	0.073029	0.073827	0.989192	0.322569	NA
ZNF776	311.5254	0.073028	0.052994	1.378053	0.168187	NA
PODXL2	311.956	0.073	0.048007	1.520605	0.128359	NA
DNAJC19	49.53886	-0.07299	0.06813	-1.07131	0.284031	NA
CELF4	486.8195	0.072987	0.045851	1.591816	0.111426	0.460575
CAMLG	104.7705	-0.07298	0.054044	-1.35042	0.176881	NA
SLC46A1	83.99166	0.072975	0.057371	1.271985	0.203378	NA
TBCCD1	57.79543	0.072975	0.064942	1.123696	0.261142	NA
COG5	236.6896	0.072972	0.044329	1.646145	0.099734	NA
LOC10012	122.1222	-0.07295	0.066187	-1.10217	0.270387	NA
KLHDC8A	89.9314	0.072949	0.072401	1.007575	0.313659	NA
FRMPD4	108.8206	-0.07295	0.084155	-0.86682	0.386043	NA
GOLGA6B	0.884649	-0.07294	0.053111	-1.37337	0.169638	NA
NRG2	244.8471	-0.07294	0.055576	-1.31245	0.189369	NA
CREBZF	693.0136	-0.07294	0.052594	-1.38678	0.165508	0.52098
CALB1	565.8692	-0.07292	0.083171	-0.87679	0.380602	0.699727
GPR37L1	92.10092	-0.07292	0.080732	-0.90326	0.366387	NA
EDEM2	64.96465	0.072918	0.059236	1.230981	0.21833	NA
ERC2-IT1	25.1904	-0.07292	0.081991	-0.88932	0.373831	NA
NRG3	55.15134	-0.07291	0.083893	-0.8691	0.384792	NA
SACM1L	211.1039	0.072906	0.044872	1.624753	0.104215	NA
C17orf47	2.511982	0.072899	0.072297	1.008328	0.313297	NA
ARSE	0.869635	-0.07289	0.047561	-1.53258	0.125379	NA
RASSF8	238.2167	0.072891	0.061947	1.176653	0.239334	NA
ARHGEF26	88.36776	-0.07288	0.07283	-1.00075	0.316947	NA
OST4	21.40892	-0.07288	0.079225	-0.91991	0.357618	NA
ATP2B2	2825.399	0.072875	0.05622	1.296243	0.194892	0.544304
LSM14A	526.0933	0.072871	0.043975	1.657096	0.0975	0.432168
FBXO15	8.371365	0.072866	0.08465	0.860793	0.389352	NA

SGTB	232.6573	-0.07286	0.066584	-1.09427	0.273835	NA
PTER	71.3814	0.072859	0.084824	0.858934	0.390377	NA
ORAOV1	90.76364	-0.07283	0.061833	-1.17792	0.238829	NA
RPA3	20.28928	-0.07282	0.081253	-0.89623	0.370131	NA
CDC42BPA	1715.805	0.072821	0.036747	1.981682	0.047515	0.337641
ZDHHC15	47.85936	0.072806	0.066141	1.100769	0.270997	NA
FLJ42393	18.99668	-0.0728	0.081238	-0.89608	0.370208	NA
SLC16A13	16.99511	-0.07279	0.081107	-0.89752	0.369444	NA
LOC10050	38.83585	-0.07277	0.072261	-1.00702	0.313925	NA
GHR	8.077713	-0.07275	0.084178	-0.86425	0.38745	NA
PIGO	72.22736	-0.07273	0.065632	-1.10815	0.267796	NA
RFX7	635.4735	0.072727	0.047833	1.520429	0.128403	0.48338
RALGPS1	397.1519	0.072719	0.042979	1.691968	0.090652	0.415466
GPATCH2L	163.722	0.072713	0.043267	1.680587	0.092843	NA
HUWE1	2707.005	0.072704	0.030941	2.349774	0.018785	0.231397
TAB3	181.8762	0.072701	0.061802	1.176346	0.239457	NA
DPY19L2P	53.73869	0.072698	0.078722	0.923467	0.355764	NA
RAB9B	161.8414	-0.07269	0.050136	-1.44976	0.147125	NA
GOPC	253.032	0.072668	0.042848	1.695947	0.089896	NA
TULP3	212.1511	-0.07267	0.057239	-1.26956	0.204243	NA
PRPF3	428.6336	-0.07264	0.0527	-1.37846	0.168062	0.521454
HMG20B	24.67988	0.072625	0.083597	0.868753	0.384982	NA
MID2	110.9053	0.072624	0.057599	1.260841	0.207366	NA
ERI2	16.31664	-0.07262	0.084878	-0.85558	0.392229	NA
NTNG2	50.88201	-0.07261	0.074899	-0.96946	0.332317	NA
GJD2	1.701499	-0.07261	0.065237	-1.11302	0.265698	NA
FGFR1OP2	246.4369	0.072598	0.042363	1.713687	0.086586	NA
FBL	50.44039	-0.07259	0.067675	-1.07264	0.283431	NA
UBE2V2	88.19899	0.072583	0.07176	1.011472	0.311791	NA
TCEAL8	135.9845	-0.07258	0.065989	-1.09982	0.271412	NA
ZFP64	59.44108	0.072569	0.059299	1.223791	0.221031	NA
RABEP2	28.81455	-0.07257	0.074338	-0.97617	0.32898	NA
RGL2	121.552	-0.07255	0.056345	-1.28769	0.197855	NA
POLR3E	146.5962	-0.07255	0.050184	-1.44569	0.148263	NA
ATXN7L2	58.2934	0.072549	0.072399	1.002077	0.316307	NA
SMIM3	22.12541	-0.07255	0.078147	-0.92832	0.353242	NA
SNORD82	4.085682	-0.07254	0.078706	-0.92162	0.356725	NA
ABCC11	2.267898	-0.07253	0.07127	-1.01774	0.308802	NA
STX1B	1300.699	0.072533	0.056355	1.287075	0.198068	0.545847
LOC14869	233.2426	-0.07253	0.078074	-0.92902	0.352881	NA
SLURP1	1.960778	0.072526	0.067125	1.080465	0.279935	NA
ZBTB9	18.45428	0.072525	0.080598	0.899835	0.368208	NA
PLCB1	300.7123	0.072517	0.07377	0.98301	0.325603	NA
PSMD2	287.9615	0.072516	0.051984	1.394956	0.163029	NA
TRAPPC4	25.31247	-0.07251	0.078564	-0.92298	0.356019	NA
COLEC12	72.75413	-0.0725	0.082563	-0.87816	0.379859	NA
DARS	188.9475	-0.07247	0.072087	-1.00531	0.314748	NA

TVP23A	43.3026	-0.07247	0.082077	-0.88292	0.377282	NA
SLC35A3	115.2877	0.072465	0.060168	1.204374	0.228445	NA
VEGFB	72.96221	-0.07246	0.064017	-1.13195	0.257653	NA
B3GALT4	19.59138	-0.07246	0.079426	-0.91232	0.361602	NA
FAM174B	17.29687	0.072447	0.081104	0.89326	0.371718	NA
LRIF1	81.86787	0.072423	0.067021	1.080603	0.279874	NA
IRAK1BP1	30.05435	0.072411	0.075202	0.962887	0.335604	NA
C12orf54	1.174218	0.072368	0.058545	1.236114	0.216416	NA
CUL1	409.9153	0.072361	0.033502	2.159925	0.030779	0.277495
ZNF391	24.89916	0.072357	0.07858	0.920812	0.357149	NA
DDX25	65.62694	-0.07235	0.065411	-1.10612	0.268674	NA
GLTP	46.821	-0.07235	0.0699	-1.03505	0.300647	NA
PLEKHF2	36.12519	-0.07235	0.071473	-1.01225	0.311418	NA
GDI1	866.8343	0.07234	0.048868	1.480305	0.138792	0.490222
PIM3	36.99874	-0.07233	0.077293	-0.93584	0.349357	NA
THEMIS2	7.997798	-0.07233	0.080727	-0.89598	0.370261	NA
IKZF2	142.8859	-0.07233	0.058926	-1.22741	0.219669	NA
PCDHAC1	54.6349	0.07231	0.068663	1.05311	0.292291	NA
MKX	9.054541	-0.07231	0.084332	-0.85739	0.391228	NA
TMEM107	38.04695	-0.0723	0.084855	-0.85202	0.394205	NA
MIR324	2.66096	-0.07228	0.070283	-1.02847	0.303729	NA
ARHGAP12	368.514	-0.07228	0.038574	-1.87369	0.060974	0.369265
MIR4786	0.914506	-0.07226	0.05298	-1.36399	0.172569	NA
SVOPL	0.435089	0.072252	0.038237	1.88958	0.058814	NA
SYNE1	18375.06	0.072244	0.06149	1.174887	0.24004	0.589893
GSTA4	154.8478	-0.07223	0.057145	-1.26406	0.20621	NA
TCEB3B	4.378209	-0.07223	0.078966	-0.91473	0.360335	NA
GSTO1	54.35633	-0.07223	0.075877	-0.95191	0.341143	NA
N4BP2	220.7989	-0.07222	0.05902	-1.22358	0.22111	NA
HAS1	10.23259	0.072202	0.084867	0.850765	0.3949	NA
MIR620	2.839209	0.072196	0.07471	0.966349	0.333869	NA
E2F8	3.547968	0.072193	0.068161	1.059163	0.289526	NA
VPS28	66.4146	-0.07219	0.059824	-1.20673	0.227535	NA
FAM181B	37.7536	-0.07219	0.082128	-0.87901	0.379394	NA
IGFLR1	4.486063	-0.07219	0.082077	-0.87953	0.379114	NA
CHMP2B	70.1336	-0.07219	0.066006	-1.09367	0.274099	NA
SAMD9L	33.43362	-0.07218	0.08459	-0.85331	0.39349	NA
CCNL1	300.8497	-0.07217	0.050506	-1.42894	0.153021	NA
TRMT2B	33.53404	-0.07215	0.072573	-0.99415	0.320151	NA
SLIT3	601.7153	0.072144	0.044119	1.635216	0.102004	0.443332
OXSM	9.040467	0.072103	0.084869	0.849578	0.39556	NA
RBBP6	668.8297	0.0721	0.051876	1.389846	0.164576	0.52098
LOC10013	28.22641	-0.07209	0.075372	-0.95652	0.338807	NA
COG8	68.33969	-0.07209	0.059888	-1.20379	0.22867	NA
MT1G	2.897747	-0.07208	0.064525	-1.11705	0.263973	NA
FPR1	4.143082	-0.07207	0.067273	-1.07135	0.284013	NA
ARL4A	16.93682	-0.07207	0.08417	-0.85623	0.391869	NA

RRAGD	467.6959	0.072066	0.05018	1.43615	0.15096	0.503569
USP7	650.8569	0.072057	0.031974	2.253647	0.024218	0.246583
NKAPL	16.41799	0.072046	0.084932	0.848275	0.396285	NA
CCT6P1	48.32304	-0.07201	0.062155	-1.15857	0.246632	NA
ORAI2	198.2315	0.071998	0.083854	0.858611	0.390555	NA
FSTL5	1302.126	0.071963	0.037644	1.91169	0.055916	0.3598
AKT2	387.9575	-0.07196	0.055739	-1.29098	0.196709	0.545356
UXS1	172.8389	0.071941	0.056451	1.274398	0.202522	NA
SUGP2	775.154	-0.07194	0.04848	-1.48387	0.137844	0.489381
TMEM14B	44.11726	-0.07193	0.075587	-0.95168	0.341258	NA
TRIM4	141.1575	0.071931	0.059861	1.201622	0.22951	NA
HS2ST1	159.381	0.071923	0.049623	1.449396	0.147227	NA
AKR1C2	6.616748	0.07192	0.082432	0.872476	0.382949	NA
CELSR3	636.4883	0.071918	0.055155	1.303922	0.19226	0.542492
FAM122C	8.041846	-0.07191	0.084723	-0.84878	0.396005	NA
SLC47A1	11.77595	-0.07189	0.084525	-0.85055	0.395019	NA
LNX1-AS1	1.532119	0.071883	0.06382	1.126339	0.260022	NA
FAM69B	109.7489	-0.07187	0.056589	-1.27	0.204084	NA
NDUFAF2	30.63845	-0.07185	0.072262	-0.99436	0.320047	NA
YEATS2	591.9202	0.071829	0.03258	2.204682	0.027476	0.263821
CXCR4	4.938491	-0.07183	0.077545	-0.92628	0.354302	NA
WDFY1	164.0762	0.071822	0.050686	1.417008	0.156481	NA
FZD5	8.496127	-0.07182	0.08346	-0.86053	0.389494	NA
ZC3H7B	523.7142	0.071806	0.049105	1.462297	0.14366	0.495647
THSD4	24.6105	0.071789	0.084752	0.847043	0.396971	NA
PGAM4	1.211986	-0.07179	0.058678	-1.22342	0.221172	NA
TIMP1	36.74039	-0.07178	0.081257	-0.88337	0.377036	NA
MRPL45	43.91351	-0.07178	0.071764	-1.00022	0.317203	NA
RPRD1A	413.17	0.071764	0.051662	1.389115	0.164798	0.52098
ITPA	15.44473	-0.07176	0.08313	-0.8632	0.38803	NA
SEC14L1P1	28.1926	-0.07175	0.073478	-0.97645	0.328844	NA
SNORA24	8.301323	-0.07174	0.077581	-0.92472	0.355111	NA
TAC1	0.653799	0.071725	0.044499	1.611841	0.106997	NA
CTSK	22.53913	-0.07172	0.084743	-0.84637	0.397345	NA
ARL6IP6	19.46311	-0.07171	0.079455	-0.90258	0.366751	NA
CNPY4	43.2886	-0.07171	0.070463	-1.01769	0.308828	NA
VIT	6.120105	0.071696	0.074823	0.9582	0.337962	NA
HLA-DPB2	15.22212	-0.07169	0.083404	-0.85957	0.390025	NA
ZNF692	214.9703	-0.07168	0.063369	-1.13118	0.257981	NA
MIR4517	3.626001	-0.07168	0.07882	-0.90937	0.363155	NA
PSG3	1.183348	-0.07168	0.056278	-1.27361	0.2028	NA
CCDC112	86.188	0.071668	0.056874	1.260127	0.207624	NA
IRGC	2.008547	0.071664	0.05716	1.253749	0.209933	NA
MARS	241.2964	0.071663	0.048827	1.467678	0.142192	NA
SLC35A5	85.21202	0.07163	0.069974	1.023664	0.305994	NA
TNFSF12	21.83232	0.0716	0.080098	0.893907	0.371371	NA
DISC2	1.442566	-0.07156	0.058857	-1.2159	0.224021	NA

GINS2	10.22238	-0.07154	0.084528	-0.84635	0.397355	NA
TNFRSF18	1.57465	-0.07153	0.056656	-1.26252	0.206762	NA
B9D2	2.380317	-0.07153	0.070812	-1.01011	0.312443	NA
CORO2B	533.6897	0.071528	0.062209	1.149791	0.25023	0.592704
GATM-AS1	7.765831	-0.07153	0.084429	-0.84717	0.396902	NA
MRPS36	15.63298	-0.07151	0.083733	-0.85405	0.393075	NA
CCDC177	182.386	0.071511	0.046947	1.523239	0.127699	NA
VN1R2	0.374424	-0.07151	0.035339	-2.02348	0.043023	NA
NGEF	17.01617	0.071496	0.081772	0.874334	0.381937	NA
NTAN1	35.98766	-0.07148	0.071691	-0.99709	0.318719	NA
EBLN1	0.401723	0.071471	0.035878	1.99204	0.046367	NA
RNF181	25.88235	-0.07146	0.076399	-0.9353	0.349634	NA
ITGB3BP	28.34451	-0.07145	0.078049	-0.91546	0.359949	NA
PSMA5	61.23973	0.071447	0.081644	0.87511	0.381514	NA
EDF1	298.5192	-0.07143	0.045512	-1.56939	0.116558	NA
SUCLG1	101.8364	-0.07142	0.056441	-1.26538	0.205736	NA
MASP1	13.39906	-0.07142	0.084823	-0.84195	0.399815	NA
CCDC136	1112.026	0.071417	0.055336	1.290609	0.196839	0.545356
ZNF543	170.1907	0.071405	0.051508	1.386281	0.165661	NA
ZP2	72.88245	-0.0714	0.081758	-0.87337	0.382464	NA
MAP3K2	421.9163	0.071404	0.034721	2.056524	0.039732	0.305061
NBN	190.6641	-0.07139	0.054413	-1.31206	0.1895	NA
CT60	0.863888	-0.07138	0.044079	-1.61926	0.105391	NA
TTPAL	117.6894	-0.07137	0.049322	-1.44707	0.147877	NA
GABRG2	436.4788	0.07135	0.062949	1.133467	0.257018	0.598301
GSTM4	50.82435	-0.07134	0.072307	-0.98669	0.323796	NA
ZNF750	42.89577	-0.07134	0.074982	-0.9514	0.341399	NA
LOC64335	15.38638	0.071336	0.083826	0.850996	0.394771	NA
KLHL6	3.705333	-0.07133	0.073644	-0.9686	0.332744	NA
NEXN	40.60737	-0.07133	0.076471	-0.93276	0.350945	NA
SDS	16.94716	-0.07132	0.083845	-0.85062	0.39498	NA
FOS	33.95667	-0.07132	0.071769	-0.9937	0.32037	NA
PSAT1	49.0518	-0.07131	0.081314	-0.87701	0.380479	NA
BRWD1-IT	4.445992	-0.07131	0.082266	-0.86677	0.386069	NA
NDUFA1	57.24756	-0.0713	0.078914	-0.90356	0.366228	NA
RELL1	143.7033	0.071297	0.056749	1.256371	0.208981	NA
RPL13AP6	5.106884	-0.07129	0.082408	-0.8651	0.386985	NA
MOB1B	158.0675	0.071243	0.04873	1.461993	0.143743	NA
ACADS	11.46243	-0.07124	0.084857	-0.83956	0.401153	NA
TRIM26	122.6508	0.071219	0.049093	1.450703	0.146862	NA
HIST2H2B	14.13266	-0.07122	0.08347	-0.85318	0.393557	NA
PLEKHA6	813.563	0.071201	0.067737	1.051144	0.293192	0.625901
TMEM231	49.83852	-0.0712	0.064899	-1.09707	0.272611	NA
RRAS	10.5822	-0.07119	0.08313	-0.8564	0.391777	NA
ZNF443	26.62454	0.071182	0.079515	0.895196	0.370683	NA
CACNA1A	5165.266	-0.07118	0.048254	-1.47508	0.140192	0.492301
MGC3280	5.342981	0.071178	0.082429	0.863506	0.38786	NA

LOC28385	12.78912	0.071174	0.084067	0.846634	0.397199	NA
RASL11A	19.64569	0.071167	0.080532	0.883717	0.376849	NA
MIR3692	0.376309	-0.07116	0.035338	-2.01367	0.044044	NA
EFS	82.39628	-0.07116	0.076511	-0.93003	0.352356	NA
POM121L8	0.543936	-0.07116	0.03835	-1.85547	0.063529	NA
ZNF235	86.45632	0.071154	0.065618	1.084359	0.278206	NA
MAP4K1	6.525498	-0.07115	0.082259	-0.86499	0.387046	NA
ANO6	55.54878	-0.07115	0.080264	-0.88646	0.375371	NA
DENR	100.7646	0.071126	0.05514	1.289908	0.197083	NA
TRMT11	187.4533	-0.07112	0.052351	-1.35848	0.174311	NA
HIF1A	551.2153	0.071113	0.050884	1.397547	0.162249	0.519328
SLC2A4	1.62586	-0.0711	0.064606	-1.10048	0.271123	NA
ADAM23	279.0665	0.071097	0.051454	1.381757	0.167046	NA
LOC72908	13.44869	-0.0711	0.0843	-0.84337	0.399022	NA
LARS2-AS1	3.397946	-0.07109	0.075268	-0.94448	0.344925	NA
PPRC1	107.3987	0.071085	0.064323	1.105124	0.269106	NA
TMEM167	81.11434	0.071077	0.057912	1.227336	0.219696	NA
TOLLIP	235.7331	0.071073	0.051024	1.392934	0.16364	NA
AHCTF1	528.8803	0.071055	0.042289	1.680236	0.092911	0.419831
CDHR2	35.72478	-0.07105	0.075521	-0.94078	0.346819	NA
SH3BP5L	160.0859	-0.07104	0.052614	-1.35023	0.176943	NA
STAT5B	449.1064	0.071035	0.0478	1.486102	0.137252	0.489381
TM6SF2	1.572433	0.071025	0.06411	1.107869	0.267918	NA
PPM1A	931.0199	0.071024	0.04791	1.482433	0.138225	0.489381
ACLY	209.9265	0.071013	0.058172	1.220747	0.222182	NA
ATP1A3	1115.213	0.071002	0.072734	0.976193	0.328969	0.65961
PARD6G	24.41593	0.071002	0.081874	0.867202	0.385831	NA
OSCP1	29.88752	0.071	0.06836	1.038618	0.298982	NA
LOC10050	0.57838	-0.071	0.044789	-1.58518	0.112924	NA
SSSCA1	24.93644	-0.07099	0.074981	-0.94681	0.343733	NA
LILRB2	1.909196	-0.07099	0.06846	-1.03689	0.299786	NA
CCDC71	28.95549	-0.07099	0.079958	-0.88778	0.374661	NA
B3GNT1	236.5268	0.070978	0.07248	0.979267	0.327448	NA
AQR	320.7019	0.070964	0.035049	2.024716	0.042897	NA
XRCC5	694.5448	0.070959	0.052978	1.33942	0.180434	0.53091
TBX10	1.473132	0.070956	0.060306	1.176605	0.239353	NA
PRMT3	55.17792	-0.07095	0.072354	-0.98065	0.326766	NA
PALM3	8.743572	0.07094	0.081895	0.866238	0.38636	NA
WFIKKN2	8.514253	-0.07091	0.084827	-0.8359	0.403209	NA
OVCH1	0.438347	0.07089	0.038262	1.85272	0.063923	NA
CLUAP1	251.6642	0.070881	0.043978	1.611718	0.107023	NA
RHO	4.180379	-0.07088	0.076029	-0.93224	0.351211	NA
COL2A1	2.962773	-0.07087	0.075909	-0.93367	0.350474	NA
CRYGS	10.81026	-0.07087	0.084937	-0.83443	0.404039	NA
BCL7A	165.8958	0.070869	0.057502	1.232445	0.217783	NA
DNMT3B	33.23183	0.070866	0.071986	0.984439	0.3249	NA
GPER	41.79042	0.070829	0.082688	0.856587	0.391673	NA

ABL2	466.035	0.070819	0.039011	1.815345	0.069471	0.385666
MAP2	3955.581	0.070815	0.049173	1.4401	0.149839	0.502511
C1orf105	1.384197	-0.0708	0.060608	-1.16815	0.242745	NA
C6orf48	21.13895	-0.07079	0.083562	-0.8471	0.39694	NA
GLUD1	265.0853	-0.07077	0.0603	-1.17357	0.240568	NA
C1orf229	7.511747	-0.07076	0.084268	-0.83975	0.401047	NA
ALS2CR12	29.6936	-0.07075	0.07941	-0.89093	0.372968	NA
PTCSC3	2.304617	-0.07074	0.066472	-1.06427	0.287209	NA
FLJ37201	40.46371	0.070739	0.075273	0.93977	0.347336	NA
COPS7B	68.0206	0.070726	0.054993	1.286089	0.198412	NA
ZNF181	119.6137	0.070723	0.048184	1.467759	0.14217	NA
OR2C1	1.074833	-0.07072	0.051474	-1.37392	0.169465	NA
LRRC29	12.62889	0.07072	0.0839	0.842906	0.399281	NA
ARFGAP2	319.2578	-0.07072	0.035356	-2.00022	0.045476	NA
ZDHHC4	16.85289	-0.07072	0.082056	-0.86182	0.388784	NA
AKR1A1	40.22572	-0.07071	0.068849	-1.02703	0.304408	NA
ENGASE	220.0945	0.070707	0.078353	0.902421	0.366833	NA
NAGPA	18.52549	-0.0707	0.081513	-0.86741	0.38572	NA
AXIN2	97.81756	0.070705	0.051612	1.369915	0.170714	NA
SRSF6	510.4801	0.070698	0.058355	1.211509	0.2257	0.576749
C20orf26	28.56645	-0.07069	0.080943	-0.87327	0.382514	NA
DDX4	0.889588	-0.07068	0.052989	-1.33386	0.182249	NA
MB21D2	28.56731	0.07068	0.075187	0.940051	0.347191	NA
MGA	1791.478	0.070674	0.043416	1.627842	0.103558	0.445167
EPHA7	255.5865	0.070674	0.061949	1.140844	0.253935	NA
SLC25A37	35.99905	-0.07066	0.07559	-0.93482	0.34988	NA
ARNT2	342.0665	0.070646	0.058067	1.216636	0.223743	0.573926
REPS1	351.4782	-0.07064	0.056996	-1.23936	0.215211	0.564835
WDR12	40.22801	-0.07063	0.067038	-1.05364	0.292046	NA
LOC10028	17.04771	-0.07063	0.081721	-0.86433	0.387406	NA
TMEM248	233.0389	0.070629	0.049175	1.436275	0.150924	NA
LINC00565	0.601175	0.070627	0.043337	1.629735	0.103157	NA
ACAD10	96.72892	-0.07062	0.062958	-1.12168	0.261999	NA
LBX2	4.422959	-0.07061	0.077474	-0.91145	0.362057	NA
PWP2	81.9138	0.070598	0.068201	1.035146	0.300601	NA
PEG3	1234.769	0.070594	0.053644	1.315967	0.188185	0.540227
LIN9	47.0924	0.07059	0.070872	0.996019	0.319241	NA
MIR4712	3.868042	-0.07058	0.079292	-0.89018	0.373371	NA
PTPRG-AS	10.40902	-0.07058	0.084714	-0.83317	0.404751	NA
PSMB6	43.11712	-0.07058	0.075	-0.94105	0.346678	NA
PGPEP1L	2.199722	-0.07058	0.066404	-1.06286	0.287847	NA
IAH1	51.01878	0.070577	0.063775	1.106665	0.268439	NA
C4orf48	7.418003	-0.07058	0.084907	-0.83122	0.405848	NA
MTCP1	10.06225	0.070575	0.084879	0.831482	0.405701	NA
LOC10028	85.61004	-0.07057	0.077404	-0.91173	0.361911	NA
ATF5	14.39026	0.07057	0.083082	0.849402	0.395658	NA
NUP153	387.5936	0.070565	0.03256	2.167201	0.03022	0.275997

MED1	251.4285	0.07056	0.04264	1.654783	0.097969	NA
ATP6V0C	440.8383	0.070534	0.074365	0.948491	0.34288	0.666057
RAB1A	265.1435	0.07053	0.053275	1.323882	0.185542	NA
CYP2J2	50.92636	-0.07053	0.08443	-0.83536	0.403517	NA
MAB21L3	8.017521	-0.07052	0.082334	-0.85656	0.39169	NA
ALDH1L1-	1.649393	-0.0705	0.058024	-1.21509	0.22433	NA
TNP1	6.156012	-0.0705	0.082617	-0.8533	0.393495	NA
TTC1	237.896	-0.0705	0.048053	-1.46705	0.142362	NA
DCDC1	2.247519	-0.0705	0.065913	-1.06952	0.284834	NA
FAM163A	3.308208	0.070468	0.07714	0.91351	0.360974	NA
N4BP2L2	878.3254	-0.07047	0.052605	-1.33954	0.180394	0.53091
MIR626	1.050999	0.070446	0.055409	1.271388	0.203591	NA
SNORA78	0.888362	0.070438	0.051286	1.373419	0.169622	NA
LOC92249	108.9973	0.070427	0.059362	1.186401	0.235464	NA
COPG1	187.4276	0.070404	0.059587	1.181531	0.237392	NA
RSAD1	176.0255	0.070403	0.05938	1.185644	0.235763	NA
ZNF276	146.6736	-0.0704	0.057946	-1.21492	0.224396	NA
SH3RF3-AS	1.829023	0.07039	0.063568	1.107318	0.268156	NA
CNTNAP1	596.0491	0.070385	0.043787	1.607431	0.10796	0.452973
EPB41	1882.361	-0.07038	0.049873	-1.41126	0.158168	0.514729
NHEJ1	42.77378	0.070369	0.068421	1.028475	0.303726	NA
STARD3	110.8665	-0.07036	0.073855	-0.95265	0.340769	NA
DNTTIP2	261.6705	0.070357	0.054075	1.301109	0.193221	NA
METTL12	10.38363	-0.07035	0.084909	-0.82859	0.407337	NA
CCDC66	364.4118	-0.07035	0.052559	-1.33844	0.180754	0.53091
PTGDR2	7.743056	-0.07031	0.082588	-0.85137	0.394562	NA
IFNA1	3.606585	-0.0703	0.07514	-0.93555	0.349503	NA
TLR4	87.38976	-0.0703	0.08221	-0.85508	0.392507	NA
NR2F1	274.5415	-0.07029	0.065565	-1.07199	0.283725	NA
ZEB2	481.2679	-0.07026	0.068582	-1.02447	0.305616	0.635668
HSPA5	327.8656	0.070255	0.084172	0.834663	0.403907	NA
PRKCQ	8.289695	-0.07025	0.080724	-0.87025	0.384163	NA
KLHL5	250.4617	0.070229	0.080558	0.87179	0.383323	NA
VPS26B	302.5142	0.070222	0.037292	1.883041	0.059695	NA
ZNF555	151.4005	0.070221	0.060095	1.168502	0.242604	NA
PLA2R1	462.488	-0.07021	0.059047	-1.18912	0.234392	0.585333
DOC2B	178.2218	0.0702	0.064867	1.082214	0.279158	NA
ATAT1	69.07715	0.070196	0.057274	1.225618	0.220342	NA
HIST1H2BL	2.795538	-0.07019	0.072616	-0.96663	0.333727	NA
OIP5	8.106504	0.070184	0.084613	0.82947	0.406839	NA
IKBKB	270.8751	-0.07017	0.07111	-0.98684	0.32372	NA
HSDL1	111.8008	0.070172	0.06711	1.045619	0.295737	NA
LAMA5	1024.323	-0.07016	0.067852	-1.03407	0.301102	0.632662
BCMO1	7.679966	-0.07015	0.083466	-0.84047	0.400646	NA
FOXC2	1.119065	-0.07015	0.055408	-1.26603	0.205501	NA
CDC42SE1	140.537	0.070141	0.05595	1.253643	0.209972	NA
RPLP1	157.4166	-0.07013	0.068737	-1.02023	0.307619	NA

LOC10050	1.723767	-0.07012	0.055993	-1.25234	0.210444	NA
MON1B	88.34605	0.070103	0.057245	1.224616	0.22072	NA
FGF7	0.618587	-0.0701	0.042406	-1.65313	0.098304	NA
ENOX1	33.43573	-0.0701	0.082931	-0.84529	0.397951	NA
MGAT4C	12.68756	0.07007	0.084834	0.825975	0.408818	NA
VWA9	51.24725	-0.07005	0.071842	-0.97506	0.329529	NA
CDIPT	97.36477	-0.07005	0.054541	-1.28427	0.199046	NA
PCDHB8	10.56368	0.070037	0.084749	0.826402	0.408576	NA
STOX2	341.0566	0.07003	0.039922	1.754176	0.0794	0.397742
TRIT1	110.3276	-0.07002	0.05716	-1.22501	0.220573	NA
KRCC1	73.22215	-0.07	0.063015	-1.11078	0.266665	NA
SERPINB1	50.69853	-0.06999	0.072693	-0.96288	0.335608	NA
ATR	256.369	0.069992	0.055393	1.263544	0.206394	NA
SMTNL2	1.628349	0.069991	0.063484	1.102505	0.270242	NA
ZNF589	372.0566	-0.06997	0.054717	-1.27883	0.200956	0.547633
TIGD7	73.99097	0.069965	0.055001	1.272074	0.203347	NA
NPM3	3.432551	0.069963	0.076956	0.909136	0.363279	NA
IQCF1	1.442147	0.069959	0.056269	1.243296	0.213759	NA
LAMP3	1.949274	0.069952	0.069939	1.000186	0.31722	NA
SYN1	540.5028	0.069951	0.065132	1.073991	0.282827	0.617151
SLC44A3	51.0888	-0.06995	0.078955	-0.88592	0.375661	NA
PTRH1	3.972267	-0.06994	0.078576	-0.89003	0.373448	NA
KCNA5	3.391119	-0.06994	0.076003	-0.92017	0.357484	NA
TSEN34	11.48406	-0.06993	0.084843	-0.82427	0.409786	NA
TLE4	282.4306	0.069933	0.051523	1.357328	0.174677	NA
STRIP2	108.0717	0.069918	0.076896	0.909258	0.363214	NA
KLLN	15.95188	-0.06991	0.07962	-0.87804	0.379922	NA
MYOM2	81.86993	-0.06991	0.074304	-0.94083	0.346793	NA
SCNM1	3.384033	-0.0699	0.077174	-0.90578	0.365051	NA
ZFP106	840.5572	0.0699	0.03129	2.23398	0.025484	0.24988
USP10	196.6363	0.069899	0.046033	1.518464	0.128898	NA
KCNN3	150.7612	-0.0699	0.082333	-0.84894	0.395917	NA
RSC1A1	32.26755	0.069886	0.073371	0.952504	0.340841	NA
SEC24A	125.679	0.069884	0.044444	1.572402	0.115857	NA
LOC28499	10.19099	-0.06988	0.084833	-0.82373	0.410094	NA
ZCCHC4	61.71313	-0.06987	0.062223	-1.12297	0.261452	NA
SH3RF1	40.00319	-0.06986	0.073541	-0.94995	0.342137	NA
GSG1	10.66241	0.069843	0.084153	0.829958	0.406563	NA
ZNF319	36.65523	0.06983	0.07175	0.973247	0.33043	NA
ZNF491	63.93488	-0.06983	0.066514	-1.04982	0.293802	NA
CPSF2	131.5014	0.069817	0.059825	1.167008	0.243207	NA
MIR5586	4.226612	-0.06978	0.081204	-0.85937	0.390137	NA
MIR26A1	3.9056	-0.06977	0.07696	-0.90661	0.364614	NA
C2orf68	152.2136	-0.06976	0.048131	-1.44934	0.147242	NA
PTBP2	161.2896	-0.06975	0.044233	-1.57695	0.114806	NA
RNU12	39.30886	-0.06975	0.076664	-0.90984	0.362906	NA
GRSF1	346.878	0.069745	0.035201	1.981342	0.047553	0.337641

SYCP3	41.54497	-0.06974	0.074889	-0.93128	0.351707	NA
PPP6C	193.2782	0.069715	0.061096	1.141079	0.253837	NA
RRP9	21.07084	0.069693	0.07821	0.891105	0.372873	NA
ZFYVE21	156.0256	-0.06968	0.04927	-1.41428	0.15728	NA
UBE2G2	265.41	-0.06968	0.042011	-1.65864	0.097189	NA
ANK1	1485.851	0.069665	0.049452	1.408733	0.158914	0.515834
AQP4-AS1	3.459384	-0.06966	0.071206	-0.97829	0.327929	NA
ATP13A4	74.41379	-0.06966	0.077774	-0.89564	0.370443	NA
PPP1R12C	279.0243	-0.06965	0.042632	-1.63378	0.102305	NA
FAM86B3P	7.812735	-0.06965	0.079438	-0.87679	0.380599	NA
P2RY12	16.60738	-0.06964	0.079254	-0.87866	0.379587	NA
MIR3132	0.733359	0.069635	0.047136	1.477306	0.139594	NA
RASSF3	26.34411	0.069631	0.084919	0.819966	0.412236	NA
LOC33978	1.382651	0.069622	0.056837	1.224941	0.220598	NA
SNORD51	0.816412	-0.06962	0.051844	-1.34286	0.179317	NA
EFCAB10	10.22345	0.069614	0.084448	0.824337	0.409748	NA
POLRMT	144.6041	0.069601	0.048513	1.434681	0.151378	NA
LRRC8E	1.506017	-0.06958	0.060594	-1.14834	0.25083	NA
MAGI1	922.7954	-0.06958	0.04258	-1.63412	0.102234	0.443332
43168	54.16749	0.069566	0.066442	1.047017	0.295092	NA
EEPD1	43.79047	-0.06955	0.065473	-1.06231	0.288095	NA
GSTM3	163.2994	-0.06955	0.082835	-0.83964	0.401108	NA
ASNSD1	177.5583	0.069536	0.057993	1.199048	0.230509	NA
CPT1B	5.333191	-0.06953	0.08243	-0.84347	0.398966	NA
LOC10065	0.749075	-0.06953	0.047564	-1.46176	0.143806	NA
RRP12	60.51784	0.069519	0.073041	0.951776	0.341211	NA
NRIP1	585.4545	0.069516	0.046072	1.508865	0.131333	0.487416
LATS1	455.0179	0.069514	0.038586	1.801555	0.071615	0.38806
GLA	10.77902	0.069513	0.083371	0.833779	0.404406	NA
PEAR1	9.419427	-0.06951	0.084044	-0.8271	0.408178	NA
SFT2D3	27.77288	0.069511	0.072115	0.963901	0.335095	NA
SYT1	1966.795	0.069503	0.065479	1.061441	0.28849	0.621721
UFSP2	62.03693	0.069501	0.060808	1.14295	0.253059	NA
FRK	3.339711	-0.0695	0.078038	-0.89057	0.37316	NA
LOC44108	1.409053	-0.06949	0.049421	-1.40609	0.159698	NA
LTB4R	16.17695	0.069478	0.081767	0.849709	0.395487	NA
NPAS2	104.1508	0.069477	0.062947	1.10374	0.269706	NA
MIOX	0.692949	-0.06947	0.044387	-1.56507	0.117567	NA
PIANP	118.456	0.069469	0.055973	1.241108	0.214566	NA
C12orf66	17.11491	0.069463	0.080338	0.86464	0.387236	NA
NAA50	246.5404	0.069459	0.047512	1.461935	0.143759	NA
OXR1	647.1632	0.069453	0.061264	1.133678	0.25693	0.598301
THNSL1	38.12739	0.069431	0.07618	0.911412	0.362079	NA
RSPO1	17.12148	-0.06942	0.084426	-0.82224	0.410941	NA
LOC10050	18.5466	-0.06942	0.080587	-0.8614	0.38902	NA
ACBD3	227.2123	0.069414	0.047471	1.462255	0.143671	NA
LEP	3.067059	0.069406	0.071508	0.970605	0.331745	NA

GRAMD4	85.09224	0.069399	0.065134	1.065475	0.286661	NA
ZP1	4.345458	0.069398	0.078783	0.880866	0.37839	NA
LOC10050	3.715049	-0.06939	0.071485	-0.97069	0.331702	NA
LINC00087	34.97434	0.069371	0.082838	0.83743	0.402351	NA
ASTN1	352.4054	0.06937	0.048592	1.427595	0.153408	0.506644
TLX2	2.891551	-0.06935	0.07666	-0.90468	0.365633	NA
GRIK2	1401.75	0.069349	0.044598	1.554969	0.119953	0.472798
LMTK3	324.8517	-0.06935	0.043144	-1.60733	0.107982	NA
SUSD5	26.10928	-0.06932	0.082198	-0.84338	0.399014	NA
CRADD	25.81083	-0.06932	0.074113	-0.93535	0.349608	NA
MIR1253	1.576234	0.069317	0.064152	1.080508	0.279916	NA
CEP120	322.5239	0.069317	0.037658	1.840706	0.065665	NA
TM2D1	52.399	-0.06931	0.071631	-0.96762	0.333233	NA
RABEP1	949.0109	0.069289	0.030236	2.291587	0.021929	0.23963
C17orf100	11.65046	0.069279	0.08445	0.820351	0.412016	NA
CYS1	18.9855	0.069273	0.08323	0.832307	0.405236	NA
TMEM164	52.24053	-0.06927	0.069119	-1.0022	0.316247	NA
DCAF12	68.07829	0.069261	0.05948	1.164443	0.244244	NA
UBE2V1	10.01269	0.069242	0.084119	0.823146	0.410425	NA
ZNF488	27.66769	0.069242	0.083897	0.825319	0.409191	NA
PDE5A	169.2371	-0.0692	0.056337	-1.22834	0.219318	NA
UROD	59.67353	-0.06919	0.064347	-1.07532	0.282234	NA
TNIK	685.0117	-0.06919	0.041149	-1.68151	0.092664	0.41977
SLC39A11	19.59019	-0.06917	0.083317	-0.83021	0.40642	NA
KDM4B	326.3635	-0.06917	0.04896	-1.41271	0.15774	NA
SLC10A4	1.295672	-0.06915	0.055201	-1.25273	0.210304	NA
GFER	57.7245	0.069148	0.061308	1.12787	0.259375	NA
ZNF600	144.8767	0.069144	0.061703	1.120588	0.262463	NA
GCSAML-A	8.088062	-0.06914	0.077404	-0.89324	0.371731	NA
IFNB1	0.629341	-0.06914	0.041252	-1.67592	0.093753	NA
LCN10	1.374809	-0.06913	0.056337	-1.22711	0.219783	NA
CEP76	99.94417	-0.06912	0.058466	-1.18224	0.23711	NA
TESK2	59.03389	-0.06912	0.070495	-0.98049	0.326842	NA
NUP160	228.7591	-0.06911	0.044382	-1.55716	0.119432	NA
IBSP	10.28676	0.069105	0.050955	1.356207	0.175033	NA
WASF3	727.2424	0.06908	0.042934	1.608983	0.10762	0.452973
CCDC79	2.387614	-0.06908	0.072767	-0.94929	0.342473	NA
VANGL1	4.309122	0.069064	0.078336	0.881631	0.377976	NA
WARS	140.4884	0.069057	0.081871	0.843488	0.398956	NA
SMCR5	27.75076	-0.06905	0.078102	-0.88415	0.376616	NA
C6orf70	44.16773	-0.06905	0.072937	-0.94673	0.343775	NA
THSD7A	93.75196	-0.06903	0.065997	-1.04602	0.295552	NA
SNORD116	12.06351	-0.06902	0.084716	-0.81477	0.415206	NA
PPP1R9B	881.9017	0.069022	0.048432	1.425135	0.154118	0.506644
HNRNPH2	17.18078	0.06902	0.083023	0.831329	0.405788	NA
HARBI1	5.466436	0.069013	0.080813	0.853983	0.393115	NA
MDH2	80.62595	0.06901	0.078675	0.877153	0.380403	NA

PTAFR	3.900404	-0.06901	0.079097	-0.87246	0.382956	NA
C16orf62	187.3797	-0.069	0.04372	-1.57819	0.114523	NA
COBLL1	49.52347	0.068995	0.077464	0.89068	0.373101	NA
GATSL3	9.766287	-0.069	0.084286	-0.81859	0.413023	NA
ZC3HC1	24.01234	0.068989	0.073646	0.936753	0.348886	NA
DOT1L	367.0227	0.068987	0.069966	0.985998	0.324134	0.654335
CYP11A1	3.713643	0.068982	0.078689	0.876636	0.380684	NA
LOC25602	19.10673	-0.06897	0.079424	-0.86842	0.385163	NA
URB1	204.4354	0.068973	0.058574	1.177544	0.238979	NA
FAM199X	470.699	0.068971	0.051501	1.339218	0.1805	0.53091
LOC72861	24.43981	0.068963	0.080572	0.855921	0.392042	NA
CASK	273.3191	0.068963	0.039052	1.765944	0.077405	NA
NUP98	448.8938	0.068948	0.038427	1.794261	0.072772	0.389252
CLDN20	19.17241	-0.06894	0.080723	-0.85399	0.393112	NA
FLJ45340	386.123	-0.06893	0.051239	-1.34523	0.17855	0.530729
KDM5C	654.8328	-0.06893	0.0658	-1.04751	0.294864	0.627839
SATB1	382.0322	-0.06892	0.052594	-1.31049	0.190031	0.54182
ATP6V1G1	116.3057	-0.06892	0.060957	-1.13065	0.258202	NA
FBXO25	150.3879	0.068917	0.045991	1.498495	0.134005	NA
LARS	495.5384	0.068908	0.034959	1.971127	0.048709	0.343697
NLE1	26.56237	0.068902	0.076204	0.904184	0.365898	NA
LINC00639	15.64981	-0.0689	0.084846	-0.81206	0.416757	NA
LDLRAD4	625.4612	0.068899	0.061697	1.116733	0.264109	0.606085
LEO1	398.788	0.068894	0.047256	1.457898	0.144869	0.49641
GNB1L	6.862283	0.068892	0.082884	0.831183	0.40587	NA
43345	469.812	-0.06888	0.057381	-1.20047	0.229958	0.578729
NPY1R	8.884035	-0.06888	0.081048	-0.84984	0.395416	NA
TLR8	0.854678	-0.06887	0.051065	-1.34875	0.177417	NA
ALDH3A2	151.3161	-0.06887	0.048073	-1.43268	0.151949	NA
MOCS1	57.29328	-0.06887	0.070097	-0.98245	0.325877	NA
ITIH5	47.81776	0.068848	0.084629	0.813529	0.415915	NA
C11orf92	3.723248	-0.06885	0.079242	-0.86881	0.384951	NA
ADAR	944.1389	0.06884	0.031347	2.196077	0.028086	0.266991
BEND7	62.78992	-0.06884	0.069751	-0.98692	0.323682	NA
SUDS3	258.3856	-0.06883	0.041499	-1.65856	0.097204	NA
IL1R2	2.163112	-0.06883	0.045657	-1.50747	0.131691	NA
SNORA28	2.414149	-0.06882	0.067319	-1.02228	0.306647	NA
E2F1	3.454989	-0.06881	0.078268	-0.87919	0.379298	NA
PAXBP1-A	61.38224	-0.06877	0.074748	-0.92009	0.357528	NA
CYP20A1	126.5033	-0.06877	0.052448	-1.31127	0.189767	NA
SLC35G5	0.626552	-0.06877	0.045351	-1.51641	0.129415	NA
RCN3	13.47801	-0.06877	0.0844	-0.81477	0.415201	NA
ARHGAP44	351.4209	0.06876	0.037418	1.837634	0.066116	0.375222
KIAA1875	69.84812	-0.06876	0.083996	-0.81861	0.413011	NA
CDC37	338.4964	-0.06876	0.045119	-1.52391	0.127532	0.483044
RDH10	10.6611	0.068742	0.084904	0.809648	0.418142	NA
ADPGK	55.85653	-0.06873	0.063772	-1.07773	0.281156	NA

LINC00482	0.676171	-0.06873	0.046776	-1.46928	0.141758	NA
LCN2	0.281748	-0.06872	0.030524	-2.25138	0.024362	NA
ANKRD40	506.1368	0.068714	0.045964	1.494951	0.134927	0.487416
BICD2	319.2099	0.068703	0.045192	1.520239	0.128451	NA
MAP9	191.7433	0.068699	0.063367	1.084157	0.278295	NA
FAM134C	148.0872	-0.06869	0.049984	-1.37427	0.169357	NA
ZNF728	8.120301	-0.06869	0.083076	-0.82684	0.408326	NA
PABPC3	3.084013	0.068687	0.077829	0.882533	0.377489	NA
NT5C	50.23519	-0.06869	0.073214	-0.93815	0.348166	NA
DKFZp779	0.884801	-0.06868	0.047161	-1.45639	0.145285	NA
WDR6	238.4718	0.068684	0.078214	0.878147	0.379864	NA
SLFNL1-AS	21.8854	0.06867	0.0776	0.88492	0.3762	NA
GCSAM	0.547906	0.068666	0.037056	1.853069	0.063872	NA
TGFBR3	33.86101	-0.06866	0.082431	-0.83294	0.404876	NA
ACACA	548.3876	0.068658	0.042708	1.607603	0.107922	0.452973
ERLIN1	72.85484	0.068657	0.062365	1.100895	0.270942	NA
QRICH1	418.3272	-0.06865	0.03735	-1.83797	0.066067	0.375222
MEX3A	22.20457	0.068639	0.084926	0.808228	0.418959	NA
TRPS1	241.6801	-0.06862	0.074355	-0.92281	0.356108	NA
C7orf41	2018.391	-0.06861	0.053258	-1.28821	0.197674	0.545847
GSPT1	370.1902	0.068605	0.036305	1.889666	0.058803	0.36517
MOGAT1	0.7132	-0.0686	0.043818	-1.56565	0.11743	NA
LYZ	3.042046	-0.06859	0.069409	-0.98817	0.32307	NA
RALGAPB	625.817	0.068586	0.032708	2.096903	0.036002	0.292276
ZCCHC3	76.83868	0.068585	0.058132	1.179824	0.23807	NA
LOC72881	0.757793	-0.06858	0.047961	-1.42993	0.152738	NA
PNRC2	111.9356	0.068579	0.059118	1.160027	0.246038	NA
PLA2G4C	141.9472	-0.06857	0.068103	-1.00688	0.313993	NA
SV2A	587.9604	0.068566	0.067021	1.023049	0.306285	0.635871
NOL12	142.3592	-0.06856	0.048529	-1.41282	0.157709	NA
GLI3	19.41511	0.068553	0.084421	0.812038	0.41677	NA
KIAA1522	28.54314	-0.06855	0.079012	-0.86762	0.3856	NA
EIF3K	121.4232	-0.06855	0.066751	-1.02696	0.304439	NA
MIR4750	0.445555	0.068549	0.037949	1.806361	0.070862	NA
AMZ2P1	60.9823	-0.06852	0.065178	-1.05127	0.293133	NA
IGSF6	11.64119	-0.06851	0.082521	-0.83024	0.406403	NA
TEX15	5.75204	-0.0685	0.082037	-0.83505	0.40369	NA
STAT3	266.9927	-0.06848	0.054375	-1.25949	0.207853	NA
ALDH1L2	178.625	-0.06848	0.055042	-1.24413	0.21345	NA
STMN2	1219.609	0.068479	0.071595	0.956473	0.338833	0.66305
B3GNT5	88.37322	-0.06846	0.061728	-1.10911	0.267384	NA
RPS6KL1	132.4103	0.068456	0.069331	0.987384	0.323454	NA
IGF1	8.192873	-0.06843	0.077491	-0.88303	0.377223	NA
GEMIN2	22.70644	0.068424	0.076691	0.892203	0.372284	NA
SNORD71	20.55614	-0.06842	0.07358	-0.92983	0.352457	NA
AFG3L1P	100.201	0.068412	0.070032	0.976875	0.328631	NA
C2orf47	49.95934	0.06841	0.066256	1.032512	0.301832	NA

OSBPL9	318.1639	-0.06841	0.039438	-1.73452	0.082827	NA
KLHDC10	325.3369	0.068402	0.044621	1.532961	0.125285	NA
FGF12	222.1391	0.0684	0.067595	1.011908	0.311582	NA
AKNA	40.06251	-0.06838	0.075962	-0.90016	0.368034	NA
PRMT2	227.4382	0.068361	0.060094	1.137563	0.255303	NA
NOC3L	90.32329	0.068351	0.062163	1.099542	0.271532	NA
MIR25	1.050371	0.068338	0.05579	1.224897	0.220614	NA
TNXB	530.1296	-0.06832	0.062812	-1.08768	0.276737	0.61452
ALG1L9P	14.45681	0.068305	0.083088	0.82208	0.411031	NA
NDUFB6	23.16223	-0.0683	0.078649	-0.86844	0.385154	NA
CEP57	185.7998	-0.06829	0.041312	-1.65308	0.098315	NA
ECSCR	2.786392	-0.06829	0.065462	-1.04317	0.29687	NA
C22orf46	107.8104	0.068271	0.069136	0.987481	0.323407	NA
CSRNP3	317.4218	0.068238	0.049422	1.380726	0.167363	NA
AGGF1	247.2393	0.068237	0.054512	1.251786	0.210648	NA
MIR3675	0.208362	-0.06822	0.027202	-2.50797	0.012143	NA
TNNI3K	1.612561	0.068219	0.062373	1.093738	0.27407	NA
CSAD	103.9867	-0.06822	0.062566	-1.09031	0.275575	NA
C17orf96	3.731069	-0.06822	0.080266	-0.84987	0.395395	NA
TMEFF1	3.81473	-0.06821	0.074355	-0.91737	0.358951	NA
WLS	45.8276	-0.0682	0.081187	-0.84002	0.400897	NA
LOC65022	3.442772	0.068198	0.059429	1.147547	0.251156	NA
ADD3	659.7528	-0.06818	0.055167	-1.23595	0.216479	0.566887
FER	259.6105	0.068177	0.040154	1.697892	0.089528	NA
STUB1	179.9328	0.068171	0.054592	1.248745	0.211758	NA
AJAP1	45.96712	-0.06817	0.075912	-0.89801	0.369178	NA
GTDC1	57.87168	0.068166	0.060369	1.12916	0.25883	NA
SF3A1	419.171	0.068159	0.047581	1.432478	0.152007	0.504833
NS3BP	24.11859	0.068151	0.082444	0.826631	0.408446	NA
TTC31	74.65036	-0.06814	0.063842	-1.06733	0.285823	NA
GABRA2	57.31865	-0.06813	0.081515	-0.83578	0.403279	NA
UBE2D2	186.6139	0.068115	0.046386	1.468424	0.141989	NA
SSPN	110.8273	-0.06811	0.079222	-0.85977	0.389914	NA
MED26	55.62252	0.068109	0.064252	1.060024	0.289134	NA
C3orf49	20.99705	-0.06811	0.076948	-0.88509	0.376108	NA
ERO1L	135.5694	0.068097	0.049063	1.387962	0.165149	NA
SPOCK3	159.1184	-0.06808	0.083986	-0.81064	0.417575	NA
ITPRIPL2	20.44277	-0.06808	0.084097	-0.80956	0.418194	NA
HCAR1	0.784316	-0.06808	0.046794	-1.45483	0.145715	NA
C1orf50	20.4706	-0.06808	0.077851	-0.87445	0.381875	NA
ADAM19	134.3296	-0.06807	0.064469	-1.05586	0.291034	NA
LOC90246	3.125032	-0.06804	0.075261	-0.9041	0.36594	NA
TSC22D1-A	37.49629	0.068042	0.070927	0.959322	0.337396	NA
GUSBP1	130.9693	-0.06804	0.074849	-0.90903	0.363334	NA
LOC34051	0.648864	0.068037	0.04042	1.683245	0.092328	NA
JMY	541.4027	0.068032	0.033936	2.004705	0.044995	0.333048
PLCB4	1225.873	0.068014	0.040174	1.692996	0.090456	0.415466

RRN3	129.6824	0.068	0.056244	1.209007	0.22666	NA
CYP3A5	63.07258	0.067997	0.07292	0.932493	0.351082	NA
COX20	16.29739	-0.06799	0.082364	-0.82552	0.409078	NA
APOL2	173.6976	-0.06799	0.063507	-1.07062	0.284339	NA
AMPH	348.5499	0.06799	0.05389	1.261627	0.207083	0.55642
LOC10050	23.24284	-0.06799	0.081927	-0.82987	0.406611	NA
VAX1	0.326906	-0.06798	0.033932	-2.00332	0.045143	NA
C12orf76	39.54986	-0.06798	0.070349	-0.96626	0.333914	NA
KLHL1	21.32429	-0.06798	0.084066	-0.8086	0.418746	NA
WDR67	181.3236	0.067966	0.049091	1.384487	0.166209	NA
DCT	1.99615	-0.06796	0.065277	-1.04119	0.29779	NA
MAP4K2	126.1975	0.067961	0.060139	1.130064	0.258449	NA
RAD51C	78.19428	0.067944	0.050662	1.341122	0.179881	NA
G3BP2	605.3647	0.067942	0.064402	1.054959	0.291444	0.6257
GABRA1	912.6736	0.067929	0.070128	0.968638	0.332726	0.660832
NAPG	208.3433	0.067906	0.05481	1.238926	0.215373	NA
TPD52L1	29.35824	-0.06789	0.084058	-0.80769	0.41927	NA
TGFBI	6.073303	-0.06789	0.071682	-0.94705	0.343612	NA
EML6	314.0078	-0.06787	0.048638	-1.39545	0.162879	NA
ASPH	909.8832	-0.06787	0.041873	-1.62089	0.105041	0.448055
DNAJC4	36.42264	-0.06787	0.079574	-0.85293	0.393699	NA
ZC2HC1B	0.459883	-0.06787	0.036588	-1.85503	0.063592	NA
FNDC3B	80.19344	-0.06785	0.0685	-0.99053	0.321916	NA
LINC00645	1.052372	-0.06785	0.054841	-1.23717	0.216022	NA
TMEM163	87.28461	0.067848	0.067957	0.998394	0.318088	NA
LOC33875	27.26492	0.067847	0.080452	0.84332	0.399049	NA
OPN4	0.38459	-0.06783	0.036625	-1.852	0.064026	NA
RAB3GAP1	338.2916	0.06782	0.028878	2.34849	0.01885	0.231397
BRF1	251.8908	-0.06782	0.047562	-1.4259	0.153897	NA
KIF16B	205.838	0.067793	0.051025	1.328623	0.183972	NA
GNG13	52.3151	-0.06779	0.084022	-0.80677	0.419802	NA
YIPF1	22.74844	-0.06778	0.076565	-0.88531	0.375989	NA
PDCD1	0.200955	-0.06776	0.027184	-2.49264	0.01268	NA
PTPN1	125.5026	0.067712	0.048099	1.407765	0.159201	NA
PTPRF	186.225	0.067706	0.057804	1.171299	0.241479	NA
MYO5B	48.36941	-0.06769	0.081343	-0.8322	0.405296	NA
UBASH3B	635.9182	0.06769	0.06356	1.064979	0.286885	0.620626
OLR1	9.970635	-0.06769	0.07846	-0.86267	0.388321	NA
MRFAP1L1	469.3024	0.067682	0.048675	1.390489	0.16438	0.52098
FAM24B	2.599464	0.067673	0.073969	0.91488	0.360255	NA
RBX1	40.36182	-0.06767	0.070121	-0.96505	0.334518	NA
SPICE1	219.7721	-0.06767	0.070429	-0.96077	0.336666	NA
PROCR	22.72291	-0.06766	0.084065	-0.80482	0.420922	NA
PIGX	63.13565	-0.06766	0.071211	-0.9501	0.342061	NA
RNF139	97.68938	0.067641	0.052526	1.287764	0.197828	NA
MIR654	0.771605	0.067629	0.047489	1.424103	0.154417	NA
TRHDE-AS	410.9909	-0.06763	0.065763	-1.02838	0.303772	0.63472

ATP6AP1	144.0814	0.06762	0.070611	0.957638	0.338245	NA
LSM14B	230.0302	0.067609	0.046824	1.443888	0.148771	NA
USP13	172.1565	0.067604	0.045009	1.502008	0.133095	NA
ACY3	0.595473	-0.06759	0.038454	-1.75776	0.078788	NA
TSPAN31	55.29416	-0.06758	0.063306	-1.06757	0.285714	NA
CRB1	170.1245	-0.06755	0.048394	-1.39592	0.162737	NA
PARP8	44.31066	-0.06754	0.074844	-0.90247	0.366805	NA
TBR1	0.364355	-0.06754	0.032149	-2.10093	0.035647	NA
CYP4F24P	5.038975	-0.06754	0.059016	-1.14441	0.252454	NA
HOGA1	29.00768	-0.06753	0.081716	-0.82639	0.408581	NA
TXNDC11	114.6402	0.067528	0.052608	1.283593	0.199284	NA
B3GAT1	162.0039	-0.06751	0.069609	-0.96985	0.332121	NA
SOCS2-AS1	1.356634	-0.0675	0.059226	-1.1397	0.254412	NA
SIGLEC15	8.595555	0.067495	0.084653	0.797321	0.425265	NA
BAMBI	7.184597	0.067485	0.083319	0.809955	0.417966	NA
PSG6	11.02959	0.067483	0.082065	0.822316	0.410897	NA
USP47	1175.746	0.067476	0.033944	1.987875	0.046826	0.337641
BCAT1	34.49171	-0.06748	0.082617	-0.81674	0.414079	NA
DLGAP1-A	93.67596	-0.06747	0.06701	-1.0069	0.313983	NA
GALK1	21.57596	-0.06747	0.078284	-0.86185	0.388771	NA
CHCHD1	17.7072	-0.06746	0.078989	-0.85406	0.393074	NA
MED25	112.9373	-0.06746	0.055213	-1.22182	0.221774	NA
BAIAP2L1	20.74922	-0.06745	0.081306	-0.82963	0.406748	NA
FREM2	36.77977	-0.06745	0.08297	-0.81295	0.416245	NA
DOCK1	232.455	-0.06745	0.061011	-1.10549	0.268947	NA
TRAF7	105.4759	-0.06744	0.054453	-1.23858	0.215501	NA
MOSPD2	51.01968	0.067439	0.06106	1.104471	0.269389	NA
RPF1	55.87298	-0.06739	0.063726	-1.05746	0.290302	NA
ROBO1	264.2057	0.067364	0.068817	0.978892	0.327633	NA
43354	521.0979	0.067363	0.038299	1.75885	0.078603	0.397742
FGFR2	382.643	-0.06735	0.077532	-0.8687	0.385009	0.70023
MINPP1	58.35451	0.067339	0.06799	0.990422	0.321968	NA
ERH	98.31317	-0.06733	0.077527	-0.86853	0.385104	NA
RPRD1B	80.07164	-0.06733	0.060733	-1.10862	0.267594	NA
C10orf12	197.945	0.067326	0.063476	1.060653	0.288847	NA
PLEKHM2	436.8142	-0.06733	0.040747	-1.65227	0.098479	0.434832
MPHOSPH	789.7711	-0.06732	0.064182	-1.04893	0.294208	0.627478
FOXO4	132.1641	-0.06732	0.07039	-0.95637	0.338886	NA
C1QTNF6	2.191488	0.067314	0.068683	0.980073	0.32705	NA
RSAD2	5.807928	0.067307	0.068478	0.982897	0.325658	NA
LTA4H	169.8393	-0.06731	0.050113	-1.34308	0.179246	NA
POLA2	66.80605	-0.06731	0.063297	-1.06333	0.287634	NA
TIPIN	41.09193	-0.0673	0.070587	-0.95349	0.340341	NA
LOC44089	1.368488	0.067278	0.058264	1.154717	0.248206	NA
PKNOX1	216.1425	0.067267	0.056247	1.195933	0.231723	NA
SLC4A8	645.225	0.067265	0.067146	1.001766	0.316456	0.645996
GNB4	99.7988	-0.06726	0.057903	-1.16159	0.245401	NA

PID1	19.87684	0.067247	0.084934	0.791762	0.428499	NA
PPP1R26	46.2954	-0.06725	0.071982	-0.93419	0.350205	NA
PRR13	29.82692	-0.06725	0.076326	-0.88103	0.378304	NA
DHRS4L1	8.686084	-0.06724	0.0849	-0.79205	0.428333	NA
LINC00578	0.227559	-0.06724	0.029139	-2.30765	0.021018	NA
SCARF1	23.81739	-0.06724	0.078343	-0.85825	0.390756	NA
ASPHD2	103.7347	0.067224	0.052503	1.28037	0.200415	NA
PPP1R12A	715.5559	0.067212	0.031846	2.110526	0.034813	0.288416
PPIL4	363.5792	0.067212	0.051633	1.301726	0.19301	0.542492
RALGDS	578.3593	-0.06721	0.055652	-1.20768	0.22717	0.577485
BRWD3	695.3286	0.06721	0.040678	1.652242	0.098485	0.434832
ACOT6	32.87198	0.067206	0.078599	0.855041	0.392529	NA
ACSF3	91.06863	-0.0672	0.058043	-1.15777	0.246959	NA
PLAC8L1	3.214368	0.0672	0.073455	0.914848	0.360272	NA
RGS5	222.4383	0.067194	0.082639	0.813104	0.416158	NA
LRRC37A4	296.3608	0.067194	0.071001	0.946376	0.343957	NA
MAP1A	4244.565	0.067163	0.05741	1.169892	0.242044	0.590833
NEDD4	21.11418	0.067147	0.078864	0.851431	0.39453	NA
CIZ1	639.3658	0.067135	0.034734	1.932814	0.053259	0.357127
FBXO17	87.23001	-0.06712	0.053998	-1.24302	0.213862	NA
LMNA	128.7014	-0.0671	0.06324	-1.06109	0.288651	NA
CHST14	6.291158	-0.06708	0.083916	-0.79943	0.424042	NA
GRAMD2	4.609999	-0.06708	0.07712	-0.86976	0.384433	NA
LOC10050	15.39028	0.067067	0.082633	0.811628	0.417005	NA
TP53I11	358.1257	0.067067	0.065021	1.03146	0.302325	0.633092
FLYWCH1	194.3305	-0.06706	0.065546	-1.02305	0.306285	NA
SYT5	15.6722	-0.06705	0.084891	-0.78981	0.429638	NA
PDCD2	102.4043	0.067026	0.06045	1.108779	0.267526	NA
PCMT1	217.761	0.067023	0.066187	1.01263	0.311237	NA
CD27	1.002339	-0.06702	0.052937	-1.26603	0.205504	NA
HSPA4L	576.255	0.067005	0.066976	1.000437	0.317099	0.646471
NIPSNAP1	132.4528	-0.067	0.057274	-1.16982	0.242072	NA
TRIM50	0.258953	0.066996	0.030561	2.192235	0.028363	NA
SIDT2	96.32038	-0.06696	0.066148	-1.0123	0.311396	NA
MIR3162	1.357684	-0.06694	0.061346	-1.09122	0.275176	NA
FAHD2CP	11.12005	-0.06694	0.084475	-0.79244	0.428102	NA
CIB4	1.877063	-0.06694	0.063818	-1.04888	0.294233	NA
CRY1	208.8179	0.066924	0.06736	0.993532	0.320451	NA
TET2	197.7554	0.066921	0.066856	1.000973	0.31684	NA
WBP1	3.52243	-0.06692	0.077104	-0.8679	0.385447	NA
CEP97	234.6561	0.066912	0.04565	1.465753	0.142716	NA
FEZ1	588.8263	0.066907	0.056326	1.187864	0.234887	0.585924
COPS4	93.82392	0.066899	0.064106	1.04356	0.296689	NA
TCERG1L	19.81733	-0.06689	0.084938	-0.78752	0.43098	NA
ZNF417	110.7217	0.066889	0.061558	1.086609	0.27721	NA
LOC10050	1.347691	-0.06688	0.058597	-1.14134	0.253729	NA
BSPH1	0.743083	0.066878	0.049139	1.361006	0.173512	NA

RTN1	911.3094	0.066838	0.05838	1.14488	0.252259	0.592704
TMCO4	4.389386	-0.06682	0.076138	-0.87764	0.380139	NA
GBX1	0.79064	-0.06682	0.046965	-1.42276	0.154805	NA
DCDC5	2.464088	-0.06681	0.072119	-0.9264	0.354239	NA
FAM228B	94.9574	-0.06681	0.075954	-0.87962	0.379064	NA
DHX29	244.5681	0.066809	0.037365	1.788035	0.07377	NA
SNORA50	5.14765	0.066808	0.081279	0.821959	0.4111	NA
C18orf54	28.4436	-0.06681	0.078999	-0.84565	0.39775	NA
MGAT5B	55.28618	0.066803	0.070243	0.951018	0.341595	NA
MIR1343	0.945492	-0.06679	0.051651	-1.29319	0.195944	NA
RAB32	3.05182	-0.06679	0.074077	-0.90167	0.367232	NA
PUF60	195.8454	-0.06679	0.040594	-1.64534	0.099899	NA
PMS2	113.2686	-0.06676	0.064056	-1.04214	0.297347	NA
NEK8	5.32532	0.066752	0.079107	0.843811	0.398775	NA
SLC17A5	59.21886	-0.06675	0.054236	-1.23074	0.21842	NA
RPS6KA2-I	6.736085	-0.06674	0.082304	-0.81091	0.417417	NA
CDR2L	295.1612	0.066733	0.055428	1.203956	0.228607	NA
AVPR2	53.89079	-0.06672	0.084554	-0.78911	0.43005	NA
SLC35B1	29.34478	0.066718	0.073285	0.91038	0.362622	NA
MPDU1	19.87279	0.066711	0.08096	0.824	0.40994	NA
PI4KA	982.7145	0.066698	0.043151	1.545691	0.122179	0.476542
TMSB15B	8.154756	-0.06669	0.08442	-0.79	0.42953	NA
SMIM8	52.73901	-0.06666	0.068983	-0.96634	0.333874	NA
NPHP1	15.34636	0.066638	0.082899	0.803838	0.42149	NA
ZBTB24	98.92341	0.066598	0.056047	1.188269	0.234727	NA
LOC40107	7.637299	0.066598	0.084247	0.790511	0.429229	NA
PRSS36	0.879463	0.066591	0.04734	1.40666	0.159528	NA
TMCO3	235.7178	0.066588	0.046304	1.438076	0.150412	NA
ADAMTS1	0.840775	-0.06659	0.048196	-1.3816	0.167094	NA
CREB3L3	1.501801	0.066578	0.055339	1.203097	0.228939	NA
ROS1	2.44911	0.066561	0.058455	1.138685	0.254835	NA
EIF4E	148.5809	0.066559	0.057976	1.148045	0.25095	NA
C15orf61	14.85798	0.066536	0.081962	0.811796	0.416909	NA
RASAL2	161.7722	0.066533	0.053997	1.232165	0.217887	NA
MIR3157	0.655624	-0.06652	0.044653	-1.48974	0.136292	NA
OSGIN1	2.03876	-0.06652	0.06518	-1.02053	0.307479	NA
KCNK13	0.846739	-0.06651	0.049959	-1.33135	0.183073	NA
PLEKHG4B	12.90577	-0.0665	0.077357	-0.85966	0.389979	NA
SPAG17	4.587865	-0.0665	0.081064	-0.82032	0.412034	NA
LOC10012	12.63382	-0.0665	0.084779	-0.78435	0.432837	NA
CNDP2	149.312	-0.06649	0.058884	-1.12921	0.258808	NA
CTGF	21.42766	0.066489	0.073653	0.90273	0.366669	NA
MPPED1	1.614118	0.06648	0.059798	1.111735	0.266252	NA
MED13	825.7597	0.066475	0.034398	1.932538	0.053293	0.357127
LOC96610	117.1368	-0.06645	0.061617	-1.07849	0.280816	NA
FGF14-IT1	472.8952	-0.06645	0.071192	-0.93344	0.350595	0.674334
SH3BP2	100.7203	-0.06645	0.074128	-0.89644	0.370019	NA

JOSD1	138.3564	0.066449	0.045989	1.444889	0.148489	NA
MIR5688	5.496666	-0.06644	0.081523	-0.81503	0.415054	NA
HERC2P7	6.239592	-0.06644	0.080217	-0.82828	0.40751	NA
TRIM68	45.59059	-0.06644	0.063277	-1.04998	0.293727	NA
LAMTOR5	31.85021	-0.06643	0.076968	-0.86314	0.388059	NA
ARAP1	277.1937	-0.06643	0.066671	-0.99645	0.319033	NA
LEPR	44.71837	-0.06642	0.08326	-0.7978	0.424987	NA
ESAM	15.14451	-0.06639	0.084777	-0.78308	0.43358	NA
HIST4H4	9.970572	0.066373	0.084939	0.781419	0.434556	NA
PCDH7	40.06595	-0.06636	0.082707	-0.80237	0.422341	NA
TMOD3	117.9017	0.06636	0.062593	1.060176	0.289064	NA
APBB3	90.88525	0.066312	0.060968	1.087663	0.276744	NA
BTBD11	48.82804	-0.06631	0.084533	-0.78444	0.432784	NA
RBM23	315.4197	-0.06631	0.057389	-1.15546	0.2479	NA
SUV39H1	31.53797	0.06631	0.071518	0.927184	0.353831	NA
MAST4	150.7892	-0.0663	0.068471	-0.9683	0.332894	NA
FLJ26850	1.720722	0.066299	0.065201	1.016846	0.309227	NA
GRPEL2	123.0114	0.066295	0.048394	1.369891	0.170721	NA
MIR3609	382.2901	0.066293	0.077292	0.857697	0.39106	0.702977
PMFBP1	36.54081	-0.06629	0.084594	-0.78356	0.433297	NA
HRCT1	1.557623	-0.06627	0.064515	-1.02726	0.3043	NA
FAM160A1	154.4004	-0.06627	0.084366	-0.78553	0.432146	NA
TBC1D9B	638.6995	-0.06627	0.048551	-1.36493	0.172276	0.525733
TSTD3	70.72166	0.066262	0.07015	0.944583	0.344872	NA
NUDT13	60.54634	-0.06626	0.068755	-0.96371	0.335191	NA
CWF19L1	74.34987	-0.06624	0.062413	-1.06139	0.288511	NA
GXYLT1	167.8519	0.066235	0.049345	1.3423	0.179499	NA
GMPPA	31.26216	-0.06622	0.07326	-0.9039	0.366047	NA
TJP2	472.6999	-0.06621	0.074643	-0.88704	0.375059	0.695376
G6PD	77.24287	0.0662	0.066885	0.989759	0.322292	NA
DSCR3	82.55927	0.066193	0.053704	1.232558	0.217741	NA
ABCG8	1.438657	0.066186	0.061283	1.080004	0.28014	NA
ZC3H12B	112.6109	0.066186	0.053508	1.236941	0.216109	NA
RAPGEF3	70.76791	-0.06618	0.080215	-0.82502	0.40936	NA
LOC43999	1.55799	-0.06617	0.058442	-1.13222	0.257543	NA
TOR3A	26.83739	0.066156	0.075141	0.880417	0.378633	NA
PLEKHO2	66.77602	-0.06615	0.077006	-0.85904	0.390316	NA
TEF	405.7185	0.066134	0.048591	1.361027	0.173505	0.526085
ATP5D	134.4347	-0.06613	0.07092	-0.93244	0.35111	NA
ZNF510	298.9759	0.066117	0.040112	1.64832	0.099287	NA
NAIP	23.45916	-0.06611	0.084884	-0.77886	0.436062	NA
GID4	89.21079	0.066107	0.057185	1.156025	0.247671	NA
MIR4774	2.42273	0.066094	0.071519	0.924152	0.355407	NA
BGN	25.78528	0.066093	0.084746	0.779895	0.435453	NA
ZFP92	17.94623	-0.06609	0.081441	-0.81145	0.417105	NA
KLHL12	94.12145	0.06608	0.059851	1.104088	0.269555	NA
FLCN	522.4196	0.066073	0.079512	0.83099	0.405979	0.710565

MIR623	0.750099	0.066068	0.045937	1.438219	0.150372	NA
CD33	1.439007	-0.06606	0.060724	-1.08782	0.276674	NA
LOC28368	27.52307	-0.06605	0.079223	-0.83375	0.404422	NA
MMGT1	89.21364	0.066051	0.063521	1.039814	0.298426	NA
PDK3	206.2648	-0.06605	0.045279	-1.45873	0.144639	NA
MRPS14	171.1398	-0.06604	0.044136	-1.49631	0.134572	NA
LOC10013	6.424111	0.066039	0.083029	0.795374	0.426396	NA
MATK	7.50922	0.066032	0.084811	0.778579	0.436228	NA
ACTL10	2.838272	-0.06602	0.076176	-0.86664	0.386137	NA
OSBPL1A	134.6071	-0.06601	0.068419	-0.96486	0.334616	NA
AP3B1	167.5907	-0.06601	0.04982	-1.32495	0.185187	NA
FAM212B	65.33205	-0.06601	0.070415	-0.9374	0.348554	NA
EIF2B1	140.1834	-0.066	0.044515	-1.48268	0.138159	NA
SLC15A3	5.658042	-0.06599	0.081458	-0.81014	0.417859	NA
TMEM115	76.10802	-0.06598	0.058736	-1.1234	0.261267	NA
DUSP19	18.48161	0.065962	0.082561	0.798956	0.424316	NA
SNORD31	5.949815	-0.06596	0.080118	-0.82328	0.410351	NA
RWDD2A	40.76535	-0.06595	0.066204	-0.99616	0.319171	NA
SRSF1	1179.895	-0.06594	0.038007	-1.73504	0.082734	0.403119
TRPC4AP	232.4544	-0.06594	0.045789	-1.44007	0.149848	NA
LINC00847	28.76933	-0.06593	0.074359	-0.88662	0.375285	NA
NDUFAF3	30.65665	-0.06592	0.074286	-0.88744	0.37484	NA
KYNU	1.349847	-0.06592	0.058831	-1.12048	0.262511	NA
HLA-A	166.605	0.065906	0.072953	0.903402	0.366312	NA
GTPBP3	26.68984	-0.0659	0.083677	-0.7876	0.430931	NA
KLHL31	0.967402	-0.06589	0.054074	-1.21858	0.223005	NA
KCNJ9	487.1466	0.06589	0.041368	1.59277	0.111212	0.460575
HPD	0.863224	0.065888	0.049794	1.323196	0.18577	NA
PEF1	58.69759	0.06588	0.068132	0.966951	0.333568	NA
OR6W1P	1.234209	0.065879	0.057494	1.145843	0.25186	NA
COL15A1	4.675129	0.065873	0.079982	0.823594	0.41017	NA
AQP7P3	0.360541	-0.06587	0.0342	-1.92602	0.054102	NA
ZBTB47	178.7086	0.065868	0.065885	0.999742	0.317435	NA
GIPC3	2.265329	-0.06586	0.067383	-0.97741	0.328367	NA
PEAK1	654.4107	0.065843	0.052461	1.25507	0.209453	0.558789
AQP5	2.147437	0.065835	0.066519	0.989722	0.32231	NA
ATP13A3	349.3051	0.065833	0.050669	1.299279	0.193848	0.543399
MIR107	2.844443	0.06583	0.069118	0.952433	0.340877	NA
FLJ40852	2.829682	-0.06583	0.073685	-0.89335	0.371671	NA
ERP44	390.7166	0.065821	0.039629	1.660929	0.096728	0.430429
QPRT	6.554221	-0.06582	0.082098	-0.80171	0.422719	NA
LTV1	102.1221	0.06581	0.055447	1.186889	0.235271	NA
CLASRP	223.5053	-0.0658	0.061146	-1.07619	0.281843	NA
BACH2	134.6896	-0.06579	0.063858	-1.03032	0.302858	NA
TXNDC12	52.46435	-0.06578	0.066913	-0.98311	0.325552	NA
SLC22A31	108.805	-0.06577	0.067524	-0.97396	0.330078	NA
OR7E12P	0.698282	-0.06576	0.044719	-1.47062	0.141395	NA

TMUB2	55.16507	-0.06575	0.066864	-0.9834	0.325409	NA
FAM208A	654.6123	0.065748	0.031633	2.078484	0.037665	0.299336
MAGI2-AS	3.441891	-0.06575	0.072704	-0.90431	0.365832	NA
LEPREL4	35.36341	0.065746	0.06891	0.954081	0.340043	NA
MIR194-1	0.5652	0.065741	0.042316	1.553592	0.120282	NA
ZCCHC12	93.86765	0.065741	0.081123	0.810381	0.417721	NA
INO80C	16.75385	-0.06573	0.081193	-0.80954	0.418204	NA
SOGA3	528.3486	0.065715	0.047459	1.384662	0.166156	0.52125
SNORD114	1.903065	-0.06571	0.065652	-1.00093	0.316859	NA
ADPRHL1	7.531473	0.065707	0.084325	0.779209	0.435857	NA
GPR173	106.3026	0.065688	0.052783	1.244493	0.213318	NA
CD163L1	3.43313	-0.06568	0.072869	-0.90137	0.367392	NA
POC1B	71.20552	-0.06568	0.060317	-1.08888	0.276207	NA
PPM1G	169.8804	0.065673	0.05124	1.28167	0.199958	NA
HNRNPL	363.0252	0.065669	0.047824	1.373127	0.169713	0.522992
SLC22A3	9.718616	-0.06566	0.084602	-0.77616	0.437654	NA
AIRN	6.58829	-0.06566	0.084344	-0.77845	0.436306	NA
EGFR	118.0152	-0.06566	0.082051	-0.80018	0.423607	NA
PTGER2	1.396831	-0.06565	0.057807	-1.13568	0.25609	NA
FERMT2	146.6157	-0.06563	0.05952	-1.10274	0.27014	NA
CDH22	342.9437	0.065633	0.047957	1.368564	0.171135	0.525233
AMIGO3	65.87534	-0.06563	0.066257	-0.99058	0.321893	NA
RNF103	496.405	0.065633	0.044065	1.489442	0.136371	0.489381
PLCG2	11.46458	-0.06563	0.084937	-0.77267	0.439717	NA
LINC00626	2.723856	-0.06562	0.067592	-0.97085	0.331625	NA
ASXL2	444.2112	0.065618	0.041173	1.593713	0.111	0.460575
NOP14-AS	65.87283	0.065615	0.064283	1.020719	0.307388	NA
C12orf65	71.38792	0.065608	0.066374	0.98847	0.322923	NA
MIR3922	0.309191	0.065601	0.032872	1.995643	0.045973	NA
SLC38A9	62.45485	-0.06559	0.057376	-1.14308	0.253006	NA
KRBA2	76.50194	-0.06559	0.069439	-0.9445	0.344913	NA
SNX11	17.62978	-0.06558	0.080432	-0.81537	0.414863	NA
KIAA1244	2043.487	0.065578	0.051472	1.274064	0.202641	0.549679
MED12	278.8768	-0.06555	0.055649	-1.17798	0.238804	NA
RCL1	61.81535	-0.06555	0.065957	-0.99385	0.320296	NA
ZCCHC7	231.5716	-0.06555	0.054995	-1.19186	0.233316	NA
LOC28457	16.11566	-0.06553	0.083637	-0.78345	0.433362	NA
SAMD12-A	4.348695	0.065518	0.079644	0.82263	0.410718	NA
IMP3	27.52505	-0.06551	0.076666	-0.85451	0.392822	NA
WIZ	89.8014	0.065507	0.05203	1.259034	0.208018	NA
KRR1	151.2984	0.065507	0.04391	1.491828	0.135744	NA
FTSJ1	55.20504	0.065506	0.065385	1.001852	0.316415	NA
SLC19A2	54.4864	-0.0655	0.064926	-1.00889	0.313028	NA
THUMPD2	135.8966	0.065501	0.049998	1.310062	0.190175	NA
LOC10013	0.418464	-0.06549	0.036391	-1.79976	0.071899	NA
C19orf35	5.007859	-0.06549	0.079304	-0.82586	0.408884	NA
MCC	179.0328	-0.06549	0.068958	-0.94966	0.342287	NA

ATAD3B	48.66305	-0.06549	0.07104	-0.9218	0.356632	NA
PPEF2	9.223842	-0.06548	0.08131	-0.80532	0.420638	NA
ADAMTS1	87.1048	-0.06547	0.084866	-0.77144	0.440448	NA
NFKBIA	149.6183	-0.06546	0.080215	-0.81603	0.414483	NA
POU5F1	6.506166	-0.06545	0.080223	-0.81582	0.414603	NA
NAT8L	259.1155	0.065444	0.057875	1.130779	0.258148	NA
TLR3	2.762107	-0.06544	0.073863	-0.88596	0.375638	NA
RNF165	78.96756	-0.06544	0.072515	-0.9024	0.366842	NA
KCNC4	117.4899	0.06542	0.062178	1.052141	0.292735	NA
SCRN3	87.63652	0.065414	0.066726	0.980346	0.326915	NA
RIMS3	332.6795	0.065414	0.068692	0.952285	0.340952	NA
CDKN2D	34.3472	0.065411	0.078445	0.833855	0.404363	NA
KCNN1	22.86108	-0.06541	0.081581	-0.80178	0.422679	NA
UBE2Q1	211.9794	0.06541	0.05284	1.237887	0.215758	NA
TSPAN12	14.28139	0.065399	0.084698	0.772146	0.440028	NA
LOC10050	11.1987	-0.06538	0.084717	-0.77178	0.440245	NA
EMG1	58.45024	-0.06538	0.063927	-1.02273	0.306436	NA
TSGA13	0.539924	0.065377	0.043605	1.499297	0.133797	NA
SLC9A7	374.6394	0.065359	0.052529	1.244249	0.213408	0.562782
MIR190A	0.880255	0.065359	0.052826	1.237249	0.215995	NA
CACNA2D1	214.7596	0.065358	0.05899	1.107962	0.267878	NA
AZIN1	344.7228	0.065357	0.066632	0.980869	0.326657	0.655921
RNF180	112.4988	0.065348	0.065964	0.990669	0.321847	NA
SKINTL	1.540817	-0.06535	0.062078	-1.05263	0.292509	NA
NBLA0030	4.817833	0.065337	0.055983	1.167097	0.243171	NA
GYS2	7.411194	0.065328	0.080657	0.809946	0.417971	NA
CILP	5.20136	0.065326	0.080061	0.815957	0.414525	NA
ILF3-AS1	38.73234	-0.0653	0.071809	-0.90936	0.363158	NA
SLC22A4	52.78787	0.0653	0.070093	0.931619	0.351533	NA
TBC1D5	272.1911	0.065296	0.037761	1.729168	0.083779	NA
SCRIB	185.9612	0.065291	0.0651	1.002937	0.315891	NA
PCBP1	193.9202	0.065277	0.065327	0.999226	0.317685	NA
CIB2	17.13316	0.065266	0.081306	0.802719	0.422137	NA
MIR645	1.994807	-0.06526	0.069069	-0.94488	0.344721	NA
BPNT1	29.35415	-0.06526	0.070299	-0.92829	0.353258	NA
SLC30A7	120.8206	-0.06526	0.05214	-1.25154	0.210737	NA
ARVCF	358.17	0.065245	0.053013	1.230734	0.218422	0.569521
THYN1	127.7952	-0.06521	0.049986	-1.30461	0.192027	NA
SP4	225.1119	0.065212	0.048672	1.339827	0.180302	NA
HBB	41.3071	-0.0652	0.066439	-0.98136	0.326415	NA
SRR	17.68213	-0.06519	0.083549	-0.78025	0.435243	NA
LHCGR	5.565956	0.065166	0.062576	1.041404	0.297688	NA
SRSF12	139.7038	0.065148	0.068205	0.955174	0.33949	NA
ARMCX4	312.1491	-0.06514	0.059052	-1.10307	0.269998	NA
CTTNBP2	260.7065	-0.06513	0.068192	-0.95517	0.339491	NA
CADM3	676.5823	0.065128	0.04999	1.302819	0.192636	0.542492
CDC25B	424.5477	0.065126	0.059591	1.092874	0.274449	0.613511

LOC64333	0.463383	-0.06512	0.039598	-1.64457	0.100058	NA
ADAMTS1	2.019481	-0.06512	0.057462	-1.13325	0.257111	NA
RBM10	292.8474	0.065118	0.047347	1.375326	0.169031	NA
INTS4L2	41.78926	0.065108	0.078892	0.825271	0.409218	NA
LOC40045	1.945721	0.065103	0.067802	0.960188	0.336961	NA
PPM1D	194.356	-0.0651	0.056393	-1.15432	0.248368	NA
RELN	3193.018	0.065087	0.068072	0.956162	0.338991	0.66305
APH1B	88.13383	-0.06507	0.070934	-0.91733	0.358969	NA
TRIM37	865.9018	0.065061	0.047663	1.365032	0.172243	0.525733
MIR4768	0.664112	-0.06506	0.0462	-1.40815	0.159088	NA
MFAP3	150.8909	0.065048	0.04767	1.36454	0.172398	NA
DHFRL1	43.32305	0.065043	0.077217	0.842332	0.399602	NA
ZNF607	122.1382	0.065032	0.050817	1.279728	0.200641	NA
WBP11P1	2.814745	0.065029	0.075909	0.856672	0.391626	NA
LBX2-AS1	3.631072	-0.06502	0.068595	-0.94788	0.343192	NA
CTPS1	62.49646	0.065018	0.065044	0.999597	0.317505	NA
PBDC1	42.47657	0.065018	0.070534	0.921793	0.356637	NA
FCHO1	118.4249	-0.06502	0.061241	-1.06164	0.288398	NA
ADSL	92.49746	-0.06502	0.054888	-1.18451	0.23621	NA
RDH14	10.71624	-0.065	0.084252	-0.77151	0.440404	NA
FBXO8	54.31723	-0.06497	0.058152	-1.11727	0.263878	NA
FCN3	0.582223	-0.06496	0.042494	-1.52861	0.126362	NA
IPO4	57.27003	0.064957	0.070561	0.920574	0.357273	NA
SLC25A38	82.45997	-0.06495	0.063944	-1.01574	0.309751	NA
MRPS6	59.84802	0.064942	0.084753	0.766249	0.443528	NA
GNB5	272.6907	0.064927	0.043274	1.500347	0.133525	NA
NPTN	451.8919	0.064925	0.064954	0.999562	0.317523	0.646753
CARS	74.36866	-0.06493	0.064257	-1.01039	0.312307	NA
PTGES3	347.0149	0.06492	0.063946	1.015237	0.309993	0.640085
C19orf77	0.557692	-0.06491	0.041028	-1.58212	0.113621	NA
SMPD4	121.1891	0.064908	0.051797	1.253125	0.21016	NA
ELAVL4	83.72626	-0.06491	0.070811	-0.91661	0.359347	NA
C14orf182	6.899025	0.064893	0.074932	0.866021	0.386479	NA
ATG16L1	219.0457	0.064892	0.042074	1.542318	0.122996	NA
LOC64392	8.735328	-0.06488	0.084868	-0.7645	0.444567	NA
ECT2L	24.98604	-0.06488	0.076848	-0.84422	0.398545	NA
CDH4	19.21229	0.064876	0.082474	0.78662	0.431505	NA
IMPACT	42.43802	-0.06487	0.07714	-0.84091	0.400398	NA
DACT1	191.4873	0.064848	0.062422	1.038867	0.298867	NA
ZRANB1	586.7553	0.064836	0.044934	1.442906	0.149047	0.502511
TG	1.615407	0.064836	0.061996	1.045804	0.295652	NA
PATE3	3.524748	-0.06483	0.075282	-0.86119	0.389136	NA
KCNMB2	8.205806	-0.06483	0.082598	-0.7849	0.432513	NA
NINJ1	39.63053	-0.06483	0.078208	-0.82895	0.407135	NA
CD302	1.113404	-0.06483	0.05511	-1.17631	0.23947	NA
ATP8B2	383.8466	0.064817	0.053836	1.203985	0.228595	0.577536
DNAJB14	156.1016	0.064815	0.051704	1.253561	0.210002	NA

MLL2	1684.434	0.064814	0.046667	1.388853	0.164877	0.52098
LOC38990	13.55491	-0.06481	0.08488	-0.76353	0.44515	NA
LOC10013	74.35209	0.064792	0.065085	0.995503	0.319492	NA
TOR2A	24.37682	-0.06479	0.07312	-0.88603	0.375599	NA
SNORA18	34.5913	-0.06478	0.076076	-0.85148	0.3945	NA
SNORD116	22.00367	-0.06477	0.080963	-0.79995	0.423738	NA
ADAM20	66.39808	0.064766	0.073679	0.879028	0.379386	NA
PSMD13	123.5004	-0.06476	0.053491	-1.21066	0.226025	NA
C2orf40	6.959272	-0.06476	0.077982	-0.8304	0.406314	NA
KRBA1	82.30199	0.06471	0.066733	0.969678	0.332207	NA
C20orf112	355.6812	0.064707	0.050287	1.28675	0.198181	0.545847
ZNF460	449.5416	0.064699	0.046918	1.378971	0.167904	0.521454
SNX12	72.4247	0.064691	0.064292	1.006211	0.314314	NA
GNAT2	9.609753	0.064679	0.084716	0.763478	0.445178	NA
SERPINE2	156.2893	-0.06467	0.081112	-0.79734	0.425255	NA
ALKBH4	31.1294	0.064673	0.076256	0.848096	0.396385	NA
ZCCHC14	638.0155	0.064667	0.049981	1.293841	0.19572	0.545273
ZNF264	311.8839	0.064663	0.048589	1.330812	0.183251	NA
SAT2	83.84364	-0.06466	0.067689	-0.95524	0.339457	NA
C9orf16	15.00007	0.064657	0.084935	0.761249	0.446508	NA
FLJ31485	11.5218	-0.06465	0.084926	-0.76129	0.446484	NA
HPCAL4	625.8356	0.064631	0.067306	0.96025	0.336929	0.66305
KCNQ4	26.56421	0.064617	0.076812	0.841231	0.400218	NA
ALG13	205.9792	-0.06462	0.050295	-1.28473	0.198886	NA
GPR12	213.4753	0.06459	0.052289	1.235249	0.216738	NA
LOC64303	0.780786	-0.06459	0.047906	-1.34821	0.177591	NA
FAM47E	5.30691	-0.06459	0.083596	-0.7726	0.439761	NA
SNORD101	5.940413	-0.06458	0.083975	-0.76907	0.441851	NA
ZNF843	10.81551	-0.06458	0.084436	-0.76484	0.444366	NA
C3orf20	4.597346	-0.06458	0.078724	-0.82031	0.412041	NA
LINC00624	0.325426	-0.06457	0.034243	-1.88556	0.059354	NA
RMI2	0.548336	-0.06456	0.038797	-1.66403	0.096107	NA
LOC15837	5.775458	-0.06456	0.079231	-0.81479	0.41519	NA
LOC10050	0.56489	-0.06455	0.042983	-1.50185	0.133135	NA
APBA1	542.9509	0.064551	0.05126	1.259281	0.207929	0.55642
USP32	379.27	0.064548	0.040228	1.604547	0.108594	0.452973
OR1D5	0.907016	0.06452	0.050536	1.276719	0.201701	NA
ARPC2	145.7297	-0.06452	0.061342	-1.05179	0.292897	NA
BAD	40.91355	-0.0645	0.06742	-0.95666	0.338737	NA
LOC40132	0.396709	0.064489	0.036929	1.746282	0.080762	NA
EP400	1047.774	0.064485	0.044379	1.453052	0.146209	0.498741
RNF182	91.75449	0.064481	0.066097	0.975561	0.329282	NA
STAU2	336.6446	0.06448	0.037975	1.697974	0.089513	0.415114
AFAP1	113.9127	0.064473	0.075699	0.85171	0.394375	NA
MIR128-2	5.703493	-0.06447	0.083627	-0.77095	0.440739	NA
PCDHA11	110.803	0.064465	0.064976	0.992131	0.321134	NA
VNN2	1.184951	-0.06446	0.05251	-1.22765	0.219578	NA

ZZZ3	346.3974	0.064461	0.034639	1.860944	0.062752	0.37053
EIF1AD	63.29415	0.064452	0.06244	1.032225	0.301967	NA
KCTD6	183.0921	0.064437	0.064516	0.998778	0.317902	NA
HSPB2	22.30816	-0.06443	0.080444	-0.80096	0.423152	NA
LINGO2	37.23694	0.06443	0.049511	1.301322	0.193148	NA
NOP9	40.53179	-0.06442	0.066456	-0.96942	0.332335	NA
ZNF592	277.9417	0.064416	0.045198	1.425196	0.1541	NA
BRD9	402.752	-0.06442	0.059509	-1.08245	0.279053	0.615438
M1AP	1.743255	-0.06441	0.06078	-1.05971	0.289279	NA
PCGF1	24.5696	0.064402	0.072091	0.893338	0.371676	NA
TP53TG1	6.601333	-0.0644	0.082324	-0.78228	0.434052	NA
TAS2R60	1.001899	-0.06439	0.047624	-1.35213	0.176334	NA
MIR23B	4.601221	-0.06439	0.081503	-0.79007	0.429486	NA
ARMCX3	261.2914	0.064393	0.047691	1.350197	0.176953	NA
HAAO	6.356373	-0.06438	0.084415	-0.76268	0.445653	NA
XPO1	703.9891	0.064351	0.064926	0.991148	0.321613	0.651336
CXorf23	365.2859	0.064328	0.044051	1.460314	0.144204	0.495647
NDUFS5	287.3808	-0.06433	0.061974	-1.03797	0.299282	NA
ZNF674	53.18935	-0.06432	0.059759	-1.07635	0.281769	NA
STOM	174.36	-0.06431	0.080289	-0.80093	0.423171	NA
ELL	159.4012	-0.06431	0.05914	-1.08734	0.276887	NA
SNTG2	4.31973	0.0643	0.065185	0.986416	0.323929	NA
LPIN1	507.9941	0.06429	0.054423	1.1813	0.237484	0.586807
KCTD8	162.7426	0.064286	0.061679	1.042259	0.297292	NA
ZNF646	159.1813	0.064283	0.058052	1.107328	0.268152	NA
SEPSECS	42.5778	-0.06427	0.079606	-0.80738	0.419449	NA
TMEM33	185.5376	0.064267	0.053988	1.1904	0.233889	NA
GIPR	1.924442	0.064256	0.062063	1.035346	0.300507	NA
AGPAT1	90.19656	0.064235	0.064226	1.000143	0.317241	NA
STARD8	18.30311	-0.06423	0.08484	-0.75705	0.44902	NA
IMPG1	8.327318	0.064218	0.084848	0.756859	0.449135	NA
ZNF263	137.6278	0.06421	0.041034	1.564775	0.117636	NA
CYP2E1	48.65863	-0.0642	0.079716	-0.80542	0.420578	NA
RALA	84.90244	0.064198	0.058102	1.104919	0.269195	NA
SATL1	2.63473	0.06419	0.072727	0.882618	0.377443	NA
FAM193B	297.2228	-0.06416	0.063891	-1.00417	0.315296	NA
WDR5	108.2699	0.064149	0.059008	1.087116	0.276985	NA
PPM1N	0.704285	0.064147	0.047658	1.345971	0.178312	NA
IWS1	359.7293	0.064143	0.043875	1.461956	0.143753	0.495647
DHRS1	23.52094	-0.06414	0.077601	-0.82652	0.408507	NA
ITCH	357.2495	0.064131	0.032589	1.967854	0.049085	0.34527
MIR138-1	0.712021	0.064116	0.047506	1.349648	0.177129	NA
HRH4	0.62511	0.064095	0.043939	1.458725	0.144641	NA
PTPN3	20.3433	-0.06409	0.072445	-0.88471	0.376315	NA
IFI30	6.036238	-0.06409	0.063201	-1.01407	0.310551	NA
TUBA3E	2.577866	0.064089	0.073635	0.870365	0.384101	NA
IL18R1	6.21009	-0.06407	0.068918	-0.92962	0.352569	NA

TACR2	13.56008	-0.06406	0.082891	-0.77284	0.439616	NA
LOC14547	10.93301	-0.06405	0.084908	-0.75432	0.450655	NA
AHCY	37.9646	-0.06405	0.077615	-0.82518	0.409267	NA
TMX3	249.6822	0.064043	0.059871	1.069685	0.284761	NA
UBA2	478.2289	0.06403	0.037743	1.696488	0.089793	0.415114
VPS13A	1001.637	0.063977	0.032141	1.990507	0.046535	0.337641
MYO3B	0.896137	-0.06396	0.051124	-1.25117	0.210873	NA
C11orf54	73.07566	-0.06396	0.063997	-0.99945	0.317575	NA
VPS8	310.216	0.063959	0.035064	1.824045	0.068145	NA
USP38	77.48574	0.063954	0.051981	1.230325	0.218575	NA
OBSL1	387.5806	-0.06393	0.060739	-1.05255	0.292546	0.6257
LPCAT3	95.78585	-0.06393	0.065257	-0.97963	0.327269	NA
IRF2BPL	162.3532	0.063926	0.065977	0.968903	0.332594	NA
AP2M1	710.2836	0.063924	0.046131	1.385697	0.165839	0.52098
EDNRA	6.397106	-0.06392	0.084128	-0.75978	0.447384	NA
TMPRSS11	0.271941	-0.06392	0.030141	-2.12064	0.033952	NA
MYLK2	0.676953	0.063917	0.047234	1.353191	0.175995	NA
ATAD3A	48.02569	0.063916	0.068706	0.930291	0.35222	NA
SPRY4	42.96737	-0.06391	0.084515	-0.75624	0.449508	NA
MIR1286	3.640953	0.063905	0.078491	0.81417	0.415548	NA
MIR3178	1.273041	0.063895	0.059106	1.08101	0.279693	NA
PAQR3	96.43778	0.063892	0.054495	1.172431	0.241024	NA
NPNT	3.095246	0.063891	0.071639	0.891846	0.372476	NA
TRIM72	35.7991	-0.06389	0.078948	-0.80921	0.418397	NA
RNF141	111.7198	-0.06386	0.076023	-0.83997	0.400923	NA
SNORD11	7.874968	-0.06385	0.084924	-0.75189	0.452114	NA
PRDX2	173.2085	-0.06385	0.070963	-0.89972	0.368271	NA
ADAM33	20.80983	-0.06385	0.08475	-0.75335	0.451237	NA
TMEM220	25.45325	0.063844	0.079882	0.79923	0.424157	NA
COL1A1	6.598414	-0.06384	0.08214	-0.77715	0.437069	NA
LPPR1	10.19399	-0.06383	0.083785	-0.76185	0.446152	NA
SNORD15A	20.8165	-0.06382	0.084456	-0.7557	0.449827	NA
SNORD24	10.09285	-0.06382	0.075588	-0.84432	0.39849	NA
TMEM204	17.62861	-0.06381	0.084341	-0.75662	0.449277	NA
ADAMTS1	0.607419	0.063806	0.044408	1.436799	0.150775	NA
TCTEX1D2	12.4742	-0.06379	0.082864	-0.76978	0.441432	NA
ERLIN2	101.2304	0.063775	0.07306	0.872908	0.382713	NA
DUSP5P1	2.82222	-0.06375	0.063086	-1.01051	0.312249	NA
DYM	239.9385	0.063749	0.034876	1.827871	0.067569	NA
RAD54L	40.96066	0.063717	0.083604	0.762138	0.445978	NA
SPDYE7P	2.473073	0.063714	0.0659	0.96683	0.333629	NA
NUPR1L	7.045708	-0.06371	0.084909	-0.75035	0.453046	NA
DPP9	121.9705	-0.06368	0.047693	-1.33523	0.181801	NA
IL12A	1.175859	0.063676	0.052895	1.203823	0.228658	NA
GOLIM4	349.5893	-0.06367	0.070538	-0.9027	0.366685	0.689818
CCDC132	170.0714	0.063659	0.049642	1.282372	0.199712	NA
ALMS1	1638.124	0.063655	0.050649	1.256792	0.208829	0.55778

GFPT2	196.0224	0.063652	0.081449	0.781501	0.434508	NA
LCTL	0.980705	-0.06363	0.050804	-1.25254	0.210374	NA
FGD5	20.71931	0.06363	0.080714	0.788344	0.430496	NA
MIR3650	2.089483	-0.06362	0.071572	-0.88897	0.37402	NA
EPB49	249.5368	0.063621	0.045997	1.38316	0.166616	NA
MRPS27	95.26115	-0.0636	0.06041	-1.05286	0.292406	NA
YTHDC2	350.1873	-0.06358	0.046949	-1.3543	0.175639	0.529097
NFATC4	45.04141	-0.06358	0.078466	-0.81031	0.417762	NA
DOK1	3.783879	-0.06358	0.079934	-0.79542	0.426371	NA
NEDD8	5.425708	-0.06358	0.081882	-0.77648	0.437466	NA
HIST2H2B	62.28009	0.063579	0.077248	0.82305	0.410479	NA
LEF1	14.37316	0.063577	0.083891	0.757857	0.448537	NA
POLR3B	74.89868	0.063572	0.062943	1.009983	0.312503	NA
KIAA1456	3236.63	0.06357	0.045491	1.397426	0.162285	0.519328
TAF9B	109.4127	-0.06354	0.052631	-1.20737	0.22729	NA
ESD	46.05443	-0.06354	0.070908	-0.89608	0.370208	NA
HIST1H4E	223.7313	0.063538	0.067771	0.937537	0.348482	NA
C11orf24	56.79892	0.063537	0.065179	0.974807	0.329656	NA
SEC24B	287.4682	0.063537	0.041237	1.540753	0.123377	NA
TXNDC5	10.33411	0.063533	0.084369	0.753043	0.451424	NA
COX14	40.57424	-0.06353	0.074183	-0.85639	0.391781	NA
OR4M2	0.635882	-0.06352	0.037425	-1.69727	0.089646	NA
ZBTB1	260.0377	0.063515	0.061999	1.02445	0.305623	NA
TGFB1	28.23529	-0.06351	0.080476	-0.78917	0.430012	NA
SEC62	739.3451	0.063503	0.054669	1.161575	0.245408	0.592704
DLG2	1012.894	-0.06348	0.07489	-0.84766	0.396629	0.705708
AP5B1	182.3983	0.06348	0.049681	1.277763	0.201333	NA
PNMAL2	122.1446	0.063476	0.075198	0.844119	0.398603	NA
RGS17	43.79347	-0.06346	0.073185	-0.86716	0.385854	NA
ATP6V0A1	594.4581	0.063459	0.036158	1.755044	0.079252	0.397742
PARP11	44.94457	0.063458	0.065858	0.963564	0.335264	NA
SCUBE3	107.8323	-0.06345	0.072141	-0.87959	0.379081	NA
NAA20	79.13402	0.063444	0.052798	1.201645	0.229501	NA
HIST1H2B	0.681885	-0.06344	0.042404	-1.49612	0.134623	NA
DUSP23	7.750108	-0.06344	0.084691	-0.74908	0.453812	NA
DHX15	422.1672	0.06343	0.034812	1.822084	0.068442	0.382769
UBXN2B	237.8159	0.063428	0.046504	1.363944	0.172585	NA
RND2	74.28727	-0.06341	0.078301	-0.80987	0.418016	NA
COMMD2	116.6523	0.063391	0.052847	1.199517	0.230327	NA
MED24	306.4269	0.063361	0.056055	1.130334	0.258335	NA
EDAR	3.37817	0.063355	0.07664	0.82666	0.40843	NA
NCAPD3	129.7553	0.063347	0.061054	1.037544	0.299482	NA
CASP7	17.5227	-0.06334	0.084604	-0.7487	0.454037	NA
SLC1A3	2162.493	-0.06332	0.081182	-0.77998	0.435404	0.738166
FAM188B	1.404916	0.063309	0.059119	1.070886	0.284221	NA
FAM154A	0.416966	0.063283	0.037481	1.688379	0.091338	NA
NCR3LG1	346.4972	-0.06327	0.051439	-1.22997	0.218707	0.569521

KCNJ1	0.944299	-0.06326	0.051265	-1.23407	0.217178	NA
MVK	20.73064	-0.06326	0.077934	-0.81173	0.416948	NA
EGR1	30.43844	-0.06326	0.076356	-0.82849	0.407394	NA
PPID	101.3615	0.063252	0.074764	0.846025	0.397539	NA
ZFP62	163.9644	0.063243	0.051179	1.235739	0.216556	NA
RNU4ATA	24.59107	-0.06324	0.076442	-0.82726	0.408091	NA
CST7	13.48755	0.063233	0.084108	0.75181	0.452165	NA
MIR4668	0.351344	0.063207	0.0322	1.962928	0.049654	NA
MTMR12	210.2649	0.063194	0.051312	1.231583	0.218105	NA
LOC10050	7.644236	0.063184	0.084489	0.747836	0.454559	NA
TUBB	332.2742	0.063183	0.060931	1.036962	0.299753	NA
MIR493	1.877131	0.063174	0.06734	0.938132	0.348176	NA
STX3	177.742	-0.06317	0.052486	-1.2035	0.228782	NA
SLC19A3	9.12731	-0.06316	0.084122	-0.75086	0.452736	NA
SNORA6	6.981833	-0.06315	0.082662	-0.76401	0.444863	NA
HEXIM1	180.3757	-0.06315	0.050454	-1.25164	0.210703	NA
EPM2AIP1	506.3557	0.063145	0.033443	1.888137	0.059008	0.36517
PRRC2A	1089.88	0.063144	0.041995	1.503615	0.132681	0.487416
SCAMP5	404.9983	0.063138	0.052928	1.192896	0.23291	0.583563
GLE1	166.1403	0.06313	0.046762	1.350042	0.177002	NA
UNC93B1	7.444914	-0.06313	0.082892	-0.76159	0.446308	NA
SDF4	309.7651	-0.06312	0.063594	-0.99255	0.320931	NA
FANCF	13.22176	0.063118	0.083773	0.753447	0.451181	NA
MIR548AO	1.996218	-0.0631	0.065922	-0.95716	0.338486	NA
ORC6	10.61048	-0.06309	0.084926	-0.74284	0.457577	NA
CCDC163P	8.298494	-0.06308	0.076484	-0.82477	0.4095	NA
VAPB	456.9435	0.063074	0.053403	1.181076	0.237573	0.586807
STXBP3	131.4487	0.063071	0.052535	1.200543	0.229928	NA
LOC10012	4.697785	0.063064	0.081801	0.770953	0.440735	NA
ZNF423	181.0134	0.063059	0.066075	0.954352	0.339906	NA
KRT18	22.04899	0.063056	0.082052	0.768495	0.442193	NA
CSK	143.1464	-0.06304	0.062556	-1.00782	0.313542	NA
MAN1B1	142.4296	-0.06304	0.051686	-1.21966	0.222592	NA
LOC10013	21.31769	-0.06304	0.078649	-0.80152	0.422829	NA
A1BG-AS1	13.95166	0.063038	0.082751	0.761784	0.446189	NA
SHARPIN	60.86336	-0.06303	0.057804	-1.09036	0.275554	NA
SLC2A12	185.329	-0.06302	0.06546	-0.96278	0.335659	NA
THAP10	26.21616	0.06302	0.081899	0.769488	0.441604	NA
MYCBP	1.371035	-0.06301	0.060431	-1.0427	0.297086	NA
GOLGB1	2060.248	-0.06301	0.05279	-1.19357	0.232644	0.583543
SLC7A5P1	26.90026	0.063	0.079072	0.796739	0.425603	NA
CBR3-AS1	14.27744	-0.063	0.084843	-0.74255	0.457756	NA
DISP2	329.9663	0.062995	0.054676	1.152164	0.249254	NA
UROC1	0.599173	0.062986	0.039181	1.607565	0.107931	NA
SBK1	359.4512	0.062981	0.064842	0.971298	0.3314	0.660832
ADAMTS1	17.28089	0.062981	0.084938	0.741493	0.458395	NA
WDR60	363.6011	-0.06297	0.057677	-1.09178	0.274929	0.613511

HSD11B1L	32.17999	-0.06297	0.069201	-0.90996	0.362845	NA
SLC5A11	18.65077	-0.06296	0.08441	-0.74592	0.455718	NA
CABIN1	652.6292	0.062951	0.056305	1.118045	0.263548	0.606026
SIGLEC9	2.970357	-0.06294	0.066795	-0.94236	0.34601	NA
LOC73127	24.88919	-0.06293	0.084303	-0.74651	0.455359	NA
LUM	0.832045	0.062931	0.049533	1.270489	0.20391	NA
EME2	39.51419	-0.06292	0.075272	-0.83587	0.40323	NA
ADH6	0.468404	-0.06292	0.040725	-1.54492	0.122365	NA
RP9	22.32003	0.062914	0.076866	0.81849	0.413077	NA
IL12RB2	3.416384	0.062914	0.071706	0.877379	0.380281	NA
MRO	112.4416	-0.06291	0.082417	-0.76335	0.445257	NA
MGLL	369.657	0.062903	0.054495	1.154291	0.248381	0.592704
HAVCR1	1.750734	0.062883	0.063999	0.982569	0.32582	NA
RBL1	168.8715	0.062883	0.064953	0.968134	0.332977	NA
STX16	231.8056	-0.06288	0.042113	-1.49314	0.135401	NA
ESPNP	3.072276	-0.06288	0.062546	-1.00527	0.314767	NA
ALDH5A1	240.7104	-0.06287	0.054049	-1.16312	0.24478	NA
LOC10028	13.56066	-0.06286	0.084271	-0.74596	0.455691	NA
AMZ2	202.1885	0.062858	0.048944	1.284274	0.199046	NA
ATP7B	173.5323	0.06285	0.05825	1.078964	0.280604	NA
SPTB	1980.412	0.06284	0.044374	1.416146	0.156733	0.513748
PPP2R5D	193.6408	0.06284	0.0505	1.244352	0.21337	NA
ENPP2	70.96306	-0.06284	0.084698	-0.74192	0.458136	NA
GPR35	8.619494	-0.06283	0.083628	-0.75128	0.452486	NA
ATG9B	7.255232	-0.06283	0.084199	-0.74617	0.455562	NA
LOC14814	4.815689	0.062808	0.074823	0.839416	0.401236	NA
PPOX	31.0007	-0.06279	0.075287	-0.83396	0.404305	NA
RRM1	159.5679	0.062776	0.053198	1.180035	0.237986	NA
DBT	214.8051	0.062773	0.049185	1.27627	0.20186	NA
SPAG16	64.75204	-0.06277	0.060546	-1.03676	0.299848	NA
KREMEN1	57.24012	-0.06277	0.072342	-0.8677	0.385561	NA
ZNF8	125.2899	0.062769	0.060483	1.037799	0.299364	NA
MIR5690	1.44354	-0.06277	0.051095	-1.22846	0.219275	NA
ELP2	245.7689	-0.06276	0.05579	-1.12501	0.260586	NA
OTUD1	70.16811	-0.06275	0.060043	-1.04516	0.29595	NA
UBE2W	249.739	0.062737	0.04316	1.453587	0.146061	NA
SLC26A7	0.666858	0.062736	0.040905	1.533693	0.125105	NA
LOH12CR1	66.48785	-0.06273	0.071601	-0.87607	0.380992	NA
RBFA	142.3641	0.062709	0.053823	1.165082	0.243986	NA
GPM6A	1835.849	0.062707	0.068271	0.918512	0.358351	0.682359
TDP1	76.56919	-0.0627	0.059818	-1.04821	0.29454	NA
PSMG2	42.29246	-0.06269	0.071141	-0.8812	0.378211	NA
C6	2.202542	-0.06269	0.067542	-0.92814	0.353333	NA
INTS12	34.96577	0.062677	0.068318	0.917421	0.358922	NA
IDH1	57.36616	0.062671	0.06193	1.011958	0.311558	NA
HLCS	209.8323	0.062671	0.049952	1.254608	0.209621	NA
PTPRT	45.03453	-0.06267	0.084932	-0.73788	0.460586	NA

MFSD12	117.2949	-0.06267	0.057487	-1.0901	0.275668	NA
ATF2	384.2251	0.062666	0.040716	1.539097	0.123781	0.477296
MIR3909	1.003875	0.062628	0.055495	1.128543	0.25909	NA
RAD52	114.111	-0.06263	0.069677	-0.89883	0.368745	NA
ABAT	599.9109	-0.06261	0.053635	-1.16736	0.243066	0.591982
ABCF2	132.5102	0.06261	0.052664	1.18886	0.234495	NA
MUSK	0.660865	-0.06261	0.043883	-1.4267	0.153666	NA
ATP11B	385.9474	0.062603	0.042681	1.46679	0.142433	0.495647
PTPRE	86.70805	-0.0626	0.065059	-0.96223	0.335936	NA
SNAI1	6.710785	-0.0626	0.081333	-0.76965	0.441507	NA
SGOL2	27.86668	-0.0626	0.074207	-0.84355	0.398922	NA
C6orf165	3.271173	0.062593	0.075881	0.824886	0.409436	NA
HCG27	22.86453	-0.06259	0.084631	-0.73957	0.459563	NA
STARD13	58.82517	-0.06259	0.074895	-0.83568	0.403337	NA
SMOC2	9.964136	-0.06259	0.084681	-0.7391	0.459847	NA
ACBD6	159.6101	-0.06257	0.047144	-1.32711	0.184474	NA
CHRM4	1.076881	-0.06256	0.054311	-1.15194	0.249346	NA
RFPL3	0.67776	0.062563	0.044698	1.399672	0.161612	NA
XPA	177.4331	-0.06255	0.060431	-1.03503	0.300653	NA
THOP1	139.8933	-0.06255	0.057865	-1.08091	0.279737	NA
NPIPL3	116.8398	0.062544	0.063579	0.983711	0.325258	NA
FLJ30679	1.258676	0.062543	0.057655	1.084777	0.27802	NA
TASP1	47.6073	0.062543	0.067687	0.924008	0.355482	NA
ZNF628	16.58241	-0.06254	0.083554	-0.74848	0.454169	NA
TPM3P9	70.599	-0.06253	0.07811	-0.80057	0.423381	NA
SNHG8	34.42071	-0.06253	0.084469	-0.74029	0.459123	NA
FBXL20	487.9105	-0.06253	0.042175	-1.48261	0.138178	0.489381
C1orf85	14.17646	-0.06252	0.082692	-0.75608	0.4496	NA
LINC00571	5.153439	0.062521	0.083024	0.753049	0.45142	NA
NRK	103.9404	-0.06252	0.084318	-0.74147	0.458409	NA
P2RY4	1.099638	-0.06251	0.056548	-1.10551	0.268939	NA
MAST2	225.4077	0.062502	0.046291	1.350191	0.176955	NA
RNF215	37.41019	0.062488	0.070831	0.882212	0.377662	NA
IDS	1063.763	0.06248	0.064532	0.968214	0.332938	0.660832
CACNA2D3	10.5579	-0.06247	0.084333	-0.74079	0.458822	NA
TNFRSF10	1.164765	-0.06246	0.054594	-1.14406	0.252599	NA
PABPC4	161.522	-0.06244	0.043034	-1.45091	0.146805	NA
RUVBL2	88.8413	0.062427	0.052548	1.188009	0.23483	NA
CBR1	49.00292	-0.06243	0.075904	-0.82242	0.410837	NA
C1orf43	215.8212	-0.06242	0.069928	-0.89269	0.372024	NA
SLC7A11-A	14.35521	-0.06242	0.084928	-0.73498	0.462351	NA
LRRC3-AS1	1.979487	0.062403	0.059778	1.043907	0.296528	NA
GAD1	132.1212	-0.0624	0.08441	-0.73924	0.459762	NA
MFSD10	73.75031	-0.0624	0.065721	-0.94945	0.342392	NA
KNTC1	89.23341	-0.06238	0.067149	-0.92899	0.352895	NA
PML	75.8689	0.062379	0.075611	0.825008	0.409367	NA
PCBP1-AS1	79.11023	0.062366	0.072764	0.857104	0.391387	NA

RACGAP1P	1.743853	-0.06236	0.065188	-0.95668	0.338728	NA
FRMD3	40.66116	-0.06236	0.077482	-0.80484	0.420914	NA
ARPC1B	20.73144	-0.06235	0.083036	-0.75091	0.452704	NA
COL24A1	9.400675	0.062335	0.084001	0.742079	0.45804	NA
LINC00665	45.40728	-0.06233	0.06311	-0.98766	0.323317	NA
TSEN15	38.65392	0.06231	0.069834	0.892258	0.372255	NA
VAV2	25.5768	-0.06229	0.081987	-0.75981	0.447368	NA
VASN	21.58303	-0.06229	0.082373	-0.75621	0.449521	NA
TMEM252	4.082762	-0.06228	0.07718	-0.807	0.419667	NA
MIR1193	1.384792	-0.06228	0.061657	-1.01018	0.312409	NA
COL1A2	14.04521	0.062274	0.083356	0.747089	0.45501	NA
MIRLET7A	7.963449	0.062272	0.084041	0.740963	0.458716	NA
RPP25L	16.71161	-0.06227	0.081193	-0.76693	0.443122	NA
EXOSC1	35.90275	-0.06225	0.068881	-0.90375	0.366127	NA
C1GALT1C	29.26185	-0.06224	0.070634	-0.88116	0.378232	NA
TRIM66	178.7758	-0.06224	0.073239	-0.84981	0.395431	NA
RPP21	3.199412	0.062234	0.078467	0.793123	0.427706	NA
SNORD58A	16.80455	-0.06223	0.084793	-0.73392	0.462995	NA
AGRN	36.43157	0.062225	0.081608	0.762478	0.445775	NA
LRP11	129.4017	0.062223	0.049094	1.267424	0.205004	NA
PTENP1	21.00797	0.062213	0.07661	0.812078	0.416747	NA
PTPRR	334.8941	0.062211	0.04261	1.460005	0.144289	0.495647
PIGN	109.695	-0.06221	0.05949	-1.04568	0.29571	NA
UBXN11	38.76582	-0.06218	0.075857	-0.81971	0.412384	NA
MIDN	81.97417	-0.06217	0.067195	-0.92523	0.354845	NA
AFF4	1237.988	0.062167	0.033754	1.841767	0.065509	0.375222
BMP2	4.039502	-0.06216	0.075414	-0.82428	0.40978	NA
LOC54147	1.922101	-0.06216	0.068725	-0.9044	0.365782	NA
FASTKD5	93.99033	0.062151	0.062715	0.991006	0.321682	NA
FMO2	6.772298	0.062137	0.077572	0.801019	0.423121	NA
ASB16-AS1	37.0451	-0.06211	0.071776	-0.86534	0.386852	NA
ERGIC3	90.00853	-0.06209	0.063454	-0.97846	0.327846	NA
KCTD9	118.1993	-0.06208	0.058534	-1.06054	0.288898	NA
PPP1R3D	31.59107	-0.06207	0.068208	-0.91006	0.362788	NA
RYR3	688.0579	-0.06207	0.073591	-0.84339	0.399008	0.707162
SH2D1A	2.142942	0.06206	0.06631	0.935906	0.349321	NA
RPSAP58	24.40721	-0.06205	0.077289	-0.80289	0.422038	NA
REEP1	62.33067	0.062049	0.074477	0.833124	0.404775	NA
TP53BP2	168.6553	-0.06205	0.063924	-0.97067	0.331714	NA
VDAC1	201.8268	0.062044	0.068159	0.910283	0.362673	NA
ECE1	60.30378	-0.06204	0.068004	-0.91231	0.361604	NA
ZNF766	88.68491	0.06204	0.055539	1.11704	0.263977	NA
ANKRD24	29.31174	-0.06202	0.080877	-0.76687	0.44316	NA
ZNF226	313.3568	0.062017	0.058195	1.065691	0.286564	NA
LOXL3	18.70449	0.062016	0.082907	0.748013	0.454452	NA
POLR2C	101.844	-0.06201	0.047096	-1.31676	0.187918	NA
TAOK3	805.5644	-0.06201	0.049846	-1.24407	0.213473	0.562782

SEC16B	4.81253	-0.06201	0.081895	-0.75718	0.448941	NA
RILPL1	89.82993	0.062007	0.056819	1.091306	0.275138	NA
CNIH	62.07025	-0.06199	0.074175	-0.83575	0.403296	NA
C19orf71	33.20949	-0.06198	0.07914	-0.78313	0.433551	NA
CDKN2C	19.67161	-0.06196	0.082958	-0.74692	0.455115	NA
CSNK1G3	249.0914	0.061948	0.045907	1.349417	0.177203	NA
ORC2	220.4622	-0.06195	0.050533	-1.22585	0.220255	NA
ARL1	190.2532	0.061942	0.060315	1.026985	0.304428	NA
TRAF3IP2	112.5616	-0.06194	0.07314	-0.84681	0.397101	NA
NAA40	94.2759	-0.06193	0.053752	-1.15205	0.2493	NA
KGFLP1	3.627651	-0.06192	0.071781	-0.86268	0.388316	NA
CLEC1A	2.059694	-0.06192	0.063284	-0.9785	0.327828	NA
RASGRF2	46.57589	-0.06192	0.072327	-0.85616	0.391908	NA
GPSM2	125.4806	0.061917	0.061907	1.000159	0.317234	NA
APOBEC3D	1.020263	-0.06192	0.053484	-1.15767	0.246999	NA
GRIK3	147.4161	-0.06189	0.075324	-0.82164	0.411284	NA
LOC44046	17.89569	0.061878	0.084692	0.730632	0.465004	NA
MAFB	13.01021	0.061876	0.079555	0.777779	0.436699	NA
CTBP1	347.2015	0.06187	0.045397	1.362871	0.172923	0.525733
LOC28303	2.407469	-0.06187	0.072041	-0.8588	0.390451	NA
LYG2	34.01451	0.061867	0.073347	0.843475	0.398963	NA
STXBP5	948.6301	0.061856	0.05063	1.221726	0.221811	0.570264
CKAP2	148.8467	-0.06185	0.061275	-1.00933	0.312818	NA
CHRDL1	40.77521	-0.06185	0.084349	-0.73321	0.463433	NA
RPPH1	1391.624	-0.06184	0.08492	-0.72823	0.466474	0.755844
LRRC31	0.657695	-0.06184	0.044594	-1.38664	0.16555	NA
PDZD9	15.73273	-0.06184	0.082679	-0.7479	0.454519	NA
NUDT18	27.6108	0.061834	0.078518	0.78751	0.430984	NA
UTP6	197.7554	-0.06183	0.050308	-1.22899	0.219076	NA
DDX26B	213.1387	0.061822	0.059269	1.043069	0.296917	NA
PHC3	1228.205	0.061822	0.032416	1.907109	0.056506	0.361261
ZNF682	55.77402	0.061807	0.059913	1.031617	0.302252	NA
IRGQ	452.6092	0.061804	0.04628	1.33544	0.181732	0.530978
CBS	434.1805	-0.0618	0.050262	-1.22962	0.218838	0.569521
SNORD81	2.893859	-0.0618	0.074454	-0.83003	0.406523	NA
FCGR2B	1.164016	-0.0618	0.049297	-1.25353	0.210015	NA
CDC42BPB	812.8091	0.06178	0.038266	1.614471	0.106425	0.452973
RLBP1	7.377533	0.061771	0.084512	0.730909	0.464835	NA
ZNF395	89.32179	-0.06176	0.058336	-1.05863	0.289766	NA
ZFAT	75.36465	0.06175	0.064386	0.959058	0.33753	NA
PCBD2	24.7076	-0.06175	0.079319	-0.77849	0.436281	NA
CKM	2.864001	0.061748	0.068151	0.906047	0.364911	NA
C11orf63	62.47976	0.061742	0.060553	1.019641	0.307899	NA
ZNF502	65.14409	0.061738	0.06285	0.982301	0.325952	NA
CA10	635.8642	0.061736	0.045838	1.346832	0.178034	0.530729
DHRS9	0.508663	-0.06173	0.037512	-1.64549	0.099868	NA
LOC64338	1.43388	-0.06172	0.056336	-1.09553	0.273286	NA

SYNGR4	1.441811	-0.06171	0.056136	-1.09928	0.271646	NA
TSTA3	25.12027	-0.0617	0.076634	-0.80512	0.420753	NA
ZNF410	117.7505	-0.0617	0.060975	-1.01183	0.311618	NA
RUFY1	202.8596	-0.06163	0.044313	-1.3908	0.164287	NA
LHFPL3	21.69977	-0.06163	0.08359	-0.73728	0.460952	NA
SLC5A2	15.32125	0.061628	0.084795	0.726797	0.46735	NA
TTC21B	664.5599	-0.06163	0.050869	-1.21149	0.225708	0.576749
MGC4580	1.998448	-0.06163	0.060285	-1.02225	0.306662	NA
WWC2	79.53044	-0.06162	0.07234	-0.85184	0.394301	NA
GPC5	22.98676	-0.06162	0.084502	-0.72918	0.46589	NA
NOS1	136.3571	-0.06162	0.08491	-0.72567	0.468042	NA
MIR3687	170.5717	-0.06161	0.080071	-0.76951	0.441593	NA
GPR182	14.335	-0.0616	0.084165	-0.73194	0.464205	NA
ERCC5	17.34772	-0.0616	0.081722	-0.75376	0.450996	NA
HIST3H3	1.505613	-0.0616	0.062709	-0.98228	0.325963	NA
ZNF267	63.40851	0.061578	0.068883	0.893961	0.371342	NA
FOXF1	6.955391	-0.06157	0.082519	-0.7461	0.455607	NA
CA5BP1	14.077	0.061558	0.08095	0.760448	0.446987	NA
SIAH1	60.04215	0.061555	0.057618	1.068322	0.285375	NA
GREM1	21.6535	-0.06155	0.084932	-0.7247	0.468637	NA
CLTA	217.838	-0.06155	0.059323	-1.03753	0.299488	NA
GNL2	109.5507	0.061542	0.057619	1.068091	0.285479	NA
AADAT	6.288881	0.061537	0.084314	0.729857	0.465477	NA
SCARNA5	58.34374	-0.06153	0.08191	-0.75114	0.452566	NA
COLEC11	9.685657	-0.06153	0.077119	-0.79781	0.424981	NA
CWC15	100.0581	-0.06152	0.060438	-1.01799	0.308682	NA
ING5	208.7251	-0.06152	0.06561	-0.93771	0.348395	NA
CA3	8.309574	0.061517	0.084928	0.724351	0.468851	NA
LOC10013	18.90522	-0.06151	0.083597	-0.73582	0.46184	NA
MBD3	203.3577	0.061506	0.060828	1.011148	0.311946	NA
MAEL	6.634646	-0.06151	0.08417	-0.73074	0.464939	NA
SGSH	15.42324	-0.0615	0.084937	-0.7241	0.469006	NA
RNF133	83.29049	-0.0615	0.067661	-0.90897	0.363367	NA
DDX39A	51.51474	-0.06148	0.068011	-0.904	0.365997	NA
WFDC2	1.55358	-0.06148	0.060847	-1.01037	0.312319	NA
DHCR24	227.4585	0.061476	0.061519	0.999303	0.317648	NA
LGALS4	2.286849	-0.06147	0.063929	-0.96146	0.336319	NA
SF3B3	470.8571	0.061461	0.036723	1.673658	0.094198	0.421656
USPL1	118.9514	0.061451	0.050328	1.221025	0.222077	NA
SSH2	334.6783	0.061448	0.043952	1.398066	0.162093	0.519328
MAGOH2	6.099824	-0.06144	0.083349	-0.7372	0.461	NA
UNC13D	51.27973	-0.06144	0.08402	-0.73129	0.464599	NA
METAP2	259.0886	-0.06144	0.046138	-1.33173	0.182948	NA
C10orf32	40.53058	-0.06144	0.070095	-0.87655	0.380733	NA
ANKFN1	5.288126	-0.06142	0.081074	-0.75761	0.448682	NA
TMEM182	9.203129	0.06142	0.084079	0.730503	0.465083	NA
SREBF2	612.2107	0.061411	0.061176	1.003848	0.315452	0.645996

ELOVL6	78.01506	0.0614	0.06343	0.967994	0.333047	NA
ICT1	46.23565	-0.06139	0.069482	-0.88352	0.376954	NA
MIR4737	2.146013	0.061386	0.062631	0.980119	0.327027	NA
SCARNA9	87.22977	-0.06137	0.077224	-0.79476	0.426751	NA
MIB1	585.0394	0.061369	0.041403	1.482227	0.13828	0.489381
NAA38	656.6142	0.061369	0.055482	1.106114	0.268677	0.610306
HMGCLL1	71.96227	0.061368	0.068551	0.89522	0.370669	NA
SALL2	489.7171	0.061365	0.048708	1.259851	0.207723	0.55642
MEN1	100.972	-0.06135	0.050434	-1.21643	0.223823	NA
KLHL17	30.62304	-0.06135	0.084316	-0.72756	0.466881	NA
ALG9	152.491	0.061334	0.046024	1.332654	0.182645	NA
ACSM3	0.658992	0.061316	0.042823	1.431826	0.152194	NA
GNG2	79.25196	-0.06131	0.084939	-0.72184	0.470395	NA
MYH10	1038.936	0.061305	0.037662	1.627752	0.103577	0.445167
CXCL11	0.627035	0.061301	0.033879	1.809424	0.070385	NA
ENPP5	170.7837	0.061292	0.070308	0.871766	0.383336	NA
TGM1	38.69151	-0.06129	0.076445	-0.80171	0.422723	NA
CBLL1	179.3458	0.06127	0.045933	1.333891	0.18224	NA
HAUS7	24.47324	-0.06127	0.07403	-0.82762	0.407885	NA
TAF1B	78.48369	0.06126	0.0668	0.917073	0.359104	NA
CFC1	0.36932	-0.06125	0.036027	-1.7002	0.089094	NA
MKI67	1.909852	-0.06125	0.039784	-1.53959	0.123659	NA
RXRB	96.44506	-0.06124	0.055992	-1.0938	0.274043	NA
HS3ST4	22.95144	-0.06124	0.084874	-0.72155	0.470574	NA
PNISR	2620.96	-0.06124	0.069434	-0.88199	0.377781	0.69794
RGS9	44.74703	-0.06124	0.084842	-0.7218	0.470415	NA
HGF	4.44317	-0.06123	0.073493	-0.83317	0.404751	NA
GRIK4	23.88165	-0.06123	0.081643	-0.74993	0.453297	NA
AGPAT4-IT	13.576	-0.06122	0.083369	-0.73435	0.462735	NA
C8orf34	69.75957	0.061219	0.082696	0.740287	0.459126	NA
DZANK1	21.39637	-0.06121	0.078549	-0.7793	0.435805	NA
CDK5RAP2	622.4585	-0.06121	0.060172	-1.01723	0.309042	0.639252
LOC10013	0.781051	-0.0612	0.045557	-1.34348	0.179117	NA
SLC24A3	81.04538	-0.06119	0.083364	-0.73398	0.462964	NA
TSPAN9	726.9658	-0.06118	0.058108	-1.05294	0.292368	0.6257
LOC10013	0.760961	-0.06115	0.046297	-1.32082	0.186562	NA
OR52D1	0.719423	-0.06114	0.048215	-1.26814	0.204749	NA
PPP1R3G	3.579366	-0.06114	0.076429	-0.79989	0.423772	NA
FAM91A2	0.390273	-0.06112	0.036725	-1.66436	0.096041	NA
NEK11	41.47361	-0.06112	0.06998	-0.87334	0.382479	NA
SLC34A3	9.392044	0.061116	0.084102	0.726689	0.467417	NA
KLHL9	163.3183	0.061116	0.042541	1.436642	0.15082	NA
PCAT1	0.695233	0.061106	0.047128	1.29659	0.194772	NA
CXorf30	0.984958	-0.06109	0.051294	-1.19106	0.233629	NA
GYPC	4.866309	-0.06109	0.077892	-0.78424	0.432897	NA
PNO1	32.41087	0.061069	0.074479	0.819951	0.412244	NA
HEY1	56.14603	-0.06106	0.069018	-0.88468	0.376328	NA

C5orf30	37.96336	0.061054	0.068427	0.892238	0.372265	NA
THAP4	89.1653	0.061047	0.048602	1.256076	0.209089	NA
PUS10	42.8299	-0.06105	0.069689	-0.87599	0.381037	NA
LSM2	23.27133	-0.06105	0.083622	-0.73003	0.465372	NA
TRPV5	1.721652	-0.06103	0.064499	-0.94628	0.344005	NA
DHDDS	292.5525	-0.06102	0.054075	-1.12853	0.259096	NA
FDXACB1	4.062194	0.061024	0.07865	0.775889	0.437814	NA
TNRC18P1	76.53236	-0.06101	0.073409	-0.83113	0.405898	NA
WDR59	264.7949	-0.06101	0.065364	-0.93339	0.35062	NA
PMS2P1	60.41277	-0.06101	0.068766	-0.88722	0.374963	NA
NF1	1220.926	0.061009	0.038349	1.590882	0.111636	0.460575
AMOT	117.6189	-0.061	0.07506	-0.81271	0.416385	NA
ZBED6	364.3836	-0.061	0.077203	-0.7901	0.429468	0.73339
LOC44033	17.1389	-0.061	0.084856	-0.71884	0.472239	NA
ITGBL1	324.1496	0.060995	0.055683	1.095401	0.273341	NA
EZR	74.56864	-0.06098	0.082799	-0.73643	0.461467	NA
MMP9	4.753751	-0.06096	0.076674	-0.79509	0.426558	NA
GLYATL2	1.076677	0.060956	0.054327	1.122018	0.261855	NA
FLJ33360	13.61827	-0.06094	0.084524	-0.72103	0.470893	NA
EBF1	281.5694	-0.06093	0.071896	-0.84753	0.396699	NA
PDGFRA	55.70568	-0.06093	0.075838	-0.8034	0.421744	NA
FOXK2	324.2943	0.060923	0.050465	1.207253	0.227335	NA
SGK196	86.31225	0.060917	0.061338	0.99313	0.320647	NA
LOC10013	1.441149	0.060911	0.056956	1.069423	0.284879	NA
ZDHHC7	134.7959	0.060899	0.050971	1.194781	0.232173	NA
LOC40055	0.836249	-0.0609	0.048089	-1.26637	0.20538	NA
ING1	64.71818	-0.0609	0.065346	-0.93194	0.351366	NA
STAU2-AS	7.620406	0.060891	0.0777	0.783674	0.433231	NA
ANKEF1	39.3224	0.060891	0.070118	0.868403	0.385174	NA
PFDN4	72.76782	-0.06089	0.070342	-0.86562	0.386696	NA
TECPR2	312.3123	0.060888	0.034207	1.780011	0.075074	NA
TSSK3	82.78954	-0.06089	0.081863	-0.74378	0.45701	NA
CDC34	127.4063	-0.06089	0.048187	-1.26356	0.206388	NA
MSL2	312.5103	0.060879	0.04933	1.234107	0.217163	NA
FGD5-AS1	379.0412	0.06086	0.053939	1.128325	0.259183	0.599641
DENND2A	130.6099	-0.06086	0.06641	-0.91643	0.359442	NA
MAPKAPK	34.77475	-0.06085	0.066825	-0.91063	0.36249	NA
LOC10050	80.41098	0.060848	0.083499	0.728728	0.466168	NA
TRUB2	59.99698	-0.06085	0.058173	-1.04594	0.295591	NA
CRYM	157.5273	0.060845	0.073828	0.824149	0.409855	NA
STX18	126.8572	-0.06084	0.057339	-1.0611	0.288646	NA
SOX2	135.8933	-0.06084	0.073646	-0.82607	0.408762	NA
PLEKHA3	91.35664	0.060831	0.057182	1.063825	0.287408	NA
PSMD5	96.94714	-0.06082	0.050273	-1.20982	0.226349	NA
AWAT1	2.408149	-0.06082	0.072971	-0.83343	0.4046	NA
MYL3	2.469176	-0.06081	0.071673	-0.8485	0.39616	NA
HIPK3	487.8217	0.060814	0.043052	1.41259	0.157776	0.514729

SERINC5	84.21545	-0.06079	0.060596	-1.00313	0.315797	NA
DYNC1I1	515.1584	-0.06078	0.050576	-1.20182	0.229434	0.57805
ATP6V1C2	6.781518	-0.06078	0.08291	-0.73305	0.463529	NA
ROBO2	185.7093	0.060772	0.083902	0.724314	0.468873	NA
ATG2A	176.0568	-0.06077	0.055144	-1.10203	0.270447	NA
RRM2	0.405845	-0.06075	0.03178	-1.91148	0.055943	NA
TOX2	5.25543	-0.06074	0.079996	-0.7593	0.44767	NA
CDK11A	33.39078	0.060731	0.082041	0.740244	0.459152	NA
MN1	23.28785	0.060707	0.084878	0.71523	0.474467	NA
MMEL1	0.578496	-0.06071	0.041377	-1.46714	0.142339	NA
SNORD113	11.17917	-0.0607	0.083155	-0.72995	0.46542	NA
TMPRSS4	0.80803	-0.06068	0.045399	-1.33663	0.181343	NA
LEPREL2	219.4531	-0.06068	0.064552	-0.94003	0.347202	NA
TPST2	19.8188	0.06068	0.083285	0.728588	0.466253	NA
LY86	1.550423	-0.06068	0.058172	-1.04305	0.296924	NA
ARL13A	3.489916	-0.06066	0.077979	-0.77791	0.43662	NA
SCG2	139.9588	0.06066	0.084913	0.714384	0.47499	NA
SUOX	40.35594	-0.06066	0.064306	-0.94327	0.345545	NA
HELZ	1093.37	0.060643	0.029366	2.065115	0.038912	0.300865
NCOA4	186.104	0.060643	0.048771	1.243422	0.213712	NA
HBA2	1.195444	-0.06064	0.059951	-1.01147	0.311792	NA
MRPL40	35.57852	-0.06063	0.075354	-0.80455	0.42108	NA
H1FX	259.85	0.060618	0.064051	0.946395	0.343947	NA
DGCR6	57.92664	-0.06062	0.065999	-0.91844	0.35839	NA
MIR663B	18.34503	-0.06061	0.077971	-0.77737	0.436943	NA
C9orf24	15.67155	-0.06061	0.08151	-0.74356	0.457143	NA
MIR181A2	16.38917	0.060605	0.082322	0.736195	0.461612	NA
TRIP11	677.653	0.060601	0.03929	1.542398	0.122977	0.476745
FLJ42289	5.837472	-0.06058	0.082818	-0.73145	0.464504	NA
RNU6-66	0.464991	-0.06056	0.036062	-1.67935	0.093083	NA
WEE1	26.37798	-0.06055	0.079539	-0.76122	0.446527	NA
MIR383	1.116311	-0.06053	0.053812	-1.12486	0.260647	NA
LOC10013	37.20985	-0.06052	0.073785	-0.82024	0.412081	NA
ZBTB10	247.8611	0.060504	0.044147	1.370523	0.170524	NA
ANKRD13D	107.8461	-0.0605	0.059844	-1.01088	0.312075	NA
TIMM17A	42.98917	0.060487	0.08088	0.74786	0.454544	NA
LIPA	51.66361	-0.06047	0.073881	-0.81843	0.413113	NA
TRIM36	4.456944	0.060442	0.079942	0.756074	0.449605	NA
PAK3	53.76546	-0.06044	0.084913	-0.71181	0.476585	NA
LOC28458	37.93564	0.060437	0.044756	1.350371	0.176897	NA
LINC00675	0.894452	-0.06043	0.05088	-1.18779	0.234917	NA
SCAND3	73.68448	0.060404	0.057327	1.053663	0.292037	NA
TAPBPL	13.88013	-0.0604	0.084537	-0.71452	0.474908	NA
MAP7D2	305.0706	-0.0604	0.057762	-1.04571	0.295695	NA
ACMSD	19.12883	0.060401	0.084696	0.71315	0.475753	NA
MCRS1	43.2197	0.060386	0.068413	0.882671	0.377414	NA
EEA1	671.056	0.060384	0.041225	1.46475	0.142989	0.495647

RAB8B	112.3658	0.060369	0.055692	1.083979	0.278374	NA
MIR218-2	1.663476	0.060351	0.065689	0.918743	0.35823	NA
NAGPA-AS	11.64508	0.060348	0.084887	0.710925	0.477131	NA
UBXN1	232.5585	-0.06032	0.058293	-1.03482	0.300752	NA
DRAXIN	5.465669	0.060309	0.068074	0.885929	0.375656	NA
PWRN1	1.075885	0.060308	0.050883	1.185227	0.235928	NA
CAMSAP1	596.1575	0.060301	0.038193	1.578839	0.114373	0.465042
MRPL36	12.10754	0.060296	0.084089	0.717055	0.47334	NA
BCDIN3D-	12.47616	0.060293	0.084686	0.711957	0.476492	NA
SDK2	12.60885	0.060292	0.084651	0.712243	0.476315	NA
PCF11	932.3786	0.060279	0.044895	1.342662	0.179382	0.53091
NOS2	7.00519	-0.06027	0.077311	-0.77952	0.435671	NA
INF2	53.07821	-0.06026	0.079313	-0.75977	0.447389	NA
RPL6	321.6345	-0.06025	0.060077	-1.00281	0.315954	NA
OTUD7B	335.209	-0.06023	0.050747	-1.18692	0.235259	0.58613
CTDSP2	648.2055	-0.06023	0.056325	-1.06937	0.284903	0.619296
ZSCAN12	146.8065	0.060228	0.045454	1.325036	0.185159	NA
DPPA5	0.387583	-0.06023	0.036632	-1.6441	0.100156	NA
DFNB31	67.49568	-0.06021	0.084696	-0.71095	0.477118	NA
COA5	122.8183	-0.0602	0.046073	-1.30654	0.191368	NA
SNORD116	79.22999	-0.06018	0.084932	-0.70861	0.478566	NA
DLEU7	7.004067	0.060184	0.084499	0.712238	0.476317	NA
PARP2	159.8995	-0.06018	0.065744	-0.91538	0.35999	NA
LOC10050	3.96673	-0.06018	0.080974	-0.7432	0.457358	NA
TNFSF13	5.715434	-0.06018	0.083833	-0.71786	0.472845	NA
CNTNAP4	411.7747	0.060174	0.078621	0.765373	0.44405	0.743291
NSG1	295.0886	-0.06016	0.077438	-0.77693	0.437201	NA
UGDH	70.22341	0.060162	0.058863	1.022067	0.306749	NA
SNORD114	14.93468	-0.06013	0.083579	-0.71942	0.471882	NA
FGFBP3	10.76247	0.060117	0.084867	0.708372	0.478714	NA
EVI2A	65.29615	-0.0601	0.072187	-0.83262	0.405061	NA
TUBGCP5	96.84758	-0.06009	0.077816	-0.77227	0.439957	NA
DUS1L	80.62249	-0.06008	0.056605	-1.06141	0.288505	NA
PCID2	138.7089	-0.06007	0.056445	-1.06429	0.287196	NA
SLC25A23	254.0901	0.060068	0.052688	1.140062	0.254261	NA
ALG11	26.27198	0.060051	0.076263	0.787427	0.431032	NA
LOC55422	0.621565	-0.06004	0.044959	-1.33544	0.181733	NA
CAPN14	5.414666	-0.06003	0.083506	-0.7189	0.472205	NA
IFFO2	319.5578	0.059996	0.055374	1.083471	0.278599	NA
ANGPT2	69.09524	-0.05999	0.084747	-0.70784	0.479044	NA
COPS2	352.1148	0.059986	0.050404	1.190098	0.234008	0.58502
GPN3	29.00303	-0.05998	0.07251	-0.82726	0.408091	NA
ASCL5	1.289988	-0.05998	0.049759	-1.2055	0.228009	NA
CLN5	97.78883	-0.05998	0.061664	-0.97275	0.330678	NA
MATR3	2016.65	0.059982	0.03832	1.565303	0.117512	0.472599
USP48	621.6522	-0.05998	0.039603	-1.51454	0.12989	0.487086
TLK2	186.3523	-0.05996	0.045926	-1.30564	0.191674	NA

ABCD1	9.112449	-0.05996	0.08478	-0.7072	0.479443	NA
SGSM1	314.6986	0.059954	0.060858	0.985149	0.324551	NA
DPH2	18.24576	0.059954	0.080476	0.744993	0.456276	NA
NUP62CL	1.279246	-0.05995	0.059193	-1.01274	0.311187	NA
KCNK9	174.9609	0.059921	0.061141	0.980061	0.327056	NA
EIF4ENIF1	261.6805	0.059917	0.041735	1.435672	0.151096	NA
HOXD-AS1	5.76413	0.059915	0.07814	0.76676	0.443224	NA
SRSF2	152.675	-0.05991	0.056282	-1.06447	0.287114	NA
CCNK	117.5551	-0.05991	0.054461	-1.09998	0.271339	NA
C3orf52	1.199597	0.059904	0.047505	1.261002	0.207308	NA
DGKZ	330.5836	-0.0599	0.05657	-1.05894	0.289628	NA
TIAM2	162.1943	0.059898	0.055063	1.08781	0.276679	NA
PLN	25.53891	0.059895	0.078306	0.764889	0.444338	NA
CPNE2	42.81207	-0.05989	0.075948	-0.78861	0.430343	NA
ANKS1A	389.3058	-0.05989	0.038721	-1.5467	0.121935	0.476542
NLGN3	157.4529	0.059888	0.064826	0.923834	0.355573	NA
TMEM25	176.5591	0.059888	0.042297	1.415883	0.15681	NA
MAP3K13	235.4005	0.059877	0.045813	1.306981	0.191219	NA
LYRM4	26.70841	0.059866	0.074338	0.805319	0.420635	NA
SLC24A6	42.22203	-0.05985	0.073093	-0.81882	0.412889	NA
CWC25	105.9283	-0.05985	0.04982	-1.20127	0.229647	NA
AKR1C1	10.15413	-0.05984	0.082957	-0.72134	0.470702	NA
USP32P1	208.1625	-0.05984	0.080832	-0.74024	0.459152	NA
MORN5	1.112451	0.059817	0.054417	1.099233	0.271666	NA
LSM1	41.8098	-0.05981	0.07044	-0.84913	0.395809	NA
PIP4K2B	548.1077	0.059812	0.040214	1.487332	0.136927	0.489381
ADCY8	85.43975	0.059811	0.072424	0.825842	0.408894	NA
RNF31	134.1354	-0.05981	0.049412	-1.21038	0.226132	NA
PLEKHA8P	1.35911	-0.05981	0.060244	-0.99275	0.320833	NA
NIPBL	1391.592	0.059805	0.036579	1.634941	0.102061	0.443332
GOT2	229.4507	0.059789	0.064663	0.924615	0.355166	NA
HCRTR2	0.417452	0.059788	0.035975	1.661927	0.096527	NA
ADCK5	25.84884	0.059785	0.077626	0.770166	0.441201	NA
POLG2	37.87352	-0.05977	0.075226	-0.79457	0.426866	NA
EHD4	16.59258	0.059771	0.084128	0.710471	0.477412	NA
LOC72905	1.122793	0.059769	0.05554	1.076147	0.281861	NA
RGMB	166.6742	0.059759	0.058931	1.014036	0.310566	NA
MIR491	1.909269	0.059752	0.06943	0.8606	0.389458	NA
SNORA61	8.188483	-0.05974	0.084816	-0.70438	0.481197	NA
FAM86C1	10.73833	-0.05974	0.084807	-0.70445	0.481153	NA
UCN2	1.175827	-0.05974	0.057121	-1.04586	0.295624	NA
ACVR1B	152.5927	0.059724	0.055018	1.085546	0.27768	NA
ZNF660	70.88863	0.059714	0.072647	0.821975	0.411091	NA
TMC8	5.192676	-0.05971	0.079172	-0.7542	0.450728	NA
LRRC6	41.54034	-0.0597	0.07504	-0.79563	0.426248	NA
EEF1A2	975.9244	0.0597	0.06733	0.886679	0.375252	0.695376
PDLIM1	7.792264	-0.05969	0.068838	-0.86707	0.385901	NA

MIR4499	0.866597	-0.05967	0.050004	-1.19335	0.232731	NA
PSMD14	80.88023	-0.05964	0.073253	-0.81416	0.415552	NA
GPR83	103.7027	-0.05963	0.078543	-0.75919	0.447741	NA
MRPS24	1.507694	0.059627	0.059194	1.007309	0.313786	NA
RALGAPA1	397.8366	0.059623	0.042937	1.388618	0.164949	0.52098
SLX4IP	24.27658	0.059613	0.081166	0.734459	0.462669	NA
GGT5	12.44484	-0.05961	0.084877	-0.70225	0.482521	NA
CASD1	212.5307	0.059595	0.058518	1.018416	0.30848	NA
PDHX	151.5811	0.059593	0.04989	1.194499	0.232283	NA
RARB	39.04558	0.05958	0.078206	0.761824	0.446165	NA
ANO5	29.88679	0.059575	0.080586	0.739271	0.459743	NA
ARHGEF2	490.3016	-0.05957	0.039695	-1.50071	0.133429	0.487416
TRIM33	629.715	0.059561	0.048655	1.224158	0.220893	0.569906
ACOT13	122.0821	-0.05956	0.054765	-1.0875	0.276817	NA
COIL	110.1806	-0.05955	0.053735	-1.10825	0.267754	NA
CSMD1	244.7575	-0.05955	0.078281	-0.76072	0.446825	NA
CERS3	4.493181	-0.05954	0.05866	-1.01509	0.310061	NA
DPAGT1	17.12093	0.05954	0.079552	0.748448	0.45419	NA
MEAF6	189.2944	0.059538	0.057808	1.029924	0.303045	NA
DLG1	1185.616	-0.05952	0.041318	-1.44057	0.149706	0.502511
WDR55	46.43652	-0.05952	0.063211	-0.9416	0.346395	NA
SPATA2	96.25017	0.059515	0.070648	0.84242	0.399553	NA
LETM1	263.0518	0.059512	0.048724	1.221411	0.22193	NA
TFDP2	273.3766	-0.05951	0.04713	-1.26269	0.206702	NA
NCEH1	104.1625	0.05949	0.065948	0.902083	0.367013	NA
ALG3	24.14196	0.059488	0.078978	0.753221	0.451317	NA
FSHB	0.665625	0.059487	0.040719	1.460928	0.144035	NA
ANXA5	87.33341	-0.05948	0.0799	-0.74442	0.456622	NA
KIAA1614	181.1767	-0.05947	0.063893	-0.93085	0.351932	NA
TRDMT1	137.9332	-0.05947	0.067931	-0.87549	0.381306	NA
SLC5A8	8.788682	0.059472	0.061013	0.974733	0.329693	NA
SNORD116	381.2217	-0.05947	0.084765	-0.70157	0.482947	0.764934
MIR5093	1.311663	0.059468	0.055312	1.07514	0.282312	NA
VPS33B	104.2247	-0.05946	0.056378	-1.05462	0.2916	NA
AOAH-IT1	1.061422	0.059452	0.053348	1.114421	0.265099	NA
MLIP	3.177474	-0.05944	0.071365	-0.83292	0.404891	NA
CP	107.4453	-0.05943	0.074549	-0.79723	0.425319	NA
PPP2R2A	191.8927	0.059378	0.03907	1.519777	0.128567	NA
SNORD114	10.55535	-0.05937	0.084316	-0.70415	0.481342	NA
ZNF664	665.1356	0.059365	0.044237	1.341972	0.179605	0.53091
GPX7	8.553642	-0.05936	0.084939	-0.69886	0.484638	NA
POLQ	60.70743	0.059357	0.075445	0.786757	0.431424	NA
EEF1D	410.2273	-0.05935	0.046513	-1.27602	0.201949	0.549117
SPIB	0.459707	-0.05935	0.038238	-1.55206	0.120649	NA
BDP1	2296.982	0.059343	0.03344	1.774602	0.075964	0.391931
RPL21P44	34.46358	-0.05931	0.076601	-0.77427	0.438771	NA
S1PR2	2.722217	0.059308	0.071283	0.832009	0.405404	NA

MIR103A1	0.287598	-0.0593	0.031186	-1.90163	0.05722	NA
SWSAP1	13.33075	0.059304	0.084395	0.702699	0.482243	NA
COX10	57.76752	0.059291	0.067536	0.877925	0.379985	NA
WDR70	178.0916	-0.05929	0.043496	-1.36311	0.172848	NA
SHMT2	69.13624	-0.05929	0.061526	-0.96361	0.335243	NA
KLHDC8B	15.20481	0.059269	0.083153	0.71277	0.475988	NA
ANKRD34A	86.05061	0.059247	0.064188	0.923015	0.355999	NA
AIMP1	91.07565	-0.05925	0.061772	-0.95908	0.337516	NA
RNF2	72.39372	0.059245	0.068397	0.866183	0.38639	NA
SLC25A25	92.22116	0.059244	0.05868	1.009607	0.312683	NA
SLC9A7P1	4.908157	0.059242	0.080488	0.736031	0.461712	NA
ATG7	95.58801	-0.05924	0.051571	-1.14863	0.250708	NA
FAM57B	70.92283	0.059236	0.059471	0.99605	0.319226	NA
SRRM4	1328.55	-0.05922	0.045192	-1.31036	0.190073	0.54182
MYNN	175.3382	0.059213	0.045961	1.28831	0.197638	NA
PIGH	36.11471	0.05921	0.072154	0.820616	0.411865	NA
ICAM2	2.693269	0.059199	0.071999	0.822218	0.410953	NA
ERN2	1.023729	-0.0592	0.055684	-1.06311	0.287732	NA
CCDC25	119.8481	-0.05919	0.056887	-1.04051	0.298104	NA
TMEM27	1.049299	0.059187	0.050654	1.168459	0.242622	NA
GLUD1P7	1.144538	-0.05918	0.054336	-1.08912	0.276101	NA
MESP2	0.975059	-0.05918	0.048409	-1.22244	0.22154	NA
CATSPER1	4.44529	-0.05918	0.079381	-0.74546	0.455992	NA
TMEM221	11.15771	0.059174	0.084819	0.69765	0.485396	NA
CCDC144B	575.1858	0.059172	0.081451	0.72648	0.467545	0.755844
LINC00441	3.051613	0.059169	0.075013	0.788785	0.430238	NA
MAML3	422.5231	-0.05917	0.051651	-1.14549	0.252006	0.592704
ZNF200	37.42725	-0.05916	0.066767	-0.88611	0.375559	NA
PSME3	158.6658	0.05916	0.054151	1.092509	0.27461	NA
TFDP1	165.1054	0.059154	0.050409	1.173493	0.240598	NA
MYPOP	56.27404	-0.05915	0.062996	-0.93892	0.347774	NA
LINC00654	15.09776	-0.05915	0.083201	-0.7109	0.477147	NA
SAMD12	330.2113	0.059145	0.050414	1.17318	0.240724	NA
SLC38A10	133.2887	-0.05914	0.058597	-1.00929	0.312834	NA
CNTD2	28.16342	-0.05914	0.078824	-0.75022	0.453124	NA
LINC00629	1.446631	-0.05913	0.059159	-0.99959	0.31751	NA
COMMD6	24.02823	0.059133	0.082197	0.71941	0.471889	NA
ANKRD26P	0.883379	0.059133	0.047267	1.251051	0.210916	NA
EFCAB6-AS	0.710467	-0.05912	0.047308	-1.24966	0.211425	NA
LINC00493	22.46071	-0.05911	0.083701	-0.70616	0.480089	NA
ELL2	176.9689	-0.0591	0.064009	-0.92328	0.355864	NA
ANKRD30B	96.33612	-0.05909	0.080789	-0.73136	0.464561	NA
RASGRF1	1237.018	0.059082	0.040468	1.459976	0.144297	0.495647
RBPMS2	3.379431	0.059079	0.078037	0.757059	0.449015	NA
C7orf60	113.3318	-0.05906	0.056369	-1.0478	0.294733	NA
IL1RL2	0.485311	0.059057	0.036775	1.605895	0.108297	NA
SOX10	76.4751	-0.05906	0.074289	-0.79495	0.42664	NA

BBS5	41.66272	-0.05905	0.073667	-0.80151	0.422834	NA
PPP1R1C	42.749	0.059029	0.082928	0.711818	0.476577	NA
RIPK2	15.82511	0.05902	0.084691	0.696892	0.48587	NA
HIF1A-AS2	5.643029	-0.05902	0.082705	-0.71357	0.475493	NA
CEACAM2	1.451087	0.059014	0.060161	0.980943	0.326621	NA
KCTD3	436.295	-0.05901	0.044189	-1.33544	0.181734	0.530978
PIK3R6	1.578017	0.059011	0.062586	0.942871	0.345747	NA
UNC119B	120.9246	0.05901	0.046482	1.269526	0.204253	NA
MRPL43	62.61129	-0.05901	0.064209	-0.91899	0.358101	NA
CUEDC2	98.83353	-0.059	0.072782	-0.81069	0.417544	NA
MRPS15	70.27062	-0.05899	0.059292	-0.99486	0.319806	NA
CDCA7	1.493474	0.058984	0.061545	0.958383	0.33787	NA
ASIC1	145.3607	0.058982	0.065277	0.903574	0.366221	NA
STARD10	95.06728	-0.05897	0.073855	-0.79843	0.424623	NA
PKN3	60.63452	0.058966	0.081766	0.721157	0.470813	NA
PPP2R5E	206.5903	0.05896	0.043796	1.346255	0.17822	NA
FAM211A	33.91788	0.058956	0.080816	0.729516	0.465686	NA
COL4A3BP	328.5357	0.058955	0.038896	1.515723	0.12959	NA
PINX1	34.89855	-0.05895	0.069248	-0.85124	0.394636	NA
U2SURP	833.0979	0.058942	0.054741	1.076727	0.281602	0.616856
CYBASC3	18.60466	-0.05892	0.080884	-0.72842	0.466356	NA
LOC15257	1.503014	-0.0589	0.059563	-0.98885	0.322736	NA
ISCA2	25.73216	0.058896	0.071581	0.82279	0.410627	NA
UPRT	58.14718	0.058895	0.059342	0.992467	0.32097	NA
PHLDB1	592.0052	-0.05889	0.056367	-1.04484	0.296098	0.62855
SYP	578.992	0.058893	0.065697	0.896436	0.37002	0.694363
ZDHHC5	159.1486	0.058887	0.048601	1.211632	0.225653	NA
CDK11B	34.4698	0.05888	0.077367	0.761055	0.446624	NA
C20orf166	0.213455	-0.05887	0.024801	-2.37373	0.017609	NA
MFN2	409.9429	0.058868	0.054289	1.084333	0.278217	0.614792
SNORD77	0.827906	0.058867	0.048044	1.225259	0.220478	NA
KCTD14	0.594082	-0.05885	0.043153	-1.36387	0.172607	NA
TRIM43	0.610033	0.058849	0.040234	1.462665	0.143559	NA
RAF1	402.9626	0.058832	0.046025	1.278257	0.201159	0.547633
ZMAT1	167.6521	-0.05882	0.0812	-0.72445	0.468792	NA
MKL2	232.7669	0.058824	0.052768	1.114774	0.264947	NA
LOC72817	0.954326	-0.05881	0.049392	-1.19065	0.233791	NA
LOC10050	5.384868	-0.0588	0.078464	-0.74934	0.453654	NA
NHP2	39.3271	0.058793	0.065398	0.898997	0.368654	NA
SHISA9	9.603624	-0.05879	0.083211	-0.70648	0.47989	NA
MIR573	9.36985	-0.05877	0.084939	-0.69193	0.488982	NA
TCEAL1	98.24242	-0.05877	0.069375	-0.84707	0.396954	NA
TTBK1	379.1832	0.058759	0.040371	1.455479	0.145537	0.497196
GBAS	132.4052	-0.05876	0.066193	-0.88769	0.374705	NA
CHD2	1700.579	-0.05875	0.037612	-1.56211	0.118261	0.472599
ANKRD62P	0.933421	0.058748	0.052217	1.125074	0.260558	NA
TRIM13	349.2278	0.058743	0.051282	1.145477	0.252012	0.592704

KCNJ13	62.99861	-0.05874	0.069074	-0.85041	0.395097	NA
QSOX1	54.56935	-0.05873	0.068405	-0.85849	0.39062	NA
ACTL7B	0.245626	-0.05872	0.029441	-1.99443	0.046105	NA
PCDHB3	27.9922	-0.0587	0.07737	-0.75871	0.448026	NA
SENP1	89.74501	0.058697	0.053723	1.092582	0.274577	NA
SETX	1047.574	0.058693	0.025821	2.273107	0.02302	0.23963
MRS2P2	10.87889	-0.05869	0.083858	-0.69984	0.484028	NA
JAKMIP2-A	18.77638	-0.05868	0.079693	-0.73628	0.461558	NA
DIS3L	169.7059	0.058669	0.049661	1.181397	0.237445	NA
NR2C2	680.7411	0.058653	0.032544	1.802231	0.071509	0.38806
CCDC164	3.104827	-0.05864	0.07174	-0.81742	0.413687	NA
SCN8A	1423.988	0.058639	0.040627	1.443344	0.148923	0.502511
FAM43A	27.16218	0.058637	0.080678	0.726802	0.467347	NA
RFC1	182.3923	0.058629	0.04956	1.182988	0.236814	NA
NPAS4	3.390862	0.058629	0.057263	1.023858	0.305903	NA
MAN2B2	52.80867	-0.05863	0.067819	-0.86445	0.387341	NA
PMS2P4	31.28811	-0.05862	0.073217	-0.80063	0.423349	NA
TMEM187	9.007143	-0.05861	0.084938	-0.69005	0.490163	NA
EIF1	296.644	0.058596	0.061441	0.953695	0.340238	NA
C16orf91	10.32256	-0.05859	0.084795	-0.69097	0.489586	NA
SEMA4B	108.9605	-0.05859	0.055992	-1.04637	0.29539	NA
MSI1	15.54127	0.058587	0.082289	0.711972	0.476482	NA
ST6GALNA	215.7069	-0.05858	0.045091	-1.29912	0.193904	NA
NR2C1	345.42	-0.05857	0.048651	-1.20395	0.228609	0.577536
IL1RAPL2	2.846463	-0.05855	0.069328	-0.8446	0.398333	NA
DPYD-AS1	0.323334	-0.05855	0.032574	-1.79749	0.072258	NA
DIABLO	114.7114	-0.05855	0.049288	-1.18794	0.234856	NA
CYP27C1	0.34648	-0.05855	0.031785	-1.84207	0.065465	NA
DET1	28.8113	-0.05855	0.07479	-0.78285	0.433717	NA
CTAGE5	135.2161	0.058547	0.048469	1.207926	0.227076	NA
USP12	268.5582	0.058539	0.047506	1.232242	0.217859	NA
SNAP25	5855.152	0.058538	0.075233	0.778088	0.436517	0.738506
MOB3A	95.88001	0.058538	0.061437	0.952817	0.340683	NA
ADAM22	2310.632	0.058532	0.050353	1.162448	0.245053	0.592704
KIF3A	697.6669	0.058514	0.030403	1.924634	0.054275	0.357364
HAUS8	16.57377	-0.0585	0.081369	-0.71892	0.472189	NA
SCGB2B2	1.211808	-0.05849	0.05966	-0.98031	0.326931	NA
IGSF1	11.81144	-0.05847	0.08214	-0.71186	0.476549	NA
TFEB	50.39997	-0.05847	0.076505	-0.76428	0.444699	NA
NFU1	62.23965	-0.05846	0.069373	-0.84262	0.399442	NA
RLIM	458.4189	0.058447	0.047746	1.224116	0.220908	0.569906
AKD1	134.272	-0.05844	0.057099	-1.02347	0.306085	NA
LOC44145	24.37671	0.058425	0.083482	0.699848	0.484022	NA
PRDM1	74.39144	0.058424	0.083142	0.70271	0.482236	NA
PROK2	0.652858	-0.05842	0.044555	-1.31121	0.189788	NA
H2BFXP	0.591193	-0.05841	0.04275	-1.3664	0.171814	NA
LAGE3	15.30966	0.058411	0.084566	0.690711	0.489747	NA

ZNF394	122.4769	-0.05841	0.063918	-0.91379	0.360828	NA
SEC31B	310.4625	-0.0584	0.06842	-0.85355	0.393354	NA
MYEF2	550.8732	0.058388	0.042874	1.361845	0.173247	0.526011
C9orf129	26.85665	-0.05839	0.069611	-0.83875	0.401612	NA
MIR4526	1.445829	0.058382	0.059873	0.975105	0.329508	NA
CDC42EP3	17.73025	-0.05836	0.083818	-0.69623	0.486284	NA
KLHL36	64.21074	0.058348	0.064405	0.905959	0.364957	NA
CENPC1	532.1027	-0.05834	0.06683	-0.87301	0.382658	0.70023
PABPC5	13.62532	-0.05833	0.084861	-0.68735	0.491861	NA
HAT1	87.82045	0.058323	0.052508	1.110745	0.266678	NA
MCOLN3	0.262363	-0.05832	0.028953	-2.01419	0.04399	NA
ATP5E	16.82471	0.058314	0.084352	0.691315	0.489368	NA
TMEM39B	18.56499	0.058307	0.077908	0.748409	0.454214	NA
EBP	33.72509	-0.0583	0.070254	-0.82991	0.406589	NA
ASPM	1.909571	-0.0583	0.057817	-1.00832	0.313303	NA
MEX3C	134.8717	0.058293	0.05283	1.103414	0.269848	NA
FAIM	44.90178	-0.05828	0.069993	-0.83264	0.405046	NA
WNT2	2.210901	-0.05828	0.060347	-0.96573	0.334177	NA
DBNDD2	18.85891	-0.05827	0.081145	-0.71804	0.472734	NA
YTHDF2	121.8623	0.058265	0.042996	1.355108	0.175383	NA
AK7	4.1924	-0.05825	0.07938	-0.73387	0.46303	NA
TRIM39	45.88135	-0.05825	0.064006	-0.91007	0.362785	NA
CPSF4	80.75647	-0.05824	0.060363	-0.9648	0.334644	NA
EXOSC7	54.83779	0.058227	0.066441	0.876374	0.380827	NA
OCEL1	21.16367	0.058219	0.080482	0.723382	0.469445	NA
FAM8A1	213.9734	0.058211	0.055515	1.048555	0.294383	NA
FOXL1	1.24596	-0.05821	0.053825	-1.08145	0.279497	NA
SERP1	144.4633	-0.05821	0.057435	-1.01346	0.310841	NA
MIR421	12.78674	-0.05821	0.084456	-0.68918	0.49071	NA
KDM2B	337.9519	-0.05819	0.044704	-1.30162	0.193046	0.542492
LOC28576	0.761273	-0.05818	0.048019	-1.21169	0.225631	NA
CRYBB3	0.596864	0.058183	0.043688	1.331761	0.182939	NA
IL12B	3.069054	0.05817	0.068117	0.853973	0.39312	NA
EIF3F	76.61619	-0.05817	0.063155	-0.92103	0.357032	NA
AMACR	4.147844	0.058161	0.080796	0.719851	0.471617	NA
UBR1	536.2921	0.05816	0.032683	1.779523	0.075154	0.391143
MKKS	112.3243	0.058144	0.06015	0.966641	0.333723	NA
FOXK1	352.8442	-0.05814	0.045483	-1.27825	0.201161	0.547633
C9orf172	350.8895	0.058126	0.059914	0.970155	0.331969	0.660832
CUTC	19.91405	-0.05812	0.076705	-0.75765	0.44866	NA
LINC00599	1798.75	-0.05811	0.047473	-1.22409	0.220917	0.569906
SARS2	31.34779	-0.05811	0.072044	-0.80661	0.419891	NA
DSCC1	28.50464	0.05811	0.080263	0.723998	0.469067	NA
GCK	18.60401	0.058102	0.084568	0.687047	0.492053	NA
SNORA44	29.06903	-0.05808	0.084141	-0.69027	0.490025	NA
DCHS1	97.89527	-0.05808	0.078858	-0.73651	0.461422	NA
CAPRIN2	654.7327	-0.05808	0.052792	-1.10014	0.271272	0.612593

L2HGDH	104.9825	-0.05807	0.053903	-1.07738	0.281309	NA
SNORA71D	5.617768	-0.05806	0.072521	-0.80063	0.423345	NA
MRPS23	66.87459	-0.05806	0.067485	-0.86033	0.389605	NA
DNTTIP1	42.74704	-0.05804	0.068894	-0.84249	0.399511	NA
POLE	803.7392	0.058037	0.083412	0.695796	0.486557	0.764934
CHCHD10	99.61614	-0.05801	0.073727	-0.78688	0.431353	NA
P4HA1	158.1804	-0.05801	0.079011	-0.73414	0.462865	NA
SEC22C	157.8211	0.058001	0.05653	1.026024	0.30488	NA
MRPL14	15.39627	0.058001	0.081778	0.709246	0.478172	NA
MIR644A	1.434135	0.057998	0.061715	0.939766	0.347338	NA
ZNF768	65.48246	0.057991	0.065135	0.890331	0.373288	NA
SLC7A2	51.1802	-0.05799	0.084734	-0.68434	0.493758	NA
ABCA11P	33.87833	0.057973	0.080883	0.716747	0.47353	NA
CCDC37	5.067257	-0.05797	0.080923	-0.71639	0.473751	NA
CSE1L	336.8632	0.057971	0.047561	1.218873	0.222892	0.572393
ACSBG1	57.97329	-0.05795	0.078586	-0.73739	0.460885	NA
SLC14A2	5.20962	-0.05794	0.077042	-0.75212	0.451982	NA
C16orf92	3.794074	0.057936	0.077086	0.751573	0.452308	NA
KHDC1L	0.928745	0.057934	0.049549	1.169231	0.24231	NA
THEM4	66.95302	-0.05792	0.074906	-0.77329	0.439351	NA
G2E3	97.18977	-0.05792	0.051659	-1.12116	0.262221	NA
SNORD60	11.71497	-0.05792	0.082566	-0.70147	0.483011	NA
FAM110C	1.757537	-0.05791	0.062749	-0.92283	0.356095	NA
ZNF812	1.655215	0.057891	0.059848	0.967304	0.333392	NA
PLA2G6	78.9236	-0.05789	0.066859	-0.86586	0.386565	NA
C5orf47	0.423117	-0.05789	0.036498	-1.58606	0.112726	NA
LOC28344	8.370003	0.057883	0.084787	0.68269	0.494802	NA
FAM114A2	39.49164	-0.05788	0.070004	-0.82682	0.408341	NA
ARHGAP35	907.9039	0.057854	0.044934	1.287513	0.197915	0.545847
KCNQ2	496.5663	0.057848	0.052091	1.110519	0.266775	0.607287
FDX1L	27.76263	0.05784	0.072892	0.793498	0.427487	NA
MTRF1L	81.86839	0.057826	0.062643	0.923111	0.355949	NA
TP73-AS1	119.5123	0.057826	0.063988	0.903698	0.366155	NA
SLC38A4	2.061596	0.057825	0.062429	0.926248	0.354317	NA
PRPH	2.939684	0.057817	0.0599	0.965236	0.334427	NA
SHFM1	136.044	-0.05781	0.051182	-1.12955	0.258664	NA
FOCAD	296.032	-0.05781	0.056341	-1.02608	0.304856	NA
SOX17	2.182434	0.057809	0.070038	0.825386	0.409152	NA
ZNF512B	199.289	-0.0578	0.066954	-0.86331	0.387967	NA
GLI1	24.1072	-0.05779	0.078275	-0.73835	0.460304	NA
LMO3	85.31492	-0.05776	0.074674	-0.77353	0.439209	NA
PIGQ	60.18272	-0.05776	0.061163	-0.94439	0.344972	NA
SPIN1	367.526	0.057748	0.04629	1.247535	0.212201	0.562782
CDC25C	0.804944	-0.05775	0.047517	-1.21529	0.224255	NA
ATP5SL	31.29729	-0.05774	0.06984	-0.82678	0.408362	NA
FKSG29	3.506302	0.057739	0.079395	0.727238	0.46708	NA
SOS2	347.4697	0.057732	0.039637	1.45652	0.145249	0.496962

ZNF827	256.8366	0.057731	0.042741	1.350736	0.17678	NA
LOX	15.79357	0.057721	0.083519	0.691107	0.489498	NA
BCORL1	148.7888	0.057714	0.062772	0.919427	0.357872	NA
LINC00485	0.221947	-0.05771	0.027522	-2.09686	0.036006	NA
RP1-177G	1377.453	-0.05771	0.072085	-0.80057	0.423381	0.727586
CCND3	55.5621	-0.0577	0.061002	-0.94595	0.344175	NA
SKI	1757.13	0.0577	0.048664	1.185687	0.235746	0.58613
BST2	14.69082	0.05769	0.08458	0.682073	0.495193	NA
METTL3	222.8854	-0.05769	0.052045	-1.1084	0.267691	NA
HSPA9	544.7498	0.057678	0.045058	1.280094	0.200512	0.547633
NRIP3	176.8356	0.057669	0.076637	0.752492	0.451755	NA
TMEM92	1.191762	-0.05767	0.039532	-1.45871	0.144646	NA
SERBP1	414.6283	0.057665	0.047076	1.224918	0.220606	0.569906
TLR7	2.911066	-0.05766	0.066804	-0.86317	0.388044	NA
LOC10019	0.812631	-0.05766	0.04867	-1.18465	0.236156	NA
EMR4P	1.007514	-0.05765	0.052617	-1.09574	0.273194	NA
TRMT5	103.4488	0.057653	0.052006	1.108591	0.267607	NA
ALKBH7	41.88117	0.057651	0.069475	0.82981	0.406646	NA
ZCWPW1	92.01311	-0.05764	0.057321	-1.00555	0.314633	NA
PIKFYVE	588.9629	0.057638	0.029456	1.95671	0.050382	0.34688
FAXC	361.6323	0.057631	0.04964	1.16099	0.245646	0.592704
RESP18	0.335818	-0.05763	0.034549	-1.66803	0.095311	NA
THRA	775.3727	-0.05763	0.041499	-1.38859	0.164957	0.52098
MIR3648	423.0644	-0.05762	0.079512	-0.72469	0.468643	0.756038
NAV2	607.3804	-0.05761	0.0647	-0.89039	0.373256	0.695376
ARHGEF40	196.6292	0.057598	0.05715	1.007853	0.313525	NA
NLRP2	6.440042	-0.05759	0.072548	-0.79385	0.427282	NA
ZRSR2	229.5496	0.057584	0.072145	0.79817	0.424772	NA
ATP2C2	15.19399	0.05758	0.084212	0.683747	0.494135	NA
SNORD116	607.8315	-0.05758	0.08278	-0.69557	0.4867	0.764934
CSNK1A1	564.7875	0.057577	0.041831	1.376435	0.168687	0.522164
CBX1	214.4683	0.057555	0.054399	1.05801	0.290051	NA
CELF2-AS2	78.68762	-0.05754	0.067407	-0.85366	0.393293	NA
MT1JP	0.410975	0.057536	0.034636	1.661168	0.09668	NA
MRPL21	47.76036	-0.05754	0.077587	-0.74156	0.458351	NA
CDC40	118.7488	-0.05751	0.042972	-1.33833	0.18079	NA
PLEKHM3	311.3554	-0.05751	0.034379	-1.67279	0.094368	NA
C14orf169	54.4284	0.057508	0.064318	0.894129	0.371253	NA
MYADML2	5.633993	0.057507	0.078015	0.737129	0.461044	NA
SMNDC1	113.483	0.057502	0.047977	1.198538	0.230708	NA
EXOSC10	549.8686	-0.05749	0.052936	-1.08606	0.277453	0.614792
SMARCAL1	44.67109	0.057481	0.064247	0.894691	0.370952	NA
TUBB3	119.9705	0.057475	0.072973	0.787621	0.430918	NA
ECHDC3	8.305802	-0.05747	0.084252	-0.68215	0.495145	NA
MMS19	337.8842	-0.05747	0.047003	-1.22274	0.221429	0.570018
KRTAP5-5	0.414432	-0.05747	0.031872	-1.80306	0.071379	NA
PKHD1L1	2.125315	-0.05747	0.057372	-1.00164	0.31652	NA

USP33	682.3341	-0.05746	0.04649	-1.23591	0.216493	0.566887
BMPR1B	133.7637	-0.05745	0.075277	-0.76323	0.445328	NA
RNF212	38.9692	-0.05745	0.084241	-0.682	0.49524	NA
DSE	35.31619	0.057446	0.074416	0.771957	0.44014	NA
CEP170	749.9632	0.05744	0.032787	1.751889	0.079793	0.397742
CPLX2	1943.272	-0.05741	0.082234	-0.69815	0.485083	0.764934
LOC10028	11.4469	-0.0574	0.080468	-0.71338	0.475613	NA
RPL23AP8	15.6369	0.057402	0.083696	0.68584	0.492814	NA
PRRT1	167.8727	0.057398	0.0577	0.994758	0.319854	NA
FBXW11	237.5064	0.057397	0.051478	1.114972	0.264862	NA
CEP135	91.82645	0.057392	0.058821	0.975707	0.32921	NA
SCEL	1.218285	-0.05738	0.054591	-1.05118	0.293174	NA
MSL3P1	1.1904	-0.05737	0.058643	-0.97836	0.327899	NA
APLP2	2208.374	0.057367	0.056276	1.019375	0.308025	0.638314
MEIG1	1.41278	-0.05736	0.062735	-0.91437	0.360525	NA
BRD7P3	31.63953	0.057359	0.07005	0.818837	0.412879	NA
MRPL9	41.55983	0.057357	0.07157	0.801409	0.422895	NA
TCF12	270.2975	-0.05734	0.043457	-1.31949	0.187004	NA
DANCR	30.18092	0.057334	0.073199	0.783264	0.433472	NA
SHQ1	34.3123	-0.05731	0.066819	-0.85773	0.39104	NA
ACOT9	44.26097	-0.05731	0.064846	-0.88374	0.376836	NA
GPR132	4.072696	-0.0573	0.078635	-0.72867	0.466202	NA
RND3	15.34129	0.057297	0.084722	0.676287	0.498858	NA
ADARB1	822.0578	0.057287	0.04678	1.224591	0.22073	0.569906
MIR4736	1.157272	-0.05728	0.05563	-1.02972	0.30314	NA
PER3	1273.476	0.05727	0.048808	1.173376	0.240645	0.589893
C9orf116	1.974127	-0.05726	0.06931	-0.8261	0.408748	NA
MAP2K5	155.7102	-0.05725	0.048115	-1.1899	0.234085	NA
GDPD2	5.169635	-0.05725	0.082457	-0.69431	0.487488	NA
DAZAP2	171.1829	-0.05723	0.071373	-0.80182	0.42266	NA
RADIL	131.5847	-0.05723	0.056699	-1.0093	0.312831	NA
IQCD	7.82828	0.057224	0.084904	0.673986	0.50032	NA
NOB1	78.14521	0.05722	0.055924	1.023179	0.306223	NA
CTTN	627.1052	-0.05721	0.042367	-1.35026	0.176932	0.529395
MYBPHL	0.517569	-0.05719	0.038469	-1.48663	0.137113	NA
CLVS2	472.2419	0.057187	0.055738	1.025994	0.304895	0.635668
TUFT1	46.3575	-0.05717	0.069399	-0.82377	0.410073	NA
NBEAL1	375.4063	0.057166	0.041388	1.381214	0.167213	0.521454
SLC27A3	51.43412	-0.05716	0.073399	-0.77878	0.436111	NA
HPCAL1	426.8228	0.057157	0.061144	0.93478	0.349902	0.674334
CNEP1R1	57.5841	0.057156	0.064499	0.886157	0.375533	NA
GLCE	731.3491	0.057154	0.04894	1.167819	0.24288	0.591982
GOSR1	203.0639	0.05715	0.045501	1.25602	0.209109	NA
LOC10050	12.97942	-0.05714	0.084922	-0.67289	0.501015	NA
ACCSL	0.410818	-0.05714	0.036595	-1.56135	0.118442	NA
BCL9L	467.8083	0.057129	0.047921	1.192147	0.233204	0.583654
WASL	357.4101	0.057126	0.043629	1.309354	0.190414	0.54182

ZNF576	50.47413	-0.05712	0.065958	-0.86608	0.386448	NA
LINC00051	0.947932	-0.05712	0.043687	-1.30755	0.191026	NA
SNORA80	2.098885	-0.05712	0.067423	-0.84713	0.39692	NA
C20orf194	410.0845	-0.05711	0.034025	-1.67859	0.093232	0.419831
L1TD1	7.618809	0.057114	0.066548	0.858237	0.390761	NA
ZNF32-AS2	15.4234	0.057109	0.083249	0.686005	0.49271	NA
MME	29.14387	0.057109	0.066634	0.857048	0.391419	NA
ZSCAN30	188.8284	-0.05711	0.050367	-1.13381	0.256876	NA
XPO7	350.7679	0.057103	0.036187	1.577996	0.114567	0.465042
TM9SF4	258.9669	0.057096	0.038095	1.498789	0.133928	NA
GDF7	0.780934	0.057075	0.043631	1.30813	0.190829	NA
PHF21A	445.1923	-0.05707	0.041528	-1.37428	0.169356	0.522992
TMC3	19.03821	-0.05707	0.082564	-0.69122	0.489427	NA
DHX9	517.5777	0.057068	0.039489	1.445173	0.148409	0.502511
DENND5B	740.4388	0.057047	0.033731	1.691217	0.090795	0.415466
COMMD3	14.08075	-0.05704	0.082876	-0.68831	0.491256	NA
AFF1	277.7141	-0.05704	0.057559	-0.99096	0.321707	NA
AP3M1	148.2941	0.057033	0.043378	1.314798	0.188578	NA
TONSL	35.49093	-0.05703	0.077828	-0.7328	0.463679	NA
FZD1	98.9254	0.057021	0.069878	0.816002	0.414499	NA
ZNF398	344.5157	0.057015	0.050247	1.134705	0.256499	0.598301
PPP1R13L	22.56054	0.057014	0.084897	0.671573	0.501855	NA
ADAT3	9.623821	-0.057	0.084939	-0.67112	0.502147	NA
SLC4A11	10.85654	0.056994	0.08473	0.672662	0.501163	NA
ADAMTS2	21.75411	-0.05699	0.084937	-0.67101	0.502214	NA
ZNF415	174.8623	0.056981	0.046012	1.238391	0.215571	NA
LPPR2	100.2358	-0.05696	0.052339	-1.08832	0.276453	NA
USMG5	49.8228	-0.05696	0.080411	-0.70836	0.478722	NA
MIR3155A	0.189396	-0.05694	0.02572	-2.21369	0.02685	NA
RNF11	279.0998	0.056901	0.059648	0.953945	0.340111	NA
GALR1	0.494241	-0.05689	0.03903	-1.45761	0.144947	NA
NOP2	75.2895	0.056882	0.079082	0.719281	0.471968	NA
C17orf89	35.67312	-0.05688	0.072356	-0.78607	0.431825	NA
UPF2	488.0727	0.056856	0.038169	1.489614	0.136326	0.489381
MAGI2	621.9168	-0.05685	0.043826	-1.29711	0.194592	0.544136
LOC64671	193.4607	0.056843	0.063421	0.896283	0.370102	NA
CNNM3	293.3302	-0.05682	0.057802	-0.98307	0.325574	NA
TSSC1	91.74072	-0.05681	0.052936	-1.07321	0.283176	NA
ANKRA2	76.37019	-0.0568	0.067048	-0.8472	0.396881	NA
WBSCR27	11.62508	-0.0568	0.082933	-0.6849	0.493409	NA
CD151	48.8672	0.056798	0.079826	0.711519	0.476762	NA
C10orf10	94.68919	-0.05679	0.079029	-0.71864	0.472362	NA
MAP3K14	77.88434	0.056791	0.059869	0.948593	0.342828	NA
POLD3	57.61141	0.05679	0.059629	0.952389	0.3409	NA
MYH7B	153.5759	-0.05678	0.073885	-0.76852	0.442176	NA
PPP2R2B	169.0838	-0.05678	0.055378	-1.02532	0.305214	NA
ZNF594	188.8367	0.056775	0.060837	0.933225	0.350704	NA

SOX21	10.268	-0.05677	0.084918	-0.66847	0.503835	NA
CNIH2	31.33938	0.056755	0.074511	0.761698	0.44624	NA
PTPN6	11.76676	-0.05675	0.084678	-0.67024	0.502707	NA
PTCD1	1.224492	-0.05674	0.05788	-0.98032	0.32693	NA
APPBP2	276.3231	0.05673	0.047357	1.197923	0.230947	NA
CIAPIN1	61.72937	0.056725	0.067499	0.840385	0.400692	NA
TOP1P1	0.845508	-0.05672	0.048691	-1.16484	0.244084	NA
PSMC3IP	38.75525	0.056716	0.079169	0.71639	0.473751	NA
DCAF10	191.939	0.056715	0.037647	1.506512	0.131936	NA
UACA	68.23368	-0.05671	0.068116	-0.83259	0.405074	NA
ZNF26	75.14554	0.056704	0.061899	0.916071	0.359629	NA
TAZ	45.74804	-0.0567	0.063399	-0.89438	0.371121	NA
MMP24	601.3187	0.056696	0.049363	1.148572	0.250733	0.592704
DLL4	40.80529	-0.05669	0.070554	-0.80355	0.421656	NA
ANKS6	483.3816	0.056679	0.068279	0.830112	0.406476	0.710565
PIK3C2G	1.715718	-0.05668	0.059263	-0.95636	0.33889	NA
ZNF644	813.098	0.056663	0.035262	1.606944	0.108067	0.452973
BEX1	310.3949	0.056663	0.073776	0.768043	0.442461	NA
TGFBR1	244.0902	0.056661	0.046953	1.206766	0.227522	NA
ASAP2	391.9817	0.056648	0.043658	1.297534	0.194447	0.544136
KIAA0247	514.8457	0.056648	0.048424	1.169823	0.242072	0.590833
SSBP3	219.9804	-0.05664	0.053644	-1.05586	0.291033	NA
KCNS1	2.53131	0.056628	0.073058	0.775117	0.43827	NA
ZNF469	26.40495	-0.05663	0.084912	-0.66688	0.504847	NA
CHTOP	124.2452	0.056623	0.048339	1.171378	0.241447	NA
MAGIX	49.84085	-0.05662	0.076346	-0.74162	0.458317	NA
PNLDC1	6.696483	0.056615	0.070757	0.800145	0.423627	NA
KCTD1	120.3222	0.056605	0.054812	1.032716	0.301737	NA
RABL2B	49.08676	0.056589	0.066151	0.85545	0.392302	NA
SLC5A3	349.7003	0.056585	0.081046	0.698184	0.485062	0.764934
C5orf46	5.908997	-0.05658	0.074712	-0.75732	0.448858	NA
DNASE2	20.83216	0.056573	0.080939	0.698955	0.48458	NA
ASB7	90.09511	0.05657	0.049976	1.13194	0.25766	NA
EFNA1	28.22224	-0.05657	0.080158	-0.70568	0.48039	NA
ECRP	0.229817	-0.05656	0.027447	-2.06085	0.039318	NA
GABPA	120.713	0.056564	0.046781	1.20911	0.22662	NA
SLIRP	85.93043	-0.05656	0.074576	-0.75847	0.448171	NA
GRWD1	58.30464	0.056543	0.065075	0.868896	0.384904	NA
TFAP2B	41.69561	-0.05654	0.084937	-0.66566	0.505631	NA
IFI6	37.95872	0.05653	0.084646	0.667833	0.50424	NA
LOC10028	3.316895	-0.05653	0.076411	-0.73977	0.459441	NA
MIR4482-1	8.012245	-0.05652	0.084266	-0.67073	0.502394	NA
ZNF551	144.6769	0.056514	0.056732	0.99615	0.319177	NA
CNOT1	907.4613	0.056512	0.034099	1.657279	0.097463	0.432168
MLLT6	771.1279	-0.05649	0.048718	-1.15954	0.246236	0.592704
ICAM1	6.517292	0.056489	0.064014	0.882446	0.377536	NA
ORC4	298.9214	0.05648	0.049318	1.145206	0.252124	NA

MIR1914	5.130481	-0.05648	0.080119	-0.70492	0.480861	NA
FOSL2	85.43983	-0.05647	0.080991	-0.69729	0.485624	NA
DPM3	14.72196	-0.05646	0.082011	-0.68844	0.491173	NA
HVCN1	25.98784	-0.05646	0.084935	-0.66474	0.50622	NA
MIR30B	0.398175	-0.05645	0.035368	-1.59612	0.110462	NA
JPH3	541.7002	0.056446	0.049405	1.142512	0.253241	0.594395
DKFZP434	3.356964	-0.05641	0.076945	-0.73307	0.463513	NA
EFCAB12	43.10029	-0.0564	0.083478	-0.67561	0.499287	NA
B3GALNT1	139.0194	0.056398	0.056982	0.98975	0.322296	NA
BRIP1	3.888023	-0.0564	0.079296	-0.71123	0.476941	NA
BCAT2	82.00938	-0.0564	0.067737	-0.83257	0.405088	NA
GAL3ST1	3.791668	-0.05639	0.078487	-0.71849	0.472453	NA
PREX2	332.4866	-0.05639	0.067917	-0.83028	0.406379	NA
PCCB	69.83555	0.056379	0.064805	0.86998	0.384311	NA
KCTD7	135.6424	-0.05638	0.060855	-0.92643	0.354225	NA
ECM2	67.5122	-0.05637	0.06899	-0.81707	0.413891	NA
TRIM29	1.140187	-0.05636	0.052571	-1.07206	0.283692	NA
ATP6V1E2	2.663843	-0.05636	0.072396	-0.77844	0.43631	NA
WDR52	232.5822	-0.05635	0.071073	-0.79288	0.427851	NA
LRIG1	289.6318	-0.05635	0.058243	-0.9675	0.333293	NA
C14orf2	85.5523	-0.05635	0.079565	-0.70818	0.478832	NA
LOC33987	16.91868	-0.05635	0.084058	-0.67033	0.502647	NA
PMCH	0.408061	0.056334	0.038139	1.477064	0.139659	NA
LSAMP-AS	8.110626	-0.0563	0.083198	-0.67671	0.498588	NA
LOC34007	0.406892	0.0563	0.036484	1.543147	0.122795	NA
ATP8B4	41.86799	-0.05629	0.078727	-0.71502	0.474597	NA
LGI1	145.5897	0.056287	0.059347	0.948438	0.342906	NA
SPDYE8P	0.73278	0.056282	0.042874	1.312733	0.189273	NA
SAMHD1	108.049	-0.05628	0.069968	-0.80437	0.421181	NA
CACNB4	808.9373	0.056277	0.058225	0.966546	0.333771	0.660832
SLFN12L	1.233371	0.056263	0.055809	1.008128	0.313393	NA
CREB3L1	5.135762	0.056252	0.079961	0.703498	0.481745	NA
MEOX2	1.516397	0.056252	0.056899	0.988622	0.322848	NA
ZDHHC18	53.98697	0.056236	0.069564	0.80841	0.418854	NA
IPPK	122.7486	-0.05623	0.056503	-0.9951	0.319689	NA
TMEM55B	43.25575	-0.05622	0.06252	-0.89916	0.368569	NA
MIR339	2.512569	0.056207	0.072008	0.78056	0.435061	NA
CRBN	316.7615	-0.0562	0.041998	-1.33822	0.180825	NA
PSMD7	181.079	0.056202	0.045776	1.227778	0.21953	NA
GPD1L	142.7782	0.056189	0.066246	0.848188	0.396333	NA
SLC22A5	135.4096	-0.05617	0.055054	-1.02035	0.307564	NA
FBXL16	539.1777	0.056151	0.048145	1.166299	0.243493	0.592387
VARS2	246.6957	0.056127	0.063436	0.884785	0.376273	NA
PHF15	391.7522	0.056126	0.055767	1.006431	0.314208	0.644056
ZNF540	200.7851	-0.05612	0.046148	-1.21618	0.223916	NA
GHRL	5.852022	-0.05612	0.084097	-0.66733	0.50456	NA
PCDHB5	38.6066	0.056117	0.068775	0.815949	0.414529	NA

MIR1292	0.886373	0.05611	0.046687	1.201845		0.229424	NA
BAZ1B	1069.671	0.056104	0.034102	1.645182		0.099932	0.436963
CLPTM1	250.4163	0.056077	0.047955	1.16938		0.24225	NA
A1CF	2.40792	-0.05608	0.070685	-0.79334		0.427582	NA
PLXNA2	902.1899	-0.05607	0.05996	-0.93515		0.349713	0.674334
WWP1	228.9379	0.056064	0.04241	1.32196		0.186182	NA
EML4	146.1011	-0.05606	0.060914	-0.92035		0.357388	NA
FGF10	0.321861	-0.05605	0.031549	-1.7767		0.075618	NA
MS4A4A	1.989091	-0.05605	0.062989	-0.88976		0.373593	NA
ARHGAP25	6.896085	-0.05604	0.08331	-0.67265		0.50117	NA
PILRA	53.08273	0.056038	0.069483	0.806503		0.419953	NA
DHRS11	14.61676	-0.05602	0.084527	-0.66281		0.507455	NA
MEIS2	194.8007	0.056017	0.060387	0.927637		0.353596	NA
FAM173B	86.3719	0.055992	0.067513	0.82935		0.406906	NA
SCAMP3	59.94909	-0.05599	0.063318	-0.88429		0.376541	NA
VGF	6.272621	0.055983	0.064553	0.867244		0.385809	NA
FHL2	8.88263	-0.05598	0.084589	-0.66182		0.508086	NA
BLZF1	83.61153	0.05598	0.053198	1.052284		0.292669	NA
43351	848.378	0.055974	0.042285	1.323718		0.185597	0.536751
TDGF1	0.917066	0.055962	0.0508	1.101625		0.270625	NA
TBPL2	0.516821	-0.05596	0.041357	-1.35316		0.176004	NA
HR	196.2879	-0.05595	0.072005	-0.77705		0.43713	NA
C10orf131	3.631675	0.055948	0.07393	0.756775		0.449185	NA
TRIM46	67.28272	-0.05594	0.058568	-0.95519		0.33948	NA
PHKA2	163.1339	-0.05594	0.057508	-0.97277		0.330669	NA
TRAIP	7.926815	-0.05594	0.084928	-0.65869		0.510096	NA
C14orf159	272.728	-0.05594	0.05271	-1.06127		0.288569	NA
KIT	256.4912	-0.05594	0.074928	-0.74654		0.455344	NA
TREML4	0.173065	-0.05593	0.023109	-2.42036		0.015505	NA
PHYH	60.9004	-0.05593	0.074446	-0.75128		0.452485	NA
WBP11	216.091	0.055919	0.048152	1.161317		0.245513	NA
KLRD1	3.816417	0.055906	0.072941	0.766453		0.443407	NA
MARK4	190.3957	0.055897	0.045887	1.218137		0.223172	NA
A1BG	20.49313	-0.05588	0.080491	-0.69428		0.487508	NA
GOLPH3	159.3788	0.055883	0.050337	1.110181		0.266921	NA
PLRG1	165.0867	0.055872	0.04878	1.145399		0.252044	NA
HMBS	15.12636	0.055857	0.081261	0.687379		0.491844	NA
MBD4	151.8619	-0.05585	0.047125	-1.18523		0.235925	NA
CLIC1	17.27435	-0.05585	0.081986	-0.68123		0.495724	NA
PPIEL	57.16998	-0.05585	0.076762	-0.72758		0.466873	NA
ANKRD36B	5.416245	-0.05585	0.080952	-0.68989		0.490266	NA
EMX1	1.353984	-0.05584	0.058686	-0.95158		0.34131	NA
ZNF281	206.8657	0.05583	0.049354	1.131207		0.257968	NA
DOCK11	53.79034	-0.05582	0.07293	-0.76534		0.444072	NA
TANGO6	212.6905	-0.05581	0.054723	-1.01983		0.307807	NA
CDK19	250.9418	0.055797	0.055364	1.00782		0.313541	NA
RBM34	79.14212	0.055793	0.055037	1.013732		0.310711	NA

NSUN6	127.9552	-0.05579	0.058611	-0.95184	0.341178	NA
SPOCK1	1770.128	0.055768	0.062466	0.892767	0.371982	0.695376
NUFIP2	722.7098	0.055765	0.052593	1.060303	0.289007	0.622244
SNORD116	968.4048	-0.05574	0.084938	-0.65623	0.511675	0.78307
CHST12	85.15735	-0.05574	0.067737	-0.82285	0.410592	NA
RGR	0.811785	0.055716	0.049105	1.134633	0.256529	NA
GPR64	9.230334	-0.05571	0.084922	-0.65602	0.511812	NA
GLB1L2	28.94355	-0.05571	0.081225	-0.68586	0.492803	NA
FOXRED2	105.2022	0.055693	0.060948	0.913781	0.360832	NA
ZFYVE9	626.1507	0.05569	0.03198	1.741409	0.081612	0.401988
GRINA	266.0405	0.055681	0.060502	0.92032	0.357406	NA
LOC10050	3.424401	-0.05568	0.07814	-0.71256	0.476117	NA
DAB2	91.59862	-0.05568	0.079085	-0.70401	0.481427	NA
LOC10014	2.582825	0.055656	0.063306	0.879159	0.379315	NA
GTF2H2	3.273283	-0.05565	0.07512	-0.7408	0.458812	NA
SNRPF	46.94031	-0.05565	0.068296	-0.81479	0.41519	NA
PTGIS	2.092891	-0.05564	0.063249	-0.87977	0.378982	NA
MAB21L1	255.5369	0.055632	0.042536	1.307886	0.190912	NA
SLC6A9	28.11728	0.055624	0.083211	0.668469	0.503834	NA
LOC44030	3.475789	-0.05561	0.07683	-0.72385	0.46916	NA
SPAG5-AS	10.69618	0.055611	0.083713	0.664311	0.506492	NA
AGPAT5	147.2132	0.05561	0.054174	1.026503	0.304654	NA
ZMYM6	142.1341	-0.05561	0.044604	-1.24673	0.212496	NA
SAFB2	971.5452	-0.05559	0.071889	-0.77321	0.439397	0.74018
TOP1MT	12.93569	-0.05558	0.084647	-0.65663	0.511421	NA
ADD2	1032.887	0.05558	0.038608	1.439613	0.149977	0.502511
FBXO22	106.9858	-0.05558	0.059773	-0.92982	0.352463	NA
ESYT2	381.5348	0.055573	0.036863	1.507571	0.131664	0.487416
C4BPA	13.88639	-0.05557	0.082377	-0.67456	0.499956	NA
TXNL4B	53.56485	-0.05556	0.066949	-0.82991	0.406588	NA
MAMLD1	61.23213	0.055558	0.076151	0.729567	0.465655	NA
CLCNKB	47.0093	0.055556	0.081784	0.679296	0.49695	NA
BRPF3	345.2768	0.055551	0.057969	0.958288	0.337917	0.66305
RNF167	113.5185	0.05555	0.051091	1.087291	0.276908	NA
WDR11	272.2515	-0.05554	0.046617	-1.19148	0.233464	NA
MOAP1	277.2151	0.055541	0.070085	0.792479	0.428082	NA
MIR454	2.153917	0.055508	0.071189	0.779728	0.435551	NA
ASGR1	22.15829	-0.05551	0.074093	-0.74917	0.453757	NA
OSCAR	2.342202	-0.0555	0.063903	-0.86851	0.385116	NA
LOC38772	3.045242	0.05549	0.073236	0.757692	0.448636	NA
TWSG1	40.58249	0.055486	0.072887	0.76126	0.446502	NA
COG1	204.138	-0.05548	0.055325	-1.00289	0.315915	NA
ANKRD55	2.230536	-0.05547	0.066074	-0.83958	0.401144	NA
WNT5A	19.96719	-0.05547	0.084806	-0.65405	0.513077	NA
LOC10012	58.49678	0.055463	0.068511	0.809548	0.4182	NA
RAET1G	2.865611	0.055462	0.070436	0.78741	0.431042	NA
ELAVL3	1670.032	0.05545	0.053685	1.032866	0.301667	0.632662

NAT8	13.28317	-0.05545	0.084514	-0.65609	0.511763	NA
FAM151A	10.11469	-0.05543	0.084928	-0.65266	0.513973	NA
TAS2R46	10.46394	0.055416	0.08478	0.653639	0.513345	NA
MRAP2	35.77526	-0.05541	0.084879	-0.65286	0.513844	NA
SLCO5A1	0.765917	0.055406	0.049444	1.120588	0.262463	NA
LOC10050	0.70159	0.055402	0.042698	1.297546	0.194443	NA
RBM19	348.7116	-0.0554	0.054642	-1.01386	0.31065	0.640823
LOC72973	6.494656	0.055394	0.072431	0.764784	0.4444	NA
DHRS12	41.76742	0.055375	0.080287	0.68972	0.49037	NA
LOC10028	17.81695	-0.05537	0.081091	-0.68287	0.49469	NA
LINC00235	5.030519	0.055372	0.078914	0.701677	0.482881	NA
ZKSCAN8	308.9164	0.055368	0.050828	1.089309	0.276018	NA
TUFM	224.6639	-0.05537	0.05095	-1.08669	0.277176	NA
ST7	125.3216	-0.05536	0.055321	-1.0007	0.316973	NA
ADRA2B	0.953158	-0.05536	0.052753	-1.04938	0.294005	NA
AMPD3	49.54512	-0.05534	0.07462	-0.74165	0.4583	NA
LOC10050	43.36036	0.05533	0.068679	0.805625	0.420459	NA
HEATR1	236.5156	0.055325	0.04085	1.354331	0.175631	NA
LPAR3	1.124147	0.055322	0.0539	1.026376	0.304714	NA
TYW5	99.4102	0.055312	0.064071	0.863285	0.387981	NA
ALG12	76.74458	0.055303	0.066914	0.826477	0.408533	NA
SLC2A1	117.317	-0.05529	0.077894	-0.70982	0.477816	NA
GAS8	169.7722	-0.05529	0.066633	-0.82973	0.406689	NA
KIAA0922	4.757311	-0.05529	0.081516	-0.67824	0.497622	NA
ENPP4	129.3672	0.055285	0.057742	0.95745	0.33834	NA
LOC10087	12.29815	-0.05528	0.084924	-0.65098	0.515057	NA
AQP8	1.324332	0.055281	0.057521	0.961056	0.336524	NA
WDFY3	1693.671	0.055281	0.030757	1.797355	0.072279	0.388866
LOC10014	5.781828	-0.05528	0.079923	-0.69167	0.489147	NA
ADCY10P1	89.16217	-0.05528	0.074736	-0.73963	0.459528	NA
TRIM7	124.1855	-0.05528	0.074382	-0.74314	0.457398	NA
CTRB1	1.339889	0.055273	0.057473	0.961724	0.336188	NA
FBXO43	0.831701	-0.05527	0.048831	-1.13179	0.257722	NA
FUNDC2	149.451	-0.05525	0.079847	-0.69199	0.488945	NA
CHMP4B	243.4693	0.055251	0.055904	0.988317	0.322997	NA
KIF27	69.41482	-0.05525	0.072274	-0.76441	0.444626	NA
CKAP5	862.1798	0.055239	0.036998	1.493036	0.135428	0.488446
SCARNA7	188.8423	-0.05523	0.067383	-0.81963	0.412425	NA
FLT3	159.6687	-0.05522	0.066374	-0.83198	0.405419	NA
GPR89A	13.2933	-0.05521	0.08305	-0.66477	0.506199	NA
SENP3-EIF	1.496085	-0.0552	0.062191	-0.88766	0.374726	NA
TOP3A	132.4252	0.0552	0.043837	1.259205	0.207956	NA
HACE1	304.5566	-0.0552	0.051326	-1.07544	0.28218	NA
HDHD3	16.6127	-0.0552	0.081818	-0.67464	0.499902	NA
PHLDA1	73.11453	-0.05519	0.074424	-0.74161	0.458325	NA
MRPS10	71.85762	0.055182	0.062078	0.888913	0.37405	NA
LINC00029	0.202576	0.055182	0.025482	2.165549	0.030346	NA

CA2	26.40615	0.05517	0.084793	0.65064	0.515279	NA
POLR2B	437.465	0.055164	0.051116	1.079189	0.280503	0.616835
RAB43	8.328308	0.055156	0.084937	0.649374	0.516097	NA
LRCH3	290.8747	-0.05515	0.041181	-1.33917	0.180516	NA
UNC5B-AS	11.39677	-0.05514	0.084897	-0.6495	0.516015	NA
CXCR6	3.063297	-0.05514	0.073071	-0.7546	0.450488	NA
ZSCAN21	24.77753	0.055135	0.077584	0.710646	0.477304	NA
SYNE4	38.43361	0.055132	0.068228	0.808055	0.419059	NA
SLC35E4	22.18939	0.055126	0.080479	0.68498	0.493356	NA
TRAF2	59.30875	0.055121	0.077625	0.710095	0.477645	NA
GANC	135.251	-0.05511	0.062406	-0.88314	0.377162	NA
ISM1	4.301078	0.055097	0.077648	0.709574	0.477968	NA
LOC10012	33.37642	-0.05508	0.076338	-0.72159	0.470549	NA
BACH1	138.0583	-0.05508	0.060728	-0.90705	0.364378	NA
NOL3	43.20734	-0.05508	0.073166	-0.75278	0.451582	NA
MIR4319	4.230953	0.05507	0.080716	0.682272	0.495067	NA
RBM27	329.9211	0.055066	0.040238	1.368512	0.171152	NA
AZI2	163.559	-0.05506	0.063995	-0.86041	0.389565	NA
PGPEP1	75.29353	-0.05505	0.065723	-0.83766	0.402224	NA
IVNS1ABP	329.8713	0.055053	0.047142	1.167829	0.242876	NA
HMHA1	34.63126	-0.05505	0.080893	-0.68047	0.49621	NA
C19orf44	57.12062	-0.05504	0.066076	-0.83299	0.404851	NA
OTOF	13.98911	0.05504	0.084863	0.64858	0.51661	NA
TRPC5	22.54501	-0.05503	0.084876	-0.64835	0.516756	NA
SH2D7	3.139482	0.055025	0.066691	0.825065	0.409335	NA
LOC10012	5.382468	0.055024	0.082622	0.665977	0.505426	NA
KAT2A	208.7166	-0.05502	0.062781	-0.8764	0.380815	NA
MAPK7	56.28247	0.055019	0.073192	0.751698	0.452233	NA
RPL36A	13.08931	0.055015	0.081449	0.675449	0.49939	NA
WNK1	1855.534	0.055011	0.048016	1.14567	0.251932	0.592704
FRG2B	0.85704	-0.05501	0.050445	-1.09048	0.275503	NA
ARF5	76.89603	-0.05501	0.071361	-0.77086	0.440792	NA
WDR3	105.7254	0.055003	0.05564	0.988549	0.322884	NA
C22orf32	53.71257	-0.05498	0.069338	-0.79295	0.427807	NA
LINC00310	5.540845	0.054976	0.078725	0.698334	0.484968	NA
ENY2	98.79773	-0.05497	0.058965	-0.93219	0.35124	NA
EXOG	145.0317	0.05496	0.059564	0.922706	0.356161	NA
FBRSL1	483.7716	-0.05496	0.049666	-1.10657	0.268479	0.610306
DEPDC4	1.497655	-0.05494	0.063915	-0.85953	0.390047	NA
TBC1D24	199.2773	0.054934	0.047076	1.166908	0.243247	NA
AP2B1	721.4187	0.054934	0.042152	1.303215	0.192501	0.542492
MIR9-1	1.64449	-0.05493	0.06356	-0.86426	0.387447	NA
TPBG	0.923464	0.054932	0.050632	1.084927	0.277954	NA
LOC10012	10.41152	0.054927	0.084637	0.64897	0.516358	NA
USP11	595.5499	-0.05491	0.054803	-1.00192	0.316383	0.645996
CAPN13	1.97232	-0.05491	0.066537	-0.82519	0.409261	NA
HCG22	2.887067	-0.05489	0.069646	-0.78816	0.430603	NA

LOC10050	10.13469	0.054887	0.084924	0.646309	0.518079	NA
SMIM18	10.86883	0.054884	0.084059	0.652928	0.513803	NA
MIR4645	0.317813	-0.05488	0.031202	-1.75897	0.078583	NA
WDR91	130.8074	0.054882	0.053322	1.029241	0.303366	NA
OLFML2B	3.217798	-0.05487	0.077568	-0.70744	0.479295	NA
ZYG11A	10.74087	-0.05486	0.084657	-0.64797	0.517005	NA
NSL1	379.7975	0.054854	0.037462	1.464269	0.14312	0.495647
NUDT11	27.62955	0.054853	0.076298	0.718931	0.472183	NA
BAZ2A	723.6721	-0.05483	0.038124	-1.43829	0.150352	0.503024
FAM20C	115.7169	0.054828	0.066701	0.821993	0.411081	NA
MIR5699	4.948205	-0.05482	0.082191	-0.66702	0.504759	NA
PLCD4	26.27431	-0.05481	0.075081	-0.73001	0.465386	NA
LOC64898	7.104974	-0.05479	0.084526	-0.64822	0.516845	NA
ZEB2-AS1	0.328952	0.054784	0.033939	1.61417	0.106491	NA
C9orf85	20.06309	-0.05478	0.077606	-0.70588	0.480261	NA
EVI5L	189.5363	0.054781	0.058961	0.929092	0.352842	NA
GBP1P1	1.050603	0.054769	0.047648	1.149466	0.250364	NA
SNORD114	9.765446	0.054767	0.084939	0.644786	0.519066	NA
MPP7	227.0377	-0.05476	0.080099	-0.68369	0.494172	NA
RINL	12.30244	0.054762	0.084542	0.647749	0.517147	NA
MRGPRE	0.563568	0.054755	0.043063	1.271519	0.203544	NA
NPC1L1	5.356021	0.054753	0.066905	0.818371	0.413145	NA
LOC10028	9.149869	0.054743	0.084698	0.646329	0.518066	NA
EPHB2	13.60254	0.054738	0.084935	0.644475	0.519267	NA
ZMYM4	630.0918	0.054731	0.036109	1.51572	0.12959	0.486769
SEBOX	3.780408	-0.05472	0.07876	-0.69481	0.487175	NA
VTRNA2-1	0.421503	0.05472	0.037434	1.461751	0.143809	NA
SEMA3D	6.89227	0.054689	0.074457	0.734516	0.462635	NA
RBM42	79.06857	0.054687	0.054113	1.010596	0.31221	NA
LOC10012	114.6067	0.054684	0.083649	0.653736	0.513282	NA
PRHOXNB	2.563559	0.054679	0.072045	0.758957	0.447878	NA
JMJD8	43.47879	0.054677	0.072806	0.750995	0.452656	NA
SPAG4	6.416169	-0.05467	0.083955	-0.65119	0.514927	NA
NUDT7	9.335073	-0.05467	0.08455	-0.64656	0.517918	NA
LCA5L	24.91612	0.054666	0.083439	0.655161	0.512364	NA
CACNG7	106.5262	0.054665	0.055358	0.987493	0.323401	NA
LINC00601	1.464822	-0.05464	0.059614	-0.91655	0.359376	NA
SLC22A1	1.285057	-0.05464	0.06003	-0.9102	0.362719	NA
RPL29P2	0.459354	-0.05462	0.03709	-1.47271	0.140829	NA
PLAC1	0.837584	0.054622	0.049557	1.102221	0.270366	NA
BRAT1	87.25917	-0.05462	0.065067	-0.83944	0.401221	NA
SYT17	6.785192	-0.05461	0.082457	-0.6623	0.507777	NA
PLEKHG4	7.969224	-0.05461	0.08434	-0.64749	0.517317	NA
M6PR	80.65608	-0.05461	0.061719	-0.88478	0.376275	NA
LOC10028	2.675532	-0.05461	0.070926	-0.76992	0.441347	NA
SNRPG	31.24357	-0.0546	0.083185	-0.65636	0.511591	NA
TIGD1	27.01529	0.054593	0.082068	0.665208	0.505917	NA

LINC00839	1.640213	0.05459	0.063871	0.85468	0.392729	NA
DYRK4	23.64432	-0.05458	0.075243	-0.72533	0.468251	NA
TDRG1	15.87418	-0.05457	0.083945	-0.6501	0.515626	NA
CCDC38	21.63827	0.054569	0.08433	0.647091	0.517573	NA
MIR4731	0.285276	0.054562	0.031118	1.753356	0.079541	NA
PLAC4	15.00035	-0.05454	0.082429	-0.66164	0.508202	NA
SARM1	128.4985	0.054526	0.052694	1.034767	0.300778	NA
NAPRT1	38.60959	0.054517	0.083863	0.650071	0.515646	NA
DHX40	131.6766	0.054508	0.052813	1.032097	0.302027	NA
GPAM	106.9434	0.054502	0.059431	0.917063	0.359109	NA
SLC12A2	547.6227	0.0545	0.07946	0.68587	0.492795	0.770843
LOC10050	0.571498	-0.0545	0.044246	-1.23167	0.218072	NA
ABHD1	31.58054	0.054489	0.08041	0.677643	0.497998	NA
THRB	54.3567	0.054461	0.078861	0.690597	0.489819	NA
RAB39A	17.43979	0.054453	0.083353	0.653284	0.513573	NA
SLC5A10	0.781027	-0.05445	0.051429	-1.05869	0.289739	NA
BLM	47.7969	-0.05445	0.075663	-0.71958	0.471785	NA
MFNG	1.94325	0.054444	0.068978	0.789292	0.429942	NA
CLEC14A	8.033412	-0.05444	0.084581	-0.64368	0.519782	NA
SIX1	0.477438	-0.05444	0.041305	-1.318	0.187503	NA
OTX2-AS1	0.640442	-0.05442	0.038174	-1.42567	0.153963	NA
NCOA7	849.1169	0.054422	0.045478	1.196666	0.231437	0.581158
SNORD26	95.78245	-0.05442	0.083861	-0.64891	0.516394	NA
PGD	120.4625	0.054412	0.069862	0.778843	0.436072	NA
LOC28480	111.2172	-0.0544	0.078767	-0.69067	0.489772	NA
C21orf49	6.710923	-0.0544	0.082506	-0.65932	0.509692	NA
AMOTL1	280.594	-0.0544	0.066474	-0.8183	0.413185	NA
UQCRBP1	22.74875	0.054375	0.079484	0.684098	0.493914	NA
NIM1	41.52116	-0.05437	0.068086	-0.79853	0.424562	NA
NRD1	477.4175	0.054366	0.033772	1.60982	0.107437	0.452973
MIR1275	4.121124	-0.05436	0.0729	-0.74565	0.455878	NA
LOXL2	47.03684	-0.05435	0.073436	-0.74016	0.459204	NA
PPP1R3F	119.3482	0.054336	0.049506	1.097566	0.272394	NA
HCCS	43.64773	-0.05433	0.074766	-0.72664	0.467444	NA
WDR52-AS	1.790384	-0.05431	0.060919	-0.89156	0.372631	NA
MIR4644	1.807733	-0.0543	0.063969	-0.84892	0.395928	NA
LETM2	82.20619	0.054304	0.066385	0.818018	0.413347	NA
CHCHD6	36.80407	-0.05429	0.076466	-0.71	0.477705	NA
C12orf52	39.78722	0.054289	0.066339	0.818351	0.413157	NA
LOC64516	6.371661	-0.05428	0.076812	-0.70665	0.479782	NA
ST3GAL1	74.59762	0.054278	0.073269	0.740808	0.45881	NA
USP50	0.530443	-0.05428	0.041491	-1.30816	0.19082	NA
GPR155	309.0251	0.054273	0.058656	0.925276	0.354822	NA
IL17D	74.41567	-0.05427	0.075919	-0.71479	0.474737	NA
CDKN2AIP	43.13667	-0.05427	0.071687	-0.75699	0.449058	NA
ZNF24	519.8274	0.054263	0.048562	1.117386	0.263829	0.606058
TTLL7	482.1409	0.054262	0.052967	1.024443	0.305626	0.635668

ARID1A	787.5449	0.054261	0.03951	1.373365	0.169639	0.522992
TRPV2	5.31994	0.054258	0.080358	0.675198	0.49955	NA
DICER1	951.2535	0.054254	0.038517	1.408567	0.158963	0.515834
CSTF3-AS1	0.588038	-0.05425	0.043281	-1.25344	0.210044	NA
IGF2BP3	19.6764	0.054246	0.081399	0.66642	0.505143	NA
PDS5A	674.8442	0.054243	0.056799	0.955005	0.339575	0.66305
MTBP	79.54835	-0.05422	0.068054	-0.79672	0.425613	NA
ANXA3	13.44132	-0.05421	0.083497	-0.64929	0.516149	NA
H2AFY	242.2815	0.054212	0.055644	0.974265	0.329925	NA
NUDCD3	423.7383	0.054202	0.040698	1.331829	0.182916	0.532526
GORASP1	86.42187	-0.0542	0.049734	-1.08978	0.27581	NA
MAX	246.0764	-0.05418	0.050826	-1.06599	0.286427	NA
PTPN18	391.7623	-0.05418	0.050731	-1.06796	0.285537	0.619984
GRIN2C	749.8126	0.054174	0.056719	0.955139	0.339507	0.66305
RNF4	173.054	0.054173	0.050636	1.069835	0.284694	NA
TAPT1	107.1845	0.054172	0.054981	0.985286	0.324483	NA
NABP2	48.47136	0.05417	0.070412	0.769325	0.4417	NA
RTN4	1726.931	0.054164	0.048298	1.121454	0.262094	0.605759
PARK2	53.94927	-0.05416	0.078679	-0.68838	0.491211	NA
ANKRD2	2.121575	0.054159	0.063655	0.850818	0.39487	NA
BAAT	5.682788	-0.05414	0.081739	-0.6624	0.507713	NA
DNAJC15	107.2471	0.054137	0.073295	0.738617	0.46014	NA
RGPD4	1.930873	0.054137	0.061917	0.874349	0.381928	NA
ZDHHC14	137.3135	0.054136	0.054192	0.998968	0.31781	NA
NAT2	0.59955	0.054121	0.041633	1.299953	0.193617	NA
C2orf43	11.18636	0.054114	0.083363	0.649139	0.516248	NA
LOC10013	7.013271	-0.05411	0.084872	-0.63758	0.523745	NA
MRPL50	143.4158	0.054111	0.052671	1.027356	0.304253	NA
NPBWR2	9.425668	-0.05409	0.084016	-0.64385	0.519675	NA
NACC1	159.0654	0.054093	0.05036	1.074117	0.28277	NA
MED4-AS1	1.228018	-0.05409	0.058526	-0.92422	0.355371	NA
ZNF197	260.8497	0.054087	0.040593	1.332414	0.182724	NA
MURC	23.3741	0.054086	0.079913	0.676802	0.498531	NA
ZNF70	129.3207	0.054085	0.062762	0.861749	0.388826	NA
OR11H4	0.896003	-0.05408	0.05015	-1.07843	0.280841	NA
CEP57L1	67.44185	0.054063	0.064557	0.837448	0.402341	NA
ZNF501	49.23719	0.054062	0.071807	0.752872	0.451527	NA
TNFRSF19	7.726973	-0.05405	0.084935	-0.63642	0.524502	NA
VN1R5	0.574439	-0.05405	0.041175	-1.31279	0.189255	NA
MIR578	5.841512	-0.05405	0.08429	-0.64126	0.521354	NA
EMX2OS	16.86451	-0.05405	0.084066	-0.64296	0.520247	NA
CIDECP	8.6211	-0.05405	0.084689	-0.63819	0.523348	NA
XPOT	261.2276	0.054023	0.048073	1.123775	0.261109	NA
PASK	90.54601	0.053995	0.077693	0.694985	0.487065	NA
WDR83OS	12.77694	0.053986	0.082796	0.652038	0.514377	NA
HGC6.3	2.495231	-0.05396	0.048771	-1.10643	0.26854	NA
P2RY6	0.77365	0.05396	0.047581	1.134066	0.256767	NA

INTS9	31.59947	-0.05394	0.071345	-0.75606	0.44961	NA
MZT2B	7.413493	0.053934	0.084872	0.635473	0.52512	NA
ATRX	2579.884	0.053929	0.039844	1.353492	0.175899	0.529097
GCNT7	15.22641	-0.05391	0.082818	-0.65094	0.515087	NA
ASIP	2.055769	0.053899	0.063932	0.843077	0.399186	NA
PXMP4	31.61873	-0.05389	0.078089	-0.69016	0.490096	NA
CKAP4	64.5395	0.053892	0.068913	0.782027	0.434199	NA
SMC6	395.4144	0.053889	0.043913	1.227169	0.219759	0.569906
PCNA	39.60931	-0.05388	0.070779	-0.76128	0.446492	NA
DFFA	103.2853	0.053867	0.054034	0.996912	0.318807	NA
TBX1	3.460536	-0.05386	0.0725	-0.74286	0.457568	NA
NAT9	74.83923	0.05385	0.066986	0.803907	0.421451	NA
MAK	161.9096	-0.05384	0.058268	-0.92403	0.355469	NA
CREBBP	1570.495	0.053841	0.043932	1.225556	0.220366	0.569906
TRIM2	1273.441	0.053807	0.048452	1.110515	0.266777	0.607287
CCHCR1	65.90747	-0.0538	0.064659	-0.83212	0.40534	NA
VPS26A	222.5325	-0.0538	0.062424	-0.86186	0.388763	NA
TRAPPC2P	9.338499	0.053798	0.084855	0.633996	0.526083	NA
KDELR1	68.33304	-0.05377	0.061843	-0.8695	0.384575	NA
NFRKB	198.8665	-0.05377	0.060835	-0.88389	0.376753	NA
HCRT	0.436464	0.053771	0.037145	1.447577	0.147735	NA
DBX2	7.185631	-0.05377	0.082729	-0.6499	0.515759	NA
SNORA7B	10.01975	-0.05376	0.084926	-0.63303	0.526717	NA
P2RX1	1.292922	-0.05376	0.056631	-0.9493	0.34247	NA
EIF2AK4	293.1602	0.053752	0.040561	1.325226	0.185096	NA
WWC1	82.90549	-0.05375	0.073944	-0.7269	0.467288	NA
PARP3	18.75074	0.053749	0.080008	0.671799	0.501712	NA
ZNF639	105.4705	0.053746	0.053485	1.004874	0.314958	NA
CCSER2	582.2437	0.053735	0.039758	1.351553	0.176518	0.529395
EEF1A1	1058.738	-0.05371	0.077791	-0.69046	0.489904	0.76738
CHID1	73.67578	-0.0537	0.060622	-0.88578	0.375736	NA
SLC40A1	15.86861	-0.05369	0.083902	-0.63995	0.522207	NA
CDKL2	84.88564	0.053692	0.063952	0.839566	0.401152	NA
COX7C	425.1678	-0.05368	0.075897	-0.70733	0.47936	0.760949
A2M	320.7342	0.053684	0.076276	0.703816	0.481547	NA
LAMB3	6.30529	-0.05368	0.084495	-0.63535	0.525199	NA
BEND3P3	226.4759	0.053684	0.068643	0.782073	0.434171	NA
STK17A	149.6228	0.05367	0.049936	1.074786	0.282471	NA
CLPTM1L	71.66648	0.05367	0.063234	0.848754	0.396018	NA
EVC2	6.514448	-0.05367	0.081106	-0.66168	0.508175	NA
BUB1	0.824381	-0.05366	0.049428	-1.08553	0.277686	NA
TBX2	10.33212	0.053644	0.083799	0.640157	0.522071	NA
ADCY10	18.60057	-0.05364	0.080754	-0.66423	0.506546	NA
FTCD	9.228648	0.053638	0.076974	0.696831	0.485908	NA
CLEC11A	13.95494	-0.05363	0.081518	-0.65794	0.510578	NA
SOBP	359.1134	0.05363	0.039347	1.362985	0.172887	0.525733
MIR4296	1.826161	-0.05362	0.064415	-0.83238	0.405194	NA

MSC	0.317522	-0.05362	0.032688	-1.64022	0.10096	NA
CD200	96.80536	0.053608	0.060168	0.890967	0.372947	NA
C4orf47	10.99155	0.053586	0.084412	0.634816	0.525548	NA
TRAPPC8	340.7591	0.053581	0.03788	1.414494	0.157217	0.51459
LOC28333	12.13323	-0.05357	0.082814	-0.64691	0.517691	NA
TCN1	1.960264	-0.05357	0.061196	-0.87543	0.381338	NA
SLC9A2	12.73266	0.053558	0.084283	0.635463	0.525126	NA
ID3	8.037529	0.053554	0.080375	0.666299	0.50522	NA
GLDN	24.96865	-0.05355	0.08359	-0.64066	0.521745	NA
CCDC93	443.4142	-0.05354	0.051696	-1.03575	0.300321	0.632181
CEP44	107.7597	-0.05354	0.063565	-0.84224	0.399656	NA
PAF1	207.0009	0.053528	0.043445	1.232104	0.21791	NA
DPH3P1	5.844254	0.053519	0.082266	0.65056	0.515331	NA
WSCD1	314.5209	0.05351	0.075692	0.706951	0.479597	NA
CCDC85C	447.8155	0.053502	0.049308	1.085072	0.27789	0.614792
WIPF3	66.92711	-0.05349	0.073556	-0.7272	0.467101	NA
SPDYE5	18.72646	0.053483	0.082417	0.648931	0.516383	NA
LOC10050	0.827481	0.053469	0.049682	1.076234	0.281823	NA
HMOX2	142.0004	0.053467	0.050964	1.049116	0.294125	NA
LINC00667	172.0293	0.053463	0.059564	0.897579	0.36941	NA
PWWP2A	418.1326	0.053461	0.053099	1.006814	0.314024	0.644056
REEP2	211.6239	0.053459	0.055001	0.971951	0.331075	NA
LINC00840	0.992759	0.053456	0.053479	0.999563	0.317522	NA
LOC39971	12.78301	-0.05345	0.07854	-0.68061	0.496121	NA
CXXC11	1.695878	-0.05345	0.064072	-0.83427	0.404127	NA
MIR5694	0.609806	-0.05345	0.043798	-1.22045	0.222293	NA
CYSLTR1	0.756444	0.053451	0.043705	1.222988	0.221334	NA
EIF4EBP2	550.0716	-0.05344	0.039723	-1.34542	0.17849	0.530729
NCOA1	949.1437	0.053411	0.035912	1.487272	0.136943	0.489381
LIFR	544.814	-0.05341	0.067682	-0.78906	0.430078	0.733529
H6PD	143.5131	-0.0534	0.076035	-0.70227	0.482511	NA
PIGT	107.5141	0.053382	0.061338	0.870281	0.384147	NA
SNHG10	53.76981	-0.05337	0.078739	-0.67784	0.497873	NA
CASP8	16.72367	-0.05336	0.082894	-0.64377	0.519723	NA
MAP3K7	259.9161	0.053358	0.039809	1.34033	0.180138	NA
SLC10A1	35.65013	-0.05335	0.084326	-0.63264	0.526966	NA
SEC31A	366.5902	0.053345	0.035092	1.520157	0.128471	0.48338
HYDIN2	17.54025	-0.05334	0.084064	-0.63447	0.525775	NA
SLMAP	427.8393	0.053335	0.032036	1.66483	0.095947	0.427793
LRRC14B	1.079939	-0.05333	0.055116	-0.96761	0.333237	NA
UFL1	187.8754	-0.05333	0.059545	-0.8956	0.370468	NA
MFSD9	46.53523	0.053327	0.06509	0.819276	0.412629	NA
DVL3	287.0063	-0.05332	0.037862	-1.40841	0.159011	NA
CLK3	146.3528	0.053325	0.052419	1.017278	0.309021	NA
ZNF890P	1.298212	-0.05332	0.046435	-1.14835	0.250824	NA
PROSAPIP	838.9414	0.05332	0.039082	1.364295	0.172475	0.525733
TMPRSS6	3.986142	-0.05332	0.07653	-0.69669	0.485995	NA

EGLN1	332.5996	0.0533	0.045329	1.175856	0.239653	NA
MMP21	8.553696	0.053297	0.084936	0.627503	0.53033	NA
AGPAT9	39.14334	0.053286	0.067224	0.792673	0.427968	NA
LOC10050	5.390614	-0.05328	0.082105	-0.64895	0.516373	NA
PCNXL2	606.9436	-0.05328	0.04417	-1.20618	0.227747	0.577536
HOMER3	197.5125	-0.05325	0.082515	-0.64536	0.518691	NA
SNED1	283.4205	-0.05325	0.057656	-0.92356	0.355717	NA
TRMU	79.06511	-0.05325	0.061142	-0.8709	0.383806	NA
MIR4669	0.273185	-0.05323	0.028917	-1.84093	0.065631	NA
OSGIN2	88.66251	0.053233	0.062402	0.853063	0.393624	NA
VPRBP	192.2091	0.053223	0.041579	1.280046	0.200529	NA
C17orf102	6.447507	-0.05322	0.078616	-0.67693	0.49845	NA
CXCL5	1.31744	0.053218	0.05444	0.977548	0.328298	NA
GTF2E2	121.9942	0.053214	0.04412	1.206116	0.227773	NA
LINC00336	0.267426	-0.05321	0.027837	-1.91153	0.055936	NA
TM9SF3	412.113	0.053212	0.051268	1.037914	0.29931	0.631658
RAD51AP1	5.609614	-0.05321	0.081159	-0.65561	0.512073	NA
WTIP	15.31877	0.053203	0.083159	0.639774	0.522319	NA
LOC20218	201.3475	-0.0532	0.048396	-1.09928	0.271647	NA
MSRA	634.4357	-0.0532	0.053815	-0.98853	0.322895	0.652416
TMX1	82.08286	0.053185	0.060268	0.882477	0.377519	NA
OSGEP	110.3887	-0.05318	0.07011	-0.75856	0.448118	NA
TAF5	74.60004	0.053167	0.05859	0.907436	0.364176	NA
PYY	0.261871	-0.05316	0.031285	-1.69937	0.08925	NA
AQP4	907.3029	-0.05315	0.084136	-0.63175	0.527552	0.791853
AFAP1L1	24.84441	-0.05315	0.083096	-0.63963	0.522411	NA
MAGEL2	1.059049	-0.05314	0.052829	-1.00597	0.314428	NA
TRIP13	2.429609	0.053142	0.070906	0.749462	0.453578	NA
MIR4535	0.725601	-0.05314	0.045706	-1.16258	0.244999	NA
LHX1	183.2411	0.053135	0.048448	1.096727	0.272761	NA
SH2B1	204.8051	-0.05313	0.042083	-1.26254	0.206754	NA
ZNF670	39.23237	0.053129	0.066792	0.795442	0.426356	NA
FLJ43663	281.6768	-0.05313	0.080051	-0.66368	0.506897	NA
RNF17	1.285546	0.053127	0.05703	0.931556	0.351566	NA
COL6A4P1	0.921005	-0.05312	0.052678	-1.00834	0.31329	NA
PCDHB4	46.42464	-0.05311	0.074646	-0.71147	0.476791	NA
VAC14	79.20294	-0.0531	0.053394	-0.9945	0.319981	NA
IZUMO4	17.86918	-0.0531	0.082892	-0.64057	0.521805	NA
CXorf21	0.951472	-0.05308	0.050703	-1.04686	0.295164	NA
LOC39066	0.197863	-0.05308	0.026709	-1.98721	0.046899	NA
MCAT	21.61183	0.05307	0.077141	0.68796	0.491478	NA
NSMCE2	31.33483	-0.05305	0.071179	-0.74536	0.456053	NA
CCDC101	79.31767	-0.05304	0.060353	-0.87888	0.379464	NA
SNORA36C	0.748584	-0.05303	0.044838	-1.18281	0.236885	NA
ATXN7L1	238.4513	-0.05303	0.043584	-1.21664	0.223742	NA
AP1B1	280.8577	-0.05302	0.047654	-1.11251	0.265917	NA
COQ9	123.9826	-0.05302	0.052859	-1.00297	0.315875	NA

DNAJA1P5	24.66391	0.053002	0.081071	0.653768	0.513261	NA
SIRT1	271.787	0.052988	0.039596	1.338212	0.180827	NA
RARRES1	27.88964	0.052982	0.07573	0.699608	0.484172	NA
LARP6	85.8649	0.052978	0.071657	0.739324	0.459711	NA
LOC64690	9.237159	0.052974	0.084617	0.626049	0.531283	NA
BATF3	2.706404	-0.05297	0.067236	-0.78785	0.430783	NA
DNAJC5	618.2793	0.05297	0.044891	1.179981	0.238008	0.587242
TIPRL	129.7003	0.052969	0.058681	0.902653	0.36671	NA
C5AR1	2.1434	-0.05296	0.066768	-0.79323	0.427646	NA
TMEM170	20.59585	0.052955	0.078252	0.676727	0.498579	NA
FEZ2	199.8818	0.052954	0.072358	0.731833	0.46427	NA
RAD51D	27.71504	0.052954	0.072872	0.726671	0.467428	NA
SMG9	144.2581	0.052953	0.054639	0.969139	0.332476	NA
GABRA5	1.536581	-0.05295	0.059698	-0.88689	0.375136	NA
PDP1	125.1404	0.052943	0.067431	0.785141	0.432371	NA
PLCH1	143.3172	0.052942	0.082778	0.639562	0.522457	NA
LOC33982	6.236081	0.052937	0.052622	1.005977	0.314427	NA
RAB13	41.65131	-0.05294	0.081545	-0.64917	0.516232	NA
SEC61A1	138.3257	0.052927	0.055469	0.954174	0.339996	NA
LSG1	90.10756	0.052924	0.068536	0.772203	0.439994	NA
ERCC6L	29.36673	-0.05292	0.078728	-0.67222	0.501445	NA
LOC10012	1.133602	-0.05292	0.056189	-0.94186	0.346267	NA
MAFK	20.32478	-0.05291	0.08193	-0.64583	0.518387	NA
TNKS1BP1	167.4257	-0.05291	0.054776	-0.96596	0.334062	NA
CDC14A	11.98562	-0.05291	0.084524	-0.62597	0.531337	NA
LCAT	58.995	-0.0529	0.076796	-0.68888	0.490899	NA
DTNBP1	73.87945	-0.0529	0.053623	-0.98649	0.323892	NA
MYCBP2-A	9.23195	0.052888	0.084814	0.623573	0.532908	NA
RBP3	2.652103	-0.05289	0.072091	-0.73361	0.463187	NA
TAP1	29.83732	0.052883	0.083465	0.633592	0.526347	NA
ZNF717	84.51908	0.052876	0.061889	0.854367	0.392902	NA
TEKT5	0.939828	-0.05288	0.044127	-1.19825	0.230818	NA
ZNF260	157.9935	0.052866	0.0509	1.038628	0.298978	NA
ASB1	118.4056	0.052863	0.047925	1.103044	0.270008	NA
ZBTB14	109.1839	0.052849	0.057362	0.921313	0.356887	NA
COA4	25.51208	-0.05285	0.073251	-0.72146	0.470629	NA
WBP2	478.4206	-0.05284	0.061092	-0.86492	0.387081	0.700314
GPR160	8.754729	0.05284	0.0849	0.622375	0.533695	NA
HUNK	44.13229	-0.05282	0.083455	-0.63291	0.52679	NA
TMEM132	92.99495	-0.05282	0.074105	-0.71273	0.476014	NA
DIRC2	44.08103	-0.05281	0.067491	-0.78246	0.433944	NA
TRIM59	8.868493	0.052791	0.082339	0.641137	0.521434	NA
PRCP	111.0687	-0.05278	0.071978	-0.73331	0.463367	NA
FJX1	13.77123	-0.05278	0.084818	-0.6223	0.533742	NA
UBR4	1678.965	0.05278	0.036502	1.44595	0.148191	0.502511
LGALS1	15.81488	-0.05278	0.084897	-0.62167	0.534158	NA
HMGN2	141.0425	-0.05277	0.071842	-0.73452	0.462634	NA

SRCIN1	922.0674	0.052768	0.049332	1.069656	0.284774	0.619296
CNKSR1	32.23349	0.052767	0.083582	0.631313	0.527836	NA
KIAA0195	340.9224	-0.05276	0.047409	-1.11296	0.265727	0.607287
PPIL1	39.74541	-0.05276	0.080406	-0.65619	0.511699	NA
TNK1	57.74394	0.052743	0.059369	0.888381	0.374336	NA
EGLN2	3.504148	0.052736	0.074001	0.712632	0.476074	NA
MOB3B	91.80938	0.052733	0.073375	0.71867	0.472344	NA
PCBD1	35.13374	0.052724	0.076597	0.688332	0.491243	NA
LRR1	5.97196	-0.05272	0.084565	-0.62346	0.532982	NA
PTTG1IP	233.4096	-0.05272	0.056464	-0.93366	0.350478	NA
INPP5J	181.3801	0.052717	0.064943	0.811743	0.416939	NA
MIR4254	0.441217	-0.05271	0.03607	-1.46119	0.143963	NA
ARMC3	0.742792	-0.05269	0.045741	-1.152	0.24932	NA
PLEKHA7	39.22498	-0.05268	0.080969	-0.65061	0.515297	NA
PELP1	273.156	-0.05268	0.045046	-1.16939	0.242248	NA
PRR14	100.584	0.052674	0.057833	0.910807	0.362397	NA
RPP38	33.85607	-0.05267	0.073833	-0.71337	0.475616	NA
ARL8B	247.6752	0.052651	0.055656	0.946006	0.344145	NA
SYT15	71.37284	0.052646	0.080727	0.652142	0.51431	NA
TMEM38B	45.14909	0.052645	0.068127	0.772742	0.439675	NA
SUCLA2	174.5039	0.052644	0.062146	0.8471	0.396939	NA
HLTF	505.7692	0.05264	0.045241	1.163537	0.244612	0.592704
THBS1	33.40604	-0.05264	0.084914	-0.61989	0.535327	NA
CLDND2	5.259961	-0.05264	0.080593	-0.65312	0.513682	NA
LIPT2	5.227665	-0.05263	0.083024	-0.63397	0.5261	NA
EIF4E1B	16.84499	-0.05263	0.082827	-0.63543	0.525147	NA
CATSPERG	34.57703	-0.05263	0.084534	-0.62259	0.533556	NA
CD300C	0.431838	0.052626	0.037804	1.39207	0.163901	NA
SF3A2	158.8992	-0.05262	0.051057	-1.03061	0.302723	NA
RIBC2	1.344133	0.052619	0.050874	1.034301	0.300996	NA
GFRA1	27.58224	-0.05261	0.084774	-0.62056	0.534889	NA
FAM188A	189.292	0.052607	0.047702	1.102823	0.270104	NA
EIF3E	326.8711	-0.0526	0.070035	-0.75112	0.452581	NA
LOC10050	62.99372	0.052571	0.064632	0.813402	0.415988	NA
C2orf57	0.337563	0.052568	0.032946	1.595578	0.110583	NA
SAMD5	8.070688	0.052568	0.083313	0.630963	0.528065	NA
ZNF689	46.98695	0.052564	0.062092	0.846549	0.397247	NA
SNORD50B	2.455174	-0.05255	0.072594	-0.72383	0.469169	NA
PAPD4	142.5369	-0.05254	0.060291	-0.87137	0.383554	NA
ERG	19.02441	-0.05253	0.08301	-0.63288	0.526814	NA
PRRT3	174.152	0.052533	0.04047	1.298079	0.19426	NA
GRM5-AS1	0.745886	-0.05253	0.044329	-1.18496	0.236032	NA
TIA1	351.4145	-0.0525	0.045633	-1.15046	0.249955	0.592704
BMPR1A	99.42376	0.052491	0.061934	0.847533	0.396698	NA
SEPT7L	0.960853	0.052485	0.050388	1.041615	0.29759	NA
SPATA6L	109.4718	0.05245	0.078901	0.664753	0.506209	NA
FAM166A	1.496555	-0.05244	0.060954	-0.86027	0.389639	NA

TTC16	1.298087	-0.05242	0.059825	-0.87623	0.380906	NA
MAN2A2	516.52	-0.05242	0.045427	-1.15391	0.248539	0.592704
CDKN2B	5.382525	-0.05241	0.08046	-0.65137	0.514805	NA
ACSL5	70.14311	-0.0524	0.079225	-0.66142	0.508346	NA
KAT6A	1165.429	0.052396	0.037095	1.412484	0.157807	0.514729
AP1G2	444.57	-0.05239	0.078997	-0.66319	0.507206	0.780812
C12orf75	16.63007	-0.05239	0.084823	-0.61761	0.536835	NA
LRRC8C	23.52718	0.052383	0.078846	0.664368	0.506455	NA
ACBD4	66.34705	-0.05238	0.06876	-0.76176	0.446204	NA
RUNDC3B	205.6131	-0.05237	0.044442	-1.17849	0.238603	NA
TNFSF9	5.846392	0.052374	0.083878	0.624409	0.532359	NA
PAFAH1B1	1172.82	0.05237	0.048148	1.087686	0.276734	0.61452
DPY19L2	105.5281	0.052344	0.077068	0.679189	0.497018	NA
SCN4A	4.283512	0.052338	0.068374	0.765466	0.443994	NA
EME1	2.978311	0.052331	0.074803	0.69959	0.484183	NA
IFIT5	157.2763	0.052313	0.056718	0.922342	0.35635	NA
SH2D2A	1.031608	0.052311	0.054174	0.965611	0.334239	NA
MIR4748	0.481782	-0.0523	0.037025	-1.41268	0.157751	NA
SUGT1	219.3254	0.052304	0.054387	0.961691	0.336205	NA
TUBGCP2	190.0295	0.052298	0.053936	0.96963	0.332231	NA
AQP7P1	5.346842	0.052287	0.075228	0.69505	0.487024	NA
ZNF234	126.792	0.052281	0.061594	0.848803	0.395991	NA
BAHD1	47.62615	0.052275	0.067487	0.774588	0.438583	NA
PSORS1C2	0.340268	-0.05227	0.034358	-1.52123	0.128203	NA
SHISA7	71.29206	-0.05226	0.081443	-0.64173	0.521046	NA
EED	123.0807	0.052259	0.052265	0.999894	0.317362	NA
KIAA0513	2070.176	0.052258	0.051808	1.008694	0.313121	0.643552
SGPL1	137.4375	-0.05225	0.044401	-1.17673	0.239303	NA
TCEAL5	150.4798	0.05224	0.073276	0.712927	0.475891	NA
CAV2	8.788857	-0.05222	0.083604	-0.62467	0.532187	NA
PPDPF	137.8299	-0.05222	0.074274	-0.70311	0.481985	NA
LOC10050	5.116605	0.05222	0.07954	0.656524	0.511487	NA
EFHD2	115.4144	-0.05222	0.063813	-0.81832	0.413173	NA
CCDC162P	0.461718	-0.05222	0.037823	-1.3806	0.167401	NA
CHD9	1852.43	0.052218	0.026808	1.947835	0.051435	0.349849
BZRAP1	757.3506	0.052214	0.067555	0.772919	0.439571	0.74018
EIF2S3	283.3194	-0.05221	0.068739	-0.75953	0.447533	NA
TMEM120	378.7987	-0.0522	0.051015	-1.02321	0.306208	0.635871
ME1	59.32899	0.052185	0.075615	0.69014	0.490106	NA
ZNF596	59.82307	-0.05218	0.076116	-0.68556	0.49299	NA
WDR62	10.755	0.052179	0.084199	0.619713	0.535447	NA
C2orf62	2.191883	-0.05218	0.065505	-0.79651	0.425735	NA
CEP164	345.52	-0.05217	0.060043	-0.86888	0.38491	0.70023
TP53TG3D	1.237505	-0.05217	0.057015	-0.91498	0.360204	NA
PRICKLE2-	1.808154	-0.05216	0.067483	-0.77291	0.439573	NA
ADAM20P	45.82893	-0.05214	0.077619	-0.67178	0.501724	NA
ARL17A	31.59263	-0.05214	0.084803	-0.61479	0.538692	NA

DDX41	151.802	-0.05213	0.043391	-1.2015	0.229558	NA
EFCAB6	40.1424	0.052132	0.076104	0.685007	0.49334	NA
HS3ST2	0.733404	-0.05213	0.045491	-1.1459	0.251836	NA
CBX8	5.2194	0.052124	0.081662	0.638288	0.523286	NA
CDHR3	104.5005	-0.05212	0.082615	-0.63084	0.528142	NA
LRRD1	6.042584	0.052105	0.083833	0.621534	0.534248	NA
MAP4K5	374.9309	-0.05208	0.047688	-1.09208	0.274799	0.613511
CACNG4	24.06585	0.052065	0.083629	0.622571	0.533567	NA
ZBTB7C	4.272073	0.052061	0.078538	0.662868	0.507415	NA
MTHFD1L	532.5361	-0.05206	0.04943	-1.05313	0.29228	0.6257
COL28A1	49.3721	-0.05206	0.083903	-0.62044	0.534968	NA
ZNF696	70.16432	-0.05205	0.066283	-0.78532	0.432268	NA
DSTYK	346.1476	0.052042	0.04603	1.130611	0.258219	0.59833
LRRC16B	240.8303	0.052039	0.072069	0.722079	0.470246	NA
C12orf71	0.679552	-0.05203	0.044283	-1.17501	0.23999	NA
KAT6B	619.9093	0.052021	0.038145	1.363784	0.172636	0.525733
POLR2F	91.64955	-0.05202	0.072056	-0.72191	0.47035	NA
MYBPC2	0.954441	0.052015	0.051265	1.014625	0.310285	NA
CHCHD7	103.6565	0.051999	0.051591	1.007911	0.313497	NA
GGA2	517.3786	-0.05199	0.052815	-0.98445	0.324897	0.655185
SASS6	62.90858	0.051993	0.060337	0.861714	0.388845	NA
C1orf27	310.1977	-0.05198	0.043326	-1.1998	0.230216	NA
GNG12-AS	2.121663	-0.05198	0.06602	-0.78729	0.43111	NA
ADORA2A	25.97175	0.051972	0.084939	0.611879	0.540618	NA
CCNA1	1.074626	0.051971	0.05148	1.009533	0.312719	NA
ZNF259	80.8611	0.051968	0.063135	0.823121	0.410439	NA
POLR1B	75.41144	0.051955	0.065472	0.79355	0.427457	NA
FAM104B	22.8286	0.051955	0.079052	0.657233	0.511031	NA
HSDL2	81.86312	-0.05195	0.073905	-0.70296	0.482084	NA
GATM	290.6229	0.05195	0.060629	0.85684	0.391533	NA
NUDCD1	83.31303	0.051949	0.051078	1.017054	0.309128	NA
TMEM140	19.91899	-0.05195	0.081986	-0.63361	0.526335	NA
STAC3	45.01207	-0.05194	0.074874	-0.69377	0.487829	NA
LIN7B	23.38254	-0.05194	0.075892	-0.68438	0.493734	NA
RPRM	0.91265	0.051936	0.052285	0.993315	0.320556	NA
CAPN10	66.06518	-0.05193	0.065191	-0.79657	0.425698	NA
E2F3	9.011465	-0.05193	0.084937	-0.61135	0.540966	NA
R3HDM1	492.4594	0.051907	0.047487	1.093085	0.274356	0.613511
GYPE	0.499765	-0.0519	0.041097	-1.26293	0.206613	NA
SECTM1	1.820361	-0.0519	0.059891	-0.86656	0.386185	NA
RASSF1	39.51166	-0.0519	0.068359	-0.75915	0.447763	NA
ACTR5	38.37639	0.051882	0.067278	0.771162	0.440611	NA
MYO3A	0.786945	0.051882	0.047843	1.084416	0.27818	NA
KCTD16	10.29081	-0.05185	0.084857	-0.61099	0.541209	NA
TRIM23	397.4453	0.051824	0.040295	1.286113	0.198404	0.545847
MAP1S	101.6637	0.051812	0.056003	0.925162	0.354882	NA
FAM135A	339.3158	0.051806	0.034249	1.512635	0.130372	0.487283

RAB3A	294.5061	0.051803	0.072759	0.711983	0.476476	NA
DEPDC1	0.470353	-0.0518	0.039929	-1.29736	0.194509	NA
NYAP2	121.4084	-0.0518	0.056271	-0.92048	0.357324	NA
CABP4	6.203848	-0.05179	0.084365	-0.61389	0.539291	NA
ARL3	185.6197	-0.05177	0.049535	-1.04518	0.295942	NA
OLIG1	39.70355	-0.05176	0.080273	-0.64484	0.51903	NA
NSUN3	12.95912	0.051752	0.083155	0.62235	0.533712	NA
WFDC13	0.76482	0.051751	0.043361	1.193511	0.232669	NA
PRDX3	63.21294	-0.05174	0.079427	-0.65138	0.514804	NA
PCYT1B	47.9814	-0.05173	0.076245	-0.67847	0.497471	NA
GMPS	324.8268	0.05173	0.032368	1.59819	0.110001	NA
TLE1	84.42783	0.05173	0.058206	0.888741	0.374142	NA
ARHGEF17	344.949	0.051726	0.047354	1.092326	0.27469	0.613511
TWIST1	0.810976	-0.05172	0.049553	-1.04383	0.296565	NA
DTD2	43.19499	0.051716	0.062028	0.833752	0.404421	NA
REEP3	264.5441	-0.0517	0.041634	-1.24179	0.214314	NA
MIB2	175.9011	-0.0517	0.055228	-0.93606	0.349245	NA
FAM78B	25.74534	0.051689	0.077704	0.665213	0.505915	NA
CCNJ	52.38802	-0.05168	0.072851	-0.7094	0.478078	NA
C5orf34	9.2185	-0.05168	0.084917	-0.60856	0.542819	NA
TFCP2	212.2296	0.051663	0.053671	0.962587	0.335755	NA
43165	1049.925	0.051646	0.047568	1.085721	0.277602	0.614792
DAPK1	70.78699	-0.05164	0.07059	-0.73155	0.464443	NA
SS18	202.3678	0.051639	0.046433	1.112128	0.266083	NA
NDNF	5.236404	-0.05164	0.072989	-0.70747	0.479275	NA
KDM4D	5.205408	0.051637	0.077448	0.666728	0.504946	NA
TFAM	143.5507	0.051633	0.048735	1.059462	0.289389	NA
GTPBP4	106.94	0.05163	0.058691	0.879694	0.379025	NA
PPP2R1B	209.0553	-0.05163	0.063206	-0.81685	0.414015	NA
HIP1	270.8467	-0.05162	0.063387	-0.81435	0.415445	NA
NT5C2	405.548	-0.05161	0.034597	-1.49188	0.13573	0.488758
RRP7A	101.4259	-0.05159	0.074661	-0.69103	0.489545	NA
UROS	50.60574	-0.05158	0.077426	-0.66623	0.505264	NA
CPNE4	0.428925	0.051583	0.035372	1.458321	0.144752	NA
GHDC	48.22081	-0.05157	0.060979	-0.84577	0.397683	NA
GNG7	150.1622	-0.05156	0.050884	-1.01319	0.310968	NA
ACTC1	6.061175	-0.05156	0.082941	-0.62159	0.534214	NA
LOC10013	0.456308	-0.05155	0.037195	-1.38591	0.165776	NA
SSTR4	0.921007	0.051543	0.051757	0.995874	0.319311	NA
SLC20A1	198.3322	-0.05154	0.068898	-0.74802	0.454446	NA
SNF8	71.6642	0.051533	0.060617	0.850139	0.395248	NA
PAH	2.368277	-0.05152	0.064266	-0.80174	0.422704	NA
LILRB4	2.898877	-0.05152	0.070493	-0.73081	0.464898	NA
PGP	222.9257	-0.05152	0.059693	-0.86302	0.388129	NA
SLC30A9	313.473	0.051512	0.06454	0.798135	0.424792	NA
WHSC1	904.3215	0.051507	0.040071	1.285378	0.19866	0.545847
C19orf59	1.390878	0.051503	0.05992	0.859525	0.390051	NA

MUT	188.3861	0.051498	0.052905	0.973407	0.330351	NA
CTR9	167.1048	0.051497	0.045588	1.129623	0.258635	NA
CARD10	4.663695	-0.0515	0.079081	-0.65118	0.514929	NA
C3orf58	218.722	0.051492	0.052839	0.974515	0.329801	NA
SAMD1	89.36831	0.051488	0.074054	0.695271	0.486885	NA
SPDYE3	33.47165	-0.05148	0.070819	-0.72691	0.46728	NA
ADAM17	210.7565	0.051476	0.044929	1.145702	0.251919	NA
MBD6	110.0852	-0.05145	0.062315	-0.82571	0.408967	NA
TSPAN11	82.31284	0.051434	0.084835	0.606284	0.544326	NA
NKAIN3	4.096834	-0.05143	0.078058	-0.65886	0.509987	NA
FRZB	8.3469	0.051429	0.082124	0.626241	0.531157	NA
CAMK2G	509.2916	0.05142	0.060396	0.851373	0.394562	0.705623
LOC28488	17.90832	-0.05142	0.083908	-0.61277	0.54003	NA
SNORD115	6.024116	-0.05142	0.083591	-0.61509	0.538498	NA
HEPACAM	0.310821	-0.05141	0.030511	-1.68501	0.091987	NA
C15orf38	19.36123	-0.0514	0.080337	-0.63986	0.522261	NA
ANKAR	87.31954	-0.0514	0.060588	-0.84844	0.396194	NA
RCBTB2	27.76847	0.051404	0.077347	0.66459	0.506313	NA
ACAP1	61.86659	-0.0514	0.074508	-0.68985	0.490287	NA
D2HGDH	25.53186	-0.0514	0.08078	-0.63628	0.524592	NA
SND1-IT1	101.5897	0.051392	0.066058	0.777991	0.436574	NA
TOX	17.76629	-0.05139	0.082071	-0.62618	0.5312	NA
ZNRF2P1	20.30593	0.051387	0.082433	0.62338	0.533035	NA
LEMD3	218.3371	0.051384	0.04117	1.248104	0.211993	NA
SNW1	210.7331	0.051368	0.044895	1.144172	0.252552	NA
YIPF6	144.3669	0.051367	0.056234	0.913441	0.361011	NA
CASQ2	10.3538	-0.05135	0.080961	-0.63429	0.52589	NA
CNTROB	87.33677	-0.05135	0.058087	-0.88403	0.376679	NA
VWA7	19.42634	0.05134	0.084929	0.604503	0.545509	NA
P2RX2	3.394826	-0.05134	0.073	-0.70328	0.48188	NA
OR52W1	2.342286	0.051334	0.068855	0.745535	0.455948	NA
GSK3A	120.9331	0.051315	0.054358	0.944025	0.345157	NA
FPGT	39.39772	0.0513	0.076143	0.673732	0.500482	NA
TBC1D23	193.3077	-0.05129	0.043768	-1.17187	0.241248	NA
TNFSF14	0.73221	-0.05128	0.042433	-1.20859	0.226819	NA
RMRP	678.5137	-0.05128	0.080237	-0.63915	0.522724	0.787214
ZSCAN2	47.83719	-0.05128	0.062625	-0.81886	0.412866	NA
UFC1	39.54887	0.05127	0.077959	0.657646	0.510766	NA
ZNF613	35.78053	0.051268	0.07194	0.712653	0.476061	NA
SLC26A5	50.04965	0.051266	0.082859	0.618715	0.536104	NA
CXorf28	0.698577	-0.05126	0.045496	-1.12663	0.2599	NA
MIR4705	20.79739	-0.05125	0.080719	-0.63497	0.525445	NA
DNASE1L2	28.39111	0.051251	0.08434	0.607671	0.543406	NA
STX12	211.3658	-0.05124	0.060493	-0.84704	0.396973	NA
NPAT	208.9314	0.051237	0.047306	1.083103	0.278763	NA
CCDC86	17.58262	0.051222	0.080616	0.635389	0.525175	NA
ELOVL5	216.1709	0.051217	0.05248	0.975934	0.329097	NA

FSIP2	51.83332	-0.05122	0.080817	-0.63373	0.526259	NA
CFH	55.11775	-0.0512	0.084389	-0.60676	0.544011	NA
ABCA13	1.686888	-0.0512	0.053664	-0.954	0.340086	NA
GTF3C5	177.4082	-0.05119	0.062108	-0.82425	0.4098	NA
C1orf64	6.931942	-0.05118	0.081205	-0.63028	0.528513	NA
ZNF816	37.65849	0.051173	0.070953	0.721226	0.47077	NA
RAPGEF4-	34.27637	-0.05117	0.068422	-0.74788	0.454532	NA
DDX12P	3.870777	0.051165	0.071453	0.716072	0.473947	NA
LOC64320	4.568338	0.051163	0.081387	0.62864	0.529585	NA
RSPO3	56.04752	0.051162	0.070213	0.728663	0.466208	NA
MAPK13	93.04224	-0.05115	0.082958	-0.6166	0.537499	NA
HERC1	2159.915	0.051143	0.029788	1.716906	0.085996	0.410051
CATSPER3	4.333939	0.05114	0.074707	0.684538	0.493635	NA
ZNF599	25.93957	0.051126	0.074665	0.684738	0.493509	NA
REREP3	21.24466	0.051124	0.041076	1.244632	0.213267	NA
CRYZ	18.5888	0.051113	0.084935	0.601792	0.547313	NA
LOC10026	12.26866	-0.05111	0.083678	-0.61083	0.54131	NA
C1orf101	17.73074	-0.0511	0.083573	-0.61147	0.540888	NA
FCRLB	5.254246	0.0511	0.081092	0.630144	0.5286	NA
WISP2	2.815095	0.051095	0.075839	0.673734	0.500481	NA
SNORD116	1606.773	-0.05109	0.084911	-0.6017	0.547374	0.803817
MIR4284	0.744495	0.051089	0.048321	1.057291	0.290379	NA
LOC90499	0.333447	0.051087	0.032497	1.572041	0.115941	NA
LHFPL1	0.872582	-0.05108	0.049532	-1.03126	0.302417	NA
SPATA19	0.283739	-0.05107	0.031402	-1.62637	0.10387	NA
HCN2	173.8632	0.051068	0.068301	0.747691	0.454647	NA
TRNT1	122.2333	0.051067	0.046875	1.089431	0.275964	NA
LARP4	219.3393	0.051067	0.04671	1.093266	0.274277	NA
CACNG5	0.688939	-0.05106	0.045541	-1.12127	0.262172	NA
TMPRSS12	1.150291	0.051056	0.05261	0.970473	0.331811	NA
NUP133	246.5093	-0.05105	0.040285	-1.26725	0.205067	NA
SETD3	214.0774	0.051044	0.038844	1.314078	0.18882	NA
GCHFR	48.40641	-0.05104	0.067371	-0.75765	0.448662	NA
KLF8	54.98605	-0.05103	0.061058	-0.8358	0.40327	NA
AAGAB	68.34854	0.05101	0.073583	0.69323	0.488165	NA
SUSD1	19.20035	0.051008	0.081821	0.623405	0.533018	NA
TIMM44	106.86	-0.051	0.06163	-0.82753	0.407936	NA
BSN	2269.716	0.051001	0.052803	0.965865	0.334112	0.660929
ZNF517	26.65989	-0.051	0.072606	-0.70238	0.482444	NA
CYP3A7	3.793212	0.050988	0.07955	0.640959	0.521549	NA
TMEM70	51.99153	0.050986	0.070133	0.726989	0.467233	NA
USP17L2	5.062285	-0.05098	0.074051	-0.68851	0.491134	NA
SLC18A2	67.95914	0.050976	0.079107	0.644395	0.519319	NA
ZNF749	63.66996	0.050974	0.062854	0.810979	0.417378	NA
HIST1H2AL	0.541152	-0.05097	0.036139	-1.4104	0.158422	NA
GPR137C	255.3066	0.050962	0.051804	0.983747	0.32524	NA
NDUFA12	83.4537	-0.05095	0.077068	-0.66116	0.508512	NA

CDHR1	21.2633	0.050953	0.084788	0.600952	0.547872	NA
UBE2D1	55.33169	0.050945	0.073467	0.693444	0.488031	NA
RBCK1	242.8604	0.050945	0.060385	0.843665	0.398857	NA
CEP192	584.4538	0.050945	0.047798	1.065843	0.286495	0.620373
ASPHD1	57.86025	0.050925	0.067897	0.750035	0.453233	NA
IL22	0.626715	-0.05091	0.0413	-1.23275	0.217668	NA
AKAP2	1.418993	-0.05091	0.060971	-0.83491	0.403768	NA
CDKAL1	139.4084	0.050884	0.049133	1.035638	0.300371	NA
CCDC28B	27.70394	-0.05088	0.073462	-0.69257	0.488578	NA
DPF3	544.8724	0.050867	0.053712	0.947047	0.343615	0.666913
UQCC	103.733	-0.05082	0.049838	-1.01967	0.307883	NA
YAP1	97.06027	-0.0508	0.077491	-0.6556	0.512084	NA
NRDE2	222.3047	-0.0508	0.042144	-1.20537	0.228061	NA
SPRED2	134.7429	0.050795	0.0809	0.627875	0.530086	NA
RXFP2	2.0461	-0.05079	0.069219	-0.73381	0.463067	NA
KLK10	11.85296	-0.05079	0.081315	-0.62462	0.532219	NA
SCO1	58.48948	-0.05079	0.063509	-0.79975	0.423855	NA
DSC1	1.066536	-0.05079	0.052191	-0.97312	0.330496	NA
MRPS31P5	78.04462	-0.05077	0.058747	-0.8643	0.387423	NA
TREX2	1.190892	-0.05077	0.051101	-0.99358	0.32043	NA
LOC38902	6.174632	0.05077	0.079661	0.637327	0.523912	NA
RBFOX3	868.8845	0.050769	0.06036	0.841101	0.400291	0.708328
ACO1	177.6491	-0.05075	0.052788	-0.96133	0.336388	NA
C21orf2	73.41622	0.05074	0.066749	0.760165	0.447156	NA
BRSK1	359.5178	-0.05074	0.048831	-1.03901	0.298801	0.631658
SLC4A4	776.8204	-0.05072	0.068718	-0.7381	0.460453	0.754105
ARFGEF1	546.6571	0.05071	0.028692	1.76739	0.077163	0.396313
C4orf33	57.46096	0.05071	0.062225	0.814944	0.415104	NA
MED7	55.27796	0.0507	0.059586	0.850866	0.394844	NA
VIPR2	6.830067	0.050694	0.081287	0.623638	0.532865	NA
NAB2	88.37638	-0.05069	0.076252	-0.66473	0.506223	NA
VMO1	1.777673	-0.05068	0.066721	-0.75962	0.447484	NA
RBBP7	104.0131	-0.05068	0.057155	-0.88667	0.375257	NA
FGF11	6.861054	-0.05067	0.07975	-0.63542	0.525155	NA
ACR	0.970265	-0.05066	0.051457	-0.98458	0.324832	NA
PUS7	128.3709	0.050661	0.057607	0.879434	0.379166	NA
PCK1	3.129022	-0.05066	0.050466	-1.00386	0.315444	NA
SLC16A6	28.16133	0.050659	0.08493	0.596474	0.550858	NA
CXCL13	1.562133	0.050656	0.061166	0.828177	0.407571	NA
TCEA2	140.074	0.050653	0.046723	1.084116	0.278313	NA
KATNAL1	270.3128	0.05065	0.047588	1.064348	0.287171	NA
LCN12	3.36985	-0.05065	0.078063	-0.64882	0.516454	NA
TCFL5	83.59754	-0.05064	0.067182	-0.75377	0.450985	NA
GALNT18	152.4599	-0.05063	0.061967	-0.81704	0.413905	NA
SORBS1	1231.869	-0.05063	0.059905	-0.84512	0.398041	0.706622
BCL3	5.411474	-0.05062	0.07937	-0.63775	0.523633	NA
RGS19	12.68812	0.050618	0.082585	0.612923	0.539927	NA

ZNF787	34.12062	-0.05062	0.071736	-0.7056	0.480435	NA
SMAD4	432.7223	0.050614	0.043537	1.16254	0.245016	0.592704
FLJ22447	3.267046	-0.0506	0.075899	-0.66671	0.504957	NA
FAM194A	2.583519	-0.05059	0.07323	-0.69085	0.489657	NA
43349	194.9107	0.050588	0.052596	0.961826	0.336137	NA
IGSF21	378.555	0.050583	0.05799	0.872271	0.383061	0.70023
DNAJC17	130.7234	-0.05058	0.060182	-0.84049	0.400633	NA
MIR4762	1.293449	0.050581	0.058356	0.866767	0.38607	NA
BMP2K	134.7596	-0.05058	0.05648	-0.89554	0.370501	NA
RIC8B	203.4719	0.050573	0.041176	1.228214	0.219367	NA
FAM109A	17.17104	0.050563	0.08133	0.621699	0.53414	NA
POLD1	33.25039	-0.05055	0.08018	-0.63048	0.52838	NA
DKK1	1.219731	0.05055	0.05567	0.908013	0.363871	NA
LOC28376	4.824037	-0.05054	0.079985	-0.63185	0.527487	NA
GSK3B	618.8052	0.050537	0.037776	1.337821	0.180955	0.53091
SNORA2B	0.640953	-0.05053	0.045232	-1.11706	0.263967	NA
PACSIN2	270.9344	0.050524	0.039348	1.284035	0.19913	NA
TMEM191	1.082567	-0.05052	0.04799	-1.05276	0.292452	NA
CASP2	63.43763	0.050521	0.05813	0.869097	0.384794	NA
FAM127B	50.9114	0.05052	0.064575	0.782345	0.434012	NA
SSB	178.0084	0.05052	0.054605	0.925186	0.354869	NA
PPIL3	28.46887	-0.05051	0.078	-0.64754	0.51728	NA
RPAIN	123.0826	-0.05051	0.052807	-0.95643	0.338856	NA
NOM1	146.9744	0.050506	0.05105	0.989343	0.322495	NA
PYDC1	7.807629	-0.0505	0.084187	-0.59991	0.548568	NA
BZW1	76.53706	0.050502	0.06981	0.723418	0.469423	NA
43348	18.86157	0.050498	0.077425	0.65222	0.514259	NA
KIAA1524	39.99112	-0.05048	0.080725	-0.62536	0.531735	NA
TRMT2A	99.34503	-0.05048	0.053301	-0.94707	0.343602	NA
FKBPL	6.694287	0.050473	0.082026	0.615336	0.538333	NA
ALOX15B	1.290229	-0.05045	0.044895	-1.12371	0.261136	NA
TMEM147	15.58475	-0.05044	0.083797	-0.60197	0.547196	NA
EFR3B	488.3034	0.05044	0.058436	0.863174	0.388042	0.700889
SPR	3.775412	-0.05042	0.076871	-0.65592	0.511875	NA
LOC10050	2.980781	-0.05041	0.075207	-0.67032	0.502651	NA
WDR34	15.23287	-0.05041	0.080132	-0.62912	0.529272	NA
ADPGK-AS	4.518909	0.050409	0.08135	0.619658	0.535483	NA
PDE4A	175.2168	0.050408	0.055541	0.907581	0.3641	NA
RHPN1	22.84784	-0.05039	0.082514	-0.6107	0.5414	NA
PABPC1	766.9333	-0.05038	0.058615	-0.85943	0.390103	0.701813
TRIM41	84.64396	-0.05037	0.059304	-0.84943	0.395642	NA
DEFB136	0.606195	0.050365	0.044101	1.14203	0.253442	NA
PGF	2.298681	-0.05036	0.072202	-0.69754	0.485465	NA
CHIT1	3.491623	-0.05036	0.076155	-0.6613	0.508419	NA
USH1G	3.8198	0.050361	0.07707	0.653437	0.513474	NA
STX6	301.8609	-0.05036	0.040254	-1.25098	0.210943	NA
ARHGAP20	110.8121	-0.05035	0.053797	-0.93585	0.349351	NA

ZNF580	66.06886	-0.05034	0.062705	-0.8028	0.422091	NA
ACADM	159.8728	-0.05034	0.052841	-0.95262	0.340785	NA
TMEM79	18.79768	-0.05032	0.080522	-0.62496	0.531999	NA
NOX5	1.198431	0.05032	0.057882	0.86935	0.384656	NA
SCARF2	11.68067	-0.05032	0.084737	-0.59383	0.552626	NA
PHF10	124.3768	0.050317	0.055931	0.899634	0.368315	NA
C6orf136	65.9973	-0.05031	0.055657	-0.90384	0.36608	NA
FMN1	30.00638	-0.05029	0.084599	-0.59447	0.552196	NA
CTNNA2	184.641	0.050287	0.045873	1.096236	0.272976	NA
ASNS	204.9368	0.050278	0.042499	1.183029	0.236798	NA
POLB	282.3931	-0.05027	0.064937	-0.77412	0.438859	NA
ANXA2	14.61138	-0.05026	0.082872	-0.60652	0.544171	NA
ACTR3C	6.539366	-0.05026	0.084646	-0.59373	0.552696	NA
MIR31	0.546533	0.050256	0.042358	1.186457	0.235442	NA
ROR1	71.83951	-0.05025	0.084889	-0.59193	0.553895	NA
CCDC42B	0.416623	-0.05025	0.03895	-1.29003	0.197041	NA
TRIM16	16.26462	0.050233	0.081419	0.616974	0.537252	NA
ANKRD34B	0.862948	-0.05023	0.04985	-1.00767	0.313614	NA
TMEM108	47.71373	-0.05021	0.071149	-0.70575	0.480342	NA
STX4	66.63037	-0.05021	0.062413	-0.80452	0.421097	NA
FGF13	3.050865	-0.0502	0.073068	-0.68701	0.492074	NA
ST3GAL4	26.44772	0.050194	0.076241	0.658357	0.510308	NA
TOR1AIP1	234.1879	0.050192	0.051483	0.974911	0.329604	NA
UBE2MP1	0.332846	0.05018	0.032534	1.542377	0.122982	NA
MAFG-AS1	7.454766	-0.05018	0.084456	-0.59415	0.552412	NA
LOC40068	0.10874	-0.05017	0.019939	-2.5161	0.011866	NA
PRKAB1	47.79961	0.050168	0.071171	0.704887	0.480881	NA
NOP56	212.7652	-0.05016	0.044363	-1.13065	0.258201	NA
IL22RA1	4.255713	-0.05016	0.062469	-0.80292	0.42202	NA
LOC72840	30.73544	0.050153	0.072724	0.689637	0.490422	NA
FAM5C	85.98498	-0.05014	0.06333	-0.79181	0.428474	NA
MGC1602	7.546532	-0.05014	0.084347	-0.59445	0.552214	NA
OMG	80.84204	0.050138	0.0727	0.689648	0.490415	NA
NSMAF	189.4653	-0.05013	0.040819	-1.22813	0.219399	NA
APOL1	10.92609	0.05013	0.075307	0.665684	0.505613	NA
TTC7B	555.7116	0.050127	0.046802	1.071048	0.284148	0.618912
NAGS	7.888642	-0.05012	0.084649	-0.59212	0.553771	NA
SNORD114	19.34979	-0.05011	0.081824	-0.61246	0.540232	NA
DDA1	50.71904	-0.05011	0.058734	-0.85315	0.393577	NA
ZNF189	239.9853	0.050108	0.041761	1.199878	0.230187	NA
CYP51A1	172.3087	0.050103	0.062853	0.797151	0.425363	NA
ZNRF3-AS1	30.9481	-0.0501	0.076998	-0.65063	0.515288	NA
ZKSCAN3	44.78477	0.050092	0.061928	0.808869	0.41859	NA
RASGEF1A	88.71166	-0.05009	0.061707	-0.81172	0.416955	NA
UBIAD1	38.39788	-0.05008	0.065261	-0.76737	0.442862	NA
CNOT6L	391.7434	0.050075	0.047552	1.053057	0.292315	0.6257
MRPL34	28.35479	0.05004	0.082498	0.606563	0.544141	NA

RER1	128.3347	-0.05004	0.042121	-1.18797	0.234844	NA
SAV1	74.57275	0.050038	0.05625	0.88957	0.373697	NA
ZNF829	92.13738	0.050031	0.060702	0.824213	0.409819	NA
BCL6	886.0877	-0.05003	0.066228	-0.75537	0.450028	0.749346
CDC5L	340.2026	0.050018	0.04757	1.051456	0.293049	0.625901
MIR1185-1	0.916319	0.050015	0.050794	0.984667	0.324787	NA
OPRL1	28.21986	-0.05001	0.080435	-0.62174	0.534113	NA
PROX1	421.5402	0.050007	0.049258	1.0152	0.31001	0.640085
NELFA	103.8745	-0.05001	0.052078	-0.96021	0.336948	NA
CLEC4M	1.068113	0.050001	0.046347	1.078845	0.280657	NA
BRD3	254.0016	0.049994	0.052281	0.956262	0.33894	NA
DDB1	419.481	0.049994	0.04703	1.063011	0.287777	0.621249
LOC72901	5.674935	-0.04998	0.082489	-0.60595	0.544546	NA
SH3GL1	141.904	0.049983	0.058896	0.848659	0.396071	NA
ZNF804B	1.861915	0.049978	0.065328	0.765028	0.444255	NA
CDH13	3.473783	-0.04998	0.074059	-0.67483	0.499784	NA
COQ5	43.89993	0.049977	0.075559	0.661428	0.508338	NA
EMCN	10.76418	0.049963	0.084374	0.592164	0.553741	NA
GPIHBP1	11.12194	-0.04996	0.081633	-0.61198	0.540552	NA
ZNF236	357.1694	0.049939	0.041258	1.210424	0.226116	0.576749
SIGLEC1	2.217678	-0.04994	0.066062	-0.75594	0.449684	NA
RSBN1L	166.2091	0.049936	0.045526	1.096865	0.2727	NA
ALKBH2	20.72216	0.049936	0.080428	0.620873	0.534683	NA
FLT3LG	2.654631	0.049933	0.072067	0.69287	0.488391	NA
TUBA3FP	22.21236	0.049929	0.084796	0.588811	0.555988	NA
AIM2	4.044058	0.049921	0.078884	0.632839	0.526839	NA
C19orf80	0.648579	0.049919	0.043035	1.159982	0.246056	NA
B3GNTL1	32.13758	-0.04991	0.075858	-0.65799	0.510543	NA
TMEM200	1.952639	0.049904	0.065674	0.759865	0.447335	NA
PARP6	236.9905	0.049898	0.039273	1.270554	0.203887	NA
SLC7A1	179.5964	0.049892	0.057696	0.864741	0.387181	NA
MSANTD4	333.2608	0.049857	0.041463	1.202459	0.229186	NA
AGFG2	117.1872	0.049855	0.074818	0.666349	0.505188	NA
LOC72985	45.18784	-0.04985	0.067667	-0.73667	0.461321	NA
TEAD3	6.935537	0.049847	0.084797	0.587843	0.556638	NA
ARHGEF3	304.0504	0.049846	0.062618	0.796027	0.426016	NA
LOC10014	21.42535	-0.04984	0.078395	-0.63578	0.524923	NA
SYTL1	117.3151	-0.04984	0.078788	-0.63258	0.527008	NA
PRPF39	318.7885	-0.04983	0.057188	-0.8714	0.383534	NA
GBP7	2.046015	0.049833	0.066171	0.753096	0.451392	NA
ABHD16B	14.87376	-0.04983	0.084547	-0.58937	0.555614	NA
CDC42EP1	16.54383	-0.04983	0.084239	-0.59152	0.554175	NA
MED16	132.9265	0.049828	0.061892	0.80507	0.420779	NA
CADM2-AS	6.462577	0.049824	0.084464	0.589884	0.555269	NA
DDOST	78.64968	0.049821	0.060504	0.82343	0.410264	NA
OBSCN	1536.403	0.049814	0.067285	0.740342	0.459093	0.754035
ABCB10	86.37866	0.049771	0.057567	0.864564	0.387278	NA

CCDC88C	15.38023	0.04977	0.084382	0.589819	0.555312	NA
NME2	9.193989	-0.04976	0.084883	-0.58618	0.557752	NA
LOC10050	14.3182	-0.04975	0.083257	-0.5976	0.550108	NA
FBXO24	6.362965	-0.04975	0.083582	-0.59519	0.55172	NA
MTRR	97.38613	-0.04973	0.049218	-1.01032	0.31234	NA
USP17L1P	1.150538	0.04971	0.052633	0.944463	0.344933	NA
DDR2	55.98007	-0.0497	0.079638	-0.62403	0.532605	NA
CACYBP	319.0411	0.049695	0.073621	0.675009	0.49967	NA
MIR3176	0.653376	0.049691	0.042968	1.15646	0.247493	NA
GPRC5C	85.69453	0.049687	0.068938	0.720748	0.471064	NA
ATXN8OS	1.494441	0.049684	0.055759	0.891061	0.372897	NA
DUSP27	0.422439	-0.04968	0.036318	-1.36795	0.171329	NA
CYB5R2	12.2973	-0.04967	0.08412	-0.59048	0.554866	NA
PATL1	173.8888	0.049662	0.049938	0.994463	0.319997	NA
PTGFR	0.746929	-0.04966	0.043	-1.15492	0.248125	NA
NSUN5	27.83794	0.049656	0.074483	0.666673	0.504981	NA
RLTPR	62.93044	0.049648	0.064255	0.772667	0.439719	NA
DEFB134	0.589246	-0.04965	0.042751	-1.16126	0.245534	NA
YWHAH	817.0998	0.049637	0.07607	0.652509	0.514073	0.784091
MIR3925	0.268947	0.049634	0.029696	1.671395	0.094644	NA
OSBPL5	49.74731	-0.04963	0.075735	-0.65533	0.512254	NA
C16orf58	122.0862	-0.04963	0.059263	-0.83746	0.402334	NA
SGK3	2.0894	0.049623	0.069665	0.712302	0.476278	NA
PRPS1	85.18595	0.04962	0.063701	0.778948	0.43601	NA
RMND5B	75.43898	-0.04962	0.053084	-0.93472	0.34993	NA
LAMA1	22.24751	0.049615	0.084847	0.584759	0.55871	NA
SLK	793.8225	0.049612	0.047716	1.039745	0.298458	0.631658
IRF5	9.894554	-0.04961	0.08487	-0.5845	0.558883	NA
METTL21A	34.03411	0.049595	0.076686	0.646731	0.517806	NA
RPS6KA6	109.9326	0.049594	0.057341	0.864885	0.387102	NA
ABHD14B	35.79162	-0.04959	0.078255	-0.63374	0.526249	NA
LOC65227	64.1461	-0.04958	0.067013	-0.73984	0.459398	NA
IGSF8	266.1788	0.049575	0.043115	1.149819	0.250218	NA
ABL1	152.4826	-0.04957	0.049012	-1.01136	0.311846	NA
RSU1	71.15541	-0.04956	0.063654	-0.77866	0.43618	NA
GNAS	1442.44	0.049556	0.063136	0.784919	0.432501	0.736097
CYP21A1P	0.535252	0.049548	0.039754	1.246348	0.212637	NA
FOXD4	4.142638	-0.04955	0.074924	-0.66127	0.508439	NA
RNF112	437.1592	0.049543	0.059502	0.832625	0.405056	0.710565
MIR3177	1.363393	0.049543	0.060682	0.816437	0.41425	NA
C3orf62	64.05449	0.049526	0.074796	0.662148	0.507876	NA
ABCB7	113.064	-0.04952	0.050093	-0.98865	0.322837	NA
STAM2	199.6534	0.049519	0.048518	1.020644	0.307423	NA
ZBTB12	25.76883	-0.04952	0.076282	-0.64912	0.516264	NA
PABPC1L2	8.581785	0.049514	0.084936	0.582962	0.559919	NA
ZCCHC8	116.8382	0.049509	0.058767	0.842466	0.399527	NA
SON	1953.743	0.049502	0.028261	1.751584	0.079845	0.397742

SNN	243.5878	0.0495	0.058436	0.847081	0.39695	NA
AP4M1	15.05105	-0.04949	0.081799	-0.60497	0.545196	NA
SNORD114	6.382174	-0.04948	0.083148	-0.59507	0.551796	NA
EPN2	315.6979	0.049471	0.048667	1.016528	0.309378	NA
ZDHHC3	194.6549	-0.04947	0.058387	-0.8473	0.39683	NA
NOVA2	57.93347	0.049464	0.062139	0.796017	0.426022	NA
HDDC2	120.9356	-0.04945	0.069519	-0.71132	0.476888	NA
KIAA1279	192.554	0.049448	0.05689	0.869194	0.384741	NA
ABCG5	2.474862	0.049438	0.072432	0.682535	0.494901	NA
TLK1	384.0653	0.049428	0.039656	1.246418	0.212611	0.562782
RECK	78.48267	-0.04942	0.05539	-0.89222	0.372273	NA
ZNF418	207.4266	0.049407	0.055089	0.896852	0.369798	NA
CRYGN	0.212021	0.049399	0.02719	1.816783	0.06925	NA
KCNAB3	142.5957	-0.04939	0.06877	-0.71822	0.472622	NA
STK38	179.6333	-0.04938	0.067077	-0.73623	0.461593	NA
YY1AP1	486.8517	0.049374	0.051187	0.964568	0.334761	0.661635
PLXDC2	48.89767	-0.04936	0.07569	-0.65207	0.514358	NA
PAPSS1	127.8928	0.049354	0.062201	0.793467	0.427506	NA
APOL3	11.28713	0.049339	0.082981	0.594579	0.552125	NA
C14orf105	6.257649	-0.04934	0.080907	-0.60978	0.542005	NA
INPP5K	67.3969	-0.04933	0.061367	-0.80389	0.421463	NA
LOXHD1	9.035914	0.049325	0.074463	0.662414	0.507706	NA
ZNF214	19.85287	-0.04932	0.081484	-0.60522	0.545036	NA
COCH	4.28537	0.049315	0.077202	0.638777	0.522968	NA
CBR3	15.54994	-0.04931	0.084758	-0.58182	0.560689	NA
MIR1537	1.950828	-0.04931	0.068889	-0.71576	0.474137	NA
OR13D1	1.920764	-0.0493	0.065415	-0.75366	0.451051	NA
ATP1B2	1411.099	-0.0493	0.058306	-0.84554	0.39781	0.706622
DNAJC24	56.45747	-0.0493	0.059126	-0.83382	0.404383	NA
PSORS1C1	0.526534	0.049299	0.039782	1.239204	0.21527	NA
MIR5089	0.273493	-0.0493	0.029784	-1.65516	0.097891	NA
MIR124-3	0.351335	0.049295	0.03473	1.419393	0.155785	NA
FAF2	191.1658	0.049289	0.049323	0.999316	0.317641	NA
LCMT2	39.01815	0.049287	0.068864	0.715707	0.474172	NA
MEA1	81.52265	0.049259	0.069771	0.706018	0.480177	NA
TBC1D29	0.704393	0.049259	0.043982	1.119972	0.262726	NA
SULT1C2	0.83225	-0.04926	0.049444	-0.99624	0.319135	NA
MYO5A	1979.188	0.049247	0.043005	1.145134	0.252154	0.592704
SLC4A10	493.5496	-0.04924	0.051626	-0.95386	0.340154	0.663608
LOC10050	0.44579	-0.04924	0.03245	-1.51738	0.129172	NA
PLEK2	3.050595	-0.04923	0.075247	-0.65426	0.512946	NA
TMEM156	2.726249	0.049225	0.07175	0.686066	0.492671	NA
ETV3	67.98659	0.049222	0.061018	0.806675	0.419854	NA
SYNPR-AS1	6.844826	0.049221	0.083724	0.587899	0.5566	NA
CLEC4G	13.79305	0.049204	0.066513	0.73977	0.45944	NA
NMT1	314.4465	0.049172	0.045782	1.074035	0.282807	NA
AP1S1	29.74612	-0.04917	0.076818	-0.64007	0.522128	NA

ZNF578	37.31878	0.049168	0.080096	0.613861	0.539307	NA
GATAD1	204.8484	-0.04916	0.04335	-1.13402	0.256784	NA
VSTM2L	32.45876	-0.04915	0.083352	-0.58965	0.555423	NA
LOC10050	5.084652	0.049146	0.083372	0.589475	0.555543	NA
PVT1	1.222866	-0.04914	0.060314	-0.81468	0.415256	NA
RRN3P2	8.880789	0.049136	0.084715	0.580009	0.561909	NA
WDR86	2.692529	-0.04913	0.069944	-0.70239	0.482436	NA
PART1	3.591386	0.049123	0.072591	0.676707	0.498592	NA
C17orf72	1.575229	-0.04912	0.063274	-0.77629	0.43758	NA
NOSTRIN	14.72334	-0.04912	0.084934	-0.57828	0.563077	NA
HKDC1	9.085352	-0.04911	0.082696	-0.5939	0.552577	NA
EYA2	12.28861	0.049108	0.084548	0.580831	0.561354	NA
MIR4486	0.285858	0.049086	0.02984	1.644959	0.099978	NA
WHAMMP	40.26755	-0.04909	0.067712	-0.72492	0.468503	NA
ZNF484	346.245	0.04908	0.06893	0.712029	0.476447	0.759174
NUDC	287.8952	0.049053	0.054962	0.892489	0.372131	NA
ATAD2	76.62626	-0.04905	0.076266	-0.6431	0.52016	NA
SP3	321.3327	0.049046	0.040053	1.224544	0.220747	NA
ANKS3	92.60516	0.049044	0.0604	0.811991	0.416797	NA
UBE2E1	132.2089	0.049029	0.06478	0.756855	0.449137	NA
HK3	39.21198	0.049026	0.084619	0.57937	0.56234	NA
TMEM81	27.49976	0.049016	0.079295	0.618148	0.536478	NA
TSC22D3	240.5418	-0.04901	0.06543	-0.74909	0.453804	NA
RNASEK-C	1.870397	0.04901	0.06322	0.775237	0.4382	NA
KBTBD13	1.482239	-0.04901	0.053361	-0.91845	0.358383	NA
WSB1	313.3542	-0.04901	0.073056	-0.67085	0.502319	NA
GATA2	7.266156	-0.04901	0.083546	-0.58661	0.557465	NA
MIR3662	0.891608	-0.049	0.051673	-0.94819	0.343035	NA
NBL1	7.839043	0.048982	0.084897	0.576959	0.563967	NA
IGLL3P	0.971171	0.048976	0.033784	1.449678	0.147148	NA
OPA1	590.0833	0.048975	0.056324	0.869523	0.384561	0.70023
OR10AD1	15.43008	-0.04897	0.084908	-0.57678	0.564088	NA
KHSRP	486.1448	0.048964	0.037182	1.316896	0.187874	0.540017
CPT2	35.39148	-0.04896	0.066668	-0.73441	0.462697	NA
MIR4784	2.675535	-0.04896	0.074658	-0.65579	0.511962	NA
NPAS1	6.684409	-0.04896	0.084444	-0.57979	0.56206	NA
PRADC1	16.53334	-0.04895	0.081981	-0.5971	0.550439	NA
SAAL1	35.03462	-0.04895	0.070651	-0.69278	0.488449	NA
CPSF1	331.1028	0.048942	0.078785	0.621214	0.534459	NA
TOP2A	0.869119	-0.04894	0.037954	-1.28951	0.197221	NA
IMP4	51.545	-0.04894	0.066724	-0.73347	0.463271	NA
ZNF833P	14.67112	-0.04894	0.084787	-0.5772	0.563802	NA
IGFN1	2.462537	0.048936	0.066291	0.738211	0.460386	NA
KLHL13	25.50731	-0.04893	0.082534	-0.59288	0.553264	NA
BEGAIN	156.1013	-0.04893	0.05998	-0.81579	0.414622	NA
TPP1	115.7587	-0.04892	0.068974	-0.70928	0.478152	NA
RBM14	290.6858	0.048916	0.083303	0.587205	0.557066	NA

PARD6G-A	14.9596	-0.04891	0.084846	-0.57647	0.564301	NA
ZNF846	92.1463	0.048891	0.065606	0.74522	0.456139	NA
LINC00312	6.091775	-0.04889	0.074887	-0.65284	0.513862	NA
IL27RA	9.316142	0.048885	0.084935	0.575552	0.564918	NA
VOPP1	33.15215	0.048875	0.07628	0.640739	0.521693	NA
HCFC1	485.2382	0.048862	0.041553	1.175892	0.239638	0.589893
ZNF573	132.9054	0.048861	0.047644	1.025543	0.305107	NA
SH3BP5	128.4311	-0.04886	0.06212	-0.78655	0.431545	NA
PPP1R9A	521.6491	0.048855	0.042854	1.140046	0.254267	0.595569
CCS	57.11627	-0.04885	0.060918	-0.8019	0.422612	NA
FLJ31813	1.33586	0.04885	0.059069	0.826997	0.408239	NA
LOC10050	16.00019	-0.04885	0.081433	-0.59987	0.54859	NA
NR1H2	131.0876	0.048843	0.053898	0.906225	0.364817	NA
USP51	22.97529	0.048827	0.082606	0.59108	0.554467	NA
ZNF519	248.3621	0.04881	0.062251	0.784078	0.432995	NA
CCBL1	71.30138	-0.04881	0.06563	-0.74369	0.457063	NA
DDX27	299.2878	-0.04879	0.055653	-0.87673	0.380636	NA
UCK2	131.6399	0.048785	0.061767	0.789822	0.429632	NA
SKAP2	82.14837	0.048783	0.075493	0.646198	0.518151	NA
WT1	0.202922	-0.04878	0.02574	-1.89522	0.058064	NA
CCND2	155.2441	-0.04878	0.080826	-0.60351	0.54617	NA
ZNF521	1459.802	0.048775	0.038996	1.250774	0.211017	0.560498
MID1IP1	39.86433	-0.04877	0.074926	-0.65097	0.515065	NA
PITPNM2	292.0319	0.048773	0.073466	0.663889	0.506761	NA
WDR44	222.2055	0.048761	0.040119	1.215424	0.224204	NA
HIST1H2BJ	1.343559	-0.04876	0.051477	-0.94722	0.343525	NA
GK	35.8719	-0.04874	0.070422	-0.69218	0.488824	NA
TSFM	71.0227	0.048715	0.060282	0.808112	0.419026	NA
AMIGO2	3.983347	-0.04871	0.07466	-0.65248	0.514092	NA
LINC00658	0.530169	-0.04871	0.041466	-1.17479	0.240079	NA
GHITM	215.4585	-0.04871	0.069997	-0.69588	0.486503	NA
SKAP1	0.726364	0.048701	0.044824	1.086493	0.277261	NA
SLC9B1	7.288879	-0.0487	0.084507	-0.57629	0.56442	NA
ZNHIT2	21.96492	-0.0487	0.076805	-0.63406	0.526044	NA
TBCE	81.20048	-0.04869	0.054239	-0.89774	0.369327	NA
SCAF4	267.6854	0.048692	0.034563	1.408767	0.158904	NA
PKIB	294.0251	0.048689	0.067139	0.725203	0.468327	NA
RBKS	21.66728	-0.04868	0.078226	-0.62233	0.533727	NA
ARID2	445.7133	0.04868	0.033759	1.442003	0.149302	0.502511
ZFYVE26	338.532	-0.04868	0.045043	-1.08069	0.279837	0.616311
MIR2278	4.785933	0.048677	0.080918	0.601555	0.547471	NA
LINC00086	25.84961	0.048672	0.084305	0.577331	0.563716	NA
ACVR2A	235.8241	-0.04867	0.052254	-0.93142	0.351637	NA
NACC2	352.1965	0.048664	0.05626	0.864993	0.387042	0.700314
IER5L	9.05015	-0.04864	0.082496	-0.58962	0.555443	NA
LRFN2	50.66882	-0.04864	0.080493	-0.60424	0.545682	NA
SLC25A21	0.43104	0.048633	0.035324	1.376783	0.168579	NA

TMEM211	3.602944	-0.04863	0.076409	-0.63643	0.524494	NA
GPHN	283.7867	-0.04863	0.057993	-0.83852	0.401736	NA
MNAT1	155.2584	-0.04862	0.053851	-0.90282	0.36662	NA
SLC25A14	27.34136	-0.0486	0.074874	-0.64909	0.516278	NA
SNX24	38.03094	-0.04859	0.075905	-0.64014	0.522079	NA
HNRNPA3	900.1273	0.048585	0.031727	1.531329	0.125688	0.478748
ELN	133.5083	-0.04858	0.061578	-0.78886	0.430191	NA
CYP2U1	95.0105	0.048576	0.056958	0.852838	0.393749	NA
VSIG10	42.45167	-0.04856	0.072078	-0.67369	0.500506	NA
MXD4	439.3884	0.048557	0.056687	0.856583	0.391676	0.703039
GDAP1	366.728	0.048553	0.035385	1.372125	0.170024	0.523241
MPP4	20.46348	-0.04855	0.082074	-0.59157	0.554137	NA
RAB17	0.710002	0.048552	0.048621	0.998564	0.318006	NA
TCEB2	94.5667	-0.04855	0.070666	-0.68701	0.492077	NA
CETN4P	1.118609	-0.04853	0.049278	-0.98478	0.324732	NA
TMEM235	5.624461	-0.04853	0.078029	-0.62193	0.53399	NA
LOC93622	46.09122	-0.04852	0.067129	-0.72286	0.469767	NA
PADI1	0.353776	-0.04851	0.034628	-1.40094	0.161232	NA
CNOT8	129.9552	0.048509	0.051211	0.947223	0.343525	NA
ZNF878	7.24815	-0.04851	0.075741	-0.64045	0.52188	NA
LOC14583	3.772477	0.048505	0.07918	0.612597	0.540143	NA
WDR87	1.320781	-0.0485	0.050935	-0.95224	0.340977	NA
EEF1G	373.9598	-0.0485	0.069122	-0.7017	0.482869	0.764934
TMEM179	26.84884	0.048499	0.072591	0.668108	0.504064	NA
HERC4	227.3064	0.048495	0.059026	0.821584	0.411314	NA
CCDC175	486.4965	0.048492	0.045835	1.057966	0.290071	0.62335
C9orf47	2.568484	0.048487	0.067996	0.713087	0.475792	NA
PROSC	97.69447	0.048478	0.055461	0.874094	0.382067	NA
FAM13C	134.6735	-0.04846	0.056643	-0.85545	0.392303	NA
ZBED3	11.29557	-0.04845	0.083627	-0.57936	0.562344	NA
TMED1	18.87323	0.048443	0.082285	0.588729	0.556043	NA
SRRM2-AS	1.916881	0.04844	0.066466	0.728796	0.466127	NA
LOC55011	18.65432	0.048438	0.079854	0.606588	0.544124	NA
CHRNB4	0.190229	-0.04844	0.025665	-1.88722	0.059131	NA
AATK	390.2252	-0.04843	0.048038	-1.00813	0.313394	0.643552
PCDHGB6	110.2853	0.048425	0.082187	0.589206	0.555723	NA
TAS2R1	0.512075	-0.04842	0.040969	-1.18193	0.237232	NA
TTC14	364.7339	-0.04842	0.057502	-0.84198	0.399798	0.708008
SERPINF1	22.86382	0.048393	0.07735	0.625639	0.531552	NA
FAM149B1	114.7811	0.048387	0.053565	0.903338	0.366347	NA
VSTM4	29.74202	0.048385	0.081113	0.59651	0.550834	NA
NPM2	178.5026	-0.04838	0.065006	-0.74426	0.456721	NA
ALDH18A1	69.91203	-0.04837	0.0539	-0.89741	0.369501	NA
USP24	1345.091	-0.04836	0.037339	-1.29528	0.195223	0.544558
VPS39	304.8003	-0.04836	0.060736	-0.7962	0.425917	NA
NAP1L4	423.1643	0.048344	0.031486	1.535417	0.124681	0.47732
HNRNPH1	1161.666	0.048343	0.030373	1.591653	0.111463	0.460575

JAGN1	18.62199	0.048337	0.078041	0.619381	0.535665	NA
DESI2	154.0072	0.048337	0.052869	0.914277	0.360571	NA
B9D1	22.28032	-0.04834	0.076658	-0.63054	0.528344	NA
RHBDF1	41.75996	-0.04832	0.082712	-0.58425	0.559052	NA
TUBB2A	152.1692	0.048313	0.07404	0.652527	0.514061	NA
NAALADL2	0.515168	-0.04831	0.039732	-1.21596	0.223999	NA
LOC28643	49.10224	0.04831	0.067511	0.715581	0.47425	NA
COL6A1	712.5975	-0.04831	0.066397	-0.72755	0.466889	0.755844
NOP14	458.0179	-0.04831	0.054406	-0.88789	0.374601	0.695376
WARS2	28.34703	-0.04829	0.075837	-0.63673	0.524299	NA
RASGEF1C	221.0623	0.048283	0.05954	0.810932	0.417405	NA
CARD6	16.49756	0.048278	0.08447	0.57154	0.567634	NA
KIF22	52.20517	-0.04827	0.060296	-0.8006	0.423365	NA
PTOV1	312.6035	0.048267	0.070591	0.683757	0.494129	NA
C3AR1	4.898161	-0.04826	0.082054	-0.58817	0.556419	NA
CLEC2B	10.56657	-0.04824	0.082688	-0.58342	0.559607	NA
FAM132A	2.002589	0.048237	0.070097	0.688151	0.491358	NA
CHMP4C	0.951636	0.048234	0.048421	0.996147	0.319179	NA
CIITA	19.06512	-0.04823	0.084652	-0.56979	0.568821	NA
BARD1	8.383322	-0.04823	0.08441	-0.57139	0.567738	NA
TARS	152.0256	0.048225	0.047769	1.009548	0.312712	NA
FKBP9	50.08737	-0.04822	0.066695	-0.72301	0.469674	NA
ALDOA	804.0366	-0.04821	0.069281	-0.69587	0.486511	0.764934
LINC00642	0.567887	0.048206	0.040881	1.179172	0.23833	NA
ACVRL1	19.93206	-0.0482	0.084521	-0.57025	0.568511	NA
SLC24A1	84.03052	-0.0482	0.056234	-0.85708	0.3914	NA
TCEA3	19.53992	-0.04819	0.081587	-0.5907	0.55472	NA
ITGB1BP1	82.47819	0.048193	0.066895	0.720432	0.471259	NA
LOC10013	8.563368	-0.04819	0.084898	-0.56764	0.570276	NA
HDAC1	23.16251	-0.04818	0.078013	-0.61764	0.536816	NA
ARHGEF11	579.2581	0.048183	0.03589	1.342496	0.179435	0.53091
CD164	179.515	-0.04817	0.050655	-0.95097	0.341622	NA
POU2F3	1.320859	-0.04816	0.059824	-0.80507	0.420777	NA
HECTD4	3138.958	0.048162	0.040615	1.185816	0.235695	0.58613
ANXA2P2	0.287449	-0.04815	0.030086	-1.60036	0.10952	NA
AMIGO1	117.9125	0.048139	0.06405	0.751596	0.452294	NA
SLCO1A2	97.51206	-0.04813	0.084539	-0.56937	0.569104	NA
ALPK3	27.33821	-0.04813	0.08483	-0.5674	0.570443	NA
TRIO	2309.308	0.048131	0.04604	1.045426	0.295826	0.62855
GCNT3	1.223524	0.048126	0.054524	0.882665	0.377417	NA
MIR301A	0.667383	-0.04812	0.045948	-1.0472	0.295008	NA
KLHDC2	150.4911	-0.04811	0.043402	-1.1084	0.267689	NA
TNRC6B	1940.368	-0.0481	0.035618	-1.35044	0.176873	0.529395
LINC00176	91.28063	-0.04809	0.073393	-0.65523	0.512323	NA
THOC2	451.9833	-0.04808	0.034215	-1.40508	0.159999	0.516971
HSD17B13	22.58183	0.048071	0.082699	0.581272	0.561057	NA
EMP2	30.03601	-0.04806	0.082725	-0.58102	0.56123	NA

TCEB1	42.57705	-0.04806	0.077411	-0.62079	0.534735	NA
GALNT2	159.7854	-0.04805	0.057501	-0.8357	0.403322	NA
GLIS2	73.5465	0.048049	0.06426	0.747723	0.454627	NA
RANBP3L	33.20916	-0.04805	0.082814	-0.58017	0.561801	NA
RNF144B	29.47218	-0.04804	0.080597	-0.59611	0.5511	NA
COLEC10	1.081066	-0.04804	0.056427	-0.85143	0.394532	NA
LOC28618	0.638782	0.04804	0.039882	1.204538	0.228382	NA
CMBL	43.70122	0.048039	0.066594	0.721367	0.470684	NA
WNT3	5.611882	-0.04803	0.081711	-0.58785	0.556634	NA
CXADR	28.94029	0.048026	0.080735	0.594854	0.551941	NA
PDCD4	334.5943	0.048017	0.052132	0.921057	0.35702	0.680826
LINC00689	1.012843	-0.04802	0.048586	-0.98826	0.323023	NA
HMP19	527.0028	0.048016	0.062162	0.77243	0.43986	0.74018
ARHGAP32	316.0008	-0.04801	0.069948	-0.68642	0.492446	NA
AGPAT4	267.1457	-0.04801	0.053727	-0.89364	0.371517	NA
TEKT4	0.356556	0.048	0.033641	1.426834	0.153628	NA
ATP2C1	361.259	0.047998	0.039733	1.208008	0.227044	0.577485
HIST1H2B	2.618989	-0.048	0.072169	-0.66505	0.506016	NA
LINC00159	1.694078	0.047996	0.059259	0.809938	0.417976	NA
NPHP3	33.14908	-0.04798	0.070317	-0.68236	0.495011	NA
RFC2	36.28857	-0.04797	0.06896	-0.69556	0.486704	NA
TMEM65	143.9072	0.047962	0.060367	0.79451	0.426899	NA
GPATCH8	1178.206	-0.04795	0.040531	-1.18316	0.236746	0.586807
HOXC4	0.608022	0.047938	0.039832	1.203509	0.22878	NA
RPL32P3	72.99205	-0.04794	0.064291	-0.74564	0.455883	NA
GLRA3	1.861193	0.047936	0.059567	0.804739	0.42097	NA
ANKRD28	610.9584	-0.04793	0.043956	-1.0905	0.275494	0.614168
UEVLD	39.08515	-0.04793	0.078955	-0.60709	0.543794	NA
TMEM98	20.78379	0.047932	0.083931	0.571086	0.567941	NA
DPY30	47.27374	-0.04793	0.071743	-0.66811	0.504066	NA
TBC1D22B	60.73413	0.04793	0.058895	0.813818	0.415749	NA
CD274	22.03763	-0.04792	0.084609	-0.56639	0.57113	NA
SDAD1	152.4274	0.047911	0.049897	0.960196	0.336956	NA
MIR497	0.280643	0.047906	0.029863	1.604226	0.108664	NA
GAS2L1	63.39478	-0.04789	0.067656	-0.70778	0.479083	NA
SNORA2A	1.822154	0.047883	0.063605	0.75282	0.451558	NA
DCST2	3.099694	0.047876	0.06952	0.688661	0.491037	NA
CPS1	10.65307	0.047873	0.084892	0.563923	0.572806	NA
SPON1	95.81964	-0.04786	0.084916	-0.56357	0.573044	NA
DPH3	48.61567	0.04783	0.06969	0.686324	0.492509	NA
KLRC3	0.973898	-0.04782	0.055223	-0.86599	0.386495	NA
STAMBPL1	73.10514	0.047819	0.054198	0.882289	0.37762	NA
TPSD1	0.6255	0.04781	0.043263	1.105103	0.269115	NA
C17orf67	33.82653	0.047801	0.084804	0.563666	0.572982	NA
EGFEM1P	14.46107	-0.04779	0.084293	-0.56697	0.570734	NA
MIR3140	1.302497	0.047791	0.059374	0.804924	0.420863	NA
OR2L8	2.305129	-0.04779	0.046556	-1.02651	0.304651	NA

RBM26-AS	12.4299	-0.04778	0.081713	-0.58476	0.558706	NA
PKD1P1	667.0102	-0.04777	0.058623	-0.81494	0.415106	0.720147
EXOSC4	21.8098	0.047773	0.075651	0.631499	0.527714	NA
ETS2	92.75965	0.047772	0.074337	0.642632	0.520463	NA
KIAA1715	140.6794	0.04777	0.057537	0.830245	0.4064	NA
SFTPA1	4.116249	0.04777	0.076669	0.623064	0.533242	NA
C6orf226	3.948739	0.047769	0.075841	0.629862	0.528785	NA
LPHN3	508.7426	0.047769	0.047301	1.009902	0.312542	0.642968
HOOK2	121.2317	-0.04776	0.0739	-0.64622	0.518137	NA
RNF14	211.323	-0.04776	0.055208	-0.86501	0.387032	NA
DENND6B	81.50522	-0.04775	0.059931	-0.79682	0.425554	NA
DIAPH3	1.686719	-0.04775	0.057701	-0.82757	0.407913	NA
PROM1	6.640849	-0.04775	0.078635	-0.60724	0.543693	NA
CFTR	1.726494	-0.04775	0.063597	-0.75074	0.452806	NA
IMMT	184.2946	-0.04774	0.0504	-0.94729	0.343491	NA
HIST2H2A	30.7877	-0.04774	0.084937	-0.56208	0.574064	NA
KCNA4	2.823876	0.047731	0.06894	0.692353	0.488716	NA
ZRANB2	490.9068	-0.04773	0.04471	-1.06745	0.285768	0.619984
DLG1-AS1	0.691457	0.047724	0.044219	1.079269	0.280468	NA
MGC5734	89.1998	-0.04772	0.063762	-0.74846	0.454182	NA
TNPO1	466.9396	0.047719	0.035991	1.32588	0.18488	0.535489
UBFD1	189.8471	0.047664	0.052403	0.909575	0.363047	NA
KIAA1755	43.10268	-0.04766	0.079367	-0.60055	0.548142	NA
XPO6	205.4259	0.04766	0.045481	1.047917	0.294677	NA
PCGF2	46.48589	0.047654	0.065283	0.729956	0.465417	NA
KBTBD2	192.3183	-0.04765	0.046909	-1.01578	0.309732	NA
TMEM178	660.9997	0.047646	0.053795	0.8857	0.375779	0.695376
RNU11	275.4431	-0.04765	0.079525	-0.59913	0.549087	NA
GPR152	0.6861	-0.04763	0.044591	-1.06826	0.285404	NA
SLC6A15	244.4264	-0.04762	0.05328	-0.89368	0.371495	NA
DYRK2	95.07057	0.047615	0.057062	0.83445	0.404027	NA
SRGAP2	970.2793	0.047609	0.051005	0.933436	0.350595	0.674334
RNASEK	3.636417	-0.0476	0.079114	-0.60161	0.547434	NA
MYT1	1114.994	-0.04759	0.063637	-0.74785	0.45455	0.750995
LSS	198.2084	-0.04758	0.065937	-0.72162	0.470529	NA
C5orf42	876.7177	-0.04758	0.040233	-1.18263	0.236955	0.586807
FAM124B	0.958708	-0.04757	0.049477	-0.96147	0.336316	NA
TRPM2	240.5853	0.04757	0.081389	0.584485	0.558894	NA
CCDC154	27.17905	-0.04756	0.082475	-0.57669	0.564146	NA
MIS12	45.03567	-0.04756	0.064762	-0.73441	0.462701	NA
VPS37A	288.1076	0.047557	0.041366	1.149651	0.250288	NA
DYNLRB2	4.745691	0.047553	0.078642	0.604683	0.54539	NA
PHF16	29.85489	0.047546	0.071357	0.666312	0.505212	NA
PPAPDC2	83.2436	0.047546	0.051468	0.923788	0.355597	NA
SLC38A2	487.6992	0.047536	0.08101	0.586799	0.557339	0.808857
COPZ1	54.65171	0.04753	0.068453	0.694345	0.487466	NA
RUFY2	551.2266	-0.04753	0.043453	-1.09381	0.274039	0.613511

TMEM59	183.2386	-0.04753	0.065667	-0.72377	0.46921	NA
ZNF665	41.87787	0.047516	0.079402	0.598428	0.549554	NA
PIGP	16.18994	0.047515	0.083247	0.570779	0.56815	NA
TRPM3	1040.728	0.047508	0.041623	1.141401	0.253703	0.594863
ELMO1	328.2793	-0.04751	0.074027	-0.64174	0.521039	NA
BTF3	152.8979	-0.0475	0.065056	-0.73013	0.465308	NA
GPD1	27.74496	-0.0475	0.081437	-0.58326	0.559719	NA
IDNK	28.54035	-0.0475	0.084076	-0.56494	0.572117	NA
ALAD	162.9306	-0.04749	0.045235	-1.04995	0.293742	NA
NAT1	1.839841	0.047488	0.05741	0.827163	0.408145	NA
LINC00174	50.68446	-0.04748	0.071982	-0.65963	0.509493	NA
LAMA4	101.9337	-0.04747	0.079231	-0.59916	0.549068	NA
ATPIF1	283.7942	-0.04746	0.054269	-0.87458	0.381804	NA
AQP6	3.142145	0.047462	0.069989	0.678141	0.497682	NA
RHNO1	17.11351	-0.04745	0.0832	-0.57037	0.56843	NA
MTFR1L	82.62783	-0.04745	0.050662	-0.93663	0.348948	NA
YAF2	259.9345	-0.04744	0.044733	-1.06055	0.288894	NA
KRTCAP3	18.89815	0.047429	0.084915	0.558538	0.576477	NA
CHRD	393.7084	0.04741	0.068877	0.688332	0.491244	0.768948
GAPT	1.084899	-0.04741	0.050775	-0.9337	0.350456	NA
R3HCC1	76.03397	-0.04741	0.056243	-0.84288	0.399298	NA
CCDC65	18.08819	-0.04741	0.079291	-0.59787	0.549927	NA
SNRPB2	56.57827	-0.04738	0.067195	-0.70509	0.480755	NA
POLR2M	29.51445	0.04736	0.07623	0.621279	0.534416	NA
PPIA	238.9933	0.047345	0.067321	0.703281	0.481881	NA
IQSEC2	485.8586	-0.04734	0.05429	-0.87204	0.383189	0.70023
RPS6KC1	195.8187	0.047341	0.043384	1.091196	0.275187	NA
MIR4765	0.154375	0.047337	0.023662	2.000581	0.045438	NA
ACAT2	37.37452	0.047324	0.071669	0.660315	0.509051	NA
DHX30	363.9564	0.047318	0.043071	1.098591	0.271946	0.612663
ZWILCH	15.21491	0.047307	0.084538	0.559593	0.575757	NA
MIR29C	29.37223	-0.0473	0.082529	-0.57313	0.566559	NA
INHBE	2.678823	-0.0473	0.071044	-0.66574	0.505575	NA
TRIP12	966.2847	0.047291	0.031989	1.478329	0.13932	0.490761
LOC44092	0.858166	0.047287	0.049173	0.961643	0.336229	NA
NADKD1	151.8496	0.047277	0.070107	0.674357	0.500085	NA
LOC10050	6.465418	-0.04727	0.083423	-0.56666	0.570947	NA
CACNA1F	9.337697	0.047268	0.08489	0.55681	0.577657	NA
SLC35A1	89.30496	0.047268	0.059144	0.799197	0.424176	NA
NMRAL1	41.87457	-0.04726	0.080986	-0.58357	0.559512	NA
EIF5	705.1567	0.047251	0.049741	0.949951	0.342137	0.665757
FOXP2	14.00465	-0.04724	0.084928	-0.55627	0.578029	NA
ADAD2	0.205854	-0.04721	0.02599	-1.81655	0.069286	NA
ZNF487P	35.48485	-0.04721	0.071945	-0.65614	0.511736	NA
KCNK12	55.07672	0.047194	0.05911	0.798413	0.424631	NA
TNFAIP8L2	7.075823	-0.04719	0.084648	-0.55752	0.577169	NA
ASB5	2.109968	0.047193	0.065063	0.725347	0.468239	NA

ACSF2	101.2326	-0.04719	0.07095	-0.66515	0.505954	NA
PSMD3	212.4409	-0.04719	0.043074	-1.09554	0.273282	NA
AK5	30.91183	-0.04716	0.082698	-0.5703	0.568475	NA
ARHGDIA	170.1055	-0.04715	0.04584	-1.02864	0.303651	NA
GTF2F2	42.53009	-0.04715	0.061182	-0.77069	0.440892	NA
C9orf106	1.209807	-0.04715	0.054468	-0.86564	0.386689	NA
PLSCR3	0.901832	-0.04715	0.054118	-0.87118	0.383654	NA
IGFBP1	0.202329	-0.04714	0.025877	-1.82189	0.068472	NA
SSBP1	155.2087	-0.04713	0.050412	-0.93488	0.349851	NA
COA6	27.54595	-0.04712	0.077855	-0.60527	0.544998	NA
MGME1	21.37569	-0.04712	0.075731	-0.62219	0.533817	NA
ZNF211	111.5182	0.047116	0.056922	0.827738	0.407819	NA
LMBR1	329.3151	0.047109	0.037292	1.263263	0.206495	NA
FAM206A	30.45594	-0.0471	0.06989	-0.67397	0.500329	NA
GABRD	385.8449	0.047101	0.064078	0.735064	0.462301	0.755844
MIR4480	2.061904	-0.0471	0.071035	-0.66305	0.507298	NA
HPSE2	21.05248	-0.04709	0.084775	-0.55547	0.578571	NA
GUSBP4	21.90329	-0.04707	0.076894	-0.61218	0.540418	NA
VIP	5.950217	0.047066	0.073463	0.640672	0.521736	NA
MFGE8	25.51685	-0.04706	0.081254	-0.57919	0.562463	NA
MBOAT4	4.514812	-0.04706	0.069844	-0.67378	0.500453	NA
FXYD2	0.972771	-0.04706	0.050525	-0.93137	0.351664	NA
PBOV1	75.75154	-0.04705	0.066198	-0.71079	0.477211	NA
GALC	117.8931	0.047045	0.065346	0.719934	0.471565	NA
CETP	1.07035	0.047031	0.047257	0.995204	0.319637	NA
TECTA	29.32016	-0.04702	0.072738	-0.64647	0.517973	NA
SLC9A3R1	155.8115	0.047014	0.054762	0.85851	0.390611	NA
PARPBP	4.423198	0.047004	0.080209	0.58602	0.557862	NA
PPP1R1A	49.64449	0.046987	0.083929	0.559842	0.575587	NA
BLOC1S4	10.34833	-0.04697	0.083998	-0.55922	0.576011	NA
PC	194.4675	0.046968	0.063206	0.743104	0.457419	NA
CPE	1690.734	-0.04697	0.065782	-0.71399	0.475236	0.759174
ZNF544	347.2169	0.046953	0.050415	0.931321	0.351687	0.675579
GAL3ST4	6.925491	0.046945	0.083489	0.562295	0.573915	NA
TMEM14C	49.61524	-0.04694	0.079007	-0.59416	0.552406	NA
CCL28	0.46904	-0.04694	0.040677	-1.15402	0.248491	NA
TRPC7	0.49147	-0.04694	0.036829	-1.27459	0.202454	NA
SGCD	24.26398	-0.04694	0.083205	-0.56409	0.572692	NA
PLEKHM1	192.0375	0.046919	0.067469	0.695418	0.486793	NA
DUSP15	5.844787	-0.04692	0.083451	-0.56222	0.573966	NA
PPP2CB	276.5609	0.046908	0.067665	0.693237	0.488161	NA
ZNF773	25.56489	0.046894	0.07218	0.649685	0.515896	NA
IPP	41.55011	-0.04689	0.072467	-0.64709	0.517573	NA
CUX1	1485.445	0.04689	0.037739	1.242483	0.214058	0.562782
TUB	516.2026	-0.04689	0.036922	-1.26987	0.20413	0.553056
TUT1	38.13194	-0.04687	0.071166	-0.65855	0.510184	NA
UVRAG	191.5107	0.046862	0.049452	0.947624	0.343321	NA

SLITRK1	69.63778	-0.04686	0.084923	-0.55179	0.581093	NA
OCIAD2	19.2301	-0.04685	0.081288	-0.5764	0.564347	NA
LOC44043	72.90496	0.046849	0.065888	0.711047	0.477055	NA
PNKP	69.79938	-0.04685	0.066519	-0.70427	0.481262	NA
ANP32E	552.1832	0.046832	0.06252	0.749071	0.453814	0.750995
RERG	4.346734	-0.04683	0.078462	-0.59682	0.550626	NA
KIAA1147	568.074	0.046825	0.048394	0.967589	0.33325	0.660832
HNRNPK	1021.074	0.046819	0.053491	0.875255	0.381435	0.70023
CERS4	81.0915	0.046813	0.054607	0.857269	0.391296	NA
KLF13	691.0896	0.046813	0.063774	0.734049	0.462919	0.755844
HLA-L	102.3192	-0.04681	0.063849	-0.73313	0.46348	NA
SNORA79	3.739167	-0.0468	0.076839	-0.60913	0.54244	NA
PPIAL4G	0.780447	0.046804	0.049017	0.954851	0.339653	NA
PYGM	38.30792	0.046803	0.084021	0.557038	0.577502	NA
PBRM1	605.4378	0.046801	0.033869	1.38182	0.167027	0.521454
PPP1R42	0.531267	-0.0468	0.040889	-1.14457	0.252389	NA
MTMR14	116.4043	-0.0468	0.046648	-1.0032	0.315767	NA
SMYD2	47.63228	0.046788	0.06368	0.734746	0.462494	NA
USP32P2	100.8302	0.046787	0.057788	0.809629	0.418154	NA
UPF3B	279.4037	-0.04678	0.054981	-0.85079	0.394888	NA
ALOX15	1.429724	0.046771	0.04592	1.018528	0.308427	NA
LOC14448	3.014153	0.046754	0.076186	0.613678	0.539428	NA
EGF	0.911355	-0.04675	0.050209	-0.9311	0.351803	NA
LANCL1	570.0499	0.046743	0.074825	0.624698	0.53217	0.794571
MEI1	4.48029	0.04674	0.081426	0.574023	0.565952	NA
PCDHA10	62.53802	0.046738	0.081889	0.570748	0.568171	NA
DDO	6.411218	-0.04674	0.084335	-0.55419	0.57945	NA
CDC20	2.860097	-0.04673	0.075424	-0.61961	0.535517	NA
FGF18	1.669367	0.046717	0.061037	0.765391	0.444039	NA
LHFPL4	79.95561	0.046713	0.056215	0.830972	0.40599	NA
HJURP	0.281481	-0.04671	0.028262	-1.65257	0.098417	NA
PKMYT1	8.292918	0.046696	0.082134	0.568531	0.569675	NA
ARFIP2	95.52901	-0.04669	0.049181	-0.9493	0.342468	NA
BIRC5	2.193151	-0.04668	0.070972	-0.65767	0.510751	NA
GAB3	8.235054	-0.04668	0.083796	-0.55701	0.577519	NA
YIF1A	28.80385	0.046672	0.074169	0.629266	0.529175	NA
MLC1	247.9505	-0.04667	0.079035	-0.5905	0.554855	NA
CADM1	950.0063	0.046642	0.040623	1.148162	0.250902	0.592704
TREM1	0.327374	-0.04664	0.032478	-1.43606	0.150985	NA
P4HB	251.9406	0.046632	0.049978	0.933045	0.350797	NA
FAM26F	0.719829	-0.04663	0.042172	-1.10566	0.268873	NA
INSR	574.7796	-0.04663	0.039378	-1.18408	0.23638	0.586807
MROH7	3.901789	-0.04663	0.076554	-0.60905	0.542493	NA
TRNAU1AP	81.1523	-0.04662	0.062376	-0.74742	0.454811	NA
LOC10027	37.98633	-0.04662	0.082423	-0.56556	0.571693	NA
DMBT1	0.650042	0.046612	0.041235	1.130399	0.258308	NA
LOC38881	0.290923	-0.04661	0.031707	-1.46993	0.141581	NA

ZNF497	49.36058	0.046604	0.063773	0.730785	0.464911	NA
PPP1R15A	115.8195	0.046604	0.075238	0.619418	0.535641	NA
IMPAD1	492.996	0.046602	0.065231	0.714418	0.474969	0.759174
MIIP	30.60217	0.0466	0.071842	0.648646	0.516567	NA
BCL2A1	0.933803	-0.0466	0.043628	-1.06808	0.285485	NA
INPP5A	317.1191	-0.0466	0.066682	-0.69881	0.484673	NA
CEL	16.09135	0.046592	0.083401	0.558654	0.576398	NA
PDCD6	81.54237	-0.04659	0.068658	-0.67859	0.497397	NA
TADA3	99.0852	-0.04659	0.051874	-0.89813	0.369114	NA
PARP1	374.0646	-0.04659	0.04861	-0.95842	0.337852	0.66305
DCAF6	314.783	0.046583	0.043691	1.066204	0.286331	NA
SMG1P1	23.83695	0.046564	0.084925	0.548297	0.583488	NA
BHMT2	12.35666	0.046564	0.084687	0.549829	0.582436	NA
GALNT10	54.30171	-0.04656	0.067381	-0.69098	0.489576	NA
WDR92	65.19034	0.046549	0.059927	0.776764	0.437298	NA
RAB40C	138.8591	-0.04655	0.061144	-0.76125	0.44651	NA
C7orf62	0.405157	-0.04654	0.038018	-1.22424	0.220862	NA
RPA2	81.78909	-0.04654	0.068055	-0.68388	0.494051	NA
DIP2A-IT1	3.828528	-0.04654	0.079014	-0.58898	0.555875	NA
KCTD20	189.3798	0.046537	0.039306	1.18397	0.236425	NA
SLC13A3	30.57985	-0.04652	0.084897	-0.54799	0.583697	NA
NKX2-5	0.160589	-0.04651	0.021288	-2.18457	0.02892	NA
DYNC1LI2	807.3732	0.046501	0.042932	1.08311	0.27876	0.61539
CSTF3	157.4707	-0.0465	0.047384	-0.98125	0.326468	NA
LOC10012	1.786798	0.046493	0.062308	0.746178	0.45556	NA
DUSP9	1.067437	-0.04649	0.047311	-0.98266	0.325773	NA
IQCB1	107.6374	-0.04647	0.057192	-0.81259	0.416453	NA
CLEC16A	422.302	-0.04647	0.044423	-1.04605	0.29554	0.628543
TOM1L2	382.9938	-0.04647	0.048761	-0.95294	0.340623	0.66395
NUPL1	195.001	0.046457	0.043915	1.057899	0.290101	NA
RAB30	73.17997	0.046437	0.053127	0.874076	0.382077	NA
HLA-E	201.5561	-0.04643	0.074556	-0.6227	0.533483	NA
GSTM2P1	1.056603	-0.04643	0.052888	-0.87781	0.380049	NA
SLC35E3	56.34704	-0.04642	0.059014	-0.7865	0.431573	NA
LRRC47	133.1752	0.046403	0.052429	0.885063	0.376123	NA
LOC40002	100.3632	-0.04639	0.059956	-0.77381	0.439045	NA
NRSN1	71.45448	0.046388	0.08213	0.564818	0.572197	NA
CHST1	15.36093	-0.04638	0.083639	-0.55455	0.579203	NA
DAPL1	0.290377	-0.04638	0.030084	-1.54155	0.123183	NA
ANPEP	3.557743	0.046355	0.075346	0.615225	0.538406	NA
IMPDH2	136.3914	-0.04635	0.04826	-0.96049	0.336807	NA
C1orf173	303.7833	0.046329	0.054236	0.854212	0.392987	NA
NPTX1	1590.442	-0.04631	0.064424	-0.71891	0.472195	0.759174
LOC10013	8.972214	-0.04631	0.082995	-0.55801	0.57684	NA
UBN2	1220.04	0.046309	0.029525	1.568472	0.116771	0.472599
TPI1P3	0.496894	-0.04629	0.040769	-1.13533	0.256235	NA
GSTM1	8.39849	-0.04628	0.033483	-1.38228	0.166884	NA

MRPL39	81.24876	0.046281	0.051295	0.902242	0.366928	NA
C5orf54	67.39096	0.046281	0.066042	0.70078	0.483441	NA
ERVK13-1	342.4258	-0.04627	0.063393	-0.72996	0.465415	0.755844
SMLR1	1.953287	0.046274	0.06368	0.726662	0.467433	NA
SNORD114	2.290521	-0.04627	0.071524	-0.64695	0.517666	NA
NKX6-2	22.50678	-0.04627	0.084907	-0.54496	0.585781	NA
PLAC8	2.239592	-0.04626	0.057845	-0.79981	0.423821	NA
EMC6	1.848979	0.046264	0.068143	0.678919	0.497189	NA
DIO2	29.92854	0.04626	0.082724	0.559203	0.576023	NA
DDX11L2	6.876233	-0.04625	0.082216	-0.56249	0.573786	NA
DNER	260.0302	-0.04624	0.081186	-0.56959	0.568954	NA
SNORD114	1.744198	-0.04624	0.062866	-0.73551	0.462026	NA
NCOA3	283.1614	0.046239	0.038351	1.205686	0.227939	NA
ARNTL	134.0352	-0.04624	0.054936	-0.84168	0.399965	NA
PRKRIR	66.59159	0.046237	0.064224	0.719939	0.471562	NA
SEMA4C	153.8534	0.046237	0.060413	0.765348	0.444065	NA
CELF5	209.0988	0.046234	0.056066	0.824638	0.409577	NA
ITGA2	40.22622	0.04623	0.072966	0.633577	0.526357	NA
ALOX15P1	1.296712	-0.04622	0.057392	-0.80535	0.420616	NA
TXLNA	286.5192	0.04622	0.05654	0.817467	0.413662	NA
CMC4	10.91772	-0.04622	0.083508	-0.55343	0.579972	NA
KCNB1	163.7293	0.046214	0.06546	0.705994	0.480192	NA
PERP	33.17747	-0.04621	0.075454	-0.61242	0.540262	NA
C8orf12	4.19612	-0.04621	0.073886	-0.62538	0.531719	NA
SCARB2	447.0647	0.046204	0.056385	0.819444	0.412533	0.717656
LOC40065	0.150356	-0.0462	0.021114	-2.18827	0.02865	NA
POLE4	9.641855	-0.0462	0.083874	-0.55082	0.581755	NA
FAM212A	7.51341	-0.04619	0.080979	-0.57044	0.568379	NA
PLCG1	1515.727	-0.04619	0.077686	-0.59462	0.552099	0.805737
MIR648	29.14324	-0.04619	0.084071	-0.54944	0.582701	NA
PANK2	163.6886	0.046181	0.054498	0.847392	0.396777	NA
COPS8	81.48552	-0.04617	0.067906	-0.67996	0.49653	NA
ME3	125.2342	-0.04617	0.057245	-0.80657	0.419916	NA
JMJD1C	3018.383	0.046167	0.036429	1.267322	0.20504	0.553506
SNORD57	20.57988	-0.04617	0.083773	-0.55109	0.58157	NA
VPS54	365.7958	0.046162	0.04452	1.036876	0.299794	0.631658
TLL1	460.2987	-0.04615	0.053332	-0.86539	0.386825	0.700314
ZNF182	125.2846	-0.04614	0.054823	-0.8417	0.399956	NA
TAGLN2	31.28296	-0.04614	0.08491	-0.54338	0.586865	NA
CLIC4	133.7861	-0.04612	0.082484	-0.55917	0.576048	NA
ARHGAP28	6.804936	0.04612	0.083094	0.555028	0.578876	NA
SLITRK5	305.084	-0.04611	0.075333	-0.61214	0.540446	NA
NEURL	241.9686	-0.04609	0.061737	-0.74649	0.45537	NA
CLDN14	1.220592	-0.04607	0.053802	-0.85635	0.391803	NA
HERC3	611.4107	-0.04607	0.039848	-1.15609	0.247643	0.592704
SIKE1	199.864	0.046068	0.049756	0.925894	0.354501	NA
NFKB1	107.0254	0.046067	0.047248	0.974997	0.329562	NA

MAMDC4	252.8558	0.046049	0.079246	0.581094	0.561177	NA
ZBTB8B	16.34603	0.046038	0.084697	0.543563	0.586743	NA
ID4	250.4731	-0.04603	0.072583	-0.63422	0.525938	NA
PHF17	337.3538	0.046024	0.042301	1.087995	0.276597	0.61452
KSR1	198.3861	-0.04602	0.067484	-0.68194	0.495275	NA
LMX1A	0.932994	0.046013	0.050389	0.913154	0.361162	NA
PIGM	47.52399	0.046012	0.061704	0.745694	0.455852	NA
GTF2H2D	0.410556	0.04601	0.036031	1.276932	0.201626	NA
BGLAP	1.056828	0.045985	0.054624	0.841837	0.399879	NA
KDM6A	224.8004	-0.04598	0.061254	-0.75067	0.452854	NA
OSBPL3	10.7338	-0.04597	0.084671	-0.5429	0.587198	NA
ANKRD20A	5.879884	-0.04597	0.07636	-0.60199	0.547183	NA
NEK6	102.0196	-0.04597	0.062664	-0.73354	0.463226	NA
CCL4	0.186967	-0.04595	0.023419	-1.96204	0.049758	NA
ABCA12	1.127174	-0.04595	0.052784	-0.87047	0.384042	NA
NPPA	3.358471	0.04594	0.0741	0.619964	0.535282	NA
FLJ31662	0.394482	-0.04594	0.033392	-1.37577	0.168893	NA
C17orf66	17.07831	0.045926	0.083385	0.550768	0.581793	NA
SNCA	262.1687	0.045909	0.071458	0.64246	0.520575	NA
RHOV	2.96332	0.045905	0.073917	0.62103	0.53458	NA
MYOCD	2.057335	-0.0459	0.067079	-0.68425	0.493815	NA
MIR5010	1.084968	0.045885	0.055321	0.829427	0.406863	NA
SNX10	128.9419	0.045879	0.066101	0.694084	0.48763	NA
SIRPB1	11.05791	-0.04588	0.052268	-0.87771	0.380104	NA
HIRA	219.2488	0.045874	0.043646	1.051048	0.293236	NA
DVL1	157.5606	0.045871	0.056322	0.814435	0.415396	NA
VGLL4	99.91434	-0.04587	0.057891	-0.79232	0.428175	NA
BCORP1	0.552922	0.045866	0.03094	1.482394	0.138235	NA
SNORD18A	0.874693	-0.04585	0.050248	-0.91257	0.361471	NA
CALB2	76.18095	0.045854	0.076304	0.600935	0.547883	NA
PURG	155.4563	-0.04585	0.051635	-0.88796	0.374562	NA
SZT2	583.9479	-0.04585	0.050845	-0.90168	0.367225	0.690261
LANCL3	3.349942	0.045839	0.073311	0.62526	0.5318	NA
P2RY13	22.74779	-0.04583	0.078565	-0.58335	0.559659	NA
ZNF623	191.9746	0.045825	0.048447	0.945892	0.344204	NA
REPIN1	165.1753	-0.04582	0.048659	-0.94173	0.346331	NA
HTR2A	2.862038	0.045821	0.073817	0.620735	0.534774	NA
LSAMP	1404.415	0.045816	0.061975	0.739266	0.459745	0.754035
MLK7-AS1	1.303243	-0.04582	0.060308	-0.7597	0.447434	NA
MPLKIP	27.20332	0.045812	0.07393	0.619675	0.535472	NA
MTMR8	13.77834	-0.04581	0.082639	-0.55431	0.579365	NA
SLC22A6	1.390275	-0.04581	0.056846	-0.80581	0.420352	NA
FAM89B	36.7408	-0.04581	0.066973	-0.68393	0.49402	NA
DYRK1A	638.7161	0.0458	0.034187	1.339694	0.180345	0.53091
GPR27	9.527063	-0.0458	0.084936	-0.5392	0.589746	NA
SEH1L	103.1156	0.045793	0.059106	0.774763	0.43848	NA
HIATL1	65.37399	0.04579	0.063715	0.71866	0.47235	NA

METTL2A	31.1407	-0.04579	0.073461	-0.62329		0.533096	NA
USP27X	133.8753	0.045783	0.06018	0.760771		0.446794	NA
ZPBP	1.489834	0.045782	0.061631	0.742829		0.457585	NA
RPE	97.36031	-0.04577	0.05686	-0.80492		0.420865	NA
PHC1	98.98909	-0.04576	0.054502	-0.83961		0.401128	NA
RAB3IL1	8.528459	-0.04575	0.084246	-0.54309		0.587069	NA
UHRF1BP1	328.1278	0.045736	0.036263	1.261215		0.207231	NA
DNAJB8-A	0.840655	-0.04572	0.050784	-0.90031		0.367956	NA
LRRN4CL	4.431255	0.045706	0.079657	0.573787		0.566112	NA
HSD3B7	76.74074	-0.0457	0.083855	-0.54504		0.585725	NA
EFCAB11	17.67828	0.045702	0.081311	0.562062		0.574074	NA
GRM3	85.61289	0.0457	0.067956	0.672492		0.501271	NA
POLH	129.5165	0.045697	0.057616	0.793134		0.4277	NA
SNRPE	34.26139	0.045692	0.067883	0.673097		0.500886	NA
LIF	0.604857	-0.04568	0.032202	-1.41864		0.156005	NA
LOC28371	2.959164	0.04568	0.074816	0.61057		0.541485	NA
LRRC3	1.390576	-0.04568	0.05616	-0.81338		0.415998	NA
MPND	71.12253	0.045666	0.065112	0.701353		0.483083	NA
SCGB1B2P	2.84337	-0.04567	0.075292	-0.60652		0.544168	NA
C21orf15	5.539417	0.045662	0.051578	0.885301		0.375994	NA
LOC10013	42.04003	-0.04566	0.077814	-0.58677		0.557361	NA
DEGS2	37.08557	0.04565	0.069708	0.654874		0.512549	NA
AAK1	1735.384	0.045645	0.03233	1.411858		0.157992	0.514729
CASC5	2.590948	-0.04564	0.06784	-0.67279		0.501083	NA
LOC10050	0.215561	-0.04564	0.024483	-1.86409		0.062309	NA
MBNL1	374.9537	0.045613	0.035498	1.284926		0.198818	0.545847
CLIC2	7.995972	0.045608	0.082893	0.5502		0.582182	NA
LSR	38.44105	-0.0456	0.07381	-0.61787		0.536661	NA
SLC25A11	80.51635	-0.0456	0.061454	-0.74202		0.458074	NA
TCTE1	31.61459	-0.04559	0.081126	-0.56202		0.574099	NA
IRF6	120.9231	0.045584	0.084076	0.542178		0.587696	NA
VPS72	100.4921	0.045579	0.058159	0.783707		0.433212	NA
TTF1	102.6383	0.045579	0.050916	0.895174		0.370694	NA
MIR631	2.008238	-0.04557	0.06904	-0.66004		0.50923	NA
YIPF4	136.5123	0.045568	0.058372	0.780656		0.435005	NA
PECR	41.70942	-0.04556	0.072698	-0.62674		0.530833	NA
RPS6KA5	425.9538	-0.04555	0.058517	-0.77838		0.436342	0.738506
CAMKK1	256.0975	-0.04554	0.0605	-0.75281		0.451565	NA
MID1	228.0309	0.04554	0.057313	0.79459		0.426852	NA
MIR29B1	0.971642	-0.04554	0.050538	-0.90108		0.367546	NA
TELO2	93.853	-0.04554	0.059983	-0.7592		0.447736	NA
TCTEX1D1	6.112719	-0.04554	0.082044	-0.55504		0.578864	NA
MIR548A2	0.366116	-0.04554	0.034446	-1.322		0.186168	NA
SNORA41	0.527041	0.045532	0.042663	1.067258		0.285855	NA
HSD17B4	268.9959	-0.04553	0.043885	-1.03751		0.299498	NA
DZIP3	405.7574	0.04551	0.04267	1.066564		0.286169	0.620261
EHD1	265.0049	0.045508	0.044101	1.031904		0.302117	NA

AUTS2	415.0585	0.045503	0.045418	1.00186	0.316411	0.645996
KLHL22	101.1181	-0.0455	0.065507	-0.6946	0.487304	NA
CTIF	564.632	0.0455	0.038752	1.174127	0.240344	0.589893
TOP2B	1206.376	0.04549	0.05284	0.86089	0.389299	0.701813
GSTT2	0.277948	-0.04549	0.029947	-1.51895	0.128776	NA
PSPN	6.053827	-0.04549	0.082756	-0.54964	0.582568	NA
POGZ	1198.288	-0.04548	0.034901	-1.30326	0.192487	0.542492
GAGE10	0.481043	0.045482	0.040522	1.122402	0.261691	NA
SAMD4A	564.5476	0.045478	0.062171	0.731499	0.464474	0.755844
WDR41	106.6345	-0.04547	0.04923	-0.92358	0.355704	NA
ACSM1	2.456868	-0.04546	0.065354	-0.69562	0.486665	NA
ZNF706	310.4028	0.045444	0.052402	0.867217	0.385823	NA
AGRP	2.729055	-0.04544	0.072314	-0.62842	0.52973	NA
FLJ31306	320.3502	-0.04543	0.043403	-1.04675	0.295216	NA
APITD1	1.02978	-0.04543	0.052446	-0.86617	0.386398	NA
PKNOX2	117.1919	-0.04543	0.052696	-0.86206	0.388657	NA
NECAB2	2.687313	0.045406	0.071268	0.637123	0.524044	NA
MIR5088	0.299468	-0.0454	0.032541	-1.39505	0.163002	NA
LHFPL3-AS	1.929023	0.045389	0.060792	0.746631	0.455286	NA
GALNT4	1.432425	-0.04537	0.062262	-0.72875	0.466154	NA
C10orf95	10.715	-0.04537	0.083646	-0.54242	0.587531	NA
GRIK1-AS1	2.421477	0.045371	0.068602	0.661361	0.508381	NA
LOC10000	10.22926	0.045366	0.084693	0.535659	0.592194	NA
RHBDD2	46.63818	0.045365	0.067836	0.668734	0.503665	NA
RAB6C	1.375761	-0.04536	0.062192	-0.72941	0.465751	NA
CABYR	14.76191	-0.04536	0.084849	-0.53461	0.592917	NA
GOLGA7B	347.3025	-0.04536	0.082666	-0.54871	0.583206	0.824332
INHBA	4.150668	0.04536	0.080358	0.564467	0.572437	NA
SRC	63.29066	0.045357	0.064804	0.699917	0.483979	NA
SORBS3	162.5098	-0.04535	0.046244	-0.9806	0.326789	NA
CBLN2	2.137075	-0.04534	0.05237	-0.86574	0.386632	NA
TMEM97	23.10024	0.045335	0.072986	0.621149	0.534502	NA
CSGALNAC	317.3331	0.045317	0.047073	0.962702	0.335697	NA
COX8C	1.530995	0.045317	0.058727	0.771659	0.440316	NA
DHX34	107.3383	0.045309	0.072597	0.624119	0.532549	NA
TNN	0.515325	0.045296	0.039481	1.147304	0.251256	NA
FOXRED1	91.88444	0.045284	0.061673	0.734258	0.462791	NA
WFDC1	22.24424	-0.04528	0.07903	-0.573	0.566645	NA
SMAD5	348.3494	0.045282	0.038694	1.170266	0.241894	0.590833
MINK1	911.5674	-0.04528	0.04451	-1.01733	0.308995	0.639252
CRMP1	302.3684	0.045268	0.061141	0.740389	0.459064	NA
OTOP3	0.553404	0.045266	0.038871	1.164513	0.244216	NA
CRTAC1	28.30393	-0.04526	0.076241	-0.59363	0.552762	NA
BEX5	43.99376	-0.04526	0.083683	-0.54081	0.58864	NA
RNF19B	119.1984	0.045253	0.055616	0.813668	0.415835	NA
PABPN1L	3.245427	-0.04523	0.073002	-0.61963	0.535502	NA
ZSCAN10	1.050032	0.045231	0.0549	0.823879	0.410008	NA

GEMIN5	120.6457	0.04522	0.047815	0.945741	0.344281	NA
MIR4473	1.07281	0.04522	0.054946	0.822986	0.410516	NA
TBX5	2.206503	-0.0452	0.060907	-0.74215	0.457998	NA
MIR605	0.189394	-0.0452	0.026156	-1.72794	0.084	NA
FOXD4L1	1.790725	0.045191	0.067421	0.670284	0.502677	NA
PPT1	157.5059	0.045189	0.074844	0.603775	0.545993	NA
CNTLN	302.3173	-0.04518	0.046103	-0.97998	0.327098	NA
LOC64284	13.58829	-0.04516	0.064724	-0.69777	0.485322	NA
ABHD11	25.66247	0.045162	0.072855	0.61988	0.535337	NA
ELK3	88.09066	0.045154	0.059672	0.756696	0.449232	NA
VPS51	143.0621	-0.04515	0.058487	-0.77203	0.440099	NA
P4HA2	76.9205	0.045153	0.079726	0.566355	0.571153	NA
LOC10050	148.523	0.045152	0.048144	0.937842	0.348325	NA
LINC00538	6.854274	-0.04515	0.08386	-0.53836	0.590329	NA
TOMM20	233.1094	-0.04514	0.072667	-0.6212	0.53447	NA
RAI1	346.9441	0.04512	0.05094	0.885742	0.375757	0.695376
BHLHB9	146.0563	0.045115	0.055313	0.815639	0.414707	NA
HES5	4.045666	-0.04511	0.074136	-0.60846	0.542886	NA
VAMP5	5.395811	-0.04511	0.079616	-0.56654	0.571024	NA
MAPK8IP3	2205.041	-0.04511	0.041148	-1.09619	0.272996	0.613429
TRMT44	126.1201	-0.0451	0.066743	-0.67565	0.49926	NA
VWC2L-IT1	0.154328	-0.04509	0.021126	-2.13453	0.032799	NA
KCNU1	0.26905	-0.04509	0.028768	-1.56752	0.116993	NA
LRRC59	91.82038	0.045082	0.061091	0.737939	0.460552	NA
FAM115A	92.50225	0.04508	0.056201	0.802129	0.422478	NA
MOV10	22.24161	-0.04508	0.081424	-0.55363	0.579832	NA
SCARNA20	1.611221	0.045065	0.059799	0.753604	0.451087	NA
CPS1-IT1	0.19869	0.045057	0.023099	1.950615	0.051103	NA
FAM151B	14.49719	0.045052	0.081848	0.550442	0.582016	NA
MPI	56.93794	-0.04505	0.06811	-0.66146	0.508319	NA
C11orf58	348.7546	-0.04504	0.061352	-0.73419	0.462831	0.755844
ATG13	266.1212	-0.04504	0.043015	-1.04706	0.295074	NA
CCT6B	13.58347	-0.04503	0.083707	-0.53801	0.590571	NA
SFXN5	171.803	-0.04503	0.058141	-0.77443	0.438676	NA
TRADD	3.284059	-0.04502	0.075625	-0.59533	0.551623	NA
WASF1	226.1245	0.045017	0.049198	0.915019	0.360182	NA
PPFIBP1	75.19444	-0.04501	0.079772	-0.56428	0.572565	NA
ZNF57	85.84579	-0.04501	0.066299	-0.67894	0.497173	NA
SLC6A20	3.397297	-0.04501	0.06871	-0.65509	0.512409	NA
SMCR7	46.13357	0.045	0.067095	0.670693	0.502416	NA
RAD21-AS	2.52662	-0.045	0.06889	-0.65316	0.513655	NA
DHX16	173.0786	-0.04499	0.045197	-0.99553	0.319479	NA
TMEM214	73.88123	0.044991	0.058876	0.764163	0.44477	NA
IPMK	83.85981	0.04499	0.060897	0.738781	0.46004	NA
LOC40095	4.399633	-0.04497	0.073608	-0.61091	0.541258	NA
LZTS1	7.290757	0.044956	0.082262	0.5465	0.584722	NA
LHFPL3-AS	1.688835	-0.04495	0.059962	-0.74969	0.45344	NA

ARMC2	90.95282	-0.04495	0.065904	-0.68207	0.495198	NA
GUCA1A	0.456645	0.044946	0.035694	1.259178	0.207966	NA
MARCKSL1	145.1739	-0.04494	0.06224	-0.7221	0.470232	NA
WDR38	2.377353	-0.04494	0.064568	-0.69606	0.486391	NA
TMEM9	76.60843	-0.04494	0.063006	-0.71322	0.47571	NA
USP15	357.903	-0.04493	0.032399	-1.38662	0.165558	0.52098
ACIN1	957.2915	-0.04492	0.040774	-1.10176	0.270565	0.611606
CWC27	177.5527	-0.04492	0.044776	-1.0032	0.315762	NA
RPL15	614.8472	-0.04492	0.073894	-0.60786	0.543281	0.803134
GNA11	244.3952	0.044912	0.048395	0.928014	0.3534	NA
MIR148B	0.330133	-0.04491	0.032505	-1.38159	0.167097	NA
TOPORS	319.9875	0.044904	0.047086	0.953649	0.340261	NA
RRAGA	120.893	0.0449	0.06642	0.676004	0.499038	NA
SETD6	149.2013	0.044886	0.081915	0.547967	0.583715	NA
GJB5	2.547425	-0.04489	0.071603	-0.62688	0.53074	NA
PIP4K2C	79.01167	0.044863	0.063376	0.707886	0.479016	NA
LOC72898	26.15419	0.044862	0.076523	0.58626	0.557701	NA
ABRACL	3.634396	-0.04486	0.074933	-0.59865	0.549406	NA
ZBTB7B	75.38845	-0.04486	0.06837	-0.65611	0.511751	NA
TRPV3	22.70442	0.044857	0.084898	0.52836	0.597249	NA
LOC10012	8.403449	-0.04485	0.080441	-0.55756	0.577143	NA
SLC30A8	0.398226	-0.04485	0.035375	-1.26785	0.204853	NA
TBKBP1	87.52211	-0.04485	0.058207	-0.77045	0.441034	NA
C11orf94	4.570959	0.044843	0.07856	0.57082	0.568122	NA
RASSF7	30.57269	-0.04484	0.080627	-0.55614	0.578115	NA
ZNF275	123.0493	0.044837	0.058095	0.771793	0.440237	NA
INTS8	202.2566	0.04482	0.048508	0.923987	0.355493	NA
ARIH1	509.588	0.044811	0.030859	1.45214	0.146463	0.498854
KIAA0895L	320.9747	0.044792	0.07637	0.586506	0.557535	NA
SNORD45B	3.717281	0.044783	0.076056	0.588815	0.555985	NA
OMA1	94.52581	-0.04477	0.072296	-0.61928	0.535733	NA
PUSL1	43.21865	0.04477	0.06591	0.679257	0.496975	NA
STX2	100.2114	-0.04477	0.051433	-0.87042	0.384071	NA
EXD2	151.5804	0.044767	0.047	0.952494	0.340846	NA
MECOM	26.71798	-0.04475	0.082474	-0.54261	0.587398	NA
ARRB1	706.8829	-0.04475	0.048508	-0.92248	0.356279	0.680826
PSMD12	167.0697	0.044727	0.049506	0.903464	0.36628	NA
SECISBP2	310.724	-0.04473	0.05108	-0.87559	0.381251	NA
CIRBP-AS1	19.54369	-0.04472	0.080814	-0.55336	0.580018	NA
ZNF793	220.8203	0.044716	0.044565	1.003375	0.31568	NA
SERPINH1	25.26678	-0.04471	0.079592	-0.56179	0.57426	NA
CCT3	336.2489	0.044708	0.066311	0.674224	0.500169	0.778081
VIMP	43.68897	0.044705	0.068809	0.649694	0.51589	NA
DKK3	1001.975	0.044691	0.059944	0.745546	0.455942	0.751607
MYZAP	17.4779	-0.04469	0.081996	-0.54501	0.585744	NA
AIRE	0.438252	-0.04469	0.039142	-1.14169	0.253585	NA
MYH16	1.418557	0.044672	0.060675	0.736251	0.461578	NA

TMEM165	89.10639	-0.04464	0.060868	-0.73346	0.463276	NA
CCNH	263.1448	-0.04464	0.048957	-0.9119	0.361819	NA
SMAD9	156.096	-0.04463	0.071484	-0.62429	0.532439	NA
AP2A1	276.4367	0.044622	0.038558	1.157257	0.247168	NA
C14orf178	2.245324	-0.04462	0.066835	-0.66754	0.504424	NA
NFKBIE	32.53361	0.044601	0.073293	0.608533	0.542834	NA
TMEM175	109.6707	-0.04459	0.068768	-0.64847	0.51668	NA
C19orf47	60.96922	0.044587	0.06264	0.711801	0.476588	NA
FAM217A	0.551766	-0.04457	0.036667	-1.21565	0.224118	NA
IPO7	541.1181	0.044568	0.04659	0.956594	0.338772	0.66305
PRRX1	44.85142	-0.04457	0.08009	-0.55647	0.577891	NA
MDGA1	2571.218	-0.04456	0.074878	-0.59516	0.55174	0.805737
MYT1L	888.2931	0.044559	0.035805	1.244489	0.21332	0.562782
GRIA3	90.96731	-0.04456	0.077771	-0.57293	0.566691	NA
FGF19	0.240271	0.044545	0.029201	1.525426	0.127153	NA
SNORD115	131.8632	-0.04454	0.084017	-0.53018	0.595989	NA
INCA1	11.35546	0.044539	0.08421	0.528912	0.596866	NA
SLC7A7	29.38933	-0.04454	0.077213	-0.5768	0.564077	NA
TPH1	2.806519	-0.04452	0.067693	-0.65771	0.510725	NA
LPAR1	53.41359	0.044518	0.084756	0.525247	0.599412	NA
CCDC91	182.2373	0.044513	0.050947	0.87371	0.382276	NA
SREK1	704.4206	-0.04451	0.032832	-1.35561	0.175223	0.529097
XPO5	153.2424	0.044504	0.049788	0.89387	0.371391	NA
UHMK1	295.2526	0.044499	0.040868	1.088855	0.276218	NA
PHF20L1	453.0626	0.044487	0.039972	1.112955	0.265728	0.607287
RCE1	41.96951	0.044484	0.075009	0.593043	0.553152	NA
P4HTM	197.2283	-0.04448	0.049546	-0.89781	0.369285	NA
RPS10	1.256672	-0.04447	0.060739	-0.73216	0.464069	NA
CNTN5	5.462787	0.044461	0.081075	0.548388	0.583425	NA
KRIT1	499.2752	0.044458	0.028605	1.554185	0.12014	0.472798
SHANK2-A	1.049849	-0.04445	0.055745	-0.79744	0.425198	NA
SNORD12C	6.769225	-0.04445	0.074789	-0.59435	0.552279	NA
KIAA1737	450.7725	0.044438	0.041822	1.062528	0.287996	0.621249
COMMD7	82.869	0.044436	0.060287	0.737076	0.461076	NA
KIRREL3-A	24.56006	-0.04443	0.084747	-0.52432	0.600056	NA
TMED9	68.26305	0.044413	0.066845	0.664415	0.506425	NA
HNRNPR	589.921	0.04441	0.036888	1.203918	0.228621	0.577536
ZNF624	41.43705	0.044394	0.080378	0.552316	0.580732	NA
ZFYVE16	453.7106	0.04439	0.04611	0.962704	0.335696	0.661751
CTRC	0.807559	-0.04438	0.047272	-0.93887	0.347797	NA
SPINK14	0.309621	0.044376	0.031233	1.420826	0.155367	NA
OS9	364.2535	-0.04438	0.039185	-1.13247	0.257438	0.59833
ARHGAP27	44.45706	-0.04436	0.084666	-0.52397	0.600298	NA
CYP26C1	1.105247	-0.04436	0.055562	-0.79839	0.424644	NA
DECR1	52.53017	-0.04434	0.070977	-0.62477	0.532119	NA
MKRN3	5.369296	-0.04433	0.083392	-0.53153	0.595051	NA
NUP210	212.8231	0.04432	0.055757	0.794872	0.426688	NA

TVP23C	115.1138	-0.04432	0.067498	-0.6566	0.51144	NA
NPLOC4	216.5113	0.044317	0.051203	0.865522	0.386753	NA
FARSB	152.0098	-0.04432	0.071488	-0.61992	0.535311	NA
NCBP2	124.1297	0.044317	0.058056	0.763343	0.445259	NA
MST1R	6.184123	0.044311	0.081119	0.546247	0.584896	NA
ZGLP1	18.54495	0.044302	0.084583	0.523768	0.60044	NA
NNAT	51.70788	-0.04428	0.064335	-0.68834	0.491237	NA
PRSS45	1.238624	-0.04427	0.053786	-0.82308	0.41046	NA
TCEANC2	24.51043	0.04427	0.078686	0.562611	0.5737	NA
PTBP3	151.8569	0.044265	0.06543	0.676531	0.498703	NA
LXN	29.5406	0.044265	0.081778	0.541275	0.588318	NA
DNAJB12	200.8049	-0.04426	0.04651	-0.95169	0.341253	NA
CRHR1-IT1	251.0808	-0.04425	0.056377	-0.78498	0.432468	NA
NDE1	28.66535	-0.04425	0.077967	-0.56758	0.570324	NA
MRPL18	60.38758	0.044248	0.074663	0.592639	0.553423	NA
LILRA5	0.447891	-0.04424	0.036037	-1.22776	0.219537	NA
TLE3	140.2429	-0.04424	0.060085	-0.73636	0.461512	NA
CUEDC1	55.83521	-0.04424	0.07153	-0.61854	0.536222	NA
MIR4513	2.328158	-0.04424	0.070076	-0.63127	0.527865	NA
CNGB3	1.843496	0.044224	0.063512	0.69631	0.486235	NA

UBTD2	397.9249	0.04422	0.048642	0.909101	0.363297	0.686402
CTSZ	102.4706	0.044212	0.061834	0.715015	0.4746	NA
LOC10029	17.47582	0.044206	0.084215	0.524911	0.599645	NA
TNFRSF4	1.232792	-0.0442	0.05537	-0.79833	0.424679	NA
HARS2	108.3828	-0.0442	0.05307	-0.83289	0.404905	NA
DUSP4	44.03569	-0.04419	0.083976	-0.52622	0.598732	NA
CDK16	362.1911	0.044183	0.051941	0.850641	0.394969	0.705623
FBXO44	298.2558	-0.04418	0.051331	-0.86065	0.389431	NA
APCDD1	52.17083	-0.04416	0.082119	-0.53775	0.590752	NA
STK10	854.0387	-0.04414	0.05504	-0.80203	0.422538	0.726689
CCDC67	0.247087	0.044135	0.028826	1.531068	0.125752	NA
GTF3A	110.1274	-0.04412	0.055547	-0.79434	0.426996	NA
LINC00548	0.196074	-0.04411	0.025178	-1.75176	0.079816	NA
MTDH	489.8921	0.044106	0.050243	0.87785	0.380025	0.699234
RANBP9	270.637	0.044102	0.037151	1.187091	0.235192	NA
MREG	182.0628	0.044097	0.062572	0.704749	0.480967	NA
CEP350	1371.686	0.044092	0.037299	1.182106	0.237164	0.586807
FLJ41649	12.54671	0.04409	0.08071	0.546272	0.584879	NA
VWA8	357.9279	0.044078	0.053007	0.831545	0.405666	0.710565
KIAA2026	807.6073	0.044066	0.03663	1.203004	0.228975	0.577536
MRPL46	46.40619	-0.04406	0.064545	-0.68258	0.494875	NA
PDIA6	127.1587	0.044054	0.068043	0.647438	0.517348	NA
UQCRC1	174.1978	-0.04405	0.052614	-0.83716	0.402503	NA
ACOT4	21.69534	0.044043	0.083646	0.526542	0.598512	NA
USP16	217.6813	0.044041	0.049582	0.888237	0.374413	NA
TRAF3	195.904	-0.04403	0.053473	-0.82347	0.410238	NA
TARP	1.143202	-0.04402	0.055209	-0.79739	0.425227	NA

AKR7A3	15.88673	-0.04402	0.082984	-0.53046	0.595795	NA
GNAI2	234.5608	-0.04402	0.05202	-0.84616	0.397465	NA
TPRX1	0.368294	0.044015	0.032924	1.336875	0.181264	NA
TSEN2	169.7363	-0.04401	0.06068	-0.72535	0.468236	NA
PRSS33	0.216368	-0.044	0.024363	-1.80604	0.070912	NA
KCNMB1	4.054283	-0.04399	0.080225	-0.54836	0.583444	NA
SLC16A3	8.208967	-0.04399	0.082298	-0.53455	0.592964	NA
PPP1R37	154.8018	0.043985	0.048685	0.903455	0.366285	NA
TNFAIP3	13.03821	0.043976	0.079916	0.550277	0.58213	NA
TRPC5OS	1.731285	-0.04397	0.062093	-0.70816	0.478844	NA
PEBP1	725.466	-0.04397	0.063017	-0.69772	0.485352	0.764934
PRPF6	660.4973	-0.04397	0.031292	-1.40511	0.15999	0.516971
DNAL4	17.84742	-0.04397	0.082608	-0.53224	0.594562	NA
GBA3	0.17343	0.043965	0.022826	1.926069	0.054096	NA
MRPL38	109.1594	0.043944	0.047306	0.928941	0.35292	NA
AQP2	0.28505	0.043926	0.028612	1.535229	0.124728	NA
PDE6G	0.328663	-0.04391	0.032551	-1.34911	0.177301	NA
GLRX3	57.16694	-0.04391	0.055581	-0.79007	0.429489	NA
LPPR4	33.01596	-0.04391	0.083521	-0.52571	0.599093	NA
CCK	0.355694	-0.04391	0.032773	-1.33972	0.180338	NA
NCF4	2.531725	-0.0439	0.06783	-0.64726	0.517464	NA
TSNAXIP1	29.44467	0.043903	0.079126	0.554853	0.578995	NA
FAM71E1	5.697436	-0.0439	0.084456	-0.51979	0.603211	NA
SEPT7P2	122.8548	-0.0439	0.056338	-0.77916	0.435885	NA
TSPAN3	293.9964	0.043888	0.062965	0.697022	0.485789	NA
CHD4	897.105	0.043886	0.037358	1.174763	0.240089	0.589893
CTH	22.06703	0.043881	0.0793	0.553349	0.580024	NA
CCNT1	243.0305	0.04387	0.034685	1.264842	0.205928	NA
PNPLA6	231.9495	0.04386	0.066678	0.657793	0.510671	NA
VASH1	209.4515	0.043842	0.059492	0.73693	0.461165	NA
SHANK3	1820.798	-0.04384	0.051678	-0.84834	0.39625	0.705623
CD3G	0.256934	0.043829	0.029359	1.492881	0.135468	NA
CSNK2A1	163.872	0.043822	0.043192	1.014586	0.310303	NA
SNORD32B	0.820413	-0.04382	0.046267	-0.94712	0.343579	NA
ZNF32-AS1	1.387769	-0.04381	0.060839	-0.72014	0.471438	NA
MED29	150.0796	-0.0438	0.064223	-0.68202	0.495225	NA
GABRR1	4.191228	0.0438	0.071181	0.615331	0.538336	NA
UPF1	506.9265	0.0438	0.042378	1.033555	0.301344	0.632662
IGLL1	0.21992	-0.0438	0.025407	-1.72388	0.08473	NA
IFFO1	157.9317	-0.0438	0.047689	-0.9184	0.358409	NA
SIGLEC17P	3.149767	0.043793	0.066762	0.655962	0.511848	NA
SNORD38A	6.450474	-0.04379	0.079484	-0.55091	0.581698	NA
LOC49375	96.19039	-0.04378	0.055891	-0.78332	0.43344	NA
IGF2	0.807693	0.043777	0.04706	0.930246	0.352244	NA
MRPL12	40.83859	-0.04378	0.063893	-0.68515	0.493248	NA
NAA35	150.675	0.043773	0.041894	1.044846	0.296094	NA
IZUMO2	0.437013	0.043762	0.038745	1.129501	0.258687	NA

MIR4654	0.655751	-0.04376	0.044924	-0.97406	0.330026	NA
C20orf197	0.867819	-0.04375	0.045753	-0.95627	0.338934	NA
APOLD1	127.0657	-0.04375	0.08447	-0.51796	0.604489	NA
DTNB	207.854	-0.04374	0.048515	-0.90155	0.367295	NA
ZNF702P	184.3364	-0.04372	0.069839	-0.62606	0.531278	NA
ZFAND2B	54.37107	-0.04371	0.071694	-0.60974	0.542032	NA
IFNA2	0.785328	-0.04371	0.040193	-1.08747	0.276829	NA
EXOC6	131.2712	-0.0437	0.066376	-0.65843	0.510265	NA
DNAH10	151.2954	0.043702	0.054076	0.808158	0.419	NA
JPX	38.16283	-0.0437	0.070536	-0.61955	0.535553	NA
PP7080	44.23667	-0.0437	0.063862	-0.68422	0.493837	NA
MUM1L1	1.72707	-0.04369	0.062798	-0.69574	0.486593	NA
SNORA10	3.300142	-0.04369	0.077028	-0.56718	0.57059	NA
METTL21E	17.37832	-0.04367	0.083768	-0.52138	0.602105	NA
PSEN2	98.09756	-0.04367	0.060241	-0.72495	0.468481	NA
DBC1	316.8406	0.043665	0.048549	0.899396	0.368442	NA
VHL	189.4942	0.043651	0.042268	1.032736	0.301727	NA
PRH1	0.410771	-0.04364	0.038906	-1.12177	0.261962	NA
UCHL1	161.3337	-0.04364	0.06961	-0.62697	0.53068	NA
RAB27A	25.51453	-0.04364	0.07719	-0.56535	0.571835	NA
HTR1D	0.372346	-0.04363	0.035092	-1.24342	0.213712	NA
C6orf47	41.68299	0.043616	0.066943	0.651544	0.514695	NA
LINC00486	0.911691	-0.04361	0.051002	-0.85516	0.392465	NA
TYW1B	36.98412	-0.04361	0.078945	-0.55245	0.580637	NA
CEACAM2	0.278095	-0.04361	0.030915	-1.41051	0.158388	NA
MAP2K7	222.0104	-0.0436	0.045522	-0.95786	0.338131	NA
TIMM21	128.0146	0.043603	0.060375	0.722211	0.470165	NA
S1PR3	12.39555	-0.04358	0.083245	-0.52356	0.600582	NA
ILKAP	137.5847	0.043582	0.052219	0.834588	0.40395	NA
SPTBN4	1384.682	-0.04358	0.052023	-0.83768	0.402212	0.709466
COL26A1	9.33945	-0.04358	0.083873	-0.51955	0.603376	NA
NPY	0.323633	0.043566	0.031223	1.395331	0.162916	NA
GPR62	14.91706	-0.04357	0.084419	-0.51606	0.605812	NA
TRIM32	234.0961	0.043564	0.044741	0.97371	0.330201	NA
FAM35DP	0.515151	-0.04355	0.042461	-1.02569	0.305038	NA
SP1	616.4833	0.043551	0.050251	0.866666	0.386125	0.700314
SLC51A	10.99098	0.043549	0.084714	0.514063	0.607208	NA
ARF4	134.1633	0.043537	0.062634	0.695113	0.486984	NA
GOLGA4	1849.548	-0.04353	0.04665	-0.93321	0.350713	0.674334
SLC12A5	1002.766	0.043531	0.046906	0.928057	0.353378	0.677732
PPP1R2P3	0.605272	-0.04353	0.045348	-0.95988	0.337114	NA
MAPK11	101.6089	-0.04352	0.067437	-0.64536	0.518692	NA
MIR4257	0.705762	-0.04352	0.047596	-0.91435	0.360535	NA
SMA5	3.104833	-0.04352	0.071299	-0.61035	0.541629	NA
C6orf1	30.40383	-0.04351	0.070351	-0.61845	0.536281	NA
CHRAC1	22.30933	-0.0435	0.07704	-0.56467	0.572296	NA
PLOD3	31.28368	-0.0435	0.08026	-0.54199	0.587827	NA

PTGDR	1.103876	-0.0435	0.05394	-0.80637	0.420029	NA
JKAMP	133.933	-0.04346	0.049334	-0.88102	0.378307	NA
TCP11L1	210.8655	0.04346	0.042648	1.019037	0.308186	NA
ZNF546	80.85929	0.043457	0.054359	0.799445	0.424032	NA
TMEM86A	21.56437	-0.04345	0.078696	-0.5521	0.580882	NA
SNORD88B	0.443708	0.043447	0.036621	1.186389	0.235469	NA
PAR-SN	748.9343	-0.04345	0.049705	-0.87409	0.382068	0.70023
RBM41	272.6003	-0.04345	0.042356	-1.02572	0.305022	NA
GATAD2A	148.3771	-0.04344	0.046197	-0.94023	0.347101	NA
AHCYL2	178.7906	0.043428	0.041963	1.034916	0.300708	NA
CATSPERD	0.328732	-0.04342	0.031857	-1.36313	0.172842	NA
CENPB	153.21	0.043423	0.054164	0.801684	0.422736	NA
AURKAPS1	19.12374	0.043421	0.078494	0.553181	0.58014	NA
CYBRD1	63.60429	-0.04342	0.083183	-0.52199	0.60168	NA
ST5	232.8958	-0.04341	0.048897	-0.88784	0.374627	NA
INSIG1	162.2452	0.043411	0.067793	0.64034	0.521952	NA
ETNK1	283.3851	0.043403	0.052087	0.83329	0.404681	NA
BAI3	1357.987	-0.04336	0.042129	-1.02924	0.303368	0.634468
CSDE1	1117.892	0.043359	0.056756	0.763952	0.444896	0.743291
CEP104	305.4037	0.043348	0.047181	0.918758	0.358222	NA
PDE10A	191.8415	-0.04334	0.083264	-0.52046	0.602741	NA
SNORD1B	0.909139	0.043334	0.052364	0.827545	0.407928	NA
KLB	29.77142	0.043333	0.081171	0.533849	0.593446	NA
SNORD115	35.83764	-0.04333	0.084511	-0.51274	0.608133	NA
LRTOMT	39.37027	0.043328	0.075772	0.571826	0.56744	NA
SHC1	88.55312	-0.04333	0.059557	-0.7275	0.46692	NA
MRPL45P2	35.29649	0.043319	0.08099	0.534865	0.592743	NA
CCDC61	84.75642	-0.04331	0.070856	-0.61128	0.541017	NA
SCRN1	1546.71	0.043304	0.064389	0.672533	0.501244	0.778081
MXD3	8.667937	-0.0433	0.084658	-0.51149	0.609008	NA
LDOC1	77.00793	0.0433	0.063753	0.679185	0.497021	NA
TGIF2	6.454097	-0.0433	0.083995	-0.51549	0.606213	NA
HSPA1L	21.29352	0.043288	0.080215	0.539655	0.589435	NA
NFYC	152.0271	0.043279	0.046771	0.925342	0.354788	NA
RCOR3	373.8405	-0.04327	0.054792	-0.78975	0.429673	0.73339
SLC9A9-AS	0.407267	0.043268	0.036861	1.173828	0.240464	NA
CNTF	0.490668	-0.04325	0.038182	-1.13279	0.257303	NA
LGALS16	0.829078	0.043249	0.047993	0.901158	0.367505	NA
FMNL2	940.3643	0.043247	0.044671	0.968133	0.332978	0.660832
DUT	77.01717	-0.04324	0.062789	-0.68871	0.491006	NA
CHUK	97.69578	0.043237	0.063284	0.683229	0.494462	NA
MOK	93.66281	-0.04323	0.064253	-0.67278	0.501086	NA
LOC10049	1.011076	0.043216	0.050447	0.856656	0.391635	NA
TTYH3	299.2214	0.043199	0.065677	0.657742	0.510704	NA
POU6F1	250.8944	0.043197	0.047826	0.903203	0.366418	NA
PRPF31	243.7879	-0.0432	0.056441	-0.76535	0.444066	NA
UNK	186.6349	0.043194	0.052077	0.829417	0.406869	NA

IFI35	11.19472	-0.04317	0.084939	-0.50825	0.611276	NA
MIR5587	0.687241	0.043159	0.04304	1.002766	0.315974	NA
C17orf85	389.6763	0.043157	0.038182	1.130295	0.258352	0.59833
SIK2	273.6991	0.043156	0.043425	0.993807	0.320317	NA
MRPL3	74.21142	0.043154	0.062727	0.687967	0.491473	NA
KDM5B	319.6668	-0.04313	0.042584	-1.01291	0.311104	NA
PFDN2	100.3589	0.043131	0.06655	0.648105	0.516917	NA
MMP17	9.033989	0.043128	0.084862	0.508221	0.611299	NA
LOC10050	9.507516	-0.04312	0.084939	-0.50768	0.611678	NA
COQ3	32.69353	0.043118	0.072978	0.59084	0.554628	NA
RNF44	142.0713	-0.04312	0.049692	-0.86767	0.385574	NA
SNIP1	92.81592	0.043109	0.056562	0.762162	0.445963	NA
RAD18	105.2887	-0.04311	0.067544	-0.63823	0.523322	NA
DPY19L2P	1.644971	0.043107	0.061683	0.698841	0.484651	NA
CELF3	329.9617	0.0431	0.04476	0.962916	0.33559	NA
MIXL1	1.267403	-0.04309	0.056104	-0.76805	0.44246	NA
PPP1R27	0.891457	0.043085	0.051633	0.834457	0.404024	NA
SENP8	16.34454	0.043084	0.080953	0.532214	0.594577	NA
SNORA11	12.43286	-0.04308	0.082358	-0.52309	0.600909	NA
ZNF771	30.74553	0.043078	0.072353	0.595376	0.551592	NA
RGAG4	322.9019	0.043076	0.046954	0.917421	0.358922	NA
FAM175B	78.57148	0.04307	0.055494	0.776115	0.437681	NA
RABL5	25.78065	0.043065	0.080496	0.534994	0.592654	NA
MAGED2	159.2571	-0.04306	0.06431	-0.66964	0.503086	NA
MIR5579	0.312854	0.043057	0.030986	1.389537	0.164669	NA
IL13RA1	25.75101	-0.04305	0.08204	-0.52479	0.599727	NA
ACADSB	269.4231	-0.04304	0.070525	-0.61025	0.541698	NA
ITPR2	430.5689	-0.04304	0.072944	-0.59	0.555188	0.808857
CCDC104	127.4374	-0.04303	0.055931	-0.76935	0.441684	NA
CCDC74B	1.52137	-0.04303	0.062231	-0.69146	0.489276	NA
ANKRD11	1472.768	-0.04303	0.050063	-0.8595	0.390067	0.701813
CCDC40	78.21428	-0.04301	0.083494	-0.51517	0.606431	NA
C14orf132	986.5496	-0.04301	0.058435	-0.73608	0.461682	0.75557
ANGPTL3	8.704879	-0.04299	0.084737	-0.50734	0.611916	NA
PBX1	404.5858	-0.04299	0.039165	-1.09759	0.272385	0.612663
NUP155	138.1275	0.042985	0.046814	0.918202	0.358513	NA
CHRM1	0.877609	-0.04298	0.049203	-0.87355	0.382362	NA
DMXL1	635.3104	0.042978	0.028737	1.495564	0.134767	0.487416
KCNK2	2.403115	-0.04297	0.064572	-0.6654	0.505796	NA
LINC00032	5.449422	0.042964	0.082142	0.523045	0.600943	NA
FKBP9L	2.202538	-0.04295	0.057599	-0.74575	0.455818	NA
LOC10012	151.4435	0.042955	0.081246	0.528698	0.597015	NA
RFNG	56.94028	-0.04295	0.072247	-0.59444	0.55222	NA
CSRNP1	39.24899	-0.04294	0.077622	-0.55324	0.580097	NA
SWT1	81.74426	0.04294	0.058501	0.734013	0.462941	NA
CTDSPL2	338.9322	0.042935	0.038662	1.110535	0.266769	0.607287
POLG	188.7892	0.042934	0.053076	0.808924	0.418559	NA

NREP-AS1	0.36247	0.042934	0.028736	1.494094	0.135151	NA
ISLR	3.35481	0.042932	0.066631	0.644324	0.519365	NA
ZNF587	336.7421	-0.04293	0.048176	-0.89113	0.372861	0.695376
ARL10	45.60739	-0.04292	0.073118	-0.58705	0.557167	NA
NAA11	0.426413	-0.04292	0.033682	-1.27433	0.202547	NA
ZC3H13	1353.817	0.042919	0.037775	1.136192	0.255876	0.598301
MTMR6	112.909	0.042917	0.055415	0.774465	0.438656	NA
HDAC8	136.6552	0.042913	0.043076	0.996217	0.319145	NA
NPAS3	94.17074	-0.04291	0.07874	-0.54491	0.585814	NA
ANKRD36B	449.5839	-0.0429	0.067568	-0.63488	0.525508	0.789308
CENPN	10.28154	0.042892	0.084848	0.50552	0.613194	NA
LOC10050	32.88897	-0.04289	0.0835	-0.51366	0.607491	NA
CSDC2	139.5899	-0.04287	0.065441	-0.65517	0.512357	NA
SIT1	0.194192	-0.04287	0.025217	-1.70024	0.089085	NA
MIR4742	1.926907	0.042873	0.063789	0.672104	0.501517	NA
PLA2G12B	3.486258	0.042871	0.071621	0.59859	0.549446	NA
METTL25	70.94541	0.042868	0.077967	0.549824	0.58244	NA
MIR543	0.423065	-0.04286	0.034515	-1.24182	0.214303	NA
COPS5	88.59291	-0.04286	0.052176	-0.82136	0.411441	NA
NAB1	469.1042	0.04285	0.041921	1.022166	0.306702	0.636154
LAMP2	295.5761	-0.04283	0.057621	-0.7433	0.457301	NA
GZMM	4.63931	0.042822	0.079105	0.541329	0.588281	NA
C3orf70	151.5964	-0.04282	0.060391	-0.70904	0.478298	NA
KIF6	15.72786	-0.04282	0.08005	-0.53489	0.592728	NA
PDGFB	56.8817	-0.04282	0.0709	-0.60391	0.545902	NA
LINC00238	4.91238	-0.04281	0.081353	-0.52625	0.598714	NA
GPC5-AS1	1.051621	-0.04281	0.054328	-0.78792	0.430743	NA
KIF25	10.04282	-0.0428	0.049184	-0.8702	0.384192	NA
ZNF799	19.61912	0.042797	0.081658	0.524108	0.600204	NA
GPR124	74.70739	0.042788	0.065999	0.648322	0.516777	NA
AEBP1	62.67268	0.042786	0.084476	0.506486	0.612515	NA
MAP3K11	79.47235	0.042772	0.054389	0.786411	0.431627	NA
ADRA1A	38.69249	0.042771	0.081265	0.526312	0.598671	NA
LDLR	100.811	0.042768	0.08417	0.508118	0.611371	NA
NR3C2	366.4323	-0.04277	0.043583	-0.98125	0.326472	0.655921
MIR3943	0.716838	-0.04276	0.04627	-0.92424	0.355361	NA
SGIP1	411.5346	0.042764	0.039811	1.074158	0.282752	0.617151
PMM1	78.25108	-0.04275	0.064436	-0.66349	0.507016	NA
AURKB	0.266423	-0.04275	0.026656	-1.60382	0.108754	NA
ECEL1	0.302489	-0.04275	0.02888	-1.48029	0.138796	NA
VPS41	262.8488	0.042746	0.047358	0.902598	0.366739	NA
PIGV	19.4672	0.042745	0.077118	0.554282	0.579386	NA
TNFRSF10	6.31228	-0.04274	0.083513	-0.51181	0.608787	NA
GIPC1	56.69342	-0.04274	0.071385	-0.59871	0.549368	NA
MIR4446	3.427337	-0.04273	0.075247	-0.56791	0.570095	NA
OSBPL11	108.8343	-0.04273	0.050884	-0.83974	0.401052	NA
ZNF778	112.4276	0.042718	0.065954	0.647685	0.517188	NA

OGFOD2	25.89541	0.042711	0.08049	0.530637	0.59567	NA
CPA2	0.328097	-0.04271	0.030031	-1.42216	0.15498	NA
ZNF321P	0.246817	0.042704	0.028707	1.487575	0.136863	NA
CSRP3	4.79578	0.042674	0.081726	0.522157	0.601561	NA
COL8A1	6.135421	-0.04267	0.081975	-0.52053	0.602695	NA
GTF2IRD1	1.128088	-0.04267	0.053766	-0.79363	0.427412	NA
CENPL	11.12182	-0.04267	0.084113	-0.50727	0.611968	NA
TMEM215	3.664987	-0.04266	0.071605	-0.59581	0.551303	NA
CD207	1.593231	0.042653	0.051873	0.822257	0.410931	NA
CWC22	293.2312	0.042653	0.038336	1.112611	0.265876	NA
DIXDC1	764.8057	0.04264	0.065996	0.646093	0.518219	0.785288
AGAP4	3.398158	-0.04264	0.069773	-0.61107	0.541156	NA
TRIM54	0.61268	0.042626	0.044487	0.958166	0.337979	NA
RAB2B	72.12737	-0.04262	0.057697	-0.73875	0.460057	NA
RNF34	176.2832	0.042621	0.0409	1.042066	0.297381	NA
STAT1	181.071	0.042612	0.077531	0.54962	0.58258	NA
METTL14	155.2487	0.04261	0.046204	0.922204	0.356422	NA
SYT14	319.6281	0.042607	0.053628	0.794477	0.426918	NA
LUZP2	38.9515	0.042605	0.083905	0.507772	0.611613	NA
GCH1	4.122091	0.042603	0.07458	0.571243	0.567835	NA
C1orf146	3.144318	-0.0426	0.07132	-0.59731	0.550298	NA
MIR3676	0.868011	0.042598	0.047905	0.889204	0.373894	NA
WHAMMP	47.69062	-0.0426	0.07156	-0.59527	0.551666	NA
THBD	4.696173	0.042592	0.07822	0.544511	0.58609	NA
COG2	248.8099	-0.04259	0.048722	-0.87412	0.382055	NA
LOC64686	1.837311	-0.04258	0.066385	-0.64145	0.521229	NA
RBPJ	328.4214	0.042582	0.04355	0.977766	0.32819	NA
NDUFS1	496.2453	0.042581	0.046238	0.920913	0.357096	0.680826
AACS	101.2681	0.042581	0.063924	0.666117	0.505336	NA
LOC10013	0.23931	-0.04258	0.028204	-1.50959	0.131149	NA
MGC2734	206.5531	0.042536	0.060123	0.70748	0.479268	NA
MATN3	4.022287	0.042517	0.077651	0.547545	0.584004	NA
EHF	1.366841	0.042507	0.052464	0.810221	0.417813	NA
ZNF320	132.5695	0.042507	0.068924	0.61672	0.537419	NA
TRIM25	143.8418	-0.0425	0.056871	-0.74738	0.454832	NA
STEAP1B	1.197066	0.042503	0.053156	0.799583	0.423952	NA
WDR89	72.50662	0.042503	0.056693	0.749695	0.453438	NA
NIT2	20.75397	-0.04249	0.080388	-0.52862	0.597066	NA
GALNT11	170.665	0.042493	0.052788	0.804983	0.42083	NA
CTSH	48.4254	0.042478	0.082949	0.512095	0.608585	NA
AGTPBP1	420.5506	-0.04246	0.054774	-0.77516	0.438248	0.74018
IGBP1P1	3.339444	0.042449	0.076648	0.553813	0.579707	NA
MLLT3	132.9584	-0.04244	0.057971	-0.73216	0.464073	NA
HCG18	547.7537	0.042438	0.053115	0.798987	0.424298	0.728057
NXNL1	1.195144	-0.04242	0.056995	-0.74422	0.456741	NA
ASNA1	56.55801	-0.04242	0.072906	-0.58178	0.560715	NA
SIN3A	386.8532	-0.04241	0.046192	-0.91822	0.358502	0.682359

UTS2D	1.070632	0.042411	0.05536	0.766094	0.44362	NA
THOC5	274.6565	-0.04241	0.063786	-0.6648	0.506179	NA
PSMD1	334.5141	0.042403	0.050393	0.841454	0.400093	NA
SPECC1	667.6363	0.0424	0.053797	0.788153	0.430607	0.733879
LPCAT1	190.2091	0.042394	0.054189	0.78233	0.434021	NA
TSPYL5	92.54919	0.042392	0.061361	0.690859	0.489654	NA
ADAL	45.69996	0.042391	0.066552	0.63696	0.524151	NA
KCTD12	413.6286	-0.04237	0.070711	-0.59925	0.549009	0.803817
FENDRR	9.329841	-0.04237	0.082837	-0.51153	0.60898	NA
ZNF648	0.864239	-0.04237	0.049503	-0.85598	0.392011	NA
EIF2A	266.3053	-0.04237	0.060678	-0.69825	0.485023	NA
TCF3	62.94592	0.042362	0.062876	0.673738	0.500478	NA
HAO2	0.187511	-0.04236	0.02301	-1.84086	0.065642	NA
SYT7	375.4666	0.042337	0.068904	0.614428	0.538932	0.799922
NPR2	217.6157	0.042334	0.070404	0.601291	0.547646	NA
CRAMP1L	450.1329	-0.04233	0.047567	-0.88984	0.373552	0.695376
POLI	141.5231	-0.04232	0.062033	-0.68228	0.49506	NA
DPP10	48.52913	0.042318	0.084152	0.502882	0.615047	NA
MIR5094	0.683907	0.042313	0.041803	1.012213	0.311436	NA
KIF3B	329.5349	0.042311	0.054996	0.769352	0.441685	NA
YY2	18.98455	-0.04231	0.08161	-0.51844	0.604151	NA
ZDHHC9	96.55304	-0.04231	0.05626	-0.75199	0.452055	NA
MTX3	217.2694	0.042299	0.055189	0.766444	0.443412	NA
ESRP2	25.71141	-0.04227	0.07913	-0.53423	0.593184	NA
HSPE1	8.819439	-0.04227	0.082648	-0.51146	0.60903	NA
AKAP14	2.273455	0.042271	0.071677	0.589737	0.555367	NA
LRRC8A	549.1235	-0.04227	0.071713	-0.58941	0.555584	0.808857
PDGFD	2.983865	0.042267	0.060116	0.703085	0.482003	NA
DTHD1	2.741974	0.042266	0.065976	0.640629	0.521763	NA
IPO13	153.0341	0.042264	0.053566	0.789013	0.430104	NA
ARIH2	264.052	-0.04226	0.03696	-1.14343	0.25286	NA
MIR4744	0.960879	-0.04226	0.052178	-0.80985	0.418028	NA
LOC10013	20.54639	-0.04225	0.083473	-0.50615	0.612753	NA
PCLO	6325.996	0.042246	0.044932	0.940217	0.347106	0.672536
ANAPC16	176.6775	-0.04224	0.051311	-0.82328	0.410349	NA
CACNA2D3	102.0209	-0.04224	0.077726	-0.54345	0.586822	NA
MSN	168.1902	-0.04223	0.075332	-0.56061	0.575062	NA
CPPED1	21.37282	-0.04222	0.082645	-0.51086	0.609449	NA
ABHD5	137.7499	0.04221	0.054954	0.768091	0.442433	NA
ARSB	62.46196	0.042204	0.063571	0.663883	0.506765	NA
IMMP1L	13.92832	0.042198	0.083803	0.503535	0.614588	NA
ATG3	148.5255	0.042196	0.062981	0.669987	0.502866	NA
ZNF439	161.1755	-0.0422	0.047554	-0.88733	0.374901	NA
PMP2	549.8236	-0.04219	0.084884	-0.49701	0.619179	0.839739
ALDH3A1	0.419629	-0.04219	0.036746	-1.14802	0.250962	NA
SMPD3	128.0563	0.042178	0.059908	0.704043	0.481406	NA
UTP23	118.8184	0.042174	0.051453	0.819655	0.412413	NA

TMEM184	228.9411	0.04217	0.062103	0.679033	0.497117	NA
RHBDL2	33.21461	-0.04216	0.077692	-0.54272	0.587324	NA
NCSTN	73.44254	0.042164	0.058134	0.725297	0.46827	NA
LHX6	2.017824	0.042158	0.063808	0.660705	0.508802	NA
HECTD2	167.8193	0.042154	0.047149	0.894054	0.371293	NA
C1orf95	38.57217	-0.04215	0.08489	-0.49655	0.61951	NA
HCAR2	0.706886	0.042137	0.043085	0.978006	0.328071	NA
OR2A4	0.431655	0.042137	0.038523	1.093794	0.274045	NA
SMC1A	1447.689	0.042129	0.042306	0.99583	0.319333	0.649272
CBFA2T3	291.2521	-0.04213	0.060215	-0.69958	0.484191	NA
GIMAP8	10.63624	0.042123	0.084597	0.497928	0.618535	NA
SYDE2	2.308898	0.042123	0.072287	0.582724	0.560079	NA
KCP	49.2985	-0.04212	0.082926	-0.50793	0.611501	NA
LOC10065	0.233585	-0.04212	0.027141	-1.55185	0.120698	NA
LOC10028	6.04416	0.042112	0.083903	0.501906	0.615734	NA
SNAPC4	163.7325	-0.04211	0.05263	-0.80008	0.423665	NA
IFITM1	28.87495	-0.04211	0.083353	-0.50517	0.613438	NA
MIR4793	0.293839	0.042106	0.030715	1.370833	0.170427	NA
DCTN4	216.1832	0.042105	0.044924	0.937249	0.348631	NA
IQSEC3	809.2597	-0.0421	0.07241	-0.58136	0.561001	0.810528
LOC10028	27.42093	0.042092	0.076819	0.54794	0.583733	NA
LOC28562	5.392528	0.042087	0.075712	0.555884	0.57829	NA
DOLK	24.73845	-0.04208	0.083223	-0.50558	0.613151	NA
AEBP2	242.8031	0.042074	0.042014	1.001435	0.316617	NA
MIR542	0.407896	0.042064	0.034054	1.235227	0.216746	NA
SLMO2	28.23165	0.042051	0.073778	0.569962	0.568703	NA
CLDN6	2.887826	0.04205	0.071265	0.590056	0.555153	NA
GUCY1B2	1.502389	-0.04204	0.062482	-0.67289	0.501014	NA
PSMD8	155.0011	-0.04204	0.072423	-0.58052	0.561564	NA
SNORD115	2.035751	-0.04204	0.067546	-0.62242	0.533668	NA
PCSK6	103.9101	-0.04204	0.076277	-0.55112	0.581554	NA
SNX18	100.5135	0.042032	0.054912	0.765445	0.444007	NA
DLGAP3	267.9857	0.042029	0.058579	0.717475	0.473081	NA
TMEM192	101.4411	-0.04202	0.060953	-0.68942	0.490562	NA
SNORA71C	0.766083	0.042021	0.049993	0.840541	0.400605	NA
C7orf26	42.12132	0.042017	0.066131	0.635363	0.525192	NA
LOC33896	2.573162	0.042017	0.068354	0.614691	0.538759	NA
RPL38	253.3151	-0.04201	0.064462	-0.65174	0.514571	NA
EIF4E2	102.9584	0.042009	0.052764	0.796172	0.425932	NA
HSD17B12	80.27789	-0.04201	0.072738	-0.57753	0.563581	NA
SKP1	591.1316	-0.04201	0.069215	-0.60692	0.543904	0.803134
ECHDC1	100.498	-0.042	0.05601	-0.74994	0.453292	NA
NME1	7.005836	0.042004	0.084098	0.499461	0.617455	NA
NEDD9	70.40845	-0.042	0.074036	-0.56724	0.570552	NA
CDR2	90.97048	0.041989	0.066332	0.633016	0.526723	NA
MAST3	31.98465	-0.04198	0.076048	-0.55207	0.580898	NA
CDH3	22.73501	-0.04198	0.084061	-0.49942	0.617486	NA

AMFR	177.9975	0.041975	0.059674	0.703408	0.481801	NA
ZNF362	146.9482	0.041975	0.057187	0.733996	0.462951	NA
GKAP1	243.5232	-0.04197	0.050761	-0.82689	0.408301	NA
EPRS	512.0808	0.041973	0.043037	0.975273	0.329425	0.65961
TOMM22	46.31905	0.041963	0.077941	0.538386	0.590311	NA
MIR5581	1.067765	0.041959	0.055117	0.761264	0.446499	NA
SNORA70	11.13703	0.041959	0.08449	0.496609	0.619465	NA
OR2AE1	0.858315	-0.04196	0.047124	-0.89035	0.373276	NA
ZNF354B	95.36759	0.041954	0.053951	0.777621	0.436793	NA
FLJ33630	109.8991	-0.04195	0.0628	-0.66792	0.504182	NA
IQGAP3	0.621205	-0.04194	0.036093	-1.16211	0.245191	NA
GYLTL1B	2.248813	-0.04194	0.069956	-0.59952	0.548823	NA
CREG2	1.415704	0.041924	0.061281	0.684133	0.493891	NA
TTLL5	302.6568	-0.04192	0.047253	-0.88717	0.374986	NA
GGPS1	73.8248	-0.04192	0.062221	-0.67367	0.500519	NA
FYB	28.55594	-0.04191	0.079124	-0.52972	0.596303	NA
DRD5	0.161574	-0.04191	0.022611	-1.85364	0.063791	NA
SBSPON	30.83214	-0.04191	0.076683	-0.54657	0.584673	NA
H2AFJ	33.84138	0.041912	0.079839	0.524951	0.599617	NA
MTUS2	440.0416	0.041907	0.059801	0.700779	0.483441	0.764934
DRG1	108.8179	-0.0419	0.055104	-0.76032	0.447061	NA
ATAD5	180.0347	-0.04189	0.053653	-0.78082	0.434907	NA
BRMS1	54.62944	0.041892	0.073075	0.573278	0.566457	NA
KLHL8	151.3368	0.04189	0.054043	0.775125	0.438266	NA
MINA	56.91571	0.04189	0.060777	0.689243	0.490671	NA
MIR1197	2.786117	-0.04189	0.072224	-0.57996	0.561942	NA
TMED3	43.80829	0.041882	0.073307	0.57132	0.567783	NA
NDN	8.735089	0.041873	0.082759	0.505966	0.61288	NA
KCNC2	4.532619	-0.04187	0.072252	-0.57951	0.562244	NA
TNRC18	1035.153	-0.04186	0.049817	-0.84035	0.400711	0.708517
LOC28619	1.074687	-0.04186	0.056974	-0.73476	0.462486	NA
SNX21	118.6465	-0.04186	0.052699	-0.79434	0.426999	NA
MBOAT7	49.81322	0.04186	0.06139	0.681872	0.49532	NA
IGSF11	26.92721	-0.04185	0.083279	-0.50249	0.615326	NA
ME2	256.7622	0.041839	0.047476	0.881256	0.37818	NA
MYOM3	3.931317	-0.04183	0.059255	-0.70588	0.480264	NA
CCDC144A	216.0563	-0.04182	0.084694	-0.49383	0.621428	NA
FRYL	631.0135	-0.04182	0.036187	-1.15576	0.247778	0.592704
TRA2A	280.2914	-0.04181	0.05696	-0.73394	0.462986	NA
SERPINA3	92.5964	-0.0418	0.047001	-0.88937	0.373806	NA
SLC25A35	4.194598	-0.0418	0.078832	-0.53023	0.595949	NA
IFT57	59.26056	0.041795	0.061244	0.682433	0.494965	NA
MAP4K3	480.3323	-0.04179	0.03868	-1.08033	0.279993	0.616311
GRIP1	10.47138	-0.04179	0.084937	-0.49196	0.62275	NA
GGACT	19.95842	-0.04176	0.080712	-0.51744	0.604846	NA
MECP2	714.1547	0.041764	0.03823	1.09244	0.27464	0.613511
FN3KRP	61.94105	-0.04176	0.062714	-0.66594	0.505451	NA

PET117	10.54314	-0.04176	0.084939	-0.49164	0.622973	NA
MIR4660	0.446475	0.04175	0.033882	1.232245	0.217858	NA
LRRIQ4	0.392943	0.041747	0.034542	1.208583	0.226823	NA
LILRA2	1.16597	-0.04174	0.057928	-0.72061	0.471148	NA
ZNF587B	140.0987	0.041739	0.063065	0.66184	0.508074	NA
UMPS	126.8391	0.041736	0.061406	0.679669	0.496714	NA
MYBPH	18.0934	0.04173	0.079705	0.52356	0.600584	NA
ATG14	175.6233	-0.04173	0.053142	-0.7852	0.432337	NA
NACA2	0.859278	0.04172	0.051126	0.816021	0.414488	NA
SH3GLB2	224.6327	-0.04172	0.063055	-0.6616	0.508225	NA
EP400NL	42.77951	0.041713	0.06871	0.607088	0.543792	NA
PRKRIP1	11.49161	0.041712	0.084004	0.496543	0.619511	NA
ATP1B4	0.324394	-0.04171	0.031009	-1.34513	0.178584	NA
MIR541	1.151022	-0.04171	0.055667	-0.74928	0.453689	NA
EFCAB13	112.8743	-0.04171	0.066968	-0.62281	0.533411	NA
KRTAP5-10	0.253125	0.041707	0.027555	1.513616	0.130123	NA
FKBP7	29.04924	-0.04171	0.07411	-0.56276	0.573598	NA
CYB5R1	35.88453	-0.04169	0.067623	-0.61652	0.537552	NA
CDH10	309.3455	-0.04169	0.048477	-0.85999	0.389792	NA
EXD1	0.168168	-0.04168	0.022779	-1.82977	0.067284	NA
ZNF99	6.779375	0.041676	0.075116	0.554824	0.579015	NA
CDCA7L	79.59704	0.041674	0.084312	0.49429	0.621101	NA
MIR624	1.243876	-0.04167	0.06066	-0.68696	0.492105	NA
GPRC5A	0.41335	-0.04167	0.035648	-1.16895	0.242422	NA
GNPDA1	42.73762	-0.04167	0.068413	-0.60907	0.542479	NA
NOC4L	30.00977	-0.04167	0.072789	-0.57243	0.567028	NA
PRRT4	12.44618	-0.04167	0.084443	-0.49341	0.621721	NA
C6orf52	2.721239	0.041656	0.071717	0.580845	0.561345	NA
MTUS2-AS	9.385014	-0.04165	0.08485	-0.49087	0.623515	NA
C9orf163	1.789233	-0.04163	0.068072	-0.61162	0.540792	NA
AQP11	37.51679	-0.04163	0.072066	-0.5776	0.563532	NA
ALG1L	0.535295	-0.04162	0.041559	-1.00147	0.316598	NA
ATG12	196.6735	-0.04161	0.049527	-0.84019	0.400804	NA
CLN6	44.64339	0.04161	0.071236	0.584112	0.559145	NA
TEX10	111.1617	0.041603	0.053551	0.77688	0.43723	NA
CCBE1	3.278775	-0.0416	0.078601	-0.52927	0.596619	NA
WAPAL	561.8276	0.041595	0.034437	1.20783	0.227113	0.577485
IL17RB	102.8738	-0.04159	0.070829	-0.58725	0.557038	NA
OAS3	30.93141	0.04159	0.084299	0.49337	0.621751	NA
MOB1A	49.32705	0.041579	0.060911	0.68262	0.494847	NA
POMZP3	35.74289	0.041579	0.08308	0.500466	0.616747	NA
ATP8A2	1116.056	0.041578	0.036016	1.154439	0.24832	0.592704
C3orf80	21.17502	0.041578	0.08104	0.513046	0.607919	NA
SAMD8	308.7072	0.041577	0.040796	1.019161	0.308126	NA
SDR39U1	25.68448	0.041566	0.07589	0.547716	0.583887	NA
PCP4L1	18.17017	-0.04155	0.079852	-0.52034	0.602826	NA
RAD51	13.54642	-0.04154	0.084868	-0.48951	0.624478	NA

MIR31HG	29.63905	0.041543	0.084528	0.49147	0.623094	NA
ICA1L	512.4544	0.041542	0.047721	0.870521	0.384016	0.70023
UBE2D3	430.6214	0.041542	0.04812	0.863288	0.387979	0.700889
TXNDC2	0.919441	0.041538	0.047951	0.866262	0.386347	NA
PEX26	91.07651	-0.04154	0.053256	-0.77994	0.435426	NA
CHST13	1.052386	0.041521	0.054234	0.765584	0.443924	NA
KRTAP5-4	0.535593	0.041511	0.040752	1.018637	0.308375	NA
C1orf63	312.1313	-0.04151	0.072507	-0.57246	0.567008	NA
DHX32	72.3168	0.041503	0.059211	0.700925	0.48335	NA
ARHGEF7	1105.298	0.041498	0.047264	0.87799	0.379949	0.699234
NUP37	8.251025	0.041495	0.084135	0.493193	0.621876	NA
CYP2F1	0.282025	-0.04149	0.024013	-1.72789	0.084007	NA
NEB	112.2106	0.041491	0.079054	0.524847	0.59969	NA
MPV17L2	6.926244	-0.04149	0.084142	-0.49309	0.621949	NA
LGALS8-AS	0.643725	-0.04149	0.046806	-0.88641	0.375395	NA
TBC1D2	50.13037	-0.04149	0.081537	-0.50884	0.610866	NA
INTS1	521.5551	-0.04148	0.067939	-0.61062	0.541454	0.802088
ISOC2	32.35598	0.041483	0.074416	0.557449	0.577221	NA
MAP3K9	459.0419	0.041481	0.064273	0.645394	0.518672	0.785288
FLJ34503	0.968639	-0.04148	0.052765	-0.78604	0.431841	NA
ST18	1247.54	0.041472	0.053859	0.770007	0.441296	0.742006
PRICKLE1	80.28709	0.041471	0.065198	0.636074	0.524728	NA
IER2	40.04394	0.041464	0.076534	0.541771	0.587976	NA
MARVELD	3.041505	0.041459	0.07625	0.543732	0.586626	NA
NEFH	527.5095	-0.04146	0.084752	-0.48917	0.624722	0.841451
MOCS2	102.9183	0.041453	0.060038	0.690444	0.489915	NA
ALG10	19.16614	0.041448	0.077473	0.534999	0.592651	NA
DNAH14	13.43747	0.041447	0.084935	0.487983	0.625562	NA
DHDH	3.531364	0.041429	0.075774	0.54674	0.584558	NA
DOCK3	957.3709	0.041424	0.034206	1.211003	0.225894	0.576749
NANOS1	29.78362	0.041423	0.077713	0.533027	0.594015	NA
MAP4K4	919.5009	-0.04141	0.048758	-0.84937	0.395674	0.705623
HDAC10	83.26316	0.041413	0.080352	0.515396	0.606276	NA
FAM49A	106.9033	-0.0414	0.054548	-0.75902	0.447843	NA
SLC6A5	4.428197	-0.0414	0.070011	-0.59136	0.554276	NA
NDUFA10	436.1813	-0.0414	0.038436	-1.07705	0.28146	0.616856
FGF9	165.1793	0.041392	0.048466	0.854039	0.393083	NA
TNKS2	415.2128	0.041381	0.044308	0.933939	0.350335	0.674334
CCNB1IP1	42.77055	0.041379	0.075212	0.550164	0.582207	NA
TRAPPC12	188.3316	-0.04137	0.0547	-0.75636	0.449435	NA
ABCC3	2.232829	-0.04137	0.069728	-0.5933	0.552982	NA
HIVEP3	473.5026	0.041355	0.046796	0.883741	0.376836	0.696762
CALY	63.87648	-0.04135	0.075451	-0.54808	0.583637	NA
TMEM168	60.04327	-0.04134	0.0662	-0.62451	0.532291	NA
APBB2	190.0422	-0.04134	0.064728	-0.63863	0.523061	NA
IFNA13	0.165188	0.041336	0.022154	1.865871	0.062059	NA
MIR659	0.964094	-0.04133	0.051614	-0.80068	0.423319	NA

ZBTB4	714.9037	0.041321	0.033495	1.233664	0.217328	0.568416
KAT2B	443.4563	-0.04132	0.047406	-0.8716	0.383425	0.70023
MAPKAP1	189.174	0.041311	0.045125	0.915472	0.359944	NA
LYPLA2	23.47566	0.041279	0.075059	0.549953	0.582352	NA
RAP1A	102.4663	0.041278	0.060573	0.681459	0.495581	NA
HMSD	0.395375	-0.04126	0.035425	-1.16483	0.244087	NA
DDIT3	54.01256	0.041256	0.079561	0.518546	0.604077	NA
PRSS46	1.07002	-0.04125	0.054138	-0.76202	0.446049	NA
VAMP1	114.2489	0.041249	0.079264	0.520402	0.602784	NA
MIR4288	1.189978	0.041227	0.054317	0.758999	0.447853	NA
UNC45A	199.9372	-0.04122	0.049844	-0.82702	0.408226	NA
MIR590	2.147423	0.041216	0.070793	0.582199	0.560433	NA
C3orf37	68.62796	0.041197	0.062689	0.657164	0.511075	NA
SNORD12B	6.548515	-0.04118	0.076232	-0.54025	0.589021	NA
AMICA1	63.41146	-0.04118	0.083919	-0.49069	0.623643	NA
MIR3671	3.112267	-0.04117	0.07334	-0.56139	0.574531	NA
PPCS	45.0938	0.041172	0.07125	0.577843	0.56337	NA
TMEM87B	76.03932	0.041154	0.066857	0.615562	0.538183	NA
SLC16A9	138.1741	-0.04115	0.075724	-0.54347	0.586808	NA
DHX58	11.93977	-0.04115	0.08357	-0.49236	0.622466	NA
BORA	16.42598	-0.04114	0.079854	-0.51525	0.606381	NA
FOXP1	172.8297	-0.04114	0.051059	-0.80576	0.420382	NA
LIMCH1	814.9537	0.041135	0.043637	0.942661	0.345854	0.670685
DNPEP	36.68095	-0.04113	0.069279	-0.59367	0.552732	NA
KLRAP1	39.52758	0.041128	0.077422	0.531223	0.595264	NA
FCAR	0.533601	-0.0411	0.037896	-1.08443	0.278172	NA
ZNF740	189.8587	-0.04108	0.041761	-0.98368	0.325272	NA
LOC10013	9.893783	-0.04108	0.084284	-0.48735	0.626008	NA
RBM12B-A	33.32347	0.041076	0.076084	0.53988	0.58928	NA
MIR26B	0.451286	-0.04106	0.038216	-1.07451	0.282594	NA
GOLGA8EP	1.935349	-0.04106	0.067449	-0.60876	0.542682	NA
C1orf106	0.625585	-0.04105	0.042316	-0.97014	0.331977	NA
TBC1D26	1.58578	-0.04105	0.064881	-0.63268	0.526941	NA
BRD8	394.2375	-0.04104	0.039573	-1.03702	0.299724	0.631658
IRS4	0.545573	-0.04104	0.038963	-1.05323	0.292237	NA
SRRM1	936.5895	-0.04103	0.048992	-0.83749	0.402318	0.709466
RFPL3S	1.701726	-0.04103	0.064187	-0.63918	0.522707	NA
KPNA7	0.150301	-0.04102	0.023121	-1.77425	0.076022	NA
FLJ41278	15.7435	-0.04102	0.084085	-0.48785	0.625653	NA
LINC00856	1.373626	-0.04102	0.059627	-0.68789	0.491522	NA
CORO7	2.323424	-0.04102	0.070679	-0.58031	0.561703	NA
LHFPL5	0.356874	-0.04102	0.032533	-1.26074	0.207403	NA
LINC00662	29.20521	0.041009	0.06874	0.596571	0.550794	NA
FAM98C	35.06166	-0.041	0.069492	-0.59001	0.555183	NA
LOC72887	0.695065	0.040995	0.044068	0.930277	0.352228	NA
LOC28569	2.046221	-0.04099	0.064448	-0.63605	0.524746	NA
ACD	63.55785	0.04099	0.057178	0.716879	0.473449	NA

ANKRD36	1256.586	-0.04098	0.054613	-0.75044	0.452992	0.750995
RANGAP1	175.3039	0.040982	0.056235	0.728775	0.46614	NA
COX11	118.6903	-0.04098	0.055877	-0.73332	0.463365	NA
LOC72789	15.90949	-0.04097	0.081159	-0.50487	0.613651	NA
SNORA70G	5.56333	-0.04097	0.084091	-0.48719	0.626122	NA
KCNIP4	317.7645	0.040967	0.058079	0.705372	0.480579	NA
ACTL7A	0.908729	-0.04095	0.049616	-0.82539	0.409148	NA
GBGT1	37.87767	-0.04095	0.071799	-0.57034	0.568448	NA
GAK	401.1813	-0.04095	0.052705	-0.77693	0.437203	0.739003
EMR2	7.188817	-0.04094	0.079061	-0.51786	0.604558	NA
ULBP2	3.167746	0.040939	0.076792	0.533111	0.593957	NA
SNORD61	29.1134	-0.04094	0.083813	-0.48845	0.625234	NA
C19orf21	0.435217	0.040908	0.037433	1.092847	0.274461	NA
YY1	265.2744	0.040902	0.044818	0.912615	0.361445	NA
DROSHA	473.9957	0.040902	0.0317	1.290278	0.196954	0.545356
MX1	36.63139	-0.04089	0.084909	-0.48161	0.63008	NA
NUFIP1	40.15422	0.040892	0.070165	0.582795	0.560031	NA
TMEM106	496.9093	0.04089	0.061554	0.664292	0.506504	0.780812
SHOC2	517.4143	0.040887	0.045008	0.908419	0.363657	0.686402
RBM25	1282.258	-0.04088	0.060604	-0.67459	0.499934	0.778081
PEX3	90.88754	-0.04088	0.056899	-0.7184	0.472511	NA
SRXN1	69.56163	0.040871	0.071329	0.572993	0.566649	NA
HTR7P1	7.119896	0.040865	0.077421	0.527828	0.597619	NA
EIF3J	136.2022	0.040856	0.043116	0.947586	0.34334	NA
CCZ1B	27.24164	0.040852	0.079834	0.511717	0.608849	NA
ZNF541	5.29376	-0.04083	0.0771	-0.52959	0.596394	NA
IFNLR1	61.29317	-0.04083	0.057892	-0.70522	0.480672	NA
IFIT3	73.08994	-0.04083	0.06421	-0.63582	0.524894	NA
SEL1L2	0.25041	-0.04082	0.029295	-1.39351	0.163466	NA
TBC1D4	258.9995	0.040806	0.066855	0.610362	0.541622	NA
CBR4	131.4803	-0.04079	0.056461	-0.72249	0.469996	NA
C1orf227	0.898215	-0.04079	0.048882	-0.83448	0.404011	NA
TEX30	20.00598	-0.04079	0.084531	-0.48255	0.629413	NA
AKIRIN2	83.43682	0.040788	0.063469	0.642651	0.520451	NA
PRC1	154.4921	-0.04077	0.051106	-0.79782	0.424978	NA
ZNF385B	231.6071	0.040769	0.048875	0.834153	0.404195	NA
ZNF30	99.08485	0.040768	0.066391	0.61407	0.539169	NA
IGFBP7	29.41914	0.040767	0.081702	0.498973	0.617799	NA
FBRS	202.6491	0.040767	0.056932	0.71606	0.473954	NA
ANXA13	0.148121	0.040763	0.022004	1.852523	0.063951	NA
RBM48	103.5174	0.04076	0.055973	0.728198	0.466492	NA
SNRNP200	1043.083	0.040757	0.033784	1.206399	0.227664	0.577536
AGMAT	4.029233	-0.04075	0.077605	-0.52513	0.59949	NA
MCMDC2	44.74794	-0.04075	0.084083	-0.48466	0.62792	NA
RGS3	36.30427	-0.04075	0.068359	-0.59611	0.551099	NA
EXOC6B	473.281	0.040748	0.04625	0.881047	0.378292	0.698315
SETD7	349.2878	0.040747	0.05455	0.746964	0.455086	0.750995

LOC10028	1.432018	0.040733	0.061498	0.662349	0.507747	NA
TMF1	505.2123	0.040733	0.035329	1.152942	0.248934	0.592704
ASIC2	54.81963	0.04073	0.079747	0.510738	0.609534	NA
NAPSB	17.35261	-0.04072	0.084751	-0.48047	0.630892	NA
ARHGEF10	815.212	0.040716	0.056771	0.717201	0.47325	0.759174
CCDC170	4.395797	-0.04072	0.075109	-0.54209	0.587756	NA
ZNF876P	73.10262	0.040711	0.069615	0.584804	0.558679	NA
CACNA1H	11.88562	-0.04071	0.084922	-0.47938	0.631669	NA
INPP5E	114.0856	0.040708	0.063885	0.637209	0.523989	NA
IFNGR2	51.33673	-0.0407	0.062768	-0.64836	0.516751	NA
MTO1	146.7443	0.040686	0.053039	0.767099	0.443023	NA
FAIM2	718.7484	0.040681	0.056038	0.72596	0.467863	0.755844
CNOT7	256.5342	0.040664	0.041322	0.98406	0.325086	NA
UBE3D	27.65501	-0.04066	0.075551	-0.53815	0.590472	NA
MAGEA8	5.360734	0.040645	0.080193	0.506837	0.612269	NA
EPC1	332.3466	0.040643	0.035298	1.151434	0.249554	NA
TIMELESS	49.29141	0.040633	0.075458	0.538488	0.59024	NA
CCBL2	64.07578	-0.04063	0.074668	-0.5441	0.586372	NA
BPTF	3467.843	0.040627	0.031071	1.307549	0.191026	0.542245
FAM13A-A	128.3441	-0.04062	0.055892	-0.72677	0.467368	NA
RGL1	299.2122	-0.04061	0.043693	-0.9295	0.352628	NA
CEP95	374.9967	-0.04061	0.054388	-0.74671	0.455239	0.750995
MYL7	1.805931	-0.04058	0.066407	-0.61114	0.541106	NA
PCGF6	26.69438	-0.04058	0.078081	-0.51967	0.603293	NA
ZBTB26	27.96129	0.040547	0.07209	0.56244	0.573816	NA
LOC10105	0.853428	-0.04054	0.048155	-0.84197	0.399804	NA
PDCL3	21.37534	0.040542	0.082235	0.493008	0.622007	NA
HHATL	21.6665	0.040529	0.084862	0.477589	0.632943	NA
WDFY3-AS	24.47791	-0.04053	0.079351	-0.51074	0.609531	NA
SSBP2	405.7505	0.040523	0.039296	1.031235	0.302431	0.633092
HAMP	1.643541	-0.04052	0.045016	-0.90016	0.368036	NA
PLBD1	4.642113	0.040507	0.08196	0.494231	0.621143	NA
TWISTNB	49.9403	0.040503	0.0672	0.602731	0.546688	NA
STRBP	334.0006	0.040496	0.043586	0.929116	0.352829	NA
OXSR1	259.7572	0.040487	0.046851	0.864165	0.387497	NA
MTRNR2L	24.58262	-0.04049	0.08172	-0.49541	0.620308	NA
SPIN3	205.3123	-0.04048	0.04528	-0.89409	0.371275	NA
RSPRY1	62.23824	-0.04047	0.065203	-0.62068	0.534809	NA
PLIN1	12.73934	-0.04047	0.08166	-0.49559	0.620183	NA
ANKRD20A	3.061252	-0.04047	0.075051	-0.53923	0.589727	NA
RAB11B	219.4278	-0.04047	0.049458	-0.81822	0.413232	NA
C3orf67	2.538483	0.040467	0.069745	0.580215	0.56177	NA
ZNF225	71.00043	0.040464	0.05163	0.783723	0.433203	NA
KLF2	20.1539	0.040461	0.080134	0.504917	0.613617	NA
HRAS	101.4654	-0.04046	0.052996	-0.76344	0.445199	NA
RSF1	1100.547	0.040456	0.035648	1.134854	0.256436	0.598301
MRPL51	35.70055	-0.04044	0.080154	-0.50455	0.613876	NA

NYNRIN	425.9309	0.040441	0.068478	0.590575	0.554805	0.808857
RANGRF	1.294347	-0.04043	0.058954	-0.68583	0.492821	NA
RAC2	1.860235	-0.04043	0.058794	-0.68766	0.491665	NA
C1orf35	92.70626	0.040428	0.069974	0.577757	0.563429	NA
CHN1	203.3341	0.040421	0.08161	0.495291	0.620395	NA
RAB11B-A	3.504203	-0.04041	0.077685	-0.52023	0.602901	NA
GBP5	1.475221	0.040414	0.064119	0.630299	0.528499	NA
KIRREL3-A	0.213827	0.040411	0.025325	1.595695	0.110557	NA
ARFGEF2	441.4409	0.040411	0.049487	0.816595	0.41416	0.71938
INTS7	65.33389	-0.0404	0.073693	-0.54825	0.583522	NA
EID2	47.53556	0.040394	0.076563	0.52759	0.597784	NA
GPR150	2.604186	-0.04039	0.071406	-0.56569	0.571607	NA
BBS2	251.4379	-0.04039	0.054585	-0.73988	0.459375	NA
USP36	323.7403	-0.04038	0.047778	-0.84515	0.398026	NA
ATF7IP2	27.53982	0.040378	0.083098	0.485911	0.627031	NA
ZNF250	192.2481	0.04036	0.044929	0.898298	0.369027	NA
ARPC3	142.3862	-0.04036	0.069726	-0.57878	0.56274	NA
PAK2	290.3181	0.040343	0.040153	1.004731	0.315026	NA
ZNF77	65.34006	-0.04034	0.066662	-0.60518	0.545059	NA
ZMAT2	161.7201	0.040334	0.053626	0.752137	0.451969	NA
NOTUM	2.382367	-0.04033	0.066208	-0.60913	0.542436	NA
CDK20	35.90764	-0.04033	0.069584	-0.57953	0.562229	NA
C9orf37	36.20601	-0.04032	0.072371	-0.55714	0.577433	NA
SLED1	4.054098	-0.04031	0.079834	-0.50497	0.613579	NA
MIR29B2	21.28033	0.040311	0.08399	0.479954	0.63126	NA
C9orf57	1.713973	0.040302	0.063582	0.633858	0.526173	NA
SNORD115	36.14178	-0.0403	0.084347	-0.4778	0.632789	NA
POMT1	190.8416	-0.0403	0.058012	-0.69463	0.487286	NA
TYMP	31.81852	-0.04028	0.083443	-0.48274	0.629281	NA
CYTH2	171.9903	0.04028	0.073128	0.550809	0.581765	NA
KLHL14	1.455191	-0.04027	0.060796	-0.66235	0.507744	NA
SHOX2	0.380968	-0.04026	0.031423	-1.28132	0.20008	NA
NEIL1	100.8734	-0.04026	0.062475	-0.64441	0.519312	NA
TEX38	2.026982	0.040256	0.066719	0.603363	0.546267	NA
TAF3	185.5761	-0.04025	0.053437	-0.75321	0.451322	NA
SRRD	51.34016	0.040242	0.064972	0.619379	0.535667	NA
ACTR3B	64.68927	-0.04024	0.058316	-0.69001	0.49019	NA
KCNH3	48.0471	0.04023	0.083382	0.482479	0.629466	NA
TOB2P1	2.987319	0.04023	0.074387	0.540822	0.588631	NA
ERCC6	336.1653	-0.04022	0.045012	-0.89354	0.371568	0.695376
AP5Z1	130.8304	-0.04021	0.061002	-0.6592	0.50977	NA
PTPDC1	108.1279	-0.04021	0.049545	-0.8116	0.41702	NA
C20orf27	17.74906	0.040193	0.080408	0.499857	0.617176	NA
ANXA11	173.6185	-0.04019	0.069135	-0.58131	0.561031	NA
LENG9	5.207611	-0.04017	0.082396	-0.48755	0.625865	NA
CDC42BPG	270.3106	0.04017	0.059743	0.672384	0.501339	NA
SMARCA5	721.51	0.040163	0.030879	1.300656	0.193376	0.542747

PPHLN1	242.0598	0.040159	0.046303	0.867324	0.385765	NA
CYCS	207.6235	0.040159	0.072774	0.551826	0.581068	NA
FAM72A	1.042444	0.040158	0.054456	0.737443	0.460853	NA
STIM1	150.2707	0.040152	0.049352	0.813597	0.415876	NA
SUPT3H	105.4759	-0.04015	0.059453	-0.67536	0.499449	NA
CDT1	22.00562	-0.04015	0.082893	-0.4843	0.628172	NA
GNA14	4.927089	-0.04014	0.08157	-0.4921	0.622647	NA
C9orf84	85.1977	0.040139	0.084933	0.4726	0.636499	NA
FLJ41200	2.668596	0.040136	0.069769	0.575268	0.56511	NA
MYLPF	1.764628	0.040131	0.062611	0.640956	0.521551	NA
PXT1	0.907216	-0.04012	0.052134	-0.76957	0.441555	NA
DNAJC14	95.14762	0.04011	0.047473	0.844908	0.398162	NA
KCNJ10	179.4401	0.040107	0.077755	0.515813	0.605985	NA
PCYOX1L	65.91883	-0.0401	0.083541	-0.47997	0.63125	NA
HIST1H4I	0.66487	-0.04008	0.042006	-0.95403	0.340067	NA
CNOT3	318.4808	-0.04007	0.054954	-0.72924	0.465856	NA
GCLM	34.92101	0.040049	0.073051	0.548241	0.583527	NA
THBS2	25.60533	0.040048	0.083195	0.481376	0.63025	NA
MPP3	260.2881	0.040043	0.059022	0.678448	0.497488	NA
FLI1	17.79273	-0.04004	0.084788	-0.47224	0.636753	NA
GPR88	0.239841	-0.04002	0.029263	-1.36755	0.171454	NA
USP39	66.9083	0.040013	0.054061	0.740143	0.459213	NA
CADPS	751.0775	0.040012	0.048961	0.817225	0.4138	0.719306
RTF1	408.7547	-0.04001	0.060406	-0.66229	0.507786	0.780812
SNORA5B	0.334629	0.040001	0.03378	1.184157	0.236351	NA
RRNAD1	39.31578	0.039986	0.067378	0.593456	0.552876	NA
SMC3	590.067	0.039985	0.038711	1.032933	0.301635	0.632662
RPH3AL	34.42183	-0.03998	0.075589	-0.52885	0.596912	NA
MIR450B	0.221589	0.039973	0.028624	1.396526	0.162556	NA
SPEF1	4.208235	0.039932	0.076609	0.521246	0.602196	NA
PLA2G10	3.716017	0.039931	0.077495	0.515266	0.606367	NA
VSTM2B	71.29574	-0.03993	0.084534	-0.47234	0.636687	NA
ROCK1P1	7.644446	0.039928	0.075178	0.531116	0.595338	NA
MOSPD1	12.36724	-0.03993	0.08246	-0.4842	0.628241	NA
RPS6KA1	94.62857	-0.03992	0.065558	-0.60899	0.542528	NA
RAPH1	492.0098	0.039916	0.038309	1.041963	0.297429	0.630193
LOC10013	39.24088	0.039896	0.078237	0.509945	0.61009	NA
OSBPL8	421.8088	0.039895	0.043195	0.923606	0.355691	0.680826
RAB18	307.1292	0.039889	0.0429	0.929815	0.352467	NA
HIF1AN	177.0119	-0.03988	0.04946	-0.80641	0.420008	NA
TNFRSF13	4.546092	0.039882	0.080454	0.495711	0.620098	NA
SCARNA8	4.427605	-0.03988	0.073463	-0.54283	0.587249	NA
SLC17A4	5.909436	-0.03985	0.07461	-0.53416	0.593232	NA
PRCC	149.39	-0.03985	0.045656	-0.87282	0.382761	NA
CEPT1	140.3569	-0.03985	0.047197	-0.84426	0.398526	NA
SEZ6	297.6559	0.03983	0.065108	0.61176	0.540697	NA
MYPN	1.391215	-0.03983	0.046826	-0.85056	0.395016	NA

PCCA-AS1	9.292114	-0.03983	0.084706	-0.47016	0.638241	NA
TOMM40	70.20264	-0.03982	0.061019	-0.6526	0.514014	NA
ESCO2	0.558918	-0.03981	0.042092	-0.94576	0.34427	NA
INPP4A	809.9504	-0.0398	0.034581	-1.15105	0.249712	0.592704
SLC2A4RG	77.55211	0.039795	0.076094	0.522968	0.600997	NA
CTNNBL1	125.3563	0.03979	0.050283	0.791329	0.428752	NA
PIN1P1	1.241934	-0.03979	0.058978	-0.6746	0.49993	NA
CBX7	529.2553	-0.03978	0.043783	-0.90858	0.363571	0.686402
CYP4Z2P	0.288345	-0.03974	0.028985	-1.37111	0.170342	NA
KIF12	2.712568	-0.03973	0.056399	-0.70446	0.481143	NA
CHPF	51.01914	0.039729	0.068691	0.578376	0.563011	NA
ZNF107	107.4513	0.039716	0.067153	0.59143	0.554232	NA
NUP210L	53.11312	0.039712	0.084718	0.468761	0.639241	NA
DAO	102.5256	-0.03969	0.081113	-0.48937	0.62458	NA
MED17	120.7111	0.039694	0.052804	0.751711	0.452225	NA
KIF1B	4822.659	0.039687	0.03195	1.242153	0.21418	0.562782
GTSE1	2.433231	0.039672	0.071296	0.556438	0.577912	NA
FLJ37453	29.25776	-0.03966	0.077419	-0.51229	0.60845	NA
ANKRD18A	33.02233	0.039658	0.07313	0.542298	0.587613	NA
GIMAP1	3.197941	0.039657	0.075887	0.522575	0.60127	NA
ZNF131	191.7982	0.039656	0.044074	0.89976	0.368248	NA
MZF1	136.8676	0.039655	0.059376	0.667869	0.504217	NA
FMO5	58.08133	0.039652	0.072722	0.545256	0.585577	NA
MIR181B2	1.38159	-0.03965	0.059557	-0.66578	0.505551	NA
GOLGA6L5	5.748728	-0.03965	0.080574	-0.49204	0.622694	NA
NLRP1	12.44962	-0.03965	0.084939	-0.46675	0.640682	NA
ZNF780B	450.9745	0.039637	0.040314	0.983205	0.325507	0.655185
ADCY3	168.1697	-0.03962	0.054165	-0.73154	0.464447	NA
MVB12A	16.34379	-0.03962	0.080538	-0.49193	0.622766	NA
ANKRD23	134.1206	-0.03961	0.070659	-0.56065	0.575039	NA
FXR1	703.4809	0.03961	0.048126	0.823043	0.410484	0.715365
MIR29A	0.748976	-0.0396	0.048979	-0.8086	0.418745	NA
SNORD37	2.679365	0.039601	0.073008	0.542412	0.587535	NA
RFFL	11.03012	0.039574	0.084779	0.466791	0.640649	NA
MYL12B	206.4571	-0.03957	0.077024	-0.51377	0.607412	NA
ABCB8	204.773	-0.03957	0.062586	-0.63226	0.527214	NA
DNAJC27-	56.43452	-0.03957	0.078634	-0.50316	0.614848	NA
CSNK1G2-	7.724539	0.039563	0.084901	0.465991	0.641222	NA
SEC22B	162.0375	0.039554	0.043413	0.911097	0.362244	NA
FAM66B	15.18724	-0.03955	0.084928	-0.46573	0.641408	NA
RBM38	60.42058	0.039551	0.061129	0.64702	0.517619	NA
B3GALNT2	85.8905	-0.03955	0.050987	-0.77569	0.437932	NA
SAA2	0.525746	-0.03955	0.037231	-1.06229	0.288103	NA
TRMT13	139.3957	-0.03955	0.049942	-0.7919	0.42842	NA
LIN54	134.9808	0.039545	0.050314	0.78597	0.431885	NA
STRADB	39.75663	0.039531	0.077219	0.511937	0.608695	NA
PDCD2L	14.3433	0.03953	0.081533	0.484832	0.627796	NA

DCAF4L1	12.25294	0.039528	0.081131	0.48722	0.626103	NA
CD7	0.591635	-0.03952	0.044521	-0.88775	0.374675	NA
LRRFIP2	468.2569	0.039516	0.045232	0.873616	0.382328	0.70023
C12orf44	28.35303	0.039512	0.078777	0.501565	0.615974	NA
RGS9BP	2.09201	0.039491	0.069076	0.571696	0.567528	NA
PSMA7	113.7065	-0.03949	0.053638	-0.73622	0.461595	NA
UBE2J2	84.6525	0.039486	0.051107	0.772603	0.439757	NA
TMEM8A	75.86138	0.039478	0.070133	0.562909	0.573497	NA
SLC2A9	2.031211	-0.03948	0.069363	-0.56912	0.569275	NA
PVR	196.8496	0.039475	0.048939	0.806603	0.419895	NA
SLC25A13	78.44671	-0.03947	0.062382	-0.63271	0.526925	NA
HIST1H3B	0.184567	-0.03947	0.021258	-1.85667	0.063359	NA
LIPT1	27.89146	0.039466	0.078286	0.504122	0.614176	NA
CAMTA2	568.6684	0.039461	0.038512	1.024645	0.305531	0.635668
CLEC18C	0.51565	-0.03946	0.038352	-1.02879	0.303579	NA
ASB2	1.189784	-0.03946	0.04963	-0.79502	0.426605	NA
PTPMT1	45.51497	-0.03945	0.071037	-0.5554	0.57862	NA
BBS9	170.9368	0.039448	0.056656	0.696263	0.486264	NA
MRPL22	45.54823	0.039442	0.069011	0.571523	0.567645	NA
TLR1	6.653389	0.039422	0.082187	0.479666	0.631465	NA
PKD1L3	13.84042	-0.03942	0.083298	-0.47326	0.636026	NA
ALB	0.561538	-0.03942	0.038422	-1.02596	0.304911	NA
TCEA1	65.79346	0.039413	0.058327	0.67572	0.499218	NA
AKR7L	22.24743	-0.0394	0.076024	-0.51822	0.604306	NA
FAM13A	442.7633	-0.03939	0.051965	-0.75798	0.448461	0.747436
C2orf74	42.63228	0.039382	0.07769	0.506904	0.612222	NA
HMGN5	13.90105	0.03938	0.083506	0.471585	0.637223	NA
KCTD4	6.108337	-0.03937	0.083572	-0.47112	0.637558	NA
DNAJB2	350.9354	0.03937	0.055282	0.712169	0.47636	0.759174
TBP	105.415	-0.03936	0.051661	-0.76193	0.446102	NA
C7orf25	54.31235	0.039357	0.072563	0.542384	0.587554	NA
METRNL	17.78727	0.039355	0.084697	0.464658	0.642176	NA
CDC27	348.9859	0.03935	0.042276	0.9308	0.351957	0.675579
SUN1	815.7255	-0.03934	0.038949	-1.01015	0.312423	0.642968
NLGN2	360.5142	-0.03934	0.048355	-0.8135	0.415929	0.720795
RNF130	315.7051	0.039336	0.037238	1.056352	0.290807	NA
LZTFL1	81.96982	0.039336	0.059149	0.665032	0.50603	NA
SLC6A8	151.3204	-0.03934	0.047801	-0.82289	0.41057	NA
DCLRE1C	113.835	-0.03933	0.050639	-0.7767	0.437336	NA
XRCC2	14.43906	-0.03933	0.084799	-0.46377	0.64281	NA
RHPN2	44.03646	0.039312	0.069551	0.565222	0.571923	NA
SPATA5L1	60.99939	0.039311	0.06014	0.653653	0.513335	NA
FERMT1	10.87823	-0.0393	0.084902	-0.46293	0.643413	NA
ATF4	254.8862	-0.0393	0.056628	-0.69396	0.487705	NA
ZNF736	67.64192	-0.0393	0.063869	-0.61526	0.538382	NA
PCDHGA4	67.42984	0.039275	0.081281	0.483204	0.628951	NA
LOC10013	0.208654	-0.03927	0.02742	-1.43225	0.152073	NA

PSMC1	67.25331	0.03927	0.054794	0.716672	0.473576	NA
LOC10050	1.903181	0.039266	0.058361	0.672803	0.501073	NA
KIAA0226L	14.02714	-0.03926	0.0849	-0.46245	0.643757	NA
ANO4	40.64756	-0.03926	0.081973	-0.47895	0.631972	NA
TSPAN19	0.693272	-0.03926	0.046676	-0.84107	0.400309	NA
ZNF772	199.1258	0.039253	0.045826	0.85656	0.391688	NA
STK4-AS1	0.571766	0.039244	0.042336	0.926957	0.353949	NA
PGM3	214.3895	-0.03923	0.050663	-0.77442	0.438683	NA
RGPD3	0.996614	0.03921	0.053009	0.739683	0.459493	NA
FMO6P	1.315921	-0.03921	0.055418	-0.70747	0.479272	NA
EP300	1184.963	0.039204	0.037757	1.038318	0.299122	0.631658
ATP6V0A4	0.277977	0.039202	0.027229	1.439691	0.149955	NA
PTX4	0.909514	0.039192	0.053506	0.732482	0.463874	NA
GOLGA3	1033.966	0.039192	0.071744	0.546266	0.584883	0.824332
RRP36	70.49516	0.039191	0.057193	0.685231	0.493198	NA
FLJ35390	21.46922	0.03918	0.076417	0.512708	0.608156	NA
ASB9P1	7.720758	0.039179	0.084217	0.46522	0.641774	NA
MOBP	347.5281	-0.03916	0.08024	-0.48798	0.625563	0.84189
UTS2R	3.002434	-0.03915	0.069259	-0.56528	0.571886	NA
CD69	0.569263	-0.03913	0.041943	-0.93287	0.350887	NA
SRSF7	216.947	0.039125	0.047987	0.81532	0.414889	NA
SLC25A12	281.0983	0.039123	0.06051	0.64655	0.517923	NA
LINC00628	1.054717	0.039118	0.049738	0.786488	0.431582	NA
SAA1	0.289737	0.039116	0.029862	1.309888	0.190234	NA
ACER2	25.11805	0.039108	0.081439	0.480215	0.631075	NA
LTBP4	12.84674	-0.0391	0.083938	-0.4658	0.641359	NA
PGAM1	57.38881	0.039098	0.07199	0.543102	0.587059	NA
SETMAR	43.60821	-0.03909	0.06787	-0.57599	0.56462	NA
LOC10028	3.554799	0.039086	0.078474	0.498077	0.61843	NA
EFTUD1P1	2.067847	-0.03908	0.066112	-0.59117	0.554408	NA
VWDE	0.73159	0.03908	0.042698	0.915273	0.360048	NA
HIST1H3H	0.6799	-0.03908	0.043486	-0.89868	0.368825	NA
LOC10012	5.851126	-0.03908	0.084057	-0.46487	0.642023	NA
CD276	29.30456	0.039071	0.076517	0.510612	0.609623	NA
ELMO2	347.7421	0.039068	0.039734	0.983247	0.325486	0.655185
ACOT2	34.10713	0.039064	0.073379	0.532354	0.594481	NA
TRIM16L	5.013899	-0.03906	0.083079	-0.4702	0.638215	NA
CLK2	205.6792	0.039063	0.056318	0.69361	0.487927	NA
LRRC23	48.90572	-0.03906	0.067992	-0.57445	0.565663	NA
TMCC2	362.4368	0.039056	0.052675	0.741459	0.458415	0.754035
TDP2	131.889	0.039039	0.062813	0.621504	0.534268	NA
ZNF207	461.2452	-0.03903	0.042342	-0.92188	0.356593	0.680826
C19orf48	14.21163	-0.03903	0.081631	-0.47812	0.632562	NA
CATSPER4	0.342164	-0.03902	0.033597	-1.16155	0.245419	NA
SPTLC2	185.4578	0.039013	0.060617	0.643585	0.519844	NA
NSFL1C	221.1699	-0.03901	0.04312	-0.90472	0.365615	NA
ZNF343	148.0901	-0.03901	0.050671	-0.76984	0.441392	NA

C10orf76	216.2883	-0.03901	0.049268	-0.79177	0.428496	NA
CHRND	0.350247	-0.039	0.030383	-1.28378	0.199218	NA
TFB1M	42.63088	-0.039	0.067294	-0.57958	0.562201	NA
MSH5-SAP	0.603677	-0.03899	0.045538	-0.85627	0.39185	NA
MMS22L	24.60065	-0.03899	0.08097	-0.48156	0.630118	NA
CA4	295.6935	0.03899	0.082594	0.472065	0.636881	NA
C20orf78	0.226525	-0.03898	0.025648	-1.51987	0.128543	NA
MAP1LC3B	421.0406	-0.03898	0.0663	-0.58793	0.55658	0.808857
RASGEF1B	44.2779	0.038979	0.081736	0.476888	0.633442	NA
FANCA	52.00411	-0.03898	0.063475	-0.61408	0.539161	NA
CARD16	2.520624	-0.03898	0.068517	-0.56889	0.569432	NA
PHLDA3	19.25799	-0.03897	0.081638	-0.47736	0.633109	NA
MTTP	0.989776	-0.03897	0.050515	-0.77144	0.440445	NA
MYOM1	92.13158	-0.03897	0.066838	-0.58303	0.559875	NA
FPGS	79.76545	-0.03896	0.065887	-0.59135	0.554289	NA
EIF4G1	698.1554	0.038957	0.043083	0.904236	0.36587	0.688858
NOL4	210.847	0.038956	0.044156	0.882222	0.377657	NA
RAI14	166.7202	0.038949	0.084622	0.460276	0.645318	NA
LOC10013	2.473038	-0.03894	0.066451	-0.58605	0.557839	NA
APOL4	1.802761	-0.03894	0.053168	-0.73233	0.463967	NA
NFKBIZ	46.91959	-0.03893	0.069582	-0.5595	0.575821	NA
FMN2	565.6217	0.038924	0.042218	0.921993	0.356532	0.680826
SDE2	100.2309	0.038921	0.053457	0.728082	0.466563	NA
DRAP1	153.8508	0.038916	0.053677	0.725009	0.468446	NA
PPAPDC1A	59.00503	-0.03891	0.063345	-0.61419	0.539087	NA
CNPY2	90.58774	-0.0389	0.046038	-0.84501	0.398104	NA
CCDC64	157.1072	0.038894	0.074489	0.522151	0.601565	NA
SNORA33	5.431383	-0.03889	0.082849	-0.46937	0.638808	NA
TSPAN18	323.7804	0.038885	0.056675	0.686115	0.49264	NA
IRF9	50.35621	-0.03887	0.077075	-0.50431	0.614041	NA
C15orf27	276.5296	0.038867	0.049281	0.788681	0.430298	NA
GPR32	0.516104	-0.03887	0.040468	-0.96039	0.336858	NA
HTR1B	0.804652	0.038862	0.047608	0.816283	0.414339	NA
ERF	26.7251	-0.03885	0.079295	-0.49	0.624133	NA
ZNF25	422.9447	0.038853	0.058959	0.658981	0.509908	0.781767
KLRC4	1.04337	-0.03885	0.048715	-0.79751	0.425157	NA
PKN2	513.1921	-0.03885	0.051156	-0.75938	0.447626	0.746593
TOMM20L	3.780477	-0.03883	0.079135	-0.49071	0.623634	NA
LINC00478	182.2709	-0.03883	0.060638	-0.64036	0.521942	NA
NUP88	168.8789	0.038822	0.061479	0.631469	0.527734	NA
SERPINB6	134.5619	-0.03881	0.050511	-0.76831	0.442301	NA
FGF17	125.5883	-0.0388	0.076859	-0.50486	0.613656	NA
C9orf66	0.654494	0.038801	0.04575	0.848108	0.396378	NA
ENTPD4	309.2454	0.038795	0.048004	0.808157	0.419	NA
HIVEP1	231.0736	0.038777	0.062448	0.620942	0.534637	NA
C11orf82	0.843682	0.038771	0.051255	0.756421	0.449397	NA
RANBP10	243.393	-0.03876	0.049362	-0.78513	0.43238	NA

PDC	17.06643	-0.03875	0.082404	-0.47026	0.638169	NA
USB1	18.8257	-0.03875	0.078243	-0.49522	0.620442	NA
SLC27A2	20.43559	-0.03874	0.083571	-0.46351	0.642995	NA
CCL5	2.661228	0.038734	0.070231	0.551525	0.581274	NA
CUL5	373.1625	0.038718	0.045523	0.85052	0.395036	0.705623
TEAD4	7.575061	0.038716	0.081487	0.475116	0.634705	NA
IL21R	2.784403	-0.03871	0.072017	-0.53758	0.59087	NA
ORC5	66.86116	-0.03871	0.062859	-0.61576	0.538052	NA
HTT	1095.225	0.038702	0.035977	1.075733	0.282047	0.617151
MRPS9	104.7343	-0.0387	0.056134	-0.68942	0.490556	NA
PHLDB3	13.18858	0.0387	0.084685	0.456982	0.647684	NA
MIR4694	1.217063	-0.0387	0.058651	-0.65978	0.509395	NA
LOC10013	2.118297	0.038695	0.067529	0.573013	0.566636	NA
ZNF404	43.71807	-0.03869	0.073842	-0.52399	0.600288	NA
TMPRSS11	6.911369	-0.03868	0.079365	-0.48734	0.626018	NA
ATL2	305.668	0.038674	0.057119	0.67709	0.498349	NA
LOC25541	0.496081	-0.03867	0.039349	-0.98282	0.325694	NA
C6orf118	1.010245	-0.03867	0.050231	-0.76982	0.441408	NA
CDA	1.235684	0.038667	0.059334	0.651687	0.514603	NA
FLJ23867	22.04351	-0.03866	0.08017	-0.48227	0.629612	NA
LINC00707	4.429244	0.038655	0.077838	0.496607	0.619466	NA
XPR1	254.4275	0.038654	0.039328	0.982848	0.325682	NA
CCDC64B	0.838134	0.038649	0.051902	0.744653	0.456481	NA
KIAA1429	342.479	0.038643	0.039642	0.9748	0.329659	0.65961
CACNA2D2	292.3095	0.038634	0.058923	0.655662	0.512042	NA
NGLY1	118.1256	0.03862	0.049825	0.775124	0.438266	NA
SULT1C2P	0.432355	-0.0386	0.036824	-1.04835	0.29448	NA
PLEKHA8	190.7722	0.038599	0.060283	0.640304	0.521975	NA
RGPD8	0.201474	0.038598	0.021428	1.801344	0.071649	NA
EML2	72.43121	-0.03859	0.06104	-0.63229	0.527198	NA
ARHGEF9	457.1415	0.038589	0.055569	0.694437	0.487408	0.765058
LRTM2	4.907159	-0.03858	0.074693	-0.51652	0.605489	NA
KCNJ5	1.411835	-0.03858	0.060595	-0.6367	0.524323	NA
TERF1	167.9267	-0.03858	0.055998	-0.68888	0.4909	NA
ATP5H	128.3367	-0.03857	0.065478	-0.5891	0.555796	NA
TTC8	60.5522	-0.03857	0.076407	-0.50477	0.613723	NA
SERPINI1	46.49878	-0.03856	0.079778	-0.48335	0.628846	NA
SARS	393.9556	-0.03856	0.04241	-0.90916	0.363265	0.686402
TMEM62	96.02647	0.038552	0.071417	0.539816	0.589324	NA
PARS2	12.44051	0.038529	0.083753	0.460035	0.645491	NA
CLASP1	1173.361	-0.03853	0.040014	-0.96282	0.335638	0.661751
C19orf70	17.42321	0.038512	0.080897	0.476057	0.634034	NA
SCRT1	309.7625	0.038503	0.057306	0.671882	0.501659	NA
THAP3	48.07842	-0.0385	0.067912	-0.56687	0.570806	NA
KCNG4	2.268932	0.038489	0.065235	0.590017	0.55518	NA
SGSM3	363.9882	-0.03849	0.064568	-0.59606	0.551137	0.805737
HSPA12B	10.62838	-0.03849	0.084374	-0.45614	0.648291	NA

LOC79015	1.386027	0.03848	0.054706	0.703397	0.481808	NA
LGALS8	223.256	-0.03848	0.047982	-0.80191	0.422605	NA
MARVELD	9.194587	0.038476	0.084886	0.453266	0.650358	NA
SPSB4	1.425284	-0.03848	0.060136	-0.63981	0.522296	NA
KDM4E	0.489471	0.038475	0.03938	0.977028	0.328555	NA
HCST	0.582285	-0.03847	0.04354	-0.88367	0.376872	NA
CPNE6	130.1515	0.038471	0.068508	0.561559	0.574417	NA
ANAPC13	32.84423	0.038466	0.081719	0.470709	0.637849	NA
TAF7	412.1757	0.038458	0.054958	0.699759	0.484078	0.764934
KIF21B	131.9745	0.03845	0.066147	0.581287	0.561047	NA
AGO3	254.9809	0.038444	0.048532	0.79214	0.428279	NA
HERC2P9	62.01286	-0.03844	0.078899	-0.48725	0.62608	NA
EPAS1	224.5023	-0.03844	0.074775	-0.51407	0.607205	NA
RNF24	79.87519	-0.03842	0.078957	-0.48662	0.626529	NA
HTRA3	0.570086	-0.03839	0.043313	-0.88645	0.375376	NA
VTN	20.88399	-0.03839	0.0798	-0.48105	0.630482	NA
XRCC6	377.6558	0.038387	0.052916	0.725429	0.468189	0.755844
ZNF831	4.936289	-0.03838	0.063181	-0.60745	0.543555	NA
GOSR2	161.5482	-0.03838	0.049267	-0.77894	0.436016	NA
FAM127C	23.37313	-0.03837	0.073069	-0.52507	0.599533	NA
POU2AF1	0.440463	-0.03837	0.034485	-1.11251	0.265919	NA
SNORA52	1.579087	-0.03836	0.056719	-0.67637	0.498805	NA
BAZ2B	2075.399	-0.03836	0.039808	-0.96358	0.335256	0.661751
SLC2A5	11.22511	-0.03835	0.079594	-0.48188	0.629891	NA
C15orf54	0.722895	0.038334	0.044278	0.865759	0.386622	NA
C8orf46	32.48836	-0.03833	0.080449	-0.47646	0.633748	NA
RSBN1	421.3427	0.038327	0.03827	1.001508	0.316581	0.645996
TACO1	28.38955	-0.03832	0.073695	-0.51998	0.603078	NA
HFE2	0.915734	0.038319	0.051235	0.747917	0.45451	NA
MIR3173	0.929584	0.038318	0.052903	0.724298	0.468883	NA
ZNF441	169.0333	0.038306	0.064659	0.59244	0.553556	NA
LZTR1	252.5401	0.038306	0.057539	0.665734	0.505581	NA
C14orf93	11.17925	0.038293	0.083946	0.456166	0.648271	NA
HIST2H3D	0.632098	-0.03829	0.042855	-0.89348	0.371602	NA
ACOT12	1.009203	-0.03829	0.051418	-0.74465	0.456486	NA
USO1	293.1368	0.038285	0.047268	0.809961	0.417963	NA
PLA1A	4.615203	0.038283	0.0659	0.580924	0.561292	NA
NOX1	2.013525	0.038279	0.068478	0.558999	0.576163	NA
PALLD	147.5593	0.038274	0.053432	0.716301	0.473806	NA
OMP	2.350312	-0.03827	0.068769	-0.55647	0.577889	NA
NOTCH2	160.3107	-0.03826	0.077782	-0.49189	0.622798	NA
TREX1	41.14435	-0.03825	0.071076	-0.53812	0.590494	NA
EDARADD	0.829526	0.038243	0.048789	0.783846	0.433131	NA
CACNA1C-	4.375753	-0.03824	0.079413	-0.48155	0.630128	NA
AMDHD2	34.27507	-0.03824	0.073979	-0.51687	0.605246	NA
TRIM52	234.8418	-0.03823	0.059725	-0.64006	0.522131	NA
FAM122B	111.4531	-0.03822	0.051143	-0.74734	0.454858	NA

CD180	1.135167	-0.03822	0.050999	-0.74945	0.453583	NA
TMEM44	8.483652	-0.03822	0.084727	-0.4511	0.651916	NA
PVRL2	15.77324	-0.03821	0.084082	-0.45443	0.64952	NA
C5orf22	94.83586	0.038207	0.067529	0.565788	0.571538	NA
COMMD5	29.90359	0.038207	0.071153	0.536968	0.59129	NA
TXLNG	273.5664	-0.0382	0.0491	-0.77809	0.436516	NA
MIR4649	0.271429	-0.0382	0.028124	-1.35831	0.174367	NA
SCGB1A1	0.755006	-0.03819	0.047494	-0.80407	0.421356	NA
ATAD1	213.815	0.038188	0.056121	0.680467	0.496209	NA
FECH	80.28872	-0.03818	0.060024	-0.6361	0.524713	NA
RFESD	14.78404	-0.03817	0.082044	-0.46529	0.641722	NA
UTF1	0.271738	0.038174	0.02959	1.290121	0.197009	NA
KITLG	52.10527	0.038172	0.084484	0.451825	0.651395	NA
PTPRVP	1.500664	-0.03816	0.060571	-0.63001	0.528689	NA
RNF8	259.3429	0.038157	0.043079	0.885739	0.375758	NA
C2orf69	102.391	0.038153	0.059712	0.638945	0.522858	NA
MIR5187	0.131514	0.038152	0.021321	1.789364	0.073556	NA
GAL	0.511133	-0.03815	0.040506	-0.94188	0.346255	NA
TTK	1.008271	-0.03815	0.047559	-0.80217	0.422452	NA
TMOD4	2.032181	-0.03815	0.069537	-0.5486	0.583278	NA
FCER1A	0.349434	0.038147	0.035774	1.066338	0.286271	NA
RIMKLB	369.9158	-0.03814	0.045555	-0.83715	0.402511	0.709466
ZNF136	121.6463	0.038129	0.060468	0.630571	0.528321	NA
TTC21A	44.96739	0.038128	0.079773	0.477957	0.632681	NA
SLC25A10	11.37481	0.038118	0.083276	0.457725	0.64715	NA
STAG3L3	29.73392	0.038116	0.079779	0.477769	0.632814	NA
LOC14870	2.699877	0.038108	0.07436	0.512472	0.608321	NA
FUBP1	733.366	0.038105	0.059209	0.64357	0.519854	0.786028
TMEM200	3.208873	-0.0381	0.072688	-0.52419	0.600149	NA
WWOX	172.7479	-0.0381	0.048045	-0.79298	0.427789	NA
FLJ12334	4.185801	0.038097	0.080325	0.474291	0.635292	NA
PKD2L2	6.757767	-0.03809	0.083917	-0.45387	0.64992	NA
LY9	0.243669	-0.03808	0.025943	-1.46796	0.142116	NA
NT5DC3	80.65337	-0.03808	0.069185	-0.55043	0.582022	NA
TDRD1	1.630712	-0.03807	0.059799	-0.63661	0.524379	NA
ZKSCAN7	59.75177	-0.03806	0.065018	-0.58541	0.558272	NA
MIR4441	2.357308	-0.03805	0.065742	-0.57877	0.562743	NA
MAATS1	71.42027	-0.03804	0.078313	-0.48577	0.627132	NA
FLJ21408	0.806604	0.038042	0.046971	0.809892	0.418003	NA
SNORD115	149.4685	-0.03803	0.084401	-0.45063	0.652258	NA
MIR487A	1.202416	-0.03803	0.053658	-0.7088	0.478447	NA
ZSWIM7	35.91818	-0.03803	0.06642	-0.57253	0.566963	NA
C14orf101	85.30824	-0.03802	0.059648	-0.6374	0.523864	NA
DLGAP5	0.462396	0.038017	0.033975	1.118978	0.263149	NA
HDAC6	279.602	-0.03801	0.048282	-0.78727	0.431122	NA
WAC-AS1	207.7126	-0.03801	0.080997	-0.46927	0.638874	NA
ASH1L	2644.039	0.038008	0.026582	1.429825	0.152767	0.506203

LYSMD4	38.15862	-0.038	0.072119	-0.5269	0.598263	NA
TBCC	46.54277	0.037998	0.062197	0.610923	0.541251	NA
TPD52	354.3631	0.037989	0.047143	0.805823	0.420345	0.724567
RHBDL3	132.6227	-0.03797	0.082553	-0.45998	0.645532	NA
WDR96	32.12389	0.037972	0.075807	0.5009	0.616442	NA
KCNC1	1114.443	0.037969	0.047855	0.793427	0.427529	0.731937
TJAP1	95.05665	-0.03797	0.064782	-0.58605	0.557842	NA
MYO15A	61.69574	-0.03796	0.084689	-0.44826	0.653966	NA
C1orf162	25.27146	-0.03796	0.078965	-0.48074	0.630702	NA
NIF3L1	57.64484	-0.03796	0.062866	-0.60376	0.546005	NA
PTGER1	0.873148	0.037955	0.04758	0.797704	0.425042	NA
LOC10050	3.785333	0.037946	0.079979	0.474444	0.635183	NA
FAM131C	64.68027	-0.03794	0.061331	-0.61864	0.536155	NA
NBPF24	0.570457	0.037938	0.041223	0.920317	0.357407	NA
CD8A	13.32328	0.037923	0.084914	0.446601	0.655163	NA
MLKL	12.04062	-0.03792	0.079493	-0.47701	0.633352	NA
FAM43B	52.29073	0.037917	0.084428	0.449103	0.653357	NA
LINC00588	0.340429	0.03791	0.028321	1.338595	0.180703	NA
LOC10050	18.41202	0.037894	0.080712	0.469493	0.638718	NA
ZNF212	43.46994	0.037888	0.066672	0.568272	0.569851	NA
ASB8	167.9658	-0.03788	0.04118	-0.91996	0.357595	NA
C10orf128	1.743796	0.037882	0.063858	0.59323	0.553027	NA
CRX	0.233098	0.03788	0.027926	1.356463	0.174952	NA
RYK	96.66621	0.037879	0.056092	0.675295	0.499489	NA
CNDP1	39.16976	0.037866	0.084558	0.447813	0.654288	NA
BRDT	0.313757	-0.03786	0.026936	-1.4057	0.159813	NA
MIR4804	1.872886	0.037864	0.066638	0.568199	0.5699	NA
MIR628	6.285259	-0.03786	0.079683	-0.47517	0.634665	NA
C2orf16	10.64929	0.037861	0.08406	0.450404	0.652419	NA
FAM133B	7.277846	-0.03786	0.084772	-0.44656	0.65519	NA
C17orf61-	1.398873	-0.03785	0.058395	-0.64824	0.51683	NA
PPP3CC	130.0231	-0.03785	0.050851	-0.74439	0.456643	NA
ATP5A1	452.7897	-0.03785	0.066901	-0.56574	0.571571	0.81719
PDPN	28.295	-0.03784	0.084904	-0.44574	0.655786	NA
SNORD115	13.99446	-0.03784	0.084212	-0.44932	0.653201	NA
WDR17	364.7746	-0.03784	0.043987	-0.86016	0.3897	0.701813
ARMC6	45.67194	-0.03781	0.062425	-0.60571	0.544708	NA
GBP2	14.22969	-0.03781	0.084447	-0.44768	0.654381	NA
SNORD116	306.4246	-0.0378	0.083732	-0.45144	0.65167	NA
MIR4323	0.55904	0.037795	0.037796	0.999969	0.317326	NA
LOC10013	51.21923	0.037795	0.069625	0.542832	0.587245	NA
LINC-ROR	1.466416	0.037792	0.055376	0.68247	0.494942	NA
TAL2	0.165617	-0.03779	0.023965	-1.57691	0.114816	NA
ZBTB3	20.52659	0.037771	0.076167	0.495894	0.619969	NA
FAM32A	98.71668	0.037768	0.05482	0.688943	0.490859	NA
BCLAF1	1047.533	-0.03777	0.046742	-0.808	0.419089	0.724567
ASAP1-IT1	6.977682	-0.03776	0.08421	-0.44841	0.653854	NA

CCNE1	21.63554	0.037759	0.076867	0.491227	0.623266	NA
VWA3B	2.089443	0.037758	0.060582	0.623252	0.533119	NA
AVEN	48.89291	-0.03775	0.071787	-0.52588	0.598974	NA
ABCG4	75.23626	0.037741	0.060964	0.619065	0.535874	NA
ISYNA1	19.28828	-0.03774	0.08466	-0.44575	0.65578	NA
CCDC102B	18.03717	-0.03772	0.082803	-0.45558	0.648693	NA
LOC38879	22.50074	-0.03772	0.080574	-0.46816	0.639671	NA
GJC3	2.592737	0.03772	0.072065	0.523419	0.600683	NA
SIGLEC16	7.927998	0.0377	0.082127	0.459044	0.646203	NA
LOC39132	4.586015	-0.0377	0.068343	-0.55162	0.581211	NA
PHACTR1	109.1206	-0.03769	0.074622	-0.50504	0.613532	NA
NRXN1	1907.644	0.037687	0.034117	1.10463	0.26932	0.610306
FAM213B	24.78485	0.037686	0.076374	0.493438	0.621703	NA
DHTKD1	163.1473	0.037685	0.043981	0.856833	0.391537	NA
KIF20B	153.6832	-0.03768	0.068607	-0.54922	0.582855	NA
SLC9A1	112.7993	0.037669	0.06089	0.618638	0.536155	NA
THAP7-AS	28.68701	0.037662	0.079779	0.472085	0.636866	NA
WASH2P	49.05852	-0.03766	0.070566	-0.5337	0.593552	NA
FASTK	70.72961	-0.03765	0.066252	-0.56828	0.569848	NA
SELT	148.8993	0.037647	0.07505	0.501631	0.615927	NA
RCAN3AS	0.682159	0.037647	0.044763	0.841023	0.400335	NA
FAM172A	229.8909	-0.03765	0.042469	-0.88644	0.375382	NA
ZSCAN26	254.2955	0.037644	0.044093	0.853743	0.393247	NA
PLCB3	132.8158	0.037643	0.062311	0.604113	0.545769	NA
SEMA6D	105.4898	-0.03764	0.077	-0.48881	0.624978	NA
LOC10012	3.452344	-0.03764	0.074759	-0.50345	0.614647	NA
CCRL2	0.756624	0.037636	0.049078	0.766873	0.443157	NA
TBC1D12	179.776	0.037635	0.043881	0.857666	0.391077	NA
KAT5	93.72766	0.037634	0.04956	0.759361	0.447636	NA
INHBB	6.867079	0.03763	0.080837	0.465503	0.641571	NA
UBE2S	22.31896	-0.03763	0.083618	-0.44998	0.652725	NA
SUPT6H	866.1981	0.037623	0.032812	1.14664	0.25153	0.592704
C8orf47	1.558725	0.037621	0.054919	0.685022	0.49333	NA
SCHIP1	21.2246	0.037611	0.075713	0.496759	0.619359	NA
COPG2	73.96218	0.037599	0.06582	0.571241	0.567836	NA
PNPLA1	1.006249	0.037595	0.052684	0.713602	0.475473	NA
ATP1B3	190.8921	0.037594	0.062306	0.603365	0.546266	NA
GUCA1B	16.84022	-0.03759	0.08479	-0.44337	0.6575	NA
MIR330	1.039277	0.037574	0.055702	0.674555	0.499958	NA
PDCL	91.47605	0.037567	0.060937	0.616478	0.537579	NA
CDC25A	3.558912	0.037565	0.076431	0.491486	0.623083	NA
PAR4	7.034819	-0.03756	0.083979	-0.44724	0.654704	NA
FAM218A	30.43616	0.037554	0.083971	0.447226	0.654712	NA
LINC00299	9.092415	0.037549	0.083569	0.449323	0.653198	NA
SPDL1	90.45438	0.037539	0.059794	0.627811	0.530128	NA
OR56B4	0.446172	-0.03753	0.039167	-0.95833	0.337895	NA
SNORD114	9.372831	-0.03753	0.082502	-0.4549	0.649179	NA

DDX20	96.04523	0.037526	0.056376	0.665631	0.505647	NA
C12orf68	49.36604	0.037525	0.082918	0.452557	0.650868	NA
ENDOD1	366.7083	0.037524	0.048456	0.774383	0.438704	0.74018
ADCYAP1R	410.4843	0.037515	0.052178	0.718991	0.472147	0.759174
SNORA74B	5.398922	0.037514	0.081764	0.458814	0.646368	NA
CA5B	37.56167	-0.03751	0.070884	-0.52919	0.596671	NA
BCL2L12	1.607915	-0.03751	0.064988	-0.57715	0.56384	NA
HDDC3	11.12167	0.037504	0.084694	0.442813	0.657901	NA
DSCR9	10.28668	-0.0375	0.083762	-0.44767	0.654391	NA
DDI2	76.07024	0.037496	0.057984	0.646653	0.517856	NA
LOC59510	22.04672	0.037489	0.084541	0.443441	0.657447	NA
SIL1	45.98594	-0.03748	0.072774	-0.51508	0.606497	NA
G6PC2	0.437849	0.037483	0.035276	1.062562	0.287981	NA
GUSBP2	2.757435	0.037477	0.07487	0.500554	0.616685	NA
NFIA	1751.354	0.037477	0.034872	1.074699	0.282509	0.617151
ACSM4	1.013565	-0.03748	0.049275	-0.76054	0.446933	NA
FBXO18	191.0536	0.037472	0.037053	1.011311	0.311868	NA
ZNF616	70.2149	0.037466	0.060233	0.622013	0.533933	NA
PPP2R3A	171.6443	-0.03746	0.066704	-0.56166	0.574349	NA
DKFZP434	42.64419	-0.03746	0.078244	-0.47878	0.632095	NA
PHB	56.49677	-0.03746	0.06774	-0.55295	0.580296	NA
INTS10	249.2464	-0.03744	0.052174	-0.71767	0.472958	NA
LINC00423	0.196005	0.037438	0.024543	1.525407	0.127158	NA
SNRNP48	189.2349	0.037434	0.065417	0.572238	0.567161	NA
POLDIP2	89.78219	-0.03743	0.054318	-0.68914	0.490734	NA
PDE7A	139.1746	0.037423	0.057993	0.645299	0.518734	NA
SNORD113	7.957309	-0.03742	0.083695	-0.44707	0.654826	NA
SLC7A6	243.1017	-0.03741	0.044006	-0.85017	0.395233	NA
C2orf78	0.346556	-0.03741	0.033369	-1.12114	0.262227	NA
ENOX2	25.03134	-0.0374	0.081464	-0.45912	0.646151	NA
INTS5	24.72338	0.037398	0.075486	0.495434	0.620294	NA
HNRNPA2	2888.044	0.037395	0.036399	1.027365	0.304249	0.635137
NFE2	0.696015	0.037391	0.044431	0.841562	0.400033	NA
NIPSNAP3	23.05445	-0.03739	0.080593	-0.46394	0.642693	NA
BLID	3.173062	-0.03739	0.075592	-0.49459	0.62089	NA
TAF6	131.3771	0.037354	0.055574	0.672145	0.501491	NA
MIR4311	5.880316	-0.03735	0.083604	-0.44673	0.655071	NA
MLLT1	428.6059	0.037342	0.049499	0.754405	0.450606	0.749356
DPCD	18.86634	0.037328	0.077407	0.482225	0.629646	NA
LRFN5	41.08183	-0.03732	0.083703	-0.4459	0.655671	NA
PCDHB14	57.0555	-0.03732	0.071988	-0.51843	0.604156	NA
FAM120A	265.6511	0.037307	0.040349	0.924591	0.355179	NA
PRICKLE2-	1.871619	0.037304	0.066205	0.563465	0.573118	NA
KCNA7	0.203833	-0.0373	0.026542	-1.40543	0.159893	NA
TCAM1P	2.779783	0.0373	0.072634	0.513529	0.607581	NA
SPATA4	2.555844	0.037295	0.069264	0.53844	0.590274	NA
KLHL2	104.7283	0.03729	0.056117	0.6645	0.50637	NA

ZNF429	85.61189	0.037283	0.078158	0.477024	0.633345	NA
NANOS2	0.327494	0.037283	0.032499	1.147187	0.251304	NA
LRRN2	49.83128	0.037283	0.081531	0.457279	0.647471	NA
MAT1A	0.114348	0.037273	0.02111	1.765649	0.077455	NA
CAPZA1	141.2922	-0.03726	0.044885	-0.83017	0.406443	NA
PREB	80.97617	-0.03725	0.055413	-0.67232	0.501383	NA
SLC25A34	35.06348	0.037254	0.083002	0.448829	0.653555	NA
SGCB	299.3145	0.037237	0.046059	0.808452	0.41883	NA
GRIA4	1439.419	0.037233	0.044487	0.836928	0.402633	0.709466
PLCL2	478.649	0.037232	0.046185	0.806152	0.420155	0.724567
THSD7B	5.354997	0.037231	0.080784	0.460874	0.644889	NA
MTMR1	142.7386	-0.03723	0.04465	-0.83376	0.404415	NA
SLC9A3R2	253.0397	0.037227	0.066459	0.560138	0.575385	NA
SYCE1	84.46998	-0.03723	0.084332	-0.44141	0.658914	NA
CDH15	284.5599	-0.03722	0.061706	-0.60317	0.546392	NA
SSTR3	2.466348	0.03722	0.063695	0.584344	0.558989	NA
PABPC4L	2.451631	0.037216	0.071818	0.518201	0.604318	NA
EDEM3	353.6885	0.037216	0.038346	0.970539	0.331778	0.660832
GNAT1	0.254783	0.037212	0.027096	1.373375	0.169636	NA
PDF	6.116975	-0.0372	0.083207	-0.44713	0.654785	NA
BCL2L1	176.0714	-0.0372	0.058331	-0.63775	0.523637	NA
MIR3126	1.142073	0.037195	0.052896	0.703161	0.481955	NA
LONRF1	117.1067	0.037186	0.056011	0.663903	0.506752	NA
SPG20	343.622	0.037184	0.049742	0.747535	0.45474	0.750995
ATPBD4	41.1457	-0.03717	0.08487	-0.438	0.661388	NA
APAF1	180.9023	-0.03717	0.051438	-0.72263	0.469908	NA
MPC2	106.8621	0.037169	0.062824	0.591627	0.5541	NA
HM13	118.0927	-0.03717	0.049785	-0.74652	0.455355	NA
GAS6-AS1	5.080699	0.03716	0.081112	0.458135	0.646856	NA
EVA1B	13.64827	-0.03716	0.084234	-0.44115	0.659102	NA
SCUBE2	150.7028	0.037154	0.06069	0.612193	0.54041	NA
ZNF137P	29.41359	0.03715	0.082127	0.452356	0.651013	NA
TMEM9B	85.50022	0.03715	0.060703	0.612003	0.540536	NA
SERAC1	67.93216	0.037147	0.061192	0.60706	0.543811	NA
PCCA	243.5394	-0.03714	0.043826	-0.84752	0.396703	NA
DHX33	95.43465	0.037142	0.059016	0.629352	0.529119	NA
DLEU2	102.816	0.037141	0.074356	0.499498	0.617429	NA
COL12A1	15.65377	0.037135	0.083734	0.443492	0.65741	NA
L3MBTL1	116.48	-0.03712	0.065716	-0.56481	0.572204	NA
KIAA1033	300.485	-0.0371	0.040386	-0.91872	0.358241	NA
TNK2	506.3119	-0.03709	0.055094	-0.67322	0.500804	0.778081
GPC4	15.69976	-0.03709	0.084929	-0.43673	0.662308	NA
SPEM1	0.357339	-0.03709	0.034381	-1.07865	0.280742	NA
CHMP3	26.72008	0.037084	0.080982	0.45793	0.647002	NA
GPBP1L1	351.8713	0.037069	0.053019	0.699158	0.484453	0.764934
MGP	8.697044	-0.03706	0.072272	-0.51282	0.608077	NA
MNF1	70.17294	-0.03706	0.074513	-0.49739	0.618916	NA

LACE1	25.32187	-0.03706	0.07342	-0.50479	0.613709	NA
RAN	162.4524	0.037057	0.058498	0.633469	0.526427	NA
OR6B2	0.174724	0.037056	0.024501	1.512415	0.130428	NA
HIGD1C	5.105158	0.037054	0.080008	0.463131	0.643271	NA
EEFSEC	32.93766	-0.03705	0.071461	-0.5185	0.604112	NA
CMYA5	233.0003	-0.03705	0.061974	-0.59784	0.549949	NA
PEX12	45.34707	-0.03705	0.066645	-0.55591	0.578271	NA
NDUFB5	110.4167	-0.03704	0.082176	-0.45076	0.652159	NA
GDF5	0.207751	-0.03703	0.026626	-1.39089	0.164259	NA
GPKOW	131.5214	-0.03703	0.042079	-0.87995	0.378886	NA
APOOL	57.63402	0.037022	0.070007	0.528836	0.596919	NA
IFNA8	3.229637	-0.03702	0.066921	-0.55317	0.580149	NA
SOD3	5.178096	-0.03702	0.079147	-0.46771	0.639995	NA
RPS6KA3	299.863	0.03701	0.043481	0.851177	0.394671	NA
GDAP2	304.6325	0.037008	0.043859	0.843789	0.398787	NA
FUCA1	28.51073	-0.03699	0.069039	-0.53578	0.592111	NA
FNDC5	183.4821	0.036989	0.070375	0.525598	0.599167	NA
TDRD10	2.259968	0.036985	0.070754	0.522724	0.601166	NA
NAPA-AS1	15.90305	-0.03698	0.082159	-0.45011	0.652629	NA
TPPP2	0.333165	0.036974	0.03316	1.115028	0.264838	NA
EIF3B	272.9518	0.03697	0.039222	0.942572	0.3459	NA
TMEM45A	0.506001	-0.03696	0.040246	-0.91847	0.358374	NA
ZFAND5	513.3483	0.036961	0.046776	0.790172	0.429427	0.73339
TRAPPC6B	356.1996	-0.03696	0.040314	-0.91677	0.359263	0.683233
STK11IP	74.46606	0.036955	0.069261	0.533568	0.59364	NA
GLT8D2	3.158008	0.036952	0.07775	0.47527	0.634595	NA
WBSCR22	58.99142	0.03695	0.063435	0.582481	0.560243	NA
MPRIP	1943.926	0.036948	0.037331	0.989733	0.322304	0.651803
TMEM53	4.525847	0.036946	0.082915	0.445592	0.655892	NA
CD52	0.435083	0.036943	0.037	0.998474	0.31805	NA
ENTHD2	23.51959	-0.03693	0.075219	-0.49094	0.623466	NA
FAM81A	6.199208	0.036926	0.083628	0.44155	0.658815	NA
PLA2G12A	249.6783	0.036926	0.041389	0.892166	0.372304	NA
GPA33	0.531208	-0.03692	0.041282	-0.89427	0.371179	NA
NR4A1	29.1975	0.036905	0.066328	0.556396	0.57794	NA
PTDSS1	109.152	0.036903	0.058344	0.632512	0.527053	NA
RPIA	25.8441	0.036883	0.074147	0.497431	0.618885	NA
PRG1	1.044009	0.036881	0.053525	0.689043	0.490796	NA
BIRC2	192.4188	0.03688	0.041171	0.895793	0.370363	NA
TSHZ1	472.1369	0.036877	0.056991	0.647073	0.517585	0.785288
TMEM194	64.92874	-0.03687	0.066941	-0.55085	0.581733	NA
RASIP1	35.74493	0.036874	0.069091	0.533702	0.593548	NA
ADCY5	111.9323	0.036873	0.072052	0.511756	0.608822	NA
MIR597	0.573903	0.036872	0.043268	0.85218	0.394114	NA
HYAL2	23.22419	0.036872	0.0838	0.44	0.659937	NA
PLAA	106.8774	0.036865	0.051705	0.712975	0.475861	NA
MIR595	0.554472	-0.03686	0.042718	-0.86295	0.388164	NA

TAPBP	167.0106	0.036862	0.043784	0.841889	0.39985	NA
VPS52	170.1809	-0.03686	0.048728	-0.7564	0.449412	NA
PNP	33.36725	-0.03686	0.081717	-0.45103	0.651966	NA
IL34	8.722666	0.036856	0.084862	0.434309	0.664064	NA
WDR45	65.9127	-0.03685	0.0614	-0.60023	0.54835	NA
ZNF283	114.5483	-0.03685	0.054138	-0.68076	0.496026	NA
C16orf96	8.390189	0.036853	0.083772	0.439917	0.659998	NA
LINC00499	1.019385	0.036846	0.033103	1.113063	0.265681	NA
CCDC36	14.24944	0.036844	0.084856	0.434199	0.664144	NA
SLC9C2	2.536216	0.036842	0.071788	0.513214	0.607802	NA
MTMR2	152.7829	0.036841	0.047469	0.776109	0.437685	NA
GLP2R	0.187981	0.036836	0.02469	1.49196	0.13571	NA
MIR496	0.674175	-0.03683	0.046039	-0.80007	0.423672	NA
ZBED3-AS1	6.230392	0.036825	0.083548	0.440757	0.659389	NA
SAYSD1	29.61626	-0.03682	0.075211	-0.48955	0.624454	NA
H3F3B	706.3902	-0.03681	0.069903	-0.52655	0.598509	0.829672
WTAP	272.7698	0.036801	0.038559	0.954409	0.339877	NA
LOC15506	81.89893	-0.03679	0.075077	-0.49002	0.624118	NA
LOC28441	23.68174	0.036785	0.084938	0.433076	0.66496	NA
LINGO3	35.66383	-0.03678	0.083787	-0.43902	0.660648	NA
TIMM8A	11.6617	0.036781	0.084009	0.437825	0.661513	NA
RPL36AL	200.5304	-0.03678	0.067091	-0.54822	0.58354	NA
SEC16A	549.7849	-0.03678	0.037121	-0.9908	0.321786	0.651336
VARS	92.15639	0.036778	0.054664	0.672803	0.501073	NA
ZFP14	225.0732	0.036765	0.048492	0.758162	0.448354	NA
GLTSCR1L	654.8088	0.03676	0.034586	1.062871	0.28784	0.621249
NDUFB3	30.42914	0.036757	0.083059	0.442533	0.658103	NA
FAM182A	4.79268	-0.03675	0.067293	-0.54618	0.584942	NA
LOC28618	16.17199	0.036754	0.084834	0.433245	0.664837	NA
PPP6R2	428.7889	-0.03675	0.050376	-0.72956	0.465659	0.755844
C2orf44	42.12922	0.036746	0.066339	0.553903	0.579645	NA
EEF1E1	11.43763	0.036743	0.084456	0.435061	0.663518	NA
GMEB1	98.87235	0.036743	0.0512	0.717641	0.472978	NA
TMEM52B	0.313284	0.036736	0.031036	1.183667	0.236545	NA
GPR1	0.490416	-0.03673	0.038872	-0.94488	0.344722	NA
AKAP3	7.592294	-0.03673	0.084615	-0.43405	0.664253	NA
GNG12	36.37246	-0.03672	0.080321	-0.45718	0.647542	NA
NEUROG2	4.754724	0.036709	0.073941	0.496465	0.619566	NA
SLC27A1	194.2707	0.036708	0.062524	0.587096	0.557139	NA
IFNE	17.54382	0.036699	0.084793	0.432806	0.665155	NA
HINT3	162.4723	0.036685	0.054972	0.667339	0.504556	NA
ODF2L	235.7484	-0.03668	0.050192	-0.73082	0.464891	NA
RASSF5	55.51512	-0.03668	0.06352	-0.57743	0.563649	NA
PAG1	836.5508	0.036671	0.037749	0.971443	0.331328	0.660832
STON1-GT	1.619848	-0.03667	0.061034	-0.60077	0.547996	NA
TTI1	98.23864	0.036666	0.050504	0.726	0.467839	NA
ATOH7	1.65154	-0.03666	0.063405	-0.57823	0.563107	NA

AKAP7	93.33453	0.036658	0.058432	0.627363	0.530421	NA
ABCD3	189.6611	0.036657	0.052125	0.703252	0.481899	NA
KIAA0391	109.0384	-0.03665	0.050681	-0.72324	0.469534	NA
GPR107	367.6033	-0.03664	0.040499	-0.90472	0.365615	0.688858
ZNF585A	80.31633	0.036636	0.058166	0.629859	0.528787	NA
CLCA3P	2.687038	-0.03663	0.071991	-0.50875	0.61093	NA
TTLL10	0.199612	-0.03662	0.025328	-1.44595	0.148192	NA
MAML1	648.6383	-0.03662	0.063788	-0.5741	0.565901	0.814521
PDK1	85.64991	-0.03661	0.057973	-0.63157	0.527671	NA
C3orf35	47.3061	-0.03661	0.066764	-0.54839	0.583421	NA
ZNF611	118.17	0.036611	0.052245	0.700746	0.483461	NA
PANK3	559.2461	0.036607	0.042739	0.85651	0.391716	0.703039
NKX3-1	1.93448	-0.0366	0.067767	-0.54014	0.589101	NA
C9orf131	73.39198	-0.0366	0.076437	-0.47884	0.632053	NA
FAM21A	34.80531	0.036597	0.074102	0.493872	0.621396	NA
TIAL1	360.4014	0.036595	0.050283	0.727791	0.466742	0.755844
POLA1	151.5085	0.036595	0.045878	0.797661	0.425067	NA
ETNK2	4.592905	-0.03659	0.080688	-0.45353	0.650168	NA
MANBA	150.0048	-0.03659	0.047408	-0.77191	0.44017	NA
GTPBP5	82.61927	0.036578	0.052809	0.692648	0.488531	NA
RNMT	225.8891	0.036565	0.041972	0.87118	0.383656	NA
DPF2	117.0089	0.03656	0.058938	0.620322	0.535046	NA
SCCPDH	103.4736	-0.03656	0.058958	-0.62011	0.535188	NA
OCRL	172.1706	0.036557	0.052365	0.698121	0.485101	NA
PPARGC1B	322.1507	-0.03654	0.048339	-0.75583	0.449751	NA
ETAA1	113.4072	-0.03653	0.066454	-0.5497	0.582528	NA
APOC1P1	0.328077	-0.03651	0.03103	-1.17671	0.239312	NA
PANK4	114.2908	-0.03651	0.053158	-0.68676	0.492235	NA
EDN1	23.01278	-0.0365	0.079564	-0.45874	0.646421	NA
BIRC3	3.795594	0.036498	0.069685	0.52376	0.600446	NA
DLST	201.5588	-0.03649	0.03963	-0.92078	0.357164	NA
TXNRD2	80.91016	-0.03649	0.065449	-0.55746	0.577211	NA
ZDHHC17	436.4838	0.036473	0.036581	0.997033	0.318748	0.648666
STAC2	64.32451	-0.03647	0.078124	-0.46676	0.640668	NA
MEGF9	411.0367	-0.03646	0.058154	-0.62696	0.530683	0.793922
LINC00617	28.52751	0.036457	0.082515	0.44183	0.658612	NA
BAX	14.13317	-0.03645	0.083173	-0.43822	0.661228	NA
CCDC47	470.1112	0.036444	0.057375	0.635189	0.525305	0.789308
RNPEPL1	43.19961	-0.03644	0.069548	-0.5239	0.600347	NA
ZSCAN29	151.7693	0.03643	0.048035	0.758404	0.448209	NA
CHRNA2	26.56325	-0.03643	0.082685	-0.44058	0.659517	NA
STXBP5-AS	14.54321	-0.03642	0.082657	-0.44067	0.659454	NA
MYL12A	57.4976	0.036424	0.076478	0.476267	0.633884	NA
SNORD18C	0.349858	0.036422	0.034157	1.066337	0.286271	NA
ZNF582-AS	19.2801	0.036413	0.08045	0.452619	0.650823	NA
ALAS1	208.7907	0.036406	0.056682	0.642292	0.520683	NA
SH2D1B	0.654374	0.036397	0.042754	0.851318	0.394593	NA

CTSS	13.12706	-0.03639	0.08345	-0.43602	0.662826	NA
KIAA1468	382.51	0.036384	0.046491	0.782606	0.433859	0.736097
C9orf173	0.701884	-0.03638	0.046512	-0.78213	0.434138	NA
FSD1	80.99254	-0.03637	0.075059	-0.48459	0.627968	NA
KIDINS220	1845.472	0.036365	0.027019	1.345901	0.178335	0.530729
NUDT5	42.14435	0.036359	0.063464	0.5729	0.566713	NA
SERINC1	1135.088	0.036351	0.064302	0.565325	0.571853	0.81719
SLC9B2	134.4216	-0.03634	0.044048	-0.82507	0.40933	NA
SRRT	496.1813	-0.03633	0.062972	-0.57699	0.563947	0.813231
MIR558	0.564103	-0.03633	0.04214	-0.86215	0.388604	NA
SH2D6	2.619548	-0.03633	0.067622	-0.53721	0.591125	NA
SLC22A18	0.512202	-0.03632	0.041395	-0.87748	0.380224	NA
DND1	17.34944	0.036319	0.083889	0.432939	0.665059	NA
FBXL6	40.04985	-0.03632	0.074268	-0.48901	0.624833	NA
DIAPH2	20.24825	0.036305	0.083069	0.43705	0.662075	NA
AKT1S1	80.27396	-0.0363	0.061834	-0.58712	0.557121	NA
SART1	270.5743	0.036303	0.054163	0.670253	0.502696	NA
HNRNPM	960.8564	0.03629	0.043464	0.834936	0.403754	0.709466
NANP	36.13054	0.036288	0.07687	0.472069	0.636878	NA
MPHOSPH	100.1589	-0.03629	0.050803	-0.71424	0.475076	NA
SLC16A1	102.012	0.036278	0.078851	0.460087	0.645454	NA
ANKRD49	46.06301	-0.03628	0.064305	-0.56412	0.572671	NA
PREPL	1135.878	0.036275	0.063672	0.569712	0.568873	0.815505
HELQ	85.77656	0.036271	0.054333	0.667561	0.504414	NA
ADAMTSL2	49.17881	0.03627	0.083128	0.436313	0.66261	NA
YRDC	52.50404	0.036265	0.063612	0.570099	0.56861	NA
PPIB	59.10208	-0.03626	0.070894	-0.51145	0.609034	NA
MAEA	156.2281	0.036251	0.048724	0.744014	0.456868	NA
CDH7	167.6881	0.036241	0.057634	0.628805	0.529476	NA
FANCL	22.34184	0.036239	0.082408	0.43975	0.660118	NA
SURF2	34.10818	-0.03624	0.080865	-0.4481	0.654079	NA
ATP7A	33.11196	-0.03622	0.071432	-0.50711	0.612074	NA
RARG	60.26509	0.036224	0.06128	0.591116	0.554443	NA
PCIF1	165.448	-0.03621	0.057295	-0.63195	0.527419	NA
TES	3.945351	-0.0362	0.073347	-0.49356	0.621615	NA
LOC10013	5.389692	0.036195	0.079994	0.452477	0.650925	NA
TMEM245	443.3459	0.036195	0.056553	0.640015	0.522163	0.787214
DLGAP4	197.1815	-0.03619	0.055012	-0.65794	0.510579	NA
AMELX	0.375395	-0.03618	0.03303	-1.09552	0.27329	NA
B4GALNT1	236.1389	0.03618	0.046318	0.781127	0.434728	NA
PRSS8	10.30528	-0.03618	0.084155	-0.42992	0.667254	NA
HIST1H2B	7.037691	0.036177	0.082036	0.440993	0.659218	NA
TUBG1	60.20369	-0.03617	0.075018	-0.4822	0.629663	NA
MAPKBP1	1169.637	0.036168	0.070052	0.516301	0.605644	0.831231
SLC35B3	50.26836	0.036157	0.065912	0.548565	0.583304	NA
PA2G4P4	17.52216	0.036151	0.083469	0.433103	0.66494	NA
INO80B	1.399284	0.036148	0.051722	0.698899	0.484615	NA

FIGNL2	13.1619	-0.03614	0.084307	-0.42872	0.668128	NA
VDAC2	112.2993	0.036099	0.060932	0.592445	0.553553	NA
SNORD114	1.231109	0.03609	0.057007	0.633072	0.526687	NA
RRN3P1	39.1817	-0.03609	0.078358	-0.46052	0.645142	NA
LOC72855	3.754041	-0.03608	0.077778	-0.46391	0.642711	NA
KLHDC4	123.2596	-0.03607	0.064496	-0.55926	0.575987	NA
GDE1	85.20656	-0.03606	0.065574	-0.54995	0.582354	NA
C9orf117	8.017944	-0.03606	0.084153	-0.42851	0.668283	NA
GNPTAB	330.9038	0.036055	0.03338	1.080139	0.28008	NA
CD101	1.528002	-0.03605	0.063802	-0.56505	0.57204	NA
KIF13A	626.6365	0.03605	0.037256	0.96764	0.333224	0.660832
F2RL1	0.245843	-0.03604	0.027175	-1.32641	0.184702	NA
C1orf116	0.596855	-0.03604	0.041191	-0.87492	0.381615	NA
CHMP7	144.7559	0.036036	0.042867	0.840649	0.400544	NA
RNF121	84.88366	0.036031	0.057526	0.626354	0.531083	NA
FAM46A	105.3234	0.036029	0.080243	0.448998	0.653433	NA
HBE1	0.348975	-0.03602	0.034388	-1.04754	0.29485	NA
PRKAA2	321.8675	0.036013	0.042461	0.848154	0.396352	NA
LY86-AS1	0.490937	-0.03601	0.032215	-1.11786	0.263629	NA
ZNF641	111.2161	0.03601	0.06369	0.565399	0.571803	NA
FGF14	501.5289	-0.03601	0.037564	-0.95857	0.337774	0.66305
EMR1	0.286689	-0.036	0.031113	-1.1572	0.247192	NA
PPYR1	8.76158	0.036	0.083696	0.430132	0.6671	NA
SMARCC1	479.2731	-0.036	0.052008	-0.69219	0.488817	0.766208
KIF1A	3003.704	0.035995	0.041132	0.875123	0.381507	0.70023
SORD	20.44523	-0.03599	0.082706	-0.43511	0.663484	NA
LOC10065	10.89398	0.035983	0.082359	0.436907	0.662179	NA
WNK3	120.2838	0.03598	0.061085	0.589019	0.555848	NA
TLL2	4.459015	0.035978	0.079419	0.453016	0.650537	NA
MEIOB	4.838586	0.035973	0.082182	0.43772	0.66159	NA
C3orf45	3.647633	0.035969	0.077713	0.462847	0.643474	NA
SYNJ2BP	365.091	0.035964	0.043034	0.835711	0.403318	0.709466
DLX6	0.52628	0.035964	0.040143	0.895898	0.370307	NA
LOC10012	37.73711	-0.03596	0.080066	-0.44915	0.65332	NA
REST	50.75432	0.035961	0.070599	0.509364	0.610497	NA
CD300LG	0.767338	-0.03596	0.047015	-0.76484	0.444365	NA
CCNDBP1	167.2818	-0.03596	0.059724	-0.60205	0.547144	NA
FAM181A	2.583495	-0.03595	0.068856	-0.52216	0.601557	NA
NOL9	160.0321	0.035949	0.043598	0.824546	0.40963	NA
TCF24	0.260831	-0.03595	0.030383	-1.18314	0.236755	NA
TSC22D1	724.7791	-0.03594	0.041545	-0.86519	0.386936	0.700314
MRPL49	44.7963	-0.03594	0.066842	-0.53772	0.590768	NA
VWA5B1	0.477074	-0.03593	0.035239	-1.01965	0.307894	NA
PTGS2	3.733978	0.035928	0.065406	0.549308	0.582794	NA
OR2AT4	0.342634	-0.03592	0.034655	-1.03649	0.299972	NA
C12orf43	51.76433	0.03592	0.063206	0.568297	0.569833	NA
PCBP2	1078.796	0.03592	0.050517	0.71104	0.477059	0.759174

WSB2	157.5867	0.035918	0.058126	0.61793	0.536621	NA
ZNF407	410.3583	0.035917	0.051492	0.697513	0.485482	0.764934
SHF	354.6595	0.035913	0.057475	0.624841	0.532075	0.794571
CCL25	0.174274	-0.03591	0.020917	-1.71691	0.085995	NA
ATP6V1G2	440.4272	0.035907	0.07596	0.472708	0.636421	0.846919
RNF222	3.242833	0.035906	0.073819	0.486401	0.626683	NA
SLC39A8	60.11525	-0.03591	0.079559	-0.4513	0.65177	NA
LOC65378	2.400614	0.035899	0.06589	0.544836	0.585866	NA
CCDC142	78.31332	-0.0359	0.060443	-0.59389	0.552583	NA
DDX54	62.8522	0.035895	0.069162	0.518997	0.603763	NA
CDON	732.783	0.035891	0.054443	0.659244	0.509739	0.781767
MFSD1	60.51729	0.035886	0.058042	0.618277	0.536392	NA
EXOSC8	33.60028	-0.03589	0.07436	-0.48259	0.629388	NA
DHRS4-AS	9.288287	0.035878	0.084925	0.422462	0.672688	NA
PNKD	66.67715	-0.03587	0.065305	-0.54934	0.582773	NA
ANAPC1P1	3.886648	-0.03587	0.038467	-0.93252	0.351066	NA
GPR19	53.30331	-0.03587	0.05817	-0.61658	0.537513	NA
CLEC17A	0.553475	-0.03587	0.041202	-0.87048	0.384036	NA
CHRNA6	17.03754	0.035864	0.071532	0.501368	0.616112	NA
WDR24	90.13377	-0.03586	0.065885	-0.54429	0.586241	NA
LOC10012	1.417721	0.035859	0.058283	0.615264	0.538381	NA
POU5F2	17.79307	-0.03586	0.079437	-0.45139	0.651709	NA
MCCD1	1.619881	-0.03586	0.060534	-0.59234	0.553625	NA
MGAT1	115.8629	-0.03585	0.062544	-0.57326	0.56647	NA
PLS3	42.6484	-0.03585	0.07783	-0.46067	0.645038	NA
ZNF80	0.354162	-0.03585	0.034402	-1.04218	0.297329	NA
FLJ46906	3.024793	-0.03585	0.072719	-0.49296	0.622042	NA
DAAM1	431.3975	0.035841	0.043993	0.814713	0.415237	0.720147
SNORD2	12.38487	-0.03584	0.084627	-0.42347	0.671954	NA
JAG1	48.41174	-0.03583	0.078866	-0.45436	0.649567	NA
RFTN2	164.5078	-0.03583	0.076005	-0.47142	0.637344	NA
RPUSD3	55.50102	-0.03582	0.061267	-0.58465	0.558782	NA
SRGAP3	1512.281	0.035807	0.050827	0.704482	0.481133	0.763141
FAM83H	2.215719	0.035799	0.062841	0.569676	0.568897	NA
SUPT16H	794.4418	0.035794	0.04307	0.831065	0.405937	0.710565
PARM1	17.14555	0.035794	0.084684	0.422674	0.672533	NA
CMC2	31.95857	-0.03579	0.080811	-0.44285	0.657877	NA
ZNF302	232.0749	-0.03579	0.046631	-0.76743	0.442829	NA
ISY1	28.95827	-0.03578	0.074566	-0.47982	0.631355	NA
BRAF	391.5159	0.035777	0.040264	0.888559	0.37424	0.695376
DGAT2	0.800004	-0.03578	0.048171	-0.7427	0.457665	NA
FCN1	0.831975	-0.03577	0.046171	-0.77481	0.43845	NA
PMS2P3	50.84972	-0.03577	0.069474	-0.51492	0.606606	NA
LOC64699	1.639647	0.035771	0.054545	0.655797	0.511955	NA
RBM26	777.178	0.035762	0.031928	1.12006	0.262688	0.60594
RC3H1	880.1611	-0.03575	0.040641	-0.87976	0.378989	0.699032
TADA2A	97.98791	-0.03575	0.050795	-0.70388	0.481509	NA

KHK	76.57081	0.035753	0.052294	0.683684	0.494175	NA
RFC5	60.85979	-0.03575	0.062072	-0.57595	0.564651	NA
MTIF2	62.41433	0.035746	0.065333	0.547132	0.584288	NA
BOK	68.49081	0.035746	0.069176	0.516737	0.60534	NA
LINC00439	0.163851	-0.03575	0.024486	-1.45983	0.144337	NA
PGAM1P5	0.305903	0.035735	0.032317	1.105788	0.268818	NA
TFR2	42.00566	-0.03573	0.076956	-0.46433	0.64241	NA
LINC00085	116.1016	0.035731	0.072791	0.490867	0.62352	NA
ZNF729	1.541009	0.035729	0.062956	0.567533	0.570352	NA
LINC00673	36.55385	-0.03573	0.073205	-0.48805	0.625515	NA
SLC18A1	0.104199	-0.03572	0.019504	-1.83167	0.067001	NA
ARL4D	7.52098	0.035723	0.084749	0.421514	0.67338	NA
CASC4	651.9323	0.035712	0.044292	0.80629	0.420076	0.724567
KIAA1804	126.5223	0.035704	0.063041	0.566366	0.571145	NA
AFAP1-AS1	17.7656	-0.03568	0.083298	-0.42839	0.668366	NA
LOC10012	90.13925	-0.03568	0.056388	-0.63284	0.526841	NA
MAPKAPK	87.59275	0.035682	0.062225	0.57343	0.566354	NA
SSRP1	257.942	0.035677	0.03799	0.939117	0.347671	NA
LOC10019	3.517215	0.035674	0.069808	0.511027	0.609332	NA
LRPPRC	694.5959	0.035657	0.058361	0.610973	0.541218	0.802088
MFSD7	9.301686	-0.03566	0.084239	-0.42327	0.672102	NA
TRAFD1	84.83911	0.03565	0.056477	0.631238	0.527885	NA
HOXB3	0.79162	0.035646	0.04318	0.825521	0.409076	NA
SPATA5	206.0136	0.035643	0.04976	0.716306	0.473803	NA
MBP	4328.387	-0.03564	0.083128	-0.42877	0.668093	0.860768
MIR4466	0.966535	0.035642	0.05087	0.700643	0.483526	NA
MZT1	100.9386	0.03564	0.0776	0.459285	0.64603	NA
NCL	2091.11	0.035638	0.048672	0.732203	0.464045	0.755844
BAG1	153.6659	-0.03563	0.069756	-0.5108	0.609494	NA
SNORD114	2.669707	-0.03563	0.07188	-0.49569	0.620112	NA
PRMT7	160.2812	-0.03563	0.0495	-0.7197	0.471711	NA
KLHL20	89.11333	-0.03562	0.053561	-0.66509	0.505992	NA
MIR3167	1.698994	0.035622	0.067065	0.531153	0.595313	NA
MSMP	16.41956	-0.03561	0.082912	-0.42953	0.667534	NA
RLF	444.0836	0.035613	0.042639	0.835235	0.403586	0.709466
ZNF366	12.77848	-0.03561	0.084347	-0.4222	0.672882	NA
LRRC49	185.1489	0.035609	0.048573	0.733095	0.463501	NA
CALCRL	35.04844	-0.03561	0.082805	-0.43003	0.667176	NA
TMEM238	5.634921	0.035606	0.082368	0.432278	0.66554	NA
CCDC27	0.247967	0.035604	0.030286	1.175611	0.23975	NA
MIR4779	0.110075	-0.0356	0.019624	-1.81431	0.06963	NA
SLC30A4	88.5352	0.035601	0.055617	0.640103	0.522105	NA
P2RY2	1.274212	0.035596	0.048015	0.741353	0.458479	NA
SLC31A2	48.62342	-0.0356	0.064455	-0.55225	0.580777	NA
LARGE	130.8345	-0.03559	0.077472	-0.45943	0.645927	NA
ZNF324	85.66619	-0.03559	0.057708	-0.61674	0.537407	NA
LMO1	0.648398	0.035591	0.043731	0.813858	0.415726	NA

MAP7D1	766.4783	0.035575	0.049583	0.717488	0.473073	0.759174
HNF4A	0.537918	0.035566	0.042859	0.829832	0.406634	NA
PLA2G15	40.84816	-0.03557	0.067748	-0.52496	0.599609	NA
FAM19A2	7.178546	-0.03556	0.082828	-0.42932	0.667689	NA
ACTL6A	36.0173	0.035559	0.069166	0.514109	0.607176	NA
ZNF384	199.8375	0.035556	0.043271	0.821694	0.411251	NA
MBOAT2	153.1637	-0.03556	0.054871	-0.64798	0.517001	NA
SNORD1C	7.071045	-0.03555	0.074426	-0.47764	0.632907	NA
LOC28500	1.489919	0.035542	0.057604	0.617009	0.537229	NA
GGT1	29.12089	-0.03553	0.082136	-0.43256	0.665336	NA
PGBD5	225.4796	0.035526	0.055213	0.643446	0.519935	NA
PAQR8	266.3444	-0.03553	0.064586	-0.55005	0.582287	NA
CLPX	192.3738	-0.03552	0.049183	-0.72222	0.47016	NA
SPSB2	9.862438	0.035515	0.08447	0.420451	0.674156	NA
HINFP	111.3284	-0.03551	0.053734	-0.66083	0.50872	NA
ARHGAP26	1193.152	-0.0355	0.054919	-0.64647	0.517977	0.785288
HP	3.652702	0.035501	0.075189	0.472155	0.636816	NA
CRYBB2P1	71.06016	0.035495	0.06232	0.569567	0.568972	NA
WDR85	66.11205	-0.03549	0.061127	-0.58065	0.561477	NA
ZC3H15	337.3393	0.035483	0.044191	0.802949	0.422004	0.726322
CFD	6.197576	0.03548	0.083461	0.425113	0.670754	NA
GALNT13	728.3979	0.035479	0.043991	0.806497	0.419956	0.724567
HSBP1	127.6157	0.035475	0.068349	0.519027	0.603742	NA
DIO1	8.577405	-0.03547	0.084939	-0.41764	0.676212	NA
A2M-AS1	31.1922	-0.03547	0.078609	-0.4512	0.651844	NA
SRPK2	657.3559	0.035454	0.039782	0.891208	0.372818	0.695376
PIK3CA	242.4064	-0.03545	0.0403	-0.87963	0.37906	NA
HIST1H3D	8.750444	-0.03545	0.080463	-0.44057	0.659527	NA
GMDS	38.50027	0.035446	0.071252	0.497474	0.618855	NA
SNRNP25	55.05976	0.035445	0.077144	0.459465	0.6459	NA
ZNF284	66.9339	0.035438	0.063957	0.554101	0.57951	NA
PKP1	0.425692	-0.03544	0.037901	-0.93497	0.349803	NA
C3orf55	0.28593	-0.03544	0.030042	-1.17953	0.238188	NA
TMPRSS11	3.325393	-0.03542	0.071603	-0.49472	0.6208	NA
PMPCB	278.9792	-0.03542	0.040417	-0.87631	0.38086	NA
VAMP3	86.16569	-0.03541	0.060794	-0.58251	0.560226	NA
NCOR2	1772.654	0.035406	0.044831	0.789755	0.429671	0.73339
REN	0.240617	0.035405	0.026955	1.313483	0.18902	NA
TMEM72	1.11636	-0.0354	0.055253	-0.64063	0.521766	NA
CDC123	72.11267	-0.0354	0.063288	-0.55928	0.575974	NA
DAPK2	10.2377	0.035395	0.08371	0.422832	0.672418	NA
BRICD5	41.0352	-0.03539	0.079011	-0.44786	0.654252	NA
TRABD	126.4896	0.035384	0.063404	0.558071	0.576796	NA
NDFIP1	749.1216	0.03537	0.07412	0.477199	0.63322	0.845517
C5orf4	78.87115	0.035369	0.072575	0.487344	0.626015	NA
GK3P	6.242206	0.035366	0.084014	0.420949	0.673792	NA
CGN	264.9892	-0.03536	0.071944	-0.49154	0.623044	NA

IL17REL	0.744815	0.03536	0.048071	0.73559	0.46198	NA
SOS1	609.8671	0.035356	0.045157	0.782946	0.433659	0.736097
FAM170B-	0.192557	-0.03535	0.022606	-1.56381	0.117863	NA
ADH5	89.82642	-0.03534	0.057778	-0.61174	0.540713	NA
AAR2	29.20981	0.035338	0.072332	0.488553	0.625158	NA
BCRP3	4.908087	0.035326	0.081054	0.435836	0.662955	NA
WDR73	140.1	-0.03533	0.054351	-0.64995	0.515726	NA
SERPINA5	1.257474	-0.03532	0.051869	-0.68104	0.495848	NA
FLVCR1	102.0671	-0.03532	0.056185	-0.62865	0.529581	NA
MIR3682	4.087703	0.035314	0.080343	0.439546	0.660266	NA
EIF3H	222.6242	-0.03531	0.065838	-0.53637	0.591699	NA
CEP250	875.2494	0.035314	0.048865	0.722669	0.469883	0.757498
C11orf21	0.311761	-0.03531	0.033807	-1.04444	0.29628	NA
POLDIP3	167.5129	-0.03531	0.05035	-0.70125	0.483149	NA
CYP46A1	25.74845	-0.0353	0.081009	-0.4358	0.662981	NA
FOXO3	482.811	0.035304	0.042932	0.82231	0.4109	0.715365
FRA10AC1	190.051	0.0353	0.051992	0.678955	0.497166	NA
DDX50	139.6771	0.035295	0.050853	0.694065	0.487641	NA
ZNF492	4.138269	0.035294	0.071371	0.494516	0.620942	NA
ADAT1	63.96133	0.035287	0.067414	0.523444	0.600666	NA
POM121C	147.5273	0.035284	0.052438	0.672866	0.501032	NA
TMEM257	6.492149	0.035267	0.084738	0.416182	0.677277	NA
MIR101-2	0.257857	-0.03526	0.030397	-1.15984	0.246113	NA
WDR53	19.62917	-0.03525	0.078115	-0.45122	0.651832	NA
MRPL20	47.48127	-0.03522	0.066681	-0.5282	0.597363	NA
MATN2	76.41989	-0.03522	0.075523	-0.46633	0.64098	NA
ITM2B	1584.759	0.035216	0.064967	0.542063	0.587775	0.825363
DNAJB7	6.808463	0.035214	0.084768	0.415421	0.677834	NA
NAA15	422.6099	0.035205	0.036828	0.955931	0.339107	0.66305
VPS16	79.4265	-0.0352	0.055763	-0.6313	0.527844	NA
PPP4R1L	16.27007	-0.0352	0.079168	-0.44463	0.656588	NA
ALDH8A1	37.06858	0.035199	0.084838	0.414894	0.678219	NA
ARSG	98.44678	-0.03519	0.076	-0.46309	0.643302	NA
LINC00595	0.45426	0.035192	0.039079	0.900553	0.367826	NA
BTBD7	291.5064	0.03519	0.042488	0.828239	0.407535	NA
CCDC127	54.78885	-0.03519	0.063423	-0.55485	0.579	NA
FAF1	237.3934	0.035185	0.042808	0.821924	0.41112	NA
TOMM34	25.68355	0.035183	0.076959	0.45717	0.647549	NA
TMEM198	47.27781	0.035182	0.068247	0.515514	0.606194	NA
ATRAID	31.52147	-0.03518	0.076277	-0.46117	0.644676	NA
PCSK5	39.31966	-0.03518	0.082856	-0.42454	0.671173	NA
SNAR-C3	0.337218	-0.03517	0.028608	-1.22951	0.218879	NA
RHCE	3.37187	-0.03517	0.076686	-0.45866	0.646478	NA
SNORA70D	0.522731	-0.03517	0.041906	-0.83931	0.401295	NA
CLCN6	225.1087	-0.03517	0.036565	-0.96183	0.336135	NA
MGST2	19.53945	0.03515	0.080823	0.434903	0.663633	NA
TMEM242	84.18541	0.035148	0.057256	0.613871	0.5393	NA

MYO1E	44.66364	-0.03515	0.083079	-0.42304	0.672267	NA
AFG3L2	285.4149	0.03514	0.061857	0.568082	0.569979	NA
MIR4769	0.365443	-0.03512	0.03434	-1.02285	0.306378	NA
NR2E3	3.098553	0.035117	0.07214	0.486791	0.626407	NA
LOC28493	67.9204	0.035117	0.083959	0.418264	0.675754	NA
SRRM2	7523.151	-0.0351	0.053871	-0.65162	0.514647	0.784438
ZNF223	47.76788	0.035102	0.062545	0.561225	0.574644	NA
RBMS3	25.63399	-0.0351	0.079323	-0.44246	0.658159	NA
AGTR2	0.713273	-0.03509	0.045947	-0.76365	0.445073	NA
SOX12	213.0454	-0.03509	0.063806	-0.54988	0.582404	NA
LOC10028	1.123944	0.035078	0.056377	0.622206	0.533806	NA
SCARB1	55.28684	-0.03508	0.067668	-0.51837	0.604203	NA
ARHGAP31	167.4298	-0.03507	0.073682	-0.47597	0.634093	NA
RBM5	964.6646	-0.03507	0.040751	-0.86056	0.38948	0.701813
CCDC15	13.96987	-0.03506	0.084892	-0.413	0.679605	NA
VTRNA1-2	0.139412	0.035058	0.020445	1.714716	0.086397	NA
LOC44031	0.602545	-0.03506	0.041372	-0.84735	0.396798	NA
UBL4B	0.782778	-0.03505	0.049637	-0.70619	0.480073	NA
NAA10	74.46949	0.035049	0.058014	0.604147	0.545746	NA
PCDHGC5	58.21026	-0.03504	0.074623	-0.4696	0.638644	NA
ZNF763	21.9419	-0.03504	0.080538	-0.43504	0.663533	NA
CCL2	1.928069	0.035032	0.050913	0.688071	0.491408	NA
ZNF274	81.31858	0.035023	0.056781	0.616799	0.537367	NA
MIR643	0.612533	0.035015	0.043366	0.807429	0.419419	NA
PCGF5	410.8072	0.035007	0.045833	0.763794	0.44499	0.743291
CCDC88A	925.6948	0.035004	0.052321	0.669018	0.503484	0.780021
NEU4	8.228707	0.035002	0.084938	0.412091	0.680273	NA
IL1A	0.574816	0.035001	0.043349	0.807433	0.419417	NA
CXorf58	0.238695	-0.03499	0.027128	-1.28987	0.197097	NA
KCTD18	35.19501	-0.03499	0.072081	-0.48539	0.6274	NA
ZNF781	116.6458	0.034987	0.064115	0.54569	0.585279	NA
FAM227A	38.87706	0.03498	0.082338	0.424832	0.670959	NA
MIR106B	1.30777	0.034978	0.054607	0.640545	0.521818	NA
CLN8	91.00239	-0.03497	0.064617	-0.54121	0.588365	NA
ZNF346	213.7636	-0.03497	0.048138	-0.72647	0.467552	NA
LOC10013	23.85153	0.034966	0.08145	0.429296	0.667708	NA
LOC10050	9.631999	-0.03496	0.084519	-0.41368	0.679105	NA
KCNA1	613.3875	0.034952	0.056181	0.622138	0.533851	0.795509
MTIF3	120.1888	0.034946	0.05067	0.689666	0.490404	NA
HHEX	2.982684	0.034941	0.074869	0.466691	0.640721	NA
SRP72	257.8153	-0.03493	0.046424	-0.75238	0.451823	NA
CAV3	0.811203	-0.03493	0.050192	-0.69584	0.48653	NA
IL4	1.613632	0.034919	0.062965	0.554577	0.579184	NA
MIR636	0.268776	0.034918	0.025525	1.367972	0.171321	NA
ZNF695	3.120705	-0.03491	0.062712	-0.55672	0.577719	NA
C20orf118	13.62016	-0.03491	0.083734	-0.41691	0.676743	NA
DLD	249.6023	0.034909	0.046381	0.752664	0.451652	NA

CCL21	0.464834	-0.0349	0.038179	-0.91425	0.360585	NA
WRAP73	39.40578	0.034886	0.067659	0.51561	0.606127	NA
FLVCR2	4.40987	0.034884	0.077836	0.448174	0.654027	NA
RASAL2-AS	1.255597	0.03488	0.058985	0.591329	0.5543	NA
ZIC5	465.6153	0.034876	0.052952	0.65863	0.510133	0.781767
FAM118B	73.19545	0.034868	0.071092	0.490457	0.62381	NA
HCG17	13.80199	-0.03487	0.082486	-0.4227	0.672513	NA
GEMIN8P4	4.668713	-0.03486	0.081325	-0.42861	0.668205	NA
SNORD65	1.541688	-0.03486	0.063034	-0.55296	0.580291	NA
AVPR1B	1.085855	-0.03485	0.049804	-0.69975	0.484082	NA
SNORD91A	1.578477	-0.03485	0.05716	-0.60968	0.542071	NA
FLVCR1-AS	2.093042	-0.03484	0.069744	-0.4996	0.617359	NA
LRRC58	230.9828	0.034831	0.050368	0.691526	0.489235	NA
TCOF1	258.595	0.03483	0.047085	0.73972	0.45947	NA
ZBTB25	69.81627	-0.03481	0.052863	-0.65848	0.510228	NA
TBX18	7.271114	-0.03481	0.081665	-0.42622	0.669945	NA
CCDC176	49.43611	-0.03481	0.068436	-0.50858	0.611045	NA
SRD5A3	40.90004	-0.0348	0.082533	-0.42167	0.673265	NA
ZNF383	43.80823	0.034796	0.064961	0.535647	0.592203	NA
ENSA	445.9742	0.034789	0.037102	0.937647	0.348426	0.673941
CECR7	68.03585	0.034783	0.064983	0.535254	0.592474	NA
RCN2	164.909	-0.03478	0.064563	-0.53873	0.590071	NA
FAM149A	55.7922	0.034777	0.080614	0.431399	0.666179	NA
YIPF7	9.979902	-0.03477	0.083034	-0.41872	0.675424	NA
LINC00346	3.407668	0.034764	0.077347	0.449459	0.653101	NA
NDST4	0.718759	-0.03476	0.046089	-0.75411	0.450782	NA
LOC39975	6.615987	-0.03475	0.079825	-0.4353	0.663345	NA
KAZALD1	0.484606	-0.03473	0.038468	-0.90293	0.366562	NA
HAUS2	39.30209	-0.03473	0.064287	-0.54028	0.589002	NA
NDC80	0.211402	-0.03473	0.024179	-1.43641	0.150885	NA
FAM114A1	46.55779	-0.03473	0.082786	-0.41951	0.674842	NA
HEMGN	0.266687	-0.03472	0.029511	-1.17645	0.239413	NA
MIR99A	0.627762	0.034718	0.043816	0.792349	0.428157	NA
TMED11P	7.790727	-0.03472	0.081431	-0.42632	0.669875	NA
SLC25A31	0.228255	0.034715	0.02692	1.289555	0.197205	NA
USP17L7	14.27249	-0.03471	0.079276	-0.43784	0.661504	NA
PSME2	33.53167	-0.03471	0.073487	-0.47231	0.636707	NA
PPM1K	526.5597	0.034696	0.046756	0.742067	0.458047	0.754035
SCARNA1	3.157102	-0.03469	0.077499	-0.44766	0.654402	NA
KIAA1919	93.68448	-0.03469	0.065267	-0.53153	0.595052	NA
GIMAP7	12.90588	0.03469	0.084247	0.41176	0.680516	NA
XRN2	268.7148	-0.03468	0.035917	-0.96561	0.334237	NA
PARL	61.32856	-0.03468	0.061598	-0.56294	0.573478	NA
TBCA	149.4893	-0.03466	0.064268	-0.53926	0.589709	NA
G3BP1	230.6171	0.034646	0.049896	0.694375	0.487447	NA
PARVG	13.22393	-0.03464	0.079607	-0.43519	0.663427	NA
DCAF5	385.2942	-0.03464	0.036133	-0.95875	0.337684	0.66305

POLE2	16.11581	0.034639	0.080588	0.429831	0.667319	NA
OACYLP	0.466467	0.034638	0.03875	0.893902	0.371375	NA
RIPPLY2	23.26078	-0.03463	0.082187	-0.42137	0.673488	NA
FAM221B	12.71803	0.03463	0.08374	0.413539	0.679212	NA
STEAP1	2.766392	-0.03463	0.075049	-0.46142	0.644495	NA
MIR548H3	0.997231	-0.03462	0.05044	-0.6864	0.492463	NA
LOC10050	1.798161	-0.03462	0.064911	-0.53331	0.59382	NA
FANCE	17.67781	0.034607	0.079579	0.434874	0.663654	NA
ZBTB44	459.257	0.034606	0.047611	0.726859	0.467313	0.755844
VAV3	68.26136	-0.0346	0.07669	-0.45122	0.651829	NA
FAM102A	138.5911	0.034598	0.054496	0.634876	0.525509	NA
GBP1	15.17206	0.034586	0.082171	0.420901	0.673828	NA
RFX5	116.1595	0.034581	0.073427	0.470956	0.637672	NA
OTUD6B	82.63602	0.034576	0.055543	0.622498	0.533614	NA
LPAL2	0.515672	0.034566	0.04078	0.847629	0.396645	NA
MIR3192	0.800154	-0.03456	0.049049	-0.70464	0.481036	NA
HFE	4.033435	-0.03456	0.080319	-0.43029	0.666982	NA
SNORD110	26.64003	-0.03455	0.080849	-0.4274	0.669092	NA
MLLT10	447.733	0.03455	0.037754	0.915141	0.360118	0.683565
TCEAL2	302.6058	0.034546	0.068198	0.506547	0.612473	NA
ALKBH3	82.34161	-0.03454	0.059047	-0.58504	0.558523	NA
CCDC23	41.57843	-0.03454	0.075433	-0.45792	0.647007	NA
PLEKHA2	35.32034	-0.03454	0.071767	-0.48131	0.630295	NA
MSL1	471.9503	-0.03453	0.037738	-0.9151	0.360141	0.683565
SNORD114	2.926252	-0.03453	0.07566	-0.45635	0.648139	NA
NRIP2	240.9127	0.034511	0.040865	0.844508	0.398386	NA
SOX2-OT	149.0676	-0.0345	0.07896	-0.43697	0.662134	NA
TMEM209	50.06738	-0.0345	0.07288	-0.47336	0.635956	NA
ARL6IP1	589.0007	-0.03449	0.073721	-0.46783	0.639907	0.848092
GIMAP6	10.00497	0.034482	0.084389	0.408604	0.68283	NA
CEACAM2	1.039907	-0.03448	0.054135	-0.63695	0.524157	NA
KIAA0020	232.0572	0.03448	0.054816	0.629009	0.529343	NA
FAM209B	0.4929	-0.03448	0.037082	-0.92981	0.35247	NA
HP07349	0.716584	0.034477	0.046124	0.747498	0.454763	NA
EBAG9	76.94797	0.034476	0.056901	0.605887	0.54459	NA
NUP205	292.482	-0.03447	0.038121	-0.90418	0.365898	NA
C3orf22	0.458629	-0.03447	0.036275	-0.95019	0.342016	NA
CLDN23	0.306526	-0.03446	0.033808	-1.01941	0.308006	NA
H19	1.878373	-0.03446	0.055665	-0.61906	0.535877	NA
FAM73A	345.3481	0.034448	0.04495	0.766358	0.443463	0.743291
PLD6	93.37341	0.034445	0.083348	0.413262	0.679415	NA
SRD5A2	0.322384	0.034436	0.031308	1.099906	0.271373	NA
KIAA1549L	504.652	0.034433	0.076229	0.451701	0.651485	0.853449
FDPS	82.44413	-0.03443	0.065469	-0.52589	0.598966	NA
DYNLRB1	195.6568	0.034425	0.065433	0.526114	0.598809	NA
TF	232.7095	-0.03442	0.084936	-0.40521	0.685324	NA
FLJ10038	118.6385	0.034404	0.04962	0.693353	0.488088	NA

C1orf114	86.38033	0.034399	0.065473	0.525385	0.599316	NA
MPC1	66.56683	-0.03439	0.067634	-0.50851	0.611098	NA
ZBED5	254.1375	0.034389	0.036717	0.936601	0.348964	NA
TM2D3	97.25528	0.034386	0.055382	0.620896	0.534668	NA
NEFM	554.1484	0.034385	0.082157	0.418528	0.675561	0.863513
CENPV	48.72101	-0.03438	0.065885	-0.52185	0.601776	NA
SNORD55	135.4019	-0.03437	0.083588	-0.41117	0.680945	NA
NDUFA5	211.5561	-0.03437	0.071113	-0.48328	0.628896	NA
SLC44A5	3.766944	-0.03437	0.072582	-0.4735	0.635859	NA
SUMO1P3	9.894507	0.034365	0.084742	0.405525	0.685092	NA
ZDHHC19	0.275517	-0.03436	0.027148	-1.26573	0.205611	NA
CD46	210.0496	-0.03436	0.059424	-0.57823	0.563108	NA
NSMCE1	49.26694	0.034353	0.070857	0.484815	0.627807	NA
SMEK2	406.163	0.034351	0.035206	0.975713	0.329206	0.65961
SLC6A7	241.9579	0.034346	0.069069	0.497278	0.618993	NA
METAP1	84.28636	-0.03434	0.058523	-0.58684	0.557312	NA
SPATS1	2.136658	-0.03434	0.067834	-0.50628	0.612657	NA
RPL22	135.8604	-0.03434	0.067003	-0.51254	0.608273	NA
AGBL1	1.346137	0.034341	0.056886	0.603675	0.54606	NA
FLJ14186	7.798395	0.03434	0.083538	0.41107	0.681021	NA
AIDA	38.85869	0.034335	0.065594	0.523445	0.600665	NA
ABCD4	164.0284	-0.03433	0.063606	-0.53977	0.589355	NA
HIST1H2A	0.477042	-0.03433	0.039414	-0.87097	0.383771	NA
R3HCC1L	138.2259	0.034322	0.047559	0.721684	0.470489	NA
VDAC3	206.7683	-0.03432	0.060166	-0.57034	0.568445	NA
DEFB135	0.60378	0.034311	0.04401	0.779622	0.435614	NA
PDIA4	135.4714	0.03431	0.078618	0.43641	0.662539	NA
NBPF14	76.02054	0.034305	0.080277	0.427339	0.669132	NA
MYLIP	43.34227	0.034299	0.063947	0.536357	0.591712	NA
STRN4	155.7494	-0.03429	0.056527	-0.6066	0.544119	NA
DDX11	56.32547	-0.03428	0.077831	-0.44046	0.659606	NA
MCFD2	191.2719	0.03428	0.055996	0.612191	0.540411	NA
FASTKD3	16.99147	-0.03428	0.079712	-0.43001	0.667191	NA
LOC10027	6.72962	0.034262	0.083141	0.412094	0.680271	NA
NTMT1	16.85983	0.034257	0.078905	0.43416	0.664172	NA
TMEM189	42.99387	0.034254	0.068259	0.501818	0.615795	NA
ZRANB2-A	1.891169	0.034252	0.062332	0.549512	0.582654	NA
SIRT5	97.56683	-0.03425	0.055882	-0.61293	0.539921	NA
LOC10050	15.69096	-0.03425	0.081634	-0.41951	0.67484	NA
RPL19P12	60.65467	-0.03424	0.082808	-0.41354	0.679208	NA
HIST3H2B	18.18832	0.034241	0.08457	0.404889	0.685559	NA
CACNG2	241.1035	-0.03423	0.065485	-0.52275	0.601151	NA
DPY19L4	95.67978	0.034232	0.058412	0.586039	0.55785	NA
ZNF18	36.20747	0.034231	0.076854	0.445402	0.656029	NA
KIAA1257	11.04023	0.034224	0.084719	0.403965	0.686239	NA
DOK4	83.76378	0.034222	0.057483	0.595346	0.551612	NA
PIK3R2	200.5593	0.034221	0.046942	0.728995	0.466005	NA

SNORD116	203.5805	-0.03421	0.072172	-0.47407	0.635447	NA
TRIM35	73.82187	0.034215	0.059377	0.576229	0.56446	NA
CYP2C18	2.022203	0.034213	0.067416	0.507495	0.611808	NA
PTRHD1	6.34149	0.034207	0.084022	0.407118	0.683921	NA
BNIP3	246.9541	-0.03421	0.068779	-0.49732	0.61896	NA
CIRH1A	65.33283	0.0342	0.059613	0.573705	0.566168	NA
SUMO3	201.424	0.034192	0.069692	0.490621	0.623695	NA
OR2AG1	0.25587	-0.03418	0.029501	-1.15865	0.246597	NA
ZNF652	550.19	0.03417	0.037389	0.913893	0.360773	0.683809
DCBLD2	199.6444	-0.03417	0.045943	-0.74367	0.457079	NA
LOC40089	11.94417	0.034155	0.084416	0.404605	0.685768	NA
NME5	21.24192	-0.03415	0.081536	-0.41888	0.675307	NA
MIR5190	9.730277	-0.03415	0.084714	-0.40311	0.686864	NA
SNORD116	203.9236	-0.03415	0.042577	-0.80202	0.42254	NA
MMADHC	54.18249	-0.03415	0.068476	-0.49866	0.618018	NA
NME6	24.92557	0.034143	0.074374	0.459072	0.646183	NA
SYNDIG1	142.4222	0.034142	0.062992	0.542009	0.587812	NA
ZNF154	260.3467	0.034137	0.051448	0.663531	0.50699	NA
ZDHHC1	16.24466	0.034126	0.08102	0.421211	0.673601	NA
MAVS	496.5442	-0.03413	0.051935	-0.65708	0.511127	0.78276
HDGFRP3	259.3038	0.034124	0.049597	0.688016	0.491443	NA
OR5K2	1.951947	0.034109	0.061167	0.557634	0.577094	NA
KRT10	36.52894	0.034099	0.072832	0.468187	0.639651	NA
CIT	749.4092	-0.03409	0.056496	-0.60346	0.546201	0.803341
MIR4295	0.303999	0.034092	0.033229	1.025972	0.304905	NA
RMND1	66.97607	0.03409	0.061815	0.551483	0.581303	NA
LRRC26	7.398321	0.034074	0.082376	0.413636	0.679141	NA
PIK3CB	295.2366	0.03407	0.044877	0.759188	0.44774	NA
AMMECR1	74.23981	0.034057	0.053287	0.63912	0.522745	NA
GCSH	12.99937	-0.03405	0.08315	-0.40946	0.682204	NA
ITIH4	56.87782	-0.03405	0.081227	-0.41915	0.675109	NA
GPR141	0.234545	-0.03402	0.027774	-1.22488	0.22062	NA
ADCY4	14.0867	-0.03402	0.084854	-0.40089	0.688498	NA
NRGN	1.710165	0.034004	0.063602	0.53464	0.592899	NA
C15orf59	50.98304	-0.034	0.078335	-0.43402	0.664275	NA
FAM120B	430.3307	0.033998	0.044674	0.761014	0.446649	0.745512
CACUL1	239.0391	0.033997	0.041274	0.823672	0.410126	NA
LOC10050	0.303771	0.033993	0.032617	1.042181	0.297328	NA
WFDC6	0.180801	0.033988	0.023574	1.441746	0.149374	NA
SLC23A2	410.43	0.033986	0.055003	0.617898	0.536642	0.797569
SLC17A6	0.795587	-0.03399	0.039718	-0.85567	0.392179	NA
RAB3B	71.63389	-0.03398	0.077475	-0.43858	0.660962	NA
LAMB2	103.0817	0.033973	0.074952	0.45327	0.650354	NA
TMEM132	54.15203	0.033972	0.076376	0.444798	0.656466	NA
CD6	2.754333	-0.03397	0.073445	-0.46255	0.643687	NA
TBX21	0.224716	0.033971	0.026433	1.285203	0.198721	NA
JAK1	615.0097	0.033971	0.061358	0.553658	0.579813	0.822777

LOC10050	31.89806	0.033967	0.075057	0.452557	0.650868	NA
BCL7C	41.82354	-0.03397	0.064629	-0.52557	0.599186	NA
SDR9C7	2.560344	-0.03397	0.07029	-0.48324	0.628928	NA
KIF7	18.3157	0.033957	0.080687	0.420846	0.673868	NA
43167	173.3879	0.033952	0.073314	0.463102	0.643291	NA
LOC28483	42.24446	-0.03395	0.076887	-0.44157	0.658798	NA
LRRC73	6.678867	-0.03394	0.084418	-0.4021	0.687609	NA
AGXT2L2	62.48966	-0.03394	0.068467	-0.49573	0.620087	NA
TRPM5	1.303093	-0.03393	0.057365	-0.59155	0.55415	NA
MRRF	38.48257	0.033933	0.065219	0.520291	0.602861	NA
PSMF1	196.0011	0.033919	0.040028	0.847376	0.396785	NA
LOC64621	240.4521	0.033919	0.08481	0.399937	0.689203	NA
TTLL9	8.023364	-0.03391	0.084139	-0.40303	0.686924	NA
ACER3	63.57529	0.033905	0.067515	0.50219	0.615534	NA
LACTB	35.94229	-0.0339	0.075573	-0.4486	0.653721	NA
FMR1	653.4521	0.033901	0.053316	0.635847	0.524876	0.789308
PRMT1	148.6158	0.033891	0.049298	0.687466	0.491789	NA
RHBDD1	63.06338	-0.03387	0.062746	-0.53988	0.589282	NA
TRRAP	1175.238	0.033872	0.055814	0.606877	0.543933	0.803134
AATF	238.4864	0.033853	0.045184	0.749213	0.453729	NA
PSMD6-AS	103.1163	0.033826	0.074214	0.455787	0.648543	NA
LOC28299	1.061009	0.033825	0.05553	0.609126	0.542441	NA
ADAM7	1.14995	-0.03382	0.054791	-0.6172	0.5371	NA
WDR7	511.2578	0.033797	0.041809	0.808365	0.41888	0.724567
YPEL2	342.0103	-0.0338	0.04525	-0.74689	0.455127	0.750995
GNL3	130.3295	0.033793	0.049515	0.682488	0.49493	NA
RAB5A	157.0578	0.033777	0.056682	0.595903	0.55124	NA
MTRNR2L	274.0622	-0.03378	0.070716	-0.47762	0.632919	NA
ACYP2	36.92956	-0.03377	0.079208	-0.42633	0.669868	NA
ATM	1018.178	0.033754	0.048694	0.693192	0.488189	0.765754
LOC38989	0.759027	-0.03375	0.046638	-0.72373	0.469229	NA
YBX2	0.216484	-0.03375	0.025294	-1.33436	0.182087	NA
SLC25A36	184.8869	-0.03375	0.061706	-0.54696	0.584408	NA
ANKRD26	449.0873	-0.03375	0.059302	-0.56911	0.56928	0.815571
FAM133A	13.0931	-0.03374	0.084625	-0.39872	0.690099	NA
43358	100.5163	-0.03374	0.075807	-0.44509	0.656252	NA
TOP3B	80.30374	-0.03374	0.067804	-0.49762	0.618749	NA
SCNN1G	54.483	0.03374	0.076229	0.442612	0.658047	NA
CXorf31	0.311797	-0.03374	0.033105	-1.01917	0.30812	NA
LMF2	65.91745	-0.03374	0.054521	-0.61881	0.536041	NA
C8orf40	42.56943	-0.03374	0.072125	-0.46776	0.639956	NA
LOC10012	32.54214	-0.03373	0.084473	-0.39933	0.689651	NA
WWP2	204.5472	-0.03372	0.042411	-0.79505	0.426583	NA
BOC	47.68164	0.033715	0.083742	0.402608	0.687237	NA
KDM4C	837.2639	-0.03371	0.056169	-0.60024	0.54835	0.803817
SAP25	18.52699	-0.0337	0.084467	-0.39898	0.68991	NA
DCTN6	138.5029	0.033697	0.065697	0.512917	0.608009	NA

CSNK1A1P	3.859865	0.033695	0.077369	0.435509	0.663193	NA
TYW3	93.15572	0.033691	0.0635	0.530575	0.595713	NA
C19orf45	3.189466	0.033688	0.075019	0.449058	0.65339	NA
ZNF20	0.186186	0.033685	0.023672	1.423002	0.154735	NA
SLC3A2	105.3216	-0.03368	0.056426	-0.59697	0.55053	NA
LRRC20	44.71245	-0.03368	0.079822	-0.42198	0.673042	NA
TCF4	1411.722	0.033669	0.036531	0.921651	0.356711	0.680826
GPRIN1	223.4988	0.033669	0.056713	0.593664	0.552737	NA
TAS2R4	24.79456	0.033663	0.079491	0.423487	0.67194	NA
LILRA4	0.667888	-0.03365	0.039395	-0.85424	0.392972	NA
CAMK1D	203.8415	-0.03365	0.048828	-0.68916	0.490721	NA
HAPLN3	1.553265	-0.03365	0.054335	-0.61932	0.535709	NA
ARHGEF19	3.034484	-0.03365	0.076589	-0.43932	0.660431	NA
HLA-B	181.0212	-0.03365	0.079414	-0.42367	0.671805	NA
MED6	145.4163	0.033642	0.04647	0.723952	0.469095	NA
SNORA51	6.547107	-0.03364	0.083211	-0.40429	0.686001	NA
STRN	575.9107	0.033629	0.039821	0.844498	0.398391	0.706622
MIPOL1	48.4468	0.033625	0.084936	0.395888	0.692188	NA
RIC3	266.0091	-0.03362	0.040909	-0.82185	0.411165	NA
NALCN-AS	7.542287	-0.03362	0.084876	-0.39611	0.692027	NA
STK25	244.6591	-0.03361	0.042465	-0.79155	0.428623	NA
PTPRH	2.668608	0.033605	0.065207	0.515357	0.606304	NA
PLAC9	14.96548	-0.0336	0.082678	-0.40645	0.684411	NA
LGR6	11.55305	-0.0336	0.080758	-0.41611	0.677326	NA
PAQR7	25.14554	0.033596	0.075055	0.447619	0.654428	NA
DACT2	5.771494	-0.03358	0.082966	-0.40469	0.685703	NA
NACAP1	1.474655	-0.03357	0.061275	-0.54793	0.583738	NA
KISS1R	0.805674	-0.03357	0.049055	-0.68438	0.493732	NA
TRIOBP	176.918	-0.03357	0.06232	-0.53869	0.590103	NA
SBDSP1	79.87063	-0.03357	0.060797	-0.55213	0.580862	NA
ATP13A5	3.419479	0.033567	0.078298	0.428713	0.668132	NA
UBA6	262.7542	0.033559	0.044957	0.746455	0.455393	NA
GFRAL	1.853233	-0.03356	0.066395	-0.50539	0.613286	NA
PSMC2	152.1127	0.033554	0.059881	0.560349	0.575242	NA
ST3GAL6-A	4.449709	-0.03355	0.081269	-0.4128	0.679756	NA
LINC00609	1.663389	-0.03355	0.063966	-0.52443	0.599983	NA
INO80D	698.9539	0.033543	0.033832	0.99144	0.321471	0.651336
MIR4759	0.282253	0.033539	0.029496	1.137066	0.255511	NA
IL18RAP	0.560307	-0.03353	0.033154	-1.01146	0.311798	NA
YLPM1	1869.968	-0.03353	0.03111	-1.07788	0.281086	0.616856
TARSL2	267.0365	-0.03353	0.042812	-0.78316	0.433535	NA
CNOT10	105.5489	-0.03353	0.048302	-0.69411	0.487615	NA
MIR3170	0.173382	-0.03352	0.023561	-1.42261	0.15485	NA
C16orf80	54.08196	0.033514	0.075543	0.44364	0.657303	NA
JSRP1	0.486874	0.033513	0.036836	0.909787	0.362935	NA
NTNG1	34.31183	0.033512	0.081857	0.409403	0.682244	NA
LOC72843	4.937955	-0.03351	0.080526	-0.41614	0.67731	NA

WTH3DI	1.291515	0.033495	0.059829	0.559838	0.57559	NA
SNRPB	99.27267	-0.03348	0.075694	-0.44236	0.658225	NA
MIR4681	0.207562	-0.03348	0.024004	-1.39467	0.163115	NA
HTR3D	0.184136	0.033472	0.023676	1.413763	0.157431	NA
MIMT1	4.761859	0.033455	0.082293	0.406533	0.684351	NA
SNORA39	1.679234	0.033448	0.060004	0.557433	0.577231	NA
TINCR	3.099159	-0.03345	0.074829	-0.44699	0.654883	NA
MAN1A1	33.64065	-0.03344	0.084574	-0.3954	0.692549	NA
GFOD2	303.0525	0.033438	0.052582	0.635916	0.524831	NA
FZD9	4.035082	-0.03344	0.079835	-0.41882	0.675349	NA
SMARCAD	339.6581	0.033436	0.036734	0.910216	0.362708	0.686402
CACNA1I	258.3899	0.033432	0.08044	0.41562	0.677688	NA
SCN9A	14.83061	-0.03343	0.084895	-0.39379	0.693734	NA
MTA1	308.1323	-0.03343	0.042965	-0.77809	0.436518	NA
BYSL	14.2596	-0.03343	0.083889	-0.3985	0.690264	NA
ATCAY	470.1082	-0.03343	0.0377	-0.88662	0.375285	0.695376
RGS14	1.842721	0.033423	0.06031	0.554192	0.579447	NA
CFB	40.19736	-0.03342	0.078173	-0.42754	0.668985	NA
PEA15	932.9295	0.033413	0.043723	0.764199	0.444749	0.743291
UBE2T	6.811441	-0.03341	0.084126	-0.39718	0.691233	NA
SH3KBP1	54.46723	-0.03341	0.077904	-0.42881	0.668059	NA
DCST1	1.193743	0.033402	0.060136	0.555445	0.57859	NA
HIST1H2A	107.3074	-0.0334	0.083143	-0.40173	0.687883	NA
GSPT2	46.78946	0.033399	0.067791	0.492684	0.622236	NA
LPAR4	2.214655	-0.0334	0.070364	-0.47467	0.635023	NA
SNORD115	1.768968	0.033387	0.064997	0.51367	0.607483	NA
MIR3183	0.382417	0.03338	0.032942	1.0133	0.310917	NA
FBXW7	604.6075	0.033369	0.03833	0.870581	0.383983	0.70023
HERC6	10.5864	-0.03337	0.084939	-0.39283	0.694444	NA
SPRED3	31.91293	-0.03336	0.083074	-0.40157	0.687997	NA
ATG2B	791.6347	-0.03336	0.030962	-1.07732	0.281338	0.616856
ARL2	11.20131	-0.03335	0.084585	-0.39432	0.693343	NA
SIM2	33.9741	-0.03335	0.073287	-0.45506	0.649063	NA
NGDN	112.0591	0.033348	0.049793	0.669728	0.503031	NA
SLCO4A1	54.20687	-0.03335	0.084826	-0.3931	0.694245	NA
GPR75	86.7459	0.033344	0.060577	0.550436	0.58202	NA
CDH17	0.465889	0.033337	0.038586	0.863975	0.387602	NA
MYBPC3	4.746356	0.033326	0.082121	0.405818	0.684876	NA
BAG6	225.2457	0.033316	0.042127	0.790849	0.429032	NA
EXOC3L2	0.615315	0.033313	0.043883	0.759133	0.447773	NA
LINC00648	0.227727	-0.03331	0.028649	-1.16265	0.244971	NA
MIR486	1.719387	0.033308	0.062559	0.53242	0.594435	NA
MCMBP	133.4334	0.033303	0.04863	0.684819	0.493458	NA
TCEAL6	118.5757	-0.0333	0.066086	-0.50388	0.614349	NA
MIR4726	0.340571	-0.0333	0.03314	-1.00476	0.315012	NA
EDIL3	96.67627	0.033296	0.080366	0.414304	0.678652	NA
SKIV2L	162.8229	-0.03328	0.061064	-0.54504	0.585727	NA

PLD3	229.4972	-0.03328	0.062195	-0.5351	0.592579	NA
RBM45	34.64325	-0.03327	0.074629	-0.44587	0.655691	NA
ZNF227	179.6687	0.033271	0.046411	0.716881	0.473447	NA
POLR1A	214.0481	-0.03327	0.053189	-0.62552	0.531633	NA
ANKRD44	98.50605	-0.03327	0.069645	-0.47768	0.63288	NA
SHISA5	80.10622	-0.03325	0.072287	-0.46004	0.64549	NA
IKBIP	6.755723	0.033252	0.080577	0.412672	0.679847	NA
TIRAP	59.2878	-0.03325	0.064373	-0.51653	0.605486	NA
MIR129-1	0.236642	0.033241	0.029194	1.138618	0.254862	NA
LHFPL2	75.17192	-0.03324	0.064145	-0.51818	0.60433	NA
MLYCD	37.66925	0.033229	0.068332	0.486291	0.626761	NA
HRH3	49.06736	-0.03323	0.075544	-0.43983	0.660063	NA
MRPL55	23.80254	-0.03322	0.074451	-0.44625	0.655415	NA
LHX3	0.176022	0.033219	0.023607	1.407201	0.159368	NA
SCML4	0.215153	0.033217	0.027396	1.212464	0.225335	NA
LTN1	302.9621	0.033214	0.039098	0.849516	0.395594	NA
ITGA1	31.33798	-0.03321	0.08478	-0.39173	0.695257	NA
FRMPD1	13.36254	-0.0332	0.083057	-0.39971	0.689367	NA
POC5	52.76188	-0.0332	0.065904	-0.50374	0.614444	NA
RFX8	0.501123	-0.0332	0.037463	-0.88612	0.375551	NA
MDH1	269.042	-0.03319	0.077568	-0.42788	0.668738	NA
BTBD16	0.929325	-0.03317	0.051383	-0.64562	0.518526	NA
MIR5693	0.438366	-0.03316	0.03503	-0.94664	0.343823	NA
PFKFB1	2.327889	-0.03316	0.070707	-0.46895	0.639107	NA
CHST8	45.48469	0.033154	0.063767	0.519923	0.603117	NA
HBD	0.506616	0.033151	0.038938	0.851376	0.39456	NA
DNAI2	0.139728	-0.03315	0.020373	-1.62716	0.103702	NA
ZFPM2	1308.194	-0.03315	0.048449	-0.68416	0.493873	0.771788
DNAH2	60.87626	-0.03315	0.081989	-0.40428	0.686009	NA
ZNF486	41.12222	0.033139	0.071057	0.466366	0.640953	NA
PEX6	117.8539	0.033138	0.063378	0.522863	0.60107	NA
MLX	79.12435	0.033128	0.059127	0.560295	0.575278	NA
LOC10013	38.07795	-0.03313	0.067228	-0.49277	0.622173	NA
PSMB5	51.40805	-0.03312	0.067861	-0.48803	0.625527	NA
LINS	53.86515	-0.03312	0.070426	-0.47022	0.6382	NA
FRMD6-AS	8.739373	0.033115	0.084931	0.389908	0.696604	NA
NQO1	33.66486	0.033114	0.084311	0.392764	0.694494	NA
SCAMP2	17.35703	0.033112	0.082567	0.401038	0.688392	NA
HECW2	365.4134	0.033091	0.037285	0.887524	0.374797	0.695376
ASCC2	112.9696	-0.03309	0.049052	-0.6746	0.499932	NA
TBX19	0.950527	0.03309	0.054308	0.609299	0.542326	NA
KCNK5	0.338571	0.03309	0.023492	1.408573	0.158962	NA
NPFFR1	26.71633	0.033088	0.084929	0.389602	0.696831	NA
NF2	277.5085	-0.03308	0.0417	-0.79332	0.427592	NA
SPC24	0.334713	-0.03308	0.033896	-0.97595	0.329091	NA
C12orf70	2.05217	0.033077	0.065361	0.506071	0.612807	NA
ZNF444	45.09189	-0.03306	0.065398	-0.50557	0.613158	NA

TMEM167	297.9136	0.033063	0.065297	0.506339	0.612619	NA
TOMM70A	271.6151	0.033062	0.058226	0.567827	0.570153	NA
TMTC4	51.3193	0.033057	0.084885	0.389432	0.696957	NA
CST6	1.27677	-0.03306	0.054425	-0.60737	0.543604	NA
TMIGD2	0.584775	-0.03305	0.039129	-0.84453	0.398374	NA
THRSP	14.94538	0.033034	0.084102	0.392786	0.694478	NA
H3F3C	1.186297	-0.03303	0.055135	-0.59912	0.549091	NA
NCOR1P1	0.11269	0.033033	0.019917	1.658543	0.097208	NA
B4GALT3	69.63096	-0.03303	0.057202	-0.57739	0.563678	NA
TOMM40L	39.29986	0.033027	0.073149	0.451501	0.651629	NA
SKA3	3.344009	0.033022	0.072406	0.456067	0.648342	NA
GRASP	12.28823	-0.03302	0.083203	-0.39684	0.691483	NA
CINP	26.71276	0.033018	0.071169	0.463934	0.642695	NA
CEND1	252.921	0.033017	0.077749	0.424655	0.671088	NA
HIC2	47.45076	0.033015	0.069535	0.474793	0.634935	NA
XIRP2	0.674196	0.033012	0.040937	0.806419	0.420001	NA
NXF3	0.317782	-0.03301	0.032256	-1.02323	0.306199	NA
TSTD2	161.9904	-0.03299	0.059039	-0.5588	0.576298	NA
GCNT1	93.09127	0.032974	0.07832	0.421023	0.673739	NA
RFWD3	62.77833	0.032971	0.061662	0.534711	0.59285	NA
C9orf152	0.656076	-0.03297	0.044066	-0.74823	0.454322	NA
NEURL2	22.10059	-0.03297	0.079125	-0.41665	0.676936	NA
ZNF44	100.6706	0.032963	0.052444	0.628545	0.529647	NA
CASP6	9.992124	0.032948	0.07836	0.420468	0.674144	NA
ARMC10	31.95895	0.032946	0.073954	0.445489	0.655967	NA
HRNR	19.17833	-0.03294	0.07097	-0.4642	0.642505	NA
ARPP21	1388.44	0.032939	0.042806	0.769495	0.4416	0.742006
NDRG4	2104.233	0.032922	0.049423	0.666123	0.505333	0.780812
KCNT1	580.6095	-0.03292	0.075044	-0.43865	0.660915	0.855957
LRRC32	27.5236	-0.0329	0.084279	-0.39036	0.696267	NA
FBXO48	7.728125	-0.0329	0.084788	-0.38802	0.698004	NA
UBAP2L	540.44	0.032892	0.03697	0.889709	0.373622	0.695376
ZNF782	308.0062	0.032889	0.04615	0.712663	0.476054	NA
WBP4	129.1285	0.032888	0.056796	0.579055	0.562552	NA
RNF148	86.78478	-0.03288	0.069932	-0.47013	0.638261	NA
CDCP1	0.983415	-0.03286	0.052128	-0.63046	0.528396	NA
MAP4	845.8436	0.032863	0.045029	0.729827	0.465496	0.755844
RTEL1	8.041133	-0.03286	0.082742	-0.39717	0.691239	NA
TXNL4A	62.38473	0.032861	0.062372	0.526857	0.598293	NA
LINC00381	1.368446	-0.03285	0.062909	-0.52224	0.601503	NA
STK16	30.13	-0.03285	0.073479	-0.44707	0.654826	NA
UBE3A	745.6844	-0.03285	0.034616	-0.94896	0.342643	0.666057
TMEM66	390.3544	0.032848	0.062329	0.52701	0.598186	0.829672
PTGER4P2	1.992456	0.032844	0.063211	0.519592	0.603348	NA
OTOP1	0.209434	-0.03284	0.027681	-1.18641	0.235461	NA
RNF144A	712.6215	-0.03284	0.043855	-0.74875	0.454008	0.750995
MRTO4	57.3786	-0.03283	0.060592	-0.54183	0.587939	NA

RICTOR	949.8742	-0.03282	0.039549	-0.82997	0.406555	0.710565
PSMB7	42.93478	-0.03282	0.066928	-0.49044	0.623822	NA
PRKG2	4.826586	0.032824	0.082534	0.3977	0.690851	NA
KIAA1328	200.8499	-0.03281	0.053652	-0.61159	0.54081	NA
POR	66.83588	0.032801	0.060999	0.537726	0.590766	NA
ATP2B3	533.1306	0.032799	0.049515	0.662401	0.507714	0.780812
LOC10050	1.809722	-0.0328	0.066187	-0.49551	0.620241	NA
URGCP	177.5517	0.032796	0.044327	0.739854	0.459389	NA
PITHD1	189.2333	0.032791	0.065216	0.502811	0.615097	NA
BTAF1	462.8842	-0.03279	0.045099	-0.72708	0.467179	0.755844
OR5B21	0.328162	-0.03278	0.03199	-1.02482	0.305447	NA
FAM180B	18.96717	0.032782	0.083748	0.391436	0.695475	NA
TLN1	576.8906	-0.03278	0.05683	-0.57683	0.564056	0.813231
HECA	145.255	0.032777	0.060178	0.544658	0.585989	NA
GNPAT	130.6145	-0.03276	0.043007	-0.76165	0.446268	NA
PPP1CC	197.2638	0.032751	0.056464	0.580035	0.561891	NA
TUBA4B	0.394946	0.032746	0.035936	0.911211	0.362184	NA
EFCAB4A	13.3553	0.032739	0.084141	0.389104	0.697199	NA
OPN3	2.974359	-0.03274	0.073511	-0.44537	0.656056	NA
PRKAG1	68.0566	-0.03274	0.074874	-0.43721	0.661962	NA
MARCO	1.708618	0.03273	0.04852	0.67456	0.499956	NA
C14orf166	0.219949	-0.03273	0.028617	-1.14364	0.252774	NA
EPN2-AS1	32.35713	0.032718	0.072274	0.452691	0.650771	NA
ZNF282	109.3674	-0.03271	0.047428	-0.68972	0.490369	NA
GLYR1	314.1878	0.032711	0.041232	0.793321	0.427591	NA
HERC2	823.0855	0.032697	0.038265	0.854476	0.392841	0.704502
SOX15	16.54748	-0.03269	0.081046	-0.4034	0.686654	NA
SNORD126	0.14823	-0.03268	0.021503	-1.51996	0.12852	NA
PIK3R4	157.5546	0.032683	0.046836	0.697805	0.485299	NA
CHM	190.5967	-0.03268	0.058811	-0.55568	0.578427	NA
MIR199A1	1.80005	-0.03268	0.067171	-0.48651	0.626605	NA
CLCN3	540.5567	0.032671	0.05186	0.629979	0.528708	0.792854
NAGA	58.24671	-0.03266	0.065329	-0.50001	0.61707	NA
KIAA0319L	247.0789	-0.03266	0.05717	-0.57131	0.567792	NA
TMEM230	101.2003	0.03266	0.066915	0.488084	0.62549	NA
PLA2G4D	1.036862	0.032659	0.055693	0.586411	0.557599	NA
OLFM4	0.368528	-0.03265	0.033781	-0.96655	0.333771	NA
RNGTT	170.0156	0.03265	0.038332	0.851776	0.394339	NA
ST13P4	0.64901	-0.03265	0.046403	-0.70354	0.48172	NA
EGR3	1.869049	0.032642	0.049946	0.65354	0.513408	NA
AES	1088.747	0.032639	0.05584	0.584508	0.558879	0.808857
HPR	7.461244	-0.03264	0.08162	-0.39985	0.68927	NA
YIPF5	103.9971	0.032635	0.066735	0.48903	0.62482	NA
CD3E	0.260207	-0.03263	0.027008	-1.20834	0.226916	NA
CCR7	0.97647	-0.03263	0.053339	-0.61183	0.540648	NA
B3GALT5	2.061596	0.032628	0.062936	0.518435	0.604155	NA
RBMX2	68.73257	0.032618	0.063514	0.513561	0.607559	NA

SGK1	136.0165	-0.03261	0.052544	-0.62069	0.534801	NA
PPIP5K2	325.7839	0.032613	0.04498	0.72505	0.468421	NA
OR52A1	0.214385	-0.03261	0.027721	-1.17641	0.23943	NA
SMARCA1	411.8946	0.032609	0.050976	0.639696	0.52237	0.787214
LRRIQ3	0.391699	-0.03259	0.030837	-1.05688	0.290565	NA
HSCB	14.59036	-0.03259	0.08441	-0.38609	0.699433	NA
SNORA22	3.721971	0.032589	0.079078	0.412107	0.680261	NA
PPTC7	77.63832	0.032587	0.068128	0.478319	0.632423	NA
MIR4642	0.550912	-0.03259	0.040838	-0.79794	0.424904	NA
S1PR5	7.65897	0.032585	0.080179	0.406396	0.684452	NA
TBC1D20	247.5769	0.032579	0.045129	0.721904	0.470354	NA
NTN3	7.12058	-0.03257	0.084146	-0.38711	0.698673	NA
PLK4	1.889419	0.032572	0.065637	0.496252	0.619716	NA
TMEM243	19.11881	0.032571	0.082729	0.393707	0.693797	NA
NEURL3	1.082409	0.032569	0.052769	0.617195	0.537106	NA
GH1	0.159845	-0.03257	0.020674	-1.57526	0.115195	NA
MAP2K6	68.21378	-0.03257	0.065521	-0.49703	0.61917	NA
GGT8P	0.232147	0.032562	0.025627	1.270613	0.203866	NA
HMGCS1	178.3207	0.032561	0.076872	0.423579	0.671873	NA
PCDHB11	55.0742	-0.03256	0.07592	-0.42885	0.66803	NA
CSMD2	158.3127	0.032553	0.067745	0.480516	0.630861	NA
NCS1	382.5863	0.032548	0.058329	0.558011	0.576837	0.821271
FASTKD2	188.9203	-0.03255	0.042309	-0.76923	0.441758	NA
SNORA77	1.079917	-0.03254	0.053127	-0.61255	0.540176	NA
HNRNPA1	1.077473	-0.03254	0.051035	-0.63765	0.523702	NA
TMEM143	22.60142	-0.03254	0.075278	-0.43226	0.665553	NA
ABHD10	138.3276	0.032539	0.07409	0.439187	0.660526	NA
MIR1262	0.153749	-0.03254	0.020552	-1.58314	0.11339	NA
SLC25A29	48.95215	0.032537	0.071765	0.453381	0.650274	NA
CNTFR	282.9925	-0.03252	0.064048	-0.50781	0.611586	NA
SNX29	250.4859	-0.0325	0.045435	-0.71534	0.474402	NA
EIF2AK1	309.7711	-0.0325	0.042628	-0.76242	0.445809	NA
HSPBP1	33.95918	-0.0325	0.076542	-0.42454	0.67117	NA
ARHGEF18	131.0982	-0.03248	0.04711	-0.68945	0.490542	NA
BACE1	390.6765	0.032476	0.038251	0.849041	0.395859	0.705623
LOC10012	1.896481	-0.03247	0.062589	-0.51879	0.60391	NA
SPPL2A	39.50792	0.032467	0.067299	0.482434	0.629498	NA
JAM3	130.4601	0.032467	0.060643	0.535378	0.592389	NA
GPHB5	0.607754	0.032457	0.041455	0.782949	0.433657	NA
PEPD	66.25549	0.032453	0.075334	0.430785	0.666625	NA
PPP2R3C	69.92839	0.03245	0.067109	0.48354	0.628713	NA
DAG1	158.9827	0.032421	0.053379	0.607382	0.543598	NA
ILVBL	52.23077	-0.03242	0.062874	-0.51564	0.606109	NA
GAPDH	1156.314	-0.03241	0.073958	-0.43817	0.661262	0.855957
ARHGAP5-	16.88165	-0.03239	0.081326	-0.39831	0.690399	NA
GPR126	6.224078	0.032392	0.079036	0.409843	0.681921	NA
C5orf49	2.886025	0.032391	0.0731	0.44311	0.657687	NA

MARK3	765.412	0.032388	0.050102	0.646429	0.518002	0.785288
FGD1	92.40775	0.032379	0.054843	0.590395	0.554926	NA
TSKU	3.066584	-0.03238	0.068151	-0.47505	0.63475	NA
TMEM91	13.45001	-0.03237	0.083885	-0.38586	0.699601	NA
GLI2	22.46922	-0.03236	0.083929	-0.38557	0.699817	NA
TMEM237	140.4964	0.032352	0.053507	0.604623	0.545429	NA
GRAMD1A	111.3943	-0.03235	0.050889	-0.63567	0.524993	NA
PRND	0.624648	0.032348	0.037708	0.857856	0.390972	NA
TSKS	5.478787	0.032333	0.079954	0.404403	0.685916	NA
DAP3	80.69751	-0.03233	0.053866	-0.60017	0.548394	NA
AGTRAP	2.431135	0.032326	0.068926	0.468994	0.639074	NA
LOC25489	0.282564	-0.03233	0.029579	-1.09285	0.27446	NA
PRPF4B	852.68	-0.03232	0.055212	-0.58546	0.55824	0.808857
LINC00857	0.377006	0.032323	0.032672	0.989319	0.322507	NA
TRPM7	700.9694	0.032321	0.061985	0.521435	0.602064	0.830918
PRRG1	38.63957	0.032313	0.080073	0.403544	0.686548	NA
CDKN1B	365.289	-0.03231	0.06464	-0.49982	0.617203	0.839155
ENO2	1679.991	0.032296	0.061328	0.526603	0.598469	0.829672
SQRDL	7.332836	-0.03229	0.075263	-0.42907	0.667871	NA
GARS	250.398	0.032285	0.04868	0.663215	0.507193	NA
CPZ	0.84509	-0.03228	0.044202	-0.73038	0.465159	NA
TAS2R10	7.935222	-0.03228	0.082447	-0.39156	0.695387	NA
PLEKHG6	0.568343	-0.03228	0.038255	-0.84377	0.3988	NA
SNORA1	3.081174	0.032275	0.06981	0.462319	0.643852	NA
OCLM	15.16402	-0.03227	0.083247	-0.38769	0.698248	NA
ANKRD16	56.53164	-0.03227	0.05875	-0.54921	0.582862	NA
LRCH1	934.2619	0.032256	0.042221	0.763996	0.44487	0.743291
PRDM6	2.696833	0.032249	0.073728	0.437404	0.661818	NA
UBE2F-SCL	0.408657	0.032249	0.038978	0.827358	0.408034	NA
MTOR-AS1	5.170423	0.032249	0.081847	0.394013	0.693572	NA
FABP5	35.49807	-0.03224	0.084066	-0.38352	0.701333	NA
LDB2	6.972379	-0.03224	0.084297	-0.38243	0.702142	NA
GALNT16	157.0412	-0.03224	0.062629	-0.51472	0.606749	NA
CXCL3	0.302184	0.032234	0.026595	1.21203	0.225501	NA
FIBP	43.2541	-0.03222	0.0749	-0.43016	0.667082	NA
TMLHE-AS	1.124646	0.032216	0.056833	0.566856	0.570812	NA
ADC	34.38006	0.032216	0.073226	0.439949	0.659974	NA
LOC40216	11.3502	-0.03221	0.078594	-0.40983	0.68193	NA
XYLB	171.7155	0.03221	0.061485	0.523872	0.600368	NA
PRRG2	2.094037	-0.03221	0.067822	-0.47492	0.634844	NA
ZNF860	3.855005	0.0322	0.073821	0.436192	0.662697	NA
LOC10012	69.1104	0.032199	0.071385	0.451058	0.651948	NA
PMS2CL	46.15055	0.032195	0.069002	0.466585	0.640796	NA
EFHC2	7.107553	-0.03218	0.08404	-0.38293	0.701771	NA
FBXO9	287.1796	-0.03218	0.052923	-0.608	0.543186	NA
GPR37	27.40036	-0.03217	0.084561	-0.38049	0.703585	NA
SDHC	97.81199	-0.03217	0.062483	-0.5148	0.606695	NA

MIR591	0.308969	-0.03216	0.033141	-0.97052	0.331787	NA
APOA1BP	61.77239	-0.03215	0.064377	-0.49936	0.617526	NA
PFDN1	54.28139	0.032144	0.065832	0.488278	0.625353	NA
LINC00472	67.25479	-0.03214	0.076813	-0.41842	0.675643	NA
GFRA3	8.702416	-0.03214	0.084936	-0.37835	0.705171	NA
MGC2188	14.38839	0.032129	0.084715	0.379257	0.704497	NA
COG4	154.6762	0.032121	0.059391	0.540847	0.588613	NA
KCNJ2-AS1	1.607307	-0.03212	0.059846	-0.53672	0.59146	NA
SH2B2	22.29137	-0.03211	0.080544	-0.39866	0.690143	NA
GRB2	244.835	-0.03211	0.04557	-0.70456	0.481085	NA
ARL16	49.5961	-0.03211	0.065899	-0.48719	0.62612	NA
SETDB1	238.5092	-0.0321	0.044005	-0.72953	0.465676	NA
AP4B1	77.63719	0.032098	0.064301	0.499181	0.617652	NA
TSIX	21.7296	-0.0321	0.061501	-0.5219	0.601743	NA
OR2W3	0.147381	-0.03209	0.020669	-1.55269	0.120496	NA
MYBBP1A	120.5366	-0.03208	0.062886	-0.51019	0.609917	NA
ZSWIM5	128.6694	0.032081	0.050785	0.631696	0.527585	NA
LOC10012	0.446284	0.032053	0.03406	0.941082	0.346663	NA
ISG20	1.950732	0.032051	0.055743	0.574972	0.565311	NA
LTBR	18.70918	-0.03205	0.08458	-0.37893	0.704737	NA
MIR599	0.316438	-0.03205	0.032898	-0.97412	0.329996	NA
ZMAT4	3.086787	0.03203	0.076405	0.419211	0.675062	NA
TSTD1	3.154107	-0.03201	0.064994	-0.49257	0.622315	NA
DACH2	0.258953	-0.03201	0.029541	-1.08353	0.278572	NA
FAM222B	183.7827	0.032009	0.044225	0.723759	0.469214	NA
PSMG4	52.65978	-0.03199	0.069682	-0.45913	0.64614	NA
NMB	4.856481	-0.03199	0.078275	-0.40867	0.682781	NA
PPP1R12B	1162.499	-0.03199	0.043182	-0.74072	0.458863	0.754035
EFNA4	2.407984	0.03198	0.069816	0.45806	0.646909	NA
THAP11	35.50578	0.031979	0.070019	0.456715	0.647876	NA
HIST1H2B	25.14067	-0.03198	0.081189	-0.39386	0.693681	NA
LRRC9	6.951817	0.031976	0.084588	0.378022	0.705415	NA
KLHL3	855.8649	0.031972	0.043222	0.73972	0.45947	0.754035
NHP2L1	136.2456	0.031971	0.067442	0.474054	0.635461	NA
LOC72834	0.837485	-0.03194	0.04862	-0.65691	0.511236	NA
PHYHD1	35.30686	-0.03194	0.084446	-0.37822	0.70527	NA
ZNF737	251.5087	0.031935	0.057381	0.556534	0.577846	NA
MFAP5	1.140725	-0.03193	0.054407	-0.58695	0.557238	NA
C22orf26	68.89013	-0.03193	0.084919	-0.37603	0.706891	NA
EML5	1002.752	0.031932	0.039692	0.804507	0.421104	0.725324
PGBD3	17.2496	0.031924	0.081068	0.393785	0.69374	NA
FAM21B	10.54544	0.031919	0.040867	0.781037	0.434781	NA
UBE2E3	78.07687	-0.03191	0.063796	-0.50026	0.616891	NA
COG6	205.0159	0.031909	0.044146	0.722808	0.469798	NA
ITGB3	12.13501	-0.03191	0.081937	-0.38942	0.696966	NA
SCTR	0.301964	-0.03191	0.028583	-1.11627	0.264305	NA
FBXW12	8.604684	-0.0319	0.083887	-0.38033	0.703704	NA

SLBP	86.43873	0.031903	0.054432	0.586114	0.557799	NA
FLJ43681	0.714605	0.031889	0.042862	0.743987	0.456884	NA
HMGN1	69.40409	0.031887	0.076928	0.414501	0.678508	NA
BTG1	687.46	0.031878	0.048675	0.654905	0.512529	0.783485
GABPB2	132.8404	0.031875	0.064722	0.492493	0.622371	NA
SEMA4F	84.50641	0.031864	0.0608	0.52408	0.600223	NA
RAB27B	10.21857	0.03186	0.084346	0.377731	0.705631	NA
NCF1C	0.5173	-0.03185	0.040785	-0.78102	0.434792	NA
BVES	7.980423	0.031851	0.083112	0.38323	0.701549	NA
LOC38976	169.7897	-0.03185	0.063879	-0.49857	0.618082	NA
CD28	0.219138	-0.03185	0.025549	-1.24649	0.212583	NA
PHKB	255.3755	0.031838	0.04283	0.743371	0.457257	NA
ZFP30	129.1908	0.031836	0.049437	0.643977	0.51959	NA
AIG1	80.30634	0.031832	0.067611	0.470818	0.63777	NA
TRMT1L	118.9743	0.031828	0.057293	0.555541	0.578525	NA
LINC00640	0.502073	-0.03183	0.039285	-0.8102	0.417827	NA
CERS5	201.0314	-0.03181	0.063175	-0.50348	0.614625	NA
FAM65A	386.2954	0.031806	0.049507	0.642467	0.52057	0.786586
IRF4	0.24051	-0.0318	0.029525	-1.07708	0.281445	NA
OXA1L	65.58912	-0.0318	0.065797	-0.4833	0.628882	NA
CMAS	66.25583	0.031797	0.069127	0.459979	0.645531	NA
CBWD1	18.46732	-0.0318	0.083238	-0.38199	0.702472	NA
CYBA	6.674895	-0.03179	0.078503	-0.40502	0.685466	NA
CDK5RAP1	122.5223	0.031792	0.055618	0.571615	0.567583	NA
EGFR-AS1	2.74239	-0.03179	0.072511	-0.43842	0.66108	NA
ATPBD4-A	4.685065	0.031789	0.079102	0.401871	0.687779	NA
TCERG1	703.8993	-0.03179	0.051233	-0.62042	0.534984	0.796193
PRDM15	88.67146	0.031783	0.061336	0.518186	0.604329	NA
JAG2	65.0613	0.031782	0.074769	0.425066	0.670788	NA
COPS3	122.2625	0.031774	0.068427	0.46435	0.642397	NA
TEX14	32.25793	-0.03176	0.08352	-0.38027	0.703744	NA
LOC10013	0.505059	-0.03176	0.040576	-0.78271	0.433795	NA
TNFRSF17	2.830376	0.031759	0.053408	0.594645	0.55208	NA
LOC28569	20.17312	-0.03175	0.084756	-0.37463	0.707937	NA
DYNLL1	176.8965	0.031749	0.079373	0.399991	0.689163	NA
TMEM26	0.162909	0.031744	0.022892	1.386683	0.165539	NA
C14orf1	26.95172	-0.03174	0.080576	-0.39392	0.693643	NA
IGFL3	0.893856	-0.03174	0.051549	-0.61565	0.538125	NA
KCNG2	20.24113	0.031732	0.084892	0.373789	0.708561	NA
KCNRG	30.86103	-0.03173	0.077214	-0.4109	0.681147	NA
ZBTB46	345.9676	0.031722	0.059874	0.529809	0.596244	0.829672
TREH	16.0233	-0.03172	0.084218	-0.37664	0.706444	NA
OPRM1	121.811	0.031717	0.083921	0.377935	0.705479	NA
CELP	0.24481	0.031716	0.029519	1.074439	0.282626	NA
PRKCSH	298.6377	-0.03171	0.055555	-0.57087	0.568088	NA
CYP1B1	14.64496	-0.03169	0.078207	-0.40526	0.685284	NA
THADA	148.4642	0.031693	0.062099	0.510371	0.609792	NA

TPRG1L	106.3738	0.031691	0.062795	0.504664	0.613795	NA
TP53I3	28.96355	-0.03169	0.079668	-0.39777	0.690799	NA
NME8	0.266225	-0.03169	0.031453	-1.00749	0.313699	NA
MIR4479	0.50893	-0.03166	0.038165	-0.82965	0.406738	NA
PTP4A2	314.0771	-0.03166	0.042099	-0.75207	0.452011	NA
PPM1B	598.9982	-0.03166	0.039748	-0.7965	0.425744	0.729432
USP25	403.7084	-0.03165	0.036453	-0.86835	0.385204	0.70023
FDX1	64.3988	0.031653	0.061367	0.515796	0.605997	NA
CENPH	5.082252	-0.03165	0.07976	-0.39685	0.691479	NA
THEMIS	0.680502	0.03165	0.039768	0.795873	0.426106	NA
SPOCK2	1725.109	0.031648	0.052417	0.603771	0.545996	0.803341
RPA4	0.600964	-0.03165	0.03887	-0.81419	0.415534	NA
NR6A1	25.0308	0.031626	0.079553	0.397539	0.69097	NA
LRRC40	94.38171	-0.03163	0.060506	-0.52267	0.601201	NA
HIST1H1T	0.398845	0.031622	0.031515	1.003401	0.315668	NA
ALPK1	11.92192	-0.03162	0.084759	-0.37307	0.709094	NA
CAMK1G	5.307106	-0.03162	0.073531	-0.42996	0.667225	NA
WDR27	81.14918	-0.03161	0.065895	-0.47968	0.631452	NA
LOC33966	16.30258	-0.03161	0.084287	-0.37501	0.707651	NA
OPN1SW	1.969241	-0.03161	0.060073	-0.52614	0.598789	NA
AMN	1.781414	0.031603	0.062901	0.502425	0.615368	NA
PTGR1	112.2665	-0.0316	0.055061	-0.57388	0.566048	NA
MCTS1	241.0975	-0.0316	0.05151	-0.61342	0.539599	NA
GTF3C3	111.7432	0.031594	0.053321	0.592531	0.553495	NA
MOB3C	140.6282	-0.03159	0.075435	-0.4188	0.675365	NA
ELAC2	197.7835	-0.03159	0.041858	-0.75469	0.450435	NA
ARHGEF39	20.16877	0.031584	0.080677	0.39149	0.695435	NA
ALKBH1	35.64735	0.031584	0.068567	0.460628	0.645066	NA
BECN1	115.9583	-0.03158	0.063249	-0.49932	0.617556	NA
RPL3	1218.383	-0.03157	0.072986	-0.43258	0.665318	0.85817
AMN1	57.31752	-0.03156	0.062789	-0.50266	0.615205	NA
ABCF3	192.6004	-0.03156	0.046679	-0.67613	0.498959	NA
TRUB1	56.53313	0.03156	0.072207	0.437079	0.662054	NA
ZNF135	110.6719	-0.03156	0.079504	-0.39696	0.6914	NA
SFTPC	0.959101	0.031556	0.051101	0.617531	0.536885	NA
MIR1539	0.137715	-0.03156	0.021406	-1.47415	0.140442	NA
GAD2	66.74567	-0.03155	0.081236	-0.38842	0.697706	NA
IFI27L1	11.11168	-0.03155	0.083614	-0.37733	0.705925	NA
LPPR5	3.136238	-0.03155	0.068134	-0.46304	0.643338	NA
DTX3	200.5052	0.031546	0.048345	0.65253	0.514059	NA
PIK3C2A	866.9048	-0.03155	0.043252	-0.72936	0.465782	0.755844
KIAA1462	101.1289	-0.03154	0.081382	-0.3876	0.698316	NA
PHF14	576.3519	0.031538	0.032621	0.966816	0.333636	0.660832
DHX35	70.08467	-0.03154	0.067941	-0.46416	0.642532	NA
MIR215	0.46126	0.031535	0.038565	0.81769	0.413534	NA
BLOC1S6	154.5264	-0.03153	0.056599	-0.5571	0.577458	NA
TERF2IP	769.7586	0.031531	0.059581	0.529216	0.596655	0.829672

RAB5B	410.0239	0.031524	0.040195	0.784284	0.432873	0.736097
CPSF7	248.1785	-0.03152	0.039751	-0.79301	0.427774	NA
TUBAL3	0.505428	-0.0315	0.036953	-0.85255	0.393912	NA
EGFLAM	2.338863	0.031497	0.070652	0.445809	0.655735	NA
NBPF9	196.7916	0.031494	0.076691	0.410655	0.681325	NA
LMOD3	2.08892	0.031472	0.069072	0.455636	0.648651	NA
S100A14	0.482379	-0.03147	0.034516	-0.91178	0.361884	NA
DNMT3A	471.58	-0.03147	0.054827	-0.57391	0.566029	0.814521
LOC73015	0.221587	-0.03147	0.027784	-1.13249	0.257429	NA
HPGDS	2.34232	-0.03145	0.069419	-0.45306	0.650502	NA
ZNF790-AS	34.68943	-0.03145	0.070294	-0.44742	0.654573	NA
WDR46	70.07785	-0.03145	0.062623	-0.50221	0.615521	NA
RGS7BP	213.6189	0.031449	0.076968	0.408599	0.682834	NA
PRSS53	40.29222	-0.03145	0.077904	-0.40368	0.686446	NA
KIAA0319	218.6984	-0.03144	0.071414	-0.44031	0.659711	NA
FCGR2C	2.843156	0.031444	0.0693	0.453742	0.650015	NA
PKD1L1	38.46732	-0.03144	0.081891	-0.38393	0.701029	NA
MED4	109.894	-0.03144	0.051993	-0.60468	0.545389	NA
AP1G1	398.4353	0.031438	0.034671	0.906765	0.364531	0.68748
CAPN1	161.3332	0.031425	0.043017	0.730526	0.465069	NA
SLC25A47	0.224093	-0.03142	0.026675	-1.17801	0.238794	NA
RDM1	0.433571	0.031419	0.030246	1.038782	0.298906	NA
OR2G2	0.414362	0.031417	0.032682	0.961277	0.336413	NA
CYB5D1	182.2128	0.031415	0.046895	0.669899	0.502922	NA
LOC34459	50.39724	-0.03141	0.077569	-0.40495	0.685514	NA
SMO	25.56666	-0.03141	0.083184	-0.37758	0.70574	NA
PIN4P1	15.75632	0.031408	0.082651	0.380013	0.703936	NA
TUBBP5	0.981972	-0.0314	0.046527	-0.67487	0.499756	NA
SNAP23	107.3729	-0.0314	0.07155	-0.43885	0.660772	NA
SERGEF	167.5982	0.031397	0.056075	0.559917	0.575536	NA
FUBP3	174.0521	-0.03139	0.040824	-0.76899	0.441902	NA
TRAF1	46.50413	-0.03139	0.077493	-0.40509	0.685415	NA
NRCAM	977.6441	-0.03139	0.049859	-0.62956	0.528982	0.792854
TMEM57	355.5141	-0.03138	0.036796	-0.85272	0.393815	0.705623
TMEM30C	0.424372	0.031372	0.035844	0.87526	0.381432	NA
TUG1	688.9145	-0.03137	0.036273	-0.86488	0.387105	0.700314
PIP5K1A	201.0722	0.031372	0.040368	0.777133	0.43708	NA
ID2	54.91598	-0.03137	0.082014	-0.38251	0.702086	NA
MLST8	130.67	0.031368	0.058929	0.532303	0.594516	NA
MAP3K12	478.5323	0.03136	0.046699	0.671522	0.501888	0.778081
RAPGEF4	516.0377	0.031359	0.046206	0.678695	0.497331	0.776233
MIR4689	0.239546	-0.03135	0.024022	-1.30511	0.191855	NA
LOC10013	2.222933	0.03135	0.070201	0.446577	0.65518	NA
ZNF684	20.08455	-0.03135	0.076955	-0.40732	0.683773	NA
ENG	74.57921	-0.03134	0.077588	-0.40393	0.686261	NA
LOC41505	11.5054	-0.03134	0.084415	-0.37123	0.710466	NA
FBLN1	36.20878	-0.03133	0.08403	-0.37287	0.709248	NA

SDSL	1.814976	0.031329	0.060362	0.51902	0.603747	NA
TARBP2	51.56913	-0.03132	0.064137	-0.48834	0.625312	NA
STYXL1	29.54244	-0.03132	0.084666	-0.3699	0.711459	NA
EPHX1	99.83513	0.031317	0.07531	0.415845	0.677523	NA
PARVA	91.27884	0.031317	0.080968	0.386785	0.698915	NA
MFAP2	0.55374	0.031312	0.042322	0.739852	0.45939	NA
ZNF177	1.438007	-0.0313	0.058472	-0.53539	0.592381	NA
SLC46A3	62.6433	0.031303	0.061552	0.508556	0.611063	NA
VWCE	13.84369	-0.0313	0.082725	-0.37838	0.705151	NA
HIGD2A	25.3116	-0.0313	0.075005	-0.41731	0.676453	NA
GATA6	0.300419	0.031292	0.03191	0.980652	0.326764	NA
FUNDC1	34.07763	-0.03129	0.07654	-0.40883	0.682663	NA
LOC64939	0.814537	-0.03129	0.047642	-0.65676	0.511334	NA
SNRNP27	223.4417	-0.03128	0.051739	-0.60464	0.545417	NA
HOXD3	0.135152	-0.03128	0.021394	-1.46207	0.143722	NA
LOC10013	0.575133	-0.03126	0.04331	-0.72188	0.470365	NA
CDK2AP2	45.67838	0.031258	0.082247	0.380054	0.703906	NA
C4orf3	136.9355	0.031251	0.0647	0.483011	0.629088	NA
LOC64340	2.116791	0.031251	0.06503	0.480558	0.630831	NA
RTDR1	2.477736	-0.03124	0.071641	-0.43611	0.66276	NA
MROH8	32.45658	-0.03124	0.073122	-0.42717	0.669257	NA
FFAR1	0.140943	-0.03123	0.021517	-1.45153	0.146632	NA
KNDC1	615.7029	0.031232	0.056473	0.553044	0.580233	0.822777
SNORA66	8.635275	-0.03123	0.082245	-0.3797	0.704165	NA
TREML1	2.405697	0.031219	0.0699	0.446625	0.655146	NA
YDJC	18.86126	0.031214	0.077912	0.400629	0.688693	NA
IFT81	162.0207	0.031211	0.045226	0.690104	0.490129	NA
ROPN1L	1.047908	0.03121	0.053277	0.585811	0.558002	NA
ECI1	47.12362	0.03121	0.066427	0.469836	0.638472	NA
YES1	77.37781	-0.0312	0.07093	-0.43984	0.660051	NA
FBXW8	93.10805	0.031196	0.062474	0.49934	0.61754	NA
ARV1	72.36718	-0.0312	0.067168	-0.46444	0.642333	NA
CERKL	205.0706	0.031188	0.053732	0.580433	0.561622	NA
TMEM100	3.485535	-0.03119	0.069999	-0.44551	0.655954	NA
METTL10	179.7906	-0.03118	0.04675	-0.66705	0.50474	NA
PKD1L2	2.578939	0.031178	0.068529	0.45496	0.649138	NA
LOC10012	5.729926	0.031169	0.084154	0.370381	0.711099	NA
AK3	111.4717	-0.03116	0.053522	-0.58219	0.56044	NA
KIAA1324L	355.1532	0.031158	0.036881	0.844828	0.398207	0.706622
RGPD1	2.308965	-0.03115	0.067532	-0.4613	0.644583	NA
GLP1R	0.534563	0.031151	0.040034	0.77811	0.436504	NA
ZNRF3	165.6559	0.031147	0.053801	0.578936	0.562633	NA
IGFBP6	4.871465	0.031125	0.078708	0.395448	0.692512	NA
LOC10050	47.02571	0.031123	0.063971	0.486525	0.626595	NA
LOC34001	0.474677	0.031121	0.038024	0.818452	0.413099	NA
YBX1	318.3997	0.031119	0.055377	0.561946	0.574153	NA
HDGFRP2	225.5543	-0.03111	0.040059	-0.77664	0.437374	NA

C11orf16	3.085178	0.03111	0.074948	0.415083	0.678081	NA
C1QTNF9B	0.583048	0.031089	0.041713	0.745312	0.456083	NA
DBIL5P2	4.728898	-0.03109	0.081137	-0.38315	0.701611	NA
PGAP1	415.1939	0.031086	0.052934	0.587254	0.557033	0.808857
STS	74.76952	-0.03108	0.082072	-0.37869	0.704915	NA
TNFRSF8	0.155231	-0.03108	0.023379	-1.32932	0.183743	NA
MED10	45.10115	0.031074	0.065723	0.4728	0.636356	NA
SPAG9	1437.51	-0.03107	0.027095	-1.14677	0.251477	0.592704
CELA2A	8.628572	-0.03107	0.084229	-0.36887	0.712223	NA
UQCRB	583.7995	-0.03107	0.059713	-0.52029	0.602864	0.830918
FAM205B	0.81837	-0.03106	0.047627	-0.6522	0.514269	NA
GNAQ	211.8388	-0.03106	0.054294	-0.57212	0.567239	NA
CCSER1	345.4204	0.031059	0.053096	0.584959	0.558575	0.808857
NDUFB8	237.2268	-0.03106	0.055529	-0.55927	0.575979	NA
GOLGA6L1	3.289858	0.031039	0.073639	0.421508	0.673384	NA
LCMT1	89.1449	-0.03104	0.062444	-0.49706	0.61915	NA
SLC52A2	47.64089	-0.03101	0.074833	-0.41441	0.678576	NA
SHC3	62.60631	-0.031	0.083892	-0.36957	0.711703	NA
EPS8	130.0589	-0.031	0.056833	-0.54539	0.585484	NA
CFDP1	226.4429	0.030993	0.056358	0.549925	0.582371	NA
BZW2	28.8826	-0.03099	0.073553	-0.42136	0.673491	NA
LINC00239	0.257385	-0.03097	0.028386	-1.0911	0.275229	NA
ZNF330	132.2298	0.030967	0.059716	0.518567	0.604063	NA
C7orf71	5.003442	0.030958	0.080875	0.382787	0.701878	NA
DKFZP434I	51.32186	0.030956	0.064832	0.477478	0.633021	NA
WDR16	1.088118	0.030954	0.055156	0.561209	0.574655	NA
LRIG2	149.2615	0.030952	0.049719	0.622539	0.533587	NA
C12orf45	17.04216	-0.03094	0.083178	-0.372	0.709893	NA
FAM65C	17.26312	0.030936	0.083051	0.372496	0.709524	NA
ARHGEF4	180.0697	-0.03093	0.058196	-0.53146	0.595098	NA
TMCO6	44.11565	-0.03093	0.076458	-0.40448	0.685857	NA
SLC1A6	183.1718	-0.03092	0.084696	-0.36509	0.715044	NA
SLC2A8	47.99	0.030913	0.071828	0.430367	0.666928	NA
TMEM14A	49.78105	-0.03091	0.078741	-0.39257	0.694634	NA
SIGLEC5	0.967962	-0.03091	0.049181	-0.62849	0.529682	NA
JPH2	1.214209	0.030907	0.051927	0.595201	0.551709	NA
PDE3B	420.4624	0.030907	0.043587	0.70909	0.478269	0.760195
SOGA2	3326.901	-0.03089	0.045173	-0.68383	0.494081	0.771788
CTNNAL1	48.70815	-0.03089	0.071455	-0.43228	0.665539	NA
NR5A2	0.964736	0.030888	0.048419	0.637934	0.523516	NA
LINC00551	0.587347	0.030885	0.040167	0.768919	0.441941	NA
ID1	13.70175	0.030882	0.084929	0.363627	0.716136	NA
ZKSCAN2	91.35361	0.030874	0.064525	0.478488	0.632303	NA
EFCAB14-A	2.92421	-0.03087	0.073759	-0.41858	0.675524	NA
NOL8	192.1783	-0.03087	0.044171	-0.69893	0.484596	NA
CEP89	132.14	0.030868	0.067578	0.456773	0.647834	NA
SHROOM1	73.09175	-0.03087	0.060362	-0.51134	0.60911	NA

EFTUD2	148.3178	-0.03087	0.04457	-0.69251	0.488616	NA
TMEM50A	93.46799	-0.03086	0.065034	-0.4745	0.635141	NA
ZNF426	77.49823	0.030856	0.057219	0.539272	0.589699	NA
SNRNP35	55.46323	-0.03085	0.059657	-0.51716	0.605041	NA
PFN1	78.51947	-0.03085	0.072927	-0.42305	0.672255	NA
ASB4	1.567463	-0.03084	0.056562	-0.54516	0.585645	NA
BAZ1A	70.36083	-0.03084	0.078579	-0.39241	0.694753	NA
FRS3	53.47721	-0.03083	0.07315	-0.4215	0.673391	NA
ATP9B	226.6142	-0.03083	0.050345	-0.61229	0.540344	NA
HIGD1B	6.795697	-0.03082	0.08412	-0.36639	0.714074	NA
PELI2	341.3837	-0.03081	0.041261	-0.74679	0.45519	0.750995
FGFR4	17.50104	0.030806	0.084328	0.365315	0.714876	NA
HIST1H4K	0.661348	0.030795	0.04505	0.683558	0.494254	NA
SYAP1	111.1898	0.030791	0.061327	0.50209	0.615604	NA
TEX21P	6.564003	-0.03078	0.083667	-0.36793	0.712926	NA
TMEM106	16.37277	-0.03077	0.084826	-0.36273	0.716805	NA
CER1	3.392165	-0.03077	0.071119	-0.43264	0.665274	NA
TENM1	1373.925	0.030768	0.04766	0.645574	0.518556	0.785288
HNRNPF	245.731	0.030767	0.047597	0.646399	0.518021	NA
CERS2	98.94628	-0.03077	0.057694	-0.53325	0.593861	NA
ALDH1L1-	25.03801	0.030763	0.084266	0.36507	0.715059	NA
SNORA19	3.060806	-0.03076	0.076831	-0.40034	0.688907	NA
RBPMS	58.62993	-0.03076	0.066269	-0.4641	0.642575	NA
ZNF221	169.7604	0.03075	0.058259	0.527819	0.597625	NA
CCNG1	68.05245	-0.03075	0.064477	-0.4769	0.633434	NA
IFNA21	1.091999	0.030746	0.047264	0.650513	0.515361	NA
CHCHD3	130.5711	0.030746	0.045725	0.672411	0.501322	NA
SLC16A14	88.27232	0.030743	0.07114	0.432151	0.665632	NA
GPSM1	291.3537	-0.03073	0.060823	-0.50531	0.613339	NA
BICC1	2.543572	-0.03073	0.072449	-0.42422	0.671408	NA
CUL2	153.9735	0.030727	0.052094	0.589832	0.555303	NA
TOR1AIP2	364.9807	0.030721	0.046211	0.664807	0.506174	0.780812
PSG10P	12.86877	0.030721	0.084869	0.36198	0.717367	NA
CR1	1.744206	0.030719	0.055512	0.553386	0.579999	NA
ZDHHC22	313.9377	-0.03072	0.067476	-0.4552	0.648964	NA
PRPF40A	534.1827	0.030705	0.043416	0.707244	0.479415	0.760949
SCARNA14	0.873032	-0.0307	0.05272	-0.5823	0.560363	NA
CHPT1	107.6058	0.030693	0.053754	0.570999	0.568	NA
LOC28319	0.12909	-0.03069	0.021313	-1.44011	0.149837	NA
KCNV2	17.56678	-0.03069	0.084489	-0.36325	0.716418	NA
HCFC1R1	31.34639	-0.03068	0.075882	-0.40438	0.685935	NA
ATP6V1E1	206.721	0.030681	0.072373	0.423935	0.671614	NA
HEATR2	75.34783	0.030678	0.079518	0.3858	0.699645	NA
MFSD6	537.1577	0.030677	0.04287	0.715574	0.474255	0.759174
SPATA22	1.365358	-0.03067	0.052048	-0.58933	0.555643	NA
ZNF688	48.3544	0.030673	0.067096	0.45715	0.647563	NA
BABAM1	163.4418	-0.03066	0.049732	-0.61656	0.537528	NA

ZNF19	15.91723	0.030659	0.084813	0.361489	0.717734	NA
ADAMTS1	59.92029	-0.03065	0.074888	-0.40933	0.682297	NA
OR51M1	0.588793	0.030648	0.043761	0.700353	0.483707	NA
MIR369	0.353657	0.030647	0.033446	0.916314	0.359502	NA
CCZ1	24.99017	-0.03065	0.084059	-0.36457	0.71543	NA
LOC44051	0.123867	-0.03064	0.021182	-1.4466	0.148008	NA
DICER1-AS	19.18769	-0.03064	0.080634	-0.37998	0.703962	NA
USP30	81.28196	0.030637	0.048026	0.637912	0.523531	NA
ZNF3	131.3535	0.030635	0.050618	0.605219	0.545034	NA
CCNI	968.5056	0.03063	0.068092	0.449835	0.652829	0.853604
L3MBTL3	19.59308	0.03063	0.078399	0.39069	0.696027	NA
SORT1	300.5157	-0.03063	0.059734	-0.51272	0.608148	NA
FAM211B	19.03208	0.030625	0.078824	0.388527	0.697626	NA
LOC10013	4.886595	-0.03062	0.075465	-0.40575	0.68493	NA
COL3A1	3.009527	0.030615	0.064911	0.471655	0.637173	NA
MUSTN1	4.955803	-0.03061	0.080764	-0.37907	0.704639	NA
FHDC1	54.42316	-0.03061	0.081759	-0.37445	0.708068	NA
MIR485	0.460605	0.03061	0.037013	0.827029	0.40822	NA
MYH9	833.4981	-0.03061	0.06577	-0.4654	0.641646	0.848776
AMMECR1	50.93051	0.030609	0.070287	0.435489	0.663208	NA
TAS2R5	23.92548	-0.03061	0.0832	-0.36789	0.712956	NA
TMEM240	37.52963	-0.0306	0.07062	-0.43337	0.664749	NA
EBI3	0.505284	-0.0306	0.036731	-0.83318	0.404744	NA
EYS	132.1309	-0.0306	0.065565	-0.46674	0.640687	NA
POM121	162.7995	0.030601	0.049384	0.619659	0.535482	NA
HCG4	7.044858	0.030601	0.076253	0.401308	0.688193	NA
MGEA5	2003.452	-0.0306	0.039045	-0.78373	0.433201	0.736097
RAB9A	46.2853	-0.03058	0.063777	-0.47953	0.631564	NA
AP3S1	89.87549	-0.03057	0.049876	-0.61296	0.539905	NA
SKP1P2	0.76049	-0.03056	0.049294	-0.61989	0.53533	NA
SPDYC	0.361175	0.030556	0.033437	0.91385	0.360796	NA
TMED4	202.8409	-0.03053	0.043312	-0.70499	0.480818	NA
FBLN7	47.12833	0.030531	0.083363	0.366246	0.714182	NA
OSGEPL1	56.2312	-0.03051	0.061199	-0.49854	0.618106	NA
NCALD	23.76326	0.030509	0.082367	0.370405	0.71108	NA
FAM22G	12.04291	0.030502	0.083749	0.364203	0.715707	NA
ATXN2	615.0292	0.030492	0.032485	0.938642	0.347914	0.673527
ZNF747	42.0995	0.03049	0.064019	0.476269	0.633883	NA
LIPE	54.86474	-0.03049	0.059157	-0.5154	0.606273	NA
ANAPC2	108.1929	-0.03049	0.050487	-0.60387	0.545932	NA
OR13A1	1.25207	-0.03048	0.055531	-0.54886	0.583098	NA
SYPL1	34.18277	-0.03048	0.071997	-0.42333	0.672057	NA
ARHGEF35	0.189409	0.030475	0.022625	1.346964	0.177992	NA
MIR3939	0.848921	-0.03047	0.051463	-0.59208	0.553795	NA
HDAC4	286.4115	0.030464	0.046691	0.652474	0.514095	NA
TBC1D8B	4.381381	0.030464	0.081	0.376093	0.706848	NA
TMC6	34.82013	-0.03046	0.081892	-0.37199	0.7099	NA

SF3B4	37.80578	-0.03046	0.069963	-0.43539	0.663279	NA
VGLL2	0.787794	0.030455	0.042172	0.722167	0.470192	NA
CKS1B	1.761449	0.030449	0.068191	0.446525	0.655218	NA
KIF3C	667.026	0.030449	0.046849	0.649937	0.515733	0.785288
SLC6A17	290.1209	0.03044	0.07102	0.428613	0.668205	NA
LTA	1.488666	0.03044	0.058838	0.517343	0.604917	NA
FA2H	15.46389	-0.03044	0.084706	-0.35932	0.719356	NA
RHOXF1	8.034154	-0.03042	0.080805	-0.37647	0.706571	NA
PFKFB4	38.93203	0.030402	0.072814	0.417523	0.676296	NA
LINC00338	11.69339	-0.03038	0.084888	-0.35791	0.720411	NA
TIFA	58.75669	-0.03038	0.068834	-0.44137	0.658945	NA
GNN	7.333194	0.030379	0.084687	0.358715	0.719809	NA
SERINC4	123.5551	-0.03038	0.075952	-0.39993	0.689208	NA
UBXN2A	73.16417	-0.03037	0.064342	-0.47204	0.636898	NA
E2F4	140.6533	0.030362	0.055994	0.542239	0.587654	NA
UFM1	190.2716	-0.03036	0.043957	-0.69066	0.489776	NA
LAMTOR1	48.30841	-0.03035	0.069982	-0.43365	0.664542	NA
FEN1	54.5701	0.030345	0.068738	0.441465	0.658876	NA
RCSD1	8.947253	-0.03034	0.083452	-0.36357	0.716181	NA
RIPK1	124.0246	-0.03033	0.058753	-0.51622	0.605699	NA
LINC00240	0.658188	-0.03032	0.04586	-0.6611	0.508548	NA
CYP4B1	0.700046	-0.03032	0.042438	-0.71439	0.474988	NA
ZNF548	123.7303	0.030314	0.05206	0.582283	0.560376	NA
MIR186	1.078317	-0.03029	0.055162	-0.54917	0.58289	NA
LOC39974	16.56801	0.030289	0.082688	0.366307	0.714136	NA
C1orf100	0.529196	-0.03029	0.04049	-0.74805	0.45443	NA
FAM73B	132.9889	-0.03028	0.052985	-0.57151	0.567654	NA
MRGBP	33.54032	0.030276	0.070301	0.430666	0.666711	NA
ANKRD32	146.337	-0.03027	0.051271	-0.59032	0.554977	NA
CALHM2	11.74515	0.030263	0.083879	0.360793	0.718254	NA
RAB11A	384.6569	0.03025	0.046239	0.654198	0.512984	0.783485
CAMKV	18.42853	-0.03023	0.08474	-0.35676	0.721269	NA
MFF	160.343	0.030226	0.049528	0.61028	0.541677	NA
AGO4	272.8936	-0.03022	0.038489	-0.78508	0.432408	NA
FSCN3	4.287021	0.030211	0.072411	0.417214	0.676522	NA
COX16	18.82412	-0.03021	0.077844	-0.38804	0.697984	NA
MYSM1	413.5325	-0.03021	0.048226	-0.62634	0.531094	0.794012
C11orf42	16.61421	0.030205	0.082393	0.366591	0.713924	NA
C1orf94	0.639602	-0.0302	0.04364	-0.69201	0.488933	NA
ZNF419	49.4165	-0.03019	0.064386	-0.46896	0.639101	NA
NAA25	310.046	-0.03019	0.042897	-0.70369	0.481626	NA
RASSF9	0.59093	0.030174	0.04254	0.709306	0.478134	NA
ZNF461	93.62925	-0.03017	0.058891	-0.5123	0.608444	NA
TNFRSF10	1.834556	-0.03017	0.068009	-0.44359	0.657337	NA
LOC28373	0.754525	-0.03017	0.048895	-0.61696	0.537264	NA
DHX36	270.1226	-0.03015	0.051523	-0.58519	0.558419	NA
KREMEN2	2.159389	-0.03015	0.068685	-0.43893	0.660712	NA

TCF7L1	60.06739	-0.03014	0.080115	-0.37625	0.70673	NA
SCO2	1.05382	-0.03014	0.048888	-0.61652	0.537552	NA
ADCK2	31.63436	0.030138	0.068888	0.43749	0.661756	NA
TNKS	690.5811	0.030136	0.029772	1.012219	0.311433	0.641853
UBE2Z	159.0103	0.030134	0.044006	0.684761	0.493495	NA
OTC	0.153992	0.030131	0.022919	1.314673	0.18862	NA
POLR1C	29.8465	0.030128	0.078323	0.384668	0.700484	NA
SCAI	255.9534	-0.03012	0.045039	-0.66882	0.503607	NA
SGK223	493.725	0.03012	0.049691	0.606143	0.54442	0.803213
SLAMF8	20.95758	-0.03011	0.082967	-0.36294	0.716647	NA
DFFB	64.88842	0.030111	0.067248	0.447766	0.654322	NA
C19orf43	181.5606	-0.03011	0.060569	-0.4971	0.619116	NA
COL18A1-	2.011958	-0.0301	0.061896	-0.48636	0.626715	NA
AARD	0.44835	-0.0301	0.036254	-0.83026	0.406394	NA
ARF1	381.4106	0.030096	0.054063	0.556677	0.577748	0.821469
PHF19	75.74037	-0.0301	0.065984	-0.4561	0.64832	NA
MAGI1-AS	36.30049	-0.03009	0.074131	-0.40584	0.684861	NA
HIST1H4L	0.450417	-0.03008	0.032362	-0.92949	0.352636	NA
ANKDD1B	4.968087	0.030079	0.081038	0.371176	0.710507	NA
ST8SIA2	2.600773	-0.03007	0.068525	-0.43884	0.660778	NA
SRPR	154.2993	0.030069	0.046028	0.653263	0.513587	NA
TTLL4	91.60905	0.030063	0.063258	0.47525	0.634609	NA
SOCS3	19.44217	-0.03004	0.064371	-0.46671	0.640708	NA
PLEKHN1	0.358758	-0.03004	0.032456	-0.92559	0.354658	NA
OR1F2P	3.137432	0.030034	0.073432	0.409009	0.682533	NA
ZNF83	700.9486	-0.03003	0.065453	-0.45877	0.646402	0.849041
ADM2	0.999441	-0.03	0.051676	-0.5806	0.561507	NA
DCHS2	20.45797	-0.03	0.084752	-0.35393	0.723393	NA
APCDD1L	6.900844	0.029996	0.081934	0.3661	0.714291	NA
UCKL1-AS1	67.21436	-0.02999	0.082676	-0.36279	0.716764	NA
SRP19	198.1715	0.029993	0.067417	0.444883	0.656404	NA
PMS1	167.8334	0.029988	0.046024	0.651571	0.514678	NA
MIR4449	0.766217	-0.02999	0.046171	-0.64943	0.516057	NA
PCYT2	103.5283	-0.02998	0.063286	-0.47371	0.635708	NA
EPPIN-WF	0.274099	0.029974	0.029115	1.029475	0.303256	NA
LRRC36	3.273481	-0.02997	0.075584	-0.39656	0.691695	NA
UBR2	725.1026	-0.02997	0.052394	-0.57201	0.567316	0.814844
TIE1	18.42098	-0.02997	0.082165	-0.36472	0.715318	NA
DCTN2	196.3308	-0.02997	0.065234	-0.45936	0.645979	NA
SEC14L6	3.872832	-0.02996	0.075749	-0.39547	0.692494	NA
SNORA16B	0.945357	-0.02995	0.049619	-0.60353	0.546157	NA
CARHSP1	45.16433	-0.02994	0.071206	-0.4205	0.67412	NA
CLEC10A	0.270773	0.029941	0.031913	0.9382	0.348142	NA
POP7	40.3356	0.029937	0.078628	0.380745	0.703393	NA
DNM2	325.3601	-0.02993	0.046897	-0.63825	0.523311	NA
SLAMF7	0.261048	0.029922	0.026947	1.110396	0.266828	NA
CLIC3	1.715269	0.029917	0.064334	0.465025	0.641914	NA

RBM47	3.61334	-0.02991	0.072542	-0.41237	0.680065	NA
SIPA1L1	765.1723	0.029904	0.057362	0.521321	0.602143	0.830918
TEX101	2.446782	0.029893	0.061751	0.484087	0.628324	NA
SNRPA	69.37801	-0.02989	0.071522	-0.41795	0.675983	NA
C1QTNF3	34.49288	0.029889	0.077205	0.387142	0.698651	NA
PMVK	42.63703	0.029887	0.070593	0.423368	0.672027	NA
ZNF818P	19.14168	0.029886	0.084098	0.355372	0.722311	NA
THAP6	67.36529	-0.02988	0.066364	-0.45025	0.65253	NA
LOC44145	20.74827	0.029878	0.077587	0.385096	0.700167	NA
UPP2	7.556971	-0.02988	0.084851	-0.3521	0.724767	NA
KHDRBS2	6.300909	0.029864	0.080035	0.37314	0.709044	NA
RNF115	292.3906	-0.02986	0.04614	-0.64723	0.517486	NA
ZFYVE19	70.39006	0.029844	0.066865	0.446329	0.65536	NA
S100Z	0.389497	-0.02984	0.035657	-0.83689	0.402657	NA
ACAP2	455.8458	0.029839	0.041949	0.711325	0.476883	0.759174
HLA-DQA2	0.320353	-0.02983	0.026331	-1.13294	0.257239	NA
TRIL	118.2382	-0.02983	0.083062	-0.35909	0.719528	NA
FIG4	86.43124	0.02982	0.049774	0.599096	0.549109	NA
ZNF664-FA	1.235537	0.029807	0.05604	0.531887	0.594804	NA
MEF2BNB	21.74146	0.029801	0.079329	0.375664	0.707167	NA
MPC1L	0.432148	-0.0298	0.039667	-0.75123	0.452514	NA
C1QL4	0.426002	0.029797	0.03279	0.908736	0.363489	NA
AXIN1	193.7112	0.029796	0.059887	0.497548	0.618803	NA
TSN	147.799	0.029792	0.047048	0.633231	0.526583	NA
FAM181A-	1.599321	0.029791	0.061023	0.48819	0.625415	NA
GPATCH2	67.15726	0.029787	0.063435	0.469567	0.638664	NA
ZNF512	213.6569	0.029782	0.043164	0.689971	0.490213	NA
SETD9	4.776625	0.029779	0.082471	0.361087	0.718035	NA
PCMTD1	275.524	-0.02978	0.042095	-0.70739	0.479322	NA
USF2	406.044	-0.02977	0.037335	-0.79746	0.425186	0.729029
SPATS2	178.7632	-0.02977	0.044758	-0.66511	0.505979	NA
NHLRC2	243.642	0.029762	0.042928	0.693298	0.488122	NA
MYLK-AS1	4.951397	-0.02976	0.082008	-0.36289	0.716687	NA
NT5C1A	30.57614	-0.02976	0.080485	-0.36973	0.711584	NA
UBE2Q2P1	24.57967	0.02975	0.082055	0.362566	0.716929	NA
MAML2	461.161	-0.02975	0.045453	-0.65446	0.512817	0.783485
VWA1	25.75623	0.029746	0.084166	0.353419	0.723774	NA
AKIRIN1	105.4377	-0.02974	0.049561	-0.60012	0.548428	NA
TUBGCP6	565.0659	-0.02974	0.057684	-0.51549	0.60621	0.831231
EIF2S1	164.0468	0.029734	0.055538	0.535388	0.592381	NA
CEACAM8	3.733831	-0.02973	0.073415	-0.40492	0.685537	NA
WHAMM	143.2161	0.02972	0.043291	0.686518	0.492387	NA
SNX19	414.8205	0.029715	0.060593	0.490399	0.623852	0.841191
SOD2	266.1858	0.029692	0.070371	0.421931	0.673076	NA
LOC10065	61.88792	-0.02969	0.063752	-0.46564	0.641472	NA
ATG4D	45.749	0.029677	0.062716	0.473194	0.636075	NA
FAM131B	440.7924	0.029674	0.061228	0.484643	0.627929	0.84337

NPDC1	273.3138	0.029673	0.045547	0.651479	0.514737	NA
TMX4	363.4535	-0.02966	0.062373	-0.4756	0.634359	0.845687
SNORD113	10.76296	-0.02966	0.084937	-0.34923	0.726919	NA
GULP1	4.421003	0.02966	0.078632	0.377207	0.70602	NA
MTNR1A	7.035528	-0.02966	0.074444	-0.3984	0.690338	NA
LINC00398	3.133153	0.029649	0.07635	0.38833	0.697771	NA
DNAH8	1.249718	0.029648	0.059428	0.498881	0.617863	NA
SURF4	100.5008	-0.02965	0.051236	-0.57863	0.562837	NA
CPSF3	77.54592	-0.02965	0.065463	-0.45287	0.650645	NA
WWC2-AS	0.499526	-0.02964	0.037941	-0.78127	0.434642	NA
VPS29	100.3445	0.029641	0.061516	0.481849	0.629913	NA
KLK11	0.617973	-0.02964	0.04084	-0.72579	0.46797	NA
SIRT4	10.38977	-0.02964	0.084895	-0.34914	0.726985	NA
C14orf80	8.470116	0.029637	0.084915	0.349019	0.727075	NA
TEKT2	4.159615	0.029634	0.074649	0.396984	0.691379	NA
SHPK	108.5306	-0.02962	0.061241	-0.48368	0.628613	NA
SLC45A3	8.898285	0.029609	0.08311	0.35627	0.721638	NA
NR0B1	0.419361	-0.02961	0.03604	-0.82156	0.411328	NA
TMEM135	138.5413	0.029604	0.048357	0.612204	0.540403	NA
ZBTB7A	458.9228	0.029603	0.057758	0.512531	0.608279	0.832479
SPINK1	0.226264	0.029594	0.025381	1.165988	0.243619	NA
SHANK1	724.2261	-0.02959	0.052985	-0.55852	0.576488	0.821271
HACL1	118.0421	-0.02959	0.062566	-0.47299	0.636219	NA
CYB561D1	65.8627	-0.02959	0.0691	-0.42824	0.668474	NA
MXI1	564.54	-0.02959	0.045585	-0.64914	0.516246	0.785288
C12orf73	19.46018	0.029588	0.079814	0.370714	0.710851	NA
IL6ST	966.8262	0.029588	0.058088	0.509355	0.610503	0.834011
BRCC3	94.61665	0.02958	0.050291	0.588185	0.556408	NA
CHAC1	4.300633	-0.02957	0.082136	-0.36003	0.718827	NA
MIR1288	0.336362	-0.02957	0.032224	-0.91767	0.358792	NA
ATP6V0E2	236.1264	0.029569	0.05932	0.498469	0.618153	NA
NR2E1	0.512724	-0.02957	0.037191	-0.79505	0.426587	NA
CYP4F12	0.687179	-0.02957	0.040432	-0.73131	0.464587	NA
PNPT1	159.765	0.029566	0.061531	0.480503	0.63087	NA
LOC10030	2.516186	-0.02955	0.068721	-0.43006	0.667151	NA
MIR326	0.490861	-0.02955	0.037853	-0.78061	0.435034	NA
ACTA1	7.952398	-0.02955	0.084938	-0.34785	0.727951	NA
CLDN12	172.5067	0.029542	0.051686	0.571569	0.567614	NA
SLC6A13	8.6606	-0.02954	0.082615	-0.35757	0.720663	NA
IL16	1529.598	0.02954	0.052168	0.566258	0.571219	0.81719
SCP2	107.0222	0.029538	0.067885	0.435115	0.663479	NA
LYPD6B	0.720281	-0.02953	0.046055	-0.64127	0.521346	NA
C6orf62	214.7798	-0.02952	0.049295	-0.59892	0.549228	NA
MMP14	17.56125	-0.02952	0.084936	-0.34752	0.728204	NA
TP53INP2	162.9774	-0.02952	0.080083	-0.36857	0.712445	NA
MIR5692C	1.158266	0.029516	0.05414	0.545174	0.585634	NA
GPR26	1.401032	0.029502	0.058242	0.506538	0.612479	NA

C15orf39	8.298736	-0.0295	0.083128	-0.35489	0.722669	NA
ZNF557	104.3642	0.0295	0.051881	0.568604	0.569625	NA
ZBTB49	38.75705	0.029494	0.06616	0.4458	0.655742	NA
DDX11L9	0.152108	0.029487	0.022948	1.28492	0.19882	NA
CALML6	2.511123	-0.02949	0.071768	-0.41084	0.681187	NA
LINC00189	0.374424	-0.02948	0.035733	-0.82506	0.409339	NA
DDHD2	448.7594	0.029474	0.049092	0.600378	0.548254	0.803817
NFIX	1701.572	-0.02947	0.046099	-0.63917	0.522711	0.787214
MMRN2	35.44049	0.029465	0.084363	0.349259	0.726895	NA
TAF1L	2.052726	-0.02946	0.069553	-0.42362	0.671844	NA
KCNN2	67.33246	-0.02946	0.06862	-0.42936	0.667664	NA
KRT8P41	1.67343	-0.02946	0.061939	-0.47565	0.634326	NA
IL13RA2	0.377865	-0.02945	0.035688	-0.82509	0.40932	NA
MIR3074	0.383134	-0.02944	0.035409	-0.83153	0.405673	NA
PLK1	24.31278	-0.02943	0.080755	-0.36442	0.715548	NA
POU5F1P4	7.502722	0.029424	0.084606	0.34778	0.728005	NA
FAM120A	86.01409	0.029415	0.05396	0.545121	0.58567	NA
CTHRC1	34.22655	-0.02941	0.078473	-0.37474	0.707856	NA
MIR411	1.675211	0.029402	0.063267	0.464726	0.642127	NA
KRTAP5-6	0.209472	-0.0294	0.026694	-1.10137	0.270735	NA
VPREB3	0.322259	0.029397	0.033362	0.881141	0.378241	NA
EPG5	430.0183	-0.0294	0.039322	-0.74757	0.454718	0.750995
MIR4469	0.157643	0.029395	0.023046	1.275499	0.202133	NA
NUP54	121.6007	0.02939	0.059477	0.494143	0.621205	NA
KIAA0825	78.8657	-0.02937	0.070073	-0.41912	0.675127	NA
RRBP1	190.7861	-0.02937	0.056087	-0.5236	0.60056	NA
OR8S1	0.184686	-0.02936	0.024854	-1.1813	0.237485	NA
MROH1	237.2146	-0.02936	0.06532	-0.44941	0.653136	NA
ASPDH	33.67068	-0.02935	0.076746	-0.38243	0.702144	NA
MPP2	154.2971	0.029348	0.050981	0.575662	0.564843	NA
GPRASP2	10.13383	0.029341	0.084791	0.346041	0.729312	NA
LOC10050	2.585341	0.029341	0.07453	0.393673	0.693822	NA
FKBP8	497.1052	-0.02933	0.053396	-0.54933	0.582782	0.824332
SHMT1	69.03278	0.029331	0.062503	0.469275	0.638873	NA
EPHA5	50.87244	0.02932	0.084937	0.345195	0.729948	NA
SAMD15	11.9595	-0.02931	0.084939	-0.3451	0.730023	NA
LOC37444	4.398762	-0.02931	0.080938	-0.36214	0.717248	NA
PLGRKT	9.705402	0.029308	0.084842	0.345438	0.729765	NA
REEP6	15.31366	-0.02931	0.084868	-0.34533	0.729848	NA
ERVV-2	0.431097	-0.02931	0.034564	-0.84786	0.396514	NA
MIR875	0.097822	-0.0293	0.018151	-1.6144	0.106441	NA
ETV4	9.230729	0.029301	0.084731	0.345808	0.729487	NA
FAM117B	208.5601	0.029299	0.054542	0.537182	0.591142	NA
PIK3C3	436.0184	-0.02929	0.032644	-0.8974	0.369505	0.69397
ZSWIM8	347.9823	0.029283	0.055761	0.525151	0.599478	0.829672
STEAP3	66.49078	0.029275	0.076103	0.384671	0.700481	NA
CAAP1	71.59762	-0.02927	0.065689	-0.44558	0.655897	NA

MIR1249	0.493231	-0.02927	0.038124	-0.76773	0.442647	NA
IGSF22	16.30992	-0.02926	0.084771	-0.34521	0.729939	NA
PUM1	573.4913	0.029262	0.032808	0.891904	0.372445	0.695376
NTN4	8.73131	0.029257	0.084303	0.347049	0.728554	NA
CYP1B1-AS	0.988912	0.029252	0.053115	0.550738	0.581813	NA
MIR2355	1.112329	0.029245	0.057605	0.507679	0.611679	NA
LINC00649	2.374244	0.02924	0.070046	0.417443	0.676354	NA
TMEM185	21.69702	0.029225	0.079889	0.365818	0.714501	NA
TMEM161	68.80468	0.029222	0.067255	0.434491	0.663932	NA
FAM124A	33.80712	-0.02921	0.081447	-0.35863	0.719873	NA
FAM177A1	130.0996	0.029208	0.050508	0.578278	0.563076	NA
MOB2	52.29771	-0.0292	0.070427	-0.41466	0.678388	NA
SCN11A	3.611919	-0.0292	0.075124	-0.38874	0.69747	NA
ZAR1L	0.153974	0.029203	0.022117	1.32037	0.186712	NA
MIR3180-5	0.520862	0.029195	0.041706	0.700006	0.483924	NA
PTPRCAP	12.3047	-0.02919	0.084893	-0.34388	0.730936	NA
ATXN1	719.1762	0.029192	0.034942	0.835464	0.403456	0.709466
BRD1	452.9573	0.029185	0.052593	0.554921	0.578949	0.82266
LRRC37A2	77.16676	-0.02918	0.08479	-0.34415	0.730734	NA
GCFC2	137.9237	0.02918	0.049685	0.587302	0.557001	NA
SPIRE2	183.6623	-0.02917	0.051268	-0.56897	0.569373	NA
ATP5F1	116.8538	-0.02917	0.060885	-0.47909	0.631872	NA
NAT16	0.757667	0.029164	0.047503	0.613935	0.539259	NA
KLHL23	2.522404	-0.02916	0.070125	-0.41587	0.677503	NA
CCNO	4.264821	0.029155	0.080715	0.361211	0.717941	NA
SPEG	295.3659	-0.02914	0.0563	-0.51764	0.604713	NA
ARID1B	959.4531	0.029142	0.04096	0.711477	0.476789	0.759174
TROVE2	300.8612	0.029141	0.042276	0.689305	0.490631	NA
PAMR1	8.561575	-0.02914	0.08318	-0.3503	0.726115	NA
QRFPR	0.335085	-0.02913	0.029296	-0.99422	0.320116	NA
EHBP1	738.6172	0.029123	0.027806	1.047364	0.294932	0.627839
LOC10050	399.4184	-0.02912	0.054976	-0.52963	0.596368	0.829672
ERC2	551.4991	0.029116	0.074119	0.392835	0.694441	0.871418
MPPE1	22.19018	0.029106	0.078251	0.371958	0.709924	NA
FAHD2A	65.18567	-0.0291	0.064873	-0.44857	0.653739	NA
FAM86JP	2.985851	-0.02909	0.062777	-0.46346	0.643038	NA
GCKR	6.604335	-0.02908	0.083944	-0.34644	0.729012	NA
CASKIN2	90.32316	-0.02908	0.051864	-0.56067	0.575021	NA
SLC4A2	47.1891	0.029074	0.072699	0.39992	0.689216	NA
EPGN	1.400749	0.029071	0.060506	0.480466	0.630896	NA
MIR4729	1.684616	0.029065	0.064353	0.451643	0.651526	NA
WDR26	521.6366	0.029062	0.043858	0.662638	0.507562	0.780812
EWSR1	768.2276	0.029062	0.035192	0.825812	0.408911	0.713546
MIR3914-1	0.237378	0.029061	0.029004	1.00196	0.316363	NA
HBG1	0.156155	0.029055	0.022163	1.310922	0.189884	NA
XRCC1	139.2007	0.029055	0.047986	0.605485	0.544856	NA
BBIP1	69.37997	-0.02905	0.05603	-0.51852	0.604092	NA

FOLH1B	0.234034	0.029044	0.024179	1.201195	0.229676	NA
MIR4667	0.229808	0.029035	0.028976	1.002015	0.316336	NA
PTK6	35.72663	-0.02903	0.084858	-0.3421	0.732278	NA
PSMD4	205.404	-0.02903	0.050629	-0.57334	0.566417	NA
CCDC140	1.112529	-0.02902	0.056151	-0.51684	0.605265	NA
EVC	42.27482	-0.02902	0.075732	-0.38318	0.701587	NA
ZNF480	86.06522	-0.02901	0.058741	-0.49385	0.621409	NA
KRT20	0.20482	0.029006	0.025313	1.145879	0.251845	NA
LOC33952	1.755739	0.029004	0.064735	0.448044	0.654121	NA
CNIH3	4.12333	-0.02899	0.077777	-0.3728	0.709298	NA
OTOS	0.455458	-0.02899	0.03616	-0.8017	0.422728	NA
SH3RF2	0.565145	-0.02898	0.042166	-0.68739	0.491838	NA
C11orf74	55.95379	-0.02898	0.064102	-0.45215	0.651158	NA
GALNT7	326.7982	0.02898	0.048696	0.595121	0.551763	NA
SNRPD3	127.0154	-0.02898	0.05124	-0.56551	0.571729	NA
KDSR	157.1748	0.028969	0.052516	0.55162	0.581209	NA
ZNF23	87.30172	0.028966	0.055004	0.526619	0.598458	NA
SFXN4	46.47999	0.028965	0.062843	0.460915	0.64486	NA
ISG20L2	124.756	-0.02896	0.062911	-0.4604	0.645226	NA
APIP	21.74497	-0.02896	0.080855	-0.35822	0.720175	NA
UBA3	171.2956	0.02896	0.051067	0.567103	0.570644	NA
DNAJC18	202.3346	0.028959	0.047436	0.610478	0.541545	NA
IFNL4	0.558291	-0.02896	0.039033	-0.74187	0.458164	NA
SETD8	98.04589	-0.02895	0.056553	-0.51194	0.608692	NA
CDPF1	10.58896	-0.02895	0.084802	-0.34136	0.73283	NA
ALG1L2	0.435518	0.02892	0.038381	0.753488	0.451157	NA
MIR204	1.379179	-0.02892	0.060707	-0.47637	0.633812	NA
ZDHHC8P1	26.39698	0.028919	0.084216	0.343387	0.731307	NA
FREM1	304.2247	-0.02892	0.077719	-0.37208	0.709832	NA
PRF1	1.146266	0.028914	0.054241	0.533058	0.593994	NA
MIR4271	0.513255	-0.02891	0.038924	-0.74278	0.457614	NA
AIMP2	22.01971	-0.02891	0.075612	-0.3823	0.702238	NA
BHLHE40-	0.62279	-0.0289	0.045807	-0.63097	0.528057	NA
TGFB3	50.09993	-0.0289	0.068608	-0.4212	0.673609	NA
KCNMB4	300.6267	0.028894	0.077428	0.373178	0.709016	NA
ZNF251	209.9453	-0.02889	0.050924	-0.56731	0.5705	NA
CX3CR1	8.1281	-0.02889	0.07518	-0.38426	0.700783	NA
CYP2W1	2.869385	-0.02888	0.076355	-0.37828	0.705224	NA
MICB	13.5275	0.02888	0.060417	0.47802	0.632636	NA
MARCKS	705.3718	-0.02888	0.063061	-0.45794	0.646995	0.849041
KIF14	3.007455	-0.02887	0.071668	-0.40287	0.687045	NA
ETFDH	133.4983	-0.02887	0.052845	-0.54634	0.584832	NA
LOC10027	0.348018	-0.02887	0.032516	-0.88775	0.374675	NA
C18orf21	50.09464	0.028865	0.061989	0.465642	0.641472	NA
FAM105B	177.7722	-0.02886	0.057195	-0.50461	0.613835	NA
CFL2	179.6209	-0.02886	0.06552	-0.44047	0.659596	NA
NFKB2	19.27675	0.028859	0.084839	0.340167	0.733731	NA

PGR	6.802083	0.028853	0.081551	0.353804	0.723486	NA
MRPL53	31.95133	-0.02885	0.076871	-0.37532	0.707424	NA
ACTR6	71.87313	0.028838	0.061725	0.467205	0.640353	NA
LOC15738	0.337373	-0.02883	0.027046	-1.06595	0.286446	NA
ACE2	0.774036	-0.02883	0.050138	-0.575	0.565294	NA
SNCAIP	88.9746	0.02882	0.064532	0.446603	0.655162	NA
KLHL41	31.75517	-0.02881	0.074973	-0.38427	0.700777	NA
AIPL1	0.333021	-0.0288	0.034191	-0.84247	0.399526	NA
DLEU1	43.92598	-0.02879	0.075434	-0.3817	0.702683	NA
SHE	24.5584	-0.02879	0.084083	-0.34237	0.732075	NA
WNK4	12.53283	0.028778	0.084515	0.340509	0.733473	NA
BHLHE41	472.9649	0.028757	0.039626	0.725719	0.468011	0.755844
TSHZ2	15.82333	-0.02876	0.084452	-0.3405	0.73348	NA
CHD5	513.2232	-0.02875	0.06595	-0.43599	0.662847	0.855957
RCCD1	117.784	0.028751	0.057369	0.501159	0.616259	NA
FNBP1L	313.4433	-0.02875	0.067076	-0.42856	0.668241	NA
GJA4	18.52599	0.028743	0.084735	0.339215	0.734447	NA
FOXD2	0.589326	-0.02874	0.041324	-0.69554	0.486714	NA
SIRT3	49.77625	-0.02874	0.060271	-0.47688	0.633448	NA
SLC26A8	28.28549	-0.02873	0.080186	-0.35829	0.720128	NA
IFNK	15.03281	-0.02871	0.084739	-0.3388	0.73476	NA
C17orf80	146.4499	-0.02871	0.044779	-0.64109	0.521465	NA
GFM1	348.666	0.028701	0.054064	0.53087	0.595509	0.829672
PACSIN3	55.08212	0.028693	0.065422	0.43858	0.660966	NA
DPY19L1	159.4437	-0.02868	0.069313	-0.41378	0.679038	NA
MIR4522	0.287036	-0.02868	0.029639	-0.96748	0.333306	NA
GBP6	0.963531	0.028671	0.049982	0.573618	0.566226	NA
OR7E2P	1.918482	0.028662	0.067862	0.422366	0.672758	NA
LOC10014	40.37905	-0.02866	0.072065	-0.39772	0.690838	NA
MIR125B2	1.506411	-0.02866	0.060548	-0.47336	0.635956	NA
FOXD4L6	0.2267	-0.02866	0.026739	-1.07185	0.283787	NA
CPNE7	0.437315	-0.02866	0.037561	-0.76295	0.445492	NA
PHKA2-AS	4.931674	-0.02865	0.08272	-0.3463	0.729119	NA
FAM19A5	217.422	0.028644	0.067287	0.425701	0.670326	NA
IL15RA	2.713966	-0.02864	0.065327	-0.43843	0.661076	NA
FAM195B	57.12783	0.02864	0.0637	0.449609	0.652992	NA
DKFZp434	2.332568	0.02863	0.073068	0.391819	0.695192	NA
CSRNP2	210.3839	0.028623	0.041409	0.691229	0.489422	NA
HDGF	322.3444	0.02861	0.050549	0.565989	0.571401	NA
SLC22A11	0.366561	-0.02861	0.031149	-0.91839	0.358416	NA
AP1M1	163.131	0.028607	0.043562	0.656686	0.511383	NA
POLR3A	284.6493	-0.0286	0.036272	-0.78851	0.430396	NA
SLC6A12	27.34178	0.028588	0.083709	0.341511	0.732719	NA
LOC64464	0.144231	-0.02859	0.018757	-1.52403	0.127502	NA
KCTD2	605.6664	0.02858	0.055061	0.519061	0.603718	0.830918
BEX2	181.626	0.028578	0.075971	0.376171	0.70679	NA
FAM196B	16.61905	-0.02856	0.083079	-0.34381	0.730991	NA

CECR2	715.8835	-0.02856	0.064051	-0.4459	0.655668	0.854402
TXN2	66.26862	-0.02855	0.065764	-0.43409	0.664222	NA
AMBRA1	173.2869	0.028539	0.045363	0.629133	0.529262	NA
PSMA3	110.526	-0.02853	0.049734	-0.5737	0.566174	NA
PRR22	5.62079	-0.02852	0.082844	-0.3443	0.730624	NA
TMEM31	1.615555	0.028513	0.059382	0.480157	0.631116	NA
ACVR2B	370.9543	-0.02851	0.058885	-0.48421	0.628234	0.84337
SHH	0.580293	-0.02851	0.041539	-0.68636	0.492488	NA
CENPF	11.83184	0.02851	0.083891	0.339846	0.733972	NA
CCDC174	123.3201	0.02851	0.050605	0.56338	0.573176	NA
MIR5584	0.133374	-0.02851	0.01702	-1.67504	0.093927	NA
PRMT6	31.86458	0.028505	0.073361	0.388559	0.697603	NA
LTB4R2	3.230115	0.028505	0.077076	0.369828	0.711511	NA
DTD1	71.91222	-0.0285	0.077382	-0.36828	0.712661	NA
BNIP1	14.36068	-0.02848	0.08361	-0.34061	0.733394	NA
PNMA6C	1.578326	0.02847	0.061361	0.463972	0.642668	NA
LAX1	0.351869	-0.02847	0.034986	-0.81373	0.415798	NA
CAB39L	127.6953	-0.02846	0.06669	-0.42677	0.669544	NA
MIRLET7D	1.098568	0.02846	0.056021	0.508027	0.611435	NA
HSD17B7	16.13683	-0.02845	0.083482	-0.34084	0.733226	NA
CADM4	216.0473	0.028444	0.056566	0.502853	0.615068	NA
DEDD2	44.03017	-0.02844	0.073384	-0.38759	0.69832	NA
NCAPH2	135.7234	-0.02842	0.060518	-0.46962	0.638628	NA
PACS2	630.336	0.02842	0.045174	0.629122	0.529269	0.792854
MUTYH	78.0934	-0.02842	0.063171	-0.44986	0.652808	NA
PALB2	98.74681	0.028417	0.061175	0.464527	0.64227	NA
HSPB7	19.2858	0.028417	0.07845	0.362227	0.717183	NA
SH3GL1P2	16.44557	-0.02841	0.084932	-0.33452	0.737989	NA
MIR1250	2.74624	-0.02841	0.072339	-0.3927	0.694544	NA
SFTPB	2.480247	-0.02841	0.071054	-0.39979	0.689315	NA
ACADVL	347.6662	-0.02841	0.056692	-0.50105	0.616336	0.839018
GOLGA6L4	3.451932	-0.0284	0.071078	-0.39962	0.689439	NA
IDH3B	127.6444	-0.0284	0.051489	-0.55164	0.581193	NA
TIMM17B	21.19943	0.028392	0.079846	0.355583	0.722153	NA
DHX38	329.9056	-0.02839	0.032655	-0.8694	0.38463	NA
AVP	0.192716	-0.02836	0.024928	-1.13772	0.255236	NA
UBN1	268.5735	0.028351	0.038416	0.737997	0.460516	NA
PSMD10	47.11397	0.028351	0.074296	0.38159	0.702765	NA
ZNF513	51.18666	0.028348	0.067517	0.419873	0.674578	NA
GNPDA2	122.7313	0.028342	0.052271	0.54221	0.587674	NA
EIF4H	318.0252	0.028338	0.055587	0.509793	0.610197	NA
RASGRP2	35.06348	-0.02833	0.084123	-0.33678	0.736281	NA
MLLT4	1073.571	-0.02831	0.0375	-0.75497	0.450269	0.749346
KCNJ15	0.606105	0.02831	0.041655	0.679623	0.496743	NA
MCCC2	111.7392	-0.02831	0.076072	-0.37211	0.709811	NA
MIR299	0.792454	0.028303	0.046501	0.608644	0.542761	NA
TMEM150	5.4584	-0.0283	0.082771	-0.34193	0.732403	NA

LOC44004	0.618968	-0.02829	0.04542	-0.62294	0.533327	NA
TIGIT	5.906238	-0.02829	0.078705	-0.35945	0.719259	NA
C17orf50	2.005468	-0.02829	0.064175	-0.44082	0.659344	NA
ZNFX1	230.7955	0.028289	0.042728	0.662079	0.507921	NA
MIR4738	0.197654	0.028285	0.025294	1.118244	0.263463	NA
COQ10B	55.00559	0.028283	0.071494	0.395607	0.692395	NA
HCFC2	72.05276	0.028282	0.065406	0.432405	0.665447	NA
MT1A	0.945628	0.028269	0.044729	0.632001	0.527386	NA
ARHGAP19	32.84	0.028265	0.073704	0.383492	0.701355	NA
DOPEY1	706.8626	0.028261	0.038289	0.7381	0.460454	0.754105
HORMAD2	1.000995	-0.02826	0.049314	-0.57307	0.5666	NA
ZNF16	24.6379	0.028259	0.078443	0.360256	0.718656	NA
MIR609	0.176767	-0.02826	0.022168	-1.27471	0.202411	NA
TIMM50	42.85685	-0.02826	0.065835	-0.42922	0.667762	NA
CCDC12	77.48455	-0.02826	0.059496	-0.47492	0.634847	NA
TRIM27	87.44987	-0.02825	0.052437	-0.53877	0.590046	NA
LOXL1	0.594859	-0.02824	0.044409	-0.63594	0.524814	NA
SARNP	98.19742	-0.02824	0.053074	-0.53199	0.594734	NA
CARD8	105.5434	0.02823	0.055491	0.50873	0.610942	NA
IL17RD	39.97527	0.028214	0.078995	0.357164	0.720969	NA
BIRC6	1741.136	0.02821	0.031756	0.888317	0.37437	0.695376
HAP1	47.16143	0.028207	0.084339	0.334448	0.738041	NA
MFAP4	13.79509	-0.0282	0.084687	-0.33299	0.739141	NA
SHROOM4	44.40356	-0.02819	0.082407	-0.34206	0.732306	NA
TKTL2	0.161407	0.028186	0.02179	1.293574	0.195813	NA
CHMP1B	106.416	0.028182	0.061586	0.457599	0.64724	NA
TOB1-AS1	8.35948	0.02818	0.084816	0.332245	0.739704	NA
AKAP9	2814.055	0.028179	0.039238	0.718171	0.472652	0.759174
MAGI3	336.7425	0.028175	0.04045	0.696532	0.486096	0.764934
CCDC124	87.068	0.028159	0.059558	0.472796	0.636359	NA
ZNF33B	365.2539	-0.02816	0.046282	-0.60839	0.54293	0.803134
PWP1	126.9459	0.028158	0.056091	0.501993	0.615673	NA
DPRXP4	4.461538	0.028156	0.080928	0.347913	0.727905	NA
C15orf62	59.91142	-0.02815	0.064829	-0.43424	0.664116	NA
GRM8	8.224227	0.028148	0.081625	0.344845	0.730211	NA
GPR20	0.404116	0.028147	0.037186	0.756918	0.449099	NA
MIR4743	0.958238	0.028146	0.052903	0.532028	0.594706	NA
SALL1	48.61663	-0.02814	0.082703	-0.34019	0.733712	NA
SPRY1	29.51638	-0.02813	0.075654	-0.37189	0.709977	NA
MCF2	31.35399	0.028106	0.084927	0.33094	0.74069	NA
FIGLA	0.260162	-0.0281	0.026654	-1.05443	0.291687	NA
PCDHB13	59.7272	-0.0281	0.072906	-0.38545	0.699902	NA
BARHL2	168.5687	0.0281	0.058789	0.477984	0.632662	NA
CLEC12A	0.297268	-0.0281	0.026798	-1.04859	0.294368	NA
HHLA2	0.213681	-0.0281	0.026725	-1.05138	0.293085	NA
SPATC1L	22.14329	0.028095	0.084428	0.332774	0.739305	NA
ESYT3	279.0762	0.028095	0.084443	0.332703	0.739358	NA

TET1	294.3467	0.028088	0.042955	0.653895	0.51318	NA
GP6	25.63332	-0.02809	0.084796	-0.33122	0.74048	NA
NOXO1	24.41392	0.02808	0.078473	0.357823	0.720476	NA
TSNAX-DIS	0.466841	0.028072	0.037964	0.739445	0.459637	NA
SETD1A	359.1329	0.028067	0.054704	0.513071	0.607902	0.832465
C1orf228	8.444175	-0.02806	0.084893	-0.33058	0.740959	NA
OR1Q1	0.339572	-0.02805	0.031604	-0.88771	0.374699	NA
SLC34A2	57.54626	-0.02805	0.084345	-0.33262	0.739423	NA
SCRN2	65.23248	0.028052	0.065376	0.429095	0.667854	NA
CTAGE9	0.907822	0.028051	0.04896	0.572924	0.566696	NA
SPINK6	2.293691	-0.02805	0.070281	-0.39911	0.689809	NA
OR51B5	1.711595	-0.02804	0.062664	-0.44746	0.654544	NA
OSM	0.639812	0.028039	0.041769	0.671281	0.502042	NA
PADI4	0.343679	0.028036	0.033332	0.841091	0.400297	NA
FMR1NB	0.158086	0.028027	0.02176	1.288005	0.197744	NA
MAP3K5	454.7038	0.028017	0.051311	0.546035	0.585042	0.824332
ZNF655	745.4417	-0.02802	0.035061	-0.79905	0.424263	0.728057
SNX25	398.1345	0.028014	0.061115	0.458377	0.646682	0.849041
RHOBTB1	15.05016	0.028012	0.084391	0.331933	0.73994	NA
GIMAP4	28.29878	-0.02801	0.084577	-0.33112	0.740553	NA
RBM39	1158.392	-0.028	0.02943	-0.95134	0.34143	0.664952
FAM107B	236.027	0.027996	0.06921	0.404511	0.685837	NA
MIR4761	1.663737	-0.02798	0.060491	-0.46257	0.643669	NA
PPEF1	1.522176	-0.02798	0.051816	-0.54002	0.589186	NA
PRRC2B	2205.077	0.027977	0.039143	0.71474	0.47477	0.759174
XPC	168.8686	-0.02796	0.057081	-0.48981	0.624265	NA
SBF1P1	22.17309	-0.02796	0.078505	-0.35611	0.721762	NA
CES3	25.89986	0.027954	0.083411	0.33513	0.737527	NA
SPG20OS	1.628438	0.027948	0.064052	0.436329	0.662598	NA
HCG4B	2.9064	-0.02793	0.070128	-0.39833	0.690384	NA
IL2RB	0.436126	-0.02793	0.038782	-0.72016	0.471426	NA
UBR5	1373.607	-0.02793	0.043945	-0.63552	0.525091	0.789308
FLOT1	437.8672	0.027915	0.046553	0.599638	0.548747	0.803817
SLC22A17	348.2494	-0.02791	0.065361	-0.42703	0.669357	0.861137
PTDSS2	84.67065	-0.02791	0.072152	-0.3868	0.698906	NA
FCGR1C	0.492035	-0.0279	0.036509	-0.76431	0.444681	NA
CD320	19.02004	-0.0279	0.079435	-0.35119	0.725445	NA
SLC38A3	17.08762	-0.02789	0.082489	-0.33805	0.735326	NA
KIRREL	9.688732	-0.02787	0.084379	-0.33034	0.741144	NA
FBXW4	114.1968	0.027871	0.045972	0.606262	0.544341	NA
BMX	0.781415	-0.02786	0.045629	-0.61068	0.54141	NA
CEBPB	25.41297	-0.02786	0.084343	-0.33035	0.741138	NA
LLGL1	102.124	0.027861	0.064992	0.428676	0.668159	NA
PIGW	13.36802	0.02786	0.084862	0.328292	0.742691	NA
CXCL2	2.171016	-0.02786	0.056358	-0.49432	0.621083	NA
AIM1	43.99231	-0.02786	0.079454	-0.35059	0.725898	NA
ADAMTS8	4.556601	-0.02785	0.080814	-0.34461	0.730389	NA

DDX11L1	0.57057	0.027848	0.040378	0.689677	0.490398	NA
IQCC	50.62369	0.027839	0.069931	0.398096	0.69056	NA
ZNF141	143.2264	-0.02783	0.060861	-0.45731	0.647449	NA
SCN1A	544.4447	0.027831	0.069895	0.39819	0.69049	0.870767
LOC10050	6.252515	0.027829	0.084294	0.33014	0.741294	NA
SFN	1.827312	0.027824	0.059243	0.469664	0.638595	NA
MSH2	211.536	0.027821	0.042576	0.653433	0.513477	NA
MS4A14	4.003348	0.02782	0.070706	0.393458	0.693981	NA
SSR2	41.45304	-0.02782	0.077594	-0.35852	0.719955	NA
UBR3	791.5546	0.027815	0.028655	0.970694	0.331701	0.660832
SMC4	78.83825	-0.02781	0.057721	-0.48185	0.629915	NA
MIR569	0.762434	0.02781	0.046505	0.597995	0.549843	NA
SLITRK6	23.14196	0.027806	0.054462	0.510554	0.609664	NA
PRSS3	45.52261	-0.0278	0.076817	-0.36195	0.717391	NA
DTWD1	71.42647	0.027794	0.060087	0.462565	0.643676	NA
ACTR10	142.9903	0.027791	0.066548	0.417601	0.676239	NA
CMTM2	10.2587	-0.02779	0.084922	-0.32722	0.743504	NA
LOC10050	2.134843	-0.02778	0.06469	-0.42951	0.667555	NA
MIR181B1	0.200214	0.027781	0.025981	1.069281	0.284943	NA
METTL24	14.48133	-0.02777	0.084868	-0.32726	0.743469	NA
OR2H1	0.127493	0.027755	0.018338	1.513532	0.130144	NA
SPINK5	3.480985	-0.02775	0.078297	-0.35447	0.722986	NA
IL6	0.959021	0.027749	0.04158	0.66736	0.504542	NA
SLC35G2	63.69095	0.027747	0.059991	0.462509	0.643716	NA
CHAF1B	45.35469	-0.02774	0.074971	-0.37	0.711381	NA
FN1	182.8252	0.027737	0.078086	0.35521	0.722432	NA
MB	0.185182	-0.02774	0.024887	-1.11448	0.265074	NA
RABGEF1	175.2376	-0.02773	0.048398	-0.57297	0.566664	NA
KANK1	470.1986	0.02773	0.060248	0.460265	0.645326	0.849041
MIR647	5.050429	-0.02773	0.083141	-0.33348	0.738772	NA
UIMC1	242.3929	-0.02773	0.047762	-0.58049	0.561587	NA
RHCG	4.539459	0.027724	0.078999	0.350942	0.725632	NA
MZB1	0.354356	-0.02772	0.034201	-0.81054	0.417631	NA
COL22A1	51.20913	-0.02772	0.082039	-0.33788	0.73545	NA
TAS2R43	10.86629	-0.02772	0.081381	-0.3406	0.733404	NA
ZNF124	55.82786	0.027713	0.080451	0.344468	0.730494	NA
FBXO2	123.5671	-0.02771	0.069401	-0.39929	0.689681	NA
UCHL5	284.0967	-0.0277	0.05621	-0.49288	0.622095	NA
DDX6	1238.125	0.0277	0.041693	0.664382	0.506446	0.780812
PSMD9	41.44278	-0.0277	0.066889	-0.41412	0.678788	NA
SMPDL3A	72.45841	-0.0277	0.055144	-0.50231	0.615446	NA
SLC28A2	10.01427	-0.02769	0.075489	-0.36683	0.713749	NA
BBX	388.1442	0.02769	0.047642	0.581198	0.561107	0.810528
ZBTB34	176.718	-0.02769	0.055084	-0.50268	0.615192	NA
LYVE1	8.820327	-0.02768	0.082432	-0.33585	0.736987	NA
FBXL2	69.94962	0.027679	0.061265	0.451793	0.651418	NA
XYLT1	108.9499	0.027671	0.080234	0.344876	0.730187	NA

TNIP1	237.3974	-0.02767	0.047954	-0.57702	0.563924	NA
CNFN	0.671258	0.027669	0.043568	0.635067	0.525385	NA
BCL2L11	25.62804	0.027658	0.083704	0.330427	0.741077	NA
COMMD8	17.84362	0.027654	0.081654	0.338668	0.73486	NA
MTMR10	195.3538	0.027649	0.056131	0.492588	0.622304	NA
MIR4514	0.375759	-0.02765	0.035719	-0.77406	0.438896	NA
DBF4B	47.36101	-0.02765	0.074365	-0.37175	0.710077	NA
CCDC150	8.653329	0.027645	0.084938	0.325472	0.744824	NA
KC6	0.256644	-0.02764	0.027911	-0.99031	0.322024	NA
BTBD1	153.5062	-0.02764	0.064723	-0.42703	0.669358	NA
CD55	93.68799	-0.02764	0.058972	-0.46866	0.63931	NA
PRSS54	0.200302	0.027632	0.025138	1.099231	0.271667	NA
PDLIM2	111.0483	0.027629	0.074271	0.372006	0.709889	NA
FGD6	116.2163	-0.02763	0.053599	-0.51545	0.606235	NA
HAS3	25.45461	-0.02762	0.0789	-0.35008	0.726276	NA
LOC28437	0.173229	0.027604	0.022023	1.253388	0.210064	NA
PMM2	51.47382	-0.0276	0.073257	-0.37674	0.706364	NA
PRKAA1	258.5106	0.027593	0.068018	0.40567	0.684985	NA
PRKAR1B	306.2123	0.027592	0.054859	0.502965	0.614989	NA
TTLL3	30.37929	-0.02758	0.075586	-0.36492	0.715172	NA
TRA2B	322.6831	0.02758	0.040844	0.675243	0.499522	NA
MYADML	0.125515	0.027568	0.018289	1.507382	0.131713	NA
GTPBP2	114.1068	0.027568	0.055305	0.498479	0.618146	NA
KIAA1430	135.4733	0.027564	0.065298	0.42213	0.67293	NA
SCT	4.053016	-0.02756	0.073586	-0.37454	0.708005	NA
EID1	285.3079	0.027557	0.069009	0.399323	0.689655	NA
43357	0.588643	-0.02755	0.04074	-0.67628	0.498864	NA
TMEM55A	98.74663	0.027552	0.058028	0.4748	0.63493	NA
SRSF9	63.59834	-0.02755	0.0591	-0.46615	0.641106	NA
CHP1	77.06671	0.027549	0.074068	0.371942	0.709936	NA
KANSL2	98.08518	0.027546	0.050372	0.546859	0.584475	NA
LOC10050	12.58987	0.027542	0.084781	0.324856	0.74529	NA
C7orf53	26.91541	-0.02753	0.084712	-0.32502	0.745163	NA
ZDBF2	948.1629	-0.02753	0.045453	-0.60573	0.544695	0.803213
ALDH9A1	113.0427	-0.02753	0.061998	-0.44403	0.657022	NA
POU2F1	730.1592	0.027526	0.040899	0.673027	0.50093	0.778081
OGG1	71.93373	0.02752	0.0617	0.446028	0.655577	NA
HTATSF1P	163.7726	-0.02751	0.061403	-0.4481	0.654083	NA
CD99L2	341.2006	0.027513	0.065843	0.417855	0.676053	0.863655
BRK1	68.42214	-0.02751	0.066444	-0.41407	0.678826	NA
BRSK2	705.1173	0.027501	0.048202	0.570543	0.568309	0.815213
SETD5	1402.204	-0.0275	0.04079	-0.67406	0.500274	0.778081
UPK1A	0.673074	0.027494	0.043864	0.626801	0.53079	NA
SLC22A18	0.121421	0.027488	0.018268	1.50474	0.132391	NA
ANKRD29	11.2232	-0.02748	0.084897	-0.32372	0.74615	NA
C6orf15	0.251169	-0.02748	0.029857	-0.92043	0.357347	NA
PKP3	0.598897	-0.02747	0.041606	-0.66025	0.509094	NA

SLC30A3	0.326909	-0.02747	0.032454	-0.84645	0.397301	NA
KATNA1	67.89981	-0.02747	0.065603	-0.41872	0.67542	NA
MIA	0.283	0.027465	0.031421	0.874098	0.382065	NA
MIR548Q	2.793333	-0.02746	0.07517	-0.36536	0.714845	NA
SCN2A	3264.869	-0.02746	0.048479	-0.56649	0.571062	0.81719
MEG8	48.96978	-0.02746	0.072965	-0.37635	0.706655	NA
FAHD2B	36.02922	-0.02746	0.080174	-0.34247	0.731997	NA
CMIP	148.7179	-0.02744	0.059184	-0.46371	0.642855	NA
KLHL30	2.808461	0.027443	0.067736	0.405152	0.685366	NA
MIR367	0.274928	-0.02744	0.026645	-1.0299	0.303056	NA
PCSK4	7.751284	0.027441	0.084668	0.324104	0.745859	NA
NME7	51.55514	0.027428	0.059706	0.459376	0.645964	NA
NEU1	49.98301	-0.02742	0.066999	-0.40929	0.682324	NA
MYL6	476.3053	-0.02741	0.060967	-0.4496	0.653001	0.853604
MIR423	0.154236	0.027407	0.021772	1.258821	0.208095	NA
HS6ST1	82.53882	0.027406	0.058098	0.471715	0.63713	NA
ORAI3	17.47413	-0.0274	0.081108	-0.33787	0.735462	NA
CDHR4	0.256683	-0.0274	0.026581	-1.03087	0.302602	NA
UBXN7	428.3834	-0.0274	0.042222	-0.64892	0.516391	0.785288
HYLS1	31.84645	-0.0274	0.067023	-0.40878	0.682699	NA
LRRC4C	57.45585	0.027396	0.081997	0.334114	0.738294	NA
RBMXL3	3.285501	0.027396	0.07519	0.364356	0.715592	NA
C8orf82	38.8418	0.027393	0.07734	0.354193	0.723194	NA
THRAP3	761.4796	-0.02739	0.036486	-0.75079	0.452781	0.750995
DMAP1	114.4846	-0.02739	0.048639	-0.56303	0.573418	NA
GNAI3	205.591	0.027384	0.05168	0.529874	0.596199	NA
LOC28574	9.036556	0.027381	0.084263	0.324946	0.745222	NA
SLC25A46	166.094	0.027379	0.051752	0.529039	0.596779	NA
POMT2	90.32202	0.027378	0.067137	0.407797	0.683423	NA
SH3D19	167.863	-0.02737	0.072117	-0.37946	0.70435	NA
IDH3G	121.6529	-0.02736	0.061336	-0.44605	0.65556	NA
MPV17	53.81298	0.027359	0.070935	0.385686	0.699729	NA
SUSD4	105.9368	-0.02736	0.054408	-0.50283	0.615081	NA
TPCN2	158.9234	-0.02736	0.062892	-0.43495	0.663595	NA
LBX1	6.324361	0.027355	0.080322	0.340566	0.73343	NA
MTFR1	55.77355	0.027351	0.070787	0.386383	0.699213	NA
LOC64496	35.75917	-0.02735	0.075312	-0.36316	0.716488	NA
CYTH3	196.4632	0.027324	0.054109	0.50498	0.613573	NA
MIR21	1.236097	0.027323	0.055969	0.488171	0.625429	NA
C7orf61	5.080658	0.027317	0.080964	0.337392	0.735821	NA
CDK7	42.22727	-0.02731	0.064821	-0.42128	0.673548	NA
HEATR5B	282.2948	-0.02727	0.034031	-0.80135	0.422932	NA
GLRX2	9.857364	0.027268	0.084337	0.323319	0.746453	NA
HIST1H2A	0.395486	-0.02726	0.033647	-0.81021	0.417822	NA
SFI1	492.4994	-0.02726	0.054729	-0.498	0.618482	0.839739
FKBP15	240.3388	0.027255	0.037135	0.733934	0.462989	NA
CATSPER2	195.798	-0.02724	0.075359	-0.36144	0.717768	NA

FCF1	36.30864	0.027229	0.06908	0.394163	0.69346	NA
CRTAM	549.6172	-0.02722	0.066284	-0.41071	0.681284	0.866937
NDUFS6	39.18314	-0.02722	0.07819	-0.34815	0.727731	NA
C2orf50	1.108083	0.027221	0.054562	0.498901	0.617849	NA
KIAA0907	218.738	0.027216	0.041671	0.653118	0.51368	NA
GP5	0.31533	-0.02722	0.0342	-0.79578	0.426161	NA
IGFL2	0.655476	0.027199	0.041159	0.660812	0.508733	NA
RAVER1	92.23573	0.027179	0.054947	0.494632	0.62086	NA
NSUN4	58.76069	0.027177	0.060175	0.451636	0.651532	NA
TFIP11	125.2586	0.027173	0.045492	0.597314	0.550298	NA
NIPSNAP3	15.60232	-0.02717	0.084574	-0.32125	0.748021	NA
TRPM8	0.587648	0.02716	0.041015	0.662214	0.507834	NA
TRDN	8.223307	0.027158	0.080339	0.338043	0.735331	NA
ANKIB1	429.4691	-0.02715	0.032009	-0.8483	0.39627	0.705623
SERINC3	281.895	0.027149	0.051323	0.528972	0.596825	NA
TMEM234	29.65845	-0.02714	0.072254	-0.37567	0.70716	NA
TTL	129.4455	0.027142	0.053024	0.511887	0.60873	NA
SKIV2L2	409.6203	0.027132	0.038152	0.711156	0.476987	0.759174
MIRLET7B	177.4541	-0.02713	0.055522	-0.48863	0.625101	NA
TBC1D13	84.21646	0.027127	0.053041	0.511441	0.609042	NA
ANKRD13C	252.9222	-0.02712	0.042512	-0.63802	0.52346	NA
PAX6	993.7754	0.027121	0.036572	0.741562	0.458353	0.754035
TMEM50B	196.1966	0.027113	0.056024	0.483954	0.628418	NA
ZNF627	54.9765	-0.02711	0.068591	-0.3952	0.692692	NA
MYOG	14.79357	-0.02711	0.084746	-0.31985	0.749083	NA
C1D	19.73399	-0.0271	0.082646	-0.32795	0.74295	NA
ZNF699	65.01958	0.027093	0.057295	0.472867	0.636308	NA
POU4F1	1.703721	0.027092	0.060042	0.451217	0.651833	NA
SDHAF1	33.58073	-0.02707	0.067659	-0.40015	0.689044	NA
BCAS2	93.07803	0.027074	0.065017	0.416409	0.677111	NA
MIR4777	0.241264	0.027073	0.028076	0.964272	0.33491	NA
GDAP1L1	74.3695	-0.02707	0.061687	-0.43884	0.660779	NA
TEKT4P2	61.71169	-0.02707	0.074259	-0.36454	0.715454	NA
DSP	33.83732	0.02706	0.082511	0.327951	0.742948	NA
HDX	118.2506	0.027047	0.050186	0.538938	0.589929	NA
SLC25A41	12.61394	-0.02704	0.084885	-0.31859	0.750037	NA
PACSIN1	858.8163	0.027041	0.04809	0.562298	0.573913	0.819101
ATF7	384.2212	0.027035	0.05507	0.490909	0.623491	0.841191
SDF2	24.05446	-0.02703	0.079256	-0.34101	0.733099	NA
SNORD36A	5.119556	-0.02702	0.074508	-0.36264	0.716875	NA
CLECL1	0.481987	-0.02702	0.030758	-0.87841	0.37972	NA
KRT17	1.543389	-0.02702	0.058005	-0.46578	0.641371	NA
LOC28410	27.85749	-0.02702	0.075683	-0.35696	0.72112	NA
DEF8	399.8458	0.027015	0.0655	0.412446	0.680012	0.866757
TMEM104	73.32567	0.027003	0.070113	0.385138	0.700135	NA
ZC3H8	84.69706	0.027	0.052613	0.513188	0.60782	NA
AVPR1A	0.8378	-0.027	0.044746	-0.60331	0.546301	NA

DCAKD	72.44116	0.02699	0.071655	0.376667	0.706421	NA
SCRT2	41.42913	0.026987	0.084936	0.317736	0.750685	NA
MIR488	0.428992	-0.02698	0.038741	-0.6965	0.486119	NA
LENEP	2.183351	0.026982	0.071026	0.379887	0.704029	NA
LRRC55	3.503018	-0.02697	0.065606	-0.41108	0.681012	NA
CACNA1C-	5.517327	-0.02697	0.080544	-0.33482	0.737757	NA
HLA-DRB6	2.135265	-0.02696	0.053029	-0.50846	0.611132	NA
POM121L9	2.665521	-0.02696	0.072492	-0.37191	0.709958	NA
RALBP1	348.8293	-0.02696	0.048995	-0.55027	0.582135	0.824332
C11orf84	45.61483	0.026959	0.079182	0.340469	0.733504	NA
ZNHIT3	30.02932	0.026959	0.075506	0.357037	0.721064	NA
C16orf89	39.55687	0.026958	0.084832	0.317787	0.750646	NA
MIR4505	0.736702	-0.02695	0.046163	-0.58389	0.559292	NA
TSACC	4.258611	0.026954	0.077828	0.346324	0.729099	NA
DAP	30.79414	-0.02695	0.078225	-0.34455	0.730436	NA
SACS	792.4856	0.026951	0.070289	0.383436	0.701396	0.877714
SATB2	12.63239	0.026947	0.084552	0.318696	0.749957	NA
PDCD11	181.9345	0.026945	0.043949	0.613108	0.539805	NA
LRWD1	65.78057	0.026941	0.080413	0.335033	0.7376	NA
RANBP1	114.602	-0.02693	0.063133	-0.42658	0.669684	NA
PTH2	0.754946	-0.02693	0.046048	-0.58477	0.558705	NA
ELF3	1.449081	0.026926	0.060282	0.446671	0.655112	NA
PCDHA5	83.63488	0.026926	0.069538	0.387212	0.698599	NA
C8orf4	4.615988	-0.02693	0.067631	-0.39813	0.690537	NA
SDCBP2	2.865998	-0.02692	0.072654	-0.37053	0.710991	NA
WDFY2	77.17637	0.026917	0.067149	0.400857	0.688525	NA
RAD51AP2	3.768712	0.026909	0.078897	0.341061	0.733057	NA
LOC10012	5.714369	-0.02691	0.083492	-0.32227	0.747249	NA
LRRC69	2.778112	0.026898	0.066289	0.405768	0.684913	NA
C6orf7	2.136131	-0.02689	0.064664	-0.4159	0.677482	NA
C5orf48	1.06555	-0.02688	0.046073	-0.58346	0.559583	NA
LINC00514	19.50766	0.026882	0.084938	0.316482	0.751637	NA
IP6K1	218.2734	-0.02688	0.043603	-0.61647	0.537582	NA
PSMA1	80.47639	-0.02688	0.061169	-0.43939	0.660376	NA
CCDC82	519.3409	-0.02688	0.059892	-0.44873	0.65363	0.85379
ZNF841	274.0827	-0.02688	0.045852	-0.58613	0.557787	NA
RQCD1	146.7885	-0.02687	0.045955	-0.5847	0.55875	NA
ORAI1	2.354083	-0.02687	0.072803	-0.36904	0.712099	NA
RNU5F-1	1.271654	-0.02686	0.058285	-0.46083	0.644923	NA
MIR3944	0.197671	-0.02686	0.025007	-1.07404	0.282805	NA
FER1L5	40.76525	-0.02686	0.078914	-0.34035	0.733594	NA
PCDH20	5.102532	-0.02686	0.079706	-0.33696	0.736145	NA
UBA52	221.5253	-0.02685	0.059851	-0.44865	0.653685	NA
ARHGDIG	184.0736	-0.02685	0.064042	-0.41929	0.675005	NA
LOC10050	6.120795	0.026852	0.083598	0.3212	0.748059	NA
PAN3-AS1	39.12445	0.026842	0.069884	0.384089	0.700912	NA
MYCT1	8.97129	0.02684	0.083946	0.319726	0.749176	NA

NPPB	0.256349	-0.02683	0.029059	-0.92337	0.355813	NA
FUT7	6.416573	-0.02683	0.084582	-0.31722	0.751075	NA
MYH6	0.522891	0.026823	0.037688	0.711704	0.476648	NA
MYEOV	0.225592	-0.02681	0.025411	-1.05526	0.291304	NA
HSPA6	2.911826	0.026806	0.059795	0.448293	0.653942	NA
LOC10028	4.95003	-0.0268	0.082065	-0.32654	0.744016	NA
MIR30C2	0.438383	0.026791	0.036995	0.724172	0.46896	NA
PIGZ	54.5231	-0.02677	0.076181	-0.35141	0.725281	NA
NIPAL3	984.6655	0.026761	0.049042	0.545678	0.585288	0.824332
TTC40	13.22658	-0.02675	0.08483	-0.31533	0.75251	NA
FN3K	261.1102	-0.02675	0.044042	-0.60736	0.543609	NA
ECI2	42.90057	-0.02675	0.0682	-0.39221	0.694901	NA
FAM106A	4.681051	0.026747	0.067999	0.393346	0.694064	NA
TTN-AS1	12.49772	0.026747	0.084533	0.316406	0.751694	NA
HTR1E	13.0538	-0.02675	0.084585	-0.31621	0.751844	NA
RSRC1	209.7117	-0.02674	0.047103	-0.56768	0.570255	NA
GGCX	200.1438	-0.02674	0.053306	-0.50159	0.615954	NA
NAP1L1	885.6205	-0.02674	0.043858	-0.60963	0.542104	0.802527
BAP1	244.1101	0.026731	0.045282	0.590331	0.554969	NA
ITGA2B	29.57439	-0.02672	0.078574	-0.34005	0.733816	NA
CACNA1D	369.0158	0.026712	0.050444	0.529538	0.596433	0.829672
RIF1	1185.024	0.026708	0.031423	0.849942	0.395357	0.705623
LOC33944	0.568806	-0.02671	0.037774	-0.70702	0.479556	NA
UBE2N	144.2691	0.026704	0.056722	0.470786	0.637793	NA
SCN3A	387.0878	-0.0267	0.062576	-0.42671	0.669591	0.861137
RAB37	218.8245	-0.0267	0.064	-0.41719	0.676542	NA
STAT2	262.4901	-0.0267	0.056103	-0.47589	0.634155	NA
SHB	15.78846	-0.0267	0.082914	-0.32197	0.747477	NA
DMRTA2	1.204234	0.026679	0.056944	0.468511	0.639419	NA
C7orf31	27.19762	0.026679	0.078852	0.338345	0.735103	NA
PDE4DIP	4492.009	0.026678	0.049175	0.542497	0.587476	0.825363
RAB20	1.813713	0.026674	0.062731	0.425219	0.670677	NA
PRKCDBP	6.628552	-0.02667	0.084247	-0.31653	0.7516	NA
PITX1	0.76962	0.026663	0.039567	0.67386	0.500401	NA
MIR4733	0.152754	0.026658	0.021836	1.220791	0.222165	NA
DENND6A	218.6704	0.026655	0.040417	0.659491	0.509581	NA
TRMT1	67.53889	0.026655	0.066012	0.403786	0.68637	NA
SNORD116	48.4085	-0.02665	0.084936	-0.31374	0.75372	NA
VASH2	13.47709	0.026645	0.084851	0.31402	0.753506	NA
MIR1256	1.864298	-0.02664	0.068961	-0.38636	0.699229	NA
ZFP41	148.217	-0.02664	0.058175	-0.45791	0.647017	NA
HSPA13	129.5564	0.026639	0.069169	0.385127	0.700143	NA
NT5C1B	0.224311	0.026624	0.023768	1.120121	0.262662	NA
SNORA40	8.385448	-0.02662	0.084792	-0.31396	0.753548	NA
APOF	0.659007	0.02662	0.041332	0.64406	0.519536	NA
MIR2467	1.310531	0.026612	0.054537	0.487959	0.625579	NA
SNHG5	42.83071	-0.02661	0.067552	-0.39388	0.693672	NA

EGR2	1.967668	0.0266	0.064404	0.413025	0.679589	NA
GON4L	610.4577	-0.02659	0.032779	-0.81126	0.417215	0.722472
IFIT1	214.3124	0.026589	0.054636	0.486645	0.62651	NA
UBXN6	242.0647	-0.02659	0.049989	-0.53187	0.594818	NA
RASA2	196.7642	-0.02657	0.051996	-0.51099	0.609356	NA
CARS2	127.4558	0.026567	0.043946	0.604527	0.545493	NA
EMC1	361.439	0.026564	0.037378	0.710666	0.477291	0.759174
TBC1D15	293.4676	0.026556	0.039652	0.669731	0.503029	NA
AKT3	1774.884	0.026552	0.034666	0.765954	0.443704	0.743291
SLC33A1	101.4998	0.026542	0.052492	0.505637	0.613111	NA
FICD	10.7541	-0.02654	0.084891	-0.31266	0.754542	NA
RBM15	241.4748	-0.02654	0.059125	-0.44887	0.653528	NA
WBP5	177.2783	-0.02653	0.060281	-0.44018	0.659804	NA
EPPK1	49.94393	0.026529	0.07429	0.357104	0.721014	NA
POLM	68.74365	-0.02652	0.067927	-0.39035	0.696274	NA
GART	77.44277	0.026512	0.056628	0.468172	0.639662	NA
NEO1	379.8453	0.026509	0.045306	0.585099	0.558481	0.808857
C7orf34	1.18032	-0.02651	0.054656	-0.48497	0.6277	NA
CCDC51	18.0256	0.026505	0.079969	0.331446	0.740308	NA
SNORD96B	1.475065	0.026501	0.059195	0.447694	0.654374	NA
MIR548AD	0.30369	-0.0265	0.032626	-0.81224	0.416653	NA
LOC28636	35.17673	-0.0265	0.073553	-0.36022	0.71868	NA
RAB40B	112.2699	-0.02649	0.058438	-0.45325	0.650366	NA
OR7A5	0.755244	-0.02648	0.044959	-0.58901	0.555855	NA
FLJ44511	11.04866	-0.02648	0.081966	-0.32304	0.746664	NA
MITD1	56.15162	-0.02647	0.072047	-0.36745	0.713284	NA
PPP3R2	0.538748	0.02646	0.036701	0.720952	0.470939	NA
FNDC8	4.580091	0.026455	0.082442	0.320892	0.748293	NA
CXXC1	329.8414	-0.02645	0.065198	-0.40575	0.684929	NA
UBASH3A	0.38018	0.026442	0.03275	0.80739	0.419442	NA
SNORD114	2.793285	-0.02643	0.066911	-0.395	0.692844	NA
PPFIA4	1833.874	-0.02643	0.055157	-0.47912	0.631855	0.844999
KLK4	0.503772	-0.02642	0.042023	-0.62864	0.529585	NA
CCDC90A	146.1401	0.026414	0.065275	0.404653	0.685733	NA
CCDC152	5.430537	-0.02641	0.082225	-0.3212	0.748056	NA
KIAA1731	464.7566	0.026411	0.036926	0.715226	0.474469	0.759174
KRT72	0.376163	-0.02641	0.032776	-0.80565	0.420445	NA
AHNAK2	281.6496	0.026404	0.080166	0.329366	0.741879	NA
NAF1	49.55403	0.026401	0.064329	0.4104	0.681513	NA
DDI1	0.70881	0.026395	0.04188	0.63026	0.528524	NA
PLEKHO1	147.6782	-0.02639	0.057491	-0.45901	0.646228	NA
GLTPD2	18.02027	0.026386	0.08138	0.324234	0.745761	NA
SMPX	6.28605	-0.02638	0.082308	-0.32046	0.74862	NA
ZNF526	69.20657	-0.02637	0.062188	-0.42398	0.671578	NA
MIR155HG	13.60856	-0.02636	0.083239	-0.31671	0.751465	NA
RNASE10	0.111596	-0.02635	0.018122	-1.45413	0.14591	NA
MADD	1303.102	0.026348	0.039768	0.662551	0.507618	0.780812

PIR-FIGF	1.371446	0.026344	0.055188	0.477348	0.633114	NA
LOC10013	7.528306	0.026342	0.084917	0.310212	0.7564	NA
FAM172BP	0.077719	-0.02633	0.016345	-1.611	0.107179	NA
MIR4461	0.648791	-0.02632	0.040395	-0.65168	0.514608	NA
NLRP6	0.195539	0.026316	0.024888	1.057376	0.29034	NA
MIR873	0.605371	0.026309	0.039075	0.673299	0.500757	NA
NOTO	0.602042	0.026304	0.042148	0.624081	0.532575	NA
LOC10028	6.740858	-0.0263	0.084519	-0.31121	0.755641	NA
SNORA70E	0.908942	-0.0263	0.05058	-0.51995	0.603096	NA
CFI	5.22667	0.026298	0.077232	0.340509	0.733473	NA
HNF4G	1.78792	-0.0263	0.065854	-0.39931	0.689664	NA
NOTCH1	90.70631	-0.02629	0.073825	-0.35616	0.721719	NA
NDUFAB1	73.94814	-0.02629	0.079985	-0.32866	0.742413	NA
APOA1	0.985872	0.026282	0.051543	0.5099	0.610121	NA
NUDCD2	23.21035	0.026275	0.076677	0.34267	0.731847	NA
SLC41A2	125.41	0.026268	0.047303	0.555319	0.578676	NA
BDNF	77.11613	0.026266	0.08361	0.314153	0.753405	NA
MIR27A	0.076997	-0.02626	0.016324	-1.60892	0.107633	NA
CYP26B1	80.24716	0.026263	0.084895	0.309358	0.757049	NA
KIF4A	2.215681	-0.02626	0.069845	-0.376	0.706914	NA
PTPRN2	141.6595	-0.02626	0.064893	-0.4046	0.68577	NA
ARPC1A	160.861	0.026255	0.051992	0.504988	0.613567	NA
ZNF101	15.50506	0.026254	0.081945	0.320389	0.748673	NA
SNX29P1	1.350948	-0.02625	0.059474	-0.44138	0.65894	NA
DCTN1-AS	8.07173	0.026249	0.08456	0.310412	0.756248	NA
PPP4R1	232.7533	0.026248	0.043053	0.609672	0.542079	NA
RPS24	296.6828	-0.02625	0.07413	-0.35406	0.723295	NA
MLL	2804.676	0.026241	0.042137	0.622762	0.533441	0.795421
OR9A4	0.187644	-0.02624	0.025005	-1.04918	0.294095	NA
SELE	1.901381	-0.02623	0.035946	-0.72968	0.465587	NA
LOC64543	1.629731	0.026228	0.063058	0.415928	0.677463	NA
FAM83A	0.368189	-0.02623	0.032569	-0.80522	0.420691	NA
CARM1	153.7318	-0.02622	0.057344	-0.45725	0.647492	NA
SUPT20HL	0.152891	-0.02622	0.022857	-1.14692	0.251413	NA
LINC00052	0.075396	-0.02621	0.016307	-1.60728	0.107994	NA
SNRNP40	87.02499	-0.0262	0.050355	-0.52035	0.602821	NA
BOD1	92.73935	-0.0262	0.062021	-0.42247	0.672684	NA
ZNF222	59.10019	-0.02619	0.056047	-0.46729	0.640289	NA
CTSL2	0.481405	0.02619	0.037383	0.700598	0.483554	NA
SPPL2C	27.96797	0.02619	0.082767	0.31643	0.751676	NA
SEMA6A	467.7452	0.026189	0.05281	0.495916	0.619954	0.83983
CTSA	103.9387	-0.02619	0.058258	-0.44954	0.653044	NA
PSD	160.3282	-0.02618	0.051497	-0.50846	0.611131	NA
SVEP1	456.3455	-0.02617	0.066349	-0.39441	0.693279	0.871418
SEC13	104.3942	0.026168	0.054084	0.48385	0.628493	NA
CCDC126	36.64083	0.026167	0.069665	0.375608	0.707209	NA
ZNF528	236.9268	0.026165	0.056458	0.463442	0.643047	NA

RAD23A	195.2039	-0.02616	0.057947	-0.45148	0.651646	NA
NEDD4L	671.2206	0.02616	0.055353	0.472599	0.636499	0.846919
MIR193A	0.141669	0.026157	0.021717	1.204474	0.228406	NA
THOC3	20.95047	0.026149	0.082499	0.316964	0.751271	NA
SNORD116	3.457622	-0.02615	0.076226	-0.34302	0.731582	NA
N4BP3	87.24162	0.026146	0.071077	0.367847	0.712987	NA
EPC2	428.5085	0.026136	0.040006	0.653302	0.513561	0.783839
MIR1284	1.360271	-0.02614	0.05331	-0.49025	0.623959	NA
MIR5687	1.536933	0.026133	0.058969	0.443167	0.657645	NA
TPK1	9.018768	-0.02613	0.084854	-0.3079	0.758162	NA
FAM167B	0.745057	0.02612	0.045523	0.573768	0.566125	NA
ISPD	59.35135	0.026114	0.065454	0.398964	0.68992	NA
ERGIC2	124.7198	-0.02611	0.045012	-0.58	0.561916	NA
ABCC9	20.65918	-0.0261	0.08323	-0.31362	0.753811	NA
SLC45A2	1.957014	-0.02609	0.069075	-0.3777	0.705651	NA
DNAH9	15.34168	0.026081	0.084409	0.308976	0.75734	NA
LOC64292	0.18576	0.02608	0.020673	1.261589	0.207097	NA
OR9A2	1.043385	0.026079	0.050834	0.513024	0.607934	NA
RGL4	185.8591	-0.02608	0.061859	-0.42155	0.67335	NA
MSH3	160.8412	-0.02607	0.04689	-0.55603	0.578187	NA
CHDC2	0.439615	-0.02607	0.036454	-0.71507	0.474565	NA
S100A5	1.367692	-0.02606	0.051424	-0.50686	0.612252	NA
RUNX3	1.598572	-0.02606	0.058859	-0.44268	0.657995	NA
LOC10028	4.485006	0.02605	0.081547	0.319451	0.749385	NA
SPRN	102.5351	0.026033	0.063071	0.412754	0.679787	NA
KIAA0753	103.0203	-0.02603	0.052678	-0.49409	0.621242	NA
LRRC4B	278.0879	-0.02602	0.051133	-0.50885	0.610857	NA
TIGD5	33.01685	0.026016	0.077947	0.333757	0.738563	NA
GUCY2F	0.142089	-0.02601	0.022718	-1.14488	0.252258	NA
DDX19A	209.9693	-0.02601	0.042925	-0.60591	0.544577	NA
PACRG-AS	1.265331	-0.02601	0.058756	-0.44265	0.65802	NA
PRKD1	26.58684	-0.026	0.081627	-0.31857	0.750051	NA
MIR4279	2.273467	-0.02599	0.070361	-0.36945	0.711793	NA
PLXNC1	294.0811	-0.02599	0.048498	-0.53591	0.592023	NA
SKIL	127.2404	0.025987	0.064854	0.400705	0.688637	NA
C10orf67	0.310317	-0.02598	0.029758	-0.87317	0.382569	NA
NHLH1	3.580356	-0.02598	0.065545	-0.39642	0.691798	NA
BTN2A1	306.3049	-0.02598	0.053908	-0.48196	0.629834	NA
NPB	2.889053	-0.02598	0.075767	-0.34289	0.731679	NA
TAS2R38	0.255495	-0.02596	0.029062	-0.89315	0.371775	NA
ANP32A	307.4347	0.025952	0.057003	0.455266	0.648918	NA
KIAA0930	249.5353	0.025948	0.076009	0.341387	0.732813	NA
SMIM14	72.37347	0.025933	0.076917	0.337156	0.735999	NA
RPN1	217.4675	-0.02593	0.06146	-0.42195	0.673064	NA
LOC10013	3.781333	0.02593	0.06615	0.39198	0.695073	NA
DCAF7	250.2424	0.025929	0.04874	0.531991	0.594732	NA
KIAA1958	195.2315	-0.02593	0.063404	-0.4089	0.682613	NA

EXOC1	165.0928	-0.02592	0.056112	-0.46199	0.644086	NA
CYP8B1	0.846429	-0.02592	0.048528	-0.53419	0.593211	NA
C20orf196	7.562979	-0.02592	0.08492	-0.30524	0.760183	NA
IL13	0.324185	-0.02592	0.031603	-0.82008	0.412173	NA
GFOD1	452.8533	-0.02591	0.053333	-0.48587	0.627057	0.843303
HRH1	1.155824	-0.02591	0.054788	-0.47292	0.636269	NA
MPDZ	963.4196	0.025905	0.059283	0.436976	0.662129	0.855957
CACNA1C-	18.80895	-0.02591	0.084749	-0.30567	0.759856	NA
HTR3E	4.368898	0.025905	0.080028	0.323695	0.746169	NA
LOC10050	17.83946	0.025898	0.083167	0.311392	0.755503	NA
DDX11L10	0.093372	-0.02589	0.017697	-1.46296	0.14348	NA
C19orf52	21.78409	0.025885	0.078701	0.328907	0.742226	NA
SPPL3	114.7156	-0.02588	0.050886	-0.50865	0.610996	NA
MIR4740	2.196014	-0.02588	0.067693	-0.38236	0.702194	NA
ZSCAN1	33.71351	0.025881	0.077169	0.335387	0.737333	NA
ZSCAN9	54.23491	0.025874	0.064101	0.40365	0.68647	NA
TPRA1	48.47354	0.025866	0.064771	0.399341	0.689642	NA
SNORA38	0.555493	-0.02586	0.036811	-0.70253	0.482351	NA
CCIN	2.060702	-0.02586	0.066981	-0.38604	0.699466	NA
ALDH1A3	38.99372	-0.02586	0.084524	-0.3059	0.759682	NA
OR52E8	0.104855	-0.02585	0.017971	-1.4387	0.150235	NA
FBXO31	708.2323	-0.02585	0.058006	-0.44569	0.655822	0.854402
CCDC73	25.5475	0.025852	0.071505	0.361538	0.717697	NA
ABP1	0.090257	-0.02585	0.017684	-1.46183	0.143788	NA
AIFM1	62.15745	-0.02585	0.066982	-0.38593	0.699545	NA
PLA2G2A	0.106902	-0.02584	0.018102	-1.42763	0.153398	NA
TXK	0.611666	0.025841	0.041147	0.628009	0.529998	NA
EPX	9.701339	-0.02584	0.084325	-0.30644	0.759273	NA
UTP14A	74.44602	0.025833	0.058396	0.44237	0.658222	NA
CECR3	0.61248	0.025832	0.041211	0.626829	0.530771	NA
ZNF324B	29.55122	-0.02583	0.075406	-0.34253	0.731954	NA
TENM3	192.4431	-0.02583	0.059764	-0.43216	0.665623	NA
NOVA1	741.0726	0.025815	0.040056	0.644482	0.519263	0.785659
LOC10050	10.71502	-0.02581	0.08463	-0.30499	0.760375	NA
VPS53	1295.925	0.0258	0.046682	0.552677	0.580485	0.822777
FAM83E	0.841569	-0.02579	0.048607	-0.53063	0.595676	NA
DYNC1I2	392.8406	-0.02577	0.037081	-0.6951	0.486991	0.764934
TMEM199	35.7802	-0.02576	0.06982	-0.36897	0.71215	NA
CDC42EP2	11.02491	-0.02576	0.084539	-0.30467	0.760619	NA
LINC00158	16.60939	0.025755	0.084598	0.304434	0.760797	NA
FGFR3	289.0511	-0.02575	0.079505	-0.32389	0.746019	NA
B3GNT2	17.74611	-0.02575	0.083254	-0.3093	0.757094	NA
GGN	6.733627	-0.02575	0.081374	-0.31643	0.751679	NA
SERPINE3	7.210938	0.025732	0.082936	0.310266	0.756359	NA
RBM12B	365.5091	0.025728	0.053593	0.48006	0.631184	0.844999
PIBF1	149.0456	0.025728	0.056976	0.45155	0.651593	NA
MIR138-2	0.107172	-0.02573	0.017957	-1.43265	0.151958	NA

RPUSD2	16.65281	0.025724	0.080868	0.318105	0.750405	NA
ZNF253	183.2912	0.02572	0.062421	0.412048	0.680304	NA
SPATA31C	0.827946	0.02572	0.048304	0.532449	0.594415	NA
PPP1R3E	175.9157	-0.02571	0.059542	-0.43184	0.665859	NA
LOC65434	4.45928	0.025707	0.07801	0.329532	0.741753	NA
CYP26A1	0.287033	-0.0257	0.029689	-0.86576	0.386624	NA
TSPAN6	13.06773	-0.02569	0.08487	-0.30269	0.762127	NA
ACOT1	7.691992	-0.02568	0.078209	-0.32837	0.742629	NA
NFASC	1169.386	-0.02568	0.044086	-0.58249	0.560239	0.810308
VTI1B	134.9622	-0.02568	0.044668	-0.5748	0.565429	NA
GPC1	251.3651	-0.02567	0.058337	-0.44011	0.65986	NA
TPTE2P6	1.196638	0.025672	0.053153	0.482991	0.629102	NA
VPS4B	126.8613	-0.02567	0.065814	-0.39004	0.696509	NA
SMPDL3B	9.676384	0.02567	0.080479	0.318958	0.749759	NA
NR0B2	0.163606	-0.02566	0.023114	-1.1103	0.266869	NA
TBC1D10A	22.5879	0.025646	0.07483	0.342726	0.731805	NA
SYF2	142.7934	-0.02565	0.057493	-0.44606	0.655556	NA
C9orf142	37.78646	0.025645	0.074506	0.344193	0.730701	NA
DHX57	144.1377	0.025641	0.045504	0.563496	0.573097	NA
ERVFRD-1	41.82442	0.025635	0.068053	0.376692	0.706402	NA
ZNF532	1028.475	-0.02563	0.042548	-0.60248	0.546854	0.803779
NXN	51.86058	-0.02563	0.080322	-0.31912	0.749637	NA
RAB42	1.938356	0.025626	0.065357	0.392092	0.69499	NA
DNAJC25	52.59267	-0.02563	0.060598	-0.42288	0.67238	NA
C15orf53	0.268934	0.025625	0.02817	0.90964	0.363012	NA
OR2M2	0.101082	-0.02562	0.017895	-1.43164	0.152247	NA
UQCRC2	282.0133	-0.02562	0.047485	-0.53947	0.589563	NA
LOC65036	0.581144	0.025616	0.037017	0.692011	0.48893	NA
PDSS2	39.47273	-0.02561	0.067266	-0.38078	0.703365	NA
RGS7	328.3359	0.025612	0.046569	0.549993	0.582324	NA
ERMN	183.8062	-0.0256	0.08491	-0.3015	0.763032	NA
PKHD1	0.142746	0.0256	0.021804	1.174081	0.240363	NA
DIRAS3	1.163299	0.025594	0.053775	0.475957	0.634105	NA
MIRLET7A	0.145124	-0.02559	0.022759	-1.12457	0.260771	NA
MDM1	77.91958	0.025586	0.054877	0.466249	0.641037	NA
MRPS5	327.8989	0.025583	0.06538	0.391302	0.695574	NA
CASC3	758.4314	0.025583	0.030842	0.829488	0.406828	0.710565
SFXN3	234.0395	-0.02558	0.050752	-0.50395	0.6143	NA
DAGLA	103.4216	-0.02557	0.077666	-0.32929	0.741938	NA
QTRTD1	25.21229	-0.02557	0.078858	-0.32426	0.745743	NA
RIMKLA	123.3256	0.025563	0.071374	0.358156	0.720227	NA
MLXIP	277.8147	-0.02556	0.050014	-0.51107	0.609301	NA
KRT18P55	0.790079	0.025554	0.04766	0.536164	0.591845	NA
RNF185	54.01679	0.025552	0.058037	0.440273	0.659739	NA
SLC15A1	0.119114	-0.02555	0.016694	-1.53052	0.125888	NA
RARRES3	4.277722	-0.02555	0.073406	-0.348	0.72784	NA
RSPO2	8.797497	0.025545	0.075228	0.339567	0.734183	NA

PGBD1	89.98399	0.025544	0.062365	0.409588	0.682108	NA
SNORD69	2.875705	-0.02554	0.074593	-0.34235	0.732091	NA
SNX5	147.2557	0.025526	0.044149	0.578191	0.563135	NA
NDUFA3	31.42121	-0.02552	0.07485	-0.34098	0.733119	NA
TROAP	0.382066	-0.02552	0.033926	-0.7522	0.451933	NA
EIF3A	1103.47	-0.02551	0.044607	-0.57198	0.567333	0.814844
VIL1	2.882278	-0.0255	0.074395	-0.34273	0.731801	NA
CCDC134	48.42768	0.025493	0.068411	0.372652	0.709408	NA
PSMG3-AS	40.79614	-0.02549	0.084665	-0.30107	0.763359	NA
TRIM34	0.089616	-0.02549	0.017746	-1.43617	0.150953	NA
RAB35	90.93152	-0.02548	0.059337	-0.42945	0.667599	NA
FOPNL	73.7438	0.025479	0.054971	0.463496	0.643009	NA
RNASE1	25.82734	-0.02547	0.084873	-0.30013	0.76408	NA
GSDMA	0.218657	0.025471	0.028427	0.895995	0.370255	NA
ARHGAP33	123.5102	0.025471	0.060688	0.419698	0.674706	NA
MPHOSPH	446.3741	0.02547	0.04782	0.532622	0.594295	0.829672
RB1CC1	646.6424	0.025464	0.033159	0.767941	0.442523	0.743005
GUCY2C	1.971987	-0.02546	0.064242	-0.39637	0.691829	NA
PFN2	352.7391	0.025453	0.072637	0.35041	0.726031	0.891306
KIAA1210	0.274917	0.025449	0.031456	0.809043	0.41849	NA
PELI3	124.7955	-0.02545	0.056309	-0.45192	0.651329	NA
XCL2	0.325266	-0.02544	0.033166	-0.7672	0.442962	NA
SNORD28	5.885988	-0.02543	0.080221	-0.31705	0.751204	NA
KCND3	269.5834	0.025427	0.058615	0.433798	0.664435	NA
GRB14	0.844415	0.025425	0.049132	0.517489	0.604815	NA
LIG3	154.2325	-0.02541	0.057326	-0.44328	0.657566	NA
SENP2	205.7514	0.025409	0.042308	0.600564	0.548131	NA
LIMK1	95.71358	0.025403	0.074532	0.340832	0.73323	NA
SBF1	677.3166	-0.0254	0.046296	-0.5487	0.58321	0.824332
ZDHHC6	107.5823	-0.0254	0.051356	-0.49462	0.620866	NA
MIR4318	0.942252	0.025399	0.051973	0.4887	0.625054	NA
LOC10013	0.311161	0.025391	0.032965	0.770236	0.44116	NA
APBA3	34.43814	-0.02539	0.074508	-0.34075	0.733292	NA
B3GNT8	11.52095	-0.02538	0.084673	-0.29979	0.764341	NA
SCARNA21	67.12756	-0.02538	0.080498	-0.31526	0.752566	NA
PSG5	17.78264	0.025376	0.083561	0.303684	0.761368	NA
SNORD114	1.228323	-0.02537	0.057383	-0.44205	0.658456	NA
NCOR1	1981.715	0.025362	0.030841	0.822346	0.41088	0.715365
GUSBP11	250.6895	-0.02535	0.06119	-0.41436	0.678612	NA
MIR3128	0.582452	0.025354	0.042055	0.602888	0.546583	NA
FOXN4	1.825277	0.025352	0.067969	0.372998	0.70915	NA
IL6R	22.51466	-0.02535	0.084624	-0.29956	0.764516	NA
UGP2	257.0105	0.025337	0.058263	0.43488	0.66365	NA
VPS37C	34.57504	0.025335	0.067852	0.373393	0.708856	NA
MLL5	1868.338	0.025331	0.035562	0.712319	0.476268	0.759174
TJP1	584.0335	-0.02533	0.048951	-0.51747	0.604825	0.831231
CAPN11	0.165624	-0.02533	0.023194	-1.09207	0.2748	NA

MST1P2	5.4371	0.025325	0.080932	0.312917	0.754343	NA
PAM	280.4511	0.025316	0.070098	0.361156	0.717983	NA
IKZF5	244.3271	-0.02532	0.042773	-0.59186	0.553944	NA
P2RX7	192.6326	0.025299	0.083555	0.302783	0.762055	NA
ISM1-AS1	0.314105	-0.02529	0.028703	-0.88123	0.378194	NA
FAM168A	209.2209	0.025293	0.048563	0.520836	0.602481	NA
MINOS1	58.3645	-0.02529	0.07504	-0.33703	0.736091	NA
GMCL1	72.70762	-0.02529	0.056092	-0.45086	0.65209	NA
NBEAL2	150.1741	0.025288	0.076706	0.329676	0.741645	NA
IDH3A	326.7614	0.025287	0.065756	0.384559	0.700565	NA
ZSWIM6	172.72	-0.02529	0.040554	-0.62351	0.532947	NA
SYS1	87.24985	0.02528	0.062697	0.403211	0.686793	NA
NKX1-2	0.155371	-0.02527	0.023011	-1.09833	0.27206	NA
SLC26A9	2.349538	0.025267	0.066323	0.380973	0.703223	NA
TPX2	15.00144	0.025259	0.084847	0.297696	0.765935	NA
CACNB3	117.371	0.025256	0.063218	0.399511	0.689517	NA
CCL27	3.467953	-0.02525	0.076809	-0.32872	0.742368	NA
WDR31	12.63552	-0.02525	0.084703	-0.29808	0.765642	NA
RBM46	0.217022	0.025248	0.023678	1.066329	0.286275	NA
NMI	6.08533	-0.02524	0.075725	-0.33329	0.738918	NA
TM4SF5	0.161215	-0.02524	0.023122	-1.09137	0.275112	NA
LOC10013	0.416266	0.025217	0.035966	0.701146	0.483212	NA
RXRG	1.458544	-0.02522	0.061624	-0.40918	0.682405	NA
EXO1	0.957931	-0.02521	0.04863	-0.51844	0.604154	NA
ASF1A	85.64636	0.025207	0.058922	0.427807	0.668792	NA
TWF1	54.52345	0.025205	0.074537	0.338155	0.735246	NA
DDX46	631.5886	-0.0252	0.030387	-0.82938	0.406889	0.710565
GLTPD1	27.18604	0.025198	0.073495	0.342852	0.73171	NA
HSPB1	145.4855	-0.0252	0.076689	-0.32856	0.74249	NA
DNMT1	655.1101	-0.02519	0.051381	-0.49029	0.623929	0.841191
ZG16	0.192961	-0.02519	0.025006	-1.00737	0.313756	NA
LINC00324	4.729273	0.025188	0.08089	0.311383	0.75551	NA
SYCP1	1.11119	0.025187	0.051036	0.493503	0.621657	NA
TCTN3	31.57924	-0.02517	0.067436	-0.37331	0.708921	NA
KALRN	152.1818	-0.02517	0.067216	-0.37452	0.708016	NA
C3orf38	80.60007	0.025169	0.062289	0.404074	0.686158	NA
SNORD114	1.164076	-0.02516	0.056953	-0.44184	0.658604	NA
PRB4	0.732557	-0.02516	0.046806	-0.53756	0.590881	NA
LGALS9B	0.353696	0.02516	0.029928	0.840703	0.400514	NA
C21orf33	153.0691	0.02516	0.045036	0.558661	0.576393	NA
CFL1	430.5343	0.025158	0.067202	0.374365	0.708133	0.883299
ZFAND2A	33.17493	-0.02515	0.071074	-0.35383	0.723463	NA
PLEKHH3	50.84881	0.025138	0.069485	0.361784	0.717514	NA
NDUFS8	33.96338	-0.02513	0.075898	-0.33104	0.740611	NA
KEAP1	83.07087	0.025113	0.057074	0.440015	0.659926	NA
KLHL26	60.39581	0.025099	0.065059	0.385789	0.699653	NA
MKNK2	300.3347	-0.02509	0.060477	-0.41494	0.678184	NA

HIPK2	1449.795	-0.02509	0.050695	-0.49486	0.6207	0.839998
EFNA2	2.796798	0.025086	0.070495	0.355861	0.721945	NA
DRD1	3.631214	-0.02509	0.071991	-0.34846	0.727497	NA
LOC28629	0.225768	-0.02508	0.026781	-0.9366	0.348967	NA
NDUFC2	31.57075	-0.02508	0.073178	-0.34273	0.731802	NA
SLC37A3	77.48196	-0.02508	0.060026	-0.41782	0.67608	NA
PHOSPHO	3.597094	-0.02508	0.075996	-0.33	0.741398	NA
ACRV1	0.699227	-0.02507	0.043176	-0.58074	0.561416	NA
CNGA1	9.402019	0.025072	0.084857	0.295466	0.767638	NA
RCN1	69.61582	0.025069	0.070009	0.358082	0.720282	NA
C2CD4C	2.425484	0.025065	0.072395	0.346219	0.729178	NA
VTI1A	267.1434	0.025064	0.047371	0.529102	0.596735	NA
FLJ30838	4.112916	0.025059	0.079717	0.314352	0.753254	NA
PLB1	5.517008	0.025053	0.080365	0.311745	0.755234	NA
SUSD3	0.771466	-0.02505	0.047565	-0.52666	0.598429	NA
EIF4A2	1712.934	-0.02505	0.078134	-0.32059	0.748519	0.901327
NUDT16	276.0814	-0.02505	0.045367	-0.55213	0.58086	NA
FAM156A	2.757476	0.025046	0.072656	0.344725	0.730301	NA
LOC72917	0.510531	-0.02504	0.038488	-0.65071	0.515232	NA
IQCF5	0.430114	0.025041	0.030994	0.807907	0.419144	NA
LRRC10	0.374241	0.025037	0.036261	0.690462	0.489904	NA
MLNR	1.280204	-0.02503	0.057349	-0.43653	0.662455	NA
SEL1L	429.197	0.025031	0.056192	0.445464	0.655984	0.854402
XAB2	164.2358	-0.02502	0.049429	-0.50617	0.612737	NA
FMR1-AS1	3.759332	-0.02502	0.07972	-0.31383	0.753653	NA
MIR3180-4	0.656753	-0.02502	0.046672	-0.53598	0.591976	NA
PLK2	9.583004	-0.02501	0.08481	-0.29487	0.768097	NA
CNGA4	3.283527	0.025004	0.075352	0.331832	0.740016	NA
ANO3	1.006941	0.025001	0.049526	0.504795	0.613703	NA
MECR	42.39873	-0.02499	0.071362	-0.35025	0.726152	NA
LIG1	156.0779	-0.02498	0.076791	-0.32536	0.744907	NA
PIEZO2	27.64195	0.024978	0.081652	0.305907	0.759675	NA
YARS2	47.02798	0.024977	0.062529	0.399449	0.689562	NA
LOC44202	1.454802	-0.02496	0.055589	-0.44906	0.65339	NA
TUBGCP4	183.3554	0.024961	0.043199	0.577805	0.563396	NA
TCTEX1D4	1.884608	0.024959	0.06764	0.369003	0.712126	NA
JPH4	778.0525	-0.02496	0.056566	-0.44124	0.659039	0.855675
SNORA34	1.442294	-0.02495	0.06111	-0.40833	0.683034	NA
CTNND2	837.3624	0.024952	0.037146	0.671723	0.50176	0.778081
SLC22A15	166.3538	-0.02495	0.061637	-0.40481	0.685618	NA
CABP1	90.17955	0.024946	0.058035	0.429842	0.667311	NA
ANKRD54	66.04122	0.024942	0.066274	0.376342	0.706663	NA
KRTAP5-3	0.300352	0.024926	0.030628	0.813814	0.415752	NA
MIR195	1.188272	-0.02492	0.054827	-0.45456	0.649425	NA
IL17C	0.319673	-0.02492	0.031012	-0.80364	0.421606	NA
POLR2I	95.58028	0.024912	0.071042	0.350662	0.725842	NA
ZSCAN32	54.4631	-0.02491	0.060457	-0.412	0.680336	NA

TFE3	146.7115	0.024908	0.043564	0.57176	0.567485	NA
LINC00278	0.313205	-0.02491	0.028094	-0.88658	0.375306	NA
PPIE	83.02468	0.024903	0.04856	0.512828	0.608072	NA
SLAIN1	331.2097	0.024902	0.052247	0.476621	0.633632	NA
FAM162B	1.783632	0.024896	0.06646	0.374603	0.707956	NA
RDX	363.3552	-0.0249	0.046774	-0.53225	0.594552	0.829672
RIOK3	146.2905	-0.02489	0.041339	-0.6022	0.547041	NA
MCM8	147.8033	0.024893	0.083643	0.297614	0.765998	NA
LOC93432	0.199248	-0.02488	0.023744	-1.04805	0.294617	NA
SLC36A2	0.462945	-0.02488	0.035165	-0.70761	0.479185	NA
LMAN2L	65.28064	0.024883	0.070617	0.352357	0.72457	NA
CCDC7	258.9847	-0.02487	0.06381	-0.38976	0.696713	NA
COMP	22.00551	-0.02486	0.06442	-0.38597	0.699522	NA
SQLE	69.06768	0.024862	0.064416	0.385955	0.69953	NA
MIR7-3HG	22.16922	-0.02486	0.082734	-0.30044	0.763841	NA
MGRN1	519.0631	-0.02485	0.070706	-0.35139	0.725294	0.891227
FP588	103.2491	-0.02484	0.068572	-0.3623	0.717129	NA
TFRC	233.8035	-0.02484	0.053824	-0.46152	0.644425	NA
ABHD8	68.05949	0.024841	0.064896	0.382777	0.701885	NA
FOXQ1	15.01674	-0.02482	0.084856	-0.29253	0.76988	NA
HTR3C	0.380434	0.024814	0.034756	0.713945	0.475261	NA
CIAO1	213.037	-0.02481	0.037643	-0.6592	0.509769	NA
RDH11	128.303	-0.02481	0.050747	-0.48889	0.624916	NA
SAR1A	201.5592	0.024807	0.049402	0.502149	0.615562	NA
MIR617	3.08325	0.024807	0.07719	0.32138	0.747923	NA
ZNF471	135.0051	0.024788	0.066287	0.373952	0.70844	NA
DENND4A	942.8012	-0.02478	0.031661	-0.78274	0.433777	0.736097
TAS2R39	3.791308	0.024782	0.078764	0.314635	0.753039	NA
ELMO3	32.52001	0.024781	0.07418	0.33407	0.738327	NA
MDC1	490.6272	-0.02478	0.044485	-0.55702	0.577512	0.821469
FAM157A	2.04242	0.024773	0.066341	0.373416	0.708839	NA
GTF2I	514.3187	0.024772	0.043926	0.563937	0.572797	0.818024
ATL1	170.5712	0.024771	0.04659	0.531692	0.59494	NA
CCDC74A	3.323414	-0.02475	0.078017	-0.3173	0.751017	NA
OSBPL7	54.18324	-0.02474	0.071265	-0.34721	0.728436	NA
PGM2	55.92247	0.024736	0.068345	0.361929	0.717405	NA
CDH12	47.96769	0.024732	0.06631	0.372977	0.709166	NA
SERP2	20.83908	-0.02473	0.080236	-0.30823	0.757907	NA
C7orf63	31.57758	-0.02472	0.073101	-0.33812	0.735276	NA
GLT8D1	58.6232	-0.02471	0.058252	-0.42422	0.671405	NA
SGK110	0.581987	0.024711	0.039667	0.622952	0.533316	NA
C17orf107	23.50282	0.024704	0.081737	0.302238	0.762471	NA
FFAR4	0.603388	0.0247	0.039502	0.625287	0.531783	NA
MIR548H4	16.38977	0.024695	0.084299	0.292947	0.769562	NA
FAM193A	474.1398	-0.02469	0.045616	-0.54136	0.58826	0.825504
AASDH	126.1151	-0.02469	0.053187	-0.46423	0.642486	NA
C17orf70	94.29882	-0.02469	0.068133	-0.36239	0.71706	NA

NANOG	0.341259	-0.02468	0.033405	-0.7389	0.45997	NA
MDM4	656.3011	-0.02468	0.039284	-0.62821	0.529867	0.793224
KCTD17	27.24063	-0.02467	0.072195	-0.34177	0.732521	NA
POLR3K	28.1592	0.024671	0.076842	0.32106	0.748165	NA
FGF22	2.34376	0.024658	0.067312	0.366318	0.714127	NA
CTSB	485.8167	-0.02465	0.052973	-0.46539	0.641654	0.848776
SSTR5	0.104028	-0.02464	0.016551	-1.48883	0.136532	NA
NDEL1	221.1166	0.024638	0.046605	0.528658	0.597043	NA
RPA1	160.8147	-0.02464	0.05573	-0.44209	0.658421	NA
LOC10050	2.09303	-0.02462	0.062122	-0.39637	0.691834	NA
MCU	284.368	-0.02462	0.050014	-0.49217	0.622602	NA
FH	86.08198	-0.02461	0.077977	-0.31567	0.752254	NA
RSPH3	77.85231	-0.02461	0.060084	-0.40956	0.68213	NA
ZNF765	42.17562	-0.02461	0.0785	-0.31344	0.753943	NA
MYO16-AS	0.493814	-0.0246	0.040141	-0.61288	0.539954	NA
ZNF354C	119.733	0.024596	0.051799	0.474831	0.634908	NA
PSG2	5.441494	-0.02459	0.080599	-0.30514	0.760259	NA
DPEP2	1.84173	-0.02459	0.066004	-0.37253	0.7095	NA
LOC28544	3.710376	-0.02458	0.07039	-0.34916	0.726972	NA
TRAPPC9	251.4686	-0.02458	0.06332	-0.38813	0.697923	NA
TMEM212	0.084252	-0.02457	0.01777	-1.3829	0.166694	NA
LY6K	0.496856	-0.02457	0.038091	-0.6451	0.518861	NA
NXF1	324.3194	0.024567	0.061954	0.396544	0.691704	NA
NOG	1.659448	0.02456	0.060586	0.405378	0.6852	NA
RPS16P5	56.41979	-0.02455	0.075045	-0.32709	0.7436	NA
RBP5	5.845289	-0.02454	0.080577	-0.30458	0.760688	NA
SLC25A17	32.28984	0.02454	0.070496	0.348101	0.727764	NA
NR1I3	10.7078	0.024538	0.084934	0.288906	0.772653	NA
KCNQ5-AS	0.390427	-0.02453	0.036722	-0.66806	0.504098	NA
ASIC4	34.19594	0.02453	0.084725	0.289518	0.772185	NA
CASC2	19.34981	0.024527	0.082946	0.295698	0.767461	NA
RHOG	13.02916	-0.02453	0.083947	-0.29216	0.770165	NA
GATSL2	2.172995	-0.02453	0.06925	-0.35415	0.723223	NA
PHF3	2015.171	-0.02452	0.048006	-0.51085	0.609458	0.833588
RAPGEFL1	90.81403	-0.02451	0.058964	-0.41575	0.677589	NA
NBAS	484.8575	0.024509	0.040881	0.599528	0.548821	0.803817
OTUD5	270.9651	-0.0245	0.038951	-0.62906	0.529313	NA
LARS2	83.76168	0.024502	0.054688	0.448033	0.654129	NA
CEACAM1	0.505874	-0.0245	0.036946	-0.66312	0.507253	NA
ZNF503-AS	0.199893	-0.0245	0.02715	-0.90231	0.366894	NA
SIK3	622.289	0.024496	0.040967	0.597941	0.549879	0.804572
CENPI	2.950366	-0.02449	0.075961	-0.32241	0.747139	NA
SLN	2.196594	-0.02449	0.057582	-0.4253	0.670617	NA
MFSD5	7.994486	0.024486	0.084921	0.28834	0.773086	NA
ZNF33A	674.7725	0.024483	0.031688	0.772644	0.439733	0.74018
METAP1D	16.82202	-0.02448	0.080365	-0.30459	0.76068	NA
ENO1-AS1	0.182595	-0.02448	0.027012	-0.90614	0.364862	NA

NGF	0.116346	-0.02448	0.018034	-1.3572	0.174719	NA
ARFRP1	108.6224	0.024464	0.061261	0.399345	0.689639	NA
MIR3622B	0.090057	-0.02446	0.017828	-1.37216	0.170013	NA
DBX1	0.096083	0.024458	0.017819	1.372614	0.169872	NA
RGS22	14.39448	0.024453	0.084939	0.287883	0.773437	NA
NUDT19	27.64341	-0.02443	0.072653	-0.3363	0.736642	NA
MED27	19.29857	-0.02443	0.07994	-0.30557	0.759934	NA
CSNK1G1	126.0131	0.024423	0.054568	0.447567	0.654465	NA
ZNF48	44.53393	0.024422	0.068271	0.357724	0.72055	NA
GPR156	1.242953	0.024421	0.057985	0.421158	0.67364	NA
INPP4B	60.7415	-0.02442	0.08034	-0.30392	0.761186	NA
NHLRC3	97.82901	-0.02442	0.064336	-0.37951	0.704307	NA
TCEB3	253.6079	0.024414	0.050452	0.483907	0.628452	NA
HPS4	291.119	-0.02441	0.054603	-0.44709	0.654807	NA
SPATA17	1.325309	-0.02441	0.053665	-0.45483	0.649234	NA
EHMT2	259.7589	-0.02441	0.047768	-0.51095	0.609387	NA
TACC1	576.4812	-0.0244	0.036893	-0.66142	0.508342	0.781136
LOC40146	0.510677	-0.0244	0.039875	-0.61194	0.540575	NA
LRRC3C	0.097699	0.024399	0.017818	1.369359	0.170887	NA
FXYD3	6.410942	-0.02439	0.082086	-0.29714	0.766362	NA
CCDC116	3.97922	0.024389	0.078009	0.312644	0.754551	NA
MIR4529	0.803627	-0.02438	0.045086	-0.54077	0.588664	NA
CYP4F35P	0.448152	0.024379	0.03677	0.663012	0.507323	NA
CXCL10	1.040887	0.024378	0.021172	1.151395	0.24957	NA
TPM1	389.174	0.024371	0.056098	0.434443	0.663967	0.856915
TSPO2	2.505942	-0.02437	0.06791	-0.35883	0.71972	NA
GPR6	0.299126	-0.02436	0.032636	-0.74638	0.455437	NA
FLJ20021	14.66245	0.024359	0.084663	0.287718	0.773563	NA
CYMP	1.798735	0.024351	0.064289	0.378771	0.704858	NA
PDCD1LG2	3.471538	-0.02435	0.076959	-0.31641	0.751693	NA
LHB	3.210565	0.02435	0.074441	0.327104	0.74359	NA
NCKAP5L	96.49092	-0.02435	0.066064	-0.36855	0.712462	NA
CYP2S1	0.580052	-0.02435	0.034839	-0.69887	0.484636	NA
FAM222A	82.39789	0.024343	0.078461	0.310253	0.756368	NA
SGPP2	46.6957	0.024337	0.061974	0.392694	0.694546	NA
SMG7-AS1	2.311414	0.024336	0.072067	0.337687	0.735599	NA
MALT1	181.0685	0.024332	0.052063	0.467359	0.640243	NA
POLR2H	34.64466	-0.02433	0.068274	-0.35634	0.721582	NA
OR3A4P	0.195295	-0.02433	0.023688	-1.02695	0.304445	NA
PARP9	25.25751	-0.02431	0.083715	-0.29039	0.771519	NA
INO80E	43.36014	-0.02431	0.064423	-0.37732	0.705934	NA
EPS8L3	0.889321	-0.0243	0.049607	-0.48993	0.624182	NA
ZNF565	43.93423	0.024297	0.06982	0.347992	0.727846	NA
C8orf44-S	1.995263	-0.0243	0.068928	-0.35249	0.724473	NA
PCNT	953.1757	-0.02429	0.059141	-0.41069	0.681301	0.866937
PAR5	1247.025	0.02428	0.05092	0.476828	0.633485	0.845517
IL23A	1.243291	0.024273	0.058181	0.417194	0.676536	NA

ASB11	0.272491	-0.02427	0.029759	-0.81565	0.414702	NA
RFK	71.1812	-0.02427	0.073476	-0.33033	0.74115	NA
TRMT10C	56.26588	-0.02427	0.058404	-0.4155	0.677775	NA
TAF15	381.3723	0.024259	0.033203	0.730625	0.465008	0.755844
KCNV1	0.271498	-0.02426	0.028253	-0.85855	0.390589	NA
CYHR1	116.1381	-0.02425	0.059898	-0.40491	0.685544	NA
GLB1L3	65.90757	0.02424	0.076038	0.318782	0.749892	NA
ADH4	0.317844	0.024237	0.033666	0.719909	0.471581	NA
FAM76B	90.86127	-0.02424	0.056072	-0.43222	0.66558	NA
GHRH	0.148587	0.024235	0.021411	1.131894	0.257679	NA
CD36	1.001215	-0.02423	0.051284	-0.47242	0.636627	NA
NPC1	102.6188	-0.02423	0.055257	-0.43844	0.66107	NA
MRPS35	102.7287	-0.02421	0.059151	-0.40934	0.682288	NA
RPS4X	503.9317	-0.02421	0.080499	-0.30069	0.763653	0.910066
VSTM2A	0.971555	-0.0242	0.051575	-0.46923	0.638904	NA
NFXL1	192.7982	0.024197	0.042346	0.571414	0.567719	NA
SLC9C1	0.177815	-0.0242	0.023409	-1.03363	0.301311	NA
LOC20165	0.177917	0.024193	0.023556	1.027071	0.304387	NA
FFAR2	0.354061	0.024191	0.030905	0.782739	0.43378	NA
PLA2G4E	0.229143	0.024187	0.027637	0.875181	0.381476	NA
SMC5	643.1169	-0.02418	0.050163	-0.48202	0.629794	0.844573
GTF2B	76.26344	-0.02418	0.063332	-0.38176	0.702639	NA
DPP3	41.33654	0.024176	0.084668	0.285544	0.775228	NA
ZNF677	39.47067	0.024175	0.07058	0.342514	0.731964	NA
SNORA46	4.399524	0.024173	0.079192	0.305241	0.760183	NA
RFX1	96.72157	0.02417	0.060961	0.396475	0.691755	NA
TMTC3	186.3051	0.024161	0.0463	0.521839	0.601783	NA
MLF2	177.6984	0.024161	0.061915	0.390226	0.696369	NA
LONRF3	151.8362	0.024159	0.049886	0.484289	0.628181	NA
TENM4	47.06784	-0.02415	0.083436	-0.28942	0.772258	NA
MIR345	0.50085	-0.02415	0.040711	-0.59314	0.553086	NA
C2orf81	13.11502	0.024138	0.08237	0.293047	0.769486	NA
TSPAN15	189.0312	-0.02414	0.07282	-0.33146	0.740294	NA
ZXDA	97.75538	0.024137	0.056355	0.428298	0.668434	NA
SLC45A1	43.64691	0.024129	0.072537	0.332642	0.739405	NA
PIP	0.93449	-0.02413	0.053577	-0.45034	0.652462	NA
ZXDC	679.1407	0.024128	0.044729	0.539414	0.589601	0.826169
EMP3	7.372538	-0.02412	0.08341	-0.2892	0.772426	NA
SIAE	58.43687	-0.02412	0.063261	-0.38129	0.70299	NA
CES1	1.450189	-0.02412	0.059752	-0.40366	0.686464	NA
RNPS1	328.5318	-0.02412	0.04928	-0.48943	0.624537	NA
ZNF582	27.31294	-0.02412	0.074089	-0.32551	0.744792	NA
ZSCAN16	38.88266	-0.02412	0.07097	-0.3398	0.734008	NA
SCGB3A2	0.923288	-0.02411	0.049369	-0.48843	0.625242	NA
ATG4B	185.9538	-0.02411	0.05846	-0.41243	0.680024	NA
ZNF254	346.7797	0.024109	0.043552	0.553572	0.579872	0.822777
PTHLH	1.748717	0.024109	0.059749	0.403494	0.686585	NA

OPTN	607.5443	-0.0241	0.046353	-0.51987	0.603156	0.830918
BET1	44.55886	0.024095	0.067179	0.358669	0.719843	NA
SLC28A3	0.308078	-0.02409	0.032679	-0.7373	0.460939	NA
ECD	191.2743	0.024094	0.043533	0.553472	0.57994	NA
SLC25A30	63.82952	-0.02409	0.062545	-0.38523	0.70007	NA
ZNF847P	0.157557	0.024094	0.02039	1.181656	0.237342	NA
DRD2	3.589791	-0.02409	0.076009	-0.31688	0.751337	NA
GBP3	13.4729	-0.02408	0.084334	-0.28551	0.775257	NA
URI1	427.2989	0.024076	0.052011	0.462893	0.643441	0.848802
PRRG3	0.813518	0.024065	0.050291	0.478509	0.632288	NA
PRPF4	80.64474	0.024064	0.053931	0.446193	0.655458	NA
GBA2	312.9752	0.02406	0.048468	0.49642	0.619598	NA
REV1	584.3626	0.024058	0.046678	0.51541	0.606267	0.831231
C14orf28	22.88265	0.024057	0.081241	0.296114	0.767143	NA
CLCN7	181.6185	-0.02406	0.064956	-0.37035	0.711123	NA
ITGB8	268.8941	-0.02405	0.081735	-0.29429	0.768533	NA
GPSM3	10.35915	-0.02404	0.08481	-0.28349	0.776802	NA
CORIN	12.28187	-0.02404	0.084646	-0.284	0.77641	NA
SLC39A7	97.93953	0.024032	0.053718	0.44737	0.654608	NA
ARL5C	0.190712	0.024016	0.023679	1.014247	0.310465	NA
SZRD1	95.89031	0.024015	0.04936	0.486531	0.62659	NA
ZNF653	69.69015	0.024013	0.065656	0.365741	0.714558	NA
MIR425	0.136752	0.024007	0.021223	1.131172	0.257983	NA
MAGED1	186.1675	0.024006	0.069094	0.347439	0.728262	NA
CHERP	217.7098	0.024006	0.045224	0.530818	0.595545	NA
STAG3L2	75.41817	-0.024	0.06862	-0.34981	0.726483	NA
DPF1	43.56541	-0.02399	0.072031	-0.33299	0.739142	NA
NRN1	217.6561	0.023969	0.074805	0.320419	0.748651	NA
ALS2	1820.496	-0.02396	0.041291	-0.58033	0.561694	0.810859
LOC10012	1.066964	-0.02396	0.055592	-0.43101	0.666463	NA
RAMP2	5.510722	0.02396	0.080018	0.299438	0.764606	NA
PARP10	32.57663	0.023954	0.075562	0.317013	0.751234	NA
C2orf27A	3.428809	-0.02394	0.075465	-0.31718	0.751104	NA
ALG2	66.21711	0.023932	0.059428	0.402707	0.687164	NA
TFG	116.0975	0.023925	0.047758	0.500968	0.616394	NA
PLEKHG5	1804.034	-0.02392	0.060149	-0.39774	0.690824	0.870767
SNAPIN	24.1654	-0.02392	0.079612	-0.30044	0.763845	NA
LOC10033	71.41101	-0.02391	0.071675	-0.33363	0.738658	NA
RPP25	10.43822	0.023912	0.084931	0.281543	0.778294	NA
PTPN23	314.5093	0.023909	0.039254	0.609087	0.542467	NA
SNORD114	0.98574	-0.02391	0.050916	-0.46957	0.638663	NA
XKR6	118.6454	0.023906	0.058634	0.40771	0.683486	NA
PRB3	0.626083	-0.02391	0.042679	-0.56013	0.575394	NA
HSPB11	40.76202	-0.0239	0.070709	-0.33803	0.735341	NA
MIR5006	0.459107	0.023896	0.038897	0.61435	0.538984	NA
GPR171	27.21746	-0.0239	0.080715	-0.29605	0.767188	NA
MIR761	0.145077	0.023896	0.016954	1.409497	0.158688	NA

LINC00568	5.231241	0.023896	0.083475	0.286269	0.774672	NA
USP17L8	0.877719	0.023895	0.048164	0.496124	0.619807	NA
TUBB1	18.70972	0.023892	0.084522	0.282672	0.777428	NA
MAPK8	106.0822	0.023888	0.046337	0.515527	0.606185	NA
SLC25A39	52.76961	0.023882	0.075607	0.315865	0.752105	NA
MLEC	192.6316	0.023877	0.060316	0.395859	0.692209	NA
NAPSA	12.49371	0.023874	0.084709	0.28183	0.778074	NA
CYB5D2	53.66754	0.023871	0.063233	0.377505	0.705798	NA
GPN2	48.23515	0.023868	0.063762	0.374333	0.708157	NA
SCYL1	168.4612	0.023868	0.043281	0.551455	0.581322	NA
LOC10050	30.89994	-0.02386	0.077367	-0.30839	0.757786	NA
ALDH7A1	134.9149	-0.02385	0.067262	-0.35461	0.722879	NA
HULC	3.066056	-0.02385	0.07087	-0.33654	0.736465	NA
BVES-AS1	0.683049	0.023843	0.044956	0.530363	0.59586	NA
ZNF676	7.602068	-0.02384	0.079663	-0.2993	0.764714	NA
TAF7L	0.354692	-0.02384	0.035237	-0.67651	0.498717	NA
RNASEH1	79.61746	0.023833	0.078581	0.303299	0.761662	NA
USP45	560.2206	0.023833	0.050844	0.468742	0.639254	0.847723
PSAP	1290.897	0.02383	0.066975	0.355808	0.721985	0.890244
OR52B2	0.288702	-0.02383	0.031846	-0.74817	0.454358	NA
FGF20	9.679621	0.023826	0.084413	0.282258	0.777746	NA
TAF2	206.5733	0.023822	0.042638	0.558703	0.576365	NA
AP5S1	16.46271	-0.02382	0.079459	-0.29978	0.764342	NA
MIR4723	0.338088	0.023815	0.032975	0.722238	0.470148	NA
MAP1B	24650.62	-0.02381	0.059808	-0.39812	0.690545	0.870767
DNMBP	145.4222	0.023807	0.056534	0.421112	0.673674	NA
C9orf9	13.43208	0.023804	0.082387	0.288927	0.772637	NA
PNN	1090.675	-0.0238	0.04064	-0.58572	0.558062	0.808857
KLC3	4.343559	0.023801	0.081521	0.291966	0.770313	NA
AFMID	72.87857	-0.0238	0.067262	-0.35385	0.723449	NA
TMEM161	19.74361	-0.0238	0.079021	-0.30119	0.763273	NA
LINC00477	0.149755	0.023797	0.020309	1.17179	0.241282	NA
KHDC1	14.78589	0.023797	0.08213	0.289754	0.772005	NA
CKMT1B	67.67641	0.023794	0.084263	0.282375	0.777656	NA
MYO18A	1524.084	-0.02379	0.043469	-0.54736	0.584133	0.824332
TEX11	0.160774	0.02379	0.020294	1.172266	0.24109	NA
MFSD3	21.50102	0.023778	0.076394	0.311253	0.755608	NA
SMPD1	64.70214	-0.02377	0.058211	-0.40842	0.682969	NA
SNORA20	34.98101	-0.02376	0.084695	-0.28048	0.779108	NA
SLC8A1-AS	10.07602	0.023752	0.084801	0.280085	0.779412	NA
ANTXR2	15.92022	-0.02375	0.08343	-0.28467	0.775893	NA
FGFBP2	0.162465	-0.02374	0.021842	-1.08683	0.27711	NA
TSNAX	82.78418	-0.02374	0.077517	-0.30619	0.759457	NA
HIBCH	113.4472	0.023728	0.07649	0.310208	0.756403	NA
MICAL2	2061.618	0.023725	0.05041	0.470637	0.6379	0.846919
DGKK	1.022816	0.02371	0.045468	0.521467	0.602042	NA
STON2	60.04637	0.0237	0.079786	0.29704	0.766436	NA

PAIP1	72.20424	-0.0237	0.065416	-0.36229	0.717137	NA
TGDS	19.62582	-0.0237	0.080331	-0.295	0.767994	NA
LRRC10B	1.266512	-0.02369	0.055475	-0.42705	0.669341	NA
SAP18	245.0258	0.023689	0.058402	0.40563	0.685014	NA
C17orf105	0.564762	0.023687	0.044118	0.536914	0.591327	NA
TTC26	21.15357	-0.02369	0.076295	-0.31045	0.75622	NA
LOC10049	0.682908	0.023682	0.045836	0.516671	0.605386	NA
CCDC83	0.443743	0.023673	0.035663	0.663799	0.506819	NA
FUT8-AS1	2.275225	0.023667	0.067294	0.35169	0.72507	NA
MIR142	0.913535	-0.02366	0.052817	-0.44805	0.654116	NA
SLC25A42	71.63552	-0.02366	0.063286	-0.37392	0.708462	NA
UBE2D4	103.2297	0.023649	0.058876	0.401679	0.687921	NA
BZRAP1-A	37.20706	0.023644	0.072661	0.325395	0.744882	NA
TPSG1	0.157965	-0.02364	0.021787	-1.08496	0.277939	NA
LINC00282	18.10439	-0.02363	0.083709	-0.28228	0.777725	NA
ARF3	317.067	0.023627	0.05775	0.409128	0.682445	NA
NR4A2	99.45362	0.023625	0.084888	0.278315	0.780771	NA
EMILIN1	15.56696	-0.02362	0.083324	-0.28352	0.77678	NA
NBR1	544.7958	0.023619	0.045087	0.523853	0.600381	0.830197
YME1L1	358.6271	0.023612	0.036484	0.64719	0.517509	0.785288
ZEB1	570.8993	-0.02361	0.036845	-0.64079	0.521659	0.787214
OR1F1	4.372198	-0.0236	0.06831	-0.34546	0.729748	NA
FOXA3	0.1501	-0.0236	0.018816	-1.25403	0.20983	NA
SAFB	664.4595	0.023595	0.033141	0.711955	0.476493	0.759174
NME9	160.3457	-0.02359	0.061238	-0.38528	0.700034	NA
RIC8A	100.0898	0.023591	0.046329	0.509214	0.610602	NA
SNORD114	18.83056	0.023587	0.084419	0.279402	0.779936	NA
SNORD95	12.34386	-0.02358	0.082114	-0.28713	0.774014	NA
INADL	1409.222	-0.02358	0.037798	-0.62375	0.532793	0.794978
VPS9D1	59.48984	0.023572	0.065349	0.360717	0.718311	NA
SUPT7L	242.4044	-0.02356	0.037258	-0.63242	0.52711	NA
EHBP1L1	116.8861	0.023561	0.080362	0.293192	0.769376	NA
LOC10028	0.134593	0.02356	0.02123	1.109727	0.267116	NA
TET3	448.3367	0.023558	0.055704	0.422918	0.672355	0.862824
IFT43	17.94693	-0.02356	0.079524	-0.29621	0.767069	NA
LOC10012	20.35012	0.023544	0.084411	0.278918	0.780307	NA
NAV2-AS5	9.217644	-0.02354	0.084633	-0.27811	0.780925	NA
MIR99B	0.764425	-0.02353	0.04884	-0.48181	0.629942	NA
LOC10012	65.55723	-0.02353	0.063516	-0.37046	0.711043	NA
LOC10063	12.68686	0.023529	0.082871	0.28392	0.776472	NA
ZFP57	1.997315	-0.02351	0.062118	-0.37848	0.705072	NA
ATP6V0E1	30.83201	-0.0235	0.075843	-0.30992	0.756625	NA
SBSN	0.385124	-0.0235	0.033233	-0.70717	0.47946	NA
AGO1	419.1917	-0.0235	0.037881	-0.62037	0.535013	0.796193
OSTN	0.240007	0.023496	0.027709	0.847936	0.396474	NA
OTUD3	233.7116	-0.02349	0.047889	-0.49059	0.623718	NA
ADAT2	34.49374	0.02349	0.072468	0.324148	0.745826	NA

RNF19A	421.8823	0.023486	0.035596	0.659788	0.50939	0.781767
SLC2A1-AS	6.385409	-0.02349	0.081642	-0.28767	0.773603	NA
BPIFC	0.166514	-0.02348	0.023559	-0.99672	0.318903	NA
CCDC148-	0.419075	0.02348	0.038206	0.614582	0.538831	NA
XG	0.163857	0.023478	0.020371	1.152532	0.249102	NA
TBL3	70.05991	0.023467	0.063219	0.3712	0.710489	NA
TAS1R3	17.97335	0.023462	0.084622	0.277252	0.781587	NA
LCORL	121.9083	-0.02346	0.054962	-0.42684	0.669496	NA
TRIM3	198.4001	-0.02345	0.050015	-0.46892	0.63913	NA
NT5C3	56.45705	0.023448	0.065596	0.357461	0.720747	NA
LINC00115	19.74097	0.023445	0.082405	0.28451	0.77602	NA
TAF1C	144.9386	-0.02344	0.059494	-0.39397	0.693607	NA
NALCN	365.2507	0.023434	0.050948	0.459958	0.645546	0.849041
LRRC37A3	74.7955	0.02343	0.07963	0.29424	0.768575	NA
TOP1	596.9375	0.0234	0.043141	0.542416	0.587532	0.825363
IFI27	12.12183	-0.0234	0.084296	-0.27755	0.781361	NA
MIR5685	0.723339	0.023393	0.044971	0.520192	0.60293	NA
C2orf71	1.761329	-0.02339	0.061371	-0.38113	0.703104	NA
FBN2	2.181694	0.023383	0.063514	0.368159	0.712755	NA
POU3F4	7.181773	0.02337	0.083935	0.278434	0.780679	NA
LYPD3	0.202953	-0.02336	0.027193	-0.85901	0.390336	NA
MTNR1B	1.131931	-0.02335	0.051602	-0.45258	0.650852	NA
MIR409	0.30748	-0.02335	0.031856	-0.73303	0.46354	NA
MDGA2	37.66001	-0.02335	0.081378	-0.2869	0.774191	NA
STMN3	453.1375	0.023346	0.05595	0.417262	0.676487	0.863722
SH3BGR	19.15099	-0.02335	0.081029	-0.28811	0.773262	NA
BCHE	8.776253	0.02334	0.083566	0.279302	0.780013	NA
ASB13	53.14076	0.023338	0.081121	0.287697	0.773579	NA
P4HA3	5.097471	-0.02333	0.064891	-0.35957	0.719169	NA
DGAT2L6	1.416541	0.023332	0.05959	0.391542	0.695397	NA
ANXA9	15.39397	-0.02333	0.083422	-0.27966	0.779742	NA
IGFBP2	2.760984	-0.02332	0.075004	-0.31096	0.755831	NA
ZNF764	53.8068	-0.02332	0.060039	-0.38846	0.697676	NA
FAM133CP	0.477468	-0.0233	0.040876	-0.57014	0.568585	NA
FLNA	467.4648	0.0233	0.067369	0.345856	0.729451	0.892833
TNNC2	4.336818	-0.0233	0.078531	-0.29667	0.76672	NA
ORMDL3	99.40937	0.023288	0.053518	0.435143	0.663458	NA
GNA13	328.7137	0.023288	0.043913	0.530319	0.595891	NA
C18orf25	220.3314	0.023285	0.037587	0.619509	0.535581	NA
LOC11323	30.27555	-0.02328	0.07409	-0.31428	0.75331	NA
CYP2R1	32.1393	-0.02328	0.072308	-0.32192	0.747511	NA
BSG	466.3348	-0.02327	0.064451	-0.36104	0.718073	0.887792
BNIP2	109.7834	-0.02326	0.049349	-0.47125	0.637461	NA
LOC10050	0.196971	-0.02326	0.023796	-0.97729	0.328425	NA
SNORA36A	0.299674	-0.02325	0.031861	-0.72978	0.465522	NA
SLIT2	6.25869	0.023247	0.079354	0.292952	0.769559	NA
SNORA81	1.229013	-0.02325	0.059636	-0.3898	0.696688	NA

SLC35G1	3.01016	0.023243	0.075656	0.307223	0.758674	NA
GSC2	0.132202	0.023237	0.021213	1.095417	0.273334	NA
CECR1	54.7209	0.023236	0.084856	0.273834	0.784212	NA
VN1R1	78.53281	0.023235	0.059212	0.392404	0.69476	NA
SNTN	0.987604	0.023233	0.053964	0.430527	0.666812	NA
CMTM4	272.1883	0.02323	0.043845	0.529813	0.596241	NA
TBC1D16	541.6548	-0.02322	0.056707	-0.40946	0.682199	0.867105
SPCS2	63.48223	0.023218	0.057245	0.4056	0.685037	NA
ACYP1	35.91651	0.023216	0.071959	0.322629	0.746976	NA
LINC00612	12.51337	0.023196	0.084418	0.274781	0.783485	NA
MIR4673	0.150804	-0.0232	0.021689	-1.06949	0.284851	NA
CCR9	0.654005	0.023194	0.04375	0.530144	0.596012	NA
GOLGA7	111.2038	-0.02319	0.059196	-0.3918	0.695203	NA
CCDC70	2.410761	0.02318	0.071178	0.325666	0.744677	NA
ATP2B4	473.7696	-0.02318	0.066656	-0.3477	0.728063	0.89271
SNAI3	20.39739	0.023172	0.079881	0.290087	0.77175	NA
LOC28369	2.458807	-0.02317	0.070618	-0.32811	0.742829	NA
RAD21	906.4996	-0.02317	0.049826	-0.46501	0.641922	0.848776
AGAP3	412.2214	-0.02316	0.042952	-0.53911	0.589808	0.826169
SSR1	529.0528	0.023154	0.034171	0.677588	0.498033	0.776355
ITGA5	21.73701	-0.02315	0.084868	-0.27281	0.784999	NA
C6orf10	4.697293	0.023151	0.075574	0.306331	0.759353	NA
RGS8	20.51045	-0.02314	0.07518	-0.30783	0.758215	NA
KPNA6	397.8317	0.023141	0.034118	0.678252	0.497612	0.776233
RSL1D1	306.1448	0.023138	0.051176	0.452129	0.651176	NA
NDUFV1	211.9441	0.023136	0.053818	0.429903	0.667266	NA
TACC3	33.16818	-0.02313	0.076862	-0.30098	0.763427	NA
TMEM138	30.54374	-0.02313	0.071222	-0.3248	0.745329	NA
PSPC1	462.4178	-0.02313	0.045472	-0.5087	0.610961	0.834133
SGTA	178.8699	-0.02313	0.044389	-0.52111	0.602292	NA
FLJ30403	23.80488	-0.02313	0.082386	-0.2807	0.778938	NA
PDE11A	3.4303	-0.02312	0.070921	-0.32603	0.744399	NA
TMEM233	0.142188	-0.02312	0.021557	-1.0725	0.283496	NA
BTBD9	185.6775	0.023119	0.049109	0.470762	0.637811	NA
RAD9A	29.86785	-0.02311	0.078194	-0.2956	0.767538	NA
UGDH-AS1	21.63875	-0.02311	0.076012	-0.30406	0.761082	NA
GPR84	1.355771	-0.02311	0.05831	-0.39632	0.691869	NA
ERGIC1	232.5945	0.023109	0.049039	0.471232	0.637475	NA
IRAK4	16.08038	-0.0231	0.081346	-0.28399	0.776416	NA
CLDN19	1.052242	-0.0231	0.051631	-0.44733	0.654638	NA
SMIM7	44.07927	-0.02309	0.064026	-0.36068	0.718337	NA
FAM192A	217.3818	-0.02309	0.04937	-0.46773	0.639978	NA
MIR380	4.283349	-0.02309	0.0794	-0.29078	0.771222	NA
LOC34007	0.17026	-0.02308	0.022077	-1.04563	0.295731	NA
LRRC45	150.6799	-0.02307	0.076898	-0.30006	0.764129	NA
ALKBH8	62.53913	0.023073	0.069187	0.333494	0.738761	NA
NIN	1015.445	0.023071	0.052022	0.443493	0.657409	0.854783

NPHP3-AC	22.30008	-0.02307	0.077718	-0.2968	0.766616	NA
ZNF813	7.98198	0.023061	0.084617	0.272537	0.785209	NA
MIR548N	0.977016	-0.02306	0.054777	-0.42096	0.673781	NA
LOC90784	28.7353	0.023056	0.081177	0.284017	0.776398	NA
ADAMTS1	9.668085	0.023054	0.084937	0.27142	0.786068	NA
HIST1H2B	2.978605	0.02305	0.07443	0.309692	0.756795	NA
DMKN	5.6563	-0.02305	0.08133	-0.28336	0.776901	NA
LBH	4.888834	0.023044	0.079334	0.290465	0.771461	NA
ECHDC2	100.596	0.023035	0.073455	0.313586	0.753836	NA
RTCA	160.2621	-0.02303	0.065581	-0.35121	0.725433	NA
DERL3	22.52225	0.023031	0.084247	0.273372	0.784567	NA
GTF2IRD2	5.783572	-0.02303	0.083224	-0.27667	0.782033	NA
CYP2D6	42.93313	0.023026	0.083502	0.275751	0.782739	NA
KIAA1432	300.256	0.023023	0.039944	0.576395	0.564349	NA
PRUNE	67.21158	0.023019	0.057147	0.4028	0.687095	NA
VGLL3	7.433057	0.023013	0.081643	0.281871	0.778042	NA
TMEM130	160.5349	0.023012	0.081103	0.283735	0.776613	NA
KRTCAP2	33.77916	-0.02301	0.072968	-0.31531	0.75253	NA
S100P	2.822311	0.022993	0.072453	0.317347	0.750981	NA
STMN1	708.9327	0.022992	0.058419	0.393564	0.693903	0.871418
CCDC111	132.0882	0.022991	0.061898	0.371441	0.710309	NA
TGFBR2	36.27782	0.022986	0.084406	0.272331	0.785367	NA
OTOP2	0.196354	-0.02298	0.022006	-1.04449	0.296257	NA
SLC6A16	3.156397	-0.02298	0.077879	-0.2951	0.767914	NA
MIR548B	0.218185	0.02298	0.026558	0.865272	0.38689	NA
MGC1627	23.78603	-0.02298	0.083087	-0.27656	0.782116	NA
C1orf189	0.75522	0.022979	0.046883	0.490133	0.62404	NA
SYNGAP1	345.3831	0.022969	0.050151	0.457998	0.646954	0.849041
GPX8	1.140056	-0.02297	0.049905	-0.46024	0.645347	NA
PUS7L	212.0453	0.022967	0.059976	0.382931	0.701771	NA
LCN8	14.09463	-0.02296	0.074539	-0.30801	0.758074	NA
KCTD19	1.880583	-0.02295	0.067374	-0.34068	0.733345	NA
KIAA1377	351.2279	0.02295	0.06169	0.37203	0.70987	0.884085
GMCL1P1	0.444149	0.022948	0.034324	0.668582	0.503762	NA
STXBP6	5.783318	-0.02294	0.082205	-0.27911	0.780157	NA
VMA21	143.2299	0.022934	0.067744	0.338537	0.734958	NA
BFAR	76.18121	-0.02293	0.062474	-0.36708	0.713558	NA
SMURF1	227.6515	0.022902	0.052303	0.437872	0.661479	NA
ZNF446	58.06424	-0.0229	0.06492	-0.35276	0.724268	NA
ARMC4	2.559946	-0.02289	0.071978	-0.31801	0.750476	NA
CYP4A22	0.651034	-0.02289	0.04539	-0.50428	0.614064	NA
PRPF18	276.9706	0.022887	0.039302	0.582334	0.560342	NA
OR5B2	0.119202	-0.02289	0.019462	-1.17593	0.239624	NA
TMEM241	6.872061	0.022884	0.083275	0.274801	0.78347	NA
ANKLE2	287.8959	0.02288	0.040025	0.571638	0.567567	NA
PPP6R1	251.724	0.022879	0.058109	0.393725	0.693784	NA
MIR637	0.761164	0.022877	0.047853	0.478063	0.632606	NA

VPS36	282.3979	-0.02287	0.043741	-0.52294	0.601019	NA
FBXL22	8.854101	-0.02287	0.084529	-0.27056	0.786727	NA
MRAS	259.8278	-0.02286	0.056756	-0.40277	0.687117	NA
COASY	80.33553	-0.02286	0.053838	-0.42459	0.671133	NA
LOC15274	0.632211	-0.02285	0.043399	-0.52659	0.59848	NA
SNORD119	29.54833	-0.02285	0.084826	-0.26936	0.787655	NA
PSG7	10.93693	-0.02284	0.082774	-0.27599	0.782557	NA
FAM108B1	87.84799	0.022841	0.049791	0.458728	0.646429	NA
TBL1X	79.93061	0.022838	0.075108	0.304068	0.761076	NA
SNORA5A	3.305733	0.022837	0.075138	0.303931	0.76118	NA
ARSF	0.536598	-0.02284	0.042669	-0.5352	0.592514	NA
TCHP	246.6064	-0.02284	0.053504	-0.42681	0.66952	NA
GEMIN6	19.65993	0.022832	0.077453	0.294786	0.768157	NA
ANKRD26P	5.82057	-0.02283	0.075562	-0.30211	0.762568	NA
LOC33929	0.145989	-0.02283	0.021682	-1.05286	0.292404	NA
ST6GAL2	28.26546	0.022828	0.083736	0.272614	0.78515	NA
CDH16	0.516556	0.022827	0.040362	0.565554	0.571697	NA
LMLN	78.13877	-0.02282	0.072279	-0.31579	0.752161	NA
SLC22A14	14.25448	0.022817	0.084504	0.270012	0.787151	NA
MITF	16.28725	-0.02282	0.081204	-0.28098	0.778726	NA
LOC14688	415.1277	-0.0228	0.061935	-0.36817	0.712745	0.884585
PCED1B	1.162221	-0.0228	0.053536	-0.42594	0.670155	NA
PTP4A1	159.3218	0.022797	0.076835	0.296701	0.766694	NA
KRT39	0.637297	0.022792	0.036008	0.632955	0.526763	NA
LOC72996	0.145792	-0.02279	0.021751	-1.04786	0.294705	NA
ZFX	421.5558	0.022781	0.064574	0.352794	0.724243	0.890559
LOC10013	0.4508	-0.02278	0.038443	-0.59257	0.553466	NA
MTVR2	0.667384	-0.02278	0.044574	-0.51101	0.609342	NA
MIR2861	0.428633	0.022772	0.037592	0.605767	0.54467	NA
LOC64242	0.636768	0.022766	0.040845	0.557379	0.577269	NA
PLEKHA5	399.9869	-0.02276	0.045046	-0.50534	0.613321	0.83685
ASB18	1.248529	-0.02275	0.05613	-0.40539	0.68519	NA
ATP6V1D	135.9846	-0.02274	0.06136	-0.37064	0.710902	NA
LOC10050	12.47113	-0.02273	0.084937	-0.26767	0.788955	NA
LOC10050	1.139738	-0.02273	0.05479	-0.41491	0.678208	NA
IFITM10	92.8498	-0.02273	0.072857	-0.31193	0.75509	NA
PRR5L	18.7724	0.022726	0.082889	0.274179	0.783947	NA
PARD6A	63.43622	-0.02273	0.073788	-0.30799	0.758091	NA
IFT46	112.7701	0.022726	0.050243	0.452321	0.651038	NA
FUT10	112.5467	-0.02272	0.064427	-0.35262	0.724373	NA
AHDC1	411.4085	0.022718	0.051517	0.440978	0.659229	0.855675
CNNM4	156.3144	0.022715	0.047466	0.47856	0.632251	NA
SNRPD2P2	1.132538	0.02271	0.052587	0.43186	0.665843	NA
LILRB3	3.802015	-0.02271	0.070678	-0.32131	0.747974	NA
POU4F1-A	3.089801	0.022708	0.071601	0.317149	0.75113	NA
ZNF354A	33.8272	-0.0227	0.075276	-0.30159	0.762963	NA
MIR3935	4.863777	-0.0227	0.081779	-0.27754	0.781365	NA

THEGL	0.359625	0.022697	0.031007	0.731977	0.464183	NA
SCAPER	711.2977	0.022696	0.046752	0.485449	0.627358	0.843303
MIR103A2	0.286436	0.022687	0.028094	0.807557	0.419346	NA
CRP	0.135706	-0.02268	0.018734	-1.21079	0.225976	NA
PLEKHB2	253.3633	0.022681	0.065527	0.34613	0.729245	NA
FAM189B	51.93138	-0.02268	0.069933	-0.32425	0.745746	NA
SMURF2	156.4655	0.022672	0.047179	0.480551	0.630835	NA
HAL	1.374132	-0.02267	0.060194	-0.3766	0.706472	NA
RPUSD1	23.63296	-0.02267	0.074392	-0.30471	0.760584	NA
HTT-AS1	2.909688	-0.02266	0.073367	-0.30887	0.757417	NA
SMAD2	308.2292	-0.02266	0.049537	-0.45741	0.647375	NA
WBSCR17	458.1245	-0.02266	0.05511	-0.41111	0.680995	0.866937
WSCD2	793.2231	-0.02265	0.04942	-0.45841	0.646657	0.849041
FAM220A	43.20119	-0.02265	0.07384	-0.30679	0.759005	NA
PCSK7	116.6616	0.022648	0.050373	0.449614	0.652989	NA
SYMPK	245.9797	-0.02264	0.042046	-0.53849	0.590242	NA
TRMT10A	17.69058	-0.02264	0.079795	-0.28367	0.776661	NA
FZD7	363.2296	0.022633	0.061037	0.370808	0.710781	0.884085
FZD6	78.12592	-0.02263	0.078398	-0.28869	0.772817	NA
TNFSF8	0.798347	0.022627	0.048042	0.47099	0.637648	NA
LOC10021	287.2506	-0.02262	0.068143	-0.33198	0.739907	NA
ZNF593	18.19459	0.022603	0.081172	0.278457	0.780661	NA
MIR92B	0.485138	-0.02259	0.036957	-0.61139	0.540942	NA
NAGLU	17.23959	0.022591	0.081372	0.277627	0.781299	NA
OTUD4	345.1611	0.02259	0.051721	0.436777	0.662273	0.855957
GPR97	0.411987	0.022588	0.037634	0.600187	0.548382	NA
SCIMP	13.08232	0.022585	0.084151	0.268381	0.788406	NA
RASGRP3	22.4278	0.022583	0.084874	0.266077	0.79018	NA
GNAL	53.31132	-0.02257	0.072959	-0.30935	0.757056	NA
RBBP9	59.389	0.022569	0.062705	0.359921	0.718906	NA
SMARCC2	1290.241	-0.02257	0.029008	-0.77798	0.436582	0.738506
U2AF2	493.9621	0.022564	0.067526	0.33415	0.738266	0.895927
FLJ39739	0.253343	0.022561	0.025287	0.892175	0.372299	NA
MIR15A	0.255864	-0.02256	0.030919	-0.72965	0.465607	NA
LOC10050	315.9383	-0.02254	0.053591	-0.42064	0.674017	NA
MCM6	95.0815	0.022532	0.0677	0.332817	0.739272	NA
ATL3	70.141	0.02252	0.057569	0.391187	0.695659	NA
AURKAIP1	74.07521	-0.02252	0.063376	-0.35531	0.722359	NA
RUFY4	0.443823	-0.02252	0.033725	-0.66764	0.504363	NA
FOXD4L5	0.788935	0.022513	0.043538	0.517085	0.605097	NA
LOC33950	1.544805	0.022502	0.063403	0.354911	0.722656	NA
LOC10012	0.782541	-0.02249	0.044538	-0.50493	0.613611	NA
KCNK15	3.934087	0.02248	0.074983	0.299803	0.764327	NA
NIPA1	208.7399	0.022479	0.062064	0.362185	0.717213	NA
KIR3DX1	0.247842	-0.02248	0.026843	-0.83738	0.40238	NA
HADH	54.08347	-0.02247	0.059036	-0.38065	0.703462	NA
DLGAP1	1117.942	-0.02247	0.062801	-0.35777	0.720514	0.889839

NTHL1	19.28591	-0.02246	0.079867	-0.28122	0.77854	NA
ZNF445	564.7063	0.02246	0.039219	0.572686	0.566857	0.814844
HSD3BP4	37.38047	-0.02246	0.083359	-0.26942	0.787607	NA
FLJ13197	26.04161	0.022449	0.082087	0.273477	0.784487	NA
CHST4	0.516072	-0.02245	0.042269	-0.53108	0.595362	NA
ANO1	13.23781	-0.02245	0.08366	-0.26829	0.788477	NA
ISLR2	2.643774	0.022442	0.063619	0.35275	0.724276	NA
LINC00162	0.213289	0.022439	0.026535	0.845659	0.397743	NA
MEIS1	384.3664	-0.02243	0.044508	-0.50397	0.61428	0.837655
GRPR	1.639849	-0.02243	0.061844	-0.36267	0.716849	NA
MTHFD2L	34.29636	0.022414	0.07154	0.313305	0.754049	NA
ST6GALNA	1.263785	0.022413	0.045793	0.489431	0.624537	NA
C8orf48	3.940388	0.022412	0.078162	0.286736	0.774315	NA
DNM1L	545.8362	0.022409	0.048849	0.458747	0.646416	0.849041
C9orf41	133.5643	0.022398	0.054235	0.412981	0.67962	NA
FRMPD2	37.18165	-0.02239	0.083542	-0.26802	0.788681	NA
IL1B	1.030839	0.022391	0.042367	0.528495	0.597156	NA
ANKRD6	370.897	-0.02237	0.067936	-0.32933	0.741909	0.896704
FMNL1	150.0858	0.02237	0.053721	0.416406	0.677113	NA
LOC22072	222.0407	0.022365	0.048956	0.456825	0.647797	NA
PSMA4	68.38031	-0.02235	0.061836	-0.3615	0.717728	NA
MPZL3	12.58137	-0.02235	0.083259	-0.26845	0.788356	NA
TSPAN17	37.62785	0.022348	0.074385	0.300433	0.763847	NA
MIR548AE	0.586926	0.022345	0.043737	0.510907	0.609416	NA
GLYAT	0.208076	0.022343	0.022214	1.005793	0.314515	NA
LPCAT4	208.1127	-0.02234	0.055915	-0.39956	0.68948	NA
PRPS2	26.3105	0.022341	0.080754	0.276658	0.782043	NA
FLJ41941	0.110031	-0.02234	0.01928	-1.15873	0.246566	NA
FAM3D	0.205304	-0.02234	0.026262	-0.85062	0.394982	NA
FILIP1L	71.5775	0.022337	0.065874	0.339092	0.734541	NA
FAM189A1	52.42881	-0.02234	0.070658	-0.3161	0.751924	NA
BCL10	25.59887	-0.02233	0.071615	-0.31186	0.755149	NA
DRAM1	7.606191	-0.02233	0.082772	-0.26976	0.787341	NA
ZNF436	97.10811	-0.02233	0.077913	-0.28656	0.774448	NA
MIR548AU	0.23235	0.022323	0.027688	0.806237	0.420106	NA
THOC7	121.7109	-0.02232	0.061673	-0.36191	0.717417	NA
OGN	30.78401	-0.02232	0.081756	-0.273	0.78485	NA
PCOLCE-A	4.480507	-0.02232	0.081668	-0.27324	0.784667	NA
PHTF1	380.7555	-0.02231	0.043048	-0.51837	0.604204	0.830918
RNF43	149.8379	-0.02231	0.066115	-0.33746	0.73577	NA
CLSTN2	47.92813	-0.02231	0.082487	-0.27045	0.786813	NA
LRRC72	0.151701	-0.02231	0.021813	-1.0227	0.306449	NA
ADRA2C	2.769815	-0.0223	0.066465	-0.33552	0.73723	NA
ZFC3H1	836.2051	-0.0223	0.049612	-0.44945	0.653111	0.853604
DGUOK	33.76048	-0.0223	0.073318	-0.30413	0.761032	NA
HMGA1P7	0.482853	-0.0223	0.033442	-0.66668	0.504976	NA
LMAN2	100.8074	-0.02229	0.057444	-0.38809	0.697948	NA

LOC10050	59.41508	-0.02229	0.077518	-0.28754	0.773695	NA
DTYMK	29.29713	0.022289	0.07099	0.313977	0.753539	NA
XPNPEP1	90.853	-0.02228	0.050303	-0.443	0.657764	NA
FAM110D	1.499556	-0.02228	0.060695	-0.36713	0.713519	NA
SPA17	1.695395	-0.02228	0.063255	-0.35223	0.724667	NA
MIR2053	0.146707	-0.02226	0.021692	-1.02626	0.30477	NA
RBBP8	109.936	0.022245	0.069076	0.322035	0.747427	NA
HGD	1.489275	-0.02224	0.062523	-0.3557	0.722064	NA
KRT23	0.213911	-0.02223	0.026326	-0.84439	0.39845	NA
UBAP1L	80.19752	-0.02223	0.064251	-0.34592	0.729405	NA
MEIS3P1	16.09023	-0.02222	0.08383	-0.26508	0.79095	NA
ACOXL	1.128702	0.02222	0.053309	0.416803	0.676823	NA
CCDC125	51.18151	-0.02222	0.062122	-0.35762	0.720625	NA
SNORA53	4.063218	0.022211	0.078999	0.28115	0.778595	NA
CDK8	151.3298	0.022204	0.058067	0.382389	0.702173	NA
TGFB1I1	19.37618	-0.0222	0.084929	-0.26137	0.793807	NA
GPR151	1.269857	-0.02219	0.055987	-0.39635	0.691847	NA
LCK	0.167126	-0.02218	0.0187	-1.18624	0.235527	NA
ASB15	0.574529	-0.02218	0.042531	-0.52151	0.60201	NA
LOC10028	11.82066	-0.02218	0.084634	-0.26208	0.793264	NA
CTNNBIP1	124.6424	0.02218	0.055296	0.40112	0.688332	NA
SPATA31E	0.385766	0.02218	0.030389	0.729877	0.465465	NA
MIR941-1	5.223404	-0.02218	0.080801	-0.27448	0.783717	NA
SYT10	0.363134	-0.02218	0.035848	-0.61868	0.53613	NA
SBDS	226.597	-0.02217	0.050638	-0.43791	0.661452	NA
GLO1	104.3999	-0.02217	0.06835	-0.32443	0.745616	NA
DDX11L5	0.442179	0.022161	0.035086	0.631624	0.527633	NA
CRTC2	79.8999	0.02216	0.059376	0.37322	0.708985	NA
LRP4-AS1	2.33115	-0.02216	0.070949	-0.31233	0.75479	NA
MED22	63.01499	0.022154	0.058147	0.380998	0.703204	NA
IGLON5	238.7157	0.022148	0.068665	0.322557	0.747031	NA
GPATCH11	99.3355	0.022128	0.059719	0.370543	0.710978	NA
KANSL1L	235.0492	-0.02213	0.045296	-0.48849	0.625206	NA
LINC00167	4.765616	-0.02212	0.081727	-0.27066	0.786649	NA
RIMBP3	0.164176	-0.02211	0.024237	-0.91244	0.36154	NA
C10orf137	299.558	0.022111	0.041671	0.53061	0.595689	NA
FGF13-AS1	0.165222	0.022111	0.024249	0.911823	0.361862	NA
PLGLA	0.128937	-0.0221	0.019769	-1.11806	0.263542	NA
RNF122	37.96196	0.022098	0.08321	0.265565	0.790574	NA
LOC28307	122.6505	0.022091	0.064054	0.344877	0.730186	NA
USP28	288.7915	0.022084	0.045345	0.487014	0.626249	NA
MGC7208	24.3491	0.022076	0.079319	0.278314	0.780772	NA
RMDN1	105.7214	0.022075	0.049059	0.449967	0.652734	NA
LOC10013	43.06757	-0.02205	0.078562	-0.28073	0.778917	NA
LINC00461	252.5107	0.022054	0.051624	0.427201	0.669233	NA
SGSM2	252.5581	-0.02205	0.050244	-0.43882	0.660791	NA
MIR4305	0.66502	-0.02204	0.042986	-0.51284	0.608064	NA

TMEM194	55.58994	-0.02204	0.073625	-0.29938	0.764653	NA
ITGB6	0.145477	0.02204	0.02002	1.100877	0.27095	NA
FRY	1332.847	-0.02204	0.040239	-0.54769	0.583907	0.824332
GTF2H2C	0.478899	0.02203	0.039489	0.557868	0.576935	NA
MIR1247	0.272103	-0.02203	0.025116	-0.87707	0.380447	NA
JAM2	140.1369	-0.02203	0.068885	-0.31974	0.749162	NA
SPATA25	10.9804	-0.02203	0.084902	-0.25942	0.795309	NA
DLG4	869.686	-0.02202	0.059629	-0.36927	0.711924	0.884535
FRAT2	27.17678	-0.02201	0.077539	-0.2839	0.776489	NA
KBTBD11	336.8006	0.022011	0.07411	0.297012	0.766457	0.910822
UBXN4	511.2302	0.02201	0.038198	0.576197	0.564482	0.813328
EBF2	2.229798	-0.022	0.06526	-0.33714	0.736014	NA
MIR1276	0.489061	0.022001	0.037362	0.588848	0.555964	NA
C5orf58	0.154457	-0.022	0.021877	-1.00556	0.314627	NA
GSTTP2	0.115549	-0.02199	0.019536	-1.12556	0.260351	NA
S1PR4	0.472479	-0.02198	0.037961	-0.57907	0.562545	NA
USP19	212.2295	0.021974	0.043679	0.503081	0.614907	NA
ANKRD65	21.37006	-0.02197	0.083658	-0.26266	0.792815	NA
GPR15	0.559821	-0.02197	0.033602	-0.6539	0.513177	NA
GSDMC	0.189352	0.021971	0.02377	0.9243	0.35533	NA
SUMO4	12.88742	-0.02197	0.084793	-0.2591	0.795559	NA
TGM5	0.449754	0.021965	0.03593	0.611321	0.540987	NA
GTF2H1	175.0758	0.021962	0.060708	0.361763	0.717529	NA
ELMOD2	92.93673	0.021961	0.056373	0.389566	0.696858	NA
ZNF175	144.9708	0.021959	0.046445	0.472787	0.636365	NA
SOWAHD	0.902326	-0.02193	0.052294	-0.41933	0.674974	NA
SLC24A4	45.00528	0.021928	0.084702	0.258889	0.795721	NA
SEC24B-AS	3.178182	0.021926	0.077468	0.283033	0.777152	NA
PCDH8	2.333249	0.02191	0.056972	0.384572	0.700555	NA
CORO1C	230.3787	0.021905	0.041213	0.531512	0.595064	NA
FURIN	162.0533	-0.0219	0.058198	-0.37627	0.706713	NA
ELFN1	1.932595	0.021894	0.063163	0.346629	0.72887	NA
TBC1D3P1	0.399311	-0.02188	0.034728	-0.63011	0.528625	NA
RAB44	0.11426	-0.02187	0.019501	-1.1216	0.262034	NA
VPS13B	753.432	-0.02187	0.039198	-0.55794	0.576884	0.821271
SPRY3	0.121281	-0.02186	0.019612	-1.11447	0.265077	NA
C16orf55	34.10613	-0.02186	0.071088	-0.30744	0.758506	NA
DKFZp686	0.124258	0.021842	0.019656	1.1112	0.266482	NA
ALYREF	41.73863	0.021838	0.069003	0.31648	0.751638	NA
KIAA0100	673.6937	0.021834	0.041466	0.526547	0.598508	0.829672
TMBIM4	26.50818	0.021829	0.077622	0.281225	0.778538	NA
LZTS1-AS1	0.591698	-0.02181	0.04303	-0.50696	0.612186	NA
CD1D	0.345682	-0.02181	0.03524	-0.61899	0.535926	NA
FOXL2	0.998859	0.021812	0.052905	0.41229	0.680127	NA
TNNT2	0.163395	-0.02181	0.024237	-0.89982	0.368215	NA
FNDC1	1.412999	0.0218	0.05648	0.385984	0.699509	NA
SAR1B	198.9477	0.021789	0.053577	0.40668	0.684243	NA

LINC00622	4.569765	-0.02179	0.082478	-0.26415	0.791661	NA
MIR638	0.119154	-0.02179	0.019632	-1.10977	0.2671	NA
NLRC3	78.52496	0.021785	0.072391	0.30094	0.76346	NA
HOTAIRM1	0.389274	-0.02179	0.034028	-0.64021	0.522036	NA
SLC15A4	62.16434	-0.02178	0.060134	-0.36225	0.717169	NA
MYL9	25.62055	-0.02178	0.083298	-0.2615	0.79371	NA
LAYN	5.12124	-0.02178	0.08002	-0.27215	0.785503	NA
ZNF358	107.83	-0.02178	0.05768	-0.37754	0.705774	NA
SORBS2	220.614	0.021776	0.058112	0.37472	0.707869	NA
ADAM9	417.0569	0.021774	0.061276	0.355342	0.722334	0.890244
LDHC	0.463386	0.021773	0.034197	0.636694	0.524324	NA
MIR22HG	6.611783	-0.02177	0.082748	-0.26311	0.792467	NA
C10orf62	12.73865	-0.02177	0.084819	-0.25662	0.797474	NA
LOC38963	0.875443	-0.02176	0.052603	-0.41368	0.679107	NA
CHMP1A	62.54191	0.021754	0.061274	0.355031	0.722567	NA
MMD	20.63043	0.021745	0.084326	0.257863	0.796512	NA
C3orf18	109.1961	0.021739	0.07893	0.275421	0.782993	NA
ULBP1	27.56416	-0.02174	0.083953	-0.25893	0.795693	NA
ZGPAT	254.5313	-0.02173	0.074277	-0.29257	0.769848	NA
SCAMP4	68.26932	-0.02172	0.053415	-0.40666	0.684258	NA
MPST	10.14418	0.021721	0.08491	0.255812	0.798096	NA
MTHFR	144.0652	0.021718	0.055708	0.389853	0.696645	NA
HSF2	125.8609	0.021714	0.051942	0.418045	0.675914	NA
CDK18	142.48	-0.02171	0.076699	-0.28306	0.777128	NA
FOXN2	253.1987	0.021702	0.062716	0.346032	0.729319	NA
LDHAL6B	0.622787	-0.0217	0.043755	-0.49595	0.619927	NA
CDC14C	0.409637	0.021696	0.036984	0.586631	0.557451	NA
C21orf128	0.274328	0.021694	0.025056	0.865798	0.386601	NA
DAB2IP	1360.101	0.02168	0.048521	0.446821	0.655005	0.854402
SRF	109.1958	-0.02168	0.050288	-0.43106	0.666424	NA
MAP3K4	403.5049	-0.02167	0.0409	-0.52988	0.596192	0.829672
MAZ	263.0364	0.02167	0.052805	0.410384	0.681524	NA
TCAP	9.488881	0.021667	0.083611	0.259146	0.795523	NA
STX19	6.025102	0.02166	0.083648	0.258949	0.795675	NA
MIR4651	0.187877	0.021659	0.023767	0.911332	0.36212	NA
TMEM183	27.6324	0.021654	0.079223	0.273331	0.784598	NA
ATG10	25.05497	-0.02165	0.083837	-0.25827	0.7962	NA
MIR376A1	0.724102	-0.02164	0.04667	-0.46377	0.642814	NA
MSMO1	41.15051	0.021641	0.08143	0.26576	0.790424	NA
EIF2B4	121.5118	0.021638	0.045896	0.471453	0.637317	NA
TBCK	175.3811	0.021635	0.058396	0.370481	0.711024	NA
DPYS	0.398832	-0.02163	0.032003	-0.67601	0.499036	NA
LRRC48	48.74702	0.021633	0.064929	0.333174	0.739003	NA
SIAH3	27.31871	-0.02163	0.08452	-0.25589	0.798034	NA
MIR3186	0.91579	0.021626	0.052639	0.410846	0.681185	NA
SLC8A2	936.1625	0.021621	0.049566	0.436197	0.662694	0.855957
LYN	115.7627	0.021617	0.056665	0.381486	0.702843	NA

SRBD1	46.25921	0.021607	0.077958	0.277164	0.781654	NA
C15orf52	49.89746	0.021591	0.077278	0.279399	0.779939	NA
FANCD2OS	0.116916	-0.02159	0.019601	-1.10142	0.270714	NA
LRRC27	34.25378	-0.02158	0.067563	-0.31941	0.749416	NA
NCBP1	430.0922	-0.02158	0.039431	-0.54725	0.584204	0.824332
LOC10013	0.192868	-0.02158	0.025021	-0.86231	0.388518	NA
SLC38A7	33.77012	-0.02157	0.07093	-0.30408	0.761068	NA
ATP6V1B1	11.56922	0.021568	0.084939	0.253921	0.799557	NA
ATP1A4	11.13224	-0.02156	0.077671	-0.27763	0.781295	NA
APOA2	0.091783	-0.02156	0.016096	-1.33943	0.18043	NA
LCN15	0.55993	-0.02156	0.04179	-0.51589	0.60593	NA
LIX1	36.89098	-0.02156	0.084538	-0.25501	0.798718	NA
LOC10050	104.4059	0.021552	0.070932	0.303843	0.761247	NA
SVIP	64.45607	0.021549	0.067602	0.318767	0.749903	NA
CD2AP	191.1804	-0.02155	0.04734	-0.45513	0.649018	NA
CXCR7	5.849038	0.021543	0.082316	0.261717	0.79354	NA
FAM159A	1.167189	0.021543	0.052429	0.410905	0.681142	NA
NSDHL	27.9663	0.021542	0.071015	0.30335	0.761623	NA
CCDC178	0.31711	-0.02153	0.031906	-0.67495	0.499707	NA
ZNF43	95.60462	0.021527	0.063998	0.336375	0.736588	NA
DUS4L	12.39206	0.021523	0.08338	0.258129	0.796307	NA
TMEM254	15.7864	-0.02152	0.083747	-0.25694	0.797225	NA
NEURL4	217.5903	0.021516	0.044851	0.479719	0.631428	NA
MIR1200	1.33044	0.021514	0.060435	0.355981	0.721855	NA
PXK	266.3072	-0.02151	0.055537	-0.38732	0.69852	NA
FLJ39080	3.19172	-0.02151	0.068851	-0.31239	0.754745	NA
HBA1	0.977173	-0.02151	0.047833	-0.44963	0.652976	NA
VCAN	520.112	0.021493	0.075942	0.283026	0.777157	0.91728
SLC35A2	12.79009	0.021489	0.083534	0.257253	0.796984	NA
MIR556	0.102442	-0.02148	0.019222	-1.11746	0.263798	NA
CCDC19	13.46431	0.021478	0.084939	0.252867	0.800371	NA
MIR586	0.410341	-0.02148	0.038716	-0.55475	0.579067	NA
LOC10050	38.83769	-0.02148	0.077153	-0.27835	0.780742	NA
DYRK3	5.880626	-0.02146	0.082217	-0.26103	0.794068	NA
PIH1D2	6.777403	0.02146	0.083855	0.255916	0.798016	NA
KIAA0196	164.2792	-0.02145	0.047153	-0.45499	0.649117	NA
DDX55	99.37453	-0.02145	0.056228	-0.38154	0.702803	NA
RHBDL1	37.63415	-0.02145	0.07652	-0.28036	0.779204	NA
SCARNA6	13.73139	0.021453	0.084286	0.254521	0.799093	NA
MIR589	0.210797	0.021443	0.027614	0.776525	0.437439	NA
RTP4	0.533219	-0.02144	0.041292	-0.51927	0.603571	NA
MIR181D	0.433123	-0.02143	0.036253	-0.5912	0.554387	NA
RUNDC3A	673.7348	-0.02143	0.03548	-0.60395	0.545876	0.803341
LRRC37A1	0.266725	0.021425	0.030188	0.709719	0.477879	NA
FAR1	421.89	0.021424	0.040813	0.524926	0.599635	0.829672
DCDC2B	26.54569	0.021417	0.080168	0.267152	0.789352	NA
PTMA	657.7337	0.021416	0.060566	0.353602	0.723637	0.890297

ZNF618	187.3601	0.02141	0.064761	0.3306	0.740946	NA
ERCC8	27.90354	0.021403	0.076405	0.280126	0.779381	NA
AGFG1	324.1707	0.021401	0.034275	0.6244	0.532365	NA
BCAR4	1.172181	-0.0214	0.057451	-0.37245	0.709558	NA
STMND1	0.14032	0.021394	0.019986	1.070492	0.284398	NA
SLC2A2	1.257165	0.021394	0.052198	0.409861	0.681908	NA
LOC10050	0.714193	-0.02139	0.044174	-0.48419	0.628253	NA
ATXN3	238.2328	0.021384	0.038177	0.560137	0.575386	NA
MCM9	69.46658	-0.02138	0.052359	-0.40837	0.682999	NA
ZSWIM1	59.78202	0.021381	0.056678	0.377244	0.705992	NA
TIMP3	251.4735	0.021372	0.072982	0.292837	0.769647	NA
SSNA1	37.5828	-0.02137	0.068542	-0.31175	0.755227	NA
SNX6	113.0782	-0.02136	0.049703	-0.42984	0.667312	NA
LINC00340	2.11389	0.02136	0.068728	0.310794	0.755957	NA
LOC15476	2.418731	-0.02136	0.068423	-0.31216	0.75492	NA
RMI1	61.08163	0.021357	0.064588	0.330669	0.740895	NA
TRAPPC1	31.9012	-0.02136	0.0757	-0.28212	0.777849	NA
ZNF654	194.9026	0.021356	0.045414	0.470249	0.638177	NA
SIX2	0.350514	-0.02136	0.030994	-0.689	0.49082	NA
DEPDC5	148.1032	-0.02135	0.050064	-0.42653	0.66972	NA
NAPA	224.5723	0.021351	0.059655	0.357913	0.720409	NA
SMU1	315.5247	0.021345	0.034544	0.617908	0.536636	NA
ZNF490	137.5246	0.021337	0.051298	0.41595	0.677447	NA
BLCAP	126.7316	0.021337	0.05732	0.372239	0.709715	NA
MIR1206	0.116393	-0.02134	0.019541	-1.09184	0.274902	NA
PTCD3	405.1397	-0.02133	0.042583	-0.50097	0.616391	0.839018
NAT6	20.12386	0.021333	0.079529	0.268237	0.788517	NA
BMPER	125.0138	0.021324	0.073995	0.28818	0.773209	NA
XIRP1	0.917863	-0.02132	0.047545	-0.44837	0.653887	NA
BRWD1	1656.301	0.021313	0.036287	0.587352	0.556968	0.808857
KAT8	193.8566	-0.02131	0.049204	-0.43313	0.66492	NA
TMA16	24.11032	0.021307	0.082923	0.256944	0.797222	NA
FBXW5	178.2562	-0.0213	0.049292	-0.43219	0.665606	NA
ESR2	20.43492	0.021302	0.077326	0.275484	0.782944	NA
MUS81	78.30626	-0.0213	0.059093	-0.36044	0.718518	NA
GRK1	2.58752	0.021299	0.073041	0.29161	0.770585	NA
MIR548I1	0.137837	0.021299	0.01997	1.066542	0.286179	NA
TMEM255	1.37233	-0.0213	0.060863	-0.34994	0.726384	NA
ATP1B1	752.6968	-0.02128	0.080799	-0.26339	0.792251	0.926009
TMEM89	0.527715	-0.02128	0.039676	-0.53634	0.59172	NA
CRYAA	0.25996	-0.02128	0.029275	-0.72686	0.467313	NA
TMEM86B	22.93194	-0.02127	0.077685	-0.27385	0.784201	NA
NRXN3	2010.542	0.021261	0.032037	0.663658	0.506909	0.780812
PSMC5	234.0246	0.02126	0.046399	0.458202	0.646807	NA
MIR4796	1.295988	0.021247	0.060441	0.351535	0.725187	NA
NARG2	182.0529	0.021243	0.054556	0.389376	0.696998	NA
LOC73010	6.692469	-0.02124	0.083752	-0.2536	0.799808	NA

FKBP2	43.19599	0.021233	0.073333	0.289547	0.772163	NA
OR2M5	0.098714	-0.02123	0.016327	-1.30009	0.193569	NA
GUCY2D	0.35183	0.021225	0.036238	0.585713	0.558069	NA
DDX49	51.32482	0.021221	0.062762	0.33812	0.735273	NA
LOC10050	17.76869	-0.02122	0.081397	-0.26069	0.79433	NA
IFRD2	20.1284	0.02122	0.079451	0.267078	0.789409	NA
NTF3	18.58045	-0.02122	0.07985	-0.26573	0.790446	NA
HYPK	2.282682	0.021216	0.066796	0.317625	0.75077	NA
HIST1H2AI	0.090891	-0.02121	0.016084	-1.31872	0.187264	NA
SF3B14	44.0504	0.021205	0.071008	0.298621	0.76523	NA
LRRTM1	13.95562	-0.0212	0.082504	-0.25699	0.797189	NA
RPS6KB1	263.0698	0.021202	0.042749	0.495954	0.619927	NA
C10orf2	29.17074	-0.0212	0.07832	-0.27065	0.78666	NA
LGALS17A	0.545121	-0.0212	0.038454	-0.55122	0.581482	NA
KHDRBS1	472.4283	0.021192	0.057882	0.366128	0.71427	0.885992
TBX20	0.494874	-0.02119	0.04083	-0.51901	0.603755	NA
SOCS5	280.2304	0.021189	0.064568	0.328166	0.742786	NA
YEATS4	36.8857	-0.02118	0.079376	-0.26689	0.789555	NA
C9orf153	13.72717	0.021182	0.07816	0.271012	0.786382	NA
EXO5	32.32518	0.021179	0.072414	0.292466	0.76993	NA
MIR548G	1.930161	-0.02118	0.067378	-0.31432	0.753281	NA
EFHC1	83.92536	0.021173	0.064609	0.327719	0.743124	NA
FUT3	0.158032	-0.02117	0.020327	-1.0416	0.297597	NA
RBP7	1.697455	-0.02117	0.064661	-0.32743	0.74334	NA
SNORA11C	0.59672	-0.02117	0.043732	-0.48412	0.628299	NA
EMD	53.41512	0.021168	0.061874	0.342116	0.732263	NA
GPR146	24.21031	-0.02117	0.079542	-0.26611	0.790154	NA
RNU6-79	0.21124	-0.02116	0.026331	-0.80343	0.421726	NA
LOC10028	11.38847	-0.02115	0.084907	-0.24905	0.80332	NA
SLC41A1	247.1571	0.021138	0.054788	0.385813	0.699635	NA
WDR76	83.0784	-0.02113	0.060431	-0.34969	0.726568	NA
WFDC3	0.697583	0.021132	0.045725	0.462154	0.643971	NA
OR2M3	0.308979	0.021126	0.026847	0.786927	0.431324	NA
PLK3	13.56735	-0.02112	0.084183	-0.25093	0.801867	NA
PWRN2	0.309793	-0.02112	0.029477	-0.71648	0.473694	NA
CCT8	310.2181	0.021108	0.052163	0.404654	0.685732	NA
OR11L1	0.092726	-0.02111	0.016192	-1.30358	0.192378	NA
RRS1	31.00184	0.021105	0.080675	0.261611	0.793622	NA
CNOT11	86.22109	0.021096	0.055345	0.381166	0.70308	NA
ATP5C1	169.1146	-0.02109	0.067059	-0.3145	0.753144	NA
LINC00638	3.606824	0.021089	0.073092	0.288531	0.772941	NA
MIR4692	0.382388	0.021081	0.033822	0.623307	0.533083	NA
FAM210B	108.4726	0.021078	0.057892	0.364092	0.715789	NA
SERPINC1	1.079654	-0.02108	0.055106	-0.38245	0.702126	NA
SIVA1	16.15767	-0.02107	0.083148	-0.25339	0.799965	NA
TGM7	0.085462	-0.02107	0.016	-1.31669	0.187942	NA
TTC24	0.362506	-0.02106	0.03091	-0.68132	0.495669	NA

MIR133A1	0.660952	-0.02105	0.041408	-0.50847	0.611125	NA
SLCO1B1	0.190697	0.021042	0.023366	0.900522	0.367842	NA
RSPH9	8.430155	-0.02104	0.084595	-0.2487	0.803592	NA
MIR5684	0.110372	-0.02104	0.019415	-1.08351	0.278581	NA
SNORA14A	1.555785	0.02103	0.064061	0.328283	0.742698	NA
CNOT2	466.8917	0.021029	0.041784	0.503275	0.614771	0.837819
AAMP	136.4336	-0.02103	0.056037	-0.37524	0.707479	NA
TCEAL4	222.9461	0.021027	0.049575	0.42415	0.671457	NA
AASDHPPT	212.9709	0.021023	0.055506	0.378746	0.704876	NA
LOC65365	1.157172	-0.02102	0.054085	-0.38861	0.697566	NA
AGBL3	37.87436	0.021014	0.076333	0.275299	0.783086	NA
KIAA1467	198.6602	0.021002	0.065304	0.321597	0.747758	NA
DKFZp434	0.368384	0.020994	0.03323	0.631766	0.52754	NA
C19orf55	28.55992	0.020991	0.08024	0.261597	0.793632	NA
DKFZP686I	59.46137	-0.02099	0.06452	-0.32533	0.744932	NA
MIR320D1	1.556973	-0.02099	0.06361	-0.32993	0.741453	NA
CD164L2	0.402939	0.02098	0.037057	0.566167	0.57128	NA
GCDH	56.88583	-0.02097	0.073769	-0.2843	0.776182	NA
PRMT10	89.47175	0.020962	0.053964	0.388443	0.697688	NA
BHLHA15	0.495176	-0.02096	0.038638	-0.54236	0.58757	NA
CES5A	0.261107	0.020952	0.030179	0.694258	0.48752	NA
RPS2P32	4.80623	-0.02095	0.082265	-0.25464	0.799004	NA
FOXP3	5.465989	-0.02095	0.080814	-0.2592	0.79548	NA
SULT2B1	0.821232	-0.02094	0.051946	-0.40315	0.686838	NA
PPP2R2B-I	5.04586	-0.02094	0.080305	-0.26077	0.794271	NA
VBP1	73.8496	-0.02093	0.075189	-0.27843	0.780685	NA
GFI1	0.286433	0.020932	0.027877	0.750843	0.452747	NA
SNX2	197.2575	-0.02092	0.051507	-0.40622	0.68458	NA
LZTS2	76.35677	0.020922	0.05959	0.351108	0.725508	NA
CDRT1	3.313066	-0.02091	0.067783	-0.30855	0.757667	NA
MIR1179	0.649009	-0.02091	0.04684	-0.4464	0.655308	NA
PCDH17	134.1432	0.020909	0.078911	0.264969	0.791033	NA
TTC39C	78.90439	-0.02091	0.074421	-0.28093	0.778763	NA
PRDM11	160.1475	0.020904	0.060152	0.347522	0.728199	NA
CTDP1	80.64716	0.020901	0.065982	0.316778	0.751412	NA
GRIN3B	13.91846	-0.02089	0.084015	-0.24865	0.80363	NA
DNM1P41	0.816276	0.020886	0.04799	0.43521	0.66341	NA
SRL	3.230935	-0.02087	0.076537	-0.27272	0.785069	NA
TTC39B	181.2197	0.020869	0.060534	0.344748	0.730284	NA
MGC1614	23.16211	-0.02087	0.083836	-0.24891	0.803432	NA
DCUN1D2	179.8473	0.020865	0.051385	0.40605	0.684706	NA
MCM4	149.9408	0.020863	0.06315	0.330373	0.741118	NA
VPS45	289.0874	-0.02084	0.039721	-0.52453	0.599908	NA
ZBTB32	27.20398	0.020833	0.074162	0.280907	0.778781	NA
ZNF836	114.1836	0.020831	0.051837	0.401858	0.687788	NA
ZNF552	84.16781	-0.02083	0.065034	-0.32029	0.748747	NA
TOE1	26.80362	-0.02082	0.075775	-0.27476	0.783503	NA

DNAJA2	269.2615	-0.02081	0.053538	-0.38869	0.697506	NA
TRIM6	0.659047	-0.02081	0.042284	-0.49207	0.622672	NA
SERTAD4	13.68913	-0.02081	0.084879	-0.24512	0.806362	NA
UBE3C	446.1971	0.020803	0.042423	0.490362	0.623878	0.841191
KLRF1	0.17501	-0.0208	0.024376	-0.85332	0.393484	NA
FAS-AS1	0.727048	-0.02079	0.047551	-0.43724	0.66194	NA
MRPS26	75.29682	0.020788	0.058476	0.355495	0.722219	NA
43350	742.9824	0.020787	0.046619	0.445881	0.655683	0.854402
CLCA1	0.436792	0.020784	0.03609	0.575891	0.564689	NA
SMKR1	2.302991	0.020781	0.071372	0.291167	0.770923	NA
LOC72808	0.271523	0.02078	0.030224	0.68753	0.491749	NA
ATP6V0A2	186.6081	0.020774	0.044667	0.465099	0.64186	NA
MIR4472-1	0.09143	-0.02077	0.016229	-1.27949	0.200723	NA
TAMM41	68.36069	0.020763	0.069101	0.300474	0.763815	NA
SPINK8	6.05486	0.020763	0.083638	0.248245	0.803945	NA
KIFAP3	325.686	-0.02076	0.045814	-0.45305	0.650511	NA
FST	0.092515	-0.02075	0.016256	-1.27666	0.201721	NA
DERL1	103.3064	0.020751	0.058264	0.356159	0.721722	NA
DNAJC11	160.6424	0.020742	0.045072	0.460191	0.645379	NA
NTRK1	0.298215	-0.02073	0.032953	-0.62902	0.529339	NA
VWA5B2	106.1563	0.02072	0.066163	0.313166	0.754155	NA
RBM17	326.2977	-0.02072	0.030658	-0.6757	0.499229	NA
TIMM9	35.99585	-0.02071	0.071151	-0.29102	0.771036	NA
HNRPDL	945.3399	-0.0207	0.058458	-0.35418	0.723201	0.890244
POMP	97.26379	0.020703	0.066278	0.312372	0.754758	NA
PRSS1	0.382163	-0.0207	0.030767	-0.6729	0.50101	NA
CYC1	104.4327	-0.0207	0.065753	-0.31486	0.752865	NA
WFIKKN1	28.30131	0.020703	0.084822	0.244074	0.807173	NA
PIR	7.671418	0.020702	0.083958	0.24657	0.805241	NA
INSL3	0.704848	-0.02069	0.045212	-0.45767	0.647188	NA
ARAF	71.68592	-0.02069	0.056513	-0.36612	0.714274	NA
HLA-F	29.1748	-0.02069	0.084192	-0.24573	0.80589	NA
PDLIM3	64.27757	0.020688	0.07928	0.260949	0.794131	NA
TFAMP1	2.286245	0.020684	0.065193	0.317272	0.751037	NA
SAP130	143.1899	0.020682	0.047036	0.439711	0.660147	NA
PROX2	40.80045	0.020667	0.084935	0.243333	0.807748	NA
GALNT3	14.58147	-0.02067	0.082664	-0.24999	0.802593	NA
RRP8	48.25176	0.020659	0.067476	0.306164	0.75948	NA
SNORD42A	4.365958	-0.02066	0.079328	-0.26038	0.794569	NA
PAOX	27.34563	-0.02065	0.078387	-0.2635	0.792166	NA
TMEM123	94.84649	-0.02065	0.070237	-0.29406	0.76871	NA
AZI1	118.5283	-0.02065	0.064039	-0.32252	0.747057	NA
NMD3	223.0792	0.020651	0.047622	0.433649	0.664543	NA
C1orf52	80.32327	0.020651	0.056971	0.362486	0.716989	NA
DMXL2	935.0732	0.020651	0.049273	0.419112	0.675134	0.863513
C14orf37	146.2006	0.020646	0.078161	0.264141	0.791671	NA
PCM1	2117.49	-0.02064	0.033332	-0.61931	0.535711	0.796707

LINC00607	1.193461	0.020641	0.05258	0.392559	0.694645	NA
ZNF431	153.458	0.020632	0.054062	0.38163	0.702736	NA
NOC2L	142.0631	-0.02063	0.055054	-0.37469	0.707891	NA
OR4F21	0.083389	-0.02062	0.014474	-1.42493	0.154177	NA
ATP6V1C1	405.9558	0.020621	0.055989	0.368303	0.712647	0.884585
GSKIP	26.03876	0.020621	0.080975	0.254658	0.798987	NA
LEF1-AS1	0.135984	0.020617	0.020016	1.030054	0.302985	NA
SNHG9	1.736156	-0.02062	0.063852	-0.32289	0.746779	NA
FAM150B	26.51773	0.020617	0.084576	0.243763	0.807415	NA
ORC3	156.4224	-0.02062	0.050922	-0.40485	0.685587	NA
MXD1	230.0902	0.020612	0.054381	0.379039	0.704659	NA
NPSR1	1.05675	0.020608	0.046938	0.439054	0.660622	NA
MIR4521	0.381736	0.020608	0.031742	0.649248	0.516178	NA
CPEB2	249.5412	0.020608	0.046821	0.440152	0.659827	NA
METTL1	3.678016	-0.0206	0.074749	-0.27565	0.782815	NA
VAMP2	462.6644	0.020602	0.064761	0.318122	0.750393	0.902144
BCAM	27.03045	-0.0206	0.082963	-0.24832	0.803884	NA
RAD50	570.5033	0.0206	0.038843	0.530354	0.595867	0.829672
CCNF	60.3156	-0.02059	0.065494	-0.31443	0.753191	NA
CSNK2A3	11.73771	-0.02059	0.083286	-0.24723	0.804728	NA
OR14A16	0.103298	-0.02058	0.019349	-1.06377	0.287431	NA
FLG	9.073049	0.020575	0.067006	0.30706	0.758797	NA
FAM198B	138.1971	-0.02057	0.082157	-0.25042	0.802263	NA
USP54	346.4911	-0.02057	0.046617	-0.44133	0.658977	0.855675
FLT1	448.8237	0.020566	0.075898	0.270971	0.786413	0.924656
SLC19A1	50.53206	0.020563	0.07825	0.262792	0.792711	NA
HIST1H3C	0.871154	0.020557	0.049237	0.417504	0.67631	NA
SNORD75	1.063122	0.020544	0.055149	0.372523	0.709503	NA
CCR6	0.730773	-0.02054	0.046169	-0.44497	0.65634	NA
ATXN7	367.4177	0.020531	0.046255	0.443875	0.657133	0.854783
GPAT2	13.6266	-0.02053	0.084527	-0.24285	0.808123	NA
SNX1	332.6217	-0.02053	0.031328	-0.65521	0.512332	NA
ZNF585B	133.4843	0.020518	0.045026	0.45568	0.64862	NA
MORN4	59.25928	-0.02051	0.071638	-0.28633	0.774622	NA
TBRG1	195.4854	-0.0205	0.036274	-0.56527	0.571889	NA
STRN3	219.9106	0.020501	0.038219	0.536426	0.591664	NA
CLEC12B	0.151718	-0.0205	0.023025	-0.89036	0.373275	NA
AQP3	6.651035	-0.02049	0.078851	-0.25987	0.794964	NA
MYO1B	24.31349	0.020491	0.08338	0.245748	0.805877	NA
ICAM5	0.116323	0.02049	0.019654	1.042527	0.297168	NA
SUMF2	85.33675	-0.02049	0.071484	-0.28664	0.774392	NA
RP1	0.407337	-0.02049	0.035911	-0.57051	0.568331	NA
C11orf71	22.54471	0.020483	0.081175	0.252337	0.80078	NA
ABCC6P1	0.357234	0.020472	0.035194	0.581695	0.560772	NA
LAMB1	249.3234	-0.02047	0.079034	-0.25902	0.795622	NA
TCTN2	75.30451	-0.02047	0.06625	-0.30899	0.75733	NA
RBM8A	98.53396	0.02047	0.056739	0.360774	0.718269	NA

GPRASP1	1038.739	-0.02047	0.041069	-0.49838	0.618214	0.839739
CYP1A1	0.49202	0.020468	0.038107	0.53712	0.591185	NA
CCDC33	0.180158	-0.02047	0.024442	-0.83741	0.402361	NA
ANKRD61	11.92878	-0.02047	0.083726	-0.24446	0.806876	NA
LEKR1	3.816884	0.020467	0.077887	0.262779	0.792721	NA
P2RX6P	0.649633	-0.02046	0.0462	-0.44296	0.657795	NA
ZDHHC23	24.01084	0.020464	0.083587	0.244824	0.806593	NA
MTMR3	442.5348	-0.02046	0.042531	-0.48103	0.630497	0.844999
TRIAP1	20.12375	0.020458	0.083522	0.244942	0.806501	NA
TSEN54	92.44158	-0.02045	0.075668	-0.27024	0.786977	NA
DYNC2H1	639.286	-0.02045	0.050924	-0.40151	0.688045	0.870512
LMAN1	309.2787	0.020441	0.042951	0.47592	0.634131	NA
MC4R	0.78919	-0.02044	0.041836	-0.4885	0.625192	NA
FAHD1	45.07853	0.020436	0.067445	0.303007	0.761885	NA
NPRL2	63.57593	-0.02043	0.064213	-0.31819	0.75034	NA
MIR147A	0.079258	-0.02043	0.014406	-1.41811	0.156159	NA
PKD2L1	0.286327	-0.02043	0.026764	-0.76326	0.445309	NA
CYB561	22.39545	-0.02042	0.077323	-0.26412	0.791685	NA
TMEM217	1.02805	0.020421	0.054869	0.372174	0.709764	NA
ANKS1B	1056.952	0.020415	0.050261	0.406181	0.684609	0.86822
RUSC1	264.3698	0.020415	0.05341	0.382227	0.702293	NA
EID2B	84.14006	-0.02041	0.061298	-0.33301	0.739126	NA
C10orf118	520.9568	-0.02041	0.042661	-0.47842	0.63235	0.844999
BCR	436.7878	-0.02041	0.072049	-0.28327	0.776967	0.91728
KPNA1	366.5473	0.020409	0.033274	0.61336	0.539639	0.800446
LGI2	115.3147	-0.02041	0.084939	-0.24028	0.810116	NA
TAF1D	206.0494	-0.0204	0.071551	-0.28504	0.775611	NA
CUL7	219.0039	-0.02039	0.051313	-0.39738	0.691089	NA
LDHB	526.2085	0.020386	0.066792	0.305219	0.7602	0.90815
SNORD115	1.233181	-0.02038	0.050533	-0.40338	0.686666	NA
ADAMDEC	0.630312	-0.02038	0.041344	-0.49282	0.62214	NA
RNF128	4.500238	0.020373	0.082015	0.248412	0.803816	NA
HPS1	102.4458	0.020369	0.05144	0.395975	0.692124	NA
SEPT5-GP1	91.39611	0.020368	0.075864	0.268482	0.788329	NA
PCNP	296.2181	0.020364	0.052095	0.390909	0.695865	NA
TAF1	578.8173	-0.02035	0.047198	-0.43116	0.666354	0.859017
CDX2	0.234736	-0.02035	0.025183	-0.80802	0.419082	NA
ASAH2	0.800962	-0.02035	0.050372	-0.40394	0.686257	NA
SLC25A2	1.318266	-0.02034	0.047657	-0.42688	0.669464	NA
AKT1	218.8783	0.020342	0.048973	0.415367	0.677873	NA
KDELC1	28.05563	0.020339	0.082633	0.246136	0.805577	NA
SLC2A14	1.560214	-0.02033	0.055264	-0.36789	0.712954	NA
DDX18	192.4898	0.020325	0.046328	0.438716	0.660867	NA
LOC10024	0.095036	0.020322	0.016208	1.253802	0.209914	NA
MTCH2	164.2334	0.020322	0.059435	0.341911	0.732418	NA
MBNL2	684.4034	0.020321	0.044618	0.455451	0.648785	0.850896
NAV3	409.104	0.020317	0.048277	0.420835	0.673875	0.863513

RPAP2	271.7361	0.020315	0.034508	0.588717	0.556051	NA
CYP2C19	0.479736	0.020314	0.036351	0.558828	0.576279	NA
ELMSAN1	458.7727	-0.02031	0.06076	-0.33423	0.738207	0.895927
NABP1	8.386343	-0.0203	0.084184	-0.24118	0.809413	NA
MROH6	38.78306	0.0203	0.084099	0.241384	0.809258	NA
HAGHL	35.51014	-0.02028	0.073693	-0.27521	0.783152	NA
SPNS2	45.46475	0.020279	0.077928	0.260233	0.794684	NA
NPSR1-AS	0.637131	-0.02027	0.040839	-0.49645	0.619578	NA
MIR302D	0.39353	-0.02027	0.033777	-0.60016	0.5484	NA
SMAD6	16.78567	-0.02027	0.083958	-0.24142	0.809227	NA
PRSS42	0.459263	-0.02026	0.03224	-0.62854	0.52965	NA
USP44	73.03391	0.020263	0.070848	0.286008	0.774872	NA
SRP54	158.7396	-0.02026	0.046342	-0.4371	0.66204	NA
ACOT8	52.73763	-0.02025	0.059467	-0.34061	0.733401	NA
RILPL2	17.62856	0.02025	0.081288	0.249117	0.80327	NA
LOC65351	40.52979	0.020249	0.084576	0.239414	0.810785	NA
CDH24	40.49387	0.020241	0.073593	0.275033	0.783291	NA
FSCB	0.165944	-0.02024	0.02429	-0.83324	0.404708	NA
IL12RB1	0.491394	0.020232	0.038517	0.525282	0.599387	NA
CBWD3	0.837481	0.020231	0.048086	0.420721	0.673959	NA
GCC2	378.0965	-0.02022	0.062146	-0.32538	0.744892	0.898871
POLR2J	68.47627	0.02021	0.069901	0.28912	0.77249	NA
C4orf51	0.50755	0.020208	0.039699	0.509027	0.610733	NA
ZNF815P	43.11564	-0.02021	0.071952	-0.28085	0.778824	NA
NHLH2	37.3615	-0.02021	0.082722	-0.24428	0.807016	NA
CKMT2	38.67454	-0.02019	0.079365	-0.25445	0.799147	NA
ELF4	2.645755	-0.02019	0.072115	-0.28	0.779475	NA
ZC3H14	636.4655	0.020176	0.038913	0.518498	0.604111	0.830918
OR2S2	0.339861	0.020175	0.030731	0.6565	0.511503	NA
ZNF2	27.30796	-0.02017	0.072572	-0.27799	0.781018	NA
C8G	1.989355	0.020165	0.068424	0.294715	0.768211	NA
SCARNA11	7.145922	-0.02015	0.083629	-0.24099	0.809562	NA
SMS	202.0162	-0.02015	0.060668	-0.33213	0.739789	NA
EIF4A3	79.6195	0.020147	0.057271	0.351794	0.724993	NA
AIP	97.69371	-0.02015	0.070377	-0.28625	0.774687	NA
LMX1B	0.498534	0.020145	0.039037	0.516049	0.60582	NA
ULK4P3	0.176428	0.020134	0.023277	0.864949	0.387067	NA
MIR4655	0.127223	0.020132	0.019905	1.011387	0.311831	NA
TMEM136	77.65906	-0.02013	0.061134	-0.32929	0.741933	NA
TDGF1P3	0.313356	0.020129	0.033181	0.606655	0.54408	NA
CNN1	3.784197	-0.02013	0.063935	-0.31482	0.752902	NA
GRK7	1.372503	0.020126	0.060076	0.335006	0.737621	NA
KIAA1967	313.4005	0.02012	0.03757	0.535538	0.592278	NA
CTSE	0.097865	0.020112	0.016206	1.240993	0.214608	NA
ADAM11	371.9423	-0.02011	0.050115	-0.40124	0.68824	0.870512
SS18L2	17.43519	0.020107	0.082107	0.244885	0.806545	NA
C1orf186	0.26696	-0.0201	0.02812	-0.71492	0.47466	NA

ORM2	0.182324	-0.0201	0.024491	-0.82087	0.411722	NA
FAR2	63.39086	0.020103	0.070185	0.286425	0.774553	NA
SLC36A3	1.22547	-0.0201	0.054018	-0.37211	0.70981	NA
TMEM203	40.55629	-0.0201	0.068138	-0.29496	0.768023	NA
PARP14	84.17855	0.020097	0.084193	0.238705	0.811334	NA
DHRS2	0.347896	-0.0201	0.033648	-0.59725	0.550339	NA
LINC00652	1.405419	-0.02009	0.057468	-0.34966	0.72659	NA
RTTN	187.7651	-0.02009	0.051376	-0.39112	0.69571	NA
INTS4	106.8121	-0.02009	0.049282	-0.40771	0.683486	NA
KRTAP5-8	0.444281	-0.02009	0.038416	-0.52301	0.600965	NA
FAM200B	176.2583	0.020083	0.053971	0.37211	0.709811	NA
MIR4798	0.197247	-0.02008	0.025068	-0.80114	0.423049	NA
PLSCR5	0.102396	0.020079	0.016226	1.237471	0.215912	NA
BEND3	31.12912	0.020075	0.073344	0.273715	0.784304	NA
AIM1L	0.248015	0.020072	0.030157	0.665594	0.50567	NA
C14orf166	104.981	-0.02007	0.060889	-0.32956	0.741735	NA
PTCHD4	22.58396	-0.02006	0.084551	-0.2373	0.812426	NA
CLIC6	0.546861	-0.02006	0.039449	-0.50859	0.611038	NA
SNORD114	195.0177	-0.02006	0.083715	-0.23963	0.810615	NA
PRKACG	1.078633	0.020061	0.053643	0.373965	0.70843	NA
SLC38A6	69.87531	-0.02005	0.066736	-0.30049	0.763804	NA
FREM3	0.339181	-0.02005	0.032971	-0.60797	0.543204	NA
NXPH2	2.102722	0.020039	0.057853	0.346385	0.729054	NA
C5orf63	34.74695	-0.02004	0.075775	-0.26443	0.791449	NA
DDX31	143.221	0.020027	0.064082	0.31252	0.754645	NA
LOC10050	45.34198	0.020026	0.079403	0.252211	0.800878	NA
ZNF93	16.04336	0.020021	0.08399	0.238372	0.811593	NA
CDKN3	14.19016	0.02002	0.084924	0.235736	0.813638	NA
ARRDC4	38.03878	-0.02002	0.078727	-0.25429	0.799275	NA
RTL1	58.02847	-0.02	0.081052	-0.24674	0.805108	NA
RNF170	136.1068	-0.02	0.052214	-0.38301	0.701714	NA
UG0898H0	15.93581	-0.02	0.084939	-0.23541	0.813893	NA
TTR	1.6568	0.019993	0.045365	0.440709	0.659423	NA
SLC39A14	121.4905	0.019993	0.072653	0.27518	0.783178	NA
UFSP1	4.895112	-0.01998	0.07833	-0.25511	0.79864	NA
ZNF714	232.1877	-0.01998	0.080048	-0.24956	0.80293	NA
TANC2	345.1549	0.019971	0.050547	0.395098	0.69277	0.871418
MYCBPAP	23.2358	0.019969	0.084884	0.235258	0.814009	NA
VIPR1	123.9797	0.019969	0.061873	0.322734	0.746897	NA
FABP1	0.172314	-0.01996	0.024375	-0.81899	0.412794	NA
ITGA6	80.28679	0.019962	0.078518	0.254236	0.799313	NA
TRIM9	1545.409	-0.01996	0.041674	-0.47894	0.631984	0.844999
PSEN1	224.8048	-0.01996	0.04563	-0.43738	0.661837	NA
TOB2	586.0606	0.019949	0.055229	0.361198	0.717951	0.887792
CBLN4	0.415675	-0.01995	0.038338	-0.52033	0.602833	NA
MCF2L	2207.868	-0.01995	0.05264	-0.3789	0.704762	0.881439
IGSF11-AS	1.710816	0.019944	0.062346	0.319889	0.749053	NA

DDX51	156.7192	-0.01994	0.057894	-0.3444	0.730546	NA
SEMA4D	203.159	-0.01993	0.052951	-0.37641	0.706612	NA
FAM180A	0.257689	-0.01993	0.030226	-0.65938	0.50965	NA
MUL1	43.05549	0.01993	0.066271	0.300736	0.763616	NA
AKAP1	269.5022	0.019923	0.040065	0.49728	0.618992	NA
CABLES2	115.4325	0.019923	0.059672	0.333869	0.738478	NA
RING1	106.6909	0.01992	0.049526	0.402213	0.687528	NA
ATP8B5P	1.955928	-0.01991	0.066053	-0.30143	0.763085	NA
IMPA2	2.31022	-0.01991	0.067363	-0.29554	0.76758	NA
CMTM3	37.10647	-0.01991	0.077595	-0.25657	0.797511	NA
KIAA1704	212.8537	-0.01991	0.047749	-0.41693	0.676732	NA
ABR	446.3789	-0.0199	0.050463	-0.39439	0.69329	0.871418
HYAL1	38.17215	-0.0199	0.067607	-0.29437	0.768472	NA
ENTPD1	238.5459	-0.0199	0.048711	-0.40854	0.682874	NA
CCL3	0.069203	-0.01989	0.014216	-1.39933	0.161714	NA
NAGK	75.02779	-0.01988	0.065009	-0.30585	0.759716	NA
RAET1E	7.581139	-0.01988	0.08434	-0.23575	0.81363	NA
BDH1	205.0637	-0.01988	0.06402	-0.31054	0.756149	NA
CDSN	1.620683	0.01987	0.052426	0.379019	0.704674	NA
SPINK9	4.028173	-0.01987	0.080651	-0.24633	0.805426	NA
FKBP1B	27.95998	-0.01987	0.07421	-0.26771	0.788923	NA
CDK5	39.84771	0.019866	0.072166	0.275288	0.783095	NA
NRF1	113.7327	-0.01985	0.057766	-0.34368	0.731089	NA
KLF6	132.3106	0.019853	0.05932	0.334669	0.737875	NA
PTPN9	83.91445	0.019849	0.060219	0.329605	0.741699	NA
ZNF845	60.18073	0.019848	0.063809	0.311049	0.755763	NA
SNORD121	0.786016	-0.01985	0.049544	-0.4006	0.688713	NA
RAB33A	19.34473	-0.01985	0.081272	-0.24419	0.807082	NA
SERTM1	0.436447	0.019845	0.036882	0.538084	0.590519	NA
RNF41	206.8388	0.019842	0.043703	0.454014	0.649819	NA
RNU6-71	3.724879	-0.01984	0.070316	-0.28214	0.777837	NA
FOLR2	0.397069	0.019837	0.034621	0.572976	0.566661	NA
C9orf96	31.28304	-0.01984	0.082678	-0.23992	0.810391	NA
ARHGAP22	79.62514	-0.01984	0.082392	-0.24075	0.809752	NA
MIR7-2	0.459622	-0.01983	0.032914	-0.6025	0.546844	NA
ZNF783	92.70036	-0.01983	0.066333	-0.29895	0.76498	NA
L3MBTL2	141.5857	-0.01982	0.045922	-0.4317	0.665957	NA
SSTR2	114.6262	-0.01982	0.084232	-0.23534	0.813943	NA
RAX2	0.986155	0.019822	0.050377	0.393481	0.693965	NA
PNPLA5	1.59023	0.019816	0.062854	0.315273	0.752555	NA
IFIT1B	6.302091	-0.01981	0.083542	-0.23716	0.81253	NA
DDX42	612.8779	-0.01981	0.060106	-0.32962	0.74169	0.896704
CILP2	1.063395	0.019811	0.055277	0.358402	0.720043	NA
ACY1	1.927196	-0.01981	0.066037	-0.29998	0.764196	NA
TIGD3	14.84475	-0.01981	0.084793	-0.23359	0.815306	NA
RPL34-AS1	5.525959	0.019803	0.075591	0.261971	0.793344	NA
ALX4	9.28593	-0.01979	0.084862	-0.23325	0.815568	NA

ATP6V0D2	0.344032	0.019787	0.033295	0.594283	0.552323	NA
APTX	82.797	-0.01979	0.05676	-0.3486	0.727387	NA
PRKAR2A	114.7365	0.019774	0.048886	0.404483	0.685858	NA
NLRC4	13.19527	0.019759	0.084282	0.234438	0.814645	NA
WDTC1	231.6867	-0.01976	0.04208	-0.46955	0.638677	NA
LLGL2	72.89054	-0.01975	0.084837	-0.23284	0.815885	NA
PIP5K1C	892.5811	0.019752	0.049604	0.398198	0.690484	0.870767
LYNX1	207.5629	0.01975	0.067499	0.292594	0.769833	NA
MTFMT	45.16367	-0.01974	0.074286	-0.26579	0.790398	NA
KCNAB2	205.6795	-0.01974	0.052504	-0.37602	0.706905	NA
SNORD115	5.587527	-0.01974	0.081442	-0.24237	0.808491	NA
MS4A1	0.095186	0.019738	0.01618	1.219872	0.222514	NA
HES2	0.181389	0.019736	0.023361	0.84483	0.398206	NA
ZUFSP	75.94848	0.019728	0.053081	0.37166	0.710146	NA
CD37	6.421262	-0.01971	0.080144	-0.24596	0.805717	NA
MATN1-AS	1.539066	-0.0197	0.062844	-0.31341	0.753966	NA
ZER1	293.4265	-0.0197	0.04646	-0.42393	0.671618	NA
PIRT	19.98126	0.019692	0.084727	0.23242	0.816212	NA
MIRLET7I	0.903052	-0.01969	0.051079	-0.38543	0.699922	NA
NCAPG	12.24495	-0.01968	0.084783	-0.23216	0.816414	NA
ARHGAP24	27.34974	0.019682	0.080027	0.245942	0.805727	NA
CDC42SE2	178.2492	0.019677	0.050601	0.388869	0.697373	NA
ANKLE1	26.58095	-0.01968	0.08308	-0.23683	0.812789	NA
CROT	55.25172	-0.01967	0.06552	-0.30021	0.76402	NA
ARNT	368.4784	-0.01967	0.040654	-0.4838	0.628525	0.84337
JMJD1C-AS	19.24618	-0.01966	0.08319	-0.23638	0.813136	NA
COPRS	65.71275	0.019664	0.073937	0.265955	0.790274	NA
RAB24	74.03646	0.01966	0.066527	0.295523	0.767594	NA
SIRPD	0.221997	0.019659	0.026251	0.748903	0.453916	NA
ZFP69	27.1816	0.019655	0.074003	0.265596	0.79055	NA
GLUD1P3	98.79145	-0.01965	0.06666	-0.29484	0.768118	NA
DARS2	56.15536	-0.01965	0.062343	-0.31522	0.752593	NA
NOMO1	65.75624	0.019648	0.073232	0.268299	0.788469	NA
OGT	1199.958	-0.01965	0.061957	-0.31709	0.751177	0.902608
CELSR1	5.803569	0.019638	0.076138	0.257927	0.796463	NA
PRR7-AS1	12.73088	0.019637	0.084806	0.231549	0.816888	NA
CYTH1	334.3489	0.019625	0.05002	0.392344	0.694804	NA
SERPINF2	3.609761	0.019611	0.067511	0.290489	0.771442	NA
MIR3146	0.247233	0.019609	0.030152	0.650338	0.515474	NA
SYNRG	311.8806	-0.01961	0.040539	-0.48366	0.628627	NA
BOD1L2	0.172462	-0.01961	0.023222	-0.84434	0.398477	NA
FAM45B	1.790636	-0.01961	0.067373	-0.29102	0.77104	NA
E2F5	9.005921	-0.0196	0.084752	-0.2313	0.817078	NA
RORA	1133.908	-0.0196	0.063156	-0.31032	0.756317	0.90638
RUFY3	1051.073	-0.01959	0.029484	-0.66434	0.506472	0.780812
EID3	25.03363	-0.01959	0.077728	-0.25199	0.801051	NA
METTL23	21.05093	0.019582	0.077574	0.252431	0.800708	NA

SCMH1	111.3437	0.019581	0.058353	0.335552	0.737209	NA
AP3B2	455.6786	0.019569	0.04929	0.397009	0.691361	0.87093
PCMTD2	435.7688	-0.01956	0.047984	-0.40768	0.683511	0.867558
CELA2B	17.68681	-0.01955	0.083754	-0.23347	0.815397	NA
KCNH6	0.889444	0.019552	0.050908	0.384073	0.700924	NA
PRKCH	20.47181	0.019552	0.084436	0.23156	0.816879	NA
GPR176	180.158	-0.01955	0.06452	-0.30298	0.761908	NA
PCNXL3	214.5839	0.019546	0.054476	0.358799	0.719745	NA
BACE2	7.62229	0.019546	0.081582	0.239582	0.810654	NA
SENP7	385.4111	-0.01954	0.034218	-0.57114	0.567905	0.815149
SENP5	300.2559	0.01954	0.035477	0.550775	0.581788	NA
SAMSN1-A	0.083644	0.019538	0.0161	1.213528	0.224928	NA
PARD3B	35.74951	0.019538	0.075247	0.259648	0.795135	NA
INTS2	148.6214	-0.01953	0.054081	-0.36118	0.717964	NA
APOL6	39.16063	-0.01953	0.084706	-0.23054	0.817672	NA
C6orf123	0.383141	-0.01953	0.035232	-0.55425	0.579408	NA
ADAM21	17.34133	-0.01953	0.082059	-0.23796	0.811911	NA
H1FNT	1.312193	0.019525	0.05441	0.358851	0.719706	NA
SHISA8	159.3222	-0.01953	0.05589	-0.34935	0.726827	NA
SLA2	22.13608	0.019523	0.084918	0.229901	0.818169	NA
NTM	587.3135	0.019522	0.042951	0.454518	0.649456	0.851284
SRPK1	301.4855	0.019519	0.042938	0.45458	0.649411	NA
UCP1	5.156729	0.019512	0.076376	0.255468	0.798362	NA
OXT	0.661839	0.019512	0.045212	0.431556	0.666064	NA
SLC1A4	203.8358	0.019507	0.043487	0.448562	0.653747	NA
TMEM45B	8.113985	0.019501	0.084525	0.230708	0.817542	NA
SLC39A9	136.0742	0.019471	0.054792	0.355367	0.722315	NA
KANK2	59.72285	0.019461	0.078362	0.248353	0.803861	NA
ITFG3	68.8491	0.01946	0.061816	0.314806	0.752909	NA
SYNE3	28.44578	-0.01946	0.082015	-0.23727	0.812445	NA
IQUB	65.05246	-0.01945	0.078949	-0.24637	0.8054	NA
ATG5	83.99642	0.019449	0.059465	0.327074	0.743612	NA
TRIM24	136.2522	0.019446	0.049222	0.395066	0.692794	NA
FAM71E2	1.179774	-0.01944	0.054055	-0.35968	0.719089	NA
LRRTM4	15.17469	0.01944	0.084163	0.230981	0.817329	NA
TAGAP	12.13661	0.019433	0.084907	0.228872	0.818968	NA
GPR4	14.38279	-0.01942	0.077246	-0.25137	0.801527	NA
LOC10013	0.306418	-0.0194	0.029527	-0.6572	0.51105	NA
GPC3	1.578824	0.019401	0.057615	0.336737	0.736315	NA
FAM106CP	11.57924	-0.0194	0.08049	-0.24102	0.809536	NA
SAMD14	329.1479	0.019399	0.048839	0.397192	0.691226	NA
ZNF98	1.334772	-0.01939	0.057557	-0.33696	0.736149	NA
RBM6	872.1895	-0.01939	0.05634	-0.34424	0.73067	0.892995
SMC1B	12.34894	-0.01939	0.084462	-0.22959	0.818414	NA
SP5	3.190795	-0.01937	0.073006	-0.26536	0.790732	NA
HIST1H2A	0.27488	-0.01937	0.029288	-0.66134	0.508395	NA
MIR1322	0.195222	0.019367	0.027028	0.716569	0.47364	NA

TREML2	0.444139	0.019365	0.037789	0.512461	0.608328	NA
MAPK1IP1	266.5175	-0.01935	0.043871	-0.44113	0.659116	NA
LOC28581	13.56818	0.019353	0.084886	0.227984	0.819659	NA
SLC25A5	57.46607	-0.01935	0.083387	-0.23202	0.816523	NA
LOC38849	0.181651	0.019346	0.023402	0.82669	0.408413	NA
LOC10050	21.74314	-0.01933	0.084618	-0.22849	0.819267	NA
GPR137	48.42	-0.01933	0.064653	-0.299	0.764941	NA
C10orf129	7.500478	-0.01932	0.079444	-0.24324	0.807821	NA
F11	1.925219	-0.01931	0.062656	-0.30826	0.757882	NA
RIMS2	1413.605	0.019311	0.037112	0.520338	0.602828	0.830918
UBAP1	221.2848	0.019307	0.041229	0.468291	0.639577	NA
ARFGAP1	154.6833	-0.0193	0.051945	-0.37157	0.710216	NA
PTPRQ	0.109066	-0.01929	0.01789	-1.07847	0.280824	NA
F2R	0.966497	-0.01929	0.050186	-0.38434	0.70073	NA
HMG20A	249.0577	0.019287	0.045547	0.423459	0.67196	NA
SNORA71A	2.879207	-0.01928	0.075267	-0.25617	0.797816	NA
FAM53A	11.41912	-0.01928	0.084811	-0.22729	0.8202	NA
SYCE3	0.538318	-0.01927	0.041122	-0.46853	0.639406	NA
MFSD6L	0.080033	0.019263	0.016066	1.199024	0.230519	NA
GPR157	0.507922	0.019263	0.037523	0.513353	0.607704	NA
WRNIP1	149.8658	0.019262	0.040971	0.470153	0.638246	NA
SRFBP1	114.6544	0.019242	0.05009	0.384144	0.700872	NA
ACP2	59.76945	-0.01924	0.065522	-0.29366	0.769014	NA
TFEC	4.213695	-0.01924	0.076043	-0.25295	0.800307	NA
HERC2P3	82.50798	0.019234	0.077281	0.24889	0.803446	NA
GPR143	2.70094	-0.01923	0.069737	-0.27573	0.782753	NA
CD40LG	0.152181	-0.01923	0.020344	-0.9451	0.344608	NA
RPRD2	757.4733	0.01922	0.037727	0.509455	0.610433	0.834011
TCIRG1	13.55219	0.019214	0.084714	0.226815	0.820568	NA
TRIM21	8.331974	-0.01921	0.08396	-0.22881	0.819019	NA
MAGOHB	75.40118	0.01921	0.070687	0.271755	0.78581	NA
CD8B	2.451753	0.019207	0.061727	0.311167	0.755674	NA
CAPN7	385.3865	-0.0192	0.036563	-0.52515	0.599476	0.829672
MIR1178	0.217487	0.019197	0.027187	0.706098	0.480127	NA
FCRL5	0.074792	-0.01919	0.015861	-1.20972	0.226387	NA
TMEM229	12.61184	0.019181	0.084213	0.227764	0.81983	NA
CYP4F2	0.18724	0.019173	0.023493	0.816097	0.414445	NA
STX7	398.7225	-0.01917	0.04377	-0.43791	0.661451	0.855957
SLC44A1	247.7278	0.019166	0.053974	0.355095	0.722518	NA
PARN	157.0222	-0.01916	0.060536	-0.31652	0.751608	NA
GNL3L	163.2784	-0.01916	0.048703	-0.39336	0.694053	NA
IBA57	146.594	0.019154	0.060045	0.318995	0.74973	NA
USP6NL	57.74983	-0.01915	0.071857	-0.26655	0.789819	NA
PDIA5	6.632361	-0.01915	0.084351	-0.22706	0.82038	NA
GNS	148.08	-0.01915	0.048792	-0.39253	0.694669	NA
ZNF414	24.97109	-0.01915	0.078508	-0.24392	0.807296	NA
CN5H6.4	3.601198	0.019143	0.076624	0.24983	0.802719	NA

GET4	120.0177	-0.01914	0.04759	-0.40221	0.687527	NA
C4orf21	178.1125	-0.01914	0.057544	-0.33262	0.739424	NA
ZBBX	0.830357	0.019133	0.043182	0.443082	0.657706	NA
SQSTM1	530.3309	-0.01913	0.041288	-0.46323	0.6432	0.848802
C2CD3	264.4213	-0.01912	0.04538	-0.42139	0.673471	NA
EPHA3	10.69881	0.01912	0.084757	0.225592	0.821519	NA
SNORD70	2.223802	0.019118	0.069383	0.275539	0.782902	NA
MIR320C1	0.365208	0.019116	0.030892	0.618807	0.536043	NA
AMER1	95.72429	0.019114	0.058175	0.328563	0.742486	NA
LOC72846	1.169944	-0.01911	0.05667	-0.33721	0.73596	NA
LINC00163	0.541838	-0.0191	0.042474	-0.44977	0.652879	NA
FAM53C	146.8747	-0.0191	0.061658	-0.30981	0.756703	NA
BMS1	421.2035	0.019099	0.040537	0.471136	0.637544	0.846919
SLC11A2	179.8365	-0.0191	0.0416	-0.45904	0.646202	NA
NCCRP1	0.29383	0.01909	0.030922	0.617356	0.537	NA
IL7	19.51424	0.019075	0.08327	0.229073	0.818812	NA
DDX3X	948.5661	-0.01907	0.070868	-0.26904	0.787896	0.924656
MIR3960	1.458504	-0.01906	0.062907	-0.30297	0.761915	NA
SLC39A6	226.954	0.019043	0.049281	0.386422	0.699184	NA
MBD1	240.7979	0.019035	0.039858	0.477571	0.632956	NA
WBSCR16	31.90454	0.019034	0.075852	0.25094	0.801861	NA
USP9X	942.6519	0.019033	0.044913	0.423767	0.671736	0.862518
DNAJC2	136.0727	0.019031	0.056365	0.337634	0.735639	NA
C12orf56	2.520229	-0.01903	0.073063	-0.26043	0.79453	NA
TPM3	480.0837	-0.01903	0.047434	-0.40111	0.688338	0.870512
FBXO38	202.4934	-0.01902	0.045494	-0.41806	0.675904	NA
MAP3K14-	6.941822	0.019016	0.084875	0.224042	0.822725	NA
MON2	758.1165	-0.01901	0.038875	-0.48897	0.624865	0.841451
CCNT2	398.5826	0.019005	0.036171	0.525412	0.599296	0.829672
LSM5	63.82756	-0.01899	0.069177	-0.27453	0.783677	NA
SLC35G6	20.59824	-0.01899	0.08476	-0.224	0.822758	NA
NUDT3	66.7212	0.018981	0.06599	0.287627	0.773632	NA
PHACTR2	137.1628	0.018978	0.079069	0.240015	0.810319	NA
ARRDC1	44.34359	-0.01898	0.068594	-0.27665	0.782052	NA
ZNF204P	260.3258	-0.01897	0.045485	-0.41711	0.6766	NA
C11orf30	381.8776	0.018953	0.045277	0.418599	0.675509	0.863513
HEXIM2	12.40754	-0.01895	0.082348	-0.23011	0.818005	NA
PRKCZ	634.2479	0.018947	0.041997	0.451145	0.651885	0.853479
MIR154	0.350755	-0.01894	0.032272	-0.58685	0.557301	NA
LHPP	71.16232	0.018938	0.073662	0.257097	0.797104	NA
MIR1296	16.64643	-0.01892	0.083315	-0.22712	0.820329	NA
ANK3	7321.649	-0.01892	0.033742	-0.56081	0.574929	0.820034
NEMF	930.5752	0.018917	0.030757	0.615054	0.538519	0.799834
DDX47	93.88448	-0.01891	0.048929	-0.38657	0.699075	NA
BFSP2	0.230197	-0.01891	0.028495	-0.66372	0.506869	NA
TRIM44	809.58	0.018907	0.036146	0.523074	0.600923	0.830439
ASL	62.08149	-0.0189	0.066252	-0.28534	0.775383	NA

TMEM14E	31.16328	-0.0189	0.076935	-0.24568	0.805932	NA
GYPB	0.077648	0.0189	0.016039	1.178382	0.238644	NA
FTSJ3	134.913	-0.01889	0.068454	-0.276	0.78255	NA
SNORA70C	1.581924	-0.01889	0.056736	-0.333	0.739136	NA
WNT4	9.219306	-0.01889	0.08472	-0.22295	0.823574	NA
TMEM236	0.210742	-0.01888	0.02837	-0.66558	0.505678	NA
ACSL6	730.5172	-0.01888	0.06035	-0.31288	0.754371	0.905334
ALOX12	25.56469	-0.01888	0.079271	-0.23819	0.811737	NA
CEACAM4	0.209722	-0.01888	0.028364	-0.66561	0.505661	NA
PAGE4	0.261527	-0.01888	0.026874	-0.70247	0.482386	NA
PDZD11	29.39554	-0.01888	0.079678	-0.23692	0.812719	NA
LTBP1	11.19164	0.018876	0.084767	0.222679	0.823785	NA
DMTF1	614.6965	-0.01886	0.035806	-0.52684	0.598303	0.829672
SCARNA10	65.86418	-0.01886	0.07946	-0.23734	0.812391	NA
CYP1A2	0.117136	-0.01886	0.018092	-1.04239	0.297233	NA
CNPY1	137.4786	0.018844	0.057915	0.325372	0.744899	NA
PDCL3P4	7.636555	-0.01884	0.083	-0.22697	0.820449	NA
KDM4A	243.9097	-0.01883	0.040012	-0.4707	0.637855	NA
CD2	0.114312	0.018818	0.019245	0.977784	0.328181	NA
FAM160B1	198.1744	0.018816	0.040845	0.460679	0.645029	NA
RHOBTB2	119.0581	0.018813	0.055086	0.341517	0.732715	NA
ESM1	0.113529	-0.01881	0.018071	-1.04066	0.298034	NA
C16orf93	0.276498	0.018802	0.028142	0.668118	0.504058	NA
ZBTB37	63.94828	0.018801	0.063915	0.294151	0.768643	NA
PIH1D3	0.234963	-0.0188	0.027612	-0.6809	0.495935	NA
KISS1	0.412084	-0.01879	0.036979	-0.50809	0.611392	NA
MEGF6	15.80903	-0.01879	0.084911	-0.22124	0.824906	NA
ZNF217	12.90895	0.018773	0.084823	0.221316	0.824846	NA
MEX3D	26.32698	0.018772	0.079327	0.236641	0.812935	NA
CCDC53	41.11831	-0.01877	0.070519	-0.2662	0.790087	NA
PRDX5	188.5196	0.018771	0.073123	0.256706	0.797405	NA
CLDN3	11.95773	0.018769	0.084896	0.221086	0.825026	NA
MBLAC1	26.21	0.018763	0.079752	0.235262	0.814006	NA
SYBU	344.6798	0.018761	0.055626	0.337276	0.735909	0.895185
PROB1	6.715549	-0.01876	0.084353	-0.22238	0.824018	NA
WDR48	282.7465	0.018758	0.035158	0.53353	0.593667	NA
PAGE2B	0.155555	0.018756	0.023118	0.811341	0.41717	NA
ZNF335	276.0278	-0.01874	0.047914	-0.39114	0.695695	NA
RTN4RL2	1.609604	-0.01874	0.060122	-0.31162	0.755328	NA
HEIH	26.20469	-0.01873	0.075524	-0.24803	0.804114	NA
PRR5	4.917626	-0.01873	0.08166	-0.22932	0.818623	NA
ZNF605	429.4104	0.018724	0.053993	0.346788	0.728751	0.89271
MIR549	0.284183	-0.01872	0.031397	-0.59635	0.550944	NA
RAB11FIP5	145.0825	0.018721	0.052608	0.355863	0.721943	NA
MMP28	2.720866	-0.01872	0.072512	-0.25818	0.796271	NA
SBF2	407.9834	0.018715	0.037448	0.499776	0.617233	0.839155
STAMBP	150.2422	-0.01872	0.046331	-0.40395	0.686251	NA

ADH1A	0.105978	-0.01871	0.017869	-1.04717	0.29502	NA
SNORA36B	0.476562	0.018709	0.040883	0.457635	0.647215	NA
SNRPN	8.995949	-0.01871	0.084536	-0.22132	0.824845	NA
GIT2	780.5782	0.018708	0.037921	0.493342	0.621771	0.840783
GIMAP5	0.219048	-0.01871	0.027551	-0.67896	0.497163	NA
ZNF674-AS	16.83154	-0.0187	0.07974	-0.23458	0.814538	NA
PRR15	1.163161	-0.0187	0.03974	-0.47065	0.637891	NA
LOXL1-AS1	0.181445	0.018696	0.020175	0.926682	0.354092	NA
LMBRD1	312.9521	0.018688	0.053832	0.347143	0.728484	NA
SPATA18	1.565164	0.018687	0.063008	0.296591	0.766779	NA
FAM72D	0.890328	-0.01869	0.051785	-0.36084	0.718217	NA
KRT37	0.276757	0.018686	0.026566	0.703375	0.481822	NA
SLC5A6	42.47558	-0.01869	0.072622	-0.25729	0.796952	NA
SMAD1	76.68446	0.018679	0.060464	0.308927	0.757377	NA
CBX4	70.73282	-0.01868	0.065853	-0.28364	0.776689	NA
FAM225A	0.327392	-0.01868	0.03211	-0.58168	0.560785	NA
EIF2B5	219.9047	-0.01868	0.040197	-0.46465	0.642185	NA
SNX3	190.5808	-0.01867	0.067425	-0.27694	0.781827	NA
LOC10013	26.13846	-0.01866	0.073835	-0.25277	0.800446	NA
MLL4	465.5696	-0.01866	0.051329	-0.36353	0.71621	0.887426
EIF1B	130.1487	-0.01865	0.067137	-0.27774	0.781214	NA
RELA	127.5182	0.018614	0.067744	0.274774	0.78349	NA
COL6A4P2	6.284067	0.018605	0.063287	0.293975	0.768777	NA
MKRN7P	27.68325	-0.0186	0.081136	-0.22927	0.818663	NA
F2RL2	0.660756	-0.0186	0.043966	-0.42306	0.672249	NA
NXPH3	211.7741	0.018596	0.065427	0.284221	0.776241	NA
TPTE2P1	287.2969	0.018587	0.065767	0.28262	0.777468	NA
IRGM	0.179407	0.018586	0.021901	0.84863	0.396087	NA
POM121L2	0.433133	0.018577	0.033367	0.556732	0.57771	NA
LINC00284	0.810472	0.018576	0.047219	0.393401	0.694024	NA
PGGT1B	66.61161	0.018573	0.054354	0.341699	0.732577	NA
QRSL1	60.06949	0.018566	0.066957	0.277286	0.781561	NA
SLC13A4	45.23465	0.018565	0.078448	0.236652	0.812927	NA
FIP1L1	296.0764	0.018564	0.039001	0.475979	0.634089	NA
LINC00710	0.6357	-0.01856	0.038318	-0.48446	0.628057	NA
ACTR8	125.7715	0.018562	0.0458	0.405276	0.685275	NA
NDFIP2	132.274	0.01856	0.06522	0.284581	0.775965	NA
FLJ42875	1.9122	0.018553	0.067629	0.274339	0.783824	NA
SNORA47	18.60866	0.018544	0.083748	0.221427	0.82476	NA
CD82	19.874	-0.01854	0.084842	-0.21854	0.827008	NA
NPR3	0.66554	-0.01854	0.045178	-0.41037	0.681538	NA
GNRH2	4.938261	-0.01854	0.07813	-0.23729	0.812435	NA
MIR1-2	0.800492	-0.01854	0.048615	-0.38135	0.702946	NA
HSP90AB4	87.3638	0.018535	0.081987	0.226072	0.821146	NA
GDF15	0.375004	0.01853	0.033495	0.553224	0.58011	NA
VWA8-AS1	1.198622	0.018523	0.05853	0.31647	0.751646	NA
SDC3	348.5784	0.018521	0.072135	0.25676	0.797364	0.929506

LOC28584	2.38337	-0.01851	0.071529	-0.25884	0.795755	NA
POTEF	0.964186	-0.01851	0.037498	-0.49372	0.621506	NA
LOC64483	12.62532	-0.01851	0.084447	-0.21921	0.826485	NA
GNAI1	219.0566	0.018509	0.061002	0.303425	0.761566	NA
FOXS1	0.769454	0.018507	0.049733	0.372129	0.709797	NA
ENOPH1	105.3126	-0.01849	0.076595	-0.2414	0.809246	NA
SLC35C1	25.6173	0.01849	0.073518	0.251499	0.801428	NA
GAST	0.233417	-0.01849	0.027605	-0.66965	0.503078	NA
EIF5A	133.075	0.018484	0.057235	0.32295	0.746733	NA
CCDC87	3.471346	-0.01847	0.075358	-0.24512	0.806364	NA
MIR4715	0.154763	-0.01847	0.023077	-0.80021	0.423588	NA
SOHLH1	3.089819	-0.01846	0.071907	-0.2567	0.797407	NA
NUMB	245.1072	-0.01846	0.042118	-0.43823	0.661222	NA
ALDOB	16.4909	-0.01845	0.084939	-0.21726	0.828003	NA
MTOR	489.9679	0.018453	0.055319	0.333573	0.738702	0.895927
TBC1D25	90.00918	-0.01845	0.058056	-0.31775	0.750672	NA
MAF	189.6742	0.018444	0.071769	0.256997	0.797181	NA
CLEC9A	1.659023	0.018443	0.061104	0.30183	0.762782	NA
MFAP1	263.9078	0.018442	0.052988	0.348041	0.727809	NA
ANP32B	188.5341	-0.01844	0.060544	-0.30457	0.76069	NA
TMEM102	0.647976	-0.01844	0.045335	-0.40674	0.684196	NA
RNU6ATA	59.01487	0.018424	0.074872	0.246077	0.805622	NA
M1	10.80121	0.018417	0.08418	0.218786	0.826817	NA
STT3A	102.9431	0.018415	0.052714	0.349335	0.726837	NA
DSPP	0.797385	-0.01841	0.048941	-0.37621	0.706758	NA
TCP11	0.278231	-0.01841	0.026773	-0.68767	0.491662	NA
UBAC2	77.25882	-0.01841	0.064449	-0.28564	0.775152	NA
GRK5	49.28632	0.0184	0.073453	0.250499	0.802202	NA
CASP12	41.82973	-0.0184	0.071401	-0.25767	0.796658	NA
SOX30	0.107868	-0.01839	0.017957	-1.02427	0.305707	NA
SLC25A1	38.42829	-0.01839	0.067497	-0.2725	0.785241	NA
CLTCL1	55.725	0.018384	0.072884	0.252238	0.800857	NA
ABI1	210.7556	-0.01838	0.04417	-0.41608	0.677351	NA
POLR2K	42.02334	-0.01838	0.079934	-0.22991	0.818164	NA
HLA-DOB	1.961321	0.018375	0.052769	0.348225	0.727672	NA
PHF2	428.7001	0.018375	0.039693	0.462927	0.643416	0.848802
ARL6	29.96813	0.018369	0.073446	0.250105	0.802506	NA
USP37	270.1193	0.018368	0.036842	0.498568	0.618083	NA
SNX22	28.3018	-0.01835	0.079233	-0.23166	0.816806	NA
C12orf50	0.339317	0.018355	0.034492	0.532147	0.594624	NA
RIPK3	2.086497	0.018352	0.068313	0.268641	0.788206	NA
NXPE3	224.8732	0.018341	0.042706	0.429483	0.667572	NA
ZMYM2	642.9332	-0.01834	0.038316	-0.47862	0.632211	0.844999
HOOK3	1013.917	0.018338	0.030319	0.604836	0.545288	0.803341
ITGAM	19.10299	-0.01834	0.084411	-0.21723	0.828031	NA
MIR4671	0.591669	0.018334	0.041372	0.443156	0.657653	NA
THOC1	200.7383	-0.01833	0.048561	-0.37737	0.705895	NA

FOXR1	0.409025	0.018324	0.032685	0.56064	0.575043	NA
MAP2K2	195.4932	0.018321	0.052153	0.351299	0.725364	NA
RCBTB1	88.78133	-0.01832	0.067068	-0.2731	0.784773	NA
ZFP37	39.06699	0.018314	0.071618	0.255715	0.798171	NA
TRMT61A	41.38007	-0.01831	0.070928	-0.2582	0.796251	NA
LOC10013	6.760934	-0.01831	0.080335	-0.22794	0.819691	NA
CA11	422.3828	0.018311	0.069937	0.261824	0.793457	0.92686
PPP1R8	77.05491	-0.01831	0.064197	-0.28523	0.77547	NA
MIR933	1.068445	0.018301	0.054583	0.335286	0.737409	NA
NAT10	243.6302	-0.0183	0.04319	-0.42369	0.67179	NA
RASGRP1	550.4538	-0.0183	0.039026	-0.46887	0.639161	0.847723
CALR3	0.509918	0.018296	0.040551	0.451178	0.651862	NA
SLC35A4	64.66863	0.018289	0.0684	0.26738	0.789176	NA
C19orf38	1.669581	0.018283	0.057086	0.320265	0.748768	NA
C19orf18	5.761734	-0.01828	0.083298	-0.21944	0.826306	NA
CDC37L1	156.2305	0.018279	0.049965	0.365834	0.714489	NA
USH2A	35.22001	-0.01828	0.074601	-0.24498	0.806475	NA
MRPS17	20.1551	0.018271	0.078055	0.23408	0.814923	NA
MT1IP	0.136986	-0.01827	0.021065	-0.86731	0.385772	NA
LOC38815	0.512837	-0.01827	0.040714	-0.44871	0.653641	NA
VN1R10P	0.973844	0.018262	0.052808	0.345814	0.729483	NA
UBE3B	262.8568	0.018253	0.044292	0.412101	0.680266	NA
ANAPC5	257.9685	0.018252	0.037119	0.491707	0.622926	NA
DDN	0.70696	0.018251	0.045812	0.398386	0.690346	NA
ZNF708	223.2972	-0.01825	0.050288	-0.36292	0.716665	NA
TKT	152.7767	0.018248	0.060239	0.302921	0.76195	NA
STRIP1	215.6243	-0.01825	0.04377	-0.41686	0.676783	NA
SPACA4	0.619723	0.018241	0.047121	0.387114	0.698672	NA
PDLIM5	139.0702	-0.01824	0.068881	-0.26479	0.791172	NA
CGRRF1	47.06195	-0.01824	0.07068	-0.25802	0.796392	NA
CHPF2	75.25337	-0.01823	0.066243	-0.27526	0.783113	NA
GRID1-AS1	3.596278	-0.01823	0.075717	-0.2408	0.809711	NA
LONP2	466.9977	0.01823	0.031106	0.586074	0.557826	0.808857
RPL13AP2	0.23252	0.018229	0.026318	0.692642	0.488534	NA
ATXN2L	582.2853	-0.01822	0.03351	-0.54382	0.586563	0.825195
CHRNA5	6.679971	0.018218	0.079005	0.230592	0.817632	NA
MRPS11	69.17984	-0.01822	0.06884	-0.26462	0.791302	NA
TMEM206	28.48347	-0.01821	0.084286	-0.21604	0.82896	NA
PTGES	2.866446	0.0182	0.070242	0.259105	0.795554	NA
SIPA1L3	360.6857	-0.0182	0.066735	-0.2727	0.785083	0.924391
ZNF503-AS	1.375852	-0.0182	0.056846	-0.32009	0.7489	NA
BSCL2	31.52007	-0.01819	0.07501	-0.24253	0.808369	NA
FBXL18	117.359	0.018191	0.056741	0.320599	0.748515	NA
UBAP2	273.7068	-0.01819	0.041239	-0.441	0.659215	NA
MIR4999	0.163632	-0.01818	0.021764	-0.8355	0.403437	NA
ATP6AP1L	14.01118	0.018183	0.084866	0.21425	0.830352	NA
IKZF1	7.016369	0.018173	0.084246	0.215712	0.829212	NA

ACTG1	869.8627	-0.01816	0.070092	-0.25912	0.795539	0.928334
KCNAB1	236.8119	-0.01816	0.065784	-0.27605	0.78251	NA
DPH5	37.1466	-0.01815	0.070733	-0.25659	0.797494	NA
MS4A10	0.219498	-0.01814	0.028439	-0.63802	0.523459	NA
CCNYL1	30.41093	0.018145	0.073959	0.245336	0.806197	NA
MAP1LC3A	98.14383	0.018132	0.070637	0.256695	0.797415	NA
LINC00311	9.516706	-0.01813	0.084791	-0.21384	0.83067	NA
SCUBE1	2.192826	-0.01813	0.060384	-0.30023	0.763999	NA
C1QTNF1	18.42585	-0.01813	0.08487	-0.21359	0.830866	NA
CAMK1	73.8305	0.018127	0.056279	0.322087	0.747387	NA
SLC6A1-AS	18.85954	-0.01812	0.083284	-0.21753	0.827797	NA
ACAA1	141.0892	-0.01811	0.050106	-0.36147	0.717747	NA
NSRP1	315.8837	0.018098	0.044261	0.408883	0.682625	NA
SIGLEC14	0.743926	-0.0181	0.047286	-0.38268	0.701954	NA
CEP70	161.1563	-0.01809	0.051272	-0.35285	0.724203	NA
SUMO1	155.3167	0.018091	0.058912	0.307081	0.758782	NA
CCDC43	106.9753	0.018076	0.059982	0.301359	0.763141	NA
SCGB1C1	0.229086	-0.01807	0.028502	-0.63399	0.526084	NA
C5orf60	0.602573	0.018068	0.039589	0.456381	0.648116	NA
FABP5P3	0.667166	-0.01807	0.046513	-0.38843	0.697695	NA
SMYD5	54.52923	-0.01806	0.05846	-0.309	0.757324	NA
MYCN	3.772029	-0.01806	0.076935	-0.23476	0.814394	NA
MIR4754	0.334235	-0.01806	0.029396	-0.6143	0.539016	NA
TRPA1	0.264683	0.018056	0.027899	0.647199	0.517503	NA
S100A2	0.325637	-0.01805	0.032999	-0.54698	0.584395	NA
PSCA	0.63032	-0.01805	0.043136	-0.41835	0.675691	NA
LCN6	0.270756	0.018041	0.028155	0.64078	0.521665	NA
KIN	216.9614	-0.01804	0.054086	-0.33353	0.738734	NA
ATP6V1F	227.8147	-0.01804	0.064601	-0.27921	0.780081	NA
POF1B	0.939842	-0.01804	0.046273	-0.38977	0.696707	NA
BEND4	0.890663	-0.01803	0.047286	-0.38129	0.702987	NA
SH3BGRL3	44.43727	0.018022	0.070536	0.255503	0.798335	NA
SNORD49B	0.381956	-0.01802	0.03557	-0.50655	0.612471	NA
VAV3-AS1	0.388519	0.018015	0.034041	0.529203	0.596665	NA
SEMA3F	21.24913	-0.01801	0.084849	-0.21231	0.831865	NA
MIR499A	0.116407	0.018014	0.019406	0.928301	0.353251	NA
RFPL1	1.513291	0.018004	0.060968	0.295309	0.767758	NA
MMP13	0.116031	0.018001	0.019427	0.926589	0.35414	NA
PON3	2.161046	-0.01799	0.069645	-0.25834	0.796147	NA
USP42	168.2097	0.01799	0.045159	0.39836	0.690365	NA
SCARNA9L	2.250069	-0.01799	0.069139	-0.26018	0.794722	NA
ESR1	17.99264	-0.01799	0.083328	-0.21586	0.829094	NA
UBTD1	13.70908	0.017983	0.082899	0.216931	0.828262	NA
PHLDA2	0.822967	0.01798	0.046065	0.390325	0.696296	NA
TWIST2	0.201317	0.017977	0.025085	0.716628	0.473603	NA
PAXBP1	390.1831	-0.01797	0.044119	-0.40741	0.683705	0.867558
SNRPD1	122.7118	-0.01797	0.066633	-0.26974	0.78736	NA

RASSF10	0.216578	-0.01797	0.023685	-0.75884	0.447949	NA
C6orf223	0.989217	-0.01797	0.051171	-0.35123	0.725412	NA
WHSC1L1	1200.18	-0.01797	0.036192	-0.49654	0.619516	0.839739
RINT1	115.1025	-0.01797	0.05722	-0.31401	0.753513	NA
EPHA2	7.232584	0.017956	0.081525	0.220254	0.825673	NA
MIR4753	1.027248	-0.01795	0.054323	-0.33045	0.741062	NA
LOC34916	0.264497	0.017948	0.026601	0.67471	0.49986	NA
TPGS1	14.96576	-0.01794	0.080144	-0.22383	0.822889	NA
LARP1	593.5421	0.017935	0.040819	0.439378	0.660388	0.855957
CCP110	521.1286	0.017933	0.038386	0.467181	0.640371	0.84821
TMEM48	41.97515	-0.01793	0.06462	-0.27743	0.781454	NA
AA06	0.46272	0.017921	0.034464	0.519998	0.603065	NA
ALG14	10.39297	-0.01792	0.084818	-0.21128	0.832665	NA
LOC44160	0.130092	0.017918	0.018292	0.979536	0.327315	NA
IL17B	4.951965	0.017911	0.081764	0.219052	0.826609	NA
DOK2	0.178782	0.017905	0.02352	0.761261	0.446501	NA
PTPRJ	108.0735	-0.0179	0.076398	-0.23429	0.814756	NA
MBNL1-AS	11.8833	-0.0179	0.084192	-0.21256	0.831673	NA
LOC10050	0.298462	0.017888	0.031764	0.563153	0.573331	NA
LDLRAD3	185.1823	-0.01788	0.049688	-0.35989	0.718926	NA
LOC10028	24.5629	-0.01788	0.084059	-0.21273	0.831536	NA
SRGAP2C	8.724057	-0.01788	0.08491	-0.21053	0.83325	NA
APLP1	289.2479	0.017873	0.063862	0.279864	0.779582	NA
IPO11	87.41543	0.017871	0.051582	0.346462	0.728996	NA
SLC35B2	24.0185	0.01787	0.078916	0.226439	0.82086	NA
SPRYD4	23.09787	0.017869	0.077988	0.229127	0.81877	NA
GRHL1	20.32134	0.017867	0.084897	0.210461	0.833308	NA
USP3	459.7311	-0.01786	0.036101	-0.49469	0.62082	0.839998
CSNK1D	270.0787	0.017854	0.046973	0.380084	0.703883	NA
E2F7	0.867872	-0.01784	0.05024	-0.35518	0.722456	NA
MIR5189	0.370699	-0.01784	0.036802	-0.48484	0.627787	NA
CXCR2P1	0.239671	0.017838	0.025081	0.711203	0.476958	NA
UTP11L	84.97409	0.017822	0.059659	0.298734	0.765143	NA
LRIG3	8.179605	0.017814	0.084935	0.209738	0.833872	NA
TMEM151	41.00065	0.017813	0.069723	0.255489	0.798346	NA
TDRD12	1.132947	0.017812	0.05291	0.336653	0.736378	NA
CYSLTR2	5.002143	0.017803	0.07914	0.224961	0.822009	NA
CAGE1	0.520934	0.017797	0.041163	0.43236	0.66548	NA
C11orf80	124.6561	0.017797	0.061476	0.289494	0.772204	NA
RGSL1	0.734497	-0.01779	0.042904	-0.41475	0.678322	NA
CHIC2	90.02624	0.017786	0.061926	0.287212	0.77395	NA
LOC28495	0.751497	0.017783	0.046643	0.381258	0.703012	NA
ATIC	89.24558	-0.01778	0.057618	-0.30853	0.75768	NA
TMEFF2	51.49554	0.017767	0.064722	0.274508	0.783694	NA
FNTB	7.395464	-0.01777	0.084646	-0.20989	0.833751	NA
MPP1	97.16971	-0.01776	0.066166	-0.26848	0.78833	NA
SPATA12	0.718492	0.017762	0.041794	0.424979	0.670852	NA

CBX2	1.067959	-0.01775	0.053608	-0.33117	0.740514	NA
PAK1IP1	34.67003	-0.01774	0.078739	-0.22536	0.821695	NA
IL10	0.290379	0.017742	0.026511	0.669231	0.503348	NA
GLIS3-AS1	0.223471	0.017736	0.027263	0.650566	0.515327	NA
OSBP	276.297	0.017736	0.039634	0.447492	0.65452	NA
GRK4	109.812	0.017733	0.058457	0.303341	0.76163	NA
GCLC	72.14572	0.017732	0.060353	0.293806	0.768906	NA
CACNB1	176.2841	0.017731	0.04958	0.357633	0.720618	NA
FBXO36	17.77122	-0.01773	0.084149	-0.21071	0.833117	NA
WNT2B	19.96673	-0.01773	0.084074	-0.21087	0.832985	NA
LOC10013	0.464683	0.017716	0.03798	0.466465	0.640883	NA
TPGS2	134.5248	-0.01771	0.047289	-0.37451	0.708026	NA
KIAA0430	905.1127	0.017708	0.03274	0.54086	0.588604	0.825504
KLC4	131.4738	-0.01771	0.050823	-0.34842	0.727525	NA
LOC10018	0.63219	-0.01771	0.044013	-0.40232	0.687448	NA
FBXO11	881.1321	0.017705	0.033587	0.527125	0.598107	0.829672
CYP3A7-CY	4.01083	-0.0177	0.078639	-0.22504	0.821949	NA
RNF103-C	1.409402	-0.01769	0.05812	-0.30433	0.760878	NA
GJB7	11.20526	0.017683	0.08287	0.213377	0.831033	NA
MIR4785	0.584583	0.017677	0.044719	0.395288	0.69263	NA
TRPV6	5.126135	0.017675	0.067209	0.262987	0.792561	NA
ZFYVE28	760.6858	-0.01767	0.050349	-0.35102	0.725573	0.891227
HERC2P2	508.8907	0.017672	0.084528	0.209071	0.834393	0.944594
DNAJC28	26.79505	0.017661	0.077678	0.227355	0.820148	NA
TRMT10B	129.5135	0.017655	0.049795	0.354554	0.722924	NA
SH3YL1	52.35999	0.017646	0.060876	0.289866	0.771919	NA
SPDYE1	43.04806	0.017645	0.069881	0.252499	0.800656	NA
KLK12	0.146488	-0.01764	0.021642	-0.81523	0.414943	NA
EBF4	10.22666	0.017641	0.084914	0.207755	0.83542	NA
MIR4728	0.210463	0.017638	0.022093	0.798383	0.424648	NA
DKFZp686	0.111296	0.017635	0.019339	0.911857	0.361844	NA
FUT2	59.33577	0.017631	0.078884	0.223501	0.823146	NA
SRRM5	101.3056	0.017625	0.084939	0.207497	0.835622	NA
RPGRIP1L	95.70926	-0.01762	0.069708	-0.25283	0.800399	NA
TRIB3	9.909817	0.017624	0.084383	0.208858	0.834559	NA
FAM86EP	4.119016	0.017624	0.077148	0.228443	0.819302	NA
MRFAP1	644.2613	0.017622	0.058119	0.303215	0.761726	0.908993
NKX3-2	0.131489	0.017618	0.018216	0.967167	0.333461	NA
CRH	0.115749	-0.01762	0.018267	-0.96432	0.334888	NA
FPGT-TNN	0.780483	0.017614	0.048973	0.359678	0.719088	NA
TBL2	43.79718	-0.01761	0.067878	-0.25941	0.795321	NA
CELF2	656.0364	0.017608	0.041361	0.425716	0.670315	0.861182
DNASE1L3	0.356028	-0.0176	0.034591	-0.50887	0.610846	NA
DCK	96.68469	-0.0176	0.077383	-0.22745	0.820073	NA
HLA-C	143.5084	0.017597	0.084564	0.208094	0.835155	NA
WDR35	68.01483	0.017595	0.059713	0.294657	0.768256	NA
SOWAHC	5.12324	0.017593	0.079928	0.220108	0.825787	NA

PATZ1	78.25175	0.017587	0.058996	0.2981	0.765627	NA
OR52B4	0.111395	-0.01758	0.018146	-0.96908	0.332506	NA
CUL4B	386.6184	-0.01758	0.048582	-0.36193	0.717406	0.887792
KCNJ4	0.586893	0.017578	0.043086	0.407963	0.683301	NA
DLX4	0.160652	-0.01758	0.021593	-0.814	0.415646	NA
LOC10013	2.462714	-0.01757	0.069644	-0.25222	0.800871	NA
MTRNR2L	0.400649	-0.01757	0.036799	-0.47734	0.633123	NA
RAD54B	77.43965	-0.01757	0.062921	-0.27916	0.78012	NA
HCG26	1.546137	0.017565	0.05653	0.310713	0.756019	NA
RUNX1-IT1	0.991576	-0.01755	0.049029	-0.35797	0.720367	NA
GJB2	3.184789	0.017548	0.066028	0.265769	0.790417	NA
C9	0.131321	-0.01754	0.021047	-0.83338	0.404632	NA
FKBP10	31.67552	0.017536	0.073675	0.238018	0.811867	NA
CCM2	98.49616	0.017532	0.057457	0.305126	0.76027	NA
CCDC60	0.55995	0.017522	0.041188	0.425409	0.670539	NA
USP29	0.10821	-0.01752	0.018064	-0.96964	0.332224	NA
FRRS1	0.650196	-0.01751	0.040913	-0.42807	0.668599	NA
C12orf60	1.480688	-0.01751	0.063113	-0.27748	0.781412	NA
ITGAV	544.3022	0.017509	0.05973	0.293137	0.769417	0.912424
ZNF705G	0.109472	0.017507	0.019369	0.903872	0.366064	NA
MIR4258	0.110161	0.017504	0.019293	0.907298	0.364249	NA
LOC40075	10.39902	0.017499	0.084657	0.2067	0.836244	NA
OR2A2	0.257051	0.017496	0.029313	0.596873	0.550592	NA
AMH	23.0744	-0.01749	0.084825	-0.20624	0.836602	NA
ZNF248	634.647	-0.01749	0.039422	-0.44376	0.657219	0.854783
POLR3GL	55.42712	-0.01749	0.065062	-0.26879	0.788093	NA
DKFZP434	0.307236	-0.01749	0.026344	-0.6638	0.50682	NA
LINC00347	0.112314	0.017484	0.019338	0.904142	0.36592	NA
LOC10027	77.76411	0.01748	0.072495	0.241118	0.809464	NA
EXOC7	409.0615	0.01747	0.042271	0.413284	0.679399	0.866463
CLDN1	0.786828	0.017469	0.050174	0.348164	0.727717	NA
AP3D1	679.6434	-0.01747	0.037053	-0.47145	0.637317	0.846919
MGC3479	2.309102	0.017468	0.053561	0.326126	0.744329	NA
ZSCAN4	1.087301	0.017458	0.055241	0.316033	0.751977	NA
OR4N3P	0.550691	-0.01746	0.030373	-0.57477	0.565449	NA
C1orf233	13.12372	-0.01746	0.08484	-0.20576	0.83698	NA
PFKM	426.9138	0.01745	0.041575	0.41973	0.674683	0.863513
WNT9A	2.082481	0.017433	0.064647	0.269661	0.787421	NA
FBXO27	48.74264	0.017427	0.070945	0.24564	0.805961	NA
TMPRSS7	1.656813	-0.01741	0.052157	-0.33385	0.738493	NA
NUSAP1	10.75352	-0.01741	0.082648	-0.21067	0.833142	NA
PEMT	13.20146	-0.01741	0.08484	-0.2052	0.837413	NA
AGTR1	1.161997	-0.01741	0.055155	-0.31559	0.752311	NA
ACTG2	1.282527	-0.0174	0.046637	-0.37318	0.709014	NA
ANKRD20A	0.231722	-0.0174	0.027626	-0.6297	0.528891	NA
PIGS	62.61624	-0.01739	0.056557	-0.30747	0.758488	NA
LOC10086	48.13651	-0.01738	0.073025	-0.23801	0.81187	NA

FRMD5	88.28775	-0.01738	0.057759	-0.30092	0.763473	NA
DNM1P46	2.763588	-0.01738	0.073772	-0.23558	0.813756	NA
BASP1	341.6327	0.017377	0.071608	0.242664	0.808266	0.935326
ZNF621	201.8328	-0.01737	0.05115	-0.33965	0.734118	NA
LRRC57	67.15821	0.017366	0.067522	0.257189	0.797033	NA
TYW1	102.9357	-0.01736	0.052879	-0.32839	0.742619	NA
HTRA4	0.732018	-0.01736	0.049201	-0.35288	0.724181	NA
NIPA2	53.2254	0.01736	0.067801	0.256048	0.797914	NA
ISCU	173.7553	0.01736	0.059899	0.289824	0.771951	NA
CYB5A	107.4037	0.017357	0.066267	0.261927	0.793378	NA
SMCR7L	124.0276	0.017345	0.050909	0.340695	0.733333	NA
ZNF318	667.3659	-0.01734	0.050816	-0.34124	0.732926	0.892995
TUBGCP3	159.8461	0.017338	0.046102	0.376092	0.706848	NA
PTP4A3	9.781103	0.017336	0.082815	0.209329	0.834191	NA
PA2G4	165.0681	0.017329	0.057852	0.299539	0.764529	NA
GEMIN4	102.7299	0.017329	0.055857	0.310237	0.756381	NA
KDM6B	394.9907	-0.0173	0.063943	-0.27054	0.786748	0.924656
LINC00696	0.480957	-0.01729	0.033632	-0.51417	0.607131	NA
GAS2	6.221948	-0.01729	0.082362	-0.20995	0.833709	NA
DDX56	285.897	-0.01729	0.065956	-0.26214	0.793212	NA
RHPN1-AS	0.08971	0.017285	0.016352	1.057037	0.290495	NA
ABCE1	132.1899	0.017284	0.055036	0.314047	0.753485	NA
NUGGC	0.099802	-0.01728	0.017844	-0.96842	0.332833	NA
HTR4	0.212342	0.017278	0.027196	0.635306	0.525229	NA
VSX2	0.338406	-0.01728	0.03118	-0.5541	0.579508	NA
TOR1B	123.3232	0.017277	0.061714	0.279947	0.779518	NA
DISP1	35.14127	-0.01726	0.067188	-0.25692	0.797241	NA
MCTP2	2.069077	-0.01725	0.057498	-0.30005	0.764138	NA
RAD17	111.9845	-0.01725	0.053591	-0.32191	0.747518	NA
SLC25A19	9.257296	-0.01725	0.084858	-0.20323	0.838958	NA
RASEF	0.247465	0.017244	0.027852	0.619139	0.535825	NA
CSAG1	0.329194	0.017244	0.032048	0.538063	0.590534	NA
GANAB	165.4799	-0.01724	0.043965	-0.39204	0.695028	NA
ATP6AP2	108.5598	-0.01723	0.07899	-0.21807	0.827371	NA
MIR921	2.760717	-0.01722	0.069843	-0.24651	0.805286	NA
MIR4523	2.874352	0.017214	0.073993	0.232639	0.816042	NA
SPTA1	0.130791	-0.01721	0.019726	-0.87238	0.383003	NA
EFCAB3	0.203102	0.017205	0.027132	0.634124	0.526	NA
LOC34496	1.611496	0.017199	0.065633	0.262052	0.793281	NA
KLHDC7A	2.800261	0.017191	0.064023	0.268518	0.788301	NA
OR2L1P	2.680474	0.01719	0.052661	0.326434	0.744096	NA
CYP3A4	17.38072	-0.01719	0.083155	-0.2067	0.836245	NA
GNL1	463.44	-0.01719	0.046482	-0.36975	0.711572	0.884535
TMEM131	335.4015	-0.01718	0.036406	-0.47193	0.636978	0.846919
SNORD115	0.85547	0.017174	0.04775	0.359675	0.71909	NA
MIR4310	0.085474	0.017169	0.016298	1.053498	0.292113	NA
CATSPER2	6.700167	0.017169	0.083021	0.2068	0.836166	NA

MIR548F1	0.679034	0.017168	0.044704	0.384037	0.700951	NA
POU4F3	0.130729	0.017167	0.018397	0.933146	0.350745	NA
LOC10049	0.503259	0.017163	0.038168	0.449668	0.65295	NA
FPR2	0.51314	-0.01716	0.036688	-0.4676	0.640068	NA
PRDX4	14.81537	-0.01714	0.084917	-0.20184	0.840041	NA
PNPLA3	2.167159	0.017134	0.069545	0.24638	0.805388	NA
RPLP0P2	0.785916	-0.01713	0.048775	-0.35111	0.725503	NA
TRPC3	32.45254	0.017122	0.084905	0.201655	0.840186	NA
PDK2	529.9174	0.017119	0.062681	0.273115	0.784765	0.924391
CDC16	168.8375	0.017118	0.04472	0.382783	0.701881	NA
UBAC2-AS	0.852185	0.017116	0.052206	0.327862	0.743016	NA
PLP1	924.0236	-0.01711	0.084937	-0.20148	0.840322	0.946431
ASTN2	296.9778	-0.01711	0.07535	-0.22709	0.820357	NA
SNAP25-A	42.60374	-0.01711	0.074187	-0.23063	0.817603	NA
PYCR1	16.28727	0.017107	0.084236	0.203089	0.839066	NA
PI15	6.562186	-0.01711	0.069743	-0.24528	0.806243	NA
MIR568	22.82747	0.017105	0.078346	0.21833	0.827172	NA
PPFIA1	679.7921	0.017105	0.036129	0.473428	0.635908	0.846919
BLVRA	51.16711	-0.0171	0.060517	-0.28261	0.777479	NA
TMX2	90.48082	0.017101	0.058362	0.293023	0.769505	NA
KRT13	0.08346	0.017097	0.016351	1.045659	0.295718	NA
TNFAIP8L3	3.592738	-0.0171	0.077219	-0.22141	0.824773	NA
MIR320A	0.563547	0.017086	0.041163	0.415096	0.678072	NA
GOLGA6L9	0.36836	-0.01708	0.035564	-0.48034	0.630982	NA
DOM3Z	64.9189	0.017082	0.066161	0.258197	0.796255	NA
FASTKD1	143.341	-0.01707	0.053635	-0.31835	0.750216	NA
RHOA	297.1564	-0.01707	0.046246	-0.36917	0.712002	NA
FAM83G	4.871739	0.017067	0.079139	0.215653	0.829258	NA
CYP4Z1	2.294219	-0.01706	0.066591	-0.25613	0.79785	NA
FBXL4	97.86124	0.017051	0.057971	0.294135	0.768655	NA
FAM155A	177.1197	-0.01705	0.047714	-0.35731	0.720859	NA
TMPRSS11	1.170808	0.017048	0.055306	0.308252	0.757891	NA
MIR548W	0.086578	0.017044	0.016238	1.049628	0.293889	NA
WDSUB1	59.4521	-0.01704	0.06859	-0.24844	0.803792	NA
PSD2	327.4823	-0.01704	0.077688	-0.21935	0.826381	NA
STK38L	184.9221	0.01704	0.051487	0.330969	0.740668	NA
MVB12B	151.2597	0.017038	0.054982	0.309884	0.756649	NA
DCAF15	59.51375	-0.01704	0.058955	-0.289	0.772585	NA
RBMXL1	79.92287	0.01703	0.057227	0.297581	0.766023	NA
EIF2B2	65.3207	0.017027	0.06068	0.280604	0.779014	NA
LOC10050	5.655	0.017019	0.084075	0.202431	0.83958	NA
PSG1	18.24638	0.017019	0.083043	0.204945	0.837615	NA
ABHD16A	115.8482	-0.01702	0.054246	-0.31369	0.753757	NA
CHMP5	219.7124	0.017016	0.061062	0.278661	0.780505	NA
TMEM17	5.333803	0.017007	0.083275	0.204232	0.838172	NA
TM6SF1	54.01736	-0.017	0.066681	-0.25497	0.798746	NA
COX15	114.5994	-0.017	0.058963	-0.28829	0.773125	NA

ZNF114	4.872421	-0.017	0.079801	-0.213	0.831329	NA
PRPF38B	799.0451	0.016997	0.046826	0.362972	0.716626	0.887456
GIMAP2	3.026495	-0.01699	0.07256	-0.2342	0.814829	NA
MIR580	0.311355	0.016992	0.031782	0.534652	0.592891	NA
FAM105A	76.29044	0.016992	0.067372	0.252203	0.800884	NA
MC2R	0.0804	0.016991	0.016301	1.042372	0.297239	NA
LOC73142	0.215625	-0.01699	0.026475	-0.64163	0.521112	NA
CD79A	0.196018	-0.01698	0.024734	-0.68651	0.492392	NA
ILDR1	0.243655	-0.01698	0.028173	-0.60259	0.54678	NA
FLJ16171	0.13164	-0.01698	0.021072	-0.80559	0.420482	NA
BATF2	1.173803	0.016972	0.052169	0.325326	0.744935	NA
TMEM110	86.44017	0.016972	0.065228	0.260194	0.794714	NA
MIR4711	0.596925	-0.01697	0.041474	-0.40912	0.682454	NA
LOC44213	1.670874	0.016967	0.060633	0.279829	0.779609	NA
MYBL2	0.552976	0.016962	0.04095	0.414212	0.678719	NA
CHRFAM7	0.144688	-0.01696	0.021346	-0.79457	0.426864	NA
GPAA1	68.44414	-0.01696	0.063601	-0.26667	0.789724	NA
PRR25	0.785336	-0.01696	0.047948	-0.35365	0.723599	NA
DUOXA2	0.634982	-0.01695	0.042965	-0.39449	0.693218	NA
ZBTB40	306.6427	0.016947	0.059112	0.286694	0.774347	NA
MAGI2-AS	764.8306	-0.01695	0.041853	-0.40488	0.685569	0.868465
TMEM222	42.45759	0.016944	0.063245	0.267911	0.788768	NA
LRRC71	4.787659	-0.01694	0.082481	-0.20542	0.837241	NA
SH3BGRL2	226.6177	0.016943	0.04691	0.361186	0.717961	NA
CCM2L	53.49547	-0.01694	0.07363	-0.23003	0.818065	NA
FAIM3	0.148954	-0.01693	0.019956	-0.84842	0.396205	NA
RAB3IP	1434.278	0.016927	0.055073	0.307359	0.75857	0.908119
FAM101B	20.93841	0.016924	0.084849	0.199459	0.841904	NA
OR52A5	0.079991	0.016923	0.01618	1.045888	0.295613	NA
CHMP6	47.95674	0.016921	0.066242	0.255444	0.79838	NA
MIR1258	0.330312	-0.01692	0.033751	-0.50126	0.61619	NA
LPAR6	47.51749	0.016916	0.074327	0.227588	0.819967	NA
ITLN2	0.077075	0.01691	0.016174	1.045481	0.295801	NA
SLC10A3	8.339389	0.0169	0.08485	0.199169	0.842131	NA
BOK-AS1	1.975097	0.016894	0.065447	0.258136	0.796302	NA
MIR1236	0.320921	0.016892	0.029633	0.570055	0.568641	NA
PAGE2	0.080498	0.01689	0.016165	1.044882	0.296078	NA
OSR1	0.445194	-0.01689	0.037692	-0.44804	0.654126	NA
ZC3H11A	696.1059	0.016884	0.056682	0.297881	0.765794	0.910638
PBX3	59.03006	0.016884	0.069132	0.244226	0.807055	NA
MIR4500H	0.243517	-0.01687	0.024966	-0.67564	0.499267	NA
AXDND1	1.669089	-0.01687	0.062551	-0.26964	0.787441	NA
C5orf38	5.523904	-0.01686	0.080626	-0.20911	0.834359	NA
YJEFN3	45.13663	-0.01685	0.082557	-0.20411	0.838266	NA
NUPL2	61.16796	0.016849	0.067326	0.250262	0.802385	NA
CHAT	0.221292	-0.01684	0.027574	-0.61087	0.541284	NA
MAGEH1	70.12293	0.016841	0.070523	0.238799	0.811262	NA

C16orf74	0.59631	-0.01684	0.040462	-0.41608	0.677352	NA
MIR548I2	0.22142	-0.01683	0.027575	-0.61047	0.541554	NA
C3P1	0.160238	0.016831	0.021865	0.769758	0.441444	NA
TTC9C	48.97266	0.016831	0.066608	0.252688	0.800509	NA
GPS2	174.1437	-0.01683	0.054425	-0.30915	0.757209	NA
ADAP1	77.0811	0.016823	0.070917	0.237223	0.812484	NA
PLA2G4B	5.081812	0.016822	0.082002	0.205146	0.837458	NA
MESDC2	120.6076	-0.01682	0.048443	-0.34719	0.728452	NA
PAQR5	6.735545	-0.01682	0.084601	-0.19879	0.842424	NA
FAM183A	0.075378	0.016805	0.016126	1.042077	0.297376	NA
C15orf37	4.565796	0.016795	0.078724	0.213338	0.831063	NA
ZNF331	304.1179	0.016794	0.047651	0.352449	0.724502	NA
CCSAP	778.5501	0.016786	0.052038	0.322578	0.747015	0.900473
QSOX2	181.5844	-0.01678	0.047377	-0.35413	0.723239	NA
LOC39982	0.500708	-0.01678	0.040119	-0.41819	0.675811	NA
ZNF559	169.4663	0.016776	0.05717	0.293445	0.769182	NA
SGOL1	19.03133	0.016774	0.074465	0.225255	0.821781	NA
PYGL	21.50301	0.016773	0.082625	0.203003	0.839133	NA
INPP5F	848.8008	0.016761	0.048887	0.342847	0.731713	0.892995
ARPP19	488.1297	0.01675	0.043658	0.383669	0.701224	0.877714
SYNE2	1547.738	-0.01675	0.071645	-0.23373	0.815191	0.937263
SERINC2	1.954514	-0.01673	0.068153	-0.24552	0.806051	NA
TMEM39A	95.35888	-0.01672	0.064136	-0.26062	0.794387	NA
C2orf66	1.089215	0.016714	0.055256	0.302486	0.762282	NA
TRHR	1.033056	0.016709	0.050375	0.331695	0.740119	NA
PHYHIP	1108.521	-0.0167	0.053448	-0.31252	0.754644	0.905334
NUDT16L1	40.00131	0.0167	0.074602	0.223861	0.822865	NA
CELF1	992.8832	0.0167	0.033619	0.496753	0.619363	0.839739
LOC10013	11.38215	0.016677	0.083477	0.199775	0.841657	NA
OAZ2	182.3539	-0.01668	0.044328	-0.37618	0.706785	NA
MIR4725	0.290563	0.016662	0.029244	0.569756	0.568843	NA
CLCNKA	21.28773	-0.01666	0.074693	-0.22305	0.823495	NA
MGC4592	0.304577	-0.01666	0.030846	-0.54003	0.589177	NA
SUN3	0.076052	0.016653	0.01614	1.031803	0.302164	NA
MIR4265	0.213173	-0.01665	0.026232	-0.63475	0.525589	NA
METTL4	64.36529	-0.01664	0.068142	-0.24422	0.807058	NA
DNAAF3	0.458669	0.016637	0.033573	0.495563	0.620203	NA
PTPRA	717.1775	0.016631	0.027961	0.594813	0.551968	0.805737
BRD4	538.5505	-0.01662	0.043302	-0.38387	0.701072	0.877714
TMEM205	44.44162	-0.01661	0.062293	-0.2667	0.789697	NA
DBR1	35.28184	-0.01661	0.066665	-0.24919	0.803218	NA
TCEANC	29.7868	-0.01661	0.078608	-0.21128	0.832666	NA
MIR98	4.279837	-0.0166	0.079737	-0.20822	0.835059	NA
BBS1	185.9649	-0.0166	0.041375	-0.40123	0.688247	NA
SPINK4	0.214635	0.016599	0.025023	0.663359	0.507101	NA
CCDC97	70.65964	-0.0166	0.057862	-0.28686	0.774221	NA
LSM12	10.06965	-0.0166	0.084652	-0.19607	0.844554	NA

LOC10028	0.26134	-0.01659	0.028326	-0.58562	0.558132	NA
ZMYND19	35.03603	0.016587	0.067023	0.24748	0.804537	NA
UBE2F	28.56558	0.016583	0.073062	0.226978	0.820441	NA
HIST1H2B	0.305849	-0.01658	0.031104	-0.53315	0.593931	NA
MIR4722	0.067567	0.016582	0.016019	1.035132	0.300607	NA
IZUMO1	31.73005	-0.01657	0.07813	-0.21214	0.831997	NA
D21S2088	0.159994	0.016572	0.021851	0.758403	0.44821	NA
HOXA3	0.113988	0.016571	0.018013	0.919903	0.357623	NA
MKRN2	171.0035	0.016552	0.050982	0.324663	0.745436	NA
SETBP1	702.5464	0.016539	0.043785	0.37773	0.705631	0.882039
MEFV	1.375312	0.016537	0.052736	0.313588	0.753834	NA
CISD1	85.50121	0.016533	0.068788	0.240344	0.810064	NA
PHPT1	124.3029	0.01653	0.071848	0.230072	0.818036	NA
WDR18	41.79016	0.01653	0.067487	0.244935	0.806507	NA
HABP4	224.457	0.016526	0.055893	0.295676	0.767477	NA
ZNF428	38.09353	0.01652	0.070171	0.235424	0.813879	NA
MIR658	0.066306	0.016519	0.016007	1.031967	0.302087	NA
SMAP2	227.7278	-0.01652	0.041427	-0.39874	0.690088	NA
SNAR-F	0.341278	0.016518	0.027125	0.608959	0.542552	NA
DEPTOR	53.78796	0.016514	0.062028	0.266238	0.790056	NA
ABCB9	133.2907	-0.0165	0.058292	-0.28309	0.777104	NA
SNORD114	4.431573	-0.01649	0.075498	-0.21846	0.827073	NA
ZC2HC1A	231.7534	0.016492	0.057849	0.285085	0.775579	NA
MYOZ1	0.909881	-0.01649	0.047757	-0.34532	0.729852	NA
LINC00602	1.25392	-0.01649	0.057014	-0.28923	0.772403	NA
LOC25565	0.247061	-0.01648	0.02652	-0.62156	0.534231	NA
EXOSC2	122.7158	-0.01648	0.050018	-0.3295	0.741781	NA
FLAD1	44.50434	0.016479	0.065432	0.251841	0.801164	NA
HIST1H4D	34.54549	-0.01648	0.084881	-0.19413	0.846075	NA
C1orf168	16.04072	-0.01647	0.084723	-0.19444	0.84583	NA
UGT3A2	0.112775	0.016458	0.018003	0.914146	0.36064	NA
SLPI	0.325029	-0.01646	0.024375	-0.67513	0.499595	NA
CCDC169	0.072231	0.016436	0.016163	1.016924	0.309189	NA
UAP1	87.80589	0.016434	0.05461	0.300942	0.763459	NA
NDST2	151.7559	-0.01643	0.05143	-0.31946	0.749376	NA
PCDH1	383.1899	-0.01643	0.053674	-0.30606	0.759558	0.90815
RPUSD4	51.84913	-0.01643	0.062256	-0.26387	0.791881	NA
SLC39A2	0.591222	-0.01642	0.042819	-0.38358	0.701292	NA
SNORA49	149.4667	-0.0164	0.081982	-0.20007	0.841427	NA
SGMS1	146.4715	-0.0164	0.050229	-0.32653	0.744026	NA
GUCY1B3	162.1486	0.016394	0.0701	0.23386	0.815094	NA
SPCS3	226.3512	0.016389	0.043585	0.376015	0.706906	NA
LOC10050	0.864454	0.016381	0.048111	0.34048	0.733495	NA
DRD4	28.00018	0.01638	0.0849	0.192936	0.847009	NA
LOC10050	9.982419	0.01638	0.084937	0.192852	0.847075	NA
DHCR7	22.85023	0.016375	0.081369	0.201247	0.840505	NA
MIR374A	0.450382	0.016374	0.038372	0.426722	0.669582	NA

LAMP1	215.6753	0.016374	0.050469	0.324431	0.745612	NA
SCN3B	148.3865	0.016364	0.068102	0.24029	0.810106	NA
TRIP4	82.85462	-0.01636	0.057882	-0.28264	0.777451	NA
UNC50	70.06243	-0.01636	0.058904	-0.27772	0.781226	NA
KRT42P	0.221341	0.016357	0.023598	0.693169	0.488204	NA
UHRF1BP1	455.8298	-0.01636	0.051256	-0.31909	0.749655	0.902144
MX2	4.735528	-0.01634	0.074771	-0.21858	0.826975	NA
ZSCAN18	311.8384	-0.01634	0.056431	-0.2896	0.772122	NA
HP1BP3	586.0042	-0.01633	0.04083	-0.39988	0.689243	0.870767
MIR4264	0.136072	-0.01633	0.021214	-0.76958	0.441552	NA
ZFP42	0.30582	0.016325	0.031799	0.513372	0.607691	NA
43169	0.550736	-0.01632	0.043586	-0.37454	0.708006	NA
MMP23A	0.418355	0.016323	0.036544	0.446658	0.655122	NA
LOC28618	0.135381	-0.01632	0.021181	-0.77047	0.44102	NA
TNFRSF10	17.89188	0.016309	0.084854	0.192202	0.847584	NA
LOC72860	0.755295	-0.0163	0.045526	-0.35808	0.720281	NA
CLDN25	0.06422	0.016302	0.015982	1.01998	0.307738	NA
HILS1	3.952838	-0.0163	0.074425	-0.21903	0.826627	NA
FLJ42969	0.568982	-0.0163	0.041568	-0.39215	0.694951	NA
TLX3	21.37815	-0.0163	0.08386	-0.19438	0.845881	NA
IQCG	39.98802	-0.0163	0.067028	-0.24318	0.807865	NA
MIR300	0.784976	0.016298	0.045767	0.356104	0.721763	NA
KIAA0040	12.24943	-0.01629	0.075757	-0.21503	0.829747	NA
HES7	6.543985	-0.01629	0.077475	-0.21023	0.833486	NA
DNAJB8	0.827513	-0.01627	0.045302	-0.35924	0.719418	NA
GREM2	9.174016	-0.01627	0.082214	-0.19793	0.8431	NA
GPR55	0.163067	0.016272	0.022851	0.712103	0.476401	NA
TSG101	74.74582	-0.01626	0.055994	-0.29045	0.77147	NA
IFLTD1	5.766221	-0.01626	0.080311	-0.20246	0.839553	NA
HYAL4	0.118005	0.016249	0.014426	1.126333	0.260025	NA
AP1S2	77.76963	-0.01625	0.067295	-0.24144	0.809216	NA
HID1	441.1615	0.016245	0.067839	0.239465	0.810745	0.935952
LINC00672	33.69134	0.016235	0.082378	0.197079	0.843766	NA
TESPA1	0.254029	0.016231	0.028895	0.561729	0.574301	NA
RAE1	46.91858	-0.01623	0.064188	-0.25284	0.800394	NA
LRP5L	7.075462	-0.01623	0.079591	-0.20388	0.838445	NA
OR1L8	0.249344	0.016225	0.028882	0.561787	0.574261	NA
FAM104A	133.5852	0.016218	0.050359	0.322053	0.747413	NA
CCL8	0.160991	0.016215	0.016748	0.968185	0.332952	NA
GPR149	0.268637	-0.01621	0.028219	-0.57459	0.565566	NA
CRSP8P	0.470984	0.016211	0.038727	0.418587	0.675518	NA
CCDC171	198.1718	0.016206	0.066462	0.243836	0.807358	NA
LENG1	46.81309	0.016204	0.068777	0.235608	0.813737	NA
TMEM177	17.21844	-0.0162	0.082694	-0.19593	0.844666	NA
PICALM	485.8739	0.016186	0.037058	0.436773	0.662276	0.855957
DONSON	75.49559	-0.01619	0.078625	-0.20586	0.836901	NA
KPNB1	589.1207	-0.01618	0.043578	-0.37139	0.710346	0.884085

BRD7	308.9182	0.016185	0.032617	0.496205	0.61975	NA
UBAC1	134.3964	0.016184	0.04775	0.33894	0.734655	NA
GPR25	0.194676	-0.01618	0.024701	-0.6552	0.51234	NA
WISP1	1.927416	0.016173	0.064692	0.25	0.802587	NA
FKBP14	106.4514	-0.01617	0.060758	-0.26618	0.790101	NA
XRRA1	163.5543	0.016164	0.081601	0.198082	0.842981	NA
ZNF511	18.15488	-0.01616	0.082469	-0.19596	0.844638	NA
TBL1Y	0.595971	0.016161	0.032259	0.500981	0.616385	NA
NLK	104.6745	0.016156	0.057872	0.279167	0.780116	NA
C19orf12	101.6101	0.016156	0.048812	0.330975	0.740663	NA
PCBP3	148.4621	0.016154	0.062249	0.25951	0.795241	NA
ZFAND1	169.8132	-0.01615	0.058826	-0.2746	0.78362	NA
PLA2G2C	0.436126	-0.01615	0.037504	-0.43072	0.66667	NA
TUBA8	196.1282	0.016154	0.063811	0.253149	0.800153	NA
RCOR2	15.95782	-0.01615	0.083943	-0.19241	0.847424	NA
S100A10	10.30931	0.016147	0.082622	0.195428	0.845058	NA
PLA2G1B	0.316296	0.016134	0.029349	0.549749	0.582492	NA
SELV	0.658511	0.016127	0.04282	0.376619	0.706457	NA
RFXAP	103.794	-0.01613	0.063732	-0.25304	0.800239	NA
MIR4306	0.283719	-0.01612	0.030543	-0.52768	0.597725	NA
LOC10050	1.951162	0.016115	0.063526	0.253673	0.799748	NA
COA1	139.9835	0.016114	0.048036	0.335443	0.737291	NA
CWF19L2	372.3699	0.016112	0.041552	0.387743	0.698206	0.875657
PTCD2	26.20679	0.01611	0.079955	0.201483	0.840321	NA
PCDHB18	48.02416	0.016099	0.068825	0.233912	0.815053	NA
ZNF451	885.575	-0.0161	0.044062	-0.36532	0.71487	0.88625
TRAK1	640.378	0.016083	0.030401	0.529044	0.596775	0.829672
SLC7A11	155.2949	-0.01608	0.083064	-0.19362	0.846473	NA
KRBOX4	48.09562	-0.01608	0.064711	-0.24847	0.803772	NA
PORCN	43.33629	0.016074	0.066681	0.241063	0.809506	NA
PSG8	0.812907	-0.01607	0.03904	-0.41153	0.680681	NA
BLMH	67.22934	0.016064	0.059008	0.272228	0.785447	NA
NPPC	3.183199	0.016062	0.07025	0.228636	0.819152	NA
MIR4442	5.360504	0.016057	0.083005	0.193444	0.846612	NA
SRSF10	368.1479	-0.01605	0.038243	-0.41981	0.674622	0.863513
PPP1R15B	302.4619	0.016051	0.059039	0.271874	0.785719	NA
MIR4787	34.73832	0.016045	0.075317	0.213028	0.831305	NA
TNFSF18	0.592334	0.016043	0.039783	0.403252	0.686763	NA
APOBEC3C	2.184032	-0.01604	0.068649	-0.23367	0.815237	NA
MTERFD1	49.84811	0.016035	0.062764	0.255483	0.79835	NA
MRC1	1.001369	0.016031	0.042371	0.378361	0.705162	NA
LBP	0.136705	-0.01603	0.021234	-0.75487	0.450329	NA
ART4	0.8637	0.016028	0.04729	0.338931	0.734662	NA
PLG	0.24944	0.016025	0.028877	0.554943	0.578934	NA
ZBTB48	46.95441	-0.01602	0.067749	-0.23643	0.813096	NA
IQCE	190.1904	-0.01601	0.058827	-0.27207	0.785569	NA
NUBPL	24.75749	-0.01599	0.07733	-0.20684	0.836135	NA

PISD	523.8297	0.015993	0.041636	0.384108	0.700898	0.877714
PI4KB	280.797	0.01599	0.034011	0.470135	0.638258	NA
PEX11B	48.67159	-0.01599	0.065736	-0.24322	0.807834	NA
TMEM43	171.3545	0.015983	0.056272	0.284023	0.776392	NA
MIR3125	1.258555	-0.01598	0.058094	-0.2751	0.783239	NA
NADK	80.60281	0.015973	0.062241	0.256636	0.79746	NA
SHPRH	835.0251	-0.01597	0.06754	-0.23641	0.813115	0.936217
BLVRB	9.588329	-0.01596	0.084827	-0.18816	0.850747	NA
STAB2	6.806409	0.015953	0.078518	0.203177	0.838996	NA
SLC22A2	0.18312	-0.01595	0.024653	-0.64703	0.517615	NA
KCNH5	0.248995	0.015942	0.028875	0.552113	0.580871	NA
EMBP1	22.4035	-0.01594	0.079764	-0.19986	0.84159	NA
IL17RC	42.49633	-0.01593	0.076478	-0.20831	0.834989	NA
OTP	0.090084	-0.01592	0.014297	-1.11354	0.265477	NA
STAP1	0.281179	0.015884	0.031549	0.503469	0.614634	NA
ANKRD20A	4.414512	-0.01588	0.066976	-0.23714	0.812545	NA
GPR3	5.220448	0.015867	0.077124	0.205737	0.836996	NA
PIAS4	111.6315	-0.01587	0.053415	-0.29703	0.766443	NA
SLC35D1	90.75616	0.015865	0.052865	0.300101	0.7641	NA
QDPR	262.4592	-0.01586	0.071799	-0.22089	0.82518	NA
SMCHD1	763.7349	0.015858	0.04034	0.393104	0.694242	0.871418
NDUFB2	24.16559	-0.01586	0.083524	-0.18984	0.849434	NA
UBE2H	566.2121	-0.01585	0.042316	-0.3746	0.707958	0.883299
GYG2P1	0.550096	0.015848	0.02604	0.608609	0.542784	NA
FAM201A	5.604574	0.015846	0.062734	0.252586	0.800588	NA
H3F3A	0.167909	0.015844	0.021808	0.72653	0.467514	NA
GTF2A2	45.08527	0.015838	0.070718	0.223966	0.822784	NA
PSMB9	5.326	-0.01583	0.077433	-0.20443	0.838015	NA
CTSD	170.3752	-0.01583	0.063476	-0.24934	0.8031	NA
COPA	433.4184	-0.01583	0.044681	-0.35419	0.723198	0.890244
RWDD3	25.87676	-0.01582	0.074922	-0.21115	0.832767	NA
MIR210	0.300949	0.015818	0.029642	0.533635	0.593594	NA
CNPPD1	50.96019	-0.01582	0.071844	-0.22014	0.82576	NA
WDR45B	201.9968	0.015815	0.043218	0.365933	0.714415	NA
KRT40	0.476855	-0.01581	0.030132	-0.52483	0.599703	NA
ZNF865	127.9692	0.015805	0.052139	0.303124	0.761795	NA
CDRT4	9.99879	-0.0158	0.08477	-0.1864	0.852128	NA
SOWAHB	2.926797	-0.01579	0.071099	-0.22203	0.824293	NA
DAB1	219.3841	-0.01579	0.049139	-0.32124	0.74803	NA
LINC00313	0.359437	0.015783	0.03219	0.490303	0.62392	NA
C16orf78	0.179418	-0.01578	0.021793	-0.72421	0.468937	NA
MIR500B	0.122982	0.015776	0.018095	0.871838	0.383297	NA
ZNF433	41.79576	-0.01577	0.064064	-0.24623	0.805502	NA
RWDD4	28.66998	-0.01577	0.068547	-0.23007	0.818039	NA
LOC28635	0.14879	0.015769	0.02269	0.694955	0.487083	NA
AUP1	199.9522	0.015761	0.073188	0.215353	0.829492	NA
SNORD127	5.81544	0.015757	0.083039	0.189753	0.849503	NA

MAPK8IP2	571.3499	0.015753	0.049493	0.318298	0.750259	0.902144
C13orf45	0.186958	-0.01575	0.025715	-0.61246	0.540236	NA
C2orf70	1.024039	-0.01574	0.052044	-0.3025	0.762273	NA
SLAMF6	0.495569	0.015741	0.037284	0.42218	0.672894	NA
LOC10028	3.063999	-0.01574	0.076852	-0.20481	0.837718	NA
ATAD2B	368.4971	-0.01574	0.04532	-0.34727	0.728385	0.89271
CHD8	858.4674	0.015734	0.031934	0.492701	0.622224	0.840893
RGS11	646.62	-0.01573	0.063002	-0.24974	0.802792	0.931519
RGPD2	1.399529	0.015717	0.044486	0.353306	0.723859	NA
SPTLC1	138.4428	-0.01571	0.042312	-0.37122	0.710477	NA
C10orf68	98.15339	-0.01571	0.070571	-0.22256	0.823875	NA
GRIK5	212.5302	-0.0157	0.04375	-0.35887	0.719689	NA
MIR1251	0.347548	-0.0157	0.03036	-0.51709	0.605095	NA
IFITM4P	0.386945	-0.0157	0.035295	-0.44478	0.656477	NA
KRT35	0.226465	0.015698	0.027813	0.564406	0.572478	NA
PNCK	18.65803	0.015693	0.084671	0.18534	0.852962	NA
LOC10028	1.851635	0.015689	0.065852	0.238244	0.811692	NA
ZNF629	197.6422	-0.01569	0.051836	-0.30265	0.762155	NA
NEK4	212.2572	0.015678	0.043583	0.359729	0.71905	NA
FTSJD1	31.47486	-0.01567	0.070637	-0.2218	0.824473	NA
LOC72798	0.094376	-0.01566	0.016465	-0.95117	0.341519	NA
FABP2	0.588404	0.015641	0.042214	0.370515	0.710999	NA
HMGB1	356.6956	-0.01564	0.061381	-0.25475	0.798913	0.930701
SERHL	0.440854	-0.01564	0.036934	-0.42338	0.672021	NA
RASA4	92.01855	0.015636	0.084177	0.185748	0.852643	NA
SLFNL1	6.88955	0.015634	0.083519	0.187193	0.851509	NA
ODF2	437.2801	-0.01562	0.051288	-0.30459	0.760678	0.908243
NRG4	67.55486	-0.01562	0.084224	-0.18547	0.85286	NA
PHOSPHO	17.75101	0.015618	0.082334	0.189696	0.849547	NA
NAALAD2	46.21758	0.015608	0.084765	0.184131	0.853911	NA
LOC28554	7.741865	-0.01561	0.078948	-0.19767	0.843303	NA
GGA1	223.4859	0.015603	0.046171	0.337944	0.735405	NA
TAX1BP3	4.145551	0.015602	0.080893	0.192872	0.847059	NA
EYA4	1.955857	-0.01559	0.066245	-0.23535	0.813936	NA
POMC	4.117972	0.015589	0.072041	0.216385	0.828688	NA
HLA-DQA1	2.18447	-0.01558	0.068209	-0.22848	0.81927	NA
PAK4	60.52077	-0.01558	0.067743	-0.22999	0.818097	NA
AAED1	7.790124	-0.01558	0.084325	-0.18471	0.853455	NA
GPRC5D	0.464441	-0.01557	0.037257	-0.41803	0.675927	NA
CNBD2	1.220321	0.015569	0.058927	0.264217	0.791613	NA
OR2D2	0.462419	-0.01556	0.034373	-0.45279	0.650699	NA
FAM131A	96.56717	0.015547	0.06705	0.231875	0.816635	NA
PNMA2	309.4036	0.015543	0.083818	0.185441	0.852883	NA
KRT24	0.683059	-0.01552	0.046287	-0.33532	0.737381	NA
FBLL1	14.7678	-0.01552	0.083347	-0.18621	0.852281	NA
HIST1H2A	0.196232	-0.01552	0.021996	-0.70537	0.48058	NA
CLASP2	2043.935	0.015511	0.028448	0.545245	0.585585	0.824332

GALNT9	411.8282	-0.01551	0.061619	-0.25171	0.801266	0.930701
GTPBP10	160.7719	0.015508	0.048768	0.317985	0.750496	NA
SERTAD1	4.646655	-0.0155	0.077901	-0.19894	0.842312	NA
SRPX	35.41002	-0.0155	0.084081	-0.1843	0.853776	NA
SETD4	97.29402	-0.0155	0.053204	-0.29125	0.770859	NA
LIPI	3.164641	0.015494	0.072952	0.212391	0.831802	NA
CDK10	89.2266	-0.01549	0.069532	-0.22274	0.823738	NA
LOC28548	4.416095	0.015486	0.079432	0.194954	0.845429	NA
CSTB	46.29714	0.015476	0.0704	0.219834	0.826	NA
CCDC62	35.86767	0.015469	0.081107	0.190718	0.848747	NA
GNG10	1.715789	-0.01547	0.066753	-0.23168	0.816785	NA
LOC15221	33.61903	0.015464	0.071203	0.217177	0.828071	NA
SPACA5	0.335024	0.015461	0.034708	0.445461	0.655987	NA
NUP188	442.1392	-0.01546	0.054424	-0.28399	0.77642	0.91728
SFPQ	2018.756	0.015454	0.054183	0.285211	0.775483	0.916737
ADAM32	17.07952	0.015452	0.084876	0.182047	0.855545	NA
LINC00535	2.207559	-0.01545	0.07169	-0.21552	0.82936	NA
PRODH2	0.304313	-0.01545	0.030533	-0.50586	0.612954	NA
DLL1	12.85428	-0.01544	0.084937	-0.1818	0.855738	NA
TMOD1	253.5936	0.01544	0.065024	0.237455	0.812304	NA
SSPO	184.6964	0.015435	0.080621	0.191449	0.848174	NA
DQX1	3.926428	0.015433	0.076562	0.20158	0.840245	NA
LOC64524	0.29386	-0.01543	0.029191	-0.52861	0.597077	NA
IGF2R	314.2592	-0.01543	0.04012	-0.38452	0.700593	NA
CCDC13-A	0.506227	-0.01542	0.039949	-0.38611	0.699412	NA
IPCEF1	302.6499	0.015425	0.076986	0.200356	0.841202	NA
GRTP1	10.69779	-0.01542	0.084842	-0.18178	0.855752	NA
SEC63	367.6248	0.015422	0.046436	0.332108	0.739808	0.896078
MAP3K10	208.3325	0.015422	0.051218	0.301103	0.763336	NA
KCNK4	6.585949	0.015421	0.08379	0.184045	0.853978	NA
HIST1H3A	8.600296	-0.01542	0.084933	-0.18154	0.855945	NA
LRRC37A5	0.144411	0.015418	0.021741	0.709179	0.478213	NA
TMEM87A	109.5388	0.015417	0.046501	0.331548	0.740231	NA
EVPLL	0.086543	-0.01542	0.016337	-0.94363	0.345357	NA
RAC1	288.6532	0.015416	0.05278	0.292075	0.770229	NA
SLC27A5	20.33114	0.015414	0.077958	0.197727	0.843259	NA
FLII	352.201	0.01541	0.061105	0.252193	0.800892	0.930701
C3orf72	1.035672	0.015405	0.05497	0.280247	0.779288	NA
PRPF19	218.5977	0.015396	0.047608	0.323399	0.746393	NA
NEU3	53.21837	0.015392	0.062085	0.247919	0.804197	NA
C17orf53	11.23993	0.015391	0.084015	0.18319	0.854649	NA
TOMM5	73.54145	0.015388	0.06895	0.223173	0.823401	NA
PRR16	50.00536	-0.01538	0.075632	-0.20336	0.838851	NA
MYH3	55.6805	-0.01537	0.079251	-0.194	0.846176	NA
ACTL6B	123.6421	0.015371	0.047729	0.322036	0.747426	NA
MIR5001	0.209526	0.015369	0.023583	0.651713	0.514586	NA
AURKA	11.3748	0.015362	0.084524	0.181753	0.855777	NA

SCP2D1	0.210848	0.015361	0.025039	0.613486	0.539555	NA
PAPPA	68.28749	0.015359	0.080482	0.19084	0.848651	NA
CCNI2	33.02359	0.015349	0.069809	0.219872	0.825971	NA
CCBP2	1.482385	-0.01533	0.059895	-0.25601	0.79794	NA
LOC10050	0.08209	-0.01533	0.01629	-0.94089	0.34676	NA
KRT83	0.154808	0.015321	0.021511	0.712255	0.476307	NA
KPTN	35.70852	0.015318	0.077556	0.19751	0.843428	NA
DCAF12L1	0.083333	-0.01531	0.016282	-0.94043	0.346997	NA
USE1	39.75673	-0.01531	0.072301	-0.21173	0.832318	NA
GHRHR	0.077199	-0.01531	0.016279	-0.94024	0.347094	NA
VCP	397.2862	0.0153	0.036236	0.422237	0.672852	0.862973
CSTL1	0.133958	-0.0153	0.021248	-0.72006	0.471489	NA
CDK12	642.3231	0.0153	0.045659	0.335082	0.737563	0.895927
MIR376A2	0.286526	-0.01529	0.028235	-0.54158	0.588109	NA
DNAL1	164.1495	-0.01529	0.046604	-0.32809	0.74284	NA
ANKRD20A	9.592056	0.015287	0.052149	0.293142	0.769414	NA
SH3PXD2A	1227.236	0.015279	0.054574	0.279962	0.779506	0.919574
GGT6	0.211361	0.015275	0.023438	0.651708	0.51459	NA
LOC10013	398.7884	0.015273	0.075005	0.203624	0.838647	0.946431
MGMT	24.63775	0.015265	0.080226	0.19028	0.84909	NA
SEC61A2	137.8782	0.015265	0.048552	0.3144	0.753217	NA
LINC00575	0.373512	0.015259	0.033475	0.455839	0.648506	NA
TFPT	52.9716	-0.01525	0.061939	-0.24623	0.805507	NA
POLR3H	203.2038	0.015244	0.050691	0.300724	0.763625	NA
ZCCHC6	266.6074	-0.01524	0.043644	-0.34923	0.726919	NA
MIR3138	0.363527	0.015241	0.035306	0.43168	0.665974	NA
C7orf55	2.923366	-0.01524	0.074277	-0.20516	0.837445	NA
DLL3	1.803655	-0.01524	0.065042	-0.23428	0.814767	NA
LOC10050	4.578424	0.015235	0.079587	0.191426	0.848192	NA
RALGAPA2	409.6338	0.015223	0.044573	0.34153	0.732705	0.892995
GDPD3	41.56222	-0.01522	0.084029	-0.18114	0.856258	NA
MIRLET7G	1.01278	-0.01522	0.053806	-0.28286	0.777281	NA
NDUFA8	48.8283	-0.01522	0.068316	-0.22278	0.823704	NA
IFNAR1	269.4766	0.015216	0.044842	0.339313	0.734374	NA
ARG2	64.63931	-0.01521	0.063394	-0.24	0.81033	NA
ASB6	128.4127	0.015208	0.05596	0.271758	0.785808	NA
STK39	450.2826	0.015198	0.045847	0.331506	0.740262	0.896149
SNORD114	3.991492	0.015182	0.076394	0.198729	0.842475	NA
SNORD93	0.123736	0.01518	0.018141	0.836776	0.402718	NA
LRRC43	5.382183	0.01518	0.083024	0.182835	0.854928	NA
LOC10049	1.981443	-0.01517	0.065763	-0.23074	0.817518	NA
SNAR-D	0.441717	0.015173	0.038586	0.39322	0.694157	NA
GSTT1	50.10157	-0.01516	0.056608	-0.26775	0.78889	NA
OPA3	54.55955	0.015156	0.067652	0.224034	0.82273	NA
ZDHHC16	114.7867	-0.01515	0.053552	-0.28282	0.777312	NA
SOX7	9.03436	-0.01514	0.083105	-0.18223	0.855399	NA
KIAA1841	168.1736	0.015143	0.050544	0.299597	0.764485	NA

C5orf52	0.085597	-0.01514	0.014184	-1.06728	0.285847	NA
ACAA2	30.49753	-0.01514	0.078574	-0.19262	0.847255	NA
EPHX3	0.182074	-0.01513	0.025676	-0.58943	0.555574	NA
SH3GLB1	258.6298	0.015134	0.03689	0.410244	0.681627	NA
RBBP4	403.3212	0.015132	0.031801	0.475849	0.634182	0.845687
PLAGL1	24.76599	0.015125	0.084434	0.179136	0.857831	NA
C3orf30	0.684421	-0.01512	0.047804	-0.31635	0.751739	NA
GPATCH3	59.98412	0.01512	0.061249	0.246856	0.80502	NA
PFKL	257.985	-0.01512	0.046625	-0.32427	0.745734	NA
TMEM105	0.079413	-0.01512	0.016179	-0.93443	0.350082	NA
MCL1	268.1967	-0.01512	0.046077	-0.3281	0.742839	NA
RNF183	0.526547	-0.01512	0.03869	-0.3907	0.696022	NA
GATAD2B	564.5325	-0.01511	0.035346	-0.42758	0.668959	0.861137
KRT71	0.079183	-0.01511	0.016176	-0.93423	0.350183	NA
EXTL2	78.43284	0.015109	0.067488	0.223872	0.822857	NA
OR13C5	0.082421	-0.0151	0.01617	-0.93391	0.350349	NA
GAS7	2390.842	0.0151	0.056124	0.269042	0.787897	0.924656
EMC10	63.74821	-0.0151	0.05982	-0.25236	0.800762	NA
TMPRSS13	0.123205	0.015093	0.018224	0.82819	0.407563	NA
LOC28587	0.340602	0.015089	0.031626	0.477114	0.633281	NA
CCNJL	267.8125	-0.01508	0.062415	-0.24167	0.80904	NA
APEH	93.59862	-0.01508	0.055277	-0.27275	0.785045	NA
MIR4688	0.253142	-0.01507	0.028877	-0.52202	0.601655	NA
CACNA1G-	3.037014	0.015066	0.075679	0.199079	0.842201	NA
KHNYN	204.4428	-0.01507	0.047746	-0.31554	0.752349	NA
RNF39	15.58019	-0.01506	0.084836	-0.17754	0.85908	NA
LYL1	4.520388	-0.01506	0.07932	-0.18987	0.849415	NA
MPZL1	106.2823	-0.01506	0.051622	-0.29173	0.770493	NA
SNORA29	3.742818	0.015056	0.074372	0.202438	0.839575	NA
HIST2H2BF	23.99484	-0.01505	0.08414	-0.17887	0.858043	NA
MIR27B	3.003057	-0.01505	0.074439	-0.20214	0.839808	NA
SSBP4	58.02773	-0.01504	0.069358	-0.21691	0.828275	NA
SS18L1	357.0038	-0.01504	0.043767	-0.34373	0.731051	0.892995
RANBP3	229.1898	-0.01504	0.048095	-0.31272	0.754495	NA
KIAA0368	827.3919	0.015039	0.029158	0.515767	0.606017	0.831231
KLRB1	0.9827	-0.01504	0.054033	-0.27829	0.780786	NA
MIR1185-2	0.616062	-0.01504	0.045965	-0.32712	0.74358	NA
OR5K1	0.076242	-0.01503	0.016369	-0.91852	0.358347	NA
GLT1D1	63.68737	0.015034	0.066041	0.227653	0.819916	NA
BCKDK	101.1184	0.015033	0.065942	0.227975	0.819666	NA
DPT	1.223525	-0.01503	0.05122	-0.29342	0.769201	NA
LOC10013	0.276365	-0.01503	0.027974	-0.53716	0.591157	NA
LOC10050	42.86065	-0.01503	0.073966	-0.20314	0.839026	NA
LRRFIP1	245.3572	-0.01502	0.054538	-0.27543	0.782987	NA
CORT	6.856078	0.015017	0.084693	0.177308	0.859266	NA
CETN3	40.85536	-0.015	0.074111	-0.20242	0.839585	NA
AURKC	11.07165	0.015	0.084198	0.178157	0.858599	NA

ATAD3C	3.676504	-0.015	0.074199	-0.20215	0.839797	NA
TIMP2	108.4446	-0.015	0.071593	-0.20948	0.834076	NA
BET1L	59.12702	0.014996	0.06328	0.236974	0.812677	NA
ASCC3	364.3149	0.014993	0.036149	0.414764	0.678315	0.865568
CACTIN-AS	1.32972	0.01499	0.057685	0.259854	0.794976	NA
C22orf39	72.49046	0.014989	0.058063	0.258148	0.796293	NA
MCM3AP-	37.46569	0.014989	0.074361	0.201565	0.840257	NA
LRP6	450.7655	0.014988	0.041832	0.358298	0.72012	0.889838
POU6F2-A	0.075125	-0.01498	0.016108	-0.93027	0.352234	NA
GATA3	0.077696	-0.01498	0.016106	-0.93016	0.35229	NA
IFNA6	0.077683	-0.01498	0.016106	-0.93013	0.352306	NA
MIR4420	0.079787	-0.01497	0.016102	-0.92991	0.35242	NA
SLU7	287.604	0.014972	0.057685	0.259547	0.795214	NA
IGSF23	0.564194	0.014969	0.039761	0.376465	0.706571	NA
IQCF2	0.077942	-0.01496	0.016096	-0.92955	0.352602	NA
ZNF701	73.51108	-0.01496	0.067989	-0.22005	0.825834	NA
C1orf177	2.683389	0.014961	0.073473	0.203618	0.838652	NA
MIR744	0.552876	-0.01496	0.04212	-0.35507	0.722536	NA
GK2	0.161584	-0.01494	0.021775	-0.68627	0.492546	NA
HM13-AS1	7.858329	-0.01494	0.0825	-0.18113	0.856267	NA
CNGA3	0.511578	0.014941	0.038074	0.392423	0.694746	NA
SPRTN	58.69657	-0.01494	0.068978	-0.21659	0.828527	NA
RPAP1	57.69344	0.014937	0.064884	0.230218	0.817923	NA
PRRG4	1.883016	0.014931	0.06161	0.242344	0.808514	NA
TERF2	151.575	0.014929	0.044876	0.332663	0.739388	NA
CSTA	0.162722	-0.01492	0.021697	-0.68752	0.491757	NA
SPHK2	97.81438	-0.01492	0.060136	-0.24803	0.804115	NA
MIR656	0.320064	-0.01491	0.03406	-0.43788	0.661476	NA
GYPA	0.264008	0.014912	0.025172	0.592411	0.553576	NA
PRICKLE4	26.18141	0.01491	0.079868	0.186677	0.851914	NA
PIK3C2B	146.6299	-0.0149	0.056121	-0.26545	0.79066	NA
CPLX4	3.987131	0.014892	0.064135	0.232194	0.816387	NA
JOSD2	12.68781	-0.01489	0.084309	-0.17661	0.859816	NA
DUOX2	2.169589	-0.01489	0.065615	-0.2269	0.8205	NA
RNASEH2B	54.62941	-0.01489	0.072505	-0.20531	0.837327	NA
SAMD4B	198.924	-0.01488	0.044135	-0.33713	0.736022	NA
CIDEC	0.156423	0.014877	0.02285	0.651062	0.515007	NA
MIR1207	0.147962	0.014873	0.021441	0.693651	0.487901	NA
OR2L2	3.463638	0.014868	0.044691	0.332682	0.739375	NA
CLCC1	285.2798	-0.01486	0.042787	-0.34732	0.728348	NA
UCMA	0.153746	0.014855	0.021502	0.690877	0.489643	NA
COMMD1	51.88862	0.014852	0.068941	0.215434	0.829429	NA
LOH12CR2	3.121739	0.014852	0.076369	0.194481	0.845799	NA
MAGEB3	0.071431	-0.01484	0.016033	-0.92587	0.354515	NA
CCR4	0.078048	-0.01484	0.016031	-0.92579	0.354554	NA
NDUFB10	147.9384	-0.01484	0.068604	-0.21633	0.828729	NA
SUV39H2	114.3386	-0.01484	0.054359	-0.27301	0.784846	NA

FLJ13224	1.097278	-0.01484	0.05592	-0.26537	0.790728	NA
IL2RA	0.103711	0.014835	0.017846	0.831288	0.405811	NA
OR51B6	0.484515	-0.01483	0.034143	-0.43444	0.663973	NA
PRB2	0.475711	0.014831	0.037719	0.393182	0.694185	NA
KLRC1	0.716681	-0.01483	0.042885	-0.34581	0.729482	NA
CANX	1192.36	0.014829	0.060661	0.244459	0.806876	0.934342
ICAM4	0.070887	-0.01481	0.016016	-0.92491	0.355011	NA
LOC10013	28.473	-0.01481	0.077538	-0.19105	0.848488	NA
LOC28400	8.003382	-0.01481	0.084781	-0.17466	0.861347	NA
SLC30A2	0.339404	0.014804	0.033838	0.437493	0.661754	NA
KARS	142.9341	-0.0148	0.054561	-0.2713	0.786158	NA
ATP8B1	27.22077	0.0148	0.080604	0.183619	0.854313	NA
CR2	0.062912	-0.0148	0.014167	-1.04467	0.296175	NA
CRELD2	35.37804	0.014798	0.072005	0.205518	0.837168	NA
ASTL	0.796298	-0.0148	0.049413	-0.29946	0.764588	NA
EMR3	0.074736	-0.01479	0.01624	-0.91078	0.362409	NA
LOC72973	0.69082	0.014777	0.043811	0.337286	0.735901	NA
FUT5	0.070602	-0.01477	0.015902	-0.92894	0.352921	NA
MYH14	359.8415	0.014768	0.065525	0.225383	0.821681	0.942126
CACNA1C	319.0359	-0.01477	0.071649	-0.2061	0.83671	NA
CCDC71L	2.83216	0.014759	0.071852	0.205413	0.837249	NA
RLN3	0.066434	-0.01476	0.015819	-0.9328	0.350926	NA
TRIM31	0.446231	0.014754	0.016603	0.888655	0.374188	NA
STAR	8.877875	-0.01475	0.084472	-0.17466	0.861348	NA
MIR503	0.225205	-0.01475	0.025116	-0.58726	0.55703	NA
DNAJC12	105.3941	-0.01475	0.061926	-0.23814	0.811774	NA
LGMN	54.58144	-0.01474	0.06784	-0.21724	0.828024	NA
HEPHL1	4.52872	-0.01474	0.065345	-0.2255	0.82159	NA
FHL5	12.83317	-0.01473	0.084894	-0.17353	0.862237	NA
OR51E1	0.244559	-0.01473	0.025129	-0.58619	0.557747	NA
ITGB1BP2	6.003785	-0.01472	0.075812	-0.19421	0.846011	NA
MIR4304	0.422054	0.014723	0.038011	0.387335	0.698508	NA
CENPO	40.81906	-0.01472	0.072961	-0.20179	0.840085	NA
ELP4	103.7454	-0.01472	0.051854	-0.28386	0.776519	NA
TSR3	68.11853	-0.01472	0.067564	-0.21782	0.82757	NA
TMLHE	71.06266	-0.0147	0.056552	-0.25986	0.794972	NA
MYO1G	9.97135	0.014695	0.084886	0.173115	0.862561	NA
IQCH-AS1	29.14089	-0.01469	0.072223	-0.20336	0.838856	NA
NOD1	31.5036	-0.01469	0.079209	-0.18542	0.852902	NA
PTPRG	312.8785	-0.01468	0.072035	-0.20383	0.838485	NA
GYS1	192.7529	0.014681	0.060228	0.243765	0.807413	NA
PLEKHF1	6.529145	0.014673	0.083167	0.176426	0.859959	NA
LYRM2	95.22877	-0.01467	0.054396	-0.2697	0.787395	NA
FRMD1	0.21597	-0.01467	0.025165	-0.58288	0.559974	NA
CTAGE10P	0.46848	-0.01466	0.03856	-0.38029	0.703728	NA
CD40	28.03838	0.014663	0.082783	0.177132	0.859405	NA
TUBA1A	441.2094	0.014661	0.072463	0.202321	0.839666	0.946431

INSL5	0.06048	-0.01466	0.014101	-1.03971	0.298474	NA
WNT8A	4.538381	0.014653	0.078144	0.187519	0.851254	NA
ADAM1A	104.0238	0.014647	0.052756	0.277648	0.781283	NA
FUT6	0.060068	-0.01464	0.014093	-1.03914	0.298742	NA
SRPK3	58.30206	-0.01464	0.084538	-0.17323	0.862473	NA
POLR2J2	10.80456	0.014641	0.036939	0.396358	0.691841	NA
CGREF1	118.9766	0.01464	0.060129	0.243484	0.80763	NA
AMBP	2.663476	0.014632	0.069844	0.209499	0.834059	NA
CPLX1	374.1717	-0.01463	0.064445	-0.22699	0.820429	0.941851
POLD2	67.23695	0.014628	0.058882	0.248425	0.803805	NA
CXorf40A	12.59791	-0.01463	0.082914	-0.17639	0.859985	NA
SNORD114	3.131571	-0.01462	0.074794	-0.19549	0.84501	NA
TTC28-AS1	105.2543	-0.01462	0.063495	-0.23026	0.817891	NA
UPK1A-AS	0.429798	-0.01462	0.038024	-0.3844	0.700682	NA
NBPF1	438.3349	0.014613	0.079659	0.183444	0.85445	0.951001
LOC57453	38.89658	0.014612	0.079293	0.184284	0.853791	NA
LOC40149	0.07556	-0.01461	0.01219	-1.19849	0.230726	NA
IFT140	269.8518	-0.01461	0.061152	-0.23887	0.811209	NA
SLC7A6OS	41.8136	0.014606	0.067346	0.216884	0.828298	NA
PHIP	1676.152	0.014601	0.031709	0.460474	0.645176	0.849041
C6orf201	1.394234	0.014599	0.061555	0.237166	0.812528	NA
INO80	369.3788	-0.0146	0.03709	-0.39352	0.693934	0.871418
OR3A1	0.063571	-0.01459	0.015797	-0.92356	0.355715	NA
KIF1C	481.4589	0.014587	0.066462	0.219481	0.826275	0.944098
TYSND1	62.80474	-0.01458	0.061787	-0.23595	0.813474	NA
SEC61B	17.75286	-0.01457	0.082403	-0.17687	0.85961	NA
KRT74	0.192594	-0.01457	0.02578	-0.56532	0.571853	NA
KIAA1211	132.5452	-0.01457	0.062423	-0.23345	0.815409	NA
MIR3940	0.059126	-0.01456	0.014053	-1.03611	0.30015	NA
KLK1	0.11746	-0.01456	0.019538	-0.74505	0.456241	NA
MED13L	1533.684	0.014554	0.038843	0.374692	0.707889	0.883299
DSCR6	0.235287	0.014538	0.027846	0.522095	0.601604	NA
MIR3975	1.557477	-0.01454	0.059234	-0.2454	0.806146	NA
CDC42	152.4555	-0.01453	0.060934	-0.23848	0.811505	NA
MUC6	141.205	-0.01453	0.084235	-0.17247	0.863067	NA
ZCCHC9	40.50552	-0.01452	0.071017	-0.2045	0.837962	NA
TOX4	223.8325	-0.01452	0.04047	-0.35875	0.719783	NA
FKBP11	20.47166	0.01451	0.076579	0.189479	0.849718	NA
GALNS	57.30282	0.014497	0.068302	0.212244	0.831917	NA
LOC40101	0.187783	0.014492	0.02567	0.564531	0.572393	NA
GALNT8	7.412617	-0.01449	0.083258	-0.174	0.861862	NA
ANKRD52	284.2451	-0.01449	0.045676	-0.31717	0.751111	NA
MIR4676	1.308223	0.014485	0.057118	0.253598	0.799806	NA
GUSBP10	0.073046	-0.01448	0.016076	-0.90091	0.367634	NA
RHAG	0.057179	-0.01448	0.014013	-1.03314	0.301538	NA
HIST2H2B	20.57813	0.014476	0.084608	0.171098	0.864147	NA
TM9SF1	35.27162	-0.01448	0.065636	-0.22055	0.825441	NA

FOXB1	0.384543	-0.01447	0.034406	-0.42063	0.674028	NA
LOC72837	0.466117	-0.01447	0.035827	-0.40386	0.686312	NA
HSF1	124.9929	0.014442	0.050663	0.285069	0.775591	NA
CKMT1A	26.04823	-0.01443	0.079986	-0.18043	0.856817	NA
FAM222A-	3.157422	0.01443	0.071312	0.202354	0.83964	NA
EPB42	2.712912	-0.01443	0.070598	-0.20439	0.838052	NA
C7orf65	0.161353	0.014423	0.021628	0.666876	0.504852	NA
KCNA3	56.62469	-0.01442	0.073015	-0.1975	0.843439	NA
FBXW9	8.455446	0.014418	0.084897	0.16983	0.865144	NA
MIR548AV	0.157919	0.014416	0.020087	0.71769	0.472948	NA
MSI2	439.4817	-0.01441	0.046044	-0.31301	0.754272	0.905334
FTO	430.0253	0.014388	0.040558	0.35475	0.722777	0.890244
CCDC41	101.3861	-0.01439	0.064473	-0.22314	0.82343	NA
MIR1224	0.05625	-0.01438	0.013968	-1.02978	0.303114	NA
DPM1	65.9458	-0.01438	0.060626	-0.23721	0.812491	NA
GLRB	90.16575	0.014376	0.068494	0.209892	0.833752	NA
EBPL	11.13131	-0.01437	0.084854	-0.16936	0.865513	NA
CA9	28.30733	0.014368	0.084939	0.169162	0.865669	NA
ZNF337	411.8056	0.014367	0.057983	0.247788	0.804299	0.932553
TRIM61	1.251135	0.014365	0.054557	0.263299	0.79232	NA
MIR618	0.735853	0.014362	0.046418	0.30941	0.757009	NA
PEX13	166.1804	0.014353	0.04955	0.289677	0.772064	NA
NDUFC1	29.36257	0.014351	0.072442	0.198108	0.842961	NA
RNU6-78	1.026701	-0.01434	0.051917	-0.2762	0.782397	NA
CTLA4	0.255244	-0.01434	0.028339	-0.50587	0.61295	NA
MIR548F5	0.225173	0.014335	0.026436	0.542273	0.58763	NA
ST3GAL3	67.79439	0.014329	0.076322	0.18774	0.85108	NA
GOLGA8S	3.440344	-0.01433	0.07663	-0.18695	0.851698	NA
CLEC4D	0.13534	-0.01432	0.019873	-0.72073	0.471073	NA
SNAP29	147.4665	0.01432	0.061599	0.232471	0.816172	NA
ZNF777	95.55668	0.014312	0.060486	0.23662	0.812951	NA
MICALL1	106.1008	0.014311	0.065988	0.21688	0.828302	NA
MUC2	0.228127	-0.01431	0.027817	-0.51445	0.606934	NA
YWHAE	1437.305	0.014306	0.059974	0.238539	0.811463	0.936217
UCA1	0.150845	0.014298	0.021479	0.66568	0.505616	NA
KHDC3L	0.550381	-0.0143	0.036399	-0.39278	0.694483	NA
AP4S1	142.2645	-0.0143	0.047686	-0.2998	0.764331	NA
MXRA7	132.4484	0.014294	0.072244	0.197853	0.84316	NA
PRDM14	0.06577	-0.01429	0.015972	-0.89463	0.370984	NA
FLJ46284	0.260656	-0.01429	0.030528	-0.46804	0.639756	NA
ZCRB1	157.7052	0.014279	0.057527	0.248216	0.803967	NA
ANLN	165.6308	-0.01428	0.084722	-0.16851	0.866181	NA
PTTG1	2.976917	0.014277	0.073584	0.194017	0.846163	NA
VNN1	0.851861	-0.01427	0.042719	-0.33405	0.738342	NA
MIR4489	0.23428	-0.01427	0.02652	-0.538	0.590574	NA
METTL6	87.34143	-0.01427	0.056998	-0.25032	0.802336	NA
FAM160A2	78.31903	0.014264	0.055549	0.256782	0.797347	NA

GABRQ	0.510505	0.014261	0.040511	0.352044	0.724806	NA
ATP6V0E2	25.87601	0.014261	0.078657	0.18131	0.856124	NA
VRK3	70.10563	-0.01425	0.054711	-0.26051	0.794472	NA
OR2L3	1.257995	-0.01425	0.046986	-0.30332	0.761643	NA
AGAP9	0.378281	-0.01425	0.033448	-0.42609	0.670044	NA
CHL1	416.9197	-0.01425	0.077888	-0.18294	0.854846	0.951001
MIR548F2	0.670191	-0.01424	0.044815	-0.31769	0.750724	NA
AKTIP	164.3663	0.014236	0.050023	0.284596	0.775954	NA
OR52K1	0.053934	-0.01423	0.013894	-1.02427	0.305706	NA
CDH18	577.8073	0.01423	0.041584	0.342195	0.732204	0.892995
FOXD4L3	0.241901	-0.01423	0.02783	-0.5112	0.609208	NA
VASP	32.83552	-0.01422	0.080643	-0.17636	0.860011	NA
CDC42EP5	0.901383	-0.01422	0.051672	-0.27523	0.783139	NA
NOX4	0.546471	-0.01422	0.04139	-0.34359	0.731158	NA
FLG2	2.198048	-0.01422	0.052622	-0.2702	0.787006	NA
POU2F2	111.6319	-0.01422	0.060097	-0.23659	0.812973	NA
LIN37	48.0955	-0.01421	0.071772	-0.19802	0.843029	NA
CCDC137	49.40151	-0.0142	0.063317	-0.22432	0.822505	NA
TAGLN3	300.2865	-0.0142	0.063622	-0.22323	0.823356	NA
CXorf64	0.053517	-0.0142	0.01388	-1.0232	0.306214	NA
USP17L4	0.444637	-0.0142	0.037193	-0.38179	0.702619	NA
C12orf69	2.410404	-0.0142	0.068286	-0.20788	0.835323	NA
DKFZp566	0.155031	0.014195	0.021558	0.658442	0.510254	NA
ITGA4	136.397	-0.01419	0.054558	-0.26017	0.794735	NA
TLE2	846.7413	0.014194	0.059893	0.236989	0.812665	0.936217
PPP1R36	0.895884	-0.01419	0.049497	-0.28674	0.774313	NA
NPHP3-AS	0.398893	0.014191	0.03472	0.408718	0.682746	NA
RIMBP2	28.13487	-0.01419	0.081166	-0.17483	0.861212	NA
KANSL1	646.2376	-0.01419	0.07427	-0.19103	0.848499	0.948602
GSX1	0.067146	-0.01418	0.015914	-0.89119	0.372826	NA
MANEAL	45.06583	-0.01418	0.065278	-0.21723	0.828027	NA
CYB5RL	21.30337	0.01418	0.076756	0.184746	0.853428	NA
ANKRD63	0.50874	-0.01418	0.038921	-0.36424	0.715676	NA
LILRB5	0.662745	0.014176	0.041731	0.339702	0.734081	NA
CCDC179	0.483566	-0.01417	0.037409	-0.3787	0.70491	NA
CXCR1	0.42224	-0.01416	0.034016	-0.41639	0.677127	NA
WIBG	25.26235	-0.01416	0.075682	-0.18713	0.851562	NA
RNF145	233.5116	-0.01416	0.04889	-0.28957	0.772146	NA
ZFP112	199.5147	-0.01416	0.058315	-0.24276	0.808191	NA
LGSN	0.139154	-0.01415	0.019973	-0.70857	0.478592	NA
TMEM88	6.492218	0.014143	0.084662	0.167058	0.867325	NA
BPIFA4P	0.111157	-0.01414	0.016337	-0.86566	0.386675	NA
HIST1H3F	0.622192	0.014141	0.041175	0.343424	0.731279	NA
ANAPC4	122.3197	0.01414	0.054478	0.259553	0.795208	NA
OR2AG2	0.052451	-0.01414	0.013849	-1.02086	0.307319	NA
TPH2	0.672992	0.014124	0.046144	0.306076	0.759547	NA
PIN1	188.9182	-0.01412	0.063524	-0.22232	0.824067	NA

CXADRP3	0.11409	-0.01412	0.016524	-0.85451	0.39282	NA
SAMSN1	4.186198	-0.01412	0.076869	-0.18368	0.854266	NA
PTPN11	659.7711	0.01411	0.049035	0.28775	0.773538	0.915715
GJA9-MYC	9.967523	0.014106	0.084927	0.166091	0.868086	NA
GPATCH1	163.1908	0.014101	0.043902	0.32119	0.748067	NA
PCDH15	33.84165	0.014101	0.084935	0.166021	0.868141	NA
C17orf98	0.186666	-0.0141	0.024673	-0.57144	0.567699	NA
ITGA3	216.831	-0.01409	0.056292	-0.25027	0.802379	NA
HS6ST2	20.68855	-0.01408	0.083571	-0.16853	0.866164	NA
KDM1B	141.6331	0.014084	0.055438	0.254042	0.799463	NA
MMRN1	3.166765	0.014082	0.062963	0.223652	0.823028	NA
LOC10012	22.21982	0.014081	0.083352	0.16893	0.865852	NA
CIC	788.7768	-0.01408	0.046041	-0.30572	0.759818	0.90815
TRAPPC5	44.72441	0.014071	0.071619	0.196476	0.844238	NA
METTL21D	62.46481	-0.01407	0.063872	-0.2203	0.825636	NA
SLC22A8	0.391106	-0.01407	0.025558	-0.55056	0.581933	NA
FKRP	22.25388	-0.01407	0.080981	-0.17375	0.862062	NA
ASB10	0.051804	-0.01407	0.013815	-1.01836	0.308508	NA
UOX	1.200168	0.014065	0.057507	0.244585	0.806778	NA
PGM5-AS1	1.663959	-0.01405	0.065382	-0.2149	0.829843	NA
LRRC41	108.7764	-0.01405	0.046384	-0.30286	0.761997	NA
IARS	319.0033	0.014046	0.046925	0.299334	0.764686	NA
ELK1	237.178	0.014044	0.066837	0.210119	0.833575	NA
LINC00597	11.15976	0.014031	0.084932	0.165206	0.868782	NA
MIR4664	0.050849	-0.01403	0.013794	-1.0168	0.309248	NA
CES5AP1	3.561185	-0.01402	0.076051	-0.18436	0.853732	NA
SNX13	370.1994	0.014016	0.040122	0.349322	0.726847	0.891825
WDR75	160.1727	-0.01401	0.048551	-0.28862	0.772871	NA
EPS15L1	447.9953	0.014002	0.042514	0.32934	0.741899	0.896704
SLC25A40	92.79117	0.013992	0.065118	0.214867	0.829871	NA
MIR320B2	4.601747	-0.01399	0.080338	-0.1741	0.861789	NA
CBLB	523.3428	0.013983	0.045686	0.306073	0.759549	0.90815
BRWD1-AS	5.265226	0.013981	0.083216	0.16801	0.866575	NA
PDGFA	83.47952	-0.01398	0.0627	-0.22295	0.823574	NA
RBFOX2	1780.143	-0.01397	0.027166	-0.51419	0.607118	0.831894
CAMSAP3	111.3969	0.013965	0.049696	0.281014	0.7787	NA
DNMT3L	0.180146	0.01396	0.020324	0.68686	0.492171	NA
CTXN1	0.996675	-0.01394	0.054723	-0.25473	0.79893	NA
KAT7	462.8175	-0.01394	0.037383	-0.37282	0.709285	0.884085
DIS3L2	269.7881	-0.01394	0.056697	-0.2458	0.805837	NA
RFPL2	11.0176	0.01392	0.083444	0.166821	0.867511	NA
ZNF711	182.3732	0.013916	0.058594	0.237502	0.812267	NA
LINC00202	22.87605	0.013914	0.084364	0.164933	0.868997	NA
CD177	5.263996	-0.01391	0.07859	-0.17704	0.859479	NA
CHCHD5	6.279734	-0.01391	0.084478	-0.16464	0.869228	NA
SNX30	382.6651	0.013908	0.047224	0.294509	0.768369	0.911658
OR2B2	0.722809	0.013906	0.043762	0.317756	0.75067	NA

TCTA	53.31343	-0.0139	0.05981	-0.23245	0.816187	NA
MDM2	511.3925	0.013899	0.037446	0.371172	0.71051	0.884085
CDC42EP4	283.0511	-0.01389	0.067802	-0.20487	0.837675	NA
EPOR	20.53865	-0.01389	0.082125	-0.16911	0.865707	NA
NKX6-3	7.087671	-0.01389	0.083513	-0.1663	0.867924	NA
C21orf90	0.048984	-0.01388	0.013724	-1.01153	0.311763	NA
ZWINT	2.980471	-0.01388	0.074915	-0.18529	0.853	NA
TMEM200	20.38033	-0.01387	0.084884	-0.16336	0.870238	NA
ANKDD1A	108.5184	0.013866	0.055954	0.247802	0.804288	NA
HNRNPA3	0.252057	-0.01386	0.029724	-0.46637	0.64095	NA
FMO1	0.19199	0.01386	0.024671	0.561809	0.574246	NA
AGK	176.6901	-0.01384	0.052094	-0.26561	0.79054	NA
ZNF792	67.65774	0.01383	0.070005	0.197551	0.843397	NA
SNORD36C	6.909303	-0.01383	0.080806	-0.17113	0.864119	NA
PDE6D	34.8407	-0.01382	0.075976	-0.18195	0.855625	NA
RABL2A	26.57658	-0.01382	0.079332	-0.17416	0.861742	NA
USP5	138.1311	0.013809	0.066767	0.206831	0.836142	NA
EIF5B	483.2167	-0.0138	0.040061	-0.34446	0.730497	0.892995
PDGFC	8.013705	-0.0138	0.083847	-0.16458	0.869273	NA
C12orf23	101.8643	-0.01379	0.059644	-0.23119	0.817164	NA
MPO	2.719392	-0.01379	0.073352	-0.18799	0.850886	NA
LOC72853	22.2105	-0.01379	0.078675	-0.17527	0.860869	NA
LYRM9	27.10716	-0.01379	0.078423	-0.17582	0.860433	NA
CS	226.6605	0.013784	0.047638	0.289352	0.772312	NA
MIR665	0.916208	-0.01378	0.050288	-0.27401	0.78408	NA
MGAT4A	560.9217	0.013778	0.040813	0.337577	0.735682	0.895185
AWAT2	0.121385	-0.01377	0.017947	-0.7674	0.442844	NA
RFC4	51.4347	0.013766	0.075297	0.182822	0.854937	NA
MIR943	0.467548	-0.01376	0.039023	-0.3527	0.724311	NA
LOC34010	0.295664	0.013757	0.02277	0.604194	0.545715	NA
GCA	66.22694	-0.01376	0.07974	-0.17251	0.863037	NA
FBXO39	0.307706	0.013756	0.030504	0.45096	0.652019	NA
MYO1C	117.2085	0.013752	0.050529	0.272152	0.785505	NA
CARKD	133.6479	-0.01375	0.047842	-0.28736	0.773833	NA
PSMC6	127.7082	0.013745	0.058436	0.235213	0.814044	NA
RORB	29.55986	0.013744	0.082335	0.166924	0.86743	NA
OR13G1	0.345258	-0.01374	0.030147	-0.45589	0.648466	NA
SKP2	77.22939	0.013743	0.05148	0.266962	0.789499	NA
REM1	0.587956	0.013743	0.037776	0.363806	0.716003	NA
GINS1	24.2828	0.013737	0.077472	0.177318	0.859258	NA
CSF3	1.597668	0.013737	0.055089	0.249352	0.803089	NA
C3orf36	0.271267	-0.01373	0.028025	-0.49002	0.624119	NA
GLS2	215.6185	-0.01373	0.042283	-0.32469	0.745418	NA
AP4B1-AS1	8.110285	0.013728	0.084823	0.161847	0.871427	NA
SPOCD1	0.557192	-0.01373	0.044168	-0.31077	0.755976	NA
PTCH2	72.49125	-0.01372	0.074603	-0.1839	0.854093	NA
CCDC108	0.762315	0.013708	0.04534	0.302325	0.762404	NA

MED20	44.7418	0.013707	0.065089	0.210587	0.83321	NA
LOC28475	0.440453	-0.0137	0.038637	-0.3547	0.722816	NA
BRCA1	29.6435	0.013695	0.0727	0.188371	0.850586	NA
UMODL1	0.590223	-0.01369	0.043211	-0.31688	0.751332	NA
FAH	13.26243	0.013693	0.082843	0.165285	0.86872	NA
S100PBP	169.0425	-0.01369	0.045258	-0.30253	0.762249	NA
ANKRD53	18.00004	-0.01368	0.0812	-0.16849	0.866197	NA
MIR4520A	0.178696	0.01368	0.020076	0.681418	0.495607	NA
EIF5AL1	2.044676	0.013678	0.065747	0.208046	0.835193	NA
SCARA5	0.565716	-0.01368	0.040555	-0.33722	0.735955	NA
CLPP	47.4019	-0.01367	0.065082	-0.21002	0.833649	NA
RNASEH2A	28.95566	0.013665	0.073832	0.185084	0.853163	NA
FLJ14107	7.987406	-0.01366	0.084205	-0.16228	0.871086	NA
CMSS1	73.15456	-0.01366	0.07273	-0.18784	0.851003	NA
GTF3C1	658.8693	0.013661	0.040634	0.336203	0.736718	0.895688
BPGM	44.02244	0.013661	0.073841	0.185005	0.853225	NA
BMP5	0.942803	-0.01366	0.041505	-0.32907	0.742104	NA
TDG	40.13221	-0.01366	0.069096	-0.19765	0.843319	NA
TECRL	0.281562	0.013656	0.029419	0.464196	0.642508	NA
IL4I1	1.964983	-0.01365	0.065995	-0.20688	0.836106	NA
MTRNR2L	238.3256	-0.01365	0.043965	-0.31052	0.756169	NA
FZD4	47.49554	-0.01365	0.070914	-0.19245	0.847389	NA
MIR608	1.100778	-0.01364	0.054524	-0.25024	0.802404	NA
GALNT1	88.79301	-0.01364	0.057672	-0.23651	0.813035	NA
NNT	179.3546	0.013638	0.043635	0.312553	0.75462	NA
PEX19	140.9998	-0.01364	0.04852	-0.28104	0.778677	NA
OSBPL10-A	0.515934	0.013633	0.040115	0.339854	0.733967	NA
CXorf22	1.155845	0.013626	0.051094	0.266687	0.78971	NA
CLEC19A	1.656176	0.013623	0.056747	0.24007	0.810276	NA
FCN2	0.228393	-0.01362	0.026608	-0.51199	0.608661	NA
PCDHGC3	329.2565	0.01362	0.054287	0.250895	0.801896	NA
FLJ39051	3.360933	-0.01362	0.077134	-0.17655	0.859862	NA
SYNJ2-IT1	0.407124	-0.01361	0.035535	-0.38303	0.701701	NA
PIAS1	277.1037	-0.0136	0.037212	-0.36554	0.714711	NA
NEK10	8.451829	0.013599	0.084932	0.160113	0.872792	NA
IPO9	555.1552	0.013597	0.031896	0.426285	0.6699	0.861137
MIR593	0.535008	-0.01359	0.04267	-0.31859	0.750034	NA
DARC	194.4977	-0.01359	0.081751	-0.16621	0.867993	NA
ZNF440	74.20017	-0.01359	0.055924	-0.24297	0.808031	NA
C11orf1	13.70874	0.013587	0.081919	0.165854	0.868272	NA
AK4	144.655	0.013586	0.061006	0.222701	0.823768	NA
HERPUD2	118.4158	-0.01358	0.049302	-0.27553	0.782906	NA
TP73	48.92842	-0.01356	0.0828	-0.16379	0.869898	NA
MAPRE2	668.8695	0.013559	0.050892	0.266426	0.789911	0.9252
DHRS7	74.80469	0.013558	0.060592	0.223754	0.822949	NA
MIR5091	0.57921	0.013557	0.042272	0.320711	0.748429	NA
DAAM2	217.8657	0.01355	0.077148	0.175639	0.860578	NA

CACTIN	86.04729	0.013548	0.061557	0.220081	0.825808	NA
ACTA2	50.5857	0.013546	0.084818	0.159709	0.87311	NA
MIRLET7F	0.568493	-0.01355	0.043356	-0.31242	0.754722	NA
MIR4730	2.089805	-0.01354	0.060352	-0.22438	0.822465	NA
MALAT1	210070.8	-0.01354	0.062085	-0.21809	0.82736	0.944098
KCNJ14	12.18916	-0.01353	0.084938	-0.15932	0.873417	NA
BLK	0.754578	-0.01353	0.044267	-0.30568	0.759851	NA
MANF	33.36444	0.01353	0.084858	0.159449	0.873315	NA
TINAGL1	13.77476	0.01353	0.084383	0.160339	0.872614	NA
SLC38A1	923.3859	0.013528	0.063508	0.213008	0.831321	0.944594
HAR1B	1.185267	-0.01352	0.059445	-0.22741	0.820104	NA
ZMYND12	3.216786	-0.01352	0.075334	-0.17943	0.857602	NA
OR2A5	0.184658	-0.01351	0.02318	-0.58291	0.559953	NA
NOA1	44.53423	0.013509	0.067857	0.199074	0.842205	NA
LINC00858	0.509497	-0.0135	0.035978	-0.37514	0.707554	NA
PSKH1	57.17456	-0.01349	0.067444	-0.20005	0.84144	NA
LINC00294	105.0323	-0.01349	0.047353	-0.28487	0.775744	NA
MIR33A	2.274233	-0.01348	0.071945	-0.18739	0.851355	NA
RLN1	0.348784	0.013471	0.031958	0.421527	0.67337	NA
FBXO47	0.100701	-0.01347	0.017762	-0.75822	0.448316	NA
MIR550A3	0.410612	-0.01347	0.03503	-0.38442	0.700664	NA
YAE1D1	38.23014	0.013464	0.069546	0.1936	0.846489	NA
WBP1L	109.8638	-0.01346	0.045277	-0.29733	0.766215	NA
ERCC6L2	147.3726	0.013459	0.04936	0.272671	0.785106	NA
LOC10105	0.187254	0.013457	0.024654	0.545824	0.585187	NA
GIGYF2	882.8666	0.013456	0.032967	0.408168	0.68315	0.867558
HKR1	280.3497	0.013446	0.041856	0.321248	0.748022	NA
AKAP6	1019.31	0.013443	0.032796	0.409909	0.681872	0.867105
MBTPS2	102.2688	-0.01344	0.075793	-0.17736	0.859222	NA
CREG1	100.1022	-0.01344	0.068078	-0.19738	0.843529	NA
MT1L	1.452977	0.01343	0.060618	0.221557	0.824658	NA
RBBP8NL	0.289979	0.013416	0.031369	0.42769	0.668877	NA
ADAMTS1	414.62	0.013409	0.064862	0.206731	0.83622	0.9456
AACSP1	1.351752	-0.01341	0.040835	-0.32832	0.742668	NA
ABRA	9.11321	0.013407	0.084835	0.158031	0.874432	NA
SNORD83B	2.315251	-0.01341	0.060473	-0.22169	0.824557	NA
APBB1	553.8357	-0.0134	0.046909	-0.28569	0.775112	0.916737
GNRHR2	20.5099	0.0134	0.079034	0.169541	0.865371	NA
FRMD8	55.04829	-0.0134	0.058768	-0.22794	0.819693	NA
AJUBA	4.204672	-0.01339	0.080409	-0.16658	0.867698	NA
PHC2	222.839	0.013395	0.041014	0.32659	0.743978	NA
MIR3619	8.196459	0.013392	0.084936	0.157673	0.874715	NA
SNORD18B	0.734189	0.01339	0.046735	0.286519	0.774481	NA
ICA1	221.4528	-0.01339	0.073424	-0.18235	0.85531	NA
WDR43	161.9949	0.013387	0.046756	0.286321	0.774633	NA
FUNDC2P2	0.192194	-0.01339	0.024716	-0.54158	0.588105	NA
NEDD1	15.50363	0.013385	0.083273	0.160738	0.872299	NA

FOXJ2	252.447	-0.01338	0.049837	-0.26851	0.788309	NA
SSTR5-AS1	1.561631	0.013374	0.053324	0.250806	0.801964	NA
PCDHA8	61.52069	0.013354	0.075124	0.177756	0.858915	NA
C1QTNF4	43.98503	-0.01335	0.080731	-0.16536	0.868662	NA
ABCC5	661.0492	-0.01334	0.063554	-0.20989	0.833754	0.944594
STEAP4	40.94869	0.013338	0.084448	0.157943	0.874502	NA
SF3B1	1484.29	0.013337	0.035635	0.374276	0.708199	0.883299
LOC10013	22.815	0.013329	0.078709	0.169345	0.865525	NA
ALDH16A1	13.75428	0.013328	0.082757	0.161054	0.872051	NA
ERBB4	440.862	0.013326	0.067605	0.197115	0.843737	0.946541
AGAP8	3.362818	0.013321	0.071694	0.185799	0.852602	NA
PHKG2	79.66003	-0.01332	0.062735	-0.21233	0.831849	NA
LOC44035	1.772521	0.013313	0.067618	0.196893	0.843912	NA
MIR4678	0.140481	-0.01331	0.020084	-0.66287	0.507415	NA
ITSN2	432.2008	-0.01331	0.051824	-0.25687	0.797281	0.929506
SNORA38B	2.562709	-0.01331	0.066881	-0.19899	0.842267	NA
OAZ1	343.6215	-0.01331	0.062614	-0.21254	0.831686	0.944594
FHAD1	2.813973	-0.01329	0.074675	-0.17802	0.858706	NA
HLA-G	2.66821	-0.01329	0.064416	-0.20629	0.836562	NA
ABCC2	13.7653	-0.01329	0.083678	-0.15879	0.873831	NA
FAM86C2P	4.821949	-0.01328	0.078816	-0.16855	0.866147	NA
GLB1	54.19766	-0.01328	0.059214	-0.22432	0.822506	NA
RRP1	96.40458	-0.01328	0.05209	-0.25497	0.798748	NA
MIR555	0.291676	-0.01328	0.029473	-0.45062	0.652266	NA
FLJ38576	3.403685	0.013281	0.073687	0.180229	0.856972	NA
TNP2	0.322348	-0.01328	0.034087	-0.38949	0.696912	NA
CMPK1	235.2166	-0.01328	0.055529	-0.23908	0.811043	NA
ACTR1A	269.1715	-0.01327	0.03758	-0.35321	0.723932	NA
LOC38903	0.141243	0.013264	0.019928	0.665614	0.505658	NA
FAM208B	466.1188	0.013257	0.033333	0.397706	0.690847	0.870767
SNAPC1	91.53916	0.013255	0.057618	0.230042	0.818059	NA
GALNTL6	7.738699	-0.01325	0.084914	-0.15603	0.876008	NA
CLUHP3	125.8864	0.013246	0.066147	0.200248	0.841287	NA
ORMDL1	69.85598	-0.01324	0.063051	-0.21004	0.833636	NA
ZRANB2-A	8.150295	0.013242	0.084509	0.156698	0.875483	NA
LOC64632	91.13314	0.013242	0.083334	0.158906	0.873743	NA
OR1D2	0.6185	-0.01324	0.042545	-0.31114	0.755698	NA
SEMA5A	56.58444	-0.01323	0.079588	-0.16625	0.867957	NA
PODXL	187.5346	0.013227	0.08227	0.16078	0.872267	NA
PQLC3	19.93904	0.013222	0.078608	0.168199	0.866427	NA
CLCF1	18.42514	-0.01322	0.083823	-0.15768	0.874709	NA
NUP214	367.4579	0.013213	0.039702	0.332796	0.739288	0.895927
THAP5	190.6454	0.01321	0.057004	0.231735	0.816744	NA
MIR3973	2.526542	0.01321	0.069755	0.189372	0.849802	NA
FBXO32	40.49605	0.013206	0.079392	0.166344	0.867887	NA
HGS	270.6996	-0.0132	0.042676	-0.30921	0.75716	NA
CARD18	0.123528	-0.01319	0.017978	-0.73346	0.46328	NA

TIGD6	27.62865	-0.01319	0.080309	-0.16419	0.86958	NA
LOC72991	3.855004	-0.01318	0.079957	-0.16487	0.869043	NA
ILK	146.9144	-0.01318	0.050366	-0.26174	0.793524	NA
SCML2	17.37037	-0.01317	0.081737	-0.16117	0.87196	NA
PHACTR3	537.0303	-0.01317	0.038468	-0.34237	0.732073	0.892995
C16orf46	27.41472	0.013161	0.079412	0.165737	0.868364	NA
C9orf3	165.8693	-0.01316	0.059425	-0.22146	0.824732	NA
ATF3	8.157301	-0.01316	0.075899	-0.17339	0.862349	NA
LYPLA1	64.99488	-0.01316	0.073498	-0.17899	0.857942	NA
HBG2	0.251769	-0.01315	0.028883	-0.4553	0.648891	NA
PTPN21	136.7549	-0.01315	0.049484	-0.26573	0.790449	NA
HIATL2	13.29095	0.013149	0.081363	0.16161	0.871613	NA
ADAMTS1	1.769878	-0.01314	0.065475	-0.20067	0.840953	NA
DGKG	910.9952	-0.01314	0.049328	-0.26632	0.789994	0.9252
RNASE3	0.1914	-0.01314	0.023383	-0.5618	0.574254	NA
OCA2	0.215009	0.013136	0.021992	0.597297	0.550309	NA
ZNF17	165.6834	0.013121	0.071562	0.183354	0.854521	NA
DCLK2	167.9963	0.013106	0.05557	0.235856	0.813544	NA
LRSAM1	170.7118	0.013104	0.050759	0.25817	0.796275	NA
TPTE2	7.810298	-0.01309	0.081	-0.16165	0.871578	NA
VWA2	68.26934	-0.01309	0.079124	-0.16545	0.868591	NA
PALM2	35.25877	0.013087	0.082349	0.158925	0.873728	NA
TEP1	116.326	-0.01309	0.062615	-0.209	0.834452	NA
MARVELD	20.12926	-0.01308	0.083688	-0.15632	0.875784	NA
CEBPA-AS1	13.02387	-0.01308	0.081968	-0.15958	0.873209	NA
FLJ46066	0.531047	-0.01307	0.038453	-0.33997	0.733882	NA
LRRC70	7.919931	-0.01307	0.084919	-0.15391	0.877683	NA
FAM101A	0.108775	-0.01307	0.017844	-0.73218	0.464058	NA
C1orf87	0.248595	0.013057	0.019979	0.653539	0.513409	NA
DUSP28	28.23284	-0.01306	0.07183	-0.18176	0.85577	NA
VNN3	0.298731	-0.01305	0.026495	-0.49274	0.622199	NA
LOC15038	138.1287	0.013052	0.083904	0.155559	0.876381	NA
SAC3D1	24.61796	0.01305	0.076215	0.171229	0.864044	NA
DNAJC30	55.76676	0.013047	0.062804	0.207742	0.83543	NA
CRCP	169.7255	-0.01304	0.043637	-0.29873	0.765143	NA
C11orf73	28.12378	-0.01303	0.07864	-0.16575	0.86835	NA
FNBP1	1413.178	-0.01303	0.044673	-0.29176	0.770468	0.913192
MARK2P9	0.730477	0.013033	0.042734	0.304981	0.76038	NA
LRG1	0.604977	0.013033	0.041576	0.313477	0.753918	NA
CD2BP2	123.1325	0.013029	0.045346	0.287332	0.773858	NA
LOC10013	12.3965	-0.01303	0.082532	-0.15782	0.874597	NA
DPEP3	0.259188	-0.01302	0.029765	-0.43758	0.661691	NA
NUBP2	43.8577	-0.01302	0.070884	-0.18368	0.854267	NA
MIR1243	1.070709	0.013019	0.053739	0.242269	0.808571	NA
FOXN3	1186.634	0.013015	0.047476	0.274141	0.783976	0.924365
ING3	164.7124	0.013015	0.058889	0.221006	0.825088	NA
PLP2	16.27427	-0.01301	0.082632	-0.1575	0.874853	NA

ZNF35	65.23267	0.013013	0.064407	0.20205	0.839878	NA
ADCY6	220.8146	-0.01301	0.05609	-0.23194	0.816587	NA
LOC40198	0.320099	0.013007	0.032067	0.405632	0.685013	NA
COPZ2	2.618132	0.013002	0.075113	0.173095	0.862577	NA
SNORA56	1.112234	0.013001	0.052085	0.249605	0.802893	NA
ZYX	145.9328	-0.013	0.05986	-0.21714	0.8281	NA
NKX2-8	0.229822	-0.01298	0.026541	-0.48907	0.624795	NA
GLIPR1L2	7.660506	-0.01298	0.080997	-0.16024	0.872688	NA
C1orf192	71.59347	0.012978	0.055584	0.233475	0.815392	NA
CHCHD4	27.45078	0.012977	0.074207	0.174877	0.861176	NA
MIR129-2	0.095841	-0.01297	0.017674	-0.73408	0.462903	NA
NFIC	820.5516	0.012966	0.048472	0.267487	0.789094	0.925102
BEND2	0.102582	-0.01296	0.017914	-0.72324	0.469531	NA
KDR	15.58044	-0.01295	0.083883	-0.15437	0.877318	NA
EZH2	54.94746	-0.01295	0.067382	-0.19214	0.847635	NA
TTC30B	10.4051	0.012946	0.084336	0.153506	0.878	NA
MIR328	0.104173	-0.01295	0.017906	-0.72294	0.469719	NA
LOC14582	0.391378	0.012944	0.0346	0.37412	0.708315	NA
ANKRD20A	8.384538	0.012941	0.059693	0.216794	0.828369	NA
CLEC4F	2.887769	-0.01293	0.061496	-0.21033	0.833409	NA
ADRBK2	232.6917	0.012932	0.069644	0.185684	0.852693	NA
RBP2	4.075693	0.012928	0.077056	0.167769	0.866765	NA
NOP58	212.228	-0.01293	0.050446	-0.25625	0.797754	NA
SNORA9	1.862946	-0.01293	0.065014	-0.19882	0.8424	NA
C22orf15	2.20716	-0.01293	0.066249	-0.1951	0.845312	NA
OPN5	0.890475	-0.01292	0.044776	-0.28861	0.772883	NA
CENPJ	519.8184	0.012917	0.079348	0.162793	0.870681	0.958257
RAB8A	136.05	0.012915	0.051105	0.252705	0.800496	NA
SNORD79	8.917042	0.012906	0.08437	0.152972	0.87842	NA
ROR2	0.270272	-0.0129	0.029514	-0.43721	0.661959	NA
TTC18	41.72545	-0.0129	0.068712	-0.18775	0.851071	NA
A4GNT	0.277763	0.0129	0.03201	0.403007	0.686943	NA
LINC00152	0.284175	0.012898	0.030518	0.422618	0.672574	NA
WNT9B	1.194985	-0.01289	0.054219	-0.23778	0.812052	NA
BRMS1L	106.6144	0.012889	0.059887	0.215222	0.829594	NA
GRPEL1	55.96867	0.012878	0.06486	0.198557	0.842609	NA
KIAA0146	412.9759	-0.01287	0.057876	-0.22245	0.82396	0.943991
CPA1	0.253949	0.012872	0.026611	0.483715	0.628588	NA
ISM2	0.097162	-0.01287	0.017708	-0.7266	0.467468	NA
GPR78	1.084506	0.012866	0.048944	0.26288	0.792643	NA
C1orf159	20.08211	0.01286	0.078382	0.164069	0.869677	NA
ANXA10	0.169122	0.012858	0.020178	0.637246	0.523965	NA
CSNK1G2	298.9415	0.012858	0.054806	0.234607	0.814514	NA
POLR3D	110.3318	0.012841	0.053464	0.240183	0.810189	NA
LOC10049	37.65091	0.012834	0.076448	0.16788	0.866678	NA
WNT3A	0.182619	0.012832	0.024623	0.521148	0.602264	NA
HMGA2	0.181962	0.012822	0.020218	0.634154	0.52598	NA

XPNPEP2	0.071867	0.012821	0.014127	0.907538	0.364123	NA
MIR4766	0.357917	-0.01282	0.034086	-0.37606	0.706876	NA
FBF1	102.3976	0.012808	0.068051	0.188215	0.850708	NA
CLCN2	81.81834	0.012805	0.070089	0.182695	0.855037	NA
ZNF814	281.4142	-0.0128	0.063105	-0.20289	0.839223	NA
VMP1	129.9069	-0.0128	0.052873	-0.2421	0.808699	NA
MIR5047	44.05566	-0.0128	0.079653	-0.16069	0.872336	NA
ERC1	2529.807	-0.01279	0.042259	-0.30271	0.762109	0.908993
CNTRL	313.3916	0.012791	0.050736	0.252106	0.800959	NA
LINC00515	8.578776	0.012789	0.084883	0.15067	0.880236	NA
DNAH11	32.4789	0.012789	0.084694	0.150998	0.879977	NA
AKR7A2	70.75518	0.012788	0.055222	0.231573	0.81687	NA
C6orf203	47.70083	0.012788	0.062928	0.203212	0.83897	NA
SIRT2	138.0135	0.012786	0.059622	0.214459	0.830189	NA
NQO2	209.89	-0.01278	0.06289	-0.20325	0.838937	NA
SLFN12	4.588224	0.01278	0.078917	0.161939	0.871354	NA
NUAK2	42.6835	0.01278	0.076402	0.167269	0.867158	NA
LINC00570	0.095514	-0.01278	0.017728	-0.7208	0.471033	NA
PNPLA4	21.93973	0.012775	0.083686	0.152653	0.878672	NA
FDXR	38.68637	-0.01276	0.0663	-0.19243	0.847403	NA
YTHDF1	157.9364	-0.01275	0.039998	-0.31879	0.749887	NA
GTF2IRD1	56.03544	-0.01274	0.073227	-0.17403	0.861846	NA
MIR876	1.116266	-0.01274	0.039052	-0.32628	0.74421	NA
PKN1	212.3918	0.012741	0.052168	0.244227	0.807055	NA
ULK2	262.7953	0.012739	0.036002	0.353847	0.723454	NA
FAM219A	226.337	-0.01273	0.045632	-0.279	0.780241	NA
C4orf6	0.112663	0.012731	0.016255	0.783233	0.43349	NA
IFNL1	1.143924	-0.01273	0.053344	-0.23865	0.811375	NA
UTP18	152.9917	-0.01273	0.048598	-0.26192	0.793382	NA
CECR5-AS1	5.357531	-0.01272	0.083063	-0.15318	0.878256	NA
MIP	0.853756	-0.01272	0.048545	-0.26209	0.793255	NA
BSND	4.360957	0.012718	0.079006	0.160979	0.87211	NA
PLSCR2	0.255969	-0.01272	0.029761	-0.42727	0.669181	NA
SCNN1B	4.850646	0.012709	0.079741	0.159372	0.873376	NA
LOC10012	0.278269	-0.01271	0.030804	-0.41251	0.679969	NA
KDELR3	27.29004	0.012703	0.077595	0.163707	0.869962	NA
BTNL8	0.179492	-0.0127	0.023339	-0.544	0.586441	NA
ARMC7	59.7821	-0.01269	0.060464	-0.20994	0.833715	NA
PAK7	275.7022	0.012693	0.051478	0.246579	0.805234	NA
IFI44L	25.61629	0.012693	0.07828	0.162146	0.871191	NA
PAN2	350.2697	0.012692	0.064096	0.198015	0.843034	0.946541
TANC1	451.7235	0.012682	0.051236	0.247518	0.804507	0.932553
FOXD1	2.874615	-0.01268	0.069667	-0.18204	0.855554	NA
EXOC3L4	0.068241	0.01268	0.014032	0.903612	0.366201	NA
UNC13B	349.5632	-0.01268	0.058141	-0.21805	0.827388	0.944098
MIR601	1.386408	0.012676	0.060259	0.210367	0.833381	NA
PPP6R3	604.7339	0.012675	0.027337	0.463654	0.642895	0.848802

TMEM64	37.57796	0.012671	0.070277	0.180301	0.856916	NA
MOGAT2	0.097275	-0.01266	0.017748	-0.71358	0.475484	NA
NBPF7	0.668909	-0.01266	0.043171	-0.29333	0.769271	NA
EMC2	172.4422	-0.01265	0.053183	-0.23794	0.811926	NA
GLOD4	75.52272	-0.01264	0.066416	-0.19039	0.849007	NA
HLX	4.085267	0.012645	0.081219	0.155685	0.876281	NA
SLC35C2	67.0801	-0.01264	0.062912	-0.20096	0.840726	NA
LOC28554	0.19877	0.012642	0.023437	0.539407	0.589606	NA
GNAT3	0.169884	0.012642	0.020062	0.630147	0.528599	NA
FITM1	1.088047	-0.01264	0.055503	-0.22771	0.819868	NA
SBNO2	75.46268	-0.01263	0.06654	-0.18984	0.849431	NA
MIR574	0.093514	-0.01263	0.017688	-0.71382	0.475338	NA
MSRB3	81.42209	-0.01262	0.057496	-0.21953	0.826235	NA
PRPF38A	336.6588	0.012622	0.043664	0.289071	0.772527	0.915154
C16orf54	0.236081	-0.01261	0.026581	-0.47457	0.635093	NA
MORC4	61.59444	0.012612	0.075068	0.168008	0.866577	NA
WDR1	134.2074	-0.01261	0.056419	-0.22347	0.823173	NA
CPXM1	0.616833	0.012605	0.042173	0.298894	0.765021	NA
HIST1H2A	2.196806	0.012603	0.061769	0.204034	0.838327	NA
FABP3	60.64669	-0.0126	0.063881	-0.19723	0.843644	NA
FLJ37505	0.093537	-0.0126	0.017704	-0.71159	0.476719	NA
CEP41	30.28267	-0.01259	0.083654	-0.15054	0.880337	NA
KLRC4-KLR	0.092263	-0.01259	0.017723	-0.71025	0.477552	NA
TRIM60	0.199716	0.012576	0.025068	0.501673	0.615897	NA
LOC28479	3.549019	0.012572	0.075866	0.165711	0.868384	NA
GUSBP5	0.636361	-0.01255	0.044283	-0.28352	0.776782	NA
ZNF649	182.021	-0.01255	0.051991	-0.2414	0.809246	NA
ESRRG	141.6682	-0.01254	0.082967	-0.15111	0.879885	NA
MIR376C	0.728341	0.01253	0.046339	0.270403	0.786851	NA
PLAU	1.037495	0.012529	0.053813	0.232834	0.81589	NA
C3orf14	45.58503	-0.01253	0.070752	-0.17706	0.859462	NA
OR2G3	0.462017	-0.01253	0.038492	-0.32542	0.744862	NA
DST	7151.7	-0.01252	0.040593	-0.30853	0.757683	0.907536
CHP2	0.101599	0.012524	0.01772	0.706781	0.479703	NA
C1GALT1	110.0303	-0.01252	0.050681	-0.24711	0.804822	NA
OR52I1	2.807015	-0.01252	0.075146	-0.16664	0.867652	NA
LOC64380	0.100327	-0.01252	0.014009	-0.89386	0.371399	NA
CEBPA	14.8291	0.012522	0.083825	0.149379	0.881255	NA
NLGN4Y-A	0.88858	-0.01252	0.032126	-0.38966	0.696786	NA
HTR7	0.580033	0.012512	0.04157	0.300976	0.763432	NA
SNHG3	33.98428	-0.01251	0.08245	-0.15172	0.879411	NA
ZNF716	0.362007	0.012507	0.032217	0.388222	0.697852	NA
HECTD3	127.3707	0.012504	0.042104	0.296968	0.766491	NA
CHRNE	13.38924	-0.0125	0.084134	-0.14861	0.881858	NA
PNMT	3.483037	-0.0125	0.070914	-0.17621	0.860132	NA
NECAB3	13.67996	0.012494	0.08489	0.147176	0.882993	NA
KNG1	0.195075	0.01249	0.025762	0.484835	0.627794	NA

PITPNA-AS	13.52354	0.012489	0.083656	0.149293	0.881323	NA
LINC00536	0.092846	-0.01249	0.017693	-0.70576	0.480336	NA
ILF2	128.277	-0.01249	0.053072	-0.23528	0.813993	NA
DCTN1	2112.535	0.012485	0.036125	0.345604	0.72964	0.892833
ADRA1D	3.098252	0.012482	0.074883	0.16668	0.867622	NA
TMPPE	50.98354	0.012479	0.064298	0.194079	0.846114	NA
LOC64649	1.193062	0.012474	0.051445	0.242477	0.80841	NA
RFWD2	180.4646	0.01247	0.046995	0.265351	0.790739	NA
PRR4	0.282447	0.012466	0.031363	0.397469	0.691022	NA
ATP6V0CP	0.688064	-0.01246	0.045658	-0.27298	0.784872	NA
TRAF3IP2-	185.5749	0.012457	0.06811	0.182901	0.854875	NA
CLIP3	1212.606	0.012456	0.05644	0.220685	0.825337	0.944098
HIST1H2A	0.159801	-0.01245	0.022808	-0.54583	0.585183	NA
LOC10013	65.47809	0.012446	0.083274	0.149456	0.881194	NA
LAG3	3.259601	-0.01244	0.076749	-0.16211	0.871223	NA
TUSC3	179.3906	0.012438	0.063723	0.195184	0.845249	NA
DNAH3	4.680352	0.012425	0.077967	0.159362	0.873384	NA
OSR2	0.067755	0.012422	0.014025	0.885693	0.375783	NA
TTN	789.2347	0.01242	0.081726	0.151967	0.879213	0.959988
SNORD76	56.0242	-0.01242	0.084316	-0.14729	0.882901	NA
GOLGA6C	0.151201	-0.01242	0.022674	-0.54772	0.583882	NA
HSD17B2	0.095479	-0.01242	0.017702	-0.70156	0.482954	NA
ARRDC5	0.7511	-0.01242	0.049694	-0.24986	0.802694	NA
FAM160B2	168.1069	-0.01241	0.055602	-0.22323	0.823359	NA
TEN1-CDK	2.435268	0.012403	0.07225	0.171666	0.8637	NA
MIR611	0.094954	-0.0124	0.017756	-0.69815	0.485082	NA
SMIM9	0.140996	-0.01239	0.018454	-0.67156	0.501864	NA
PSORS1C3	1.71688	0.012392	0.0585	0.21183	0.83224	NA
MTMR7	269.7907	0.012387	0.035635	0.347622	0.728124	NA
NEGR1-IT1	4.832918	-0.01238	0.080046	-0.15467	0.877083	NA
SNORD44	6.282547	-0.01238	0.079701	-0.15532	0.876569	NA
DCAF17	109.8782	-0.01238	0.046803	-0.26442	0.791454	NA
XKRX	0.564219	-0.01237	0.043378	-0.28526	0.775447	NA
PLEKHJ1	29.45129	-0.01237	0.070745	-0.17488	0.861172	NA
LOC34105	0.143413	-0.01237	0.022566	-0.54825	0.583523	NA
LOC10013	0.091037	-0.01237	0.017663	-0.7003	0.483743	NA
PCDHB19P	46.82336	-0.01237	0.075776	-0.16318	0.870377	NA
RNF169	397.0632	0.012365	0.053126	0.232748	0.815957	0.937668
TTC22	0.356679	0.012363	0.031934	0.387139	0.698653	NA
SRPRB	44.17209	0.01236	0.067303	0.183645	0.854292	NA
RNF26	33.25222	-0.01235	0.07405	-0.16675	0.867568	NA
CD19	0.192629	0.012344	0.025745	0.479476	0.6316	NA
MIR3136	0.331234	-0.01234	0.032325	-0.38186	0.702562	NA
INTS6	256.6793	0.012343	0.043232	0.285499	0.775262	NA
GTF2IRD2	0.151345	0.012324	0.021659	0.569011	0.569349	NA
SMIM2-IT1	0.194005	0.012323	0.024687	0.49915	0.617674	NA
NDUFB2-A	8.609898	-0.01232	0.084312	-0.14614	0.883807	NA

ZNF347	206.2924	0.012319	0.051463	0.239386	0.810806	NA
COX5A	53.76079	-0.01231	0.075162	-0.16382	0.869871	NA
KIAA0101	0.448412	0.012296	0.037715	0.326029	0.744402	NA
CTDSPL	122.2941	-0.0123	0.0562	-0.21879	0.826817	NA
MIR4648	0.308682	0.012294	0.026425	0.465221	0.641773	NA
MIR1234	0.095119	-0.01229	0.016051	-0.76559	0.443923	NA
HIST1H1A	0.506535	-0.01229	0.040171	-0.30582	0.759739	NA
LOC28605	0.205746	0.012281	0.024773	0.49573	0.620085	NA
OAF	5.77211	0.01227	0.082945	0.147928	0.8824	NA
IRX2	30.40409	-0.01226	0.078666	-0.15587	0.876135	NA
MIR1277	0.515235	-0.01226	0.038759	-0.31636	0.751732	NA
MCIN	0.308099	0.012259	0.032131	0.381544	0.7028	NA
OR52M1	0.087997	-0.01225	0.017642	-0.69461	0.487301	NA
HSD17B7P	11.19933	0.012252	0.084938	0.144248	0.885305	NA
SPATA9	3.711586	-0.01225	0.077538	-0.15798	0.874475	NA
GATC	181.8498	0.012248	0.043414	0.282128	0.777845	NA
ST8SIA6-A	0.628555	-0.01225	0.043605	-0.28086	0.778821	NA
MIR4638	0.090097	0.012245	0.01753	0.698496	0.484867	NA
PDZK1P1	0.427943	0.012241	0.038017	0.321981	0.747467	NA
GGH	63.20769	-0.01224	0.080364	-0.1523	0.878949	NA
DIO2-AS1	1.674734	-0.01224	0.062874	-0.19464	0.845677	NA
MYH7	2.338322	0.012231	0.065498	0.186745	0.851861	NA
RXFP3	0.082426	-0.01223	0.017451	-0.70052	0.483604	NA
FKTN	118.1694	0.012217	0.076025	0.160701	0.872329	NA
LINC00842	10.40205	0.012208	0.081742	0.149346	0.881281	NA
ALG1	19.58504	0.012202	0.077333	0.157779	0.874631	NA
C1orf53	0.335655	-0.0122	0.033268	-0.36672	0.713826	NA
SNX17	95.67317	0.012183	0.070097	0.173805	0.862019	NA
PSMD11	201.8491	-0.01218	0.046965	-0.25936	0.79536	NA
KDM1A	356.3566	0.012179	0.035966	0.338612	0.734902	0.894921
LOC28463	17.64666	-0.01218	0.084539	-0.14403	0.88548	NA
OR51E2	0.104957	0.012174	0.017885	0.680691	0.496067	NA
GZMH	0.102038	0.012166	0.017799	0.683553	0.494258	NA
TNFRSF9	0.483906	0.012159	0.037705	0.322473	0.747095	NA
TRIM65	73.22817	-0.01216	0.060564	-0.20074	0.8409	NA
MEPE	0.340044	0.012155	0.028741	0.422921	0.672353	NA
ZNF208	20.09133	-0.01215	0.084266	-0.14421	0.885335	NA
RAB3GAP2	312.2877	0.012147	0.036073	0.336728	0.736322	NA
TFPI2	0.493602	-0.01214	0.035959	-0.33775	0.735555	NA
KIAA1551	541.4225	0.012142	0.053945	0.225081	0.821917	0.942126
SLC16A4	71.27858	-0.01214	0.072593	-0.16723	0.867185	NA
AKAP10	265.2663	-0.01214	0.032563	-0.37274	0.709346	NA
OR7E91P	0.080518	-0.01213	0.017444	-0.69563	0.486663	NA
CA13	20.09163	-0.01213	0.08182	-0.14826	0.882139	NA
GLTSCR1	147.1071	-0.01213	0.046536	-0.26062	0.794389	NA
CAMKMT	20.35074	0.012123	0.07773	0.155968	0.876059	NA
BPIFA3	0.098175	-0.01212	0.017906	-0.6769	0.498471	NA

TCHH	0.870181	-0.01212	0.049667	-0.24393	0.807287	NA
RD3	0.105255	0.01211	0.017769	0.681542	0.495529	NA
SLC38A8	0.23492	-0.01211	0.028851	-0.41972	0.67469	NA
DIP2C	685.3222	0.012099	0.057945	0.208797	0.834607	0.944594
GUF1	198.9841	-0.0121	0.04498	-0.26893	0.787981	NA
TACSTD2	0.09646	0.012085	0.017686	0.683314	0.494408	NA
LOC10028	0.936728	-0.01208	0.050421	-0.23955	0.810679	NA
MIR1278	0.206454	0.012077	0.024805	0.486869	0.626351	NA
CCDC18	59.29717	0.012073	0.064444	0.187347	0.851389	NA
TACR3	0.194449	-0.01207	0.023399	-0.51592	0.605908	NA
NXF4	0.138172	-0.01207	0.021281	-0.56716	0.570605	NA
NELL1	5.142233	-0.01207	0.076405	-0.15795	0.8745	NA
ZFP90	294.9158	0.012063	0.046664	0.258516	0.796009	NA
LRCOL1	0.159637	-0.01205	0.022807	-0.52834	0.597261	NA
MIR346	0.09076	-0.01205	0.014451	-0.83353	0.404548	NA
GTF3C2	111.7476	-0.01204	0.053752	-0.22406	0.822711	NA
MIR3907	0.099084	-0.01204	0.017784	-0.67674	0.498572	NA
OR51B2	1.152435	0.012034	0.055145	0.218219	0.827259	NA
MICU1	319.2942	0.012029	0.044944	0.267645	0.788973	NA
PTTG3P	1.330529	-0.01203	0.062817	-0.19143	0.848186	NA
ICMT	155.2414	0.012024	0.077706	0.154734	0.877031	NA
SLC9A5	91.52282	-0.01202	0.059986	-0.20042	0.841155	NA
FOXA1	0.097706	0.012014	0.017858	0.672778	0.501088	NA
LOC28329	0.100996	0.012011	0.0161	0.746012	0.45566	NA
HCP5	6.842338	0.012011	0.081933	0.146591	0.883454	NA
GADL1	0.244097	-0.012	0.02782	-0.43152	0.666089	NA
TCEAL7	134.7711	-0.012	0.0789	-0.15215	0.879068	NA
MIR3165	0.102183	0.012001	0.017863	0.671867	0.501668	NA
FLJ27352	2.062558	0.011999	0.068009	0.176432	0.859955	NA
EMC9	22.35928	-0.012	0.074536	-0.16095	0.872135	NA
PRSS38	0.101025	0.011996	0.017828	0.672874	0.501027	NA
TRAM1L1	16.78587	0.011994	0.08436	0.14218	0.886938	NA
MAP2K4P1	1.217152	0.011993	0.057459	0.208727	0.834661	NA
ANKK1	2.816538	-0.01199	0.070678	-0.16967	0.865271	NA
SNX14	343.7085	0.011989	0.040567	0.295522	0.767595	0.911218
MUC12	0.101666	0.011988	0.017801	0.673411	0.500686	NA
F3	45.21418	-0.01199	0.084823	-0.14131	0.887626	NA
SLTM	770.0235	0.01198	0.039871	0.300478	0.763813	0.910066
PAM16	0.645641	-0.01198	0.043406	-0.2759	0.782624	NA
PDIA3P	40.52124	-0.01198	0.078694	-0.15218	0.879047	NA
RFC3	78.71709	0.011972	0.066028	0.181313	0.856122	NA
MIR627	1.053713	0.011959	0.054832	0.218102	0.82735	NA
CHKA	107.8753	-0.01196	0.056132	-0.21302	0.83131	NA
NKX6-1	0.713927	0.011943	0.041902	0.285029	0.775622	NA
ZSCAN22	107.36	-0.01194	0.063043	-0.18942	0.849766	NA
SOX11	3.926596	-0.01194	0.075369	-0.1584	0.874142	NA
LIMD1-AS1	1.077256	-0.01194	0.056143	-0.2126	0.83164	NA

MIR2052	0.162754	-0.01193	0.022831	-0.52257	0.601272	NA
GPR179	41.95735	0.01193	0.082021	0.145451	0.884354	NA
GGNBP2	301.9461	0.011929	0.045286	0.263413	0.792232	NA
ABCB6	159.6272	-0.01193	0.07638	-0.15614	0.875922	NA
THTPA	91.42923	0.011914	0.070307	0.169452	0.865441	NA
OPRD1	0.084968	0.011896	0.017543	0.678123	0.497694	NA
LOC28644	0.084713	-0.01189	0.014359	-0.82818	0.407566	NA
TEX19	0.092089	-0.01189	0.017756	-0.66958	0.503127	NA
SPIRE1	559.5472	0.011885	0.038217	0.310973	0.755821	0.906265
OTOGL	2.039374	0.011884	0.063465	0.18726	0.851456	NA
PCSK9	88.89957	0.011874	0.084383	0.140718	0.888093	NA
ATF6	292.6696	0.011874	0.036755	0.323046	0.74666	NA
SMCR9	0.151075	0.01187	0.020047	0.592099	0.553784	NA
MIR4803	3.962582	-0.01186	0.080031	-0.14825	0.882142	NA
ZFHX2	575.8918	0.011864	0.062634	0.189421	0.849763	0.948602
LOC10028	2.62255	-0.01186	0.067668	-0.17527	0.860864	NA
KCNE2	1.267055	0.011858	0.056529	0.209759	0.833856	NA
KRT32	0.484596	0.011854	0.033765	0.351064	0.72554	NA
KLK5	0.086064	-0.01185	0.017509	-0.6769	0.498468	NA
SNX8	59.04551	-0.01185	0.063605	-0.18633	0.852186	NA
PPP2R1A	272.6683	0.01185	0.060712	0.195181	0.845251	NA
ESPL1	0.533772	0.011847	0.043323	0.273467	0.784494	NA
MFAP3L	131.437	0.011845	0.066255	0.178773	0.858116	NA
MSLN	0.774029	0.011845	0.042351	0.279676	0.779726	NA
CTXN2	11.70131	-0.01184	0.083775	-0.14138	0.88757	NA
PRSS56	0.362908	0.011831	0.032785	0.360877	0.718192	NA
TAS2R7	0.505604	0.011831	0.036189	0.326921	0.743728	NA
SDCCAG8	289.1173	-0.01183	0.049627	-0.2383	0.81165	NA
MDFI	1.447379	-0.01182	0.061833	-0.19122	0.848351	NA
SFSWAP	420.4864	0.011822	0.049895	0.23694	0.812703	0.936217
LTF	4.293415	-0.01182	0.039769	-0.29721	0.766307	NA
GRAMD1B	913.4782	0.011811	0.039486	0.299121	0.764848	0.910341
MIR1827	0.093087	0.011809	0.017658	0.668793	0.503628	NA
C6orf141	39.58337	0.011806	0.060408	0.195431	0.845056	NA
MIR212	0.14589	-0.0118	0.021379	-0.55195	0.580979	NA
GRIN1	1375.588	-0.01179	0.044489	-0.2651	0.790934	0.925343
SNORD58C	2.498471	-0.01178	0.071826	-0.16406	0.869683	NA
SVOP	136.5501	0.011777	0.077786	0.151404	0.879657	NA
ABHD2	854.5232	-0.01177	0.043494	-0.27065	0.786657	0.924656
UBE2U	0.079918	-0.01177	0.014284	-0.82381	0.410049	NA
MIR3919	0.095692	0.011763	0.017727	0.663596	0.506949	NA
DCP1B	56.87493	0.011762	0.066377	0.177206	0.859347	NA
MAPK14	226.0616	0.011762	0.042801	0.274812	0.783461	NA
TNF	0.185197	0.011759	0.023339	0.503853	0.614364	NA
ITIH1	0.816852	-0.01176	0.048439	-0.24273	0.808214	NA
MYO7A	23.02795	-0.01175	0.084802	-0.1386	0.889764	NA
PRR23C	0.143467	-0.01175	0.018494	-0.63548	0.525113	NA

F2	5.837611	-0.01175	0.082036	-0.14324	0.8861	NA
C10orf53	0.192535	0.01175	0.023371	0.502783	0.615117	NA
BROX	104.46	0.011749	0.04948	0.237457	0.812302	NA
C7orf69	0.299054	-0.01175	0.031763	-0.36981	0.711526	NA
TRIM8	320.0917	0.011743	0.055378	0.212055	0.832064	NA
EEF1DP3	110.1053	0.011739	0.067278	0.174487	0.861482	NA
LOC10012	0.188756	0.011738	0.024674	0.475734	0.634264	NA
DRGX	0.197705	0.011737	0.02003	0.585987	0.557884	NA
FRAS1	19.56916	-0.01173	0.067259	-0.17447	0.861494	NA
LOC10013	0.478798	0.011734	0.039405	0.297774	0.765876	NA
ISL2	0.098013	0.011731	0.017762	0.660478	0.508947	NA
FRMPD2P1	1.270552	0.011728	0.054872	0.213733	0.830756	NA
MIR1301	0.333489	0.011728	0.032054	0.365876	0.714458	NA
RABL6	828.435	0.011724	0.050062	0.234202	0.814828	0.937263
MADCAM1	7.512462	0.011724	0.081703	0.143496	0.885899	NA
CECR5	33.22873	0.011716	0.068169	0.171865	0.863544	NA
LPAR2	1.517787	-0.01171	0.062965	-0.18604	0.852417	NA
MIR572	0.256159	-0.01171	0.028936	-0.40454	0.685818	NA
OGDH	300.3486	-0.0117	0.048997	-0.23887	0.811204	NA
MIR192	0.28226	0.011699	0.028162	0.41544	0.67782	NA
SLC38A5	14.17549	-0.0117	0.084741	-0.13806	0.890193	NA
ZNF839	107.2696	0.011699	0.058536	0.199852	0.841597	NA
LEFTY1	1.418978	0.011693	0.061895	0.188925	0.850151	NA
FAM9A	0.105021	0.011687	0.017813	0.656099	0.51176	NA
GDNF	20.12687	0.011686	0.078867	0.148174	0.882205	NA
ADRA1B	2.071937	-0.01168	0.068471	-0.17064	0.864507	NA
LILRA1	1.127131	0.01168	0.051795	0.225512	0.821581	NA
GAPDHS	11.08789	-0.01168	0.084747	-0.13782	0.890383	NA
DLG5	316.8639	-0.01167	0.052991	-0.2203	0.825635	NA
DHX8	211.2435	0.011673	0.048588	0.240238	0.810146	NA
ZNF529	196.3809	-0.01167	0.043976	-0.26526	0.790806	NA
SPOP	238.7052	0.011661	0.04336	0.268935	0.78798	NA
BLOC1S2	61.73841	0.011659	0.065469	0.178089	0.858653	NA
ZBTB5	95.36126	-0.01165	0.061621	-0.18909	0.85002	NA
ITSN1	930.0516	0.011639	0.032849	0.354333	0.723089	0.890244
RPS26P11	5.340264	-0.01163	0.082687	-0.14069	0.888116	NA
HIST1H3G	0.07945	-0.01163	0.014202	-0.819	0.412785	NA
NTN1	36.68514	-0.01163	0.080191	-0.14498	0.884725	NA
SHISA3	1.807129	-0.01162	0.063212	-0.1838	0.854174	NA
POU6F2	2.706652	-0.01162	0.072648	-0.15991	0.872955	NA
HDLBP	861.3383	0.011617	0.036215	0.320772	0.748383	0.901327
ASZ1	0.448134	-0.01161	0.032371	-0.3587	0.71982	NA
DNAJC7	267.1273	-0.01159	0.039173	-0.2958	0.767385	NA
MIR639	0.083932	0.011585	0.017533	0.660752	0.508771	NA
FCRLA	0.091314	-0.01158	0.017842	-0.64899	0.516344	NA
ATP5J	97.82074	-0.01157	0.065302	-0.17722	0.859336	NA
FGF5	345.094	-0.01157	0.067272	-0.17203	0.863414	0.953967

LETMD1	65.14103	0.011563	0.075429	0.153294	0.878167	NA
OR1L1	0.28172	0.011562	0.030489	0.37922	0.704525	NA
MIOS	137.2025	0.011559	0.048863	0.236554	0.813003	NA
KDM3B	513.4605	-0.01155	0.0388	-0.29774	0.765901	0.910638
C10orf82	1.630436	-0.01155	0.060862	-0.18981	0.84946	NA
LRAT	3.549935	0.011549	0.069691	0.165721	0.868377	NA
MTF2	236.0158	0.011548	0.043967	0.262655	0.792816	NA
NXPE1	2.1433	0.011547	0.064347	0.179452	0.857583	NA
MIR181C	0.275914	0.011547	0.030519	0.378355	0.705167	NA
AQPEP	3.166664	-0.01155	0.062232	-0.18554	0.852804	NA
RAB15	523.3102	0.011544	0.06971	0.165605	0.868468	0.957242
OR13C2	0.092834	0.011535	0.017813	0.647572	0.517262	NA
DUSP12	46.19852	0.01153	0.067173	0.171647	0.863715	NA
PDZD2	385.7673	-0.01153	0.067639	-0.17046	0.864649	0.954865
RAD1	122.5407	0.011528	0.057509	0.200465	0.841117	NA
SNORD115	21.39402	-0.01153	0.082941	-0.13899	0.88946	NA
GLT25D1	147.0548	-0.01153	0.059956	-0.19223	0.847564	NA
LINC00476	150.0027	0.011522	0.071194	0.161846	0.871428	NA
NXPE2	0.864476	0.011522	0.05184	0.222266	0.824107	NA
DENND4B	246.3566	0.011514	0.052101	0.221001	0.825092	NA
LOC44100	20.72232	-0.01151	0.078831	-0.14604	0.883888	NA
ANO8	232.993	-0.01151	0.057781	-0.19918	0.842119	NA
CR1L	0.083946	0.011509	0.01753	0.656492	0.511508	NA
IARS2	111.9617	0.011503	0.05501	0.209115	0.834359	NA
GPC6	26.94014	0.011496	0.082887	0.138689	0.889696	NA
ZMYND8	689.2233	-0.01149	0.066571	-0.17266	0.862916	0.953883
TNIP3	1.270488	-0.01149	0.041441	-0.27715	0.781662	NA
CPSF3L	388.9414	0.011485	0.064674	0.177582	0.859051	0.95169
C1orf123	37.08551	-0.01148	0.074262	-0.15464	0.877107	NA
ALS2CR8	115.7389	-0.01147	0.071486	-0.1605	0.872488	NA
KRTAP3-3	0.076591	-0.01147	0.014104	-0.81332	0.416037	NA
VCX3B	0.091902	0.011471	0.017654	0.649744	0.515857	NA
AMY2A	0.604789	-0.01146	0.036533	-0.31369	0.753755	NA
MSANTD3	34.37503	-0.01146	0.065538	-0.17482	0.861222	NA
TCF15	1.154342	0.011451	0.05615	0.203942	0.838399	NA
CEBPE	0.150075	-0.01145	0.02266	-0.50526	0.613374	NA
IL8	0.858145	0.011449	0.038523	0.297198	0.766315	NA
MIR5188	0.183117	0.011447	0.024636	0.464645	0.642186	NA
CAP1	168.2959	-0.01145	0.05459	-0.20967	0.833922	NA
SMYD4	108.6868	-0.01144	0.05726	-0.19987	0.841579	NA
MLN	0.290468	-0.01144	0.032524	-0.35181	0.724979	NA
TSLP	0.492629	-0.01144	0.041402	-0.2763	0.782316	NA
DLX5	0.082158	-0.01144	0.017569	-0.65111	0.514977	NA
LOC37519	1.205066	0.011438	0.055414	0.206418	0.836465	NA
DLX1	0.09732	0.011437	0.017746	0.644492	0.519256	NA
SLC38A11	3.06984	-0.01143	0.075256	-0.15194	0.879231	NA
PIP5K1P1	7.217132	-0.01143	0.084709	-0.13499	0.892623	NA

OR51Q1	0.664301	0.011427	0.045818	0.249398	0.803053	NA
CXCL6	0.095713	0.011426	0.017691	0.645892	0.518349	NA
MTG1	141.9729	0.011422	0.051617	0.221288	0.824868	NA
MMP12	0.098798	0.011417	0.017782	0.642077	0.520823	NA
GPX5	0.090675	0.011392	0.017715	0.643081	0.520172	NA
ONECUT2	78.79639	-0.01139	0.083269	-0.13681	0.891184	NA
GDI2	378.5854	-0.01139	0.059866	-0.19029	0.849085	0.948602
EHD2	55.87028	0.01139	0.08004	0.142301	0.886843	NA
OCM	1.468471	-0.01139	0.05975	-0.19059	0.848846	NA
C6orf222	0.090794	0.011384	0.017666	0.64438	0.519329	NA
QRFP	2.220532	-0.01138	0.069468	-0.16387	0.869837	NA
TMEM220	1.433112	0.011383	0.059991	0.189744	0.84951	NA
FBXW2	280.1825	0.011382	0.0532	0.21395	0.830586	NA
OR2F1	0.089935	0.01138	0.0177	0.642924	0.520273	NA
SLC1A2	1708.175	-0.01138	0.074743	-0.15224	0.878994	0.959988
MIR500A	0.203063	-0.01137	0.024704	-0.4604	0.645229	NA
CAPN8	0.418066	0.011374	0.033744	0.337048	0.73608	NA
LRRC37B	176.9955	0.011372	0.058892	0.193103	0.846878	NA
HYMAI	7.424227	0.011371	0.078738	0.144418	0.88517	NA
MIR3942	0.325172	-0.01137	0.031226	-0.36413	0.715762	NA
CLDN7	1.971077	-0.01137	0.068779	-0.16528	0.868722	NA
WRB	95.34008	-0.01137	0.059546	-0.1909	0.848601	NA
UNQ6975	0.094855	0.011365	0.01775	0.640301	0.521977	NA
MMP23B	0.901282	-0.01136	0.047139	-0.24102	0.80954	NA
C1QTNF3-	1.164555	0.011361	0.056169	0.20227	0.839706	NA
ROPN1	0.07123	-0.01136	0.013934	-0.81509	0.415021	NA
ZDHHC24	11.90357	0.011353	0.084799	0.133886	0.893492	NA
HLA-J	12.54153	0.011351	0.084153	0.134884	0.892703	NA
CAHM	9.973404	0.011342	0.084391	0.1344	0.893086	NA
C21orf91-	0.31932	0.011339	0.029715	0.381606	0.702754	NA
IBTK	376.9373	0.011334	0.052491	0.215927	0.829045	0.944594
ZP3	5.391747	0.011334	0.073049	0.155156	0.876698	NA
METTL21C	3.875619	-0.01133	0.079556	-0.14245	0.886728	NA
SLC39A13	97.6975	0.011329	0.05544	0.204343	0.838085	NA
LOC64455	9.098323	-0.01132	0.084892	-0.13337	0.893904	NA
SNORD78	0.611807	-0.01132	0.045047	-0.25126	0.801612	NA
KLKP1	0.18884	0.01131	0.020156	0.56109	0.574736	NA
GBE1	57.13138	0.011309	0.076309	0.148203	0.882183	NA
LGR4	193.0241	-0.0113	0.050879	-0.22211	0.824227	NA
HPS3	63.82512	0.0113	0.057424	0.196784	0.843997	NA
GAN	230.7813	0.011299	0.046611	0.242402	0.808469	NA
PAICS	163.3732	0.011297	0.051881	0.217749	0.827625	NA
SNORD1A	0.368824	0.011296	0.034731	0.325233	0.745005	NA
TTC27	54.26541	-0.01129	0.071595	-0.15775	0.87465	NA
SRSF3	306.4719	0.011294	0.053854	0.209716	0.833889	NA
TADA2B	163.3658	-0.01128	0.050623	-0.22288	0.823627	NA
ST6GALNA	30.12503	0.011273	0.082381	0.136845	0.891153	NA

MIR132	0.518908	0.011273	0.03645	0.30926	0.757124	NA
LOC10050	0.067366	-0.01126	0.013973	-0.80572	0.420406	NA
LINC00598	1.673652	-0.01126	0.063258	-0.17797	0.858748	NA
MIR33B	0.143649	0.011252	0.021134	0.532416	0.594438	NA
UQCRFS1	39.80758	0.011249	0.082283	0.136716	0.891255	NA
LOC10012	16.88038	0.011247	0.081815	0.137474	0.890656	NA
FNDC9	89.26328	-0.01124	0.061786	-0.18197	0.855605	NA
GRP	0.132658	-0.01124	0.02122	-0.5295	0.596459	NA
TMEM155	0.067481	-0.01123	0.013857	-0.81063	0.417581	NA
ASAH2B	2.600807	0.011227	0.07463	0.15044	0.880418	NA
KLHDC7B	0.156314	-0.01122	0.02155	-0.52084	0.60248	NA
APOPT1	42.85165	-0.01122	0.070549	-0.15907	0.873613	NA
LOC72839	70.39721	0.011222	0.066247	0.169391	0.865489	NA
INVS	221.6246	-0.01121	0.044161	-0.25391	0.799565	NA
OR2H2	14.00824	-0.01121	0.082771	-0.13544	0.892262	NA
POU5F1P3	0.702867	-0.01121	0.046714	-0.23991	0.810399	NA
MIR1908	1.410599	-0.01121	0.058419	-0.19183	0.847878	NA
C5AR2	0.233396	0.011202	0.026409	0.42416	0.671449	NA
HSD17B10	18.30853	-0.0112	0.076988	-0.1455	0.884318	NA
RNU6-19	0.444726	-0.0112	0.037188	-0.30121	0.763257	NA
EGFL6	0.170755	0.011197	0.018244	0.613749	0.539381	NA
PPAPDC1B	108.7266	0.011171	0.058889	0.189702	0.849543	NA
HEBP1	33.98264	-0.01117	0.076513	-0.14596	0.883953	NA
SNORD115	14.48044	0.011164	0.084424	0.132238	0.894796	NA
LAMA3	17.8127	0.011162	0.084222	0.132527	0.894568	NA
OR10A4	0.839726	-0.01116	0.047894	-0.23298	0.815775	NA
LOC10050	0.135623	0.011157	0.021025	0.530664	0.595651	NA
IRF1	51.15564	0.011157	0.078699	0.141765	0.887266	NA
LRRK1	59.5065	0.011149	0.076608	0.145532	0.884291	NA
LCN9	0.294109	0.011148	0.02923	0.381401	0.702906	NA
C21orf7	4.090632	0.011135	0.07519	0.148097	0.882266	NA
UPK2	0.340556	-0.01113	0.031162	-0.35731	0.720859	NA
R3HDM2	609.3782	0.011126	0.050893	0.218617	0.826948	0.944098
MIR4424	0.181558	-0.01113	0.020214	-0.55036	0.58207	NA
ELL3	30.24891	-0.01112	0.078053	-0.14246	0.886718	NA
ATRIP	63.38486	-0.0111	0.065851	-0.16861	0.866106	NA
ABCC10	116.1692	0.011102	0.06603	0.16814	0.866473	NA
MAP3K15	3.871554	0.011099	0.065089	0.170521	0.8646	NA
LOC64465	11.45865	0.011091	0.084626	0.131057	0.89573	NA
KRT25	0.150106	-0.01109	0.021418	-0.51772	0.604657	NA
MIR412	0.533786	0.011086	0.04194	0.264337	0.79152	NA
JRKL-AS1	0.093175	-0.01108	0.015862	-0.69876	0.484701	NA
MRPS18C	36.37749	-0.01108	0.06753	-0.16408	0.869667	NA
PSMC3	128.7169	0.011079	0.057739	0.191876	0.847839	NA
CREBL2	244.7769	-0.01108	0.051163	-0.21651	0.828592	NA
MOXD1	0.533456	-0.01108	0.040666	-0.27237	0.785334	NA
MIR497HG	17.53168	0.011075	0.083265	0.133006	0.894189	NA

VEZT	431.9843	0.011071	0.033229	0.333178	0.739	0.895927
MIR4653	0.083484	0.011071	0.017584	0.629596	0.528959	NA
COL21A1	106.8267	-0.01107	0.06803	-0.16271	0.870751	NA
RNF135	15.16584	-0.01107	0.083439	-0.13265	0.894467	NA
GSX2	0.128503	0.011065	0.0209	0.529435	0.596504	NA
VSX1	111.2443	0.011062	0.072918	0.151711	0.879415	NA
MIR4511	2.050199	0.011062	0.069377	0.159455	0.873311	NA
RCOR1	121.474	-0.01106	0.054939	-0.20134	0.840434	NA
PIF1	0.694232	-0.01106	0.040411	-0.27366	0.784346	NA
CC2D1B	163.5065	0.011057	0.054447	0.203079	0.839073	NA
SNX32	34.94805	-0.01106	0.084093	-0.13148	0.895396	NA
NSA2	83.24678	0.011049	0.066268	0.16673	0.867583	NA
MIR124-1	3.986029	0.011048	0.077818	0.141976	0.887099	NA
VENTXP7	3.472253	0.011046	0.07195	0.153521	0.877988	NA
GNAS-AS1	0.775434	-0.01105	0.046871	-0.23565	0.813701	NA
UBQLN3	0.772233	0.01104	0.047398	0.232923	0.815821	NA
TMEM256	7.289185	-0.01104	0.083392	-0.13233	0.894726	NA
SEC23B	97.81023	0.011034	0.064516	0.171033	0.864198	NA
RPTOR	285.4373	0.011034	0.04252	0.259497	0.795251	NA
PPP1R3C	19.35645	0.011033	0.082998	0.132934	0.894246	NA
MIR548AC	1.735818	-0.01103	0.066529	-0.16583	0.868292	NA
VPS4A	355.4114	0.011029	0.041886	0.263299	0.79232	0.926009
IKZF4	72.17943	-0.01103	0.055456	-0.19884	0.842391	NA
ZMYM3	160.4993	-0.01102	0.052252	-0.21099	0.832898	NA
C6orf58	0.385623	-0.01102	0.036384	-0.30297	0.761912	NA
LOC64424	1.921547	0.011015	0.062025	0.17759	0.859045	NA
ANO10	106.3708	-0.01101	0.047732	-0.23075	0.817513	NA
HNRNPA1	94.62366	-0.01101	0.051046	-0.21576	0.829177	NA
CD226	4.729985	0.01101	0.080594	0.136611	0.891338	NA
C19orf69	0.277032	0.011009	0.030489	0.361069	0.718048	NA
C9orf114	144.1892	0.010999	0.070647	0.155688	0.876279	NA
LOC91450	5.317232	-0.011	0.080899	-0.13593	0.891875	NA
MAPT-AS1	25.28999	-0.01099	0.084753	-0.12963	0.896862	NA
MMP8	0.403141	-0.01099	0.022435	-0.48968	0.624359	NA
MIR670	0.357173	-0.01098	0.029133	-0.37699	0.706179	NA
LOC10050	21.63873	-0.01098	0.082978	-0.13235	0.894707	NA
PMCHL2	0.231441	0.01098	0.02641	0.415746	0.677596	NA
BMP3	9.532852	-0.01098	0.082907	-0.13239	0.894672	NA
MND1	2.438666	0.01097	0.070017	0.156677	0.8755	NA
S100G	2.072557	-0.01097	0.069086	-0.15872	0.87389	NA
HBS1L	361.0507	-0.01096	0.045584	-0.24044	0.809987	0.935621
SELENBP1	11.69764	-0.01096	0.084935	-0.12904	0.897324	NA
ZNF625-ZN	2.7709	0.010946	0.075909	0.144192	0.885349	NA
UCP3	31.10295	-0.01093	0.078891	-0.1386	0.889766	NA
ADCK4	57.60723	-0.01093	0.068478	-0.15967	0.873143	NA
DTL	0.557845	0.010934	0.041012	0.266592	0.789783	NA
FCHO2	270.7639	-0.01093	0.049189	-0.2222	0.824155	NA

LINC00446	0.141408	0.010929	0.018135	0.602667	0.54673	NA
PCOLCE2	0.681711	0.010921	0.044307	0.246487	0.805305	NA
LOC10050	0.168104	-0.01091	0.021623	-0.50455	0.613877	NA
LOC64236	21.28928	-0.0109	0.078811	-0.13829	0.890014	NA
POU5F1B	0.083898	-0.01089	0.015778	-0.69022	0.490053	NA
LOC15018	0.091904	-0.01089	0.015879	-0.68563	0.492945	NA
POU3F2	44.92673	0.010876	0.080902	0.134434	0.893059	NA
MIR379	2.075384	0.010864	0.068495	0.158608	0.873978	NA
CAPN12	5.499433	0.010856	0.080878	0.134232	0.89322	NA
HNRNPU	2713.042	-0.01085	0.026786	-0.40515	0.685369	0.868465
KCNH4	1.119847	-0.01085	0.055567	-0.19528	0.845172	NA
ETV5	82.81951	0.010851	0.084938	0.127753	0.898345	NA
SUPT5H	602.0009	-0.01084	0.040495	-0.26765	0.788965	0.925102
CLDN15	23.83145	0.010827	0.074382	0.145554	0.884274	NA
ACOX1	195.6784	-0.01082	0.046205	-0.23427	0.814779	NA
MCF2L2	304.9764	-0.01082	0.044583	-0.2427	0.808239	NA
ZNF727	66.09938	0.010818	0.078234	0.138283	0.890017	NA
LONP1	248.778	-0.01082	0.041374	-0.26144	0.793751	NA
HEATR5A	103.0125	-0.01082	0.055361	-0.19538	0.845095	NA
STOX1	48.68824	0.010808	0.079864	0.135334	0.892348	NA
DNAJB1	280.8048	0.010808	0.081913	0.131945	0.895028	NA
HMGA1	80.31648	0.010805	0.06356	0.169991	0.865017	NA
KRT8	6.666127	-0.0108	0.081106	-0.13319	0.89404	NA
EEF2	2478.08	-0.0108	0.066758	-0.16179	0.87147	0.958659
PARP16	60.0864	0.010796	0.05866	0.184047	0.853977	NA
MIR1266	0.525257	0.010793	0.04105	0.262921	0.792611	NA
MSRB1	14.79152	-0.01078	0.082186	-0.13119	0.895628	NA
IL9	0.317295	0.01078	0.02799	0.385154	0.700123	NA
ENDOV	43.23719	0.010779	0.071103	0.151591	0.87951	NA
TBC1D10B	150.6605	0.010772	0.051448	0.209376	0.834155	NA
GLRX	109.5543	-0.01077	0.068492	-0.15723	0.875065	NA
TRIM14	16.04338	-0.01077	0.079358	-0.13565	0.892094	NA
OPRK1	24.00114	0.010761	0.067933	0.158409	0.874135	NA
TNFAIP8L1	60.81578	-0.01076	0.068197	-0.15777	0.874637	NA
C20orf24	8.564324	-0.01076	0.084927	-0.12669	0.899188	NA
ELOVL1	26.03461	-0.01076	0.077629	-0.13857	0.889789	NA
MUC22	0.169592	0.010757	0.020428	0.526563	0.598497	NA
LOC10065	2.098448	0.010755	0.070003	0.153634	0.877898	NA
MIR4764	0.176614	0.010754	0.024594	0.437281	0.661908	NA
MIR603	0.16315	-0.01075	0.02161	-0.49739	0.618916	NA
APOBR	3.574251	0.010748	0.073822	0.145593	0.884243	NA
SOX1	11.48121	0.010744	0.083964	0.127955	0.898184	NA
PRSS22	1.932466	0.010742	0.065751	0.16337	0.870227	NA
LINC00630	54.63644	0.010738	0.06008	0.178728	0.858151	NA
LOC44070	0.387209	0.010727	0.034659	0.309505	0.756938	NA
CEACAM1	23.2126	-0.01073	0.080833	-0.13269	0.894442	NA
ZNF658	24.54169	0.010723	0.082631	0.129771	0.896747	NA

AHI1	975.7187	-0.01071	0.059526	-0.17998	0.85717	0.951001
LCNL1	10.75574	0.01071	0.084078	0.127384	0.898636	NA
UPK1B	7.693748	0.010699	0.079526	0.134537	0.892978	NA
ODF3	4.730502	-0.01069	0.082798	-0.1291	0.89728	NA
LOC10050	8.136115	-0.01069	0.08208	-0.13021	0.896397	NA
DEFB109P	0.83458	0.010678	0.049952	0.213761	0.830733	NA
CHRNA7	1.936397	-0.01068	0.061495	-0.17363	0.862157	NA
SNORA72	5.681872	0.010674	0.075555	0.141281	0.887648	NA
FOXC1	25.78008	0.010673	0.084161	0.126811	0.89909	NA
MMAB	49.11112	-0.01067	0.070941	-0.15041	0.880439	NA
ZIC2	952.2466	0.01067	0.053884	0.198021	0.843029	0.946541
U2AF1	155.231	-0.01066	0.054211	-0.19671	0.844053	NA
LIPJ	2.853794	0.010662	0.073347	0.145368	0.88442	NA
MIR4472-2	0.140174	0.010659	0.021107	0.50499	0.613566	NA
LYG1	27.44563	0.010656	0.082107	0.129788	0.896734	NA
FAM57A	79.72521	-0.01065	0.068104	-0.15644	0.875683	NA
GALK2	108.4825	-0.01065	0.047193	-0.22573	0.821409	NA
EIF4EBP3	4.919682	0.010652	0.082456	0.129187	0.897209	NA
SPTBN2	1651.277	-0.01065	0.055846	-0.19067	0.848781	0.948602
SPON2	58.51074	0.01064	0.081884	0.129945	0.89661	NA
RPN2	134.5983	0.010635	0.047704	0.222945	0.823579	NA
LOC38964	0.683381	0.010635	0.043231	0.246008	0.805676	NA
FBXL3	350.8235	0.010633	0.044235	0.240368	0.810045	0.935621
OR56A1	0.206292	-0.01063	0.027069	-0.39275	0.694507	NA
LOC10050	30.88446	0.010629	0.084814	0.125319	0.900271	NA
PGLYRP1	15.56728	-0.01062	0.083834	-0.12669	0.899186	NA
MANSC4	4.360252	0.01062	0.08121	0.130777	0.895952	NA
PPP5C	227.6251	0.010615	0.036424	0.291426	0.770726	NA
ATN1	1660.3	0.010602	0.03689	0.287395	0.77381	0.915715
CRYBB1	0.197107	-0.0106	0.026051	-0.40685	0.684121	NA
BICD1	784.5972	-0.0106	0.043819	-0.24186	0.80889	0.935326
PLAGL2	77.95092	0.010589	0.06097	0.173667	0.862127	NA
C3orf33	29.19932	-0.01059	0.071609	-0.14786	0.88245	NA
LOC55064	38.48053	-0.01058	0.06907	-0.15315	0.878278	NA
SPAG7	83.03511	-0.01056	0.067798	-0.15575	0.876232	NA
APBA2	653.1489	-0.01055	0.035664	-0.29596	0.767263	0.911218
KRTAP5-11	0.228334	-0.01055	0.027826	-0.37907	0.704636	NA
MIR495	0.601871	-0.01054	0.043717	-0.24119	0.809409	NA
KIAA1143	64.978	-0.01053	0.071849	-0.14662	0.883433	NA
COMT	171.7147	0.010529	0.053936	0.195218	0.845222	NA
MTERFD3	142.1653	0.010528	0.044072	0.23889	0.811191	NA
FBXO34	131.4517	0.010526	0.048624	0.216483	0.828611	NA
MIR5191	0.566012	-0.01052	0.040443	-0.26015	0.794745	NA
LOC39236	0.190045	0.010521	0.023395	0.4497	0.652927	NA
SOAT1	73.49264	-0.01052	0.05583	-0.1884	0.850567	NA
SF1	1190.213	-0.01051	0.038569	-0.27252	0.785222	0.924391
CCDC102A	4.088885	0.010511	0.070818	0.148417	0.882014	NA

ANP32D	0.455426	0.010507	0.040495	0.259477	0.795267	NA
SMIM6	3.702009	0.010497	0.079049	0.132797	0.894354	NA
PXMP2	17.77306	-0.01049	0.080142	-0.13093	0.895829	NA
ZNF449	47.45574	-0.01049	0.080791	-0.12987	0.896673	NA
KCNK6	2.91051	-0.01049	0.073572	-0.1426	0.886608	NA
TRPV4	2.293241	0.010489	0.061699	0.170005	0.865006	NA
MRPS7	50.47879	-0.01047	0.070526	-0.14842	0.882014	NA
OR1L3	0.459446	-0.01047	0.033055	-0.31663	0.751524	NA
SLC25A16	63.40525	0.010464	0.069367	0.150844	0.880099	NA
MIR1225	0.555769	-0.01045	0.040428	-0.2586	0.795942	NA
LOC10013	3.047178	-0.01045	0.07566	-0.13816	0.890114	NA
ARHGAP19	0.306335	-0.01045	0.03255	-0.32104	0.74818	NA
MIR4429	0.15539	-0.01045	0.021537	-0.48508	0.627623	NA
IL31	0.26032	-0.01045	0.030108	-0.34699	0.728599	NA
LOC10050	44.01897	0.010444	0.073605	0.141896	0.887162	NA
POT1	65.27231	0.010443	0.063356	0.164826	0.869081	NA
C1orf86	106.8032	-0.01044	0.067276	-0.15517	0.876684	NA
AOC3	4.551947	-0.01044	0.075942	-0.13746	0.890664	NA
OSBPL2	596.7555	0.010438	0.038752	0.269342	0.787667	0.924656
GRAP2	0.152493	0.010437	0.021336	0.489169	0.624722	NA
CCNY	147.9328	0.010428	0.049689	0.209857	0.833779	NA
ANKRD46	94.36858	0.010428	0.064669	0.161246	0.8719	NA
PPP1R18	37.88517	0.010425	0.080661	0.129242	0.897166	NA
C2orf53	0.061999	-0.01042	0.011901	-0.87571	0.381188	NA
TRIM56	14.91322	0.01042	0.083764	0.124399	0.900999	NA
B4GALT1	75.18166	-0.01042	0.056695	-0.18379	0.854177	NA
LOC10012	51.95339	-0.01042	0.075635	-0.13777	0.890425	NA
HEMK1	40.92606	-0.01042	0.079192	-0.13152	0.895364	NA
SH2B3	132.8759	-0.01041	0.059245	-0.17576	0.860482	NA
SLC10A2	0.184398	-0.01041	0.023398	-0.44504	0.656289	NA
OAS2	12.0688	0.010408	0.084829	0.122692	0.902351	NA
SCARNA27	4.65866	-0.0104	0.082404	-0.12627	0.899522	NA
GRM2	5.845871	0.010403	0.082929	0.125441	0.900175	NA
HDAC11	48.87561	-0.0104	0.071777	-0.1449	0.884792	NA
TLR8-AS1	0.159905	-0.0104	0.021527	-0.48309	0.629033	NA
SNORD4B	1.401676	-0.01039	0.059664	-0.17415	0.861747	NA
TPRG1-AS	0.231691	0.010381	0.02842	0.365277	0.714904	NA
FGF3	15.07379	-0.01038	0.084441	-0.12293	0.902161	NA
ZC3H10	31.00527	0.010378	0.072484	0.143171	0.886155	NA
RNASE13	0.299961	-0.01038	0.030731	-0.33764	0.735634	NA
SNORD115	6.16593	-0.01037	0.076375	-0.13581	0.891975	NA
CHEK2	2.622404	0.010367	0.074206	0.139706	0.888892	NA
FANCB	35.87847	0.010361	0.073359	0.141242	0.887679	NA
HTA	0.38055	-0.01036	0.033125	-0.31275	0.754471	NA
SNORD53	1.898521	-0.01036	0.061741	-0.16776	0.866769	NA
TRIM47	30.24496	-0.01035	0.081679	-0.12675	0.899137	NA
BTLA	1.270052	-0.01035	0.054491	-0.18998	0.849325	NA

RNF114	138.0605	-0.01035	0.047686	-0.21706	0.828163	NA
NAPEPLD	343.7507	0.01035	0.03456	0.29948	0.764574	0.910341
RLN2	0.141009	0.01035	0.021093	0.490688	0.623647	NA
TINF2	74.50517	-0.01035	0.057327	-0.18054	0.85673	NA
LARP4B	338.0293	-0.01035	0.039892	-0.25937	0.795346	0.928334
TULP1	0.092781	0.010342	0.015957	0.648131	0.5169	NA
ABHD6	114.6145	0.010333	0.069875	0.147877	0.88244	NA
LOC10028	0.143228	-0.01033	0.022587	-0.45739	0.64739	NA
CCPG1	157.7873	-0.01033	0.051638	-0.20006	0.841435	NA
POLR2E	106.8153	-0.01032	0.053219	-0.19393	0.846231	NA
RABAC1	45.73823	0.01032	0.066459	0.155281	0.8766	NA
TBC1D28	0.432752	0.010314	0.035855	0.287656	0.77361	NA
CLIP2	533.6068	0.010313	0.052927	0.194847	0.845513	0.947593
C17orf82	0.189686	0.010312	0.023379	0.441099	0.659141	NA
MIR4717	0.139364	0.010308	0.021049	0.489732	0.624324	NA
DKC1	106.487	-0.0103	0.043959	-0.23439	0.81468	NA
MUC16	0.223876	0.0103	0.028378	0.362973	0.716625	NA
ITGB1	192.5673	0.010298	0.050904	0.202303	0.83968	NA
SOX6	46.42644	-0.0103	0.077076	-0.13359	0.893725	NA
KCNJ18	1.365217	-0.0103	0.060624	-0.16983	0.86514	NA
WNT8B	5.645172	-0.0103	0.076051	-0.13538	0.892312	NA
LYRM7	135.3478	-0.01029	0.061803	-0.16647	0.867785	NA
GPHA2	10.64201	-0.01029	0.084778	-0.12134	0.90342	NA
MIR30C1	0.436032	-0.01029	0.039601	-0.25977	0.795043	NA
KCNA2	188.2028	0.010287	0.069826	0.147322	0.882878	NA
PNPLA8	169.0547	0.010285	0.053861	0.190959	0.848558	NA
RNF5P1	0.746156	0.010284	0.049877	0.206189	0.836644	NA
PPIP5K1	783.3019	-0.01028	0.057123	-0.18003	0.857131	0.951001
CCDC106	40.86953	0.010283	0.072819	0.141211	0.887703	NA
MAPK3	145.8175	0.010278	0.055532	0.185082	0.853164	NA
REP15	0.683427	0.010276	0.041523	0.247475	0.804541	NA
LY6H	0.681428	-0.01027	0.045675	-0.22487	0.82208	NA
LOC64174	0.147096	0.010264	0.019866	0.516668	0.605388	NA
TBC1D14	100.3467	0.010261	0.053927	0.190269	0.849099	NA
CHRNA4	28.89418	-0.01025	0.084441	-0.12141	0.903364	NA
PATL2	1.128516	-0.01025	0.053512	-0.19149	0.848142	NA
NEK1	334.534	-0.01025	0.040554	-0.25265	0.800536	0.930701
LOC10050	0.415789	0.010245	0.034613	0.295987	0.76724	NA
TMEM8B	123.9234	0.010241	0.058151	0.176119	0.860201	NA
GUSBP9	0.258101	0.010241	0.025153	0.407162	0.683889	NA
GOLM1	545.3904	0.010232	0.046523	0.219929	0.825927	0.944098
SMARCA4	1170.411	0.010224	0.031123	0.328491	0.74254	0.896989
MYCNOS	0.093487	-0.01022	0.016082	-0.63551	0.525094	NA
MIR4434	1.349726	0.010217	0.057065	0.179045	0.857902	NA
FGFR1OP	108.9018	0.010196	0.051401	0.198358	0.842765	NA
OR6F1	0.64524	-0.01019	0.043282	-0.2355	0.813822	NA
CACNB2	387.3589	0.010192	0.031277	0.325867	0.744525	0.898871

SLC35F2	3.199413	-0.01019	0.077467	-0.13156	0.895333	NA
RNU6-83	1.941384	0.01019	0.059973	0.169909	0.865081	NA
ASAP1	214.1033	-0.01019	0.049794	-0.2046	0.837884	NA
C22orf23	24.27457	-0.01018	0.076773	-0.13266	0.89446	NA
LOC10050	0.093106	-0.01018	0.015971	-0.63771	0.523665	NA
LINC00251	0.057917	-0.01018	0.011765	-0.86563	0.386693	NA
HEATR8-T	1.624297	-0.01018	0.063394	-0.16062	0.872393	NA
PLIN4	32.23333	0.01018	0.083006	0.122642	0.902391	NA
PAFAH2	28.91861	0.010176	0.073696	0.138081	0.890177	NA
ARHGAP8	0.066356	0.010175	0.014412	0.705989	0.480195	NA
HIST1H2AJ	0.146162	0.010174	0.019812	0.513517	0.607589	NA
DACH1	19.44439	0.010172	0.083881	0.121266	0.903481	NA
DNASE2B	0.682024	0.010171	0.041837	0.24312	0.807912	NA
LOC28576	0.626595	-0.01017	0.037505	-0.27107	0.786339	NA
MIR5691	0.122794	0.010166	0.020852	0.487511	0.625896	NA
NT5DC1	52.84178	0.010164	0.068482	0.148415	0.882015	NA
SLC9A3	53.56853	-0.01016	0.076338	-0.13311	0.894106	NA
SH3RF3	6.746315	0.01016	0.081673	0.124402	0.900997	NA
SHBG	2.4994	0.010153	0.073758	0.137656	0.890512	NA
LATS2	90.61394	-0.01015	0.080481	-0.12613	0.89963	NA
MIR1913	0.291908	0.010151	0.031407	0.3232	0.746544	NA
CERS1	32.46224	-0.01015	0.071758	-0.14141	0.887546	NA
LOC10013	29.47997	-0.01014	0.080734	-0.12561	0.900043	NA
ZNF326	227.2738	0.010136	0.050603	0.200301	0.841245	NA
C13orf35	0.13345	0.010127	0.021011	0.481995	0.629809	NA
LOC44097	0.232594	-0.01013	0.023601	-0.42904	0.667897	NA
AOC4	0.104299	0.010123	0.016095	0.628964	0.529373	NA
TTC4	6.818998	0.010122	0.084227	0.120172	0.904347	NA
MIR5702	0.063636	0.010118	0.014372	0.704007	0.481428	NA
C16orf90	1.902052	0.010116	0.066856	0.151307	0.879733	NA
MOGS	61.57901	0.010103	0.064627	0.156326	0.875776	NA
SNORA75	5.380827	-0.0101	0.077852	-0.12973	0.896778	NA
IK	364.1583	-0.0101	0.040434	-0.24978	0.802754	0.931519
HSPA14	89.28792	0.010099	0.056661	0.178227	0.858545	NA
MCM2	37.99544	-0.0101	0.077227	-0.13075	0.89597	NA
IRX1	25.36199	-0.0101	0.084145	-0.11997	0.904503	NA
AZU1	0.065225	0.010093	0.014355	0.70315	0.481962	NA
SLC17A8	0.221765	-0.01009	0.027176	-0.37136	0.710368	NA
AKNAD1	1.838437	-0.01009	0.065927	-0.15307	0.878342	NA
C1orf194	1.583128	0.010089	0.062806	0.160643	0.872374	NA
MSH5	0.147164	-0.01008	0.021403	-0.47117	0.637517	NA
PPM1H	759.0067	0.010084	0.050374	0.200189	0.841333	0.946541
PABPC1P2	0.065002	0.010077	0.014343	0.702587	0.482313	NA
REXO2	113.5481	0.010075	0.067579	0.149084	0.881487	NA
APOC4-AP	0.314413	-0.01007	0.032566	-0.30927	0.757117	NA
LOC44056	0.06455	0.010068	0.014337	0.70227	0.482511	NA
SERPINA6	0.055967	-0.01007	0.011697	-0.86061	0.389451	NA

SPACA3	0.064798	0.010063	0.014333	0.702067	0.482637	NA
RALYL	463.3149	0.010055	0.03787	0.265522	0.790607	0.925343
PPP2R4	145.9894	-0.01005	0.059982	-0.1676	0.866901	NA
ZNF671	136.5607	0.010051	0.045714	0.219873	0.82597	NA
MIR502	0.055712	-0.01005	0.011687	-0.85985	0.38987	NA
MIR378E	0.132864	0.010042	0.019661	0.510754	0.609524	NA
FAM184A	423.6413	0.010027	0.039442	0.254232	0.799316	0.930701
THAP9	25.63736	-0.01002	0.074612	-0.13429	0.893174	NA
SDHB	79.84713	0.010011	0.067126	0.149143	0.881441	NA
DDX24	789.6305	0.010006	0.051903	0.192787	0.847126	0.948463
LINC00511	0.106341	0.010005	0.016183	0.618249	0.536411	NA
PINK1	392.8597	-0.01	0.054096	-0.18489	0.853313	0.951001
ZNF397	184.0249	0.010001	0.054449	0.183686	0.85426	NA
SUCNR1	0.063932	0.01	0.014288	0.69985	0.484021	NA
PPAT	60.2814	0.009991	0.059408	0.168174	0.866446	NA
ZNF146	313.6647	0.009987	0.037527	0.266126	0.790142	NA
PSME4	211.3364	-0.00998	0.048121	-0.20747	0.835639	NA
NEK2	0.310376	-0.00998	0.029318	-0.34038	0.733573	NA
PCDHB15	45.09559	0.009978	0.074165	0.134536	0.892978	NA
BCAR1	400.2043	0.009977	0.052558	0.189834	0.849439	0.948602
MIR320C2	0.091656	-0.00998	0.016103	-0.61952	0.535577	NA
AGPAT3	368.5723	-0.00997	0.049434	-0.20159	0.84024	0.946431
GBAP1	22.0153	0.009964	0.082649	0.120558	0.904041	NA
HAUS6	105.6927	-0.00996	0.051206	-0.19457	0.845726	NA
GPR158-A	3.803934	-0.00996	0.079661	-0.12505	0.900482	NA
C1orf110	0.46391	0.009961	0.039328	0.253275	0.800056	NA
MIRLET7D	34.94755	-0.00996	0.070659	-0.1409	0.887951	NA
C11orf48	52.62614	0.009955	0.061738	0.161243	0.871902	NA
OR51V1	1.555256	-0.00994	0.058144	-0.17102	0.864206	NA
PRDX6	145.8454	-0.00994	0.06723	-0.14789	0.882428	NA
JMJD7	78.10284	-0.00994	0.069982	-0.14206	0.887033	NA
GPR39	1.414394	0.009933	0.057378	0.17312	0.862557	NA
SAMD7	0.142803	-0.00993	0.019847	-0.50034	0.616833	NA
DENND1C	3.61646	-0.00993	0.079423	-0.125	0.900523	NA
IL18BP	118.4424	0.009927	0.073652	0.134785	0.892782	NA
EDC4	316.1676	-0.00993	0.043054	-0.23056	0.817657	NA
ZNF862	192.851	0.009925	0.04544	0.218417	0.827104	NA
PRKACA	297.8233	0.009922	0.059163	0.167697	0.866822	NA
ABHD12B	4.38924	0.009919	0.072305	0.137182	0.890887	NA
RNY4	0.053584	-0.00991	0.011606	-0.85385	0.393188	NA
ZNF780A	142.2706	0.009889	0.044115	0.224159	0.822634	NA
ANTXR1	226.2276	0.009887	0.072413	0.136537	0.891397	NA
MIR5004	0.720079	0.009884	0.043301	0.228262	0.819442	NA
PTCRA	0.098439	0.009877	0.016169	0.610857	0.541294	NA
ZNF157	36.17752	0.009874	0.06785	0.145521	0.8843	NA
C3orf43	0.198231	-0.00986	0.026076	-0.37826	0.705236	NA
CCDC129	26.18815	-0.00986	0.08485	-0.11617	0.907514	NA

CD3EAP	11.49905	0.009851	0.083771	0.11759	0.906393	NA
SCAF11	796.0373	-0.00985	0.030383	-0.32404	0.745907	0.899616
SNORA84	0.085288	-0.00984	0.01591	-0.61829	0.536382	NA
PNMA3	59.11364	-0.00984	0.081178	-0.12117	0.903553	NA
C1QL2	3.794235	0.009834	0.076171	0.12911	0.89727	NA
SNORD19B	2.099149	0.009826	0.069062	0.142286	0.886854	NA
LINC00457	0.209861	-0.00982	0.023288	-0.42176	0.673198	NA
FBXL21	2.140797	0.009822	0.067199	0.146159	0.883796	NA
PMPCA	103.0102	0.009821	0.046158	0.212768	0.831508	NA
SLC12A4	110.8785	0.009814	0.076792	0.127796	0.89831	NA
FAM215A	0.057909	0.00981	0.014153	0.693152	0.488214	NA
MIR181A1	0.396464	0.009808	0.036962	0.265361	0.790732	NA
MIR3941	0.991552	0.009788	0.050991	0.191948	0.847783	NA
OSBPL10	28.38036	-0.00979	0.084814	-0.1154	0.908131	NA
PHF8	193.4763	0.00978	0.044996	0.217354	0.827933	NA
DYNC2LI1	56.13049	0.009775	0.075693	0.129146	0.897242	NA
LOC10050	0.098022	0.009775	0.016142	0.605592	0.544786	NA
LOC10013	0.80563	-0.00977	0.04529	-0.2158	0.829146	NA
RNU6-67	0.312041	-0.00977	0.030752	-0.31779	0.750647	NA
GNGT2	0.630188	0.00976	0.044528	0.219196	0.826497	NA
ZAK	70.8096	-0.00976	0.076244	-0.12796	0.898184	NA
SLC10A6	0.266957	-0.00975	0.026778	-0.36422	0.715696	NA
AICDA	0.060183	0.009746	0.014107	0.690874	0.489645	NA
CT62	0.652586	0.009746	0.047259	0.206235	0.836607	NA
SNORD80	5.020449	0.009745	0.083534	0.116664	0.907126	NA
C22orf34	2.659966	-0.00974	0.070678	-0.13787	0.890339	NA
NR1I2	0.933793	0.009741	0.053755	0.181217	0.856197	NA
LOC44060	24.25137	0.009737	0.077813	0.125129	0.900422	NA
MIR197	0.76442	0.009736	0.043204	0.225357	0.821701	NA
MAD2L1B	15.82457	-0.00973	0.079989	-0.12167	0.903164	NA
FUCA2	43.3011	-0.00973	0.06961	-0.13979	0.888828	NA
DCTPP1	13.25522	-0.00973	0.084826	-0.11469	0.90869	NA
LOC73122	0.225663	-0.00972	0.027205	-0.35745	0.720756	NA
ST7L	143.6578	-0.00972	0.047609	-0.20424	0.838166	NA
MIR149	0.222566	0.00972	0.027506	0.353385	0.723799	NA
PRICKLE2-	0.67052	0.009713	0.046794	0.207573	0.835563	NA
RHD	13.86193	0.009713	0.070003	0.138753	0.889645	NA
NFX1	374.4291	0.009712	0.046482	0.208932	0.834501	0.944594
SLC24A2	541.4386	0.009709	0.083765	0.115907	0.907726	0.971866
FGF21	0.059558	0.009694	0.01407	0.689012	0.490815	NA
CD24	26.2061	0.009694	0.029083	0.333335	0.738881	NA
C12orf10	44.36191	0.009689	0.065324	0.148321	0.882089	NA
GP9	0.094244	-0.00969	0.016057	-0.60318	0.546387	NA
LINC00521	0.128388	-0.00968	0.018328	-0.5281	0.597432	NA
CELSR2	373.3753	0.009678	0.063365	0.152741	0.878602	0.959988
UNCX	195.5052	0.009676	0.055705	0.173703	0.862099	NA
LRP2BP	334.5217	0.009673	0.06591	0.146759	0.883322	NA

IL11	6.226066	0.00967	0.082522	0.117183	0.906715	NA
PEX5	133.3624	-0.00967	0.044921	-0.21525	0.829574	NA
MAN1C1	224.2747	0.009669	0.051596	0.187396	0.85135	NA
TNC	2.545391	-0.00967	0.062985	-0.1535	0.878	NA
ZNF638	2485.568	0.009665	0.046358	0.208488	0.834848	0.944594
GPR52	38.39178	-0.00966	0.069471	-0.13911	0.889366	NA
TMPO	325.1468	-0.00966	0.050169	-0.19249	0.847362	NA
KRT15	0.276741	-0.00965	0.030555	-0.31598	0.752019	NA
LOC44249	0.451721	0.009652	0.03582	0.269446	0.787586	NA
LINC00494	0.390012	-0.00965	0.036626	-0.26344	0.792211	NA
TSSK1B	0.60016	0.009646	0.043268	0.222926	0.823593	NA
GPI	679.4397	-0.00964	0.060928	-0.15826	0.874248	0.959476
ZDHHC8	111.9829	-0.00964	0.060969	-0.15805	0.87442	NA
MIR3691	0.313735	0.009633	0.032913	0.292674	0.769771	NA
CBFB	58.06274	-0.00963	0.067143	-0.14339	0.88598	NA
LOC10013	0.090651	-0.00963	0.016045	-0.59999	0.548513	NA
MKRN1	259.1793	-0.00963	0.048899	-0.19685	0.843942	NA
DPPA4	0.090315	-0.00962	0.016127	-0.59665	0.55074	NA
RPL31P11	0.113187	-0.00962	0.019224	-0.50046	0.616751	NA
PNOC	0.769672	0.009619	0.049188	0.195567	0.844949	NA
LOC25739	8.29595	0.00961	0.084911	0.113172	0.909894	NA
ZNF485	15.87561	0.009609	0.08392	0.114496	0.908844	NA
NLRP4	0.056771	0.009602	0.014003	0.685695	0.492905	NA
MIR34A	0.214529	-0.0096	0.02714	-0.35359	0.723644	NA
BCL9	251.3946	0.009593	0.045297	0.211788	0.832273	NA
LINC00443	0.099461	0.009593	0.01612	0.595086	0.551786	NA
LCE5A	0.049044	-0.00959	0.011419	-0.83996	0.400928	NA
BET3L	0.056842	0.009591	0.013995	0.685311	0.493148	NA
CBWD6	23.16472	-0.00958	0.083833	-0.1143	0.908997	NA
TAF11	105.8079	-0.00958	0.055333	-0.17311	0.862566	NA
LINC00488	0.055727	0.009576	0.013984	0.684751	0.493501	NA
RMDN3	132.6699	-0.00957	0.044462	-0.21532	0.829521	NA
EIF3L	458.9727	-0.00957	0.071665	-0.13358	0.893736	0.967349
WDR77	49.87637	-0.00957	0.070094	-0.13655	0.891386	NA
NDUFS7	95.26726	0.00957	0.052251	0.183148	0.854682	NA
SCG5	215.6232	0.009569	0.075043	0.12751	0.898537	NA
VMAC	17.51956	-0.00957	0.079231	-0.12077	0.903873	NA
ACAT1	115.749	0.009568	0.049918	0.191679	0.847994	NA
LAIR2	0.054948	0.009567	0.013978	0.684423	0.493708	NA
OTUD7A	185.1094	-0.00955	0.054423	-0.17554	0.860655	NA
MIR4672	0.485476	0.009553	0.039287	0.243152	0.807888	NA
TACC2	329.5772	0.009552	0.042646	0.223975	0.822777	NA
PARK7	224.2603	-0.00954	0.06906	-0.1382	0.890083	NA
LOC28597	0.276694	0.009541	0.028877	0.330402	0.741096	NA
SUB1	255.4325	0.009532	0.071847	0.13267	0.894454	NA
GAL3ST2	7.340167	0.009526	0.08308	0.114659	0.908716	NA
C4orf36	0.666796	-0.00953	0.046169	-0.20631	0.83655	NA

APOM	11.03412	-0.00952	0.084938	-0.11209	0.910749	NA
ADORA2A-	26.42664	0.009518	0.081297	0.117079	0.906798	NA
MIR3154	0.05437	0.009518	0.013942	0.682666	0.494818	NA
HOXA1	0.047896	-0.00951	0.011371	-0.83634	0.402962	NA
POM121L1	0.273104	0.009509	0.02951	0.322241	0.74727	NA
PROKR1	0.082651	0.009507	0.012132	0.783572	0.433291	NA
GCNT4	1.455968	0.009499	0.058594	0.162117	0.871213	NA
RRH	6.627675	0.009482	0.083542	0.113502	0.909633	NA
UCP2	3.557027	-0.00947	0.075649	-0.12521	0.900357	NA
DHODH	56.42536	0.009463	0.081834	0.115636	0.907941	NA
ATP6V1H	139.02	-0.00945	0.065067	-0.14529	0.884484	NA
PFKP	383.7625	0.009448	0.08399	0.112489	0.910436	0.972087
MIR4297	0.148939	-0.00945	0.021417	-0.44112	0.659123	NA
SPATA31D	0.090938	0.009444	0.016075	0.587488	0.556876	NA
RABL3	81.88454	0.009442	0.071971	0.131185	0.895629	NA
ADAM15	91.84144	-0.00944	0.058929	-0.16017	0.872745	NA
OR2B11	0.305454	-0.00944	0.028992	-0.3255	0.744801	NA
ZAR1	0.468	0.009435	0.040468	0.233141	0.815652	NA
TREML3P	0.056245	0.009433	0.013881	0.679606	0.496754	NA
ZNF322	30.67073	-0.00942	0.075923	-0.12412	0.901224	NA
C3orf79	0.117719	-0.00941	0.019396	-0.48496	0.627703	NA
GFRA4	0.053372	0.009405	0.01386	0.678569	0.497411	NA
MILR1	0.530056	0.009404	0.040518	0.232093	0.816466	NA
DNAJB4	257.8515	0.009394	0.080345	0.116916	0.906926	NA
HIST1H2B	1.476136	0.009392	0.062999	0.149077	0.881493	NA
DPEP1	0.052801	0.009391	0.01385	0.678085	0.497718	NA
ZNF32-AS3	0.377458	0.009387	0.032325	0.290398	0.771512	NA
GCGR	0.217485	-0.00939	0.027159	-0.34561	0.729639	NA
CEP68	334.971	0.009384	0.05137	0.182674	0.855054	0.951001
CCNB2	3.027562	0.009383	0.076518	0.122628	0.902402	NA
LINC00314	0.055416	0.009383	0.013844	0.677782	0.49791	NA
RPE65	0.560037	0.009377	0.038011	0.2467	0.805141	NA
FLJ45445	0.978212	-0.00937	0.051768	-0.18109	0.856294	NA
TPO	0.234598	0.009369	0.023077	0.405989	0.684751	NA
REV3L	1270.661	-0.00937	0.03943	-0.23755	0.81223	0.936217
MIR920	0.423619	0.009364	0.037563	0.249285	0.80314	NA
AHSP	0.054035	0.009361	0.013827	0.676968	0.498426	NA
WNT10B	0.146958	0.009359	0.021308	0.439247	0.660483	NA
STARD4	142.2889	0.009351	0.061123	0.152985	0.87841	NA
HAO1	0.055188	0.009338	0.013811	0.676159	0.49894	NA
SLC15A2	74.30928	-0.00934	0.074087	-0.12604	0.899703	NA
WDR86-AS	0.127201	0.009337	0.02096	0.445451	0.655994	NA
ZNF205-AS	25.30174	-0.00933	0.078814	-0.11843	0.905728	NA
OASL	0.583926	-0.00933	0.042318	-0.22055	0.825441	NA
DGCR8	375.3609	-0.00933	0.066179	-0.14102	0.887856	0.964968
MIR4634	0.052805	0.00933	0.013805	0.675848	0.499137	NA
MIR581	1.655086	-0.00933	0.064271	-0.14511	0.884623	NA

ARHGEF37	116.4036	0.009325	0.068987	0.135173	0.892475	NA
VPS18	108.1967	0.009325	0.051196	0.182138	0.855474	NA
DCD	0.074847	0.00931	0.012006	0.775411	0.438097	NA
C21orf37	0.470204	0.009304	0.035786	0.259996	0.794867	NA
NDUFAF6	47.17395	0.009301	0.065977	0.140969	0.887895	NA
LOC10013	1.651548	0.009297	0.065111	0.14279	0.886456	NA
CCDC59	61.98408	-0.0093	0.064376	-0.14439	0.885191	NA
HIST1H4J	0.136119	0.009287	0.021124	0.439659	0.660184	NA
VPS13C	2033.85	0.009283	0.043769	0.212092	0.832035	0.944594
MIR3200	2.886786	-0.00928	0.075072	-0.12364	0.901602	NA
PLXDC1	55.34939	-0.00928	0.081394	-0.11397	0.909259	NA
RTP1	0.110339	-0.00927	0.019187	-0.48336	0.628841	NA
LOC10050	0.129432	0.009272	0.021003	0.441475	0.658869	NA
C7orf73	49.97398	0.009266	0.060692	0.152667	0.878661	NA
CDKN2B-A	2.095642	0.009263	0.06873	0.134773	0.892792	NA
LINC00410	0.054008	0.009255	0.01375	0.673107	0.500879	NA
TAS1R1	2.030357	-0.00925	0.064781	-0.14286	0.886402	NA
VAV1	4.222053	0.009253	0.072918	0.126899	0.89902	NA
GPRC5B	869.8005	-0.00925	0.066576	-0.13892	0.889515	0.965256
EIF3D	157.6114	0.009247	0.055416	0.166868	0.867474	NA
RIPPLY1	0.052467	0.009244	0.013742	0.672708	0.501133	NA
GPC6-AS2	0.604559	-0.00924	0.043405	-0.21295	0.831365	NA
TRIM28	224.9365	0.009242	0.048432	0.190814	0.848671	NA
CPA5	0.052393	0.009238	0.013737	0.672477	0.50128	NA
SNORD74	0.399594	0.009229	0.031823	0.290017	0.771804	NA
MCM10	1.179133	0.009228	0.057245	0.161203	0.871933	NA
UBE2NL	0.112835	0.009225	0.014411	0.640155	0.522072	NA
CBX3P2	2.135415	0.009225	0.067696	0.136273	0.891606	NA
KBTBD3	93.68484	0.009225	0.052119	0.17699	0.859516	NA
LOC34488	0.108065	-0.00922	0.019204	-0.48027	0.631033	NA
LIN28B	0.654886	0.009222	0.038715	0.238206	0.811722	NA
BHMT	1.575568	-0.00922	0.060112	-0.15341	0.878079	NA
MIR494	1.033725	-0.00922	0.054882	-0.16801	0.866578	NA
TPD52L2	240.6141	-0.00922	0.049282	-0.18709	0.851591	NA
OR5B12	0.050833	0.009219	0.013723	0.671803	0.501709	NA
HN1L	119.7089	0.009219	0.055655	0.165636	0.868443	NA
COL4A6	2.644217	-0.00922	0.071124	-0.1296	0.896884	NA
PLCXD2	9.779679	-0.00922	0.084892	-0.10858	0.91354	NA
ICK	178.9217	-0.00921	0.051635	-0.17845	0.858373	NA
FAM35BP	0.128403	0.009214	0.019609	0.469876	0.638444	NA
KRTAP16-1	0.051418	0.00921	0.013716	0.671458	0.501929	NA
INIP	45.87031	0.009209	0.065668	0.140237	0.888473	NA
FIBIN	17.62079	-0.0092	0.084659	-0.10871	0.913436	NA
PLCZ1	0.176325	0.009199	0.024251	0.379326	0.704446	NA
LEPREL1	3.616108	0.009195	0.077511	0.11863	0.905569	NA
CLGN	80.63623	-0.00919	0.080362	-0.11441	0.908915	NA
CPA6	0.051232	0.009194	0.013704	0.670863	0.502308	NA

EYA3	231.5284	-0.00919	0.040062	-0.22941	0.81855	NA
TINAG	0.050352	0.009188	0.0137	0.670662	0.502436	NA
TMEM225	0.052624	0.009186	0.013699	0.670596	0.502478	NA
STARD6	0.051967	0.009186	0.013698	0.670562	0.502499	NA
BAIAP3	8.848117	0.009185	0.08161	0.112547	0.910389	NA
FDPSL2A	4.204249	0.009181	0.081486	0.112665	0.910296	NA
TRPC2	0.12873	0.009178	0.020982	0.43743	0.661799	NA
MGC1612	0.688966	-0.00917	0.047479	-0.19304	0.846924	NA
TBX5-AS1	0.320059	0.009165	0.032	0.286399	0.774573	NA
C7orf10	4.739153	0.009161	0.082978	0.110406	0.912087	NA
MAF1	116.149	-0.00916	0.052619	-0.17409	0.861799	NA
TBC1D30	207.4375	-0.00916	0.042749	-0.21427	0.830338	NA
FHL1	181.718	-0.00916	0.057018	-0.16063	0.872386	NA
C15orf57	48.09794	0.009158	0.070396	0.130088	0.896497	NA
ABLIM1	3907.767	-0.00915	0.043436	-0.21072	0.833107	0.944594
KIF9	29.11087	0.009151	0.072388	0.12642	0.899399	NA
RECQL	47.90119	0.009151	0.064458	0.141966	0.887107	NA
LOC40113	1.160581	-0.00915	0.0541	-0.16911	0.865712	NA
E2F2	0.137032	0.009149	0.021134	0.43288	0.665102	NA
EPHB6	48.39886	-0.00914	0.077865	-0.11743	0.906516	NA
KIF9-AS1	22.99149	-0.00914	0.078031	-0.11718	0.90672	NA
CNKSR3	22.05972	0.009142	0.084862	0.107727	0.914213	NA
HDAC3	88.90323	-0.00914	0.051477	-0.17754	0.859081	NA
MTAP	61.28346	-0.00913	0.083964	-0.10873	0.913415	NA
FAM229B	55.98487	-0.00911	0.078285	-0.1164	0.907337	NA
FADS1	252.7181	-0.00911	0.06636	-0.13721	0.890866	NA
ERICH1-AS	0.222389	-0.0091	0.027198	-0.33472	0.737837	NA
DGKQ	211.0029	-0.0091	0.069184	-0.13154	0.895349	NA
LILRA6	1.604623	-0.0091	0.061338	-0.1483	0.882104	NA
MIR4326	0.241157	-0.0091	0.029179	-0.31174	0.755242	NA
B3GALT2	40.49321	-0.00909	0.070045	-0.12981	0.896713	NA
GCN1L1	428.1502	-0.00909	0.050018	-0.18178	0.855754	0.951001
FAM207A	28.61672	-0.00909	0.074419	-0.12217	0.902766	NA
SLCO3A1	145.8433	0.009088	0.057833	0.157141	0.875134	NA
TFAP2D	0.042792	-0.00909	0.011116	-0.81747	0.413657	NA
LOC64594	0.050102	0.009083	0.013622	0.666777	0.504915	NA
NWD1	29.94497	0.009067	0.083338	0.108795	0.913365	NA
AGXT2	0.139219	0.009066	0.019795	0.457997	0.646955	NA
KIF18A	1.61328	-0.00906	0.06296	-0.1439	0.885576	NA
TOPAZ1	0.113341	-0.00906	0.019236	-0.47093	0.637694	NA
RALB	87.0423	0.009053	0.063167	0.143318	0.886039	NA
PM20D2	174.0012	0.00905	0.053959	0.167722	0.866802	NA
CSTF2	60.48017	-0.00905	0.059307	-0.15259	0.878724	NA
ZC3H18	315.1691	-0.00904	0.049378	-0.18315	0.854682	NA
DCAF8L1	0.049129	0.009041	0.013591	0.66523	0.505903	NA
SNORA65	4.115768	0.00903	0.077798	0.116069	0.907598	NA
SLCO4C1	0.105147	-0.00903	0.019137	-0.47162	0.637199	NA

IGFBP4	28.07299	0.009023	0.084846	0.106348	0.915306	NA
TM4SF19-	0.271998	0.00902	0.030133	0.299336	0.764684	NA
MST1L	6.773145	0.009011	0.076488	0.117808	0.90622	NA
C4orf46	23.79156	-0.00901	0.075989	-0.11853	0.905645	NA
CCDC77	46.89126	0.009007	0.081025	0.111158	0.911491	NA
CLDN9	42.93949	-0.00901	0.080807	-0.11145	0.911259	NA
BBC3	24.67654	-0.009	0.079301	-0.11349	0.909642	NA
ZNF735	0.113927	-0.009	0.019256	-0.4673	0.640288	NA
SYDE1	10.11824	0.008998	0.084873	0.106014	0.915571	NA
KIAA1751	52.36503	0.008994	0.076438	0.117664	0.906334	NA
PSG11	10.09954	0.008987	0.083642	0.107448	0.914433	NA
GTF3C6	53.31791	-0.00898	0.065522	-0.13706	0.890987	NA
ZRANB3	45.83693	0.008976	0.065113	0.137854	0.890356	NA
LINC00264	0.106089	-0.00898	0.019168	-0.46829	0.639576	NA
MAFA	0.046064	0.008969	0.013537	0.662564	0.50761	NA
AMHR2	0.045982	0.008965	0.013534	0.662424	0.507699	NA
LINC00202	0.122487	-0.00896	0.019422	-0.46155	0.644404	NA
RASSF2	301.7326	0.008957	0.071046	0.126079	0.899669	NA
RHOH	0.149709	-0.00896	0.019906	-0.44989	0.652791	NA
LOC33997	0.896852	0.008955	0.035957	0.249051	0.803321	NA
CCR1	3.031549	-0.00895	0.060136	-0.14884	0.881683	NA
C4orf45	0.865381	-0.00895	0.047565	-0.18818	0.850739	NA
STAG3L1	9.583188	0.008949	0.056498	0.158403	0.874139	NA
DNM1	1401.659	0.008947	0.038908	0.229946	0.818134	0.939692
GAS2L2	1.413435	0.008947	0.057467	0.155685	0.876282	NA
TMEM145	281.1774	-0.00895	0.065139	-0.13733	0.890771	NA
TRIM63	0.194571	0.008941	0.021852	0.409158	0.682424	NA
VEGFC	5.013573	-0.00894	0.082262	-0.10867	0.913466	NA
RD3L	0.315494	0.008935	0.032902	0.271562	0.785959	NA
NUDT4	11.89298	-0.00893	0.084511	-0.1057	0.915824	NA
BCL6B	7.353407	-0.00893	0.08349	-0.10697	0.91481	NA
HTR3B	0.38515	-0.00893	0.032006	-0.27901	0.78024	NA
DAK	24.6849	-0.00893	0.082414	-0.1083	0.913754	NA
KIAA0355	294.296	-0.00892	0.033759	-0.26424	0.791597	NA
HESX1	0.978898	0.00892	0.051132	0.174442	0.861518	NA
ROPN1B	3.120989	0.008917	0.076213	0.117007	0.906855	NA
MIR3123	0.921467	0.0089	0.052237	0.170379	0.864712	NA
CHRM3	0.237114	0.008886	0.026434	0.336174	0.73674	NA
LOC10012	1.184932	0.008882	0.050385	0.176287	0.860068	NA
TAF4	205.3886	0.00888	0.053301	0.166599	0.867685	NA
TNFRSF6B	4.233862	0.008867	0.069634	0.127344	0.898668	NA
RAB3D	75.6816	-0.00887	0.05296	-0.16743	0.867028	NA
ST13	387.1543	-0.00887	0.057342	-0.15464	0.877107	0.959988
OR5C1	1.988327	0.008859	0.067778	0.13071	0.896004	NA
ACAD9	181.7211	0.008859	0.05201	0.170327	0.864753	NA
TAF9	89.78415	0.008854	0.052732	0.167909	0.866655	NA
MCM3	80.82849	-0.00885	0.064475	-0.13721	0.890864	NA

IRS1	272.2332	0.008845	0.060214	0.146892	0.883217	NA
MAPK15	0.175708	0.008842	0.024245	0.364699	0.715336	NA
FLJ46361	0.109552	-0.00884	0.019246	-0.45943	0.645923	NA
DOC2GP	2.675642	-0.00884	0.074383	-0.11886	0.905386	NA
ZNF295-AS	0.205935	-0.00884	0.02612	-0.33838	0.735074	NA
HPYR1	0.077443	-0.00884	0.015767	-0.5604	0.575203	NA
SPAG6	0.392812	0.008831	0.035667	0.247592	0.80445	NA
ARMC9	84.25624	-0.00883	0.053199	-0.16593	0.868211	NA
SCARNA18	1.587751	0.008827	0.063645	0.138699	0.889688	NA
ORMDL2	12.3371	0.008822	0.084897	0.103914	0.917238	NA
GUSB	68.14508	0.008821	0.063461	0.138995	0.889454	NA
FAM194B	0.163707	0.00882	0.023272	0.378981	0.704702	NA
UPK3A	0.112076	-0.00882	0.019333	-0.4562	0.648247	NA
C3orf17	160.1626	-0.00882	0.049132	-0.17945	0.85758	NA
ITGB5	58.567	0.008814	0.072786	0.121091	0.903619	NA
ANKMY2	102.6247	0.008805	0.057731	0.152515	0.878781	NA
MIR3064	58.52132	-0.00879	0.076791	-0.11451	0.908835	NA
ERLEC1	133.5725	0.008793	0.050446	0.174308	0.861623	NA
JDP2	104.8683	0.008793	0.066964	0.131306	0.895533	NA
PHYHIPL	271.2494	-0.00879	0.06164	-0.14264	0.886574	NA
TOM1L1	59.60505	-0.00879	0.07164	-0.12264	0.902396	NA
MIR135A2	0.088668	-0.00878	0.015999	-0.54905	0.582973	NA
CNTD1	13.909	-0.00878	0.084455	-0.10397	0.917195	NA
NGFR	45.44645	0.008774	0.080494	0.109008	0.913196	NA
LOC10028	0.120733	-0.00877	0.01939	-0.4525	0.65091	NA
ABT1	34.07682	-0.00877	0.067386	-0.13011	0.89648	NA
CASP5	0.451434	0.008766	0.033768	0.259583	0.795186	NA
LOC10013	15.03395	0.008762	0.08463	0.103537	0.917537	NA
CENPW	6.481222	-0.00876	0.080232	-0.10916	0.913072	NA
ITM2A	27.00886	-0.00875	0.084913	-0.10306	0.917913	NA
ASB12	0.352297	-0.00874	0.032415	-0.26973	0.78737	NA
SOLH	184.2838	-0.00874	0.063913	-0.13672	0.89125	NA
RARS2	122.9289	0.008736	0.052307	0.167016	0.867357	NA
MTRNR2L	193.913	-0.00873	0.073314	-0.11906	0.905224	NA
EIF2S2	123.1235	0.008729	0.048098	0.181477	0.855993	NA
SLC36A1	630.6715	0.008727	0.069919	0.12481	0.900674	0.970824
RAB7L1	38.14588	0.008726	0.077257	0.112953	0.910068	NA
CHD1	590.0852	-0.00872	0.04185	-0.20841	0.834913	0.944594
ADAMTS6	3.468178	0.008716	0.077952	0.111809	0.910975	NA
CALCB	1.169714	-0.00872	0.055091	-0.1582	0.874297	NA
CASZ1	15.33008	-0.00871	0.084756	-0.10281	0.918112	NA
SLC35F5	157.3561	-0.00871	0.053206	-0.16376	0.869918	NA
ACPL2	37.30546	0.008712	0.076518	0.113855	0.909353	NA
MIR505	0.118179	-0.00871	0.019397	-0.44895	0.653468	NA
ARID3C	3.773627	0.008706	0.077848	0.111834	0.910955	NA
TRPM1	1.003775	0.008702	0.044332	0.196287	0.844386	NA
DR1	157.9787	0.008697	0.060706	0.143271	0.886076	NA

BCYRN1	0.377992	-0.00869	0.034358	-0.25302	0.800254	NA
AKAP8	165.1719	-0.00869	0.056111	-0.15486	0.876934	NA
LINC00116	7.58499	-0.00869	0.084916	-0.10231	0.918507	NA
MIR455	1.250672	-0.00869	0.054335	-0.15989	0.872965	NA
FAM217B	188.4684	0.008687	0.0505	0.172013	0.863427	NA
CTTNBP2N	162.9637	0.008684	0.060583	0.143338	0.886023	NA
ZDHHC20	215.6378	0.008683	0.046678	0.186031	0.85242	NA
FAM90A1	105.7484	0.008683	0.070313	0.123483	0.901724	NA
PLXNB2	1310.196	0.00868	0.063406	0.136887	0.89112	0.965736
MIR4734	0.115362	-0.00868	0.019407	-0.44702	0.654862	NA
MET	4.475926	-0.00867	0.076622	-0.1132	0.909869	NA
CDV3	161.8984	0.008668	0.060461	0.143369	0.885999	NA
ELMOD3	52.83012	-0.00866	0.062594	-0.13833	0.889979	NA
PRDX1	175.3497	-0.00864	0.061686	-0.14014	0.888548	NA
ZNF586	174.9553	-0.00864	0.06303	-0.13712	0.890934	NA
NGFRAP1	352.7897	-0.00864	0.067503	-0.12801	0.898138	0.970091
C1orf140	0.433871	-0.00864	0.039386	-0.21939	0.826349	NA
RIIAD1	1.116463	-0.00864	0.058448	-0.14781	0.882496	NA
ANO9	0.386905	0.008631	0.034473	0.250367	0.802304	NA
ZNF503	4.840311	0.008628	0.075219	0.11471	0.908675	NA
TFF3	0.399945	-0.00863	0.035573	-0.2425	0.80839	NA
SLC35D3	0.310467	-0.00863	0.029269	-0.29472	0.768209	NA
CRHBP	0.843219	0.008622	0.049519	0.174105	0.861783	NA
ABI2	632.842	0.008619	0.038239	0.225402	0.821666	0.942126
TRAPPC11	362.0537	-0.00862	0.047178	-0.18266	0.855065	0.951001
LOC72998	0.118832	0.008615	0.019501	0.441772	0.658655	NA
RNF216	370.3187	0.008607	0.056753	0.151654	0.87946	0.959988
CCL17	0.558345	0.008604	0.038353	0.224347	0.822487	NA
UNC45B	0.108933	-0.0086	0.014563	-0.59072	0.554707	NA
RAPSN	0.410926	-0.0086	0.033156	-0.25938	0.795344	NA
CTAGE1	0.109527	-0.00859	0.019195	-0.44762	0.654425	NA
TMEM67	83.62462	0.008592	0.054927	0.15643	0.875694	NA
UBD	0.17244	0.00859	0.024217	0.354728	0.722793	NA
UBL3	281.0251	0.008589	0.047876	0.179393	0.857629	NA
PRAME	0.353592	-0.00859	0.034421	-0.24949	0.802983	NA
GALNT5	148.5041	-0.00859	0.080748	-0.10634	0.91531	NA
RAPGEF5	354.5096	0.008585	0.065377	0.13132	0.895522	0.968652
DMPK	187.7683	0.008581	0.063371	0.135417	0.892283	NA
COL18A1	182.5909	-0.00858	0.084191	-0.10188	0.918848	NA
MIR1324	0.120668	-0.00858	0.019461	-0.44063	0.65948	NA
MRPL28	46.74226	-0.00857	0.063405	-0.13518	0.892473	NA
TSR2	152.6317	-0.00856	0.058425	-0.14657	0.883473	NA
CA7	11.70885	-0.00856	0.084926	-0.10078	0.919729	NA
PHF1	120.2035	0.008541	0.063529	0.134449	0.893047	NA
DMRTA1	1.843915	0.008539	0.06551	0.130345	0.896294	NA
MAPKAPK	177.7993	0.008533	0.042647	0.20008	0.841418	NA
KSR2	837.8621	0.008528	0.040834	0.20885	0.834565	0.944594

LOC10050	1.475428	-0.00852	0.062208	-0.137	0.891028	NA
TARS2	92.005	-0.00852	0.056673	-0.1503	0.880526	NA
DDX3Y	188.5726	0.008516	0.0209	0.407452	0.683676	NA
FKBP1AP1	77.19848	-0.0085	0.077028	-0.1104	0.912096	NA
SLC7A5	169.588	-0.0085	0.077638	-0.10949	0.912812	NA
PDZD4	1142.282	-0.0085	0.046617	-0.18233	0.855327	0.951001
C8orf22	0.07349	-0.0085	0.012146	-0.69948	0.48425	NA
ZNF133	131.4549	0.008495	0.055004	0.154436	0.877266	NA
NANS	43.23658	-0.00848	0.075607	-0.1122	0.910668	NA
PAX8	0.536704	-0.00848	0.03945	-0.21499	0.829777	NA
ANXA2P1	21.97241	-0.00848	0.077733	-0.10907	0.913148	NA
AKR1B1	64.60107	0.008476	0.058001	0.146139	0.883812	NA
ZKSCAN5	144.3437	0.008468	0.063265	0.133855	0.893518	NA
IFITM5	0.087888	0.00845	0.013913	0.607353	0.543617	NA
XK	63.15712	0.008449	0.06436	0.131274	0.895559	NA
SCN7A	2.088336	-0.00845	0.058892	-0.14344	0.885941	NA
PCDH18	9.767224	0.008443	0.079106	0.106733	0.915001	NA
SESN2	30.16731	-0.00844	0.073175	-0.11529	0.908213	NA
KLF1	0.210171	-0.00844	0.026132	-0.32283	0.746826	NA
FUT8	448.5426	0.008436	0.039605	0.212995	0.831331	0.944594
RHOF	7.182379	0.008433	0.083264	0.101286	0.919324	NA
RSRC2	677.3573	-0.00843	0.039938	-0.21101	0.832878	0.944594
MIR4269	1.028438	-0.00843	0.053615	-0.15716	0.875117	NA
HDAC5	502.2796	-0.00842	0.042591	-0.19775	0.84324	0.946541
ROCK1	830.9392	0.008421	0.047554	0.177092	0.859436	0.95169
PYCRL	23.71976	-0.00842	0.075601	-0.11139	0.91131	NA
CDK13	765.0914	0.008412	0.038181	0.220316	0.825625	0.944098
EPPIN	0.454522	0.008409	0.039529	0.212725	0.831542	NA
SLC20A2	262.5989	-0.0084	0.05676	-0.14802	0.882327	NA
PCGF3	675.742	0.008401	0.052557	0.159839	0.873008	0.958954
MIR4783	0.214835	-0.0084	0.024904	-0.33714	0.736014	NA
TEX26	0.495616	0.008391	0.034911	0.240368	0.810045	NA
TPPP3	31.52688	-0.00839	0.084603	-0.09917	0.921004	NA
MEG3	7999.426	-0.00839	0.068789	-0.12191	0.902967	0.97114
AANAT	1.903573	0.008384	0.066292	0.126478	0.899353	NA
LOC28427	0.163755	0.008384	0.023002	0.364477	0.715502	NA
RFT1	73.96932	-0.00838	0.062015	-0.13517	0.892474	NA
TMEM201	69.19709	-0.00838	0.064868	-0.12918	0.897214	NA
C11orf95	103.3816	-0.00838	0.063345	-0.13226	0.894779	NA
TMEM186	11.35729	-0.00838	0.084046	-0.09966	0.920615	NA
C1orf172	4.469769	-0.00838	0.080143	-0.10451	0.916766	NA
LOC34919	0.981754	0.008372	0.041785	0.200361	0.841198	NA
TTC28	213.3581	-0.00837	0.059586	-0.14049	0.888276	NA
LOC10028	16.13427	0.008369	0.083135	0.100663	0.919818	NA
GNRHR	35.64179	0.008357	0.0843	0.099131	0.921034	NA
EPO	0.193901	0.008356	0.026326	0.317423	0.750923	NA
PLEC	2296.87	-0.00835	0.062597	-0.13338	0.893891	0.967349

SCFD2	41.92603	-0.00835	0.069997	-0.11922	0.905101	NA
ZNF219	152.9444	0.008344	0.06884	0.121214	0.903522	NA
HIST1H2B	37.69122	-0.00834	0.083541	-0.09987	0.920447	NA
LOC28464	0.260315	0.008339	0.030098	0.277068	0.781728	NA
ABCA4	2.975951	-0.00833	0.074702	-0.11157	0.911168	NA
KRT38	0.100915	-0.00833	0.019099	-0.43636	0.662578	NA
MRVI1-AS	3.176477	0.008331	0.072482	0.114943	0.90849	NA
LINC00483	0.233412	-0.00833	0.026248	-0.31737	0.75096	NA
LDLRAD1	0.287787	0.00833	0.027724	0.300472	0.763817	NA
ACAD8	118.0391	-0.00833	0.052721	-0.15798	0.874472	NA
IDO1	0.137185	0.00832	0.010075	0.825778	0.40893	NA
AGO2	264.6651	0.008316	0.04684	0.177535	0.859088	NA
C12orf40	0.831229	0.008313	0.052106	0.159539	0.873244	NA
HTATIP2	16.34656	-0.00831	0.083198	-0.09989	0.920435	NA
PCYT1A	90.14137	-0.00831	0.054013	-0.15384	0.877736	NA
CDC20B	0.115115	-0.00831	0.019297	-0.43042	0.666889	NA
ZNF678	111.0251	-0.0083	0.051931	-0.1599	0.87296	NA
XKR5	0.111695	-0.0083	0.01919	-0.43266	0.665258	NA
TRHDE	238.14	-0.0083	0.062905	-0.13195	0.895023	NA
LSM11	51.05976	0.008296	0.064145	0.129336	0.897092	NA
HHIPL1	42.59527	-0.00829	0.071039	-0.11676	0.907047	NA
SCAP	205.8064	0.008292	0.053061	0.156264	0.875825	NA
TMCO5B	0.128591	0.008286	0.017927	0.46219	0.643945	NA
MPZL2	8.892812	0.008283	0.076525	0.108245	0.913802	NA
BUD13	94.3471	0.00828	0.048108	0.172108	0.863353	NA
VSIG1	0.299996	0.008277	0.029177	0.283693	0.776645	NA
SLC41A3	119.034	0.00827	0.054027	0.153078	0.878337	NA
AIF1L	117.8046	0.008268	0.082525	0.100183	0.920199	NA
PRDM7	0.801622	-0.00826	0.048794	-0.16938	0.865497	NA
NCKAP5	85.54888	-0.00826	0.075009	-0.1101	0.912328	NA
SLC1A7	2.71345	0.008253	0.057978	0.142339	0.886812	NA
TEFM	67.829	0.008251	0.062509	0.131995	0.894988	NA
OR2L5	3.56916	0.008243	0.051004	0.161614	0.87161	NA
LOC72950	2.716233	0.008237	0.072565	0.113512	0.909625	NA
TRIM45	12.31713	0.008226	0.084549	0.097296	0.922491	NA
LINC00534	0.32973	0.008224	0.031996	0.257037	0.79715	NA
SNORD4A	6.870726	0.008224	0.084773	0.097009	0.92272	NA
LOC64354	0.833904	0.008214	0.044625	0.184076	0.853954	NA
HMX1	0.908809	-0.00821	0.051556	-0.1592	0.873512	NA
LGALS9C	0.18891	0.0082	0.024376	0.336379	0.736585	NA
DHX37	65.85998	0.0082	0.065746	0.124718	0.900747	NA
RASA1	661.3363	0.008199	0.032514	0.252164	0.800914	0.930701
NSUN5P2	0.673018	0.008197	0.034282	0.239106	0.811023	NA
RARA	68.70878	-0.0082	0.062237	-0.1317	0.895218	NA
SLC23A3	6.529619	-0.00819	0.078882	-0.10389	0.917258	NA
IKBKAP	560.1312	-0.00818	0.049405	-0.16558	0.868491	0.957242
LRRC46	26.62002	0.00818	0.084489	0.09682	0.922869	NA

MPP5	250.3641	0.008175	0.045392	0.180099	0.857075	NA
LOC10021	109.8987	-0.00817	0.071149	-0.11488	0.908542	NA
CYP7A1	1.340504	-0.00817	0.048465	-0.16854	0.866155	NA
MIR668	0.215647	-0.00816	0.026165	-0.31201	0.755031	NA
DOPEY2	513.3804	0.008163	0.050803	0.160676	0.872349	0.958695
PHF23	51.86241	-0.00816	0.066796	-0.1222	0.902741	NA
KCNG3	0.265796	0.008155	0.030122	0.270719	0.786607	NA
TCEAL3	161.0167	-0.00815	0.058162	-0.14004	0.888625	NA
FOXH1	1.630426	0.008144	0.061053	0.1334	0.893877	NA
LOC10050	1.84153	-0.00814	0.063199	-0.12885	0.897475	NA
VLDLR	76.79898	-0.00814	0.074259	-0.10958	0.912739	NA
WNK2	795.721	0.008136	0.045122	0.180313	0.856907	0.951001
TPI1	350.2704	-0.00813	0.070557	-0.11528	0.908225	0.971866
C17orf74	0.123939	-0.00813	0.01802	-0.45126	0.651803	NA
MIR140	0.252366	-0.00813	0.026532	-0.30646	0.759251	NA
GDF9	1.188726	-0.00813	0.053919	-0.15077	0.880158	NA
PARG	68.50377	0.00812	0.05934	0.136843	0.891155	NA
GCM1	0.265458	0.008117	0.029175	0.278205	0.780855	NA
CELA1	0.137163	0.00811	0.019844	0.408682	0.682773	NA
ACAP3	795.2443	0.008105	0.058794	0.137851	0.890358	0.965372
OR2M1P	0.262695	-0.0081	0.029173	-0.27777	0.781188	NA
C7orf13	12.15685	-0.0081	0.084909	-0.09542	0.923984	NA
ZBTB42	2.048216	0.008098	0.063724	0.127078	0.898878	NA
RNF138	104.5577	0.008094	0.051928	0.15587	0.876135	NA
DCTD	36.84711	0.008088	0.070335	0.114995	0.908449	NA
DSG1	0.258068	0.008087	0.02919	0.27705	0.781742	NA
MIR3153	0.111674	-0.00809	0.019323	-0.41853	0.675563	NA
B3GAT3	39.56945	0.008086	0.064839	0.12471	0.900753	NA
ACTN4	365.8272	0.008085	0.045798	0.176531	0.859877	0.95169
BRF2	24.9664	0.008083	0.07235	0.111717	0.911048	NA
AGPAT2	4.940688	-0.00808	0.076645	-0.10544	0.916028	NA
KLK6	11.98462	-0.00807	0.082083	-0.09836	0.921643	NA
IFNL2	0.260674	0.008067	0.030108	0.267928	0.788755	NA
SLC35F1	35.02077	-0.00806	0.081901	-0.09845	0.921576	NA
HTR6	0.116631	-0.00806	0.019405	-0.41542	0.677833	NA
ZADH2	241.7885	0.00806	0.056595	0.142407	0.886759	NA
C19orf26	17.83575	0.008055	0.081407	0.098946	0.921181	NA
CYB5R4	96.44439	0.008055	0.054951	0.14658	0.883464	NA
USP14	180.422	0.008045	0.04953	0.162429	0.870968	NA
MIR4701	0.548023	-0.00804	0.043232	-0.186	0.852443	NA
LOC10024	0.32222	-0.00804	0.027876	-0.28843	0.773021	NA
WWTR1-A	0.4251	0.008038	0.036039	0.223029	0.823513	NA
DKK4	22.91839	0.008033	0.084382	0.095199	0.924156	NA
ABHD12	191.0515	0.00803	0.054866	0.146365	0.883633	NA
FAM83F	0.38803	0.008019	0.031903	0.251352	0.801542	NA
XAF1	37.22974	-0.00802	0.084934	-0.0944	0.92479	NA
PAX2	8.944519	0.008013	0.083987	0.095411	0.923988	NA

C7orf57	0.55493	-0.00801	0.041698	-0.19211	0.847659	NA
EMID1	17.02742	-0.008	0.08322	-0.09616	0.923394	NA
GRHL2	0.114464	-0.008	0.017879	-0.44756	0.654474	NA
LOC10028	0.168809	-0.008	0.02418	-0.3308	0.740792	NA
AVIL	90.27574	-0.008	0.079669	-0.10037	0.920047	NA
ZNF606	90.15265	-0.00799	0.05921	-0.13496	0.892646	NA
LOC44002	1.110518	-0.00799	0.052467	-0.1522	0.879026	NA
LOC10013	0.108598	-0.00798	0.01925	-0.4146	0.678438	NA
CLUH	253.7848	0.007975	0.050039	0.159387	0.873364	NA
LOC40132	192.6315	0.007958	0.070631	0.112674	0.910289	NA
SNORD115	6.844622	0.007958	0.079253	0.100409	0.92002	NA
STT3B	172.2383	0.007957	0.044992	0.176846	0.859629	NA
FLJ40288	0.123983	-0.00796	0.019586	-0.40622	0.684582	NA
FBN3	0.94742	0.007951	0.044797	0.177492	0.859122	NA
EPN1	229.2475	0.007951	0.057593	0.138054	0.890198	NA
BLOC1S5	0.664508	0.007949	0.04318	0.184096	0.853938	NA
B4GALT7	40.54446	-0.00795	0.069701	-0.11402	0.90922	NA
IQCK	131.8767	-0.00795	0.064792	-0.12264	0.902391	NA
ANKH	700.4753	0.007944	0.033665	0.235972	0.813455	0.936217
PLA2G7	39.34765	0.007944	0.083195	0.095484	0.92393	NA
ZSCAN5B	0.25608	-0.00794	0.030099	-0.26374	0.791977	NA
PTGS1	5.874769	-0.00793	0.079976	-0.09921	0.920972	NA
GAB4	0.118294	-0.00793	0.019437	-0.408	0.683275	NA
PIP5K1B	211.9325	-0.00791	0.060629	-0.13049	0.896181	NA
BCL2L2	208.9855	0.007902	0.053688	0.147184	0.882987	NA
DDR1	117.8006	-0.0079	0.057917	-0.13642	0.891487	NA
XKR9	1.947923	-0.0079	0.066585	-0.11866	0.905544	NA
UBA5	186.3544	-0.0079	0.044913	-0.17585	0.860413	NA
CD96	0.186254	0.007887	0.024354	0.323842	0.746057	NA
TTC9B	64.42696	0.007887	0.063515	0.124174	0.901178	NA
CHML	46.56358	-0.00789	0.078715	-0.10018	0.920205	NA
OR3A3	0.487338	0.007878	0.031441	0.250557	0.802157	NA
ANKRD30B	0.165441	-0.00787	0.024149	-0.32609	0.744355	NA
OCIAD1	308.1988	-0.00787	0.050044	-0.15735	0.874965	NA
DIO3OS	0.322262	0.00787	0.031996	0.245964	0.80571	NA
TTC19	394.8126	-0.00787	0.037128	-0.21184	0.832233	0.944594
LOC10012	3.566921	0.00785	0.077636	0.101114	0.91946	NA
HIST1H2BF	13.34846	0.007847	0.084896	0.092431	0.926355	NA
TOPBP1	200.2414	0.007846	0.03856	0.203462	0.838774	NA
FAM166B	5.156425	0.007845	0.083314	0.094158	0.924984	NA
MRPL15	59.54688	-0.00784	0.0646	-0.12143	0.903352	NA
DPY19L2P	38.38042	-0.00784	0.076704	-0.10225	0.918558	NA
BSDC1	273.7042	-0.00784	0.053353	-0.1469	0.883207	NA
LOC38933	1.685426	-0.00784	0.061158	-0.12812	0.898051	NA
SNORD115	31.84763	0.007835	0.084354	0.092878	0.926	NA
PQLC2	34.25262	-0.00783	0.074088	-0.10568	0.915839	NA
ATP13A2	194.8467	0.007829	0.04793	0.163337	0.870253	NA

MIR2681	8.581241	-0.00783	0.084898	-0.0922	0.926538	NA
BPIFA1	0.126313	0.007826	0.019651	0.398261	0.690438	NA
PRB1	0.575947	-0.00782	0.040131	-0.19498	0.845408	NA
GLRA4	5.797157	0.007825	0.078423	0.099775	0.920523	NA
ZNF474	1.220108	0.007824	0.056138	0.139365	0.889161	NA
FAM111B	0.581777	-0.00782	0.037986	-0.20589	0.836877	NA
MIR577	0.196497	0.007817	0.024444	0.319797	0.749122	NA
SLC17A1	0.118345	0.007813	0.019544	0.399757	0.689335	NA
GJA5	2.964095	-0.00781	0.068773	-0.1136	0.909556	NA
NUP35	62.74742	0.007811	0.05779	0.13517	0.892478	NA
MYL4	0.120842	-0.00781	0.017909	-0.436	0.662835	NA
SNORD25	7.339262	0.007808	0.083966	0.092992	0.92591	NA
PFN1P2	37.75412	0.007801	0.08333	0.093612	0.925418	NA
ZNF630	58.87626	-0.0078	0.079819	-0.09771	0.92216	NA
MBD2	285.5741	0.007798	0.047642	0.163676	0.869986	NA
CSRP2BP	123.1724	0.007789	0.055531	0.14027	0.888447	NA
SENP3	8.356888	0.007789	0.084608	0.092056	0.926653	NA
BRAP	156.3091	-0.00778	0.040487	-0.19228	0.847521	NA
GZMA	0.182791	-0.00778	0.02342	-0.33229	0.739669	NA
LOC15409	0.308633	-0.00778	0.033632	-0.23139	0.817013	NA
MIR4640	0.179778	0.00778	0.024289	0.32029	0.748749	NA
YIPF2	40.20137	-0.00778	0.0725	-0.10726	0.914581	NA
LOC10105	0.275239	-0.00777	0.030163	-0.25753	0.796766	NA
GALM	14.35392	-0.00777	0.084481	-0.09193	0.926754	NA
ZNF536	386.4954	0.007762	0.049591	0.15653	0.875615	0.959956
ZMYM1	46.17614	0.007759	0.069235	0.112068	0.91077	NA
IFT172	385.3637	-0.00776	0.055675	-0.13935	0.889177	0.965256
ZNRD1	12.5324	0.007757	0.082863	0.093609	0.92542	NA
LINC00242	4.118133	0.007752	0.076266	0.10165	0.919035	NA
NARFL	56.42137	0.00775	0.06247	0.12406	0.901267	NA
FTLP10	0.223538	0.007744	0.02641	0.29322	0.769354	NA
BCL2L15	21.09181	-0.00774	0.082172	-0.09419	0.924954	NA
ZNF462	668.9752	-0.00774	0.043419	-0.17823	0.858545	0.95169
WASH5P	0.485799	0.007737	0.034363	0.225154	0.821859	NA
DENND1A	181.1245	-0.00773	0.044831	-0.17248	0.863058	NA
TK1	12.6273	0.007728	0.08371	0.092322	0.926443	NA
ARHGAP42	22.30983	-0.00773	0.083707	-0.09232	0.926445	NA
C4BPB	1.274047	0.007723	0.061028	0.126555	0.899292	NA
FNDC7	3.353476	-0.00772	0.074082	-0.10421	0.917001	NA
ADAMTS3	0.515525	0.007712	0.04101	0.188056	0.850833	NA
ZFPM1	74.57828	-0.00771	0.060325	-0.12782	0.898291	NA
LSM4	47.83772	-0.00771	0.077455	-0.0995	0.920738	NA
ZDHHC11	142.6085	-0.0077	0.084583	-0.09108	0.927425	NA
USP34	1858.7	0.007702	0.030373	0.253564	0.799832	0.930701
BOD1L1	1900.838	-0.00769	0.04129	-0.18624	0.852256	0.950916
C15orf48	0.259927	-0.00769	0.030106	-0.25541	0.798409	NA
SNORA70B	0.86883	-0.00768	0.052898	-0.14517	0.884578	NA

RAB10	278.2459	-0.00768	0.045417	-0.16901	0.865788	NA
C8orf69	0.085337	0.007672	0.013959	0.549621	0.58258	NA
LIPN	0.074689	0.007672	0.012526	0.612496	0.540209	NA
MYO19	89.93718	-0.00767	0.063364	-0.12105	0.903648	NA
TMEM11	35.31882	-0.00767	0.06901	-0.11112	0.911518	NA
SLC4A1AP	228.3841	0.007666	0.036376	0.210731	0.833097	NA
ARHGAP9	79.54028	0.007665	0.071257	0.107564	0.914342	NA
LOC28366	7.812515	0.007663	0.08015	0.095611	0.923829	NA
C9orf72	489.8763	-0.00766	0.069057	-0.11095	0.911655	0.972087
SDF2L1	7.37818	0.007662	0.081957	0.093484	0.925519	NA
SSU72	209.0668	0.007658	0.047374	0.161646	0.871585	NA
MIR3916	0.925648	-0.00766	0.052131	-0.14689	0.883219	NA
MYO9A	963.3987	0.007651	0.031166	0.245488	0.806079	0.933897
AP3S2	11.03414	-0.00764	0.084657	-0.09028	0.928066	NA
TAS2R9	1.095559	0.007642	0.055605	0.137432	0.890689	NA
CYP24A1	0.250371	0.007632	0.030076	0.25376	0.799681	NA
PHLPP1	200.12	0.007627	0.049411	0.154366	0.877321	NA
HIST1H3J	0.117283	-0.00763	0.019442	-0.39223	0.69489	NA
CDAN1	133.694	-0.00763	0.066368	-0.11489	0.908531	NA
ANAPC10	58.79443	0.007623	0.057158	0.133375	0.893897	NA
GBF1	413.5742	-0.00762	0.031526	-0.24176	0.808964	0.935326
C19orf10	23.62568	0.007615	0.076623	0.099387	0.920831	NA
DEK	539.015	-0.00761	0.048143	-0.15813	0.874351	0.959476
KRTAP5-7	0.604135	0.007611	0.044393	0.171439	0.863878	NA
MNT	102.521	-0.00761	0.05417	-0.14049	0.88827	NA
LGALS3BP	44.73033	-0.00761	0.081351	-0.09349	0.925512	NA
CNRIP1	118.8122	0.007599	0.059854	0.12696	0.898972	NA
CLEC3B	19.277	-0.0076	0.084793	-0.08959	0.928616	NA
ATP2A1	10.18538	-0.00759	0.08375	-0.09058	0.927827	NA
MTCH1	197.7751	-0.00758	0.059608	-0.12721	0.89877	NA
RALGPS2	130.4863	0.007583	0.05423	0.139827	0.888797	NA
RAC3	39.33403	0.007573	0.073808	0.102608	0.918274	NA
ANXA7	211.4891	-0.00756	0.055843	-0.13535	0.892332	NA
JTB	71.19752	0.007555	0.065868	0.114705	0.908679	NA
ANK2	4904.206	0.007551	0.037252	0.202689	0.839378	0.946431
FBXL5	484.877	-0.00755	0.038424	-0.19651	0.844214	0.946607
CHTF8	57.98612	0.007545	0.056288	0.134045	0.893367	NA
STOML2	45.84652	0.007545	0.065946	0.114409	0.908914	NA
RNF40	514.8256	0.007543	0.035613	0.211815	0.832251	0.944594
XYLT2	113.0475	-0.00754	0.0474	-0.15911	0.873579	NA
PHF5A	40.12846	0.00754	0.069766	0.108078	0.913934	NA
SNORD115	57.16914	-0.00754	0.067095	-0.11237	0.910534	NA
CCAR1	632.0579	-0.00753	0.037129	-0.20287	0.839235	0.946431
ADCYAP1	12.12382	0.007514	0.084639	0.088771	0.929264	NA
SLC13A1	0.256108	-0.00751	0.029175	-0.25738	0.796889	NA
ELP6	71.6397	-0.0075	0.061863	-0.12129	0.903464	NA
FITM2	19.80061	0.0075	0.077401	0.096897	0.922808	NA

ALS2CL	89.83951	-0.0075	0.08035	-0.09333	0.925639	NA
RPSAP9	1.943018	-0.00748	0.068583	-0.10901	0.913191	NA
MLH1	157.3348	0.007474	0.062574	0.119438	0.904929	NA
FAM178A	633.4617	0.007469	0.036496	0.204652	0.837844	0.946431
LPP	353.9654	0.007462	0.048416	0.154125	0.877511	0.959988
SUGT1P1	8.225974	0.007457	0.084528	0.088222	0.929701	NA
MYH4	0.067194	0.007452	0.012346	0.603619	0.546097	NA
EIF3M	109.4632	-0.00745	0.064992	-0.1146	0.90876	NA
RSPH6A	1.352807	0.007444	0.059609	0.124881	0.900618	NA
EVI5	317.8403	-0.00744	0.036168	-0.20562	0.837091	NA
C20orf195	0.190501	-0.00743	0.024839	-0.2992	0.764789	NA
DENND4C	607.3315	-0.00743	0.037651	-0.19737	0.843535	0.946541
ANXA2R	1.652121	0.007429	0.064167	0.115779	0.907828	NA
CTBS	31.87138	0.007424	0.075521	0.098304	0.921691	NA
GLG1	712.7921	-0.00742	0.03688	-0.20112	0.840607	0.946431
ANKRD18D	0.413503	0.007401	0.034633	0.213708	0.830774	NA
TUBD1	47.71341	-0.00739	0.062592	-0.11814	0.905955	NA
SNORD114	5.267638	0.007394	0.080462	0.091891	0.926784	NA
CLDN24	0.393402	-0.00739	0.033566	-0.22024	0.825685	NA
TSPAN33	29.59198	-0.00739	0.075195	-0.09829	0.921701	NA
TMEM74B	22.19334	0.007375	0.078949	0.093419	0.925571	NA
ZNF662	189.291	0.007374	0.058263	0.126563	0.899286	NA
C9orf92	0.343624	-0.00737	0.034461	-0.21393	0.8306	NA
TPTE	0.08118	0.00737	0.013965	0.527764	0.597663	NA
RNASE7	0.230949	-0.00737	0.024968	-0.29513	0.767893	NA
PLEKHA1	121.8765	-0.00737	0.046887	-0.15711	0.875157	NA
LOC33916	0.176744	-0.00736	0.023105	-0.31857	0.75005	NA
PCDH10	41.57279	-0.00735	0.084436	-0.08707	0.930613	NA
TMC5	6.001165	0.00735	0.083325	0.088206	0.929713	NA
ANKRD22	0.533217	-0.00735	0.037063	-0.1982	0.842887	NA
CLEC18B	2.654245	0.007344	0.065718	0.111756	0.911017	NA
BTBD2	246.9082	-0.00734	0.048138	-0.15257	0.87874	NA
RNF113B	0.952886	0.007344	0.050575	0.145211	0.884544	NA
MGC1588	4.487652	0.007341	0.081353	0.090234	0.928101	NA
DESI1	133.6105	-0.00734	0.054059	-0.13575	0.892022	NA
EFTUD1	71.82225	-0.00734	0.057465	-0.12768	0.898406	NA
GLRX5	27.37457	-0.00732	0.078454	-0.09327	0.925689	NA
TPP2	320.9621	-0.00732	0.044649	-0.16387	0.869834	NA
MIR3666	0.0679	0.007308	0.012226	0.597705	0.550037	NA
MBIP	85.50305	0.007306	0.053687	0.13609	0.89175	NA
TMEM41B	118.9994	0.007306	0.05785	0.126286	0.899506	NA
VKORC1	26.93032	-0.0073	0.071827	-0.10168	0.919013	NA
LOC10050	0.124283	-0.0073	0.018023	-0.40501	0.685468	NA
MIR4675	0.102243	-0.0073	0.017665	-0.413	0.679603	NA
LILRB1	3.310881	-0.00729	0.070218	-0.10383	0.917307	NA
MIR93	2.083831	0.007289	0.06816	0.106945	0.914833	NA
OR51I1	0.171755	0.007284	0.024217	0.300772	0.763589	NA

DDX52	149.8165	0.007274	0.045965	0.158258	0.874254	NA
FCGR1A	1.951092	-0.00727	0.060615	-0.11999	0.904487	NA
CALR	503.9822	0.007271	0.079233	0.091772	0.926879	0.980561
MIR4530	0.389768	-0.00727	0.033578	-0.21654	0.828563	NA
GABRR2	2.331682	0.007271	0.060836	0.11951	0.904871	NA
TMCO1	223.8531	0.00727	0.043447	0.16733	0.867111	NA
CDX1	0.157005	-0.00727	0.022939	-0.3169	0.75132	NA
RPS6KA4	179.4665	-0.00726	0.04852	-0.14966	0.881035	NA
NKIRAS2	39.32592	0.007258	0.066438	0.109243	0.91301	NA
MTERFD2	463.1135	-0.00725	0.045095	-0.16087	0.872194	0.958695
PUS3	33.94215	0.007247	0.070915	0.102188	0.918607	NA
PROC	6.240656	-0.00725	0.082566	-0.08776	0.930067	NA
LOC40004	1.622678	-0.00725	0.065102	-0.11129	0.911388	NA
C1orf204	15.1282	0.007235	0.081412	0.088869	0.929186	NA
TAS2R8	1.305032	0.007234	0.049124	0.147263	0.882925	NA
LRIT2	0.47641	-0.00723	0.038787	-0.18639	0.852137	NA
ZMAT3	280.306	0.007227	0.060164	0.120128	0.904382	NA
HNRNPD	749.947	0.007225	0.048778	0.148114	0.882253	0.961647
ZNF37A	546.8573	-0.00722	0.034404	-0.20999	0.833676	0.944594
LOC28358	0.084527	0.007223	0.013984	0.516506	0.605501	NA
OR2W5	0.216136	-0.00722	0.02474	-0.29192	0.770345	NA
ATP9A	1548.758	0.00722	0.050275	0.143614	0.885806	0.963665
SYN3	108.759	0.007219	0.082422	0.087585	0.930206	NA
DIS3	268.5754	-0.00721	0.040126	-0.17979	0.857321	NA
HES4	5.541219	-0.00721	0.081351	-0.08863	0.929374	NA
MYBPC1	12.34298	-0.00721	0.081762	-0.08818	0.929735	NA
MIR323B	0.26261	0.007205	0.030109	0.239288	0.810883	NA
SEPP1	78.65082	-0.0072	0.082562	-0.08716	0.930544	NA
IQGAP1	288.0306	-0.00719	0.055475	-0.12955	0.896921	NA
OR13C9	0.109284	0.007183	0.019391	0.37046	0.71104	NA
MIR134	1.305104	-0.00718	0.056699	-0.12659	0.899262	NA
SECISBP2L	870.0803	0.007163	0.039855	0.179722	0.857371	0.951001
FAM129C	6.358217	0.007152	0.082602	0.086589	0.930998	NA
EVL	409.3046	0.007149	0.040839	0.175045	0.861044	0.952278
NFKBIB	18.46193	0.007145	0.078569	0.090937	0.927543	NA
CD300LF	74.30826	0.007144	0.075784	0.094262	0.924901	NA
ARHGEF5	1.163018	0.007138	0.056832	0.125593	0.900054	NA
SLC2A10	6.695764	-0.00714	0.081748	-0.08731	0.930427	NA
ACOX3	63.32589	-0.00714	0.062718	-0.11379	0.909405	NA
NUDT17	20.14484	-0.00714	0.084643	-0.0843	0.932821	NA
TTLL1	58.59194	-0.00713	0.070711	-0.10084	0.919681	NA
LOC10050	2.051141	-0.00713	0.064153	-0.11109	0.911543	NA
TMEM196	1.224774	-0.00712	0.057943	-0.1229	0.902184	NA
IRF2BP2	300.447	0.00712	0.038009	0.187328	0.851404	NA
FAM53B	215.14	-0.00712	0.05172	-0.13764	0.890521	NA
CYP4A11	0.465239	-0.00711	0.038212	-0.18616	0.852321	NA
MOCS3	22.11369	0.007113	0.080581	0.088273	0.929659	NA

C18orf8	78.69826	0.007112	0.056224	0.126501	0.899336	NA
ZNF584	70.14087	0.00711	0.055929	0.127123	0.898843	NA
XIST	4734.174	-0.0071	0.035342	-0.20098	0.840715	0.946431
PI4K2A	97.68052	-0.00709	0.051685	-0.13721	0.890862	NA
MYOC	0.174541	-0.00709	0.024241	-0.2925	0.769902	NA
PSMD6	125.6299	-0.00709	0.049039	-0.14448	0.88512	NA
NMUR1	2.819807	0.007082	0.070855	0.099954	0.920381	NA
MIR1293	0.528504	0.007081	0.040202	0.176147	0.860179	NA
TMED6	7.080656	-0.00708	0.084898	-0.08339	0.933539	NA
GFM2	101.7544	0.007066	0.053089	0.133096	0.894117	NA
PPIF	35.68081	-0.00706	0.069317	-0.10188	0.918849	NA
TAAR3	1.104307	-0.00706	0.054301	-0.13003	0.896545	NA
CAD	116.2275	0.007059	0.064691	0.109111	0.913114	NA
RUVBL1	53.3996	0.007056	0.069027	0.102217	0.918585	NA
MTUS1	1283.37	0.007047	0.039046	0.180486	0.856771	0.951001
FLJ45974	0.089536	-0.00705	0.014177	-0.49703	0.619166	NA
MRPS34	130.3259	0.007045	0.05344	0.131831	0.895118	NA
COL19A1	316.2357	0.007039	0.081353	0.086523	0.93105	NA
ZNF71	69.35089	0.007036	0.057372	0.122645	0.902388	NA
TMED5	195.7103	0.007035	0.063097	0.111502	0.911218	NA
SIX4	37.38549	0.007028	0.08028	0.08754	0.930242	NA
HIST2H2A	17.8036	0.007022	0.084828	0.082779	0.934028	NA
NLGN4Y	60.51228	0.007018	0.021555	0.325591	0.744734	NA
CCNB1	34.11274	0.007013	0.075872	0.092431	0.926355	NA
SLC23A1	15.92491	0.007005	0.084426	0.082976	0.933871	NA
UBE2O	357.4275	-0.007	0.042812	-0.16361	0.870037	0.958257
MAP2K3	45.18674	0.007004	0.065678	0.106641	0.915074	NA
BTG4	0.069915	-0.007	0.0146	-0.4796	0.631514	NA
PPAP2C	2.985179	-0.007	0.07342	-0.09531	0.924067	NA
CCDC107	12.28324	0.006994	0.083691	0.08357	0.933398	NA
VCL	222.3223	-0.00699	0.050896	-0.13733	0.890767	NA
SLC26A4	0.823968	-0.00698	0.050642	-0.1379	0.890316	NA
MIR4719	0.069477	-0.00698	0.014579	-0.4789	0.63201	NA
C6orf163	16.42936	0.006974	0.082987	0.084038	0.933026	NA
ZNF549	173.0769	0.006969	0.051921	0.13423	0.89322	NA
FAM19A3	3.014934	-0.00696	0.067409	-0.10327	0.917746	NA
LOC10013	23.29505	0.006961	0.081771	0.085128	0.932159	NA
HDHD2	243.2867	-0.00696	0.041716	-0.16678	0.86754	NA
C11orf91	6.510416	-0.00694	0.079614	-0.08723	0.93049	NA
C2orf49	46.02592	0.006943	0.065606	0.105837	0.915712	NA
SNORD115	23.15307	-0.00694	0.083074	-0.08355	0.93341	NA
PTGIR	0.248397	-0.00694	0.029184	-0.23784	0.812004	NA
ACO2	274.3001	-0.00694	0.058617	-0.11835	0.905788	NA
MICALCL	5.359548	-0.00694	0.082322	-0.08426	0.932847	NA
AVL9	135.1906	0.006935	0.048339	0.143457	0.885929	NA
MIR1180	1.97601	-0.00693	0.068231	-0.10154	0.91912	NA
CEP55	0.121553	0.006926	0.017945	0.385936	0.699544	NA

CEACAM6	0.068116	-0.00692	0.014513	-0.4767	0.633573	NA
AP2A2	721.7007	0.006915	0.049678	0.139193	0.889297	0.965256
GNG3	121.8989	-0.00691	0.077966	-0.08867	0.929343	NA
LRRC18	0.257989	0.006913	0.029166	0.237029	0.812635	NA
GINM1	101.9687	0.006912	0.050216	0.137654	0.890514	NA
N6AMT2	23.13276	0.006907	0.074001	0.093335	0.925638	NA
C1orf21	792.4223	0.006906	0.045802	0.15079	0.880141	0.96027
MIR410	0.801594	-0.0069	0.051489	-0.13405	0.893363	NA
XXYLT1	26.48094	-0.0069	0.080719	-0.08544	0.93191	NA
TAOK2	410.6769	0.006885	0.041504	0.165883	0.868249	0.957242
TFAP2C	0.455147	0.006883	0.040022	0.171979	0.863454	NA
DGKB	63.193	-0.00688	0.080021	-0.08601	0.931457	NA
FGF23	2.136243	0.00688	0.059739	0.115174	0.908307	NA
LINC00691	0.87501	-0.00688	0.045999	-0.14951	0.881151	NA
DCBLD1	39.31788	-0.00687	0.084616	-0.08116	0.935312	NA
OBFC1	134.4576	0.006866	0.054902	0.125067	0.900471	NA
IGF2BP2	94.90372	0.006863	0.075727	0.090627	0.927789	NA
NUP85	160.754	-0.00686	0.063948	-0.10728	0.914564	NA
CCDC114	3.46837	0.006859	0.077065	0.089009	0.929075	NA
DCAF12L2	0.705765	0.006856	0.04336	0.158107	0.874372	NA
ANKRD30B	0.282711	-0.00685	0.029104	-0.2355	0.813819	NA
KLF14	0.066342	-0.00685	0.014441	-0.4743	0.635289	NA
SASH1	317.0605	-0.00685	0.077097	-0.08884	0.929213	NA
PIFO	1.682459	0.006848	0.06485	0.105602	0.915898	NA
CACNA1B	764.918	-0.00684	0.053607	-0.12765	0.898425	0.970091
CTSL1P2	0.475362	0.006841	0.037018	0.184814	0.853375	NA
WDR74	45.99928	-0.00684	0.063175	-0.10822	0.91382	NA
PEX1	285.2665	0.006837	0.046063	0.148417	0.882014	NA
FAM19A4	1.103872	0.006828	0.047425	0.143979	0.885517	NA
EXT2	125.4226	0.006826	0.054888	0.124371	0.901022	NA
PDCL2	0.117504	-0.00682	0.017947	-0.38005	0.703911	NA
MIR30D	0.065988	-0.00682	0.014408	-0.47319	0.636081	NA
ANGPT4	0.226455	0.006812	0.023546	0.289291	0.772359	NA
RAD54L2	236.6517	-0.00679	0.045139	-0.15053	0.880349	NA
KDM5A	715.7388	-0.00678	0.033983	-0.19956	0.841821	0.946541
C1orf127	5.424969	-0.00678	0.076638	-0.08848	0.929492	NA
DBP	127.9199	0.00678	0.061969	0.109409	0.912878	NA
LY6G6D	0.063103	-0.00677	0.014361	-0.47162	0.637201	NA
R3HDML	0.058735	0.006757	0.011759	0.574612	0.565554	NA
S100A3	0.388397	0.006757	0.032208	0.209781	0.833839	NA
SPATA32	0.353546	0.006756	0.034408	0.196356	0.844331	NA
LOC10013	0.878926	0.006752	0.049453	0.136532	0.891401	NA
ZNF705A	0.351293	0.006751	0.034877	0.193575	0.846509	NA
SLC22A16	0.226256	-0.00675	0.02352	-0.28691	0.774182	NA
NAALADL1	5.664363	-0.00674	0.083143	-0.08106	0.935394	NA
LOC15727	0.063369	-0.00674	0.014324	-0.4704	0.638073	NA
HSPA7	1.873686	-0.00674	0.061415	-0.10968	0.912664	NA

TRIM42	0.057811	0.006735	0.01174	0.573678	0.566185	NA
TEX37	0.06407	-0.00673	0.014317	-0.47017	0.638233	NA
HIRIP3	102.2197	0.006729	0.053103	0.126718	0.899164	NA
BHLHA9	0.307577	0.006719	0.030713	0.218772	0.826828	NA
MTA3	84.09721	0.006719	0.056708	0.118478	0.905689	NA
MIR450A2	0.536695	-0.00671	0.037931	-0.17695	0.85955	NA
TBC1D1	201.6572	-0.0067	0.059657	-0.11232	0.910567	NA
MAD1L1	166.8766	0.0067	0.060427	0.110871	0.911718	NA
LINC00466	0.063136	-0.00669	0.014276	-0.46881	0.639207	NA
C22orf24	2.90691	0.00669	0.072578	0.092175	0.926559	NA
OPA1-AS1	6.873739	0.006686	0.084384	0.079233	0.936848	NA
DDX5	2763.346	-0.00668	0.048213	-0.13853	0.889825	0.965256
MIR302B	0.177859	0.006671	0.021537	0.309738	0.75676	NA
LECT1	8.008983	-0.00667	0.084126	-0.07928	0.936811	NA
METTL17	95.84513	-0.00667	0.049241	-0.13541	0.892287	NA
ATP5L2	5.272957	0.006662	0.08177	0.081478	0.935062	NA
MIR184	0.345661	0.006662	0.035115	0.189727	0.849523	NA
DGCR2	219.3749	0.006662	0.051986	0.128152	0.898029	NA
SLC9A4	0.060915	-0.00666	0.014243	-0.46769	0.640007	NA
PTCHD3P1	130.9593	0.006659	0.05287	0.125956	0.899767	NA
STBD1	0.734061	-0.00665	0.046517	-0.14286	0.886398	NA
NCAPH	3.868594	0.006643	0.078686	0.084428	0.932716	NA
TOM1	105.581	0.006643	0.051976	0.12781	0.8983	NA
RFX6	0.058747	-0.00664	0.014223	-0.46703	0.640476	NA
OR52E4	0.060546	-0.00664	0.014223	-0.46701	0.64049	NA
PSIP1	1295.628	0.006634	0.042089	0.157623	0.874754	0.959476
FAM183B	0.061183	-0.00662	0.014198	-0.46618	0.641085	NA
LOC14584	0.26336	0.006617	0.027769	0.238301	0.811648	NA
C6orf147	31.4486	-0.00662	0.073187	-0.0904	0.927967	NA
43164	67.39739	0.006615	0.057723	0.1146	0.908763	NA
PRPSAP2	113.4228	-0.00661	0.056089	-0.11793	0.90612	NA
RFPL1-AS1	56.9382	0.006615	0.082773	0.079913	0.936306	NA
PRG4	6.900172	-0.00661	0.084507	-0.07823	0.937641	NA
TNS4	0.151935	0.006609	0.022917	0.288367	0.773066	NA
NMNAT1	21.28332	-0.00661	0.078239	-0.08445	0.9327	NA
LINC00583	0.061066	-0.00661	0.014184	-0.46572	0.641416	NA
C11orf44	0.057977	-0.0066	0.014179	-0.46556	0.641532	NA
BCAP31	114.554	-0.0066	0.057346	-0.11504	0.908416	NA
C11orf40	0.077095	0.006593	0.016079	0.410069	0.681755	NA
MIR4300	0.07642	0.006574	0.016054	0.409516	0.682161	NA
FOLR3	0.060165	-0.00657	0.014149	-0.46457	0.642237	NA
NCR3	0.055265	0.006573	0.011599	0.566715	0.570908	NA
LINC00359	0.06103	-0.00657	0.014148	-0.46455	0.642256	NA
MIR765	3.099106	-0.00657	0.076567	-0.08582	0.931607	NA
C1orf74	16.25971	-0.00657	0.08369	-0.07851	0.937421	NA
CUL9	290.0347	-0.00657	0.04698	-0.13984	0.88879	NA
C11orf85	1.93333	0.006569	0.059423	0.110541	0.911981	NA

MIR5087	0.363859	-0.00657	0.032437	-0.20249	0.839531	NA
LOC10019	0.454316	-0.00657	0.037471	-0.17527	0.860865	NA
NUP107	161.1222	0.006566	0.041753	0.157262	0.875038	NA
LOC10012	0.053184	0.006566	0.011592	0.5664	0.571122	NA
WDR90	75.51244	-0.00656	0.068667	-0.0956	0.923837	NA
PF4	0.092186	0.006558	0.016586	0.395394	0.692552	NA
OTOR	0.059038	-0.00656	0.01413	-0.46395	0.642686	NA
UBXN10	18.22421	0.006552	0.084917	0.077157	0.938498	NA
CPEB1	44.23235	-0.00655	0.065188	-0.10044	0.919996	NA
LINC00469	0.112495	-0.00654	0.017854	-0.36646	0.714021	NA
PDRG1	32.56118	0.006542	0.068143	0.096008	0.923514	NA
CKLF	9.203777	0.006538	0.084845	0.077054	0.93858	NA
FKBP6	0.059301	-0.00654	0.01411	-0.46327	0.643168	NA
EFCAB7	101.0863	0.006537	0.054194	0.120618	0.903994	NA
TGM2	76.02047	-0.00653	0.083364	-0.07834	0.937559	NA
LOC28426	26.87185	-0.00653	0.075246	-0.08674	0.93088	NA
KIF2B	0.060101	-0.00653	0.014098	-0.46285	0.643469	NA
DEDD	53.2404	0.006525	0.061772	0.105631	0.915875	NA
MIR222	0.101919	-0.00652	0.017656	-0.36941	0.711819	NA
RPS6KA2	291.6765	-0.00652	0.050985	-0.1279	0.898225	NA
RAB19	0.105089	0.006521	0.017708	0.368249	0.712688	NA
GABRA4	59.28716	0.006518	0.083075	0.078457	0.937464	NA
NPEPPS	440.1698	-0.00652	0.036977	-0.17626	0.860093	0.95169
MED14	162.7359	0.006517	0.049703	0.131114	0.895685	NA
DKK2	2.830571	0.006516	0.062824	0.103719	0.917392	NA
TMEM40	0.054845	0.006512	0.011545	0.564062	0.572712	NA
FAM83B	0.059269	-0.0065	0.014074	-0.46206	0.644037	NA
CLRN3	0.059399	-0.0065	0.014074	-0.46206	0.644042	NA
LOC61303	11.40474	-0.0065	0.084721	-0.07674	0.938834	NA
BTNL3	0.152514	-0.0065	0.021517	-0.30206	0.762607	NA
GDF6	0.265899	0.006497	0.030128	0.215632	0.829275	NA
SDR42E1	0.058522	-0.0065	0.014066	-0.46181	0.644218	NA
LDHA	219.8851	-0.00649	0.075546	-0.08594	0.931515	NA
LOC10065	11.01415	-0.00649	0.084866	-0.07645	0.939064	NA
CALCR	0.059276	-0.00648	0.014052	-0.46133	0.644561	NA
TIMM10B	237.3706	-0.00648	0.050923	-0.12727	0.898726	NA
FAM83C	0.939318	0.006479	0.052246	0.124007	0.90131	NA
PCSK2	103.3285	-0.00648	0.067327	-0.09621	0.923356	NA
MIR3151	1.18407	0.006477	0.048822	0.132672	0.894453	NA
BANK1	39.56804	-0.00648	0.083235	-0.07782	0.937973	NA
ODF4	0.059149	-0.00648	0.014045	-0.46109	0.644731	NA
ADAMTS2	0.164087	0.006472	0.023027	0.281069	0.778658	NA
CTSL1P8	0.059002	-0.00647	0.014036	-0.46081	0.644935	NA
MSANTD1	13.4062	-0.00646	0.084548	-0.07636	0.939137	NA
LRCH4	140.328	0.006455	0.076347	0.084549	0.93262	NA
CTAGE6P	1.108265	-0.00645	0.04965	-0.12996	0.896597	NA
C8orf87	0.057712	-0.00644	0.01401	-0.45993	0.645566	NA

KCNE1	0.973862	-0.00644	0.054362	-0.11853	0.905651	NA
MIR585	0.270498	0.006435	0.030892	0.208314	0.834984	NA
NRBF2	38.52762	0.006428	0.067956	0.094597	0.924635	NA
IL23R	0.056974	-0.00643	0.013992	-0.45935	0.64598	NA
CYP7B1	17.96166	0.006426	0.084899	0.075688	0.939667	NA
LY6D	0.057518	-0.00642	0.013987	-0.45918	0.646106	NA
DNAJA3	105.264	0.006419	0.056821	0.112962	0.910061	NA
CATSPERB	0.086403	0.006416	0.016405	0.391094	0.695728	NA
MIR501	0.071007	0.006415	0.015844	0.404866	0.685576	NA
DRD3	0.084595	0.006408	0.016442	0.389738	0.69673	NA
CSH2	0.056073	-0.00641	0.013971	-0.45864	0.64649	NA
ULBP3	0.05601	-0.0064	0.013967	-0.45852	0.64658	NA
MIR596	0.050468	0.006404	0.011449	0.559341	0.575929	NA
ATG9A	211.579	-0.0064	0.0511	-0.12529	0.900292	NA
ZKSCAN1	576.9049	0.006398	0.05923	0.108028	0.913973	0.972815
CT45A5	0.052283	0.006398	0.011444	0.559077	0.576109	NA
TIMMDC1	82.89646	0.006397	0.062479	0.102393	0.918445	NA
LOC44028	6.671002	0.006397	0.083036	0.07704	0.938592	NA
HS3ST3B1	1.349658	-0.00639	0.053727	-0.119	0.905275	NA
EI24	115.6807	-0.00639	0.050631	-0.12622	0.899558	NA
PLA2G4F	0.159219	-0.00639	0.022964	-0.27821	0.780855	NA
SNORD41	5.488945	-0.00639	0.069688	-0.09167	0.926959	NA
FAM135B	287.3376	0.006388	0.058831	0.108589	0.913528	NA
KLHL40	0.361023	-0.00639	0.033479	-0.19079	0.848692	NA
HTR1A	0.460656	0.006387	0.035927	0.177784	0.858892	NA
ITIH6	0.056839	-0.00639	0.013947	-0.45785	0.647062	NA
CCL20	0.054975	-0.00638	0.013942	-0.45766	0.647194	NA
LOC28508	0.057335	-0.00638	0.013941	-0.45765	0.647201	NA
SNORD115	9.237641	-0.00638	0.084155	-0.07582	0.939565	NA
OR52N1	0.045042	-0.00638	0.011474	-0.5559	0.578277	NA
LEFTY2	0.08492	0.006378	0.016356	0.389939	0.696582	NA
SPATA7	216.0658	-0.00638	0.078498	-0.08122	0.935264	NA
BTC	0.207378	0.006375	0.021946	0.2905	0.771434	NA
SEPN1	165.9322	-0.00638	0.046542	-0.13698	0.891048	NA
TANGO2	36.39148	-0.00637	0.074366	-0.08572	0.931692	NA
MIR583	0.169283	0.006363	0.024205	0.262874	0.792648	NA
PROKR2	0.465148	0.006362	0.038909	0.16351	0.870117	NA
LOC40062	0.056574	-0.00636	0.01392	-0.45693	0.64772	NA
ST8SIA1	349.0114	-0.00636	0.053004	-0.11999	0.904494	0.971859
UBE2B	142.702	0.006359	0.045038	0.141187	0.887722	NA
LBR	63.06645	-0.00636	0.069984	-0.09086	0.927606	NA
DDX10	212.9644	0.006357	0.046348	0.137165	0.890901	NA
TMEM202	0.084032	0.006355	0.016327	0.389236	0.697102	NA
MIR718	0.083935	0.006352	0.016324	0.389159	0.697159	NA
REL	133.9674	-0.00635	0.050933	-0.1247	0.900759	NA
SNORA23	21.95796	0.006351	0.084198	0.075434	0.939869	NA
SLC22A10	0.083868	0.006351	0.016321	0.389105	0.697199	NA

MIR3134	0.066788	0.006349	0.015757	0.402925	0.687003	NA
NRSN2	103.637	-0.00635	0.0747	-0.08498	0.932274	NA
LOC10028	0.055892	-0.00635	0.013904	-0.45639	0.648106	NA
MIR4270	0.055886	-0.00635	0.013903	-0.45638	0.648114	NA
MIR4735	0.08362	0.006344	0.016313	0.388907	0.697345	NA
MIR539	0.25843	0.00634	0.030117	0.210525	0.833258	NA
APC2	907.4389	-0.00633	0.056767	-0.11155	0.911181	0.972087
LOC64352	1.473675	-0.00633	0.063662	-0.09944	0.920792	NA
MIR5000	0.052403	-0.00633	0.013887	-0.45584	0.648507	NA
MIR660	0.082533	0.006327	0.016291	0.388376	0.697738	NA
DUXA	0.082756	0.006322	0.016284	0.388211	0.69786	NA
INTS3	418.3912	-0.00632	0.056456	-0.11194	0.910868	0.972087
PSG9	9.654965	0.006318	0.079054	0.079918	0.936302	NA
FAN1	221.8751	0.006314	0.057683	0.109466	0.912833	NA
POM121L4	0.081981	0.006314	0.016274	0.387987	0.698026	NA
FAM129B	117.7509	0.00631	0.062385	0.10115	0.919431	NA
SESN3	441.738	-0.00631	0.063997	-0.09859	0.921461	0.977889
OR2T8	0.054262	-0.00631	0.013863	-0.45505	0.649072	NA
LOC10050	0.08221	0.006307	0.016265	0.387767	0.698189	NA
SNAPC3	260.4528	0.006306	0.036314	0.17366	0.862133	NA
WDR5B	38.29196	0.006306	0.067863	0.092928	0.925961	NA
EGFL8	0.049854	0.006303	0.011359	0.554899	0.578963	NA
KLRK1	0.081531	0.0063	0.016256	0.387556	0.698344	NA
DMP1	0.322997	0.006297	0.031725	0.198496	0.842657	NA
FAM35A	63.45084	0.006294	0.059801	0.105251	0.916176	NA
RNF223	0.053348	-0.00629	0.013847	-0.45452	0.649456	NA
H2AFX	70.45213	0.006293	0.059136	0.106421	0.915248	NA
TMTC2	161.8394	0.006288	0.062346	0.100859	0.919662	NA
ZNF705D	0.052291	-0.00628	0.013833	-0.45404	0.649803	NA
PPP1CA	62.91502	0.00628	0.063841	0.098372	0.921637	NA
ARL2-SNX1	0.084008	0.006278	0.016463	0.381368	0.70293	NA
OR2A12	0.079725	0.006278	0.016276	0.385742	0.699688	NA
SLC22A25	0.0548	-0.00628	0.013827	-0.45385	0.649938	NA
ERN1	37.62243	-0.00627	0.070562	-0.08891	0.929152	NA
LOC44090	0.129236	-0.00627	0.018299	-0.34271	0.73182	NA
CLHC1	138.9974	0.00627	0.059687	0.105045	0.91634	NA
BEX4	194.5863	-0.00627	0.070788	-0.08855	0.92944	NA
TRIM22	30.2004	0.006266	0.084928	0.073784	0.941182	NA
SDHA	378.8928	-0.00627	0.05401	-0.11601	0.907644	0.971866
ETV7	0.448201	0.006253	0.036306	0.17224	0.863249	NA
OR6T1	0.05343	-0.00625	0.013799	-0.45293	0.650597	NA
LOC64662	0.628953	0.006246	0.043691	0.142963	0.88632	NA
RABGAP1	510.4012	-0.00625	0.02838	-0.22007	0.825817	0.944098
MGAT4B	185.0144	0.006239	0.059796	0.104342	0.916898	NA
ZFY	43.05041	0.006239	0.019459	0.320644	0.74848	NA
PDE1B	51.99187	-0.00624	0.084374	-0.07391	0.94108	NA
MIR4727	0.076962	0.006232	0.016217	0.384324	0.700739	NA

BSX	0.052787	-0.00623	0.013779	-0.45225	0.651086	NA
H2BFWT	0.126976	-0.00623	0.018223	-0.34172	0.73256	NA
ZCCHC13	0.07867	0.006224	0.016158	0.385217	0.700077	NA
C4orf29	100.6768	0.006224	0.056153	0.110832	0.911749	NA
LYPD8	0.076712	0.006223	0.016157	0.385188	0.700098	NA
LRBA	815.8249	-0.00622	0.04259	-0.14608	0.883862	0.962474
OTX2	17.51608	-0.00622	0.051227	-0.12137	0.9034	NA
HRC	4.513177	-0.00621	0.077834	-0.07984	0.936363	NA
MTX1	15.10352	0.006214	0.080423	0.077272	0.938407	NA
ADRB3	0.051119	-0.00621	0.013757	-0.45152	0.651618	NA
LOC40116	0.053036	-0.00621	0.013756	-0.4515	0.651628	NA
BRS3	0.076082	0.006205	0.016133	0.38463	0.700511	NA
UBB	563.7228	0.006203	0.069741	0.088948	0.929123	0.981525
GPRC6A	0.081146	0.006202	0.016366	0.378972	0.704708	NA
RGS13	0.042616	-0.0062	0.011315	-0.54813	0.583602	NA
LOC10050	0.046657	0.006197	0.011263	0.55019	0.582189	NA
HMGCS2	0.053098	-0.00619	0.013737	-0.45086	0.652092	NA
PAX9	0.05384	-0.00619	0.013734	-0.45075	0.652168	NA
IFNA16	0.077833	0.006188	0.01611	0.384087	0.700914	NA
VWC2L	0.249771	0.006185	0.023368	0.264664	0.791268	NA
CASS4	5.544883	-0.00618	0.08219	-0.07525	0.940017	NA
CTAGE4	0.052961	-0.00618	0.013727	-0.45051	0.65234	NA
ALPK2	0.076555	0.006183	0.016104	0.38394	0.701023	NA
TSPAN8	0.580019	0.006182	0.041609	0.148566	0.881896	NA
OBP2B	0.053109	-0.00618	0.013721	-0.45033	0.652475	NA
KCNE1L	3.167973	-0.00618	0.063645	-0.09706	0.92268	NA
GPR161	55.33078	0.006173	0.074701	0.082642	0.934136	NA
HOXB6	0.080025	0.006172	0.016327	0.378008	0.705425	NA
TNNI3	0.051258	-0.00617	0.013709	-0.44993	0.652762	NA
MIR4636	0.041949	-0.00616	0.01128	-0.5464	0.584793	NA
ZPBP2	0.04211	-0.00616	0.011275	-0.54616	0.584955	NA
LAD1	0.079418	0.006155	0.016305	0.377479	0.705818	NA
TAAR8	0.042425	-0.00615	0.011272	-0.54602	0.58505	NA
APOBEC3B	0.076073	0.006154	0.016066	0.383033	0.701695	NA
CDKL4	4.849783	0.006152	0.081932	0.075086	0.940147	NA
VDR	1.906894	-0.00615	0.067355	-0.09133	0.927234	NA
OR9Q1	0.0792	0.006149	0.016298	0.377289	0.705959	NA
TRAF3IP3	11.40297	-0.00615	0.083692	-0.07346	0.941438	NA
EDDM3A	0.076357	0.006146	0.016056	0.382793	0.701873	NA
TPR	1295.269	0.006145	0.032325	0.190114	0.84922	0.948602
LCE1E	0.076192	0.006141	0.016049	0.382647	0.701981	NA
FBXO16	87.52139	0.00614	0.06229	0.098578	0.921474	NA
TTLL13	6.868329	0.00614	0.084595	0.072584	0.942137	NA
HCG23	3.220873	0.006138	0.067104	0.091476	0.927115	NA
FAM81B	8.866677	0.006135	0.084855	0.072304	0.94236	NA
IL17F	0.05065	-0.00613	0.01367	-0.44861	0.653712	NA
SAMM50	67.41241	-0.00613	0.06408	-0.09564	0.923807	NA

FCRL6	1.318923	0.006128	0.053421	0.114713	0.908673	NA
CAPNS2	0.668458	-0.00613	0.046876	-0.13073	0.895989	NA
NBEAP1	3.804249	-0.00613	0.07803	-0.07851	0.937424	NA
SPANXC	0.042091	-0.00613	0.011245	-0.54473	0.58594	NA
OR1E1	0.050503	-0.00612	0.013655	-0.44811	0.654073	NA
ZNF609	608.8601	0.006118	0.031649	0.193309	0.846717	0.948463
MMP3	0.074267	0.006117	0.016018	0.381889	0.702544	NA
CRISP2	0.051269	-0.00612	0.013652	-0.44804	0.654127	NA
IQCF3	0.051942	-0.00611	0.01365	-0.44795	0.654189	NA
DDX53	16.22869	-0.00611	0.084925	-0.07197	0.942627	NA
MIR759	0.04944	-0.00611	0.013645	-0.44781	0.65429	NA
MIR4516	0.041836	-0.0061	0.011225	-0.54373	0.586624	NA
LOC73177	0.041811	-0.0061	0.011223	-0.54364	0.586692	NA
CCDC105	0.051659	-0.0061	0.01363	-0.44729	0.654663	NA
LOC10050	0.051275	-0.0061	0.013629	-0.44725	0.654692	NA
WDR20	252.5829	0.006091	0.044642	0.13645	0.891466	NA
NDUFC2-K	0.3425	0.006091	0.034131	0.178449	0.858371	NA
43435	0.04073	-0.00609	0.011213	-0.54315	0.587023	NA
UNC79	808.1779	-0.00609	0.043033	-0.14142	0.887535	0.964968
CNOT4	294.1313	0.006085	0.043229	0.140752	0.888066	NA
NLN	52.87333	-0.00608	0.075397	-0.08066	0.935709	NA
FLJ33534	0.24308	-0.00607	0.025004	-0.2429	0.808081	NA
TKTL1	0.112673	-0.00607	0.017894	-0.33934	0.734355	NA
MAGOH	51.30614	-0.00607	0.062027	-0.09787	0.922033	NA
TPI1P2	0.743395	0.006065	0.046013	0.131815	0.895131	NA
GDF2	0.073288	0.006063	0.015947	0.380218	0.703784	NA
DPYSL2	2160.7	-0.00606	0.047369	-0.12798	0.898166	0.970091
MIR3978	0.069718	0.006053	0.015983	0.378717	0.704898	NA
NSUN5P1	47.3486	-0.00605	0.069905	-0.08656	0.93102	NA
LOC10029	26.19646	0.006048	0.077964	0.077571	0.938169	NA
LOC40110	0.072279	0.006038	0.015914	0.379426	0.704371	NA
LCLAT1	53.13956	-0.00604	0.061124	-0.09877	0.921324	NA
MIR892A	0.041064	-0.00604	0.011163	-0.5407	0.588716	NA
CLNK	0.901173	-0.00603	0.049806	-0.12113	0.903588	NA
PRRC2C	2951.799	0.006032	0.036983	0.163111	0.870431	0.958257
CTAGE15P	0.131996	-0.00603	0.018367	-0.32817	0.742784	NA
STYX	106.4396	-0.00603	0.052872	-0.11399	0.909243	NA
GNAO1	1144.949	0.006023	0.048926	0.123095	0.902032	0.971056
LOC64493	0.071642	0.006019	0.015889	0.378825	0.704818	NA
PKD2	265.6333	0.006015	0.035495	0.169458	0.865437	NA
SLC35E2B	148.149	-0.00601	0.071329	-0.0843	0.932816	NA
PTGR2	71.48467	0.006008	0.070128	0.085676	0.931724	NA
FEZF1	0.585536	-0.00601	0.039268	-0.15299	0.878405	NA
MIR137HG	0.073807	0.006003	0.016095	0.372953	0.709183	NA
LOC28550	0.069742	0.006	0.015913	0.377041	0.706143	NA
SNORD115	0.047155	-0.006	0.013522	-0.4437	0.657261	NA
FAM225B	0.162811	-0.006	0.022988	-0.26079	0.794253	NA

SIRPG	0.222561	0.005989	0.024896	0.240566	0.809891	NA
NDUFV3	128.2747	0.005988	0.053765	0.111367	0.911325	NA
ABLIM3	603.0237	-0.00598	0.051946	-0.11511	0.908359	0.971866
RRAS2	39.63299	0.005979	0.069113	0.086513	0.931058	NA
PPME1	151.6121	0.005974	0.057218	0.104404	0.916848	NA
PPIC	7.352911	-0.00597	0.084664	-0.07053	0.943775	NA
PRDM13	0.072592	0.005967	0.016048	0.371795	0.710046	NA
BANP	125.5208	0.00596	0.071245	0.083661	0.933326	NA
PITRM1	251.508	0.005947	0.043524	0.136638	0.891317	NA
GUCD1	60.46519	0.005944	0.062432	0.095203	0.924154	NA
METTL7B	1.448902	0.005941	0.05673	0.104723	0.916595	NA
VSIG2	0.800511	-0.00593	0.047627	-0.1246	0.900839	NA
PRSS35	0.163008	-0.00593	0.021605	-0.27451	0.783693	NA
OR3A2	0.417783	0.00593	0.037746	0.157101	0.875165	NA
MSANTD2	48.27888	-0.00593	0.071605	-0.08275	0.934049	NA
NUP93	104.6693	-0.00592	0.058441	-0.10129	0.919322	NA
SFTA1P	0.690208	0.005919	0.046403	0.127561	0.898496	NA
C21orf59	251.0813	0.005915	0.051357	0.115184	0.9083	NA
PRKAG3	0.073871	0.005914	0.016151	0.366184	0.714228	NA
TMEM95	0.689178	-0.00591	0.045229	-0.13069	0.896018	NA
SOSTDC1	1.326726	-0.00591	0.048112	-0.12279	0.902274	NA
RPP14	135.684	-0.0059	0.049542	-0.11917	0.905137	NA
RAB4A	145.4297	-0.0059	0.056764	-0.10397	0.917195	NA
NTS	6.363364	0.005898	0.054967	0.107308	0.914544	NA
BTD	67.20829	-0.00589	0.069658	-0.08453	0.932638	NA
USP4	282.8829	0.005885	0.040632	0.144843	0.884835	NA
PTK2	620.511	-0.00588	0.043192	-0.13624	0.891629	0.965825
C2orf42	56.76441	-0.00588	0.060813	-0.09673	0.92294	NA
KRTAP19-8	0.045209	-0.00588	0.013387	-0.43923	0.660497	NA
GMIP	9.406522	-0.00588	0.08466	-0.06945	0.944634	NA
CTAGE11P	0.167955	0.005876	0.019903	0.295227	0.767821	NA
DKFZP586I	98.44092	-0.00588	0.064653	-0.09088	0.927589	NA
POLR1E	60.61308	0.005875	0.065536	0.089647	0.928568	NA
SYPL2	6.948702	0.005873	0.083814	0.070078	0.944132	NA
PKDCC	4.815722	0.005873	0.078111	0.075182	0.94007	NA
LOC72904	14.54939	-0.00587	0.084387	-0.06954	0.944559	NA
ANGEL1	87.62958	0.005863	0.065496	0.089521	0.928668	NA
CCDC17	12.29127	-0.00586	0.084823	-0.06911	0.944899	NA
CDH26	26.57894	0.005862	0.079431	0.0738	0.94117	NA
PDXK	492.4293	0.005862	0.055698	0.10524	0.916185	0.973677
KIFC3	37.724	-0.00586	0.075024	-0.07812	0.93773	NA
CES1P1	0.098681	-0.00586	0.015771	-0.37164	0.710163	NA
ZNF266	124.5057	0.005851	0.069322	0.084405	0.932734	NA
SNORA25	8.898965	0.005849	0.084585	0.069143	0.944875	NA
MAP1LC3C	0.165943	-0.00584	0.023024	-0.25385	0.799609	NA
TTI2	25.9111	-0.00584	0.073474	-0.07951	0.936629	NA
C4orf22	36.39539	0.00584	0.084141	0.069409	0.944664	NA

IL2RG	0.596624	-0.00584	0.040342	-0.14476	0.884899	NA
OR11H6	0.071824	0.00584	0.016054	0.363752	0.716043	NA
ZMIZ2	287.6586	-0.00584	0.049889	-0.11702	0.906843	NA
EPHX4	6.080352	-0.00583	0.084082	-0.06939	0.944682	NA
GPR142	2.410423	0.005833	0.06693	0.087152	0.930551	NA
MIR424	0.038721	-0.00583	0.010974	-0.53147	0.59509	NA
PEX16	23.02326	-0.00583	0.074607	-0.07816	0.937702	NA
CYP4F8	0.038237	-0.00583	0.01097	-0.53127	0.595231	NA
MYOD1	1.69766	0.005826	0.066514	0.087584	0.930207	NA
TMEM184	1.872992	0.005821	0.066006	0.088183	0.929732	NA
NVL	164.7181	0.005814	0.055951	0.103916	0.917236	NA
MIR4440	0.285675	-0.00581	0.029412	-0.1976	0.843356	NA
TMCO2	0.689474	-0.0058	0.046519	-0.12472	0.900749	NA
MIR559	0.038132	-0.0058	0.010944	-0.53002	0.596101	NA
LOC10050	0.038111	-0.0058	0.010943	-0.52995	0.596145	NA
ZNF683	0.066926	0.005798	0.01584	0.366024	0.714347	NA
SUCO	183.7039	0.005793	0.049936	0.116001	0.907652	NA
ASPG	0.170811	0.005788	0.024224	0.238946	0.811147	NA
MIR3620	0.069534	0.005785	0.015982	0.361961	0.717381	NA
C1orf210	0.037955	-0.00578	0.010928	-0.5292	0.596665	NA
LINC00323	1.070298	0.005778	0.043689	0.132246	0.89479	NA
NELFB	64.79663	-0.00577	0.058942	-0.09791	0.922001	NA
ZHX1-C8O	2.333538	-0.00577	0.072242	-0.07986	0.936349	NA
ST8SIA5	395.0641	0.005759	0.048219	0.119433	0.904932	0.971866
SH3PXD2B	300.0944	-0.00576	0.051486	-0.11184	0.91095	NA
SNORD23	0.979614	-0.00576	0.051316	-0.11219	0.910671	NA
ANKRD1	2.268311	0.00575	0.07211	0.079745	0.93644	NA
GOT1L1	0.065629	0.005748	0.015759	0.364724	0.715317	NA
TECTB	0.043259	-0.00575	0.013236	-0.4342	0.664142	NA
MIR3145	0.291944	-0.00574	0.031801	-0.18056	0.85671	NA
POM121L1	2.218668	0.00574	0.064919	0.088414	0.929548	NA
MBL2	0.05697	0.005736	0.015544	0.369022	0.712111	NA
SEC23IP	205.1258	-0.00574	0.047735	-0.12015	0.904364	NA
TBC1D3P2	0.037234	-0.00573	0.010882	-0.52699	0.5982	NA
NPFFR2	0.03721	-0.00573	0.01088	-0.52689	0.598272	NA
DCN	17.7475	-0.00573	0.084922	-0.06748	0.946199	NA
TC2N	2.274068	-0.00572	0.063506	-0.09004	0.928259	NA
NOL10	191.2636	-0.00571	0.041377	-0.13798	0.890256	NA
HOXA7	0.110105	0.005707	0.019189	0.297401	0.76616	NA
TTC29	0.563646	0.005691	0.040451	0.14069	0.888115	NA
ZBTB20	714.4105	-0.00569	0.054921	-0.10362	0.917472	0.97425
EXOSC5	12.73043	0.005691	0.083987	0.067756	0.94598	NA
SLFN13	2.667742	-0.00569	0.067182	-0.08469	0.932505	NA
TNPO3	196.9915	-0.00569	0.038307	-0.14843	0.882004	NA
LOC33980	0.113438	-0.00568	0.016005	-0.35513	0.722491	NA
PHLDB2	15.18619	0.005677	0.084931	0.066842	0.946708	NA
OR10V1	0.036246	-0.00567	0.010825	-0.52417	0.60016	NA

USP26	0.036246	-0.00567	0.010825	-0.52417	0.60016	NA
BTBD18	69.46224	-0.00567	0.061285	-0.09245	0.926338	NA
GLUD2	1.975516	0.005663	0.066118	0.085654	0.931741	NA
RPSAP52	0.036753	-0.00566	0.010809	-0.52342	0.600684	NA
LOC28429	0.036753	-0.00566	0.010809	-0.52342	0.600684	NA
LINC00853	0.219273	0.005656	0.026146	0.21633	0.828731	NA
NMBR	0.328919	0.005646	0.032033	0.176269	0.860083	NA
LTBP2	52.62979	-0.00564	0.066064	-0.08544	0.931912	NA
FGR	7.877835	-0.00564	0.079532	-0.07092	0.943459	NA
IFRD1	126.5435	-0.00564	0.069463	-0.0812	0.935284	NA
PRORSD1P	14.92298	-0.00564	0.08367	-0.06741	0.946254	NA
LOC10050	0.495262	-0.00564	0.038131	-0.14789	0.882428	NA
MIR4274	0.035838	-0.00564	0.010791	-0.52254	0.601291	NA
KRT81	0.035838	-0.00564	0.010791	-0.52254	0.601291	NA
CCL19	0.481308	0.005638	0.034278	0.164495	0.869341	NA
PET112	51.07278	-0.00564	0.066832	-0.08432	0.932799	NA
KLK3	0.036207	-0.00562	0.010773	-0.52167	0.601901	NA
CCDC166	0.11131	0.005616	0.017773	0.315987	0.752012	NA
LOC50351	0.036132	-0.00561	0.010768	-0.52143	0.60207	NA
SLC17A3	0.065277	0.00561	0.015942	0.351904	0.72491	NA
TXLNG2P	28.62874	0.005608	0.02451	0.228794	0.819029	NA
CXorf48	0.23097	0.0056	0.024789	0.2259	0.821279	NA
C17orf75	91.74392	0.005597	0.065326	0.085686	0.931716	NA
LINC00327	1.609063	0.005596	0.058924	0.094965	0.924342	NA
CYP2C9	0.035843	-0.00559	0.010746	-0.52033	0.602835	NA
ELOVL2-AS	2.19613	0.00559	0.066795	0.083687	0.933305	NA
CPED1	10.09653	-0.00559	0.084801	-0.06586	0.947487	NA
PGK1	265.0595	0.005584	0.071617	0.077977	0.937846	NA
SYNPO2L	11.09983	0.005577	0.084687	0.065851	0.947496	NA
MIRLET7C	0.533001	0.005575	0.039493	0.141177	0.88773	NA
MIR4663	0.223219	0.005575	0.026439	0.210866	0.832992	NA
HIST1H1C	146.9418	0.005572	0.083745	0.06653	0.946956	NA
LOC10013	0.564259	0.005564	0.041545	0.133931	0.893457	NA
RNF25	40.0353	0.00556	0.064186	0.086619	0.930974	NA
LOC15368	5.261115	-0.00555	0.081767	-0.06791	0.945859	NA
MIR5090	0.193093	0.005551	0.021634	0.2566	0.797487	NA
EIF1AY	9.657649	0.005541	0.019511	0.283988	0.776419	NA
SCFD1	176.5938	-0.00554	0.057669	-0.09606	0.923473	NA
KCNB2	0.662172	-0.00554	0.043761	-0.12658	0.899272	NA
PNMA6D	0.034978	-0.00553	0.010687	-0.51744	0.60485	NA
OR7C1	0.126571	-0.00553	0.019548	-0.28281	0.777325	NA
LOC37529	0.034924	-0.00553	0.010683	-0.51726	0.604978	NA
LINC00460	0.034878	-0.00552	0.01068	-0.51711	0.605079	NA
KCNS3	2.099908	-0.00552	0.066369	-0.0832	0.933693	NA
SNORD88A	0.761245	0.005519	0.047958	0.115089	0.908375	NA
LOC40117	0.034744	-0.00551	0.01067	-0.51664	0.605405	NA
GEMIN7	32.11641	-0.0055	0.070833	-0.07768	0.938086	NA

DEFB132	0.034581	-0.0055	0.010659	-0.51609	0.605795	NA
SPTBN1	5324.237	0.005499	0.036642	0.150077	0.880704	0.960421
LAMC3	7.132747	-0.0055	0.084152	-0.06532	0.947918	NA
TMEM139	6.572352	-0.00549	0.083715	-0.06562	0.947681	NA
ARHGAP17	131.3471	-0.00549	0.047788	-0.11492	0.908511	NA
LINC00582	0.429278	-0.00549	0.035049	-0.15666	0.875514	NA
FAM27A	1.026084	-0.00549	0.049807	-0.11024	0.912222	NA
RNF224	0.957838	0.005489	0.050835	0.107976	0.914015	NA
TNPO2	401.3601	-0.00549	0.044053	-0.12457	0.900867	0.970824
GATA4	0.034332	-0.00548	0.010641	-0.51523	0.606393	NA
NCAPG2	72.21222	-0.00548	0.064744	-0.08463	0.932553	NA
MIR4478	0.034278	-0.00548	0.010638	-0.51504	0.606524	NA
LOC10050	0.351575	0.005477	0.035117	0.15596	0.876065	NA
PHF6	174.9845	0.005472	0.051102	0.107072	0.914732	NA
MIR335	0.439223	0.005468	0.037573	0.145536	0.884287	NA
TMEM191	3.502713	-0.00546	0.078645	-0.06948	0.944604	NA
PIWIL3	0.033398	-0.00546	0.010623	-0.51434	0.607012	NA
FAM174A	33.67469	0.005464	0.076693	0.071243	0.943204	NA
SLC30A6	95.16715	-0.00546	0.051249	-0.10653	0.915159	NA
TRAPPC3	49.69992	0.005459	0.070375	0.077567	0.938173	NA
PCDHA2	26.8813	0.005447	0.077315	0.070453	0.943833	NA
MIR16-1	0.174565	-0.00544	0.023087	-0.23582	0.81357	NA
KDM4A-AS	10.45739	-0.00544	0.083751	-0.06499	0.94818	NA
CPA3	0.03375	-0.00544	0.010601	-0.51325	0.607778	NA
TRAT1	0.03375	-0.00544	0.010601	-0.51325	0.607778	NA
TM4SF1	28.27339	-0.00544	0.082934	-0.0656	0.947696	NA
EMCN-IT3	0.820653	0.005439	0.050366	0.107987	0.914006	NA
OXGR1	0.306327	-0.00543	0.030796	-0.17637	0.860003	NA
FAM58A	18.91862	0.005431	0.082223	0.066047	0.947341	NA
DGAT1	96.79635	0.005429	0.06307	0.086073	0.931408	NA
GAS6	126.8776	0.005428	0.060538	0.089658	0.928559	NA
LAMTOR2	14.37518	-0.00543	0.08191	-0.06625	0.947179	NA
ISX	0.033506	-0.00542	0.010583	-0.51237	0.608392	NA
MIR4321	0.126551	0.005422	0.019499	0.278076	0.780954	NA
ATP13A1	146.0307	0.005412	0.056181	0.096324	0.923263	NA
MIR4720	1.344065	-0.00541	0.057773	-0.0936	0.925427	NA
ACSBG2	17.39507	-0.00541	0.083512	-0.06474	0.948384	NA
ZNF558	85.29631	0.005404	0.059224	0.091254	0.92729	NA
KLHL21	92.06336	-0.0054	0.066782	-0.08087	0.935543	NA
ZBTB20-AS	41.1886	-0.0054	0.078201	-0.069	0.944993	NA
DNAJC9-A	15.57064	-0.00539	0.082879	-0.06509	0.948105	NA
LIMS3-LOC	0.033059	-0.00539	0.010549	-0.51074	0.609536	NA
CCDC3	11.53631	0.005386	0.084856	0.063478	0.949386	NA
SNORD27	188.0831	-0.00538	0.08483	-0.06344	0.949414	NA
RAB11FIP3	569.0305	-0.00538	0.050224	-0.10716	0.914665	0.973094
HOXD1	1.054185	-0.00538	0.055285	-0.09731	0.922477	NA
OR4C45	0.032906	-0.00538	0.010538	-0.51018	0.609923	NA

LOC55011	0.032879	-0.00537	0.010536	-0.51008	0.609999	NA
GPBAR1	2.977718	0.005373	0.07291	0.073696	0.941252	NA
RNF7	52.0189	0.005366	0.061447	0.08733	0.930409	NA
PADI6	0.032541	-0.00535	0.01051	-0.50883	0.610868	NA
LOC40112	8.474571	-0.00535	0.084882	-0.063	0.949769	NA
TBC1D3B	0.108781	-0.00535	0.017873	-0.29907	0.764887	NA
LOC72873	0.347673	-0.00534	0.03332	-0.16038	0.872578	NA
ZNF232	57.43534	-0.00534	0.063348	-0.08431	0.932814	NA
ESPNL	23.18791	0.005334	0.062961	0.084722	0.932483	NA
SPSB1	5.136965	0.005333	0.07933	0.067231	0.946398	NA
NMRK2	0.445466	-0.00533	0.038169	-0.13962	0.888962	NA
STAG1	533.3285	0.005322	0.042767	0.12445	0.900959	0.970824
SNORA60	5.82449	-0.00532	0.080743	-0.0659	0.947454	NA
SART3	410.0367	0.005313	0.031829	0.166925	0.867429	0.957242
SLC4A3	215.6971	0.005311	0.050827	0.104486	0.916784	NA
ADIRF	6.31245	0.00531	0.080648	0.065839	0.947506	NA
LINC00290	0.347238	-0.00531	0.033304	-0.15941	0.873345	NA
DZIP1	487.9993	0.005307	0.043529	0.121923	0.90296	0.97114
OLAH	1.342381	-0.0053	0.059535	-0.08898	0.929094	NA
NKPD1	15.3209	-0.0053	0.08303	-0.0638	0.949132	NA
NFE2L2	304.9558	-0.0053	0.060835	-0.08705	0.930631	NA
OR7E156P	0.125567	0.005295	0.018014	0.293959	0.768789	NA
MIR2276	0.126484	0.005288	0.019508	0.271086	0.786325	NA
ART1	2.88826	0.005285	0.069576	0.075959	0.939452	NA
PPP1R32	19.45128	0.005281	0.082713	0.063852	0.949088	NA
PDZD3	0.50907	-0.00528	0.041265	-0.12786	0.898262	NA
SEMA4G	88.33866	0.005272	0.061011	0.086406	0.931143	NA
SPINK7	7.862112	-0.00527	0.084299	-0.06252	0.950152	NA
OR1N2	0.099178	0.005269	0.014864	0.354482	0.722978	NA
PVRL3-AS1	0.544351	-0.00526	0.041876	-0.12561	0.900037	NA
FAM64A	9.845317	-0.00526	0.084719	-0.06209	0.950493	NA
DCAF11	201.7727	-0.00526	0.042567	-0.12346	0.901739	NA
KCTD13	92.03006	0.005252	0.051731	0.101522	0.919136	NA
MSH6	383.1422	0.005251	0.0477	0.110092	0.912336	0.972087
NR5A1	0.309628	0.005248	0.031713	0.165471	0.868573	NA
DZIP1L	107.4061	-0.00524	0.074803	-0.07002	0.944174	NA
PRKY	27.65506	0.005231	0.018167	0.287934	0.773397	NA
SERTAD4-	4.595956	-0.00523	0.07682	-0.06805	0.945744	NA
LOC28623	0.031019	-0.00523	0.010391	-0.50301	0.614956	NA
CHEK1	13.8303	-0.00523	0.084742	-0.06167	0.950827	NA
TMEM47	44.96591	-0.00522	0.08454	-0.06177	0.950743	NA
USP9Y	235.967	0.005218	0.018547	0.281317	0.778467	NA
SLC26A1	63.70121	-0.00522	0.072121	-0.07233	0.942343	NA
SNORD115	0.120238	-0.00521	0.016327	-0.31891	0.749794	NA
TMEM2	408.8391	0.005204	0.058738	0.088602	0.929399	0.981525
MIR651	0.105267	-0.0052	0.017777	-0.29264	0.769796	NA
TMUB1	18.02914	-0.0052	0.079865	-0.06511	0.948084	NA

CCDC167	15.11223	-0.0052	0.084064	-0.06184	0.950694	NA
ATP4B	1.7243	0.005196	0.061558	0.084414	0.932727	NA
PAPOLG	191.353	-0.00519	0.0392	-0.13251	0.894585	NA
MTSS1L	2123.532	0.00519	0.063515	0.08172	0.934869	0.984229
WNT6	0.170533	0.005189	0.023098	0.224669	0.822237	NA
FASLG	0.03052	-0.00519	0.010353	-0.50118	0.616246	NA
OR2K2	0.117814	0.005187	0.01933	0.268322	0.788451	NA
MTMR4	419.8193	0.005185	0.035452	0.146243	0.88373	0.962474
UNQ6494	0.030466	-0.00518	0.010349	-0.50096	0.616401	NA
UTY	122.2903	0.005184	0.017832	0.290723	0.771263	NA
PDP2	116.6314	0.005184	0.053482	0.096931	0.922781	NA
LINC00200	0.191197	-0.00518	0.021769	-0.23813	0.811784	NA
KDELC2	35.93421	-0.00518	0.068127	-0.07605	0.93938	NA
CHRNA10	8.928073	0.005175	0.084268	0.06141	0.951033	NA
NUCKS1	1664.629	-0.00517	0.055224	-0.09362	0.925411	0.979923
BIN3	65.08651	0.005163	0.060298	0.085633	0.931758	NA
GJB1	4.838253	0.005161	0.079074	0.065263	0.947965	NA
CCDC168	19.67414	0.00516	0.070534	0.073149	0.941688	NA
DKKL1	0.298111	-0.00516	0.034145	-0.1511	0.879898	NA
RAX	0.116138	0.005152	0.019344	0.266304	0.790005	NA
CD248	6.444653	-0.00515	0.082273	-0.06257	0.950111	NA
GALE	20.29162	0.005147	0.075338	0.068321	0.94553	NA
DOLPP1	24.22434	-0.00515	0.077175	-0.06669	0.946832	NA
ABI3BP	28.12359	-0.00514	0.081578	-0.06304	0.949732	NA
ITPRIP	9.384619	-0.00514	0.083222	-0.06178	0.950741	NA
EFCAB5	44.35923	-0.00514	0.077499	-0.06633	0.947118	NA
CRIP1	6.264008	-0.00513	0.082173	-0.06239	0.950254	NA
TRAP1	229.781	-0.00512	0.047695	-0.10743	0.914452	NA
LOC64673	0.159055	-0.00512	0.024095	-0.21235	0.831837	NA
HEATR4	104.9412	-0.00511	0.069198	-0.07385	0.941128	NA
IKBKG	30.0608	0.005107	0.072377	0.070566	0.943744	NA
CDRT15P2	0.091765	0.005104	0.01463	0.3489	0.727164	NA
BIK	3.761494	-0.0051	0.076773	-0.06637	0.947085	NA
MIR4263	0.090983	0.005086	0.014604	0.348286	0.727626	NA
LOC10050	5.520462	0.005086	0.080675	0.063044	0.949731	NA
TMEM9B-	12.79173	-0.00509	0.084939	-0.05987	0.952259	NA
FCER2	0.91646	-0.00508	0.049443	-0.10275	0.918161	NA
DNAJC5B	0.090697	0.00508	0.014594	0.348059	0.727796	NA
C3orf27	0.514012	-0.00507	0.039297	-0.12901	0.897349	NA
MIR3977	0.073592	0.005062	0.013978	0.362126	0.717258	NA
DKFZP434	0.825558	0.005049	0.049745	0.101504	0.91915	NA
ACSS2	99.15366	-0.00505	0.058551	-0.08624	0.931278	NA
WTAPP1	0.401602	0.005045	0.035524	0.142028	0.887058	NA
PTPLAD2	24.50987	0.005045	0.07501	0.067263	0.946373	NA
ESCO1	274.3497	0.005045	0.038912	0.129648	0.896845	NA
G6PC	0.263137	-0.00504	0.02807	-0.17959	0.857476	NA
OGDHL	263.6978	0.005037	0.049988	0.100759	0.919742	NA

STX17	358.2629	0.005028	0.044236	0.113653	0.909513	0.971866
MAPK10	531.8432	0.005025	0.043699	0.115	0.908445	0.971866
SLC29A1	49.51988	0.005023	0.065089	0.077172	0.938487	NA
XPNPEP3	71.96082	-0.00502	0.061878	-0.08114	0.935329	NA
ITFG1	202.57	0.005016	0.071361	0.070295	0.943958	NA
ROM1	17.80964	-0.00501	0.080212	-0.06247	0.950192	NA
LOC63930	2.817018	-0.00501	0.061113	-0.08198	0.934665	NA
NEIL3	0.08767	0.005009	0.014492	0.345621	0.729627	NA
LINC00244	0.086892	0.005005	0.014486	0.345494	0.729723	NA
OTX1	0.309623	-0.005	0.033605	-0.14874	0.881756	NA
SPANXA2-	0.871369	-0.005	0.050985	-0.09801	0.921924	NA
LGALS13	0.918021	0.004994	0.04876	0.102415	0.918427	NA
MIR4451	0.2321	-0.00499	0.028421	-0.1756	0.86061	NA
PPP2R5C	339.1166	-0.00499	0.040385	-0.12356	0.901668	0.971056
MIR143HG	30.95374	0.004982	0.08488	0.058696	0.953195	NA
CHD6	1094.775	0.004982	0.032711	0.152301	0.87895	0.959988
MIR301B	0.085609	0.004974	0.014441	0.344425	0.730527	NA
NRAP	0.314355	-0.00497	0.032914	-0.15103	0.879955	NA
TULP2	2.155588	0.004963	0.061859	0.080231	0.936053	NA
OR13H1	0.085138	0.004958	0.014419	0.343882	0.730935	NA
TTTY14	5.73935	0.004955	0.020844	0.237695	0.812118	NA
MIR4706	1.215269	-0.00495	0.058915	-0.08406	0.93301	NA
DNAJB13	1.103471	-0.00495	0.053748	-0.09213	0.926598	NA
FAM110B	155.9032	-0.00494	0.066687	-0.07406	0.940961	NA
AHCTF1P1	8.961516	-0.00494	0.084675	-0.05831	0.953504	NA
DMRT2	1.472411	-0.00492	0.054514	-0.0902	0.928126	NA
ACTB	2397.297	0.00491	0.064991	0.075546	0.93978	0.984229
ISOC1	6.581432	-0.0049	0.083076	-0.05901	0.952943	NA
HBBP1	0.660647	-0.0049	0.047423	-0.10337	0.917672	NA
LOC38890	3.700247	0.004901	0.07627	0.064255	0.948767	NA
ARHGEF15	7.046606	0.004899	0.080434	0.060902	0.951437	NA
SMYD3	90.78776	-0.0049	0.052297	-0.09366	0.925376	NA
ACAD11	15.56981	0.004898	0.081278	0.060261	0.951948	NA
LOC72832	39.3565	0.004894	0.070196	0.069716	0.94442	NA
IFNA22P	0.223631	0.00489	0.026167	0.186883	0.851752	NA
CCDC153	0.311138	-0.00489	0.032914	-0.14857	0.881889	NA
MIR5192	0.20248	0.004881	0.026066	0.187267	0.851451	NA
TMEM141	35.75325	0.004879	0.071648	0.068098	0.945708	NA
DMRTB1	0.06581	0.004876	0.013707	0.355737	0.722038	NA
MGAM	2.608857	-0.00487	0.066432	-0.07334	0.941538	NA
APOL5	0.631284	0.00487	0.040441	0.120433	0.90414	NA
DAZL	0.052891	0.004866	0.01211	0.401805	0.687828	NA
TMCO5A	0.052891	0.004866	0.01211	0.401805	0.687828	NA
WDR33	662.4155	0.00486	0.031882	0.152451	0.878831	0.959988
LOC38924	17.23239	-0.00486	0.080549	-0.0603	0.951916	NA
CCDC54	0.476859	0.004856	0.038619	0.125732	0.899944	NA
THRB-AS1	0.803725	0.00485	0.049279	0.098415	0.921603	NA

MIR4767	14.27568	0.004849	0.084548	0.057353	0.954264	NA
MIR4536-1	0.206042	0.004842	0.026072	0.185718	0.852666	NA
ARID3B	67.24329	0.004841	0.071585	0.067631	0.946079	NA
DNM3OS	201.6436	-0.00484	0.060552	-0.07994	0.936285	NA
C6orf99	0.303417	-0.00484	0.027839	-0.17385	0.861981	NA
TMEM213	0.436288	0.004837	0.035705	0.135476	0.892235	NA
KLF9	792.1339	0.004837	0.072063	0.067118	0.946488	0.988494
LOC10050	0.148586	-0.00483	0.019826	-0.24372	0.807446	NA
SPEN	2518.62	-0.00483	0.053982	-0.08945	0.928728	0.981525
MIR378D2	0.125151	-0.00483	0.019526	-0.24714	0.804798	NA
NKD1	149.8782	0.004824	0.070999	0.06795	0.945826	NA
SLC18A3	0.114708	0.00482	0.019278	0.250042	0.802555	NA
NIPAL2	42.14162	0.004816	0.070351	0.068451	0.945427	NA
RAP2B	39.81026	0.004813	0.071735	0.067101	0.946501	NA
LINC00272	1.606993	-0.00481	0.060249	-0.07987	0.936344	NA
TMEM37	0.50546	-0.00481	0.04064	-0.11839	0.905756	NA
FAM60A	15.22217	-0.00481	0.084939	-0.05664	0.954829	NA
SPRNP1	45.7162	0.0048	0.08288	0.05792	0.953812	NA
OTOA	0.555356	0.0048	0.039397	0.121825	0.903037	NA
TRPC1	159.8604	-0.0048	0.063898	-0.07511	0.940125	NA
HMBOX1	244.1751	-0.0048	0.04583	-0.10463	0.916668	NA
MIAT	317.2172	-0.00478	0.084476	-0.0566	0.954861	NA
TSPEAR	0.400386	0.00478	0.03697	0.129305	0.897116	NA
BIN1	452.752	0.00478	0.049927	0.095738	0.923729	0.9786
NOMO2	29.54066	-0.00478	0.079415	-0.06015	0.952034	NA
PHF12	269.1445	-0.00477	0.041681	-0.11452	0.908823	NA
C18orf63	0.064552	-0.00477	0.012068	-0.39548	0.69249	NA
CCDC80	83.4121	-0.00477	0.078793	-0.06051	0.951749	NA
NEBL	1232.323	0.004764	0.048736	0.097751	0.92213	0.977889
WIPI1	110.9316	0.004764	0.058326	0.081679	0.934902	NA
HSPG2	31.89766	-0.00476	0.08456	-0.05632	0.955086	NA
LIPF	0.335132	0.004762	0.032547	0.146311	0.883676	NA
FAM20A	32.25186	-0.00476	0.075465	-0.06308	0.949702	NA
PHACTR4	234.6024	0.004752	0.042158	0.112718	0.910254	NA
ADAM8	11.39032	-0.00475	0.083517	-0.05687	0.954645	NA
PMEL	9.674824	-0.00475	0.084921	-0.05593	0.9554	NA
COPS6	163.4243	0.004734	0.058594	0.080797	0.935604	NA
DYTN	0.148997	-0.00473	0.018094	-0.26158	0.793649	NA
FGGY	144.0802	-0.00473	0.062187	-0.07604	0.939385	NA
RERE	1229.111	0.004726	0.032809	0.14404	0.885469	0.963665
CALU	192.3433	-0.00472	0.044943	-0.10512	0.91628	NA
LOC64373	0.855094	-0.00472	0.051225	-0.09219	0.926544	NA
FAM216A	87.49837	0.004722	0.05895	0.080106	0.936153	NA
RABGGTB	121.2216	-0.00472	0.059653	-0.07906	0.936981	NA
LOC10065	0.071815	0.004716	0.014067	0.335266	0.737424	NA
KRT19P2	0.254381	0.004715	0.030839	0.152881	0.878492	NA
BTBD10	205.4246	0.004714	0.048591	0.097018	0.922712	NA

SNRPC	68.3895	0.004706	0.065554	0.071796	0.942764	NA
ARID4A	608.7757	0.004706	0.044181	0.106514	0.915174	0.973178
LOC28333	16.15044	-0.0047	0.083557	-0.05629	0.955115	NA
LOC10028	26.01044	0.004699	0.084851	0.055375	0.95584	NA
NCLN	128.0981	-0.0047	0.048063	-0.09774	0.922136	NA
STAG2	923.3012	0.004696	0.040125	0.117032	0.906834	0.971866
SLC6A18	0.049622	0.004685	0.011885	0.394243	0.693402	NA
RAVER2	203.1444	-0.00468	0.04288	-0.10917	0.913067	NA
OR52E6	0.089573	-0.00468	0.016605	-0.28174	0.77814	NA
C8orf58	57.6039	0.004676	0.082158	0.056921	0.954608	NA
STH	26.24418	0.004671	0.077054	0.060616	0.951665	NA
PPA1	194.4534	-0.00467	0.070296	-0.06641	0.94705	NA
SLFN14	0.049315	0.004668	0.011863	0.393512	0.693941	NA
C11orf65	2.239447	0.004661	0.071558	0.065129	0.948071	NA
HTR2C	0.504371	-0.00466	0.039217	-0.11884	0.905403	NA
PLBD2	97.9321	-0.00466	0.052107	-0.08942	0.928744	NA
ZNF670-ZN	0.117865	0.004659	0.017905	0.260215	0.794698	NA
ENO4	19.41987	-0.00466	0.08217	-0.05669	0.954789	NA
CLEC18A	1.302377	-0.00465	0.053593	-0.08682	0.930815	NA
LOC33986	0.073654	-0.00465	0.016008	-0.29052	0.771419	NA
B3GNT3	0.127809	-0.00465	0.020894	-0.22249	0.82393	NA
GRHPR	72.66771	-0.00465	0.055157	-0.08426	0.932847	NA
DDHD1	492.4231	-0.00464	0.042185	-0.10997	0.912431	0.972087
CD48	0.503455	-0.00464	0.038938	-0.11905	0.905234	NA
TTTY10	6.74542	0.00463	0.020374	0.227249	0.82023	NA
OR13C3	0.070744	-0.00462	0.015959	-0.28973	0.772021	NA
NKX2-4	0.192962	0.004614	0.026011	0.177375	0.859214	NA
LOC10063	0.087493	-0.00461	0.016487	-0.27969	0.779713	NA
MIR302C	0.327553	-0.00461	0.032911	-0.13999	0.888667	NA
VPS13A-AS	0.199563	0.004607	0.02605	0.176836	0.859637	NA
CLDN16	0.28421	-0.0046	0.029455	-0.15632	0.875779	NA
ZNF22	36.67121	-0.0046	0.071875	-0.06403	0.948946	NA
PRM2	0.071221	0.004598	0.013884	0.331158	0.740525	NA
SPG11	471.0061	0.004597	0.040255	0.114201	0.909078	0.971866
MIR4716	0.071678	-0.00459	0.015901	-0.28879	0.772744	NA
LOC10012	4.590606	0.004591	0.078213	0.058699	0.953192	NA
YPEL5	274.9595	0.00459	0.061657	0.07445	0.940652	NA
RAD51L3-	0.087047	-0.00458	0.016437	-0.27883	0.780378	NA
WNT10A	0.086807	-0.00458	0.016429	-0.27869	0.780481	NA
LOC25735	0.196098	0.004576	0.026018	0.175889	0.860381	NA
ZBTB45	36.3348	0.004571	0.069722	0.065564	0.947725	NA
NPY5R	0.45196	-0.00457	0.037575	-0.12161	0.903207	NA
STX8	60.18397	-0.00457	0.065189	-0.07007	0.94414	NA
SERPINB9	81.69938	0.004566	0.065201	0.070037	0.944164	NA
SPERT	0.068728	0.004566	0.013835	0.330009	0.741393	NA
NPW	0.137522	-0.00455	0.021032	-0.21646	0.828629	NA
BANCR	0.08358	-0.00455	0.016322	-0.27883	0.780376	NA

OVOL2	0.067414	-0.00455	0.015821	-0.2875	0.773729	NA
LOC10050	0.08359	-0.00455	0.016374	-0.27772	0.781224	NA
TMSB15A	0.106768	0.004545	0.019176	0.237012	0.812648	NA
C2orf27B	0.058676	-0.00454	0.011766	-0.38548	0.699882	NA
CCRL1	9.635449	0.004534	0.084848	0.053442	0.957379	NA
MIR4646	0.083329	-0.00453	0.01635	-0.27732	0.781536	NA
GGTA1P	2.619911	-0.00453	0.074328	-0.06098	0.951375	NA
FLJ27354	1.889798	0.004527	0.06533	0.06929	0.944759	NA
MYB	0.296958	-0.00452	0.028001	-0.16153	0.871673	NA
KRT86	1.661171	-0.00452	0.057836	-0.0782	0.937671	NA
PRKXP1	40.66096	0.004521	0.073809	0.061256	0.951155	NA
HIST1H2B	80.93844	0.004512	0.083524	0.054023	0.956917	NA
MRPS22	110.7628	0.004511	0.052819	0.085413	0.931933	NA
DPPA2P3	0.066108	-0.00451	0.015748	-0.28631	0.774639	NA
MIR374B	0.159406	-0.00451	0.021418	-0.21051	0.83327	NA
SMCP	0.434808	-0.00451	0.036059	-0.12494	0.900568	NA
DYNLT1	23.75822	-0.00451	0.074228	-0.06069	0.951602	NA
STAG3L4	63.01233	-0.0045	0.058829	-0.07655	0.938983	NA
MIR4470	0.082516	-0.0045	0.016287	-0.27621	0.782383	NA
OR52N2	0.231164	0.004497	0.024935	0.180339	0.856886	NA
TSHB	0.081409	-0.00449	0.01628	-0.2761	0.782474	NA
ARMS2	0.23105	-0.00449	0.028415	-0.15802	0.874445	NA
HS3ST3A1	0.072022	-0.00448	0.015928	-0.28149	0.778337	NA
RTFDC1	175.6105	0.004482	0.054793	0.081803	0.934803	NA
TNRC6C	778.7739	-0.00448	0.036923	-0.12128	0.903472	0.971222
CDH1	1.028483	0.004478	0.053184	0.084196	0.932901	NA
MIR3684	0.073022	-0.00448	0.015937	-0.28096	0.778745	NA
OR2D3	0.944432	0.004476	0.052371	0.08546	0.931896	NA
SIGLEC11	1.195918	-0.00447	0.052529	-0.08514	0.932148	NA
C11orf86	0.080056	-0.00447	0.016237	-0.27536	0.783042	NA
SPEF2	63.86976	0.004469	0.080469	0.05554	0.955708	NA
ZNF438	72.44139	0.004468	0.063798	0.070031	0.944169	NA
THEG	0.045791	0.004466	0.011604	0.384863	0.700339	NA
LINC00254	0.06324	-0.00447	0.015667	-0.285	0.775645	NA
NRON	16.37914	0.004462	0.082465	0.054108	0.956849	NA
MT1DP	0.080099	-0.00446	0.016216	-0.27499	0.783324	NA
ANKRD30A	0.078255	-0.00446	0.01615	-0.27588	0.782644	NA
NCR1	0.079227	-0.00445	0.016202	-0.27475	0.783506	NA
MC1R	50.27193	-0.00445	0.081763	-0.05443	0.956591	NA
TFAP2E	93.78624	-0.00445	0.074503	-0.05972	0.952376	NA
TMEM247	0.0799	-0.00445	0.016196	-0.27463	0.783598	NA
BCRP2	3.522853	-0.00445	0.061888	-0.07185	0.942718	NA
DNAJB9	84.33158	0.004445	0.066809	0.066537	0.94695	NA
LOC10014	0.076325	-0.00444	0.016187	-0.27448	0.783717	NA
LOC10099	0.481844	0.00444	0.035937	0.123563	0.901661	NA
C9orf43	7.035922	0.00444	0.083836	0.052962	0.957762	NA
TAAR5	0.070534	-0.00443	0.015837	-0.27998	0.779494	NA

LOC44029	0.076947	-0.00443	0.016171	-0.2742	0.78393	NA
FOLR1	0.465046	0.004434	0.039832	0.111314	0.911367	NA
SNORD36B	1.709022	-0.00443	0.065227	-0.06796	0.94582	NA
CYTL1	0.934336	0.004431	0.046232	0.095846	0.923643	NA
SNX31	10.84459	0.004431	0.071682	0.061811	0.950713	NA
PPARA	114.4741	0.004431	0.068972	0.064238	0.94878	NA
GOLGA6L1	0.051054	-0.00443	0.01163	-0.38097	0.703228	NA
MFHAS1	118.0263	0.00443	0.05038	0.08794	0.929924	NA
DNAJC5G	0.078961	-0.00443	0.016162	-0.27405	0.784045	NA
GATSL1	0.07753	-0.00443	0.016161	-0.27404	0.784056	NA
MIR492	0.055088	-0.00443	0.011624	-0.38077	0.703373	NA
ANGPTL6	3.205728	0.004425	0.074654	0.05927	0.952737	NA
OR6B3	0.075948	-0.00442	0.016147	-0.27379	0.784248	NA
FAM22F	0.260418	-0.00442	0.02325	-0.19013	0.849208	NA
MROH2B	0.068891	-0.00442	0.015807	-0.27947	0.779886	NA
HOXA13	0.050287	-0.00442	0.009555	-0.46229	0.64387	NA
DNAJC8	184.8735	0.004414	0.055549	0.079459	0.936668	NA
LOC72821	0.04489	0.004413	0.011536	0.382567	0.702041	NA
MIR4298	0.073128	-0.00441	0.01613	-0.27349	0.784478	NA
PPIG	818.3067	0.004408	0.045132	0.097669	0.922195	0.977889
CPSF6	980.9345	-0.00441	0.034577	-0.12747	0.898565	0.970091
CNBD1	0.044743	0.004405	0.011525	0.382191	0.70232	NA
ENPP7	0.077349	-0.0044	0.016117	-0.27327	0.784645	NA
MIR4639	0.107393	0.004403	0.019188	0.229468	0.818505	NA
DSC3	0.076295	0.0044	0.011942	0.368442	0.712544	NA
MIR634	0.077366	-0.0044	0.016104	-0.27305	0.784818	NA
MIR4790	0.05514	-0.0044	0.011583	-0.37943	0.704367	NA
GOLGA8CP	0.044542	0.004393	0.011509	0.381673	0.702704	NA
CEBPZ	230.8401	0.004391	0.04243	0.103484	0.917579	NA
RMST	63.43509	-0.00439	0.080528	-0.05453	0.956517	NA
SULT1C3	0.074221	-0.00439	0.016088	-0.27277	0.785031	NA
MIR548AN	0.070498	-0.00439	0.016025	-0.27374	0.784282	NA
BATF	0.30936	-0.00439	0.029222	-0.15012	0.880672	NA
LOC28562	0.075785	-0.00438	0.016077	-0.27258	0.785178	NA
MIR4256	1.745331	0.004379	0.062938	0.069584	0.944525	NA
EFNB3	4.717312	0.004378	0.077896	0.056202	0.955181	NA
KIR3DL2	0.044256	0.004376	0.011487	0.380931	0.703254	NA
CGB	0.075926	-0.00438	0.016064	-0.27235	0.78535	NA
MIR708	0.065761	-0.00437	0.015727	-0.27815	0.780894	NA
PSMB2	95.02599	-0.00437	0.050547	-0.08646	0.931104	NA
SPATA24	4.384792	0.004369	0.08008	0.054555	0.956493	NA
ZNF844	194.5878	0.004367	0.063508	0.06876	0.945181	NA
TNMD	0.072323	-0.00436	0.01604	-0.27193	0.78568	NA
RNU6-76	0.043994	0.00436	0.011467	0.380252	0.703758	NA
A4GALT	4.453866	0.004359	0.076107	0.057276	0.954325	NA
MT1H	0.075082	-0.00436	0.016032	-0.2718	0.785774	NA
CXorf38	50.10795	-0.00435	0.062427	-0.06975	0.944396	NA

TNS3	223.1558	0.004353	0.070589	0.061672	0.950824	NA
DUPD1	0.043816	0.00435	0.011453	0.379787	0.704103	NA
B4GALNT4	265.6516	0.004349	0.074663	0.058252	0.953548	NA
PPP1R2	109.1	0.004349	0.073325	0.059308	0.952707	NA
DYNC1LI1	215.4955	0.004348	0.043842	0.099183	0.920993	NA
HS6ST2-AS	0.481322	-0.00435	0.039574	-0.10985	0.912526	NA
MYL1	0.156107	0.004347	0.019784	0.219718	0.826091	NA
FAM71B	0.073412	-0.00435	0.016013	-0.27146	0.786037	NA
ZNF732	15.53687	-0.00434	0.075756	-0.05726	0.954342	NA
RFPL4B	0.073632	-0.00434	0.015995	-0.27115	0.786273	NA
CXorf65	0.071995	-0.00434	0.015995	-0.27115	0.786276	NA
UBE2R2	199.6932	-0.00433	0.050099	-0.08646	0.931105	NA
OR1J1	0.043502	0.004331	0.011428	0.378962	0.704716	NA
CYP19A1	0.071735	-0.00432	0.01597	-0.27072	0.78661	NA
PALM2-AK	0.07116	-0.00432	0.015968	-0.27069	0.786631	NA
KCNK17	0.069822	-0.00432	0.015907	-0.27172	0.785836	NA
MICAL3	2581.495	-0.00432	0.062238	-0.06939	0.944676	0.987397
KRTAP11-1	0.043283	0.004318	0.011411	0.378387	0.705143	NA
SCGB3A1	0.043236	0.004315	0.011407	0.378261	0.705237	NA
OR1E2	0.070856	-0.00431	0.015949	-0.27036	0.786883	NA
OR51I2	0.58743	-0.00431	0.037354	-0.11535	0.908165	NA
THEG5	0.043133	0.004309	0.011399	0.37799	0.705438	NA
OR5AK2	0.071995	-0.00431	0.015942	-0.27024	0.786978	NA
CSDAP1	0.070457	-0.00431	0.015879	-0.27125	0.786202	NA
ITK	0.447043	-0.00431	0.034723	-0.12402	0.901303	NA
PPM1M	31.97611	-0.0043	0.076765	-0.05606	0.955296	NA
MIR298	0.042977	0.004299	0.011387	0.377574	0.705747	NA
MIR4282	0.042977	0.004299	0.011387	0.377574	0.705747	NA
CABP5	0.052051	-0.0043	0.011454	-0.37517	0.707533	NA
OR5E1P	0.042924	0.004296	0.011382	0.377434	0.705851	NA
LOC10050	0.042924	0.004296	0.011382	0.377434	0.705851	NA
OR13C8	0.042924	0.004296	0.011382	0.377434	0.705851	NA
CNR2	0.108062	0.004294	0.019201	0.223632	0.823044	NA
TEX12	3.753379	-0.00429	0.080204	-0.05351	0.957328	NA
PRSS37	7.546348	0.004291	0.083947	0.05111	0.959238	NA
NXPH1	30.56094	-0.00429	0.084424	-0.05082	0.959471	NA
LOC10050	12.70559	0.004289	0.083397	0.051431	0.958982	NA
LOC33962	0.042725	0.004284	0.011367	0.376904	0.706245	NA
AFP	0.070963	-0.00428	0.015896	-0.26944	0.787592	NA
ELOF1	35.69678	-0.00428	0.067963	-0.06298	0.949783	NA
LOC25357	0.130002	-0.00428	0.020925	-0.20454	0.837929	NA
FLJ12825	1.853936	-0.00428	0.066051	-0.0648	0.948337	NA
MIR323A	0.446496	0.004278	0.039001	0.109701	0.912647	NA
MIR548X	0.068378	-0.00427	0.015874	-0.26905	0.787893	NA
ADCY9	409.3946	-0.00427	0.048245	-0.08844	0.929524	0.981525
CHSY3	0.82407	-0.00427	0.045652	-0.09344	0.925555	NA
LOC72791	0.201419	0.004265	0.027013	0.157888	0.874545	NA

PANX2	183.4673	-0.00426	0.054415	-0.0783	0.937587	NA
ENTPD6	285.2401	0.004261	0.051098	0.083387	0.933544	NA
MRPL1	33.93404	0.004261	0.072278	0.058947	0.952994	NA
FGF6	0.042281	0.004257	0.011331	0.375716	0.707128	NA
MIR3605	2.48778	-0.00426	0.071304	-0.05968	0.952413	NA
OR2AK2	4.124688	0.004254	0.044118	0.096426	0.923182	NA
MCM3AP	578.1321	-0.00425	0.035791	-0.11884	0.9054	0.971866
LY6G6E	0.042139	0.004249	0.01132	0.375332	0.707413	NA
MIR3621	0.069385	-0.00425	0.015832	-0.26832	0.788451	NA
KIAA1161	98.10737	-0.00425	0.07711	-0.05509	0.956069	NA
MIR4450	0.042053	0.004243	0.011313	0.375102	0.707585	NA
RBP4	0.129613	-0.00424	0.019579	-0.21664	0.828485	NA
FCRL3	0.066528	-0.00424	0.015815	-0.26804	0.788671	NA
PMCHL1	0.826388	0.004238	0.042977	0.09862	0.92144	NA
PEX10	20.42651	0.004233	0.078435	0.053971	0.956958	NA
LOC28445	2.392112	-0.00423	0.066213	-0.06393	0.949024	NA
DIDO1	701.3584	0.004224	0.055309	0.076366	0.939128	0.984229
C12orf39	10.6988	-0.00422	0.081893	-0.05154	0.958896	NA
CLCN5	152.5861	-0.00422	0.061744	-0.06832	0.94553	NA
MIR4531	0.347664	-0.00422	0.029014	-0.14538	0.884414	NA
MALL	0.382665	-0.00422	0.033124	-0.12728	0.898719	NA
ASAH1	321.2045	-0.00421	0.060498	-0.06953	0.944565	NA
F12	0.210247	-0.0042	0.026318	-0.15976	0.873072	NA
PCDHB1	0.306115	0.004199	0.031705	0.132442	0.894635	NA
KL	1.257633	-0.0042	0.050945	-0.08238	0.934341	NA
LOC49414	0.041049	0.004182	0.011231	0.372366	0.70962	NA
TTC5	55.88016	-0.00418	0.067113	-0.06229	0.950331	NA
DUSP13	0.041	0.004179	0.011227	0.372231	0.709721	NA
ANHX	0.061935	0.004177	0.013718	0.304523	0.760729	NA
CSF2RB	2.695779	0.004172	0.068938	0.060526	0.951737	NA
C16orf72	205.2169	0.004169	0.04575	0.091133	0.927387	NA
MIR1915	0.040809	0.004167	0.011211	0.371703	0.710114	NA
SIGLECL1	0.562264	-0.00417	0.037083	-0.11234	0.910555	NA
UTRN	275.9753	0.004166	0.056072	0.074295	0.940776	NA
MIR769	0.34376	-0.00416	0.03173	-0.13124	0.895587	NA
ZNF738	141.2177	0.004157	0.070293	0.059143	0.952838	NA
ZSWIM2	0.061211	0.004155	0.013682	0.303668	0.761381	NA
DEFB108B	0.147327	-0.00415	0.021166	-0.19626	0.844407	NA
SNAR-E	0.040533	0.00415	0.011188	0.37094	0.710682	NA
LINC00558	0.040518	0.004149	0.011187	0.370899	0.710713	NA
LPA	0.040518	0.004149	0.011187	0.370899	0.710713	NA
KCNQ1	2.244503	-0.00415	0.067305	-0.06164	0.950853	NA
LOC10052	58.05458	-0.00415	0.078653	-0.05273	0.957948	NA
KLHL7	158.3195	-0.00414	0.047981	-0.08636	0.931181	NA
HSPB2-C11	2.326471	-0.00414	0.070352	-0.05887	0.953054	NA
KCNJ2	18.87831	-0.00414	0.084892	-0.04879	0.961088	NA
MIR4760	0.040313	0.004137	0.01117	0.370326	0.71114	NA

CBFA2T2	297.1424	0.004131	0.044124	0.093622	0.92541	NA
LOC72944	0.233401	0.004129	0.024951	0.165502	0.868549	NA
SCD	982.3279	0.004128	0.077627	0.053177	0.957591	0.991078
FAM127A	127.5768	0.004128	0.069813	0.059126	0.952852	NA
ADD1	1136.472	-0.00413	0.026864	-0.15359	0.877936	0.959988
MTL5	2.090355	0.004118	0.060435	0.068139	0.945675	NA
REXO1L1	0.039925	0.004112	0.011138	0.369242	0.711948	NA
KRTAP10-1	0.039875	0.004109	0.011133	0.3691	0.712053	NA
OR1B1	0.112629	0.004108	0.017838	0.230297	0.817861	NA
EBNA1BP2	84.63599	0.004104	0.062445	0.06573	0.947593	NA
LOC64575	25.60081	-0.0041	0.08489	-0.04828	0.961491	NA
DGCR5	540.7908	-0.00409	0.067185	-0.06095	0.951401	0.990769
MIR4498	0.10201	0.004092	0.017651	0.231849	0.816655	NA
MEMO1	32.61449	0.004082	0.070112	0.058221	0.953572	NA
C7orf72	0.120048	0.004077	0.019439	0.209724	0.833883	NA
BMS1P4	71.30899	-0.00407	0.059233	-0.06873	0.945205	NA
MIR1231	0.133716	0.004062	0.019708	0.206103	0.836711	NA
ADNP2	102.1603	0.00406	0.054635	0.07432	0.940756	NA
MYO18B	1.427512	-0.00406	0.060105	-0.0675	0.946185	NA
ARMCX5-G	9.135357	-0.00405	0.084713	-0.04783	0.961853	NA
IQGAP2	8.653477	-0.00405	0.07792	-0.05199	0.958537	NA
C1QL3	51.01207	-0.00405	0.084859	-0.04768	0.961968	NA
MIR9-3	0.215394	0.004045	0.027109	0.149209	0.881388	NA
MIR4684	0.038848	0.004045	0.011046	0.366182	0.714229	NA
FAM92A1	0.038848	0.004045	0.011046	0.366182	0.714229	NA
POU4F2	0.277301	-0.00404	0.029443	-0.13732	0.890777	NA
SDHAP3	72.04824	-0.00404	0.072364	-0.05584	0.955469	NA
FBXO21	438.9383	-0.00404	0.049203	-0.08209	0.934576	0.984229
BMP6	8.839223	-0.00404	0.082001	-0.04925	0.960718	NA
GDPD4	0.996118	-0.00403	0.043263	-0.0932	0.925747	NA
ELF2	238.9217	-0.00403	0.046404	-0.0868	0.930831	NA
H2BFM	0.075718	-0.00403	0.016222	-0.24822	0.803964	NA
EFCAB2	107.7627	0.004021	0.06619	0.060753	0.951556	NA
ZNF432	94.45115	-0.00402	0.05942	-0.06767	0.946051	NA
SPIN2A	10.22064	-0.00402	0.084402	-0.04761	0.962029	NA
POLR1D	124.8984	-0.00402	0.051447	-0.0781	0.937746	NA
LOC39223	0.038388	0.004016	0.011006	0.364854	0.71522	NA
PGK2	0.045732	0.004015	0.009074	0.442532	0.658104	NA
OR2F2	0.03829	0.00401	0.010998	0.364572	0.715431	NA
MIR205HG	0.038284	0.004009	0.010997	0.364552	0.715446	NA
CHRNA1	0.104676	0.004005	0.019143	0.209231	0.834268	NA
DPCR1	0.038124	0.003999	0.010983	0.364088	0.715792	NA
CRIP3	15.89587	-0.004	0.080767	-0.04949	0.960526	NA
LOC10050	0.0381	0.003997	0.010981	0.364018	0.715845	NA
CYLC2	0.0381	0.003997	0.010981	0.364018	0.715845	NA
C21orf88	1.385587	0.003989	0.059956	0.066533	0.946954	NA
PP2D1	9.829194	-0.00398	0.08472	-0.047	0.962512	NA

MIR3921	0.037803	0.003978	0.010955	0.36315	0.716493	NA
WFDC10B	0.23749	0.003976	0.023349	0.170296	0.864777	NA
MIR1305	0.071013	-0.00398	0.01613	-0.24647	0.80532	NA
WNT1	0.037575	0.003976	0.010951	0.363018	0.716592	NA
LCE1A	0.043987	-0.00397	0.011017	-0.36074	0.718292	NA
MIR5008	0.117714	0.003974	0.0179	0.222003	0.824311	NA
HNRNPUL	491.709	-0.00397	0.036611	-0.10841	0.913669	0.972815
GPR162	169.9898	-0.00397	0.055699	-0.07124	0.943203	NA
RNF157	261.3076	-0.00397	0.055041	-0.07208	0.942541	NA
RNU86	0.648573	0.003959	0.044124	0.089731	0.928501	NA
TERT	0.03747	0.003957	0.010926	0.36217	0.717225	NA
MIR4322	0.037389	0.003952	0.010919	0.361932	0.717402	NA
LRPAP1	507.7829	-0.00395	0.052403	-0.07539	0.939906	0.984229
TNNT3	0.03736	0.00395	0.010916	0.361845	0.717468	NA
SNAR-G1	0.037311	0.003947	0.010912	0.361699	0.717577	NA
SPRYD3	280.3463	0.003944	0.065151	0.060541	0.951725	NA
EIF3IP1	0.037266	0.003944	0.010908	0.361567	0.717675	NA
ATP5EP2	0.125407	-0.00394	0.017932	-0.21986	0.825984	NA
CPEB3	395.2126	-0.00394	0.034184	-0.11532	0.90819	0.971866
LOC10028	0.359769	-0.00394	0.033206	-0.1187	0.905517	NA
SAG	0.037205	0.00394	0.010903	0.361385	0.717811	NA
PTOV1-AS	52.41	0.003938	0.075539	0.052133	0.958423	NA
OR52H1	0.206733	0.003937	0.026082	0.15095	0.880015	NA
PTPN14	58.28213	-0.00394	0.072788	-0.05409	0.956865	NA
FXYD6	140.9993	0.003936	0.06256	0.062908	0.94984	NA
STRA6	0.184048	0.003933	0.023389	0.168178	0.866443	NA
MIR150	0.037019	0.003928	0.010886	0.360834	0.718224	NA
LINC00606	0.036939	0.003923	0.010879	0.360594	0.718403	NA
MIR938	0.03688	0.003919	0.010874	0.36042	0.718533	NA
SYT14L	0.036874	0.003919	0.010873	0.360399	0.718549	NA
TEPP	0.109536	0.003919	0.017807	0.220067	0.825819	NA
LOC10012	0.361042	-0.00392	0.031647	-0.12379	0.90148	NA
MIR5582	0.447648	0.003915	0.038579	0.101483	0.919167	NA
ABCC8	399.2456	-0.00391	0.06866	-0.057	0.954543	0.991078
LINC00113	0.036638	0.003903	0.010852	0.359694	0.719076	NA
LINC00520	3.163317	0.003902	0.066589	0.058594	0.953276	NA
KLK2	0.036608	0.003901	0.010849	0.359604	0.719143	NA
IL31RA	0.03659	0.0039	0.010848	0.35955	0.719184	NA
IGLL5	0.041319	-0.0039	0.008979	-0.43424	0.664114	NA
SOX8	44.72076	0.003899	0.079383	0.04911	0.960831	NA
OR7E5P	0.036543	0.003897	0.010843	0.359406	0.719291	NA
MLF1	31.92818	-0.0039	0.081717	-0.04769	0.961965	NA
TMEM154	1.368975	-0.00389	0.060256	-0.06464	0.94846	NA
FAM24A	0.212546	0.003894	0.026118	0.149081	0.881489	NA
FZD2	0.470701	-0.00389	0.04093	-0.09511	0.924228	NA
ANKRD33B	13.14876	-0.00389	0.084266	-0.04619	0.963156	NA
SOX18	14.95379	-0.00389	0.084214	-0.04622	0.963138	NA

LOC15869	0.217759	-0.00389	0.028341	-0.1372	0.890876	NA
RPS10P7	1.571861	0.003875	0.064964	0.059649	0.952435	NA
CAPZA2	243.3587	-0.00387	0.076112	-0.05089	0.959413	NA
RPL22L1	14.63675	0.00387	0.08431	0.045904	0.963387	NA
TIAM1	4671.987	0.003869	0.036904	0.10484	0.916503	0.973677
PDPR	337.7089	-0.00387	0.082708	-0.04676	0.962702	0.991078
MTA2	142.2252	-0.00387	0.059251	-0.06524	0.94798	NA
GABRR3	0.03606	0.003866	0.0108	0.357947	0.720383	NA
IFNA7	0.127581	0.003865	0.019598	0.197212	0.843662	NA
TMEM71	0.544386	-0.00386	0.037091	-0.1042	0.917012	NA
TAS2R3	23.74715	-0.00386	0.07828	-0.04936	0.960632	NA
LGALS12	0.104444	0.003859	0.019154	0.201474	0.840328	NA
CEACAM1	0.035942	0.003858	0.010789	0.357588	0.720652	NA
ACTR1B	129.2197	-0.00385	0.071784	-0.0537	0.957178	NA
LOC10028	9.476082	-0.00385	0.0846	-0.04556	0.963661	NA
RBM20	5.060791	-0.00385	0.08004	-0.04813	0.961616	NA
LOC10050	11.67477	0.003849	0.084904	0.04533	0.963844	NA
SNX4	97.38023	-0.00385	0.069996	-0.05497	0.956159	NA
MIR1469	0.03577	0.003847	0.010773	0.357062	0.721046	NA
CXADRP2	0.106228	0.003844	0.015911	0.241621	0.809074	NA
MTM1	66.81674	-0.00384	0.062023	-0.06198	0.950577	NA
FCGR1B	1.350723	-0.00384	0.061051	-0.06295	0.949803	NA
SH2D3A	1.069149	-0.00384	0.048978	-0.07847	0.937453	NA
FSCN2	15.72981	0.003843	0.084751	0.04534	0.963836	NA
MAMDC2	16.49732	0.003839	0.082253	0.046678	0.96277	NA
PRKCI	187.9904	-0.00384	0.051219	-0.07495	0.940258	NA
ARHGEF3-	0.035581	0.003834	0.010756	0.356484	0.721478	NA
ZFHX4-AS1	0.752095	-0.00383	0.046761	-0.0819	0.934727	NA
VPS11	151.4936	-0.00382	0.049013	-0.07804	0.937799	NA
LINC00470	0.574387	0.003823	0.040016	0.095544	0.923883	NA
CAPN3	82.16917	-0.00382	0.07671	-0.04981	0.960274	NA
SYT8	0.036145	-0.00382	0.008888	-0.42982	0.667328	NA
CGB2	0.030866	-0.00382	0.008888	-0.42982	0.667328	NA
PAX7	0.031172	-0.00382	0.008888	-0.42982	0.667328	NA
LOC64626	0.034216	-0.00382	0.008888	-0.42982	0.667328	NA
SNORA71B	3.459643	-0.00382	0.074199	-0.05147	0.95895	NA
MIR5009	0.035116	0.003815	0.010728	0.355565	0.722166	NA
C16orf95	12.63965	-0.00381	0.084838	-0.04496	0.964137	NA
KRT1	0.035118	0.003804	0.010713	0.355054	0.722549	NA
MIR4515	0.035118	0.003804	0.010713	0.355054	0.722549	NA
TMEM126	26.45167	-0.0038	0.074185	-0.05119	0.959171	NA
LOC20077	0.541159	-0.00379	0.039544	-0.09593	0.923579	NA
GPR112	0.034905	0.00379	0.010693	0.354393	0.723045	NA
DEFB109P	1.595951	-0.00379	0.056634	-0.06684	0.946709	NA
DCSTAMP	0.034818	0.003784	0.010685	0.354119	0.72325	NA
CCNC	211.2503	-0.00378	0.049665	-0.07618	0.939279	NA
RAB36	21.32055	-0.00378	0.076623	-0.04936	0.960629	NA

MAPK8IP1	348.6596	0.00378	0.047267	0.079978	0.936255	0.984229
LOC10013	41.80745	0.003779	0.08231	0.045911	0.963381	NA
TBCD	333.328	-0.00378	0.062948	-0.05998	0.952172	NA
TPSAB1	0.034683	0.003775	0.010673	0.353698	0.723566	NA
AIFM3	124.1161	0.003772	0.069145	0.054557	0.956491	NA
ARMC8	718.6868	0.003771	0.030061	0.125444	0.900172	0.970824
ATP8B3	1.877633	0.00377	0.065313	0.057721	0.953971	NA
PCDH11Y	0.466617	0.003768	0.034523	0.109138	0.913093	NA
GCG	0.034497	0.003762	0.010655	0.353115	0.724002	NA
CDK2AP1	124.1785	-0.00376	0.078429	-0.04795	0.96176	NA
ATP6V1B2	348.7193	0.00376	0.063797	0.058932	0.953006	0.991074
OR6M1	0.034455	0.00376	0.010651	0.352981	0.724103	NA
HDC	0.38545	-0.00376	0.034641	-0.10852	0.913581	NA
KRTAP22-1	0.034362	0.003753	0.010642	0.352688	0.724322	NA
WDR61	64.42767	0.003753	0.062816	0.059744	0.95236	NA
RXFP1	0.03435	0.003753	0.010641	0.352651	0.72435	NA
CA5A	0.310698	0.003753	0.029283	0.128146	0.898033	NA
CRB3	0.46402	-0.00375	0.040037	-0.09368	0.925362	NA
OTUB1	133.4875	-0.00375	0.052302	-0.07164	0.942885	NA
LOC10013	0.034137	0.003738	0.010621	0.351976	0.724857	NA
LSP1P3	0.034096	0.003736	0.010617	0.351846	0.724954	NA
NSUN2	163.9962	0.003736	0.060584	0.06166	0.950834	NA
IL19	0.034062	0.003733	0.010614	0.351739	0.725034	NA
CNTNAP3	18.77736	0.003733	0.067552	0.055256	0.955934	NA
LIPM	0.034041	0.003732	0.010612	0.351672	0.725084	NA
CCDC130	76.61799	-0.00373	0.060652	-0.06151	0.950954	NA
DTWD2	58.38813	-0.00373	0.065063	-0.05727	0.954331	NA
PLEKHG3	119.5078	0.003723	0.071086	0.052371	0.958233	NA
PPBP	0.212966	0.003722	0.027102	0.137343	0.89076	NA
TP63	0.853576	-0.00371	0.048234	-0.07699	0.938628	NA
OR52E2	0.033751	0.003712	0.010584	0.350747	0.725778	NA
LOC10050	4.801475	0.003705	0.080668	0.045924	0.963371	NA
ARX	0.167254	-0.0037	0.023043	-0.16074	0.872301	NA
POLR2J3	29.50998	0.003697	0.083824	0.0441	0.964825	NA
LINC00473	227.6589	-0.0037	0.083169	-0.04444	0.964552	NA
NUDT14	4.339634	-0.0037	0.074567	-0.04956	0.960473	NA
COPB1	264.3163	-0.00369	0.038948	-0.09487	0.92442	NA
MIR221	0.159583	0.003695	0.022718	0.16263	0.87081	NA
ATP10B	68.39982	-0.00369	0.077784	-0.04746	0.962144	NA
SLC31A1	95.06683	-0.00369	0.050745	-0.07275	0.942004	NA
LOC64417	14.15218	-0.00369	0.030559	-0.12074	0.903895	NA
MAD2L1	10.2964	0.003688	0.084812	0.043488	0.965313	NA
RP1L1	0.491716	-0.00369	0.038587	-0.09552	0.923905	NA
CCDC9	91.41991	-0.00368	0.059873	-0.06149	0.950965	NA
NRP1	13.88362	-0.00368	0.084844	-0.04336	0.965415	NA
HLA-DQB2	0.152199	0.003676	0.022943	0.160216	0.872711	NA
ARSK	33.26995	-0.00367	0.070689	-0.05188	0.958623	NA

MIR3188	0.033063	0.003666	0.010518	0.348531	0.727441	NA
ZDHHC21	737.5466	-0.00367	0.0323	-0.11348	0.90965	0.971866
OR1G1	0.321124	0.003661	0.03303	0.110849	0.911736	NA
LOC28402	45.31577	-0.00366	0.077807	-0.04703	0.962492	NA
EMILIN2	9.111254	0.003654	0.084439	0.043279	0.965479	NA
CTRB2	0.032797	0.003648	0.010492	0.347663	0.728093	NA
KCNQ1OT	1674.62	-0.00364	0.074989	-0.04861	0.961233	0.991078
TMEM125	2.084483	-0.00364	0.065678	-0.05548	0.955759	NA
P2RY14	56.77157	0.003642	0.071432	0.05098	0.959342	NA
FAM27B	0.064738	-0.00364	0.013622	-0.2673	0.789238	NA
MED23	316.6447	-0.00364	0.041599	-0.08746	0.930306	NA
ZSCAN25	87.34793	0.003636	0.054205	0.067087	0.946513	NA
GADD45GI	85.25101	-0.00363	0.064892	-0.05601	0.955335	NA
CARD17	0.032596	0.003634	0.010472	0.347005	0.728588	NA
POMGNT1	121.6636	0.00363	0.050931	0.071273	0.943181	NA
RNF126P1	0.097524	0.003628	0.01905	0.190458	0.84895	NA
HOXB2	0.901494	0.003628	0.046313	0.078331	0.937565	NA
KIAA0125	0.086328	0.003627	0.012329	0.294168	0.76863	NA
CLEC3A	0.032476	0.003626	0.01046	0.346609	0.728885	NA
MIR2116	0.131949	-0.00362	0.020973	-0.17281	0.862803	NA
ARHGAP23	434.6501	-0.00361	0.044522	-0.08111	0.935352	0.984229
PRELID1	20.57999	0.00361	0.080615	0.044776	0.964286	NA
SNORD123	0.032068	0.003608	0.010434	0.345744	0.729535	NA
DCUN1D4	191.4801	0.003607	0.045285	0.079641	0.936523	NA
MOS	0.032184	0.003606	0.010431	0.345643	0.729611	NA
TMSB4Y	4.167022	0.003603	0.023734	0.151811	0.879336	NA
IL24	0.031987	0.003602	0.010426	0.345475	0.729738	NA
DDX28	14.75857	0.003599	0.083791	0.042947	0.965743	NA
LOC10050	0.031945	0.003589	0.010408	0.344848	0.730209	NA
SLC35G3	0.136983	-0.00359	0.021047	-0.17052	0.864603	NA
TYRP1	2.492016	0.003581	0.064151	0.055821	0.955484	NA
ACTN2	41.23673	0.003577	0.083265	0.042961	0.965733	NA
OR2T12	0.088496	-0.00358	0.014538	-0.24594	0.805726	NA
KDM5D	122.1758	0.003571	0.016101	0.221791	0.824477	NA
FAM227B	9.723485	-0.00357	0.084937	-0.04203	0.966471	NA
MIR592	0.07213	-0.00357	0.01393	-0.25609	0.797882	NA
TPRXL	0.856587	-0.00356	0.04824	-0.07386	0.941123	NA
CHODL	1.704228	-0.00356	0.058915	-0.06047	0.95178	NA
PAIP2	502.0039	0.003562	0.062178	0.057294	0.954311	0.991078
TMEM132	82.92418	0.003561	0.084912	0.041942	0.966545	NA
SLC16A10	32.96196	-0.00356	0.0752	-0.04735	0.962231	NA
MAPRE3	458.4403	-0.00356	0.051015	-0.06969	0.944442	0.987397
TEAD1	191.9175	-0.00355	0.060573	-0.0586	0.953274	NA
CAPN6	0.206384	-0.00354	0.026135	-0.13554	0.892182	NA
HIST1H1B	3.404372	0.003538	0.067945	0.052073	0.95847	NA
LINC00552	0.371821	-0.00353	0.035543	-0.09942	0.920804	NA
OR7E14P	3.006913	-0.00353	0.075337	-0.04689	0.962598	NA

ENO3	16.5915	0.003522	0.083244	0.04231	0.966252	NA
SNAI3-AS1	28.97432	-0.00352	0.075302	-0.04674	0.962723	NA
PAX4	0.139886	-0.00352	0.021082	-0.16676	0.867559	NA
CACNG3	6.886748	0.003514	0.072514	0.048456	0.961353	NA
FLJ40194	0.164872	0.003511	0.021639	0.162244	0.871114	NA
LOC44089	0.12997	0.003509	0.021145	0.165939	0.868205	NA
LOC10050	0.08285	-0.00351	0.014397	-0.24352	0.807602	NA
OR13F1	0.136209	-0.0035	0.019667	-0.17805	0.858687	NA
ODF3L2	0.763131	-0.0035	0.046293	-0.07561	0.939732	NA
GMNN	51.64464	-0.0035	0.072874	-0.04802	0.961697	NA
C1orf200	0.285284	-0.00349	0.031355	-0.11144	0.911265	NA
RASA3	153.6994	-0.00349	0.056238	-0.06208	0.950499	NA
MIR377	0.25417	0.003489	0.026321	0.132549	0.89455	NA
MIR3678	0.030368	0.003488	0.010261	0.339951	0.733893	NA
CPB1	0.206407	0.003487	0.023439	0.14876	0.881743	NA
C2orf91	0.460722	-0.00349	0.03747	-0.09303	0.925878	NA
SLC6A2	0.03028	0.003482	0.010252	0.339646	0.734123	NA
RPL13AP1	12.92494	-0.00348	0.084919	-0.04097	0.96732	NA
ZHX2	127.0141	-0.00347	0.051506	-0.06736	0.946297	NA
PLEKHD1	88.0798	-0.00346	0.080025	-0.04328	0.965476	NA
CD38	2.566338	-0.00345	0.063328	-0.05453	0.956509	NA
TMEM160	24.18722	0.003445	0.078396	0.043938	0.964954	NA
EIF2B3	122.0177	0.003443	0.049691	0.069286	0.944762	NA
C1orf185	0.07596	-0.00344	0.014264	-0.24122	0.809388	NA
CLIP1	2285.242	-0.00344	0.040379	-0.0852	0.932104	0.982875
CLEC2A	1.001041	0.003439	0.024435	0.140754	0.888064	NA
LOC34876	0.078856	-0.00343	0.014235	-0.24073	0.809768	NA
MIR3065	0.080161	-0.00343	0.014232	-0.24068	0.809807	NA
IGJ	0.080087	-0.00342	0.01423	-0.24063	0.809844	NA
MIR885	1.037639	0.003422	0.052032	0.065762	0.947568	NA
C22orf28	117.2644	0.003416	0.052066	0.065617	0.947683	NA
TTTY15	41.24158	0.003416	0.018826	0.181472	0.855997	NA
AGAP6	58.83319	-0.00342	0.073166	-0.04669	0.962759	NA
RNF113A	45.49353	0.003409	0.0666	0.05118	0.959182	NA
PDE4C	2.516955	0.003407	0.071144	0.047892	0.961802	NA
CACNA1S	0.328365	0.003401	0.030684	0.110828	0.911753	NA
TMEM181	69.32673	-0.0034	0.063215	-0.05376	0.957127	NA
ARID4B	889.0645	-0.0034	0.03523	-0.09644	0.923173	0.978469
INSIG2	115.9372	0.003395	0.053715	0.063208	0.949601	NA
MAGEA12	0.07849	-0.00339	0.014169	-0.23958	0.810654	NA
LHX9	0.076133	-0.00339	0.014163	-0.23948	0.810737	NA
ESRP1	0.07673	-0.00339	0.014153	-0.2393	0.810871	NA
CHI3L2	1.265867	0.003387	0.026224	0.12914	0.897247	NA
LRP8	363.347	0.003375	0.062391	0.054101	0.956854	0.991078
FADD	60.29529	0.003371	0.061748	0.0546	0.956457	NA
SLIT2-IT1	0.072422	-0.00337	0.014118	-0.23871	0.811328	NA
MIR130B	0.114887	0.003368	0.019377	0.173815	0.862011	NA

ZC3H7A	279.6219	0.003366	0.034157	0.09855	0.921496	NA
LOC72872	0.076806	-0.00336	0.014104	-0.23846	0.811527	NA
CLCA4	3.428333	-0.00336	0.077159	-0.04357	0.965245	NA
LOC10042	0.067721	-0.00336	0.013751	-0.24419	0.807085	NA
VPS33A	83.01264	-0.00336	0.057972	-0.05788	0.953843	NA
MYO9B	585.3685	0.003354	0.064122	0.052303	0.958287	0.991078
NEURL1B	7.553227	-0.00335	0.084695	-0.03958	0.968427	NA
MIR4524B	0.13568	-0.00335	0.02101	-0.1593	0.873433	NA
LOC10013	65.19229	0.003346	0.07435	0.045009	0.9641	NA
B3GNT6	0.165073	0.003341	0.022779	0.14667	0.883392	NA
LAMB4	1.492996	0.003339	0.061241	0.054521	0.95652	NA
TOR1A	105.6663	0.003333	0.051258	0.065034	0.948147	NA
LDLRAD2	0.220286	-0.00333	0.027481	-0.12128	0.903471	NA
DVL2	98.39662	-0.00333	0.060183	-0.05536	0.955851	NA
LOC91948	0.665617	-0.00332	0.041958	-0.07922	0.936856	NA
TAAR6	0.028059	0.003321	0.010013	0.331674	0.740136	NA
MIR676	0.028059	0.003321	0.010013	0.331674	0.740136	NA
FLJ37035	6.653567	-0.00332	0.084775	-0.03912	0.968796	NA
DNAJC9	106.5785	-0.00331	0.060309	-0.05494	0.956185	NA
PAN3	558.7187	-0.00331	0.038724	-0.0855	0.931861	0.982875
CDC14B	42.13739	-0.00331	0.07633	-0.04335	0.965421	NA
CRABP2	12.73291	0.003303	0.084932	0.038895	0.968974	NA
ASUN	67.91197	-0.0033	0.059584	-0.05543	0.955792	NA
SMG6	528.6193	0.003298	0.03586	0.091981	0.926713	0.980561
PYY2	0.410446	0.003298	0.037192	0.088668	0.929346	NA
TBC1D2B	134.2376	-0.0033	0.055861	-0.059	0.952954	NA
HIST1H2A	48.0244	0.003293	0.084916	0.038785	0.969062	NA
SRD5A3-A	2.849158	-0.00329	0.068742	-0.04788	0.961809	NA
C10orf107	0.514633	-0.00329	0.038048	-0.08643	0.931125	NA
CDCP2	0.565196	0.003284	0.040263	0.081572	0.934987	NA
NPVF	0.070662	-0.00328	0.013937	-0.2356	0.813746	NA
MIR548J	0.193301	0.003281	0.026958	0.12172	0.903121	NA
LOC10012	1.547515	-0.00328	0.060494	-0.05423	0.956748	NA
GRB10	341.1648	0.003278	0.041516	0.07895	0.937073	0.984229
PDGFRB	85.55636	-0.00327	0.075373	-0.04345	0.965344	NA
LINC00471	13.36571	0.003273	0.084638	0.038676	0.969149	NA
PTGES2	108.0668	-0.00327	0.059422	-0.05504	0.956107	NA
ARL6IP4	373.7174	-0.00326	0.043067	-0.07566	0.939693	0.984229
ZHX3	782.3391	0.003257	0.041614	0.078263	0.937619	0.984229
ACTN1-AS	0.066854	-0.00325	0.013865	-0.23436	0.814708	NA
MIR4718	0.139318	-0.00325	0.021086	-0.15393	0.877665	NA
C2orf54	0.14085	-0.00324	0.019777	-0.16402	0.869715	NA
RHEB	54.9442	0.003238	0.071533	0.04527	0.963892	NA
MIR365B	0.21518	0.003232	0.027113	0.119211	0.905108	NA
GPX4	322.9568	-0.00323	0.074302	-0.04343	0.965358	NA
SCAF1	300.994	0.003225	0.04391	0.073454	0.941445	NA
UTP15	69.37288	0.00322	0.058706	0.054851	0.956258	NA

LRRC16A	390.0496	0.003217	0.048747	0.066001	0.947377	0.988852
PROK1	0.615018	0.003208	0.046213	0.069411	0.944662	NA
SOX4	73.65254	0.003205	0.074712	0.042905	0.965778	NA
RERGL	3.630585	-0.0032	0.068547	-0.04666	0.962785	NA
NFIB	882.7979	0.003196	0.054187	0.058979	0.952968	0.991074
DOCK7	372.4836	-0.00319	0.047683	-0.06698	0.946598	0.988494
MIR874	4.274863	0.003193	0.081067	0.03939	0.96858	NA
GNA12	203.6072	0.003192	0.057045	0.055958	0.955375	NA
TICAM1	14.72337	-0.00319	0.083622	-0.03816	0.969561	NA
ASCL1	22.18609	-0.00319	0.084916	-0.03753	0.97006	NA
POTEE	0.112811	-0.00319	0.019377	-0.16448	0.869354	NA
MGARP	32.49726	-0.00319	0.079229	-0.04023	0.967913	NA
ABCB5	0.107518	0.003186	0.017787	0.179112	0.85785	NA
DEGS1	65.4181	0.003185	0.061055	0.052166	0.958397	NA
BNC1	0.248524	0.003183	0.026542	0.119918	0.904548	NA
SLFN11	19.16319	-0.00318	0.08424	-0.03777	0.96987	NA
ZBTB17	87.60512	-0.00318	0.057428	-0.05537	0.955843	NA
PRUNE2	1266.393	-0.00317	0.055375	-0.05722	0.954367	0.991078
C1orf170	0.207896	0.003157	0.027064	0.116664	0.907126	NA
RNMTL1	63.06115	0.003157	0.059907	0.052703	0.957969	NA
CD5	12.50664	-0.00316	0.084876	-0.03719	0.970337	NA
EMC3	66.79106	0.003155	0.060773	0.051916	0.958595	NA
SLC39A1	28.57358	-0.00315	0.072918	-0.04317	0.965564	NA
RBMXL2	0.8188	0.003146	0.046849	0.067162	0.946452	NA
IFNW1	1.232587	0.003144	0.057378	0.054798	0.956299	NA
TRIM58	3.253433	-0.00314	0.070207	-0.04478	0.964283	NA
NUDT6	11.59325	-0.00314	0.084203	-0.03733	0.970218	NA
FGF8	0.19402	0.003143	0.02601	0.120855	0.903806	NA
ZNF826P	2.185395	0.003134	0.064032	0.048949	0.96096	NA
SEC24C	312.6085	0.003129	0.03992	0.078378	0.937528	NA
STYK1	0.213861	-0.00313	0.026382	-0.1186	0.905596	NA
MIR4324	0.132924	-0.00313	0.021012	-0.14873	0.881767	NA
FRMD7	0.395203	0.003125	0.03329	0.093869	0.925213	NA
C20orf173	14.38931	0.003118	0.084678	0.036819	0.970629	NA
AQP7	29.92969	-0.00312	0.084732	-0.03677	0.970671	NA
SOD1	329.2615	-0.00311	0.065059	-0.04779	0.961884	NA
IPO8	336.1781	0.003102	0.037884	0.081888	0.934736	0.984229
LOC28503	14.52677	-0.0031	0.081108	-0.03824	0.969499	NA
FLJ11235	0.283988	-0.0031	0.029447	-0.10519	0.916223	NA
CUL4A	415.887	-0.0031	0.043235	-0.0716	0.942922	0.986475
BCAS1	263.3993	-0.00309	0.084706	-0.03653	0.970859	NA
BRIX1	59.80725	-0.00309	0.070967	-0.04357	0.965245	NA
MIR3121	0.623035	-0.00309	0.04018	-0.07692	0.938688	NA
CYB5R3	217.297	-0.00309	0.0492	-0.06279	0.949934	NA
REXO1	198.9085	-0.00309	0.051494	-0.05999	0.952164	NA
RNF146	376.0849	0.003084	0.048929	0.063026	0.949746	0.990017
CTBP2	248.5567	0.003083	0.042716	0.072181	0.942458	NA

TSPAN10	11.42581	-0.00308	0.083634	-0.03685	0.970608	NA
TP53TG5	4.440162	0.003072	0.079041	0.03887	0.968994	NA
C17orf77	0.145482	-0.00307	0.01983	-0.15478	0.876993	NA
NEK9	332.1987	0.003069	0.041871	0.073286	0.941578	NA
MDN1	1709.217	-0.00307	0.040995	-0.07482	0.940362	0.984251
FAM47C	0.119727	-0.00307	0.019457	-0.15758	0.874788	NA
LOC10012	2.141891	-0.00306	0.065786	-0.04658	0.962851	NA
MST1	58.98925	0.003059	0.076385	0.040041	0.96806	NA
PES1	96.87985	-0.00306	0.055998	-0.05457	0.956485	NA
LINC00623	0.050134	0.003049	0.009595	0.317758	0.750669	NA
SNORD124	1.36332	-0.00305	0.060253	-0.05058	0.959659	NA
OR2C3	29.18486	0.003046	0.071766	0.042447	0.966142	NA
OR2B6	0.323893	0.003045	0.030689	0.099236	0.920951	NA
PPP1R21	203.9992	-0.00304	0.043209	-0.07044	0.94384	NA
TMEM171	0.125169	-0.00304	0.019517	-0.15591	0.876106	NA
PTCHD1	337.299	-0.00304	0.058746	-0.05176	0.958721	0.991078
FLOT2	141.0881	-0.00304	0.059147	-0.05135	0.959043	NA
HOXB4	0.126992	-0.00304	0.01956	-0.15523	0.87664	NA
ALOXE3	15.4843	-0.00303	0.082886	-0.03656	0.970837	NA
POLR3C	110.1419	-0.00303	0.050333	-0.06018	0.952012	NA
AQP10	0.64843	0.003025	0.044128	0.068545	0.945352	NA
DNAJC13	372.3591	-0.00302	0.039539	-0.07648	0.939037	0.984229
ARMC5	40.24523	0.003023	0.071419	0.042329	0.966236	NA
ATG4C	128.5399	-0.00302	0.05706	-0.05294	0.957777	NA
LOC10012	0.420338	-0.00302	0.037029	-0.08148	0.935064	NA
TMEM106	74.66553	-0.00301	0.067304	-0.04473	0.964325	NA
HS6ST3	49.81193	-0.00301	0.07835	-0.03839	0.969379	NA
FLJ44313	0.238205	-0.00301	0.027562	-0.10908	0.913143	NA
FER1L6	1.464047	0.003006	0.060868	0.04938	0.960616	NA
INMT	0.824265	0.00299	0.050154	0.059622	0.952457	NA
STK4	218.9675	-0.00298	0.042364	-0.07025	0.943997	NA
SGOL1-AS	0.313582	-0.00298	0.032933	-0.09036	0.928005	NA
DOHH	15.34801	-0.00297	0.083134	-0.03573	0.971497	NA
GM140	0.564286	-0.00297	0.042256	-0.07028	0.943968	NA
GGA3	203.9242	0.002966	0.041196	0.071999	0.942603	NA
KLRF2	0.103541	0.002964	0.017741	0.167048	0.867332	NA
TAS2R30	27.22271	-0.00296	0.081129	-0.03651	0.970872	NA
PTGER3	0.148173	0.002954	0.021345	0.138381	0.88994	NA
FAM138D	0.168801	0.002947	0.022823	0.129109	0.897271	NA
DBIL5P	15.41127	0.002946	0.082291	0.035794	0.971447	NA
IL1RN	0.151314	0.002937	0.02262	0.129824	0.896705	NA
PLD5	166.6458	0.002935	0.062801	0.046739	0.962721	NA
MIR4484	0.947355	0.002935	0.052713	0.055672	0.955603	NA
ENPP3	18.96424	0.002933	0.080718	0.036339	0.971012	NA
ADCK3	379.7402	-0.00293	0.07844	-0.03738	0.970182	0.991078
RNU6-28	1.90631	0.002932	0.067021	0.043746	0.965107	NA
TNFAIP6	0.208272	0.002916	0.026116	0.111666	0.911089	NA

ZNF575	25.58442	-0.00292	0.073914	-0.03945	0.96853	NA
MRGPRX3	0.130376	-0.00291	0.020935	-0.13917	0.889315	NA
ZNF883	90.78345	-0.00289	0.07452	-0.03883	0.969029	NA
EXOC4	330.0295	0.002885	0.040914	0.070511	0.943787	NA
LINC00507	0.068584	-0.00288	0.013939	-0.20658	0.83634	NA
BMP15	0.04576	0.002877	0.009321	0.308611	0.757618	NA
PAPD7	331.355	0.002876	0.045555	0.063134	0.94966	NA
PTPRD	1948.312	-0.00288	0.032843	-0.08757	0.930222	0.981804
TM4SF19	0.276152	-0.00287	0.03047	-0.09435	0.92483	NA
FUS	557.9238	0.002869	0.058695	0.048879	0.961016	0.991078
PTGER4	3.781862	-0.00286	0.080072	-0.03578	0.97146	NA
SNRK-AS1	41.24828	-0.00286	0.080464	-0.03557	0.971627	NA
TGM3	0.209547	0.002852	0.027076	0.105321	0.916121	NA
ANGPTL5	1.667037	-0.00285	0.061002	-0.04672	0.96274	NA
TUBB8	0.134276	-0.00285	0.019634	-0.14504	0.88468	NA
LOC10028	11.32803	-0.00285	0.084417	-0.03371	0.973105	NA
LOC25455	129.8635	-0.00284	0.060086	-0.04733	0.962246	NA
MRAP	1.681277	-0.00284	0.058511	-0.04861	0.961232	NA
AGBL5	100.4741	0.002843	0.052463	0.054198	0.956777	NA
NUDT9	108.7468	0.00284	0.055362	0.051301	0.959086	NA
DUSP1	128.7929	0.002836	0.082595	0.034342	0.972604	NA
RAB40A	8.664107	0.002832	0.084674	0.033443	0.973322	NA
DEFB115	0.044604	0.00283	0.009245	0.306083	0.759542	NA
RIT1	108.5347	-0.00283	0.061056	-0.04628	0.963087	NA
ANKRD42	27.59224	0.002822	0.073572	0.038358	0.969403	NA
FAM9B	0.044319	0.002818	0.009226	0.305453	0.760021	NA
MMP2	3.928245	0.002816	0.077082	0.036529	0.97086	NA
LOC40139	15.18383	-0.00282	0.083544	-0.0337	0.973114	NA
PHGR1	0.837348	0.002812	0.045728	0.061502	0.95096	NA
DUSP14	21.97656	0.002811	0.076269	0.036855	0.970601	NA
RNF123	187.4682	0.002807	0.054524	0.051478	0.958945	NA
SLC28A1	0.116726	0.002805	0.017971	0.156091	0.875961	NA
SF3A3	251.3079	-0.0028	0.041795	-0.06704	0.946548	NA
TRAPPC13	99.43849	0.002799	0.058331	0.047977	0.961735	NA
MFSD11	121.3558	-0.00279	0.06332	-0.04402	0.96489	NA
TNNI1	0.152603	-0.00279	0.019901	-0.13997	0.888681	NA
SYT2	1644.282	0.002785	0.0667	0.04176	0.96669	0.991078
ZNF823	85.74682	-0.00277	0.050576	-0.05487	0.956246	NA
TEX13B	0.133227	-0.00277	0.019621	-0.14131	0.887623	NA
CPT1C	151.8329	0.00277	0.066005	0.041965	0.966527	NA
SCLY	2.724993	-0.00277	0.069389	-0.03992	0.968159	NA
MIR32	0.405936	0.002766	0.037188	0.074372	0.940714	NA
NLRP12	0.438813	0.002763	0.038584	0.071608	0.942914	NA
RRAD	7.205808	0.002762	0.082651	0.033423	0.973337	NA
C14orf180	7.510681	0.002756	0.08074	0.034132	0.972772	NA
MIR128-1	0.518177	0.002753	0.03923	0.070185	0.944047	NA
MRPL42	115.0414	-0.00275	0.050787	-0.05408	0.956869	NA

LOC44124	13.05305	0.002746	0.083756	0.032782	0.973849	NA
RAB38	0.129193	-0.00275	0.020919	-0.13124	0.895588	NA
ACBD5	189.0667	0.002745	0.047538	0.057747	0.95395	NA
MIR450A1	0.427628	-0.00274	0.036587	-0.07476	0.940403	NA
C1orf115	71.85354	0.002732	0.075122	0.036371	0.970987	NA
DIP2B	1044.463	0.002727	0.04454	0.061219	0.951185	0.990769
PDE2A	14.46766	0.002725	0.084939	0.03208	0.974409	NA
SLC25A22	136.9353	-0.00272	0.058191	-0.04675	0.96271	NA
TRNP1	46.38535	0.002719	0.068148	0.0399	0.968173	NA
MIR487B	0.174939	-0.00272	0.024511	-0.11082	0.911758	NA
MIR191	0.12814	-0.00271	0.019561	-0.13864	0.889738	NA
B4GALT4	46.84247	0.00271	0.064974	0.041711	0.966729	NA
GPX3	92.63347	0.002706	0.078145	0.034624	0.972379	NA
ERP27	0.316921	-0.0027	0.032697	-0.0827	0.93409	NA
LINC00620	0.147514	-0.00269	0.021216	-0.12697	0.898966	NA
LOC55310	40.7832	0.002691	0.070395	0.038221	0.969512	NA
PDAP1	226.0651	-0.00269	0.046444	-0.05782	0.953895	NA
ADPRM	27.69704	0.002684	0.077147	0.03479	0.972247	NA
GAPVD1	414.7438	0.002671	0.034777	0.076805	0.938778	0.984229
NARS	313.4977	-0.00267	0.061282	-0.04353	0.965282	NA
CRNKL1	286.797	-0.00267	0.048913	-0.05452	0.956523	NA
NLRC5	17.72852	-0.00266	0.084884	-0.03135	0.974993	NA
MIR553	3.31882	-0.00266	0.075038	-0.03545	0.97172	NA
PPP1R10	351.4485	-0.00265	0.06439	-0.04118	0.96715	0.991078
B3GAT2	4.89671	-0.00265	0.080238	-0.03302	0.973655	NA
TALDO1	111.5191	0.002648	0.05775	0.045855	0.963425	NA
RPS4Y1	48.87479	0.002647	0.017248	0.153477	0.878022	NA
FEZF2	0.506347	0.002643	0.034848	0.075852	0.939537	NA
SNORA15	0.170048	0.002639	0.022834	0.115575	0.90799	NA
EDN3	3.508123	0.002616	0.07219	0.036236	0.971094	NA
MT1P2	0.037785	0.002615	0.008888	0.294195	0.768609	NA
WFDC5	0.034931	0.002615	0.008888	0.294195	0.768609	NA
NF1P2	0.025629	0.002615	0.008888	0.294195	0.768609	NA
OR2T10	0.037785	0.002615	0.008888	0.294195	0.768609	NA
SULT6B1	0.036338	0.002615	0.008888	0.294195	0.768609	NA
FRMD6-AS	0.034704	0.002615	0.008888	0.294195	0.768609	NA
GPR82	24.3562	0.002613	0.073594	0.035508	0.971675	NA
PRCD	20.60128	-0.00259	0.084852	-0.0305	0.975669	NA
ZNF195	151.6494	0.002588	0.052672	0.049125	0.96082	NA
LAP3	67.94093	-0.00258	0.081484	-0.03171	0.974702	NA
SNTB2	149.029	-0.00258	0.060093	-0.04295	0.965741	NA
CCDC85B	74.99815	-0.00258	0.078852	-0.03273	0.97389	NA
GTF2H4	86.59434	-0.00257	0.071102	-0.0362	0.971126	NA
SGCG	13.21297	0.002571	0.084065	0.030586	0.9756	NA
GGT3P	0.758689	0.002571	0.04616	0.055701	0.95558	NA
COL4A2-A	0.244661	-0.00257	0.027577	-0.09317	0.925765	NA
MRPS18A	49.97741	-0.00257	0.062131	-0.04135	0.967013	NA

CEP290	435.8988	-0.00257	0.057997	-0.0443	0.964665	0.991078
OR2T33	0.080692	-0.00257	0.01234	-0.20817	0.835098	NA
ADCK1	15.52845	-0.00257	0.082253	-0.03122	0.975092	NA
FSD2	9.844773	0.002563	0.08471	0.030259	0.97586	NA
TAF4B	68.91902	-0.00256	0.060459	-0.04235	0.966219	NA
LRRC37BP	91.41657	-0.00256	0.056853	-0.04501	0.964099	NA
PTPN2	257.3546	0.002558	0.046689	0.054779	0.956314	NA
PTH2R	1.007852	0.002548	0.055328	0.046053	0.963268	NA
CNPY3	87.93588	-0.00255	0.054473	-0.04675	0.962711	NA
LPIN2	276.7586	0.002545	0.062559	0.040687	0.967545	NA
PPAN	5.055568	-0.00254	0.08118	-0.03133	0.975003	NA
ITGAL	21.96656	-0.00254	0.0844	-0.03008	0.976006	NA
NCOA2	461.5542	0.00253	0.042713	0.059242	0.952759	0.991074
EHMT1	356.4689	-0.00252	0.052088	-0.04838	0.961411	0.991078
RPL4	723.3411	-0.00252	0.071142	-0.03541	0.971752	0.991078
MBD5	504.6467	-0.00251	0.038959	-0.06448	0.948588	0.98966
FANCG	43.48728	0.002509	0.075774	0.033117	0.973581	NA
ERCC4	76.84529	-0.00251	0.057414	-0.0437	0.965144	NA
NCMAP	0.584938	0.002509	0.042868	0.058523	0.953332	NA
KDM2A	862.1404	-0.0025	0.050251	-0.04981	0.960278	0.991078
C20orf144	0.632704	0.0025	0.042405	0.058956	0.952987	NA
POLK	217.4751	-0.0025	0.036675	-0.06815	0.945666	NA
IRX6	0.172281	0.002494	0.022862	0.109084	0.913136	NA
FARP2	278.9242	-0.00249	0.06354	-0.03922	0.968718	NA
NR1H3	22.13293	-0.00248	0.084243	-0.02942	0.976527	NA
C12orf77	0.130689	-0.00248	0.020942	-0.11825	0.905868	NA
C2orf83	0.299337	0.00247	0.031725	0.077846	0.93795	NA
ADIPOQ	0.137577	-0.00244	0.021065	-0.11593	0.907706	NA
CNOT6	348.1112	0.002435	0.047637	0.051122	0.959229	0.991078
SPHK1	4.661045	-0.00243	0.077163	-0.03152	0.974855	NA
PIGR	0.121994	-0.00243	0.019487	-0.12481	0.900677	NA
SULT2A1	0.156061	0.002425	0.021421	0.113203	0.90987	NA
TNNI2	0.123415	-0.00242	0.019501	-0.1243	0.901082	NA
LOC25410	12.43292	0.002423	0.083263	0.029105	0.976781	NA
PPP1R16B	419.5255	-0.00242	0.055147	-0.04392	0.96497	0.991078
RTP3	0.233953	-0.00241	0.027539	-0.08756	0.930226	NA
NELFCD	199.319	-0.00241	0.035335	-0.06821	0.945622	NA
ARHGAP29	754.5123	-0.00241	0.050529	-0.04768	0.961969	0.991078
KCNH1	383.0206	-0.00241	0.048848	-0.04929	0.960689	0.991078
MBTPS1	470.8605	0.002405	0.038205	0.062952	0.949804	0.990017
MORC1	0.152277	0.002405	0.022632	0.106256	0.915379	NA
PAIP2B	369.0072	-0.0024	0.058916	-0.04069	0.967544	0.991078
TMBIM6	435.9877	-0.00239	0.052741	-0.04535	0.963829	0.991078
DOK7	63.34868	-0.00239	0.082066	-0.02913	0.976762	NA
ZC3H6	548.1224	0.00239	0.047609	0.050193	0.959968	0.991078
KIAA0226	426.5129	0.002387	0.062598	0.038132	0.969582	0.991078
EIF3G	163.5321	-0.00239	0.053018	-0.045	0.964104	NA

INHBA-AS1	1.437287	0.002381	0.059061	0.040313	0.967844	NA
MICAL1	199.9378	0.00238	0.064437	0.036935	0.970537	NA
CTPS2	94.55617	0.002375	0.059079	0.040201	0.967933	NA
NDST1	190.3774	0.002374	0.05096	0.046585	0.962844	NA
PEX5L	114.8412	0.002373	0.081341	0.029177	0.976724	NA
RBM28	233.8917	0.002362	0.055205	0.042782	0.965875	NA
GOLGA6A	0.249141	0.002356	0.028836	0.081716	0.934872	NA
TRH	1.194941	-0.00236	0.0563	-0.04184	0.966622	NA
CCR8	0.54774	-0.00235	0.042771	-0.05504	0.956107	NA
SNX27	541.2227	-0.00235	0.030998	-0.0757	0.939657	0.984229
C9orf169	0.526412	-0.00234	0.041373	-0.0566	0.954866	NA
GNG8	0.298903	-0.00234	0.028037	-0.08343	0.933507	NA
SEC24D	55.82685	-0.00234	0.070424	-0.0332	0.973514	NA
TARDBP	880.1801	-0.00233	0.029015	-0.08039	0.935931	0.984229
ERP29	160.0297	0.002329	0.067694	0.034401	0.972557	NA
PPFIA2	316.2378	0.002323	0.058471	0.039728	0.96831	NA
KRT7	0.151322	0.00232	0.019863	0.116801	0.907017	NA
FAM5B	12.86091	0.002317	0.084617	0.027383	0.978155	NA
POU3F1	4.439674	0.002314	0.07878	0.029373	0.976567	NA
ZSWIM8-A	21.00716	0.002312	0.078321	0.029521	0.976449	NA
TNFSF15	0.145279	0.002311	0.018007	0.128351	0.897871	NA
SNORD105	38.02536	0.00231	0.083497	0.027667	0.977928	NA
ALX3	5.389712	-0.0023	0.083759	-0.02751	0.978051	NA
NDUFB4	79.82402	-0.0023	0.061935	-0.03712	0.970393	NA
BCS1L	32.02581	-0.0023	0.071973	-0.03193	0.974531	NA
HIST1H2B	2.275612	0.002295	0.064725	0.035464	0.97171	NA
NDUFAF7	159.4501	-0.00229	0.047997	-0.04778	0.96189	NA
HAGH	154.7518	-0.00229	0.06314	-0.03631	0.971032	NA
CLUL1	13.74646	0.002286	0.083934	0.02724	0.978268	NA
CTXN3	0.277347	0.002286	0.025153	0.090874	0.927592	NA
DBNL	198.4973	-0.00228	0.050733	-0.04494	0.964159	NA
LIN52	49.4899	-0.00228	0.065561	-0.03476	0.972267	NA
APOA5	0.292477	0.002277	0.030802	0.07392	0.941074	NA
STK36	98.49045	-0.00227	0.057291	-0.03965	0.96837	NA
MIR3677	0.586516	-0.00227	0.042732	-0.05314	0.95762	NA
PPP1R11	60.43733	-0.00226	0.067239	-0.03368	0.973131	NA
MIR4749	0.483345	0.002263	0.040903	0.055315	0.955888	NA
SGCE	109.2015	0.002261	0.051765	0.043669	0.965169	NA
FBXO3	208.9327	0.00226	0.047456	0.047626	0.962014	NA
PGM1	95.45471	-0.00226	0.066457	-0.03395	0.972921	NA
KLHL32	92.06037	0.00225	0.055297	0.040681	0.96755	NA
PTN	279.2268	-0.00225	0.077389	-0.02904	0.976836	NA
FRAT1	19.03171	-0.00225	0.077054	-0.02915	0.976742	NA
CD244	0.292019	-0.00224	0.031848	-0.0704	0.943877	NA
ZNF334	193.709	-0.00224	0.069571	-0.03216	0.974342	NA
ARHGAP39	158.5838	0.002235	0.052065	0.042932	0.965756	NA
RCVRN	2.648551	0.002232	0.068862	0.032407	0.974148	NA

YARS	158.4353	0.002227	0.063858	0.034871	0.972183	NA
CHD7	3596.145	0.002224	0.043768	0.050807	0.959479	0.991078
OR56A3	0.12354	-0.00222	0.020843	-0.10669	0.915039	NA
MIR889	1.131403	-0.00222	0.058409	-0.03802	0.969672	NA
MACC1	0.754668	0.00222	0.043468	0.051073	0.959268	NA
HCN3	81.74021	-0.00222	0.069	-0.03214	0.97436	NA
GTPBP8	23.67805	-0.00222	0.073417	-0.0302	0.975904	NA
SPAG8	15.38577	0.002217	0.081691	0.027142	0.978346	NA
GLMN	81.85805	0.002215	0.061854	0.03581	0.971434	NA
SNORD100	3.452428	0.002212	0.071587	0.030895	0.975354	NA
NCF1B	0.180083	-0.00221	0.021708	-0.10185	0.918876	NA
ZBED4	310.1452	-0.00219	0.056839	-0.03861	0.969203	NA
MIR602	0.130306	0.002193	0.021158	0.103626	0.917466	NA
KMO	0.965972	-0.00219	0.053815	-0.0407	0.967532	NA
RAPGEF6	1021.008	0.002188	0.052463	0.041698	0.96674	0.991078
PYGO2	76.32802	0.002179	0.055756	0.039074	0.968831	NA
MCOLN2	0.119108	-0.00218	0.020774	-0.10485	0.916497	NA
IFNA4	0.109886	0.002174	0.016022	0.135704	0.892056	NA
NDOR1	73.43057	0.002154	0.063786	0.033767	0.973063	NA
CDYL	139.5882	-0.00215	0.056028	-0.03837	0.969395	NA
GDA	0.192621	-0.00214	0.023307	-0.09192	0.926761	NA
RPS23	364.579	-0.00213	0.077192	-0.02764	0.977947	0.993807
SNORD111	2.187966	0.002131	0.068782	0.030984	0.975282	NA
PGC	0.920827	0.002129	0.051424	0.041399	0.966978	NA
LOC10012	2.978277	-0.00213	0.075941	-0.02802	0.977649	NA
PROM2	31.80523	0.002118	0.078637	0.026937	0.97851	NA
SMIM10	8.006203	0.002118	0.083685	0.025307	0.97981	NA
B3GALTL	18.60121	0.002114	0.082915	0.025493	0.979662	NA
ZNF341	33.08038	0.002103	0.070743	0.029723	0.976288	NA
EXT1	74.2559	0.002091	0.065509	0.031922	0.974535	NA
PVRL4	0.220335	-0.00209	0.027472	-0.07597	0.939442	NA
PTF1A	0.175337	-0.00209	0.024505	-0.08515	0.932146	NA
NFKBIL1	56.8899	-0.00207	0.060258	-0.03442	0.972546	NA
KCNJ12	155.187	-0.00206	0.059141	-0.03484	0.97221	NA
TTC34	2.52902	-0.00205	0.073823	-0.02783	0.977795	NA
DDX23	351.6674	0.002054	0.039453	0.052049	0.95849	0.991078
OR2V2	0.14503	0.002041	0.022548	0.090504	0.927886	NA
ATP5G1	21.64553	-0.00204	0.082126	-0.02482	0.980202	NA
USP20	156.0625	0.002028	0.046544	0.04357	0.965247	NA
RASL12	25.25707	-0.00202	0.084592	-0.02388	0.980947	NA
MAFG	91.45704	-0.00202	0.060764	-0.03323	0.973491	NA
LOC40124	0.139583	-0.00202	0.018062	-0.11158	0.911155	NA
CAPSL	0.161505	0.002006	0.02274	0.088202	0.929716	NA
CHFR	146.4925	0.002003	0.063579	0.031505	0.974867	NA
DGCR6L	32.67864	-0.002	0.071585	-0.0279	0.977743	NA
DCLK1	311.5948	-0.002	0.083579	-0.02388	0.980949	NA
TRAPPC2	66.49403	0.001992	0.062627	0.031811	0.974623	NA

WDR64	0.307821	-0.00199	0.031379	-0.06342	0.949429	NA
KRT36	0.149869	0.001979	0.022605	0.087535	0.930246	NA
CD79B	0.718248	0.001978	0.04639	0.042644	0.965986	NA
SYCP2	182.2345	0.001975	0.053998	0.036573	0.970826	NA
PTCH1	517.8951	-0.00197	0.054055	-0.03647	0.970906	0.991078
BPHL	44.79757	-0.00197	0.073311	-0.02686	0.978571	NA
FAT3	358.3324	0.001966	0.072779	0.027013	0.97845	0.993807
43352	568.7199	-0.00196	0.052331	-0.03749	0.970097	0.991078
CES2	128.1796	-0.00196	0.05629	-0.03484	0.972205	NA
GRIA2	1611.936	0.001956	0.035879	0.054524	0.956518	0.991078
PPP1R13B	422.5387	0.001954	0.038356	0.05095	0.959365	0.991078
FLJ16341	0.240053	0.00195	0.023358	0.083493	0.93346	NA
SPOPL	150.5864	0.001948	0.045309	0.04299	0.965709	NA
ASB9	0.308655	-0.00195	0.030358	-0.06412	0.948871	NA
BCL7B	112.4122	0.001942	0.061865	0.031398	0.974952	NA
STX10	64.06598	-0.00194	0.063143	-0.03075	0.975473	NA
PIGU	32.07681	-0.00194	0.067951	-0.02853	0.977236	NA
GPR113	16.6961	0.001937	0.083262	0.023267	0.981438	NA
ZIC3	154.944	-0.00193	0.048799	-0.03961	0.968403	NA
ZNF775	15.60847	0.001931	0.081865	0.023587	0.981182	NA
PTK7	40.83961	-0.00193	0.074139	-0.026	0.979261	NA
FLJ42102	0.250288	0.001924	0.028839	0.066712	0.946811	NA
LOC10012	2.345025	-0.00192	0.072691	-0.02646	0.978889	NA
FUT4	40.02619	0.001915	0.063989	0.029931	0.976122	NA
ABCF1	199.3448	0.001914	0.045213	0.042332	0.966234	NA
MIR5692B	0.16981	-0.00191	0.024486	-0.07816	0.937698	NA
TNFRSF25	468.3142	-0.00191	0.065986	-0.02898	0.976879	0.993711
LOC64151	0.532299	0.001902	0.04108	0.046311	0.963063	NA
FHL3	3.505858	0.00189	0.077266	0.024462	0.980484	NA
LOC10027	52.91987	-0.00189	0.072457	-0.02606	0.979211	NA
SETD1B	469.3826	-0.00189	0.052056	-0.03622	0.971105	0.991078
PTPRO	9.761942	0.001878	0.084236	0.022298	0.98221	NA
ZW10	106.5041	-0.00187	0.050698	-0.03695	0.970523	NA
ART5	0.300328	-0.00187	0.024827	-0.07544	0.939868	NA
ITPR3	116.5304	-0.00187	0.071468	-0.02618	0.979117	NA
GALR2	2.653858	-0.00187	0.056213	-0.03324	0.973482	NA
TSPO	2.851849	0.001865	0.074838	0.024924	0.980115	NA
FAM214B	39.13642	-0.00186	0.068952	-0.027	0.978458	NA
PIWIL2	12.48702	-0.00186	0.084927	-0.0219	0.982527	NA
PPT2-EGFL	1.959191	0.001859	0.066427	0.027991	0.97767	NA
VIM	72.9488	0.001859	0.08331	0.022309	0.982202	NA
CDKN2A	1.631853	-0.00186	0.060888	-0.03047	0.975693	NA
FRG1	53.70805	-0.00185	0.070822	-0.02617	0.979125	NA
MACF1	6951.875	-0.00185	0.042592	-0.0435	0.965306	0.991078
SRSF4	516.1365	-0.00184	0.039616	-0.04645	0.962951	0.991078
MIR506	0.017473	-0.00184	0.008888	-0.20704	0.835975	NA
MIR632	0.02066	-0.00184	0.008888	-0.20704	0.835975	NA

MIR877	0.021431	-0.00184	0.008888	-0.20704	0.835975	NA
CPN2	0.020428	-0.00184	0.008888	-0.20704	0.835975	NA
ZNF733P	0.02066	-0.00184	0.008888	-0.20704	0.835975	NA
IL25	0.015433	-0.00184	0.008888	-0.20704	0.835975	NA
GUSBP3	0.020404	-0.00184	0.008888	-0.20704	0.835975	NA
MRGPRG	0.016805	-0.00184	0.008888	-0.20704	0.835975	NA
LNX1-AS2	0.015586	-0.00184	0.008888	-0.20704	0.835975	NA
MIR4262	0.018317	-0.00184	0.008888	-0.20704	0.835975	NA
NYX	0.020404	-0.00184	0.008888	-0.20704	0.835975	NA
USP17L3	0.02066	-0.00184	0.008888	-0.20704	0.835975	NA
PDHA2	0.020428	-0.00184	0.008888	-0.20704	0.835975	NA
OR8B3	0.018072	-0.00184	0.008888	-0.20704	0.835975	NA
OR8B8	0.020993	-0.00184	0.008888	-0.20704	0.835975	NA
MIR4301	0.017108	-0.00184	0.008888	-0.20704	0.835975	NA
B4GALNT2	0.017293	-0.00184	0.008888	-0.20704	0.835975	NA
MIR365A	0.015433	-0.00184	0.008888	-0.20704	0.835975	NA
TH	0.020404	-0.00184	0.008888	-0.20704	0.835975	NA
RPS4Y2	0.024637	-0.00184	0.008888	-0.20704	0.835975	NA
FGG	0.015433	-0.00184	0.008888	-0.20704	0.835975	NA
FGA	0.020993	-0.00184	0.008888	-0.20704	0.835975	NA
LOC25409	0.017526	-0.00184	0.008888	-0.20704	0.835975	NA
LOC64535	0.020404	-0.00184	0.008888	-0.20704	0.835975	NA
CAPZA3	0.018681	-0.00184	0.008888	-0.20704	0.835975	NA
FLJ43879	0.015586	-0.00184	0.008888	-0.20704	0.835975	NA
MIR2117	0.02066	-0.00184	0.008888	-0.20704	0.835975	NA
MIR296	0.021431	-0.00184	0.008888	-0.20704	0.835975	NA
TCL6	0.020404	-0.00184	0.008888	-0.20704	0.835975	NA
HELT	0.015586	-0.00184	0.008888	-0.20704	0.835975	NA
MS4A3	0.017108	-0.00184	0.008888	-0.20704	0.835975	NA
MIR4772	0.018317	-0.00184	0.008888	-0.20704	0.835975	NA
MUC17	0.025144	-0.00184	0.008888	-0.20704	0.835975	NA
COX7B2	0.020428	-0.00184	0.008888	-0.20704	0.835975	NA
LOC10050	0.02066	-0.00184	0.008888	-0.20704	0.835975	NA
MIR4739	0.018681	-0.00184	0.008888	-0.20704	0.835975	NA
C11orf88	0.012993	-0.00184	0.008888	-0.20704	0.835975	NA
MIR3912	0.020428	-0.00184	0.008888	-0.20704	0.835975	NA
MIR1287	0.017526	-0.00184	0.008888	-0.20704	0.835975	NA
NPHS2	0.015586	-0.00184	0.008888	-0.20704	0.835975	NA
HIST1H4G	0.025144	-0.00184	0.008888	-0.20704	0.835975	NA
OR9I1	0.015433	-0.00184	0.008888	-0.20704	0.835975	NA
ADAM2	0.025144	-0.00184	0.008888	-0.20704	0.835975	NA
RNASE11	0.017451	-0.00184	0.008888	-0.20704	0.835975	NA
GJD4	0.02066	-0.00184	0.008888	-0.20704	0.835975	NA
LOC10050	0.015433	-0.00184	0.008888	-0.20704	0.835975	NA
RETNLB	0.020428	-0.00184	0.008888	-0.20704	0.835975	NA
SERPINB10	0.018072	-0.00184	0.008888	-0.20704	0.835975	NA
CXorf27	0.017451	-0.00184	0.008888	-0.20704	0.835975	NA

CGA	0.016805	-0.00184	0.008888	-0.20704	0.835975	NA
KRTAP20-4	0.020428	-0.00184	0.008888	-0.20704	0.835975	NA
MIR100	0.016805	-0.00184	0.008888	-0.20704	0.835975	NA
SEMG1	0.018072	-0.00184	0.008888	-0.20704	0.835975	NA
MIR548AR	0.018317	-0.00184	0.008888	-0.20704	0.835975	NA
MIR548AL	0.012993	-0.00184	0.008888	-0.20704	0.835975	NA
SPZ1	0.020404	-0.00184	0.008888	-0.20704	0.835975	NA
MIR1910	0.025144	-0.00184	0.008888	-0.20704	0.835975	NA
KRT5	0.015433	-0.00184	0.008888	-0.20704	0.835975	NA
ODAM	0.021382	-0.00184	0.008888	-0.20704	0.835975	NA
HS3ST6	0.018317	-0.00184	0.008888	-0.20704	0.835975	NA
MAGEA10	0.017526	-0.00184	0.008888	-0.20704	0.835975	NA
KRT14	0.024637	-0.00184	0.008888	-0.20704	0.835975	NA
LOC10012	0.017526	-0.00184	0.008888	-0.20704	0.835975	NA
CIDEA	0.017451	-0.00184	0.008888	-0.20704	0.835975	NA
LINC00615	0.017293	-0.00184	0.008888	-0.20704	0.835975	NA
LINC00616	0.020428	-0.00184	0.008888	-0.20704	0.835975	NA
NSFP1	0.020428	-0.00184	0.008888	-0.20704	0.835975	NA
BAGE	0.017108	-0.00184	0.008888	-0.20704	0.835975	NA
OR10W1	0.02066	-0.00184	0.008888	-0.20704	0.835975	NA
TBC1D21	0.02066	-0.00184	0.008888	-0.20704	0.835975	NA
MIR4791	0.017473	-0.00184	0.008888	-0.20704	0.835975	NA
GSTA7P	0.016805	-0.00184	0.008888	-0.20704	0.835975	NA
WFDC10A	0.016805	-0.00184	0.008888	-0.20704	0.835975	NA
EIF3C	0.021382	-0.00184	0.008888	-0.20704	0.835975	NA
C18orf61	0.017473	-0.00184	0.008888	-0.20704	0.835975	NA
MIR3152	0.024637	-0.00184	0.008888	-0.20704	0.835975	NA
OR10G4	0.017451	-0.00184	0.008888	-0.20704	0.835975	NA
TEX33	0.018317	-0.00184	0.008888	-0.20704	0.835975	NA
XCR1	0.012993	-0.00184	0.008888	-0.20704	0.835975	NA
PADI3	0.021382	-0.00184	0.008888	-0.20704	0.835975	NA
GUCA2A	0.018681	-0.00184	0.008888	-0.20704	0.835975	NA
MDS2	0.018072	-0.00184	0.008888	-0.20704	0.835975	NA
FOXG1	0.025144	-0.00184	0.008888	-0.20704	0.835975	NA
SPINT3	0.024637	-0.00184	0.008888	-0.20704	0.835975	NA
AFM	0.024637	-0.00184	0.008888	-0.20704	0.835975	NA
GALP	0.018072	-0.00184	0.008888	-0.20704	0.835975	NA
FXYD4	0.017108	-0.00184	0.008888	-0.20704	0.835975	NA
CTAG2	0.024637	-0.00184	0.008888	-0.20704	0.835975	NA
MS4A6E	0.017108	-0.00184	0.008888	-0.20704	0.835975	NA
KRTAP4-8	0.015586	-0.00184	0.008888	-0.20704	0.835975	NA
KRTAP4-4	0.016805	-0.00184	0.008888	-0.20704	0.835975	NA
MIR4314	0.02066	-0.00184	0.008888	-0.20704	0.835975	NA
C12orf74	0.017293	-0.00184	0.008888	-0.20704	0.835975	NA
LOC10028	0.020993	-0.00184	0.008888	-0.20704	0.835975	NA
HOXA-AS3	0.018681	-0.00184	0.008888	-0.20704	0.835975	NA
MIR4801	0.018317	-0.00184	0.008888	-0.20704	0.835975	NA

MMP26	0.015433	-0.00184	0.008888	-0.20704	0.835975	NA
TBATA	0.020993	-0.00184	0.008888	-0.20704	0.835975	NA
DSCR8	0.018317	-0.00184	0.008888	-0.20704	0.835975	NA
HBZ	0.025144	-0.00184	0.008888	-0.20704	0.835975	NA
FERD3L	0.021431	-0.00184	0.008888	-0.20704	0.835975	NA
OR51L1	0.020993	-0.00184	0.008888	-0.20704	0.835975	NA
C2orf48	0.015433	-0.00184	0.008888	-0.20704	0.835975	NA
LY6G6F	0.021431	-0.00184	0.008888	-0.20704	0.835975	NA
LOC10012	0.017108	-0.00184	0.008888	-0.20704	0.835975	NA
LINC00563	0.018681	-0.00184	0.008888	-0.20704	0.835975	NA
MGC3403	0.016805	-0.00184	0.008888	-0.20704	0.835975	NA
OR2W1	0.020404	-0.00184	0.008888	-0.20704	0.835975	NA
ADAD1	0.015586	-0.00184	0.008888	-0.20704	0.835975	NA
MIR16-2	0.021382	-0.00184	0.008888	-0.20704	0.835975	NA
CARTPT	0.015433	-0.00184	0.008888	-0.20704	0.835975	NA
MIR3160-1	0.021431	-0.00184	0.008888	-0.20704	0.835975	NA
OR6Q1	0.017451	-0.00184	0.008888	-0.20704	0.835975	NA
ANKUB1	0.015433	-0.00184	0.008888	-0.20704	0.835975	NA
CD300LD	0.02066	-0.00184	0.008888	-0.20704	0.835975	NA
C15orf32	0.017108	-0.00184	0.008888	-0.20704	0.835975	NA
TEX26-AS1	0.017293	-0.00184	0.008888	-0.20704	0.835975	NA
TBX22	0.020404	-0.00184	0.008888	-0.20704	0.835975	NA
AADACL2	0.016805	-0.00184	0.008888	-0.20704	0.835975	NA
LOC64732	0.018681	-0.00184	0.008888	-0.20704	0.835975	NA
TMEM244	0.017473	-0.00184	0.008888	-0.20704	0.835975	NA
MUC7	0.017293	-0.00184	0.008888	-0.20704	0.835975	NA
TXNDC8	0.017293	-0.00184	0.008888	-0.20704	0.835975	NA
CD1C	0.015586	-0.00184	0.008888	-0.20704	0.835975	NA
CD1A	0.016805	-0.00184	0.008888	-0.20704	0.835975	NA
OR4F15	0.017451	-0.00184	0.008888	-0.20704	0.835975	NA
OR8A1	0.015433	-0.00184	0.008888	-0.20704	0.835975	NA
ENTHD1	0.018681	-0.00184	0.008888	-0.20704	0.835975	NA
EDDM3B	0.017526	-0.00184	0.008888	-0.20704	0.835975	NA
MIR4294	0.018681	-0.00184	0.008888	-0.20704	0.835975	NA
LOC34011	0.018681	-0.00184	0.008888	-0.20704	0.835975	NA
HSFY1P1	0.015433	-0.00184	0.008888	-0.20704	0.835975	NA
TNFRSF13	0.020993	-0.00184	0.008888	-0.20704	0.835975	NA
MIR3689A	0.021382	-0.00184	0.008888	-0.20704	0.835975	NA
LRRC15	0.020993	-0.00184	0.008888	-0.20704	0.835975	NA
USP17L17	0.017473	-0.00184	0.008888	-0.20704	0.835975	NA
KIR3DL1	0.017526	-0.00184	0.008888	-0.20704	0.835975	NA
H1FOO	0.016805	-0.00184	0.008888	-0.20704	0.835975	NA
TMPRSS11	0.012993	-0.00184	0.008888	-0.20704	0.835975	NA
MIR384	0.020404	-0.00184	0.008888	-0.20704	0.835975	NA
KRTAP10-1	0.017451	-0.00184	0.008888	-0.20704	0.835975	NA
KRTAP10-1	0.017473	-0.00184	0.008888	-0.20704	0.835975	NA
GJA3	0.021382	-0.00184	0.008888	-0.20704	0.835975	NA

SRY	0.015586	-0.00184	0.008888	-0.20704	0.835975	NA
SLCO1B7	0.02066	-0.00184	0.008888	-0.20704	0.835975	NA
MIR4518	0.024637	-0.00184	0.008888	-0.20704	0.835975	NA
LOC10012	0.015433	-0.00184	0.008888	-0.20704	0.835975	NA
CD99P1	0.017451	-0.00184	0.008888	-0.20704	0.835975	NA
ACTRT1	0.020428	-0.00184	0.008888	-0.20704	0.835975	NA
IZUMO3	0.015586	-0.00184	0.008888	-0.20704	0.835975	NA
LIPK	0.020428	-0.00184	0.008888	-0.20704	0.835975	NA
FOXD3	0.021382	-0.00184	0.008888	-0.20704	0.835975	NA
REG4	0.017526	-0.00184	0.008888	-0.20704	0.835975	NA
GGTLC2	0.015586	-0.00184	0.008888	-0.20704	0.835975	NA
ZNF280A	0.017293	-0.00184	0.008888	-0.20704	0.835975	NA
LGALS7B	0.017293	-0.00184	0.008888	-0.20704	0.835975	NA
AMBN	0.015433	-0.00184	0.008888	-0.20704	0.835975	NA
MIR4658	0.02066	-0.00184	0.008888	-0.20704	0.835975	NA
HOXC5	0.018072	-0.00184	0.008888	-0.20704	0.835975	NA
UBE2Q2P3	0.018681	-0.00184	0.008888	-0.20704	0.835975	NA
LRIT1	0.018317	-0.00184	0.008888	-0.20704	0.835975	NA
LOC84931	0.017473	-0.00184	0.008888	-0.20704	0.835975	NA
FLJ25363	0.012993	-0.00184	0.008888	-0.20704	0.835975	NA
IL3	0.015433	-0.00184	0.008888	-0.20704	0.835975	NA
IAPP	0.021382	-0.00184	0.008888	-0.20704	0.835975	NA
OR6K6	0.015586	-0.00184	0.008888	-0.20704	0.835975	NA
PTH	0.024637	-0.00184	0.008888	-0.20704	0.835975	NA
LOC28386	0.017108	-0.00184	0.008888	-0.20704	0.835975	NA
MIR4757	0.018072	-0.00184	0.008888	-0.20704	0.835975	NA
MIR3196	0.018317	-0.00184	0.008888	-0.20704	0.835975	NA
ABCB11	0.017451	-0.00184	0.008888	-0.20704	0.835975	NA
DLK1	0.020404	-0.00184	0.008888	-0.20704	0.835975	NA
MS4A2	0.017473	-0.00184	0.008888	-0.20704	0.835975	NA
MIR4661	0.015433	-0.00184	0.008888	-0.20704	0.835975	NA
MIR23A	0.017526	-0.00184	0.008888	-0.20704	0.835975	NA
ARL14EPL	0.015586	-0.00184	0.008888	-0.20704	0.835975	NA
KRTAP7-1	0.012993	-0.00184	0.008888	-0.20704	0.835975	NA
MIR4253	0.017526	-0.00184	0.008888	-0.20704	0.835975	NA
MIR4251	0.016805	-0.00184	0.008888	-0.20704	0.835975	NA
LINC00669	0.020404	-0.00184	0.008888	-0.20704	0.835975	NA
GC	0.018072	-0.00184	0.008888	-0.20704	0.835975	NA
RDH8	0.021431	-0.00184	0.008888	-0.20704	0.835975	NA
LOC20072	0.017451	-0.00184	0.008888	-0.20704	0.835975	NA
TMPRSS2	0.025144	-0.00184	0.008888	-0.20704	0.835975	NA
MIR1229	0.017526	-0.00184	0.008888	-0.20704	0.835975	NA
SPRR2D	0.020428	-0.00184	0.008888	-0.20704	0.835975	NA
KRT82	0.021382	-0.00184	0.008888	-0.20704	0.835975	NA
SRG7	0.016805	-0.00184	0.008888	-0.20704	0.835975	NA
KIR2DL1	0.020428	-0.00184	0.008888	-0.20704	0.835975	NA
PNLIPRP2	0.020404	-0.00184	0.008888	-0.20704	0.835975	NA

FAM138B	0.017293	-0.00184	0.008888	-0.20704	0.835975	NA
LOC15391	0.012993	-0.00184	0.008888	-0.20704	0.835975	NA
MIR3664	0.012993	-0.00184	0.008888	-0.20704	0.835975	NA
LOC15222	0.017451	-0.00184	0.008888	-0.20704	0.835975	NA
MRPS31	104.7381	-0.00184	0.055016	-0.03345	0.973319	NA
ATP5B	525.2793	0.001838	0.07508	0.024474	0.980474	0.994278
NXT1	8.402003	0.001836	0.084566	0.021708	0.982681	NA
SPINT4	0.142629	0.001832	0.022515	0.081373	0.935145	NA
MIR5002	0.137623	-0.00183	0.021069	-0.08674	0.930876	NA
MIR4685	0.649317	-0.00182	0.046493	-0.03906	0.968845	NA
LOC25513	0.131336	-0.00181	0.019652	-0.09186	0.926807	NA
CCDC138	81.74463	0.001796	0.060247	0.029815	0.976215	NA
ZNF169	42.40787	-0.0018	0.06834	-0.02627	0.97904	NA
C16orf71	27.01974	-0.0018	0.081295	-0.02208	0.982381	NA
LRP1	3042.475	0.001792	0.04583	0.039105	0.968807	0.991078
PNMA5	2.032423	-0.00179	0.068236	-0.02626	0.97905	NA
LOC40105	14.21266	0.001788	0.083073	0.021518	0.982832	NA
APOB	0.980994	-0.00178	0.046451	-0.03842	0.969353	NA
ZNF663	30.83329	-0.00178	0.081146	-0.02192	0.98251	NA
PITX3	0.073029	0.001778	0.014839	0.119839	0.90461	NA
SLC30A1	29.25921	0.001775	0.076109	0.023319	0.981396	NA
ARPC5	82.6259	-0.00177	0.061671	-0.02873	0.977078	NA
MIR4714	0.901411	-0.00176	0.049833	-0.03522	0.971902	NA
CCL22	0.206599	0.001754	0.023407	0.074942	0.940261	NA
MED15	243.7229	-0.00175	0.041616	-0.04212	0.9664	NA
LOC28541	0.460311	0.001748	0.037894	0.046125	0.96321	NA
CALHM3	0.155625	0.001739	0.022677	0.076663	0.938892	NA
OR1I1	0.179164	0.001729	0.022936	0.075388	0.939906	NA
LOC38894	0.068099	0.001722	0.014605	0.117923	0.906129	NA
NCRNA001	12.85182	0.001721	0.018842	0.091333	0.927228	NA
SPNS3	0.131737	-0.00172	0.019632	-0.08763	0.930174	NA
UGT2B17	0.067868	0.00172	0.014594	0.11783	0.906203	NA
ZNF506	201.2512	-0.00171	0.046574	-0.03682	0.970632	NA
MIR126	0.526145	-0.00171	0.0383	-0.04475	0.964308	NA
TMEM38A	35.20303	0.001713	0.069457	0.024664	0.980323	NA
SEC14L3	0.153028	0.001712	0.021393	0.080043	0.936203	NA
EREG	0.067124	0.001711	0.014557	0.117528	0.906442	NA
FLNB	515.3243	0.001704	0.059194	0.028781	0.977039	0.993711
COL25A1	7.204803	0.001703	0.084541	0.020144	0.983928	NA
MIR219-2	0.066421	0.001703	0.014522	0.117239	0.906671	NA
C8B	0.066024	0.001698	0.014502	0.117075	0.906801	NA
ZNF416	43.02515	-0.0017	0.06425	-0.02641	0.978933	NA
SMARCD3	268.1408	-0.00169	0.054694	-0.03099	0.975281	NA
ST8SIA6	3.031848	0.001695	0.076265	0.022222	0.982271	NA
KCNA10	0.065752	0.001695	0.014488	0.116962	0.90689	NA
LINC00559	0.065686	0.001694	0.014485	0.116935	0.906912	NA
IQCA1	54.89413	0.00169	0.079866	0.021166	0.983113	NA

OR4D5	0.065338	0.00169	0.014467	0.116789	0.907027	NA
THUMPD3	146.8896	-0.00169	0.051977	-0.03245	0.974116	NA
TUBA3C	0.065033	0.001686	0.014451	0.116661	0.907129	NA
CXCL9	0.200578	-0.00169	0.01148	-0.14683	0.883266	NA
NANOS3	4.910829	0.001683	0.082063	0.020507	0.983639	NA
ZNF679	0.212072	-0.00168	0.026336	-0.06386	0.949083	NA
MIR548O2	0.513535	0.001675	0.03965	0.04225	0.9663	NA
HDAC9	177.6165	0.001675	0.070082	0.023895	0.980937	NA
LINC00577	0.063869	0.001672	0.014391	0.116163	0.907523	NA
TBC1D3P5	0.063775	0.001671	0.014386	0.116123	0.907555	NA
VCX	0.063237	0.001664	0.014357	0.115892	0.907739	NA
LOC28369	21.95524	0.001662	0.084047	0.019776	0.984222	NA
RNF20	362.3108	-0.00166	0.041871	-0.03961	0.968401	0.991078
GSC	0.062672	0.001657	0.014327	0.115646	0.907933	NA
BPIFA2	0.062611	0.001656	0.014324	0.11562	0.907954	NA
RPL3L	0.062577	0.001656	0.014322	0.115604	0.907966	NA
MIR604	0.062556	0.001655	0.014321	0.115595	0.907973	NA
ZNF160	353.9704	0.001654	0.035995	0.045961	0.963342	0.991078
TTC17	521.763	-0.00165	0.036962	-0.04468	0.964365	0.991078
LOC10012	0.062177	0.001651	0.014301	0.115429	0.908105	NA
DES	3.476633	-0.00165	0.052814	-0.03124	0.975074	NA
SETDB2	122.402	0.001648	0.046239	0.03565	0.971561	NA
MIR4474	0.58649	-0.00165	0.041497	-0.03969	0.968341	NA
TNRC6A	1276.463	0.001647	0.039404	0.041795	0.966662	0.991078
DAW1	0.061854	0.001647	0.014284	0.115287	0.908218	NA
AQP12B	0.061765	0.001646	0.014279	0.115247	0.908249	NA
CCT6P3	35.67443	-0.00164	0.081404	-0.02019	0.983891	NA
TCF21	0.061367	0.001641	0.014257	0.115071	0.908389	NA
COX7A2L	57.81177	-0.00164	0.078562	-0.02086	0.983354	NA
OR1A1	0.061212	0.001639	0.014249	0.115002	0.908444	NA
PRR12	491.6999	0.001637	0.045382	0.036073	0.971224	0.991078
TAS2R16	0.060515	0.00163	0.01421	0.114689	0.908692	NA
HAR1A	13.62201	0.001629	0.084724	0.019232	0.984656	NA
GHSR	0.060254	0.001626	0.014196	0.114571	0.908785	NA
OR2A25	0.060138	0.001625	0.01419	0.114518	0.908827	NA
CIB3	0.060017	0.001623	0.014183	0.114463	0.90887	NA
ADGB	0.134547	-0.00162	0.021014	-0.07724	0.938436	NA
LOC64869	0.059855	0.001621	0.014174	0.114389	0.908929	NA
MIR223	0.196771	-0.00162	0.024648	-0.06573	0.947593	NA
PTGES3L-A	0.186505	-0.00162	0.025643	-0.06311	0.949682	NA
SDCBP2-A	50.36128	-0.00162	0.066592	-0.02427	0.980636	NA
OR10H5	0.059277	0.001614	0.014142	0.114125	0.909139	NA
HES3	0.059239	0.001613	0.014139	0.114107	0.909153	NA
OR56A4	0.059059	0.001611	0.014129	0.114024	0.909219	NA
POTED	0.058987	0.00161	0.014125	0.113991	0.909245	NA
ELP5	49.79545	0.001609	0.066889	0.024056	0.980808	NA
MIR376B	1.73725	-0.00161	0.066894	-0.02403	0.980831	NA

CRYBA2	0.058713	0.001607	0.01411	0.113864	0.909346	NA
IFNA14	0.05862	0.001605	0.014104	0.113821	0.90938	NA
MIR5681A	0.058548	0.001604	0.0141	0.113787	0.909406	NA
SOST	0.058496	0.001604	0.014097	0.113763	0.909426	NA
NT5E	26.18412	-0.0016	0.073866	-0.02169	0.982692	NA
C14orf39	0.058276	0.001601	0.014085	0.113661	0.909507	NA
RSU1P2	0.058207	0.0016	0.014081	0.113628	0.909532	NA
IGF2-AS	0.058195	0.0016	0.01408	0.113623	0.909537	NA
MIR4643	0.058036	0.001598	0.014071	0.113548	0.909596	NA
LINC00221	0.057637	0.001597	0.014069	0.113534	0.909607	NA
C8orf74	0.057951	0.001597	0.014066	0.113508	0.909628	NA
ALPP	0.057926	0.001596	0.014065	0.113496	0.909637	NA
GATA5	0.057731	0.001594	0.014054	0.113405	0.90971	NA
SLC25A3	243.4575	-0.00159	0.068485	-0.02327	0.981434	NA
TIAF1	30.89105	-0.00159	0.08263	-0.01927	0.984626	NA
SI	0.057466	0.00159	0.014038	0.113279	0.909809	NA
SRD5A1P1	0.057386	0.001589	0.014034	0.113241	0.90984	NA
LOC28557	0.057268	0.001588	0.014027	0.113185	0.909884	NA
DMWD	189.1339	0.001585	0.05802	0.027322	0.978203	NA
KCNQ1DN	0.122514	-0.00158	0.019491	-0.08119	0.935289	NA
MIR3653	0.056873	0.001582	0.014004	0.112997	0.910033	NA
C1QTNF9B	0.056832	0.001582	0.014001	0.112977	0.910049	NA
LOC10013	0.056802	0.001581	0.014	0.112963	0.91006	NA
LOC10027	0.05678	0.001581	0.013998	0.112952	0.910068	NA
CD70	0.143858	0.001581	0.021311	0.074192	0.940858	NA
C10orf55	0.056772	0.001581	0.013998	0.112948	0.910071	NA
MED30	21.18792	-0.00158	0.08025	-0.0197	0.984283	NA
COL6A5	0.056757	0.001581	0.013997	0.112941	0.910077	NA
GLOD5	0.056351	0.001575	0.013973	0.112746	0.910232	NA
MIR1468	0.056335	0.001575	0.013972	0.112738	0.910238	NA
TMEM82	0.108936	-0.00157	0.019337	-0.08139	0.935128	NA
MEP1A	0.056154	0.001573	0.013961	0.112651	0.910307	NA
AGR2	0.671454	-0.00157	0.03958	-0.03974	0.968304	NA
HBM	0.056041	0.001571	0.013955	0.112596	0.910351	NA
IL7R	1.176969	-0.00157	0.051133	-0.03073	0.975488	NA
CLDN8	0.055887	0.001569	0.013946	0.112521	0.91041	NA
KRT16	0.055432	0.001563	0.013918	0.112299	0.910586	NA
C12orf42	0.055426	0.001563	0.013918	0.112296	0.910589	NA
MIA2	0.055424	0.001563	0.013918	0.112295	0.91059	NA
PHOX2A	0.055383	0.001562	0.013915	0.112275	0.910606	NA
LOC10050	0.055177	0.00156	0.013903	0.112173	0.910686	NA
CST11	0.055159	0.001559	0.013902	0.112165	0.910693	NA
MIR3131	0.055018	0.001557	0.013894	0.112095	0.910748	NA
LOC38945	0.054995	0.001557	0.013892	0.112084	0.910757	NA
MIR4519	0.993782	-0.00156	0.051064	-0.03049	0.975675	NA
NLRP8	0.054804	0.001554	0.013881	0.111989	0.910832	NA
MRPL10	65.18314	-0.00155	0.064199	-0.02421	0.980683	NA

KIFC2	475.007	-0.00155	0.048924	-0.03177	0.974656	0.993326
OR2T4	0.054779	0.001554	0.013879	0.111977	0.910842	NA
MIR153-1	0.555729	-0.00155	0.041982	-0.03701	0.970474	NA
TBC1D8	238.6518	0.001553	0.05187	0.029945	0.976111	NA
EGFLAM-A	0.054663	0.001553	0.013872	0.111919	0.910887	NA
LINC00605	0.054618	0.001552	0.013869	0.111897	0.910905	NA
PRRX2	0.054513	0.00155	0.013863	0.111845	0.910947	NA
ARHGAP40	0.054354	0.001548	0.013853	0.111766	0.911009	NA
FAM26D	0.053973	0.001548	0.013851	0.111746	0.911025	NA
MIR4674	0.054278	0.001547	0.013849	0.111727	0.91104	NA
SPATA31D	0.054275	0.001547	0.013848	0.111726	0.91104	NA
STX11	1.387966	0.001546	0.061425	0.025171	0.979919	NA
IVL	0.054141	0.001545	0.01384	0.111659	0.911094	NA
ACER1	0.05401	0.001544	0.013832	0.111593	0.911146	NA
OCSTAMP	0.053993	0.001543	0.013831	0.111585	0.911153	NA
AGR3	0.053979	0.001543	0.01383	0.111578	0.911158	NA
FAM170B	0.149559	-0.00154	0.018061	-0.08542	0.931931	NA
RPRML	12.97174	-0.00154	0.084837	-0.01817	0.985502	NA
OR1D4	0.053821	0.001541	0.013821	0.111498	0.911221	NA
PRG2	0.053732	0.00154	0.013815	0.111453	0.911257	NA
SSX5	0.053291	0.001538	0.013809	0.111401	0.911299	NA
SMARCD1	126.4649	-0.00154	0.05328	-0.02884	0.976993	NA
CRTC1	822.4419	-0.00153	0.043379	-0.03532	0.971826	0.991078
ZNF577	229.364	-0.00153	0.058263	-0.02629	0.979024	NA
PRM3	0.053017	0.00153	0.013771	0.11109	0.911545	NA
SRMS	2.26496	0.00153	0.068971	0.022178	0.982306	NA
LUC7L2	36.34721	-0.00153	0.075195	-0.02034	0.983773	NA
SLC6A3	0.052973	0.001529	0.013768	0.111068	0.911563	NA
LOC15147	0.39744	-0.00153	0.036548	-0.04176	0.966691	NA
OTUD6A	0.052664	0.001525	0.013749	0.11091	0.911688	NA
PSMB11	0.08595	-0.00152	0.015901	-0.09585	0.92364	NA
MIR1289-2	0.547104	-0.00152	0.042089	-0.03621	0.971117	NA
KIF26A	3.126835	0.001523	0.074351	0.020487	0.983655	NA
PBK	0.052123	0.001517	0.013714	0.11063	0.91191	NA
LYZL4	0.051941	0.001515	0.013703	0.110535	0.911985	NA
SCN4B	264.5078	0.001511	0.042341	0.035698	0.971523	NA
MIR4635	0.05124	0.001509	0.013678	0.110337	0.912142	NA
MIR4732	0.051027	0.001506	0.013665	0.110224	0.912231	NA
STK35	165.5912	0.001506	0.053724	0.028032	0.977637	NA
C18orf62	0.257168	0.001505	0.029711	0.050663	0.959594	NA
HELB	5.433287	0.001501	0.080486	0.018648	0.985122	NA
WASH7P	3.753557	0.0015	0.074093	0.020247	0.983847	NA
BIN2	7.037707	-0.0015	0.084372	-0.01778	0.985818	NA
FGB	0.427084	0.001498	0.034999	0.042787	0.965871	NA
PIWIL1	0.050539	0.001495	0.013612	0.109796	0.912571	NA
HAS2-AS1	0.415923	-0.00149	0.035899	-0.04161	0.966809	NA
STX5	111.875	0.001492	0.063062	0.023657	0.981126	NA

OR56A5	0.049905	0.001485	0.013571	0.109455	0.912841	NA
RYR2	2679.438	0.001481	0.048448	0.030572	0.975611	0.993326
FBXL15	36.21768	-0.00148	0.068541	-0.02155	0.982808	NA
GSTTP1	0.318721	0.001475	0.027888	0.052905	0.957807	NA
APOC3	0.049041	0.001473	0.013513	0.108985	0.913215	NA
HTR3A	0.048718	0.001472	0.013512	0.108974	0.913223	NA
NXT2	7.292626	0.001471	0.084706	0.017362	0.986148	NA
MIR5092	0.152308	0.00147	0.021397	0.068698	0.94523	NA
ARGFX	0.351016	0.001469	0.034038	0.043171	0.965565	NA
SEMA4A	12.80122	0.001465	0.084545	0.017329	0.986174	NA
MIR30A	0.048483	0.001464	0.013475	0.108677	0.913459	NA
KLF17	0.122171	-0.00146	0.019503	-0.07507	0.94016	NA
LOC10013	9.455471	-0.00146	0.084487	-0.01728	0.986212	NA
TIMD4	0.408909	-0.00145	0.035544	-0.04074	0.967503	NA
INGX	0.37675	0.001446	0.034752	0.04161	0.96681	NA
RAB1B	115.8665	0.001443	0.06141	0.023492	0.981258	NA
PALM	283.2813	-0.00144	0.046245	-0.03113	0.975169	NA
LINC00700	0.046748	0.001438	0.013355	0.107698	0.914235	NA
ZNF205	57.49752	0.001438	0.059371	0.024223	0.980675	NA
GFI1B	0.046514	0.001435	0.013339	0.107563	0.914342	NA
PFAS	59.78372	-0.00143	0.061202	-0.02344	0.981303	NA
HSPA1A	148.136	0.001434	0.066795	0.021473	0.982869	NA
ANKRD39	54.19891	0.001433	0.065392	0.021921	0.982511	NA
NRAS	74.78065	-0.00143	0.05876	-0.02436	0.980562	NA
LOC28408	5.001897	-0.00143	0.081583	-0.01752	0.986025	NA
ZNF608	466.2344	-0.00142	0.046818	-0.03035	0.975784	0.993326
FAM66E	0.798612	-0.00142	0.042996	-0.03301	0.973663	NA
SRM	45.97279	-0.00142	0.070545	-0.0201	0.983963	NA
MATN4	0.044937	0.001415	0.013246	0.106803	0.914945	NA
PYHIN1	0.33534	0.001415	0.02422	0.058404	0.953427	NA
CALM2	2030.06	0.001414	0.068732	0.020574	0.983586	0.995611
CDK4	45.20037	-0.00141	0.062617	-0.02258	0.981986	NA
MIR219-1	0.044097	0.001401	0.013184	0.106299	0.915345	NA
ZNF622	128.283	0.0014	0.056906	0.024606	0.980369	NA
EPHA6	417.7323	0.0014	0.068731	0.020368	0.98375	0.995611
ZZEF1	702.6903	0.0014	0.045863	0.030518	0.975654	0.993326
RIPK4	0.044166	0.001398	0.013169	0.106179	0.915441	NA
TMED2	243.7867	-0.0014	0.056809	-0.02459	0.980379	NA
C1orf174	27.0421	-0.00139	0.079647	-0.01742	0.986098	NA
IFNAR2	81.46736	0.001387	0.061009	0.022742	0.981856	NA
DEAF1	329.29	0.00138	0.043353	0.031826	0.974611	NA
MIR4703	0.017465	0.001377	0.008888	0.154961	0.876852	NA
LY6G6C	0.024556	0.001377	0.008888	0.154961	0.876852	NA
GALNTL5	0.01381	0.001377	0.008888	0.154961	0.876852	NA
ALPI	0.016689	0.001377	0.008888	0.154961	0.876852	NA
KRTAP1-3	0.013728	0.001377	0.008888	0.154961	0.876852	NA
MIR1470	0.015244	0.001377	0.008888	0.154961	0.876852	NA

HOXB-AS3	0.02288	0.001377	0.008888	0.154961	0.876852	NA
PLA2G2D	0.015244	0.001377	0.008888	0.154961	0.876852	NA
KRTAP29-1	0.02476	0.001377	0.008888	0.154961	0.876852	NA
SERPINB7	0.013728	0.001377	0.008888	0.154961	0.876852	NA
DAOA	0.018893	0.001377	0.008888	0.154961	0.876852	NA
KRTAP3-2	0.01381	0.001377	0.008888	0.154961	0.876852	NA
KRTAP3-1	0.020185	0.001377	0.008888	0.154961	0.876852	NA
LGALS14	0.027824	0.001377	0.008888	0.154961	0.876852	NA
CLEC1B	0.017352	0.001377	0.008888	0.154961	0.876852	NA
FAM27C	0.020622	0.001377	0.008888	0.154961	0.876852	NA
HAND1	0.018893	0.001377	0.008888	0.154961	0.876852	NA
OR5B3	0.02476	0.001377	0.008888	0.154961	0.876852	NA
OR4D2	0.018169	0.001377	0.008888	0.154961	0.876852	NA
F13B	0.017553	0.001377	0.008888	0.154961	0.876852	NA
HOXA2	0.018374	0.001377	0.008888	0.154961	0.876852	NA
PAGE3	0.019173	0.001377	0.008888	0.154961	0.876852	NA
ZIM3	0.02476	0.001377	0.008888	0.154961	0.876852	NA
PNLIP	0.027824	0.001377	0.008888	0.154961	0.876852	NA
GOLGA8D	0.018374	0.001377	0.008888	0.154961	0.876852	NA
DLX2	0.014923	0.001377	0.008888	0.154961	0.876852	NA
DLX3	0.018217	0.001377	0.008888	0.154961	0.876852	NA
MIR4303	0.018374	0.001377	0.008888	0.154961	0.876852	NA
DEFA4	0.015244	0.001377	0.008888	0.154961	0.876852	NA
CERS6-AS1	0.018169	0.001377	0.008888	0.154961	0.876852	NA
TAAR9	0.016689	0.001377	0.008888	0.154961	0.876852	NA
VSTM1	0.019037	0.001377	0.008888	0.154961	0.876852	NA
MIR4488	0.019439	0.001377	0.008888	0.154961	0.876852	NA
C14orf23	0.014923	0.001377	0.008888	0.154961	0.876852	NA
CD5L	0.018374	0.001377	0.008888	0.154961	0.876852	NA
DEFA11P	0.019439	0.001377	0.008888	0.154961	0.876852	NA
LOC38855	0.017766	0.001377	0.008888	0.154961	0.876852	NA
NTF4	0.017352	0.001377	0.008888	0.154961	0.876852	NA
MOXD2P	0.017553	0.001377	0.008888	0.154961	0.876852	NA
MIR15B	0.020622	0.001377	0.008888	0.154961	0.876852	NA
MIR152	0.018374	0.001377	0.008888	0.154961	0.876852	NA
KLK13	0.016689	0.001377	0.008888	0.154961	0.876852	NA
MIR452	0.012815	0.001377	0.008888	0.154961	0.876852	NA
P2RX3	0.017465	0.001377	0.008888	0.154961	0.876852	NA
ALPPL2	0.020334	0.001377	0.008888	0.154961	0.876852	NA
LOC64693	0.016689	0.001377	0.008888	0.154961	0.876852	NA
KRT6C	0.013728	0.001377	0.008888	0.154961	0.876852	NA
MIR4778	0.01381	0.001377	0.008888	0.154961	0.876852	NA
MIR514B	0.018374	0.001377	0.008888	0.154961	0.876852	NA
ODF1	0.024556	0.001377	0.008888	0.154961	0.876852	NA
SDC4P	0.018169	0.001377	0.008888	0.154961	0.876852	NA
PPY2	0.023632	0.001377	0.008888	0.154961	0.876852	NA
CLEC4C	0.020334	0.001377	0.008888	0.154961	0.876852	NA

MIR551B	0.017553	0.001377	0.008888	0.154961	0.876852	NA
MIR557	0.015244	0.001377	0.008888	0.154961	0.876852	NA
HOXD8	0.016689	0.001377	0.008888	0.154961	0.876852	NA
FBP2	0.016689	0.001377	0.008888	0.154961	0.876852	NA
SLC6A19	0.018217	0.001377	0.008888	0.154961	0.876852	NA
SLC6A14	0.02216	0.001377	0.008888	0.154961	0.876852	NA
ADAM5	0.023632	0.001377	0.008888	0.154961	0.876852	NA
WFDC9	0.018893	0.001377	0.008888	0.154961	0.876852	NA
MIR320D2	0.020622	0.001377	0.008888	0.154961	0.876852	NA
FAM74A3	0.027824	0.001377	0.008888	0.154961	0.876852	NA
MIR188	0.02719	0.001377	0.008888	0.154961	0.876852	NA
NOX3	0.018169	0.001377	0.008888	0.154961	0.876852	NA
LINC00210	0.017465	0.001377	0.008888	0.154961	0.876852	NA
ZBED2	0.018893	0.001377	0.008888	0.154961	0.876852	NA
ANKRD66	0.018374	0.001377	0.008888	0.154961	0.876852	NA
MIR1204	0.022096	0.001377	0.008888	0.154961	0.876852	NA
MIR1205	0.02288	0.001377	0.008888	0.154961	0.876852	NA
MIR1203	0.019439	0.001377	0.008888	0.154961	0.876852	NA
MIR1208	0.02719	0.001377	0.008888	0.154961	0.876852	NA
CLRN2	0.025067	0.001377	0.008888	0.154961	0.876852	NA
LINC00550	0.025067	0.001377	0.008888	0.154961	0.876852	NA
DPRX	0.017465	0.001377	0.008888	0.154961	0.876852	NA
NBPF22P	0.020334	0.001377	0.008888	0.154961	0.876852	NA
LCE6A	0.017553	0.001377	0.008888	0.154961	0.876852	NA
OR14C36	0.016689	0.001377	0.008888	0.154961	0.876852	NA
LOC10012	0.022096	0.001377	0.008888	0.154961	0.876852	NA
C11orf53	0.019439	0.001377	0.008888	0.154961	0.876852	NA
DYDC2	0.019253	0.001377	0.008888	0.154961	0.876852	NA
MIR378D1	0.020334	0.001377	0.008888	0.154961	0.876852	NA
AKR1B10	0.022096	0.001377	0.008888	0.154961	0.876852	NA
C15orf43	0.017465	0.001377	0.008888	0.154961	0.876852	NA
SEMG2	0.013728	0.001377	0.008888	0.154961	0.876852	NA
MIR3189	0.019253	0.001377	0.008888	0.154961	0.876852	NA
LOC64371	0.022096	0.001377	0.008888	0.154961	0.876852	NA
OR52L1	0.020622	0.001377	0.008888	0.154961	0.876852	NA
LINC00692	0.023632	0.001377	0.008888	0.154961	0.876852	NA
PCGEM1	0.014923	0.001377	0.008888	0.154961	0.876852	NA
GLYCAM1	0.020622	0.001377	0.008888	0.154961	0.876852	NA
WBSCR28	0.022096	0.001377	0.008888	0.154961	0.876852	NA
CABP2	0.017766	0.001377	0.008888	0.154961	0.876852	NA
OR5B17	0.019037	0.001377	0.008888	0.154961	0.876852	NA
ZP4	0.023632	0.001377	0.008888	0.154961	0.876852	NA
SPAG11B	0.019439	0.001377	0.008888	0.154961	0.876852	NA
MAGEA11	0.02719	0.001377	0.008888	0.154961	0.876852	NA
LALBA	0.024556	0.001377	0.008888	0.154961	0.876852	NA
LOC64348	0.017766	0.001377	0.008888	0.154961	0.876852	NA
LILRP2	0.017352	0.001377	0.008888	0.154961	0.876852	NA

GABRP	0.027824	0.001377	0.008888	0.154961	0.876852	NA
UGT3A1	0.019173	0.001377	0.008888	0.154961	0.876852	NA
MIR5196	0.02216	0.001377	0.008888	0.154961	0.876852	NA
LINC00619	0.01381	0.001377	0.008888	0.154961	0.876852	NA
GPR50	0.02216	0.001377	0.008888	0.154961	0.876852	NA
SCGB2A1	0.017766	0.001377	0.008888	0.154961	0.876852	NA
DIAPH3-AS	0.017553	0.001377	0.008888	0.154961	0.876852	NA
ELANE	0.012815	0.001377	0.008888	0.154961	0.876852	NA
C9orf135	0.017352	0.001377	0.008888	0.154961	0.876852	NA
ARL14	0.018169	0.001377	0.008888	0.154961	0.876852	NA
DHRS7C	0.024556	0.001377	0.008888	0.154961	0.876852	NA
ANKRD33	0.020334	0.001377	0.008888	0.154961	0.876852	NA
MIR1237	0.024556	0.001377	0.008888	0.154961	0.876852	NA
LOC64340	0.01381	0.001377	0.008888	0.154961	0.876852	NA
MC5R	0.017553	0.001377	0.008888	0.154961	0.876852	NA
MIR508	0.018217	0.001377	0.008888	0.154961	0.876852	NA
FAM25A	0.018169	0.001377	0.008888	0.154961	0.876852	NA
MIR4795	0.022302	0.001377	0.008888	0.154961	0.876852	NA
MIR4799	0.022302	0.001377	0.008888	0.154961	0.876852	NA
MIR4423	0.020622	0.001377	0.008888	0.154961	0.876852	NA
POTEH	0.02719	0.001377	0.008888	0.154961	0.876852	NA
MIR3649	0.015244	0.001377	0.008888	0.154961	0.876852	NA
CLPSL1	0.01381	0.001377	0.008888	0.154961	0.876852	NA
MIR4690	0.019439	0.001377	0.008888	0.154961	0.876852	NA
FEV	0.018893	0.001377	0.008888	0.154961	0.876852	NA
LINC00333	0.017553	0.001377	0.008888	0.154961	0.876852	NA
EIF3CL	0.019439	0.001377	0.008888	0.154961	0.876852	NA
TEX36	0.019253	0.001377	0.008888	0.154961	0.876852	NA
TEX35	0.02719	0.001377	0.008888	0.154961	0.876852	NA
CHODL-AS	0.02216	0.001377	0.008888	0.154961	0.876852	NA
OR7E37P	0.02288	0.001377	0.008888	0.154961	0.876852	NA
KRT16P2	0.018507	0.001377	0.008888	0.154961	0.876852	NA
ZPLD1	0.016689	0.001377	0.008888	0.154961	0.876852	NA
CHIA	0.013728	0.001377	0.008888	0.154961	0.876852	NA
TMEM8C	0.02216	0.001377	0.008888	0.154961	0.876852	NA
HOXD13	0.02288	0.001377	0.008888	0.154961	0.876852	NA
HOXD11	0.017352	0.001377	0.008888	0.154961	0.876852	NA
NLRP5	0.017766	0.001377	0.008888	0.154961	0.876852	NA
ASCL4	0.014923	0.001377	0.008888	0.154961	0.876852	NA
NLRP10	0.020334	0.001377	0.008888	0.154961	0.876852	NA
MAGEC1	0.018893	0.001377	0.008888	0.154961	0.876852	NA
MIR208A	0.025067	0.001377	0.008888	0.154961	0.876852	NA
OR6B1	0.022096	0.001377	0.008888	0.154961	0.876852	NA
MIR942	0.018507	0.001377	0.008888	0.154961	0.876852	NA
MRGPRX4	0.02719	0.001377	0.008888	0.154961	0.876852	NA
MIR532	0.020622	0.001377	0.008888	0.154961	0.876852	NA
KRTAP13-2	0.02288	0.001377	0.008888	0.154961	0.876852	NA

MIR4540	0.016689	0.001377	0.008888	0.154961	0.876852	NA
HOXB8	0.022302	0.001377	0.008888	0.154961	0.876852	NA
SLCO6A1	0.018374	0.001377	0.008888	0.154961	0.876852	NA
MIR1265	0.014923	0.001377	0.008888	0.154961	0.876852	NA
TDO2	0.017766	0.001377	0.008888	0.154961	0.876852	NA
DEFA8P	0.015244	0.001377	0.008888	0.154961	0.876852	NA
MIR4475	0.019173	0.001377	0.008888	0.154961	0.876852	NA
MIR4802	0.019439	0.001377	0.008888	0.154961	0.876852	NA
SPACA1	0.020622	0.001377	0.008888	0.154961	0.876852	NA
MIR363	0.020185	0.001377	0.008888	0.154961	0.876852	NA
OR10J1	0.020622	0.001377	0.008888	0.154961	0.876852	NA
FAM169B	0.02288	0.001377	0.008888	0.154961	0.876852	NA
MIR4533	0.020334	0.001377	0.008888	0.154961	0.876852	NA
LOC28466	0.017352	0.001377	0.008888	0.154961	0.876852	NA
C10orf99	0.020185	0.001377	0.008888	0.154961	0.876852	NA
ATOH1	0.020334	0.001377	0.008888	0.154961	0.876852	NA
FAM216B	0.025067	0.001377	0.008888	0.154961	0.876852	NA
GIMD1	0.027824	0.001377	0.008888	0.154961	0.876852	NA
CRYGA	0.018507	0.001377	0.008888	0.154961	0.876852	NA
CRYGD	0.018893	0.001377	0.008888	0.154961	0.876852	NA
RNF5	0.017352	0.001377	0.008888	0.154961	0.876852	NA
OR5V1	0.017352	0.001377	0.008888	0.154961	0.876852	NA
RIMBP3C	0.014923	0.001377	0.008888	0.154961	0.876852	NA
LINC00837	0.022096	0.001377	0.008888	0.154961	0.876852	NA
DPPA2	0.017766	0.001377	0.008888	0.154961	0.876852	NA
MIR564	0.013728	0.001377	0.008888	0.154961	0.876852	NA
SPP2	0.013728	0.001377	0.008888	0.154961	0.876852	NA
FOXB2	0.02288	0.001377	0.008888	0.154961	0.876852	NA
NHEG1	0.019253	0.001377	0.008888	0.154961	0.876852	NA
OR12D3	0.02288	0.001377	0.008888	0.154961	0.876852	NA
TRIM77	0.013728	0.001377	0.008888	0.154961	0.876852	NA
LIM2	0.018507	0.001377	0.008888	0.154961	0.876852	NA
SHCBP1L	0.018893	0.001377	0.008888	0.154961	0.876852	NA
AKR1C4	0.025067	0.001377	0.008888	0.154961	0.876852	NA
ADH1C	0.018893	0.001377	0.008888	0.154961	0.876852	NA
LOC14709	0.019173	0.001377	0.008888	0.154961	0.876852	NA
UCN3	0.016689	0.001377	0.008888	0.154961	0.876852	NA
LOC28554	0.019439	0.001377	0.008888	0.154961	0.876852	NA
SIGLEC6	0.022096	0.001377	0.008888	0.154961	0.876852	NA
PRL	0.02288	0.001377	0.008888	0.154961	0.876852	NA
HOTAIR	0.02216	0.001377	0.008888	0.154961	0.876852	NA
DSG4	0.022302	0.001377	0.008888	0.154961	0.876852	NA
MSGN1	0.02476	0.001377	0.008888	0.154961	0.876852	NA
ADTRP	0.01381	0.001377	0.008888	0.154961	0.876852	NA
CRISP3	0.023632	0.001377	0.008888	0.154961	0.876852	NA
MIR146B	0.017766	0.001377	0.008888	0.154961	0.876852	NA
MIR146A	0.022302	0.001377	0.008888	0.154961	0.876852	NA

C7orf66	0.014923	0.001377	0.008888	0.154961	0.876852	NA
OR1A2	0.027824	0.001377	0.008888	0.154961	0.876852	NA
UGT1A6	0.014923	0.001377	0.008888	0.154961	0.876852	NA
UGT1A7	0.023632	0.001377	0.008888	0.154961	0.876852	NA
TRIM10	0.023632	0.001377	0.008888	0.154961	0.876852	NA
TRIM15	0.023632	0.001377	0.008888	0.154961	0.876852	NA
MIR490	0.018169	0.001377	0.008888	0.154961	0.876852	NA
CDH9	0.022302	0.001377	0.008888	0.154961	0.876852	NA
MIR2682	0.018169	0.001377	0.008888	0.154961	0.876852	NA
VCX2	0.017465	0.001377	0.008888	0.154961	0.876852	NA
PDE6H	0.020185	0.001377	0.008888	0.154961	0.876852	NA
TTTY19	0.02719	0.001377	0.008888	0.154961	0.876852	NA
CLLU1OS	0.02476	0.001377	0.008888	0.154961	0.876852	NA
LOC64236	0.01381	0.001377	0.008888	0.154961	0.876852	NA
AP1B1P1	0.024556	0.001377	0.008888	0.154961	0.876852	NA
HABP2	0.018374	0.001377	0.008888	0.154961	0.876852	NA
SPDYE4	0.012815	0.001377	0.008888	0.154961	0.876852	NA
KRTDAP	0.018217	0.001377	0.008888	0.154961	0.876852	NA
FAM170A	0.016689	0.001377	0.008888	0.154961	0.876852	NA
MIR892B	0.018374	0.001377	0.008888	0.154961	0.876852	NA
LOC65350	0.017352	0.001377	0.008888	0.154961	0.876852	NA
LINC00851	0.017766	0.001377	0.008888	0.154961	0.876852	NA
FGF4	0.01381	0.001377	0.008888	0.154961	0.876852	NA
APCS	0.017553	0.001377	0.008888	0.154961	0.876852	NA
C1orf158	0.019439	0.001377	0.008888	0.154961	0.876852	NA
ECEL1P2	0.018893	0.001377	0.008888	0.154961	0.876852	NA
PMS2L2	0.018374	0.001377	0.008888	0.154961	0.876852	NA
MIR3920	0.018507	0.001377	0.008888	0.154961	0.876852	NA
OR8D4	0.022302	0.001377	0.008888	0.154961	0.876852	NA
SCN10A	0.02476	0.001377	0.008888	0.154961	0.876852	NA
TMPRSS11	0.019173	0.001377	0.008888	0.154961	0.876852	NA
MIR4281	0.018893	0.001377	0.008888	0.154961	0.876852	NA
LINC00656	0.019173	0.001377	0.008888	0.154961	0.876852	NA
OR4F4	0.023632	0.001377	0.008888	0.154961	0.876852	NA
DMBX1	0.017352	0.001377	0.008888	0.154961	0.876852	NA
IL2	0.02288	0.001377	0.008888	0.154961	0.876852	NA
CFC1B	0.020622	0.001377	0.008888	0.154961	0.876852	NA
KRTAP26-1	0.016689	0.001377	0.008888	0.154961	0.876852	NA
C5orf20	0.018507	0.001377	0.008888	0.154961	0.876852	NA
SLAMF9	0.018169	0.001377	0.008888	0.154961	0.876852	NA
LINC00544	0.019439	0.001377	0.008888	0.154961	0.876852	NA
ZAN	0.023632	0.001377	0.008888	0.154961	0.876852	NA
TBX4	0.014923	0.001377	0.008888	0.154961	0.876852	NA
SLC22A24	0.020334	0.001377	0.008888	0.154961	0.876852	NA
C20orf85	0.018169	0.001377	0.008888	0.154961	0.876852	NA
MIR4462	0.018893	0.001377	0.008888	0.154961	0.876852	NA
MIR1264	0.020185	0.001377	0.008888	0.154961	0.876852	NA

FOXI2	0.022096	0.001377	0.008888	0.154961	0.876852	NA
SLC25A51	0.018217	0.001377	0.008888	0.154961	0.876852	NA
PGLYRP3	0.025067	0.001377	0.008888	0.154961	0.876852	NA
CT49	0.019439	0.001377	0.008888	0.154961	0.876852	NA
TOP1P2	0.018893	0.001377	0.008888	0.154961	0.876852	NA
MIR4721	0.017465	0.001377	0.008888	0.154961	0.876852	NA
LOC28613	0.019253	0.001377	0.008888	0.154961	0.876852	NA
C4orf40	0.025067	0.001377	0.008888	0.154961	0.876852	NA
MIR4758	0.014923	0.001377	0.008888	0.154961	0.876852	NA
CCKAR	0.02288	0.001377	0.008888	0.154961	0.876852	NA
MIR3190	0.019037	0.001377	0.008888	0.154961	0.876852	NA
MIR3193	0.018169	0.001377	0.008888	0.154961	0.876852	NA
FAM95B1	0.017352	0.001377	0.008888	0.154961	0.876852	NA
CHURC1-F	0.018217	0.001377	0.008888	0.154961	0.876852	NA
MIR664	0.023632	0.001377	0.008888	0.154961	0.876852	NA
GDF3	0.022302	0.001377	0.008888	0.154961	0.876852	NA
TMEM114	0.020334	0.001377	0.008888	0.154961	0.876852	NA
PRG3	0.019037	0.001377	0.008888	0.154961	0.876852	NA
LINC00704	0.013728	0.001377	0.008888	0.154961	0.876852	NA
LOC11643	0.020622	0.001377	0.008888	0.154961	0.876852	NA
MIR1307	0.014923	0.001377	0.008888	0.154961	0.876852	NA
CCDC42	0.018507	0.001377	0.008888	0.154961	0.876852	NA
OR10C1	0.01381	0.001377	0.008888	0.154961	0.876852	NA
ATP6V1G3	0.020185	0.001377	0.008888	0.154961	0.876852	NA
F9	0.018374	0.001377	0.008888	0.154961	0.876852	NA
CCL16	0.019173	0.001377	0.008888	0.154961	0.876852	NA
EHHADH-A	0.017766	0.001377	0.008888	0.154961	0.876852	NA
C7orf45	0.02288	0.001377	0.008888	0.154961	0.876852	NA
MYL10	0.018374	0.001377	0.008888	0.154961	0.876852	NA
OR52K2	0.019173	0.001377	0.008888	0.154961	0.876852	NA
GPR110	0.017352	0.001377	0.008888	0.154961	0.876852	NA
CYLC1	0.02719	0.001377	0.008888	0.154961	0.876852	NA
IQCF6	0.013728	0.001377	0.008888	0.154961	0.876852	NA
ABCC13	0.02719	0.001377	0.008888	0.154961	0.876852	NA
SLC2A7	0.019037	0.001377	0.008888	0.154961	0.876852	NA
TBC1D3G	0.019439	0.001377	0.008888	0.154961	0.876852	NA
CEACAM5	0.02719	0.001377	0.008888	0.154961	0.876852	NA
MIR1228	0.02288	0.001377	0.008888	0.154961	0.876852	NA
MIR1227	0.02288	0.001377	0.008888	0.154961	0.876852	NA
SPRR2G	0.027824	0.001377	0.008888	0.154961	0.876852	NA
SPRR2F	0.018169	0.001377	0.008888	0.154961	0.876852	NA
SPATA31C	0.017766	0.001377	0.008888	0.154961	0.876852	NA
OR2T6	0.017465	0.001377	0.008888	0.154961	0.876852	NA
OR2T1	0.020185	0.001377	0.008888	0.154961	0.876852	NA
C22orf42	0.02719	0.001377	0.008888	0.154961	0.876852	NA
BMP10	0.019253	0.001377	0.008888	0.154961	0.876852	NA
HOXC11	0.012815	0.001377	0.008888	0.154961	0.876852	NA

HOXC10	0.02288	0.001377	0.008888	0.154961	0.876852	NA
HOXC13	0.018217	0.001377	0.008888	0.154961	0.876852	NA
MIR4789	0.018507	0.001377	0.008888	0.154961	0.876852	NA
MYH2	0.02216	0.001377	0.008888	0.154961	0.876852	NA
MYH1	0.020185	0.001377	0.008888	0.154961	0.876852	NA
PNLIPRP1	0.02216	0.001377	0.008888	0.154961	0.876852	NA
PNLIPRP3	0.018507	0.001377	0.008888	0.154961	0.876852	NA
LINC00701	0.02288	0.001377	0.008888	0.154961	0.876852	NA
C12orf36	0.019253	0.001377	0.008888	0.154961	0.876852	NA
MIR1538	0.02288	0.001377	0.008888	0.154961	0.876852	NA
LOC28391	0.020185	0.001377	0.008888	0.154961	0.876852	NA
MCHR2	0.017766	0.001377	0.008888	0.154961	0.876852	NA
OR10H3	0.016689	0.001377	0.008888	0.154961	0.876852	NA
ZNRF2P2	0.012815	0.001377	0.008888	0.154961	0.876852	NA
FBXO6	18.3514	0.001375	0.08024	0.017131	0.986332	NA
LOC14578	46.77353	-0.00137	0.066337	-0.02069	0.983495	NA
NDUFAF5	77.80439	-0.00137	0.055431	-0.02468	0.980311	NA
KCNA6	18.60267	-0.00136	0.084399	-0.01615	0.987115	NA
VPS13D	1457.86	0.00134	0.037118	0.036112	0.971193	0.991078
EVA1A	0.345588	0.001336	0.034052	0.039227	0.96871	NA
BRI3	18.16271	-0.00133	0.079341	-0.01673	0.986654	NA
HIST1H3I	0.405163	-0.00132	0.036235	-0.03631	0.971037	NA
MIR4467	1.129616	-0.00131	0.057536	-0.02278	0.981827	NA
FLJ35424	0.122285	-0.0013	0.017887	-0.07291	0.941881	NA
SNORD72	0.350713	-0.0013	0.034792	-0.03745	0.970126	NA
CD34	28.12892	-0.0013	0.084631	-0.01534	0.987758	NA
TRAM1	92.03644	-0.0013	0.054712	-0.02367	0.981115	NA
UBC	1231.922	-0.00129	0.072882	-0.01776	0.985829	0.995611
ALG10B	95.88044	0.00129	0.053903	0.023925	0.980912	NA
UFD1L	46.39525	-0.00129	0.072482	-0.01778	0.985817	NA
ROBO4	17.38188	0.001288	0.084844	0.015186	0.987884	NA
SP9	0.075039	0.001286	0.012632	0.101799	0.918916	NA
SERPINA4	0.074784	0.001284	0.012621	0.101708	0.918989	NA
MIR5193	0.201701	-0.00128	0.024667	-0.0518	0.958688	NA
CMTM7	9.718215	-0.00128	0.081035	-0.01574	0.987438	NA
ESRRA	38.2424	0.001264	0.070548	0.017921	0.985702	NA
TMED10	222.1654	0.001264	0.057647	0.021925	0.982508	NA
SMARCE1	261.0504	0.001261	0.036973	0.034107	0.972792	NA
MAMSTR	47.36195	-0.00126	0.072013	-0.01747	0.986065	NA
ADIPOR1	164.8078	0.001255	0.050505	0.024845	0.980179	NA
FCHSD2	302.5692	-0.00125	0.040876	-0.03064	0.975555	NA
SLC25A43	38.23601	0.001248	0.070473	0.017703	0.985875	NA
GNB3	85.70173	0.001233	0.077626	0.015889	0.987323	NA
PTCHD3	0.111264	0.001229	0.01621	0.075808	0.939572	NA
HAPLN4	58.80461	-0.00123	0.084181	-0.01456	0.988383	NA
POP5	16.36258	0.001224	0.082046	0.014914	0.988101	NA
IER5	61.29267	-0.00122	0.078065	-0.01565	0.987512	NA

FCRL1	0.067585	0.001217	0.01229	0.099011	0.921129	NA
AMER2	455.0352	0.001214	0.050123	0.024229	0.98067	0.994278
AGAP7	2.935138	0.001213	0.033948	0.035726	0.971501	NA
ANKRD20A	0.172477	-0.00121	0.024484	-0.0494	0.9606	NA
MRPL44	43.1952	-0.00121	0.074257	-0.01623	0.987053	NA
CPXM2	10.65712	0.001201	0.08341	0.014404	0.988507	NA
SLITRK2	74.8694	-0.0012	0.076508	-0.01568	0.987489	NA
LOC14995	0.162567	0.001199	0.019949	0.060105	0.952072	NA
EARS2	106.2086	-0.00119	0.057038	-0.02094	0.983294	NA
ARL8A	101.5737	-0.00119	0.053193	-0.02245	0.982091	NA
RRN3P3	81.05822	0.001193	0.064957	0.018361	0.985351	NA
GLYATL3	0.06479	0.00119	0.012154	0.097901	0.922011	NA
PSMA8	0.064748	0.001189	0.012152	0.097884	0.922024	NA
HSD52	0.064544	0.001187	0.012141	0.097799	0.922092	NA
KLK14	2.358116	0.001183	0.07108	0.016641	0.986723	NA
CSMD3	152.5355	0.001181	0.074818	0.015785	0.987406	NA
PILRB	714.128	0.00118	0.066061	0.017856	0.985754	0.995611
LOC72960	15.6527	0.001179	0.084672	0.013926	0.988889	NA
SLC39A5	4.918525	-0.00118	0.08123	-0.0145	0.988428	NA
SRGAP1	164.9066	-0.00117	0.06743	-0.01735	0.986156	NA
FLJ45079	0.178071	-0.00117	0.023045	-0.05075	0.959527	NA
LOC28637	0.062392	0.001166	0.012032	0.096914	0.922795	NA
ARHGAP36	0.922774	-0.00115	0.053035	-0.02176	0.982636	NA
RTKN2	0.519095	-0.00115	0.042424	-0.02719	0.978309	NA
MIR4316	0.473956	0.001153	0.040415	0.028537	0.977234	NA
SNORA80B	0.185757	-0.00115	0.02563	-0.045	0.964111	NA
COG7	91.73752	0.001151	0.059389	0.019378	0.98454	NA
ARMCX1	62.4262	0.001147	0.065649	0.017465	0.986066	NA
NKAP	90.99025	-0.00114	0.061599	-0.01854	0.985211	NA
BNC2	6.392022	0.001139	0.084284	0.013511	0.98922	NA
COQ10A	25.96171	0.001137	0.075302	0.015097	0.987955	NA
RFX2	37.58108	-0.00113	0.083516	-0.01356	0.989183	NA
IFT52	45.36858	0.00113	0.060029	0.018821	0.984984	NA
ZNF213	69.08333	0.00113	0.058383	0.01935	0.984562	NA
GABARAPL	319.0415	0.001124	0.061652	0.018239	0.985448	NA
ORM1	0.18202	0.001124	0.011834	0.095004	0.924311	NA
TGIF1	4.865904	0.001123	0.08155	0.013769	0.989014	NA
LOC10050	0.087159	-0.00112	0.017499	-0.06408	0.948907	NA
PANX3	0.090274	-0.00112	0.017616	-0.06344	0.949415	NA
MUC13	0.052461	-0.00112	0.012083	-0.09241	0.926374	NA
HSD3B1	0.149475	0.001114	0.02136	0.052156	0.958404	NA
LOC64377	0.243932	0.00111	0.027802	0.039921	0.968156	NA
MIR554	0.62818	-0.0011	0.045037	-0.02447	0.980478	NA
AMOTL2	96.87791	0.0011	0.068449	0.016066	0.987182	NA
LOC15019	1.342256	0.001098	0.05991	0.018332	0.985374	NA
PODN	10.24108	0.001091	0.084632	0.012892	0.989714	NA
HCAR3	0.215622	-0.00109	0.024754	-0.04397	0.964932	NA

PROSER2-	8.940376	0.001088	0.084328	0.012905	0.989703	NA
SESTD1	563.0174	-0.00108	0.044724	-0.02425	0.980651	0.994278
CRYBB2	0.097203	-0.00108	0.01775	-0.0611	0.951283	NA
WDR65	0.658045	0.001082	0.044941	0.024083	0.980786	NA
CA6	0.048817	-0.00107	0.011829	-0.09045	0.927933	NA
FAM78A	12.40215	0.00107	0.082972	0.012892	0.989714	NA
GTSF1L	0.048775	-0.00107	0.011826	-0.09042	0.927952	NA
SHD	13.04684	0.001069	0.084937	0.012585	0.989959	NA
FRMD8P1	0.768573	0.001069	0.039591	0.026998	0.978461	NA
PBX2	104.0535	0.001063	0.048749	0.0218	0.982607	NA
LINC00114	0.048252	-0.00106	0.011789	-0.09013	0.928183	NA
POLR3G	40.98429	0.001059	0.065264	0.016227	0.987053	NA
CDRT15	2.064261	0.001058	0.059565	0.017769	0.985823	NA
ASPSCR1	68.81679	-0.00105	0.065994	-0.01592	0.987294	NA
LOC33959	0.047303	-0.00105	0.011719	-0.0896	0.928608	NA
ATP8A1	412.1037	0.001049	0.059457	0.017636	0.985929	0.995611
OOEP	0.050829	0.001043	0.011385	0.091651	0.926976	NA
IL27	0.206585	0.001039	0.02317	0.044834	0.96424	NA
SNORA69	0.299239	-0.00104	0.029245	-0.03552	0.971667	NA
MIR551A	0.046142	-0.00103	0.011633	-0.08893	0.929138	NA
PDZK1IP1	0.045938	-0.00103	0.011618	-0.08881	0.929232	NA
LOC40155	0.045495	-0.00103	0.011584	-0.08855	0.929437	NA
C10orf91	0.04535	-0.00102	0.011573	-0.08847	0.929505	NA
CACNG6	0.045262	-0.00102	0.011566	-0.08842	0.929546	NA
MIR5197	0.048963	0.001022	0.011269	0.09071	0.927723	NA
LINC00635	0.044932	-0.00102	0.011541	-0.08822	0.9297	NA
KCNMB2-I	0.044932	-0.00102	0.011541	-0.08822	0.9297	NA
COG3	194.994	-0.00102	0.058278	-0.01747	0.986063	NA
CEP85	136.7751	-0.00102	0.06696	-0.01519	0.987879	NA
MT1B	0.044629	-0.00101	0.011518	-0.08804	0.929843	NA
MS4A15	0.151834	0.001013	0.021395	0.047367	0.962221	NA
PKM	1122.283	-0.00101	0.061589	-0.01641	0.986906	0.996054
ASS1	15.38083	0.00101	0.084834	0.011904	0.990502	NA
MIR320E	0.110956	0.001009	0.014331	0.070385	0.943887	NA
FAM47A	0.044036	-0.00101	0.011472	-0.08769	0.930125	NA
OR5AU1	0.043891	-0.001	0.011461	-0.0876	0.930194	NA
CCDC120	77.10175	-0.001	0.072699	-0.01376	0.989019	NA
CMA1	0.190702	-0.001	0.024625	-0.04061	0.967603	NA
ETV3L	0.043527	-0.001	0.011432	-0.08738	0.930369	NA
ZNF292	1159.456	-0.001	0.036458	-0.02733	0.978198	0.993807
MIR629	0.046058	0.000994	0.011112	0.08943	0.928741	NA
ZNF479	0.043139	-0.00099	0.011402	-0.08714	0.930556	NA
SERPINB2	0.043011	-0.00099	0.011391	-0.08707	0.930619	NA
RECQL5	67.26359	0.000988	0.069406	0.014241	0.988637	NA
ZNRF1	53.44783	0.000988	0.063735	0.015506	0.987629	NA
ZFAND3	196.5857	-0.00099	0.052279	-0.01889	0.984926	NA
HOXA5	0.042697	-0.00099	0.011366	-0.08687	0.930772	NA

ADAM29	0.045937	0.000987	0.011073	0.089117	0.928989	NA
MIR1291	0.042593	-0.00099	0.011358	-0.08681	0.930823	NA
PRM1	0.042496	-0.00098	0.01135	-0.08675	0.93087	NA
CLDN10	11.06043	0.000984	0.08476	0.011605	0.990741	NA
LINC00841	0.042359	-0.00098	0.011339	-0.08667	0.930937	NA
MSMB	0.04219	-0.00098	0.011326	-0.08656	0.931021	NA
CWH43	0.04219	-0.00098	0.011326	-0.08656	0.931021	NA
TPD52L3	0.042175	-0.00098	0.011325	-0.08655	0.931028	NA
KRT80	0.042082	-0.00098	0.011317	-0.08649	0.931074	NA
HHLA1	0.042053	-0.00098	0.011315	-0.08647	0.931089	NA
TAT	0.30667	-0.00098	0.032383	-0.03015	0.975949	NA
G6PC3	27.5315	-0.00097	0.070283	-0.01387	0.988935	NA
DDIT4L	12.0722	-0.00097	0.082971	-0.01174	0.990631	NA
C2orf76	11.40757	-0.00097	0.084502	-0.01153	0.990802	NA
ANKRD19P	143.8035	0.000974	0.079048	0.01232	0.99017	NA
HFM1	97.58157	-0.00097	0.083528	-0.01165	0.990703	NA
MIR3917	0.041561	-0.00097	0.011275	-0.08617	0.931333	NA
AKR1B15	0.496675	0.00097	0.034835	0.027853	0.97778	NA
LINC00426	0.646688	0.00097	0.04392	0.022077	0.982387	NA
LOC15093	0.041281	-0.00097	0.011252	-0.08599	0.931473	NA
KCNQ5	10.59211	0.000964	0.078849	0.012231	0.990241	NA
TMPRSS11	0.040994	-0.00096	0.011228	-0.08581	0.931618	NA
OR2J3	0.040953	-0.00096	0.011225	-0.08578	0.931639	NA
CDRT15L2	0.04087	-0.00096	0.011218	-0.08573	0.93168	NA
LECT2	0.217373	-0.00096	0.026349	-0.03647	0.970906	NA
NCR2	0.04074	-0.00096	0.011207	-0.08565	0.931746	NA
LOC15148	0.153115	0.00096	0.022648	0.04237	0.966203	NA
FABP12	0.040685	-0.00096	0.011203	-0.08561	0.931774	NA
LOC49412	0.098635	-0.00096	0.01777	-0.05395	0.956978	NA
FAM71A	0.0405	-0.00096	0.011188	-0.08549	0.931868	NA
LOC15487	0.040437	-0.00096	0.011182	-0.08545	0.931901	NA
VPREB1	0.040413	-0.00096	0.01118	-0.08544	0.931913	NA
USP17L10	0.040353	-0.00095	0.011175	-0.0854	0.931944	NA
ELOVL3	0.094762	-0.00095	0.017708	-0.05381	0.957089	NA
SNORA35	0.040142	-0.00095	0.011158	-0.08526	0.932052	NA
TRIM43B	0.040099	-0.00095	0.011154	-0.08524	0.932074	NA
OR10Q1	0.040099	-0.00095	0.011154	-0.08524	0.932074	NA
HOXC6	0.040099	-0.00095	0.011154	-0.08524	0.932074	NA
GNGT1	0.039989	-0.00095	0.011145	-0.08517	0.93213	NA
CYP2B6	0.039938	-0.00095	0.011141	-0.08513	0.932156	NA
RAET1K	0.092406	-0.00095	0.017618	-0.05372	0.957159	NA
UCK1	105.2712	-0.00095	0.052023	-0.01817	0.985504	NA
STRA8	0.039697	-0.00094	0.01112	-0.08498	0.932281	NA
NPBWR1	0.039697	-0.00094	0.01112	-0.08498	0.932281	NA
OR52N5	0.039682	-0.00094	0.011119	-0.08497	0.932289	NA
P2RY10	0.039658	-0.00094	0.011117	-0.08495	0.932301	NA
C1orf180	0.039648	-0.00094	0.011116	-0.08494	0.932306	NA

LOC10050	0.039611	-0.00094	0.011113	-0.08492	0.932325	NA
C20orf166	0.039611	-0.00094	0.011113	-0.08492	0.932325	NA
CAPZB	189.9333	-0.00094	0.051186	-0.01843	0.985296	NA
VTCN1	0.03956	-0.00094	0.011109	-0.08489	0.932352	NA
GUCY2EP	0.03956	-0.00094	0.011109	-0.08489	0.932352	NA
MIR4308	0.039389	-0.00094	0.011094	-0.08477	0.932441	NA
MELK	0.039303	-0.00094	0.011087	-0.08472	0.932486	NA
TUBG2	155.2753	0.000939	0.060023	0.015647	0.987516	NA
LINC00028	0.03921	-0.00094	0.011079	-0.08466	0.932534	NA
SYCN	0.039197	-0.00094	0.011078	-0.08465	0.932541	NA
SNORD48	7.930347	-0.00093	0.076844	-0.01216	0.990299	NA
AMPD1	0.038935	-0.00093	0.011055	-0.08448	0.932678	NA
SPANXN3	0.038911	-0.00093	0.011053	-0.08446	0.93269	NA
ITPKA	16.96321	-0.00093	0.084588	-0.01104	0.991194	NA
CCL26	0.038866	-0.00093	0.01105	-0.08443	0.932714	NA
MIR3143	0.038866	-0.00093	0.01105	-0.08443	0.932714	NA
ASCL2	0.038848	-0.00093	0.011048	-0.08442	0.932724	NA
TPRG1	3.072354	0.000932	0.074185	0.012568	0.989972	NA
KLK9	0.038829	-0.00093	0.011046	-0.08441	0.932734	NA
CLEC6A	0.038694	-0.00093	0.011035	-0.08432	0.932805	NA
RPL10L	0.038573	-0.00093	0.011024	-0.08424	0.932869	NA
DDX11-AS	4.932513	0.000928	0.078036	0.011886	0.990516	NA
GDEP	0.038426	-0.00093	0.011012	-0.08414	0.932947	NA
MYRIP	305.0422	-0.00093	0.046546	-0.01988	0.984136	NA
IMPA1	57.6231	-0.00092	0.064565	-0.01431	0.988586	NA
LOC10012	0.36187	-0.00092	0.033486	-0.02757	0.978007	NA
ANKRD35	83.39893	0.000923	0.063446	0.014544	0.988396	NA
ACTBL2	0.03812	-0.00092	0.010985	-0.08393	0.93311	NA
SLC7A3	0.038012	-0.00092	0.010976	-0.08386	0.933167	NA
TMPRSS15	0.100454	-0.00092	0.016184	-0.05685	0.954668	NA
LINC00518	0.037806	-0.00092	0.010958	-0.08372	0.933278	NA
GJA8	0.037806	-0.00092	0.010958	-0.08372	0.933278	NA
PIPOX	24.46617	-0.00092	0.08445	-0.01086	0.991334	NA
LINC00671	0.235433	-0.00092	0.027553	-0.03327	0.973461	NA
CCDC63	0.037746	-0.00092	0.010952	-0.08368	0.93331	NA
WFDC8	0.090461	-0.00092	0.01761	-0.05197	0.958555	NA
OR4D1	0.037549	-0.00091	0.010935	-0.08355	0.933416	NA
SPO11	0.037549	-0.00091	0.010935	-0.08355	0.933416	NA
LYZL6	0.037549	-0.00091	0.010935	-0.08355	0.933416	NA
HRASLS2	0.173775	0.000913	0.0216	0.042281	0.966275	NA
KRTAP21-1	0.037511	-0.00091	0.010932	-0.08352	0.933437	NA
SERPINB11	0.037452	-0.00091	0.010926	-0.08348	0.933469	NA
MIR133B	0.037452	-0.00091	0.010926	-0.08348	0.933469	NA
LEAP2	14.04425	0.00091	0.084381	0.010788	0.991393	NA
LOC10013	0.037326	-0.00091	0.010915	-0.0834	0.933537	NA
PLXND1	58.47627	0.00091	0.072756	0.012505	0.990022	NA
SMAD5-AS	6.939588	-0.00091	0.083606	-0.01088	0.991319	NA

SNCB	493.365	-0.00091	0.071163	-0.01276	0.989823	0.996637
RBPJL	0.037139	-0.00091	0.010899	-0.08327	0.933639	NA
DIP2A	436.3347	-0.0009	0.05948	-0.01521	0.987863	0.99622
DCAF4L2	0.036911	-0.0009	0.010878	-0.08311	0.933763	NA
SERPINB4	0.036897	-0.0009	0.010877	-0.0831	0.933771	NA
MIR4464	0.036897	-0.0009	0.010877	-0.0831	0.933771	NA
SUPT20HL	0.03689	-0.0009	0.010876	-0.0831	0.933775	NA
FOXA2	0.03685	-0.0009	0.010873	-0.08307	0.933797	NA
CDCA5	2.198379	-0.0009	0.068049	-0.01323	0.989441	NA
POTEA	0.036547	-0.0009	0.010846	-0.08286	0.933963	NA
DPPA3	0.036486	-0.0009	0.01084	-0.08282	0.933997	NA
GZMB	0.036419	-0.0009	0.010834	-0.08277	0.934034	NA
HSD3B2	0.036362	-0.0009	0.010829	-0.08273	0.934066	NA
TMEM54	11.88124	0.000895	0.084485	0.010588	0.991552	NA
GPR33	0.036244	-0.00089	0.010818	-0.08265	0.934131	NA
IFNL3	0.036145	-0.00089	0.010809	-0.08258	0.934186	NA
OR52N4	0.087423	-0.00089	0.017505	-0.05084	0.959455	NA
CSPP1	430.07	-0.00089	0.048828	-0.01819	0.985483	0.995611
UNC5B	282.8495	-0.00089	0.053646	-0.01654	0.986802	NA
HOXA4	0.035782	-0.00089	0.010776	-0.08232	0.934389	NA
GPR119	0.035782	-0.00089	0.010776	-0.08232	0.934389	NA
FAM24B-C	0.035672	-0.00089	0.010766	-0.08225	0.93445	NA
PCNAP1	0.035648	-0.00089	0.010764	-0.08223	0.934464	NA
SIGLEC12	0.035626	-0.00088	0.010762	-0.08221	0.934477	NA
VN1R4	0.035626	-0.00088	0.010762	-0.08221	0.934477	NA
OR10G2	0.035509	-0.00088	0.010751	-0.08213	0.934542	NA
CCR2	0.184603	0.000883	0.024569	0.035938	0.971332	NA
TSPAN16	1.533747	-0.00088	0.059947	-0.0147	0.98827	NA
ZNF767	246.3802	0.000881	0.052103	0.016908	0.98651	NA
CLLU1	0.035351	-0.00088	0.010737	-0.08202	0.934631	NA
FAM3B	0.035325	-0.00088	0.010734	-0.082	0.934646	NA
UTP20	212.5609	0.00088	0.049626	0.017731	0.985854	NA
IYD	0.035295	-0.00088	0.010731	-0.08198	0.934663	NA
NCRUPAR	0.035292	-0.00088	0.010731	-0.08198	0.934665	NA
MIR1272	0.035217	-0.00088	0.010724	-0.08192	0.934707	NA
FBXO22-A	0.03516	-0.00088	0.010719	-0.08188	0.93474	NA
PRPS1L1	0.03511	-0.00088	0.010714	-0.08185	0.934768	NA
LINC00326	0.035025	-0.00088	0.010706	-0.08179	0.934817	NA
KRT77	0.035022	-0.00088	0.010706	-0.08178	0.934819	NA
RASSF6	0.035004	-0.00088	0.010704	-0.08177	0.934828	NA
DLX6-AS1	0.034917	-0.00087	0.010696	-0.08171	0.934879	NA
LINC00668	0.034917	-0.00087	0.010696	-0.08171	0.934879	NA
SLC6A10P	0.034917	-0.00087	0.010696	-0.08171	0.934879	NA
MIR4794	0.034872	-0.00087	0.010692	-0.08168	0.934904	NA
LINC00593	0.034758	-0.00087	0.010681	-0.08159	0.934969	NA
SLC25A48	2.043544	-0.00087	0.06033	-0.01444	0.988478	NA
MIR185	0.034687	-0.00087	0.010675	-0.08154	0.93501	NA

MAS1	0.034645	-0.00087	0.010671	-0.08151	0.935034	NA
APOH	0.034574	-0.00087	0.010664	-0.08146	0.935075	NA
AHSG	0.034571	-0.00087	0.010664	-0.08146	0.935077	NA
ADA	9.93221	0.000868	0.084514	0.010273	0.991804	NA
ADAM30	0.034388	-0.00087	0.010647	-0.08133	0.935183	NA
DMRT1	0.034326	-0.00086	0.010641	-0.08128	0.935218	NA
CLK2P	0.642025	-0.00086	0.041517	-0.02076	0.983434	NA
ILF3	889.5322	-0.00086	0.033298	-0.02589	0.979346	0.994271
IFIT2	49.01644	0.000861	0.070998	0.012123	0.990328	NA
MIR548A3	0.033981	-0.00086	0.010608	-0.08103	0.935419	NA
SLC22A9	0.033925	-0.00086	0.010603	-0.08099	0.935452	NA
MIR4797	0.093545	-0.00086	0.017668	-0.04857	0.961265	NA
OR14I1	0.033474	-0.00085	0.010559	-0.08066	0.935716	NA
PCDHA6	74.6334	0.000851	0.06339	0.013424	0.98929	NA
SP7	3.083661	0.000849	0.077358	0.010972	0.991246	NA
SLC15A5	0.774993	0.000849	0.046302	0.01833	0.985375	NA
CD3D	0.033219	-0.00085	0.010535	-0.08047	0.935867	NA
ZNF724P	6.344799	-0.00085	0.082665	-0.01023	0.991839	NA
DGKD	789.5461	0.000843	0.045836	0.018388	0.985329	0.995611
APOBEC3A	0.032717	-0.00084	0.010486	-0.08009	0.936167	NA
FAM122A	129.5963	0.000837	0.048176	0.01738	0.986134	NA
LOC49455	0.032491	-0.00084	0.010463	-0.07992	0.936303	NA
EVX2	0.032409	-0.00083	0.010455	-0.07985	0.936353	NA
CCDC74B-	0.032409	-0.00083	0.010455	-0.07985	0.936353	NA
NLRP11	0.032049	-0.00083	0.01042	-0.07958	0.936571	NA
APOA4	0.032031	-0.00083	0.010418	-0.07957	0.936583	NA
CYP4F22	0.032031	-0.00083	0.010418	-0.07957	0.936583	NA
SULT1E1	0.031801	-0.00083	0.010395	-0.07939	0.936724	NA
KLRG2	0.031801	-0.00083	0.010395	-0.07939	0.936724	NA
MUC21	0.031801	-0.00083	0.010395	-0.07939	0.936724	NA
LOC28455	0.031801	-0.00083	0.010395	-0.07939	0.936724	NA
TCP10	0.031728	-0.00082	0.010388	-0.07933	0.936768	NA
RNASE8	0.09085	-0.00082	0.017557	-0.04683	0.962652	NA
43166	603.8791	0.000818	0.042265	0.019356	0.984557	0.995611
JAKMIP2	804.4596	0.000817	0.051152	0.015969	0.987259	0.996054
PRSS41	0.031162	-0.00081	0.01033	-0.07889	0.937119	NA
CYP2A13	0.031162	-0.00081	0.01033	-0.07889	0.937119	NA
LOC10050	0.031162	-0.00081	0.01033	-0.07889	0.937119	NA
WIPI2	191.6945	-0.00081	0.04094	-0.01988	0.984136	NA
CALML3	0.375699	-0.00081	0.034428	-0.02361	0.981164	NA
LOC15444	0.24243	0.000812	0.026318	0.030849	0.97539	NA
MYH8	0.030887	-0.00081	0.010302	-0.07868	0.937291	NA
CAMP	0.03083	-0.00081	0.010296	-0.07863	0.937327	NA
DIO3	0.210789	-0.00081	0.026558	-0.03043	0.975725	NA
AKR1D1	0.030615	-0.00081	0.010274	-0.07846	0.937462	NA
IFNG	0.030509	-0.0008	0.010263	-0.07838	0.937529	NA
OR10S1	0.030509	-0.0008	0.010263	-0.07838	0.937529	NA

LINC00661	0.030356	-0.0008	0.010247	-0.07825	0.937626	NA
CST9L	0.030341	-0.0008	0.010246	-0.07824	0.937636	NA
MIR4781	0.030287	-0.0008	0.01024	-0.0782	0.93767	NA
NKX2-1	0.030266	-0.0008	0.010238	-0.07818	0.937684	NA
HIST1H2B	0.519897	0.000798	0.039974	0.019967	0.98407	NA
FCAMR	0.096467	-0.0008	0.017736	-0.04497	0.964134	NA
LILRA3	0.029396	-0.00079	0.010146	-0.07748	0.938245	NA
GZMK	0.029314	-0.00078	0.010137	-0.07741	0.938298	NA
NRTN	0.087377	-0.00078	0.017572	-0.04457	0.964454	NA
HIGD2B	0.029161	-0.00078	0.010121	-0.07728	0.938398	NA
HOXB7	0.029161	-0.00078	0.010121	-0.07728	0.938398	NA
LOC40092	20.41029	0.000776	0.082086	0.009448	0.992462	NA
JRK	216.5748	0.000773	0.045319	0.017049	0.986397	NA
TMC7	98.66577	-0.00077	0.069051	-0.01119	0.991075	NA
TM7SF3	100.0619	0.000767	0.045875	0.016722	0.986659	NA
LOC28368	0.028248	-0.00077	0.010022	-0.07652	0.939005	NA
ATP5G3	123.5454	-0.00077	0.057664	-0.01329	0.989395	NA
MIR4285	0.101168	-0.00076	0.016159	-0.04714	0.962401	NA
FABP4	0.235423	0.00076	0.026547	0.028633	0.977157	NA
FAM50A	277.2796	-0.00076	0.053522	-0.01418	0.988689	NA
TCN2	16.36646	-0.00076	0.084864	-0.00892	0.992884	NA
RETN	0.089781	-0.00075	0.015892	-0.04743	0.962168	NA
MIR4746	0.509935	-0.00075	0.039717	-0.01897	0.984868	NA
MIR548AI	0.026722	-0.00074	0.009851	-0.0752	0.940053	NA
TAS1R2	0.026722	-0.00074	0.009851	-0.0752	0.940053	NA
ZNF350	87.84031	0.000737	0.06236	0.011813	0.990575	NA
NUDT9P1	3.83243	0.000736	0.078983	0.009318	0.992565	NA
ZNF454	91.9928	0.000726	0.059905	0.012124	0.990327	NA
ZNF518A	380.3057	0.000725	0.058091	0.012488	0.990037	0.996637
MIR4439	0.310521	-0.00072	0.031383	-0.02304	0.981615	NA
LINC00637	2.75332	0.000722	0.060441	0.011942	0.990472	NA
IFNA5	0.228436	0.000715	0.027765	0.025764	0.979446	NA
IRF8	28.97218	-0.00071	0.082038	-0.00869	0.993064	NA
GRAP	1.964807	-0.00071	0.068013	-0.01047	0.991646	NA
LIPG	36.45048	0.000712	0.084423	0.00843	0.993274	NA
OBP2A	0.098101	-0.00071	0.016052	-0.04406	0.964855	NA
DDT	0.091783	-0.0007	0.017622	-0.03976	0.968285	NA
CLRN1	0.341942	-0.0007	0.031178	-0.02244	0.982097	NA
MKLN1	712.6931	0.000697	0.039572	0.017622	0.98594	0.995611
MUC15	0.085318	-0.0007	0.017452	-0.0399	0.968176	NA
GGNBP1	0.173091	-0.00069	0.023209	-0.02979	0.976238	NA
TPSB2	0.09462	-0.00068	0.017666	-0.03851	0.969284	NA
ISG15	3.498082	-0.00067	0.068863	-0.00978	0.992195	NA
CNNM2	311.1848	0.00067	0.054791	0.01222	0.99025	NA
ADSS	88.81041	-0.00067	0.057617	-0.0116	0.990742	NA
MIR758	0.947067	-0.00067	0.051097	-0.01302	0.989611	NA
LOC10028	26.30718	0.000657	0.084716	0.007753	0.993814	NA

CPA4	0.238372	-0.00065	0.025113	-0.02574	0.979463	NA
LOC64362	0.661596	0.000639	0.045283	0.014119	0.988735	NA
TMEM174	0.123845	-0.00064	0.017917	-0.03555	0.971641	NA
STK19	31.00518	0.000631	0.071438	0.008832	0.992953	NA
NKAPP1	55.44211	-0.00062	0.077263	-0.00805	0.993579	NA
ELOVL7	100.6987	-0.00062	0.060612	-0.01025	0.991821	NA
POSTN	0.30985	0.000619	0.030746	0.020134	0.983937	NA
CCDC50	272.8888	-0.00062	0.038135	-0.01623	0.98705	NA
CPNE3	197.5381	0.000615	0.052658	0.011672	0.990687	NA
CEMP1	12.87058	-0.00061	0.08234	-0.00745	0.994053	NA
CITED2	155.8861	-0.00061	0.062435	-0.00981	0.992172	NA
SLC12A7	30.84747	-0.00061	0.082692	-0.00739	0.994103	NA
SCOC	178.2516	0.000607	0.07674	0.007914	0.993686	NA
C2CD4B	0.586927	0.000606	0.038027	0.015946	0.987277	NA
LINC00487	0.149273	0.000601	0.021363	0.028142	0.977549	NA

0.983733 NA
0.975715 NA
0.973016 NA
0.984039 NA
0.991174 NA
0.975854 NA
0.980895 NA
0.973758 NA
0.982099 NA
0.974178 NA
0.970966 NA
0.988539 0.996458
0.974958 NA
0.987647 NA

0.99324 NA
0.974979 NA
0.980701 NA
0.983431 NA
0.992298 NA
0.982982 NA
0.993037 NA
0.994667 NA
0.994031 NA
0.97423 NA
0.977212 NA
0.995105 NA
0.977125 NA

LINC00332	0.104913	-0.0005	0.017864	-0.02801	0.977653	NA
LOC72822	0.806268	-0.0005	0.04764	-0.01049	0.991631	NA
LOC44115	0.115198	-0.00049	0.018086	-0.02728	0.978237	NA
HMGXB3	141.3725	0.000492	0.047106	0.01044	0.99167	NA
OR2M4	0.164021	0.00049	0.022779	0.021528	0.982824	NA

PCSK1N	126.1416	-0.00049	0.078296	-0.00624	0.995019	NA
LINC00670	0.163717	-0.00049	0.018124	-0.02688	0.978555	NA
SLC25A24	35.73743	-0.00049	0.08053	-0.00604	0.995178	NA
MIR1238	0.099476	-0.00049	0.017747	-0.02737	0.978163	NA
CRIM1	312.5268	-0.00048	0.060735	-0.00795	0.99366	NA
CD300LB	0.092475	0.000481	0.017648	0.027268	0.978246	NA
OR2A7	0.096239	-0.00048	0.017682	-0.02719	0.978311	NA
GPR135	48.56356	0.000479	0.068444	0.006998	0.994417	NA
DIMT1	63.11211	-0.00048	0.064393	-0.00741	0.99409	NA
MIR4444-1	0.099786	-0.00047	0.017746	-0.02674	0.978665	NA
GP2	0.101527	-0.00047	0.01779	-0.02661	0.97877	NA
KLHL4	15.45803	0.000473	0.084818	0.005581	0.995547	NA
RNF151	1.31146	0.000468	0.055787	0.008396	0.993301	NA
ZNF560	0.58045	0.000462	0.042587	0.010847	0.991345	NA
OVOL1	2.072221	0.000462	0.066906	0.006902	0.994493	NA
CLCA2	0.344613	-0.00046	0.034724	-0.01321	0.989458	NA
ASTE1	44.04293	0.000459	0.07811	0.005873	0.995314	NA
C2orf88	72.52903	0.00045	0.071857	0.006258	0.995007	NA
PCNX	695.0422	0.000442	0.034444	0.012832	0.989762	0.996637
GABRB1	75.98201	0.000439	0.069991	0.006275	0.994993	NA
ZFYVE20	405.9853	0.000433	0.05469	0.007924	0.993678	0.99897
UBE2A	91.27889	0.000427	0.057923	0.00738	0.994111	NA
TMEM180	81.003	-0.00043	0.066367	-0.00643	0.994872	NA
SLC22A12	4.674378	0.000418	0.072164	0.005797	0.995375	NA
ASH2L	167.119	0.000413	0.064611	0.006399	0.994894	NA
RNF168	353.8995	-0.00041	0.047116	-0.00867	0.993085	0.998818
GOLGA8T	6.474082	0.000403	0.084442	0.004777	0.996188	NA
LHX2	0.327329	0.000398	0.032257	0.012344	0.990151	NA
HCG25	5.220389	-0.00039	0.083024	-0.00469	0.996259	NA
SV2C	18.46673	-0.00039	0.078212	-0.00496	0.996041	NA
NAAA	39.12036	-0.00039	0.070796	-0.00548	0.995632	NA
AGL	284.8405	0.000387	0.043251	0.008956	0.992854	NA
CHMP4A	143.8522	-0.00039	0.062184	-0.00622	0.995038	NA
BPI	0.391644	-0.00038	0.032162	-0.01191	0.990497	NA
RNF207	141.8633	-0.00038	0.072888	-0.00524	0.995821	NA
HNF1A-AS	0.102434	-0.00038	0.017823	-0.02126	0.98304	NA
ZNF579	59.88969	-0.00037	0.063491	-0.0059	0.995289	NA
SLC5A7	0.199132	-0.00037	0.023329	-0.01578	0.987409	NA
ANKRD10	223.0395	0.000365	0.05289	0.006904	0.994491	NA
SMYD1	0.098072	-0.00036	0.016054	-0.02264	0.981939	NA
OR2L13	5.397697	-0.00036	0.050357	-0.00706	0.99437	NA
C1QTNF8	0.338268	0.000354	0.032982	0.010735	0.991435	NA
RPL7	305.0553	0.000354	0.068341	0.005179	0.995868	NA
PRKACB	1238.495	-0.00035	0.055927	-0.00625	0.995013	0.999679
CRNN	0.096753	-0.00035	0.017682	-0.01964	0.984327	NA
BUB3	117.6149	0.000343	0.048107	0.00714	0.994303	NA
PSTPIP2	3.766544	-0.00034	0.074422	-0.0046	0.99633	NA

SH2D4B	0.202878	0.00034	0.025775	0.013183	0.989482	NA
PLA2G2E	0.093548	-0.00034	0.017667	-0.01911	0.98475	NA
MCHR1	3.717752	-0.00034	0.069834	-0.00483	0.996144	NA
CMAHP	14.06564	0.000332	0.081805	0.004064	0.996757	NA
TICAM2	2.537947	0.000329	0.072268	0.004554	0.996366	NA
XCL1	0.151476	0.000328	0.022631	0.014503	0.988428	NA
LCE1B	0.084102	-0.00033	0.015753	-0.02083	0.98338	NA
MACROD2	107.9554	0.000327	0.063959	0.005106	0.995926	NA
NMU	0.129366	0.000324	0.017754	0.018248	0.985441	NA
C5orf28	19.10231	-0.00032	0.080695	-0.00397	0.996832	NA
SDCBP	168.6058	0.000316	0.056326	0.005616	0.995519	NA
TRIM71	0.949028	-0.00032	0.053279	-0.00593	0.995271	NA
OR2A14	0.102592	-0.00031	0.016165	-0.01949	0.984454	NA
TIPARP	84.57722	0.000306	0.059507	0.005141	0.995898	NA
LRRC3B	67.82489	0.000303	0.066164	0.004583	0.996344	NA
PI4K2B	28.90704	-0.00029	0.077474	-0.00379	0.996974	NA
PRDM2	938.4254	-0.00029	0.02919	-0.00991	0.992094	0.998264
SLC22A23	294.7566	0.000289	0.045849	0.006294	0.994978	NA
ZNF830	65.46815	-0.00029	0.061115	-0.00472	0.996237	NA
TBXA2R	0.7646	0.000288	0.046726	0.006156	0.995088	NA
NPY2R	0.216844	-0.00029	0.019954	-0.01435	0.988554	NA
PDYN	8.479809	0.00028	0.084726	0.003307	0.997361	NA
SIMC1	56.64234	-0.00028	0.05798	-0.00483	0.99615	NA
GLYATL1	0.219831	-0.00028	0.021381	-0.01308	0.989562	NA
NKG7	0.720654	0.000277	0.049074	0.005653	0.99549	NA
LOC44245	0.096488	-0.00028	0.017677	-0.01564	0.987521	NA
MFSD2B	7.146549	0.000274	0.084603	0.003239	0.997416	NA
NFE2L1	481.7746	-0.00027	0.049855	-0.00549	0.995623	0.999679
ADIG	0.105026	-0.00027	0.017882	-0.01491	0.988103	NA
C14orf142	12.81846	0.000264	0.08408	0.003143	0.997492	NA
NT5M	15.07305	0.000263	0.083256	0.003158	0.99748	NA
MAPT-IT1	102.9139	0.000254	0.065426	0.003883	0.996902	NA
LOC14764	0.099333	-0.00025	0.017747	-0.01428	0.988603	NA
MIR302A	0.338148	-0.00025	0.03403	-0.00739	0.994107	NA
MIR4682	0.200421	-0.00024	0.024682	-0.00985	0.992142	NA
BANF2	0.188056	-0.00024	0.024617	-0.00986	0.992135	NA
AARSD1	1.212899	-0.00024	0.057052	-0.00418	0.996665	NA
MAP10	16.94585	-0.00024	0.084522	-0.00282	0.997752	NA
RBM22	122.8735	-0.00024	0.04236	-0.00557	0.995552	NA
INSM2	4.537952	0.000234	0.081002	0.002892	0.997692	NA
ARIH2OS	6.586958	0.000234	0.084741	0.00276	0.997798	NA
HOMEZ	39.59182	0.000231	0.069668	0.003312	0.997357	NA
FEZF1-AS1	1.279731	0.000229	0.055001	0.004171	0.996672	NA
OR56B1	0.086825	0.000224	0.017474	0.012835	0.989759	NA
MIPEP	24.81433	0.000222	0.072967	0.003048	0.997568	NA
NAT8B	4.946219	-0.00022	0.078629	-0.0028	0.997764	NA
CXCL1	2.917989	-0.00022	0.056469	-0.00386	0.996917	NA

ARMC1	131.9899	0.000217	0.066016	0.00328	0.997383	NA
FLRT2	27.04232	0.000216	0.084756	0.002547	0.997968	NA
SBF2-AS1	23.19242	-0.00022	0.080328	-0.00269	0.997857	NA
HPCA	75.66277	0.000206	0.084856	0.00243	0.998061	NA
ATP12A	0.433165	0.000205	0.036388	0.005636	0.995503	NA
ZNF619	34.92857	-0.0002	0.07339	-0.00278	0.997778	NA
ITGAD	0.811161	-0.0002	0.048322	-0.00418	0.996662	NA
KAZN	525.5032	-0.0002	0.03913	-0.00516	0.995881	0.999679
C1QTNF9	0.18244	0.0002	0.025604	0.00783	0.993753	NA
LAPTM4B	22.26838	0.0002	0.084789	0.00236	0.998117	NA
FAM71C	53.09136	-0.00019	0.074601	-0.00259	0.99793	NA
ZNF837	10.9854	-0.00019	0.084728	-0.00228	0.99818	NA
CHURC1	181.1029	-0.00019	0.060046	-0.00318	0.997462	NA
RAB2A	351.623	-0.00019	0.057652	-0.00323	0.997422	0.999679
C1QBP	97.32076	0.000181	0.081067	0.002232	0.998219	NA
FAM66D	21.80915	-0.00018	0.084887	-0.00212	0.998312	NA
NME4	71.77554	0.000179	0.060335	0.002969	0.997631	NA
GABBR1	1234.821	0.000175	0.055336	0.003169	0.997472	0.999679
PLXNB1	294.2648	-0.00017	0.056678	-0.00308	0.997542	NA
HBQ1	1.025535	0.000168	0.054801	0.003074	0.997547	NA
ZC3H3	83.10574	0.000168	0.06282	0.002675	0.997866	NA
LOC28611	0.101038	-0.00017	0.016139	-0.01038	0.991719	NA
CRY2	288.0193	-0.00016	0.050742	-0.0032	0.997446	NA
FAM66A	9.18559	0.000159	0.07849	0.002029	0.998381	NA
IGSF9	0.662703	0.000159	0.04543	0.0035	0.997207	NA
ANKRD37	14.32044	0.000158	0.084793	0.001862	0.998514	NA
C6orf89	204.4614	0.000153	0.048188	0.003172	0.997469	NA
PDSS1	44.78789	0.000147	0.065435	0.00225	0.998205	NA
PDCD6IP	400.1365	-0.00015	0.039587	-0.00369	0.997057	0.999679
FLJ35282	0.716411	-0.00014	0.045799	-0.00312	0.99751	NA
MIR331	1.485662	-0.00014	0.06128	-0.00229	0.998176	NA
RETSAT	63.89568	-0.00013	0.059556	-0.00212	0.998305	NA
USP8	393.8455	-0.00012	0.033958	-0.00356	0.99716	0.999679
DYNAP	0.093109	0.000117	0.015943	0.007308	0.994169	NA
MIR1976	0.339272	-0.00011	0.03087	-0.00352	0.997191	NA
SORL1	1793.413	-0.00011	0.047069	-0.0023	0.998164	0.99969
DSTN	392.821	-0.00011	0.073905	-0.00145	0.998846	0.99969
OR51B4	0.915272	9.75E-05	0.046424	0.002099	0.998325	NA
XPO4	285.5687	-9.49E-05	0.040018	-0.00237	0.998107	NA
NMRK1	86.0539	-9.17E-05	0.068167	-0.00134	0.998927	NA
DNAJC10	170.6821	8.55E-05	0.069326	0.001233	0.999016	NA
CLPS	0.09636	8.45E-05	0.017701	0.004775	0.99619	NA
LYPD2	0.196226	-7.86E-05	0.025718	-0.00306	0.99756	NA
CDC73	324.053	7.33E-05	0.032382	0.002263	0.998194	NA
RASL10A	9.595067	7.14E-05	0.084611	0.000844	0.999326	NA
CASP14	0.102296	-6.94E-05	0.017845	-0.00389	0.996898	NA
MORF4L1	449.6133	6.61E-05	0.058283	0.001134	0.999095	0.99969

CLRN1-AS	0.099479	5.31E-05	0.017774	0.002986	0.997618	NA
ITGA10	51.55403	-4.52E-05	0.084939	-0.00053	0.999575	NA
OLA1	251.792	4.48E-05	0.063651	0.000704	0.999438	NA
HNF1B	0.095311	4.42E-05	0.017778	0.002487	0.998015	NA
PSKH2	0.091208	-4.17E-05	0.017558	-0.00238	0.998105	NA
FAM157B	0.300835	3.87E-05	0.031747	0.00122	0.999027	NA
NTSR1	0.295402	3.34E-05	0.030471	0.001097	0.999124	NA
SLC26A4-A	0.95867	2.91E-05	0.050651	0.000574	0.999542	NA
CLEC4GP1	58.69818	2.90E-05	0.082848	0.00035	0.999721	NA
UBE2M	102.8374	-2.68E-05	0.05823	-0.00046	0.999632	NA
YTHDC1	833.4848	-2.55E-05	0.027031	-0.00094	0.999248	0.99969
SF3B2	924.8883	1.67E-05	0.06268	0.000266	0.999788	0.999788
IFNA17	0.095385	-1.42E-05	0.016019	-0.00088	0.999294	NA
MSX2P1	0.657875	1.42E-05	0.045325	0.000314	0.999749	NA
PAX5	0.436061	-9.51E-06	0.037712	-0.00025	0.999799	NA
GTSF1	0.399387	-9.29E-06	0.025032	-0.00037	0.999704	NA
GPR128	0	0	0	0	1	NA
