# Supplementary material for: Exploring the diagnostic markers of essential tremor: A study based on machine learning algorithms
Source: Open Life Sci. 2023 Jun 22;18(1):20220622. doi: 10.1515/biol-2022-0622 (PMC10290283; doi:10.1515/biol-2022-0622)
Supplement: Supplementary Table 3 [file biol-2022-0622-sm4.pdf]

Table S3: ET-related DEGs in the GSE134878 dataset

Gene	p-value
APOE	0.006608
ZNF148	0.013042
FAM169A	0.022413
SHROOM3	0.022508
EFR3A	0.023764
SENP6	0.035061
KDM3A	0.041384
NISCH	0.049757
ZDHHC2	0.058298
SYNJ1	0.059264
CREB1	0.063827
FAM126B	0.072241
HNRNPC	0.079332
FZD3	0.085647
ZFR	0.087567
SRRM3	0.093294
NCKAP1	0.10002
FNIP1	0.104085
BTBD3	0.104497
ST8SIA3	0.110566
PKP4	0.127331
CLOCK	0.128858
ZIC4	0.128974
RYR1	0.140457
ATF7IP	0.14306
AHCYL1	0.153453
ZMYND11	0.153571
TMEM259	0.153641
ANKRD17	0.157201
ZBTB41	0.158713
RPL13	0.159053
RPS3	0.159538
UGGT1	0.160515
KLF7	0.161642
TULP4	0.170925
SCAMP1	0.17165
NBEA	0.177701
NMNAT2	0.180353
SPG7	0.182547
KCND2	0.18364
KIAA1109	0.184146
PURA	0.184159
GOLGA8A	0.18752
GFPT1	0.196532
ATP2B1	0.197352
TTC37	0.198351

LNPEP	0.201165
INCENP	0.205952
SETD2	0.210777
PTPN4	0.212357
RAB6B	0.214884
FASN	0.219158
EXOC5	0.220834
FAM214A	0.227317
CD47	0.228417
LYST	0.235553
GUCY1A2	0.239292
KLF12	0.243441
ZNF507	0.250366
CAND1	0.250406
EIF4G2	0.253002
NSD1	0.25818
MYCBP2	0.259145
CDS2	0.271204
SLC25A27	0.27221
PUM2	0.275538
MYO6	0.276767
KIF5B	0.277126
RAPGEF2	0.279859
SMG5	0.291648
NR3C1	0.293468
MEGF8	0.299965
TSPYL4	0.31167
TMEM170	0.31309
RBM12	0.32011
ABCA2	0.321058
ROCK2	0.323181
TAOK1	0.328089
APPL1	0.329786
CAMSAP2	0.332963
ADNP	0.345694
OGFRL1	0.347708
PHRF1	0.356058
KIF2A	0.359065
HECTD1	0.360876
SSH1	0.363295
DYNC1H1	0.366502
ZNF483	0.376497
GLS	0.3787
AMER3	0.391045
STXBP5L	0.392603
RPL19	0.396782
RPL31	0.397408

PRRT2	0.399084
CFLAR	0.409833
PPP1R1B	0.425064
CAPN2	0.428248
RUSC2	0.431223
TLN2	0.432474
C2CD5	0.436924
UBTF	0.43757
GPR158	0.442431
ARCN1	0.45096
USP7	0.452733
RANBP2	0.46314
PRKCB	0.469208
SEL1L3	0.469333
ENAH	0.469333
SETX	0.480567
CDK17	0.484464
LENG8	0.499507
NDRG2	0.506495
CAMTA1	0.507702
DDX17	0.507752
MAPK1	0.50947
WAC	0.509576
QSER1	0.511447
NR1D2	0.518851
ARHGEF12	0.523531
FAT2	0.526516
KLC2	0.52865
COPB2	0.536793
KLC1	0.540823
TSC1	0.541291
PIK3R3	0.548535
NEUROD1	0.551871
DCAF8	0.555467
CCDC88B	0.557011
AFTPH	0.56475
PURB	0.572383
CALN1	0.573833
AGT	0.581054
NDRG3	0.581301
MEF2A	0.584207
OPCML	0.586576
EPHA4	0.586723
UBE4B	0.586996
TCF25	0.589778
S100B	0.594139
MLH3	0.597981

DNM3	0.604636
CCNG2	0.609469
C5orf24	0.61377
BMPR2	0.625093
HNRNPH3	0.625311
LIN7C	0.625675
PRR14L	0.633425
TEX2	0.636694
LONRF2	0.642672
FYN	0.642815
EPB41L1	0.648155
SPTAN1	0.651497
AGAP1	0.652198
CERK	0.653726
CPD	0.65476
SPHKAP	0.655913
FUT9	0.659887
UNC80	0.669967
PTAR1	0.676304
CHGB	0.67701
SPTBN5	0.67959
ZHX1	0.680062
UBQLN1	0.687369
RAB11FIP4	0.688619
EEF2K	0.694693
KIF5A	0.695475
LINC00641	0.700873

EXPH5	0.703528
ANKZF1	0.706413
HIVEP2	0.723754
TAX1BP1	0.72552
NOLC1	0.730858
SYN2	0.739046
RMND5A	0.750244
NCOA6	0.766416
NUMA1	0.768849
AFF3	0.772398
EIF4G3	0.774692
TP53BP1	0.78133
SMG7	0.784259
PTGDS	0.784674
NREP	0.801081
MIA3	0.802254
RABEP1	0.802463
ZIC1	0.808393
TTBK2	0.808877
RC3H2	0.821533

BAALC	0.828904
JUN	0.831556
DOCK4	0.834153
PRKCE	0.836168
RAB3GAP1	0.837911
JARID2	0.845601
FAM13B	0.848537
SBNO1	0.859107
PAGR1	0.861909
SRSF11	0.8718
GABRB3	0.877102
ZBTB18	0.879281
TGOLN2	0.882394
TSPYL2	0.887872
MGAT5	0.900242
CALM1	0.905465
ZNF91	0.90657
YWHAG	0.908143
ADCY1	0.92318
UBP1	0.928602
PRPF8	0.928624
CALD1	0.938221
GPBP1	0.939183
SLITRK4	0.94262
FNBP4	0.944535
LMTK2	0.947666
RPS6	0.948542
RPS8	0.949781
FAM107A	0.967413
HUWE1	0.971469
CNKSR2	0.980726
KIAA0232	0.983445
