# Supplementary material for: Exploring the diagnostic markers of essential tremor: A study based on machine learning algorithms
Source: Open Life Sci. 2023 Jun 22;18(1):20220622. doi: 10.1515/biol-2022-0622 (PMC10290283; doi:10.1515/biol-2022-0622)
Supplement: Supplementary Table 4 [file biol-2022-0622-sm5.pdf]

Table S4: DEGs in the pathogenic course of ET

Symbol	base Mean	log2FoldC	lfcSE	stat	p-value	p-adj
3-Sep	1087.725	0.165123	0.055159	2.993585	0.002757	0.130106
ABCA2	1276.469	-0.11166	0.048554	-2.29977	0.021461	0.23963
ADCY1	1262.301	0.161575	0.05785	2.79299	0.005222	0.151649
ADNP	565.4917	0.085463	0.036254	2.35735	0.018406	0.231397
AFF3	776.8776	0.094434	0.040257	2.34577	0.018988	0.231397
AFTPH	351.6055	0.125007	0.044579	2.804169	0.005045	0.151649
AGAP1	758.7865	0.095945	0.038897	2.466638	0.013639	0.208729
AGT	407.1206	0.213945	0.061043	3.504835	0.000457	0.075062
AHCYL1	959.9883	-0.17657	0.075346	-2.34349	0.019104	0.231397
AMER3	366.5572	0.159166	0.06215	2.560992	0.010437	0.198661
ANKRD17	1292.872	0.123172	0.028514	4.319776	1.56E-05	0.035377
ANKZF1	416.2827	-0.22703	0.066542	-3.41182	0.000645	0.075062
APOE	911.6687	-0.21142	0.074193	-2.84961	0.004377	0.147979
APPL1	518.5237	0.08789	0.038359	2.291239	0.02195	0.23963
ARCN1	399.5768	0.138754	0.055867	2.483655	0.013004	0.205975
ARHGEF12	2088.15	0.091974	0.029469	3.12108	0.001802	0.101784
ATF7IP	700.696	0.084648	0.033329	2.539738	0.011094	0.201925
ATP2B1	1153.623	0.110789	0.046095	2.403514	0.016238	0.223611
BAALC	420.1047	-0.21555	0.074887	-2.87839	0.003997	0.14186
BMPR2	784.1397	0.165937	0.050951	3.256809	0.001127	0.092144
BTBD3	748.5475	0.179792	0.064943	2.768467	0.005632	0.155569
C2CD5	585.2146	-0.07965	0.032429	-2.45616	0.014043	0.212051
C5orf24	358.3277	0.149471	0.050871	2.938238	0.003301	0.137333
CALD1	696.9177	-0.12794	0.050908	-2.51311	0.011967	0.202283
CALM1	5734.555	0.115388	0.049712	2.321115	0.020281	0.235785
CALN1	1330.712	0.146145	0.059794	2.444144	0.01452	0.212327
CAMSAP2	1285.017	0.088738	0.03942	2.251105	0.024379	0.246583
CAMTA1	1077.72	0.133921	0.049577	2.701247	0.006908	0.170072
CAND1	583.8895	0.162285	0.043637	3.718979	0.0002	0.075062
CAPN2	360.2258	-0.12319	0.051307	-2.40107	0.016347	0.223611
CCDC88B	462.6303	-0.21961	0.082093	-2.67512	0.00747	0.171303
CCNG2	509.3523	0.135226	0.059929	2.256442	0.024043	0.246583
CD47	655.0265	0.077926	0.032638	2.387559	0.016961	0.224654
CDK17	353.746	0.097801	0.042051	2.325774	0.020031	0.235785
CDS2	956.4319	0.093597	0.040949	2.285708	0.022271	0.23963
CERK	338.2441	-0.14368	0.064155	-2.2396	0.025117	0.248425
CFLAR	1204.36	-0.11725	0.052287	-2.24248	0.02493	0.247662
CHGB	3077.89	0.204385	0.080507	2.538729	0.011126	0.201925
CLOCK	758.9671	0.14164	0.039848	3.55451	0.000379	0.075062
CNKSR2	1694.856	0.100231	0.032854	3.050796	0.002282	0.120222
COPB2	425.106	0.118734	0.046525	2.552054	0.010709	0.200462
CPD	343.3556	0.095431	0.04015	2.376839	0.017462	0.227303
CPLX3	390.2462	-0.27091	0.079155	-3.42253	0.00062	0.075062
CREB1	464.2112	0.127313	0.040619	3.13431	0.001723	0.101784
DCAF8	650.3343	-0.12017	0.047699	-2.51946	0.011754	0.201925
DDX17	3403.818	-0.10682	0.039879	-2.67862	0.007393	0.171303

DNM3	2008.576	0.151386	0.053266	2.842082	0.004482	0.14929
DOCK4	700.3528	-0.20652	0.052555	-3.92954	8.51E-05	0.075062
DYNC1H1	6394.84	0.11977	0.040211	2.978521	0.002896	0.131209
EEF2K	522.1596	-0.10103	0.042647	-2.36907	0.017833	0.2295
EFR3A	918.7088	0.146475	0.05875	2.493182	0.01266	0.205975
EIF4G2	1105.604	0.130654	0.052287	2.49876	0.012463	0.205975
EIF4G3	839.6219	0.131044	0.039149	3.347363	0.000816	0.084436
ENAH	345.7586	0.102346	0.041346	2.475372	0.01331	0.206483
EPB41L1	732.1714	0.160385	0.063518	2.525043	0.011568	0.201925
EPHA4	347.997	0.127722	0.05569	2.29346	0.021822	0.23963
ERBB2IP	344.0113	-0.18383	0.073991	-2.48451	0.012973	0.205975
EXOC5	382.7537	0.103552	0.040205	2.575605	0.010006	0.193715
EXPH5	1890.167	0.105335	0.045777	2.301042	0.021389	0.23963
FAM107A	1700.57	-0.19554	0.078695	-2.48475	0.012964	0.205975
FAM126B	623.087	0.095788	0.042123	2.274002	0.022966	0.23963
FAM13B	475.5334	0.141663	0.054064	2.620309	0.008785	0.180879
FAM169A	411.0722	0.110731	0.047528	2.329782	0.019818	0.235011
FAM214A	394.5956	-0.17679	0.053854	-3.28267	0.001028	0.092144
FAM63B	525.6363	0.108127	0.044112	2.451164	0.014239	0.212327
FASN	1301.018	-0.18637	0.066494	-2.80283	0.005066	0.151649
FAT2	6694.747	0.161156	0.059834	2.69339	0.007073	0.171303
FNBP4	841.8916	-0.10384	0.044816	-2.31693	0.020508	0.235785
FNIP1	356.7268	-0.08575	0.036774	-2.33182	0.01971	0.234966
FUT9	1979.706	0.138666	0.042996	3.225107	0.001259	0.095074
FYN	494.9973	-0.09297	0.038578	-2.40996	0.015954	0.223064
FZD3	442.836	0.113792	0.036698	3.100785	0.00193	0.104087
GABRB3	337.6968	0.132346	0.049386	2.679841	0.007366	0.171303
GATS	686.734	-0.16065	0.05583	-2.8775	0.004008	0.14186
GFPT1	351.3817	0.103057	0.036479	2.82506	0.004727	0.150804
GLS	934.4241	0.166049	0.059108	2.809225	0.004966	0.151649
GOLGA8A	724.0041	-0.25939	0.073556	-3.5265	0.000421	0.075062
GPBP1	690.9469	0.112666	0.029735	3.788987	0.000151	0.075062
GPR158	1040.506	0.199928	0.054668	3.657124	0.000255	0.075062
GUCY1A2	473.9457	0.175293	0.070031	2.50308	0.012312	0.205975
HECTD1	2060.454	0.095112	0.040082	2.372951	0.017647	0.228398
HIVEP2	1574.396	0.114412	0.047756	2.395765	0.016586	0.223611
HNRNPC	721.8801	0.10588	0.039619	2.672432	0.00753	0.171303
HNRNPH3	684.9321	0.073054	0.032556	2.243938	0.024836	0.247662
HUWE1	2707.005	0.072704	0.030941	2.349774	0.018785	0.231397
INCENP	394.4628	-0.18098	0.074055	-2.44388	0.01453	0.212327
JARID2	721.0708	-0.13598	0.053898	-2.52287	0.01164	0.201925
JUN	397.3504	0.219934	0.06629	3.317743	0.000907	0.089367
KCND2	1159.267	0.120099	0.053245	2.255606	0.024095	0.246583
KDM3A	348.2205	-0.13035	0.054555	-2.38924	0.016883	0.224654
KIAA0232	634.1604	0.145941	0.042098	3.466722	0.000527	0.075062
KIAA0947	657.5347	0.0946	0.036681	2.578984	0.009909	0.193485
KIAA1109	2380.784	0.100771	0.030978	3.25292	0.001142	0.092144

KIF2A	367.1364	0.09475	0.041579	2.278798	0.022679	0.23963
KIF5A	1974.778	0.161772	0.054688	2.958073	0.003096	0.135775
KIF5B	1249.729	0.073551	0.032248	2.280782	0.022561	0.23963
KLC1	1513.245	0.117923	0.042728	2.759884	0.005782	0.156126
KLC2	497.0602	0.124547	0.051479	2.419368	0.015547	0.218727
KLF12	358.99	0.132231	0.04961	2.665428	0.007689	0.171303
KLF7	446.4035	0.114183	0.046768	2.441455	0.014628	0.212391
LENG8	1522.465	-0.13695	0.050377	-2.71861	0.006556	0.16684
LIN7C	534.0208	0.140933	0.055993	2.516992	0.011836	0.201925
LINC00641	542.1291	0.165054	0.050671	3.25735	0.001125	0.092144
LMTK2	549.0593	0.102509	0.045811	2.237647	0.025244	0.248599
LNPEP	562.3676	0.097222	0.036632	2.654034	0.007954	0.171303
LONRF2	1586.164	0.118178	0.044285	2.668603	0.007617	0.171303
LYST	1378.301	0.081994	0.026301	3.117537	0.001824	0.101784
MAPK1	624.742	0.151417	0.051454	2.942766	0.003253	0.137333
MEF2A	459.3332	0.106281	0.045333	2.344457	0.019055	0.231397
MEGF8	547.92	0.09718	0.040065	2.425533	0.015286	0.216391
MGAT5	501.2921	0.111777	0.049755	2.246559	0.024668	0.247228
MIA3	940.0879	0.088931	0.033494	2.655135	0.007928	0.171303
MLH3	420.3315	0.100205	0.034732	2.88509	0.003913	0.14186
MUM1	420.9292	-0.14477	0.048255	-3.00008	0.002699	0.130106
MYCBP2	2509.309	0.079174	0.033295	2.377954	0.017409	0.227303
MYO6	697.3984	-0.09128	0.03859	-2.36532	0.018015	0.229527
NBEA	2085.078	0.089336	0.036802	2.427495	0.015204	0.216391
NCKAP1	1149.88	0.114533	0.048771	2.348388	0.018855	0.231397
NCOA6	589.7335	0.12372	0.03634	3.404523	0.000663	0.075062
NDRG2	1206.873	-0.2055	0.074471	-2.75944	0.00579	0.156126
NDRG3	554.2505	0.149435	0.06151	2.429455	0.015122	0.216391
NEUROD1	741.4964	0.158431	0.063992	2.475781	0.013294	0.206483
NISCH	1915.82	-0.2201	0.069944	-3.14682	0.001651	0.101784
NMNAT2	1151.673	0.126219	0.054127	2.331924	0.019705	0.234966
NOLC1	361.2226	0.142219	0.049654	2.864198	0.004181	0.14568
NR1D2	672.1388	0.127353	0.048178	2.643387	0.008208	0.173751
NR3C1	648.497	0.101073	0.039677	2.547376	0.010854	0.201504
NREP	544.0699	0.220339	0.076326	2.8868	0.003892	0.14186
NSD1	1262.152	0.092042	0.035993	2.557178	0.010553	0.199179
NUMA1	2060.662	-0.08922	0.039636	-2.25087	0.024394	0.246583
OGFRL1	544.5606	0.209664	0.069128	3.032989	0.002421	0.12465
OPCML	764.7454	0.187619	0.053434	3.511214	0.000446	0.075062
PAGR1	683.4095	-0.17224	0.068446	-2.51637	0.011857	0.201925
PHRF1	701.9736	-0.11876	0.045135	-2.63123	0.008508	0.176787
PIK3R3	588.7223	0.169586	0.045286	3.744785	0.000181	0.075062
PKP4	795.8632	0.124584	0.051894	2.400732	0.016362	0.223611
PPP1R1B	369.6548	-0.24971	0.079793	-3.12952	0.001751	0.101784
PRKCB	411.8428	0.215355	0.082283	2.617246	0.008864	0.180879
PRKCE	488.4109	0.140132	0.048692	2.877914	0.004003	0.14186
PRPF8	1322.325	0.089377	0.039046	2.289033	0.022077	0.23963

PRR14L	684.099	0.086842	0.037138	2.338351	0.019369	0.233356
PRRT2	909.0313	-0.1095	0.044505	-2.4604	0.013878	0.210965
PTAR1	483.4	0.108325	0.043703	2.478663	0.013188	0.206483
PTGDS	515.0879	-0.22657	0.075656	-2.99473	0.002747	0.130106
PTPLAD1	342.703	0.131721	0.057624	2.285883	0.022261	0.23963
PTPN4	1109.762	0.114516	0.035304	3.243727	0.00118	0.092144
PUM2	998.5968	0.102879	0.036591	2.811607	0.004929	0.151649
PURA	769.2127	0.138506	0.043982	3.149124	0.001638	0.101784
PURB	832.7022	0.161471	0.057066	2.82952	0.004662	0.150804
QSER1	916.4451	0.119166	0.052781	2.257759	0.023961	0.246583
RAB11FIP4	493.521	0.118316	0.044091	2.683458	0.007287	0.171303
RAB3GAP1	338.2916	0.06782	0.028878	2.34849	0.01885	0.231397
RAB6B	415.6792	0.149085	0.055437	2.689243	0.007161	0.171303
RABEP1	949.0109	0.069289	0.030236	2.291587	0.021929	0.23963
RANBP2	1198.221	0.105621	0.036685	2.879129	0.003988	0.14186
RAPGEF2	702.5461	0.115777	0.048252	2.399424	0.016421	0.223611
RBM12	440.2849	0.113347	0.040037	2.83107	0.004639	0.150804
RC3H2	418.3657	0.106387	0.038685	2.750105	0.005958	0.158753
RMND5A	1150.859	0.116867	0.051429	2.272377	0.023064	0.23963
ROCK2	1187.448	0.108848	0.043668	2.492594	0.012681	0.205975
RPL13	467.2537	-0.14748	0.063489	-2.32289	0.020185	0.235785
RPL19	843.0513	-0.17965	0.069613	-2.58072	0.00986	0.193485
RPL31	343.86	-0.16756	0.070857	-2.36484	0.018038	0.229527
RPS3	522.6925	-0.20377	0.07816	-2.60711	0.009131	0.183021
RPS6	937.653	-0.23455	0.083806	-2.79866	0.005132	0.151649
RPS8	358.3453	-0.22612	0.079106	-2.85848	0.004257	0.146084
RUSC2	446.7147	0.087223	0.029872	2.919908	0.003501	0.139132
RYR1	623.2078	-0.19825	0.063654	-3.11452	0.001842	0.101784
S100B	677.6063	-0.19694	0.084927	-2.31898	0.020396	0.235785
SBNO1	933.5266	0.099176	0.03131	3.167533	0.001537	0.101784
SCAMP1	395.8267	0.130224	0.057166	2.277987	0.022727	0.23963
SEL1L3	793.848	0.080208	0.035659	2.249275	0.024495	0.246583
SENP6	940.4799	0.104189	0.033232	3.135225	0.001717	0.101784
SETD2	1832.993	0.091259	0.036141	2.525105	0.011566	0.201925
SETX	1047.574	0.058693	0.025821	2.273107	0.02302	0.23963
SHROOM3	530.301	0.229575	0.068614	3.34591	0.00082	0.084436
SLC25A27	760.8912	-0.09744	0.039114	-2.49106	0.012736	0.205975
SLITRK4	539.1465	0.1473	0.058226	2.529812	0.011412	0.201925
SMEK1	436.3675	0.084906	0.035508	2.391198	0.016793	0.224654
SMG5	360.6662	-0.09922	0.042808	-2.31773	0.020464	0.235785
SMG7	554.333	0.093802	0.034559	2.71427	0.006642	0.167162
SPG7	355.0169	-0.14705	0.054249	-2.7106	0.006716	0.167166
SPHKAP	2622.335	0.203159	0.072647	2.796533	0.005165	0.151649
SPTAN1	5428.313	0.081512	0.035737	2.280874	0.022556	0.23963
SPTBN5	464.4531	0.20987	0.084452	2.485064	0.012953	0.205975
SRRM3	821.6232	-0.1403	0.054396	-2.5792	0.009903	0.193485
SRSF11	1351.532	-0.14779	0.055621	-2.65712	0.007881	0.171303

SSFA2	949.2567	-0.11842	0.043214	-2.74035	0.006137	0.161641
SSH1	537.7834	0.171143	0.048349	3.539731	0.000401	0.075062
ST8SIA3	774.601	0.111409	0.040722	2.735836	0.006222	0.161992
STXBP5L	2173.03	0.145951	0.052432	2.783601	0.005376	0.153366
SUV420H1	421.8393	0.115816	0.033766	3.42991	0.000604	0.075062
SYN2	493.5214	0.132888	0.044356	2.995962	0.002736	0.130106
SYNJ1	502.748	0.10125	0.042565	2.378698	0.017374	0.227303
TAOK1	1283.309	0.086585	0.036753	2.355856	0.01848	0.231397
TAX1BP1	436.3982	0.102148	0.040434	2.526281	0.011528	0.201925
TCF25	676.9431	-0.14531	0.052434	-2.77131	0.005583	0.155569
TEX2	343.5172	0.141904	0.049231	2.882396	0.003947	0.14186
TGOLN2	1121.833	0.098423	0.033629	2.926705	0.003426	0.138559
TLN2	470.8939	0.118724	0.052152	2.276475	0.022818	0.23963
TMEM170	414.9523	0.190855	0.059355	3.21551	0.001302	0.095139
TMEM259	340.691	-0.13395	0.042916	-3.1212	0.001801	0.101784
TP53BP1	1192.164	0.130992	0.04463	2.935063	0.003335	0.137333
TSC1	914.4638	-0.08811	0.038627	-2.281	0.022548	0.23963
TSPYL2	2269.608	-0.12982	0.056555	-2.29538	0.021711	0.23963
TSPYL4	556.4496	0.189341	0.054519	3.472966	0.000515	0.075062
TTBK2	493.0953	0.099193	0.039115	2.535952	0.011214	0.201925
TTC37	710.4581	-0.11154	0.046543	-2.3964	0.016557	0.223611
TULP4	982.859	0.137486	0.059761	2.300591	0.021415	0.23963
UBE4B	623.8662	-0.08283	0.036426	-2.27397	0.022968	0.23963
UBP1	427.2674	0.107051	0.044018	2.431957	0.015018	0.216391
UBQLN1	424.1992	0.126011	0.051527	2.445522	0.014464	0.212327
UBTF	475.2477	0.157251	0.053198	2.955945	0.003117	0.135775
UGGT1	346.119	0.121801	0.035108	3.469368	0.000522	0.075062
UNC80	2717.532	-0.1075	0.041971	-2.56127	0.010429	0.198661
USP7	650.8569	0.072057	0.031974	2.253647	0.024218	0.246583
WAC	1667.732	0.124798	0.047069	2.651361	0.008017	0.171303
YWHAG	1200.134	0.162869	0.061881	2.63195	0.00849	0.176787
ZBTB18	2066.737	0.138331	0.052093	2.655463	0.00792	0.171303
ZBTB41	494.4834	0.117798	0.045157	2.608639	0.00909	0.183021
ZDHHC2	377.4813	0.128351	0.042995	2.98525	0.002833	0.130976
ZFP106	840.5572	0.0699	0.03129	2.23398	0.025484	0.24988
ZFR	818.7619	0.103109	0.041755	2.46937	0.013535	0.208552
ZHX1	369.2954	0.14583	0.044304	3.29156	0.000996	0.092144
ZIC1	2036.337	0.187753	0.06751	2.781135	0.005417	0.153366
ZIC4	563.7506	0.180125	0.050152	3.591583	0.000329	0.075062
ZMYND11	764.1487	0.115525	0.050261	2.298511	0.021533	0.23963
ZNF148	610.9009	0.128544	0.037403	3.436762	0.000589	0.075062
ZNF483	1601.114	0.096018	0.039205	2.449151	0.014319	0.212327
ZNF507	403.2055	0.103762	0.044124	2.351585	0.018694	0.231397
ZNF91	1364.366	0.114881	0.042094	2.729125	0.00635	0.163447
