# Supplementary material for: Exploring the diagnostic markers of essential tremor: A study based on machine learning algorithms
Source: Open Life Sci. 2023 Jun 22;18(1):20220622. doi: 10.1515/biol-2022-0622 (PMC10290283; doi:10.1515/biol-2022-0622)
Supplement: Supplementary Table 5 [file biol-2022-0622-sm6.pdf]

Table S5: KEGG analysis of DEGs

ONTOLOG	ID	Description	Gene Ratio	Bg Ratio	p-value	p.adjust	q-value	Gene ID	Count
BP	GO:00508	modulation	22/202	454/18866	3.88E-09	6.60E-06	5.60E-06	ADCY1/AD	22
BP	GO:00991	regulation	22/202	455/18866	4.04E-09	6.60E-06	5.60E-06	ADCY1/AD	22
BP	GO:00481	regulation	14/202	191/18866	1.91E-08	2.08E-05	1.76E-05	ADCY1/AG	14
BP	GO:00508	positive re	13/202	172/18866	4.33E-08	3.53E-05	3.00E-05	ADCY1/AP	13
BP	GO:00076	circadian r	13/202	218/18866	6.78E-07	0.000443	0.000376	ADCY1/CL	13
BP	GO:00074	axonogene	19/202	482/18866	1.13E-06	0.000613	0.00052	ADCY1/AD	19
BP	GO:00713	cellular res	15/202	330/18866	2.87E-06	0.001337	0.001135	ADCY1/AG	15
BP	GO:19907	protein loc	15/202	340/18866	4.12E-06	0.001546	0.001312	ABCA2/AP	15
BP	GO:00602	long-term	8/202	87/18866	4.26E-06	0.001546	0.001312	ADCY1/AP	8
BP	GO:00485	rhythmic p	14/202	305/18866	5.54E-06	0.001809	0.001536	ADCY1/AD	14
BP	GO:19016	cellular res	16/202	398/18866	6.40E-06	0.001899	0.001612	ADCY1/AG	16
BP	GO:19002	regulation	6/202	48/18866	1.18E-05	0.002808	0.002384	ADCY1/AP	6
BP	GO:00074	axon guida	13/202	284/18866	1.24E-05	0.002808	0.002384	BMPR2/EN	13
BP	GO:00974	neuron pr	13/202	285/18866	1.29E-05	0.002808	0.002384	BMPR2/EN	13
BP	GO:00481	Golgi vesicle	15/202	374/18866	1.29E-05	0.002808	0.002384	ARCN1/CO	15
BP	GO:00068	retrograde	7/202	87/18866	4.12E-05	0.008407	0.007137	ARCN1/CO	7
BP	GO:00001	nuclear-tr	8/202	120/18866	4.53E-05	0.008711	0.007395	RPL13/RPL	8
BP	GO:19029	regulation	14/202	373/18866	5.19E-05	0.009423	0.007999	APOE/CAM	14
BP	GO:00995	synaptic v	10/202	199/18866	5.86E-05	0.010069	0.008548	ADCY1/CP	10
BP	GO:00109	positive re	12/202	290/18866	7.16E-05	0.011697	0.00993	ADNP/AGT	12
BP	GO:00009	nuclear-tr	10/202	210/18866	9.18E-05	0.012456	0.010575	RC3H2/RP	10
BP	GO:00313	positive re	14/202	394/18866	9.31E-05	0.012456	0.010575	ADNP/AGT	14
BP	GO:00434	response t	15/202	447/18866	9.91E-05	0.012456	0.010575	ADCY1/AG	15
BP	GO:00432	regulation	15/202	449/18866	0.000104	0.012456	0.010575	APOE/ATF	15
BP	GO:00508	cognition	12/202	302/18866	0.000105	0.012456	0.010575	ADCY1/AD	12
BP	GO:00072	neurotran	9/202	175/18866	0.000115	0.012456	0.010575	ADCY1/CP	9

BP	GO:00996 signal rele 9/202	175/18866	0.000115	0.012456	0.010575	ADCY1/CP	9
BP	GO:00076 learning or 11/202	260/18866	0.000118	0.012456	0.010575	ADCY1/AD	11
BP	GO:00069 nucleocyto13/202	354/18866	0.00012	0.012456	0.010575	AGT/AHCY	13
BP	GO:20001 regulation 5/202	46/18866	0.000128	0.012456	0.010575	ATF7IP/CA	5
BP	GO:00511 nuclear tra13/202	357/18866	0.00013	0.012456	0.010575	AGT/AHCY	13
BP	GO:00190 viral transc9/202	178/18866	0.000131	0.012456	0.010575	JUN/RANB	9
BP	GO:00323 regulation 13/202	358/18866	0.000134	0.012456	0.010575	ABCA2/C2	13
BP	GO:00990 vesicle-me 10/202	220/18866	0.000135	0.012456	0.010575	ADCY1/CP	10
BP	GO:00331 regulation 11/202	264/18866	0.000135	0.012456	0.010575	C2CD5/FY	11
BP	GO:00989 dendritic t 3/202	10/18866	0.000137	0.012456	0.010575	KIF5A/KIF5	3
BP	GO:00323 positive re 10/202	222/18866	0.000145	0.012792	0.01086	C2CD5/DY	10
BP	GO:00991 regulation 7/202	107/18866	0.000154	0.013195	0.011201	APOE/DN	7
BP	GO:00328 cellular res10/202	226/18866	0.000168	0.014036	0.011916	AGT/APPL	10
BP	GO:00488 dendrite m8/202	146/18866	0.000179	0.014653	0.012439	BTBD3/DN	8
BP	GO:00705 regulation 9/202	190/18866	0.000214	0.015867	0.01347	CAMSAP2/	9
BP	GO:00064 mRNA cat 13/202	376/18866	0.000217	0.015867	0.01347	HNRNPC/P	13
BP	GO:20003 regulation 6/202	80/18866	0.000218	0.015867	0.01347	ADCY1/CP	6
BP	GO:00986 regulation 7/202	114/18866	0.000227	0.015867	0.01347	ADCY1/CP	7
BP	GO:00726 protein loc 11/202	281/18866	0.000232	0.015867	0.01347	APPL1/C2C	11
BP	GO:00709 protein loc 8/202	152/18866	0.000236	0.015867	0.01347	MIA3/RAB	8

BP	GO:00512	spindle ass	7/202	115/18866	0.00024	0.015867	0.01347	DYNC1H1/	7
BP	GO:00512	protein de	7/202	115/18866	0.00024	0.015867	0.01347	CAMSAP2/	7
BP	GO:00456	positive re	13/202	380/18866	0.00024	0.015867	0.01347	ADNP/AGT	13
BP	GO:00072	nitric oxid	4/202	29/18866	0.000246	0.015867	0.01347	AGT/APOE	4
BP	GO:00313	positive re	3/202	12/18866	0.000248	0.015867	0.01347	C2CD5/KIF	3
BP	GO:00481	regulation	5/202	53/18866	0.000253	0.015902	0.0135	AGT/APOE	5
BP	GO:00190	viral gene	9/202	195/18866	0.00026	0.016018	0.013599	JUN/RANB	9
BP	GO:00076	locomotor	9/202	198/18866	0.000291	0.01742	0.014789	ABCA2/AP	9
BP	GO:19038	positive re	12/202	338/18866	0.000298	0.01742	0.014789	C2CD5/FY	12
BP	GO:00147	striated m	13/202	389/18866	0.000301	0.01742	0.014789	AGT/CFLA	13
BP	GO:00725	neurotran	3/202	13/18866	0.00032	0.01742	0.014789	APOE/SSH	3
BP	GO:00996	axo-dendr	3/202	13/18866	0.00032	0.01742	0.014789	KIF5A/KIF5	3
BP	GO:19002	negative r	3/202	13/18866	0.00032	0.01742	0.014789	APOE/EPH	3
BP	GO:00313	regulation	4/202	31/18866	0.00032	0.01742	0.014789	C2CD5/CP	4
BP	GO:00160	synaptic v	7/202	121/18866	0.000328	0.017552	0.0149	ADCY1/CP	7
BP	GO:00163	dendrite d	10/202	247/18866	0.000342	0.017868	0.015169	APOE/BTB	10
BP	GO:00427	regulation	7/202	122/18866	0.000345	0.017868	0.015169	ADCY1/CL	7
BP	GO:00974	synaptic v	5/202	57/18866	0.000357	0.018211	0.01546	DNM3/KIF	5
BP	GO:00725	establishm	7/202	124/18866	0.00038	0.019116	0.016228	RAB3GAP1	7
BP	GO:00108	regulation	3/202	14/18866	0.000404	0.019537	0.016585	ABCA2/AG	3
BP	GO:00458	regulation	3/202	14/18866	0.000404	0.019537	0.016585	ATF7IP/CA	3
BP	GO:19020	regulation	4/202	33/18866	0.000409	0.019537	0.016585	ABCA2/AP	4
BP	GO:00324	regulation	6/202	90/18866	0.000413	0.019537	0.016585	CALM1/IN	6
BP	GO:19054	positive re	7/202	127/18866	0.00044	0.020512	0.017413	C2CD5/FY	7
BP	GO:00066	protein tar	9/202	210/18866	0.000448	0.020589	0.017479	C2CD5/FY	9
BP	GO:00605	muscle tis	13/202	409/18866	0.000484	0.021957	0.01864	AGT/CFLA	13
BP	GO:00064	RNA catab	13/202	415/18866	0.000555	0.024819	0.02107	HNRNPC/P	13
BP	GO:00991	postsynap	8/202	173/18866	0.000563	0.024852	0.021098	APOE/CNK	8
BP	GO:19016	cellular res	6/202	97/18866	0.000616	0.026818	0.022767	ADCY1/AT	6
BP	GO:00708	transcripti	4/202	37/18866	0.000639	0.027456	0.023309	ATF7IP/CA	4
BP	GO:20001	positive re	4/202	38/18866	0.000708	0.030036	0.025499	CAND1/CR	4
BP	GO:00507	positive re	14/202	485/18866	0.000764	0.031989	0.027157	ADNP/AGT	14
BP	GO:00342	amyloid-b	4/202	39/18866	0.000783	0.032056	0.027213	ABCA2/AP	4
BP	GO:00903	positive re	8/202	182/18866	0.000785	0.032056	0.027213	C2CD5/FY	8
BP	GO:00068	neurotran	9/202	229/18866	0.000833	0.033161	0.028152	ADCY1/CP	9
BP	GO:00508	regulation	9/202	229/18866	0.000833	0.033161	0.028152	ADNP/APO	9
BP	GO:19029	regulation	4/202	40/18866	0.000862	0.033285	0.028257	ABCA2/AP	4
BP	GO:00344	steroid est	3/202	18/18866	0.000876	0.033285	0.028257	ABCA2/AG	3
BP	GO:00344	sterol este	3/202	18/18866	0.000876	0.033285	0.028257	ABCA2/AG	3
BP	GO:00344	cholestero	3/202	18/18866	0.000876	0.033285	0.028257	ABCA2/AG	3
BP	GO:00329	protein-co	11/202	330/18866	0.000891	0.033454	0.0284	CAMSAP2/	11
BP	GO:00080	axo-dendr	5/202	70/18866	0.000922	0.03422	0.029051	DYNC1H1/	5
BP	GO:00066	SRP-depen	6/202	105/18866	0.000935	0.0343	0.029118	RPL13/RPL	6
BP	GO:00328	response t	10/202	283/18866	0.00098	0.034708	0.029465	AGT/APPL	10
BP	GO:00469	regulation	6/202	106/18866	0.000982	0.034708	0.029465	ADCY1/CP	6
BP	GO:19018	negative r	5/202	71/18866	0.000983	0.034708	0.029465	CAMSAP2/	5
BP	GO:00003	response t	9/202	235/18866	0.000999	0.034708	0.029465	ANKZF1/A	9

BP	GO:00015	regulation	9/202	235/18866	0.000999	0.034708	0.029465	ADCY1/CP	9
BP	GO:00515	response t	7/202	147/18866	0.001047	0.036001	0.030563	ADCY1/AH	7
BP	GO:00613	cardiac co	7/202	148/18866	0.001089	0.037062	0.031463	AGT/AHCY	7
BP	GO:00064	translation	8/202	192/18866	0.001109	0.037163	0.031549	EIF4G2/EIF	8
BP	GO:19037	regulation	5/202	73/18866	0.001115	0.037163	0.031549	AGT/AHCY	5
BP	GO:00066	cotranslati	6/202	109/18866	0.001135	0.037252	0.031625	RPL13/RPL	6
BP	GO:00328	regulation	9/202	240/18866	0.001157	0.037252	0.031625	CAMSAP2/	9
BP	GO:00508	regulation	9/202	240/18866	0.001157	0.037252	0.031625	ADNP/APO	9
BP	GO:00901	establishm	11/202	342/18866	0.001189	0.037252	0.031625	C2CD5/FY	11
BP	GO:19046	glucose tra	6/202	110/18866	0.00119	0.037252	0.031625	APPL1/C2C	6
BP	GO:00080	retrograde	3/202	20/18866	0.001205	0.037252	0.031625	DYNC1H1/	3
BP	GO:19020	positive re	3/202	20/18866	0.001205	0.037252	0.031625	ABCA2/EP	3
BP	GO:00315	protein-co	10/202	291/18866	0.001209	0.037252	0.031625	DNM3/KIF	10
BP	GO:19054	regulation	8/202	195/18866	0.001225	0.037398	0.031748	APPL1/C2C	8
BP	GO:00484	synaptic v	4/202	44/18866	0.001239	0.037462	0.031803	DNM3/KIF	4
BP	GO:00463	glucose im	5/202	75/18866	0.001259	0.037738	0.032037	APPL1/C2C	5
BP	GO:20010	regulation	3/202	21/18866	0.001395	0.041423	0.035166	DDX17/NR	3
BP	GO:00086	hexose tra	6/202	114/18866	0.001431	0.041972	0.035632	APPL1/C2C	6
BP	GO:19018	regulation	8/202	200/18866	0.001439	0.041972	0.035632	ADNP/AGT	8
BP	GO:00070	microtubu	4/202	46/18866	0.001465	0.042028	0.035679	CAMSAP2/	4
BP	GO:19029	negative r	7/202	156/18866	0.001477	0.042028	0.035679	APOE/CAM	7
BP	GO:00435	positive re	12/202	406/18866	0.00148	0.042028	0.035679	AGAP1/AR	12
BP	GO:00063	DNA-temp	9/202	249/18866	0.001493	0.042044	0.035693	ATF7IP/CA	9
BP	GO:00512	protein po	10/202	300/18866	0.001517	0.042342	0.035946	CAMSAP2/	10
BP	GO:00157	monosacc	6/202	116/18866	0.001564	0.043298	0.036757	APPL1/C2C	6
BP	GO:00108	regulation	5/202	79/18866	0.001589	0.043611	0.037023	APPL1/C2C	5
BP	GO:00311	microtubu	6/202	117/18866	0.001634	0.044485	0.037765	CAMSAP2/	6
BP	GO:00342	carbohydr	6/202	118/18866	0.001707	0.046074	0.039114	APPL1/C2C	6
BP	GO:00713	cellular res	8/202	206/18866	0.001734	0.046431	0.039417	ATP2B1/C	8
BP	GO:00432	negative r	5/202	81/18866	0.001776	0.047019	0.039917	CAMSAP2/	5
BP	GO:00709	neuron de	11/202	360/18866	0.001785	0.047019	0.039917	ADNP/APO	11
BP	GO:00106	negative r	12/202	416/18866	0.001814	0.047073	0.039962	CAMSAP2/	12
BP	GO:19002	positive re	3/202	23/18866	0.001829	0.047073	0.039962	ADCY1/CR	3
BP	GO:19029	positive re	8/202	208/18866	0.001843	0.047073	0.039962	APOE/CD4	8
BP	GO:00450	protein tar	6/202	120/18866	0.001859	0.047073	0.039962	RPL13/RPL	6
BP	GO:00515	regulation	6/202	120/18866	0.001859	0.047073	0.039962	ADCY1/CP	6
BP	GO:00353	regulation	8/202	209/18866	0.001899	0.047706	0.040499	CALM1/CA	8
BP	GO:00076	memory	6/202	121/18866	0.001939	0.048346	0.041043	ADCY1/AD	6
BP	GO:00712	cellular res	5/202	83/18866	0.001978	0.048938	0.041545	ADCY1/EE	5
BP	GO:00429	amyloid pr	4/202	50/18866	0.001999	0.049096	0.04168	ABCA2/AP	4
BP	GO:00432	regulation	6/202	122/18866	0.002022	0.049148	0.041723	CAMSAP2/	6
BP	GO:00901	regulation	3/202	24/18866	0.002074	0.049148	0.041723	DYNC1H1/	3
BP	GO:19029	positive re	3/202	24/18866	0.002074	0.049148	0.041723	ABCA2/EP	3
BP	GO:19012	negative r	8/202	212/18866	0.002075	0.049148	0.041723	ADNP/APO	8
BP	GO:00970	dendritic s	5/202	84/18866	0.002085	0.049148	0.041723	APOE/DN	5
BP	GO:00430	regulation	13/202	481/18866	0.002102	0.049148	0.041723	AGAP1/AR	13
BP	GO:19043	regulation	6/202	123/18866	0.002107	0.049148	0.041723	ABCA2/AP	6

BP	GO:00504	amyloid-b	4/202	51/18866	0.002152	0.049836	0.042308	ABCA2/AP	4
CC	GO:00140	postsynap	17/209	337/19559	1.39E-07	3.62E-05	2.93E-05	ADCY1/BA	17
CC	GO:00322	asymmetri	17/209	343/19559	1.79E-07	3.62E-05	2.93E-05	ADCY1/BA	17
CC	GO:00995	postsynap	17/209	361/19559	3.68E-07	4.88E-05	3.94E-05	ADCY1/BA	17
CC	GO:00989	neuron to	17/209	368/19559	4.82E-07	4.88E-05	3.94E-05	ADCY1/BA	17
CC	GO:00059	cell cortex	13/209	305/19559	2.60E-05	0.002104	0.001701	C2CD5/CA	13
CC	GO:00989	glutamate	14/209	361/19559	3.60E-05	0.002428	0.001963	ADCY1/AP	14
CC	GO:00308	cortical cy	7/209	106/19559	0.000143	0.008295	0.006705	CALD1/CA	7
CC	GO:00058	kinesin co	5/209	51/19559	0.000209	0.009706	0.007846	KIF2A/KIF5	5
CC	GO:00308	cortical ac	6/209	80/19559	0.000216	0.009706	0.007846	CALD1/CA	6
CC	GO:01201	neuron pr	6/209	87/19559	0.000341	0.0138	0.011155	DYNC1H1/	6
CC	GO:00328	dendrite c	4/209	34/19559	0.000457	0.016824	0.013599	KIF5A/KIF5	4
CC	GO:01500	distal axon	11/209	309/19559	0.000513	0.017305	0.013988	CPLX3/EPH	11
CC	GO:00017	ruffle	8/209	179/19559	0.000697	0.021726	0.017561	APPL1/C2C	8
CC	GO:00058	microtubu	13/209	431/19559	0.000778	0.022496	0.018184	CALM1/CA	13
CC	GO:00226	cytosolic ri	6/209	110/19559	0.00118	0.031867	0.025759	RPL13/RPL	6
CC	GO:00056	telomeras	3/209	21/19559	0.001388	0.035128	0.028394	HNRNPC/S	3
CC	GO:00058	microtubu	7/209	159/19559	0.001632	0.038083	0.030783	DYNC1H1/	7
CC	GO:00996	postsynap	6/209	118/19559	0.001693	0.038083	0.030783	ADCY1/CN	6
CC	GO:00312	cell leadin	12/209	421/19559	0.001979	0.042186	0.0341	APPL1/ATP	12
CC	GO:00059	adherens j	7/209	166/19559	0.002086	0.042232	0.034137	BMPR2/EI	7
MF	GO:00481	tau protei	6/207	45/18352	1.08E-05	0.005287	0.004711	APOE/FYN	6
MF	GO:00156	tubulin bin	14/207	365/18352	7.19E-05	0.015986	0.014246	ADNP/APP	14
MF	GO:00503	tau-protei	4/207	22/18352	9.81E-05	0.015986	0.014246	FYN/ROCK	4
MF	GO:00037	microtubu	6/207	77/18352	0.000233	0.028522	0.025419	DYNC1H1/	6
