# Supplementary material for: Exploring the diagnostic markers of essential tremor: A study based on machine learning algorithms
Source: Open Life Sci. 2023 Jun 22;18(1):20220622. doi: 10.1515/biol-2022-0622 (PMC10290283; doi:10.1515/biol-2022-0622)
Supplement: Supplementary Table 6 [file biol-2022-0622-sm7.pdf]

Table S6: KEGG signaling pathway analysis.

ID	Description	GeneRatio	BgRatio	P-value	p.adjust	q-value	geneID	Count
hsa04270	Vascular s	10/98	133/8102	4.16E-06	0.000899	0.000587	107/183/2	10
hsa05132	Salmonella	12/98	249/8102	4.23E-05	0.004574	0.002987	1778/1064	12
hsa04360	Axon guid	10/98	182/8102	6.47E-05	0.004656	0.003041	23365/659	10
hsa04921	Oxytocin s	9/98	154/8102	9.53E-05	0.004781	0.003122	107/801/2	9
hsa04611	Platelet ac	8/98	124/8102	0.000119	0.004781	0.003122	107/23365	8
hsa04713	Circadian e	7/98	97/8102	0.000161	0.004781	0.003122	107/801/1	7
hsa04925	Aldosteron	7/98	98/8102	0.000172	0.004781	0.003122	107/183/4	7
hsa04022	cGMP-PKG	9/98	167/8102	0.000177	0.004781	0.003122	107/490/8	9
hsa05171	Coronaviru	10/98	232/8102	0.00047	0.011289	0.007372	3725/5594	10
hsa04015	Rap1 signa	9/98	210/8102	0.000951	0.01923	0.012558	107/801/9	9
hsa04728	Dopamine	7/98	132/8102	0.00105	0.01923	0.012558	801/9575/	7
hsa04024	cAMP sign	9/98	216/8102	0.00116	0.01923	0.012558	107/490/8	9
hsa04933	AGE-RAGE	6/98	100/8102	0.001281	0.01923	0.012558	183/3725/	6
hsa04371	Apelin sign	7/98	137/8102	0.001305	0.01923	0.012558	107/801/5	7
hsa04916	Melanoge	6/98	101/8102	0.001349	0.01923	0.012558	107/801/1	6
hsa05031	Amphetam	5/98	69/8102	0.001424	0.01923	0.012558	801/1385/	5
hsa05163	Human cyt	9/98	225/8102	0.001542	0.019593	0.012795	107/23365	9
hsa05223	Non-small	5/98	72/8102	0.001724	0.020683	0.013506	3798/3799	5
hsa04931	Insulin res	6/98	108/8102	0.001904	0.021642	0.014133	183/1385/	6
hsa04072	Phospholip	7/98	148/8102	0.002036	0.021984	0.014356	107/183/2	7
hsa04725	Cholinergi	6/98	113/8102	0.002395	0.024635	0.016087	107/1385/	6
hsa05130	Pathogeni	8/98	197/8102	0.00257	0.024834	0.016217	23365/253	8
hsa04150	mTOR sign	7/98	155/8102	0.002644	0.024834	0.016217	96459/797	7
hsa04510	Focal adhe	8/98	201/8102	0.00291	0.026193	0.017104	824/2534/	8
hsa04071	Sphingolip	6/98	119/8102	0.003104	0.026821	0.017515	2534/5594	6
hsa04211	Longevity	5/98	89/8102	0.004353	0.036159	0.023613	107/26060	5
hsa04912	GnRH sign	5/98	93/8102	0.005248	0.039244	0.025627	107/801/3	5
hsa04970	Salivary se	5/98	93/8102	0.005248	0.039244	0.025627	107/490/8	5
hsa05020	Prion disea	9/98	273/8102	0.005622	0.039244	0.025627	1385/2534	9
hsa04730	Long-term	4/98	60/8102	0.005849	0.039244	0.025627	2977/5594	4
hsa04210	Apoptosis	6/98	136/8102	0.005968	0.039244	0.025627	824/8837/	6
hsa04910	Insulin sig	6/98	137/8102	0.006182	0.039244	0.025627	801/2194/	6
hsa04070	Phosphati	5/98	97/8102	0.006266	0.039244	0.025627	801/8760/	5
hsa04915	Estrogen s	6/98	138/8102	0.006401	0.039244	0.025627	107/801/1	6
hsa04750	Inflammat	5/98	98/8102	0.006541	0.039244	0.025627	107/801/8	5
hsa05231	Choline m	5/98	98/8102	0.006541	0.039244	0.025627	3725/5594	5
hsa05142	Chagas dis	5/98	102/8102	0.007725	0.045098	0.02945	107/8837/	5
hsa04625	C-type lect	5/98	104/8102	0.00837	0.047577	0.031069	23365/801	5
hsa04720	Long-term	4/98	67/8102	0.008622	0.047755	0.031185	107/801/5	4
hsa04928	Parathyroi	5/98	106/8102	0.009051	0.048878	0.031918	107/1385/	5
hsa04261	Adrenergic	6/98	150/8102	0.009491	0.049109	0.032069	107/183/4	6
hsa04924	Renin secr	4/98	69/8102	0.009549	0.049109	0.032069	183/801/1	4
hsa04960	Aldosteron	3/98	37/8102	0.009901	0.049737	0.03248	5594/8503	3
