# Supplementary material for: Exploring the diagnostic markers of essential tremor: A study based on machine learning algorithms
Source: Open Life Sci. 2023 Jun 22;18(1):20220622. doi: 10.1515/biol-2022-0622 (PMC10290283; doi:10.1515/biol-2022-0622)
Supplement: Supplementary Table 7 [file biol-2022-0622-sm8.pdf]

Table S7: top 3 most prominent pathways identified through KEGG analysis.

ID	Description	Set Size	enrichment	NES	p-value	p.adjust	q-values	rank	leading_ed
hsa03010	Ribosome	128	0.59225	2.694751	1.00E-10	1.09E-08	5.61E-09	5173	tags=67%,
hsa05168	Herpes sim	480	-0.38377	-2.16456	1.00E-10	1.09E-08	5.61E-09	3017	tags=29%,
hsa05171	Coronaviru	211	0.487526	2.384142	1.00E-10	1.09E-08	5.61E-09	5207	tags=53%,
hsa00480	Glutathion	51	0.679541	2.634758	1.85E-10	1.23E-08	6.35E-09	3700	tags=61%,
hsa00280	Valine, leu	47	0.691117	2.634697	1.88E-10	1.23E-08	6.35E-09	2216	tags=49%,
hsa01212	Fatty acid	52	0.648177	2.523166	4.86E-10	2.65E-08	1.36E-08	4037	tags=58%,
hsa00190	Oxidative	103	0.532483	2.338967	1.38E-09	6.46E-08	3.32E-08	6218	tags=69%,
hsa01200	Carbon me	110	0.524136	2.317748	2.39E-09	9.75E-08	5.02E-08	5200	tags=54%,
hsa05016	Huntingto	275	0.390226	1.967957	8.54E-09	3.10E-07	1.60E-07	3826	tags=35%,
hsa03320	PPAR signa	73	0.571751	2.342069	1.44E-08	4.71E-07	2.43E-07	4559	tags=52%,
hsa05415	Diabetic ca	173	0.429572	2.039317	2.86E-08	8.35E-07	4.30E-07	4254	tags=44%,
hsa05012	Parkinson	217	0.405424	1.991651	3.06E-08	8.35E-07	4.30E-07	5875	tags=50%,
hsa05014	Amyotrop	328	0.359248	1.840604	3.93E-08	9.88E-07	5.09E-07	3759	tags=33%,
hsa05321	Inflammat	60	0.581783	2.318451	8.69E-08	2.03E-06	1.05E-06	2873	tags=42%,
hsa04145	Phagosom	146	0.447731	2.073991	1.50E-07	3.00E-06	1.54E-06	4932	tags=46%,
hsa04151	PI3K-Akt si	337	0.34366	1.763573	1.52E-07	3.00E-06	1.54E-06	4164	tags=30%,
hsa00071	Fatty acid	41	0.644171	2.382649	1.56E-07	3.00E-06	1.54E-06	4168	tags=61%,
hsa05022	Pathways	439	0.326636	1.707507	2.15E-07	3.79E-06	1.95E-06	5900	tags=40%,
hsa00640	Propanoat	33	0.676944	2.409857	2.20E-07	3.79E-06	1.95E-06	2175	tags=52%,
hsa05020	Prion disea	244	0.369686	1.845991	5.36E-07	8.77E-06	4.52E-06	5875	tags=46%,
hsa05150	Staphyloco	72	0.526009	2.157226	1.00E-06	1.56E-05	8.05E-06	4676	tags=49%,
hsa05416	Viral myoc	57	0.552923	2.17946	1.33E-06	1.98E-05	1.02E-05	2981	tags=47%,
hsa04932	Non-alcoh	143	0.428564	1.976666	2.08E-06	2.95E-05	1.52E-05	5824	tags=54%,
hsa05332	Graft-vers	33	0.642338	2.286665	2.73E-06	3.68E-05	1.90E-05	3760	tags=58%,
hsa00410	beta-Alani	29	0.67234	2.299064	2.81E-06	3.68E-05	1.90E-05	4892	tags=66%,
hsa04146	Peroxisom	81	0.50701	2.129168	4.37E-06	5.49E-05	2.83E-05	3428	tags=40%,
hsa00330	Arginine a	47	0.576071	2.196115	5.01E-06	6.06E-05	3.12E-05	3439	tags=43%,
hsa04514	Cell adhesi	136	0.417836	1.916665	5.33E-06	6.06E-05	3.12E-05	5081	tags=43%,
hsa04380	Osteoclast	125	0.435594	1.978288	5.37E-06	6.06E-05	3.12E-05	3698	tags=39%,
hsa01230	Biosynthes	71	0.510808	2.086569	6.26E-06	6.83E-05	3.52E-05	4331	tags=42%,
hsa04612	Antigen pr	62	0.516352	2.06425	1.43E-05	0.000151	7.76E-05	4828	tags=48%,
hsa04142	Lysosome	123	0.41963	1.901023	2.29E-05	0.000234	0.00012	5484	tags=46%,
hsa04060	Cytokine-c	248	0.340809	1.70054	2.46E-05	0.000244	0.000126	4929	tags=33%,
hsa00380	Tryptopha	36	0.604506	2.177651	2.72E-05	0.000262	0.000135	2126	tags=31%,
hsa04723	Retrograd	140	0.402459	1.853562	2.97E-05	0.000277	0.000143	3883	tags=36%,
hsa00982	Drug meta	43	0.564967	2.095162	3.14E-05	0.000285	0.000147	4936	tags=49%,
hsa05322	Systemic l	46	0.544433	2.061773	3.54E-05	0.000313	0.000161	2270	tags=41%,
hsa04940	Type I diab	39	0.567346	2.072827	3.76E-05	0.000324	0.000167	3789	tags=51%,
hsa00260	Glycine, se	38	0.585508	2.129047	4.02E-05	0.000337	0.000174	2169	tags=32%,
hsa05163	Human cyt	215	0.346215	1.694056	4.48E-05	0.000366	0.000189	4014	tags=31%,
hsa04933	AGE-RAGE	98	0.433032	1.89147	7.94E-05	0.000633	0.000326	2756	tags=30%,
hsa05132	Salmonella	244	0.331178	1.653707	8.87E-05	0.000691	0.000356	4573	tags=33%,

hsa04510	Focal adhe	196	0.347115	1.681305	0.000102	0.000768	0.000396	3213	tags=27%,
hsa04714	Thermoge	201	0.348084	1.693481	0.000103	0.000768	0.000396	4120	tags=34%,
hsa04640	Hematopo	84	0.450877	1.911395	0.000128	0.000927	0.000478	2873	tags=33%,
hsa05166	Human T-c	213	0.337968	1.655799	0.000141	0.001	0.000515	3827	tags=30%,

hsa04672	Intestinal i	42	0.546057	2.029784	0.000161	0.001111	0.000572	2873	tags=38%<br>,
hsa05130	Pathogeni	192	0.3538<br>9	1.71163	0.000163	0.001111	0.000572	4448	tags=32%<br>,
hsa05412	Arrhythmo	77	0.453218	1.887758	0.000168	0.001111	0.000572	2854	tags=32%<br>,
hsa05205	Proteoglyc	200	0.346841	1.689641	0.000174	0.001111	0.000572	4825	tags=32%<br>,
hsa04727	Aergic syn	88	0.428242	1.828286	0.000174	0.001111	0.000572	2554	tags=33%<br>,
hsa04659	Th17 cell d	102	0.406664	1.771587	0.000177	0.001111	0.000572	2873	tags=30%<br>,
hsa05167	Kaposi sar	186	0.350538	1.681077	0.000191	0.001125	0.000579	3857	tags=31%<br>,
hsa00010	Glycolysis	62	0.473366	1.892402	0.000191	0.001125	0.000579	2126	tags=29%<br>,
hsa04015	Rap1 signa	202	0.341101	1.660759	0.000192	0.001125	0.000579	4103	tags=31%<br>,
hsa04918	Thyroid ho	72	0.450578	1.847878	0.000196	0.001125	0.000579	3827	tags=33%<br>,
hsa05202	Transcripti	152	0.365892	1.705487	0.000199	0.001125	0.000579	3274	tags=28%<br>,
hsa00860	Porphyrin	23	0.650659	2.11129	0.0002	0.00112<br>5	0.000579	4059	tags=43%<br>,
hsa04066	HIF-1 signa	105	0.413463	1.816311	0.000208	0.0011<br>5	0.000592	2669	tags=30%<br>,
hsa05204	Chemical c	52	0.495255	1.927886	0.000219	0.001193	0.000615	4936	tags=42%<br>,
hsa00650	Butanoate	25	0.618659	2.033294	0.000227	0.001219	0.000628	1827	tags=40%<br>,
hsa05140	Leishmani	74	0.450001	1.851666	0.000252	0.001327	0.000683	3586	tags=39%<br>,
hsa05330	Allograft r	34	0.571499	2.048377	0.000325	0.001666	0.000858	3760	tags=50%<br>,
hsa05410	Hypertrop	89	0.417624	1.789576	0.000328	0.001666	0.000858	5146	tags=45%<br>,
hsa04014	Ras signali	219	0.326249	1.606768	0.000331	0.001666	0.000858	3998	tags=31%<br>,
hsa01040	Biosynthes	23	0.638598	2.072155	0.000337	0.001668	0.000859	1266	tags=39%<br>,
hsa05323	Rheumato	80	0.434598	1.817758	0.000355	0.001733	0.000893	5879	tags=48%<br>,
hsa04260	Cardiac m	81	0.444729	1.867619	0.000362	0.001742	0.000897	5146	tags=47%<br>,
hsa01521	EGFR tyros	79	0.426093	1.77651	0.000406	0.001924	0.000991	3481	tags=32%<br>,
hsa04120	Ubiquitin	135	-0.35479	-1.72692	0.000439	0.002049	0.001055	4605	tags=38%<br>,
hsa00270	Cysteine a	47	0.506351	1.930327	0.000469	0.002145	0.001105	4296	tags=45%<br>,
hsa01210	2-Oxocarb	18	0.660421	1.979903	0.000472	0.002145	0.001105	4212	tags=50%<br>,
hsa00980	Metabolis	49	0.495695	1.898529	0.000516	0.002311	0.00119	4936	tags=43%<br>,
hsa04216	Ferroptosi	40	0.5289	1.938497	0.000541	0.002391	0.001232	4787	tags=55%

			5						,
hsa04810	Regulation	210	0.330338	1.615558	0.000593	0.002567	0.001322	4103	tags=30%<br>,
hsa05169	Epstein-Ba	197	0.339989	1.645983	0.000597	0.002567	0.001322	3760	tags=29%<br>,
hsa05010	Alzheimer	336	0.289156	1.483335	0.000682	0.002898	0.001492	4228	tags=29%<br>,
hsa00830	Retinol me	45	0.509109	1.912533	0.000729	0.003055	0.001574	5777	tags=58%<br>,
hsa00020	Citrate cyc	29	0.569202	1.946383	0.000806	0.0032<br>6	0.001679	6002	tags=69%<br>,
hsa04978	Mineral ab	56	0.472956	1.856456	0.000811	0.0032<br>6	0.001679	2777	tags=30%<br>,
hsa04512	ECM-recep	87	0.405994	1.724155	0.000823	0.0032<br>6	0.001679	3770	tags=34%<br>,
hsa05170	Human im	201	0.324087	1.576736	0.000825	0.0032<br>6	0.001679	3852	tags=29%<br>,
hsa04650	Natural kil	116	0.382598	1.715093	0.000827	0.0032<br>6	0.001679	3902	tags=35%<br>,
hsa04613	Neutrophi	103	0.387083	1.700289	0.000841	0.003274	0.001686	3759	tags=39%<br>,
hsa00340	Histidine m	19	0.633001	1.91739	0.000854	0.003284	0.001692	914	tags=37%<br>,
hsa05225	Hepatocell	163	0.341736	1.613294	0.001164	0.004425	0.002279	3444	tags=24%<br>,
hsa04520	Adherens j	66	0.438036	1.775948	0.001226	0.004589	0.002364	2981	tags=33%<br>,
hsa00590	Arachidon	56	0.464409	1.822908	0.001235	0.004589	0.002364	4738	tags=45%<br>,
hsa04610	Compleme	73	0.421217	1.725436	0.001313	0.004824	0.002485	5665	tags=49%<br>,
hsa05165	Human pa	327	0.285702	1.462008	0.001396	0.005072	0.002612	3827	tags=25%<br>,
hsa05164	Influenza A	162	0.337073	1.587476	0.001418	0.005094	0.002624	3887	tags=29%<br>,
hsa04662	B cell rece	80	0.411674	1.721878	0.001525	0.005373	0.002767	3698	tags=38%<br>,
hsa04666	Fc gamma	92	0.395145	1.705333	0.001528	0.005373	0.002767	4932	tags=45%<br>,

hsa04926	Relaxin sig	127	0.354263	1.607219	0.001666	0.005795	0.002985	3827	tags=31%,
hsa05320	Autoimmu	47	0.478538	1.824295	0.001802	0.006202	0.003195	6517	tags=57%,
hsa04061	Viral prote	77	0.410983	1.71184	0.001837	0.006256	0.003222	4929	tags=38%,
hsa05032	Morphine	88	0.390914	1.668921	0.001912	0.006445	0.00332	2554	tags=28%,
hsa05218	Melanoma	70	0.411347	1.683504	0.002079	0.006939	0.003574	3594	tags=31%,
hsa04979	Cholestero	46	0.469348	1.777427	0.002141	0.007071	0.003642	4562	tags=39%,
hsa04115	p53 signali	69	0.418173	1.70586	0.002181	0.007133	0.003674	3823	tags=33%,
hsa00310	Lysine deg	48	0.450028	1.720297	0.002241	0.007254	0.003736	2245	tags=29%,
hsa00062	Fatty acid	23	0.592416	1.922299	0.002275	0.007292	0.003756	709	tags=26%,
hsa05414	Dilated car	94	0.387751	1.677217	0.002315	0.007348	0.003785	2854	tags=30%,
hsa05145	Toxoplasm	110	0.359793	1.591016	0.002517	0.007914	0.004076	2873	tags=27%,
hsa04152	AMPK sign	117	0.359172	1.608344	0.002617	0.008151	0.004198	2515	tags=25%,
hsa04217	Necroptos	130	0.351519	1.600545	0.002672	0.008243	0.004245	6493	tags=49%,
hsa05144	Malaria	48	0.440347	1.683293	0.003087	0.009433	0.004859	4164	tags=35%,
hsa05131	Shigellosis	211	0.309324	1.512683	0.003124	0.009458	0.004871	3887	tags=27%,
hsa04928	Parathyroi	102	0.363206	1.58227	0.003353	0.010058	0.00518	3965	tags=33%,
hsa04621	NOD-like r	163	0.32724	1.54486	0.003663	0.010874	0.005601	3887	tags=28%,
hsa05213	Endometri	58	0.411136	1.630129	0.003691	0.010874	0.005601	1913	tags=22%,
hsa00061	Fatty acid	17	0.624425	1.841091	0.003919	0.011441	0.005893	3936	tags=53%,
hsa00053	Ascorbate	12	0.68942	1.823452	0.004103	0.011874	0.006116	4032	tags=58%,
hsa04670	Leukocyte	107	0.365986	1.608787	0.00418	0.011991	0.006176	4493	tags=34%,
hsa00620	Pyruvate m	44	0.469804	1.755483	0.004295	0.012214	0.006291	4605	tags=45%,
hsa05310	Asthma	25	0.534564	1.756907	0.004446	0.012532	0.006455	2873	tags=40%,
hsa00983	Drug meta	56	0.428142	1.68055	0.005785	0.016107	0.008296	4647	tags=36%,
hsa05226	Gastric can	146	0.333724	1.545885	0.005812	0.016107	0.008296	3412	tags=23%,
hsa05214	Glioma	74	0.390118	1.605259	0.006203	0.017046	0.00878	3412	tags=30%,
hsa05224	Breast can	144	0.313274	1.444439	0.006967	0.018986	0.009779	2178	tags=17%,
hsa05216	Thyroid ca	37	0.486543	1.763422	0.007353	0.01987	0.010234	3412	tags=32%,
hsa05133	Pertussis	72	0.383266	1.571823	0.007664	0.020543	0.010581	5207	tags=42%,
hsa00120	Primary bi	16	0.594328	1.710852	0.00798	0.021216	0.010927	2934	tags=38%,
hsa05215	Prostate c	95	0.353922	1.528873	0.008264	0.021793	0.011224	2093	tags=21%,
hsa05203	Viral carcin	163	0.312344	1.474538	0.009218	0.024113	0.012419	3827	tags=28%,
hsa00630	Glyoxylate	27	0.510815	1.705598	0.009524	0.024702	0.012723	4482	tags=52%,
hsa00030	Pentose p	28	0.509946	1.726436	0.009659	0.024702	0.012723	4331	tags=46%,
hsa05212	Pancreatic	76	0.372213	1.544868	0.009669	0.024702	0.012723	1894	tags=21%,
hsa04068	FoxO signa	127	0.330735	1.50048	0.010183	0.025812	0.013295	2245	tags=22%,
hsa00051	Fructose a	27	0.505294	1.687163	0.01101	0.027695	0.014264	1805	tags=37%,
hsa01524	Platinum d	68	0.374759	1.529511	0.011513	0.028738	0.014801	2045	tags=21%,
hsa01523	Antifolate	29	-0.4603	-1.66546	0.012463	0.030873	0.015901	5291	tags=52%,
hsa01240	Biosynthes	123	0.331869	1.503443	0.012963	0.031705	0.01633	3499	tags=26%,
hsa05033	Nicotine a	39	0.43137	1.576033	0.013039	0.031705	0.01633	1192	tags=21%,
hsa05152	Tuberculo	175	0.300084	1.429055	0.013105	0.031705	0.01633	2270	tags=21%,
hsa05223	Non-small	72	0.370802	1.520706	0.013186	0.031705	0.01633	3412	tags=31%,
hsa04540	Gap juncti	86	0.350597	1.489501	0.013789	0.032762	0.016874	3964	tags=31%,
hsa04390	Hippo sign	153	0.311044	1.452698	0.013826	0.032762	0.016874	2669	tags=18%,
hsa05220	Chronic m	76	0.364354	1.512251	0.014056	0.033068	0.017032	2019	tags=24%,

hsa04630	JAK-STAT s	148	0.304627	1.41242	0.014367	0.03332	0.017161	4549	tags=32%,
hsa04919	Thyroid ho	121	0.322347	1.453922	0.014937	0.034397	0.017716	3412	tags=26%,
hsa05110	Vibrio chol	48	0.399041	1.525394	0.015902	0.036364	0.018729	5879	tags=54%,
hsa04218	Cellular se	151	0.310819	1.447502	0.016689	0.037898	0.019519	3773	tags=26%,
hsa04210	Apoptosis	130	0.315875	1.438248	0.018375	0.041439	0.021343	2977	tags=22%,
hsa05219	Bladder ca	39	0.418901	1.530476	0.019359	0.04336	0.022332	1913	tags=23%,
hsa05418	Fluid shea	131	0.325149	1.480611	0.019685	0.043789	0.022554	4073	tags=30%,
hsa04920	Adipocyto	69	0.3669	1.4967	0.02093	0.046244	0.023818	5207	tags=41%,
hsa05230	Central ca	68	0.356613	1.45545	0.02118	0.046482	0.023941	3419	tags=28%,
hsa03008	Ribosome	72	-0.34099	-1.5053	0.0214	0.046651	0.024028	3902	tags=33%,
hsa00230	Purine me	116	0.31853	1.427893	0.021772	0.047149	0.024284	3992	tags=28%,
hsa04964	Proximal t	23	0.525925	1.706546	0.014217	0.033206	0.017103	343	tags=22%,

core_enrichment
6232/6158/6187/6175/6150/6181/79590/6136/6159/7311/6135/6166/6128/6157/6137/6134/6138/6147/54790/8891/148254/
7186/169841/6352/57474/10793/3551/55762/1616/7625/284370/79088/148268/7565/5578/5648/1956/7132/716/6232/6158
/6187/6175/3553/715/2212/7099/6181/10379/713/6136/712/718/33418/4257/373156/2950/9588/2876/79017/2949/51056/2
946/2878/2947/2937/51060/2879/493869/6240/4329/3032/3030/217/223/18/10449/1962/501/1892/64087/3712/11112/594/
3157/1629/586/35/38/224/53032/79966/3030/54898/9415/23205/1374/10449/1962/6319/51/9524/1892/3295/3992/2194/63
42/35/384700/6391/479/4701/4720/4726/1339/1327/374291/54539/4695/7381/7385/10975/1355/1350/8992/472/4329/8801
/3418/26227/2023/230/80201/2746/50/6391/51/29968/226/83440/1892/84706/9104/847/4967/2890/347733/6507/6506/101
26/2915/4700/2876/7802/6391/9776/4701/148327/2878/84617/5719/4720/79966/5360/9415/1593/1622/23205/1374/1962/6
319/123/51/5346/3157/6342/5465/4023/51129/116519/5578/4088/1509/5166/7042/4700/6391/4701/6517/4720/4726/7040/
2597/5465/4689/1536/1339/489/132/347733/4128/9246/2771/4700/6391/4701/25828/120892/84617/5719/4129/4720/7311/
4726/5693/5685/2890/347733/6506/71/10126/400916/4700/7132/2876/596/7802/6391/9776/4701/5608/11337/2878/71340
88/7042/4772/7100/3123/3553/3459/7099/3119/3113/3566/7040/3127/3122/8809/3108/64127/6778/6347733/71/81035/706
0/3133/3123/715/2212/7099/3119/60/84617/3690/3920/3113/718/821/3105/3127/2247/5578/2064/2263/4915/3696/2261/36
91/56034/5521/284/7060/55970/2246/1956/1975/596/3908/103032/3030/217/223/23205/1374/10449/1962/501/51/1892/10
455/35/38/33/2180/224/36/124/2181/2330/2890/347733/5578/9246/10126/2915/4700/7132/2876/596/1460/7802/6391/9776
/4701/3553/5608/11334329/8801/3030/18/79611/1962/51/1892/3945/84532/594/1629/8802/35/38/5096/84693/347733/468
5/2534/4700/1460/6391/4701/3553/148327/84617/5719/713/4720/116444/712/64764/4726/5648/1672/716/3123/715/2212/
3119/713/2358/712/3113/718/3127/3122/2214/3108/714/3689/3109/640/71/2534/3133/3908/3123/3119/60/4625/6442/3113
/3105/3127/3122/1605/3108/3107/1756/3689/3106/34700/7132/6391/4701/3553/6720/5565/51094/4720/840/4726/7040/54
65/3953/1339/1327/374291/7960/3133/3123/3553/3119/3113/3105/3127/3122/3108/3107/3106/3109/3821/940/942/3115/3
111/3134/355/4329/3030/217/223/18/55748/1962/501/51/1892/35/224/1806/219/2572/339896/57571/218/2571/3418/1610/
373156/51268/1962/1384/51/5192/1891/3295/10455/847/92960/5052/26063/6342/283927/8217/5625/1610/223/4128/5574
8/6303/501/29920/8659/51056/1152/4129/224/4953/6723/112483/6611/56385/4685/3696/9672/6402/4897/5010/3133/3123
/54413/50848/6382/3119/3113/9076/3105/4756/3127/7042/2534/7132/4772/11026/1436/3553/5608/4982/3459/2212/10288
/10379/3690/4773/11006/6688/283418/26227/2023/2752/230/29920/50/875/29968/226/84706/113675/2597/7167/586/5315
/4548/7086/501508/3133/3123/972/3119/5721/5720/3113/821/8625/3105/3127/3122/3108/3107/5641/3106/3109/567/1519
9516/2760/2799/9741/1509/10577/1200/1508/7805/967/2629/285362/6556/1075/8722/3920/162/3658/3977/90865/2662/35
56/7042/3590/53832/7132/3625/9173/53342/3597/1436/9180/3553/4982/3459/3030/217/223/4128/1962/501/1892/4129/84
7/38/224/2890/5578/108/2565/2555/2564/55970/2915/2771/4700/4701/2557/2560/51764/4720/2783/4726/2567/54257/412
8/373156/2950/2949/2946/4129/2947/1558/2941/1559/4259/2948/124/1576/316/128/119391/2716/3123/715/2212/3119/71
3/712/3113/718/3127/3122/2214/3108/714/3109/940/942/2209/3115/3133/3123/3553/3119/1363/3113/3105/3127/3122/310
8/3107/3106/3109/940/942/3115/3111/3134/355/26227/1610/4128/51268/501/875/29968/4129/113675/2653/63826/2731/5
578/108/1871/11214/55970/2771/1956/7132/4772/3133/1026/3553/5608/148327/3690/4773/51764/285578/4088/113026/5
333/2152/7042/4772/596/3553/7423/2308/5292/7040/1286/5595/1536/6774/51196/347733/388/26084/71/6934/7132/596/7
100/10006/3553/5608/25828/7099/60/84617/5286/840/3071/964/5578/2064/71/3696/3691/56034/7060/7410/2534/1956/59
6/399694/3908/3910/3679/60/29780/3690/742108/71/1374/4700/6391/4701/5346/148327/5565/60/4720/5592/63976/2885/
64764/4726/4881/1339/132/3590/928/3123/1436/3553/960/3119/3690/3113/966/3554/3566/3127/3122/3108/930/4254/952/
1435/31108/4088/1871/4488/7042/7132/4772/3133/7538/3123/1026/148327/3119/9700/4773/2224/6688/1031/3

3123/3119/3113/7040/3127/3122/3108/4055/3109/23308/940/942/3115/8741/3601/3111
347733/7009/71/10972/2534/7132/5010/7456/7100/10163/10006/3553/5781/3059/4651/2212/7099/60/8
71/6934/3696/3691/1495/3908/2697/3679/60/3690/6442/1605/489/10369/1824/1756/3685/29119/27091
2247/6385/5578/2064/71/4478/7410/7042/1956/1975/10855/1026/967/5781/3059/960/6382/7099/60/36
6538/5578/108/2565/2555/18/2752/2564/55970/2771/11337/2557/2550/2560/51764/2783/2567/81539/6
4088/3556/4772/53342/3123/3553/3459/3119/4773/3113/3572/3554/3566/7040/3127/3122/3108/5595/6
2247/1871/6934/57580/55970/7132/4772/3133/7538/1026/5608/11337/3459/10379/4773/51764/718/35
217/223/2023/230/80201/501/226/83440/3945/84532/2597/7167/5315/2203/5106/3099/5162/224
2247/5578/2263/108/71/2261/56034/284/7410/2771/2246/57568/1956/1436/5608/54518/23683/60/3690
482/477/5578/108/8458/2876/148327/2878/821/64764/7849/493869/432/2877/5330/5582/2882/468/370
1647/26471/5077/2120/1026/1436/3398/6935/4208/5090/6688/1031/4616/2308/1051/4005/1643/3486/9
9843/3162/326625/1356/210/1355/645/7389/2235/7390

5578/2064/2023/230/284/80201/112399/1956/596/1026/226/5209/3945/3459/7099/3162/2597/5595/153
4257/373156/2950/873/2949/3290/2946/2947/64816/1558/2941/1559/4259/2948/124/2052/1576/128/15
3030/18/7915/56898/1962/54988/1892/3157/35/38

7042/3123/3553/65108/3459/2212/7099/3119/1915/3113/718/7040/3127/3122/2214/3108/5595/4689/15
3133/3123/3119/3113/3105/3127/3122/3108/3107/3106/3109/940/942/3115/3111/3134/355
71/3696/3691/7042/3908/3679/5565/60/3690/4625/6442/7040/1605/489/10369/1756/3685/27091/3693/
2247/5578/2263/23179/4915/2261/56034/284/55970/2246/1956/399694/1436/5781/7423/51764/2885/3
79966/54898/9415/6319/51/9524/3295/3992/6342
284/7042/3123/3553/7099/3119/3113/7040/3127/3122/3108/3689/1435/6374/3109/8992/940/942/3115/
482/477/4625/1339/489/1327/10369/444/7381/7385/10975/27091/1350/7134/7384/93589/1340/7139/13
2247/558/5578/2064/2263/2261/56034/1956/596/399694/2885/5595/6774/5290/3265/3479/5582/4233/6
8453/4591/8916/7326/9817/8452/7320/92912/55294/89910/9820/23327/29882/7322/65264/51588/2589
10768/7263/26227/55256/875/29968/3945/2937/113675/586/4548/27430/6723/58478/191/6611/4191/2
3418/50/84706/586/3417/3421/3419/3420/137362

4257/373156/2950/873/2949/3290/2946/2947/29785/2941/1559/1572/4259/2948/124/2052/1576/128/11
2495/30061/23657/6303/2512/3162/1356/2937/2879/1536/2180/23516/7419/112483/2729/2181/55240/
2247/2263/9459/71/4478/3696/2261/3691/56034/55970/7410/2246/1956/2934/4342/10163/3679/60/369
1871/1647/3133/596/3123/1026/5608/9541/960/3119/10379/5719/4616/3113/1643/3105/3127/3122/328
348/347733/2915/4700/7132/1460/6391/9776/4701/51107/3553/84617/5719/4720/840/4726/2597/5693/
216/9249/51109/220/8630/29785/1558/54884/10901/1559/124/10170/1576/316/128/8608/56603/15883
8801/3418/50/6391/4967/8802/5106/5162/6389/4191/3417/3421/3419/3420/4190/2271/6390/6392/47/5
482/477/2495/30061/9843/4501/79901/2512/4502/4493/3162/4499/4496/538/115019/6550/4489
6385/3696/3691/7060/3908/960/3910/6382/3679/3690/1286/1605/10319/3685/1293/1284/7143/3693/31
5578/7465/55970/2771/7132/4772/3133/596/5608/7099/7133/4773/51764/494551/162/60489/3105/278
5578/7410/2534/4772/6452/3133/399694/5781/3459/4773/2885/3105/2214/5595/3107/3456/3689/5290/
5578/71/2212/7099/60/3690/2358/718/2214/5595/4689/1536/1182/3689/5290/6850/27180/79885/6404/
217/223/4128/55748/3176/501/4129
5578/4257/4088/1871/1647/71/6934/2950/7042/1956/399694/1026/2949/2946/60/3162/4616/2885/2947
2064/4088/71/6934/2534/1956/1495/1460/10163/60/56288/7082/7525/5595/7454/387/29119/10580/579
5730/873/2876/2878/1573/391013/1558/493869/242/2877/8398/1559/5742/2882/5322/6916/8644/4048/
1191/5648/2152/716/710/7035/715/713/2157/712/718/966/714/5055/11326/3689/1380/7450/728/722/5
23493/6934/3696/3691/5521/7060/4853/1956/7132/3133/23462/3908/1026/26508/3910/148327/3679/1
5578/71/90865/9230/7132/79671/3123/3553/3459/7099/3119/60/10379/2224/3113/55916/3127/3122/45
975/7410/4772/11026/3635/118788/10288/4773/11006/2885/23547/5595/974/930/353514/5290/6850/3
5578/55616/7410/2934/10163/3635/65108/4651/2212/4082/2214/5595/85477/7454/3055/5290/6850/55

5578/108/55970/2771/1956/399694/1910/148327/7423/51764/409/2885/64764/2783/7040/1286/10022/
3133/3123/3119/3113/3105/3127/3122/3108/3107/3106/3109/940/942/3115/3111/3445/3134/355/3135/
53832/7132/1436/7133/3572/5473/8809/1435/3588/4055/1230/6374/1234/3587/9547/1524/8740/3606/2
5578/108/2565/2555/8622/5142/5144/2564/55970/2771/2557/2550/2560/51764/409/2783/2567/2775/27
2247/1871/1647/56034/2246/1956/1026/4616/1643/1029/5595/5290/1869/3265/3479/4233/3082/8074/5
348/5360/1593/10577/3931/4023/51129/341/7419/6272/3988/345/338/7416/337/19/336/6646
1647/596/1026/3732/4616/84883/1643/1029/3486/894/6241/3479/64393/900/63970/5054/8795/55240/9
3030/217/223/51268/1962/501/10157/8424/1892/5352/63976/38/224/80854
3032/3030/54898/10449/9524/1892
108/71/3696/3691/7042/3908/3679/60/3690/4625/6442/7040/1605/489/10369/1756/3685/27091/3693/3
2771/7042/7132/596/3908/3123/5608/3910/3459/7099/3119/3113/7040/3127/3122/3108/5595/10454/27
79966/10890/1374/5521/9230/6319/5209/148327/6720/5565/6517/51094/2308/148/64764/81617/2194/
2495/90865/2752/2746/7132/2512/596/3553/3459/7099/10379/5834/1536/6778/6774/3456/815/25978/3
975/7060/7042/3553/6382/7099/7040/3689/5175/3606/100528032/4233/3082/3039/7057/2995/7059
113026/71/5333/80201/1956/7132/596/7100/10163/826/3553/664/3059/11337/960/7099/60/718/831/23
5578/108/11214/5142/5144/2771/1956/596/1026/9247/148327/4208/409/4323/64764/2768/4666/5595/5
10628/1508/84168/79671/596/3428/3553/11337/25828/7099/10379/59082/5595/1536/10454/64127/345
2064/1647/6934/1956/1495/1026/4616/2885/1643/5595/5290/29119/3265
23205/2194/2180/55301/54995/2181/23305/32/84869
217/223/501/9104/224/219/55586
5578/71/4478/7410/2771/1495/5010/5781/50848/60/9076/4689/1536/83692/3689/5290/387/29119/5175
217/223/501/2739/3945/197257/84532/5315/5106/38/5162/224/124/4191/219/32/128/4190/9380/2271

3123/3119/3113/3127/3122/3108/3109/2207/3115/3111
4257/2950/7084/2949/2946/2947/6240/2941/6241/4259/1806/1890/2948/1854/4831/7172/50484/1576/4
2247/2064/2263/4088/1871/1647/6934/2246/7042/1956/1495/596/399694/1026/4616/2885/1643/7040/5
5578/1871/1647/1956/399694/1026/4616/2885/1643/1029/5595/815/5290/1869/3265/3479/5582/6464/5
2247/2064/23493/1871/1647/6934/4853/2246/1956/23462/399694/1026/26508/4616/2885/1643/182/32
1647/6934/1026/4616/1643/7849/5595/3265/6256/7175/6257/5605
2771/716/710/3553/715/7099/713/712/718/840/714/5595/3689/6374/387/834/722/1072/725/808/35337
1593/3295/9023/6342/6718/9420
2064/2263/1871/6934/2950/56034/1956/596/1026/148327/6935/2308/2885/64764/5595/5290/1869/102
2934/3133/3249/1026/28973/148327/10379/718/3572/387332/2885/64764/27044/3105/1029/894/5595/
2752/50/847/84532/2653/38/5096/2731/84693/4191/132158/4190/9380/112817
230/226/9104/51071/2203/7086/5226/221823/6888/132158/2821/2539/22934
2064/4088/1871/9459/1647/7042/1956/1026/4616/1643/1029/7040/5595/6774/5290/1869
1901/4088/1647/7042/1956/1026/664/11337/5565/6517/847/4616/2308/2885/7040/894/5595/6774/1134
6652/230/80201/226/5209/55556/7167/5208/2203/3099
2064/4257/2950/596/1026/2949/2946/7153/2947/1029/5595/2941/5290/538
10057/113235/6472/471/4524/4790/3551/1719/9429/2618/6470/1244/6573/4363/1147
217/9249/51109/158067/29968/55163/8630/4522/9104/326625/10797/2937/210/586/5315/122481/1101
2890/2565/2555/2564/2557/2560/116444/2567
1509/7042/7132/596/3123/972/3553/3459/2212/7099/3119/3920/3113/718/1051/8625/7040/3127/3122/
5578/2064/1871/1647/1956/1026/4616/2885/1643/1029/5595/6774/5290/1869/3265/6256/5582/4233/30
347733/5578/108/56034/2915/2771/1956/57369/2697/84617/5592/2885/7082/5595/7846/3265/84790/5
658/4088/71/6934/7159/5521/23286/154796/2246/7042/1495/10413/3398/25937/60/56288/6657/7040/8
4088/1871/1647/7042/399694/1026/5781/4616/2885/1643/1029/7040/5595/5290/1869/1027/3265/861
482/8671/477/358/2746

3977/3590/1956/53832/596/53342/3597/1026/9180/2670/5781/3459/10379/3572/2885/3566/5292/894/3
482/477/5578/113026/71/5333/4853/60/3690/4625/53919/2308/7067/10499/5595/489/5208/51196/3685
5578/71/9414/60/10945/7082/8992/155066/534/5336/9550/90423/50617/5567/51382/23480/375/1080/5
4088/677/1871/1647/7042/678/4772/3133/1026/5608/4773/4616/2308/3105/1029/7040/3486/894/5595/
143/5783/1519/1647/71/1509/7132/1508/596/60/1075/8722/4616/840/1512/5595/56616/5290/7846/326
2064/1871/2261/1956/1026/1029/5595/1869/3265
6385/4257/658/71/2950/7132/596/2949/3553/5608/2946/25828/6382/60/3690/3162/4208/2947/3554/36
23205/1374/7132/5781/5565/6517/7133/51094/5465/3953/6774/79602/5106/9021/5588/2180/6256/625
2064/3418/2263/2261/80201/1956/3945/6510/5595/5315/5290/3099/3265/5162/51548/4233/5156/5605/
83732/51367/51096/283106/4931/10528/29102/29889/29107/6023/55127/23195/55131/134430/5822/57
108/8622/5142/5144/131870/51292/158067/957/4860/10606/4881/2766/318/5315/27115/6240/122481/

4460/6202/6122/6143/6209/9349/6205/6124/3921/6183/2197/9045/6142/6224/6169/23521/6203/6132/6
/7638/162972/7738/5610/10000/9668/5199/10520/7773/7748/9310/8890/58500/126017/284406/84775/6
572/6159/7311/6135/6166/4599/6128/714/5595/6157/1536/6137/6774/3456/6134/6138/6147/6202/6122
/2941/6241/2877/5226/4953/4259/2882/6723/2948/6611/2729/3417/50484/2936 096/84693/36
/33/2180/36/54995/79993/2181/23305/51144/9374/84869/1376
3/56901/4724/7384/1340/1329/1337/4702/29796/1351/6389/4725/4713/155066/64077/534/9167/4711/1
/84532/113675/2597/8802/7167/5315/2653/2203/35/7086/3099/38/5162/5226/5096/2731/84693/221823
387332/64764/4726/5693/5436/5685/1339/5689/1327/374291/493869/51164/54539/7846/4695/7381/550
5106/10580/33/5467/2180/2171/6256/2173/7316/345/6257/2167/2181/23305/4973/440503/1376/2170/3
7/374291/815/54539/5290/4695/7381/5580/7385/5162/10975/1350/4723/56901/4724/1277/7384/1340/5
1339/5689/1327/374291/815/54539/7846/4695/7381/5687/7385/10975/1350/4723/84790/56901/5710/47
3/60/84617/5719/847/4720/55916/4726/5693/5685/1339/5689/1327/374291/493869/25978/11345/51164
774/3109/3115/3606/6772/6095/3111
3122/2214/3108/4689/1536/5878/3107/3685/3689/7846/3106/3109/10333/3693/8992/84790/4074/2209/
26/1436/9180/118788/3910/148327/3679/7099/3690/7423/51764/2885/64764/1942/2783/3566/1286/11
5/219/1376/128/113612
7/120892/2878/7133/84617/5719/847/4720/840/7311/4726/5693/5595/1536/5685/1339/5689/489/1327/3

5693/714/5595/4689/1536/5685/1339/5689/1327/374291/54539/5290/7846/4695/7381/5580/5687/7385/
4/2209/3115/5724/8689/728/2357/2359/3111/820/3880/2213/25984/3882/727/9103
109/940/942/3115/6444/25/3111/5880
2/54539/5290/9021/4695/7381/7385/10975/1350/4723/56901/4724/7384/1340/6256/1329/1337/3643/47

3594/10901/6648/2180/5824/6647/5264/10654/11264/4598/3155/2181/23305/3417/4358
832/219
3385/3122/3108/5133/3107/2734/3685/83692/3689/3106/3109/23308/940/942/3655/6404/3115/5175/96
85/3554/7040/2214/23547/5595/4689/10454/3456/3726/353514/1435/5290/9021/6850/3455/7305/3454/
53/27430/221823/5832/3417/6888/3421/3419/445/3420/137362/22934
3821/3115/920/3111/811/4261/3134/10437/3306/6890/3135
074/1512/8763/5641/5660/968/950/6448/4074/427/22901/2581/8905/53/6272/23659/3988/1203/410/93
130399/7133/3572/5473/3554/3566/7040/8809/3953/1271/3456/1435/3588/4055/1230/6374/1234/8771

595/2775/374291/54539/4695/140679/4723/54331/56901/4724/2788/5330/2787/5582/4702/2785/4725/
329/218/1544

257285/64764/2768/3105/2783/3554/1029/5595/2775/3107/6774/3456/3685/3588/1230/5290/3106/387/1234
5290/7424/5580/1284/1027/3265/1277/5330/1282/5054/6772
8/23339/2597/5595/257364/10454/57381/5878/51164/302/5290/7846/51429/387/9367/10093/3265/103
3/2885/1286/4638/894/5595/10319/3685/5290/7424/1293/1284/387/7143/3265/3693/3479/3655/10298/
7/374291/54539/4695/7381/7385/3265/10975/28958/1355/1350/2180/4723/56901/4724/7384/1340/132
09/1380/3655/2209/3115/920/3672/7850/3111
113/821/387332/64764/3105/3554/1029/7040/3127/3122/3108/894/5595/2113/3107/3689/4055/5290/31

4617/840/4542/9076/2768/3071/3554/2597/7082/5595/10454/7846/64005/387/10093/27128/84790/834
/3693/3655/93589/6444/3672/22801
90/2885/7040/5962/7078/5595/6774/815/51196/3685/5290/387/3265/3693/3479/1277/7476/7430/5582/
529/2775/11345/140679/54331/23710/2788/2787/9001/5582/2785
778/6774/3109/5588/861/3115/196/6256/920/6772/3662/6095/3111
72/7311/3105/2783/9976/5595/3107/6774/3456/3055/11345/1230/5290/3106/6850/1234/1869/3265/345

/7423/56288/1942/5595/2775/51196/4254/3689/1435/5290/7424/387/3265/3479/9863/5216/5330/3643/
8/7276/2776/2936/90993/5567
915/7849/894/5087/1027/861/3479/942/55589/2209/597/6256/7850/7030/4291/4149/5218/904/4233/42

6/6774/815/4055/5290/3099/7076/1027/5162/3479/3643/5582/5054
51/119391/218/1544

36/10454/3689/3109/2209/3115/6772/3111/2353/2002/1535

3479/3655/7134/93589/6444/7139/4634/3672/22801/3680/488/5564/7137/786/778/59283/10368/1906/5
91013/1942/2783/5595/2113/5878/51196/4254/8437/1435/5290/7424/387/3265/8398/3479/54331/10298

8741/3606/3111/155066/534/2353/9550/90423/50617/51382/3600/6348/527/9114/533/3458/9296/941/5
29/1337/4634/29796/1351/9167/1345/7386/488/7137/786/778/59283/481/1349/3270/10368/7170/7388/
464/3791/3082/5156/5336/5605/3084
8/51433/22954/9039/51434/55236/6502/10054/8451/140739/8651/9616/63893/51465/7325/7329/26091/
729/23743/137362/4190

9391/218/1544
23305/10162/84557/81631/643246
0/2768/3071/5962/4638/5595/85477/3685/3689/10152/5290/387/3985/10093/3265/3693/3655/10298/52
0/3108/894/10454/3107/6774/3456/930/5290/3106/6850/3109/1869/1027/3455/3454/567/1380/5710/69
4023/5595/1536/5685/1339/83464/5689/489/1327/374291/1435/54539/55851/5290/7846/4695/7381/55
5/1551/6121/112724/1544/8228/317749/125/5959

160
61/3655/1277/7450/1282/3672/341640/22801/3680/22987/7057/1287
3/5595/10454/2775/3107/3456/85417/8450/5290/3106/3985/1234/3265/567/54331/10298/2788/2787/89
3106/6850/3265/3455/7305/3454/3821/2207/5582/2185/100528032/8795/6464/7409/5880/5336/5605/9
2209/7450/834/51284/5330/728/79792/7419/5582/2357/10105/2359/5880/837/5336/820/5605/1535/292
/1643/1029/7040/5595/2941/5290/1869/3265/122011/7476/5582/4259/4233/2948/6464/3082/5336/1452 7/3643/4233/5880

4051/30814/113612/5320/283748/2687/151056
054/5265/2162/725/2155/5627/727/7448/9002/3075/2153/717/2149/2244/730/3687
0379/3690/387332/2308/2885/10474/64764/56288/3105/182/4599/1286/3280/894/5595/3107/3456/1031
99/11100/3108/5595/3456/5290/9021/3109/3455/3454/6041/3115/834/51284/10241/3606/6772/8795/31
636/3265/1380/695/10990/27071/7409/5880/5336/2353/5605/11025/5879
80/3636/3985/10093/8398/2209/5582/10109/1793/5338/1072/7409/5880/10094/5336/5879/10810/5788/
3640/5595/2775/5290/7424/1284/3265/54331/1277/2788/5330/2787/1282/2785/468/6464/2353/5605/40
3452/958/941/3447/3443/356/3439/3002
9949/8795/1236/8764/3570/7852/6370/1233/10563/1235/6348
115/140679/54331/2788/2787/5582/2785
156/5605/4286/2253
83/50484/7057/355/143686
479/3655/7134/93589/6444/7139/4634/3672/22801
75/6774/10319/3588/1234/3109/3655/3587/3115/10105/6772/3111
92335/3953/5208/79602/5290/2203/5106/1938/3479/5525/7248/3643
455/3454/7128/8837/834/7419/7188/6772/197259/8795/8878/353376/292/3445/3718/7416/355/29108/8

08/7311/3554/5595/10454/3456/11345/51196/5290/5580/387/55062/3099/10093/5588/27128/23710/521/744/387/5330/6256/
5582/5338/468/3708/8074/6257/2353/2776/408/90993/5567/8606/11345/388646/7205/5580/387/3455/3454/6041/23710/7128
/7295/834/5330/79792/7419/58484/114769/7430/5582/2185/7409/5880/58494/5336/10411/1003/1535/5879/7852/4688/9071/
7122/1364/394
353/119391
595/5290/29119/1869/1027/3265/122011/7476/6256/4233/6464/3082/8074/6257/1452/5605
156/808/5336/5605
80/5595/5290/10683/1869/3265/3479/122011

6/2353/29108/114548/727/148022/1073/929/717/5602
7/3265/3479
85477/3107/6774/5315/4055/5290/3106/6850/8850/387/1234/1027/3265/79885/2967/7419/1739/7531/7
5/85417/5290/5106/1027/3265/6648/3479/23710/80854
9/203/1355/224/27430/23475/54995/8942/60490/7389/2729/2235/4831/7360/219/79717
2214/26253/3108/5595/64127/5878/3456/815/3689/3588/6850/387/3109/10333/2209/3587/2207/3115
82/6257/5336/5605
330/5582/3708/5156/5605/2776/983/5567/1813/5607
94/3689/29119/64398/286204/653/7476/1739/7531/5054
953/1271/6778/6774/3456/3588/5290/9021/3265/3455/3454/3587/1443/6772/29949/3601/1489/2688/51
/5290/8850/3265/1735/5330/6256/5582/6772/25942/6257/5336/560527/5566/9114/533/10952/2778/9296/51606
2113/3107/85417/5290/3106/1869/3265/7248/7419/5054/5883/3708/8878/808/4683/5605/983/292/3134
5/84790/8837/597/63970/468/8795/4803/3708

85/2941/5290/387/5175/7295/4259/7850/2948/3791/5880/8878/808/2353/1003/1535/5879/445/5607/46
7/2181/23305/32/5564/8660/4852/6258/5571/6794/5602
3417
455/55341/6949/166378/10482/10799/10813/10940/9790
6241/203/4907/5167/353/221823/377841/115024/5147/4831/132/56953/272/50484/2987/5153
141/6217/6191/11224/6230/6130/6167/6171/6168/51318/6125/25873/6129/51023/6146/51073/6223/61
4170/79724/91975/8427/80264/286075/284371/51276/6428/163051/90592/63934/92285/162962/14792
/5290/6143/6209/6850/9349/6205/6124/3921/3455/3454/2197/9045/6142/6224/6169/7450/834/51284/2

345/4717/55967/7386/4729/27068/9550/4728/4708/4710/4697/4698/126328/90423/50617/51382/4722/1

/6389/2098/4191/3417/6888/3421/3419/132158/3420/128/2821/137362/2539/4190/22934/2271/2026/13
62/5687/7385/83544/2877/11258/10975/6648/1350/4723/84790/56901/5710/4724/7384/163/10120/6647
36/6258
330/1329/1337/7419/3643/5582/4702/29796/1351/6389/10105/4725/4713/5880/9167/4711/1345/4717/5
24/7384/7295/1340/1329/1337/5706/7419/4702/29796/1351/6389/10105/4725/4713/468/7316/5691/370
/54539/7846/4695/7381/55062/5687/7385/83544/2877/11258/79023/10975/1350/4723/23710/84790/569

3115/9341/3111/155066/5869/534/5868/23673/811/9550/4973/1535/5879/7057/3134/90423/50617/4688
140/894/5595/3456/930/4254/10319/3685/1435/5290/5106/7424/1293/6850/1284/7143/1027/3265/3455

74291/493869/815/25978/11345/51164/1435/54539/7846/4695/7381/55062/5687/7385/3265/83544/287

10975/1350/4723/84790/56901/5710/4724/7384/6647/1340/1329/1337/5706/7419/4702/29796/1351/638

02/29796/1351/6389/4725/4713/468/9167/4711/1345/4717/55967/2353/7386/4729/4728/4708/4710/469

5/5797/920/3111/6614/58494/3680/1003/152404/3134/3384/5788/84628/9071/7122/1364/4099/3135/79
54209/695/10990/2209/2355/6772/8878/5336/2353/4286/3937/1535/11025/5879

74/50617/8546/256471/2517/138050/10239/3423/3482/4126/1211/527/9179/8943/1201/58511/9114/117
/3455/3454/3623/23765/3587/9547/653/1524/920/8740/8741/3606/939/1443/7850/29949/58191/8795/36

4713/3708/4711/4717/55967/4729/2776/4728/4708/4710/4697/4698/126328/5567/11343/1869/3265/567/54331/3587/2788/

5330/2787/7248/5582/2185/2785/7188/468/3708/5880/5156/808/811/

33/11258/27128/84790/4074/5216/7295/10120/834/79792/58484/10109/3606/1639/197259/8795/10121/

1277/7450/1282/5582/1793/3672/22801/4233/6464/3791/7409/5880/3082/3680/5156
9/1337/7248/51548/4702/29796/1351/6389/4725/4713/123096/9167/4711/1345/4717/55967/2181/7386/

06/8850/3109/1869/3265/567/3115/7419/64682/1739/920/7850/468/3601/3111/2353/811/5605/2002/55
/4691/7430/10109/3606/25/55930/8795/55971/10094/837/2353/6188/55845/4646/5879/355/10810/2910

4233/3791/7409/3708/3082/5336/5605/2002/5879/7057/355/5567/818/6608/7474/10818/7484/6194/309

5/3454/54331/23710/942/2788/2787/2785/6772/7316/3708/808/5336/2353/5605/5879/3445/3134/355/47

5582/2357/4803/4233/3791/7409/5880/3082/8074/5156/808/10411/23566/5605/2776/5028/3937/375189

21/3087/5081/25942/6257/4094
571/7170/779
/2788/2787/3643/5582/2785/5322/25/22821/5338/4803/4233/5869/6464/3791/64926/5880/3082/8074/51606779/11065/571
54/57448/51619/11059/134111/10055/378884/8945/867/6477/83737/8924/118424/4214/845416/7114/7430/10109/81624/1
793/3672/1730/1072/22801/7409/5880/8074/10094/128239/3680/5156/565/7128/3115/965/5706/7188/6772/377841/3111/5
709/25942/5336/811/7431/11317/5879/3445/3134/371
062/5687/7385/3265/10975/1350/4723/84790/56901/5710/4724/122011/7384/1340/7476/5330/1329/133

05/5582/2185/2785/920/7188/1072/164668/3708/5880/808/5336/2353/811/5605/2776/983/1642/5879/34
62/3937/5879/3445/355/3384/5879/7416/5605/2002

9/10134/3685/5315/5290/3106/1293/1284/7143/1869/1027/3265/3455/3454/3693/8992/3655/122011/64
11/8480/55998/5605/4261/5646/292/3445/7416/355/29108/2213/283748/5058/1073/10095/653361

8/1287/1281/90993/556718/114548/283748/9825/51510/148022/51652/331/2747/79643/1540/27243/5602/8737/3452/3458/
87976/834/5330/58484/10241/199746/10109/1793/3606/7188/7316/3708/8878/10094/837/5336/375189/587/7531/3606/718
8/6772/3708/837/820/51393/1535/3445/7416/29108

188/468/11317/983/55697/1642/5879/3134/3718/90993/5567

56/3445/3718/8554/3570/1441/316/2690/4170/3600/3596/3976/7416/477688
60/6206/6161/6188/6152/29088/51081/6222/6170/65003/6156/6133/6154/6194/6165/6201/64963/6231/
9/170959/637/730051/162967/79898/65243/7772/168417/842/27300/10795/7770/84503/284307/54811/6
3521/6203/6132/728/6141/6217/5582/6191/11224/6230/6130/6167/6171/6772/6168/6125/25873/6129/6

349/6390/7388/4715/527/4712/4718/9114/533/4709/51079/1347/9296/1537/6392/51606/4719/526/4694
73/6390/2747/3098/9563
/1340/5330/1329/1337/5706/7419/9001/4702/2882/29796/1175/1351/6389/10105/1639/4725/4713/569

5967/7386/4729/4728/4708/4710/1535/4697/292/4698/5879/2936/126328/7416/1281/818/488/1376/46
8/9167/5709/4711/1345/808/2861/4717/55967/7386/4729/65018/4728/4708/4710/4697/11315/292/4698
01/5710/4724/4744/7384/5216/10120/6647/834/1340/1329/1337/5706/9001/4702/2882/29796/1351/638

/2213/7059/51382/23480/7879/64581/4353/6890/9103/3135/929/653361
/3454/3693/3479/54331/3655/9863/1277/7450/2788/5525/6256/2787/7248/1282/3643/2785/7531/3672/

7/11258/10975/1350/4723/23710/84790/56901/5710/4724/122011/4744/7384/53349/10120/6647/1340/7

9/10105/4725/4713/468/5691/3708/5880/9167/5709/4711/1345/4717/55967/7386/4729/4728/4708/4710

7/4698/5879/126328/355/3570/5564/8660/4722/1349/6390/5571/7388/4715/5602/4712/4718/4709/5107

679/214/6693/23562/1370754/53301/4803/10148/1489/2688/8784/1236/3445/355/392255/8764/3570/7852/1441/6370/655/26
90/84818/1

5605/2776/2002/5879/3445/3134/355/4776/90993/5567/3570/7852

5869/10094/837/5868/2353/6188/5605/79026/10671/10540/375189/55845/55823/4646/5879/27072/108

23305/4729/4728/4708/4710/4697/4698/126328/90993/5567/11343/51287/5564/1376/10818/6194

697/292/3134/3718/7416/4776/2114/90993/5567
8/4640/57121/9071/114548/9871/7122/375/13641/5058/7448/3709/81029/2549/5727/2065
76/285/7314/2253/5879/7057/285/1813/1945/5217/57121
156/808/5868/5336/5605/2002/2253/5879/30814/285/2114/5567/51365/1945/10401/8925
05/375189/2253/55845/5879/221178/1132/7852/5217/571218/355
7/5706/7419/3643/4702/29796/1351/6389/22943/10105/4725/4713/468/5691/3708/10888/9167/5709/4745/3134/355/4776/65
00
398/1277/7450/92359/7476/5525/7248/1282/1739/3672/6772/22801/155066/534/5527/3680/1452/5

605//9131/8605/3447/3443/9525/142/824/356/3439/5837/290829/7416/6500/7314/29108

6234/51264/6233/28998/6155/6176/6210/63931/6208/6139/51187/6207/6164/6189/4736
432/158431/388566/374900/80032/7988/10482/8503/126231/117608/7587/22869/7733/342892/30832/2
146/6223/2162/5336/6160/6206/59272/6161/2353/6188/6152/3445/6222/6170/6156/3570/6133/6154/61

1/10121/3708/9167/5709/4711/1345/3766/25942/4717/55967/7386/4729/10671/2776/1770/10540/4728/88/7137/2539/51660
/126328/7416/5567/1813/7314/818/9627/5683/10213/6263/5688/2774/4722/7345/3709/5714/1349/6233
9/348995/310/1639/4725/4713/468/55255/5691/10121/5630/8480/7175/9167/5709/8878/4711/1345/559

1443/468/22801/4803/4233/3791/3082/5527/8074/3680/2688/5156/23566/5605/5518/2253/5879/7057/3

476/5330/1329/1337/5706/7419/9001/5582/4702/2882/29796/1351/6389/22943/10105/1639/4725/4713/

/1535/4697/292/4698/5879/126328/7416/90993/5567/4688/5683/10213/6263/3306/5688/778/727/4722/

9/10062/1347/51422/1537/6392
233/3600/3596/3976/9966/10563/970/1235/2660/634810/6500/29108/5294/5788/5217/5287/114548/140735/7879/375/5058/5
1626

11/1345/808/1452/4717/55967/7386/4729/5605/2776/4728/4708/4710/4697/292/4698/126328/7416/355
9550/11317/5518/7057/3445/3134/90423/355/50617/1287/90993/5567

01514/79818/57343/155061/6431/374928/81856/125919/256051/339559/147687/3717/348327/79862/75
94/6165/6201/6231/114548/6234/727/6233/6155/6176/6210/6208/6139/51187/6207/6164/6189/717/473

4708/4710/1742/4697/292/4698/126328/7416/90993
/7317/6390/7388/4715/5602/4712/4137/9927/4718/5695/5566/4709/51079/5682/2778/1347/1537/6392/
98/5868/4717/55967/7386/4729/10671/1770/3084/10540/65018/4728/4708/4710/375189/4697/4698/58

445/3718/1287/285/90993/3570/1945/5294/1441/57121/6194/7059
468/7316/55255/5691/10121/5630/3708/6310/9167/5709/8878/4711/1345/808/5868/2861/1452/4717/55
3709/5714/1349/653361/6390/7388/4715/779/5602/4712/2904/1388/4718/5695/5566/4709/730/51079/5
/8883/488/7474/7484/8660/23621/5683/10213

51/171392/7699/7596/345462/83744/90233/10780/146434/7592/7556/152687/93134/7559/7769/197320
6/5602
5694/113457
79/126328/7416
967/7386/4729/5605/10671/2776/1770/10540/65018/4728/4708/4710/1742/4697/11315/292/4698/5879
682/1347/1537/6392/5694/113457
/253639/6940/255403/7554/90075/6732/7743/7710/285676/7633/7574/163049/388567/388558/6885/284
/126328/7416/355/7314/818/488/9627/7474/7484/5683/10213/6263/5688/778/4722/4218/7345/342371/
306/163087/57541/147923/9451/51710/1147/7637/170960/136051/163081/388536/140612/7561/34734

3709/5714/1349/6233/81029/7317/6390/177/7388/4715/779/5602/4712/7483/4137/5173/2904/9927/203

4228/4718/5695/6712/1454/4709/51079/5682/1347/5861/4741/9706/1020/1537/6392/5694/113457/9973
