# Supplementary material for: Exploring the diagnostic markers of essential tremor: A study based on machine learning algorithms
Source: Open Life Sci. 2023 Jun 22;18(1):20220622. doi: 10.1515/biol-2022-0622 (PMC10290283; doi:10.1515/biol-2022-0622)
Supplement: Supplementary Table 8 [file biol-2022-0622-sm9.pdf]

Table S8: pathways involved in ZNF148 identified by GSEA.

ID	Description	set Size	enrichment	NES	p-value	p.adjust	q-values	rank	leading_edcore_enrichment
hsa04120	Ubiquitin	135	0.490261	2.3432	1.51E-10	4.95E-08	4.00E-08	5093	tags=51%, 996/3093/8065/51465/55284/25898/7329
hsa04610	Compleme	73	-0.50406	-2.11946	1.74E-06	0.000285	0.00023	4776	tags=47%, 714/3426/7035/5265/718/2/2152/2162/2
hsa05010	Alzheimer	336	0.295072	1.595812	2.44E-05	0.002457	0.001985	4165	tags=29%, 10000/3845/5601/3799/5332/8851/6622/
hsa05017	Spinocere	137	0.375298	1.797281	3.40E-05	0.002457	0.001985	3897	tags=33%, 10000/5579/5601/23369/5332/5718/5291
hsa03013	RNA trans	154	0.365644	1.780681	3.76E-05	0.002457	0.001985	3965	tags=31%, 2332/1977/10762/55520/9972/7329/5180
hsa04512	ECM-recep	87	-0.43647	-1.90558	5.26E-05	0.002869	0.002318	5651	tags=46%, 7057/1297/2335/6696/6385/1292/158326
hsa03040	Spliceosom	129	0.373762	1.777878	6.90E-05	0.002952	0.002385	5078	tags=42%, 220988/3190/5356/6627/51729/6632/730
hsa05012	Parkinson	217	0.326111	1.663659	7.68E-05	0.002952	0.002385	5681	tags=40%, 5601/3799/51465/6622/5718/6571/836/3
hsa00590	Arachidon	56	-0.4871	-1.92534	8.12E-05	0.002952	0.002385	5326	tags=48%, 2877/1557/2678/5743/5319/1559/240/10
hsa04144	Endocytos	239	0.309575	1.605733	0.000162	0.005292	0.004275	3765	tags=31%, 3799/8027/22841/10097/5584/57403/419
hsa05014	Amyotrop	328	0.284884	1.532922	0.000273	0.008128	0.006567	2960	tags=22%, 220988/29110/3799/10762/9972/9782/55
hsa05110	Vibrio cho	48	0.479886	1.864508	0.000318	0.008674	0.007009	4006	tags=48%, 11014/23545/523/528/375/2778/527/60/
hsa05022	Pathways	439	0.259385	1.428149	0.000398	0.010021	0.008097	3870	tags=25%, 29110/3845/5579/5601/3799/51465/5332
hsa00190	Oxidative	103	0.381295	1.743816	0.000461	0.010771	0.008703	5681	tags=41%, 10063/23545/523/528/4719/4726/4704/4
hsa04060	Cytokine-c	248	-0.30182	-1.5553	0.000615	0.013152	0.010627	5541	tags=33%, 3976/55504/653/10220/60401/3452/959/
hsa04721	Synaptic v	77	0.400077	1.723695	0.000669	0.013152	0.010627	4877	tags=47%, 6571/23545/523/440279/528/1212/1213/
hsa04720	Long-term	66	0.431115	1.8047	0.000684	0.013152	0.010627	2701	tags=35%, 3845/5579/814/5500/5332/5532/673/817
hsa03015	mRNA sur	92	0.377332	1.693509	0.000959	0.015812	0.012776	5285	tags=43%, 2935/5515/11051/5500/23283/5529/5520
hsa04141	Protein pr	161	0.33239	1.625503	0.000973	0.015812	0.012776	3804	tags=33%,

								5601/51465/10427/10802/7322/29979/70
hsa04640	Hematopo	84	-0.39305	-1.6984	0.000978	0.015812	0.012776	6965 tags=46%, 3109/3552/2209/947/1435/3113/3108/92
hsa04140	Autophagy	131	0.337218	1.606504	0.001015	0.015812	0.012776	3801 tags=31%, 58528/29110/10000/3845/5601/83452/55
hsa05332	Graft-vers	33	-0.53438	-1.88723	0.001372	0.020388	0.016473	6965 tags=70%, 5551/3109/3552/942/3458/3824/3113/31
hsa05031	Amphetam	67	0.393489	1.647514	0.001696	0.02398	0.019376	4011 tags=39%, 5579/814/5500/1385/5532/6571/817/306
hsa04012	ErbB signa	82	0.385972	1.684402	0.00176	0.02398	0.019376	3633 tags=32%, 10000/3845/5579/5601/5291/673/8503/8
hsa05020	Prion dise	244	0.278707	1.449594	0.002619	0.034253	0.027676	4225 tags=28%, 5601/3799/1385/5532/5718/5291/836/85
hsa04360	Axon guid	180	0.30328	1.511026	0.003199	0.038609	0.031195	4114 tags=33%, 659/3845/2048/5532/5291/2045/7220/60
hsa04114	Oocyte me	118	0.336879	1.576143	0.003281	0.038609	0.031195	3749 tags=31%, 996/5515/5500/5532/7532/5529/817/560
hsa00910	Nitrogen m	17	-0.60339	-1.81619	0.003306	0.038609	0.031195	6128 tags=65%, 768/11238/760/763/759/377677/767/275
hsa05330	Allograft r	34	-0.49509	-1.77111	0.003466	0.039081	0.031576	6965 tags=65%, 3109/942/3458/3113/3108/7124/958/959
hsa03320	PPAR sign	73	-0.38712	-1.62777	0.003978	0.043362	0.035036	6167 tags=51%, 729359/2169/1593/7350/2168/364/4023/
hsa04961	Endocrine	52	0.416431	1.655574	0.004261	0.044947	0.036317	2707 tags=29%, 5579/5332/476/8766/490/1212/1213/277
hsa00072	Synthesis	10	0.711538	1.835131	0.004875	0.049817	0.040251	6098 tags=100%5019/39/3157/64064/622/3155/545 11/56

/4193/9690/329/8453/4591/7322/10075/10054/9039/331/10273/55294/8452/10055/140739/1161/55585/83737/4214/23291/134111/11059/9320/7155/624/728/712/2147/5329/
462/715/1361/3053/713/1191/3684/3689/5327/729/716/5054/11326/730/710/5345/7450/10544/966/3075
5532/5718/5291/10313/836/673/8503/3831/5686/2932/1965/4719/5604/10381/291/4726/57142/4704/5713/5534/5594/5701/808/7280/1499/4714/8503/5686/291/10939/564
9/5713/4976/5701/22863/7417/5295/5707/9706/342371/25814/6310/5708/8678/2904/3516/116443/2911/11047/2903/
8/79023/59343/11171/54913/6613/1964/1965/11260/55998/26999/10921/2733/5901/6612/7341/53371/79897/9513/8672/81929/51068/5903/863
/3674/6382/1277/960/1285/22987/131873/1293/3693/1291/3695/2811/80144/7143/3675/3678/3655/1311/3911/3913/3339/2815/1282/7450/2280
7/9716/10594/10291/23350/23020/25804/27258/11325/3183/9092/10569/10286/6634/199746/10929/23450/10523/10450/25949/22938/10915/84
831/817/5686/9927/1965/4719/10381/291/4726/4704/5713/818/5701/808/2778/10131/7280/4714/4723/801/203068/7417/3800/7277/5707/317/5
0137049/8644/283748/5322/50487/2053/2878/123745/239/873/1558/5321/8398/1555/5730/4056/11283/1573/2687/8605
3/51652/10096/8723/382/6456/8766/29924/1212/57132/375/10564/83737/1213/9559/55737/116987/11059/9135/8394/8411/8976/9765/9829/380
32/5718/836/29979/79023/3831/5686/1965/4719/10381/4726/4704/55998/5713/5534/2733/5701/7280/4714/310/4723/9217/127602/10010/55860
534/535/50617/71/5566/5567/9296/51606/90423/525/9114/10952/537/529/526
/8851/6622/5532/5718/836/673/3831/817/5686/2932/9927/1965/4719/5604/10381/291/4726/4704/5713/818/5534/5594/5701/808/10131/7280/1
714/4723/527/534/1355/1353/535/50617/479/9377/4698/496/9296/51606/90423/525/9114/537/84701/4724/4706/529/1345/526/1350/4728/9550
53833/2057/8797/10850/3590/7043/1443/3441/3598/2690/163702/7040/1230/83729/53832/3953/4803/1489/56832/3977/4804/8995/943/10803/3
527/6857/534/6812/26052/112755/535/5864/50617/8218/1173/339302/9296/8775/51606/90423/246213/525/9114/57030/6616/4905/529/526/108
/5604/107/818/5534/5594/808/801/5906/2904/5530/2911/2903/5566/5567/4893
/80335/53981/55998/11052/1477/10921/2733/1478/81608/8731/10284/5518/8761/5523/8732/5516/29101/5527/2521/26986/55110/2107/5501/14
95/10273/10970/11231/1965/3998/10905/51128/10484/9373/81567/7991/80267/267/258010/55666/7415/51360/79139/6745/51009/7326/55968/
0/7124/3570/2323/925/3112/2057/933/4254/3590/3674/3115/3111/960/929/3123/912/2811/3684/3675/1441/3678/3655/3119/3554/2815/924/785
15/3146/5562/5291/8503/1965/5604/64121/5594/84938/5728/7405/10010/22863/5563/5295/9706/6885/8678/116442/5599/5566/5567/4893/9821
08/7124/3112/3115/3111/3002/3105/3123/3106/3821/3107/3133/356/355/3119/940
6/818/5534/1386/808/2778/801/2904/5530/116443/2903/5566/5567/23411/5173/805/776/2354/5501
17/2932/5604/5058/818/5594/6416/5062/2002/4690/5295/8440/53358/5599/4893/867/1398/6777/5290
03/3831/5686/2932/1965/4719/10381/291/4726/4704/5713/5534/5594/1386/5701/7280/4714/4723/4684/203068/7417/5295/3800/7277/5707/317
91/8503/817/55740/2932/285220/23365/5058/818/5534/5594/10512/5062/6259/4690/5295/5921/8440/1072/7976/5530/1949/4233/84612/998/39
4/107/7534/818/5534/5594/808/23291/10971/801/898/7529/25847/9126/5530/132864/8697/4085/6500/10393/5566/5567/8454/5518/7531/5516/
2/762/771/761
/3112/3115/3111/3002/3105/3123/3106/3107/3133/356/355/3119/3593/940
2173/1582/6257/33/9415/11001/2170/10873/30/8309/51129/2180/5105/1622/5360/5465/28965/1962/440503/8310/1374/1375/5467/376497/1058
8/26052/3817/8218/5566/1173/5567/478
898/3158/38

334/54926/7428/25847/7326/9063/27338/378884/8697/6500/10393/7325/26272/9616/92912/7316/55958/867/8554/8454/7321/27339/64750/733

/4723/89780/5663/801/324/4040/203068/22863/322/7417/5295/3800/7277/5707/9706/351/317/5708/8324/8678/2904/7976/5530/10376/102/145
11317/5599/9821/6908/54205/6095/5704/7416/5173/9698/5290/7419/115209/7436
7/3837/8661/1975/9631/10284/57122/51095/11218/8761/1915/1983/10248/1968/2521/26986/55110/9688
1/3161/3672/3691/8515/1284/1286/3679/3908

844/10772/9128/6635/10285/10946/6628/27339/3192/9343/2521/55110/1665/27316/10189/153527/8559/10992/10907/22916/6428/6434/55660/
708/7326/10376/11047/112714/5599/25828/5566/5567/10382/9377/7316/4698/54205/5704/7416/9451/805/10383/7419/84701/4724/4706/1345/8

0/9372/26052/23362/27183/137492/51028/10254/5868/378/25978/10193/84612/998/51534/8218/51699/29934/1173/6457/84440/51100/867/273
/203068/23064/22863/60/7415/3800/7277/51164/5707/9706/53371/79139/81929/317/5708/5217/8678/2904/5530/10376/5868/4842/11047/2903

499/4714/4723/89780/9217/127602/5663/10010/801/324/55860/4040/203068/22863/7417/7415/3800/7277/51164/5707/9706/79139/342371/351/
/4711/4725/4707/4705/6389/1337/9167/7381
56/2826/643/4050/3460/1271/284340/3604/3587/2688/8809/654/656/355/3601/8795/8784/1441/58985/658/55540/6351/970/4982/3556/8740/35
15/6844/9550/774/6572

79/23708/53918/10189/22916/5522/5528/5499/26019/55844
5886/54431/56886/6500/5599/27102/10294/5887/573/821/8454/11253/3703/23645/55768/56681/5610/7321/10961/440275/10952/9451/6184/554
0/3672/2208/3566/966
/140775/51100/6009/440275/5516/5861/9451/5290/203228/64422

/5708/2904/5530/10376/116443/1457/11047/2903/112714/1459/5599/5566/5567/10382/9377/4698/5879/54205/779/5704/7416/5621/727/9451/5
84/64101/6586/56896/223117/10154/9860/4893/1808/5879/54434/4636/1948/1946/5781/7224/57715/50855/5290/1073/9475/64218/9353/6387/5
5527/10459/805/97480/6256/10062/51703/126129/123

7/6502/1642/148581/51434/8924/7319/8450/7314/51619/51588/57448/10477/9817/7323/246184

7/4842/11047/2903/112714/1459/5599/3416/4893/10382/9821/9377/4698/54205/83464/5610/1452/779/5704/7416/8883/9451/7480/805/5290/74

6637/23658/22827
42/5682/7846/1350/1616/5717/4728/2773/7314/7345/5695/4711/816/4725/2861/3798/4707/4705/10105/6389/5706/27429/1337/5689/90550/727

/128866/377/7046/60682/7037/5878/26286/30845/6011/28964/2348/9744/58533/50855/9525/64750/10015/2868/9922/64744
/25978/116442/112714/5903/1768/71/10540/10671/9631/10382/9821/9377/57122/4698/140775/5879/54205

6310/317/5708/8324/8678/2904/7976/5530/7326/10376/2911/5868/1457/4842/11047/2903/25978/116442/112714/1459/1768/5599/10540/1067154/10148/4055/8771/8793/

3593/7132/9180/9173/132014/7048/10663/7042/8718/23529/7850/3456/2662/3566/94/1234/7133/1236/84818

32290/10383/776/7419/84701/4724/4706/1345/842/5682/7846/1350/747/84448/91584/3983

76/22943/10383/776/7419/84701/4724/4706/1345/4843/842/1133/5682/784

8/5714/9167/738/4893/10382/9821/9377/7316/4698/140775/5879/54205/1452/779/5704/7416/5621/5861/5173/9451/7480/805/7476/22943/10383/776/2521/7419/203228/847
01/4724/470
