# Supplementary material for: Exploring the diagnostic markers of essential tremor: A study based on machine learning algorithms
Source: Open Life Sci. 2023 Jun 22;18(1):20220622. doi: 10.1515/biol-2022-0622 (PMC10290283; doi:10.1515/biol-2022-0622)
Supplement: Supplementary Table 9 [file biol-2022-0622-sm10.pdf]

**Table S9:** Relationship between diagnostic markers and immune infiltrating cells by Pearson correlation analysis

| ID | Description | set Size | enrichment | NES | p-value | p.adjust | q-values | rank | leading_edcore_enrichment |
|---|---|---|---|---|---|---|---|---|---|
| hsa04144 | Endocytos | 239 | 0.437016 | 2.140103 | 1.04E-10 | 3.40E-08 | 1.85E-08 | 3608 | tags=34%, 8976/5869/57403/23362/10096/3799/101 |
| hsa04919 | Thyroid ho | 121 | 0.510087 | 2.280938 | 1.16E-09 | 1.90E-07 | 1.03E-07 | 4256 | tags=46%, 8648/5594/9969/3091/1499/8503/476/52 |
| hsa04071 | Sphingolip | 117 | 0.517483 | 2.29327 | 1.87E-09 | 2.04E-07 | 1.11E-07 | 4341 | tags=45%, 5594/8503/5295/5604/5515/3845/5579/5 |
| hsa04120 | Ubiquitin | 135 | 0.48243 | 2.172641 | 4.38E-09 | 3.58E-07 | 1.95E-07 | 4036 | tags=44%, 331/3093/10075/55284/55585/134111/84 |
| hsa04722 | Neurotrop | 116 | 0.500674 | 2.221639 | 7.21E-09 | 4.72E-07 | 2.57E-07 | 4250 | tags=47%, 5594/11108/10818/801/8503/5295/5604/ |
| hsa05132 | Salmonella | 244 | 0.406587 | 1.999811 | 1.11E-08 | 5.51E-07 | 3.00E-07 | 4135 | tags=35%, 23118/8976/5594/10810/10787/6416/269 |
| hsa04140 | Autophagy | 131 | 0.482556 | 2.167854 | 1.18E-08 | 5.51E-07 | 3.00E-07 | 4040 | tags=41%, 58528/5594/3091/8503/5295/64121/5604 |
| hsa05131 | Shigellosis | 211 | 0.41155 | 1.984666 | 5.28E-08 | 2.16E-06 | 1.17E-06 | 4040 | tags=32%, 58528/23118/8976/5594/3098/8936/8503 |
| hsa05010 | Alzheimer | 336 | 0.364195 | 1.849642 | 6.51E-08 | 2.28E-06 | 1.24E-06 | 3682 | tags=27%, 5594/324/8851/801/7976/1499/8503/379 |
| hsa03015 | mRNA sur | 92 | 0.517582 | 2.201807 | 6.96E-08 | 2.28E-06 | 1.24E-06 | 2805 | tags=37%, 80335/2935/23283/5515/11051/5528/237 |
| hsa03013 | RNA trans | 154 | 0.447744 | 2.073828 | 1.02E-07 | 2.87E-06 | 1.56E-06 | 4058 | tags=36%, 9513/51808/10762/26999/2332/11260/19 |
| hsa04068 | FoxO signa | 127 | 0.466818 | 2.095023 | 1.05E-07 | 2.87E-06 | 1.56E-06 | 3913 | tags=38%, 5594/4089/8503/5295/26260/5604/3845/ |
| hsa04012 | ErbB signa | 82 | 0.536711 | 2.245515 | 1.40E-07 | 3.53E-06 | 1.92E-06 | 4112 | tags=51%, 5594/6416/8503/6777/5295/5604/3845/5 |
| hsa05211 | Renal cell | 65 | 0.57186 | 2.282923 | 1.82E-07 | 4.24E-06 | 2.31E-06 | 3974 | tags=55%, 5594/54583/3091/8503/5295/5604/3845/ |
| hsa05017 | Spinocere | 137 | 0.451747 | 2.044222 | 2.67E-07 | 5.82E-06 | 3.16E-06 | 4599 | tags=39%, 23369/9698/8503/5295/3516/5579/5332/ |

| | | | | | | | | |
|---|---|---|---|---|---|---|---|---|
| hsa05205 | Proteoglyc | 200 | 0.404934 | 1.939206 | 2.88E-07 | 5.89E-06 | 3.21E-06 | 3894 tags=34%, 5594/10818/23365/3091/7976/1499/8503 |
| hsa05022 | Pathways | 439 | 0.330623 | 1.71998 | 3.80E-07 | 7.07E-06 | 3.85E-06 | 4786 tags=33%, 5594/324/8851/801/7976/1499/3799/383 |
| hsa04728 | Dopamine | 129 | 0.451369 | 2.033151 | 3.89E-07 | 7.07E-06 | 3.85E-06 | 4316 tags=41%, 9575/801/1385/2782/1386/3799/5515/27 |
| hsa04360 | Axon guid | 180 | 0.407171 | 1.910347 | 6.40E-07 | 1.10E-05 | 5.99E-06 | 4066 tags=33%, 659/5594/23365/7976/8503/5295/3845/5 |
| hsa04390 | Hippo sign | 153 | 0.424646 | 1.961239 | 8.62E-07 | 1.38E-05 | 7.51E-06 | 3541 tags=32%, 659/324/4089/7532/7976/1499/64398/55 |
| hsa04720 | Long-term | 66 | 0.547759 | 2.205387 | 8.86E-07 | 1.38E-05 | 7.51E-06 | 3739 tags=44%, 5594/801/5604/3845/5579/5332/5534/81 |
| hsa04141 | Protein pr | 161 | 0.425683 | 1.970471 | 1.11E-06 | 1.65E-05 | 8.96E-06 | 5497 tags=47%, 5887/7095/29978/5610/80267/10970/104 |
| hsa05120 | Epithelial c | 68 | 0.522105 | 2.109962 | 1.49E-06 | 2.11E-05 | 1.15E-05 | 4363 tags=44%, 6416/28964/527/5879/836/528/5781/523 |
| hsa04150 | mTOR sign | 150 | 0.418452 | 1.919312 | 1.87E-06 | 2.46E-05 | 1.34E-05 | 4410 tags=38%, 58528/5594/7976/8503/5295/64121/5604 |
| hsa05130 | Pathogeni | 192 | 0.402426 | 1.915428 | 1.88E-06 | 2.46E-05 | 1.34E-05 | 1745 tags=22%, 23118/8976/5594/10810/10787/23365/89 |
| hsa04261 | Adrenergic | 146 | 0.426142 | 1.945774 | 1.96E-06 | 2.47E-05 | 1.34E-05 | 4917 tags=47%, 5594/801/1385/476/1386/5515/2778/533 |
| hsa04550 | Signaling p | 135 | 0.429556 | 1.934519 | 2.08E-06 | 2.47E-05 | 1.34E-05 | 4517 tags=39%, 659/5594/324/5080/4090/84333/4089/79 |
| hsa05203 | Viral carci | 163 | 0.412868 | 1.917475 | 2.12E-06 | 2.47E-05 | 1.34E-05 | 2937 tags=30%, 5594/2957/7532/1385/8503/1386/6777/5 |
| hsa05110 | Vibrio cho | 48 | 0.594022 | 2.23348 | 2.43E-06 | 2.74E-05 | 1.49E-05 | 4984 tags=54%, 11014/2778/375/527/528/523/5566/60/5 |
| hsa04910 | Insulin sig | 133 | 0.425411 | 1.918956 | 2.57E-06 | 2.77E-05 | 1.51E-05 | 3913 tags=39%, 5594/5577/801/3098/8503/5295/5604/38 |
| hsa05225 | Hepatocel | 163 | 0.411298 | 1.910183 | 2.66E-06 | 2.77E-05 | 1.51E-05 | 3497 tags=33%, 5594/324/4089/7976/1499/8503/5295/56 |
| hsa04137 | Mitophagy | 65 | 0.542298 | 2.164906 | 2.71E-06 | 2.77E-05 | 1.51E-05 | 4208 tags=52%, 3091/3845/2309/9927/10370/8887/1457/ |
| hsa04810 | Regulation | 210 | 0.381589 | 1.841515 | 3.72E-06 | 3.55E-05 | 1.93E-05 | 3593 tags=30%, 8976/5594/324/10787/23365/2909/8936/ |

| | | | | | | | | |
|---|---|---|---|---|---|---|---|---|
| hsa04022 | cGMP-PKG | 159 | 0.411075 | 1.906487 | 3.73E-06 | 3.55E-05 | 1.93E-05 | 3577 tags=35%, 5594/801/2767/1385/476/1386/5604/533 |
| hsa04915 | Estrogen s | 127 | 0.430777 | 1.933275 | 3.80E-06 | 3.55E-05 | 1.93E-05 | 5440 tags=51%, 8648/5594/801/9568/1385/8503/1386/52 |
| hsa04540 | Gap juncti | 86 | 0.475525 | 2.009603 | 5.76E-06 | 5.15E-05 | 2.80E-05 | 4112 tags=43%, 5594/2767/203068/5604/2778/3845/5579 |
| hsa03040 | Spliceosom | 129 | 0.42296 | 1.905185 | 5.83E-06 | 5.15E-05 | 2.80E-05 | 4219 tags=40%, 10594/10291/23020/220988/51729/23450 |
| hsa04218 | Cellular se | 151 | 0.410609 | 1.889713 | 7.41E-06 | 6.38E-05 | 3.47E-05 | 3479 tags=30%, 204851/5594/801/8503/5295/5604/3845/ |
| hsa04917 | Prolactin s | 63 | 0.525888 | 2.086695 | 9.82E-06 | 8.24E-05 | 4.48E-05 | 3253 tags=41%, 5594/9306/8503/6777/5295/5604/3845/2 |
| hsa04310 | Wnt signal | 162 | 0.395819 | 1.836844 | 1.02E-05 | 8.34E-05 | 4.54E-05 | 2897 tags=27%, 324/1487/4089/7976/1499/5579/5332/23 |
| hsa04721 | Synaptic v | 77 | 0.489288 | 2.031657 | 1.42E-05 | 0.000113 | 6.17E-05 | 4718 tags=51%, 527/1173/528/6844/5864/6571/523/163/ |
| hsa04662 | B cell rece | 80 | 0.481223 | 2.010394 | 2.11E-05 | 0.000164 | 8.93E-05 | 3945 tags=35%, 5594/8503/5295/5604/3845/5579/5534/2 |
| hsa04961 | Endocrine | 52 | 0.53918 | 2.063177 | 2.23E-05 | 0.00017 | 9.23E-05 | 4703 tags=54%, 476/2778/5579/5332/1173/490/163/2605 |
| hsa05012 | Parkinson | 217 | 0.356025 | 1.724087 | 3.10E-05 | 0.000231 | 0.000125 | 4786 tags=35%, 801/3799/3831/203068/2778/5717/6389/ |
| hsa04114 | Oocyte me | 118 | 0.41163 | 1.825736 | 5.51E-05 | 0.000397 | 0.000216 | 3703 tags=36%, 5594/801/9126/7532/5604/5515/23291/5 |
| hsa04066 | HIF-1 signa | 105 | 0.429701 | 1.865268 | 5.69E-05 | 0.000397 | 0.000216 | 4112 tags=38%, 5594/3098/54583/3091/8503/5295/5604/ |
| hsa05210 | Colorectal | 85 | 0.459951 | 1.933074 | 5.70E-05 | 0.000397 | 0.000216 | 3593 tags=38%, 5594/324/26060/4089/1499/8503/5295/5 |
| hsa05223 | Non-small | 72 | 0.478067 | 1.958553 | 6.13E-05 | 0.000417 | 0.000227 | 3329 tags=39%, 5594/8503/3799/6777/5295/5604/3845/5 |
| hsa04935 | Growth ho | 116 | 0.40858 | 1.812988 | 7.14E-05 | 0.000476 | 0.000259 | 3722 tags=34%, 5594/2767/6416/1385/8503/1386/6777/5 |
| hsa05213 | Endometri | 58 | 0.526393 | 2.077432 | 7.44E-05 | 0.000487 | 0.000265 | 3253 tags=45%, 5594/324/1499/8503/5295/5604/3845/29 |
| hsa05163 | Human cyt | 215 | 0.348018 | 1.687296 | 7.94E-05 | 0.000509 | 0.000277 | 3370 tags=27%, 5594/23365/801/2767/1385/2782/1499/8 |
| hsa05230 | Central ca | 68 | 0.467875 | 1.890806 | 8.36E-05 | 0.000525 | 0.000286 | 3253 tags=35%, 5594/3098/3091/8503/5295/5604/3845/5 |
| hsa05014 | Amyotrop | 328 | 0.317425 | 1.612415 | 8.80E-05 | 0.000543 | 0.000295 | 5249 tags=35%, 10762/9782/3799/3831/203068/29978/22 |
| hsa05220 | Chronic m | 76 | 0.468203 | 1.942908 | 9.11E-05 | 0.000552 | 0.0003 | 3465 tags=38%, |

| | | | | | | | | | | |
|---|---|---|---|---|---|---|---|---|---|---|
| | | | | | | | | | | 5594/1487/4089/8503/6777/5295/5604/3 |
| hsa04024 | cAMP sign | 209 | 0.350907 | 1.690193 | 9.56E-05 | 0.000568 | 0.000309 | 3943 | tags=31%, | 5594/801/9568/1385/8503/476/5295/560 |
| hsa04960 | Aldosteron | 35 | 0.583985 | 2.073371 | 0.000104 | 0.000607 | 0.00033 | 4570 | tags=54%, | 5594/8503/476/5295/3845/5579/5291/51 |
| hsa05135 | Yersinia in | 132 | 0.396717 | 1.785504 | 0.000119 | 0.000685 | 0.000373 | 1715 | tags=20%, | 23118/8976/5594/23365/6416/8503/1009 |
| hsa01522 | Endocrine | 93 | 0.422083 | 1.799543 | 0.000173 | 0.000976 | 0.000531 | 4040 | tags=40%, | 5594/8503/5295/5604/2778/3845/5291/1 |
| hsa05418 | Fluid shea | 131 | 0.384074 | 1.725427 | 0.000178 | 0.000988 | 0.000537 | 4135 | tags=34%, | 659/801/6416/1499/8503/5295/5879/529 |
| hsa05100 | Bacterial i | 69 | 0.464189 | 1.88331 | 0.000207 | 0.00113 | 0.000614 | 1512 | tags=29%, | 8976/8936/1499/8503/10096/5295/10097 |
| hsa05161 | Hepatitis B | 160 | 0.369687 | 1.714174 | 0.000217 | 0.001153 | 0.000627 | 4176 | tags=34%, | 23118/5594/4089/6416/1385/8503/1386/ |
| hsa04921 | Oxytocin s | 150 | 0.366562 | 1.681305 | 0.000222 | 0.001153 | 0.000627 | 4142 | tags=39%, | 5594/801/5604/2778/3845/5579/5332/55 |
| hsa05168 | Herpes sim | 480 | 0.281298 | 1.473377 | 0.000222 | 0.001153 | 0.000627 | 4992 | tags=31%, | 23118/57786/10838/8503/7743/388566/7 |
| hsa05214 | Glioma | 74 | 0.456226 | 1.886227 | 0.000236 | 0.001208 | 0.000657 | 3313 | tags=38%, | 5594/801/8503/5295/5604/3845/5579/52 |
| hsa05221 | Acute mye | 65 | 0.47213 | 1.884789 | 0.00026 | 0.001307 | 0.000711 | 3253 | tags=35%, | 5594/8503/6777/5295/5604/3845/5291/1 |
| hsa04512 | ECM-recep | 87 | -0.3965 | -1.78709 | 0.000277 | 0.001374 | 0.000748 | 5885 | tags=51%, | 3676/960/3688/3161/7148/3371/3672/66 |
| hsa05165 | Human pa | 327 | 0.303454 | 1.541849 | 0.000328 | 0.001602 | 0.000872 | 4410 | tags=31%, | 5594/324/1385/7976/1499/8503/64398/5 |

| | | | | | | | | |
|---|---|---|---|---|---|---|---|---|
| hsa05152 | Tuberculo | 175 | 0.345203 | 1.623824 | 0.000374 | 0.001764 | 0.00096 | 4379 tags=29%, 5594/23365/801/4801/1385/5869/5534/5 |
| hsa04152 | AMPK sign | 117 | 0.388908 | 1.72348 | 0.000376 | 0.001764 | 0.00096 | 3432 tags=32%, 1385/8503/1994/5295/5515/51719/5528/ |
| hsa04070 | Phosphati | 93 | 0.408671 | 1.74236 | 0.00038 | 0.001764 | 0.00096 | 1770 tags=20%, 8760/801/8503/5295/5579/5332/22876/8 |
| hsa04010 | MAPK sign | 286 | 0.310755 | 1.562452 | 0.000388 | 0.001764 | 0.00096 | 4135 tags=28%, 23118/57551/5594/5494/6416/1386/5604 |
| hsa01521 | EGFR tyros | 79 | 0.442603 | 1.840099 | 0.000388 | 0.001764 | 0.00096 | 4112 tags=39%, 5594/8503/5295/5604/3845/5579/2932/5 |
| hsa04926 | Relaxin sig | 127 | 0.373253 | 1.675113 | 0.000411 | 0.00184 | 0.001001 | 4045 tags=37%, 5594/6416/1385/2782/8503/1386/5295/5 |
| hsa05020 | Prion dise | 244 | 0.321111 | 1.579393 | 0.000418 | 0.001848 | 0.001005 | 1852 tags=18%, 5594/4684/1385/8503/1386/3799/3831/5 |
| hsa05031 | Amphetam | 67 | 0.455674 | 1.837842 | 0.000465 | 0.002014 | 0.001096 | 2132 tags=30%, 801/1385/1386/2778/5579/5534/3066/81 |
| hsa05142 | Chagas dis | 98 | 0.402669 | 1.721972 | 0.000468 | 0.002014 | 0.001096 | 4045 tags=33%, 5594/2767/6416/8503/5295/5515/2778/5 |
| hsa05167 | Kaposi sar | 186 | 0.342175 | 1.620088 | 0.000489 | 0.002078 | 0.00113 | 4135 tags=30%, 5594/801/6416/1385/3091/2782/1499/85 |
| hsa05016 | Huntingto | 275 | 0.308513 | 1.543735 | 0.000505 | 0.002117 | 0.001152 | 4786 tags=31%, 5430/1385/3799/3831/203068/5332/3066 |
| hsa04966 | Collecting | 26 | 0.598216 | 1.958971 | 0.000528 | 0.002184 | 0.001188 | 4364 tags=50%, 527/528/523/534/535/9114/155066/8992 |
| hsa05160 | Hepatitis C | 153 | 0.355205 | 1.640521 | 0.000536 | 0.002193 | 0.001193 | 3313 tags=29%, 5594/7532/1499/8503/5295/5604/5515/3 |
| hsa04666 | Fc gamma | 92 | 0.410965 | 1.748254 | 0.000592 | 0.002368 | 0.001288 | 2190 tags=24%, 5594/10810/8936/8503/10096/5295/5604 |
| hsa04929 | GnRH secr | 62 | 0.458324 | 1.823751 | 0.000594 | 0.002368 | 0.001288 | 3253 tags=32%, 5594/2767/9568/8503/5295/5604/3845/5 |
| hsa04660 | T cell rece | 97 | 0.402719 | 1.720854 | 0.000623 | 0.002454 | 0.001335 | 3959 tags=33%, 5594/8503/5295/5604/3845/5062/5534/2 |
| hsa05215 | Prostate c | 95 | 0.406981 | 1.742194 | 0.00063 | 0.002454 | 0.001335 | 4040 tags=37%, 5594/1385/1499/8503/5295/5604/3845/2 |
| hsa04730 | Long-term | 57 | 0.468864 | 1.845725 | 0.000642 | 0.002469 | 0.001343 | 4112 tags=42%, 5594/2767/2781/5604/5515/2778/3845/5 |
| hsa04916 | Melanoge | 99 | 0.401136 | 1.714573 | 0.000712 | 0.002707 | 0.001473 | 5050 tags=43%, 5594/801/1385/7976/1499/5604/2778/38 |
| hsa04973 | Carbohydr | 43 | 0.504023 | 1.867328 | 0.000734 | 0.00276 | 0.001501 | 3253 tags=35%, 3098/8503/476/5295/5579/5332/5291/10 |
| hsa04725 | Cholinergi | 113 | 0.388339 | 1.704385 | 0.000765 | 0.002844 | 0.001547 | 4112 tags=35%, |

| | | | | | | | | |
|---|---|---|---|---|---|---|---|---|
| | | | | | | | | 5594/2767/1385/2782/8503/5295/5604/3 |
| hsa04664 | Fc epsilon | 63 | 0.456051 | 1.809587 | 0.000806 | 0.002962 | 0.001611 | 3253 tags=32%, 5594/6416/8503/5295/5604/3845/5879/5 |
| hsa05206 | MicroRNA | 245 | 0.309466 | 1.522817 | 0.000873 | 0.003171 | 0.001725 | 3253 tags=23%, 659/5594/324/7976/8503/5295/5604/384 |
| hsa05235 | PD-L1 exp | 89 | 0.3992 | 1.692361 | 0.000905 | 0.003253 | 0.00177 | 4135 tags=35%, 5594/3091/8503/5295/5604/3845/5534/5 |
| hsa04930 | Type II dia | 44 | 0.507575 | 1.881898 | 0.001035 | 0.003677 | 0.002 | 3253 tags=36%, 5594/3098/8503/5295/5291/122809/5581 |
| hsa04625 | C-type lect | 101 | 0.388912 | 1.670805 | 0.00107 | 0.003761 | 0.002046 | 3943 tags=30%, 5594/23365/801/8503/5295/1540/3845/5 |
| hsa05166 | Human T-c | 213 | 0.324703 | 1.567184 | 0.001115 | 0.003879 | 0.00211 | 3972 tags=28%, 331/5594/4089/4801/6416/1385/8503/13 |
| hsa04933 | AGE-RAGE | 98 | 0.387356 | 1.656488 | 0.00123 | 0.004235 | 0.002304 | 4040 tags=31%, 5594/4089/8503/6777/5295/3845/5579/5 |
| hsa04934 | Cushing sy | 152 | 0.347049 | 1.599827 | 0.00128 | 0.004362 | 0.002373 | 4410 tags=32%, 5594/324/2767/1385/7976/1499/1386/56 |
| hsa04110 | Cell cycle | 124 | 0.368213 | 1.650029 | 0.0013 | 0.004382 | 0.002384 | 3506 tags=30%, 4089/9126/7532/5885/3066/2932/7529/8 |
| hsa05170 | Human im | 201 | 0.322882 | 1.547868 | 0.001497 | 0.004994 | 0.002717 | 4135 tags=28%, 23118/5594/801/2767/2782/8503/5295/5 |
| hsa04062 | Chemokin | 172 | 0.334701 | 1.565267 | 0.001761 | 0.005818 | 0.003165 | 3703 tags=26%, 5594/2782/8503/6777/5295/5604/2931/3 |
| hsa05219 | Bladder ca | 39 | 0.495038 | 1.80412 | 0.001942 | 0.006351 | 0.003455 | 3593 tags=41%, 5594/5604/3845/4193/1029/9252/6714/6 |
| hsa05226 | Gastric can | 146 | 0.342775 | 1.56512 | 0.002018 | 0.006535 | 0.003555 | 4483 tags=34%, 5594/324/4089/7976/1499/8503/5295/56 |

| | | | | | | | | |
|---|---|---|---|---|---|---|---|---|
| hsa05212 | Pancreatic | 76 | 0.411814 | 1.708908 | 0.00214 | 0.006861 | 0.003732 | 3682 tags=33%, 5594/4089/8503/5295/5604/3845/10928/ |
| hsa04014 | Ras signali | 219 | 0.3116 | 1.507218 | 0.002343 | 0.00744 | 0.004047 | 3718 tags=27%, 5594/801/5869/2782/8503/5295/5604/38 |
| hsa05231 | Choline m | 94 | 0.395482 | 1.690703 | 0.002501 | 0.007792 | 0.004239 | 3361 tags=30%, 5594/10810/8936/3091/8503/5295/5604/ |
| hsa04912 | GnRH sign | 90 | 0.386301 | 1.639728 | 0.002502 | 0.007792 | 0.004239 | 3399 tags=31%, 5594/801/2767/6416/5604/2778/3845/55 |
| hsa04530 | Tight junct | 158 | 0.332655 | 1.543506 | 0.0026 | 0.00802 | 0.004363 | 3959 tags=28%, 10096/64398/5515/1741/10097/51762/58 |
| hsa04610 | Compleme | 73 | -0.37361 | -1.61995 | 0.002931 | 0.008956 | 0.004872 | 7513 tags=47%, 7448/727/3426/716/2160/717/730/710/3 |
| hsa04380 | Osteoclast | 125 | 0.354238 | 1.590116 | 0.002965 | 0.008976 | 0.004883 | 4135 tags=29%, 23118/5594/1385/8503/5295/5604/1540/ |
| hsa04210 | Apoptosis | 130 | 0.33745 | 1.516154 | 0.003103 | 0.009308 | 0.005064 | 4176 tags=30%, 331/5594/8503/142/5295/5604/3845/529 |
| hsa04213 | Longevity | 61 | 0.427461 | 1.696355 | 0.00337 | 0.010018 | 0.00545 | 5232 tags=49%, 8503/5295/3845/3066/5291/2309/23411/ |
| hsa04211 | Longevity | 87 | 0.384139 | 1.621217 | 0.00343 | 0.010106 | 0.005498 | 3703 tags=34%, 26060/1385/8503/1386/5295/3845/5291/ |
| hsa03010 | Ribosome | 128 | -0.31228 | -1.49434 | 0.003573 | 0.01035 | 0.00563 | 4906 tags=36%, 64960/6137/6224/6143/64983/6165/2197 |
| hsa00511 | Other glyc | 16 | -0.61159 | -1.80961 | 0.003603 | 0.01035 | 0.00563 | 3195 tags=38%, 10825/2720/64772/4126/3073/4123 |
| hsa04510 | Focal adhe | 196 | 0.310257 | 1.47708 | 0.003608 | 0.01035 | 0.00563 | 3593 tags=26%, 394/331/5594/2909/1499/8503/5295/560 |
| hsa04145 | Phagosom | 146 | 0.332164 | 1.516667 | 0.003809 | 0.01083 | 0.005892 | 4104 tags=27%, 5869/203068/527/8411/5879/10382/528/ |
| hsa05218 | Melanoma | 70 | 0.40636 | 1.651313 | 0.00434 | 0.012233 | 0.006655 | 3313 tags=33%, 5594/8503/5295/5604/3845/5291/5728/1 |
| hsa05224 | Breast can | 144 | 0.331032 | 1.508376 | 0.004396 | 0.012285 | 0.006683 | 4483 tags=33%, 8648/5594/324/7976/1499/8503/5295/56 |
| hsa03018 | RNA degra | 74 | 0.399604 | 1.652127 | 0.004573 | 0.012672 | 0.006894 | 4771 tags=38%, 167227/246175/1656/29883/23019/694/5 |
| hsa00590 | Arachidon | 56 | -0.39796 | -1.64048 | 0.004914 | 0.013502 | 0.007346 | 6459 tags=43%, 8644/1557/64600/283748/5319/4051/532 |
| hsa04371 | Apelin sign | 131 | 0.336861 | 1.513325 | 0.005214 | 0.01411 | 0.007676 | 3913 tags=33%, 5594/801/4089/2782/5604/3845/5332/42 |
| hsa04971 | Gastric aci | 74 | 0.39711 | 1.641818 | 0.005221 | 0.01411 | 0.007676 | 4256 tags=38%, 801/476/2778/5579/5332/818/808/107/5 |
| hsa04350 | TGF-beta s | 91 | 0.366361 | 1.55707 | 0.005277 | 0.014143 | 0.007694 | 3465 tags=27%, |

| | | | | | | | | | | |
|---|---|---|---|---|---|---|---|---|---|---|
| | | | | | | | | | | 659/5594/285704/4090/4089/5515/5518/ |
| hsa05415 | Diabetic ca | 173 | 0.31346 | 1.46662 | 0.005434 | 0.014446 | 0.007859 | 4112 | tags=28%, | 2673/8503/142/5295/5579/5332/2932/58 |
| hsa04015 | Rap1 signa | 202 | 0.30228 | 1.449849 | 0.005512 | 0.014534 | 0.007907 | 4135 | tags=28%, | 5594/801/1499/8503/5295/5604/2778/38 |
| hsa04726 | Serotoner | 111 | 0.344009 | 1.50868 | 0.005629 | 0.014726 | 0.008011 | 4316 | tags=32%, | 2562/5594/2782/5604/2778/3845/5579/5 |
| hsa04370 | VEGF signa | 58 | 0.43922 | 1.733403 | 0.006039 | 0.015673 | 0.008526 | 3253 | tags=34%, | 5594/8503/5295/5604/3845/5579/5534/5 |
| hsa04520 | Adherens j | 66 | 0.406066 | 1.634903 | 0.006195 | 0.015951 | 0.008677 | 2676 | tags=30%, | 8976/5594/10810/4089/8936/1499/11717 |
| hsa04650 | Natural kil | 116 | 0.341634 | 1.515928 | 0.007481 | 0.019111 | 0.010397 | 3253 | tags=23%, | 5594/8503/5295/5604/3845/5579/5534/5 |
| hsa04924 | Renin secr | 65 | 0.402607 | 1.607247 | 0.007703 | 0.019443 | 0.010577 | 4295 | tags=32%, | 801/1385/2778/5332/5534/5532/808/134 |
| hsa04613 | Neutrophi | 103 | 0.357891 | 1.544363 | 0.007729 | 0.019443 | 0.010577 | 2190 | tags=22%, | 5594/8503/1183/5295/5604/5579/5332/3 |
| hsa04727 | GABAergi c | 88 | 0.359926 | 1.523984 | 0.008543 | 0.021325 | 0.011601 | 5065 | tags=44%, | 2562/9568/2782/57468/5579/2561/2566/ |
| hsa00670 | One carbo | 18 | -0.54883 | -1.70299 | 0.00896 | 0.022196 | 0.012075 | 2889 | tags=33%, | 160428/10840/4524/10797/4548/275 |
| hsa05034 | Alcoholism | 104 | 0.358466 | 1.552948 | 0.009628 | 0.023671 | 0.012877 | 3703 | tags=31%, | 5594/801/1385/2782/1386/5604/2778/38 |
| hsa04710 | Circadian r | 30 | 0.502014 | 1.712101 | 0.009822 | 0.02397 | 0.01304 | 3913 | tags=50%, | 9575/1385/23291/6095/26224/79365/140 |
| hsa02010 | ABC transp | 44 | -0.40941 | -1.60369 | 0.011483 | 0.027815 | 0.015132 | 2143 | tags=36%, | 6890/1080/225/85320/1672/23460/6833/ |

| | | | | | | | | |
|---|---|---|---|---|---|---|---|---|
| hsa04611 | Platelet ac | 118 | 0.328645 | 1.457664 | 0.012281 | 0.029529 | 0.016065 | 1715 tags=20%, 5594/23365/2909/8503/5295/2778/5332/ |
| hsa04928 | Parathyroi | 102 | 0.342973 | 1.484251 | 0.014883 | 0.035524 | 0.019326 | 1715 tags=21%, 5594/2767/1385/1386/5604/2778/5579/5 |
| hsa04925 | Aldosteron | 96 | 0.342132 | 1.46575 | 0.016114 | 0.038182 | 0.020772 | 2505 tags=24%, 801/2767/1385/476/1386/2778/5579/533 |
| hsa05216 | Thyroid ca | 37 | 0.449281 | 1.623771 | 0.017734 | 0.04172 | 0.022697 | 3000 tags=35%, 5594/1499/5604/3845/8031/8030/673/55 |
| hsa05169 | Epstein-Ba | 197 | 0.288744 | 1.375682 | 0.017948 | 0.041922 | 0.022806 | 4589 tags=29%, 23118/6416/8503/5295/3516/79685/5610 |
| hsa05032 | Morphine | 88 | 0.342678 | 1.450955 | 0.019566 | 0.045377 | 0.024686 | 6245 tags=49%, 2562/9568/2782/2778/5579/2561/2566/1 |
| hsa04974 | Protein dig | 97 | -0.30876 | -1.42182 | 0.020464 | 0.047124 | 0.025636 | 4899 tags=35%, 1299/6505/91522/1360/1803/1284/1302/ |
| hsa05146 | Amoebiasi | 89 | 0.341024 | 1.445733 | 0.020897 | 0.047687 | 0.025943 | 1320 tags=16%, 2767/5869/8503/5295/2778/5579/5332/5 |
| hsa04962 | Vasopressi | 44 | 0.418783 | 1.55269 | 0.021 | 0.047687 | 0.025943 | 2079 tags=32%, 1385/5869/2778/140735/6844/51164/587 |
| hsa00970 | Aminoacyl | 24 | -0.4935 | -1.67479 | 0.021624 | 0.048766 | 0.02653 | 4302 tags=42%, 23395/55157/23438/51091/80222/10667/ |

core_enrichment

93/28964/6456/8027/10097/375/22841/84440/55737/8411/112936/128866/26286/64744/10938/9829/1173/137492/51652/11059/3800/5878/163/
95/5604/7067/3845/5579/5332/3066/2932/5291/5170/10000/4193/5469/5566/1827/60/3685/71/25942/10499/6714/8202/9611/1387/478/5595/52
332/5528/5879/5291/253782/5581/8560/5728/5520/1509/5170/10000/5518/10672/81537/134/387/5601/9475/55844/5529/5516/5595/5527/9517/
52/7428/25898/23291/10055/7329/10273/4591/8554/54926/10054/9320/55958/11059/4193/7328/1642/7326/51465/867/7317/83737/27339/8065
3845/2932/5879/5291/1399/814/5781/5906/2309/1398/818/808/5170/10000/9500/387/2885/5601/4908/9252/673/57498/5595/816/207/4893/566
99/5869/1499/10096/3799/3831/203068/3839/5604/3840/10097/10640/375/5879/5291/10382/140735/836/7280/10376/257397/51164/7277/1434
/5515/3845/5291/5861/116442/5728/1509/5170/10000/10670/1965/5566/5601/3146/7879/26100/140775/5516/8878/29110/5595/51100/83452/98
/10096/5295/64121/2931/5332/23291/10097/375/2932/5879/5291/1399/5581/92610/2309/257397/11335/1398/10000/4193/10670/63916/387/560
9/3831/5295/203068/5604/3845/5332/5610/5534/2932/5291/5717/6389/291/351/10382/836/5532/10313/7280/10376/57142/4719/2903/7277/380
08/10921/5520/9887/5500/26019/5518/5501/53918/8731/9939/55844/340529/5529/53981/5516/26986/29101/51585/5527/22916/5976/4440/147
64/7329/1982/10921/3837/1983/1981/6613/6612/5903/26019/9972/1965/1977/10248/9939/11171/340529/8672/59343/26986/10605/23636/8666
7874/5291/901/5728/2309/5170/23411/10000/4193/80854/2885/5601/1027/1387/673/5595/9456/1032/207/4893/664/5563/5565/8660/6655/5296
579/5062/2932/5291/1399/2002/1398/818/10000/2885/867/5601/1027/6714/8440/673/4690/5595/816/207/27/4893/817/6655/5296/5605/2066/19
7428/5062/5879/5291/1399/5781/5906/1398/10000/2885/9915/1387/673/5595/207/4893/8453/4233/6655/5296/7030/5605/5290/112399/5058/25
5291/6310/5717/291/342371/6095/2903/11047/5718/10000/5701/5601/5708/5707/26100/7416/7417/6667/116443/9821/207/10939/5713/9706/90
/5295/5604/3845/5579/5879/5291/4659/2002/836/5781/5500/818/5170/10000/4193/5501/6383/387/2885/27250/5566/867/60/8324/3685/9475/7
1/203068/5604/3845/5579/5332/5534/79139/2932/5879/6310/5717/6389/5861/291/342371/351/116442/10382/836/5532/7280/10376/4719/2903/
78/2931/5579/5332/5528/2932/5532/5520/5500/6571/2903/3800/818/808/10000/5518/5501/5566/3760/5601/408/55844/5529/5516/5530/5527/8
062/5534/2932/5879/5291/64101/5532/5781/55740/818/5170/1072/1948/387/9475/6091/1808/56896/3983/6714/8440/4690/5595/5530/8482/544
15/1741/23291/2932/5520/5500/7529/5518/5501/92597/60/8324/71/55844/5584/5516/7534/8321/7533/122786/4087/84612/10971/657/999/5522
4/5532/5906/5500/2903/818/808/5501/107/5566/1387/673/5595/5530/816/4893/817/5605/5499/5567/2033/2911
27/51128/79139/10273/11231/10802/10905/3998/7415/5034/80331/10484/1965/6400/7326/51465/5601/5886/27102/29979/55432/56893/267/73
/5601/102/534/6714/4067/535/9114/537/4233/155066/8992/526/1956/5058/5970/3725/50617/4792/529/525/3579/51606
/3845/51719/5579/2932/5291/528/5728/523/5170/10000/10670/8649/387/2885/1977/8324/534/220441/8321/673/5595/1975/207/253260/4893/8
36/26999/10096/203068/10097/5062/375/10427/4691/5879/10802/5861/10382/836/7280/5781/10376/257397/7277/10383/10672/387/5601/60/10
2/5528/5520/5500/490/818/808/10000/5518/5501/107/5566/183/9254/55844/9252/5529/5516/59284/10369/478/5595/781/5527/785/784/816/207
76/1499/8503/5295/5604/7994/3845/2932/5291/10000/2885/55183/8324/648/8321/7547/5595/4087/3716/3572/657/207/93/4893/3717/7473/102
295/3516/3845/5610/7874/3066/5879/5291/1654/836/7529/3190/1108/4193/1642/387/2885/5566/2961/1027/1029/22938/6714/7534/4067/1387/
34/71/535/9114/537/155066/8992/526/29927/5567/50617/529/525/51606/9414/115/6558/10945
45/2932/5291/1399/122809/2002/2645/5573/5500/1398/808/5170/10000/5501/2885/5566/867/5601/1977/10211/5575/5584/2319/673/5595/207/
04/3845/5579/2932/5291/2002/5728/55193/10000/6598/7296/2885/55274/1029/60/8324/71/6595/8289/8321/673/9817/5595/4087/3481/114112/
5601/1459/7879/8878/6714/6667/29110/4077/55288/84749/65018/7316/4893/664/22808/8678/7030/23710/9101/64786/5970/3725/22800/10133/
26999/8503/10096/5295/5604/28964/3845/10097/5062/5879/5291/8396/1399/4659/5500/55740/1398/1072/5501/10672/387/60/10152/3685/9475

2/5534/4209/4659/291/5581/5532/150/4205/27345/5500/490/808/10000/5501/10672/107/134/387/1910/9475/7416/7417/478/5595/5530/2977/12
95/5604/2778/3845/5332/5291/1509/3880/808/10000/107/2885/5566/3760/3885/10499/6714/8202/6667/5595/207/3881/4893/6655/3858/5296/5
/5332/10382/7280/10746/10376/7277/10383/107/2885/5566/10381/6714/5595/2977/56034/4893/5598/6655/153/5605/1956/2773/2771/1950/556
/3183/3190/6434/10569/10523/988/25949/10907/10929/10992/9939/9092/9785/5093/22938/9416/27339/10285/1665/5356/22916/6429/11325/5
23291/5534/5291/291/5532/5728/132660/2309/5500/808/23411/10000/4193/5501/10114/5810/1029/8878/7416/7417/898/5595/5530/4087/5928/
932/5291/122809/2645/2309/10000/2885/5601/6714/30837/5595/207/4893/9655/6655/3717/5296/5605/5290
291/5534/2932/5879/5532/818/1457/1501/387/5566/5601/1459/8324/340419/55366/9475/59343/8321/1387/5530/7091/6422/57680/6477/816/22
26052/1212/534/1213/4905/535/6812/9114/6857/10815/160/339302/155066/6511/6506/8992/526/6616/112755/161/440279/6804/57030/50617/8
932/5879/5291/5532/10000/2885/4067/5595/5530/207/4893/6655/5296/930/5605/4793/5290/5970/3725/4792/10990
2/1212/5566/3817/1213/478/6546/491/482/160/480/8766/493/161/5567/6543/7421/5582/481/2776/115
291/10382/836/7280/10376/6571/4719/7277/9927/3800/818/11047/808/5718/10383/5701/1965/5566/7326/51465/5601/6622/10381/7317/5708/5
528/5534/5532/5500/7529/8243/818/808/5518/5501/107/5566/80315/5529/5516/7534/26271/898/996/7533/5595/5530/25847/5527/10971/816/8
5579/7428/5291/818/10000/1977/3945/1027/1387/5595/5213/816/207/226/2023/817/8453/2597/5296/2026/5605/1956/5290/230/112399/229/19
604/3845/2932/5879/5291/836/10000/387/2885/5601/673/5595/4087/207/4893/6655/5366/10297/5296/5605/5898/1956/5290/7046/1950
579/5291/2309/3800/5170/10000/2885/1029/673/5595/207/4893/4233/6655/5296/6789/5605/1956/5290/5925/3798
295/5604/2778/3845/5579/5332/2932/5291/1399/1398/10000/107/2885/5566/5601/1387/5595/207/4893/4214/8660/6655/3717/5296/6752/5605/
32/5291/2002/5728/2309/5170/10000/2885/673/5595/207/4893/999/6655/10297/5296/5605/1956/5290
503/1386/5295/5604/2778/3845/5579/5332/5534/2932/5879/5291/1399/2002/836/5532/1398/808/10000/4193/10672/107/387/2885/5566/1029/3
291/5728/2645/10000/3945/5595/5213/2744/5315/207/4893/4233/3417/5296/5605/1956/5290
0988/5534/79139/5879/5717/6389/5861/116442/10382/836/5532/7280/10376/4719/2903/51164/7277/5903/7415/3800/11047/5718/10383/9972/5
845/3066/5291/1399/5781/1398/10000/4193/2885/867/1027/1029/673/5595/207/4893/6655/5296/5605/5290/5925/7046
4/2778/5879/5291/4659/814/5906/5500/2903/490/818/808/10000/5501/107/10021/134/387/5566/5601/51/9475/1387/673/478/5595/116443/816/
70/478/5595/482/480/5296/3667/5290/9351/5582/481/3291
6/5295/5604/10097/2932/5879/5291/1399/1398/10000/63916/5585/387/5601/60/382/9475/71/6714/29110/5595
0000/4193/107/5469/2885/5566/5601/1027/1029/6714/8202/9611/6667/673/5595/207/4893/10683/6655/5296/5605/1956/5290/5925/4313/3714/
1/6613/6612/4205/808/10000/6383/387/5601/60/3685/71/8878/6714/9817/51588/657/445/207/7341/93/4208/5563/5598/4880/5296/5590/5290/4
/5879/5291/1399/1398/26052/63916/387/1212/867/60/71/1213/6714
6777/5295/5604/3845/5579/5291/1654/2002/836/7529/10000/1642/2885/5601/6714/7534/1387/898/29110/673/5595/3716/10971/3454/8900/537
34/4659/2002/814/5532/5500/818/808/5501/107/387/5566/3760/1827/60/9254/9475/71/6714/59284/10369/5595/5530/781/785/784/2977/816/48
644/5295/57343/7559/5610/5291/57693/390980/162239/836/7738/5781/5500/163227/10172/7695/155054/10000/5451/51427/5501/7556/163051
91/814/5728/818/808/10000/4193/2885/1029/673/5595/816/207/4893/817/6655/5296/5605/1956/5290/5925
0000/2885/862/3728/673/5595/1050/8900/4353/207/4893/6655/5296/8864/5605/5290
96/1299/7450/1284/948/3914/1293/1292/1282/3909/1298/7057/3339/51206/1287/80144/3691/8515/341640/2815/8516/3910/22801/3695/1291/3
295/5604/3516/5515/2778/3845/1741/5610/5528/3066/527/2932/5291/836/528/5728/5520/523/1108/10000/4193/5518/2885/5566/1027/8324/36

27/8411/836/5532/1509/5878/818/808/10000/3313/1054/387/5601/7879/6714/1387/535/5595/5530/3716/5868/9114/816/537/207/817/3717/3920
5291/3156/51552/5520/2309/5170/23411/10000/5518/51094/55844/5529/5516/10890/5213/5527/5862/8900/207/5522/5563/5565/8660/5523/799
526/5291/8396/4952/5728/8867/1040/808/253430/9807/3612/22908
/3845/5579/5062/5534/5879/1399/2005/2002/836/5532/10746/5906/1845/1849/1398/10000/3727/8649/2885/5566/5601/4908/408/9254/9252/59
291/5728/2309/10000/2885/1977/6714/673/5595/3716/56034/207/4893/4233/6655/3717/5296/5605/1956/5290/1950/2549/596/5582
604/2778/3845/5332/5291/10000/107/2885/5566/5601/408/1910/6714/5595/4087/207/4893/2786/6655/5296/5605/6013/5590/2790/1956/2783/5
295/203068/5534/2932/5879/5291/5717/6389/291/10382/836/5532/7280/10376/4719/2903/7277/3800/11047/5718/10383/1457/5701/1965/5566/

4/5532/5500/6571/2903/818/808/23411/5501/5566/5530/116443/816
332/5291/5520/10000/5518/107/5601/55844/5516/5595/4087/207/3827/5522/5296/5290/2773/2775/7046/2771/5970/3725/919/4792/2770
03/5295/5604/3845/5610/5534/2932/5879/5291/836/5532/808/10000/5601/1827/6714/4067/1387/29110/5595/5530/7316/3716/3572/3454/207/4
/5431/5717/6389/291/10382/836/1173/7280/10376/4719/51164/7277/3800/11047/5718/163/10383/5701/1212/5601/7019/10381/5708/1639/5707
/526/50617/529/525/1188 845/10197/5610/2932/5291/8554/836/5520/7529/10000/5518/1965/2885/55844/27102/5516/7534/29110/673/9071/7533/5595/3716/10971/3454/
/5579/10097/5879/5291/1399/5581/4082/1398/10000/1072/382/4067/5595/65108/207 579/5332/5291/10000/3760/348980/408/5595/207/4893/5296/5605/5290
932/5291/5532/5170/10000/387/2885/5601/8440/4690/5595/5530/207/4893/6655/5296/5605/4793/5290/5058/5970/3725/919/4792/5609
932/5291/5728/5170/10000/4193/2885/1027/1387/898/673/5595/6935/56034/207/4893/6655/5296/5605/1956/5290/5925/1950/5970/2033/3326/
579/5332/5518/10672/5516/4067/673/5595/2977/4893/5605/2773/2775/2771/2911/2770/5582
45/5579/5332/2932/818/808/107/5566/8324/1910/8321/1387/5595/816/4893/817/7473/7476/5605/2773/2775/11211/2771/5567/2033/7474/2770
000/478/207/482/80834/480/5296/5290

845/5579/5332/5291/814/818/10000/107/5566/3760/5595/816/207/4893/3768/3761/817/2786/3717/5296/2790/2783/5290/2773/60482/2775/277
291/5170/10000/2885/5601/4067/5595/207/4893/6655/5296/5605/5290
5/5579/7329/3066/5291/1399/5581/836/5728/4082/3190/1398/1591/23411/4193/387/2885/27250/1027/1029/9252/648/23405/1387/898/5595/74
291/5532/5728/5781/10000/1457/1459/5595/5530/3716/207/4893/3717/5296/5605/4793/1956/5290/1950/5970/3725/919/4792/5608
/2645/5601/5595/5315/8660/5296/5590/3667/5290
534/5291/5532/5781/808/10000/4193/387/5601/6714/5595/5530/207/4893/9261/22808/5296/5290/5058/5970/3725/22800/4792
86/6777/5295/5604/3845/5534/5291/291/2005/2002/5532/5728/115650/10000/107/5566/5601/1029/7416/1387/7417/898/996/5595/5530/4087/3
332/5879/5291/5581/836/10000/5601/1027/183/5595/4087/207/4893/3717/5296/5590/5290/4313/7046/5970/3725/1278/596
04/2778/5332/2932/5906/57552/818/5929/107/5566/5087/1027/1029/8324/183/8321/6667/898/673/5595/816/817/7473/10297/7476/5605/9070/1
243/10735/4193/4173/1027/1029/4172/5000/7534/1387/898/996/7533/4087/25847/1032/10971/8900/7027/5591/8454/4175/10459/4171/7531/59
604/3845/5579/23291/5062/5534/5879/5291/164/1399/836/5532/1398/808/10000/1072/1642/5601/8065/29110/5595/5530/8450/207/4893/8454/
845/5579/5332/2932/5879/5291/1399/5906/2309/1398/10000/107/387/2885/5566/408/9475/6714/4067/673/5595/207/4893/2786/6655/3717/529
73/5595/4893/999/5605/1956/5925/4313/1950
04/3845/2932/5291/10000/2885/3728/1027/8324/8321/898/673/5595/4087/207/4893/2254/999/4233/4040/6655/1452/7473/10297/5296/7476/56

5879/5291/10000/5601/1029/673/5595/4087/3716/207/5296/5898/1956/5290/5925/7046/1950/5970
45/5579/10928/5062/5879/5291/2002/5781/5906/2903/8036/5878/808/10000/387/2885/5566/5601/4908/382/29110/5595/3481/5868/81579/5603
3845/5579/8526/5879/5291/5170/10000/2885/5601/6667/5595/56034/207/4893/6655/23446/5296/5605/1956/5290/60482
79/5332/2002/10746/818/808/107/2885/5566/5601/6714/5595/816/4893/817/4214/5598/6655/5605/1956/4313
79/5581/5520/10376/5906/7277/5518/387/5566/5601/60/9475/71/55844/5584/5516/6714/9071/54566/7430/84612/4628/5522/3308/5563/4214/5
689/9002/624/2151/5627/729/735/5329/7450/715/5054/2162/629/3684/5265/11326/1378/3075/5345/3053/2155/722/10544/5327/1361/2147
5534/5879/5291/140885/814/5532/10000/3727/2885/5601/8878/5595/5530/3716/3454/207/5296/2274/5290/7046/5468/5970/55423/3725/4792/1
1/836/10376/7277/1509/5170/10000/1965/5601/60/71/5595/6709/1616/1508/207/84823/2021/4893/329/5366/5296/5605/4001/5290/1522/5970/3
10000/107/5566/207/81570/4893/5563/5565/8660/5296/1979/6647/3667/5290/5564/5567/5562/3312/115/108/3643/6198/3306
814/2309/143686/23411/10000/107/51094/5566/1977/9821/207/4893/5563/10919/5565/8660/5296/3667/5290/5564/5468/5970/5567
/28998/6154/6217/6233/6171/6234/3921/6230/4736/6188/6150/6164/51069/6206/6208/51264/6223/54460/6210/6155/11224/6202/6189/6193/61

4/5579/5062/2932/5879/5291/1399/4659/2002/5728/5906/5500/1398/5170/10000/5501/387/2885/5601/60/3685/9475/71/6714/673/5595/56034/5
7280/10376/7277/5878/523/10383/60/10381/3685/7879/534/71/1783/535/8417/5868/9114/4353/537/155066/821/3920/8992/526/29927/729238/

0000/4193/1029/673/5595/56034/207/4893/2254/999/4233/5296/5605/1956/5290/5925
04/3845/2932/5291/5728/10000/2885/8324/8202/8321/6667/673/5595/207/4893/2254/10683/4040/6655/1452/7473/10297/5296/7476/5605/1956
7472/55802/10140/3313/219988/340529/26986/9337/5213/115752/27258/2023/2026/54512/4850/4848/25804/10766/22894/51010/131870/11846
0/1555/100137049/1573/1558/391013/2877/5321/2687/5743/874/5322/239/4056/11283/8398/2053/8605
09/5581/814/4205/808/10000/10672/107/5566/7019/5595/4087/207/6546/4893/4208/999/340156/5563/5565/22808/2786/8678/5605/2790/8862/
566/60/887/71/54207/478/7430/816/482/340156/817/480/6752/2773/3766/2771/5567/2770/5582/481
387/5516/1387/6667/5595/4087/657/7027/93/8454/79875/91/269/64750/3625/3400/9372/7046
79/5291/6389/291/5728/5500/4719/1509/818/10000/5501/5601/183/7416/7417/6667/4087/816/4723/207/4704/817/2597/5296/4726/5590/9882/4
45/5579/5332/5879/5291/1399/5906/2903/55740/1398/808/10000/107/387/60/71/5584/260425/6714/673/5595/84612/56034/5216/207/5217/489
332/351/836/2561/6571/5566/3760/673/5595/4893/2786/3360/2790/2783/2773/1562/2775/3751/3351/2771/5567/3356/5742/3361/121278/2770/
879/5291/5532/10000/6714/5595/5530/207/4893/9261/5296/5605/5290
8/5879/1457/387/1459/60/71/6714/1387/5595/10458/999/5770/4233
879/5291/836/5532/5781/2885/80329/673/5595/5530/259197/80328/3454/4893/6655/5296/51744/5605/3448/5290
/5566/183/5136/5530/2977/1508/153/2773/2771/5567/117/2770/9635 066/5879/5291/291/1182/10000/3146/60/71/7416/6714/7417/5595/8520/4353/207
107/5566/54407/6714/4905/2744/18/2786/2554/2559/2790/2783/2773/81539/2775/2563/23710/2771/5567/2558/2770/2752/5582/6529/2785/654

45/3066/814/5500/6571/2903/5569/808/5501/2885/5566/673/5595/8520/116443/4893/2786/6655/2790/2783/2773/2775/5499/2771/5567
8/1407/8454/8863/5563/5565/8864/5564/5562
5826/10350/10351/89845/10257/11194/23457/10057/20

5291/4659/5906/5500/10000/5501/10672/107/387/5566/60/9475/71/5584/6714/4067/5595
332/4209/4324/4205/1591/10672/107/3727/387/5566/408/6667/673/5595
2/5581/814/490/818/808/107/5566/183/478/816/466/51305/491/482/817
95/4893/999/7175/10342/5605
/7874/3066/5879/5291/5717/836/11047/5718/10000/4193/5701/5601/1027/5708/5707/25942/22938/4067/898/29110/3716/7431/3454/8900/207/
07/134/5566/3760/408/5136/5141/2786/2554/2559/2790/2783/2773/2775/2563/2771/5567/2558/2770/5582/2785/115/27115/108/2870/2565/181
10008/1300/1293/1292/1282/1298/1301/6510/9056/5644/1287/1305/340267/1307/1291/1289/1297/1294/50509/23439/3783/23428/51032/1361/3
291/836/5878/107/5052/5566/7879
8/5566/1639/4905/1783/5868/10540/84516
57176/57038/118672/57505

4193/26052/29924/387/1212/867/27183/408/382/7879/377/155382/29934/1213/5584/83737/116987/6714/9135/26056/30845/4087/10890/51100/
13/207/4893/482/9440/23389/10231/480/5296/5605/9882/488/5290/5567/2033/1733/6567/1735/4853/117247/5582/29079/481
427/207/4893/3827/79603/5522/5523/130367/5296/5605/5590/5290/2773/2771/5970/29956/596/2770/5582/5526/166929
/9817/996/22954/7322/7316/25847/7332/7321/8450/51588/6477/11065/27338/9039/8454/8451/4214/8453/329/7323/51619/64750/378884/8697/
3/9261/817/4214/5598/6655/5296/7531/5605/4793/3667/5290/4804/5970/2549/3725/4792/5609/596/6272/2889
/3800/5878/10383/10000/63916/5585/387/5601/127829/64746/60/10381/83658/1639/382/7879/9475/71/55207/1783/5595/5868/10540/84516/52
21/9342/1508/207/4893/664/5563/9706/8660/22808/64422/10010/5296/3920/8678/5605/3667/5290/3916/23710/5567/22800/5562/596
1/60/382/26100/9475/71/9252/8878/7416/6714/29110/5595/7322/7316/7321/5216/207/5217/8454/664/7307/5296/7323/8678/51619/4793/1956/
0/11047/808/5718/10383/10000/1457/5701/1965/5601/1459/6622/102/8324/10381/5708/5707/26100/7416/8321/7417/673/4137/5595/5530/1088
7/5522/1478/10284/5523
/1975/10289/22916/8661/8894/5976/7341/84321/81929/9669/5901/10284/7175/10209/8637/1979/1917/96764/10189/22794/55520/79023/55916/
/6789/5605/3667/1956/5290/5564/7046/604/23710/1950/9454/2033/2911/10733/5562 56/5290/5058/1950/2549/3725/5609/5582

49/2033/3725/112398
550/6511/25814/5296/8678/4976/488/5290/10105/6315/5700/5649/5709/2911/23152/7436/5582/5704/5702/3752/7419/2776/5686
1/6714/8321/673/5595/4087/7430/3481/1975/816/207/4893/7078/817/3549/4233/22808/6655/7473/5296/7476/5605/2066/1956/5290/4313/288/5
51164/7277/7415/9927/3800/818/11047/808/5718/10383/1457/5701/1965/7326/51465/5601/1459/6622/8324/10381/7317/5708/1639/5707/26100
16/207/5522/817/2786/5523/2790/2783/2773/3798/2775/5499/2771/5567/2770/5582/1816/5526/4129/2785
34/84612/2045/2043/816/4893/817/54910/10501/4233/3897/2048/10512/5296/9901/285220/5590/7852/5290/2773/2771/5058/6586/7474/9860/2
/84962/26524/9113/329/7003/7473/10297/7476/7531/5590/6657/7159/7046/5499/11211/166824

22/7321/56681/258010/51009/6745/10294/8454/10961/6184/23640/573/821/23471/7323/55666/51619/29927/3703/26232/7991/22872/3326/1013

1929/5563/9706/4040/6655/7473/5296/7476/5605/526/3667/9663/5290/11211/7474/5562/529/6520/525/5582/28956/7481/51606/1857/7472
381/382/9475/71/7009/6714/8440/4644/9071/4690/5595/10972/7430
/6546/491/482/5522/6330/817/5523/480/153/488/493/2773/186/5499/6324/146/2771/5567/7171/6543/10368/596/2770/5526/481/782/2776/115
97/5296/7476/91/5605/3625/5290/3400/6657/11211/6929/7474/3976/7481/8313/1857/3977/7472/3398/6498
898/7533/5595/3716/5315/3572/10971/5902/9114/8900/4893/9261/5366/5296/7531/2965

5576/4893/5563/5770/5565/8660/6655/5296/5605/89801/5590/3667/5290/5564/5499/5567/5313/5834/5562
196528/207/4893/6605/4233/4040/6655/1452/7473/10297/5296/7476/5605/7015/6601/1956/5290/5925/7046/11211
192111/7314/665
/71/6714/673/5595/7430/54434/4628/56034/5216/5217/10458/4893/3827/2254/340156/22808/79837/6655/10297/5296/5605/7852/1956/5290/46

58/207/6546/3827/491/4208/482/340156/9569/4880/8660/480/153/5605/488/3667/493/2773/10105/5499/146/2771
605/1956/5290/2773/2775/4313/390792/8688/2771/5567/2911/3725/3326/2288/54474/596/2770/3859/3312/3868/2776/115/3866/108/9166/5894
7/2911/3356/2697/2770/5582
1340/84321/27258/7307/55660/9716/153527/10946/10286/22827/10262/10713/6635/6100/10189/6634/10772/25804/6632/3178/23451/6628
8900/207/4893/9261/22808/90550/286826/5296/5605/144715/5290/5925/7046/5499

39/8454/6423/5663/817/144165/4040/1452/7473/10297/7476/79718
775/529/525/6529/51606/6540/6507

707/7416/7417/4137/65018/7316/7332/1616/816/4723/4704/5713/817/90550/4726/64837/2773/10105/3798/5700/2771/7384/5709/4718/5567/73
454/817/10459/7531/9748/22849/8697/5499/132864/5567
50/5230/5970/2033/112398/596/5582

3667/5290/2773/5449/2771/5567/2033

685/9475/6714/6667/29110/5595/5530/3716/207/4893/2786/6655/5296/5731/5605/2790/7852/3448/1956/2783/5290/2773/5925/2775

701/1965/60/10381/5708/1639/5707/26100/71/29979/140775/8878/7416/10280/29110/23636/56893/5530/65018/4741/9821/5868/9217/1616/10

1258/207/2696/5141/491/482/817/480/5296/153/6752/5605/3360/488/493/5290/2773/2641/5499/3351/2771/5058/5970/5567/2033/117/7432/37

5567/4853/3725/596
313/5970/6382/3725/5562/3326/5609/596/5608

/207/4893/4214/6655/3717/5296/5605/3448/5290/5925/7046/5970/2033/3725/4792/5609/596/3447/5582/5608/317
93/3768/4208/3761/340156/817/5563/5598/5565/5605/1956/2773/2775/1938/5564/5499/2771/5567/10368/3725/5562/2770/5582/57118
/1965/27153/54753/10929/163049/163050/219749/7594/27102/84775/148268/63934/22835/6714/7733/7773/7553/148254/30832/29110/57677/2

674/7143/1297/3679/2811/3912/3908/3678/3911/1286/7059/158326
85/534/55844/55502/5529/5584/5516/8321/1387/535/898/29110/5595/84612/3716/5315/5527/9114/3454/8900/537/207/4893/5522/5663/155066

/5993/3448/3916/5970/2033/3117/7421/50617/596/3447/317/4802/51606/3439/1379 66/5296/3667/5290/1938/5564

284/673/10369/4137/5595/5530/781/3481/785/4149/784/1616/56034/207/4893/2254/4208/9261/3925/4214/5598/23542/4233/22808/6655/6789/

290/2773/2775/4313/7046/2771/5970/5567/59350/3725/4792/5609/1278/2770
5601/1459/10381/5708/5707/5621/7416/7417/5595/5530/116443

893/9261/64422/2786/3717/5296/8678/5605/2790/3448/2783/5290/5925/23710/5970/2033/3725/4792/5609/7314/3447/5608
/26100/5434/1213/25942/7416/1387/7417/6667/9821/5433/10540/84516/4723/4704/5713/55860/160/9706/6506/8678/4726/5439/64837/6647/10

207/4893/5522/6655/644672/5296/7531/5605/3448/1956/5290/5925

4792/596

/5582/7481/1857/7472/2776/115/108/5894/7480 1/5567/596/2770/1141/5582

30/6935/7431/2744/406905/6659/900/4893/7078/406969/406957/3925/5598/8660/4233/6655/7473/10297/5296/113130/5605/3667/1956/5290

716/5902/8900/207/4893/4214/5901/8498/821/5296/5605/7015/5290/5925/8697/9184/7046/2114/5970/5567/6929/2033/23373/3117/3725/4792/

956/2773/5925/11211/9101/3284/2771/5567/3739/7474/2770/7481/8313/1857/7472
25/4998/8697/9184/7465
8451/2786/5296/5605/2790/7852/3448/2783/5290/2773/2775/7465/2771/5058/5970/3725/919/4792/5609/596/2770/3447/5582/5608
6/5590/2790/4793/7852/2783/5290/2773/2771/5058/5970/5567

05/7015/1956/5290/5925/7046/11211/1950/2549/7474/596/26291/7481/8313/1857/7472/2253

4/207/27/4893/2254/4233/22808/2786/6655/5296/6789/5605/2790/5898/1956/2783/5290/5923/8399/5058/1950/2114/4804/55770/5970/5567/25

565/644672/5590/9368/4627/5564/123720/5567/3725/9693/5562/5609

0990/5609/799/5608
725/4792/8739/596/317

33/65003/51263/9553/6160/6222/6152/25873/6203/6194/6201/6139/6227/6235/63931

5742/207/340156/329/4233/6655/5296/3918/256076/1956/5290/3694/5499/5649/5923/5058/1950
3916/4973/3117/50617/1778/529/525

/5290/5925/3714/11211/1950/4853/3725/7474/26291/7481/8313/1857/7472/2253 0

2783/2773/5564/7046/23710/2771/5567/6543/22800/5562

88/3667/5290/10105/4313/7046/5499/7384/4718/5970/1278/6390/4698/5582
3/2254/999/4233/22808/5296/5605/5590/23566/5898/1956/5290/2773/9170/2775/2771/1950/4804/2846/9693/2770/26291/5582/5608
5582/4129/27850/115/2572/2571/108/6539/2565

5713/5296/930/4793/3448/5290/5925/5700/5709/5970/956/3117/3725/919/4792/5609/596/3447/5608/317/5704/3439/5702/4794/5971
2/51764/10681/5578/200959/55970/5153/140679/54331/2550
769/1286/57642
84612/5868/378/60682/832/8723/57132/160/5867/147179/6643/8766/5119/5590/11031/829/64750/7852/1956/9372/3798/161/84249/7046/25978

10477/26232/7334/246184/140739/7314

16/6990/207/5217/55823/55860/9367/329/57617/2597/5605/113146/399/64837/5898/5290/3798/5058/55770/5970/2318/79026/9265/3725/3326/

826/5290/23710/5970/7336/9265/7334/3725/4792/7314/596
8/322/9821/4723/207/4704/4893/5663/5713/9706/8660/4040/90550/1452/7473/2597/10297/5296/7476/8678/4726/5605/64837/23621/488/3667/

54913/79897

499/11211/5058/2318/5567/2549/6382/22800/7474
/140775/5621/8878/7416/8321/7417/10280/29110/673/4137/5595/5530/65018/7316/7332/4741/9821/5868/9217/1616/10540/84516/816/4723/4 770/5696

0/5609/55768/596/23190/56886/23193/201595/3312/6500/10134/55968/51360/51035/8720/81567/22824/3301/3306/6185/3320/1409/10483/560

/147/27092/4635/4624/10827/9170/3694/5499/5058/1950

/5580/3306/3320/3875/10488/3860

45/11315/7314/2770/6390/4698/317/4129/5704/5702/4702/10131/7311/7419/5686/598/6391/4728/5705

540/84516/4723/5216/4704/5217/5713/55860/81929/23064/9706/90550/6506/10010/7175/8678/4726/64837/6647/2066/3798/5700/25978/23710/

25/22800/4792

84370/147949/148266/3716/57335/140612/90649/163081/55786/340252/22869/284323/1616/6429/3054/342892/3454/163087/207/7678/284306/

/6655/1452/5523/7473/10297/5296/7476/3918/5605/256076/7015/8992/5590/9368/526/3448/1956/5290/5925/3694/5700/11211/5649/1950/5970

5605/2066/1956/9479/7046/5923/5058/1950/4804/5970/2318/5567/10368/3725/9693/22800/5609/5495/26291/5582/5608

105/3798/161/5700/3766/6804/7384/5709/5468/4718/2033/5609/6390/4698/317/5704/246721/10891/5702/4702/5438/7419/2776/5686/6391/65
246184
49/11021/9101/9922/27

1778/4792/5609/596/5287/560

5290/10105/3798/5700/11211/7384/5709/4718/597

704/4893/5663/5713/55860/817/9706/4040/90550/1452/7473/10010/10297/7476/8678/4726/5605/64837/6647/488/10105/3798/5700/25978/1121

7384/310/5709/4718/10189/79023/55916/10133/596/6390/4698/3178/5608/317/5704/5702/4702/5686/598/8480/6391/10671/4728/1769/2733/57

7561/10795/7581/7711/51276/84924/440515/329/57615/79088/171392/3717/57711/56242/374900/5296/80032/80264/388569/374879/155061/11

/5567/2033/4853/50617/7474/1278/3447/529/525/5526/7481/8313/51606/1857/3439/747

07/10671/4728/1769/570

1/23710/6804/7384/5709/4718/5970/7345/11315/2911/7474/5609/10133/7314/596/6390/4698/5582/5608/317/7481/8313/5704/1857/7472/5702/
05/6432/9377/83544/5695/4842/4714/5688/10121/4218/7846/3084/2343
5509/152687/7766/3448/147686/79862/5290/93134/9668/170960/79973/284406/5499/10308/10189/5970/7637/126070/3117/90592/148206/5934

4702/10131/7311/7419/2776/5686/598/6391/10671/4728/1769/570

8/4792/83744/79898/596/3447/26149/80110/57541/317/286075/91664/7625/90333/3439/147660/90338/91120/598/90576/54106/25888/147923/

7189/253639/55900/4049/80095/7592/6885/6432/10794/148198/58500/136051/1079
