# Supplementary material for: Exploring the diagnostic markers of essential tremor: A study based on machine learning algorithms
Source: Open Life Sci. 2023 Jun 22;18(1):20220622. doi: 10.1515/biol-2022-0622 (PMC10290283; doi:10.1515/biol-2022-0622)
Supplement: Supplementary Table 10 [file biol-2022-0622-sm11.pdf]

Table S10: correlation among TNFSF4, TNFRSF14, and the diagnostic markers

Gene	immune cells	correlation	p-value
APOE	Tgd	0.564245	1.32E-05
APOE	Macrophages	0.421668	0.00185
APOE	TFH	0.37437	0.006253
APOE	Eosinophil	0.355588	0.009681
SENP6	Cytotoxic cell	-0.36105	0.008547
SENP6	T helper cell	0.379066	0.005584
SENP6	TFH	-0.3339	0.015557
ZNF148	CD8 T cells	0.387006	0.004593
